# Distinct Transcriptional Changes in Response to Patulin Underlie Toxin Biosorption Differences in *Saccharomyces cerevisiae*

**DOI:** 10.3390/toxins11070400

**Published:** 2019-07-10

**Authors:** Christian I. Oporto, Carlos A. Villarroel, Sebastián M. Tapia, Verónica García, Francisco A. Cubillos

**Affiliations:** 1 Departamento de Biología, Facultad de Química y Biología, Universidad de Santiago de Chile, Santiago 9170022, Chile; 2 Millennium Institute for Integrative Biology (iBio), Santiago 7500574, Chile; 3 Centro de Estudios en Ciencia y Tecnología de Alimentos (CECTA), Universidad de Santiago de Chile (USACH), Santiago 9170201, Chile; 4 Departamento de Ciencia y Tecnología de los Alimentos, Universidad de Santiago de Chile (USACH), Santiago 9170022, Chile

**Keywords:** *Saccharomyces cerevisiae*, mycotoxins, biocontrol, yeast, transcriptome

## Abstract

Patulin (4-hydroxy-4H-furo[3,2c]pyran-2[6H]-one) is a mycotoxin produced by a suite of fungi species. Patulin is toxic to humans and is a sporadic contaminant in products that were made from fungi-infected fruits. The baker yeast *Saccharomyces cerevisiae (S. cerevisiae)* has been shown to decrease patulin levels likely by converting it to the less harmful E-ascladiol, yet this capacity is dependent on the strain utilized. In this study we show that four representative strains of different *S. cerevisiae* lineages differ in their ability to tolerate and decrease patulin levels in solution, demonstrating that some strains are better suitable for patulin biocontrol. Indeed, we tested the biocontrol capacities of the best patulin-reducer strain (WE) in contaminated apple juice and demonstrated their potential role as an efficient natural biocontrol solution. To investigate the mechanisms behind the differences between strains, we explored transcriptomic changes of the top (WE strain) and worst (WA strain) patulin-biocontroller strains after being exposed to this toxin. Large and significant gene expression differences were found between these two strains, the majority of which represented genes associated with protein biosynthesis, cell wall composition and redox homeostasis. Interestingly, the WE isolate exhibited an overrepresentation of up-regulated genes involved in membrane components, suggesting an active role of the membrane towards patulin detoxification. In contrast, WA upregulated genes were associated with RNA metabolism and ribosome biogenesis, suggesting a patulin impact upon transcription and translation activity. These results suggest that different genotypes of *S. cerevisiae* encounter different stresses from patulin toxicity and that different rates of detoxification of this toxin might be related with the plasma membrane composition. Altogether, our data demonstrates the different molecular mechanisms in *S. cerevisiae* strains withstanding patulin exposure and opens new avenues for the selection of new patulin biocontroller strains.

## 1. Introduction

Mycotoxins are toxic secondary metabolites produced by fungi. These compounds represent a threat to food security as they recurrently contaminate human food and animal feed, especially when those products have been produced from fungal-infected agricultural commodities, such as fruits, grains and vegetables [1]. The main foodborne mycotoxins of public health concern are aflatoxins, fumonisins, ochrartoxins, zearalenone, tricothecenes, deoxynivalenol and patulin (PAT), which are primarily produced by fungi of the *Aspergillus*, *Fusarium* and *Penicillium* genera [2,3]. In particular, PAT is produced by several fungal species belonging to the genus *Byssochlamys*, *Aspergillus* and *Penicillium* and among these, *Penicillium expansum* (*P. expansum*) is one of the most prominent PAT producers [4]. *P. expansum* is the causal agent of blue mold rot in apple fruits and additionally it can infect pears, grapes, apricots and peaches [5]. PAT is highly toxic to human and animals. Several reports have shown genotoxic, cytotoxic, neurotoxic and teratogenic effects of PAT [6,7], however it is not considered a carcinogenic compound [8]. Due to the health risk that it poses, the concentration of PAT in food products is regulated in many countries, the majority of which have set a maximum allowed concentration of 50 µg/L [9,10]. Despite these regulations, PAT contamination is still found in food products in many countries [11], which in the case of apple products often occurs due to the mishandling of rotten fruits along the production chain [12].

Several approaches have been developed to diminish PAT levels at different stages before and along the apple juice production process [13]. The application of synthetic fungicides to control blue mold before and after harvest is a traditional strategy [14]. The overuse of fungicides is hazardous to human health and promotes fungicide resistance in phyto-pathogenic fungi [15]. PAT reduction in industrial settings includes physical processes such as pulsed light [16], ultraviolet radiation [17] and filtration by activated carbon [18]; however, the effectiveness of these techniques remains uncertain [13]. Recently, biological control strategies have been investigated to absorb PAT, in particular the use of microbial organisms such as lactic acid bacteria, yeast and fungi [19,20] or to metabolize it into less harmful compounds [21]. The baker’s yeast *Saccharomyces cerevisiae* is a promising microorganism to be used for PAT biocontrol. Early reports indicate that PAT is completely absent in apple juice that has been fermented by *S. cerevisiae* [22,23,24]. In addition, yeast cells can adsorb PAT, likely by binding it to cell wall components. Studies have shown that the abundance of PAT can be reduced by over 50% after 24 h of incubation with heat-killed *S. cerevisiae* cells [20]. Biocontrol using inactive yeast is amenable for industrial processes in which the products of yeast metabolic activity, such as ethanol, are unwanted. Despite this, the use of inactive yeasts in industrial settings is still in its infancy, mostly because only few strains have been evaluated and further studies are needed before yeast can be efficiently applied to industrial conditions.

Increasing efforts aimed to reveal the molecular effects of PAT in eukaryotic cells, including diverse cell lines and microbiological models, have shown that the accumulation of reactive oxygen species (ROS) is a common effect to PAT exposure [25]. PAT has been shown to react with sulfhydryl groups of nucleophiles, such as those present in the amino acids cysteine and methionine and in the antioxidant macromolecule glutathione [26]. The accumulation of ROS can lead to cell death due to oxidative damage on DNA, proteins and membrane lipids. In this context, the effect of PAT on the global gene expression pattern has also been investigated in two yeast species. The basidiomycete yeast *Sporobolomyces* sp. responds to PAT exposure by increasing the expression of genes related to oxidation-reduction processes, while lowering the expression of genes related to protein biosynthesis, RNA processing, translation, protein phosphorylation and the biosynthesis of amino acids, suggesting a reduction in general metabolic activity [27]. Similarly, in the *S. cerevisiae* laboratory strain several genes showing a function on oxidation-reduction were upregulated by PAT, in conjunction with genes associated with heat-shock and sulfur metabolism [28], suggesting the activation of defense mechanisms to resist PAT toxicity. Two main mechanisms of PAT detoxification have been proposed: i) a PAT adsorption at the cell wall and ii) PAT degradation by unknown mechanisms in cells. The yeast cell wall is known to bind mycotoxins, such as zearalenone [29], aflatoxin B1 [30] and PAT [20,31]. Furthermore, an array of factors have been shown to influence yeast’s mycotoxin adsorption, such as cell morphology and cell size [32] and particularly the relative content of beta-glucans and mannans in the cell wall [29,30,32]. Contrastingly, less is known about the detoxification of PAT after its uptake in yeast cells. It has been shown that during yeast cider fermentation, PAT is most likely metabolized through an unknown mechanism, into E-ascladiol [23], which is not toxic to human cells [24]. In addition, PAT was shown to be degraded by the yeast species *Rhodosporidium kratochvilovae* and *Sporobolomyces* sp into desoxypatulinic acid (DPA) and (Z)-ascladiol [27,33]. Knock-out mutants of *Sporobolomyces* sp for the *S. cerevisiae* homologous genes *YCK2*, *PAC2*, *DAL5* and *VPS8* were shown to be susceptible to PAT. Yet, these genes were proposed to function in PAT tolerance rather than detoxification [25].

In the past decade, the natural diversity of *S. cerevisiae* has been comprehensively investigated [34,35]. Six major yeast lineages have been discovered and these include two lineages that are strongly associated with human activities, that is, Wine/European (WE) and Sake (SA) and four undomesticated populations, that is, North American (NA), Chinese, Malaysian and West African (WA) [35,36]. With the aid of sequencing technologies and high-throughput phenotyping, several studies have exploited isolates from these lineages to understand genetic, ecological and evolutionary processes in yeast [37]. Comprehensive genetic resources have allowed the discovery of key alleles that influence traits relevant for industrial applications [38,39,40]. In this context, traits associated with mycotoxin biological control, such as mycotoxin resistance, detoxification and adsorption, can vary among yeast strains [41]. Although an enourmous genetic diversity is available in yeast for biocontrol studies, only a limited subset of strain has been explored to assess PAT biocontrol, which mostly consist of commercial strains related to beer or wine fermentation [22,23,31]. Hence, by exploring natural variation in *S. cerevisiae*, isolates with improved biocontrol capabilities can be discovered. 

With the aim of determining the molecular bases of PAT biocontrol differences across isolates and determine the efficiency of genetically diverse yeasts to decrease PAT concentrations in apple juice, we explored the natural variation of PAT reduction capacity in a set of *S. cerevisiae* isolates. More specifically, we assessed PAT reduction and global gene expression patterns in *S. cerevisiae* strains that were representatives of four major yeast lineages (i.e., WE, WA, NA and SA). We found large differences in PAT reduction, utilizing live cells, among *S. cerevisiae* isolates. In particular, we found that the WE and WA isolates were the most and least effective strains, respectively. Moreover, these two strains showed a different transcriptome profile in response to PAT and we analyzed these differences in the context of PAT resistance and detoxification. Finally, as a proof of concept, we assessed the capacity of inactive WE cells to diminish PAT concentration in apple juice, evidencing its potential as a biocontrol agent and its applicability to industrial apple juice production.

## 2. Results

### 2.1. Natural Variation in PAT Biocontrol in Saccharomyces Cerevisiae

**Figure 1:**
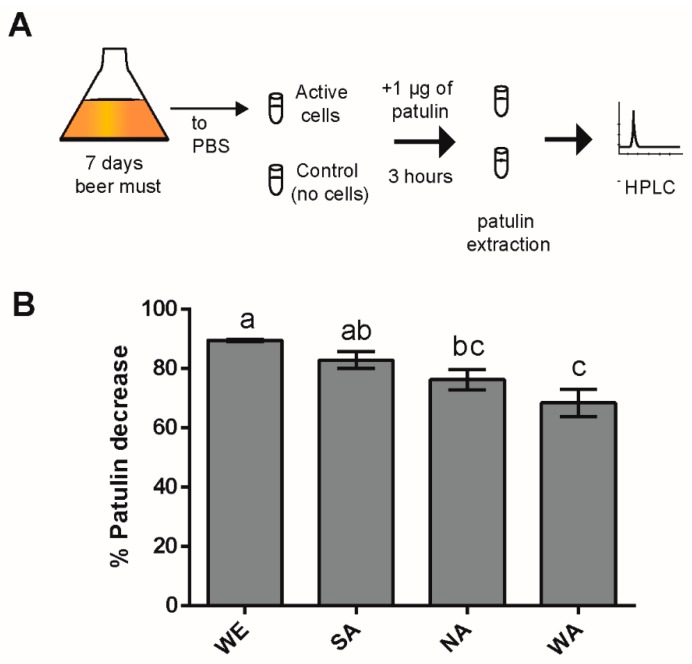
Patulin (PAT) concentration decreases in PBS medium. (**A**) Schematic representation of the experiment to determine PAT reduction in PBS by different isolates of *S. cerevisiae*. (**B**) The bars indicate the percentage of decrease in PAT concentration by isolates of the North American (NA), Wine/European (WE), Sake (SA) and West African (WA) lineages. Isolates were incubated in PBS. PAT was added to the medium prior to the incubation with live cells. Percentages are relative to the control, which was not treated with yeast cells. Letters indicate significant differences among strains (ANOVA, *p*-value < 0.05). Error bars show the standard error (n = 5).

Yeast natural variation can be exploited to discover strains that possess improved capabilities to absorb or detoxify mycotoxins. In an attempt to find strains showing different capacities for PAT biocontrol, we assessed the extent of *Saccharomyces cerevisiae* natural variation in the concentration decrease of this mycotoxin in liquid media. The strains chosen were representatives of the North American (NA), Wine/European (WE), Sake (SA) and West African (WA) lineages (Table 1); thus, an attempt was made to capture a large proportion of the genetic diversity described to date in *S. cerevisiae* [35,42]. First, we utilized Phosphate-Buffered Saline (PBS buffer) as matrix to assess PAT concentration decrease. Yeast cells were incubated in 1 mL PBS containing approximately 1 µg of PAT (see methods, Figure 1A). After three hours of incubation, the concentration of PAT diminished in the four strains, yet significant differences were found across them (ANOVA, *p*-value < 0.05). Specifically, the WE strain was the most effective at reducing the concentration of PAT (concentration of PAT decreased by 89.4%) while the WA strain was the less effective (concentration of PAT decreased by 68.3%) (Figure 1B). Altogether, these results suggest extensive variation in the ability of *S. cerevisiae* natural isolates to decrease the concentration of PAT in PBS medium.

### 2.2. Effect of PAT on Growth of WE and WA Strains

**Figure 2:**
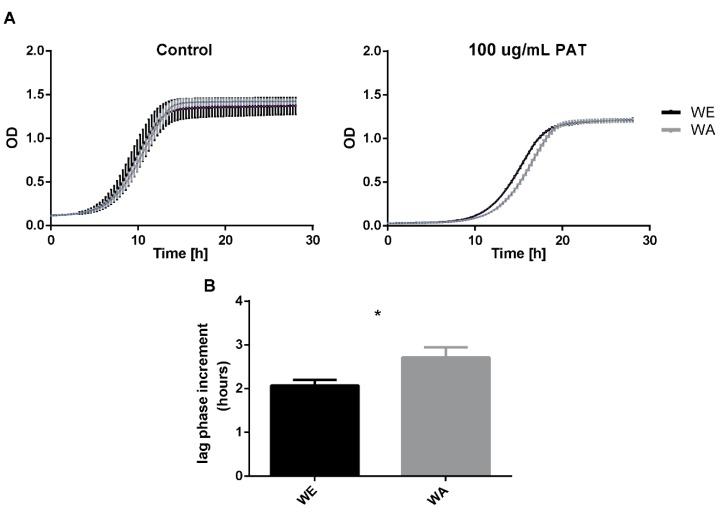
Effect of PAT on the fitness of WA and WE *S. cerevisiae* strains. (**A**) Growth curves of isolates WE (black) and WA (grey) lineages. Isolates were grown in Yeast Extract, Peptone, Dextrose media (YPD) as control (n = 2) and YPD supplemented with 100 µg/mL of PAT (n = 3). (**B**) Increment on the duration of the lag phase of isolates corresponding to the WE and WA lineages grown in presence of 100 µg/mL of PAT. Values are relative to their growth in control condition (YPD). (*) indicates significant differences due to treatment with PAT between strains (Student’s t test, *p*-value < 0.05). Error bars indicate the standard deviation (n = 2 and n = 3 in control and PAT treatments, respectively)

The PAT biocontrol differences observed between strains after PAT exposure could be associated with different levels of resistance to this mycotoxin. In order to evaluate the resistance to PAT of the two strains exhibiting the greatest differences for PAT biocontrol, WA and WE strains were compared for their growth capacity in microcultivation plates in the presence of 100 µg/mL of PAT. For this, strains were incubated during 24 h in the presence of the mycotoxin and optical density (OD) measurements were recorded every 20 min. For both strains the maximum OD diminished equally in the presence of PAT when compared to the control condition (Figure 2A). In addition, we observed that PAT treatment increased the duration of the lag phase in both strains, however this increment was longer in the WA strain (Figure 2B, *p*-value < 0.05). This demonstrates the greater PAT sensitivity in the WA genetic background and validates the greater PAT biocontrol potential in the WE background.

### 2.3. Effect of Heat-Killed Cells on PAT Biocontrol in Apple Juice

**Figure 3:**
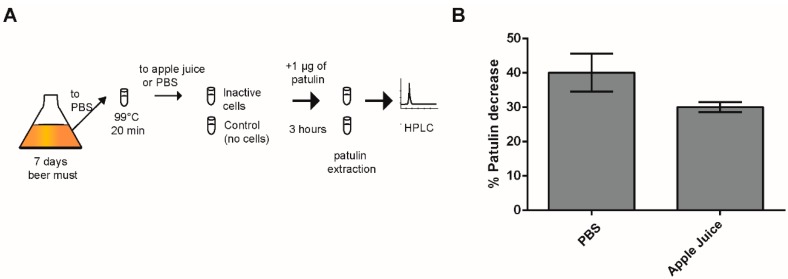
PAT reduction by inactive yeast. (**A**) Schematic representation of the experiment to determine PAT reduction in apple juice by inactive yeast cells. (**B**) The bars show the percentage of PAT reduction in PBS (n = 4) and in apple juice (n = 9) by inactive cells of the WE isolate. Percentages are relative to the control, which was not treated with yeast cells. Error bars indicate the standard error.

We showed that yeast cells, in particular those of the WE strain, quickly decreased a significant fraction of the PAT available in solution. However, yeast cells that are in a metabolic inactive state might be preferable as biocontrol agent in the food industry. It has been shown that inactive yeast can still substantially reduce PAT in apple juice [20]. Here, as a proof of concept, we assessed whether inactive WE yeast cells were able to decrease the available PAT in PBS solution and in commercial apple juice. For this, we added 1 µg/mL of PAT into a commercial apple juice (PAT free) or PBS and estimated PAT biocontrol by inactive WE cells (Figure 3A). For this, yeast cells were inactivated by heat exposure at 99°C for 20 min and then incorporated into both solutions containing PAT. After three hours of incubation with heat-killed WE cells, we observed a 40% to 30% decrease in the concentration of PAT in PBS and apple juice, respectively (Figure 3B). The decrease of PAT in apple juice was lower compared to that observed in PBS media (40%), yet this difference was not significant (p = 0.057, Figure 3B). Apple juice and PBS greatly differ in pH (pH 3 and pH 7.4 respectively), which could impact PAT reduction by yeast cells. Even though the decrease in PAT concentration in apple juice was less than in PBS, the inactive yeast still had a negative effect on PAT after only three hours incubation. These results confirm the potential utilization of inactive yeast in industrial processes to remove PAT from solutions.

### 2.4. Contrasting Transcriptomic Changes after PAT Exposure in WA and WE Strains

**Figure 4:**
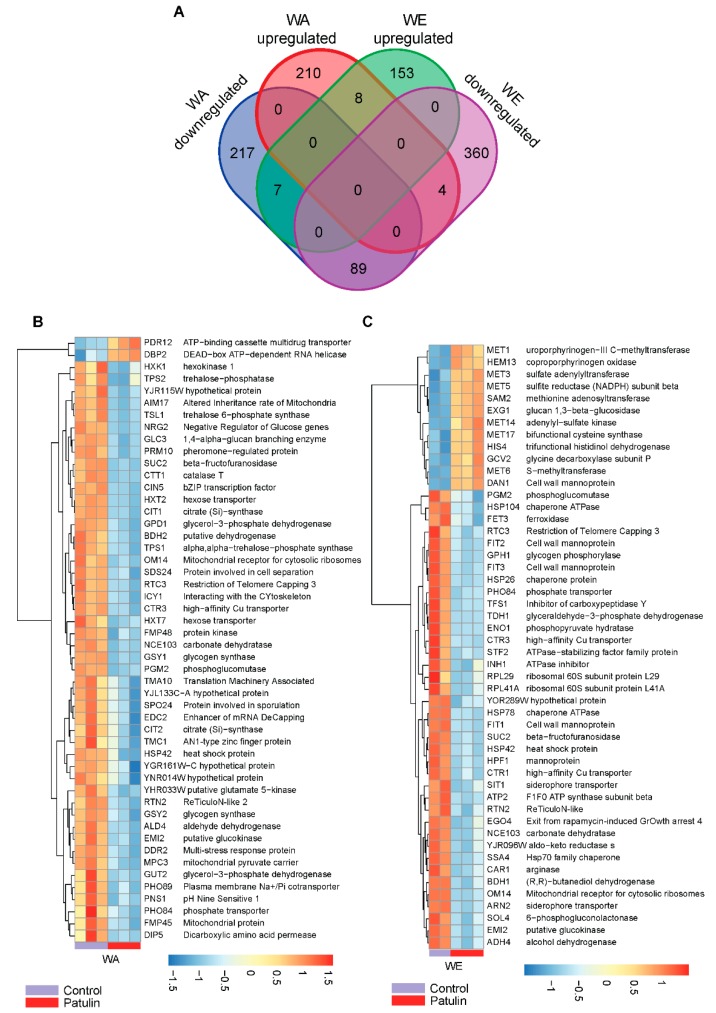
Transcriptome profile of the WE and WA isolates after PAT exposure. (**A**) A Venn diagram indicating the number of Differentially Expressed Genes (DEGs) upregulated or downregulated in WE and WA strains after incubation of 3 h with PAT. (**B**,**C**) Heatmap of hierarchical clustering shows the top 50 most significant genes between untreated and PAT treated WA and WE strains. Red indicates up-regulation and blue indicates down-regulation. Expression values were converted to z-scores and scaled by rows (genes).

In order to investigate the possible mechanisms underlying the differences in PAT biocontrol and susceptibility between the WE and WA strains, we studied their transcriptome after an incubation period of three hours with 100 µg/mL of PAT. A total of 12 samples, consisting of two strains grown in triplicates without PAT exposure (controls) and exposed to PAT, were used for transcriptome sequencing using Illumina HiSeqX (Table S1). A total of 427 and 513 differently expressed genes (DEG, adjusted *p*-value < 0.05) in response to PAT exposure respect to the control condition were found in WA and WE, respectively (Figure 4A). The fraction of up and down-regulated genes differed in both strains (Table S2). Among upregulated genes, 210 and 153 were upregulated in WA and WE respectively, however only eight of those genes were common in both strains. Among these, we found *MNN1*, a gene encoding for an Alpha-1,3-mannosyltransferase and upregulated 1.3-fold in PAT compared to the control condition in both strains. In a similar manner, 217 and 360 DEG were downregulated in WA and WE strains respectively, among which only a small set of 89 genes were shared by both strains, some of them with hexokinase activity (i.e., *HXK1*, *EMI2* and *GLK1*). A hierarchical cluster analysis of the 50 most significant DEGs in the WA strain shows that most of them (48 genes) were repressed (Figure 4B), many of which corresponded to genes associated with sugar metabolism and transport. On the other hand, cluster analysis of the 50 most significant DEGs in the WE strain highlights the upregulation of sulfur-metabolism related genes in response to PAT and the differential regulation of genes that encode for mannoproteins (Figure 4C). In general, these results indicate a clear distinct transcriptomic regulation in response to PAT between the strains that showed the highest and lowest levels of PAT reduction. 

**Figure 5:**
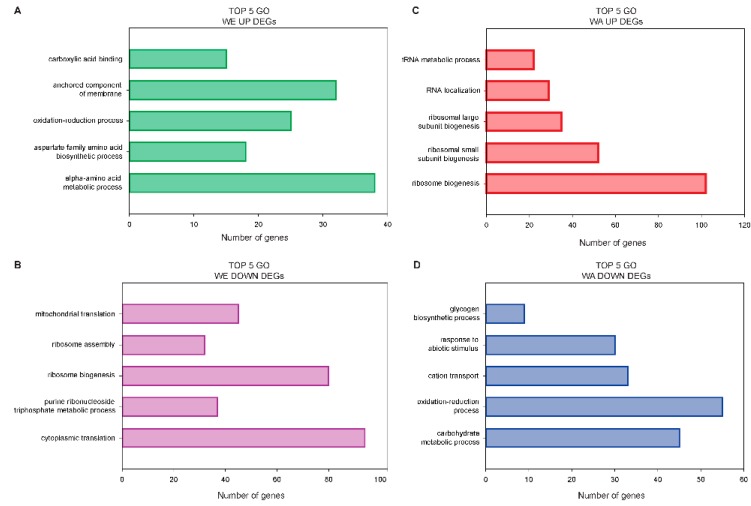
The bar plots show the 5 most enriched GO-terms of the type “Biological Process” in upregulated and downregulated DEGs in the strains WE and WA after PAT exposure. (**A**) WE Upregulated, (**B**) WE Down regulated, (**C**) WA Upregulated and (**D**) WA Downregulated.

Next, we assessed each genotype and conditions separately to identify molecular and biological processes enrichment in functionally related genes. For this, GO-term enrichment analysis of DEGs further demonstrated the distinct transcriptomic responses of both strains. The most enriched terms within the “Biological Process” (BP) category among WE upregulated DEGs were those associated with alpha amino-acid metabolic process (38 genes, Log P = −22.29, Figure 5 and Table S2). Among genes in this category, many also contained the GO term “sulfur compound metabolic process” (15 genes) and “drug metabolic process” (20 genes). In addition, among WE induced genes, the “extracellular region” was the solely enriched term within the category “Cellular Component” (CC) (13 DEGs, LogP = −4.59), of those, 11 DEGs also contained the GO term “anchored component of membrane,” suggesting an active role of the membrane composition. A different transcriptome landscape is observed by GO-enrichment analysis among WA upregulated genes (Figure 5). In this case, the majority of genes were associated with RNA metabolism, principally “ribosomal biogenesis” (102 genes, LogP = −62.58) and “RNA localization” (24 genes, LogP = −10.51). A few common enriched GO terms were observed among suppressed genes in both strains, such as “carbohydrate metabolic process” and “iron coordination entity transport.” However, downregulated genes in WE were predominantly associated with the BP “cytoplasmic translation” (94 genes, Log P = −56.43), “ribosome biogenesis” (80 genes, Log P = −18.13) and “mitochondrial translation” (45 genes, Log P = −14.38), whereas suppressed genes in WA were mostly associated with redox processes (55 genes, Log P = −10.17) and “carbohydrate metabolic process” (45 genes, Log P = −11.87). Within the CC category, in WA most repressed DEGs fell in the terms “Plasma membrane” or “Storage Vacuole” while in WE most suppressed DEGs fell in the terms “ribosomal subunit” or “mitochondrial protein complex.”

**Figure 6:**
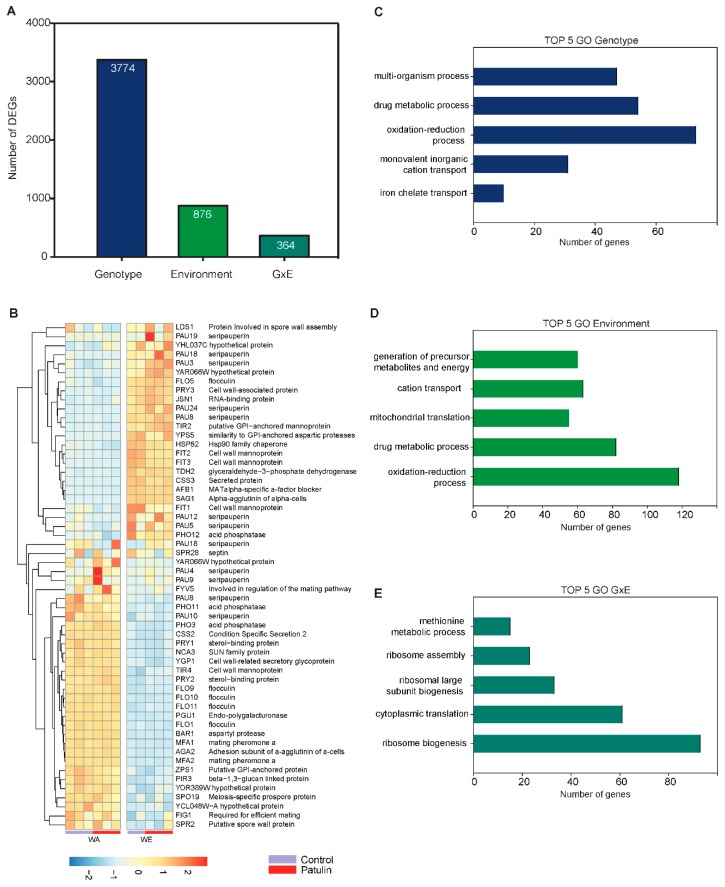
Gene-Environment interaction in the response to PAT in WE and WA strains. (**A**) Number of genes that show a significant (FDR < 0.05) “Genotype” (WE or WA) effect, “Environment” (PAT) effect or Gene and Environment (GxE) interaction effect. (**B**) Heatmap of hierarchical clustering shows DEGs classified with the GO-term “Cell-wall” exhibiting a “Genotype” effect. Red indicates up-regulation and blue indicates down-regulation. Expression values were converted to z-scores and scaled by rows (genes). (**C**–**E**) The five most enriched GO-terms of the type “Biological Process” in upregulated and downregulated genes that show a significant effect in Genotype, Environment and the interaction GxE. For GO-term enrichment of “Genotype” effect genes, we selected those genes which had an absolute fold change greater than 2-fold (706 genes).

In order to determine the effect of the Genotype (strain) and the interaction with the environment (control condition and PAT exposure) and detect genes exclusively responding to the mycotoxin treatment, we performed a GxE analysis. In this analysis, we dissected ‘G’ genes whose expression differed by genotype but not by an environmental change. ‘E’ genes exhibiting similar expression levels for both strains but varied between treatments. Finally, ‘GxE’ genes were defined as those having a genotypic and environmental difference. This analysis identified a total of 3,774 genes affected by G, 876 genes affected by E and 364 genes showing a significant GxE interaction (FDR < 0.05, Figure 6A). Overall, the large fraction of genes affected by PAT between both genotypes demonstrates the differential impact of this mycotoxin depending on the genotype.

Next, we explored which GO terms were enriched among DEGs that showed a G, E or GxE effect. First, as there was an extensive number of DEGs that showed a significant “Genotype” effect, we selected those DEGs that showed a change of at least 2-fold (706 genes) for GO enrichment analysis in this condition (Figure 6C). The terms “plasma membrane” (94 genes, Log P = −14.27), “cell wall” (40 genes, Log P = −12.95) and “vacuole” (70 genes, Log P = −6.23) were significantly enriched within the CC category, which suggest that WA and WE differ considerably at their cell wall and plasma membrane composition (Figure 6B). Hierarchical clustering of the “Genotype” genes within the “cell wall” category further illustrates the distinct expression of genes encoding for mannoproteins, seripauperins and flocculins (Figure 6B). Genes that showed a significant “Environmental” effect were mostly mitochondrial (174 genes, Log P = −22.5) or associated with the plasma membrane (104 genes, Log P = −4.25). In the BF category, there were 118 genes containing the term with “oxidation-reduction process,” which was also enriched among genes that had a “Genotype” effect (73 genes). Finally, among genes that showed a significant GxE interaction, the terms “ribosome biogenesis” (93 genes, Log P = −34.63) and “cytoplasmic translation” (61 genes, Log P = −28.96) exhibited the lowest p-values in the BP category, suggesting that these two represent the most significant differences between strains and conditions.

## 3. Discussion

The mycotoxin PAT is a harmful contaminant in fruits and processed food products and it is especially prevalent in products derived from apple fruit such as apple juice, compote, dried apples and cider. PAT has been shown to have carcinogenic and teratogenic effects in animals [6,43]. Due to the health risk that it poses, several studies have investigated how to diminish PAT levels along the food production process. These studies have taken different approaches, generally using UV radiation or adsorption by carbon filters [13]. Biocontrol, which includes the adsorption and/or degradation of toxins by microorganisms, provides an environmentally friendly approach. Making it a promising biological agent to control PAT, *S. cerevisiae* can adsorb [31] and metabolize this mycotoxin [23]. In this study, we have shown that the concentration of PAT decreases by different naturally occurring isolates of *S. cerevisiae*. We demonstrated that active cells of natural occurring isolates of *S. cerevisiae* from diverse geographic regions and industrial settings can diminish PAT concentrations after only three hours of incubation. Furthermore, we observed extensive variation among *S. cerevisiae* isolates in their capacity to diminish PAT concentration in solution, which suggests the possibility to genetically improve yeast for PAT biocontrol. In particular, the Wine/European isolate was particularly efficient at decreasing the concentration of PAT in solution. In this context, Stinson et al. [22] reported a complete reduction of PAT levels in apple juice after being fermented by yeast. More recently, Moss and Long [23] have shown that PAT is converted to more polar compounds, such as E-ascladiol, during yeast fermentation. In our experiments, a significant fraction of the PAT levels in PBS medium decreased after 3 h (70–90%). Moreover, PAT was no longer detected when we tried longer incubations periods (24 h, data not shown). Genetic variation among yeast strains could account for the different efficiencies in PAT reduction that we and others have found. Indeed, we showed that the transcriptional profile after PAT exposure differs extensively between two divergent *S. cerevisiae* strains, which implies that PAT elicit distinct responses in yeast and these differences could be due to dissimilar stresses that these strains encounter in their original environments.

### 3.1. Contrasting Transcriptome Responses after PAT Exposure of Two Yeast Strains

**Figure 7:**
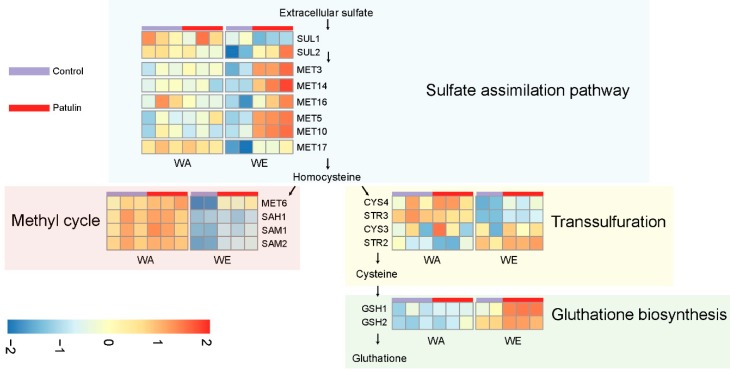
Differential expression of genes related to sulfur metabolism in response to PAT in WA and WE strains. The heatmaps show the gene expression values which were converted to z-scores and scaled by rows (genes).

The transcriptome profile of the isolates belonging to the WE and WA lineages after PAT exposure revealed that these strains develop different mechanisms in response to this toxin. In particular, a shift towards protein metabolism was observed among WE upregulated genes, which contrast to the shift towards RNA metabolism which was predominant among WA upregulated genes. The effect of PAT in eukaryotic cells has been largely studied [25]. PAT is a strong electrophile which is able to interact with different types of macromolecules, among which PAT is highly reactive with free thiol groups, such as those contained in cysteine residues [44]. Interestingly, in response to PAT, the WE isolate induced the expression of several genes involved in different steps of glutathione, methionine and cysteine biosynthesis, such as those conforming the sulfur assimilation pathway *MET3*, *MET14*, *MET16*, *MET5*, *MET10* and *MET17* (Figure 7). Also, the high affinity sulfate permease *SUL2*, had increased expression in WE after exposure to PAT. Contrastingly, none of these genes responded to the PAT treatment in the WA strain. The concerted upregulation of the sulfur pathway in WE could be a response to a deficit in functional sulfur-containing proteins or amino acids, such as cysteine [45], which agrees with PAT reactivity with these types of molecules. Alternatively, the sulfate pathway could have been induced to suffice a higher demand of glutathione, which yeast uses to tolerate redox stress, similar to what was previously found in yeast exposed to the thiol-reactive metals: arsenite [46] and cadmium [47].

The WA isolate showed a higher number of upregulated DEGs than the WE isolate (210 vs 153), the majority of which were not highly induced, that is, only 8 DEGs in WA were induced 1.4-fold or higher compared to 40 DEGs in WE. The gene *PDR12*, which encodes for a multi-drug transporter necessary to develop weak-acid resistance [48], was the highest induced gene in WA (2-fold). Moreover, genes related with ribosome biogenesis were also upregulated in the WA strain, likely as a compensatory mechanism to the toxic effects of PAT. A more substantial effect of PAT upon gene expression was observed among repressed DEGs in the WA strain, out of which 142 were at least 1.4-fold downregulated (of a total of 217 repressed DEGs). Among these, the hexose transporters *HXT11*, *HXT2*, *HXT7* and *HXT6* were downregulated. Interestingly, 82 genes showing a sort of functions but whose abundance are known to increase under DNA replication stress, were repressed by PAT in WA, while only eleven of those were also downregulated in WE. Many of these genes were also repressed under uracil or sulfur limitation in yeast grown in chemostats, which commit to cell cycle arrest after exhausting the limiting nutrients [49]. This set of genes might be involved in promoting yeast mitotic grow and their suppression might be a response of WA cells to survive the stress provoked by PAT. We observed that PAT exposure diminished the total biomass and incremented the duration of the lag phase in both WA and WE isolates; although for the lag phase the negative effect of PAT was stronger in the WA strain. Still, both *S. cerevisiae* strains were able to grow in the presence of high concentrations of PAT, suggesting that both transcriptional responses develop mechanisms to withstand this mycotoxin. In fact, *S. cerevisiae* is known to be resistant to various mycotoxins [41,50]. 

In comparison to a previous transcriptome study on *S. cerevisiae* response to PAT, we found similarities among the induced genes of the WE isolate, in particular the upregulation of the sulfate pathway and redox stress responsive genes [28]. In that work, the laboratory strain S288c was used, which is genetically closer to WE lineage than to the WA. In addition, a recent report showed that PAT strongly induces the gene *RPN2*, whose expression is known to be positively correlated with 20s proteasome activity; suggesting that PAT has a proteotoxic effect [44]. However, this gene was not among those DEGs either in the WE or WA strains. In our study, DEGs encoding for cell wall and plasma membrane components explained most of the variation between strains, demonstrating that cell wall composition is essential for an efficient PAT detoxification. 

### 3.2. Basal and Induced Expression of Plasma Membrane Protein Components Could Determine Differences in PAT Biosorption

Previously, it has been reported that inactive yeast can reduce up to 70% of PAT in apple juice after 24 h of incubation [20], suggesting that components of the yeast cell wall can efficiently remove PAT. Further to this, the structure and components of the cell wall have been shown to be major factors contributing to the adsorption of mycotoxins [31,36]. The yeast cell wall consists of two layers where the inner layer contains β-D-glucans and the outer layer contains mannoproteins. Both components have been found to be essential for PAT biosorption [31]. Furthermore, Luo et al [32] showed that the relative content of 1,3-β-glucan in the cell wall positively correlated with PAT adsorption among different yeast species. However, in our study we found that the majority of the genes involved in the biosynthesis of 1,3-β-glucan and 1-6-β-glucan were not differentially regulated by PAT exposure, nor had a significant “Genotype” effect. Yet, we found in the WE isolate an enrichment for the GO term category “anchored component of membrane,” where *DAN1*, *TIR2 and TIR4* genes encoding for mannoproteins were significantly upregulated by PAT exposure, while in WA these genes did not significantly respond (*TIR2* and *TIR4*) or were less induced by PAT (*DAN1*, “GxE” FDR = 0.008). These mannoproteins have been shown to be induced by anaerobic growth [51], cold shock [51] and hydrostatic pressure [52]. In contrast, in both strains the mannoprotein encoding genes *FIT2* and *FIT3*—which play a crucial role in iron assimilation [53]—were downregulated, although the basal levels of transcription of both genes greatly differed between WE and WA strains (20 and 26 fold higher in WE), as shown by their highly significant “G” effect (“G” FDR < 0.001). Furthermore, the analysis of both transcriptomes by “Genotype” component demonstrated that both strains had transcriptional landscapes widely different at the basal control condition, in which a significant proportion of DEGs encoded for plasma membrane or cell wall proteins. Overall, these gene expression differences should translate into a distinct membrane composition of both strains before and after being exposed to PAT, first largely due to the different genotypes (“G” effect genes) but also due to the contrasting response to PAT exposure. A different composition of the membrane and cell wall could explain the differences in PAT detoxification between the WE and WA strains. Further research could show if different mannoproteins, their abundance or other membrane components, have different impact at diminishing PAT in solution and if their overexpression boost the capacities of yeast to control PAT in media.

### 3.3. PAT Biocontrol Using Inactive WE Cells

In our study the utilization of inactive yeast for biocontrol was shown to not affect juice quality by the fermentation process, making it a preferable method, than for instance using, active yeast [20]. The adsorption of mycotoxins by microorganisms is considered an important contributor to the reduction of these compounds in animal and human food products [36]. Here, after three hours of incubation, PAT reduction by inactive WE yeast was considerably lower than that achieved by active WE cells. This suggests that some biological and chemical processes, such as enzymatic reactions, influence the reduction of PAT at early stages of incubation. Still, we showed that inactive WE cells could reduce the concentration of PAT in solution after a short incubation period, constituting a convenient procedure to complement strategies for PAT control under industrial settings.

Overall, the distinct transcriptome responses found in this work and in previous reports suggest significant variation in gene expression after PAT exposure among *S. cerevisiae* strains. Moreover, these differences are highly enriched for genes encoding for proteins involved in cell wall and membrane composition, together with the sulfur assimilation pathway. This demonstrates that yeasts cells differ in their biocontrol potential and that in our case, strains obtained from processes associated with wine fermentation might be the best candidates for patulin biocontrol.

## 4. Materials and Methods

### 4.1. Yeast Strains and PAT Stock Solutions

*Saccharomyces cerevisiae* strains utilized in this study are depicted in Table 1. For each experiment, strains were recovered from glycerol stocks and grown on YPDA (Yeast extract, Peptone, Dextrose, Agar) plates for 48 h. *Saccharomyces* spp. strains are available upon request [54,55].

The PAT (P163 - Sigma-Aldrich, Darmstadt, Germany) mycotoxin used in this work was dissolved in chloroform to stock concentrations of 100 μg/mL, 3 mg/mL and 10 mg/mL depending on the assay and were then aliquoted into 1 mL screw-capped cryotubes and stored at −80 °C. Before each experiment and depending on the assay, aliquots were diluted in either YPD or PBS to achieve a concentration of 100 μg/mL or 1 μg/mL, respectively and immediately utilized to avoid any chloroform evaporation.

### 4.2. Growth Assays

The microcultivation phenotyping assay was performed as previously described [39]. Briefly, cells were pre-cultivated in 200 μL of YPD medium for 24 h at 28 °C. For the experimental run, yeast strains were inoculated in triplicates in 96-well plates to an optical density (OD) of 0.03–0.1 (wavelength 600 nm) in 200 uL of YPD or YPD containing 100 μg/mL of PAT (1% chloroform). The effect of growing yeast cells in YPD containing 1% chloroform was tested and no differences were found (*p*-value > 0.05, ANOVA) when compared to the YPD control (Figure S1). Plates were incubated at 28 °C for 48 h. Absorbance was measured in a Tecan Sunrise microplate reader (TECAN, Austria). Optical density (O.D.) was obtained every 20 min. Growth rate parameters were estimated using the software GrowthRates ver. 3.0 [56].

### 4.3. PAT Biocontrol Assay

Yeast biomass for the biocontrol experiments was generated from beer fermentation. For this, 1 × 10^6^ cells/mL of each strain were inoculated into *Pilsener* beer must (Muntons®, Stowmarket, UK) and were grown for seven days (Figure 2A). Cells were collected after seven days of fermentation. Subsequently, cells were washed, and total biomass was weighed.

For the biocontrol assay, cells were resuspended to achieve a final concentration of 50 mg FW/mL in PBS buffer containing 1 μg/mL of PAT (1% chloroform) [20]. Previous studies determining the ability of *S. cerevisiae* and lactic acid bacteria to reduce Aflatoxin B1 levels have used this solution [57,58]. Cells and PAT were incubated for three hours at 25 °C with constant shaking (1050 rpm). A negative control sample containing PAT and no cells was utilized (Figure 1A). Assays were performed in triplicates and independent experiments were run three times (overall nine assays). After three hours, yeast cells were removed by centrifugation at 13,000 rcf for five minutes; after which 200 μL of supernatant were collected for PAT extraction.

### 4.4. Apple Juice Biocontrol Assay

Yeast cells were killed by heat shocking at 99 °C for 20 min with constant shaking (700 rpm). Subsequently, the cells were centrifuged at 13,000 rcf at 4 °C for five minutes. The biocontrol assay was performed as previously explained except that 1 mL of apple juice containing 1 μg/mL of PAT was used instead of PBS buffer. Three replicates were prepared for each isolate type (each 300 μL) and independent experiments were performed three times. After three hours, the assays were centrifuged and 200 μL of supernatant were collected for PAT extraction.

### 4.5. RNA-Seq

Cells of *S. cerevisiae* strains WE and WA were grown overnight in liquid YPD at 28 °C to reach an O.D of 0.6, after which 5 × 10^6^ cells were inoculated in 2 mL of YPD (3% chloroform) or YPD containing 100 µg/mL of PAT (3% chloroform). These cells were incubated for three hours at 28 °C with shaking. Subsequently, yeast cells were washed three times with PBS buffer and total RNA was extracted following a hot-formamide protocol [59] and were further treated using GeneJET RNA Cleanup and Concentration Micro Kit (Thermo Fisher Scientific). RNA samples were sent for library preparation and sequencing to BGI (Shenzhen, China). Samples that did not meet quality criteria (RIN) were discarded from subsequent sequencing. Raw reads were stored in NCBI’s SRA database and can be found with the code PRJNA540854.

Read quality was visually assessed with FASTQC (http://www.bioinformatics.babraham.ac.uk/projects/fastqc/). Raw reads were processed with fastp (adaptor trimming, low quality 3’ end trimming) [60]. The global gene expression of three control samples of WA, two control samples of WE, three PAT-treated samples of WA and three PAT-treated samples of WE were analyzed. RNAstar ver. 2.7.0 [61] was used to map reads to *S. cerevisiae* R64-2-1 reference genome using default settings. Subsequently gene counts were obtained with featurecounts ver. 1.6.4 [62] using R64-2-1 gene annotations. Summary statistics of raw and processed reads and mapping are shown in Table S1. Statistical tests on differential gene expression found in the comparison of PAT-treated or untreated samples for each strain were done with DESeq2 package in R [63]. Genes showing an adjusted p-value of 0.05 or less were considered as Differential Expressed Genes (DEGs). The statistical analysis of the “Genotype” effect, ”Environment” effect or the interaction of both effects (GxE), were done with the EdgeR package [64] using the glmFit function. Hierarchical clustering and heatmaps were calculated and rendered using the pheatmap R package. GO-term enrichment analyses and graphical outputs were obtained using Metascape webserver (min overlap = 3, *p*-value cutoff = 0.01,min enrichment = 1.5) [65].

### 4.6. HPLC PAT Quantification

A total of 200 μL of supernatant obtained from biocontrol assays were collected from each sample and added to a 1.2 mL PAT extraction solution containing: ethyl acetate-Hexan in a 95:5 (*v*/*v*) ratio, 1 mL of PBS buffer and 3.5 mM of NaHCO_3_. Samples were mixed by vortexing for two minutes and were then centrifuged for two minutes at 200 rcf. Subsequently, 700 μL of the upper phase of the mixture were obtained and transferred to a new falcon tube containing 8 μL of acetic acid. Finally, the resulting solution was dried utilizing nitrogen and stored at −20 °C before HPLC analysis was conducted.

PAT final concentrations were estimated using HPLC analyzer (Shimadzu Prominence) connected to a UV absorber set at 275 nm. An INERTSIL ODS-3 column with an internal diameter of 4.6 mm was used. The mobile phase had a 1 mL/min flow. A 90 μL subsample was injected for each sample. The PAT decrease (D%) was calculated using the following equation:

(1) $$D\%=\frac{C_{0}-C_{f}}{C0}\cdot 100$$

where *C*_0_ and *C_f_* are the initial and final concentrations of PAT (mg/L), respectively.

### 4.7. Statistical Analysis

The growth rate (µmax), lag phase duration and PAT decrease levels were compared by analysis of variance (ANOVA). Additionally, comparisons of PAT decrease levels between live and dead WE cells, and between PBS buffer and apple juice, were performed using Student’s *t*-tests. All analyses were performed using GraphPad Prism (version 6.01, 2012). *p* Values less than 0.05 were considered statistically significant.

## Figures and Tables

**Table 1:** *Saccharomyces cerevisiae* strains used in this work.

Strain Code	Lineage	Species	Reference
DBVPG6765	Wine/European	*S. cerevisiae*	[35]
YPS128	North American	*S. cerevisiae*	[35]
DBVPG6044	West African	*S. cerevisiae*	[35]
Y12	Sake	*S. cerevisiae*	[35]

## Supplementary Materials

The following are available online at https://www.mdpi.com/2072-6651/11/7/400/s1, **Figure S1**: Effect of 1% chloroform on the growth of the WE and WA strains; **Table S1**: Summary statistics of RNA sequencing reads and mapping to the *S. cerevisiae* reference genome; **Table S2**: Differential gene expression data and GO-enrichment analysis of differentially expressed genes.
