# Supplementary material for: Distinct Transcriptional Changes in Response to Patulin Underlie Toxin Biosorption Differences in Saccharomyces cerevisiae
Source: Toxins (Basel). 2019 Jul 10;11(7):400. doi: 10.3390/toxins11070400 (PMC6669508; doi:10.3390/toxins11070400)
Supplement: Supplementary file 1 [file toxins-11-00400-s001.zip › toxins-523720-si/Figure S1.pdf]

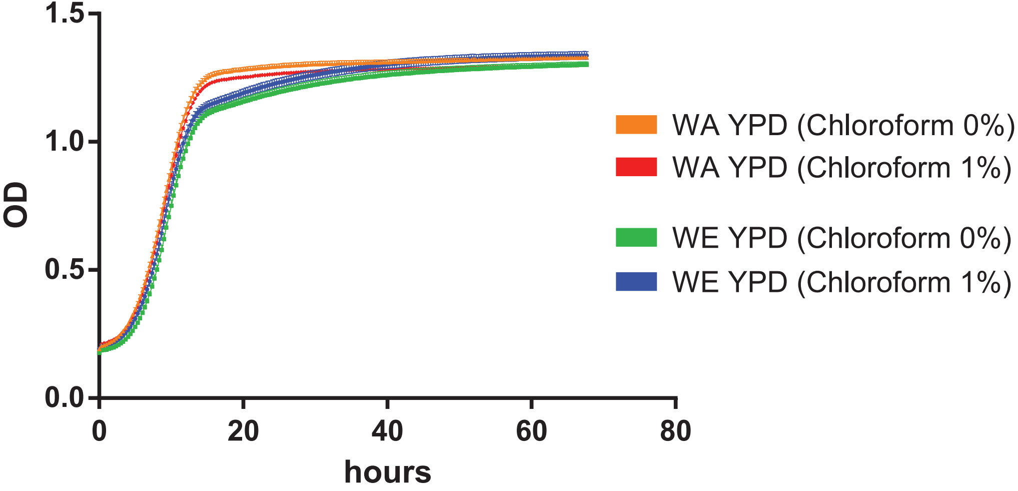

Figure S1. Effect of chloroform on the growth of the WE and WA strains. Isolates were grown in YPD control and in YPD containing 1% of chloroform (n=3). Error bars indicate the SEM.
