# Supplementary material for: Distinct Transcriptional Changes in Response to Patulin Underlie Toxin Biosorption Differences in Saccharomyces cerevisiae
Source: Toxins (Basel). 2019 Jul 10;11(7):400. doi: 10.3390/toxins11070400 (PMC6669508; doi:10.3390/toxins11070400)
Supplement: Supplementary file 1 [file toxins-11-00400-s001.zip › toxins-523720-si/Table_S1.pdf]

Table S1.Summary statistics of RNA sequencing reads and mapping to S. cere

Sample	Replicate	Treatment	Number Raw Reads	Q20 %	Total read count
WA	2	Control	16831498	97.17	14161892
WA	3	Control	18613634	97.23	15546563
WA	4	Control	19217926	96.81	16036387
WE	3	Control	18302888	97.26	15620111
WE	4	Control	21871922	97.23	18515199
WA	1	Patulin	22181400	97.1	18373123
WA	3	Patulin	16877538	97.18	14084672
WA	4	Patulin	19104102	97.27	16208961
WE	2	Patulin	20767430	97.1	17594079
WE	3	Patulin	19596870	97.09	16725189
WE	4	Patulin	17801742	97.27	14983188

visiae genome
Mapping percentage
83.70%
83.80%
83.60%
83.90%
84.10%
84.00%
83.60%
83.90%
84.30%
84.10%
83.30%
