# Supplementary material for: Distinct Transcriptional Changes in Response to Patulin Underlie Toxin Biosorption Differences in Saccharomyces cerevisiae
Source: Toxins (Basel). 2019 Jul 10;11(7):400. doi: 10.3390/toxins11070400 (PMC6669508; doi:10.3390/toxins11070400)
Supplement: Supplementary file 1 [file toxins-11-00400-s001.zip › toxins-523720-si/Table_S2.pdf]

Table S2 Differential gene expression data and GO-enrichment analysis of differentially

Gene ID	WA_2	WA_3	WA_4	WA_PT_1	WA_PT_3	WA_PT_4	Ratio	WE_3	WE_4
YOR383C	338.5586	451.9001	387.3183	302.6557	324.9383	303.3148	-0.33935	14855.08	12529.08
YOR382W	277.5259	192.8239	183.7786	167.4456	157.8601	134.0456	-0.50998	6615.249	5494.531
YDR036C	3.45468	5.933043	5.928341	0	0	0	#NUM!	1038.832	928.0471
YIR019C	56694.75	54082.65	58124.42	46720.9	47855.81	56035.93	-0.16534	2063.049	2475.963
YAL065C	1756.129	1586.1	1502.834	1736.241	1865.515	1720.088	0.135411	20.23699	36.87605
YOR388C	8526.15	9835.996	8319.438	8130.514	7652.182	6443.971	-0.26355	751.0174	707.6689
YKL209C	2771.805	3054.528	3175.615	3490.39	3865.844	4638.759	0.414123	233.8497	185.2582
YGL089C	134.7325	145.3595	108.6863	126.2558	114.0741	117.4122	-0.12003	17207.07	14433.46
YNL145W	10129.12	10983.05	9085.183	10317.16	11653.99	9812.722	0.073873	6.745665	22.82803
YOR133W	29096.46	26801.53	25935.5	28990.47	27838.69	26966.64	0.034186	4238.526	3476.006
YAL063C	2642.83	3004.097	3076.809	2904.778	3365.762	4024.302	0.238904	70.82948	84.2881
YKR102W	3071.21	2705.467	3469.068	2586.9	2831.111	3368.751	-0.07346	0	0
YHL047C	3.45468	0	1.976114	3.581724	2.304527	1.956869	0.530265	665.5723	672.5488
YJR161C	26.48588	25.70985	41.49839	51.03957	36.87243	43.05113	0.48313	1491.916	1521.576
YGL032C	1722.734	1778.924	1525.56	1712.064	2066.008	1710.304	0.126619	11.24277	5.268006
YFL026W	10858.06	11938.27	11004.98	12237.86	12800.49	11007.39	0.09275	40.47399	40.38805
YGR289C	0	1.977681	0	0	0	0	#NUM!	2132.754	1882.434
YOL158C	0	0	1.976114	1.790862	0	1.956869	0.923352	2100.15	2141.445
YIL015W	1955.349	2455.291	2062.075	2593.168	2639.836	2411.842	0.240117	40.47399	52.68006
YGR234W	2890.415	3558.837	2716.168	2551.978	2548.807	2527.297	-0.26488	23660.42	20705.02
YKL178C	42.60772	38.56478	34.58199	29.54922	38.02469	28.37461	-0.27074	4355.451	4002.807
YJR004C	397.2882	389.6031	259.8589	282.0608	303.0453	368.8699	-0.13389	15703.91	17069.22
YIL014C-/	710.5125	648.6793	588.8819	696.6453	730.535	717.1926	0.138508	2.248555	0
YLR040C	318.9821	254.132	304.3215	256.9887	262.7161	219.1694	-0.24796	7258.335	6542.864
YKR097W	1780.312	2140.84	1896.081	1530.292	1482.963	1174.122	-0.47429	50.59249	71.11809
YOL159C-/	0	0	0	0	0	0	#DIV/0!	377.7572	378.4185
YFL062W	130.1263	146.3484	120.5429	136.1055	102.5514	120.3475	-0.1452	2029.321	1741.076
YDR037W	2117.719	1969.77	1886.2	2176.793	2139.753	1966.654	0.072877	21746.9	20316.94
YEL073C	1166.53	1003.673	1142.194	954.5295	1026.667	1098.782	-0.10496	47.21965	35.12004
YFL059W	4094.947	4052.268	3991.75	4257.775	3913.087	3595.748	-0.04495	51.71676	59.70407
YCL049C	2862.778	2638.226	2546.222	2794.64	2766.585	2655.472	0.030067	97.81214	168.5762
YLR237W	16960.17	23002.41	18706.88	20975.47	21099.1	16786.03	0.004692	1785.353	1762.148
YPL187W	16.12184	67.24115	17.78502	36.71267	36.87243	13.69809	-0.21269	52034.93	46566.54
YDR461W	947.7338	1157.932	759.8157	913.3396	1107.325	865.9147	0.010583	6.745665	4.390005
YGR287C	65.63892	57.35275	48.41478	53.72586	66.83128	76.31791	0.199859	1459.312	1475.92
YAL064W-I	593.0534	615.0587	677.807	651.8738	707.4898	672.1846	0.107312	25.85838	22.82803
YCR097W	717.4218	524.0854	543.4313	496.9642	614.1564	588.0393	-0.07105	0	0
YDR380W	3990.155	4749.401	4055.973	4829.955	4930.535	4251.299	0.131001	773.5029	748.9349
YJL218W	461.7755	378.7259	383.366	323.2506	301.893	351.2581	-0.3259	0	0
YGR288W	0	0	0	0	0	0	#DIV/0!	277.6965	262.5223
YCL026C-I	403.046	378.7259	403.1272	463.8333	455.1441	348.3228	0.096994	2798.327	2443.477
YJR153W	383.4695	391.5808	382.378	392.1988	338.7655	289.6167	-0.18153	0	0
YNL333W	470.988	443.0005	381.3899	386.8262	414.8148	363.9777	-0.15228	8.99422	3.512004
YPR192W	1824.071	2658.003	1912.878	1568.795	1433.416	1076.278	-0.6489	35.97688	28.09603
YJL048C	4504.902	5724.397	5033.161	4562.221	4223.046	4343.272	-0.21728	673.4422	679.5728
YDR385W	13407.61	13209.92	14025.47	14470.17	14473.58	14959.29	0.111314	49333.3	45224.96
YLR411W	6432.614	6115.978	6297.874	4673.255	4696.626	4446.986	-0.44786	1915.769	1671.714
YGR295C	435.2897	496.3979	472.2912	411.0028	382.5515	311.1422	-0.34587	2.248555	4.390005
YFR055W	3698.811	4069.078	3999.654	3527.103	3554.733	3632.928	-0.13521	1095.046	1129.109
YMR303C	8092.012	9139.852	7691.034	7857.407	8444.939	8860.705	0.013835	36661.56	32444.77

YDR539W	300.5571	368.8375	258.8709	256.9887	244.2799	273.9617	-0.25991	1532.39	1454.848
YJL222W	371.9539	328.295	261.8351	299.074	269.6297	280.8108	-0.17952	0	1.756002
YEL077C	362.7414	531.0073	425.8525	358.1724	409.0535	409.9641	-0.16475	6.745665	5.268006
YCL054W-/	315.5274	287.7526	261.8351	320.5643	337.6132	249.5009	0.069289	1.124277	0
YBR297W	87.51856	137.4488	132.3996	62.68017	108.3128	98.82191	-0.40544	1304.162	1146.669
YDR038C	36.84992	31.64289	23.71336	16.11776	18.43622	19.56869	-0.76863	472.1965	433.7325
YGR052W	750.8171	713.9428	679.7831	325.9369	407.9013	366.913	-0.96218	2010.208	2015.89
YLL026W	4016.641	3957.339	3832.672	3262.951	3014.321	3349.182	-0.29452	13223.75	13583.55
YBR296C	697.8453	1025.428	787.4813	357.277	444.7737	348.3228	-1.12602	17.98844	9.658012
YPR182W	1316.233	1110.468	1101.683	1147.047	1099.259	962.7798	-0.13685	175.3873	167.6982
YDL168W	1552.303	1492.16	1411.933	1537.455	1425.35	1321.865	-0.05669	5634.879	5218.838
YDL222C	1307.021	1827.377	1572.986	881.9996	852.675	734.8045	-0.93072	107.9306	117.6521
YMR058W	1938.075	2070.632	1996.863	2023.674	2127.078	2211.262	0.083183	8152.136	8736.111
YML054C	1772.251	2131.94	1912.878	1619.835	1586.667	1433.407	-0.3262	311.4249	247.5963
YLR136C	96.73103	80.09608	81.02066	60.88931	62.22223	68.49043	-0.42841	720.6619	682.2068
YHR043C	207.2808	183.9243	189.7069	223.8578	191.2757	170.2476	0.011057	1245.699	1211.641
YGR256W	1422.177	1835.288	1418.85	1307.329	1314.733	1171.186	-0.30194	215.8613	224.7683
YBR118W	40671.95	36844.19	38868.18	43067.55	38614.65	36092.5	0.017133	9767.723	9109.261
YPR080W	41621.98	39627.78	41299.79	45290.01	40923.79	40800.73	0.051628	133061.6	121791.9
YAR050W	772.6967	966.0971	823.0513	875.7315	865.3499	771.985	-0.02773	109.0549	130.8222
YBR298C	41.45616	42.52014	18.77308	36.71267	35.72017	22.504	-0.11409	673.4422	582.9927
YDR033W	12128.23	15008.62	13523.53	16415.94	16971.69	15244.01	0.258271	3991.185	4071.291
YOL159C	0	0	0	0	0	0	#DIV/0!	192.2514	205.4523
YBR299W	152.0059	87.01796	112.6385	103.87	64.52675	45.008	-0.7206	1986.598	1930.724
YPL189C-/	1220.654	1238.028	1228.155	1272.407	1238.683	1155.531	-0.00793	287.815	302.9104
YFL060C	138.1872	187.8797	166.9816	171.9228	201.6461	203.5144	0.22705	0	1.756002
YIL117C	114.0044	88.99564	106.7101	94.91569	108.3128	101.7572	-0.02218	540.7775	553.1407
YHR216W	305.1634	427.1791	356.6885	389.5125	374.4856	406.0504	0.103523	1644.818	1563.72
YDR070C	2784.472	2913.124	2613.41	2462.435	2350.617	2071.346	-0.27169	720.6619	710.3029
YPL240C	3855.423	3850.545	3750.664	3453.677	2931.358	2891.275	-0.30456	12223.14	11766.97
YFR015C	2888.112	2905.213	2918.72	1935.922	2008.395	1964.697	-0.56009	5957.546	6138.106
YOR161C	1348.477	1636.531	1430.706	880.2087	990.9466	910.9227	-0.66648	285.5665	292.3744
YKR059W	13103.6	11861.14	12248.94	12898.68	12485.93	12725.52	0.034341	4997.413	4470.781
YDR171W	3286.552	3210.765	3164.746	2568.096	2136.296	2104.613	-0.50489	10909.99	10247.15
YNR060W	447.9568	500.3533	387.3183	335.7866	331.8519	334.6247	-0.41426	1657.185	1635.716
YNL335W	130.1263	155.2479	143.2682	128.9421	134.8148	108.6063	-0.20306	0	0
YNL034W	16.12184	33.62057	26.67753	23.28121	20.74074	23.48243	-0.17897	267.578	271.3023
YMR015C	28814.33	35607.16	30080.4	33498.07	33218.6	31015.4	0.048489	8816.584	8861.665
YPL095C	1949.591	2371.239	2028.481	2390.801	2205.432	1881.53	0.028896	519.4162	457.4386
YCR021C	82.91232	85.04028	81.02066	78.79793	106.0082	98.82191	0.18801	813.9769	743.6669
YGR079W	4174.405	4577.342	4475.897	5051.126	4840.659	4890.217	0.160285	1553.751	1411.826
YJL034W	8596.395	8086.737	8718.613	9211.299	9001.482	9768.692	0.139544	22944.25	22637.5
YDR119W-/	2684.286	2686.679	2384.181	2069.341	2183.539	2030.252	-0.30367	474.4451	418.8065
YOR009W	4040.824	3981.072	3652.846	4276.579	4535.309	3885.364	0.121126	1012.974	787.567
YGL253W	11102.19	10370.96	11929.8	10373.57	10411.85	11432.03	-0.05213	30180.1	28036.33
YGR292W	5.7578	7.910723	10.86863	3.581724	2.304527	4.892174	-1.18682	381.1301	259.8883
YJL116C	1505.089	2251.59	1722.183	2038.896	1965.761	1764.118	0.074389	272.0751	287.9844
YKR046C	13325.85	13586.67	12712.34	13441.32	13087.41	11398.76	-0.06316	4548.827	4492.731
YFR020W	2772.956	2464.19	2612.422	2568.992	2598.354	2431.41	-0.04685	661.0752	516.2646
YJL138C	7223.735	6598.532	7485.519	7924.565	7524.28	7960.545	0.135707	18936.21	18164.96
YKR039W	2896.173	4500.213	3048.155	2698.829	2689.383	2313.02	-0.43959	480.0665	483.7786
YER188W	0	0	0	0	0	0	#DIV/0!	157.3988	139.6022
YHR092C	6574.256	6856.62	6769.177	7582.51	7955.227	7014.398	0.158905	17789.44	17172.82
YPR193C	404.1975	473.6546	373.4855	333.9958	366.4198	334.6247	-0.27378	34.8526	40.38805

YHR021W-/	277.5259	236.3329	234.1695	162.073	201.6461	161.4417	-0.51033	15.73988	9.658012
YKR013W	9503.824	8894.62	9378.635	10290.29	11163.13	10207.03	0.188787	3868.639	3732.383
YGR043C	2040.564	2539.342	2050.218	1481.043	1530.206	1365.895	-0.59905	538.5289	528.5566
YKR104W	178.4918	123.6051	129.4354	157.5959	134.8148	117.4122	-0.07447	0	0
YHR112C	643.722	743.608	618.5236	660.8281	641.8108	645.7669	-0.04192	1645.942	1694.542
YLR205C	3801.299	4659.416	3685.452	3759.019	3560.494	3138.819	-0.21585	1045.578	973.7032
YFR057W	137.0356	118.6609	121.531	102.9746	145.1852	115.4553	-0.05302	1.124277	1.756002
YMR321C	472.1396	419.2683	335.9393	424.4343	376.7902	408.0073	-0.02145	56.21387	46.53406
YLL052C	606.8721	770.3067	678.795	933.0391	899.9178	875.6991	0.397755	120.2977	86.04411
YNL160W	21238.22	25097.76	21774.8	21868.22	21676.38	19582.39	-0.10963	7822.723	7780.846
YDL039C	1768.796	2337.619	2104.561	2094.413	2002.634	2479.354	0.082478	268.7023	302.0324
YFL014W	5892.532	5589.915	5571.652	3925.57	3865.844	3296.347	-0.62115	646.4596	249.3523
YJR048W	17006.24	17185.06	15559.92	17766.25	17933.83	15119.75	0.03066	6172.283	5189.864
YHR219W	2.30312	9.888404	2.96417	7.163448	2.304527	5.870608	0.017305	140.5347	158.0402
YKR103W	111.7013	138.4377	165.0055	142.3735	133.6626	208.4066	0.222713	0	0
YOL083W	465.2302	429.1567	510.8254	377.8719	440.1646	395.2876	-0.21182	82.07226	94.82412
YER186C	719.725	770.3067	682.7473	635.756	631.4404	752.4163	-0.10546	152.9017	166.8202
YLL053C	939.6729	888.9675	829.9677	1309.12	1123.457	930.4914	0.339107	165.2688	115.8961
YOR010C	181.9465	179.969	188.7189	236.3938	163.6214	167.3123	0.043087	745.396	674.3048
YEL065W	366.1961	421.246	345.8199	222.0669	270.7819	279.8323	-0.55254	1569.491	1758.636
YMR011W	7799.515	8274.617	8063.532	4396.566	4345.186	4068.332	-0.91401	13671.21	13566.87
YML123C	4607.391	6722.137	5044.03	3657.836	3358.848	3089.897	-0.69607	1550.379	1367.926
YGL184C	3332.614	4516.034	3475.984	3293.395	2854.157	2683.846	-0.35875	619.4769	626.8928
YBR054W	1317.385	1538.636	1401.065	1635.057	1429.959	1330.671	0.046223	365.3902	375.7845
YLR046C	952.3401	1109.479	943.5943	936.6209	996.7079	850.2598	-0.11062	320.4191	280.0823
YOL058W	2529.977	2783.586	2635.148	1989.648	2128.231	1937.301	-0.39255	840.9596	888.5371
YDR343C	191.1589	137.4488	153.1488	102.0791	78.35392	76.31791	-0.90793	828.5925	656.7448
YNL111C	20919.24	24630.04	21142.44	25861.84	26174.82	20966.88	0.130459	7540.529	7114.443
YJL186W	761.1811	877.1015	842.8125	899.0128	767.4075	894.2893	0.045567	2224.945	2384.651
YLR420W	3506.5	3547.959	3509.578	3761.706	3709.136	3730.772	0.084545	7536.032	6987.133
YBR177C	4012.035	4515.045	4220.979	4205.84	3872.758	3809.046	-0.10081	1677.422	1476.798
YNL336W	97.88259	82.07376	55.33118	57.30759	78.35392	72.40417	-0.17739	444.0896	462.7066
YDR133C	42232.31	39483.41	38554.97	37559.75	37574.16	34656.16	-0.13154	17517.37	15242.98
YLR056W	27699.62	33784.72	28583.5	31854.06	30941.73	26217.16	-0.017	10923.48	10174.28
YHR022C	337.4071	363.8933	448.5778	277.5836	241.9753	274.9402	-0.53336	31.47977	43.90005
YBR069C	2582.949	3064.417	2881.174	2988.053	3044.28	3549.761	0.168042	1246.824	1330.172
YDL248W	177.3402	160.1922	175.8741	171.0273	164.7737	170.2476	-0.02083	29.23121	22.82803
YDR258C	1824.071	1868.908	1908.926	1548.2	1395.391	1535.164	-0.32282	4705.101	4720.134
YER187W	449.1084	451.9001	403.1272	433.3886	492.0165	392.3523	0.014991	102.3093	93.06811
YNR034W-/	5160.14	4710.836	5166.549	4046.453	4016.79	3568.351	-0.37052	15426.21	14854.9
YBR092C	3962.518	4321.233	3940.371	4812.046	4498.437	4265.975	0.151376	1950.621	1670.836
YJR009C	78961.31	67508.14	73232.8	77641.03	72929.06	70455.13	0.008661	189353.1	161408.2
YNL052W	9218.237	9974.433	8963.652	9493.36	9170.865	7778.556	-0.09059	3860.769	3414.546
YGR157W	3210.549	3436.221	3272.444	3800.209	3372.675	3496.926	0.105237	1509.905	1300.32
YBR066C	718.5734	655.6012	658.0458	359.9633	341.07	364.9561	-0.93086	1231.084	1191.447
YOL165C	214.1901	131.5158	100.7818	138.7918	140.5761	178.0751	0.034972	0	0
YFL027C	861.3668	1044.215	930.7495	861.4046	899.9178	907.9874	-0.08756	287.815	194.9162
YIL082W	132.4294	167.114	86.949	121.7786	114.0741	84.14539	-0.27238	0	0
YGR088W	4430.051	4867.073	4738.721	2718.529	2776.955	2749.402	-0.76754	1443.572	1371.438
YJL078C	650.6314	682.2999	725.2337	683.2139	633.7449	688.818	-0.0372	1541.384	1669.958
YML075C	14632.87	17569.72	15876.1	18230.98	18826.83	20132.27	0.250367	7315.674	7587.685
YML131W	291.3447	346.0942	320.1304	372.4993	251.1934	275.9186	-0.09007	1164.751	1027.261
YHR137W	1657.095	2056.788	1753.801	1812.352	2002.634	1662.361	0.002547	644.211	698.0109
YKL163W	1573.031	2293.121	1886.2	1742.509	1488.724	1325.779	-0.33606	366.5145	356.4684

YJL185C	650.6314	630.8802	516.7537	551.5855	530.0412	613.4786	-0.08523	154.026	181.7462
YPL277C	66.79048	85.04028	83.98483	111.9289	110.6173	117.4122	0.527699	338.4075	403.0025
YDR502C	22212.44	27143.67	23384.34	26542.37	25870.62	24118.42	0.073294	8632.202	8903.809
YLR036C	62.18424	43.50898	34.58199	25.9675	24.19753	15.65496	-1.09166	273.1994	289.7404
YER079W	525.1113	448.9336	467.3509	385.9308	331.8519	328.7541	-0.46184	1282.801	1403.046
YLR228C	452.5631	440.034	496.0045	412.7937	404.4445	485.3036	-0.0923	1077.058	1322.27
YPL028W	25980.34	31007.07	26250.69	29740.85	28958.69	25486.27	0.016333	12155.69	11197.15
YLR460C	78.30608	47.46434	43.4745	89.5431	96.79013	82.18852	0.665926	0	0
YML066C	70.24516	98.88404	86.949	89.5431	95.63787	86.10225	0.083215	414.8584	343.2984
YHL040C	325.8915	494.4202	386.3302	393.9897	409.0535	445.1878	0.048887	1573.988	1627.814
YPR010C-/	3117.273	3236.475	2993.812	2910.151	3165.268	2558.607	-0.11456	1280.552	1268.712
YHL044W	13.81872	23.73217	25.68948	11.6406	4.609054	7.827478	-1.39318	125.9191	110.6281
YMR195W	895.9136	779.2063	827.0036	385.9308	369.8766	303.3148	-1.24028	2020.327	1727.028
YCL069W	81.76076	79.10723	52.36701	79.69336	77.20165	67.512	0.073673	0	1.756002
YDR453C	1236.775	1300.325	1292.378	1183.76	1134.98	1138.898	-0.14736	538.5289	523.2886
YLR367W	6408.431	5610.681	5632.912	5443.325	5745.186	5017.413	-0.12331	2841.049	2353.921
YBR145W	275.2228	315.4401	262.8231	290.1197	271.9342	238.7381	-0.09194	836.4624	742.7889
YDR492W	7395.318	9368.274	7719.688	9394.862	9087.902	7537.861	0.087859	2600.454	2519.863
YOR065W	6571.953	7826.672	6851.186	6743.491	7062.223	6287.421	-0.08075	3023.182	3197.68
YKR018C	1044.465	929.51	1042.4	981.3924	913.7449	1004.852	-0.05677	2102.399	2208.173
YOR317W	5983.505	7665.491	6798.819	6353.083	5957.202	5881.371	-0.16867	2842.173	2921.11
YKR105C	57.578	63.28579	51.37895	50.14414	71.44034	54.79234	0.034218	0	0
YHL021C	1430.237	1347.79	1522.596	781.7113	738.6009	818.9499	-0.87849	2291.278	2341.629
YPR151C	153.1575	181.9466	165.0055	105.6609	126.749	113.4984	-0.53185	16.86416	17.56002
YLR413W	7298.587	7427.18	7685.106	7244.037	7584.198	8557.39	0.061423	14524.54	15465.99
YMR244W	251.0401	353.016	280.6081	359.9633	334.1564	277.8755	0.135819	31.47977	45.65606
YOR209C	2401.002	2310.92	2438.524	2442.736	2418.601	2561.542	0.053945	4897.353	4338.203
YNL074C	811.8498	751.5187	862.5736	748.5803	772.0165	887.4403	-0.01069	1768.488	1880.678
YGR182C	2870.839	2450.347	2424.691	2510.789	2492.346	2186.802	-0.10745	1143.39	1098.379
YPL117C	8651.67	9590.763	8421.208	9179.063	9047.573	8838.201	0.021546	4545.454	4239.867
YLL056C	1180.349	1490.183	1092.791	1220.472	1087.737	816.993	-0.26806	288.9393	199.3062
YNL280C	4352.897	5350.616	4350.414	4950.838	4799.177	4261.083	-0.0044	2028.197	1857.85
YPR009W	659.8438	597.2596	616.5475	638.4423	603.7861	544.0097	-0.06893	1326.647	1399.534
YER103W	631.0548	753.4964	736.1023	682.3184	581.8931	646.7453	-0.15021	2881.523	2605.029
YGR049W	19675.55	21940.39	19416.3	22544.27	22103.87	17599.11	0.028438	7194.252	6999.425
YBR214W	2363.001	2186.326	2239.925	1507.01	1570.535	1361.003	-0.61316	3710.116	3750.821
YEL039C	5092.198	4635.684	4662.64	3664.999	3618.107	3379.514	-0.43256	2079.913	1756.002
YJL079C	3879.605	4634.695	3605.419	3775.137	3841.646	2995.967	-0.19156	1249.072	1180.911
YJL196C	6295.578	7135.473	6485.605	6493.666	6411.194	5529.135	-0.11161	2509.387	2131.787
YCR005C	663.2985	856.3358	675.8309	470.1013	364.1153	293.5304	-0.96108	1862.928	1612.01
YKL104C	2280.089	2256.534	2318.969	2466.912	2386.338	2756.251	0.150521	4521.844	4548.924
YLL012W	4473.81	5993.362	4844.443	5499.737	5513.581	5017.413	0.066214	2070.919	1878.922
YOR142W	4280.348	4211.471	4272.358	3795.732	3759.836	3810.025	-0.16743	2302.52	2123.885
YOR154W	1020.282	984.8851	1120.456	1255.394	1268.642	1393.291	0.325726	465.4509	524.1666
YNR043W	12683.28	13367.14	12105.67	13282.82	13680.82	13326.28	0.078506	6990.757	6394.482
YGL261C	1.15156	5.933043	1.976114	6.268017	1.152263	7.827478	0.7509	75.32659	64.97208
YGR183C	4532.54	4684.137	4182.445	4228.225	4407.408	3848.184	-0.10208	2079.913	1875.41
YOR049C	725.4828	950.2757	772.6604	683.2139	512.7572	427.576	-0.5927	146.1561	129.9442
YGL187C	5525.185	5843.058	5083.552	5527.496	5316.544	4720.947	-0.07994	2809.569	2757.801
YHR211W	90.97324	62.29695	60.27147	35.81724	53.00412	55.77078	-0.56253	243.9682	262.5223
YKL220C	102.4888	97.8952	97.81763	102.9746	102.5514	108.6063	0.075084	373.2601	363.4924
YGR180C	10486.1	9739.089	9735.324	9558.726	9457.779	9440.917	-0.07426	18725.97	16188.58
YLR038C	4108.766	3958.328	4022.379	3836.027	4271.441	3771.866	-0.0253	2168.731	1755.124
YDR441C	315.5274	383.6701	369.5333	312.5054	276.5432	254.393	-0.34154	1025.341	874.4891

YGL263W	528.566	432.1233	533.5507	537.2586	487.4074	485.3036	0.015108	145.0318	153.6502
YMR256C	2075.111	1996.469	1766.646	1857.124	1978.436	1800.32	-0.05089	983.7428	863.0751
YPL279C	127.8232	142.393	111.6504	203.2628	187.8189	153.6143	0.512383	17.98844	24.58403
YDL181W	2558.766	2128.973	2279.447	2160.675	1998.025	1905.991	-0.20014	1223.214	1050.089
YPR127W	1741.159	1975.703	1636.222	1874.137	1756.05	1582.129	-0.03845	849.9538	777.909
YHL035C	232.6151	248.1989	177.8502	181.7725	221.2346	265.1558	0.020657	894.9249	1033.407
YDR077W	39617.12	44035.04	42135.68	37306.34	38162.97	39054.22	-0.13535	71156.65	74148.95
YCR102C	82.91232	93.93984	102.7579	117.3015	137.1194	98.82191	0.337244	365.3902	289.7404
YLR231C	1009.918	1136.178	1100.695	1032.432	1007.078	822.8636	-0.1818	338.4075	346.8104
YMR006C	4887.22	5162.736	5178.406	5678.824	5757.861	5169.071	0.124923	8502.911	8641.287
YML125C	2250.148	2103.264	2110.489	2546.606	2422.058	2129.074	0.134954	4900.726	4226.697
YLR010C	515.8989	421.246	513.7895	469.2059	453.9918	429.5328	-0.10111	96.68786	64.97208
YDL174C	4464.598	5442.578	4463.053	4404.625	4403.951	3666.195	-0.20407	2043.936	2123.007
YBR300C	6.90936	2.966521	0	2.686293	0	0	-1.87829	65.20809	60.58207
YOR238W	780.7576	701.0879	636.3086	721.7174	679.8354	635.9826	-0.05598	251.8382	280.9603
YDR497C	6201.15	6329.568	6579.47	6653.053	6793.745	6571.168	0.066953	3296.382	3472.494
YHR205W	1490.119	1336.912	1263.725	1457.762	1335.473	1367.852	0.024593	2803.948	2678.781
YJR155W	839.4872	1014.55	758.8276	757.5346	588.8066	601.7373	-0.42358	229.3526	177.3562
YEL040W	5549.367	5557.283	5740.61	5792.543	6040.165	5550.66	0.045194	9853.168	10344.61
YDL223C	976.5228	1007.628	1132.313	725.2991	807.7367	1060.623	-0.26492	282.1936	327.4944
YMR175W	87.51856	93.93984	79.04455	98.49741	67.98354	73.3826	-0.11909	5.621387	8.780011
YMR134W	2060.141	2100.297	1827.905	1895.627	1991.111	1652.576	-0.11245	767.8815	813.907
YGL158W	48.36552	42.52014	51.37895	17.90862	23.04527	52.83547	-0.60108	299.0578	353.8344
YGL039W	458.3209	538.918	526.6343	631.2789	493.1688	519.5488	0.109464	1192.858	1136.133
YOL143C	4165.192	3502.473	4020.403	3891.543	3855.474	3420.608	-0.06571	9763.226	8095.17
YML126C	22101.89	25522.96	22343.92	25852.88	27108.15	25452.02	0.164383	12514.33	12046.17
YPL278C	39.15304	44.49782	38.53422	40.2944	24.19753	21.52556	-0.50637	266.4538	192.2822
YLR265C	718.5734	638.7909	705.4726	624.1154	720.1647	658.4866	-0.04264	281.0694	265.1563
YDL110C	5562.035	5832.181	5505.453	4532.672	4507.655	4117.253	-0.3611	2723	2573.421
YJL026W	6650.259	6188.163	6420.393	6175.788	6073.581	5870.608	-0.08794	11203.43	9734.398
YDR040C	80.6092	74.16303	60.27147	77.00707	54.15638	62.61982	-0.15019	2.248555	7.024009
YNR014W	422.6225	385.6478	337.9154	246.2435	203.9506	146.7652	-0.94114	972.5	989.5072
YGL101W	2325	2276.311	2191.51	2239.473	2268.807	2454.893	0.035734	1162.503	905.2191
YHR140W	238.3729	160.1922	169.9458	141.4781	134.8148	154.5927	-0.39988	23.60983	22.82803
YFL067W	41.45616	49.44202	46.43867	34.02638	62.22223	58.70608	0.174128	0	0
YML008C	22279.23	23948.73	21675	24210.66	23808.07	21979.56	0.043845	13023.63	12110.27
YNL194C	153.1575	213.5895	198.5994	127.1512	81.81071	90.99443	-0.91438	680.1879	604.9427
YPL091W	2571.433	2741.066	2578.828	2514.37	2538.436	2600.679	-0.04415	4986.171	4481.317
YCR012W	26910.8	28801.96	31184.06	28623.35	28122.14	30958.65	0.013342	57339.28	52392.96
YBR230C	1645.579	1418.986	1491.966	898.1173	955.2264	881.5697	-0.73644	738.6503	731.3749
YFL034C-/	3215.155	3254.274	2979.979	3438.455	3371.523	2590.895	-0.00743	1468.306	1388.998
YHR120W	1081.315	1172.765	1146.146	1227.636	1282.469	1394.269	0.199461	590.2457	568.0667
YLL063C	33.39524	51.4197	27.66559	34.92181	29.95885	29.35304	-0.25536	179.8844	198.4282
YLR030W	209.5839	212.6007	225.277	203.2628	207.4074	188.8379	-0.11102	52.84104	47.41206
YOR032C	259.101	284.786	236.1456	241.7664	186.6667	234.8243	-0.23397	503.6763	575.9687
YDL234C	485.9583	421.246	480.1956	486.219	410.2058	372.7836	-0.12845	1035.46	1146.669
YOL118C	194.6136	209.6342	140.3041	182.6679	164.7737	131.1103	-0.18639	31.47977	24.58403
YIR030C	116.3076	89.98448	108.6863	93.12483	107.1605	69.46886	-0.22361	13.49133	8.780011
YLR395C	4002.822	4580.309	4187.385	4161.963	4431.605	3939.178	-0.02711	2658.916	2330.215
YPL134C	1134.287	1191.553	1063.149	989.4513	988.6421	862.001	-0.25492	523.9133	446.0245
YLR216C	2212.147	1881.763	2035.397	2000.393	1955.391	1853.155	-0.07745	4167.697	3829.841
YJL141C	596.508	507.2751	632.3564	429.8069	412.5103	444.2094	-0.4324	1184.988	1259.054
YHR027C	2846.656	3041.673	2981.955	2818.817	3069.63	3397.125	0.06601	5164.931	5472.581
YOL155C	6766.566	6743.892	6749.416	7008.539	7308.807	7457.629	0.104046	5306.59	4987.924

YDR534C	101.3373	145.3595	130.4235	62.68017	91.02881	64.57669	-0.78881	558.7659	586.5047
YIR016W	1319.688	1221.218	1209.382	955.4249	876.8725	805.2518	-0.5078	2700.515	2283.681
YFR047C	529.7176	490.4649	493.0404	422.6434	396.3786	335.6031	-0.3902	1163.627	966.6792
YBR117C	447.9568	638.7909	528.6104	412.7937	462.0577	453.9937	-0.28168	143.9075	134.3342
YOR178C	259.101	233.3663	203.5397	146.8507	140.5761	186.881	-0.55328	543.026	655.8668
YMR041C	584.9924	578.4717	572.0849	556.0627	580.7408	547.9234	-0.04288	222.6069	228.2803
YOL091W	35.69836	42.52014	55.33118	62.68017	41.48148	64.57669	0.337411	186.6301	189.6482
YJL036W	530.8691	562.6502	581.9655	516.6637	448.2305	503.8939	-0.18995	945.5174	1169.497
YFL061W	0	0	0	1.790862	0	0	#DIV/0!	42.72254	34.24204
YCL030C	1936.924	2095.353	1999.827	1705.796	1858.601	1862.94	-0.15242	2893.89	2701.609
YGR060W	29297.99	35613.09	30469.7	35032.84	35301.9	30917.56	0.086184	16824.81	16421.25
YLR437C	1347.325	1191.553	1261.749	1333.297	1199.506	1014.637	-0.09946	2775.841	2545.325
YKL150W	5299.479	4954.091	5011.424	4783.393	4923.622	4386.323	-0.11521	3304.252	2847.357
YOL016C	1022.585	1170.787	921.857	842.6006	536.9548	655.5513	-0.61423	2185.595	2401.333
YFR007W	753.1202	874.1349	752.8993	778.1296	717.8601	741.6535	-0.08908	332.7861	320.4704
YML133C	1.15156	0	2.96417	3.581724	4.609054	0	0.992852	35.97688	51.80206
YHR001W-/	2445.913	2840.939	2192.498	2191.12	2336.79	1969.589	-0.20303	1134.396	880.6351
YCR100C	29.94056	26.69869	21.73725	22.38578	35.72017	29.35304	0.158185	84.32081	98.33612
YHR051W	3819.724	4091.822	3683.476	3964.073	3846.255	3434.306	-0.04427	2391.338	1976.38
YGL051W	9.21248	16.81029	12.84474	8.058879	16.13169	14.67652	-1.5E-05	131.5405	84.2881
YEL070W	39.15304	49.44202	46.43867	13.43147	19.58848	41.09426	-0.8655	186.6301	268.6683
YCR091W	203.8261	201.7234	185.7547	181.7725	195.8848	203.5144	-0.02494	499.1792	676.9388
YNR075C-/	0	3.955362	0	7.163448	1.152263	1.956869	1.376917	44.9711	34.24204
YFL058W	66.79048	82.07376	49.40284	42.98069	41.48148	54.79234	-0.50972	2.248555	3.512004
YLR031W	195.7652	200.7346	177.8502	169.2365	137.1194	157.528	-0.30817	52.84104	45.65606
YLR149C	163.5215	173.0471	195.6353	93.12483	108.3128	86.10225	-0.88822	365.3902	478.5106
YMR145C	5138.26	6100.157	5503.477	5457.652	5685.268	5299.202	-0.02607	3695.5	3724.481
YJR145C	42965.85	38099.03	40922.35	43802.69	41362.8	40412.29	0.041851	27540.3	24370.68
YML051W	810.6982	865.2354	877.3945	904.3853	816.9548	800.3596	-0.01798	1470.555	1495.236
YJR091C	794.5764	867.2131	1027.579	789.7702	770.8643	1239.677	0.05832	2289.029	3098.466
YML004C	2958.357	2948.722	3069.893	2574.364	2636.379	2499.901	-0.21938	5595.529	4694.672
YDL124W	1716.976	1659.274	1640.174	1399.559	1370.041	1330.671	-0.29094	3309.873	2885.99
YER053C-/	1546.545	2280.266	1562.118	2074.714	2098.272	1605.611	0.100721	491.3093	470.6086
YBR151W	2498.885	2372.228	2393.074	2333.493	2264.198	2179.953	-0.10002	4039.529	3988.759
YIR027C	224.5542	312.4736	216.3844	196.0994	238.5185	120.3475	-0.44104	47.21965	24.58403
YPL247C	884.398	982.9074	874.4303	719.9265	666.0083	594.8883	-0.46899	1425.584	1619.034
YHL043W	158.9153	265.0092	208.48	205.0537	229.3004	189.8163	-0.01891	555.3931	572.4567
YGL001C	8940.711	9790.509	8496.301	9215.776	9544.198	7919.451	-0.02934	4708.474	4083.583
YBR301W	0	0	0	0	0	0	#DIV/0!	24.7341	26.34003
YBR105C	1260.958	1389.321	1257.796	1036.909	883.7861	912.8796	-0.46383	474.4451	489.0466
YLR300W	14442.86	16158.64	15403.81	17843.25	17364.61	16576.64	0.17072	8398.353	8475.344
YNL098C	7170.764	6916.939	6974.693	6591.268	6244.116	5874.522	-0.17087	4276.752	4086.217
YGL055W	35833.09	45093.1	39759.41	42853.54	45631.94	47391.46	0.171047	21947.02	21123.83
YOR107W	631.0548	699.1102	734.1262	835.4371	783.5392	842.4323	0.253838	248.4653	309.9344
YKL110C	1305.869	1316.147	1298.307	1477.461	1594.733	1419.709	0.196354	2314.887	2563.763
YDL210W	100.1857	134.4823	92.87734	55.51672	69.13581	51.85704	-0.89195	8.99422	10.53601
YJL214W	123.2169	200.7346	183.7786	175.5045	163.6214	209.385	0.111459	25.85838	44.77805
YHL032C	806.092	826.6706	778.5888	683.2139	699.4239	708.3867	-0.20563	1308.659	1408.314
YMR090W	4241.195	3615.201	3856.386	3707.98	3805.926	3524.322	-0.08558	2475.659	2133.543
YCL027W	345.468	315.4401	340.8796	285.6425	358.3539	302.3363	-0.08216	693.6792	834.979
YDL079C	335.1039	353.016	331.9871	207.74	226.9959	203.5144	-0.67653	658.8266	619.8688
YER134C	183.098	187.8797	201.5636	186.2497	191.2757	173.1829	-0.05609	476.6937	462.7066
YGR244C	5293.721	5078.684	4928.427	4555.953	4377.449	4541.894	-0.18329	3145.728	2957.108
YNR018W	9970.206	9615.484	9666.16	12152.79	11012.18	9877.298	0.175785	6274.593	6008.161

YDR047W	4318.35	4784.999	4434.399	4553.267	4573.334	4186.722	-0.02412	2679.153	2592.737
YGL255W	10709.51	12118.24	10902.22	10426.4	10688.4	9031.931	-0.16203	5756.301	5472.581
YNL134C	4394.353	4843.34	4224.931	4488.796	4360.165	3827.637	-0.08679	2359.858	2263.487
YDL037C	208.4323	345.1053	462.4106	397.5714	347.9836	680.9906	0.489699	13.49133	36.87605
YNL195C	647.1767	787.117	715.3531	566.8078	533.498	455.9506	-0.46602	301.3064	274.8143
YOL151W	772.6967	880.068	863.5617	744.1032	732.8396	751.4379	-0.17532	422.7283	298.5204
YGR175C	25416.08	30286.2	27976.83	30700.75	29766.42	29309.99	0.101481	14938.27	14912.85
YLR153C	19506.27	23675.81	21425.02	23686.84	24129.55	25052.82	0.173616	12488.47	11854.77
YPL141C	323.5883	376.7482	360.6407	290.1197	379.0947	411.921	0.027153	684.685	971.9472
YGR249W	209.5839	232.3775	307.2857	168.341	197.0371	204.4929	-0.39481	498.0549	627.7708
YER143W	632.2064	663.5119	658.0458	568.5987	583.0453	573.3627	-0.17965	1119.78	1024.627
YHR190W	8787.554	9855.773	9064.433	9782.584	10093.83	9198.265	0.069473	5606.772	5523.505
YMR202W	22432.39	20910.02	21076.24	25250.26	25232.27	23694.75	0.203499	16484.16	14024.31
YHL042W	180.7949	150.3037	134.3757	131.6284	153.251	139.9162	-0.13193	422.7283	331.8844
YPL220W	6203.453	4316.288	5593.39	6333.384	5434.075	4895.109	0.048374	15269.94	12477.27
YKR091W	442.199	411.3576	414.9839	383.2445	376.7902	364.9561	-0.17326	698.1763	652.3548
YKL145W	2814.412	2995.198	3130.164	2931.641	2994.733	3398.104	0.060785	4727.587	4872.028
YHR033W	953.4916	1111.457	903.0839	505.0231	425.1852	463.7781	-1.09029	363.1416	360.8584
YMR186W	22373.66	18612.94	22197.68	21455.42	20630.13	25145.77	0.089568	43218.35	41088.69
YNR047W	1020.282	930.4988	1188.632	989.4513	977.1194	1486.242	0.137277	373.2601	392.4665
YMR220W	4356.351	5022.321	4387.96	4874.727	4872.922	4659.306	0.06559	2874.778	2607.663
YKR011C	755.4233	714.9316	770.6843	598.1479	501.2346	577.2765	-0.41858	1279.428	1247.64
YLL039C	2645.133	2649.104	2343.671	2248.427	1836.708	1881.53	-0.35625	4858.003	4289.035
YPL009C	1355.386	1257.805	1484.061	1398.663	1432.263	1580.172	0.106481	2170.98	2408.357
YNL040W	700.1484	647.6905	553.3118	653.6646	638.354	746.5457	0.100681	272.0751	244.9623
YGR095C	1630.609	1504.026	1627.33	1883.987	1765.268	1557.668	0.128874	959.0087	962.2892
YNL007C	2677.377	2964.544	2255.734	2243.055	1905.844	1660.404	-0.44306	4778.179	5117.868
YHR044C	82.91232	103.8282	91.88928	120.8832	96.79013	93.92973	0.16136	476.6937	316.9584
YHR039C	7622.175	9848.851	8366.865	9443.216	9912.923	9321.548	0.15044	4508.353	4712.232
YKL065W-/	126.6716	148.3261	112.6385	98.49741	117.5309	118.3906	-0.21305	35.97688	20.19402
YIR018W	399.5913	466.7327	380.4019	350.1135	236.214	247.544	-0.58025	857.8237	794.591
YCL050C	4503.751	3913.83	4310.892	4079.584	4219.589	4063.439	-0.04208	7138.038	6497.208
YCL035C	3147.213	2734.144	2947.374	2971.936	3068.478	2648.623	-0.02301	6565.78	5914.215
YJR150C	2321.545	2076.565	2236.961	3109.832	3249.383	2565.456	0.427687	962.3815	943.8512
YLR134W	878.6402	888.9675	800.326	1082.576	1164.938	1024.421	0.349536	601.4885	461.8286
YBR045C	47.21396	65.26347	59.28341	68.05276	59.9177	70.4473	0.20814	183.2572	201.9402
YFR033C	11368.2	10934.6	10373.61	11218.86	10868.15	9334.267	-0.05651	7136.913	6498.964
YPL223C	354.6805	535.9515	368.5452	328.6232	292.6749	201.5576	-0.61377	109.0549	69.36208
YFL054C	105.9435	127.5604	173.898	119.9878	115.2263	105.6709	-0.25717	377.7572	402.1245
YLR177W	659.8438	505.2975	629.3922	461.147	394.0741	368.8699	-0.5519	991.6127	1027.261
YOR054C	213.0386	220.5114	257.8828	170.1319	191.2757	199.6007	-0.30157	418.2312	434.6105
YDR529C	7753.453	7963.132	7862.956	8135.886	7693.663	7080.932	-0.04153	5804.645	5253.08
YER062C	12290.6	13277.16	12974.17	10234.78	9866.832	9106.292	-0.40007	7212.24	6491.062
YEL024W	3400.557	4346.943	3925.55	3615.75	3490.206	3196.546	-0.18019	2251.928	1980.77
YJL171C	1218.35	1315.158	1005.842	1173.91	1113.087	1103.674	-0.06191	2580.217	2141.445
YAR066W	6.90936	1.977681	4.940284	0.895431	5.761317	3.913739	-0.38748	24.7341	28.97404
YLL010C	701.3	993.7846	887.275	836.3326	853.8272	832.6479	-0.03366	351.8989	420.5625
YER004W	1532.726	1714.649	1437.623	1201.668	1488.724	1311.103	-0.22751	2718.503	2543.569
YMR272C	7321.618	8287.472	7044.845	7523.411	7427.49	6546.707	-0.07559	4235.153	4003.685
YIL029C	206.1292	223.4779	191.683	168.341	186.6667	143.8299	-0.3167	71.95376	85.1661
YDL021W	575.78	490.4649	499.9568	356.3815	320.3292	347.3443	-0.61298	1122.029	897.3171
YDR259C	511.2926	454.8666	500.9448	421.748	301.893	434.425	-0.34125	853.3266	964.9232
YDL247W-/	34.5468	33.62057	26.67753	21.49034	14.97943	25.4393	-0.61542	0	0
YDR490C	1400.297	1600.933	1422.802	1360.16	1386.173	1362.96	-0.10647	757.763	762.1049

YGR177C	735.8468	846.4474	893.2034	1024.373	1084.28	1282.728	0.454154	390.1243	469.7306
YNL146C-/	41.45616	32.63173	26.67753	31.34009	19.58848	32.28835	-0.27605	4.49711	0
YIL040W	986.8869	1017.517	950.5107	941.9934	967.9013	767.0928	-0.14251	507.0491	374.0285
YIL118W	3208.246	3050.573	2931.565	3070.433	3174.486	2785.604	-0.02532	1967.486	1742.832
YER053C	2011.775	2052.833	1963.269	1810.562	1931.194	1637.9	-0.16414	1053.448	993.0192
YER098W	263.7072	260.065	280.6081	204.1583	223.5391	269.0695	-0.2072	502.552	580.3587
YER021W	2237.481	2134.906	2302.172	2206.342	1964.609	2234.745	-0.05932	3494.254	3398.742
YIR034C	916.6417	983.8962	864.5497	795.1428	903.3746	796.4459	-0.14831	1229.96	1278.37
YER044C	9005.199	8239.018	8621.784	9524.7	9549.96	8573.045	0.096103	5878.847	5030.068
YOR027W	3393.647	3670.576	3525.387	3073.119	3039.671	3419.629	-0.15174	5426.887	5620.963
YML121W	1536.181	1772.991	1523.584	1511.488	1553.251	1324.801	-0.13878	801.6098	808.639
YMR322C	28.789	27.68753	13.8328	24.17664	23.04527	23.48243	0.008083	0	0
YAL044C	3002.117	3008.053	2612.422	3065.956	2817.284	2403.036	-0.0574	1620.084	1505.772
YNR074C	1304.717	1316.147	1211.358	1240.172	1336.626	1140.855	-0.04379	719.5376	687.4748
YGL162W	165.8246	219.5226	173.898	154.0141	139.4239	106.6494	-0.48317	324.9162	390.7105
YOR356W	2197.176	2849.838	2152.976	2324.539	2254.98	2125.16	-0.10283	1158.006	1162.473
YBR203W	285.5869	259.0762	297.4051	241.7664	322.6338	462.7996	0.286708	866.8179	829.711
YOL154W	308.6181	361.9156	314.2021	258.7796	253.498	205.4713	-0.45626	111.3035	68.48408
YOR377W	284.4353	305.5517	313.214	294.5968	308.8066	360.064	0.093189	583.5	634.7948
YDR516C	6494.798	7047.466	6479.677	5043.963	5131.029	4798.244	-0.4192	11484.49	11058.42
YOR267C	879.7918	920.6104	852.693	879.3133	836.5433	1004.852	0.036306	1499.786	1870.142
YGR142W	369.6507	410.3688	288.5126	376.9765	195.8848	153.6143	-0.55664	1829.199	1758.636
YHR213W	4.60624	0	0.988057	1.790862	0	0	-1.6433	21.36127	27.21803
YDL243C	72.54828	76.14071	55.33118	53.72586	41.48148	50.87861	-0.48189	163.0202	132.5782
YKL062W	700.1484	586.3824	727.2098	503.2322	577.284	652.616	-0.2165	1084.928	1213.397
YDR423C	885.5496	1203.419	875.4183	1001.987	925.2676	773.9419	-0.13413	358.6445	436.3665
YNL117W	267.1619	265.9981	217.3725	256.0933	212.0165	180.032	-0.21161	75.32659	64.97208
YMR002W	9506.127	9269.39	9602.924	9213.09	8790.618	8029.035	-0.12447	6181.278	5850.999
YMR070W	2670.467	2885.436	2595.625	2440.05	2466.996	2464.677	-0.14507	1336.766	1562.842
YIR028W	74.8514	62.29695	52.36701	60.88931	39.17696	60.66295	-0.23768	10.1185	1.756002
YJL144W	82.91232	82.07376	65.21175	42.98069	33.41564	37.18052	-1.01921	207.9913	182.6242
YLR130C	3522.622	3815.935	3698.297	4008.845	3863.539	3564.438	0.051357	2240.685	2042.23
YDR085C	217.6448	329.2839	252.9425	239.0801	256.9548	187.8595	-0.22599	675.6908	716.4489
YOR192C	42.60772	41.5313	42.48644	60.88931	43.78601	42.07269	0.212773	157.3988	212.4763
YKL043W	2145.356	1940.105	2184.594	2037.106	2061.399	2009.705	-0.03773	3138.983	3137.098
YDL042C	1066.345	994.7735	827.0036	983.1833	1024.362	1098.782	0.105079	608.2341	444.2685
YLR168C	1539.636	1760.136	1450.467	1486.416	1409.218	1388.399	-0.14903	909.5405	886.7811
YJL035C	374.257	335.2169	334.9513	308.9237	351.4404	268.0911	-0.1698	694.8035	670.7928
YPR195C	124.3685	174.0359	163.0294	103.87	162.4691	152.6358	-0.13926	32.60405	22.82803
YDL025C	560.8097	491.4537	643.225	491.5916	571.5227	628.1551	-0.00359	966.8786	1108.037
YCR039C	3.45468	3.955362	0.988057	7.163448	1.152263	0	-0.01422	71.95376	44.77805
YER020W	1267.867	1202.43	1314.116	1169.433	1114.239	1091.933	-0.16492	1747.127	2058.035
YMR278W	704.7547	698.1213	562.2043	577.553	574.9795	562.6	-0.19627	1012.974	1081.697
YER141W	4753.639	7350.051	4974.866	5185.441	5110.288	4273.803	-0.22923	2179.974	2166.029
YLR286C	543.5363	575.5051	590.858	475.4739	412.5103	452.0368	-0.35166	827.4682	896.4391
YHR042W	12951.59	13440.32	13220.2	14737	15436.87	14372.23	0.169358	8841.318	9206.719
YFR010W	2095.839	1892.641	2106.537	2054.119	1995.72	2110.484	0.015376	3120.994	3101.978
YOR355W	3559.472	3392.712	3482.9	3493.076	3356.543	3363.859	-0.03097	2011.332	2365.335
YPL170W	4202.042	4473.514	3982.857	4431.488	4067.49	3337.441	-0.09686	2223.821	1929.846
YLL029W	1932.318	2084.476	1955.364	1834.738	1942.716	1828.694	-0.09124	2851.168	3025.592
YHL033C	29803.52	28261.06	28912.52	32855.16	30129.39	27981.28	0.064689	21985.25	19529.38
YBR009C	11534.02	11027.55	11206.54	11979.97	12553.91	11310.71	0.086094	7964.382	6813.288
YBL081W	1794.13	1791.779	1776.526	1954.726	1874.733	1799.341	0.069939	1198.48	1247.64
YLR180W	19455.61	23987.29	21068.34	25555.6	24175.64	21540.24	0.143775	12286.1	12129.58

YGR097W	970.765	1013.561	1085.874	907.0716	1031.276	1184.884	0.024707	1517.775	1948.284
YOR344C	968.4619	972.0301	1077.97	933.0391	914.8972	939.2973	-0.11498	1401.974	1792.878
YJL225C	20.72808	23.73217	31.61782	28.65379	44.93828	33.26678	0.490154	2.248555	3.512004
YJL191W	5831.5	5339.738	5162.597	6379.946	6305.186	5367.693	0.144361	4077.754	3679.703
YMR311C	2457.429	1891.652	2055.158	2020.988	1921.975	1871.746	-0.13932	3563.96	3336.404
YHR037W	2093.536	2650.092	2402.954	2062.178	2097.12	2060.584	-0.20037	1314.28	1258.176
YEL044W	2931.872	2676.791	2789.284	3061.479	2953.251	2422.604	0.006751	1685.292	1521.576
YEL036C	5710.586	6211.896	5986.636	6531.274	6062.058	5786.463	0.037426	3553.841	3650.728
YOL032W	423.7741	420.2572	358.6646	291.9105	298.4362	250.4793	-0.51639	836.4624	734.8869
YGL188C	488.2614	433.1121	405.1033	439.6566	407.9013	370.8268	-0.12263	233.8497	219.5003
YDL241W	223.4026	255.1208	224.2889	189.8314	163.6214	176.1182	-0.40832	447.4624	393.3445
YJL053W	536.6269	532.985	575.0491	563.2261	541.5638	573.3627	0.029084	981.4942	889.4151
YPR005C	305.1634	282.8084	296.417	244.4527	252.3457	275.9186	-0.19474	561.0145	512.7526
YNL332W	25.33432	25.70985	32.60588	20.59491	16.13169	17.61182	-0.62239	0	0
YMR265C	123.2169	201.7234	185.7547	163.8639	188.9712	198.6222	0.110787	413.7341	412.6605
YER091C	20977.97	26173.62	24230.12	27885.51	27348.97	30686.65	0.267459	11028.04	11157.64
YDR540C	173.8856	166.1252	213.4203	158.4913	139.4239	142.8515	-0.32839	55.0896	41.26605
YDL070W	3192.124	2737.11	3364.333	3277.278	3557.037	4019.41	0.223886	1860.679	1998.33
YHR174W	264712.5	223286.1	249745.2	262218	247694	241179.3	0.025868	434733.4	374781.8
YLR259C	7775.333	8865.943	7599.145	7606.687	7723.622	6833.388	-0.12922	14241.22	13511.56
YOR360C	1715.824	1781.89	1769.61	1588.495	1729.547	1615.396	-0.09448	991.6127	1034.285
YDR436W	391.5304	403.4469	389.2944	280.2699	265.0206	337.56	-0.42376	617.2283	652.3548
YBR072W	4569.39	4223.337	4127.113	4168.231	3678.025	3330.592	-0.20907	10125.24	9032.875
YPL106C	14187.22	13439.33	13759.68	13937.38	13832.92	15108.01	0.051098	21730.04	21817.45
YAR031W	29.94056	32.63173	21.73725	26.86293	19.58848	22.504	-0.29003	0	0
YLR120C	950.0369	1108.49	1041.412	927.6665	948.3128	1109.545	-0.05426	1509.905	1650.642
YMR170C	916.6417	771.2955	875.4183	787.9793	814.6503	690.7749	-0.16054	414.8584	270.4243
YGR281W	2601.374	2984.32	2725.061	2570.782	2487.737	2913.779	-0.05998	1497.538	1640.984
YEL030W	306.3149	297.641	223.3008	231.9166	187.8189	220.1478	-0.37053	627.3468	562.7987
YDR168W	1271.322	1166.832	1435.647	1280.466	1388.477	1495.048	0.104218	2298.023	2296.851
YFR050C	2631.314	2630.316	2786.32	2514.37	2579.918	2495.009	-0.08466	3958.581	3798.233
YKL152C	27638.59	23806.33	30696.95	30364.07	28848.07	32101.46	0.152712	50666.69	48476.2
YBR239C	1074.405	1336.912	1167.883	1144.361	1171.852	1224.022	-0.01579	573.3815	609.3327
YNL142W	613.7814	923.577	612.5952	548.0038	462.0577	422.6838	-0.58552	252.9624	218.6223
YPR160W	10859.21	11236.19	10436.84	9931.226	9233.087	9020.19	-0.20697	8893.035	8067.074
YMR180C	252.1916	260.065	239.1098	246.2435	246.5844	264.1774	0.010787	504.8006	495.1926
YDR459C	704.7547	654.6124	613.5833	573.0759	586.5021	557.7078	-0.20022	364.2659	249.3523
YCL036W	1379.569	2171.494	1596.7	2101.577	2183.539	2079.174	0.306054	867.9422	932.4371
YHR097C	856.7606	946.3203	902.0959	752.1621	782.3869	698.6024	-0.27664	1527.893	1603.23
YOL059W	2980.237	3511.372	3212.173	3141.172	2979.753	2969.549	-0.09419	4783.801	4771.936
YGL196W	1140.044	1225.173	1272.617	1250.022	1306.667	1267.073	0.071912	753.2659	573.3347
YOL014W	955.7947	888.9675	826.0155	967.9609	913.7449	653.5944	-0.0751	2211.454	1736.686
YOR362C	4217.012	4154.119	4082.651	3935.419	3866.996	3584.985	-0.12915	6183.526	5817.635
YMR104C	211.887	248.1989	192.6711	210.4263	172.8395	249.5009	-0.04487	485.6879	506.6066
YML048W	4537.146	4496.257	4249.632	4273.892	4182.716	4002.776	-0.09235	6330.806	6472.624
YPL049C	2898.476	3047.606	2761.619	3280.859	2768.889	2609.485	-0.00805	4030.535	5001.972
YEL071W	4253.862	5191.412	4697.222	4275.683	3855.474	4298.264	-0.18628	5989.026	5966.017
YIL162W	7523.141	7988.842	8067.484	4578.339	4697.778	4499.821	-0.77538	5358.306	5190.742
YNR073C	76.00296	88.99564	63.23564	35.81724	31.11111	37.18052	-1.13242	172.0145	172.0882
YJL166W	3734.509	3754.627	3519.458	3474.272	3454.486	2938.239	-0.15795	2470.038	2354.799
YDR358W	591.9018	543.8622	574.061	460.2515	417.1194	495.088	-0.31709	838.711	1019.359
YNR020C	1043.313	882.0457	769.6963	710.9722	799.6708	831.6695	-0.20238	431.7226	439.0005
YGL012W	7617.569	7424.214	7177.245	7210.011	7493.169	6649.442	-0.05738	5204.28	4605.994
YIR038C	1996.805	1982.625	1795.299	1899.209	1794.074	1701.498	-0.09819	1288.422	1188.813

YKR076W	865.9731	850.4028	733.1382	670.6778	661.3992	548.9019	-0.38101	337.2832	413.5385
YBR196C	25592.27	24122.76	25135.18	24383.48	23998.19	25221.11	-0.02425	37763.36	35512.51
YMR196W	2935.326	2912.135	2915.756	2285.14	2225.021	2492.073	-0.32365	1851.685	1863.118
YGL011C	4036.218	3700.241	3838.601	3712.457	3696.461	3664.238	-0.06395	5759.674	5385.659
YER081W	1935.772	1926.261	2038.361	1743.404	1760.659	1773.902	-0.16083	1000.607	1047.455
YIR003W	1137.741	1063.992	1217.286	1131.825	1085.432	1202.496	0.00031	1716.772	1880.678
YLL024C	37091.75	29974.72	34888.29	34078.31	31162.97	32770.71	-0.0569	56707.43	53475.53
YPR174C	362.7414	375.7594	420.9122	390.4079	422.8807	443.2309	0.116039	656.578	722.5949
YGR127W	280.9806	230.3998	270.7276	232.8121	207.4074	215.2556	-0.25483	460.9538	464.4626
YML032C	826.82	923.577	829.9677	740.5215	737.4486	774.9203	-0.1958	1131.023	1256.42
YHR203C	29641.15	26652.22	29360.11	31211.14	30256.13	29225.84	0.082481	49200.63	44970.34
YPL058C	3022.845	3302.727	3368.286	5870.446	6759.178	7482.09	1.052893	6710.812	8026.686
YML132W	13.81872	12.85493	19.76114	20.59491	20.74074	9.784347	0.138682	79.8237	65.85008
YBL071W-/	788.8186	853.3693	657.0578	868.5681	865.3499	701.5377	0.083031	463.2023	429.3425
YDR381C-/	550.4457	625.936	433.7569	398.4668	455.1441	379.6327	-0.38473	254.0867	212.4763
YCL048W-/	49.51708	47.46434	65.21175	37.6081	38.02469	33.26678	-0.57471	4.49711	5.268006
YPR078C	0	9.888404	5.928341	13.43147	9.218108	5.870608	0.85053	50.59249	46.53406
YBL045C	7896.247	7553.752	8444.922	8003.363	7874.569	8198.304	0.010906	6027.252	6213.614
YMR023C	293.6478	278.853	280.6081	313.4009	315.7202	302.3363	0.12676	164.1445	161.5522
YEL058W	1953.046	2067.665	1875.332	2072.027	1988.807	2007.748	0.041612	3260.405	2903.55
YGR286C	2062.444	2117.107	1959.317	2150.825	2008.395	1925.56	-0.01277	2647.673	2536.545
YDR330W	963.8557	995.7623	1149.11	837.228	854.9795	964.7366	-0.22656	1416.59	1575.134
YAL017W	631.0548	542.8734	619.5116	505.0231	450.535	542.0528	-0.26007	933.1503	1029.017
YIL135C	1137.741	1128.267	1263.725	1071.831	1185.679	1073.343	-0.08367	1506.532	1584.792
YEL017W	1409.509	1395.254	1287.438	1451.494	1431.111	1338.499	0.044743	908.4162	818.297
YDL048C	435.2897	444.9782	538.491	617.8474	442.4692	602.7158	0.229187	891.552	1220.421
YJL170C	125.52	141.4042	104.734	130.7329	175.144	101.7572	0.133298	24.7341	22.82803
YOR036W	613.7814	568.5832	604.6908	503.2322	449.3828	452.0368	-0.34737	969.1272	882.3911
YPR071W	537.7785	600.2261	534.5387	613.3703	645.2675	599.7805	0.152032	308.052	324.8604
YMR305C	6357.762	6867.497	6633.814	7200.161	7504.692	7571.128	0.165691	10315.25	10022.38
YBR208C	2470.096	2642.182	2725.061	2360.356	2448.56	3049.781	0.003926	1620.084	1665.568
YPL078C	5811.923	5527.618	5881.902	5415.567	5382.223	5024.262	-0.12227	4491.489	4058.121
YLL006W	347.7711	442.0117	456.4823	337.5775	371.0288	413.8779	-0.15092	735.2775	757.7149
YDL087C	208.4323	262.0427	222.3128	220.276	195.8848	259.2852	-0.03657	427.2254	466.2186
YPL081W	9212.48	10054.53	8962.663	10938.59	10124.94	9028.996	0.092193	6548.916	5412.877
YER012W	2196.025	1769.036	1869.404	1855.333	1735.309	1810.104	-0.11144	2933.24	2859.649
YHR031C	1342.719	1425.908	1367.471	1437.167	1500.247	1667.253	0.154827	778	827.955
YOR387C	6.90936	4.944202	0.988057	0.895431	4.609054	4.892174	-0.30471	33.72832	35.12004
YBR034C	5096.804	5333.805	5198.167	5857.91	5851.194	5810.924	0.1648	4165.448	3973.833
YKL213C	1006.463	1001.695	1019.675	956.3203	964.4445	1009.745	-0.04713	1381.737	1548.794
YIR029W	271.7681	267.9758	217.3725	154.0141	197.0371	151.6574	-0.59079	96.68786	73.75209
YNR001C	2152.266	2217.969	2187.558	1482.834	1479.506	1408.946	-0.58515	2999.572	2679.659
YHR180W	302.8603	343.1276	321.1185	288.3288	289.2181	230.9106	-0.2585	83.19653	136.9682
YLR303W	8959.136	10277.02	9024.911	10048.53	9218.108	8705.134	-0.01484	4371.191	3802.623
YCR099C	52.97176	32.63173	39.52227	33.13095	41.48148	22.504	-0.36559	125.9191	113.2621
YKL059C	724.3312	729.7642	725.2337	687.691	624.5268	694.6886	-0.11891	1080.431	987.7512
YGR013W	705.9062	656.59	640.2608	719.0311	621.07	701.5377	0.02774	1062.442	1116.817
YPR035W	21527.26	21155.25	20981.39	20500.89	19880	21157.67	-0.04898	26622.89	26174.97
YPL075W	802.6373	798.9831	1012.758	862.3001	927.5721	1031.27	0.109811	1432.33	1572.5
YBL030C	10296.1	12602.77	9985.302	10179.26	10300.08	8173.844	-0.1987	6915.431	6379.556
YNR036C	3500.742	2887.414	3103.486	2972.831	3100.741	2741.574	-0.10667	2495.896	2053.645
YAL038W	23106.05	24855.49	29319.6	28661.85	29486.42	35547.51	0.277868	44343.75	48021.39
YDL067C	2815.564	2859.727	2736.917	2611.077	2758.519	2469.569	-0.10178	2120.387	1935.114
YPR048W	559.6581	718.887	593.8222	752.1621	670.6173	695.6671	0.178143	1096.171	1199.349

YDR050C	75932.71	60613.94	66797.58	71103.49	66015.48	63485.74	-0.01957	126580.2	103132.6
YPR140W	754.2718	960.1641	798.3499	901.699	768.5597	752.4163	-0.05269	411.4856	453.9266
YDL007W	3455.831	3471.819	3419.665	3623.809	3459.095	3358.966	0.013124	4914.217	4546.29
YNL231C	3438.558	3669.587	3341.608	3453.677	3022.387	2825.719	-0.16789	2001.214	1903.506
YKL146W	674.8141	791.0723	682.7473	645.6058	707.4898	699.5808	-0.06591	1109.662	1161.595
YIL066C	285.5869	271.9311	222.3128	196.9948	215.4733	225.04	-0.29072	344.0289	364.3704
YBR183W	900.5199	1272.638	968.2957	1148.838	1054.321	816.993	-0.05681	433.9711	367.0044
YJR104C	26569.94	23875.55	23645.19	27921.33	27135.8	23532.33	0.085044	18502.23	15005.92
YLR073C	1494.725	1846.165	1362.53	1754.149	1626.996	1375.679	0.016289	830.8411	783.177
YIL075C	4282.651	4193.672	4143.91	3931.838	4130.865	4095.728	-0.05378	5584.286	6079.279
YGR122C-/	1.15156	5.933043	2.96417	0	0	0.978435	-3.3604	20.23699	28.09603
YOR389W	422.6225	508.264	435.7331	437.8658	477.0371	390.3955	-0.06623	212.4884	170.3322
YKL193C	921.248	662.5231	808.2305	732.4626	755.8848	736.7613	-0.10434	1299.665	1296.808
YIL145C	1647.882	1591.044	1574.963	1672.665	1751.44	1678.994	0.084171	1070.312	1050.967
YHR125W	28.789	14.83261	26.67753	28.65379	14.97943	13.69809	-0.29418	0	0
YML030W	1236.775	973.019	1138.241	1029.746	969.0536	1020.507	-0.1491	722.9104	657.6228
YDL085W	193.4621	283.7972	221.3247	193.4131	173.9918	127.1965	-0.49817	65.20809	65.85008
YOR035C	416.8647	433.1121	446.6017	458.4607	413.6626	508.7861	0.090909	744.2717	783.177
YIL166C	293.6478	309.5071	334.9513	284.7471	360.6585	410.9426	0.171262	105.6821	106.2381
YML128C	3347.585	3774.404	3333.704	3032.825	2589.136	2798.323	-0.31235	5552.806	5460.289
YDR225W	16628.53	14438.06	14847.53	15927.03	15549.8	13379.12	-0.03364	10467.02	8998.633
YMR181C	652.9345	685.2664	612.5952	538.154	473.5803	497.0448	-0.37068	876.9364	927.1691
YMR020W	629.9033	683.2887	607.6549	576.6576	498.9301	491.1742	-0.29396	966.8786	875.3671
YMR251W-/	17628.08	20188.17	17040.03	15422.01	15425.35	14096.31	-0.28754	11520.47	10968.87
YOR390W	80.6092	111.739	103.746	99.39284	119.8354	108.6063	0.146912	329.4133	219.5003
YOR259C	3735.66	4031.502	3919.621	3987.354	3724.116	3927.437	-0.00592	5790.029	5124.892
YDR286C	295.9509	250.1766	314.2021	242.6618	239.6708	282.7676	-0.16924	143.9075	137.8462
YGL036W	646.0251	611.1034	616.5475	645.6058	536.9548	612.5001	-0.06184	894.9249	1068.527
YAR073W	20.72808	25.70985	21.73725	12.53603	20.74074	35.22365	0.006866	0	2.634003
YEL017C-/	5410.029	5286.341	5288.08	6186.533	6524.116	5285.504	0.171019	4003.552	3497.078
YGR186W	1699.702	1837.266	1854.583	1833.843	1786.008	2142.772	0.096025	2873.653	2809.603
YOR043W	2197.176	2231.813	2016.624	2138.289	1962.305	2032.209	-0.07177	1410.968	1418.85
YDR394W	3215.155	3113.859	3039.263	3197.584	3062.716	3216.115	0.016558	4157.578	4175.773
YDR461C-/	193.4621	201.7234	130.4235	133.4192	144.0329	101.7572	-0.471	60.71098	53.55807
YGR091W	665.6016	676.3669	745.9829	688.5865	713.2511	695.6671	0.006586	362.0173	375.7845
YPL061W	63798.72	76179.28	64348.19	61170.48	61580.42	57823.53	-0.17828	36707.66	36201.74
YBR244W	1490.119	1394.265	1509.751	1826.679	2070.617	1724.002	0.355324	1091.673	872.7331
YGL067W	1077.86	1030.372	1001.89	1119.289	1132.675	941.2542	0.03804	634.0925	582.1147
YMR016C	968.4619	807.8826	1040.424	1053.922	1004.774	1263.159	0.237948	1481.798	1999.208
YGR136W	537.7785	590.3377	610.6191	566.8078	546.1729	550.8587	-0.06352	790.3671	822.687
YNL067W	12585.4	11941.24	12578.95	14733.42	13991.94	12712.8	0.159323	10302.88	8975.805
YMR171C	1235.624	1434.807	1368.459	1347.624	1368.889	1637.9	0.108519	758.8873	885.9031
YHR115C	1312.778	1341.856	1211.358	1233.009	1168.395	1084.106	-0.14947	749.8931	814.785
YJR154W	25.33432	31.64289	53.35507	41.18983	42.63375	43.05113	0.201549	88.81792	159.7962
YOR338W	511.2926	818.7599	537.5029	525.618	448.2305	431.4897	-0.41023	220.3584	211.5983
YMR152W	2292.756	2503.744	2337.742	2365.729	2281.482	2247.465	-0.04928	1668.428	1532.112
YDR004W	948.8854	1063.992	1014.734	978.7061	899.9178	895.2678	-0.12627	554.2688	512.7526
YPL256C	1138.893	1098.602	1063.149	1225.845	1230.617	1319.908	0.194253	1635.824	1742.832
YMR266W	1935.772	1968.781	1998.839	2013.824	2072.922	2301.278	0.113826	2925.37	3342.55
YOR247W	7109.731	6493.715	7426.235	7663.099	8623.54	8248.205	0.222405	11854.38	12382.45
YCR075C	664.4501	583.4159	615.5594	514.8728	472.428	461.8212	-0.36278	305.8035	281.8383
YOL106W	24.18276	24.72101	21.73725	23.28121	20.74074	26.41774	-0.00412	0	3.512004
YCR028C	837.1841	1135.189	937.6659	1043.177	1002.469	917.7718	0.026224	545.2746	587.3827
YLR100W	2873.142	3618.167	3061.988	3458.155	3468.313	3172.085	0.080078	2039.439	2020.28

YMR025W	200.3714	212.6007	257.8828	160.2822	194.7325	232.8675	-0.19047	104.5578	93.06811
YDL126C	6951.967	7283.799	7205.898	6943.172	6776.461	7525.141	-0.01331	9663.165	10387.63
YGL174W	458.3209	411.3576	400.163	487.1145	464.3622	442.2525	0.134302	716.1648	662.0128
YPL054W	52.97176	65.26347	53.35507	36.71267	27.65432	19.56869	-1.03161	110.1792	121.1641
YDL089W	354.6805	372.7928	373.4855	362.6496	361.8107	320.9266	-0.07472	553.1445	621.6248
YBL107C	1252.897	1109.479	1204.441	1355.683	1258.272	1189.777	0.092778	817.3497	732.2529
YNR034W	351.2258	435.0898	311.2379	308.9237	372.1811	309.1854	-0.14837	709.4191	676.9388
YOR208W	282.1322	275.8865	255.9067	206.8446	233.9095	264.1774	-0.20741	417.1069	531.1906
YJR113C	1285.141	1381.41	1183.692	1176.596	987.4898	866.8932	-0.34517	744.2717	654.1108
YNL130C	2160.326	2319.82	2118.394	2223.355	2514.239	2038.08	0.038218	1482.922	1360.024
YNL100W	1281.686	1498.093	1327.948	1155.106	1216.79	1064.537	-0.25743	865.6937	827.077
YMR182C	244.1307	247.2101	315.1901	309.8191	303.0453	331.6894	0.227903	477.8179	681.3288
YFL053W	20.72808	31.64289	49.40284	37.6081	20.74074	28.37461	-0.23087	88.81792	122.0421
YNL055C	17547.47	17598.39	18100.21	17829.82	17434.9	17337.86	-0.01754	13842.1	11981.2
YBR010W	18732.43	16844.9	17653.61	20207.19	18945.52	16659.81	0.068324	12181.55	11504.45
YGR248W	1456.723	1311.202	1428.73	1208.832	1190.288	1020.507	-0.2954	2519.506	2326.703
YAL046C	852.1544	791.0723	797.3619	805.8879	876.8725	737.7398	-0.01192	580.1272	431.0985
YGR123C	2056.686	2008.335	2072.943	2183.956	2238.848	2171.147	0.103383	1579.61	1508.406
YNL208W	7784.545	7958.188	7047.809	7632.654	6940.083	5907.789	-0.15418	11563.19	10144.42
YGR223C	599.9627	550.7841	530.5865	407.4211	443.6214	478.4546	-0.33873	817.3497	748.9349
YLR414C	1520.059	1692.895	1227.167	1327.924	1272.099	1154.553	-0.24195	2508.263	2191.491
YOL085C	34.5468	49.44202	45.45061	35.81724	33.41564	16.63339	-0.59211	3.372832	2.634003
YMR250W	6189.635	7117.673	6049.872	5757.621	5315.391	4921.527	-0.27529	4718.593	4516.438
YAR023C	99.03415	137.4488	97.81763	62.68017	81.81071	55.77078	-0.73926	276.5723	211.5983
YIL077C	795.7279	872.1573	775.6246	647.3966	659.0947	571.4059	-0.37984	481.1908	381.0525
YOR385W	266.0103	344.1165	335.9393	241.7664	256.9548	227.9753	-0.38059	509.2977	529.4346
YER126C	5475.668	5530.585	5131.967	6456.058	5950.289	5757.11	0.170557	4311.604	3649.85
YMR208W	2495.43	3175.167	2524.485	2770.464	2942.881	2522.405	0.007141	1671.801	1655.91
YKR070W	971.9166	977.9632	999.9135	941.098	904.5268	826.7773	-0.14248	636.3411	564.5547
YIL010W	1576.486	1314.169	1343.757	1265.244	1251.358	1277.836	-0.15828	950.0145	863.9531
YOR071C	233.7667	346.0942	323.0946	372.4993	266.1729	267.1127	0.004514	516.0434	535.5807
YJR115W	423.7741	397.5139	502.9209	203.2628	178.6008	217.2125	-1.14432	555.3931	663.7688
YOR285W	7128.156	5718.464	6511.294	5668.974	5954.898	5509.566	-0.17611	4900.726	4024.757
YOL112W	628.7517	760.4183	693.6159	653.6646	755.8848	618.3707	-0.03851	1054.572	1063.259
YPL068C	1240.23	1247.917	1141.206	1266.139	1171.852	1031.27	-0.06508	662.1994	792.835
YKL219W	542.3847	483.543	496.9926	554.2718	432.0988	434.425	-0.10014	1050.075	841.125
YLR407W	1653.64	1824.411	1788.383	1969.053	1844.774	1610.504	0.042619	1012.974	1111.549
YPL177C	1870.133	1934.172	1708.35	1703.11	1631.605	1536.143	-0.17857	2307.017	2378.505
YJL106W	36.84992	50.43086	50.3909	59.09845	58.76544	52.83547	0.310226	98.93642	97.45812
YDL097C	3363.707	3278.006	3273.432	3056.106	3151.441	3438.22	-0.03974	4517.347	4371.567
YDR277C	335.1039	338.1834	385.3422	260.5704	235.0617	282.7676	-0.44361	468.8237	647.9648
YDR241W	20.72808	29.66521	28.65365	28.65379	25.3498	17.61182	-0.14244	92.19075	89.55611
YMR132C	435.2897	433.1121	396.2108	438.7612	474.7325	454.9721	0.113864	256.3353	199.3062
YGL090W	219.9479	243.2547	213.4203	254.3024	259.2593	176.1182	0.027575	540.7775	473.2426
YDR377W	2278.937	2137.873	2386.157	2303.944	2360.988	2041.993	-0.02051	1804.465	1548.794
YCR101C	12.66716	2.966521	1.976114	8.95431	5.761317	4.892174	0.15505	29.23121	72.87409
YGR033C	1870.133	1772.991	1534.452	1620.73	1794.074	1679.972	-0.02326	1225.462	903.4631
YML097C	538.9301	595.2819	637.2967	556.9581	616.461	656.5297	0.046825	1095.046	899.0731
YHL030W	571.1737	532.985	579.0013	510.3957	553.0865	689.7965	0.058883	930.9018	978.0932
YNR044W	1946.136	2183.36	2072.943	2595.855	2835.72	3089.897	0.458267	1405.347	1789.366
YIL107C	282.1322	207.6565	275.6679	241.7664	269.6297	317.9913	0.115726	436.2197	510.1186
YEL048C	392.6819	465.7438	396.2108	413.6891	483.9507	454.9721	0.108478	261.9567	236.1823
YCR102W-/	2.30312	4.944202	2.96417	2.686293	4.609054	0	-0.48515	19.11272	28.09603
YIL094C	2769.502	2680.746	2838.687	2843.889	2743.539	2603.615	-0.01714	3696.624	3644.582

YPR191W	6612.257	7219.524	6739.536	6975.408	6611.688	6069.231	-0.06564	5223.393	5086.26
YBR170C	679.4204	758.4406	670.8906	617.8474	594.5679	591.953	-0.2249	976.9971	911.3651
YNL331C	198.0683	221.5003	164.0174	163.8639	147.4897	182.9673	-0.2395	62.95954	49.16806
YDR078C	271.7681	177.0024	264.7992	264.1522	185.5144	226.0184	-0.0787	82.07226	93.06811
YCL009C	6915.117	6252.438	5935.257	6121.166	5793.581	5022.305	-0.1736	10474.89	9199.695
YHR094C	80.6092	89.98448	85.96094	76.11164	58.76544	70.4473	-0.32136	263.0809	212.4763
YDR298C	4705.274	4415.173	4404.757	4264.043	4149.301	3916.674	-0.13348	3793.312	3252.994
YLR005W	917.7933	950.2757	904.072	808.5742	907.9836	886.4619	-0.09081	1258.066	1231.836
YGR230W	176.1887	151.2926	115.6026	151.3278	182.0576	136.9809	0.086205	61.83526	65.85008
YMR282C	813.0013	915.6662	843.8005	841.7052	788.1482	797.4243	-0.08381	491.3093	539.9707
YNL030W	14690.45	12643.31	13046.3	14013.5	14005.76	12647.25	0.010198	10232.05	8485.88
YGL048C	3451.225	3594.435	3422.629	3596.051	3482.14	3662.281	0.037032	5051.379	4576.142
YJR003C	644.8736	564.6279	686.6995	740.5215	759.3416	787.6399	0.270662	1082.679	1100.135
YMR314W	4271.136	3639.922	3818.84	3876.321	3746.009	3552.696	-0.06991	5441.503	5296.102
YJL082W	505.5348	537.9292	534.5387	411.8983	479.3416	420.7269	-0.26637	734.1532	746.3009
YDR345C	4060.4	5371.381	4856.299	6110.421	5722.14	5194.51	0.253018	8599.598	8229.504
YKL144C	1204.532	1078.825	1219.262	1142.57	1264.033	1300.34	0.081796	819.5983	740.1549
YPR108W-/	10.36404	17.79913	16.79697	38.50353	40.32922	19.56869	1.130033	0	1.756002
YAR071W	207.2808	223.4779	154.1369	178.1908	124.4445	135.024	-0.41837	60.71098	68.48408
YDL200C	259.101	258.0874	222.3128	239.0801	251.1934	205.4713	-0.08799	125.9191	134.3342
YDR019C	1547.697	2032.067	1505.799	1618.939	1659.259	1374.701	-0.12828	797.1127	842.881
YBL058W	2966.418	2884.448	2970.099	2988.053	2737.778	2864.857	-0.03816	3657.275	3785.941
YDR524W-(	15125.74	12222.07	13709.29	15462.3	14533.5	12323.39	0.04368	9941.986	9031.997
YLR105C	1035.252	1073.881	993.9852	1018.105	1002.469	909.9443	-0.08256	661.0752	633.0388
YPL201C	57.578	39.55362	42.48644	34.02638	50.69959	41.09426	-0.15012	15.73988	1.756002
YML100W	1833.283	1659.274	1933.627	936.6209	871.1112	953.9738	-0.97438	2158.613	2298.607
YLR348C	435.2897	390.592	311.2379	317.878	299.5885	312.1207	-0.29072	618.3526	502.2166
YMR083W	6238	6650.941	5961.935	6482.921	6075.885	5848.104	-0.03438	9876.778	8477.1
YML083C	49.51708	80.09608	67.18786	57.30759	46.09054	55.77078	-0.30618	134.9133	116.7741
YMR286W	1402.6	1132.222	1068.089	1089.74	1073.91	974.521	-0.19924	914.0376	782.299
YFL017C	1316.233	1220.229	1176.776	1264.349	1346.996	1172.165	0.027047	888.1792	848.149
YDL185W	12389.63	11825.54	12573.02	13164.63	12989.47	13654.06	0.113821	9265.171	9262.911
YGR086C	19479.79	20630.18	19870.81	18033.09	17376.13	17367.22	-0.18461	13977.02	14103.33
YJR073C	4322.956	4085.889	4211.098	4084.061	4085.926	3700.44	-0.08833	2956.85	2276.657
YDR404C	2303.12	2044.922	2226.092	2145.453	2208.889	1869.789	-0.07893	1455.939	1466.262
YNL191W	2391.79	2513.632	2319.957	2469.599	2237.696	1912.84	-0.12621	1379.488	1301.198
YKL187C	386.9241	383.6701	365.581	237.2892	238.5185	303.3148	-0.54426	120.2977	133.4562
YMR174C	792.2732	866.2242	822.0633	723.5083	820.4116	641.8532	-0.18252	529.5347	461.8286
YEL057C	87.51856	139.4265	96.82957	95.81112	94.4856	84.14539	-0.23849	287.815	266.0343
YOL064C	1444.056	1429.863	1509.751	1670.874	1509.465	1479.393	0.088108	2104.647	1817.462
YJR077C	8116.194	9674.815	8295.725	8742.989	8438.025	6856.87	-0.11801	6342.049	5932.653
YBR265W	2341.121	2471.112	2194.474	2335.284	2299.918	2321.826	-0.01027	1647.067	1540.892
YMR135C	514.7473	458.822	368.5452	322.3552	402.14	384.5248	-0.27522	692.5549	679.5728
YDR533C	806.092	754.4853	751.9112	1041.386	763.9507	805.2518	0.174929	1660.558	1247.64
YJL098W	1429.086	1569.29	1462.324	1887.569	1789.465	2160.384	0.38806	1006.228	1229.202
YLR052W	1108.952	1230.117	1199.501	1224.054	1161.482	1227.936	0.030219	911.789	784.933
YOL077W-/	1544.242	1337.901	1215.31	1261.662	1534.815	1308.167	0.00253	993.8613	1017.603
YDR524C-I	61834.16	56766.36	61549.02	62762.55	63621.08	58456.58	0.037084	47250.01	41125.57
YER142C	210.7355	113.7166	111.6504	98.49741	70.28807	107.6278	-0.65784	340.6561	296.7644
YBR033W	342.0133	334.2281	349.7721	250.7207	276.5432	306.2501	-0.29977	170.8902	168.5762
YCL037C	525.1113	535.9515	613.5833	632.1743	506.9959	510.7429	-0.02147	259.7081	312.5684
YDR287W	269.465	277.8642	326.0588	285.6425	270.7819	274.9402	-0.07114	573.3815	539.9707
YAL016W	1615.639	1627.631	1759.729	1514.174	1564.774	1736.722	-0.05506	2355.361	2319.679
YKL140W	1019.131	936.4319	1105.636	933.9346	1055.473	988.2191	-0.03993	1415.465	1536.502

YHR018C	1692.793	1916.373	1944.496	1772.953	1621.235	1648.662	-0.1392	2193.465	2027.304
YOL113W	312.0727	356.9714	413.9958	286.5379	301.893	328.7541	-0.2398	478.9422	540.8487
YAR028W	572.3253	528.0408	466.3628	535.4678	439.0124	439.3172	-0.14818	957.8844	785.811
YJR148W	5122.139	5484.109	4913.607	4945.466	4679.342	4524.282	-0.13341	7975.624	7378.721
YDR406W	965.0072	1018.506	991.021	627.6971	672.9219	907.009	-0.43017	456.4567	500.4606
YNR041C	794.5764	931.4877	705.4726	676.0504	793.9095	550.8587	-0.26693	391.2486	286.2284
YDL202W	1591.456	1580.167	1657.959	1542.828	1518.683	1397.205	-0.11527	1242.327	1246.762
YMR029C	618.3877	566.6056	671.8786	584.7165	568.0659	627.1767	-0.06103	844.3324	916.6331
YMR009W	2976.782	3142.535	2831.771	2860.007	2658.272	2296.386	-0.19588	1758.37	1655.91
YBL075C	1870.133	2221.924	1709.338	1654.757	1350.453	1417.752	-0.39139	1011.85	921.9011
YKL201C	1729.643	1948.016	2066.027	1935.026	1916.214	2440.216	0.131417	851.0781	1129.987
YKL212W	2414.821	2312.898	2536.342	2612.868	2796.543	3070.328	0.223244	3598.812	3785.063
YML098W	1365.75	1302.303	1298.307	1263.453	1276.708	1061.602	-0.13911	809.4798	807.761
YML108W	1036.404	944.3426	961.3793	1241.963	1130.37	954.9523	0.177487	725.159	618.1128
YNL192W	1366.902	1389.321	1592.748	1583.122	1707.654	2116.354	0.314189	2296.899	2938.67
YIR014W	29.94056	28.67637	50.3909	35.81724	41.48148	44.02956	0.154484	67.45665	122.9202
YKR085C	1895.468	1538.636	1619.425	1618.044	1496.79	1610.504	-0.09687	1319.902	1234.47
YHL039W	1419.873	1473.372	1441.575	1553.573	1704.198	1825.759	0.229858	951.1387	1018.481
YFL021W	705.9062	684.2776	707.4487	650.9784	670.6173	554.7725	-0.16082	360.8931	323.1044
YNL156C	3460.438	3774.404	3535.267	3328.317	3252.84	3082.069	-0.15646	2423.942	2290.705
YJR127C	543.5363	441.0228	570.1088	500.5459	527.7367	725.0201	0.173469	901.6705	1037.797
YOR289W	456.0177	468.7104	516.7537	393.9897	395.2264	361.0424	-0.32559	335.0347	342.4204
YDR422C	549.2941	522.1077	576.0371	603.5205	603.7861	788.6184	0.276832	866.8179	997.4092
YBR229C	827.9716	857.3247	1001.89	818.424	955.2264	1015.615	0.053789	1325.523	1448.702
YBL107W-/	1.15156	0.98884	0	0.895431	0	0	-1.25723	10.1185	21.07203
YBL043W	168.1278	176.0136	191.683	158.4913	135.9671	93.92973	-0.46426	52.84104	37.75405
YCL040W	9441.64	10703.21	9678.017	8239.756	7285.762	7148.444	-0.39538	14252.47	13972.51
YPL193W	976.5228	917.6439	893.2034	974.229	962.14	976.4778	0.063525	608.2341	664.6468
YFL036W	1204.532	1234.073	1302.259	996.6147	1032.428	1449.062	-0.10507	1949.497	2174.809
YJL164C	735.8468	560.6725	575.0491	376.081	342.2223	385.5033	-0.76176	279.9451	268.6683
YKL068W-/	1082.466	1369.544	1049.316	1078.099	1039.342	749.481	-0.2884	529.5347	439.0005
YGR240C	10782.06	10088.15	10900.24	10756.81	10891.19	12606.15	0.108594	15462.19	15450.18
YOR303W	3075.817	3533.127	2936.505	2967.458	2752.757	2617.313	-0.19519	1986.598	2038.718
YOL055C	613.7814	600.2261	645.2011	584.7165	603.7861	514.6567	-0.12648	292.3121	362.6144
YLR025W	2064.747	2011.301	2117.406	2043.374	1875.885	2049.821	-0.05324	2810.694	2872.82
YOR117W	3097.696	3238.452	2998.752	3046.256	2977.449	2942.153	-0.05819	4073.257	3922.031
YLR350W	2896.173	2861.704	2704.312	2403.337	2346.008	2004.813	-0.32525	3945.09	3401.376
YAR068W	111.7013	117.672	140.3041	96.70655	47.2428	95.8866	-0.62422	30.35549	26.34003
YGL141W	428.3803	387.6254	453.5181	326.8323	326.0906	377.6758	-0.3008	561.0145	618.1128
YNL179C	18.42496	19.77681	9.880568	20.59491	17.28395	10.76278	0.016685	46.09538	62.33808
YOR212W	3301.522	3383.812	3153.877	3031.034	2940.576	3432.349	-0.06527	4446.517	4214.405
YPL084W	1088.224	1008.617	1198.513	1012.732	1017.449	1136.941	-0.05726	1645.942	1561.964
YNR035C	3238.187	2692.613	3231.934	3132.218	2829.959	2688.739	-0.08293	4361.072	4479.561
YMR110C	2337.667	2220.936	2269.567	1944.876	1877.037	2183.866	-0.18515	1481.798	1411.826
YDR031W	271.7681	262.0427	263.8112	253.407	229.3004	263.1989	-0.09671	127.0434	175.6002
YOR323C	2657.8	2730.188	2677.634	2732.855	2658.272	2852.137	0.03143	3919.231	3551.514
YGL183C	58.72956	55.37506	85.96094	58.20302	65.67902	55.77078	-0.15526	25.85838	10.53601
YHR114W	670.2079	708.0098	715.3531	677.8413	682.14	855.1519	0.081428	1220.965	1110.671
YDL147W	3247.399	3317.56	3258.611	3199.375	3171.029	3554.653	0.014828	4555.572	4476.927
YKL132C	61.03268	51.4197	77.06843	59.99388	58.76544	69.46886	-0.00987	155.1503	129.0662
YKL143W	2615.193	2534.398	2595.625	3062.374	2902.552	3009.665	0.212541	2146.246	2097.545
YKL089W	321.2852	385.6478	343.8438	337.5775	312.2634	378.6542	-0.03092	587.9971	589.1387
YCR053W	6479.828	6246.505	6413.477	6706.778	6710.782	6694.45	0.071481	8432.081	8417.396
YDR039C	4.60624	13.84377	13.8328	4.477155	10.37037	7.827478	-0.50966	58.46243	31.60804

YGR019W	1723.885	1724.538	1644.127	1305.538	1457.613	1249.461	-0.34385	945.5174	940.3392
YER096W	119.7622	188.8685	136.3518	102.9746	95.63787	93.92973	-0.6051	57.33815	46.53406
YGR124W	7508.171	7180.959	7182.185	7081.069	6649.712	7065.277	-0.07273	10413.06	9424.464
YNL173C	3684.992	3524.227	3524.399	3103.564	2703.21	2870.727	-0.30678	2378.971	2335.483
YLR009W	5883.32	5889.534	5093.433	6630.667	6395.062	5504.674	0.135752	4569.064	4372.445
YOR348C	1070.951	1686.962	1121.444	663.5144	704.033	481.3899	-1.06912	351.8989	388.9545
YJL128C	1083.618	1049.16	1036.472	980.497	1017.449	1227.936	0.025552	1434.578	1489.968
YNL180C	2429.791	2312.898	2271.543	2191.12	2329.877	2096.786	-0.08394	1540.26	1618.156
YDR368W	2847.808	2536.376	2698.383	2653.162	2836.873	2592.852	5.71E-05	3842.78	3547.124
YMR189W	1574.182	2229.835	1783.443	1732.659	1609.712	1689.757	-0.15102	764.5087	726.9849
YMR004W	936.2182	946.3203	992.0091	937.5163	971.3581	980.3916	0.007368	1359.251	1387.242
YPR069C	2974.479	2594.717	2851.532	2864.484	2922.14	2658.407	0.004158	4733.208	3937.835
YBR201C-/	115.156	166.1252	96.82957	72.52991	92.18108	74.36104	-0.66136	287.815	208.9643
YPL271W	2274.331	1870.886	1831.857	1871.451	1886.255	1746.506	-0.1189	1566.119	1313.49
YPL183C	1712.37	1991.525	1939.556	2019.197	2183.539	2166.254	0.174487	1416.59	1444.312
YMR317W	158.9153	188.8685	195.6353	160.2822	147.4897	170.2476	-0.185	281.0694	273.9363
YIL105C	629.9033	569.5721	722.2695	526.5134	527.7367	663.3787	-0.162	882.5578	976.3372
YKL029C	2323.848	3258.229	2666.765	3369.507	3552.428	3380.492	0.320721	3702.246	4240.745
YDR475C	538.9301	651.6458	696.5801	630.3834	644.1153	708.3867	0.071388	896.0492	1093.111
YHR169W	2003.714	2470.123	2220.164	2788.372	2741.235	2585.025	0.277657	1702.156	1746.344
YNL058C	648.3282	628.9025	651.1294	777.2341	715.5556	613.4786	0.127315	1053.448	980.7272
YLR433C	665.6016	708.9986	636.3086	656.3509	634.8972	703.4946	-0.01165	1075.934	880.6351
YPL221W	1697.399	1869.897	1816.048	1645.802	1635.062	1780.751	-0.08891	2355.361	2619.077
YDR115W	624.1455	589.3489	521.694	599.0434	601.4815	603.6942	0.056282	421.6041	400.3685
YJR114W	27.63744	32.63173	21.73725	25.9675	26.50206	20.54713	-0.16751	5.621387	2.634003
YGR194C	579.2346	517.1635	515.7657	372.4993	373.3334	455.9506	-0.42382	833.0896	797.225
YLR179C	5222.324	4889.816	4589.524	5229.317	5184.033	4340.336	0.005096	7836.214	6714.952
YCL057C-/	2774.108	2236.757	2510.652	2444.527	2493.498	2217.133	-0.07204	2069.795	1842.046
YOR226C	776.1514	752.5076	850.7169	989.4513	1118.848	978.4347	0.375498	513.7948	487.2906
YDR127W	2546.099	2403.871	2472.118	2410.5	2646.749	2665.256	0.057244	1670.676	1717.37
YDR145W	1582.243	1306.258	1632.27	1618.939	1791.77	2101.678	0.286108	2662.289	2585.713
YCL054W	3483.469	3925.697	3513.53	4401.043	4635.556	4619.19	0.322184	2835.428	3225.776
YLR364W	437.5928	497.3867	349.7721	530.0952	451.6873	360.064	0.06273	214.737	179.1122
YPR103W	2958.357	2911.146	2882.162	2753.45	3048.889	2857.029	-0.0153	3777.572	3672.678
YCR018C-/	38.00148	42.52014	42.48644	30.44465	39.17696	35.22365	-0.23049	11.24277	1.756002
YLL028W	1787.221	2439.469	1901.021	2263.65	1948.478	1918.71	0.000736	1131.023	1331.928
YNL106C	383.4695	371.804	420.9122	402.0485	353.7449	446.1662	0.031273	580.1272	636.5508
YNL038W	998.4025	878.0903	849.7289	885.5813	825.0206	764.1575	-0.13961	574.5058	527.6786
YKL015W	584.9924	472.6657	552.3238	484.4282	511.605	604.6727	-0.00834	811.7283	929.8031
YNL306W	1726.188	1612.799	1699.458	1360.16	1406.914	1288.599	-0.31304	1288.422	1057.113
YGL047W	591.9018	431.1344	470.315	401.1531	357.2017	379.6327	-0.39207	1003.98	721.7169
YCL043C	18575.81	16957.62	17455.01	19771.12	19523.95	17783.05	0.10726	27538.05	24044.94
YGR039W	141.6419	135.4711	72.12815	130.7329	106.0082	90.01599	-0.096	32.60405	26.34003
YER043C	24500.59	28538.92	26382.11	28091.46	27957.37	26172.15	0.049975	19036.27	19597.86
YPL002C	564.2644	607.148	661.9981	605.3114	596.8725	635.9826	0.003738	376.633	364.3704
YMR169C	1127.377	1214.296	993.9852	843.496	866.5021	794.489	-0.41346	807.2312	735.7649
YJL156W-/	211.887	210.623	179.8263	166.5502	203.9506	177.0967	-0.13745	124.7948	76.38609
YNL279W	249.8885	297.641	224.2889	217.5897	212.0165	238.7381	-0.20767	102.3093	142.2362
YNR019W	5879.865	8648.398	6109.155	7247.619	7183.21	6542.793	0.023313	3805.679	3736.773
YIL097W	162.37	191.835	183.7786	156.7004	130.2058	130.1318	-0.36738	287.815	341.5424
YGR176W	70.24516	53.39738	52.36701	67.15733	58.76544	47.9433	-0.01768	19.11272	17.56002
YOR353C	649.4798	619.0141	636.3086	575.7621	649.8766	755.3516	0.05658	944.3931	965.8012
YGL227W	329.3461	300.6075	295.429	207.74	215.4733	301.3579	-0.35292	475.5694	515.3866
YBR267W	2503.491	2601.639	2203.367	3094.61	2674.404	2488.159	0.176073	2049.558	1735.808

YGL180W	78.30608	114.7055	79.04455	99.39284	61.06996	91.97286	-0.10799	174.263	166.8202
YGR146C	202.6745	254.132	258.8709	210.4263	254.6502	223.0831	-0.05657	410.3613	379.2965
YGL160W	1129.68	1291.426	1169.859	1112.125	1139.589	1008.766	-0.13929	776.8757	646.2088
YEL060C	5291.418	6590.621	5613.151	5255.285	5288.889	5347.146	-0.13872	7306.679	7635.975
YBR236C	877.4887	744.5968	996.9493	828.2737	786.9959	868.85	-0.0763	1358.127	1316.124
YLR304C	7159.248	7866.226	7278.027	7141.062	7050.7	7222.805	-0.05868	5544.937	5639.401
YPL222W	257.9494	347.083	274.6798	291.0151	258.107	275.9186	-0.09257	134.9133	149.2602
YJL001W	3034.36	2412.771	2626.255	2445.422	2491.194	2380.532	-0.14189	3956.332	3585.756
YGL208W	228.0089	166.1252	179.8263	150.4324	144.0329	164.377	-0.32295	88.81792	71.99609
YAL068C	25.33432	31.64289	13.8328	12.53603	18.43622	11.74122	-0.72926	3.372832	1.756002
YGR215W	611.4783	517.1635	610.6191	556.0627	558.8478	478.4546	-0.1264	408.1127	333.6404
YOR367W	960.401	853.3693	829.9677	872.1498	852.675	779.8125	-0.07798	607.1098	597.0407
YBL022C	2405.609	2306.965	2599.578	2324.539	2465.844	2933.347	0.079002	3291.884	3738.529
YER083C	2009.472	2128.973	1990.935	2027.256	2026.831	1908.926	-0.0397	2975.962	2596.249
YHR104W	1273.625	1130.245	1173.812	983.1833	997.8602	948.1032	-0.28854	2030.445	1699.81
YOR157C	3370.616	3208.787	3100.522	3031.929	3097.284	2775.819	-0.12037	4180.064	4093.241
YPL024W	756.5749	706.0321	728.1979	634.8606	677.5309	483.3467	-0.28688	428.3497	367.8825
YPL036W	126.6716	181.9466	183.7786	198.7857	182.0576	184.9242	0.200388	64.08382	89.55611
YLR019W	1152.711	1414.042	1290.402	1297.48	1239.835	1284.685	-0.01321	771.2544	787.567
YHR087W	4735.214	3965.25	4120.197	2219.774	2189.301	1956.869	-1.01002	3326.737	2528.643
YPL041C	479.0489	439.0452	400.163	440.5521	477.0371	388.4386	-0.01345	234.974	232.6703
YGL259W	0	0	0	0	0	0	#DIV/0!	10.1185	11.41401
YJR025C	80.6092	122.6162	136.3518	138.7918	138.2716	94.90817	0.131453	273.1994	280.9603
YPR184W	2839.747	3099.026	2861.413	2389.01	2374.815	2452.936	-0.28618	2087.783	2230.123
YDR055W	1928.863	2121.063	2047.254	2236.787	2269.959	2075.26	0.110385	1362.624	1343.342
YDR542W	50.66864	27.68753	33.59393	34.02638	32.26338	23.48243	-0.31852	0	9.658012
YML034W	880.9434	825.6818	858.6214	666.2007	761.6462	822.8636	-0.18872	1138.893	1209.885
YIL014W	840.6388	873.1461	761.7918	801.4108	878.0248	1017.572	0.123595	449.711	532.9467
YDR329C	1318.536	1162.876	1267.677	1263.453	1308.971	1173.143	-0.00136	1681.919	1714.736
YIR033W	1269.019	1277.582	1342.769	1384.336	1512.922	1910.883	0.305943	1886.538	2089.643
YIL139C	490.5645	382.6812	387.3183	435.1795	457.4486	406.0504	0.042975	206.8671	252.8643
YDL194W	171.5824	170.0806	164.0174	123.5695	102.5514	138.9377	-0.4701	283.3179	245.8403
YOL103W	1087.073	1346.801	1251.868	1296.584	1307.819	1389.377	0.1158	1743.754	1914.92
YOR051C	5053.045	5073.74	5188.286	5733.445	5133.334	5295.289	0.07766	3783.194	3776.283
YML081C-/	1497.028	1087.724	1308.187	1227.636	1400	1351.218	0.031493	1110.786	824.443
YDR432W	3028.603	4005.793	3758.568	4022.276	3800.165	3855.033	0.113637	4899.601	5271.518
YHR210C	67.94204	49.44202	66.19981	56.41215	57.61317	67.512	-0.01617	105.6821	151.0162
YIL169C	1702.006	1847.154	1821.977	1788.176	1759.506	1967.632	0.038216	1251.321	1486.456
YLR341W	95.57947	105.8059	95.84151	79.69336	77.20165	97.84347	-0.22255	210.2399	165.0642
YFR052W	1767.645	1727.504	1830.869	1684.306	1652.346	1553.754	-0.1231	2377.847	2411.869
YNR076W	2.30312	0	0	0	0	0	#NUM!	8.99422	7.90201
YDR376W	831.4263	855.347	737.0904	748.5803	707.4898	765.1359	-0.12597	517.1676	467.0966
YFL030W	241.8276	442.0117	350.7602	275.7928	315.7202	227.9753	-0.33628	150.6532	107.9941
YGR239C	622.9939	673.4003	596.7863	593.6708	587.6544	483.3467	-0.18557	371.0116	382.8085
YIL116W	1275.928	1315.158	1326.96	1580.436	1542.881	1484.285	0.233882	1848.312	1813.072
YPR133W-/	1691.642	1492.16	1380.315	1378.964	1546.338	1510.703	-0.04107	1278.303	1138.767
YLR174W	140.4903	145.3595	98.80568	119.9878	133.6626	101.7572	-0.11409	44.9711	52.68006
YCR052W	1642.124	1644.442	1597.688	1787.28	1677.696	1738.678	0.091387	2000.09	2137.933
YPR030W	92.1248	96.90636	96.82957	77.00707	89.87655	46.96487	-0.41872	204.6185	214.2323
YBL071C	363.8929	370.8152	287.5245	427.1206	371.0288	309.1854	0.115368	223.7312	173.8442
YLR050C	1938.075	1723.549	1842.726	1789.967	1965.761	1858.048	0.028399	1495.289	1227.446
YPR020W	1788.373	1668.174	1659.935	1588.495	1592.428	1392.313	-0.16194	1237.83	1100.135
YGR038W	910.8839	882.0457	847.7528	785.293	882.6338	769.0497	-0.11582	625.0983	514.5086
YGL150C	1506.24	1290.437	1527.536	1584.017	1479.506	2084.066	0.251459	2213.702	2821.895

YKL094W	2387.184	2593.728	2634.159	2586.005	2433.58	2330.631	-0.05107	1865.176	1708.59
YGR200C	1931.166	2267.411	2050.218	2501.834	2488.889	2541.973	0.269588	1554.876	1713.858
YKL156W	5283.357	4701.936	4638.927	5243.644	5452.511	4904.893	0.093283	4343.084	3562.05
YGL037C	14624.81	13051.7	13793.27	11570.76	10872.76	10013.3	-0.35354	21649.09	19316.9
YHR007C	34531.83	41265.3	38269.42	43312.89	43047.41	41649.03	0.166373	28164.28	28854.63
YGR253C	3231.277	2584.829	3074.833	2981.785	2775.803	2710.264	-0.07034	4544.33	4093.241
YDL192W	13581.5	10437.21	12172.86	12822.57	13132.35	11998.54	0.068578	10042.05	8953.855
YDR261C	1266.716	1119.367	1336.841	1291.212	1329.712	1405.032	0.112895	1837.069	1754.246
YOR076C	404.1975	397.5139	424.8644	435.1795	382.5515	487.2605	0.089404	598.1156	649.7208
YLR377C	194.6136	202.7123	152.1608	137.8964	106.0082	110.5631	-0.63243	84.32081	53.55807
YPL246C	2766.047	3049.584	2556.103	3068.642	3070.782	2406.949	0.029786	2083.286	1754.246
YGR174W-/	12.66716	22.74333	5.928341	9.849741	14.97943	20.54713	0.134441	66.33237	61.46008
YOL155W-/	12.66716	5.933043	11.85668	9.849741	8.065844	5.870608	-0.35664	67.45665	34.24204
YMR209C	982.2806	963.1306	872.4542	963.4838	1018.601	935.3836	0.050114	677.9393	648.8428
YCL057W	1531.575	1668.174	1687.601	1547.305	1533.663	1684.865	-0.03632	2113.642	2221.343
YFR026C	82.91232	79.10723	81.02066	60.88931	63.37449	92.9513	-0.16207	31.47977	20.19402
YOR145C	3818.573	3659.698	3229.958	3859.308	3943.046	3540.955	0.083122	2938.861	2607.663
YHR003C	1706.612	1823.422	1712.302	2026.36	1920.823	1758.247	0.122126	1299.665	1307.344
YAL060W	2265.118	2360.362	2290.316	1969.053	1742.222	1547.884	-0.39506	1830.324	1824.486
YOR019W	275.2228	295.6633	230.2172	262.3613	251.1934	198.6222	-0.16975	151.7775	119.4081
YKL130C	1380.72	1247.917	1129.349	1180.178	1151.111	1079.213	-0.13998	791.4913	726.9849
YGL043W	1515.453	1422.941	1601.64	1592.076	1340.082	1479.393	-0.04142	1961.864	2108.959
YNL244C	11775.85	9933.891	10479.33	11100.66	11621.73	9742.274	0.012299	9054.931	7556.077
YLR400W	72.54828	59.33043	64.22369	87.75224	53.00412	74.36104	0.133517	23.60983	29.85204
YOL126C	1118.165	1427.886	1254.832	1167.642	1041.646	915.8149	-0.28243	1629.078	1814.828
YDR111C	356.9836	368.8375	343.8438	282.0608	306.5021	359.0855	-0.17474	581.2515	546.1167
YLR106C	2433.246	2065.688	2518.557	2342.448	2941.729	2896.167	0.221206	3714.613	4195.967
YLR257W	4911.403	4554.599	4739.709	4215.689	3673.416	3565.416	-0.31055	6804.127	6030.111
YMR165C	686.3297	712.954	693.6159	671.5733	647.5721	739.6966	-0.02367	905.0434	991.2632
YGL073W	594.2049	544.8511	623.4639	479.0556	601.4815	734.8045	0.042602	875.8122	1060.625
YBR273C	1114.71	1131.233	1167.883	1146.152	1049.712	1107.588	-0.04742	1618.96	1452.214
YGR243W	278.6775	325.3285	245.0381	100.2883	71.44034	62.61982	-1.85718	498.0549	341.5424
YOR007C	6794.204	6130.811	6423.357	6614.549	6531.029	6152.397	-0.00376	9097.653	8312.914
YKR061W	611.4783	656.59	606.6669	661.7235	736.2964	575.3196	0.073952	404.7399	425.8305
YKL016C	4648.847	4311.344	4403.769	4036.603	4151.605	3451.918	-0.19924	3439.165	3029.982
YGR130C	2874.294	2489.9	3294.181	2456.167	2545.35	3209.266	-0.07658	3891.124	4646.382
YNR072W	32.24368	9.888404	21.73725	17.90862	16.13169	29.35304	-0.01079	60.71098	93.06811
YBR082C	10243.13	8886.709	9403.337	10029.72	9623.704	9152.278	0.013714	14545.9	13227.96
YLR324W	853.3059	980.9297	936.6779	808.5742	754.7326	671.2062	-0.3104	1216.468	1123.841
YHR196W	2708.469	2915.102	3075.821	3529.789	3789.795	3842.313	0.359596	2456.546	2609.419
YMR103C	242.9791	222.4891	217.3725	150.4324	167.0782	131.1103	-0.60605	406.9884	336.2744
YDR452W	3080.423	2874.559	3283.313	2911.942	2788.478	3175.021	-0.05781	2014.705	2310.021
YEL047C	2402.154	2674.813	2442.476	2447.213	2147.819	2128.096	-0.16149	3282.89	3067.736
YDL131W	2160.326	2182.371	2185.582	2428.409	2371.358	2304.214	0.121925	2829.806	2671.757
YLR145W	602.2658	481.5653	534.5387	527.4089	579.5885	534.2254	0.02023	354.1474	353.8344
YJL108C	994.9478	1005.651	917.9048	580.2393	521.9754	571.4059	-0.80226	1070.312	1037.797
YBR195C	639.1158	672.4115	572.0849	732.4626	757.0371	693.7102	0.212949	498.0549	429.3425
YBR296C-/	17.2734	34.60942	27.66559	11.6406	10.37037	7.827478	-1.41466	0	0.878001
YDR177W	3121.879	2519.565	2756.679	2682.711	2622.552	2405.971	-0.12311	2045.061	1905.262
YLR213C	529.7176	541.8846	464.3867	535.4678	382.5515	386.4817	-0.23567	220.3584	214.2323
YLR114C	1075.557	1028.394	1181.716	1118.393	1182.222	1470.587	0.198839	1557.124	1709.468
YNL062C	2275.482	2354.429	2222.14	2916.419	2772.346	2802.237	0.309399	2076.541	1856.972
YNL035C	613.7814	720.8647	639.2728	702.0179	630.2881	577.2765	-0.04781	390.1243	389.8325
YJL013C	449.1084	447.9447	391.2705	412.7937	418.2716	408.0073	-0.05623	648.7081	616.3568

YER080W	1179.197	1152.988	1262.737	1229.427	1214.486	1036.162	-0.04684	1605.468	1772.684
YBR065C	176.1887	163.1587	236.1456	201.472	171.6873	271.0264	0.162678	294.5607	474.9986
YGL191W	5825.742	5498.942	5538.059	5429.894	5795.885	5123.084	-0.04465	4883.861	4449.709
YBR210W	314.3759	323.3508	341.8677	286.5379	291.5227	323.8619	-0.11918	514.9191	508.3626
YER045C	485.9583	428.1679	536.5149	511.2911	520.8231	522.4841	0.099851	596.9913	732.2529
YFL052W	74.8514	90.97332	67.18786	49.24871	61.06996	46.96487	-0.56704	125.9191	139.6022
YIL051C	5444.575	5438.622	5184.334	5592.862	5431.77	4660.285	-0.03477	8532.142	7224.193
YGL251C	116.3076	149.3149	127.4593	75.21621	88.72429	63.59826	-0.78872	238.3468	187.0142
YHR082C	1158.469	897.8671	1133.301	888.2676	999.0124	1238.698	-0.02908	1629.078	1837.656
YGL058W	1164.227	1106.512	1084.886	1214.204	1107.325	953.9738	-0.03487	1684.168	1588.304
YBR149W	8930.347	8517.871	8168.266	7709.661	7502.387	7913.58	-0.14758	6897.442	6033.623
YDR284C	2020.988	2018.223	1960.305	1905.477	1822.881	1878.595	-0.09763	1412.093	1196.715
YJR008W	307.4665	291.7079	243.062	219.3806	188.9712	162.4202	-0.56131	417.1069	467.9746
YDL167C	2140.75	2107.219	2080.848	2398.86	2737.778	2719.07	0.311806	1553.751	1823.608
YKL182W	24835.69	24832.75	25407.88	27462.87	28024.2	32244.32	0.224735	19013.78	18366.9
YDR071C	2923.811	2618.449	2745.81	2773.15	2778.107	2693.631	-0.00754	4094.619	3616.486
YHL014C	441.0475	480.5765	406.0914	386.8262	450.535	342.4522	-0.17039	286.6908	273.9363
YNL054W	578.0831	580.4493	677.807	553.3764	557.6955	653.5944	-0.05744	782.4971	821.809
YLR450W	1847.102	1405.142	1713.291	1553.573	1700.741	1812.061	0.029005	2607.199	2467.183
YHR202W	482.5036	556.7172	441.6614	441.4475	472.428	542.0528	-0.02452	303.5549	319.5924
YMR140W	219.9479	247.2101	266.7753	218.4852	215.4733	292.552	-0.01467	340.6561	521.5326
YJL174W	4054.643	4357.82	4251.609	4538.94	4317.531	3947.006	0.015794	3275.02	3158.17
YOR342C	2296.211	2368.273	2431.608	2222.46	2494.65	2312.041	-0.01367	1768.488	1770.928
YAL005C	11226.56	9278.29	9842.034	8681.204	7706.338	7767.793	-0.32921	15848.94	14102.45
YGR040W	582.6893	680.3222	641.2489	650.0829	662.5515	672.1846	0.059777	797.1127	925.4131
YER107C	784.2123	764.3737	714.3651	808.5742	744.3622	813.0792	0.064254	1197.356	1024.627
YLR260W	549.2941	558.6948	646.1892	601.7296	694.8149	763.1791	0.231656	894.9249	914.8771
YER033C	176.1887	98.88404	162.0413	104.7654	126.749	168.2908	-0.12871	257.4595	410.9045
YHR106W	673.6626	725.8089	682.7473	692.1682	539.2593	551.8372	-0.2236	1033.211	962.2892
YNL334C	166.9762	135.4711	141.2921	134.3147	168.2305	175.1398	0.106347	79.8237	65.85008
YMR136W	642.5704	676.3669	662.9861	479.951	439.0124	511.7214	-0.4702	831.9653	866.5871
YPL260W	1687.035	1562.368	1608.557	1501.638	1516.379	1475.48	-0.11251	1930.384	2171.297
YBR130C	1429.086	1370.533	1481.097	1307.329	1423.045	1534.186	-0.00546	1870.798	1899.994
YJL047C-/	43.75928	45.48666	58.29535	43.87612	49.54733	36.20208	-0.18677	10.1185	26.34003
YJR112W	880.9434	696.1437	705.4726	769.1753	825.0206	689.7965	0.000905	521.6648	472.3646
YMR053C	102.4888	105.8059	82.00872	95.81112	71.44034	80.23165	-0.23023	163.0202	168.5762
YGR062C	567.719	664.5008	582.9535	619.6383	615.3087	558.6862	-0.01722	408.1127	402.1245
YLR247C	512.4442	523.0966	549.3596	499.6505	578.4363	609.5648	0.090625	717.289	818.297
YOL156W	26.48588	33.62057	19.76114	12.53603	9.218108	1.956869	-1.75205	61.83526	72.87409
YDL098C	344.3164	334.2281	327.0468	311.61	368.7243	305.2716	-0.02896	201.2457	179.1122
YEL019C	160.0668	217.5449	228.2411	203.2628	185.5144	214.2772	-0.00668	70.82948	114.1401
YLR108C	666.7532	712.954	610.6191	589.1936	603.7861	544.0097	-0.19642	816.2254	716.4489
YJR116W	1933.469	2102.275	1847.666	2140.08	2204.28	1862.94	0.077313	1149.012	1197.593
YIL160C	185.4012	240.2882	189.7069	229.2303	160.1646	156.5496	-0.17276	98.93642	84.2881
YDR272W	1620.245	1698.828	1764.669	1557.155	1388.477	1417.752	-0.22044	1186.113	1074.673
YIL053W	25173.1	24811	24697.47	23810.41	22682.31	20860.23	-0.14901	34321.94	29809.89
YKR005C	110.5498	117.672	105.7221	141.4781	132.5103	166.3339	0.398954	76.45087	81.6541
YGL096W	97.88259	101.8506	104.734	79.69336	76.04939	97.84347	-0.26381	137.1619	134.3342
YAL049C	2293.907	2211.047	1966.233	1909.059	1921.975	1843.371	-0.18956	3150.225	2827.163
YIL119C	193.4621	202.7123	159.0771	191.6222	206.2552	265.1558	0.25594	462.078	346.8104
YDR381W	8557.242	8002.686	8624.748	8557.634	8406.914	8606.312	0.021955	11234.9	10728.3
YLR309C	833.7294	723.8312	956.439	896.3265	955.2264	1239.677	0.298196	1437.951	1619.912
YGR085C	25953.86	20244.53	23223.29	26201.21	25978.93	23524.51	0.124996	20170.66	17690.84
YBR223C	99.03415	145.3595	128.4474	121.7786	96.79013	107.6278	-0.19282	207.9913	194.9162

YKR052C	559.6581	772.2844	639.2728	610.684	547.3251	510.7429	-0.24032	349.6503	374.0285
YER158C	588.4471	741.6303	645.2011	641.1286	621.07	722.0848	0.006562	364.2659	400.3685
YHR076W	1649.034	1512.926	1526.548	1584.913	1479.506	1422.644	-0.06336	1122.029	1096.623
YJL203W	188.8558	249.1878	233.1814	186.2497	167.0782	202.536	-0.27206	309.1763	307.3004
YGL077C	3199.034	4477.469	3457.211	4030.335	3756.379	3322.764	-0.00314	2100.15	2245.927
YDR044W	7448.29	6783.445	7618.906	6695.138	6234.898	5909.746	-0.21389	4110.358	4048.463
YBL095W	251.0401	330.2727	240.0978	264.1522	300.7408	188.8379	-0.12405	114.6763	103.6041
YNL024C-/	1119.316	990.8181	1075.006	1088.844	1141.893	987.2406	0.014798	825.2197	689.2308
YML111W	609.1752	685.2664	772.6604	726.1946	773.1688	971.5857	0.257456	1023.093	1200.227
YER154W	2538.038	2917.079	2600.566	2539.442	2604.115	2044.929	-0.16432	1622.332	1877.166
YLR455W	735.8468	744.5968	720.2934	711.8677	732.8396	676.0984	-0.05337	534.0318	539.9707
YOR261C	2434.398	2335.641	2590.685	2406.919	2442.799	2700.48	0.036667	3088.39	3279.334
YNL006W	673.6626	652.6347	730.174	667.9915	636.0494	641.8532	-0.07974	1092.798	923.6571
YPL183W-/	1163.076	1063.003	910.0003	1065.563	1004.774	848.3029	-0.10367	845.4567	725.2289
YLL043W	1688.187	1854.076	1798.263	1790.862	1786.008	1908.926	0.038719	2359.858	2402.211
YMR221C	1383.023	1525.781	1451.455	1513.278	1569.383	1311.103	0.011043	1047.827	1046.577
YAL067C	127.8232	224.4668	151.1727	157.5959	130.2058	148.7221	-0.20582	87.69364	82.5321
YIR020C	19.57652	38.56478	33.59393	34.92181	14.97943	25.4393	-0.28405	77.57515	103.6041
YPR139C	1658.246	1695.861	1682.661	1769.372	1701.893	1783.686	0.061179	1365.997	1311.734
YJR005C-/	47.21396	39.55362	54.34313	17.01319	16.13169	20.54713	-1.39405	83.19653	105.3601
YDR435C	348.9227	430.1456	423.8764	361.7541	368.7243	407.0288	-0.08069	742.0231	610.2107
YKR075C	78.30608	90.97332	62.24758	77.00707	114.0741	69.46886	0.17038	159.6474	155.4062
YLR449W	3948.699	3658.71	3968.036	4369.703	4235.721	4509.606	0.180153	5754.052	5639.401
YKL085W	3960.215	3425.343	3886.027	3562.92	3358.848	3305.152	-0.14032	5781.035	5451.509
YJL167W	16091.9	15541.61	15539.17	16476.83	17070.78	16357.47	0.081235	12539.07	11840.72
YGR189C	5566.641	5725.386	5548.927	6003.865	5889.219	5399.981	0.03822	6841.228	6772.9
YEL011W	2721.136	2766.776	2718.144	1782.803	1625.844	1745.528	-0.67095	2944.483	2863.162
YHR072W	4559.026	6108.067	5213.976	5633.157	5716.379	5616.215	0.095317	3617.925	3813.159
YPR157W	92.1248	127.5604	90.90123	126.2558	130.2058	88.05912	0.149596	269.8266	238.8163
YPL093W	8334.991	8809.579	9059.493	10834.72	11088.23	11803.84	0.364104	7555.145	7762.407
YJL130C	10844.24	11038.43	11544.46	10481.92	10520.17	12463.3	0.00165	13103.45	13930.37
YLR439W	1722.734	1936.15	1664.876	1827.575	1772.181	1654.533	-0.01895	1331.145	1428.508
YKL211C	2105.052	1995.48	1992.911	2090.831	2067.161	2046.885	0.026145	2540.867	2418.893
YOL128C	572.3253	632.8579	487.112	528.3043	604.9383	546.945	-0.01036	355.2717	335.3964
YOR131C	806.092	717.8982	808.2305	868.5681	769.712	660.4434	-0.02087	1222.09	1129.987
YGL125W	821.0622	1129.256	1048.328	873.9407	859.5885	962.7798	-0.15333	557.6416	539.0927
YML124C	3802.451	3723.973	3775.365	3879.007	3850.865	3753.276	0.022967	4663.503	4466.391
YGL035C	2118.87	1925.272	2293.28	1922.49	1819.424	1987.201	-0.14558	2287.905	2814.871
YJL189W	12.66716	18.78797	7.904455	15.22233	14.97943	5.870608	-0.12582	46.09538	35.99804
YDR156W	3004.42	3071.338	2775.452	3268.323	3161.811	2588.938	0.027104	2486.902	2165.151
YOL109W	33612.88	28000.01	32446.8	37095.92	34542.55	29636.79	0.106633	46709.23	44509.39
YLR452C	1167.682	1084.758	1067.101	1118.393	1242.14	1471.566	0.207151	1615.587	1721.76
YGR048W	807.2435	745.5857	717.3293	668.887	652.1811	579.2334	-0.25656	1110.786	906.0971
YJL133C-/	739.3015	804.9161	659.0339	488.0099	413.6626	344.409	-0.82224	478.9422	364.3704
YKL060C	275495.7	223791.4	264985	276603.1	260110.8	264426.9	0.067969	401364.8	354953
YIR025W	231.4635	274.8976	229.2292	194.3085	222.3868	226.0184	-0.19473	382.2543	305.5444
YGR094W	8853.193	8156.945	9078.266	9461.124	9662.881	11528.9	0.232615	12835.88	13272.74
YGR263C	427.2287	393.5585	391.2705	405.6303	346.8313	338.5384	-0.15181	218.1098	235.3043
YGL057C	370.8023	346.0942	347.796	376.9765	334.1564	365.9346	0.016672	194.5	258.1323
YLL061W	2330.757	3542.026	2505.712	3104.459	2773.498	2294.429	-0.03593	1421.087	1377.584
YEL069C	78.30608	64.27463	93.8654	46.56241	41.48148	50.87861	-0.76723	30.35549	21.95003
YBR158W	914.3386	994.7735	976.2001	932.1437	1027.819	1047.904	0.060013	1270.434	1370.56
YHL004W	2350.334	2588.784	2075.907	2000.393	2070.617	1827.716	-0.25004	1685.292	1555.818
YPR004C	1757.28	2073.598	2019.588	1989.648	2011.852	1724.98	-0.0309	1493.04	1424.118

YOR125C	633.358	646.7016	728.1979	623.22	591.1112	554.7725	-0.18293	939.896	863.0751
YMR293C	350.0742	354.0049	369.5333	374.2902	357.2017	309.1854	-0.04495	267.578	217.7443
YMR276W	4643.09	4598.108	4574.703	4975.015	4567.572	4224.881	-0.00507	5904.705	5797.441
YMR105C	1632.912	1550.502	1569.034	857.8229	897.6132	924.6208	-0.82641	2463.292	2138.811
YPL196W	393.8335	357.9602	325.0707	286.5379	299.5885	247.544	-0.36929	544.1503	435.4885
YMR267W	1016.827	1195.508	1113.54	1106.753	1139.589	1071.386	-0.00354	905.0434	847.271
YGR070W	294.7993	209.6342	243.062	137.8964	172.8395	205.4713	-0.53411	381.1301	338.0304
YML057W	2076.263	1893.629	1846.678	1773.849	1771.029	1635.943	-0.16699	1267.061	1304.71
YGL029W	2377.971	2092.386	2049.23	2535.861	2541.893	2138.858	0.146541	1831.448	1841.168
YDR246W	844.0934	726.7977	922.8451	747.6849	788.1482	741.6535	-0.13087	1064.691	1233.592
YBL002W	13104.75	10971.18	12171.87	12816.3	13035.56	12085.63	0.06573	9949.856	8636.897
YOR045W	2512.704	2033.056	2014.648	2199.179	2167.408	1951.977	-0.05419	1779.731	1835.9
YMR127C	199.2199	247.2101	239.1098	239.9755	214.321	236.7812	0.011608	338.4075	415.2945
YEL075C	3.45468	7.910723	6.916398	8.95431	8.065844	12.71965	0.701987	0	0
YMR263W	571.1737	523.0966	413.0078	492.4871	546.1729	472.584	0.003791	806.107	727.8629
YGR117C	464.0787	432.1233	465.3748	462.9378	462.0577	549.8803	0.115315	707.1705	652.3548
YPR013C	941.976	850.4028	948.5346	832.7509	954.0742	846.346	-0.05786	1188.361	1111.549
YMR039C	2563.372	2070.632	2550.175	2445.422	2294.157	2387.381	-0.01154	3418.928	3273.188
YOL052C-/	2215.601	2388.05	2100.609	1198.087	1096.955	959.8445	-1.04247	1307.535	1154.571
YJR063W	2361.849	2071.621	2091.716	2458.854	2484.28	2164.298	0.123309	1837.069	1694.542
YBL033C	906.2777	1122.334	975.2121	1114.812	949.4651	809.1655	-0.06402	1277.179	1338.952
YLR121C	534.3238	539.9069	540.4671	593.6708	636.0494	576.298	0.161548	753.2659	734.8869
YDR185C	442.199	434.101	379.4138	355.4861	336.4609	264.1774	-0.39324	220.3584	213.3543
YBR235W	870.5793	903.8002	933.7137	909.7579	851.5227	814.0577	-0.07252	706.0463	676.9388
YJR059W	1421.025	1264.727	1391.184	1351.205	1325.103	1479.393	0.027607	1716.772	2096.667
YDR244W	488.2614	500.3533	645.2011	541.7358	542.7161	607.608	0.050535	788.1185	723.4729
YHL050C	74.8514	119.6497	143.2682	94.91569	107.1605	186.881	0.203573	34.8526	41.26605
YOL133W	779.6061	588.3601	694.6039	619.6383	591.1112	604.6727	-0.18414	464.3266	387.1985
YBR271W	1029.495	1095.635	1011.77	1112.125	1228.313	1270.008	0.202838	810.6041	784.933
YOR064C	839.4872	879.0791	848.7408	856.0321	754.7326	551.8372	-0.24749	520.5405	445.1465
YCL026C-/	17.2734	13.84377	26.67753	7.163448	16.13169	13.69809	-0.64368	76.45087	63.21608
YFL051C	33.39524	33.62057	57.3073	24.17664	33.41564	54.79234	-0.14565	74.20231	93.94611
YBL102W	770.3936	756.4629	777.6007	770.9661	695.9671	734.8045	-0.06578	1061.318	1122.963
YHR136C	118.6107	142.393	156.113	69.84362	55.30865	66.53356	-1.12171	77.57515	29.85204
YLL040C	2185.661	2139.851	2181.629	2013.824	2006.091	2027.317	-0.10575	1648.191	1599.718
YKL168C	255.6463	333.2392	298.3932	214.008	290.3704	349.3012	-0.05569	483.4393	496.9486
YHR215W	38.00148	48.45318	38.53422	49.24871	17.28395	26.41774	-0.42727	157.3988	79.0201
YGL116W	614.933	635.8244	588.8819	708.2859	705.1852	699.5808	0.199905	834.2139	924.5351
YDR482C	1389.933	1224.184	1271.629	1315.388	1129.218	1023.443	-0.16407	863.4451	823.565
YNL144C	82.91232	91.96216	96.82957	68.94819	63.37449	61.64139	-0.48625	106.8064	165.0642
YOL049W	1829.829	1795.734	1912.878	1813.248	1910.453	2084.066	0.068504	2403.705	2419.771
YBR302C	0	1.977681	5.928341	4.477155	2.304527	7.827478	0.885849	16.86416	28.09603
YDR005C	663.2985	588.3601	762.7799	616.0565	715.5556	688.818	0.004285	1002.856	946.4852
YPL103C	584.9924	752.5076	627.4161	690.3773	648.7243	640.8747	0.011016	474.4451	446.0245
YLR466W	0	0	0.988057	0	0	0	#NUM!	5.621387	8.780011
YMR210W	902.823	921.5993	888.2631	854.2412	754.7326	808.1871	-0.16641	636.3411	590.8947
YPL056C	149.7028	153.2703	134.3757	116.406	122.1399	139.9162	-0.20864	275.448	222.1343
YJL037W	20.72808	7.910723	0	3.581724	6.913581	3.913739	-0.991	69.7052	64.09408
YLR410W	1464.784	1476.339	1579.903	1520.442	1487.572	1755.312	0.075319	1929.26	2151.981
YDR121W	698.9969	582.427	528.6104	674.2596	638.354	582.1687	0.066015	1122.029	950.8752
YJL101C	1110.104	1315.158	1167.883	1228.531	1237.531	1255.332	0.050596	1317.653	1436.41
YGL225W	5864.895	6561.945	6094.335	6604.699	6951.606	5698.404	0.056036	4838.89	4270.597
YBR234C	3838.149	3448.087	3580.718	3568.293	3597.367	3354.074	-0.04685	5318.957	4790.374
YAL009W	571.1737	543.8622	533.5507	565.9124	513.9095	522.4841	-0.04108	391.2486	347.6884

YHR146W	2399.851	1940.105	2570.924	2226.042	2182.387	2249.421	-0.05381	2749.983	3037.884
YNL317W	507.8379	456.8443	496.9926	396.6759	472.428	418.7701	-0.18263	676.815	636.5508
YBR240C	161.2184	219.5226	132.3996	100.2883	109.465	107.6278	-0.69314	24.7341	56.19207
YPL150W	566.5675	585.3935	649.1533	539.0495	550.7819	648.7022	-0.05102	748.7688	818.297
YCL073C	4.60624	7.910723	5.928341	8.95431	13.82716	1.956869	0.423495	0	0
YOL140W	1361.144	1408.109	1174.8	1244.649	1151.111	998.9818	-0.21638	959.0087	809.517
YOR185C	1492.422	1503.037	1402.053	1246.44	1207.572	1039.098	-0.33218	1278.303	918.3891
YDR092W	1454.42	1167.821	1357.59	1386.127	1322.798	1212.281	-0.02141	2033.818	1737.564
YLL042C	57.578	77.12955	69.16398	71.63448	77.20165	60.66295	0.039284	41.59827	24.58403
YDL212W	4086.886	3661.676	3853.422	4110.028	4190.782	3733.707	0.052807	3442.538	2924.622
YGR167W	3742.57	3574.658	3770.425	3639.927	3454.486	3378.535	-0.08229	4986.171	4529.608
YOL123W	3759.843	2899.28	3693.356	3836.027	3915.391	4203.356	0.20761	4789.422	5377.757
YBR035C	2791.381	2275.322	2341.695	2386.324	2367.901	2430.432	-0.04424	3699.997	3222.264
YGR161C	1073.254	1598.955	921.857	590.9845	315.7202	240.6949	-1.64725	1551.503	1417.972
YKR029C	434.1381	454.8666	514.7776	453.0881	546.1729	582.1687	0.17191	600.3642	720.8389
YGL103W	1055.98	1324.057	1132.313	1104.066	1102.716	1048.882	-0.10948	896.0492	784.055
YDL005C	1220.654	1095.635	1176.776	1103.171	1216.79	1193.69	0.008478	1439.075	1676.982
YHR181W	917.7933	943.3538	879.3706	1030.641	1063.539	900.1599	0.12779	799.3613	647.0868
YGL193C	133.581	167.114	143.2682	101.1837	134.8148	103.7141	-0.38613	55.0896	64.97208
YKL073W	1406.055	1295.381	1428.73	1358.369	1540.576	1749.441	0.17053	1819.081	2035.206
YOR005C	604.569	617.0364	652.1175	595.4616	667.1605	546.945	-0.05026	391.2486	460.9506
YER026C	2620.95	3003.108	2428.644	2469.599	2282.634	2206.37	-0.21068	1920.266	1529.478
YPR186C	709.3609	677.3557	650.1414	811.2605	868.8067	833.6264	0.303463	609.3584	528.5566
YGR187C	2262.815	2217.969	2145.071	2526.011	2647.901	2607.529	0.231931	1930.384	1892.97
YCR097W-/	9.21248	10.87724	20.74919	3.581724	19.58848	1.956869	-0.7007	0	0
YPL218W	5643.795	5121.205	4860.252	5158.578	5362.634	4784.546	-0.0298	4457.76	3722.725
YKR019C	264.8588	287.7526	236.1456	307.1328	278.8478	312.1207	0.187297	366.5145	372.2725
YOR313C	193.4621	224.4668	196.6233	183.5634	197.0371	244.6087	0.024803	50.59249	67.60608
YGL148W	7078.639	6495.693	6788.938	7524.307	7134.815	7153.336	0.099183	8866.052	8772.987
YJL180C	742.7562	839.5255	817.123	741.4169	745.5145	640.8747	-0.17331	540.7775	582.9927
YNL015W	3863.484	3865.377	3936.418	4052.721	4100.906	3552.696	0.005067	3431.295	3241.58
YML042W	321.2852	331.2615	277.644	205.0537	236.214	175.1398	-0.59364	415.9827	423.1965
YPL135W	2457.429	3008.053	2402.954	1914.432	1707.654	1506.789	-0.61743	1680.795	1642.74
YDR169C	619.5392	566.6056	670.8906	545.3175	556.5433	717.1926	-0.02981	793.7399	944.7292
YBR173C	2825.928	2488.911	2658.861	2572.573	2495.803	2390.316	-0.09633	3692.127	3422.448
YIL165C	84.06388	126.5716	99.79374	108.3472	70.28807	77.29634	-0.27851	49.46821	47.41206
YOR192C-(	1.15156	1.977681	0	0	3.45679	0.978435	0.503195	13.49133	15.80402
YIL108W	794.5764	922.5881	836.8841	749.4758	780.0824	749.481	-0.16436	924.1561	958.7772
YOR234C	9181.387	8507.983	8411.328	9186.227	9768.89	8483.029	0.072095	7754.142	6872.992
YKL098W	929.3089	1082.78	907.0362	842.6006	758.1894	728.9339	-0.32538	657.7023	561.9207
YNR066C	127.8232	169.0917	204.5278	135.2101	208.5597	183.9457	0.073676	62.95954	81.6541
YDR478W	704.7547	666.4785	634.3325	690.3773	685.5968	586.0824	-0.03164	473.3208	440.7565
YDL182W	3263.521	3065.405	2984.92	3011.335	2976.297	2791.474	-0.0853	4031.659	3910.617
YML060W	619.5392	740.6415	692.6278	722.6128	721.3169	656.5297	0.033106	431.7226	365.2484
YPL123C	517.0504	496.3979	417.948	359.0678	316.8725	375.7189	-0.44476	636.3411	656.7448
YJL031C	368.4992	306.5405	395.2227	468.3104	345.679	403.1151	0.185489	583.5	528.5566
YOL124C	1464.784	1475.35	1647.091	1783.699	1731.852	1728.894	0.193168	1244.575	1319.636
YMR225C	1106.649	974.0078	941.6182	896.3265	879.177	787.6399	-0.23772	790.3671	703.2789
YHL036W	397.2882	550.7841	348.7841	324.146	281.1523	226.9969	-0.63985	453.0838	543.4827
YBR225W	179.6434	212.6007	270.7276	193.4131	233.9095	251.4577	0.033998	301.3064	382.8085
YKR042W	34478.86	45927.68	34706.48	39749.08	39412.02	31459.61	-0.05743	26248.51	26199.55
YML117W	969.6135	965.1083	1189.62	981.3924	1066.996	1290.555	0.095839	1352.506	1638.35
YPR015C	47.21396	54.38622	69.16398	64.47103	50.69959	64.57669	0.073965	92.19075	77.26409
YFL031W	9986.328	11639.64	9607.865	8962.369	8931.194	7584.826	-0.29384	8050.951	6851.042

YOR113W	313.2243	297.641	363.6049	343.8455	345.679	471.6055	0.25284	464.3266	598.7967
YIL113W	138.1872	161.181	191.683	109.2426	125.5967	97.84347	-0.56173	247.341	194.9162
YGL135W	52345.31	44639.22	48337.72	53188.6	49874.57	47868.94	0.054644	45480.4	37203.54
YOL052C	1573.031	1672.129	1561.13	1659.234	1605.103	1558.647	0.005002	1969.734	2135.299
YNL110C	5415.786	5002.544	4898.786	5777.321	5485.926	5264.957	0.109785	4696.107	4466.391
YKR087C	694.3906	808.8715	714.3651	677.8413	732.8396	534.2254	-0.18932	460.9538	500.4606
YNL070W	1294.353	1162.876	998.9255	1087.053	1118.848	1089.976	-0.06851	893.8006	812.151
YGR205W	163.5215	166.1252	227.2531	120.8832	165.9259	127.1965	-0.42777	333.9104	286.2284
YGL026C	3393.647	3496.54	3592.575	3746.483	3470.618	3638.799	0.05046	4174.442	4196.845
YLR390W-/	16610.1	16933.89	15563.87	16125.82	16581.07	13600.24	-0.08472	13160.79	12226.16
YDR279W	346.6195	346.0942	385.3422	417.2709	414.8148	399.2014	0.191735	601.4885	513.6306
YLL015W	1840.193	1860.998	1633.258	1578.645	1424.198	1779.773	-0.15754	1216.468	1231.836
YLR397C	1541.939	1762.114	1482.085	1634.162	1654.65	1586.043	0.026497	1210.847	1142.279
YGR150C	936.2182	1046.193	1006.83	936.6209	1002.469	1134.006	0.039913	710.5434	700.6449
YLR113W	3504.197	3749.683	3597.515	3506.508	3283.951	3303.196	-0.10443	4584.804	4301.327
YBR071W	625.297	681.3111	727.2098	653.6646	663.7038	676.0984	-0.02891	821.8468	783.177
YOL070C	1260.958	1242.972	1150.098	1250.022	1372.346	1280.771	0.095147	1309.783	1548.794
YHR047C	2498.885	2315.864	2395.05	2368.415	2359.836	2689.717	0.041065	3407.685	3227.532
YER042W	1472.845	1456.562	1410.945	1447.017	1535.967	1277.836	-0.02668	1074.809	923.6571
YNR001W-/	2.30312	0	0	2.686293	3.45679	2.935304	1.978847	20.23699	7.90201
YOR230W	8856.647	8961.861	8952.783	8817.309	9295.309	9015.297	0.019092	11670	10773.95
YPR145C-/	51.8202	71.19651	41.49839	48.35328	42.63375	32.28835	-0.41634	19.11272	28.09603
YBL103C	352.3773	465.7438	514.7776	419.9572	445.926	462.7996	-0.00457	583.5	679.5728
YOR205C	994.9478	973.019	998.9255	993.033	966.749	907.9874	-0.04902	700.4249	740.1549
YLR220W	1501.634	1597.966	1393.16	1237.486	1186.831	1100.739	-0.34996	1043.33	816.541
YFL007W	1678.974	1655.319	1810.12	1718.332	1696.132	1941.214	0.058063	2407.078	2262.609
YKR071C	3228.974	3346.236	3165.734	3658.731	3790.947	4012.561	0.234755	2477.908	2611.175
YER064C	1634.064	1664.218	1715.267	1484.625	1455.309	1420.687	-0.2013	1870.798	1974.624
YDR514C	810.6982	1030.372	959.4032	1082.576	1140.741	1112.48	0.252361	726.2833	683.0848
YJL057C	294.7993	274.8976	290.4887	250.7207	247.7366	270.048	-0.16259	378.8815	352.0784
YJR005W	720.8765	661.5342	723.2576	681.423	700.5762	768.0713	0.030106	885.9307	971.9472
YER018C	700.1484	805.905	740.0546	619.6383	758.1894	599.7805	-0.18367	468.8237	457.4386
YBR089C-/	944.2792	708.0098	753.8874	745.894	743.2099	642.8316	-0.17458	544.1503	563.6767
YDR324C	2768.35	3004.097	3055.072	3545.011	3514.404	3915.696	0.314156	2355.361	2564.641
YDL122W	2728.045	2967.51	2775.452	3259.369	3279.342	3606.51	0.260195	2321.633	2485.621
YJR039W	207.2808	195.7904	223.3008	138.7918	203.9506	177.0967	-0.26895	317.0462	286.2284
YGR237C	669.0563	567.5944	612.5952	559.6444	516.214	585.104	-0.15492	744.2717	865.7091
YNL293W	309.7696	381.6924	397.1988	340.2638	361.8107	301.3579	-0.11761	482.315	525.0446
YDL154W	344.3164	395.5362	377.4377	358.1724	380.2469	297.4442	-0.10917	231.6012	183.5022
YBR146W	2819.019	2941.8	2870.305	2750.764	2761.976	2450.001	-0.11628	2593.708	2378.505
YGL192W	507.8379	832.6036	679.7831	561.4353	592.2634	548.9019	-0.24678	350.7746	376.6625
YJR105W	16342.94	17118.81	16102.36	17049.01	16431.28	15212.7	-0.02558	13404.76	12443.91
YJL066C	376.5601	378.7259	418.9361	308.0283	342.2223	296.4657	-0.3107	535.1561	539.0927
YKL161C	157.7637	213.5895	196.6233	179.0862	133.6626	183.9457	-0.19347	276.5723	316.9584
YPL186C	690.936	675.378	603.7027	570.3896	427.4897	435.4034	-0.45888	441.841	417.0505
YPL130W	40.3046	35.59826	39.52227	44.77155	34.5679	24.46087	-0.15315	1.124277	10.53601
YBR076C-/	268.3135	240.2882	249.9784	241.7664	281.1523	243.6302	0.015076	179.8844	151.8942
YDR186C	1085.921	1063.003	1220.25	1096.008	1122.305	1378.615	0.09437	1298.54	1684.884
YIL031W	473.2911	331.2615	474.2673	452.1927	502.3869	641.8532	0.320039	689.1821	940.3392
YPL161C	327.043	291.7079	331.9871	279.3745	267.3251	340.4953	-0.0998	454.2081	475.8766
YDR460W	1067.496	984.8851	809.2185	876.627	789.3005	750.4594	-0.24397	593.6185	568.9447
YNL079C	13939.63	10966.24	12398.14	14022.45	12939.92	12130.63	0.06758	17986.19	16329.06
YOR163W	1335.81	1188.586	1365.495	1313.597	1298.601	1109.545	-0.06375	1024.217	919.2671
YKL010C	1229.866	1237.039	1233.095	1327.924	1213.333	1365.895	0.078592	1476.176	1631.326

YLR435W	1878.194	1736.404	1651.043	1852.647	1960	1660.404	0.055736	1604.344	1423.24
YKL018C-/	469.8365	436.0786	412.0197	422.6434	432.0988	387.4601	-0.08538	301.3064	342.4204
YDR088C	367.3476	381.6924	355.7005	347.4272	364.1153	349.3012	-0.0585	531.7832	505.7286
YBR120C	855.609	728.7754	724.2457	685.9002	699.4239	605.6511	-0.21356	583.5	511.8746
YLR055C	879.7918	1172.765	891.2273	998.4056	1033.58	806.2302	-0.05269	573.3815	609.3327
YGR081C	1646.731	1511.937	1500.858	1860.706	1813.663	1743.571	0.217561	1386.234	1345.976
YER051W	275.2228	285.7749	275.6679	217.5897	253.498	216.2341	-0.28367	359.7688	354.7124
YCR073W-/	1254.049	1200.452	1083.898	1112.125	1123.457	936.362	-0.15773	1585.231	1463.628
YOR220W	1168.833	1350.756	1092.791	971.5427	838.8478	701.5377	-0.52415	1461.561	1402.168
YDL225W	1364.599	1237.039	1163.931	1368.219	1425.35	1399.162	0.155022	1672.925	1705.956
YLR335W	2909.992	2664.925	3193.4	3250.415	3351.934	4073.224	0.283942	4080.003	4366.299
YJL155C	377.7117	332.2504	296.417	277.5836	319.177	312.1207	-0.14701	529.5347	425.8305
YPR188C	1248.291	927.5323	1054.257	1139.884	1019.753	918.7502	-0.0694	831.9653	662.0128
YLR117C	665.6016	651.6458	560.2282	633.0697	583.0453	620.3276	-0.03188	808.3555	874.4891
YOR339C	73.69984	74.16303	83.98483	58.20302	40.32922	48.92174	-0.65291	17.98844	42.14405
YGR037C	9483.096	7963.132	8171.23	8987.441	8563.622	7973.264	-0.00525	7021.113	6211.858
YKL012W	918.9448	939.3984	976.2001	964.3792	966.749	1083.127	0.088685	1260.315	1302.076
YDL113C	204.9777	231.3887	282.5843	216.6943	236.214	275.9186	0.019684	344.0289	418.8065
YJL212C	2893.87	3598.39	3336.668	3283.546	3157.202	3345.268	-0.00631	2233.939	2302.997
YKL088W	688.6328	707.0209	639.2728	643.8149	708.642	799.3812	0.080593	912.9133	993.8972
YDL205C	1013.373	1145.077	952.4868	1079.89	947.1606	920.7071	-0.07773	718.4133	724.3509
YCR104W	27.63744	16.81029	13.8328	16.11776	24.19753	22.504	0.108194	37.10116	32.48604
YHR159W	342.0133	258.0874	375.4616	299.9694	263.8683	373.7621	-0.05726	485.6879	529.4346
YBL084C	360.4383	413.3353	358.6646	389.5125	377.9424	435.4034	0.087034	525.0376	591.7727
YOR216C	907.4292	741.6303	1001.89	836.3326	964.4445	1040.076	0.099815	1332.269	1312.612
YFL044C	519.3535	481.5653	496.9926	500.5459	488.5597	499.0017	-0.00947	713.9162	661.1348
YLR408C	198.0683	214.5784	227.2531	137.8964	135.9671	119.369	-0.70246	114.6763	110.6281
YLR432W	6046.841	5776.806	6124.964	6636.935	6741.894	6901.878	0.176236	5357.182	4951.926
YJR046W	884.398	1035.316	916.9167	958.1112	920.6585	836.5617	-0.06305	1227.711	1253.786
YGR078C	3057.392	2996.187	2540.294	3170.721	2992.428	2511.642	0.013521	2060.801	2072.961
YFL001W	1211.441	1279.56	1116.504	1168.537	1050.864	1146.725	-0.09991	885.9307	888.5371
YBR096W	1460.178	1223.196	1220.25	1211.518	1380.412	1188.798	-0.04615	1043.33	869.2211
YLL055W	753.1202	1160.899	813.1708	745.894	557.6955	542.0528	-0.56329	442.9653	422.3185
YMR173W	299.4056	386.6366	461.4225	416.3754	548.4774	765.1359	0.592313	179.8844	230.9143
YGR029W	791.1217	576.494	633.3444	684.1093	594.5679	589.9961	-0.09868	1168.124	938.5831
YJR103W	1873.588	2146.773	1939.556	2157.093	1903.539	1776.837	-0.02995	2404.83	2619.077
YLR115W	450.2599	574.5163	574.061	484.4282	526.5844	611.5217	0.021226	671.1937	842.003
YHR108W	3201.337	3312.615	3662.727	3802.896	4045.597	4463.619	0.274811	5070.491	4718.378
YGR165W	1822.919	1822.433	1857.547	1752.359	1795.226	1706.39	-0.06678	1581.858	1556.696
YLR095C	1117.013	1109.479	1125.397	1002.883	1228.313	1195.647	0.031906	1423.335	1539.136
YLR072W	920.0964	760.4183	880.3586	854.2412	789.3005	820.9067	-0.05537	641.9624	561.9207
YMR107W	145.0966	201.7234	169.9458	79.69336	76.04939	100.7788	-1.01043	93.31503	61.46008
YDR253C	686.3297	785.1393	693.6159	730.6717	572.6749	551.8372	-0.22286	321.5434	391.5885
YMR081C	74.8514	101.8506	79.04455	77.9025	91.02881	46.96487	-0.24438	129.2919	182.6242
YJL123C	2789.078	2711.4	2753.714	2999.694	2937.12	2756.251	0.074738	3418.928	3596.292
YOR173W	1004.16	1115.412	1125.397	828.2737	755.8848	831.6695	-0.42569	862.3208	731.3749
YGL023C	1336.961	1278.571	1368.459	1390.604	1287.078	1475.48	0.059996	1530.142	1989.55
YDR251W	1478.603	1362.622	1465.288	1271.512	1252.51	1440.256	-0.11946	1653.812	1770.05
YGL117W	347.7711	291.7079	302.3454	321.4597	240.8231	292.552	-0.13981	514.9191	459.1946
YDR116C	1723.885	1796.723	1708.35	1704.901	1689.218	1659.425	-0.04923	1500.91	1471.53
YJL107C	297.1025	382.6812	517.7418	190.7268	259.2593	487.2605	-0.35356	577.8786	786.689
YKL108W	391.5304	365.871	400.163	375.1856	338.7655	441.2741	-0.00292	503.6763	497.8266
YNL056W	1092.83	1039.271	1023.627	1009.151	1035.885	864.9363	-0.11697	1562.746	1375.828
YKL217W	145.0966	202.7123	187.7308	114.6152	107.1605	147.7436	-0.53534	302.4306	331.0064

YLR240W	454.8662	474.6434	539.479	405.6303	459.7531	544.9881	-0.05875	663.3237	654.1108
YDL085C-/	963.8557	945.3315	992.9971	1008.255	951.7696	917.7718	-0.01217	765.633	607.5767
YNL031C	20435.58	18040.4	19611.94	20444.48	20056.3	17778.16	0.004736	15784.86	14182.35
YLL013C	791.1217	711.9651	805.2663	777.2341	834.2388	1070.408	0.216381	636.3411	618.9908
YOL053W	209.5839	260.065	237.1336	182.6679	179.7531	134.0456	-0.50957	291.1879	331.8844
YGR268C	800.3342	747.5634	690.6517	648.2921	630.2881	517.592	-0.31764	966.8786	922.7791
YAR029W	48.36552	60.31927	38.53422	59.99388	50.69959	52.83547	0.151582	26.98266	17.56002
YML129C	1214.896	1057.07	957.4271	856.0321	928.7244	831.6695	-0.30367	843.2081	708.5469
YGL025C	948.8854	885.0122	864.5497	904.3853	925.2676	765.1359	-0.05651	1159.13	1134.377
YKR093W	3661.961	4770.166	3722.01	3449.2	3182.552	3045.867	-0.32872	2484.653	2284.559
YGL245W	9340.303	8449.642	8928.081	9487.092	9508.478	9850.881	0.11058	12644.75	11740.63
YML013W	454.8662	574.5163	558.2521	451.2972	487.4074	600.7589	-0.04445	682.4364	768.2509
YBR025C	11722.88	11022.6	12447.54	12844.06	12979.1	12659.97	0.128936	15803.97	14784.66
YLR246W	551.5972	628.9025	612.5952	556.9581	533.498	499.0017	-0.17392	395.7457	382.8085
YML019W	1090.527	1101.568	1240.011	1035.118	1191.44	1188.798	-0.00706	875.8122	812.151
YBR103W	808.3951	784.1505	812.1827	843.496	770.8643	889.3972	0.058221	1053.448	1080.819
YKL071W	143.945	243.2547	201.5636	220.276	201.6461	251.4577	0.193733	93.31503	136.9682
YKR024C	1366.902	1650.375	1715.267	1834.738	1989.959	2127.117	0.330714	1351.382	1334.562
YFL013C	204.9777	276.8753	328.0349	233.7075	297.284	405.072	0.208884	372.1358	545.2387
YGR196C	452.5631	524.0854	487.112	564.1215	532.3457	664.3572	0.266572	711.6676	781.421
YLR011W	176.1887	158.2145	187.7308	179.9816	165.9259	144.8083	-0.08953	260.8324	296.7644
YKL034W	373.1054	376.7482	376.4497	337.5775	313.4157	322.8835	-0.20978	449.711	418.8065
YCR061W	846.3966	1229.129	1002.878	928.562	871.1112	872.7638	-0.20403	623.974	529.4346
YOR359W	1316.233	1491.171	1488.014	1548.2	1614.321	1690.735	0.176155	936.5231	1034.285
YDR293C	2638.224	2481.989	2778.416	2548.397	2608.724	3330.592	0.103774	3165.965	4325.033
YBR108W	675.9657	612.0922	679.7831	578.4484	543.8684	620.3276	-0.17534	690.3064	854.295
YDL229W	15506.91	16792.49	18525.08	18635.71	18927.08	21810.29	0.224286	23021.83	23557.65
YMR205C	9365.637	9755.9	10110.79	10140.76	10258.6	12037.68	0.150078	12787.53	13832.03
YLR187W	346.6195	312.4736	367.5571	306.2374	282.3045	330.7109	-0.15941	399.1185	515.3866
YIL123W	6574.256	6457.128	6587.375	6559.032	6838.684	6615.197	0.028697	7685.561	7849.33
YIL137C	1424.48	1412.064	1467.264	1421.049	1537.119	1623.223	0.090172	1689.789	1880.678
YHR078W	918.9448	1022.461	1136.265	1002.883	1131.523	1079.213	0.06236	716.1648	779.665
YMR160W	287.89	244.2436	274.6798	221.1715	286.9136	258.3068	-0.07415	357.5202	395.1005
YGL222C	685.1782	760.4183	546.3954	560.5398	561.1523	443.2309	-0.34812	408.1127	368.7605
YGL216W	855.609	795.0277	798.3499	719.9265	745.5145	765.1359	-0.13477	572.2572	518.8986
YGR008C	1669.762	1074.87	1462.324	856.9275	913.7449	972.5641	-0.6169	2683.65	2133.543
YKR022C	502.0801	496.3979	516.7537	466.5196	441.3169	457.9074	-0.14985	760.0116	643.5748
YJL080C	6600.742	6136.744	6591.327	6459.639	6642.799	8207.11	0.140747	8601.847	9090.823
YNL037C	3778.268	4229.271	3750.664	3592.469	3415.309	3178.934	-0.20698	2957.974	2973.79
YMR320W	29.94056	37.57594	21.73725	29.54922	27.65432	41.09426	0.139246	59.58671	43.02205
YBR029C	2472.399	2895.325	2601.554	2893.138	2674.404	2376.618	-0.00455	1789.85	1968.478
YDR306C	575.78	607.148	642.2369	493.3825	530.0412	557.7078	-0.20707	792.6156	730.4969
YOR283W	1454.42	1568.301	1472.205	1604.612	1841.317	1540.056	0.149581	1234.457	1214.275
YPL200W	3.45468	10.87724	6.916398	9.849741	2.304527	15.65496	0.388215	0	0
YPL272C	41.45616	40.54246	35.57005	20.59491	41.48148	44.02956	-0.148	82.07226	79.8981
YIR005W	322.4368	368.8375	278.632	342.9501	278.8478	286.6814	-0.09439	190.0029	209.8423
YNL036W	7844.426	8342.847	7219.731	3253.996	3601.976	3436.263	-1.18538	6482.584	5887.875
YGL045W	195.7652	263.0316	237.1336	223.8578	244.2799	220.1478	-0.01594	349.6503	352.9564
YPR144C	2745.319	3098.037	2538.318	3105.355	3021.235	2901.059	0.107112	2386.841	2225.733
YHR179W	7811.031	8128.268	7843.195	8011.421	7913.746	7977.178	0.007252	9994.827	9556.164
YDR369C	184.2496	197.7681	226.265	187.1451	209.712	270.048	0.132739	360.8931	358.2244
YOL092W	2437.852	2647.126	2602.542	2934.327	2768.889	2304.214	0.058821	2101.275	1942.138
YOR365C	24.18276	53.39738	47.42673	67.15733	42.63375	51.85704	0.370849	91.06648	119.4081
YMR241W	4272.287	4178.84	3967.048	4028.544	3892.346	3444.09	-0.12786	3258.156	2888.624

YJL029C	894.7621	916.6551	857.6333	873.0453	911.4404	980.3916	0.050889	1097.295	1124.719
YNR067C	856.7606	856.3358	937.6659	943.7843	929.8766	1098.782	0.165242	513.7948	577.7247
YLR191W	686.3297	802.9384	735.1143	810.3651	793.9095	722.0848	0.064669	657.7023	506.6066
YJR117W	2517.31	2801.385	2625.267	2697.038	2776.955	2652.537	0.032781	3237.919	3454.934
YPL139C	903.9746	824.6929	871.4661	989.4513	1022.058	1080.192	0.249815	782.4971	592.6507
YNR053C	5398.513	6246.505	5683.303	6615.444	6539.095	6163.16	0.156791	5110.965	4659.552
YKL148C	2272.028	2615.483	2406.906	2095.309	2026.831	1939.258	-0.26714	2809.569	3348.696
YMR082C	5.7578	7.910723	3.952227	1.790862	2.304527	0.978435	-1.79613	26.98266	14.04802
YIR015W	175.0371	237.3217	134.3757	187.1451	168.2305	177.0967	-0.03813	252.9624	313.4464
YPL267W	354.6805	330.2727	340.8796	356.3815	344.5268	356.1502	0.04326	450.8353	424.0745
YPR115W	773.8483	639.7798	801.3141	599.9388	607.2428	831.6695	-0.11951	1015.223	1142.279
YGR121W-/	43.75928	37.57594	26.67753	38.50353	24.19753	21.52556	-0.35885	10.1185	3.512004
YDR398W	4397.807	4321.233	4690.306	5247.226	5167.902	5273.763	0.226504	4071.009	3992.271
YOR106W	906.2777	808.8715	797.3619	846.1823	815.8025	741.6535	-0.06391	1075.934	1060.625
YKR096W	655.2376	621.9806	697.5681	656.3509	580.7408	778.834	0.029746	860.0723	933.3151
YHR007C-/	123.2169	117.672	122.519	166.5502	138.2716	154.5927	0.338206	103.4335	77.26409
YML071C	773.8483	718.887	725.2337	760.2209	786.9959	835.5832	0.103419	891.552	1117.695
YCR030C	2490.824	2153.694	2516.581	2466.912	2396.708	3113.379	0.155665	3172.711	3544.49
YNR013C	1833.283	2194.237	2189.534	2231.414	2348.313	2701.458	0.227942	2575.72	2787.653
YBR156C	441.0475	449.9224	475.2553	493.3825	477.0371	557.7078	0.161569	591.37	642.6968
YIL060W	5.7578	2.966521	4.940284	4.477155	2.304527	10.76278	0.360572	0	0
YEL050C	1702.006	1979.659	1874.344	1993.229	1832.099	1733.786	0.000806	1540.26	1381.974
YHR073W	1067.496	960.1641	1227.167	1044.073	1221.399	1537.121	0.224403	1667.304	1747.222
YDL043C	642.5704	745.5857	682.7473	757.5346	709.7943	763.1791	0.107112	516.0434	518.8986
YBL093C	3025.148	2817.206	2646.016	2979.994	2737.778	2294.429	-0.08329	2099.026	1978.136
YDL091C	559.6581	464.755	503.909	566.8078	498.9301	599.7805	0.124023	773.5029	683.0848
YGL258W-/	0	0	0.988057	0	0	0	#NUM!	5.621387	3.512004
YIL026C	633.358	503.3198	639.2728	548.8992	638.354	907.009	0.237851	803.8584	1090.477
YLR344W	6827.599	5682.866	5852.261	5840.897	6753.416	5848.104	0.006248	5975.535	4691.16
YJL104W	1966.864	1954.938	1984.018	2026.36	2025.679	1860.983	0.001758	1760.619	1709.468
YBR032W	33.39524	28.67637	43.4745	45.66698	25.3498	56.74921	0.27563	19.11272	11.41401
YER084W	88.67012	93.93984	63.23564	51.03957	46.09054	44.02956	-0.80042	116.9249	123.7982
YDR349C	2073.959	2285.21	2267.59	2273.499	2463.539	2308.127	0.08833	1790.974	1768.294
YEL038W	2300.817	2225.88	2109.501	2054.119	2085.597	1823.802	-0.15419	1737.009	1529.478
YDR517W	2237.481	2494.844	2227.08	2250.218	2274.568	2100.699	-0.07094	2960.223	2864.918
YOR340C	3474.256	3569.714	3295.17	4310.605	4133.169	3639.777	0.224928	3131.113	3059.834
YDR305C	644.8736	556.7172	562.2043	550.6901	472.428	461.8212	-0.24828	433.9711	369.6385
YJL151C	1660.549	1460.517	1678.709	1645.802	1560.165	1292.512	-0.09353	2346.367	2028.182
YFL028C	604.569	559.6837	496.0045	644.7103	589.9589	534.2254	0.091441	960.133	838.491
YML020W	621.8424	516.1747	539.479	539.0495	495.4733	535.2038	-0.0958	659.9509	733.1309
YIL101C	84.06388	112.7278	89.91317	81.48422	74.89713	86.10225	-0.24168	159.6474	144.8702
YOR324C	446.8053	462.7773	451.542	495.1734	428.642	374.7405	-0.06789	246.2168	323.9824
YDR289C	643.722	443.0005	538.491	514.8728	444.7737	548.9019	-0.10747	788.1185	733.1309
YKR021W	654.086	643.7351	699.5442	671.5733	699.4239	947.1248	0.214858	881.4335	991.2632
YMR027W	2320.393	2121.063	2123.334	1878.614	1931.194	2036.123	-0.16732	2723	2595.371
YPR108W	2757.986	2528.465	2708.264	2586.005	2575.309	2812.021	-0.00386	3144.604	3242.458
YEL012W	331.6493	361.9156	333.9632	279.3745	296.1317	325.8188	-0.18906	502.552	430.2205
YLR069C	1680.126	2163.583	1872.368	1769.372	1727.243	1760.204	-0.12084	1448.069	1532.112
YJR094C	32.24368	29.66521	44.46256	44.77155	18.43622	17.61182	-0.39633	12.36705	9.658012
YGL136C	482.5036	578.4717	507.8612	512.1865	510.4527	478.4546	-0.06368	319.2948	379.2965
YNL059C	1073.254	954.231	1231.119	1036.909	1160.329	1414.817	0.148566	1534.639	1522.454
YER152C	831.4263	925.5546	746.971	695.7499	781.2346	577.2765	-0.28559	634.0925	435.4885
YJL088W	406.5007	546.8288	377.4377	348.3227	405.5967	305.2716	-0.3293	197.8728	188.7702
YLR060W	8410.994	8061.027	9122.729	9350.091	9622.552	11614.02	0.257055	11973.56	11851.26

YLR154C	1501.634	1114.423	1221.238	1234.799	1274.403	1099.761	-0.08851	1002.856	839.369
YDR148C	2462.035	2398.927	2432.596	2398.86	2427.819	2451.957	-0.00295	2932.116	3057.2
YDL072C	2274.331	1941.094	2199.414	1952.935	2185.844	1780.751	-0.11593	3459.402	2643.661
YHR041C	1546.545	1577.2	1269.653	1592.076	1326.255	1076.278	-0.13728	1050.075	759.4709
YMR279C	97.88259	100.8617	147.2205	139.6872	97.94239	127.1965	0.076584	195.6243	176.4782
YDR030C	301.7087	276.8753	261.8351	288.3288	371.0288	354.1934	0.270238	204.6185	136.9682
YIL076W	2698.105	2178.415	2600.566	2825.085	2554.568	2652.537	0.103317	3698.873	3142.366
YJL201W	618.3877	668.4561	635.3205	639.3378	659.0947	603.6942	-0.01512	432.8468	447.7805
YCR023C	542.3847	709.9874	606.6669	650.0829	573.8272	608.5864	-0.02075	962.3815	847.271
YNL275W	292.4962	312.4736	313.214	194.3085	243.1276	282.7676	-0.35038	403.6156	431.0985
YOR078W	2664.71	2584.829	2699.371	3001.485	2916.379	2720.049	0.119926	2290.153	2353.921
YMR059W	331.6493	307.5294	286.5365	274.8973	282.3045	181.0104	-0.32653	193.3757	189.6482
YDR224C	18193.5	17421.39	16318.75	17185.11	17002.8	15108.99	-0.07517	13778.02	12601.95
YHR069C	1840.193	2298.065	2028.481	2347.82	2166.255	1931.43	0.063786	1593.101	1601.474
YBR087W	723.1796	833.5925	793.4096	900.8036	875.7202	814.0577	0.140504	1081.555	1171.253
YPL172C	476.7458	512.2193	474.2673	495.1734	480.4939	400.1798	-0.08884	287.815	363.4924
YNL138W-/	460.624	391.5808	392.2586	399.3622	392.9218	328.7541	-0.15069	300.1821	236.1823
YMR153W	794.5764	864.2465	784.5171	794.2473	830.782	1014.637	0.111501	974.7486	1112.427
YHR160C	247.5854	165.1364	192.6711	136.1055	161.3169	134.0456	-0.48862	105.6821	80.7761
YDR001C	2357.243	2426.614	2381.217	2069.341	2090.206	2083.088	-0.19883	2853.416	2731.461
YEL006W	1061.738	1147.055	1079.946	1074.517	1126.914	1084.106	-0.00141	905.0434	807.761
YIL090W	1477.451	1847.154	1559.154	1688.783	1617.778	1353.175	-0.06774	1163.627	1226.568
YER136W	2413.67	2511.655	2614.398	2256.486	2310.288	2459.785	-0.10169	2917.5	2952.718
YOR127W	802.6373	855.347	960.3912	914.2351	965.5968	1251.418	0.258067	1286.173	1331.05
YJR126C	1131.983	1266.705	1195.549	1258.976	1366.584	1521.466	0.206392	1524.52	1743.71
YPR128C	1324.294	1410.086	1291.39	1381.65	1466.831	1294.469	0.041394	1155.757	923.6571
YHL015W-/	24.18276	33.62057	45.45061	13.43147	16.13169	14.67652	-1.22278	4.49711	7.90201
YJL038C	71.39672	63.28579	69.16398	89.5431	69.13581	76.31791	0.205158	35.97688	38.63205
YHR068W	4924.07	4554.599	4871.12	5404.822	4745.021	4505.692	0.030416	6753.535	5951.091
YFR004W	2159.175	2266.422	2159.892	2181.27	2090.206	1809.126	-0.11508	2755.604	2814.871
YMR289W	730.089	762.396	680.7712	676.9459	784.6914	694.6886	-0.01128	500.3035	479.3886
YLR387C	1140.044	1115.412	1032.519	1036.909	1043.951	1159.445	-0.02107	1452.567	1423.24
YCL047C	552.7488	548.8064	527.6223	535.4678	600.3293	486.2821	-0.0063	428.3497	331.8844
YLR018C	1842.496	1791.779	1801.228	2101.577	1877.037	1854.134	0.101762	1485.171	1446.946
YML092C	3044.724	2920.046	2916.744	2797.327	2795.391	2701.458	-0.09871	3870.887	3382.938
YDR227W	610.3268	509.2528	724.2457	776.3387	728.2305	1140.855	0.520797	893.8006	1227.446
YBL028C	2307.726	2428.592	2171.749	2663.012	2712.428	2210.284	0.135005	2126.009	1898.238
YLR264W	3998.216	3601.357	3942.347	4541.626	4496.132	4744.43	0.255922	3749.465	3689.361
YFL034C-I	879.7918	956.2087	911.9765	966.1701	966.749	757.3085	-0.03064	1204.101	1200.227
YBR242W	2491.976	2616.472	2345.647	2884.183	2770.041	2323.782	0.097995	2111.393	1758.636
YBR112C	2774.108	2583.84	2847.58	2787.477	2707.819	3294.39	0.099215	3137.858	4098.509
YMR238W	1773.402	2045.911	1800.24	1856.229	1683.457	1602.676	-0.12802	1378.364	1344.22
YMR164C	462.9271	463.7662	553.3118	360.8587	449.3828	525.4194	-0.14805	561.0145	804.249
YKL067W	1308.172	1154.966	1272.617	1053.922	1073.91	1083.127	-0.2184	1808.962	1628.692
YIL088C	1442.905	1538.636	1487.026	1422.84	1400	1241.634	-0.13674	1142.266	1015.847
YOL152W	434.1381	559.6837	484.1478	319.6689	336.4609	350.2796	-0.5544	285.5665	252.8643
YJR119C	188.8558	213.5895	191.683	182.6679	197.0371	237.7596	0.055582	116.9249	139.6022
YIR006C	4948.253	4120.498	4871.12	4784.288	5052.675	5873.544	0.172512	6186.899	6780.802
YBR132C	102.4888	136.46	110.6624	91.33396	61.06996	59.68452	-0.72109	184.3815	156.2842
YKL100C	1145.802	1197.486	1081.922	1069.145	977.1194	1101.717	-0.12177	1408.72	1369.682
YDL040C	2845.505	2579.885	2852.52	2771.359	2854.157	3321.786	0.112186	3493.13	3675.312
YAL033W	536.6269	462.7773	540.4671	547.1084	599.177	524.441	0.117666	420.4798	384.5645
YGR093W	1003.009	1066.959	941.6182	1003.778	1106.173	1181.949	0.128397	786.9942	836.735
YDR043C	831.4263	960.1641	702.5084	666.2007	592.2634	517.592	-0.48984	365.3902	442.5125

YOR380W	505.5348	451.9001	481.1837	430.7023	468.9712	392.3523	-0.15505	304.6792	335.3964
YGL040C	3698.811	4228.282	3800.067	3699.921	3727.572	3322.764	-0.12548	3071.526	2821.017
YDL178W	2137.295	2612.516	2159.892	2157.093	2190.453	2057.648	-0.10938	1588.604	1621.668
YPR028W	10092.27	8484.251	9956.649	10351.18	10592.76	10076.9	0.120598	9279.786	8458.662
YKL202W	21.87964	8.899564	6.916398	9.849741	10.37037	8.805912	-0.37705	42.72254	30.73004
YDR333C	842.9419	908.7444	934.7018	758.4301	884.9383	1091.933	0.026032	1107.413	1275.736
YLR380W	2582.949	2603.617	2662.813	2678.234	2839.177	2665.256	0.059993	3321.116	2871.942
YHR080C	861.3668	815.7934	1075.006	822.0057	829.6297	1107.588	0.003695	612.7312	604.0647
YFR017C	1655.943	1425.908	1423.79	1243.754	1013.992	915.8149	-0.50563	1104.04	918.3891
YHR005C-/	5070.318	4449.782	4421.554	4518.345	4674.733	4345.229	-0.04235	4391.428	4004.563
YNL014W	149.7028	204.69	170.9338	144.1644	106.0082	135.024	-0.44762	73.07804	76.38609
YNR004W	259.101	291.7079	305.3096	281.1653	261.5638	267.1127	-0.08017	191.1272	205.4523
YGR203W	859.0637	856.3358	950.5107	981.3924	872.2634	770.0281	-0.02303	704.922	630.4048
YDR262W	409.9553	536.9404	489.0881	396.6759	414.8148	328.7541	-0.3327	625.0983	575.9687
YDL059C	256.7979	299.6187	225.277	213.1126	229.3004	189.8163	-0.30615	264.2052	345.0544
YDL150W	1087.073	1093.658	1018.687	1447.017	1344.691	1199.561	0.319039	996.1098	885.0251
YGR010W	242.9791	313.4624	273.6917	298.1785	269.6297	267.1127	0.008296	322.6676	356.4684
YDL143W	4904.494	5182.513	5288.08	5627.784	5587.326	5667.094	0.134909	6524.182	6373.41
YNL162W-/	423.7741	496.3979	411.0316	471.8922	475.8848	365.9346	-0.01908	351.8989	312.5684
YKR014C	3310.735	2876.537	3262.564	2795.536	3001.646	2605.572	-0.16943	2346.367	2128.275
YPL080C	202.6745	187.8797	132.3996	230.1258	190.1235	150.6789	0.126626	120.2977	82.5321
YJR085C	1635.215	1567.312	1544.333	1439.853	1414.98	1395.248	-0.15948	1432.33	1210.763
YKR028W	1657.095	1811.556	1702.422	1779.221	1913.91	1940.236	0.123534	1344.636	1453.092
YOR119C	1346.174	1416.02	1393.16	1504.324	1444.938	1635.943	0.142015	1113.035	1179.155
YER100W	1543.09	1523.803	1542.357	1595.658	1477.202	1418.73	-0.03731	2121.512	1808.682
YNL027W	726.6343	710.9763	770.6843	700.2271	685.5968	880.5912	0.037479	1068.064	983.3612
YMR318C	4024.702	4854.218	4086.603	4489.691	4125.103	4016.475	-0.03768	3599.936	3353.964
YER169W	1782.615	1809.578	1822.965	1776.535	1723.786	1768.032	-0.03965	1119.78	1427.63
YBR148W	126.6716	119.6497	124.4952	83.27509	92.18108	99.80034	-0.42993	137.1619	146.6262
YNL125C	1257.503	1461.506	1221.238	1298.375	1292.84	1283.706	-0.02412	1020.844	1078.185
YPR068C	223.4026	247.2101	254.9187	257.8841	223.5391	287.6598	0.084101	156.2746	117.6521
YPR065W	4629.271	5053.963	5518.297	4521.031	4551.441	5308.987	-0.08001	5175.049	6231.174
YCR027C	716.2703	693.1771	608.643	568.5987	573.8272	579.2334	-0.22919	491.3093	520.6546
YPR033C	5078.379	4925.414	4780.219	5202.454	4893.663	5066.335	0.036465	6357.789	5912.459
YAR064W	18.42496	29.66521	34.58199	42.98069	34.5679	26.41774	0.330643	8.99422	15.80402
YKL216W	12501.33	13425.49	13178.7	14085.13	14122.14	13448.59	0.091147	17522.99	15577.5
YLR143W	626.4486	629.8914	674.8428	658.1418	710.9466	754.3732	0.136933	902.7948	906.9751
YFL033C	1122.771	978.952	1220.25	1056.609	1194.897	1337.52	0.111552	622.8497	776.1529
YOR290C	1115.862	1303.292	1237.047	1129.139	1126.914	1298.383	-0.04072	1448.069	1661.178
YJL008C	2717.681	3323.493	3364.333	3168.93	2978.601	3500.839	0.03678	3973.197	3968.565
YOR222W	1684.732	1616.754	1656.971	1683.41	1532.51	1367.852	-0.11336	2103.523	1927.212
YML087C	153.1575	179.969	122.519	131.6284	132.5103	123.2828	-0.23401	60.71098	79.0201
YJR044C	1460.178	1348.778	1418.85	1437.167	1398.848	1284.685	-0.03702	1263.688	1038.675
YBR263W	5753.193	6114	5911.544	6072.813	5436.379	5258.108	-0.0845	4770.309	4787.74
YPL033C	5.7578	7.910723	12.84474	13.43147	9.218108	1.956869	-0.10768	0	0
YNR055C	1324.294	1431.841	1332.889	1380.755	1464.527	1549.841	0.104147	1814.584	1836.778
YDR042C	56.42644	67.24115	73.11621	62.68017	59.9177	36.20208	-0.3094	20.23699	21.07203
YOR004W	2443.61	2375.195	2269.567	2801.804	2737.778	2518.491	0.184981	2264.295	2045.743
YML005W	565.4159	639.7798	624.4519	607.9977	639.5062	649.6807	0.052294	433.9711	415.2945
YLR176C	214.1901	207.6565	250.9664	202.3674	213.1687	253.4146	-0.00831	310.3006	317.8364
YLR178C	4214.709	4159.063	4054.985	3429.501	3275.885	3172.085	-0.33147	6211.633	5396.195
YOR260W	2532.28	2629.327	2605.506	2728.378	2946.338	3001.838	0.159744	3341.353	3541.856
YGR202C	3293.461	3189.999	3214.149	3219.97	2988.971	3049.781	-0.06682	2414.948	2464.549
YDR046C	3720.69	4548.666	3940.371	3892.439	3581.235	3434.306	-0.16265	2494.772	2609.419

YDR104C	333.9524	375.7594	464.3867	413.6891	334.1564	476.4977	0.060455	231.6012	229.1583
YJL210W	561.9613	526.0631	508.8493	458.4607	429.7943	325.8188	-0.39539	362.0173	250.2303
YOR006C	1582.243	1608.843	1440.587	1725.496	1706.502	1501.897	0.091193	1374.991	1249.396
YOR108W	2073.959	2581.862	2321.934	2558.246	2795.391	2582.089	0.185599	1645.942	1830.632
YCL018W	847.5481	756.4629	806.2544	884.6859	713.2511	821.8852	0.005709	1056.821	878.8791
YGR016W	41.45616	48.45318	48.41478	55.51672	47.2428	42.07269	0.06633	119.1734	74.63009
YIL009W	1294.353	1398.22	1561.13	1398.663	1561.317	1714.218	0.135999	1833.697	1847.314
YCR017C	2137.295	2081.509	2246.841	2149.034	2104.033	1992.093	-0.05006	2565.601	2484.743
YEL031W	4943.647	4786.977	5057.863	5009.937	5374.157	6183.707	0.163908	6275.717	6858.066
YCL008C	760.0296	781.1839	801.3141	757.5346	828.4774	753.3947	-0.00192	1030.962	954.3872
YNL065W	938.5214	1385.365	1103.659	1476.566	1285.926	1270.008	0.234498	809.4798	839.369
YKL106W	1296.656	1514.904	1397.112	1514.174	1605.103	1693.67	0.193555	1122.029	1144.913
YNR046W	3499.591	3108.914	3179.567	3753.647	3613.498	3241.554	0.116151	3160.344	2781.507
YDR487C	2524.219	2315.864	2415.799	2522.429	2317.202	2192.672	-0.04515	3777.572	2929.012
YBL091C	4155.98	3822.857	3982.857	4240.761	4301.4	4404.913	0.114204	5557.304	5096.796
YDL017W	191.1589	263.0316	244.05	222.9623	195.8848	237.7596	-0.08869	306.9278	352.0784
YJR096W	2082.02	2013.279	2051.206	1777.431	1682.305	1558.647	-0.29254	2890.517	2748.143
YDR347W	1676.671	1819.466	1841.738	1704.005	1691.523	1520.488	-0.11878	1430.081	1390.754
YPR118W	1670.913	1678.062	1418.85	1618.939	1492.181	1424.601	-0.072	2410.451	1910.53
YOR153W	2891.567	3406.555	3241.814	3067.747	2562.634	3186.762	-0.11367	3481.887	4072.169
YER092W	973.0681	1101.568	920.869	915.1305	727.0782	744.5888	-0.32772	582.3757	662.8908
YJR012C	380.0148	390.592	390.2824	384.1399	349.1358	352.2365	-0.09685	281.0694	199.3062
YCL021W-/	32.24368	21.75449	11.85668	21.49034	24.19753	9.784347	-0.24752	59.58671	66.72808
YJL156C	789.9701	952.2533	953.4748	825.5874	928.7244	1119.329	0.092221	631.8439	623.3808
YFL055W	115.156	208.6453	169.9458	148.6416	111.7696	126.2181	-0.35282	55.0896	68.48408
YPL206C	1331.203	1474.361	1491.966	1207.041	1284.774	1268.051	-0.19283	1719.02	1612.888
YOR213C	278.6775	365.871	422.8883	371.6039	394.0741	384.5248	0.107738	600.3642	563.6767
YAR042W	1059.435	1143.1	1147.134	1198.982	1232.922	1468.631	0.219653	1324.399	1748.1
YMR176W	567.719	590.3377	593.8222	498.7551	565.7614	669.2493	-0.01499	363.1416	414.4165
YKL013C	2422.882	2367.284	2542.27	2183.061	2263.045	2216.155	-0.13828	3472.893	2921.11
YDR264C	1220.654	1344.823	1423.79	1148.838	1243.292	1451.997	-0.05347	1531.266	1708.59
YNL045W	2034.806	1835.288	1747.873	1735.345	1593.58	1738.678	-0.14874	2282.283	2094.911
YLL051C	604.569	627.9137	644.2131	521.1409	565.7614	582.1687	-0.16915	766.7572	805.127
YKR099W	978.8259	959.1752	1111.564	1105.857	1062.387	1303.275	0.186964	761.1359	848.149
YPR146C	111.7013	86.02912	71.14009	74.32078	86.41976	86.10225	-0.12332	55.0896	38.63205
YMR003W	343.1649	310.4959	376.4497	379.6628	424.033	335.6031	0.145347	224.8555	259.8883
YMR194W	8924.59	7059.332	7699.927	8246.92	8547.49	7824.542	0.055866	7629.347	6461.21
YPL214C	864.8215	869.1907	834.908	826.4828	808.889	825.7989	-0.06182	673.4422	575.9687
YKR036C	445.6537	406.4134	404.1152	464.7287	435.5556	466.7134	0.121965	580.1272	649.7208
YDR173C	86.367	114.7055	91.88928	73.42534	103.7037	76.31791	-0.20903	132.6647	168.5762
YFR024C-/	1879.346	2135.895	1836.798	1790.862	1787.161	1537.121	-0.19416	2412.699	2310.021
YKR043C	4134.1	4008.759	4109.328	4197.781	4104.362	3640.756	-0.03689	5078.361	4946.658
YLR349W	8.06092	12.85493	16.79697	20.59491	8.065844	15.65496	0.232764	43.84682	42.14405
YJR146W	82.91232	85.04028	63.23564	70.73905	51.85186	56.74921	-0.36637	25.85838	40.38805
YGR140W	450.2599	434.101	483.1598	490.6962	533.498	580.2118	0.230477	745.396	654.9888
YKL129C	504.3833	513.2082	588.8819	433.3886	429.7943	452.0368	-0.28859	546.3989	743.6669
YLR021W	404.1975	422.2349	386.3302	461.147	445.926	424.6407	0.134987	649.8324	534.7027
YLR173W	454.8662	518.1524	465.3748	491.5916	548.4774	499.9801	0.098518	297.9335	331.0064
YHR034C	902.823	811.838	748.9471	750.3712	709.7943	630.112	-0.23708	604.8613	481.1446
YKL117W	8025.221	7332.252	7702.891	7248.514	7462.058	7114.199	-0.07945	10417.56	9499.972
YKL019W	2284.695	2599.661	2330.826	2510.789	2437.037	2230.831	-0.00732	1959.616	1788.488
YKL204W	1526.968	1523.803	1579.903	1437.167	1700.741	1737.7	0.07436	1848.312	2000.964
YBR192W	884.398	948.298	928.7734	963.4838	914.8972	969.6288	0.044518	774.6272	741.9109
YPR129W	1083.618	1131.233	1266.689	1324.342	1202.963	1320.887	0.144455	1390.731	1626.936

YBR276C	1266.716	1111.457	1255.82	1252.708	1298.601	1397.205	0.119754	1520.023	1624.302
YKL218C	158.9153	226.4445	190.695	223.8578	164.7737	137.9593	-0.12952	402.4913	338.0304
YPR145W	11724.03	11342	12390.23	12410.67	12657.61	13590.46	0.124754	13880.33	14588.87
YKR078W	329.3461	255.1208	278.632	265.943	283.4568	304.2932	-0.01581	380.0058	412.6605
YOL036W	1049.071	1026.416	1070.066	1064.667	1270.947	1300.34	0.20902	1258.066	1535.624
YCR051W	2197.176	2163.583	1986.982	2674.652	2176.626	1670.188	0.038953	3396.442	3075.638
YKL142W	2224.814	1912.417	1945.484	1660.129	1576.296	1591.913	-0.33319	2761.225	2411.869
YBR162W-/	2076.263	1787.824	1614.485	1746.986	1909.301	1518.531	-0.08229	1432.33	1328.416
YMR274C	398.4397	460.7996	401.1511	376.081	417.1194	377.6758	-0.10628	321.5434	251.9863
YMR275C	771.5452	809.8603	703.4965	795.1428	742.0577	1009.745	0.156636	971.3757	1088.721
YMR148W	886.7012	872.1573	767.7202	799.6199	735.1441	647.7238	-0.21121	630.7197	538.2147
YOR239W	2847.808	2334.652	3085.701	3118.786	3303.539	4175.959	0.358192	3723.607	4338.203
YGR161W-(	830.2747	857.3247	784.5171	502.3368	451.6873	295.4873	-0.98438	980.37	892.9271
YBR067C	14047.88	16030.09	13453.38	17568.36	16987.82	13846.81	0.153041	19201.54	16659.19
YJR093C	981.1291	876.1126	841.8244	808.5742	899.9178	836.5617	-0.08476	641.9624	618.1128
YJL122W	4836.552	4998.588	4744.649	6053.114	5840.824	4798.244	0.195203	4787.174	4483.073
YOR316C-/	14.97028	24.72101	11.85668	23.28121	11.52263	24.46087	0.201258	1.124277	2.634003
YER040W	418.0163	326.3173	425.8525	399.3622	345.679	532.2685	0.12637	492.4335	648.8428
YEL032W	1220.654	1301.314	1441.575	1434.481	1305.515	1580.172	0.124297	1023.093	1009.701
YER048C	2713.075	2429.581	2705.3	2597.645	2586.831	2654.493	-0.00165	3572.954	3274.066
YLR193C	1207.986	1105.524	1037.46	892.7447	994.4034	807.2086	-0.31464	803.8584	912.2431
YPR042C	268.3135	345.1053	330.999	316.0872	298.4362	458.8859	0.184703	366.5145	590.0167
YBR219C	32.24368	41.5313	31.61782	61.78474	44.93828	52.83547	0.598309	12.36705	22.82803
YLR165C	297.1025	286.7637	263.8112	250.7207	246.5844	217.2125	-0.24655	156.2746	198.4282
YPL198W	3037.815	3244.385	3505.626	3698.13	3599.671	3607.489	0.155968	3228.925	3089.686
YNL039W	901.6714	867.2131	945.5704	936.6209	1034.733	1075.3	0.166563	1115.283	1354.756
YGR023W	213.0386	182.9355	174.8861	184.4588	177.4486	109.5847	-0.2759	242.8439	332.7624
YIL058W	0	0	0	0	0	0	#DIV/0!	2.248555	2.634003
YDR101C	3888.818	4346.943	3923.574	4658.928	4397.037	4726.818	0.180803	3759.584	3750.821
YGR092W	627.6002	709.9874	637.2967	670.6778	710.9466	715.2358	0.086463	892.6763	804.249
YJL112W	1126.226	1130.245	1074.018	1055.713	1139.589	1216.194	0.034671	921.9075	798.103
YCL025C	2184.509	2846.872	2426.668	1926.072	1848.231	1636.921	-0.46284	2498.145	2329.337
YKR012C	5.7578	4.944202	7.904455	2.686293	13.82716	10.76278	0.551841	0	0
YKR073C	9.21248	15.82145	20.74919	11.6406	14.97943	13.69809	-0.18339	1.124277	7.024009
YER177W	26233.69	19998.31	25582.77	24982.53	23950.95	23738.78	0.017124	20576.53	19560.11
YMR155W	481.3521	481.5653	389.2944	453.0881	475.8848	400.1798	-0.02481	310.3006	302.9104
YJR078W	43.75928	77.12955	83.98483	65.36646	53.00412	49.90017	-0.28395	104.5578	103.6041
YML052W	5646.098	6685.55	5772.228	6187.428	6117.367	5002.737	-0.0649	4517.347	4051.097
YLR074C	4649.999	4841.363	4573.715	5747.772	5710.618	4290.436	0.163127	4213.792	4092.363
YCR046C	2417.124	2189.293	2249.805	2522.429	2243.457	1774.881	-0.06795	1975.356	1711.224
YJR082C	552.7488	661.5342	657.0578	676.0504	591.1112	646.7453	0.032448	883.6821	800.737
YAL062W	389.2273	537.9292	351.7482	385.9308	322.6338	305.2716	-0.33508	247.341	224.7683
YDL219W	616.0846	530.0185	534.5387	539.0495	574.9795	500.9586	-0.05749	845.4567	678.6948
YJL028W	13.81872	7.910723	15.80891	17.01319	8.065844	4.892174	-0.32479	2.248555	1.756002
YLR049C	786.5154	743.608	699.5442	773.6524	881.4816	869.8285	0.179433	505.9249	532.0687
YCR047C	1183.804	1518.859	1036.472	1613.567	1427.654	1156.51	0.166905	982.6185	930.6811
YBL086C	494.0192	415.313	480.1956	424.4343	381.3992	423.6622	-0.17653	575.6301	596.1627
YNL256W	1596.062	1573.245	1704.398	1596.554	1709.959	1611.482	0.013051	1258.066	1326.66
YLR142W	359.2867	579.4605	401.1511	314.2963	297.284	212.3203	-0.70158	214.737	185.2582
YDL015C	3728.751	3658.71	3612.336	3852.144	4068.642	3821.766	0.094269	3309.873	3136.22
YGL017W	460.624	489.476	422.8883	476.3693	410.2058	399.2014	-0.09468	577.8786	548.7507
YML018C	2719.985	3822.857	2967.135	3738.425	3566.255	3136.862	0.134821	2269.916	2540.057
YCR024C-/	5291.418	6319.679	5828.547	5985.061	6619.754	5503.695	0.054297	5198.659	4681.502
YML080W	1285.141	1483.261	1394.148	1596.554	1605.103	1401.119	0.145036	1241.202	1030.773

YBL101C	624.1455	596.2708	694.6039	603.5205	519.6708	558.6862	-0.18729	754.3902	777.031
YNL245C	541.2332	547.8176	537.5029	485.3236	528.8889	493.1311	-0.10981	366.5145	392.4665
YOR198C	9407.093	7351.04	9538.701	9631.256	9800.001	11401.7	0.229584	13065.23	12866.23
YMR292W	762.3327	707.0209	807.2424	873.0453	875.7202	735.7829	0.126105	675.6908	538.2147
YJR130C	606.8721	537.9292	550.3477	476.3693	472.428	577.2765	-0.15159	718.4133	657.6228
YGL226W	522.8082	383.6701	364.593	371.6039	472.428	297.4442	-0.15514	270.9509	260.7663
YBR186W	236.0698	222.4891	160.0652	220.276	230.4527	160.4633	-0.01744	120.2977	112.3841
YDR428C	404.1975	408.3911	527.6223	523.8272	421.7284	453.9937	0.062502	716.1648	641.8188
YML053C	609.1752	522.1077	585.9177	616.0565	583.0453	569.449	0.042509	742.0231	784.933
YJR101W	1579.94	1502.049	1533.464	1441.644	1439.177	1362.96	-0.12112	1333.393	1249.396
YGL252C	1410.661	1527.758	1545.321	1339.565	1571.687	1459.825	-0.03671	1861.804	1758.636
YDR098C	2109.658	1992.513	1966.233	2092.622	2053.334	1823.802	-0.02364	2724.124	2493.523
YPL057C	1173.44	1272.638	1198.513	1129.139	941.3993	961.8013	-0.26533	1238.954	1495.236
YJL145W	2890.415	2484.956	2414.811	2781.209	2604.115	2423.583	0.003464	2108.02	2002.72
YNL147W	1104.346	1028.394	1030.543	968.8564	1107.325	1070.408	-0.00763	923.0318	833.223
YGL105W	10885.7	10515.33	10470.44	11925.35	11312.92	10342.05	0.075351	14303.06	13429.9
YDR280W	1735.401	1857.042	1840.75	2082.773	2052.181	1739.657	0.112693	1552.627	1386.364
YDL236W	2755.683	2838.961	2587.721	2707.783	2697.449	2537.081	-0.04296	2372.225	1972.868
YOR336W	1121.619	1056.082	1131.325	1068.249	1042.798	1216.194	0.00792	683.5607	872.7331
YDL099W	1646.731	1720.582	1951.412	1938.608	1825.185	1776.837	0.05897	2244.058	2184.467
YNL016W	4342.533	4051.279	4541.109	4464.619	4739.26	5513.48	0.186248	5333.572	5945.823
YMR042W	765.7874	913.6886	817.123	835.4371	815.8025	710.3436	-0.08021	620.6012	622.5028
YPR179C	378.8632	365.871	371.5094	353.6953	356.0494	442.2525	0.045485	481.1908	524.1666
YBR079C	8964.894	9153.696	9776.822	10622.5	10313.91	11492.69	0.217261	12473.86	12451.81
YPL070W	445.6537	397.5139	524.6582	453.9835	436.7079	524.441	0.049053	534.0318	675.1828
YGL060W	382.3179	410.3688	376.4497	393.0942	419.4239	401.1582	0.053941	540.7775	546.1167
YKR015C	85.21544	71.19651	103.746	65.36646	42.63375	46.96487	-0.74744	116.9249	99.21412
YDL233W	458.3209	461.7885	412.0197	342.9501	368.7243	291.5735	-0.40906	550.896	542.6047
YNL251C	2017.533	2146.773	2081.836	2095.309	2297.613	2239.637	0.0866	1573.988	1751.612
YMR271C	813.0013	816.7822	793.4096	850.6595	617.6132	536.1822	-0.2737	1372.743	1082.575
YPR029C	1500.483	1283.515	1485.049	1559.841	1376.955	1658.447	0.106227	1738.133	1839.412
YJR033C	488.2614	464.755	532.5626	489.8008	521.9754	565.5353	0.086442	589.1214	705.9129
YGR080W	1411.812	1475.35	1409.957	1550.887	1394.239	1361.981	0.003349	1744.879	1691.03
YBL050W	1893.165	1809.578	1830.869	1563.423	1630.453	1672.145	-0.18548	2223.821	2029.938
YMR273C	658.6923	615.0587	581.9655	631.2789	504.6914	688.818	-0.02425	802.7341	886.7811
YBL051C	1249.443	1265.716	1608.557	1443.435	1534.815	1835.544	0.22323	1645.942	2194.125
YJL129C	1511.998	1449.64	1550.261	1618.939	1632.757	1948.064	0.20471	1174.87	1404.802
YGL179C	168.1278	193.8127	198.5994	150.4324	135.9671	134.0456	-0.4149	267.578	281.8383
YCL014W	733.5437	689.2218	770.6843	768.2798	757.0371	998.9818	0.202681	979.2457	1102.769
YGL169W	1163.076	1312.191	1214.322	1284.944	1353.91	1276.857	0.085814	1106.289	1119.451
YOL082W	1004.16	985.8739	977.1882	733.358	633.7449	838.5186	-0.42793	1133.272	1168.619
YCR076C	679.4204	660.5454	575.0491	680.5276	596.8725	509.7645	-0.09968	953.3873	789.323
YOL122C	1459.026	1863.964	1474.181	1721.018	1718.025	1521.466	0.048304	1170.373	1193.203
YOL142W	1556.909	1538.636	1493.942	1571.481	1744.527	1366.873	0.029064	1301.913	1136.133
YJR006W	918.9448	876.1126	859.6094	880.2087	896.461	804.2733	-0.04063	998.3584	1086.965
YOL105C	413.41	378.7259	349.7721	419.9572	435.5556	377.6758	0.110947	536.2804	507.4846
YGL071W	715.1187	585.3935	659.0339	737.8352	776.6256	847.3245	0.269358	829.7168	1093.111
YPR074C	17102.97	17875.27	17691.16	18711.82	19263.54	20097.05	0.140888	20573.15	21664.68
YGR155W	14127.34	16931.91	14867.29	17166.31	17100.74	15678.44	0.121026	11897.1	12142.75
YHR022C-/	2.30312	2.966521	5.928341	5.372586	8.065844	10.76278	1.111841	34.8526	10.53601
YDL100C	4117.978	3522.25	4088.579	3783.196	4009.877	4201.399	0.032313	5044.633	4906.27
YMR297W	10678.42	11384.52	10609.75	11211.69	10805.93	10947.71	0.012864	10286.01	9558.798
YOL146W	597.6596	546.8288	572.0849	533.6769	472.428	487.2605	-0.20096	423.8526	439.0005
YOR358W	826.82	1045.204	870.4781	832.7509	747.819	682.9474	-0.27693	609.3584	629.5268

YOR018W	532.0207	620.9918	580.9774	462.0424	569.2182	629.1335	-0.06257	332.7861	400.3685
YPL016W	470.988	402.4581	517.7418	440.5521	555.391	679.0337	0.267824	644.211	785.811
YNL124W	2358.395	2863.682	2390.109	2719.424	2612.181	2488.159	0.038814	2072.043	2123.885
YDR297W	3255.46	2953.666	3086.69	2855.53	2748.148	2766.035	-0.1514	2392.462	2329.337
YGL082W	1541.939	1492.16	1649.067	1475.67	1586.667	1439.277	-0.05704	1928.136	1829.754
YOR311C	1469.39	1599.944	1511.727	1628.789	1544.033	1353.175	-0.01745	1231.084	1234.47
YMR121C	1647.882	1618.732	1379.327	1590.286	1530.206	1367.852	-0.04979	1377.24	1108.915
YDL052C	2372.213	2720.3	2329.838	2416.768	2538.436	2009.705	-0.09177	1940.503	1777.952
YKL175W	2369.91	2429.581	2293.28	2191.12	2379.424	2289.537	-0.04812	1898.905	1817.462
YNL237W	271.7681	351.0384	326.0588	291.0151	324.9383	242.6518	-0.14421	187.7543	176.4782
YBL089W	434.1381	565.6167	541.4551	534.5723	564.6091	426.5975	-0.01452	319.2948	377.5405
YJR094W-/	10426.22	8385.367	9300.579	10157.77	10362.31	9556.372	0.097439	9521.506	7916.936
YJL147C	300.5571	230.3998	260.847	188.9359	202.7984	211.3419	-0.3928	116.9249	174.7222
YAR007C	1569.576	1366.577	1487.026	1412.99	1391.934	1816.953	0.063395	1679.671	1927.212
YAL028W	204.9777	144.3707	219.3486	140.5827	124.4445	138.9377	-0.49343	237.2225	262.5223
YMR268C	400.7429	393.5585	382.378	428.016	468.9712	442.2525	0.186693	251.8382	287.9844
YEL045C	5.7578	5.933043	5.928341	5.372586	4.609054	9.784347	0.165873	11.24277	29.85204
YBR061C	1523.514	1636.531	1501.846	1874.137	1775.638	1654.533	0.186249	1421.087	1313.49
YIL033C	3659.657	4054.246	3937.406	3846.772	3675.72	3945.049	-0.02294	4875.991	4918.562
YBR028C	978.8259	1006.64	896.1675	934.83	926.4198	1015.615	-0.00239	733.0289	701.5229
YGR250C	800.3342	770.3067	755.8635	710.9722	738.6009	868.85	-0.00502	1161.379	1271.346
YJR152W	317.8305	349.0607	230.2172	235.4984	241.9753	218.1909	-0.36689	152.9017	189.6482
YMR150C	502.0801	437.0675	509.8373	436.9703	420.5762	438.3388	-0.1611	365.3902	295.8864
YPR025C	659.8438	642.7463	684.7234	673.3641	712.0988	609.5648	0.005589	831.9653	814.785
YAL036C	2510.401	3057.495	2665.777	3093.714	3084.609	2813.978	0.127154	2249.679	2332.849
YDR140W	918.9448	817.771	838.8602	860.5092	888.3951	823.842	-0.00159	710.5434	622.5028
YNL112W	9582.13	10899.99	10471.43	13231.78	13399.67	13788.1	0.384949	10323.12	10401.68
YKL103C	2543.796	2401.893	2935.517	2500.043	2316.05	2776.798	-0.05377	3343.601	3432.984
YBL085W	1697.399	1349.767	1851.618	1650.279	1777.943	2191.694	0.198124	1924.763	2605.029
YLR294C	650.6314	547.8176	547.3835	574.8667	500.0823	468.6702	-0.1776	472.1965	446.0245
YLL011W	1450.966	1625.654	1448.491	1743.404	1607.408	1542.013	0.112715	1182.74	1324.904
YHR147C	1486.664	1378.444	1399.088	1240.172	1116.543	1071.386	-0.31486	1134.396	991.2632
YDR124W	148.5512	165.1364	182.7905	140.5827	179.7531	153.6143	-0.067	174.263	198.4282
YBR011C	26066.71	21985.88	24256.8	25017.45	24301.24	24951.06	0.038592	32587.18	28752.78
YPL119C	79.45764	111.739	114.6146	98.49741	102.5514	107.6278	0.013455	151.7775	180.8682
YOR287C	1247.139	1100.579	1226.179	1370.009	1436.873	1255.332	0.184768	1046.702	908.7311
YGL054C	3037.815	2986.298	3053.096	3175.198	3369.218	2866.814	0.052135	2811.818	2446.989
YBR259W	226.8573	271.9311	298.3932	266.8384	262.7161	266.1342	-0.0027	332.7861	380.1745
YIL161W	549.2941	577.4828	579.9894	590.089	550.7819	523.4626	-0.03632	430.5983	470.6086
YMR219W	886.7012	775.2509	793.4096	826.4828	818.1071	956.9092	0.083408	915.1619	1304.71
YLR354C	13118.57	11884.87	12783.48	13600.7	13015.97	13240.18	0.076941	10682.88	9575.48
YOR095C	3466.195	3713.096	3273.432	3664.104	3831.276	3225.899	0.036598	3164.841	2833.309
YPL021W	6.90936	17.79913	7.904455	12.53603	4.609054	4.892174	-0.5655	3.372832	0
YGR255C	1229.866	1369.544	1312.139	1096.903	1205.268	1118.351	-0.19352	993.8613	952.6312
YLL007C	199.2199	173.0471	214.4083	221.1715	214.321	196.6654	0.107723	249.5896	288.8624
YGR122W	375.4085	453.8778	482.1717	376.081	312.2634	364.9561	-0.31625	521.6648	599.6747
YLL018C	8508.876	8338.891	8393.543	9483.51	9299.918	10333.25	0.206059	10633.42	10874.04
YGL068W	443.3506	390.592	405.1033	389.5125	410.2058	405.072	-0.04045	391.2486	326.6164
YER127W	2444.762	2643.17	2372.324	3069.538	2839.177	2737.66	0.21287	2210.33	2288.949
YML113W	1874.74	2157.65	1868.415	2219.774	2042.963	1602.676	-0.00868	1292.919	1460.994
YGR009C	1232.169	1261.76	1361.542	1306.434	1303.21	1547.884	0.108818	1611.09	1688.396
YPL066W	443.3506	513.2082	556.276	437.8658	463.2099	543.0313	-0.06708	591.37	628.6488
YOR241W	1303.566	1314.169	1380.315	1447.017	1429.959	1387.42	0.093045	1667.304	1540.892
YHR197W	1936.924	2066.677	2044.29	2503.625	2230.782	2502.836	0.259008	2004.587	1889.458

YFL019C	18.42496	13.84377	13.8328	13.43147	4.609054	11.74122	-0.63039	21.36127	36.87605
YML027W	662.147	780.1951	775.6246	901.699	857.284	903.0952	0.263315	878.0607	976.3372
YJL073W	665.6016	707.0209	684.7234	762.0118	887.2429	826.7773	0.267246	873.5636	976.3372
YDR032C	6284.063	6132.788	6055.8	6179.37	5745.186	4971.427	-0.12871	5397.656	4478.683
YPR175W	504.3833	487.4983	518.7298	469.2059	496.6256	569.449	0.02337	630.7197	588.2607
YNL321W	1264.413	1359.656	1453.432	1302.852	1291.687	1698.563	0.074336	1697.659	1862.24
YBL011W	1779.16	1756.181	1739.968	1744.3	1806.749	1758.247	0.009265	2099.026	2184.467
YOR159C	1106.649	1000.707	1092.791	1157.792	1124.609	1003.874	0.038315	872.4393	835.857
YLR034C	1302.414	1523.803	1358.578	1341.356	1375.803	1229.892	-0.08438	1621.208	1607.62
YKR001C	2987.146	2766.776	3050.131	3114.309	3152.593	3602.597	0.164809	3831.538	3721.847
YDR237W	1901.225	1817.489	1915.842	1809.666	1768.724	1634.964	-0.11209	1722.393	1482.944
YOR229W	838.3356	1052.126	837.8722	868.5681	902.2223	849.2813	-0.05841	629.5954	643.5748
YFR029W	437.5928	426.1902	452.53	405.6303	467.819	420.7269	-0.02447	550.896	565.4327
YDL193W	665.6016	606.1592	599.7505	677.8413	686.749	666.314	0.117919	881.4335	718.2049
YNL243W	2805.2	3005.086	3020.49	2844.784	2839.177	3030.212	-0.01918	3300.879	3278.456
YAL025C	5344.39	5456.422	5034.15	6519.633	6085.103	5780.592	0.215442	4996.289	4855.346
YHR207C	684.0266	610.1145	610.6191	646.5012	662.5515	713.2789	0.08641	914.0376	782.299
YOR102W	8.06092	3.955362	7.904455	2.686293	10.37037	2.935304	-0.31692	1.124277	0
YOR337W	784.2123	821.7264	815.1469	707.3905	731.6873	898.2031	-0.05082	1012.974	975.4592
YHL049C	9.21248	0	1.976114	1.790862	2.304527	1.956869	-0.88648	6.745665	7.90201
YKR023W	601.1143	514.197	584.9296	522.9317	450.535	633.0473	-0.08181	685.8093	748.9349
YGR005C	1423.328	1511.937	1636.222	1507.906	1557.86	1620.288	0.03571	1962.988	1821.852
YBR062C	523.9598	425.2014	461.4225	526.5134	480.4939	371.8052	-0.03287	666.6965	575.9687
YLR314C	2440.156	2378.161	2321.934	2440.05	2472.757	2468.591	0.047919	2636.431	2991.35
YER155C	2107.355	1796.723	2498.796	2342.448	2665.185	3509.645	0.411673	3094.012	3646.338
YBR238C	1478.603	1347.79	1816.048	1849.065	1827.49	2556.65	0.425091	2068.671	2506.693
YHR214C-I	0	0	0	0	0	0	#DIV/0!	4.49711	0.878001
YDR399W	5550.519	5377.314	5250.534	6320.848	5667.984	5016.435	0.071916	5260.494	4496.244
YGL240W	47.21396	67.24115	74.10426	40.2944	35.72017	51.85704	-0.56032	75.32659	86.04411
YBR255C-/	313.2243	298.6298	282.5843	276.6882	239.6708	200.5791	-0.31913	213.6127	158.9182
YDR183C-/	36.84992	56.3639	42.48644	50.14414	78.35392	35.22365	0.270822	15.73988	26.34003
YBR269C	777.303	637.8021	637.2967	587.4028	482.7984	381.5895	-0.49948	456.4567	403.8805
YOL096C	543.5363	514.197	472.2912	465.6241	421.7284	381.5895	-0.26993	712.7919	631.2828
YMR199W	795.7279	690.2106	904.072	719.0311	861.8931	1004.852	0.113581	907.2919	1048.333
YPR043W	96.73103	192.8239	134.3757	123.5695	106.0082	113.4984	-0.3053	74.20231	66.72808
YLL049W	498.6255	443.0005	407.0794	431.5978	457.4486	437.3603	-0.02405	477.8179	307.3004
YPL096C-/	168.1278	206.6677	148.2085	147.7461	148.642	178.0751	-0.14053	104.5578	102.7261
YIL006W	154.309	171.0694	201.5636	176.3999	125.5967	161.4417	-0.18527	237.2225	263.4003
YML062C	749.6655	767.3402	768.7082	873.0453	807.7367	727.9554	0.075632	997.2341	1026.383
YJL052W	33477	33094.51	31617.82	33560.75	31339.26	28888.28	-0.06616	63274.34	53099.75
YIL114C	672.511	797.9942	699.5442	610.684	757.0371	585.104	-0.15217	939.896	842.881
YGR036C	494.0192	616.0476	426.8405	422.6434	506.9959	388.4386	-0.22159	269.8266	318.7144
YGR147C	1780.312	1794.745	1800.24	1781.908	1522.14	1444.17	-0.17896	1378.364	1381.974
YDR096W	461.7755	446.9559	506.8732	385.9308	434.4033	515.6351	-0.08353	594.7428	670.7928
YMR246W	11095.28	12894.48	12356.64	13687.56	13658.93	14474.96	0.202431	8436.578	9418.318
YNL183C	751.9686	909.7332	953.4748	843.496	770.8643	979.4131	-0.01186	998.3584	1144.035
YGR152C	673.6626	635.8244	669.9025	641.1286	614.1564	644.7885	-0.059	711.6676	810.395
YFR012W	8.06092	22.74333	7.904455	15.22233	9.218108	13.69809	-0.02141	26.98266	33.36404
YOR225W	8.06092	4.944202	5.928341	16.11776	13.82716	13.69809	1.204812	2.248555	1.756002
YDR223W	259.101	273.9088	168.9577	214.008	218.9301	224.0615	-0.09551	143.9075	99.21412
YFL025C	520.5051	492.4425	478.2195	474.5784	489.712	617.3923	0.085018	632.9682	717.3269
YNR009W	1111.255	1165.843	1232.107	1098.694	1134.98	1082.149	-0.08178	776.8757	900.8291
YKL171W	204.9777	217.5449	266.7753	216.6943	258.107	275.9186	0.123147	288.9393	396.8565
YBR292C	28.789	28.67637	15.80891	16.11776	21.89301	24.46087	-0.23011	4.49711	8.780011

YNL207W	1818.313	2169.516	1784.431	2154.407	2145.515	2076.238	0.143552	1771.861	1600.596
YPL053C	2199.479	2144.795	2165.821	2293.199	2464.692	2581.111	0.172905	2663.413	2731.461
YLR266C	408.8038	370.8152	389.2944	301.7603	284.6091	322.8835	-0.36241	514.9191	466.2186
YBR233W-/	709.3609	618.0253	505.8851	629.488	640.6585	619.3492	0.043581	491.3093	462.7066
YKL007W	1675.52	1659.274	1562.118	1462.239	1466.831	1648.662	-0.09724	2106.896	1848.192
YJL139C	667.9048	683.2887	617.5355	678.7367	700.5762	636.961	0.034427	536.2804	485.5346
YLR164W	190.0074	263.0316	242.0739	148.6416	195.8848	112.52	-0.60491	101.185	76.38609
YPR154W	1444.056	1775.957	1434.659	1289.421	1206.42	946.1464	-0.43544	1966.361	2061.547
YOR094W	887.8527	934.4542	965.3315	947.366	1025.514	926.5777	0.05674	827.4682	724.3509
YEL004W	248.7369	251.1655	257.8828	271.3156	309.9589	248.5224	0.130969	142.7832	184.3802
YCR059C	1467.087	1667.185	1420.826	1693.26	1473.745	1202.496	-0.06001	2041.688	2012.378
YDL170W	506.6864	565.6167	523.6701	450.4018	485.1029	487.2605	-0.16574	564.3873	543.4827
YOR090C	1151.56	1098.602	1073.03	1103.171	1113.087	1111.502	0.001982	1345.76	1352.122
YJL159W	19060.62	22637.52	20135.61	21296.04	21230.45	20245.77	0.021733	25973.06	24734.17
YML112W	587.2956	609.1257	549.3596	578.4484	596.8725	590.9746	0.016854	693.6792	745.4229
YGL118C	33.39524	9.888404	10.86863	8.95431	8.065844	10.76278	-0.96282	6.745665	3.512004
YLR328W	846.3966	930.4988	1004.854	970.6472	1007.078	853.1951	0.025279	1318.777	1090.477
YDR195W	978.8259	892.9229	1015.722	1176.596	1208.724	1266.095	0.338649	789.2428	857.807
YBR185C	779.6061	810.8492	791.4335	750.3712	707.4898	647.7238	-0.17789	720.6619	705.0349
YHL046W-/	0	0	0	0	0	0	#DIV/0!	4.49711	1.756002
YOR306C	773.8483	940.3873	779.5768	790.6656	813.498	864.9363	-0.01437	531.7832	688.3528
YKL138C	830.2747	594.2931	718.3173	710.9722	659.0947	610.5433	-0.11361	593.6185	510.9966
YNL320W	526.2629	570.5609	575.0491	548.8992	600.3293	449.1015	-0.0649	391.2486	386.3205
YMR118C	131.2778	184.9132	150.1846	142.3735	139.4239	115.4553	-0.23144	236.0983	215.9883
YLR351C	2805.2	2693.601	2901.923	3002.38	2771.194	2824.741	0.03354	2548.737	2173.931
YBR022W	500.9286	481.5653	459.4464	558.749	498.9301	442.2525	0.056885	408.1127	342.4204
YGR294W	0	2.966521	0	0	0	0	#NUM!	8.99422	2.634003
YNR007C	384.621	364.8821	340.8796	301.7603	303.0453	227.9753	-0.38883	420.4798	459.1946
YIL045W	233.7667	205.6788	248.9903	136.1055	139.4239	197.6438	-0.54095	296.8093	283.5943
YLR353W	938.5214	860.2912	985.0927	868.5681	1054.321	1233.806	0.181305	654.3295	705.0349
YLR008C	2262.815	2188.304	2085.788	2253.8	2097.12	1883.487	-0.06836	1993.344	1716.492
YKL052C	891.3074	1096.624	987.0688	831.8554	791.605	629.1335	-0.4013	550.896	641.8188
YKL191W	2667.013	3148.468	2944.409	3210.12	3327.737	3041.954	0.129084	2472.286	2636.637
YOR374W	9670.8	11408.25	9661.22	5076.198	4990.453	4396.107	-1.08779	9297.775	9811.662
YBR222C	1518.908	1514.904	1462.324	1474.775	1402.305	1291.534	-0.10912	1639.197	1655.91
YAR033W	9.21248	29.66521	22.72531	24.17664	26.50206	14.67652	0.085302	46.09538	37.75405
YJL024C	1014.524	1114.423	1119.468	1052.131	1098.107	1030.292	-0.03047	874.6879	808.639
YPL215W	1039.859	1090.691	1078.958	1093.321	1003.621	984.3053	-0.05884	854.4509	836.735
YFL040W	49.51708	83.0626	71.14009	44.77155	58.76544	54.79234	-0.36366	17.98844	35.12004
YOR301W	632.2064	700.099	665.9503	659.0372	694.8149	635.0041	-0.0068	731.9046	820.931
YDR273W	133.581	147.3372	147.2205	156.7004	107.1605	95.8866	-0.25109	94.43931	41.26605
YML006C	808.3951	826.6706	853.6811	847.9732	810.0412	833.6264	0.001677	620.6012	673.4268
YBR003W	1355.386	1353.723	1482.085	1293.002	1328.56	1295.448	-0.09761	1158.006	1119.451
YBR110W	671.3594	728.7754	585.9177	627.6971	686.749	560.6431	-0.08294	804.9827	819.175
YGL224C	892.459	780.1951	812.1827	688.5865	825.0206	783.7262	-0.11319	650.9567	525.9226
YPR055W	599.9627	616.0476	685.7114	589.1936	610.6996	809.1655	0.079214	803.8584	886.7811
YDR320C-/	423.7741	397.5139	373.4855	351.9044	444.7737	342.4522	-0.0688	362.0173	277.4483
YFR002W	1567.273	1670.151	1577.927	1681.619	1783.704	1879.573	0.150521	1971.983	2010.622
YDR157W	11.5156	24.72101	13.8328	22.38578	18.43622	13.69809	0.122858	13.49133	8.780011
YDR202C	416.8647	431.1344	398.1869	446.8201	406.749	363.9777	-0.03354	610.4827	490.8026
YHR072W-/	4028.157	3453.031	3401.88	3880.798	3869.301	3587.92	0.059083	3840.532	2929.89
YLR375W	2098.142	2362.34	1972.161	2044.269	1806.749	1619.309	-0.23378	1611.09	1512.796
YNL298W	1013.373	1231.106	1177.764	1191.819	1140.741	1349.261	0.105477	1280.552	1438.166
YJL046W	411.1069	399.4915	442.6495	458.4607	382.5515	372.7836	-0.04615	320.4191	277.4483

YLL032C	476.7458	373.7817	418.9361	418.1663	426.3375	489.2174	0.071238	534.0318	600.5527
YOL157C	52.97176	15.82145	19.76114	26.86293	23.04527	39.13739	0.007981	62.95954	105.3601
YPL230W	230.312	257.0985	231.2053	123.5695	159.0124	144.8083	-0.74967	98.93642	120.2861
YMR098C	748.514	783.1616	840.8364	717.2403	807.7367	836.5617	-0.00669	638.5896	553.1407
YPR027C	452.5631	435.0898	361.6288	327.7278	335.3087	239.7165	-0.4687	246.2168	282.7163
YDR023W	8320.021	8115.413	8429.113	8974.01	9251.523	9707.051	0.167859	10676.14	10671.23
YBL056W	2907.689	3023.874	3101.51	2782.104	2892.181	2681.89	-0.11237	3536.977	3651.606
YOR371C	138.1872	187.8797	200.5755	187.1451	148.642	234.8243	0.115685	216.9856	333.6404
YBR205W	1117.013	1156.943	1283.486	1367.323	1282.469	1257.289	0.135251	1558.249	1543.526
YCR089W	431.835	424.2125	474.2673	474.5784	483.9507	543.0313	0.174695	322.6676	360.8584
YGR050C	201.523	193.8127	176.8622	222.0669	243.1276	169.2692	0.149023	110.1792	118.5301
YOR315W	1297.808	1393.276	1321.032	1646.698	1716.873	1683.886	0.331193	1514.402	1735.808
YDR181C	703.6031	670.4338	794.3977	826.4828	822.7161	813.0792	0.18334	603.737	553.1407
YCR068W	161.2184	279.8418	318.1543	209.5309	222.3868	323.8619	-0.00654	348.526	427.5865
YLR026C	1187.258	1213.307	1221.238	1321.656	1147.654	1136.941	-0.00621	1533.514	1367.926
YER008C	1134.287	1062.015	1164.919	1099.589	1320.494	1631.051	0.269341	1419.962	1693.664
YDR052C	974.2197	1009.606	1077.97	1019.896	912.5927	1092.912	-0.01725	1210.847	1370.56
YNL143C	125.52	104.8171	106.7101	137.0009	86.41976	74.36104	-0.1787	73.07804	61.46008
YKL084W	178.4918	159.2033	163.0294	162.9684	207.4074	196.6654	0.179437	286.6908	257.2543
YNL215W	1050.223	988.8404	1029.555	1208.832	1175.309	1098.782	0.182709	1404.223	1363.536
YPL257W	9.21248	25.70985	18.77308	19.69948	20.74074	17.61182	0.112548	25.85838	37.75405
YGR238C	527.4145	554.7395	473.2792	518.4546	496.6256	495.088	-0.04261	685.8093	613.7228
YLR004C	160.0668	197.7681	156.113	139.6872	110.6173	135.024	-0.41553	113.552	86.92211
YBR196C-I	40.3046	35.59826	43.4745	34.92181	51.85186	51.85704	0.215718	73.07804	74.63009
YAR027W	880.9434	859.3023	788.4693	770.0707	759.3416	638.9179	-0.22182	654.3295	539.0927
YHR200W	3610.14	3310.638	3326.787	3435.769	3389.959	3025.32	-0.05693	4090.121	3998.417
YJR072C	2701.56	2690.635	2709.252	3001.485	3005.103	2773.862	0.116115	3629.168	3172.218
YIL009C-/	494.0192	441.0228	403.1272	343.8455	377.9424	375.7189	-0.28603	607.1098	521.5326
YBR268W	233.7667	185.902	191.683	182.6679	214.321	231.889	0.040778	179.8844	146.6262
YBL039C	4701.819	5162.736	4782.195	5559.731	5681.811	5771.786	0.216085	4463.382	4449.709
YBR262C	842.9419	789.0947	708.4367	658.1418	731.6873	502.9154	-0.30632	592.4942	521.5326
YGR012W	889.0043	806.8938	820.0872	843.496	829.6297	732.8476	-0.0645	1075.934	952.6312
YGR236C	31.09212	47.46434	29.6417	13.43147	17.28395	18.59026	-1.13385	76.45087	65.85008
YNL278W	412.2585	450.9112	450.5539	382.349	472.428	513.6782	0.058887	475.5694	570.7007
YNL064C	7052.153	7617.038	6895.649	7786.668	7177.449	7008.528	0.027028	9357.361	9090.823
YPL011C	583.8409	667.4673	580.9774	640.2332	602.6338	537.1607	-0.04174	699.3006	856.929
YNL287W	3581.351	3413.477	3937.406	3370.402	3956.873	4081.051	0.061499	4483.619	4595.458
YBR094W	839.4872	845.4586	716.3412	912.4442	835.391	879.6128	0.129854	595.8671	664.6468
YBR169C	946.5823	946.3203	940.6301	702.9134	751.2758	693.7102	-0.39968	1174.87	1332.806
YLL018C-/	527.4145	822.7152	567.1446	612.4748	474.7325	411.921	-0.35493	436.2197	445.1465
YIL099W	25.33432	51.4197	42.48644	48.35328	40.32922	44.02956	0.154426	61.83526	76.38609
YPR016C	4746.73	5575.082	5040.078	5303.638	5249.712	4402.956	-0.0386	4430.778	3688.483
YPL265W	2809.806	3893.065	3340.62	2141.871	2212.346	2116.354	-0.6343	2537.494	3068.614
YDR518W	1210.289	1151.999	1060.185	1288.525	1279.012	1256.31	0.159986	1437.951	1352.122
YCL055W	928.1573	1014.55	1016.71	1092.426	1084.28	1051.817	0.125561	860.0723	763.8609
YBR286W	17970.09	17340.31	18943.03	18049.2	18295.64	18398.49	0.012969	22332.65	22524.24
YKL017C	422.6225	347.083	356.6885	346.5318	371.0288	366.913	-0.05472	449.711	468.8526
YAR035W	54.12332	92.951	84.97289	68.05276	43.78601	33.26678	-0.67732	137.1619	114.1401
YBL015W	1138.893	1080.803	912.9645	991.2421	943.7038	693.7102	-0.25306	617.2283	729.6189
YBR280C	236.0698	298.6298	232.1934	195.204	208.5597	183.9457	-0.38392	272.0751	304.6664
YGR209C	8345.355	6158.498	7026.072	7684.589	7854.98	7321.627	0.086558	6945.786	5605.159
YPL120W	587.2956	575.5051	598.7624	526.5134	536.9548	687.8396	-0.00842	405.8642	436.3665
YBL076C	4759.397	5211.189	5483.715	5660.02	5697.943	6772.725	0.230425	6483.708	7066.153
YBR166C	984.5837	931.4877	912.9645	1021.687	865.3499	942.2326	0.000119	1149.012	1067.649

YJR108W	49.51708	71.19651	41.49839	29.54922	26.50206	29.35304	-0.9255	77.57515	57.07007
YDR146C	864.8215	996.7512	910.9884	928.562	912.5927	1142.812	0.106012	1110.786	1230.08
YML122C	127.8232	161.181	130.4235	162.073	193.5803	154.5927	0.282771	109.0549	70.24009
YNL119W	899.3683	847.4363	771.6724	895.431	928.7244	931.4698	0.129828	769.0058	658.5008
YLR438C-/	2192.57	1399.209	1948.448	2009.347	2021.07	1747.484	0.0606	3129.989	2705.121
YDR504C	163.5215	157.2256	100.7818	160.2822	148.642	167.3123	0.176046	101.185	79.0201
YLR352W	504.3833	531.9962	576.0371	460.2515	426.3375	481.3899	-0.23718	592.4942	549.6287
YIL078W	10772.84	10070.35	10660.15	11131.1	11430.45	13128.64	0.180023	14464.95	13407.08
YPR082C	794.5764	560.6725	612.5952	645.6058	695.9671	562.6	-0.04745	449.711	435.4885
YGL095C	848.6997	797.0054	724.2457	733.358	674.0741	760.2438	-0.12871	908.4162	893.8051
YMR154C	94.42792	138.4377	127.4593	140.5827	137.1194	161.4417	0.285395	174.263	205.4523
YGL010W	238.3729	220.5114	220.3367	210.4263	187.8189	152.6358	-0.30214	314.7977	267.7903
YBR039W	4714.486	4366.719	4481.826	4362.54	4491.523	4379.474	-0.03548	4368.942	3911.495
YFR013W	897.0652	917.6439	999.9135	849.764	1001.317	1082.149	0.059549	1172.621	1109.793
YPR124W	4866.492	4580.309	4593.476	4008.845	4073.251	3778.715	-0.24337	4268.882	4123.971
YOL067C	117.4591	164.1475	174.8861	183.5634	169.3827	252.4362	0.407255	204.6185	304.6664
YOR391C	8.06092	19.77681	0	8.95431	11.52263	5.870608	-0.07937	38.22543	28.97404
YNL081C	1733.098	1449.64	1477.145	1409.408	1341.235	1270.987	-0.21251	1405.347	1173.009
YEL050W-/	13.81872	10.87724	5.928341	8.058879	11.52263	12.71965	0.076909	0	5.268006
YER025W	6391.158	6436.362	6745.464	6973.617	7360.659	8152.318	0.200201	8401.726	8272.526
YDR439W	415.7131	337.1946	408.0675	420.8526	425.1852	489.2174	0.201778	565.5116	610.2107
YHR029C	272.9197	177.9913	224.2889	226.544	222.3868	226.0184	-0.00054	385.6272	316.9584
YGR141W	348.9227	354.9937	367.5571	338.4729	338.7655	333.6462	-0.08398	447.4624	468.8526
YBR133C	1204.532	1300.325	1272.617	1259.871	1264.033	1211.302	-0.01623	981.4942	1017.603
YDR049W	909.7324	1025.428	934.7018	922.294	928.7244	903.0952	-0.05939	1201.853	1221.299
YDL065C	952.3401	905.7778	963.3554	980.497	949.4651	918.7502	0.013861	1128.775	1224.811
YIL096C	859.0637	1038.282	927.7854	1074.517	1120	1059.645	0.203968	923.0318	841.125
YLR150W	49233.79	39260.92	47907.91	50280.24	46859.1	49333.66	0.102763	65501.53	58655.74
YLR086W	670.2079	616.0476	792.4216	709.1814	733.9918	925.5992	0.188474	852.2023	979.8492
YDR375C	844.0934	896.8783	952.4868	856.9275	825.0206	787.6399	-0.12519	740.8989	763.8609
YNL148C	490.5645	535.9515	496.0045	589.1936	538.107	362.9993	-0.03086	372.1358	325.7384
YER119C-/	0	1.977681	0	0	2.304527	5.870608	2.047433	8.99422	8.780011
YBL097W	117.4591	150.3037	161.0533	119.0923	134.8148	205.4713	0.099324	218.1098	239.6943
YLR253W	724.3312	822.7152	810.2066	703.8088	729.3828	646.7453	-0.18057	989.3642	995.6532
YDL204W	2772.956	3111.881	3007.645	1993.229	1903.539	1777.816	-0.64807	3373.957	3584.878
YER133W-/	44.91084	34.60942	51.37895	45.66698	58.76544	56.74921	0.300231	82.07226	77.26409
YDL171C	2981.389	3212.743	3212.173	3277.278	3381.893	4106.49	0.194737	3970.948	4025.635
YMR206W	32.24368	19.77681	60.27147	16.11776	16.13169	23.48243	-1.01068	82.07226	53.55807
YNL272C	992.6447	931.4877	1035.484	957.2158	988.6421	1181.949	0.079742	1189.486	1250.274
YML061C	932.7636	993.7846	1099.707	925.8757	1144.198	1213.259	0.117627	690.3064	767.3729
YHL024W	223.4026	315.4401	181.8025	231.9166	169.3827	167.3123	-0.34185	134.9133	122.9202
YGL170C	44.91084	45.48666	35.57005	42.98069	34.5679	42.07269	-0.07458	57.33815	88.67811
YPR133C	2567.979	2570.985	2505.712	2945.968	2733.169	2877.576	0.162601	3180.581	3065.102
YJL193W	544.6879	564.6279	470.315	565.017	587.6544	434.425	0.006802	348.526	358.2244
YBR084W	4655.757	5211.189	4949.177	5428.103	5600	6048.683	0.204869	4643.266	4679.746
YOL160W	0	0	0	0	0	0	#DIV/0!	4.49711	0.878001
YIL157C	813.0013	801.9496	733.1382	677.8413	653.3334	598.802	-0.2829	684.685	696.2549
YOL034W	481.3521	419.2683	451.542	477.2647	492.0165	639.8963	0.251055	619.4769	640.9408
YIR030W-/	3.45468	0.98884	3.952227	7.163448	1.152263	1.956869	0.291068	0	0
YOR272W	3208.246	3597.401	3052.108	3790.36	3722.963	3584.006	0.170881	3203.067	2969.4
YML086C	1678.974	2010.313	1655.983	1825.784	1797.531	1664.317	-0.01564	1400.85	1570.744
YLR041W	2.30312	7.910723	3.952227	4.477155	8.065844	2.935304	0.127808	1.124277	0
YOR124C	1169.985	881.0568	1042.4	998.4056	940.247	1133.027	-0.01018	1164.751	1423.24
YMR240C	571.1737	669.445	670.8906	632.1743	596.8725	633.0473	-0.03779	766.7572	798.981

YMR251W	133.581	175.0248	100.7818	96.70655	64.52675	49.90017	-0.95531	83.19653	64.09408
YPL166W	27.63744	36.5871	58.29535	47.45784	35.72017	45.98643	0.076193	80.94798	98.33612
YJL105W	224.5542	294.6744	221.3247	212.2172	244.2799	240.6949	-0.08705	123.6705	138.7242
YPR187W	3974.033	3626.078	3589.61	4089.433	3978.766	3825.68	0.088045	3716.861	3359.232
YKR056W	2191.419	2198.192	2206.331	2601.227	2846.091	2827.676	0.32718	2097.902	2207.295
YHR028C	1995.653	1640.486	2008.72	1766.685	1918.519	2281.71	0.080047	1517.775	1513.674
YBR129C	801.4857	791.0723	770.6843	689.4819	738.6009	665.3356	-0.17491	609.3584	519.7766
YAL007C	2508.098	2147.761	2436.548	2328.121	2399.013	2240.615	-0.02558	3144.604	2880.722
YKL039W	2377.971	2772.709	2475.082	2471.39	2487.737	2203.435	-0.09041	2145.121	2012.378
YGL021W	830.2747	995.7623	1108.6	1009.151	1049.712	1350.24	0.21621	1172.621	1324.026
YLR295C	2403.306	2698.546	2563.019	2434.677	2425.515	2274.861	-0.10334	2310.39	2297.729
YPL185W	14.97028	16.81029	13.8328	4.477155	6.913581	11.74122	-0.97957	3.372832	4.390005
YLR332W	817.6076	891.9341	775.6246	715.4494	835.391	835.5832	-0.05849	1084.928	1167.741
YNL204C	126.6716	76.14071	93.8654	153.1187	155.5556	144.8083	0.612151	104.5578	94.82412
YGR201C	1017.979	1019.494	1092.791	894.5356	763.9507	741.6535	-0.38317	1363.749	1169.497
YJR083C	456.0177	514.197	464.3867	514.8728	501.2346	536.1822	0.113748	552.0202	580.3587
YGL038C	1540.787	1357.678	1465.288	1387.918	1442.634	1435.364	-0.03271	951.1387	1095.745
YPR122W	1150.408	1136.178	1218.274	1127.348	1118.848	1264.138	0.002251	762.2601	906.0971
YPR196W	247.5854	399.4915	269.7395	307.1328	299.5885	261.2421	-0.079	207.9913	164.1862
YJL165C	1149.257	1072.892	1339.805	1313.597	1317.037	1896.206	0.345836	1578.486	1982.526
YDR217C	548.1425	416.3018	534.5387	546.2129	574.9795	793.5106	0.353136	638.5896	863.9531
YOL063C	418.0163	490.4649	428.8167	413.6891	430.9465	406.0504	-0.0966	628.4711	561.9207
YOR162C	399.5913	390.592	509.8373	404.7348	449.3828	464.7565	0.020772	529.5347	579.4807
YDL235C	1380.72	1220.229	1328.936	1262.558	1458.766	1409.924	0.07209	1196.231	1099.257
YGL066W	602.2658	531.9962	805.2663	623.22	642.963	812.1008	0.099687	816.2254	903.4631
YIL016W	1394.539	1295.381	1370.435	1392.395	1372.346	1173.143	-0.04419	1143.39	1056.235
YOL010W	1875.891	2355.418	1975.126	2310.212	2195.062	1886.422	0.042434	1762.867	1662.934
YGR280C	3994.761	4065.123	4033.248	4881.89	4519.177	4390.237	0.189571	3735.974	3797.355
YGR002C	883.2465	785.1393	773.6485	726.1946	770.8643	775.8987	-0.10351	943.2688	1035.163
YNL258C	845.245	901.8225	938.654	873.9407	851.5227	1108.567	0.077545	1091.673	1191.447
YLL008W	3015.935	3310.638	2922.672	3659.627	3743.704	3872.645	0.285845	2464.416	2916.72
YKR053C	469.8365	440.034	429.8047	550.6901	463.2099	511.7214	0.187514	349.6503	352.9564
YGL175C	36.84992	53.39738	64.22369	51.935	81.81071	91.97286	0.547189	85.44509	115.0181
YML089C	8.06092	20.76565	9.880568	21.49034	5.761317	13.69809	0.081255	6.745665	1.756002
YJR010C-/	735.8468	800.9608	680.7712	627.6971	702.8807	564.5568	-0.22668	594.7428	499.5826
YDL045W-/	1249.443	1208.363	1174.8	1190.923	1259.424	1003.874	-0.07264	1160.254	941.2172
YAR019C	229.1604	279.8418	296.417	249.8253	305.3498	275.9186	0.045271	338.4075	440.7565
YDR006C	1211.441	1329.002	1344.745	1240.172	1340.082	1472.544	0.060934	1621.208	1684.006
YBR147W	73.69984	80.09608	79.04455	66.2619	70.28807	38.15895	-0.41439	94.43931	135.2122
YJL110C	1332.355	1421.953	1464.3	1443.435	1348.148	1327.736	-0.03436	1162.503	1109.793
YOR062C	389.2273	499.3644	384.3541	318.7734	337.6132	259.2852	-0.47527	256.3353	188.7702
YLR132C	162.37	254.132	156.113	123.5695	140.5761	139.9162	-0.50299	195.6243	206.3303
YDR391C	1128.529	903.8002	909.0123	890.0584	963.2923	890.3756	-0.10034	974.7486	852.539
YBR167C	494.0192	580.4493	524.6582	529.1997	523.1276	454.9721	-0.08532	417.1069	373.1505
YKL096W	24016.93	21622.97	22157.17	17703.57	18031.77	15362.4	-0.40796	25355.83	22975.53
YLR430W	1362.295	1376.466	1528.524	1547.305	1516.379	1576.258	0.120788	1151.26	1272.224
YML041C	327.043	305.5517	278.632	273.1065	284.6091	263.1989	-0.15058	476.6937	316.9584
YER058W	651.7829	574.5163	467.3509	530.0952	510.4527	426.5975	-0.20712	477.8179	336.2744
YDR379C-/	475.5943	429.1567	424.8644	325.0415	402.14	362.9993	-0.28644	348.526	303.7884
YGR090W	2564.524	2508.688	2895.995	2965.668	3268.971	3737.621	0.323484	2257.549	2430.307
YOR092W	700.1484	670.4338	898.1437	791.561	829.6297	878.6344	0.139945	948.8902	1041.309
YKL135C	753.1202	768.329	882.3347	805.8879	813.498	1106.61	0.181476	953.3873	1109.793
YIL129C	928.1573	944.3426	1077.97	973.3335	967.9013	1179.014	0.080716	1226.587	1280.126
YKL137W	792.2732	862.2689	777.6007	749.4758	735.1441	653.5944	-0.18582	792.6156	708.5469

YJL176C	910.8839	886.9899	876.4064	1019.896	823.8684	1101.717	0.139353	1156.882	1237.104
YHR171W	162.37	186.8908	173.898	119.9878	161.3169	152.6358	-0.26975	248.4653	209.8423
YPR006C	138.1872	153.2703	113.6265	77.00707	138.2716	85.12382	-0.43132	212.4884	168.5762
YJL124C	3121.879	2869.615	3142.021	3101.773	3136.461	2882.469	-0.00203	3730.353	3679.703
YHR107C	2334.212	2123.04	2412.835	2592.273	2480.823	2467.612	0.134372	2718.503	2817.505
YDL056W	707.0578	827.6594	891.2273	766.489	808.889	1030.292	0.103108	898.2977	1115.061
YNL053W	917.7933	972.0301	931.7376	900.8036	997.8602	888.4187	-0.01774	733.0289	682.2068
YPR149W	10252.34	11532.85	10803.41	9997.487	10582.39	8957.57	-0.14182	8750.252	8655.335
YPL248C	222.2511	198.7569	192.6711	151.3278	195.8848	150.6789	-0.30165	245.0925	272.1803
YPR141C	431.835	418.2795	462.4106	422.6434	433.2511	565.5353	0.114998	554.2688	591.7727
YOL166C	2.30312	5.933043	0	0	6.913581	4.892174	0.519446	0	0
YBL074C	139.3388	158.2145	161.0533	148.6416	138.2716	200.5791	0.088123	50.59249	85.1661
YHL001W	15845.46	13391.87	14676.6	16100.75	15651.19	13304.76	0.037063	14102.94	12332.4
YJL070C	259.101	238.3105	319.1424	232.8121	231.605	272.0049	-0.14902	305.8035	421.4405
YBL016W	3763.298	4352.876	3872.195	4292.696	4193.087	3625.101	0.014669	3280.642	2958.864
YHR008C	19485.55	15443.71	19183.12	20128.39	19026.17	18782.03	0.098516	16032.2	15176.25
YDR527W	1725.037	1896.596	1790.359	1764.895	1756.05	1674.102	-0.05902	1517.775	1376.706
YKL186C	2147.659	1975.703	2083.812	2053.223	2040.659	1744.549	-0.08836	2548.737	2364.457
YDR402C	24.18276	20.76565	15.80891	31.34009	12.6749	28.37461	0.252724	4.49711	10.53601
YPL252C	596.508	554.7395	518.7298	615.1611	530.0412	455.9506	-0.06072	478.9422	459.1946
YGR179C	340.8617	316.4289	347.796	274.0019	274.2387	352.2365	-0.15856	389	463.5846
YGR089W	1611.032	1600.933	1886.2	1738.927	1656.955	1966.654	0.072937	1223.214	1444.312
YGL166W	221.0995	265.9981	231.2053	185.3542	171.6873	137.9593	-0.53716	136.0376	133.4562
YNL242W	234.9182	298.6298	237.1336	189.8314	205.1029	226.9969	-0.30938	326.0405	310.8124
YKL064W	1425.631	1361.633	1579.903	1306.434	1380.412	1931.43	0.080656	1824.702	2128.275
YML130C	1748.068	1839.243	1919.794	2350.506	2289.548	2480.332	0.370661	2488.026	2626.979
YKL155C	841.7903	911.7109	800.326	815.7377	770.8643	791.5537	-0.10282	623.974	719.0829
YHR011W	344.3164	425.2014	325.0707	368.9176	362.963	356.1502	-0.00867	240.5954	282.7163
YLR278C	842.9419	827.6594	884.3109	892.7447	783.5392	973.5425	0.052624	938.7717	1196.715
YLR224W	1408.358	1518.859	1475.169	1328.82	1346.996	1240.655	-0.16873	1288.422	1129.109
YOR392W	0	0	0	0	0	0	#DIV/0!	0	4.390005
YHR149C	1665.156	1785.846	1823.953	1935.922	2019.918	2167.233	0.215097	1273.806	1495.236
YGL250W	382.3179	395.5362	303.3334	313.4009	347.9836	283.7461	-0.19403	540.7775	484.6566
YJR121W	13599.92	13862.55	14041.28	12841.38	13223.38	13404.56	-0.07251	13055.11	13546.68
YML022W	4664.969	4574.376	4484.79	5115.597	5028.478	4104.534	0.054106	4502.731	3784.185
YOR370C	2717.681	3144.513	2867.341	3098.191	3192.922	3277.756	0.132444	2521.754	2428.551
YMR261C	2194.873	2019.212	2128.274	1745.195	1767.572	2238.659	-0.1411	2556.607	2624.345
YPL088W	963.8557	868.2019	877.3945	1075.413	971.3581	854.1735	0.098521	1297.416	1102.769
YER130C	696.6938	829.6371	741.0426	833.6463	717.8601	708.3867	-0.00477	566.6358	561.0427
YLR183C	814.1529	797.0054	1041.412	990.3467	970.2058	1095.847	0.204442	1086.052	1127.353
YDR089W	903.9746	796.0165	840.8364	794.2473	944.856	1078.235	0.149034	902.7948	1055.357
YHR131C	356.9836	390.592	430.7928	367.1267	309.9589	442.2525	-0.07414	439.5925	579.4807
YGL232W	1256.352	1210.341	1225.19	1284.944	1426.502	1372.744	0.145693	1196.231	1184.423
YFL064C	5.7578	2.966521	3.952227	1.790862	2.304527	6.849043	-0.21196	0	0
YPL132W	770.3936	815.7934	797.3619	822.0057	811.1935	703.4946	-0.02864	654.3295	620.7468
YOR052C	1515.453	1821.444	1333.877	1112.125	925.2676	820.9067	-0.7085	1533.514	1377.584
YNL175C	1802.191	1721.571	1684.637	2004.87	2086.749	1882.508	0.197888	2710.633	2221.343
YHR040W	1492.422	1560.39	1336.841	1715.646	1681.152	1375.679	0.120632	1235.581	1304.71
YFL045C	15745.28	13992.09	15167.66	15024.44	14879.18	14376.14	-0.02023	13900.57	12671.31
YKR026C	2270.876	2342.563	2166.809	2265.44	2303.375	2047.864	-0.03523	1962.988	1705.078
YOL104C	6.90936	0.98884	9.880568	8.058879	4.609054	12.71965	0.513968	38.22543	10.53601
YGR109C	184.2496	154.2591	166.9816	125.3603	144.0329	193.7301	-0.12629	245.0925	185.2582
YKR016W	2193.722	2149.739	2316.993	2364.833	2302.222	2620.248	0.129765	1997.841	2058.913
YBR017C	2075.111	2207.092	2209.295	2183.956	2280.329	2488.159	0.098969	2586.962	2625.223

YKL109W	825.6685	841.5032	807.2424	779.025	818.1071	949.0817	0.041267	1082.679	1303.832
YKR034W	14.97028	13.84377	7.904455	2.686293	2.304527	3.913739	-2.04389	0	3.512004
YOR199W	52.97176	60.31927	82.99677	75.21621	93.33334	108.6063	0.497727	26.98266	56.19207
YPL108W	641.4189	588.3601	579.9894	692.1682	689.0536	476.4977	0.037728	372.1358	482.9006
YOR187W	5716.344	6528.325	6248.471	5949.244	5847.737	5471.407	-0.09886	5856.361	5702.617
YNL259C	442.199	410.3688	317.1662	327.7278	362.963	422.6838	-0.07124	413.7341	262.5223
YLR301W	3216.307	2746.999	2949.35	3039.093	2897.943	2940.196	-0.00575	3714.613	3360.988
YLR182W	831.4263	935.4431	945.5704	908.8625	981.7285	1218.151	0.19674	1137.769	1253.786
YBR246W	1244.836	1260.772	1221.238	1251.813	1194.897	1265.116	-0.00583	1051.199	1040.431
YPL146C	2814.412	2349.485	2552.151	2920.896	2979.753	3260.144	0.24761	2346.367	2500.547
YDR476C	1665.156	1656.308	1417.862	1431.794	1538.272	1255.332	-0.16559	2056.304	1820.974
YNL103W	1221.805	1279.56	1279.534	1289.421	1141.893	1298.383	-0.01967	867.9422	985.9952
YHR161C	1173.44	1089.702	1060.185	844.3915	872.2634	894.2893	-0.34806	1032.087	1251.152
YIL152W	497.4739	383.6701	462.4106	440.5521	395.2264	365.9346	-0.16096	555.3931	552.2627
YOL012C	2502.34	2261.478	2391.098	2400.651	2306.831	1888.379	-0.11737	1945	1784.098
YNL089C	25.33432	21.75449	8.892511	17.01319	14.97943	14.67652	-0.26248	6.745665	3.512004
YPL105C	2741.864	2629.327	2869.317	2946.864	2816.132	3336.462	0.143047	3091.763	3531.32
YKL025C	898.2168	800.9608	824.0394	736.9397	748.9713	998.0034	-0.02265	1077.058	1066.771
YHR085W	1248.291	1424.919	1302.259	1420.154	1455.309	1296.426	0.069576	1175.994	1152.815
YJR035W	294.7993	270.9423	348.7841	344.7409	300.7408	476.4977	0.294951	389	540.8487
YER180C-/	81.76076	107.7836	109.6743	103.87	118.6831	122.3043	0.2048	155.1503	169.4542
YLR157W-I	5.7578	3.955362	3.952227	0.895431	0	3.913739	-1.50667	0	0
YMR323W	27.63744	24.72101	21.73725	17.90862	14.97943	17.61182	-0.55311	4.49711	12.29202
YCL032W	674.8141	796.0165	627.4161	665.3053	591.1112	557.7078	-0.20991	957.8844	819.175
YMR022W	1103.194	948.298	1001.89	1049.445	989.7943	964.7366	-0.02353	1363.749	1163.351
YNL305C	1378.417	1417.997	1222.226	1125.557	1199.506	1004.852	-0.27122	1496.413	1547.916
YKL055C	128.9747	201.7234	175.8741	153.1187	147.4897	133.0671	-0.22415	101.185	119.4081
YER049W	3965.972	4111.599	4301.011	4950.838	5165.597	5701.339	0.353701	3890	3905.349
YPL163C	3740.267	3074.305	3386.071	3586.201	3543.21	3585.963	0.071022	2687.023	2727.949
YBR294W	161.2184	137.4488	116.5907	98.49741	170.535	136.9809	-0.03248	93.31503	106.2381
YBR182C	152.0059	206.6677	187.7308	170.1319	200.4938	189.8163	0.036596	207.9913	237.0603
YML059C	1408.358	1365.589	1649.067	1413.886	1583.21	2145.707	0.217525	1043.33	1166.863
YPL283C	1.15156	0	0.988057	0.895431	1.152263	0	-0.06335	0	4.390005
Q0275	0	0	0	0	0	0	#DIV/0!	6.745665	5.268006
YDR489W	365.0445	383.6701	343.8438	359.0678	323.786	276.897	-0.18698	429.474	428.4645
YLR017W	2529.977	2560.108	2581.792	2705.097	2712.428	2450.001	0.03633	2407.078	2139.689
YDR323C	228.0089	194.8016	230.2172	181.7725	170.535	182.9673	-0.28686	276.5723	273.0583
YKL105C	834.881	600.2261	764.756	681.423	737.4486	945.1679	0.10384	860.0723	1188.813
YGL014W	1267.867	1571.267	1489.99	1595.658	1457.613	1451.019	0.057224	1106.289	1312.612
YIL044C	1093.982	1128.267	1140.218	1088.844	1040.494	1026.378	-0.09155	1288.422	1294.174
YIR020W-/	4.60624	14.83261	11.85668	10.74517	4.609054	2.935304	-0.77494	22.48555	10.53601
YBR281C	679.4204	712.954	775.6246	830.96	712.0988	895.2678	0.169527	829.7168	913.9991
YMR112C	457.1693	390.592	360.6407	397.5714	352.5926	353.2149	-0.13117	571.133	498.7046
YOR232W	4215.861	3506.428	3966.06	4004.368	4098.601	3827.637	0.029596	5466.237	5006.362
YNR016C	15250.11	14611.11	15666.63	16324.6	16367.9	18959.13	0.182065	12815.64	13327.18
YKL118W	6.90936	5.933043	6.916398	4.477155	4.609054	4.892174	-0.4993	0	2.634003
YJR144W	2274.331	2172.482	2158.904	2134.708	2019.918	1977.417	-0.10735	1902.277	1770.928
YHR096C	84.06388	133.4935	97.81763	107.4517	108.3128	87.08069	-0.05849	159.6474	136.9682
YDL180W	454.8662	477.6099	449.5659	395.7805	375.6379	480.4114	-0.14276	278.8208	345.0544
YMR093W	1888.558	2123.04	1941.532	2138.289	2007.243	2204.413	0.093096	1716.772	1673.47
YJR107W	340.8617	333.2392	361.6288	348.3227	341.07	333.6462	-0.01779	293.4364	208.0863
YDR412W	163.5215	171.0694	155.1249	198.7857	212.0165	173.1829	0.253987	122.5462	121.1641
YOR214C	21.87964	14.83261	10.86863	22.38578	9.218108	15.65496	-0.0098	2.248555	1.756002
YMR190C	519.3535	432.1233	568.1327	504.1277	510.4527	591.953	0.08025	608.2341	718.2049

YOR069W	771.5452	685.2664	763.7679	644.7103	680.9877	793.5106	-0.06741	808.3555	877.1231
YGL215W	1569.576	2041.955	1587.807	1598.344	1673.087	1568.431	-0.10336	1201.853	1544.404
YKL136W	14.97028	8.899564	12.84474	17.90862	4.609054	3.913739	-0.4741	1.124277	6.146008
YKL167C	937.3698	876.1126	863.5617	719.9265	704.033	704.473	-0.33085	751.0174	740.1549
YJL115W	1122.771	1319.113	1240.011	1328.82	1484.115	1205.432	0.126161	1111.91	967.5572
YDR528W	857.9122	633.8467	854.6692	825.5874	849.2182	963.7582	0.169287	584.6243	662.8908
YNL018C	150.8544	204.69	150.1846	87.75224	99.09466	135.024	-0.65188	170.8902	233.5483
YOR368W	472.1396	419.2683	415.9719	402.944	307.6543	346.3659	-0.30675	240.5954	269.5463
YOR273C	1539.636	2131.94	1619.425	1691.469	1522.14	1160.424	-0.27458	1021.968	1060.625
YPR200C	34.5468	50.43086	31.61782	30.44465	56.46091	43.05113	0.156519	26.98266	18.43802
YNL304W	268.3135	346.0942	328.0349	361.7541	306.5021	346.3659	0.106466	353.0231	446.0245
YCL001W	1432.541	1480.294	1317.08	1484.625	1472.593	1259.245	-0.0046	1175.994	1145.791
YNL202W	43.75928	46.4755	38.53422	55.51672	53.00412	65.55513	0.434932	21.36127	37.75405
YHR214W	5.7578	0	1.976114	7.163448	3.45679	9.784347	1.399623	0	0.878001
YMR269W	1131.983	1007.628	991.021	1154.211	1164.938	1126.178	0.138187	965.7544	806.005
YJL051W	872.8824	626.9248	813.1708	842.6006	757.0371	952.9954	0.142235	1102.916	1082.575
YPL152W	353.5289	326.3173	364.593	287.4334	336.4609	270.048	-0.22447	372.1358	367.0044
YPR119W	856.7606	783.1616	977.1882	958.1112	1035.885	1280.771	0.323418	1258.066	1367.926
YLR063W	658.6923	758.4406	714.3651	765.5935	858.4363	733.826	0.145608	552.0202	548.7507
YPR202W	0	0	0	0	0	0	#DIV/0!	0	0
YPR105C	1163.076	1074.87	1166.895	1036.014	1099.259	1365.895	0.040249	1226.587	1482.066
YPR185W	469.8365	398.5027	557.264	478.1602	478.1893	579.2334	0.107214	499.1792	640.9408
YCR085W	18.42496	27.68753	5.928341	11.6406	20.74074	19.56869	-0.00252	0	5.268006
YOR194C	2060.141	1965.815	2160.88	2119.485	2033.745	2040.036	0.001499	2456.546	2697.219
YDL231C	2108.506	2314.875	2316.993	2115.904	2359.836	2629.054	0.075964	1939.379	1947.406
YDL232W	773.8483	814.8045	749.9351	773.6524	863.0453	742.6319	0.024918	702.6734	558.4087
YOR202W	1644.428	1541.602	1481.097	1512.383	1540.576	1301.318	-0.1001	1907.899	1799.024
YIL037C	99.03415	102.8394	86.949	100.2883	78.35392	39.13739	-0.40731	62.95954	57.94807
YBR282W	1097.437	1041.249	1073.03	930.3528	946.0083	797.4243	-0.26446	916.2861	898.1951
YNL176C	1099.74	1058.059	1067.101	1154.211	1085.432	1294.469	0.132093	1175.994	1359.146
YER071C	418.0163	346.0942	298.3932	313.4009	358.3539	335.6031	-0.07689	550.896	439.0005
YLR185W	16652.71	11936.29	13403.98	14896.39	15070.45	14109.03	0.069841	14902.3	12515.03
YKR100C	1160.772	1189.575	1191.597	1230.322	1155.72	1214.237	0.023568	983.7428	1077.307
YLR285C-/	40.3046	25.70985	43.4745	35.81724	40.32922	17.61182	-0.22377	16.86416	16.68202
YJR112W-/	839.4872	667.4673	653.1056	676.9459	737.4486	668.2709	-0.05264	646.4596	497.8266
YHR188C	1551.151	1744.315	1617.449	1694.156	1826.338	1667.253	0.078529	1945	2088.765
YCR090C	865.9731	732.7308	778.5888	801.4108	906.8313	749.481	0.048003	628.4711	693.6208
YIL063C	1690.49	1516.881	1622.389	1625.207	1434.568	1414.817	-0.1102	1837.069	1850.826
YDR357C	259.101	221.5003	237.1336	261.4659	276.5432	240.6949	0.117624	216.9856	194.9162
YHL002W	1043.313	891.9341	888.2631	813.0514	829.6297	797.4243	-0.21055	957.8844	1028.139
YIL109C	3082.726	3446.109	3598.503	3619.332	3758.683	4658.328	0.249142	3995.682	4711.354
YLR401C	1457.875	1784.857	1427.742	1841.006	1760.659	1690.735	0.180353	2001.214	2115.105
YBL041W	4311.44	4076	4155.767	4227.33	4047.902	3764.038	-0.05916	5101.971	4611.262
YKL031W	28.789	23.73217	11.85668	23.28121	9.218108	18.59026	-0.33354	33.72832	29.85204
YMR149W	4364.412	4083.911	4406.733	4585.502	4907.49	4422.525	0.114359	3841.656	3792.965
YDR322C-/	2092.384	1650.375	1857.547	1712.96	1737.613	1649.641	-0.13495	1769.613	1590.938
YKR106W	2.30312	5.933043	3.952227	3.581724	0	0.978435	-1.41835	0	0
YMR124W	837.1841	705.0432	853.6811	820.2148	775.4733	972.5641	0.100214	966.8786	1126.475
YGL139W	1150.408	1184.631	1221.238	1202.564	1334.321	1559.625	0.204027	1351.382	1590.06
YNL322C	4867.644	4963.979	4959.057	5497.051	5158.684	4556.57	0.04055	4379.061	4123.093
YPR121W	226.8573	207.6565	217.3725	180.8771	210.8642	246.5655	-0.03037	102.3093	169.4542
YGL256W	3029.754	3117.814	2988.872	2645.103	2616.79	2469.569	-0.24089	2911.879	2813.993
YGL243W	446.8053	489.476	490.0762	480.8465	518.5186	444.2094	0.01731	394.6214	375.7845
YLR070C	133.581	227.4333	195.6353	168.341	147.4897	178.0751	-0.17253	152.9017	151.0162

YLL009C	1257.503	1190.564	1079.946	1070.936	981.7285	962.7798	-0.22649	1216.468	895.5611
YLR064W	1386.478	1478.316	1365.495	1545.514	1591.276	1250.44	0.052554	1047.827	1174.765
YNL325C	204.9777	267.9758	259.8589	237.2892	265.0206	186.881	-0.08854	128.1676	161.5522
YCL034W	2222.511	2264.445	2144.083	2106.949	1903.539	1914.797	-0.16235	2436.309	2347.775
YNL224C	541.2332	574.5163	547.3835	543.5266	561.1523	688.818	0.108872	670.0694	830.589
YIL150C	97.88259	133.4935	85.96094	83.27509	85.2675	93.92973	-0.27385	102.3093	143.9922
YKL078W	846.3966	1059.048	949.5226	1103.171	1238.683	1330.671	0.363298	993.8613	918.3891
YJL103C	200.3714	135.4711	201.5636	151.3278	141.7284	136.9809	-0.32155	286.6908	227.4023
YNL128W	39.15304	65.26347	52.36701	74.32078	73.74486	62.61982	0.426317	23.60983	24.58403
YML068W	232.6151	279.8418	312.226	230.1258	263.8683	274.9402	-0.10098	475.5694	377.5405
YNL284C	1414.116	1547.535	1489.99	1228.531	1206.42	1142.812	-0.31528	1396.353	1174.765
YDR427W	2030.2	1933.183	2087.764	1956.517	2080.988	1798.363	-0.05226	2584.714	2242.415
YML010W	1073.254	1168.809	1343.757	1356.578	1477.202	2214.198	0.493402	1337.89	2095.789
YDR245W	3901.485	3651.788	3823.78	4182.558	4150.453	4241.514	0.144377	3550.468	3468.982
YHR088W	2092.384	2162.594	2100.609	2615.554	2654.815	2662.321	0.319785	2179.974	2173.931
YNL122C	734.6952	688.2329	682.7473	762.0118	667.1605	675.12	-0.00095	628.4711	661.1348
YGR135W	4268.833	3716.062	4034.236	3927.36	4089.383	3988.1	-0.00172	5353.809	4496.244
YDR334W	1037.556	1003.673	1156.026	949.1569	1079.671	1456.889	0.124622	1396.353	1501.382
YCR010C	39.15304	33.62057	28.65365	27.75836	23.04527	38.15895	-0.18917	12.36705	22.82803
YBR080C	1484.361	1581.156	1807.156	1592.972	1595.885	1905.991	0.064326	2013.581	1938.626
YGL145W	686.3297	630.8802	694.6039	676.9459	627.9836	833.6264	0.08814	780.2486	912.2431
YJL097W	1515.453	1595.988	1659.935	1650.279	1803.292	1487.221	0.050337	1262.564	1269.59
YPR022C	680.5719	603.1927	762.7799	813.0514	834.2388	1044.968	0.395627	473.3208	593.5287
YGR241C	1551.151	1344.823	1463.312	1246.44	1141.893	1178.035	-0.28964	1509.905	1597.084
YNL084C	1912.741	1620.709	1530.5	1549.096	1648.889	1703.455	-0.04706	2215.951	1964.966
YGR162W	5958.171	5698.687	7293.835	7124.945	7695.968	10605.25	0.424063	8119.532	10262.95
YML096W	666.7532	717.8982	773.6485	763.8027	776.6256	768.0713	0.09706	950.0145	918.3891
YMR139W	1021.434	882.0457	1016.71	760.2209	846.9136	832.6479	-0.25931	1118.656	1161.595
YFR040W	591.9018	609.1257	679.7831	680.5276	613.0042	740.6751	0.113112	726.2833	938.5831
YER149C	330.4977	304.5629	394.2347	398.4668	296.1317	362.9993	0.039134	421.6041	419.6845
YBR038W	955.7947	1282.526	1227.167	1263.453	1269.794	1445.148	0.199128	1354.754	1610.254
YLR099C	1720.431	2404.86	1623.377	1867.869	1455.309	1159.445	-0.35888	1038.832	1019.359
YOR182C	6058.357	4734.568	4935.344	5616.143	5671.441	5996.826	0.136112	6025.003	5162.646
YMR069W	100.1857	142.393	85.96094	88.64767	107.1605	79.25321	-0.25631	28.10694	69.36208
YOL013C	525.1113	513.2082	532.5626	472.7876	525.4321	455.9506	-0.11138	576.7543	578.6027
YHR032W	2975.631	3766.493	3701.261	4241.657	4292.181	4564.398	0.326783	2895.015	3051.932
YGR066C	184.2496	250.1766	226.265	102.9746	97.94239	111.5416	-1.08031	139.4104	118.5301
YDL101C	742.7562	640.7686	771.6724	652.7692	694.8149	891.354	0.054995	902.7948	820.931
YBL079W	1500.483	1604.888	1581.879	1759.522	1745.679	2094.829	0.256693	1258.066	1416.216
YGR278W	579.2346	443.9894	645.2011	630.3834	573.8272	629.1335	0.135991	381.1301	495.1926
YDL106C	508.9895	422.2349	521.694	440.5521	506.9959	620.3276	0.109857	554.2688	622.5028
YKR020W	587.2956	526.0631	529.5985	498.7551	532.3457	482.3683	-0.11844	665.5723	696.2549
YHR170W	3583.655	3687.386	3631.109	4158.382	4171.194	4212.161	0.202125	3846.153	3649.85
YPL180W	747.3624	707.0209	810.2066	708.2859	640.6585	861.0225	-0.03523	901.6705	946.4852
YDL161W	1347.325	1379.432	1339.805	1574.168	1516.379	1489.178	0.171451	1593.101	1660.3
YCR067C	1099.74	1280.548	1298.307	1296.584	1319.342	1543.97	0.177393	1409.844	1713.858
YDR510W	3372.919	2814.24	3142.021	2880.602	2957.86	2804.194	-0.11028	4012.546	3755.211
YMR200W	2077.414	2292.132	2241.901	2368.415	2246.914	2139.837	0.031025	1882.04	1894.726
YEL015W	1820.616	1934.172	1856.559	1862.497	1978.436	2352.157	0.142312	2304.769	2275.779
YDR027C	808.3951	652.6347	725.2337	724.4037	697.1194	836.5617	0.046632	875.8122	921.9011
YMR144W	711.664	665.4896	762.7799	699.3316	618.7655	625.2198	-0.13904	403.6156	514.5086
YDR354W	1098.588	1002.684	973.236	976.0198	816.9548	822.8636	-0.23308	1133.272	1108.037
YPL074W	462.9271	526.0631	530.5865	483.5328	502.3869	667.2925	0.121602	596.9913	673.4268
YER022W	677.1172	652.6347	809.2185	728.8809	739.7531	969.6288	0.188937	971.3757	1014.091

YDR308C	861.3668	706.0321	667.9264	710.9722	743.2099	665.3356	-0.07675	709.4191	581.2367
YOL019W	1566.122	1689.928	1739.968	1824.888	1895.473	1800.32	0.144068	1597.598	2028.182
YDR075W	710.5125	848.4251	862.5736	739.626	800.8231	832.6479	-0.02914	676.815	640.0628
YLR343W	104.792	114.7055	85.96094	82.37965	72.5926	70.4473	-0.43836	79.8237	58.82607
YNL169C	1680.126	2030.089	1954.376	1849.96	1789.465	1800.32	-0.05843	1515.526	1374.072
YGR044C	510.1411	527.052	524.6582	481.7419	480.4939	505.8507	-0.08932	632.9682	571.5787
YOR292C	229.1604	272.92	231.2053	242.6618	235.0617	203.5144	-0.10622	341.7804	258.1323
YIL154C	1410.661	1387.343	1050.304	1341.356	1158.025	1088.019	-0.10129	1629.078	1510.162
YBL004W	2308.878	2154.683	2362.444	2843.889	2914.074	3490.077	0.438106	2120.387	2549.715
YLR464W	1.15156	1.977681	1.976114	1.790862	0	7.827478	0.913777	0	0
YNL075W	1798.737	2031.078	1905.962	2430.2	2322.963	2305.192	0.299343	1967.486	1783.22
YDR307W	534.3238	654.6124	550.3477	646.5012	589.9589	653.5944	0.119934	441.841	466.2186
YPL047W	544.6879	623.9583	584.9296	576.6576	584.1976	457.9074	-0.11541	465.4509	412.6605
YJR068W	1540.787	1238.028	1396.124	1363.741	1438.025	1388.399	0.005252	1723.517	1770.928
YNR022C	1354.234	1253.85	1275.581	1313.597	1250.206	1105.631	-0.08186	1224.338	1120.329
YKR092C	6764.263	7704.056	7413.39	8383.025	8007.079	7406.751	0.121045	5856.361	6592.032
YML101C	834.881	758.4406	785.5052	791.561	818.1071	606.6295	-0.1021	672.3179	677.8168
YDR144C	1859.769	2119.085	2073.931	2240.368	2320.659	2443.151	0.210617	2474.535	2711.267
YAL059W	652.9345	626.9248	518.7298	721.7174	680.9877	605.6511	0.159149	654.3295	519.7766
YGL236C	617.2361	779.2063	479.2076	600.8342	520.8231	499.0017	-0.21081	413.7341	450.4146
YML047W-/	9.21248	4.944202	3.952227	11.6406	13.82716	5.870608	0.791231	5.621387	1.756002
YBL044W	9.21248	13.84377	7.904455	10.74517	5.761317	8.805912	-0.29059	4.49711	7.024009
YFL020C	21.87964	19.77681	14.82085	10.74517	10.37037	17.61182	-0.54432	56.21387	18.43802
YAR002W	2140.75	2114.141	2361.456	2274.395	2416.296	2722.005	0.163964	2661.165	2639.271
YOR275C	727.7859	611.1034	763.7679	644.7103	719.0124	801.338	0.042194	888.1792	891.1711
YNL301C	5699.07	5811.415	5637.852	6410.391	5979.095	5241.475	0.040042	7669.821	7061.763
YCR072C	1358.841	1739.37	1576.939	1786.385	1885.103	1763.139	0.217169	1414.341	1338.952
YCR033W	1225.26	1222.207	1460.348	1233.904	1275.556	1838.479	0.15397	1577.361	1928.09
YJR050W	609.1752	502.3309	465.3748	494.2779	489.712	487.2605	-0.10003	601.4885	611.9667
YIR012W	3227.823	3851.533	3488.829	4154.8	3684.939	3746.427	0.132676	3080.52	3205.582
YPL006W	1125.074	1157.932	1179.74	1025.269	1062.387	1229.892	-0.0618	957.8844	877.1231
YGL228W	3056.24	3089.138	3512.542	3172.512	3302.387	3722.944	0.07848	3656.15	3824.573
YML023C	758.878	744.5968	756.8515	735.1489	830.782	673.1631	-0.01362	614.9798	562.7987
YOR171C	895.9136	924.5658	1039.436	1088.844	962.14	1268.051	0.214792	1108.538	1258.176
YGR159C	11994.65	12079.67	11109.71	14020.66	13206.09	11992.67	0.156647	12343.44	10516.7
YMR231W	495.1708	483.543	643.225	509.5003	601.4815	702.5161	0.161056	725.159	721.7169
YLR133W	637.9642	717.8982	866.5258	628.5926	701.7285	738.7182	-0.10315	844.3324	803.371
YDR294C	2526.523	2625.371	2529.425	2684.502	2649.054	2289.537	-0.01098	1948.373	2043.987
YPL184C	1627.154	1618.732	1771.586	1781.012	1849.383	1741.614	0.098501	1617.835	1449.58
YDL013W	594.2049	695.1548	653.1056	619.6383	599.177	632.0688	-0.06967	670.0694	687.4748
YHR209W	345.468	281.8195	375.4616	269.5247	362.963	317.9913	-0.07723	222.6069	234.4263
YJL133W	1194.168	1276.593	1289.414	1224.95	1277.86	1155.531	-0.03961	1054.572	963.1672
YBR053C	1323.142	1252.861	1304.235	1341.356	1276.708	1212.281	-0.01867	1172.621	1073.795
YMR259C	1136.59	1171.776	1257.796	1245.545	1395.391	1621.266	0.257227	968.0029	1146.669
YPR040W	886.7012	791.0723	725.2337	805.8879	831.9342	724.0417	-0.02492	976.9971	973.7032
YIL067C	391.5304	346.0942	371.5094	393.0942	377.9424	335.6031	-0.00325	455.3324	488.1686
YBL059W	318.9821	294.6744	264.7992	258.7796	314.5679	225.04	-0.13788	216.9856	200.1842
YNL115C	1011.07	1160.899	1054.257	950.9477	982.8807	915.8149	-0.17907	722.9104	825.321
YNL133C	513.5957	525.0743	576.0371	459.3561	538.107	358.1071	-0.25237	353.0231	332.7624
YML038C	1186.107	1334.935	1147.134	1245.545	1194.897	1134.984	-0.03695	978.1214	971.9472
YDL201W	2029.049	1942.083	1897.069	2397.964	2122.469	2065.476	0.166465	1914.645	1712.98
YNL181W	1021.434	1004.662	931.7376	1053.027	1000.165	1003.874	0.047607	1250.197	1188.813
YPL233W	358.1351	415.313	440.6733	376.9765	339.9177	383.5464	-0.14183	198.9971	278.3263
YDR106W	35.69836	51.4197	37.54616	45.66698	53.00412	44.02956	0.194945	23.60983	26.34003

YOR134W	32.24368	93.93984	64.22369	30.44465	23.04527	31.30991	-1.16695	73.07804	88.67811
YMR319C	1927.711	1940.105	1962.281	1770.267	1867.819	1760.204	-0.11102	1496.413	1343.342
YNL277W-/	6.90936	16.81029	11.85668	5.372586	8.065844	1.956869	-1.20843	15.73988	24.58403
YPL046C	468.6849	433.1121	407.0794	433.3886	388.3128	318.9697	-0.19845	362.0173	320.4704
YOL117W	193.4621	146.3484	174.8861	133.4192	135.9671	213.2988	-0.09264	188.8786	261.6443
YPR116W	84.06388	85.04028	91.88928	65.36646	80.65844	80.23165	-0.20605	40.47399	48.29006
YLR099W-/	656.3892	586.3824	573.073	573.0759	493.1688	449.1015	-0.26099	543.026	480.2666
YJL118W	285.5869	296.6521	269.7395	281.1653	218.9301	212.3203	-0.2581	205.7428	169.4542
YJR100C	692.0875	792.0612	715.3531	652.7692	609.5474	625.2198	-0.22067	620.6012	541.7267
YJL025W	777.303	960.1641	916.9167	923.1894	956.3787	901.1384	0.067074	730.7804	712.0589
YKR058W	414.5616	279.8418	310.2498	220.276	223.5391	260.2636	-0.51289	408.1127	360.8584
YDL164C	738.1499	741.6303	741.0426	878.4178	739.7531	863.9579	0.160484	829.7168	921.0231
YER189W	0	0	0.988057	0	0	0	#NUM!	1.124277	4.390005
YDR275W	466.3818	560.6725	556.276	514.8728	525.4321	429.5328	-0.10731	447.4624	446.0245
YDR471W	19254.08	15585.11	17387.82	19472.04	18951.28	17460.17	0.097626	18374.07	15655.64
YIL043C	4987.406	4793.898	5057.863	5366.318	5523.951	5172.984	0.114354	4907.471	3978.223
YIL034C	2744.167	2594.717	2561.043	2697.038	2457.778	2481.31	-0.049	3321.116	2893.014
YDR448W	498.6255	648.6793	613.5833	529.1997	540.4116	525.4194	-0.14272	462.078	354.7124
YJR032W	731.2406	821.7264	719.3054	822.9011	1003.621	768.0713	0.191373	965.7544	904.3411
YBR168W	313.2243	317.4178	351.7482	323.2506	364.1153	233.8459	-0.09276	229.3526	216.8663
YKL051W	1195.319	1349.767	1146.146	978.7061	1232.922	973.5425	-0.21273	987.1156	859.5631
YOL057W	1840.193	1780.902	1727.123	1637.743	1845.926	1808.147	-0.0153	1933.757	2049.255
YLR190W	1582.243	1528.747	1913.866	1796.235	1879.342	2266.055	0.241777	2085.535	2454.013
YCR071C	1043.313	901.8225	884.3109	867.6727	828.4774	707.4083	-0.23535	883.6821	766.4949
YOR314W	13.81872	29.66521	34.58199	26.86293	19.58848	25.4393	-0.11889	14.61561	8.780011
YDR425W	135.8841	137.4488	206.5039	120.8832	164.7737	172.2045	-0.06763	203.4942	176.4782
YAL031C	351.2258	355.9826	380.4019	264.1522	298.4362	359.0855	-0.23883	360.8931	515.3866
YDR100W	674.8141	464.755	494.0284	395.7805	422.8807	396.2661	-0.42718	431.7226	362.6144
YIL148W	29166.71	22823.43	24671.78	26300.6	28165.93	25465.72	0.060268	27495.33	22830.66
YGL165C	16.12184	16.81029	18.77308	8.058879	11.52263	7.827478	-0.91566	7.869942	6.146008
YOR307C	1646.731	1757.169	1644.127	1800.712	1696.132	1736.722	0.052075	1394.104	1466.262
YKL063C	2044.019	1794.745	1986.982	2171.42	2125.926	1842.393	0.075734	1653.812	1626.936
YGR139W	1.15156	1.977681	7.904455	9.849741	9.218108	6.849043	1.231977	7.869942	9.658012
YNL049C	864.8215	850.4028	1052.281	960.7975	1061.235	1320.887	0.272523	1179.367	1192.325
YDL008W	779.6061	626.9248	579.0013	673.3641	599.177	632.0688	-0.06003	510.422	486.4126
YGR216C	596.508	737.675	606.6669	604.4159	576.1317	629.1335	-0.10095	468.8237	463.5846
YOR293C-/	52.97176	35.59826	36.5581	51.03957	39.17696	27.39617	-0.08936	75.32659	81.6541
YHR101C	495.1708	410.3688	450.5539	478.1602	433.2511	483.3467	0.040558	336.159	324.8604
YGR133W	555.0519	553.7506	562.2043	504.1277	462.0577	435.4034	-0.25365	435.0954	419.6845
YBR081C	1045.616	1048.171	1209.382	1001.987	1058.93	1320.887	0.033942	1238.954	1415.338
YMR194C-I	304.0118	318.4066	284.5604	239.0801	206.2552	184.9242	-0.52512	216.9856	193.1602
YFR025C	1001.857	1036.305	1073.03	961.6929	1013.992	915.8149	-0.10565	769.0058	767.3729
YDL054C	419.1678	478.5988	412.0197	486.219	480.4939	350.2796	0.007916	493.5578	536.4587
YDR321W	2504.643	2516.599	2328.85	2546.606	2532.675	2326.718	0.010932	3164.841	2755.167
YNL281W	3529.531	3072.327	3219.089	3408.01	3295.474	2925.52	-0.02848	4520.72	3776.283
YHR150W	323.5883	435.0898	419.9242	316.9826	329.5474	391.3739	-0.1834	456.4567	504.8506
YJL184W	1433.692	1074.87	1287.438	1322.552	1222.552	1174.122	-0.02948	996.1098	1046.577
YOR381W	240.676	335.2169	274.6798	212.2172	191.2757	214.2772	-0.46136	424.9769	374.9065
YMR291W	160.0668	170.0806	206.5039	163.8639	162.4691	126.2181	-0.2459	261.9567	194.0382
YOR164C	1255.2	1289.448	1388.22	1296.584	1413.827	1433.407	0.075379	1198.48	1013.213
YGR282C	17099.51	18034.47	18283	18580.19	18985.85	17921.99	0.054878	17096.89	16066.54
YMR113W	687.4813	789.0947	790.4455	786.1884	788.1482	729.9123	0.023499	683.5607	537.3367
YML069W	2349.182	1926.261	2177.677	2227.832	2280.329	2651.558	0.149906	2559.98	2599.761
YJL005W	2314.635	1939.116	2380.229	2022.779	2210.041	3135.883	0.151536	2855.665	3209.972

YOL015W	118.6107	108.7724	53.35507	57.30759	77.20165	86.10225	-0.34772	150.6532	126.4322
YOR101W	2082.02	2084.476	2158.904	2185.747	2162.799	2172.125	0.043864	1734.76	1698.932
YDR020C	582.6893	724.82	610.6191	636.6515	631.4404	710.3436	0.044661	474.4451	557.5307
YCR092C	444.5021	564.6279	521.694	500.5459	492.0165	710.3436	0.15369	662.1994	665.5248
YMR300C	2904.234	3860.433	2899.947	3424.128	3109.959	2973.463	-0.02364	2467.789	2305.631
YHR016C	1174.591	1367.566	1232.107	967.0655	1025.514	953.9738	-0.35717	1036.584	966.6792
YKR077W	290.1931	350.0495	363.6049	318.7734	371.0288	322.8835	0.012646	376.633	439.0005
YDR442W	40.3046	43.50898	39.52227	34.02638	42.63375	44.02956	-0.03129	21.36127	28.97404
YIL020C	745.0593	754.4853	724.2457	821.1103	619.9177	664.3572	-0.07894	927.5289	863.0751
YOR266W	202.6745	233.3663	191.683	243.5572	278.8478	253.4146	0.305591	326.0405	287.9844
YJL215C	21.87964	31.64289	31.61782	42.08526	28.80659	18.59026	0.071756	13.49133	25.46203
YMR230W	11212.74	9761.833	10463.52	11522.41	11246.09	10609.17	0.086369	11541.83	9970.58
YCR063W	148.5512	205.6788	157.101	136.1055	197.0371	155.5711	-0.06527	110.1792	116.7741
YAL035W	5211.96	5099.45	5902.651	6207.128	6186.503	8002.618	0.331058	6894.069	7853.72
YKL176C	1024.888	1112.445	1048.328	1123.766	1100.412	1185.863	0.098196	820.7226	983.3612
YBR137W	1004.16	925.5546	992.0091	923.1894	972.5104	856.1304	-0.08643	1485.171	1083.453
YDR403W	25.33432	60.31927	59.28341	53.72586	42.63375	33.26678	-0.16107	31.47977	8.780011
YFR030W	792.2732	1136.178	1019.675	857.8229	967.9013	840.4754	-0.14501	830.8411	943.8512
YIL164C	56.42644	88.99564	80.0326	89.5431	82.96297	76.31791	0.142288	37.10116	48.29006
YER027C	1330.052	1381.41	1531.488	1415.676	1473.745	1585.064	0.076654	1759.494	1673.47
YDR151C	761.1811	765.3625	748.9471	880.2087	760.4939	708.3867	0.045924	934.2746	1015.847
YLL019C	709.3609	766.3513	827.9916	693.0636	823.8684	865.9147	0.048731	546.3989	732.2529
YNL196C	48.36552	51.4197	44.46256	87.75224	64.52675	33.26678	0.363226	92.19075	101.8481
YPL245W	1570.728	1672.129	1513.703	1826.679	1736.461	1745.528	0.158431	1490.792	1363.536
YDL220C	133.581	131.5158	135.3638	178.1908	130.2058	191.7732	0.320758	170.8902	211.5983
YHL020C	3087.332	3597.401	3054.084	3673.058	3167.572	2847.245	-0.00757	2357.61	2468.939
YBR228W	447.9568	450.9112	345.8199	393.9897	407.9013	366.913	-0.09075	337.2832	313.4464
YDR129C	4458.84	4443.849	4708.091	4087.643	4269.136	4644.63	-0.06608	5080.61	5192.498
YOR179C	583.8409	598.2485	552.3238	573.9713	515.0618	485.3036	-0.1397	511.5463	454.8046
YLL058W	800.3342	1013.561	777.6007	886.4767	761.6462	633.0473	-0.18401	532.9075	554.8967
YGL247W	298.254	294.6744	291.4768	261.4659	256.9548	231.889	-0.23722	218.1098	251.1083
YPL144W	476.7458	405.4246	374.4735	453.9835	410.2058	350.2796	-0.04925	337.2832	273.0583
YFL048C	1822.919	1742.337	1827.905	1821.307	1870.124	1865.875	0.043254	1634.699	1531.234
YLR241W	990.3415	1036.305	935.6898	1027.955	939.0947	1310.124	0.145717	1231.084	1181.789
YNL268W	2776.411	3438.198	2895.006	3264.742	2924.445	2635.903	-0.04578	3316.619	3413.668
YKL091C	416.8647	354.0049	385.3422	368.9176	313.4157	303.3148	-0.23026	577.8786	503.0946
YNL164C	608.0236	621.9806	551.3357	677.8413	583.0453	579.2334	0.046837	463.2023	478.5106
YMR130W	938.5214	843.4809	869.49	889.163	888.3951	895.2678	0.011561	718.4133	788.445
YKL183C-/	43.75928	42.52014	27.66559	33.13095	34.5679	36.20208	-0.13313	20.23699	27.21803
YBR063C	279.8291	246.2213	279.6201	334.8912	329.5474	318.9697	0.287601	451.9595	408.2705
YML011C	371.9539	452.8889	392.2586	325.9369	360.6585	315.056	-0.28107	326.0405	254.6203
YGR104C	619.5392	582.427	634.3325	620.5337	624.5268	647.7238	0.043709	494.6821	445.1465
YGL257C	798.031	909.7332	917.9048	848.8686	920.6585	910.9227	0.02979	1189.486	974.5812
YOL134C	1.15156	3.955362	0	1.790862	5.761317	0.978435	0.740196	0	0
YNL211C	175.0371	160.1922	136.3518	119.0923	108.3128	123.2828	-0.42732	110.1792	90.43411
YBR171W	1275.928	1172.765	1326.96	1364.637	1433.416	1215.216	0.088051	1713.399	1605.864
YNL294C	612.6299	691.1995	582.9535	587.4028	616.461	625.2198	-0.04481	766.7572	762.9829
YOL011W	556.2035	708.9986	598.7624	628.5926	589.9589	599.7805	-0.03576	649.8324	710.3029
YOR097C	550.4457	526.0631	541.4551	470.1013	492.0165	422.6838	-0.2245	430.5983	381.9305
YOL056W	499.777	599.2373	498.9687	441.4475	493.1688	389.417	-0.27131	598.1156	562.7987
YHR067W	354.6805	380.7036	381.3899	345.6364	307.6543	346.3659	-0.15983	278.8208	253.7423
YHR004C	588.4471	584.4047	671.8786	519.35	520.8231	562.6	-0.20284	619.4769	654.9888
YMR012W	4541.752	4054.246	4948.189	4883.681	5128.725	6568.232	0.291826	5836.124	6659.638
YJR007W	5603.491	5794.605	5124.063	5676.137	5544.692	4907.829	-0.03478	5007.532	4607.75

YOR308C	249.8885	259.0762	343.8438	285.6425	254.6502	464.7565	0.236972	409.237	432.8545
YOR250C	601.1143	676.3669	589.8699	540.8403	602.6338	510.7429	-0.17484	418.2312	452.1706
YOR014W	1193.016	1320.102	1536.428	1327.029	1579.753	1914.797	0.251745	1636.948	1981.648
YHL034C	3589.412	3951.406	4469.969	4093.015	3892.346	4351.099	0.038598	4837.766	4744.718
YOR050C	103.6404	97.8952	97.81763	100.2883	101.3992	123.2828	0.118459	70.82948	57.94807
YOL098C	1851.708	1702.783	1852.607	1847.274	2013.004	2482.289	0.230212	2166.483	2361.823
YOL081W	1964.561	1675.096	1808.144	1567.9	1561.317	1881.53	-0.12065	1571.74	1500.504
YJR065C	3901.485	3783.303	4051.033	3819.909	3658.437	3656.411	-0.07585	4403.795	4404.053
YOL020W	1789.524	2652.07	1990.935	2010.243	2147.819	1968.611	-0.07028	1569.491	1561.086
YPR062W	3878.454	3466.875	3600.479	3830.654	3701.07	3188.719	-0.03001	3562.835	2840.333
YPL217C	3362.555	3326.459	3667.667	4113.61	4132.017	5178.855	0.374305	3047.916	3561.172
YGL070C	1013.373	943.3538	973.236	1030.641	952.9219	907.009	-0.01953	960.133	755.9589
YFR003C	1196.471	979.9409	1327.948	1093.321	959.8355	922.6639	-0.23586	1436.827	1271.346
YJL154C	1403.752	1230.117	1330.913	1270.617	1231.77	1425.579	-0.01346	1364.873	1579.524
YHL009C	570.0222	614.0699	565.1685	572.1804	642.963	680.9906	0.116316	453.0838	521.5326
YCR019W	365.0445	391.5808	428.8167	432.4932	391.7696	346.3659	-0.01814	492.4335	487.2906
YPL273W	6898.996	6364.177	6935.171	6877.806	6826.009	6658.248	0.011647	6130.685	5908.069
YHR157W	29.94056	33.62057	51.37895	51.03957	29.95885	58.70608	0.281496	28.10694	17.56002
YLR459W	724.3312	831.6148	698.5562	830.0646	890.6997	795.4674	0.158456	603.737	662.8908
YNR012W	863.67	1103.546	1001.89	1225.845	1093.498	998.0034	0.160001	843.2081	813.029
YGR004W	430.6834	675.378	595.7983	556.9581	634.8972	564.5568	0.045519	419.3555	454.8046
YHR070C-/	199.2199	166.1252	164.0174	194.3085	145.1852	145.7868	-0.12544	104.5578	138.7242
YFL016C	1342.719	1822.433	1458.372	1280.466	1149.959	941.2542	-0.45553	1705.529	1811.316
YML007W	1665.156	1663.23	1803.204	1444.33	1516.379	1746.506	-0.12453	1706.653	1882.434
YGL088W	27.63744	43.50898	13.8328	45.66698	25.3498	28.37461	0.226011	92.19075	78.1421
YDR274C	108.2466	143.3819	119.5549	92.2294	101.3992	48.92174	-0.61385	139.4104	117.6521
YBR040W	13.81872	11.86609	5.928341	8.95431	12.6749	7.827478	-0.10193	0	5.268006
YBR213W	580.3862	788.1058	529.5985	507.7094	536.9548	445.1878	-0.34938	296.8093	327.4944
YHR105W	49.51708	132.5046	104.734	95.81112	64.52675	112.52	-0.07167	112.4277	194.9162
YAL061W	976.5228	862.2689	779.5768	453.9835	428.642	385.5033	-1.04597	865.6937	833.223
YDR512C	470.988	455.8554	414.9839	342.9501	440.1646	301.3579	-0.30721	667.8208	524.1666
YJL153C	224.5542	352.0272	261.8351	341.1592	280	293.5304	0.125615	202.3699	154.5282
YDL242W	10.36404	21.75449	17.78502	12.53603	12.6749	18.59026	-0.18817	21.36127	21.07203
YGR251W	635.6611	624.9472	722.2695	874.8361	784.6914	770.0281	0.293097	566.6358	665.5248
YLR238W	384.621	338.1834	330.999	322.3552	333.0041	299.401	-0.1424	382.2543	411.7825
YBR212W	685.1782	704.0544	738.0784	548.8992	576.1317	569.449	-0.32819	640.8382	833.223
YOL038W	3932.577	3752.649	3957.168	3983.773	3786.338	3422.565	-0.05683	4444.269	4297.815
YNL252C	1973.774	2042.944	1647.091	1768.476	1749.136	1471.566	-0.18297	1669.552	1503.138
YOR059C	753.1202	838.5367	642.2369	713.6585	667.1605	707.4083	-0.09728	834.2139	790.201
YNR056C	154.309	193.8127	140.3041	99.39284	124.4445	133.0671	-0.4526	168.6416	251.9863
YPR017C	553.9003	489.476	551.3357	474.5784	471.2758	462.7996	-0.17898	394.6214	406.5145
YNR040W	745.0593	652.6347	713.377	717.2403	557.6955	591.953	-0.17734	620.6012	545.2387
YDR188W	4647.696	5818.337	4499.611	5376.168	5178.272	4824.662	0.039317	3859.645	4223.185
YPR125W	943.1276	1270.66	1117.492	1218.682	1094.65	1123.243	0.044895	837.5867	903.4631
YHR135C	3640.081	3513.35	3491.793	3215.493	3501.729	3250.36	-0.09489	3830.413	3957.151
YLR042C	1476.3	1432.83	1465.288	1351.205	1538.272	1281.749	-0.06862	1763.991	1596.206
YLR212C	545.8394	619.0141	687.6876	622.3246	667.1605	807.2086	0.178611	754.3902	856.929
YKL028W	1548.848	1704.761	1489.99	1457.762	1503.704	1643.77	-0.04271	1865.176	1839.412
YOL148C	712.8156	764.3737	772.6604	773.6524	668.3128	784.7046	-0.01494	800.4856	995.6532
YDR462W	1507.392	1312.191	1444.539	1304.643	1148.807	1010.723	-0.29974	1146.763	1086.965
YCR098C	62.18424	103.8282	75.09232	69.84362	73.74486	41.09426	-0.38461	112.4277	122.0421
YDL198C	2019.836	2194.237	2050.218	2141.871	2023.375	1937.301	-0.03774	1864.052	1533.868
YOR298C-/	7327.376	7254.133	7056.702	6196.383	5526.256	4646.586	-0.4026	7649.584	7111.809
YKR003W	1191.865	1118.379	1257.796	1103.171	1071.605	1165.316	-0.09524	876.9364	822.687

YDL033C	398.4397	422.2349	434.745	404.7348	352.5926	320.9266	-0.21947	254.0867	280.9603
YMR047C	1969.167	1906.484	2265.614	1995.02	2002.634	2510.663	0.083749	2009.084	2907.94
YBR150C	276.3744	257.0985	294.4409	223.8578	190.1235	267.1127	-0.28163	301.3064	342.4204
YLR296W	19.57652	14.83261	7.904455	2.686293	9.218108	5.870608	-1.25127	1.124277	3.512004
YIL111W	1725.037	1699.817	1627.33	1449.703	1349.301	1084.106	-0.37969	1342.387	1125.597
YOR114W	274.0713	298.6298	231.2053	232.8121	213.1687	262.2205	-0.18287	321.5434	287.1064
YKL047W	464.0787	388.6143	535.5268	427.1206	481.6461	517.592	0.039101	516.0434	577.7247
YEL056W	1195.319	1182.653	1133.301	1130.034	940.247	993.1112	-0.19686	1244.575	1266.078
YJL111W	4516.418	4345.954	4475.897	4447.606	4930.535	4642.673	0.071999	5484.226	5087.138
YOR056C	2852.414	2745.021	2376.277	3069.538	3048.889	2823.763	0.165377	2377.847	2367.091
YMR214W	1816.01	1839.243	1874.344	2193.806	2172.017	1788.579	0.154444	2318.26	2285.437
YDR364C	713.9672	765.3625	760.8038	840.8097	833.0865	925.5992	0.214647	611.6069	705.9129
YDR172W	6247.213	6402.742	7118.949	7191.207	7329.548	9481.032	0.279909	7847.457	8969.659
YNR038W	1195.319	1297.359	1261.749	1434.481	1588.971	1641.813	0.313367	1145.639	1188.813
YNL067W-/	2.30312	4.944202	5.928341	5.372586	2.304527	0.978435	-0.60618	1.124277	0
YOL004W	2207.54	1991.525	2248.817	2000.393	2125.926	2651.558	0.072008	2374.474	2709.511
YFR005C	840.6388	935.4431	869.49	873.9407	813.498	885.4834	-0.04017	701.5491	716.4489
YFR035C	216.4933	155.2479	173.898	191.6222	160.1646	163.3986	-0.08286	167.5173	122.0421
YDR012W	31860.21	27454.17	25735.92	30156.33	29096.96	25003.9	-0.01352	27337.93	23058.06
YDR200C	213.0386	222.4891	314.2021	291.9105	307.6543	331.6894	0.312804	243.9682	406.5145
YDR409W	451.4115	432.1233	433.7569	423.5389	425.1852	488.2389	0.021385	482.315	619.8688
YER135C	63.3358	52.40854	48.41478	46.56241	72.5926	64.57669	0.162506	32.60405	25.46203
YER161C	1035.252	1203.419	1099.707	1167.642	1015.144	1216.194	0.025955	1289.546	1287.15
YPL268W	485.9583	346.0942	449.5659	490.6962	543.8684	462.7996	0.224458	544.1503	658.5008
YDR079C-/	191.1589	156.2368	172.9099	166.5502	192.428	211.3419	0.132412	315.922	270.4243
YOR236W	1206.835	1132.222	1271.629	1328.82	1163.786	1108.567	-0.00381	1686.416	1393.388
YJL137C	462.9271	409.3799	407.0794	315.1917	391.7696	361.0424	-0.26054	276.5723	288.8624
YDR449C	1302.414	1331.968	1313.128	1709.378	1576.296	1426.558	0.255468	1261.439	1216.909
YOR349W	406.5007	515.1859	510.8254	399.3622	511.605	525.4194	0.003897	414.8584	365.2484
YLR104W	816.456	791.0723	903.0839	893.6402	1009.383	801.338	0.107248	726.2833	670.7928
YIR011C	999.554	1092.669	1075.994	967.0655	1085.432	938.3189	-0.08313	1286.173	1180.033
YER170W	298.254	251.1655	295.429	225.6486	261.5638	263.1989	-0.17101	329.4133	350.3224
YER015W	149.7028	185.902	150.1846	151.3278	139.4239	114.4769	-0.2616	185.5058	185.2582
YPL250C	908.5808	1364.6	732.1501	806.7834	517.3663	357.1287	-0.83797	409.237	402.1245
YML064C	692.0875	624.9472	619.5116	540.8403	526.5844	557.7078	-0.25293	453.0838	447.7805
YLR456W	195.7652	166.1252	166.9816	144.1644	138.2716	103.7141	-0.45376	240.5954	193.1602
YDR302W	903.9746	831.6148	737.0904	861.4046	850.3704	685.8827	-0.04445	753.2659	626.0148
YIL163C	5.7578	19.77681	23.71336	6.268017	9.218108	8.805912	-1.01958	32.60405	15.80402
YHR163W	5131.351	4453.737	4720.936	5017.1	4618.272	4595.708	-0.00758	4314.977	3806.135
YLR068W	541.2332	566.6056	649.1533	749.4758	622.2223	643.81	0.198036	618.3526	575.9687
YMR079W	4020.096	4351.887	4049.057	4083.165	4187.325	3851.119	-0.0352	3612.304	3312.698
YKR055W	266.0103	288.7414	286.5365	298.1785	217.7778	286.6814	-0.06785	335.0347	326.6164
YPL249C-/	149.7028	147.3372	109.6743	147.7461	157.8601	106.6494	0.019523	107.9306	69.36208
YNR077C	0	0	0	0	0	0	#DIV/0!	2.248555	1.756002
YBL098W	427.2287	443.0005	447.5897	451.2972	463.2099	507.8076	0.110089	636.3411	635.6728
YAL041W	1364.599	1291.426	1280.522	1358.369	1402.305	1655.512	0.16587	1407.595	1689.274
YDR135C	2498.885	2226.869	2551.163	2678.234	2602.963	3002.816	0.187003	2109.145	2227.489
YPR083W	404.1975	376.7482	397.1988	376.9765	360.6585	395.2876	-0.05647	473.3208	419.6845
YNR075W	81.76076	85.04028	73.11621	84.17052	80.65844	88.05912	0.075962	78.69942	102.7261
YLR361C	495.1708	516.1747	580.9774	501.4414	541.5638	590.9746	0.037257	392.3728	359.9804
YGL144C	336.2555	307.5294	370.5213	321.4597	301.893	338.5384	-0.07655	227.1041	200.1842
YPL077C	163.5215	137.4488	169.9458	170.1319	150.9465	133.0671	-0.05232	164.1445	213.3543
YDR122W	944.2792	890.9452	840.8364	607.1022	675.2264	756.33	-0.39249	784.7457	1040.431
YKR072C	2024.442	1920.328	1876.32	1940.399	1859.753	1860.004	-0.04045	1627.954	1639.228

YGL254W	207.2808	195.7904	210.4561	201.472	253.498	191.7732	0.076065	246.2168	273.0583
YGL207W	2911.144	2605.595	3182.531	2839.412	3199.836	3695.548	0.162256	3395.318	3711.311
YKR068C	1412.964	1277.582	1201.477	1186.446	1321.646	1112.48	-0.1043	1133.272	1090.477
YLR051C	1934.621	1986.58	1923.747	2304.839	2169.712	2201.478	0.1918	2018.078	1680.494
YBR154C	4349.442	4100.721	4046.093	4862.19	4562.963	4315.876	0.136994	4131.72	3827.207
YKL197C	565.4159	654.6124	559.2402	509.5003	527.7367	584.1255	-0.13408	397.9942	433.7325
YER060W	966.1588	1105.524	965.3315	933.0391	1037.037	1015.615	-0.02459	770.1301	843.759
YKL179C	1194.168	1068.937	1292.378	1165.851	1181.07	1668.231	0.175409	1581.858	1459.238
YDR468C	1105.498	1082.78	1124.409	1031.537	995.5556	1022.464	-0.1194	999.4827	830.589
YDR513W	2572.585	2441.447	2235.973	2213.505	2218.107	2038.08	-0.16428	2294.65	1934.236
YPR166C	579.2346	583.4159	549.3596	594.5662	667.1605	535.2038	0.069843	672.3179	461.8286
YMR177W	491.7161	463.7662	482.1717	522.0363	471.2758	561.6215	0.113136	499.1792	561.9207
YJR090C	875.1856	779.2063	842.8125	783.5021	792.7573	967.6719	0.026746	864.5694	1065.893
YDR511W	977.6744	811.838	890.2392	791.561	748.9713	757.3085	-0.22182	829.7168	692.7428
YNL182C	2090.081	2140.84	2458.285	2611.077	2626.008	2522.405	0.214127	2058.552	2098.423
YDR356W	624.1455	396.525	523.6701	640.2332	670.6173	924.6208	0.533588	820.7226	762.9829
YOL131W	4.60624	3.955362	4.940284	3.581724	3.45679	4.892174	-0.17848	12.36705	15.80402
YDR126W	413.41	279.8418	320.1304	347.4272	399.8354	321.905	0.07731	225.9798	222.1343
YPL162C	525.1113	553.7506	531.5746	524.7226	574.9795	423.6622	-0.08019	675.6908	628.6488
YLR250W	1601.82	1631.587	1448.491	1685.201	1653.498	1455.911	0.03432	2142.873	1973.746
YHR098C	2178.751	2015.257	2061.087	2142.766	2076.379	2375.639	0.076294	2410.451	2497.035
YKL138C-/	628.7517	514.197	520.7059	479.0556	618.7655	492.1527	-0.06535	484.5636	433.7325
YNL185C	982.2806	834.5813	793.4096	768.2798	755.8848	683.9259	-0.2414	781.3728	695.3769
YFR008W	337.4071	341.1499	367.5571	371.6039	349.1358	318.9697	-0.00886	299.0578	191.4042
YGR296W	1.15156	0.98884	2.96417	3.581724	0	0.978435	-0.16271	4.49711	10.53601
YNL310C	1138.893	938.4096	1031.531	936.6209	910.2881	852.2166	-0.20388	1229.96	1176.521
YJR131W	762.3327	738.6638	705.4726	774.5478	704.033	739.6966	0.0077	623.974	570.7007
YMR031C	2933.023	2896.314	3259.599	2502.73	2628.313	3281.67	-0.11154	3250.286	3694.629
YNL067W-I	3.45468	4.944202	0	0	0	4.892174	-0.77972	0	0
YCL056C	322.4368	357.9602	301.3573	296.3877	311.1111	213.2988	-0.25834	267.578	233.5483
YPL258C	203.8261	194.8016	215.3964	205.0537	125.5967	200.5791	-0.20896	134.9133	139.6022
YGL203C	1241.382	1244.95	1320.044	1186.446	1376.955	1283.706	0.015356	1079.306	1055.357
YLR225C	815.3044	910.722	962.3673	801.4108	821.5639	823.842	-0.13584	993.8613	878.0011
YOR130C	712.8156	648.6793	561.2163	665.3053	675.2264	444.2094	-0.10743	453.0838	435.4885
YDR466W	502.0801	539.9069	617.5355	480.8465	587.6544	732.8476	0.118309	549.7717	811.273
YMR192W	523.9598	574.5163	593.8222	650.0829	536.9548	619.3492	0.094123	704.922	619.8688
YCL002C	626.4486	701.0879	589.8699	647.3966	613.0042	550.8587	-0.08216	483.4393	520.6546
YPL216W	398.4397	417.2907	511.8134	390.4079	459.7531	559.6647	0.086757	257.4595	346.8104
YOR148C	450.2599	508.264	428.8167	376.081	470.1235	403.1151	-0.15118	366.5145	395.9785
YDR415C	735.8468	832.6036	683.7353	778.1296	808.889	732.8476	0.042716	600.3642	575.0907
YNL232W	1750.371	1871.875	1771.586	1954.726	1954.239	1918.71	0.11161	1719.02	1627.814
YMR018W	41.45616	43.50898	41.49839	37.6081	26.50206	25.4393	-0.49796	20.23699	13.17002
YLR454W	1497.028	1271.649	1584.843	1412.99	1587.819	1877.616	0.164233	1189.486	1254.664
YNL291C	718.5734	670.4338	744.9948	752.1621	848.0659	802.3165	0.171002	965.7544	894.6831
YKL032C	2262.815	2280.266	2457.297	2490.194	2630.617	2850.18	0.187326	2509.387	2950.084
YMR193W	1320.839	1182.653	1275.581	1232.113	1202.963	1119.329	-0.08842	1237.83	1181.789
YJR002W	2928.417	2917.079	3026.418	3797.523	3569.712	4162.261	0.378012	3988.936	4138.897
YPR085C	627.6002	672.4115	496.0045	555.1672	625.6791	573.3627	-0.03398	523.9133	427.5865
YGR120C	732.3921	705.0432	714.3651	750.3712	728.2305	624.2413	-0.0332	603.737	586.5047
YDR002W	10889.15	9374.207	10677.93	11626.28	10710.29	10381.19	0.080541	13631.86	12405.28
YAL010C	274.0713	249.1878	294.4409	292.8059	277.6955	280.8108	0.058117	338.4075	336.2744
YMR272W-I	8.06092	0	0.988057	0	2.304527	2.935304	-0.78823	0	0
YMR137C	356.9836	263.0316	275.6679	265.943	263.8683	336.5815	-0.04797	151.7775	205.4523
YAL021C	2136.144	2288.177	2398.014	2379.16	2518.848	2933.347	0.198997	2546.488	2822.773

YEL016C	330.4977	459.8108	329.0229	334.8912	329.5474	388.4386	-0.0883	490.185	465.3406
YLR406C	13815.26	11236.19	12841.77	13654.43	13866.34	12444.71	0.076814	13248.49	11631.76
YAR014C	1385.327	1532.703	1472.205	1526.71	1559.012	1476.458	0.055426	1524.52	1800.78
YPR204W	6.90936	6.921883	13.8328	7.163448	10.37037	9.784347	-0.01815	8.99422	21.95003
YHR152W	1146.954	988.8404	1042.4	1137.197	1176.461	998.9818	0.059774	1359.251	1297.686
YLR097C	437.5928	465.7438	428.8167	465.6241	494.321	381.5895	0.010124	647.5838	528.5566
YCL052C	931.612	885.0122	971.2599	929.4574	894.1565	920.7071	-0.02272	1129.899	1034.285
YLR390W	503.2317	443.9894	482.1717	437.8658	453.9918	361.0424	-0.19013	433.9711	417.9285
YDL134C	1985.289	1824.411	1941.532	1911.745	1955.391	1850.22	-0.00852	1742.63	1662.934
YBR056W	1574.182	1578.189	1575.951	1464.925	1293.992	1395.248	-0.18677	1448.069	1201.983
YPR019W	847.5481	872.1573	827.0036	796.9336	988.6421	1036.162	0.14795	1073.685	1100.135
YPL190C	1251.746	1521.825	1732.064	1549.096	1648.889	2059.605	0.222671	1602.095	2114.227
YNL337W	0	0	0	0	0	0	#DIV/0!	0	5.268006
YHR204W	1083.618	1329.99	1136.265	1180.178	1288.231	1086.063	0.001867	852.2023	946.4852
YDR247W	102.4888	133.4935	121.531	120.8832	103.7037	106.6494	-0.11014	147.2803	153.6502
YCR024C-I	6470.615	6240.572	5961.935	6365.619	6873.252	5201.359	-0.01811	6267.847	5428.681
YBL073W	1.15156	14.83261	0.988057	8.95431	8.065844	8.805912	0.605652	0	0
YEL002C	4023.55	3466.875	3858.362	3829.758	3792.099	3942.113	0.027099	4733.208	4231.087
YDR213W	432.9865	432.1233	474.2673	428.9115	390.6173	547.9234	0.029928	496.9306	590.8947
YPL264C	101.3373	144.3707	132.3996	108.3472	102.5514	124.2612	-0.17395	69.7052	82.5321
YBR047W	125.52	113.7166	132.3996	105.6609	96.79013	86.10225	-0.36505	127.0434	64.97208
YLL002W	267.1619	251.1655	291.4768	275.7928	240.8231	296.4657	0.005827	304.6792	304.6664
YIL127C	1971.471	1923.295	1825.929	2401.546	2159.342	2029.274	0.204124	1776.358	1888.58
YNL165W	536.6269	401.4692	499.9568	480.8465	473.5803	446.1662	-0.03808	610.4827	567.1887
YER066W	160.0668	139.4265	162.0413	187.1451	125.5967	126.2181	-0.07235	96.68786	97.45812
YNR006W	1021.434	812.8268	865.5378	867.6727	887.2429	913.858	-0.01667	959.0087	1040.431
YPL128C	1000.706	1214.296	1039.436	1153.315	1243.292	1268.051	0.171271	928.6532	986.8732
YDR017C	1053.677	1139.144	1229.143	1190.028	1137.284	1666.274	0.22286	1370.494	1586.548
YDL240W	460.624	414.3241	567.1446	463.8333	457.4486	682.9474	0.153716	570.0087	721.7169
YPL239W	2424.034	2413.759	2330.826	2664.803	2555.72	2162.341	0.042485	3151.35	2817.505
YJR139C	5384.694	5210.2	5070.708	5504.215	5155.227	4608.428	-0.0371	4870.37	4106.411
YOL031C	1188.41	1101.568	1275.581	1361.951	1386.173	1367.852	0.207106	1309.783	1402.168
YJR158W	0	1.977681	5.928341	0	2.304527	1.956869	-0.89163	0	0
YMR032W	657.5407	620.003	661.01	736.9397	732.8396	770.0281	0.208394	730.7804	823.565
YBR193C	550.4457	537.9292	524.6582	502.3368	521.9754	473.5624	-0.10686	638.5896	637.4288
YNL177C	1476.3	1450.629	1497.894	1378.068	1342.387	1191.733	-0.17764	1267.061	1184.423
YIL068C	587.2956	487.4983	630.3803	604.4159	574.9795	846.346	0.248531	665.5723	714.6929
YGL033W	3.45468	21.75449	15.80891	18.80405	18.43622	18.59026	0.444794	6.745665	1.756002
YJL089W	41.45616	58.34159	75.09232	34.92181	33.41564	17.61182	-1.02489	12.36705	14.04802
YPR199C	686.3297	769.3179	715.3531	667.0961	772.0165	634.0257	-0.06654	600.3642	588.2607
YNR063W	74.8514	83.0626	91.88928	83.27509	93.33334	123.2828	0.263647	94.43931	158.9182
YDR234W	2633.618	3406.555	2758.655	3283.546	3086.914	3059.565	0.09995	2240.685	2371.481
YLR124W	12.66716	10.87724	28.65365	11.6406	19.58848	17.61182	-0.09591	30.35549	25.46203
YPL112C	1175.743	1294.392	1236.059	1361.951	1330.864	1269.03	0.096234	1470.555	1489.09
YDR080W	278.6775	314.4513	372.4974	334.8912	375.6379	354.1934	0.140941	403.6156	510.1186
YPR131C	787.667	889.9564	862.5736	981.3924	860.7408	778.834	0.045159	761.1359	667.2808
YJR064W	4272.287	4450.771	4387.96	4646.392	4763.457	5019.37	0.138213	5656.24	4994.07
YLR424W	432.9865	468.7104	460.4345	474.5784	512.7572	626.1982	0.244358	290.0636	372.2725
YLR189C	1180.349	1234.073	1273.605	1196.296	1145.35	1296.426	-0.01968	789.2428	950.8752
YOL149W	849.8512	825.6818	1007.818	879.3133	882.6338	800.3596	-0.06659	1145.639	981.6052
YCL033C	718.5734	756.4629	638.2847	643.8149	676.3787	595.8667	-0.14137	1007.353	858.6851
YLR412C-/	2.30312	3.955362	2.96417	10.74517	0	1.956869	0.461807	12.36705	5.268006
YIL158W	1122.771	1037.294	1155.038	1242.858	1288.231	1016.594	0.097823	1566.119	1328.416
YNL167C	648.3282	636.8132	729.1859	699.3316	697.1194	727.9554	0.076762	778	915.7551

YNL282W	329.3461	476.6211	451.542	492.4871	486.2552	463.7781	0.198023	283.3179	339.7864
YDR103W	756.5749	801.9496	806.2544	858.7184	939.0947	975.4994	0.229905	1023.093	947.3632
YBL099W	6372.733	6658.851	6410.513	6124.748	6204.939	5769.83	-0.10323	6481.46	6238.198
YPL107W	742.7562	776.2397	712.389	711.8677	592.2634	604.6727	-0.22527	590.2457	502.2166
YDR312W	1175.743	1150.021	1237.047	1321.656	1318.189	1452.976	0.20008	1089.425	1115.939
YPR079W	802.6373	703.0655	699.5442	720.822	789.3005	601.7373	-0.06243	773.5029	843.759
YOR046C	2641.679	2930.923	2919.708	3143.858	3090.371	3265.037	0.161659	3701.121	3297.772
YJR124C	915.4902	1143.1	848.7408	1075.413	936.7902	746.5457	-0.07568	771.2544	762.1049
YMR230W-/	0	4.944202	0	1.790862	2.304527	1.956869	0.291736	5.621387	5.268006
YPL159C	1270.171	1448.651	1239.023	1109.439	1106.173	992.1328	-0.30316	1082.679	945.6072
YLR298C	541.2332	546.8288	551.3357	539.0495	553.0865	493.1311	-0.04844	648.7081	645.3308
YPR156C	586.144	797.9942	641.2489	769.1753	783.5392	625.2198	0.104763	519.4162	530.3126
YJR135W-/	920.0964	699.1102	756.8515	649.1875	805.4322	814.0577	-0.06672	816.2254	683.9628
YKL114C	928.1573	1136.178	1061.173	1010.046	1056.626	876.6775	-0.08663	923.0318	880.6351
YOL110W	596.508	756.4629	509.8373	688.5865	564.6091	466.7134	-0.11515	447.4624	352.0784
YHR019C	10473.44	9949.712	9960.601	10930.53	10649.22	10507.41	0.078696	11540.71	11859.16
YER118C	1247.139	1327.024	1292.378	1480.147	1473.745	1431.45	0.181646	1609.965	1561.964
YBR005W	1016.827	1129.256	821.0752	859.6138	750.1235	569.449	-0.44529	1254.694	991.2632
YNR037C	1125.074	1025.428	970.2718	899.9082	958.6832	890.3756	-0.18301	1002.856	820.053
YDL066W	1597.214	1951.971	1758.741	1777.431	1648.889	1409.924	-0.13426	2139.5	2035.206
YCR064C	3.45468	8.899564	13.8328	8.058879	10.37037	10.76278	0.156722	10.1185	0
YLR126C	456.0177	480.5765	373.4855	417.2709	414.8148	374.7405	-0.11844	625.0983	572.4567
YNL247W	4175.556	4056.223	4377.092	4621.32	4397.037	4743.451	0.126231	4715.22	5096.796
YMR252C	460.624	419.2683	416.96	406.5257	382.5515	318.9697	-0.227	344.0289	298.5204
YLR157W-I	0	3.955362	1.976114	2.686293	2.304527	0	-0.24911	0	0
YJR061W	530.8691	475.6322	465.3748	593.6708	467.819	615.4354	0.188162	623.974	602.3087
YER076C	280.9806	327.3062	276.6559	245.3481	262.7161	212.3203	-0.29682	190.0029	259.0103
YPR138C	1031.798	1468.428	988.0568	981.3924	1091.194	777.8556	-0.29133	774.6272	798.981
YNL118C	2179.903	2166.549	2222.14	2018.302	2218.107	2440.216	0.023535	2308.142	2651.563
YBR101C	1341.567	1447.662	1122.433	1236.59	1164.938	931.4698	-0.23096	1627.954	1689.274
YDR507C	1548.848	1302.303	1761.705	1648.489	1719.177	2196.586	0.270527	1656.061	2094.033
YPR031W	731.2406	622.9695	699.5442	703.8088	606.0906	774.9203	0.021659	550.896	568.9447
YIL138C	2481.612	2351.463	2492.867	2461.54	2553.416	2478.375	0.032593	3085.017	2622.589
YKL210W	3227.823	3033.762	3739.795	3281.755	3554.733	4207.269	0.143032	3647.156	4029.147
YGR191W	2668.164	3059.472	2554.127	2819.712	2886.42	2729.833	0.026615	2519.506	2369.725
YCR048W	811.8498	1089.702	919.8809	1021.687	1007.078	969.6288	0.087762	1032.087	1063.259
YKL223W	2.30312	0	0	3.581724	0	3.913739	1.702428	0	0
YPL189W	162.37	201.7234	176.8622	231.0212	209.712	189.8163	0.221099	112.4277	135.2122
YDR541C	310.9212	339.1723	327.0468	301.7603	330.6996	370.8268	0.038096	368.763	409.1485
YDR153C	2404.457	2352.451	2508.676	2495.566	2401.317	2395.208	0.005254	1916.893	2166.907
YGR003W	475.5943	471.6769	475.2553	419.9572	445.926	544.9881	-0.01187	535.1561	633.0388
YJL004C	708.2094	692.1883	696.5801	661.7235	656.7902	555.7509	-0.16199	562.1387	575.9687
YLR360W	575.78	517.1635	497.9806	516.6637	503.5391	524.441	-0.04259	429.474	371.3945
YOR128C	4637.332	5211.189	4565.811	4927.557	4841.811	4552.657	-0.00927	5090.728	4958.95
YJL208C	1273.625	1359.656	1251.868	1290.316	1352.757	1190.755	-0.01918	1119.78	1179.155
YCR020C	188.8558	188.8685	171.9219	188.9359	207.4074	183.9457	0.078268	152.9017	127.3102
YOL028C	388.0757	555.7283	540.4671	521.1409	549.6297	594.8883	0.166338	477.8179	732.2529
YBR004C	621.8424	691.1995	517.7418	513.082	415.9671	429.5328	-0.43036	473.3208	381.0525
YML021C	553.9003	486.5095	522.6821	540.8403	488.5597	567.4921	0.030864	589.1214	639.1848
YGL028C	241.8276	297.641	266.7753	214.9034	247.7366	295.4873	-0.08878	194.5	166.8202
YGL244W	2300.817	2076.565	2380.229	2433.782	2483.128	2674.062	0.167771	2608.324	2703.365
YOR217W	681.7235	713.9428	907.0362	858.7184	812.3457	1312.081	0.373506	819.5983	1210.763
YJR054W	672.511	623.9583	559.2402	589.1936	655.6379	693.7102	0.063001	727.4075	766.4949
YLR276C	3686.143	3631.022	3735.843	4131.519	4243.786	4566.355	0.227584	3689.879	3253.872

YAR075W	0	3.955362	0	0	0	9.784347	1.306666	0	0
YOL084W	80.6092	87.01796	45.45061	50.14414	39.17696	17.61182	-0.99467	129.2919	131.7002
YER056C-/	19114.74	15117.39	16877.99	18002.64	18572.18	17456.25	0.08018	18971.06	16524.86
YPR041W	6080.236	6272.215	6080.502	6947.649	6443.457	6762.941	0.128782	7627.098	7247.021
YOR297C	572.3253	521.1189	519.7179	475.4739	523.1276	464.7565	-0.14061	426.1012	470.6086
YHL023C	617.2361	492.4425	719.3054	618.7428	607.2428	788.6184	0.139454	694.8035	851.661
YNR052C	2012.927	2025.145	2298.22	2221.564	2080.988	2168.211	0.030297	2390.214	2325.825
YGL091C	749.6655	785.1393	669.9025	770.0707	707.4898	635.9826	-0.06092	853.3266	866.5871
Q0045	3.45468	1.977681	0.988057	1.790862	6.913581	0	0.439085	8.99422	12.29202
YMR227C	620.6908	662.5231	651.1294	620.5337	593.4157	556.7294	-0.12754	706.0463	721.7169
YDL208W	8770.28	8214.297	8543.727	10155.98	9823.046	8098.504	0.137318	8673.801	7794.016
YJL140W	3643.536	2967.51	3528.351	3517.253	3149.136	3075.22	-0.05774	3206.439	2624.345
YMR086C-/	34.5468	24.72101	29.6417	27.75836	18.43622	36.20208	-0.10975	39.34971	50.92406
YNL123W	1543.09	1830.344	1701.434	1799.816	1783.704	2050.799	0.150871	1196.231	1558.452
YNL141W	3135.698	3711.118	3023.454	3920.197	4037.531	3266.015	0.185393	3296.382	3018.568
YER133W	2877.748	3159.345	3216.125	3087.446	3328.889	3221.007	0.05868	3685.382	3663.02
YOR093C	580.3862	543.8622	624.4519	595.4616	561.1523	839.497	0.190909	697.052	839.369
YHR184W	78.30608	75.15187	43.4745	60.88931	82.96297	45.008	-0.06038	49.46821	47.41206
YML090W	14.97028	9.888404	21.73725	8.95431	19.58848	27.39617	0.263649	3.372832	11.41401
YGL168W	58.72956	53.39738	57.3073	42.98069	47.2428	50.87861	-0.26399	52.84104	17.56002
YHR110W	1940.378	1501.06	1834.822	1697.737	1728.395	1520.488	-0.09307	1427.832	1434.654
YJR098C	245.2823	282.8084	311.2379	277.5836	267.3251	299.401	0.008537	191.1272	218.6223
YDR068W	970.765	863.2577	787.4813	981.3924	888.3951	776.8772	0.013781	1183.864	1034.285
YER068C-/	0	0	0	0	0	0	#DIV/0!	4.49711	0.878001
YNL265C	251.0401	244.2436	325.0707	253.407	285.7613	244.6087	-0.0658	355.2717	289.7404
YOR381W-/	2.30312	4.944202	0	6.268017	11.52263	5.870608	1.707007	22.48555	14.92602
YDL245C	8.06092	9.888404	9.880568	10.74517	9.218108	17.61182	0.433142	20.23699	19.31602
YLL014W	947.7338	974.0078	815.1469	924.9802	941.3993	765.1359	-0.05664	788.1185	646.2088
YJL205C	178.4918	193.8127	199.5875	181.7725	203.9506	130.1318	-0.14878	173.1387	172.0882
YKL125W	1297.808	1589.067	1560.142	1600.135	1593.58	1565.496	0.097885	1289.546	1360.902
YHR045W	923.5511	963.1306	1008.806	1086.158	1028.971	1186.841	0.189521	1213.095	1151.059
YBR050C	128.9747	99.87288	63.23564	44.77155	50.69959	47.9433	-1.02619	48.34393	40.38805
YPR070W	829.1232	780.1951	807.2424	733.358	857.284	899.1815	0.043088	888.1792	949.9972
YDR543C	2.30312	1.977681	0	0	4.609054	1.956869	0.617117	0	0
YPR178W	307.4665	298.6298	286.5365	313.4009	269.6297	306.2501	-0.00543	386.7515	347.6884
YNL270C	47.21396	50.43086	41.49839	31.34009	9.218108	19.56869	-1.21049	49.46821	67.60608
YMR296C	4803.157	4944.202	4774.291	5716.432	5542.387	4955.772	0.159087	5593.28	5821.147
YIL122W	369.6507	412.3465	386.3302	400.2577	304.1976	312.1207	-0.20073	394.6214	445.1465
YDL149W	240.676	205.6788	277.644	197.8903	171.6873	213.2988	-0.3128	259.7081	329.2504
YMR299C	485.9583	453.8778	448.5778	511.2911	456.2963	456.929	0.037035	359.7688	365.2484
YLR192C	6484.434	6455.15	6173.379	6518.738	6423.869	5804.075	-0.02792	5814.763	5480.483
YGL194C-/	128.9747	140.4153	100.7818	147.7461	132.5103	153.6143	0.22907	128.1676	129.9442
YHL037C	0	0.98884	0	0	3.45679	1.956869	2.452795	3.372832	6.146008
YDL244W	20.72808	27.68753	3.952227	16.11776	13.82716	9.784347	-0.39848	49.46821	35.12004
YBL108W	3.45468	11.86609	3.952227	3.581724	5.761317	2.935304	-0.65046	2.248555	0
YNL206C	1266.716	1181.664	1190.608	1281.362	1122.305	1188.798	-0.01856	1427.832	1358.268
YGR054W	4620.058	5278.43	4807.885	5312.592	5358.025	5478.256	0.134992	4298.113	4643.748
YNL019C	92.1248	95.91752	143.2682	107.4517	72.5926	45.98643	-0.55167	48.34393	48.29006
YEL076C-/	0	0	0	0	1.152263	0.978435	#DIV/0!	5.621387	1.756002
YBR013C	44.91084	61.30811	63.23564	56.41215	48.39507	65.55513	0.007708	73.07804	81.6541
YFR037C	1900.074	2119.085	2536.342	2573.469	2589.136	2834.525	0.286776	2385.717	2955.352
YPL253C	405.3491	350.0495	407.0794	372.4993	482.7984	481.3899	0.201459	473.3208	488.1686
YFR016C	1273.625	980.9297	1303.247	1212.414	1192.593	1534.186	0.146914	1286.173	1550.55
YMR052W	88.67012	107.7836	135.3638	96.70655	133.6626	100.7788	-0.00291	140.5347	126.4322

YOL060C	1574.182	1749.259	1693.529	1530.292	1474.897	1393.291	-0.18981	1070.312	1354.756
YOL037C	6.90936	8.899564	6.916398	3.581724	6.913581	5.870608	-0.47361	6.745665	10.53601
YDR034W-I	116.3076	88.99564	61.25952	71.63448	63.37449	91.97286	-0.2319	41.59827	67.60608
YCL001W-/	2.30312	13.84377	14.82085	2.686293	10.37037	11.74122	-0.32055	17.98844	14.04802
YBL082C	622.9939	820.7376	710.4129	792.4565	744.3622	717.1926	0.06538	896.0492	873.6111
YBR199W	2861.626	2746.999	2959.23	2861.798	2906.008	3183.827	0.063217	3445.91	3180.12
YPL228W	852.1544	673.4003	876.4064	677.8413	788.1482	992.1328	0.033344	939.896	1021.993
YLR330W	3348.736	3118.803	3530.327	3313.99	3438.354	3860.903	0.086174	3947.338	3892.179
YOL100W	272.9197	287.7526	388.3063	302.6557	339.9177	475.5193	0.236592	321.5434	610.2107
YPL019C	2046.322	1891.652	2163.844	1592.076	1569.383	1860.983	-0.28085	1613.338	1678.738
YFR021W	602.2658	700.099	596.7863	635.756	676.3787	585.104	-0.00145	791.4913	674.3048
YPR180W	674.8141	696.1437	738.0784	624.1154	677.5309	669.2493	-0.09773	761.1359	823.565
YKR084C	1341.567	1371.522	1350.674	1440.749	1442.634	1592.892	0.139482	1567.243	1487.334
YKR081C	3864.635	4115.554	3540.208	4608.783	4368.231	3713.16	0.139521	3943.965	3645.46
YOR357C	785.3639	769.3179	766.7321	719.0311	845.7614	681.969	-0.04716	648.7081	588.2607
YER101C	219.9479	288.7414	259.8589	258.7796	304.1976	274.9402	0.124672	191.1272	215.1103
YKL180W	35060.39	31313.61	33673.96	34663.03	34411.2	32081.9	0.015892	36012.86	30718.62
YJL127C-I	1151.56	1016.528	1019.675	1011.837	1110.782	932.4483	-0.06134	1036.584	834.979
YDR233C	15561.03	15241.99	14599.53	17021.25	17085.76	15530.69	0.128663	15757.87	14186.74
YOR295W	669.0563	636.8132	552.3238	656.3509	601.4815	571.4059	-0.02266	535.1561	451.2926
YLR365W	14.97028	10.87724	16.79697	7.163448	4.609054	13.69809	-0.74353	1.124277	10.53601
YFR032C-/	7558.839	6023.027	6154.606	6558.137	6760.33	6669.989	0.018303	8239.83	6232.052
YNL309W	550.4457	462.7773	569.1207	507.7094	511.605	545.9666	-0.01564	535.1561	616.3568
YNL043C	41.45616	31.64289	25.68948	40.2944	46.09054	19.56869	0.101018	26.98266	18.43802
YDR139C	1365.75	1116.401	1208.393	1240.172	1323.951	1252.396	0.048424	1318.777	1048.333
YLR412W	1074.405	903.8002	938.654	891.8493	989.7943	979.4131	-0.02787	864.5694	852.539
YPL043W	2893.87	3042.662	3046.179	3665.895	3625.021	3739.577	0.296274	3225.552	3055.444
YFL037W	7490.897	7160.194	7435.128	7302.24	7418.272	7286.403	-0.00519	8382.613	8166.288
YOR126C	564.2644	446.9559	458.4584	462.9378	526.5844	515.6351	0.034414	627.3468	590.8947
YDR208W	1328.9	1155.954	1330.913	1264.349	1381.564	1769.988	0.210735	1513.277	1681.372
YDL022W	16710.29	16404.86	15957.12	11207.21	10648.07	10024.06	-0.62229	15313.78	14425.56
YLR318W	216.4933	255.1208	209.468	265.943	221.2346	212.3203	0.038491	147.2803	172.0882
YDL004W	3311.886	2920.046	2963.182	2780.313	2782.716	2762.121	-0.14339	3099.633	2829.797
YNL187W	393.8335	356.9714	360.6407	321.4597	341.07	332.6678	-0.15938	215.8613	268.6683
YLR297W	409.9553	390.592	288.5126	403.8394	440.1646	366.913	0.153017	312.5491	235.3043
YEL013W	1016.827	1125.3	1073.03	1113.916	1086.584	1039.098	0.010926	827.4682	977.2152
YJL069C	2569.13	2695.579	2599.578	3026.557	3283.951	3290.476	0.287866	2583.59	2658.587
YDR328C	1848.254	1729.482	1937.579	1816.83	1847.078	1766.075	-0.0225	2138.376	2079.985
YFL017W-/	367.3476	376.7482	337.9154	357.277	386.0083	306.2501	-0.04396	356.396	235.3043
YDR456W	1000.706	1006.64	923.8331	851.5549	940.247	850.2598	-0.14982	776.8757	810.395
YJR074W	1171.136	1019.494	1086.863	1182.864	1068.148	864.9363	-0.07292	870.1908	725.2289
YDR138W	576.9315	557.706	616.5475	581.1347	627.9836	786.6615	0.188621	573.3815	788.445
YKR008W	876.3371	885.0122	1051.292	931.2483	1024.362	1162.38	0.148691	1070.312	1191.447
YBR248C	1766.493	2098.319	1895.093	1829.366	1878.189	1780.751	-0.06968	1913.52	2094.911
YGL143C	490.5645	606.1592	550.3477	530.9906	536.9548	492.1527	-0.07827	484.5636	479.3886
YOL093W	1541.939	1801.667	1647.091	1881.301	1871.276	1745.528	0.139693	1504.283	1355.634
YLR267W	199.2199	200.7346	193.6591	132.5238	173.9918	133.0671	-0.43339	128.1676	131.7002
YBL111C	1.15156	0	0	0	1.152263	0	0.000881	7.869942	0
YER120W	8394.872	6982.202	6890.708	7526.993	7240.824	6553.556	-0.06266	7123.422	5975.675
YLR440C	947.7338	849.4139	868.5019	949.1569	956.3787	968.6504	0.108666	798.237	664.6468
YDR109C	508.9895	635.8244	537.5029	510.3957	488.5597	549.8803	-0.11927	362.0173	434.6105
YDR309C	1991.047	2140.84	2269.567	2511.684	2499.259	2908.886	0.30707	1754.997	1950.04
YOL145C	2054.383	1728.493	1980.066	2045.164	1933.498	2339.437	0.132685	2183.347	2331.971
YPL227C	1362.295	1479.305	1547.297	1502.533	1580.905	1603.654	0.094835	1424.46	1267.834

YBR247C	3108.06	3419.41	3166.722	3764.392	3932.675	3837.421	0.250761	3091.763	3360.988
YBR252W	2017.533	1724.538	1804.192	2012.929	2024.527	1828.694	0.080898	1879.792	1545.282
YOR060C	184.2496	128.5493	145.2444	141.4781	159.0124	184.9242	0.083734	151.7775	251.1083
YHR001W	1771.099	1692.895	1703.41	1600.135	1534.815	1521.466	-0.15022	1407.595	1485.578
YNL271C	1199.925	1090.691	1323.008	1171.224	1317.037	1552.776	0.161279	1321.026	1716.492
YBL106C	363.8929	319.3955	346.8079	310.7146	347.9836	330.7109	-0.05814	283.3179	234.4263
YLR053C	88.67012	85.04028	76.08038	69.84362	81.81071	76.31791	-0.13186	148.4046	86.92211
YLR146C	703.6031	834.5813	715.3531	837.228	874.568	774.9203	0.14205	699.3006	681.3288
YFR011C	634.5095	606.1592	624.4519	635.756	565.7614	524.441	-0.11187	598.1156	550.5067
YKL124W	101.3373	188.8685	165.0055	164.7593	122.1399	153.6143	-0.04735	185.5058	197.5502
YDR021W	774.9998	924.5658	819.0991	1035.118	972.5104	1012.68	0.262037	818.474	703.2789
YJL188C	63.3358	34.60942	17.78502	58.20302	61.06996	48.92174	0.539367	43.84682	28.97404
YMR044W	1036.404	1082.78	1107.612	1123.766	1201.811	1236.741	0.142714	1308.659	1322.27
YBR207W	1053.677	1426.897	1169.859	1134.511	1058.93	1010.723	-0.18812	1017.471	997.4092
YOR183W	2.30312	0.98884	0.988057	1.790862	4.609054	1.956869	0.965332	10.1185	5.268006
YGL076C	36070.31	29193.54	35071.08	36861.31	35492.02	35312.69	0.101739	45516.37	40707.64
YFL011W	0	4.944202	0	1.790862	6.913581	1.956869	1.108575	4.49711	3.512004
YOR057W	984.5837	872.1573	820.0872	960.7975	882.6338	835.5832	0.001178	1156.882	1007.067
YDR182W	1203.38	1095.635	1191.597	1176.596	1374.65	1297.404	0.140873	1322.15	1379.34
YDR069C	369.6507	353.016	397.1988	328.6232	333.0041	519.5488	0.076899	550.896	498.7046
YHR198C	658.6923	737.675	581.9655	568.5987	592.2634	519.5488	-0.23547	550.896	598.7967
YPL231W	29350.96	30593.73	27553.94	31616.77	29865.52	30636.75	0.074239	25125.35	23951.87
YJL157C	3202.488	3116.825	3117.319	3632.764	3545.515	4109.426	0.258408	2702.763	2928.134
YBR289W	808.3951	791.0723	935.6898	879.3133	850.3704	1189.777	0.203626	921.9075	1172.131
YPL104W	893.6105	901.8225	935.6898	744.1032	876.8725	843.4107	-0.14827	773.5029	735.7649
YJL062W-/	1624.851	1482.272	1592.748	1442.539	1416.132	1192.712	-0.21421	1585.231	1309.978
YGR026W	2386.032	2215.991	2049.23	2421.245	2476.214	2339.437	0.121745	3001.821	2574.299
YKL044W	92.1248	91.96216	92.87734	99.39284	79.50618	87.08069	-0.05838	84.32081	146.6262
YMR258C	697.8453	800.9608	750.9232	610.684	693.6626	734.8045	-0.14178	537.4046	587.3827
YML036W	527.4145	544.8511	632.3564	481.7419	500.0823	505.8507	-0.19639	459.8295	383.6865
YJL041W	2116.567	2019.212	2228.068	2260.068	2325.268	2887.361	0.23173	2378.971	2817.505
YDL128W	1681.278	2095.353	1757.753	1909.954	1860.906	1743.571	-0.00521	1651.564	1610.254
YOL001W	254.4947	250.1766	217.3725	271.3156	290.3704	204.4929	0.085595	366.5145	297.6424
YNL082W	332.8008	357.9602	348.7841	349.2181	390.6173	517.592	0.274523	418.2312	465.3406
YDL190C	1233.321	1174.742	1380.315	1188.237	1261.728	1379.593	0.015598	1372.743	1546.16
YHR103W	1279.383	954.231	1378.339	1378.068	1457.613	1756.29	0.346335	1435.702	1703.322
YHR062C	1505.089	1683.995	1375.375	1844.588	1656.955	1530.272	0.140635	1417.714	1350.366
YIL131C	1171.136	1075.858	1142.194	1110.334	1184.527	1057.688	-0.01568	854.4509	976.3372
YDR418W	5700.222	4128.409	4752.553	5420.939	4919.013	4626.039	0.03758	5222.269	4277.621
YDL206W	191.1589	203.7011	211.4442	135.2101	126.749	169.2692	-0.49159	202.3699	214.2323
YHL048W	96.73103	106.7948	128.4474	115.5106	124.4445	129.1534	0.152978	69.7052	86.04411
YHR064C	7915.823	8529.738	8923.141	9374.267	9018.766	9195.329	0.12101	9614.821	9966.19
YBR083W	2017.533	2195.226	2132.227	2287.826	2434.733	2432.389	0.173324	2193.465	2379.383
YOR184W	4677.636	4810.709	4690.306	4613.261	4368.231	4352.078	-0.08866	4934.454	4879.93
YER128W	482.5036	496.3979	440.6733	538.154	482.7984	446.1662	0.047527	337.2832	369.6385
YOL119C	564.2644	689.2218	572.0849	649.1875	581.8931	512.6998	-0.06613	389	434.6105
YHR166C	398.4397	378.7259	331.9871	397.5714	334.1564	266.1342	-0.15255	517.1676	414.4165
YKL003C	1064.041	909.7332	1011.77	892.7447	977.1194	874.7206	-0.12141	936.5231	786.689
YNL097C-I	56.42644	103.8282	95.84151	82.37965	82.96297	62.61982	-0.16789	49.46821	66.72808
YBR250W	26.48588	28.67637	31.61782	27.75836	20.74074	21.52556	-0.3095	13.49133	19.31602
YBL088C	665.6016	639.7798	697.5681	641.1286	682.14	833.6264	0.10683	512.6705	567.1887
YHL006C	305.1634	238.3105	271.7156	226.544	253.498	217.2125	-0.22545	203.4942	173.8442
YDR079W	624.1455	684.2776	565.1685	549.7946	621.07	510.7429	-0.15597	584.6243	554.8967
YDL020C	1031.798	1007.628	954.4629	883.7904	890.6997	760.2438	-0.24019	957.8844	1079.941

YLL031C	1121.619	935.4431	1175.788	1070.936	1128.066	1459.825	0.178574	1270.434	1313.49
YLR363W-/	930.4604	1021.472	985.0927	1116.602	1227.161	909.9443	0.147729	964.6301	912.2431
YGL205W	48.36552	31.64289	22.72531	44.77155	51.85186	28.37461	0.282995	47.21965	54.43607
YJR136C	400.7429	373.7817	391.2705	430.7023	358.3539	346.3659	-0.03809	294.5607	318.7144
YGL081W	101.3373	103.8282	65.21175	101.1837	77.20165	66.53356	-0.14267	41.59827	64.09408
YGL137W	3841.604	3902.953	3991.75	4204.049	3975.309	4226.838	0.080082	4372.315	4808.812
YML102W	644.8736	943.3538	696.5801	889.163	757.0371	668.2709	0.01861	536.2804	529.4346
YLR357W	532.0207	543.8622	646.1892	651.8738	662.5515	897.2246	0.360977	572.2572	958.7772
YHR192W	849.8512	825.6818	901.1078	907.0716	925.2676	855.1519	0.060769	1041.081	998.2872
YNL276C	0	4.944202	0.988057	0	0	3.913739	-0.60003	0	0
YPR100W	64.48736	57.35275	50.3909	61.78474	54.15638	56.74921	0.003843	52.84104	38.63205
YDL209C	438.7443	605.1703	491.0642	412.7937	477.0371	394.3092	-0.25742	368.763	390.7105
YML009C	1032.949	898.856	879.3706	882.895	962.14	864.9363	-0.0529	1503.159	1346.854
YOR352W	376.5601	367.8486	354.7124	302.6557	320.3292	317.9913	-0.22412	439.5925	431.0985
YPL261C	13.81872	11.86609	3.952227	3.581724	12.6749	1.956869	-0.70239	0	3.512004
YLR093C	1501.634	1498.093	1471.217	1463.134	1389.63	1295.448	-0.10809	1677.422	1605.864
YNL288W	1361.144	1581.156	1401.065	1631.475	1568.231	1515.595	0.118537	1938.254	1587.426
YDL112W	1601.82	1570.279	1577.927	1818.62	1996.873	2346.286	0.375412	2108.02	2272.267
YDR035W	3048.179	3117.814	3131.152	3448.305	3293.169	3266.994	0.106361	3481.887	3496.2
YLR333C	11289.89	9875.549	9925.031	11077.38	11251.85	10236.38	0.066877	11555.32	9722.984
YMR162C	912.0355	818.7599	881.3467	768.2798	796.2141	1091.933	0.024254	577.8786	705.9129
YIL041W	6540.86	5977.54	5811.75	6340.547	6297.12	5339.318	-0.02807	5818.136	4732.426
YGL080W	1210.289	1180.675	1281.51	1199.878	1083.128	937.3405	-0.18954	1527.893	1372.316
YDR270W	442.199	514.197	512.8015	448.6109	417.1194	472.584	-0.13461	514.9191	648.8428
YBR044C	730.089	783.1616	842.8125	741.4169	782.3869	647.7238	-0.11767	644.211	654.9888
YHR026W	5479.122	5164.714	5116.158	5242.749	5216.297	4845.209	-0.04233	5397.656	4318.887
YBR215W	776.1514	643.7351	841.8244	733.358	838.8478	969.6288	0.168456	794.8642	1038.675
YNL197C	976.5228	953.2422	1250.88	974.229	1206.42	1491.135	0.207162	1117.532	1548.794
YOL047C	9.21248	5.933043	11.85668	10.74517	6.913581	5.870608	-0.19862	15.73988	25.46203
YDR119W	2033.655	2386.072	2211.271	2340.657	2500.412	2568.391	0.160142	2023.699	2005.354
YBR159W	3304.977	3085.182	2911.803	3156.394	3203.292	3100.66	0.024358	2947.856	2824.529
YER117W	26809.47	21166.13	24435.63	25129.38	25512.27	24598.83	0.055295	26839.88	22480.34
YDR142C	322.4368	359.9379	323.0946	263.2567	307.6543	282.7676	-0.2361	223.7312	270.4243
YLR116W	765.7874	912.6997	723.2576	772.757	678.6832	835.5832	-0.07061	884.8064	932.4371
YER076W-/	10.36404	5.933043	4.940284	3.581724	11.52263	0.978435	-0.40109	21.36127	13.17002
YIL056W	1500.483	1544.569	1437.623	1344.042	1177.613	1141.833	-0.29114	1364.873	1597.084
YGR207C	1715.824	1682.018	1482.085	1499.847	1457.613	1323.822	-0.18882	1381.737	1236.226
YPR104C	946.5823	932.4765	1020.663	970.6472	1111.934	1385.464	0.258208	810.6041	893.8051
YLL003W	709.3609	639.7798	861.5856	736.0443	723.6215	741.6535	-0.00615	573.3815	565.4327
YER095W	1440.601	1874.841	1362.53	1231.218	1303.21	1124.221	-0.35457	1669.552	1516.308
YDR386W	391.5304	393.5585	525.6462	470.9967	417.1194	576.298	0.159947	344.0289	343.2984
YOL051W	1503.937	1419.975	1944.496	1780.117	2109.794	2973.463	0.495468	1748.251	2806.969
YMR151W	6.90936	15.82145	12.84474	9.849741	5.761317	15.65496	-0.18629	4.49711	2.634003
YER115C	723.1796	667.4673	532.5626	522.9317	497.7778	509.7645	-0.32954	494.6821	421.4405
YDR066C	285.5869	266.9869	262.8231	298.1785	280	247.544	0.018155	230.4769	182.6242
YCR009C	3139.152	2750.954	3255.647	3040.884	3120.329	3068.371	0.013164	3619.049	3639.314
YDL197C	608.0236	540.8957	641.2489	642.9195	599.177	599.7805	0.041082	447.4624	496.9486
YML058W-/	367.3476	304.5629	300.3693	284.7471	292.6749	224.0615	-0.2787	254.0867	165.9422
YMR013W-/	6.90936	6.921883	4.940284	8.95431	9.218108	0.978435	0.028863	1.124277	0.878001
YDR370C	829.1232	712.954	633.3444	649.1875	702.8807	722.0848	-0.06877	622.8497	546.1167
YDR503C	665.6016	669.445	625.44	635.756	619.9177	548.9019	-0.11955	541.9017	395.1005
YBR190W	3.45468	7.910723	5.928341	0.895431	8.065844	9.784347	0.116303	1.124277	1.756002
YJL199C	291.3447	337.1946	268.7515	285.6425	250.0412	165.3555	-0.35608	173.1387	215.1103
YPR014C	46.0624	22.74333	41.49839	27.75836	47.2428	41.09426	0.073824	75.32659	47.41206

YIL065C	1166.53	860.2912	995.9613	954.5295	829.6297	796.4459	-0.22817	1262.564	1096.623
YER075C	681.7235	765.3625	871.4661	762.0118	844.6091	1124.221	0.236122	844.3324	1072.917
YOR180C	169.2793	147.3372	96.82957	130.7329	192.428	126.2181	0.120233	107.9306	115.8961
YOL007C	1411.812	1489.194	1739.968	1623.416	1676.543	1993.072	0.189666	1788.725	1807.804
YML015C	1305.869	1194.519	1273.605	1349.415	1226.008	1315.016	0.043841	919.659	1144.035
YJR129C	742.7562	684.2776	724.2457	877.5224	737.4486	727.9554	0.123117	541.9017	658.5008
YBR164C	1651.337	1541.602	1666.852	1679.829	1698.436	1449.062	-0.00967	2101.275	1811.316
YGR158C	1583.395	1584.122	1529.512	1785.489	1735.309	1613.439	0.128401	1897.78	1766.538
YOR210W	2399.851	2089.42	2017.612	2487.507	2534.98	2352.157	0.180607	2363.231	1930.724
YPL175W	538.9301	600.2261	516.7537	700.2271	622.2223	589.9961	0.207794	498.0549	454.8046
YLR312W-/	1346.174	1459.528	1320.044	1294.793	1230.617	1050.839	-0.20621	1308.659	1242.372
YKL133C	105.9435	150.3037	134.3757	127.1512	99.09466	107.6278	-0.22647	116.9249	93.06811
YBR287W	1164.227	1441.729	1205.429	1089.74	981.7285	862.9794	-0.37723	1545.882	1439.044
YBR037C	1336.961	1405.142	1232.107	1154.211	985.1853	827.7558	-0.42159	1043.33	931.5591
YNL009W	146.2481	173.0471	118.5668	92.2294	81.81071	81.21008	-0.77856	137.1619	174.7222
YBR049C	1646.731	1523.803	1847.666	1688.783	1855.144	2409.885	0.246644	1876.419	2236.269
YCR043C	1011.07	908.7444	1043.388	1078.994	1079.671	955.9307	0.071888	1072.561	871.8551
YNL002C	3803.602	4193.672	4049.057	4574.757	4614.815	3984.186	0.129073	3928.226	3840.377
YKR060W	1150.408	1089.702	1134.289	1284.048	1379.259	1222.065	0.203422	994.9856	1067.649
YCR022C	10.36404	9.888404	1.976114	15.22233	19.58848	2.935304	0.763914	15.73988	20.19402
YNL152W	529.7176	507.2751	583.9416	551.5855	503.5391	581.1902	0.013625	619.4769	633.0388
YGR102C	849.8512	782.1728	788.4693	834.5417	813.498	711.322	-0.0369	765.633	743.6669
YOL137W	483.6552	608.1369	530.5865	671.5733	549.6297	637.9394	0.196527	498.0549	436.3665
YPL124W	294.7993	277.8642	277.644	224.7532	263.8683	285.7029	-0.13505	310.3006	325.7384
YDR141C	747.3624	749.541	854.6692	697.5408	724.7737	875.6991	-0.03324	835.3382	890.2931
YGL260W	8.06092	1.977681	6.916398	0.895431	8.065844	3.913739	-0.39714	10.1185	9.658012
YPR172W	1039.859	1004.662	1094.767	1021.687	986.3375	784.7046	-0.16876	948.8902	773.5189
YNL025C	168.1278	195.7904	210.4561	175.5045	140.5761	195.6869	-0.1665	196.7486	281.8383
YKR025W	1789.524	1847.154	1841.738	1832.947	1805.597	1818.91	-0.00553	1681.919	1695.42
YER066C-/	10.36404	7.910723	4.940284	8.058879	8.065844	5.870608	-0.07786	11.24277	14.92602
YPL025C	26.48588	31.64289	19.76114	14.3269	50.69959	14.67652	0.033198	20.23699	12.29202
YEL005C	271.7681	260.065	251.9545	237.2892	328.3951	226.0184	0.014496	382.2543	316.0804
YDR498C	1303.566	1307.247	1227.167	1325.238	1187.984	1191.733	-0.05089	1009.601	1011.457
YMR061W	1498.179	1451.618	1604.604	1595.658	1580.905	1904.034	0.157737	1296.292	1360.902
YMR243C	6854.085	7096.908	7251.349	8008.735	8000.165	7670.928	0.159435	7042.474	6668.418
YKL097C	2.30312	16.81029	7.904455	17.01319	12.6749	5.870608	0.396289	4.49711	5.268006
YGL221C	2464.338	2154.683	2426.668	2529.593	2384.033	2436.302	0.060989	3115.373	2579.567
YML088W	810.6982	986.8628	995.9613	1007.36	920.6585	1052.796	0.093621	963.5058	1006.189
YGR192C	458323.2	384483.9	424012.7	430373.7	409114.6	406248.1	-0.02421	436610.9	365004.4
YEL072W	178.4918	278.853	177.8502	162.073	215.4733	145.7868	-0.27947	248.4653	235.3043
YBR163W	522.8082	396.525	522.6821	505.0231	513.9095	495.088	0.070298	364.2659	413.5385
YLR006C	796.8795	707.0209	734.1262	590.089	610.6996	610.5433	-0.30518	676.815	780.543
YKL072W	488.2614	432.1233	410.0436	399.3622	417.1194	511.7214	-0.00242	562.1387	496.9486
YGR271C-/	1011.07	906.7667	978.1763	1277.78	1356.214	1102.696	0.367693	1041.081	1002.677
YKL092C	405.3491	345.1053	425.8525	470.9967	460.9054	542.0528	0.325428	505.9249	503.0946
YIL092W	396.1366	473.6546	398.1869	531.886	486.2552	457.9074	0.21921	344.0289	333.6404
YNL209W	37972.69	36548.53	38208.16	40826.28	39757.7	42435.69	0.126026	47381.55	43342.52
YKL001C	1290.899	1424.919	1434.659	1327.029	1413.827	1123.243	-0.10314	1158.006	1144.913
YGR001C	1690.49	1608.843	1602.628	1819.516	1629.301	1663.339	0.060573	1504.283	1527.722
YKL070W	260.2525	329.2839	328.0349	246.2435	328.3951	227.9753	-0.19311	233.8497	221.2563
YHR208W	3346.433	3274.051	3315.919	3372.193	3427.984	3478.335	0.048836	3407.685	3559.416
YOR219C	537.7785	449.9224	591.846	565.9124	566.9136	626.1982	0.155265	602.6127	676.0608
YBR275C	815.3044	699.1102	721.2815	780.8159	846.9136	1090.955	0.282184	857.8237	1019.359
YBR143C	7305.496	7507.277	7328.417	7851.139	7579.589	7931.192	0.077426	7471.948	6996.791

YJR142W	675.9657	558.6948	607.6549	527.4089	521.9754	560.6431	-0.19443	671.1937	697.1329
YPL097W	510.1411	635.8244	544.4193	457.5653	474.7325	434.425	-0.30663	431.7226	494.3146
YLR222C-/	29.94056	12.85493	15.80891	15.22233	16.13169	19.56869	-0.2027	10.1185	10.53601
YPL012W	3528.38	3636.955	3980.881	4504.913	4695.474	5541.854	0.403402	3440.289	4223.185
YJR110W	430.6834	439.0452	420.9122	336.6821	297.284	465.7349	-0.23098	492.4335	480.2666
YGR190C	50.66864	55.37506	47.42673	51.03957	39.17696	58.70608	-0.0434	47.21965	80.7761
YNL095C	444.5021	521.1189	434.745	438.7612	428.642	418.7701	-0.12272	538.5289	543.4827
YMR092C	2280.089	2038	2332.802	2209.924	2129.383	2249.421	-0.01355	1880.916	1808.682
YER088C	2215.601	2194.237	2389.121	2336.18	2377.12	2319.869	0.048861	2445.304	2764.825
YDR340W	14.97028	7.910723	6.916398	7.163448	8.065844	6.849043	-0.43256	0	7.024009
YOL013W-/	29.94056	18.78797	19.76114	21.49034	32.26338	13.69809	-0.02203	2.248555	7.024009
YBR018C	11.5156	9.888404	3.952227	1.790862	4.609054	3.913739	-1.29778	17.98844	7.024009
YBR141C	540.0816	620.9918	526.6343	545.3175	548.4774	459.8643	-0.11939	520.5405	431.0985
YNL327W	2891.567	3027.829	3158.818	3177.885	3542.058	3999.841	0.239795	3253.659	3703.409
YPL060W	255.6463	217.5449	253.9306	256.0933	266.1729	243.6302	0.074952	201.2457	193.1602
YGL013C	760.0296	813.8157	920.869	786.1884	797.3663	1108.567	0.109869	798.237	1106.281
YNL263C	2185.661	2209.07	2341.695	2303.049	2484.28	2307.149	0.074713	2033.818	1907.896
YMR173W-/	32.24368	13.84377	19.76114	21.49034	19.58848	13.69809	-0.26558	17.98844	7.024009
YDR029W	39.15304	27.68753	38.53422	26.86293	34.5679	57.72765	0.177352	22.48555	69.36208
YDR481C	1711.218	2012.29	1797.275	1698.633	1680	1564.517	-0.15944	1897.78	1807.804
YJR125C	1963.41	1947.027	2094.68	1873.242	1925.432	1838.479	-0.09123	2399.208	2117.739
YHR063W-/	11.5156	3.955362	3.952227	15.22233	6.913581	8.805912	0.671778	8.99422	1.756002
YKR064W	480.2005	513.2082	598.7624	592.7753	601.4815	744.5888	0.284203	592.4942	755.0809
YGR146C-/	6.90936	11.86609	9.880568	17.90862	14.97943	5.870608	0.435681	4.49711	6.146008
YMR135W-/	0	1.977681	1.976114	3.581724	0	0	-0.14258	1.124277	5.268006
YGR063C	47.21396	51.4197	36.5581	48.35328	59.9177	39.13739	0.124811	8.99422	37.75405
YBR046C	656.3892	795.0277	743.0187	547.1084	562.3046	569.449	-0.38637	609.3584	544.3607
YAR018C	748.514	952.2533	804.2783	799.6199	815.8025	806.2302	-0.04884	667.8208	698.0109
YLR429W	5339.783	5094.506	5335.507	5120.97	5294.651	5081.011	-0.02521	4174.442	4570.874
YGL204C	94.42792	100.8617	90.90123	74.32078	123.2922	96.86504	0.041182	60.71098	71.11809
YJL127W-/	27.63744	35.59826	51.37895	16.11776	21.89301	32.28835	-0.70521	26.98266	64.97208
YOL085W-/	21.87964	18.78797	6.916398	16.11776	16.13169	12.71965	-0.08154	14.61561	5.268006
YDL071C	31.09212	42.52014	46.43867	25.07207	33.41564	26.41774	-0.49972	15.73988	25.46203
YDR433W	168.1278	108.7724	126.4713	143.269	129.0535	85.12382	-0.17438	214.737	193.1602
YJR037W	0	0	0	0	0	2.935304	#DIV/0!	0	4.390005
YNR026C	1004.16	966.0971	1132.313	1117.498	1073.91	1261.202	0.154223	1285.049	1197.593
YGL044C	793.4248	710.9763	692.6278	734.2534	634.8972	697.624	-0.08817	861.1965	755.9589
YIL102C	8.06092	12.85493	18.77308	0	0	0.978435	-5.34212	0	3.512004
YDR175C	1128.529	1297.359	1246.928	1104.066	1061.235	1059.645	-0.18761	1141.142	976.3372
YAL034W-/	259.101	250.1766	213.4203	215.7989	253.498	226.9969	-0.0537	182.133	198.4282
YDR074W	5719.798	5006.499	5839.416	3229.82	3159.506	4204.334	-0.645	5106.468	5621.841
YBR209W	46.0624	56.3639	50.3909	39.39897	39.17696	32.28835	-0.46301	33.72832	30.73004
YLL021W	2116.567	1656.308	2335.766	2146.348	2394.403	3150.56	0.332378	2331.751	2883.356
YDL024C	87.51856	76.14071	76.08038	51.03957	64.52675	34.24522	-0.67832	35.97688	71.11809
YHR050W	1552.303	1669.163	1595.712	1517.756	1526.749	1485.264	-0.08875	1328.896	1353
YGR220C	808.3951	847.4363	865.5378	801.4108	712.0988	765.1359	-0.14603	789.2428	718.2049
YFR036W	521.6567	458.822	455.4942	479.951	542.7161	387.4601	-0.0262	737.526	533.8247
YDR359C	772.6967	704.0544	644.2131	755.7438	804.2799	834.6048	0.175082	556.5174	684.8408
YBR140C	1410.661	1212.318	1384.268	1170.328	1327.408	1547.884	0.013749	1109.662	1169.497
YJL131C	560.8097	471.6769	529.5985	472.7876	460.9054	415.8348	-0.21102	453.0838	417.0505
YMR077C	401.8944	384.6589	338.9035	364.4404	298.4362	313.0991	-0.20559	455.3324	448.6585
YNR029C	954.6432	996.7512	1070.066	1241.067	1346.996	1242.612	0.342353	1032.087	1006.189
YLR398C	1102.043	1164.854	1182.704	1093.321	1201.811	1431.45	0.111424	1225.462	1417.972
YGR254W	37618.01	34497.68	36623.31	37033.24	33536.63	32580.9	-0.07611	52384.58	46500.69

YNL154C	3004.42	2767.764	3017.526	2849.262	2760.823	2856.051	-0.05411	3280.642	3339.916
YEL049W	41.45616	62.29695	54.34313	63.5756	40.32922	65.55513	0.100141	13.49133	46.53406
YMR247C	1077.86	993.7846	1133.301	1026.164	1054.321	1325.779	0.08789	1199.604	1381.096
YIL061C	658.6923	708.0098	634.3325	714.554	607.2428	648.7022	-0.02219	521.6648	539.0927
YJL083W	282.1322	266.9869	280.6081	251.6161	230.4527	305.2716	-0.07565	287.815	331.0064
YOL054W	525.1113	548.8064	524.6582	547.1084	566.9136	578.2549	0.082178	631.8439	661.1348
YDR486C	1752.674	1512.926	1553.225	1523.128	1547.49	1343.391	-0.12659	1358.127	1338.952
YHR217C	0	0	0	0	0	0	#DIV/0!	0	0
YKL165C-/	26.48588	30.65405	31.61782	32.23552	26.50206	20.54713	-0.16283	5.621387	19.31602
YNL104C	2945.69	3331.403	2771.499	2766.882	2778.107	2593.83	-0.15287	3008.567	3049.298
YER044C-/	21.87964	36.5871	59.28341	24.17664	29.95885	59.68452	-0.04897	22.48555	29.85204
YER150W	5686.403	6194.096	5389.85	5107.539	4491.523	3836.443	-0.36225	5914.824	4965.096
YLR199C	868.2762	947.3091	790.4455	833.6463	827.3252	749.481	-0.11255	1106.289	842.003
YLR388W	7011.848	5635.402	5760.371	6088.931	6486.091	5987.042	0.012054	7002	5786.027
YBR109C	5474.516	4486.369	5156.669	5114.702	4940.906	4643.651	-0.04048	6724.304	5861.535
YBR115C	1843.647	1968.781	2044.29	1984.275	2017.613	2275.839	0.10015	1983.225	2239.781
YLR082C	259.101	330.2727	311.2379	217.5897	241.9753	268.0911	-0.30765	375.5087	336.2744
YML127W	1117.013	1199.463	1304.235	1178.387	1228.313	1286.642	0.028653	1385.11	1340.708
YOL114C	16.12184	16.81029	16.79697	24.17664	18.43622	16.63339	0.252634	14.61561	47.41206
YDR389W	991.4931	874.1349	1057.221	1005.569	1079.671	1121.286	0.133636	1070.312	1248.518
YNL063W	374.257	383.6701	330.011	371.6039	327.2428	328.7541	-0.08232	336.159	318.7144
YPL116W	992.6447	1041.249	1012.758	891.8493	1061.235	1104.653	0.00524	770.1301	798.103
YDR378C	1479.755	1247.917	1250.88	1348.519	1385.021	1410.903	0.058935	1317.653	1147.547
YDL214C	152.0059	132.5046	163.0294	143.269	155.5556	134.0456	-0.04808	198.9971	230.0363
YJL095W	765.7874	685.2664	824.0394	783.5021	754.7326	994.0897	0.154537	969.1272	956.1432
YGL094C	742.7562	662.5231	821.0752	733.358	723.6215	994.0897	0.138728	890.4278	893.8051
YGL182C	0	8.899564	3.952227	9.849741	2.304527	0.978435	0.031194	0	0.878001
YDL092W	2351.485	2004.38	2055.158	2260.068	2416.296	1982.309	0.05468	2258.673	1956.186
YOL030W	6176.968	6565.9	6622.945	7254.782	7478.19	6804.035	0.153306	6026.127	5368.977
YPL182C	4.60624	6.921883	3.952227	7.163448	8.065844	11.74122	0.800945	2.248555	0.878001
YHR201C	1968.016	2002.402	1703.41	1846.379	1962.305	1792.492	-0.01859	2497.02	1992.184
YDR390C	1026.04	941.3761	1115.516	992.1376	1093.498	1194.669	0.089527	1205.225	1118.573
YOR168W	2354.94	2620.427	2889.078	3044.465	3224.033	4172.046	0.40878	3335.731	3699.897
YGR082W	4035.066	3721.995	3665.691	4013.322	3850.865	3364.837	-0.02468	3975.445	3548.002
YPL169C	2427.488	2615.483	2571.912	2598.541	2604.115	2688.739	0.051458	2717.379	2765.703
YPL067C	268.3135	336.2057	252.9425	285.6425	285.7613	236.7812	-0.08539	364.2659	356.4684
YGR116W	2341.121	1919.339	2688.503	2371.997	2833.416	3925.48	0.393958	2915.252	3351.33
YDL157C	1260.958	980.9297	1114.528	1104.962	1039.342	928.5345	-0.12735	1576.237	1259.054
YHR183W	16117.23	15418	14904.84	17083.03	15820.58	14304.72	0.023671	13534.05	12604.58
YLR148W	453.7146	404.4357	513.7895	449.5064	554.2387	657.5081	0.276055	516.0434	687.4748
YER132C	708.2094	724.82	846.7647	880.2087	835.391	1004.852	0.254943	643.0867	723.4729
YGR053C	70.24516	61.30811	54.34313	48.35328	34.5679	41.09426	-0.58398	77.57515	86.04411
YCR028C-/	3110.363	2669.869	2956.266	3002.38	3023.539	2717.113	0.001079	2793.83	2591.859
YIL143C	950.0369	1156.943	1083.898	1086.158	1037.037	1168.251	0.044768	1149.012	1283.638
YLR043C	7679.753	6566.889	7123.89	7115.99	7783.54	7028.097	0.037127	7685.561	6087.181
YPL203W	1515.453	1478.316	1516.667	1158.688	1223.704	1420.687	-0.2461	1450.318	1626.058
YER016W	1234.472	1268.682	1187.644	1187.342	1239.835	1052.796	-0.08486	1434.578	1293.296
YOL108C	536.6269	534.9627	548.3715	484.4282	462.0577	357.1287	-0.31344	672.3179	586.5047
YLR112W	190.0074	196.7792	203.5397	178.1908	154.4033	143.8299	-0.30927	147.2803	100.0921
YJL216C	109.3982	98.88404	130.4235	111.9289	130.2058	168.2908	0.277088	91.06648	81.6541
YIL024C	150.8544	158.2145	133.3877	134.3147	156.7078	128.1749	-0.07791	187.7543	231.7923
YNL289W	1278.232	1355.7	1267.677	1411.199	1329.712	1276.857	0.042325	1162.503	979.8492
YLR109W	38123.54	30335.65	33023.82	34225.16	33697.95	31040.84	-0.03626	52122.63	44473.39
YDR388W	5165.898	5017.376	4909.654	5056.499	5129.877	4681.81	-0.02164	5665.234	5222.35

YLL060C	254.4947	300.6075	256.8948	269.5247	212.0165	250.4793	-0.14959	196.7486	168.5762
YPL022W	741.6046	700.099	721.2815	620.5337	644.1153	904.0737	0.003822	737.526	1025.505
YJR052W	629.9033	590.3377	581.9655	551.5855	632.5926	648.7022	0.024348	706.0463	652.3548
YHR036W	523.9598	730.7531	736.1023	746.7895	660.247	730.8907	0.102853	494.6821	565.4327
YOR265W	1060.587	834.5813	911.9765	852.4503	947.1606	894.2893	-0.05941	801.6098	765.6169
YPL121C	13.81872	6.921883	15.80891	5.372586	16.13169	1.956869	-0.63958	2.248555	0.878001
YNL136W	966.1588	851.3916	862.5736	842.6006	803.1276	796.4459	-0.13413	928.6532	1082.575
YLR084C	1144.651	1255.827	1304.235	1312.702	1304.362	1681.929	0.214637	1245.699	1748.1
YFL034W	702.4516	691.1995	656.0697	644.7103	668.3128	677.0768	-0.04259	476.6937	484.6566
YGL159W	490.5645	501.3421	545.4074	504.1277	571.5227	470.6271	0.008387	499.1792	410.9045
YOR034C	732.3921	635.8244	620.4997	648.2921	700.5762	702.5161	0.04476	575.6301	547.8727
YHL026C	692.0875	1004.662	764.756	1068.249	896.461	795.4674	0.16522	709.4191	706.7909
YKL002W	2044.019	1802.656	1806.168	1918.909	1879.342	1768.032	-0.02226	2262.046	2049.255
Q0255	0	0	0	0	0	0	#DIV/0!	2.248555	1.756002
YMR234W	665.6016	695.1548	776.6127	770.0707	672.9219	718.1711	0.015972	552.0202	624.2588
YFR039C	815.3044	841.5032	761.7918	827.3783	790.4527	848.3029	0.028079	659.9509	658.5008
YHR014W	25.33432	60.31927	27.66559	59.09845	36.87243	25.4393	0.099497	4.49711	23.70603
YNL092W	157.7637	130.5269	113.6265	106.5563	127.9012	107.6278	-0.23254	183.2572	168.5762
YPL188W	854.4575	1052.126	931.7376	1078.994	1071.605	942.2326	0.123891	795.9885	796.347
YEL009C	19232.2	16990.26	16544.02	18187.99	17130.7	15002.34	-0.06846	17935.6	15195.56
YDR411C	1095.134	1455.573	1323.008	1179.283	1140.741	1003.874	-0.22084	985.9913	920.1451
YOR143C	851.0028	908.7444	779.5768	825.5874	890.6997	858.0872	0.019777	793.7399	741.9109
YJL099W	431.835	386.6366	383.366	326.8323	380.2469	358.1071	-0.17414	404.7399	479.3886
YMR226C	5412.332	5040.12	5279.188	5418.253	5169.054	4867.713	-0.02559	5266.116	4175.773
YML047C	0	1.977681	0	1.790862	0	1.956869	0.922208	2.248555	2.634003
YER125W	2086.627	2287.188	2331.814	2117.694	2180.082	2599.701	0.040696	2591.46	2768.337
YOR166C	596.508	624.9472	715.3531	615.1611	671.7696	825.7989	0.125427	516.0434	534.7027
YHR148W	1115.862	1527.758	1358.578	1567.004	1595.885	1411.881	0.192907	1216.468	1204.617
YDL093W	608.0236	733.7196	632.3564	652.7692	625.6791	568.4706	-0.09607	582.3757	551.3847
YGR208W	2047.474	2111.174	1894.105	2097.995	2016.461	2107.548	0.039788	2373.35	2117.739
YNR002C	209.5839	241.2771	216.3844	159.3867	91.02881	97.84347	-0.93806	237.2225	243.2063
YNL086W	204.9777	208.6453	175.8741	140.5827	132.5103	152.6358	-0.46955	254.0867	221.2563
YIR039C	71.39672	82.07376	57.3073	84.17052	66.83128	70.4473	0.071252	40.47399	33.36404
YDR205W	619.5392	787.117	664.9622	667.0961	645.2675	630.112	-0.09286	537.4046	552.2627
YNL330C	1737.704	1634.553	1751.825	1645.802	1553.251	1612.46	-0.0908	1444.697	1421.484
YBR122C	1859.769	1728.493	1824.941	1789.071	1680	1523.423	-0.11672	1756.121	1555.818
YJL020C	1313.93	1124.312	1411.933	1209.727	1236.379	1712.261	0.111093	1288.422	1701.566
YEL027W	9618.98	10305.69	9667.148	10824.87	10341.56	8238.42	-0.00914	9608.075	8138.192
YLR013W	3.45468	7.910723	3.952227	0	2.304527	7.827478	-0.59627	0	1.756002
YGR065C	764.6358	1053.115	869.49	939.3071	889.5474	832.6479	-0.01388	780.2486	640.0628
YML118W	195.7652	193.8127	218.3606	185.3542	117.5309	141.873	-0.4509	139.4104	145.7482
YLR373C	593.0534	603.1927	701.5203	651.8738	746.6667	900.1599	0.276516	695.9278	935.0711
YAL042W	4153.677	4240.148	3942.347	4178.081	4000.659	3468.551	-0.0829	5041.26	4270.597
YPL027W	9.21248	22.74333	26.67753	17.90862	17.28395	17.61182	-0.15106	15.73988	5.268006
YLR014C	419.1678	482.5541	421.9003	482.6373	570.3704	599.7805	0.32041	362.0173	392.4665
YLR386W	647.1767	605.1703	727.2098	635.756	676.3787	818.9499	0.10641	795.9885	856.929
YOR223W	295.9509	358.9491	325.0707	272.211	259.2593	260.2636	-0.30772	372.1358	322.2264
YER088C-/	126.6716	157.2256	128.4474	168.341	114.0741	112.52	-0.06223	124.7948	123.7982
YEL033W	497.4739	418.2795	312.226	380.5582	459.7531	282.7676	-0.12883	399.1185	274.8143
YER174C	941.976	875.1238	886.287	900.8036	739.7531	733.826	-0.18722	708.2948	662.0128
YHR143W	388.0757	447.9447	291.4768	279.3745	303.0453	318.9697	-0.3229	242.8439	251.1083
YGL156W	684.0266	580.4493	632.3564	511.2911	531.1935	494.1095	-0.30386	717.289	697.1329
YAR030C	27.63744	20.76565	16.79697	26.86293	31.11111	29.35304	0.421556	21.36127	17.56002
YGL056C	2600.222	3097.048	3043.215	2842.994	2738.93	2894.21	-0.04431	2909.63	3109.002

YDL139C	377.7117	285.7749	270.7276	257.8841	285.7613	246.5655	-0.24152	359.7688	410.9045
YGL153W	165.8246	228.4221	219.3486	191.6222	193.5803	185.9026	-0.10353	277.6965	243.2063
YLL046C	1.15156	1.977681	2.96417	8.058879	2.304527	5.870608	1.413698	0	0
YGR228W	4.60624	0.98884	0	0.895431	3.45679	2.935304	0.38127	0	0.878001
YJL012C	3830.088	3993.927	3898.872	3353.389	3242.469	3917.653	-0.15708	3236.795	3202.948
YBR059C	1843.647	1550.502	1955.364	1516.86	1478.354	1834.565	-0.14745	1873.046	2008.866
YDR018C	52.97176	72.18535	69.16398	63.5756	70.28807	39.13739	-0.16766	113.552	88.67811
YLR404W	498.6255	341.1499	404.1152	354.5907	286.9136	351.2581	-0.32534	276.5723	223.8903
YMR099C	3120.727	2685.691	2971.087	2989.844	2965.926	2825.719	0.000655	2838.801	2535.667
YOL115W	832.5778	867.2131	1032.519	933.9346	988.6421	1079.213	0.135702	1099.543	1076.429
YML078W	4464.598	3734.85	3883.063	3908.556	3710.288	3419.629	-0.13038	4172.194	3615.608
YGR144W	33.39524	21.75449	25.68948	34.02638	27.65432	25.4393	0.107949	68.58093	45.65606
YDR067C	444.5021	543.8622	458.4584	450.4018	503.5391	424.6407	-0.0697	589.1214	596.1627
YCR082W	1848.254	1376.466	1605.592	1419.258	1586.667	1455.911	-0.11448	1928.136	1801.658
YGR138C	1149.257	1644.442	1275.581	1552.677	1470.288	1202.496	0.054336	1533.514	1719.126
YIL140W	1263.261	1188.586	1427.742	1315.388	1410.37	1728.894	0.199409	1351.382	1513.674
YNR045W	279.8291	322.362	332.9752	199.6811	251.1934	246.5655	-0.42315	314.7977	375.7845
YLL035W	566.5675	702.0767	696.5801	662.619	679.8354	688.818	0.04769	608.2341	533.8247
YGL059W	173.8856	196.7792	214.4083	127.1512	138.2716	162.4202	-0.45154	192.2514	177.3562
YOR246C	2528.826	2728.211	2501.76	2712.261	2619.095	2300.3	-0.02384	2186.72	2160.761
YLL041C	1728.491	1732.448	1799.251	1531.187	1463.375	1539.078	-0.21445	1450.318	1490.846
YKR065C	2103.9	1818.478	1930.663	2024.57	1896.626	1502.876	-0.10981	1842.691	1777.952
YLR203C	2369.91	2486.934	2358.492	2356.774	2205.432	2250.4	-0.08286	2045.061	2361.823
YIR036C	973.0681	806.8938	834.908	843.496	760.4939	604.6727	-0.24357	862.3208	635.6728
YDL189W	1581.092	1527.758	1532.476	1661.025	1637.366	1705.412	0.108488	1225.462	1475.92
YER188C-/	0	1.977681	0	2.686293	2.304527	0	1.335467	0	0
YLR022C	1501.634	1472.383	1551.249	1596.554	1544.033	1490.156	0.033241	1500.91	1267.834
YBL078C	261.4041	223.4779	274.6798	133.4192	177.4486	182.9673	-0.62114	250.7139	315.2024
YPL110C	262.5557	270.9423	340.8796	214.9034	252.3457	340.4953	-0.11436	277.6965	325.7384
YHR017W	424.9256	430.1456	453.5181	474.5784	500.0823	380.6111	0.05057	580.1272	542.6047
YOR030W	480.2005	516.1747	463.3987	472.7876	498.9301	688.818	0.185904	563.263	616.3568
YJL090C	462.9271	456.8443	376.4497	428.016	523.1276	484.3252	0.14721	290.0636	423.1965
YGL053W	4.60624	22.74333	7.904455	14.3269	25.3498	31.30991	1.009759	13.49133	7.024009
YJR056C	496.3223	450.9112	389.2944	470.1013	440.1646	391.3739	-0.03816	324.9162	345.9324
YDL176W	594.2049	552.7618	529.5985	445.0292	455.1441	611.5217	-0.14934	406.9884	423.1965
YPL045W	722.0281	783.1616	791.4335	779.025	858.4363	940.2758	0.166591	849.9538	868.3431
YOR074C	883.2465	704.0544	748.9471	866.7772	705.1852	684.9043	-0.04987	632.9682	563.6767
YLR024C	268.3135	230.3998	249.9784	223.8578	202.7984	303.3148	-0.03653	259.7081	334.5184
YMR086W	1880.497	1811.556	2342.683	2179.479	2203.128	3100.66	0.310378	1515.526	1814.828
YIL133C	22078.86	19203.28	19791.77	22255.04	21221.24	19555.97	0.045534	22223.59	19162.37
YLR098C	385.7726	342.1388	386.3302	376.9765	358.3539	382.568	0.004727	381.1301	391.5885
YOR330C	698.9969	732.7308	820.0872	682.3184	644.1153	763.1791	-0.10785	666.6965	843.759
YPL005W	170.4309	173.0471	168.9577	152.2233	133.6626	126.2181	-0.31436	195.6243	186.1362
YDR454C	6261.031	5025.287	5625.008	6180.265	5801.647	5825.6	0.074496	5968.789	5155.622
YDR196C	1122.771	862.2689	1115.516	1037.805	897.6132	939.2973	-0.10911	972.5	834.101
YCR084C	4621.21	4608.985	5341.435	5106.643	5466.338	6436.144	0.223147	5260.494	6179.372
YIL149C	1562.667	1210.341	1776.526	1693.26	1986.502	2494.03	0.440438	1654.936	2636.637
YFR052C-/	33.39524	12.85493	12.84474	11.6406	24.19753	12.71965	-0.28333	13.49133	8.780011
YPR052C	1683.581	1287.47	1310.163	1481.938	1502.552	1027.356	-0.09375	1298.54	1064.137
YGL063W	216.4933	348.0718	229.2292	300.8648	262.7161	156.5496	-0.14051	138.2861	172.9662
YDL155W	1141.196	1109.479	1144.17	1078.994	1096.955	1143.79	-0.03228	1241.202	1196.715
YML074C	7984.917	7465.745	8121.827	7754.433	7510.453	7894.011	-0.02554	8940.255	8549.096
YPL242C	1122.771	1101.568	1155.038	1230.322	1208.724	1454.932	0.204488	1271.558	1548.794
YMR325W	0	0	0	0	0	0	#DIV/0!	0	0

YGR110W	186.5527	212.6007	148.2085	154.9096	149.7943	100.7788	-0.43286	119.1734	99.21412
YGL009C	1252.897	1294.392	1413.909	1317.179	1391.934	1390.356	0.0495	1253.569	1451.336
YMR306W	131.2778	183.9243	180.8144	171.9228	154.4033	161.4417	-0.02419	224.8555	187.8922
YDR467C	41.45616	22.74333	28.65365	30.44465	17.28395	24.46087	-0.36316	19.11272	10.53601
YEL020C-I	0	3.955362	5.928341	0	6.913581	0.978435	-0.32466	1.124277	0
YPL115C	643.722	622.9695	764.756	694.8545	755.8848	1037.141	0.292409	794.8642	985.1172
YIR002C	679.4204	662.5231	800.326	710.9722	769.712	892.3325	0.147582	898.2977	886.7811
YMR264W	2171.842	1667.185	1996.863	2079.191	2018.766	1719.11	-0.00466	2357.61	2189.735
YKR027W	426.0772	441.0228	407.0794	370.7084	399.8354	560.6431	0.063145	543.026	549.6287
YDL195W	4174.405	3920.752	4439.339	4127.042	4417.778	5595.668	0.173928	4623.029	5481.361
YLR227C	481.3521	566.6056	461.4225	472.7876	388.3128	414.8563	-0.24238	417.1069	378.4185
YKR047W	18.42496	31.64289	12.84474	19.69948	17.28395	17.61182	-0.20457	25.85838	45.65606
YDR350C	426.0772	448.9336	399.175	368.9176	315.7202	453.0153	-0.16351	260.8324	349.4444
YKL050C	168.1278	178.9801	163.0294	126.2558	202.7984	215.2556	0.093543	109.0549	111.5061
YKL190W	1948.439	1668.174	1609.545	1573.272	1765.268	1514.617	-0.10683	1667.304	1463.628
YKL120W	846.3966	896.8783	745.9829	876.627	974.8149	817.9714	0.100807	619.4769	611.0887
YER119C	735.8468	726.7977	777.6007	603.5205	610.6996	527.3763	-0.36325	568.8844	593.5287
YKL126W	1882.801	1887.696	2095.669	1842.797	2029.136	2189.737	0.047298	2213.702	2166.907
YKL093W	336.2555	426.1902	413.9958	331.3095	296.1317	242.6518	-0.43519	277.6965	270.4243
YOR304W	1013.373	911.7109	1213.334	1053.027	1155.72	1381.55	0.194066	1188.361	1301.198
YOR189W	1075.557	867.2131	877.3945	882.895	990.9466	820.9067	-0.06563	833.0896	772.6409
YLR356W	1429.086	1474.361	1266.689	1124.661	1199.506	988.2191	-0.33222	1063.566	934.1931
YGR035W-/	105.9435	129.5381	89.91317	99.39284	85.2675	91.97286	-0.23422	87.69364	91.31211
YGR071C	511.2926	464.755	592.8341	559.6444	562.3046	828.7342	0.314243	408.1127	491.6806
YPL113C	110.5498	127.5604	116.5907	87.75224	84.11523	104.6925	-0.35901	76.45087	50.04606
YDL226C	2483.915	2653.059	2494.843	2682.711	2514.239	2214.198	-0.04233	2156.364	2111.593
YNL198C	1.15156	0	1.976114	0	4.609054	0	0.559381	0	0
YPL241C	352.3773	441.0228	371.5094	395.7805	381.3992	364.9561	-0.02848	278.8208	316.0804
YNL213C	1034.101	779.2063	968.2957	913.3396	871.1112	796.4459	-0.10804	760.0116	849.027
YLR372W	5980.051	6684.561	5815.702	6218.768	6238.354	5158.308	-0.06915	5452.746	5390.927
YNL050C	1141.196	1328.013	1176.776	1319.865	1255.967	1166.294	0.03755	1160.254	1092.233
YER124C	140.4903	183.9243	181.8025	138.7918	159.0124	143.8299	-0.1969	118.0491	100.9701
YER074W	35502.59	28264.03	31625.72	33551.8	33446.75	31234.57	0.042336	35112.31	30056.61
YDR026C	931.612	865.2354	936.6779	966.1701	940.247	1090.955	0.132935	991.6127	1068.527
YBR139W	1133.135	1144.088	1195.549	952.7386	1093.498	894.2893	-0.24001	1255.818	1302.954
YDR494W	2269.725	1922.306	2095.669	2111.426	2170.864	2231.81	0.051034	2060.801	1999.208
YNR003C	754.2718	781.1839	844.7886	899.0128	894.1565	859.0657	0.156099	914.0376	916.6331
YFR028C	1737.704	1736.404	1760.717	1890.255	1819.424	1737.7	0.057421	1331.145	1619.912
YNL162W	20685.47	15115.41	18213.84	19970.8	19549.3	17572.69	0.079956	20344.93	17010.39
YAL051W	244.1307	352.0272	366.5691	331.3095	419.4239	512.6998	0.392151	402.4913	402.1245
YPL133C	473.2911	471.6769	497.9806	508.6048	460.9054	452.0368	-0.02156	335.0347	381.0525
YOR329C	841.7903	733.7196	813.1708	789.7702	743.2099	903.0952	0.028345	774.6272	884.1471
YDL129W	462.9271	542.8734	508.8493	559.6444	563.4568	536.1822	0.131576	492.4335	431.9765
YGL151W	990.3415	756.4629	1070.066	985.8696	1007.078	1144.769	0.155622	1046.702	1167.741
YJL043W	4.60624	15.82145	23.71336	27.75836	18.43622	19.56869	0.575161	37.10116	35.12004
YMR280C	148.5512	95.91752	119.5549	119.9878	79.50618	89.03756	-0.3353	141.659	135.2122
YCL066W	0	0	0	0	0	0	#DIV/0!	3.372832	1.756002
YLR305C	1520.059	1552.479	1567.058	1433.585	1641.975	1902.077	0.101462	1897.78	1776.196
YJR157W	2.30312	1.977681	3.952227	0	4.609054	0	-0.83695	0	0
YGR164W	3.45468	0	5.928341	6.268017	0	14.67652	1.15845	0	0
YDL116W	827.9716	852.3805	931.7376	999.301	935.6379	1144.769	0.237589	1175.994	1119.451
YAL024C	415.7131	385.6478	428.8167	380.5582	449.3828	570.4274	0.18694	401.3671	611.9667
YER055C	5001.225	5000.566	4863.216	4967.851	4773.828	4218.032	-0.09065	5773.165	5232.886
YMR172W	514.7473	531.0073	552.3238	493.3825	542.7161	654.5728	0.081258	538.5289	608.4547

YLR263W	317.8305	260.065	330.999	302.6557	298.4362	266.1342	-0.0677	194.5	251.1083
YBR026C	525.1113	513.2082	470.315	427.1206	365.2675	327.7756	-0.42953	366.5145	367.0044
YER031C	3326.857	2764.798	3011.597	2827.771	2934.815	2655.472	-0.11289	3838.283	3188.9
YDR346C	3450.074	3321.515	3519.458	3700.816	3929.218	4170.089	0.197412	3915.858	4321.521
YHL006W-/	70.24516	39.55362	48.41478	22.38578	42.63375	41.09426	-0.57626	33.72832	34.24204
YNR008W	1077.86	1290.437	1214.322	1297.48	1279.012	1321.865	0.121852	926.4046	1129.987
YDR451C	477.8974	480.5765	471.3031	493.3825	498.9301	550.8587	0.110108	529.5347	512.7526
YNL126W	324.7399	457.8331	536.5149	385.0353	467.819	524.441	0.062297	314.7977	334.5184
YJL060W	1581.092	1611.81	1507.775	1381.65	1359.671	1337.52	-0.20471	1098.419	1189.691
YNR015W	1630.609	1839.243	1624.365	1577.749	1540.576	1555.711	-0.12419	1424.46	1440.8
YOR233W	961.5525	977.9632	1115.516	1036.014	972.5104	1211.302	0.075795	860.0723	802.493
YGL237C	636.8126	531.0073	589.8699	528.3043	452.8395	453.0153	-0.29348	609.3584	640.9408
YDR166C	667.9048	609.1257	754.8754	718.1357	713.2511	952.9954	0.230782	761.1359	892.9271
YHR023W	849.8512	722.8424	902.0959	776.3387	891.8519	1017.572	0.118026	840.9596	1074.673
YDR421W	533.1723	542.8734	580.9774	521.1409	524.2799	632.0688	0.01771	535.1561	452.1706
YLR204W	1069.799	951.2645	926.7973	911.5488	1000.165	912.8796	-0.06163	1349.133	1242.372
YDL169C	163.5215	163.1587	150.1846	137.8964	119.8354	109.5847	-0.37656	245.0925	181.7462
YPL195W	893.6105	934.4542	895.1795	923.1894	928.7244	1025.4	0.079396	1057.945	1061.503
YCR044C	946.5823	862.2689	969.2837	933.9346	875.7202	882.5481	-0.04533	1187.237	937.7051
YMR084W	4.60624	11.86609	19.76114	26.86293	6.913581	3.913739	0.056869	28.10694	28.97404
YDR084C	901.6714	890.9452	959.4032	931.2483	933.3334	810.1439	-0.0411	935.3989	752.4469
YNL266W	5.7578	0.98884	1.976114	5.372586	3.45679	0	0.017528	0	0.878001
YER078W-/	0	0	0.988057	0	2.304527	0	1.221805	5.621387	3.512004
YHR143W-/	2922.659	3113.859	2811.022	3317.572	3463.704	2878.555	0.126722	3142.356	2774.483
YAL064C-/	0	0.98884	2.96417	0	3.45679	0	-0.19352	0	0
YPL052W	277.5259	344.1165	322.1065	310.7146	253.498	310.1638	-0.11015	387.8757	322.2264
YGL127C	696.6938	868.2019	703.4965	758.4301	773.1688	573.3627	-0.10788	991.6127	904.3411
YCR041W	2.30312	0	4.940284	7.163448	1.152263	0	0.199172	0	0.878001
YDR488C	203.8261	270.9423	223.3008	214.008	261.5638	282.7676	0.119474	186.6301	275.6923
YLL047W	2.30312	3.955362	0.988057	0	0	0	#NUM!	6.745665	3.512004
YGL119W	849.8512	1120.356	939.642	919.6077	857.284	922.6639	-0.10822	721.7861	805.127
YIL103W	1043.313	1105.524	959.4032	1093.321	1138.436	1234.785	0.157399	1006.228	968.4352
YBR090C	0	2.966521	0.988057	0.895431	2.304527	3.913739	0.847076	1.124277	0
YJR010W	1117.013	1433.819	1326.96	1551.782	1383.868	1431.45	0.17144	897.1734	1128.231
YOR177C	59.88112	74.16303	68.17592	59.99388	49.54733	43.05113	-0.40624	43.84682	48.29006
YNL324W	0	0	0	0	0	0	#DIV/0!	0	4.390005
YOR245C	703.6031	655.6012	729.1859	683.2139	615.3087	588.0393	-0.14663	605.9856	633.0388
YOR033C	865.9731	737.675	831.9438	768.2798	928.7244	845.3676	0.061903	992.737	901.7071
YHR121W	1211.441	1067.948	1170.847	1241.963	1032.428	1214.237	0.015965	1409.844	1311.734
YPR072W	2673.922	2309.931	2826.831	2761.509	2926.749	3473.443	0.230167	2996.199	3105.49
YJR036C	117.4591	65.26347	93.8654	55.51672	101.3992	63.59826	-0.32687	97.81214	137.8462
YLR096W	1133.135	1284.504	1263.725	1409.408	1472.593	1867.832	0.367637	1344.636	1763.026
YOL077C	4076.522	4101.71	3741.771	4615.052	4401.646	4180.852	0.146885	4182.312	3920.275
YFR044C	6076.782	5911.288	6073.585	6199.964	6104.692	5513.48	-0.01958	7452.835	6479.648
YDR317W	135.8841	175.0248	133.3877	154.9096	119.8354	91.97286	-0.27685	64.08382	85.1661
YBR243C	876.3371	1057.07	903.0839	866.7772	881.4816	746.5457	-0.18518	786.9942	754.2029
YMR122W-/	12387.33	12194.38	11730.21	12828.84	13361.65	11275.48	0.045138	13883.7	13309.62
YGR111W	963.8557	941.3761	1054.257	980.497	927.5721	967.6719	-0.04141	975.8729	853.417
YCR045C	234.9182	252.1543	237.1336	214.9034	241.9753	151.6574	-0.25106	169.7659	155.4062
YOR254C	3553.714	3263.173	3802.043	3939.897	4158.519	4639.737	0.262518	4723.09	4293.425
YER110C	4842.31	4945.191	5473.835	6034.31	6034.404	6983.089	0.320046	6246.486	6666.662
YOR089C	2709.621	2738.099	2432.596	2649.58	2496.955	2388.359	-0.06467	2486.902	2188.857
YLR118C	1126.226	989.8293	1029.555	1020.791	1004.774	978.4347	-0.06646	1027.59	906.0971
YGL248W	903.9746	990.8181	951.4987	746.7895	902.2223	839.497	-0.1938	780.2486	790.201

YJR066W	861.3668	891.9341	902.0959	778.1296	829.6297	884.505	-0.09147	901.6705	1023.749
YGL176C	541.2332	523.0966	592.8341	574.8667	580.7408	643.81	0.118814	417.1069	539.0927
YJL219W	52.97176	39.55362	49.40284	37.6081	18.43622	21.52556	-0.87159	26.98266	28.09603
YJL127C	255.6463	255.1208	354.7124	295.4922	357.2017	510.7429	0.426821	368.763	400.3685
YDR102C	14.97028	24.72101	13.8328	17.90862	17.28395	20.54713	0.058517	8.99422	7.90201
YPR112C	3134.546	3005.086	3139.057	3762.601	3682.634	4803.136	0.400597	2992.827	3202.948
YPR161C	1194.168	1227.151	1121.444	1103.171	1191.44	1116.394	-0.05468	1278.303	1294.174
YDR320C	1131.983	1008.617	1116.504	1068.249	1116.543	1209.345	0.059455	1235.581	1295.052
YDR028C	2519.613	1817.489	2246.841	2198.283	1955.391	2792.453	0.077257	2341.87	2791.165
YMR131C	5536.7	5752.085	6079.514	6882.283	7119.836	7716.915	0.322503	5872.101	5997.625
YPL160W	5863.743	5533.551	5949.09	6460.535	6660.083	8310.824	0.305094	7100.937	7155.709
YDR397C	3.45468	11.86609	5.928341	6.268017	2.304527	8.805912	-0.2901	3.372832	2.634003
YGL052W	2.30312	1.977681	0.988057	0	0	1.956869	-1.42894	0	0
YGL211W	1153.863	1322.08	1308.187	1481.938	1423.045	1406.011	0.188059	1164.751	1223.055
YKL087C	1274.777	1219.24	1253.844	1202.564	985.1853	1056.709	-0.20809	1036.584	1084.331
YLR292C	1809.101	1686.962	1603.616	1669.083	1674.239	1560.603	-0.05647	2039.439	1741.076
YMR072W	5594.278	4941.236	4984.747	5054.708	5112.593	4400.999	-0.09132	4790.546	4557.704
YDR097C	757.7264	644.724	775.6246	764.6981	853.8272	1064.537	0.300827	822.9711	916.6331
YOR066W	855.609	918.6328	875.4183	909.7579	995.5556	1138.898	0.200261	1007.353	1054.479
YPL014W	239.5245	245.2324	244.05	188.0405	273.0864	284.7245	0.033352	301.3064	292.3744
YGR100W	890.1558	866.2242	993.9852	794.2473	827.3252	889.3972	-0.13138	942.1445	1005.311
YLR128W	223.4026	293.6856	275.6679	268.6293	239.6708	252.4362	-0.05948	327.1647	323.9824
YIR024C	360.4383	376.7482	330.011	379.6628	367.572	335.6031	0.02099	315.922	323.1044
YDL053C	1660.549	1659.274	1465.288	1679.829	1712.264	1392.313	-0.00021	1523.396	1413.582
YDL207W	787.667	905.7778	852.693	830.0646	859.5885	947.1248	0.050466	910.6648	1012.335
YKL061W	329.3461	268.9646	328.0349	237.2892	205.1029	261.2421	-0.39672	286.6908	254.6203
YBR157C	234.9182	244.2436	234.1695	142.3735	135.9671	128.1749	-0.81126	115.8006	179.9902
YDR265W	445.6537	414.3241	351.7482	354.5907	380.2469	280.8108	-0.25466	529.5347	492.5586
YGR059W	12.66716	17.79913	26.67753	17.01319	13.82716	7.827478	-0.56346	4.49711	10.53601
YDR189W	1767.645	1495.127	1941.532	1838.32	1864.362	2262.141	0.196774	2061.925	1975.502
YGR290W	0	0	0	0	0	0	#DIV/0!	3.372832	0
YHR059W	783.0608	775.2509	822.0633	787.9793	772.0165	637.9394	-0.11504	712.7919	743.6669
YHR038W	545.8394	553.7506	518.7298	411.8983	439.0124	366.913	-0.41019	573.3815	626.8928
YKR050W	522.8082	634.8356	591.846	617.8474	547.3251	566.5137	-0.01476	432.8468	525.0446
YNL311C	298.254	335.2169	338.9035	274.0019	268.4774	337.56	-0.14394	308.052	409.1485
YBR057C	906.2777	831.6148	862.5736	885.5813	878.0248	935.3836	0.053649	968.0029	959.6552
YNL166C	1371.508	1317.135	1544.333	1472.089	1520.988	1633.008	0.128119	1514.402	1536.502
YHL028W	125.52	211.6119	175.8741	216.6943	179.7531	148.7221	0.087729	168.6416	210.7203
YCR003W	981.1291	827.6594	823.0513	804.0971	804.2799	754.3732	-0.1556	829.7168	746.3009
YJR060W	826.82	844.4697	942.6062	800.5153	785.8437	852.2166	-0.10016	577.8786	719.0829
YMR304W	785.3639	757.4518	817.123	695.7499	690.2058	946.1464	-0.01712	847.7052	878.8791
YMR294W-/	19.57652	24.72101	17.78502	34.92181	21.89301	16.63339	0.242539	12.36705	14.04802
YGL163C	477.8974	630.8802	649.1533	556.9581	549.6297	575.3196	-0.06378	522.789	719.9609
YDR045C	802.6373	814.8045	900.1198	994.8239	957.5309	877.6559	0.16878	906.1676	778.787
YMR087W	165.8246	144.3707	201.5636	179.0862	156.7078	173.1829	-0.00786	234.974	176.4782
YOL144W	1031.798	1189.575	959.4032	1314.493	1313.58	1198.583	0.266705	1119.78	1039.553
YNL220W	3689.598	4652.494	3909.741	4335.677	4149.301	3458.767	-0.03674	3349.223	3247.726
YBR254C	523.9598	580.4493	455.4942	479.0556	517.3663	541.0744	-0.02087	514.9191	386.3205
YBR113W	13.81872	15.82145	11.85668	17.90862	12.6749	40.11582	0.768695	15.73988	26.34003
YLR172C	3977.488	3864.388	3729.915	3966.759	3963.786	3220.029	-0.05349	3865.266	3350.452
YDL084W	8771.432	7940.389	8853.977	8271.992	8204.116	8825.481	-0.01499	7886.807	7653.535
YNL267W	1264.413	1413.053	1400.077	1258.976	1491.029	1781.73	0.152364	1417.714	1650.642
YGL155W	760.0296	717.8982	621.4877	727.9854	682.14	789.5968	0.067334	863.4451	771.7629
YDR209C	635.6611	444.9782	483.1598	637.5469	498.9301	505.8507	0.070687	464.3266	352.0784

YDR197W	485.9583	511.2305	424.8644	494.2779	504.6914	435.4034	0.012445	464.3266	417.0505
YDL055C	30872.17	24083.21	32402.34	31662.44	31239.01	32558.39	0.127959	33494.47	31845.98
YOR104W	133.581	223.4779	168.9577	206.8446	140.5761	225.04	0.122068	122.5462	142.2362
YOR116C	1695.096	1951.971	2020.576	2140.976	2326.42	2457.828	0.289112	1641.445	1871.02
YLR047C	211.887	297.641	238.1217	217.5897	183.2099	289.6167	-0.1149	229.3526	370.5165
YCR073C	605.7205	545.8399	544.4193	499.6505	585.3498	651.6375	0.034178	683.5607	588.2607
YPR011C	492.8677	434.101	544.4193	468.3104	425.1852	358.1071	-0.2334	383.3786	403.8805
YLR438W	3939.487	4621.84	3701.261	3764.392	3341.564	2926.498	-0.28959	3786.567	3094.954
YBR293W	409.9553	545.8399	395.2227	320.5643	460.9054	268.0911	-0.36426	308.052	263.4003
YGR168C	363.8929	297.641	273.6917	300.8648	282.3045	281.7892	-0.11268	359.7688	352.9564
YPR113W	4575.148	4376.608	4082.651	4376.867	4529.548	3849.162	-0.0312	5817.012	4565.606
YPL042C	603.4174	761.4071	766.7321	674.2596	626.8313	668.2709	-0.11418	643.0867	755.0809
YKL006W	34646.98	24420.4	30933.1	33944.89	31433.75	29857.91	0.081583	34769.41	27292.66
YLR431C	406.5007	441.0228	385.3422	379.6628	421.7284	447.1447	0.018222	283.3179	353.8344
YOR197W	2175.297	2283.233	2139.143	2115.008	2155.885	2324.761	-0.00044	2526.251	2288.949
YKL054C	4332.168	3682.442	4592.488	4810.255	5102.223	6019.33	0.337674	3206.439	4390.005
YOR020C	5931.685	4514.057	5400.719	5360.946	5127.572	5005.672	-0.03243	8483.798	6863.334
YPL100W	434.1381	458.822	496.9926	418.1663	409.0535	527.3763	-0.03717	516.0434	535.5807
YIL038C	1811.404	1759.147	1865.451	2026.36	2148.971	2666.235	0.331781	1998.965	2463.671
YFR031C	543.5363	569.5721	574.061	641.1286	668.3128	851.2382	0.35688	674.5665	757.7149
YDR424C	642.5704	583.4159	661.01	617.8474	712.0988	685.8827	0.095282	674.5665	541.7267
YKL004W	1968.016	2467.157	1900.033	2367.52	2514.239	1784.665	0.073521	2021.451	1891.214
YOR393W	0	0	0	0	0	0	#DIV/0!	1.124277	0.878001
YLR402W	70.24516	95.91752	56.31924	81.48422	54.15638	59.68452	-0.18781	37.10116	40.38805
YLR342W-/	163.5215	129.5381	82.99677	134.3147	149.7943	103.7141	0.044449	125.9191	86.04411
YMR078C	915.4902	824.6929	896.1675	861.4046	859.5885	1107.588	0.101536	629.5954	739.2769
YGR188C	393.8335	312.4736	314.2021	299.9694	360.6585	509.7645	0.197703	248.4653	305.5444
YER036C	12145.5	12209.21	12078.99	13173.58	13483.79	13216.7	0.130177	12170.3	11925.01
YER090W	2847.808	2502.755	2730.989	2603.913	2691.687	2895.188	0.01937	2915.252	2849.991
YKL127W	1935.772	1958.893	2169.773	2000.393	2276.873	2175.06	0.089445	2145.121	2208.173
YOR011W	178.4918	273.9088	211.4442	226.544	261.5638	281.7892	0.21382	139.4104	143.9922
YJL161W	157.7637	259.0762	184.7666	162.073	140.5761	174.1614	-0.3354	305.8035	300.2764
YPL020C	468.6849	481.5653	413.0078	483.5328	415.9671	546.945	0.085453	544.1503	557.5307
YDR440W	368.4992	353.016	425.8525	380.5582	359.5062	455.9506	0.059908	437.3439	474.1206
YEL028W	65.63892	77.12955	67.18786	74.32078	63.37449	34.24522	-0.28818	91.06648	87.80011
YLR327C	474.4427	547.8176	344.8318	196.0994	137.1194	90.01599	-1.69158	430.5983	398.6125
YHR176W	310.9212	316.4289	346.8079	288.3288	312.2634	276.897	-0.15077	263.0809	257.2543
YLR045C	1091.679	939.3984	1145.158	999.301	1144.198	1344.369	0.135028	1115.283	1292.418
YDL086W	852.1544	788.1058	929.7615	1024.373	917.2017	927.5561	0.158833	867.9422	806.883
YDR537C	2.30312	2.966521	1.976114	4.477155	4.609054	0.978435	0.474088	4.49711	7.024009
YDR260C	631.0548	654.6124	574.061	660.8281	511.605	581.1902	-0.08475	697.052	642.6968
YDR379W	930.4604	806.8938	949.5226	864.9864	975.9672	1179.014	0.168603	921.9075	1062.381
YDR530C	1015.676	1120.356	986.0807	1002.883	907.9836	869.8285	-0.16708	910.6648	847.271
YML003W	49.51708	31.64289	41.49839	38.50353	50.69959	82.18852	0.482651	58.46243	80.7761
YDR114C	0	0.98884	0	6.268017	0	1.956869	3.056186	1.124277	0
YNR061C	996.0993	1008.617	920.869	926.7711	1012.84	862.9794	-0.06196	1201.853	928.0471
YBL003C	17507.17	13083.35	15805.95	16270.88	15907	15017.02	0.024616	15666.81	13012.85
YMR035W	368.4992	462.7773	367.5571	374.2902	329.5474	330.7109	-0.21263	313.6734	288.8624
YPR061C	208.4323	268.9646	221.3247	166.5502	170.535	140.8946	-0.54777	105.6821	148.3822
YCL064C	3476.559	5078.684	3850.457	4040.185	3750.618	3286.562	-0.16339	4522.968	4539.266
YGR027C	25492.08	19736.27	21881.51	23221.21	23407.08	20782.93	0.006464	25944.95	20412.65
YNL071W	6848.327	6125.866	6742.5	6608.281	6261.4	6142.613	-0.05249	6899.691	7486.715
YKL165C	1220.654	1221.218	1215.31	1206.146	1211.029	1548.862	0.116966	1345.76	1523.332
YIL036W	792.2732	715.9205	737.0904	671.5733	655.6379	668.2709	-0.17016	740.8989	764.7389

YOR176W	2092.384	2148.75	2226.092	2009.347	2026.831	1811.083	-0.14539	1784.228	1762.148
YML034C-/	1.15156	0	0	0	1.152263	1.956869	1.432923	6.745665	2.634003
YDL144C	1149.257	1180.675	1089.827	1125.557	973.6626	847.3245	-0.21487	1002.856	927.1691
YPR008W	1384.175	1323.068	1537.416	1485.52	1423.045	1643.77	0.100958	1495.289	1684.884
YML063W	41224.69	35178.99	38328.7	41011.64	39831.44	38263.65	0.053982	42284.08	36187.69
YNL139C	570.0222	473.6546	633.3444	558.749	660.247	813.0792	0.277053	564.3873	820.053
YJL096W	1204.532	1044.215	1157.015	985.8696	1040.494	1007.788	-0.16668	1153.509	958.7772
YKL095W	170.4309	177.9913	210.4561	250.7207	233.9095	287.6598	0.466609	186.6301	282.7163
YDL103C	1407.206	1307.247	1460.348	1484.625	1418.436	1713.239	0.14503	1614.462	1618.156
YIR023W	551.5972	556.7172	676.8189	616.0565	670.6173	793.5106	0.22068	617.2283	762.9829
YMR281W	419.1678	371.804	392.2586	359.9633	364.1153	254.393	-0.27413	440.7168	417.9285
YOL033W	513.5957	514.197	430.7928	417.2709	391.7696	444.2094	-0.2189	415.9827	388.0765
YNL044W	1093.982	1157.932	1077.97	998.4056	1107.325	1054.753	-0.07533	1012.974	871.8551
YGR156W	734.6952	823.7041	701.5203	723.5083	640.6585	737.7398	-0.10457	522.789	598.7967
YDR384C	1459.026	1506.993	1280.522	1447.912	1372.346	1044.968	-0.13574	1056.821	1176.521
YDR239C	549.2941	494.4202	521.694	459.3561	587.6544	673.1631	0.136015	575.6301	680.4508
YDR325W	701.3	670.4338	628.4041	688.5865	707.4898	740.6751	0.095319	550.896	562.7987
YGL242C	927.0058	890.9452	885.2989	950.9477	836.5433	916.7933	0.000552	869.0665	792.835
YDR473C	477.8974	421.246	381.3899	450.4018	386.0083	394.3092	-0.05724	460.9538	475.8766
YPL224C	718.5734	746.5745	680.7712	577.553	655.6379	585.104	-0.23901	776.8757	737.5209
YNL140C	1.15156	4.944202	6.916398	7.163448	4.609054	4.892174	0.356933	2.248555	3.512004
YER176W	1271.322	1202.43	1389.208	1378.964	1540.576	1530.272	0.204038	1313.156	1597.084
YNL297C	1001.857	754.4853	920.869	782.6067	962.14	1084.106	0.079486	943.2688	1086.965
YJL183W	1943.833	1998.447	2043.302	2095.309	2132.84	2089.937	0.077996	1953.994	1752.49
YMR285C	865.9731	1047.182	902.0959	985.8696	1033.58	983.3269	0.093034	1086.052	1053.601
YOR149C	353.5289	467.7215	496.0045	470.1013	504.6914	442.2525	0.105351	378.8815	406.5145
YHR124W	87.51856	172.0582	104.734	162.9684	169.3827	78.27478	0.172655	85.44509	96.58012
YMR201C	719.725	787.117	683.7353	742.3123	710.9466	571.4059	-0.11363	556.5174	555.7747
YMR218C	426.0772	485.5207	569.1207	508.6048	623.3745	708.3867	0.313695	568.8844	686.5968
YDR091C	3205.943	3510.384	3696.321	3942.583	4257.614	4561.463	0.293479	4205.922	4444.441
YCR107W	588.4471	539.9069	583.9416	533.6769	551.9342	605.6511	-0.01783	493.5578	507.4846
YDL018C	590.7502	566.6056	604.6908	579.3439	573.8272	569.449	-0.03265	537.4046	519.7766
YOL130W	1259.807	1335.923	1281.51	1329.715	1378.107	1792.492	0.214996	1309.783	1733.174
YAL043C	1927.711	2000.424	2116.418	2026.36	1927.737	2063.519	-0.00644	1945	2195.881
YDR299W	2565.676	2669.869	2649.968	3063.27	3372.675	3456.81	0.327168	3436.916	3257.384
YOR363C	456.0177	476.6211	480.1956	366.2313	440.1646	447.1447	-0.17258	408.1127	490.8026
YKL215C	310.9212	450.9112	415.9719	283.8516	347.9836	433.4466	-0.14486	436.2197	469.7306
YOR008C	2303.12	2407.826	2468.166	2381.847	2494.65	2412.82	0.021978	2382.344	2713.023
YGL161C	1810.252	1705.75	1919.794	1793.548	1754.897	1796.406	-0.02434	2158.613	1936.87
YER017C	1535.029	1969.77	1892.129	1652.07	1705.35	1730.851	-0.08496	1407.595	1560.208
YBL001C	1867.83	1539.625	1761.705	1547.305	1573.992	1556.69	-0.14404	1838.194	1548.794
YGL049C	1788.373	1632.576	1962.281	1875.928	1829.794	2379.553	0.176851	2051.806	2102.813
YDR112W	11.5156	13.84377	10.86863	8.058879	3.45679	5.870608	-1.05916	23.60983	10.53601
YDR252W	161.2184	177.9913	132.3996	149.537	137.1194	138.9377	-0.14811	204.6185	174.7222
YOR193W	245.2823	206.6677	215.3964	195.204	178.6008	170.2476	-0.29469	167.5173	155.4062
YLR103C	489.413	469.6992	501.9329	499.6505	435.5556	607.608	0.078564	503.6763	551.3847
YLR175W	7479.382	8057.072	7701.903	8127.827	7984.034	7160.185	0.00209	7641.714	7000.303
YER094C	3793.238	3470.83	3492.781	3541.43	3438.354	3106.53	-0.09286	4219.413	3751.699
YJL204C	321.2852	358.9491	301.3573	347.4272	388.3128	471.6055	0.298644	337.2832	460.0726
YDL016C	230.312	197.7681	186.7427	196.9948	243.1276	191.7732	0.039516	210.2399	161.5522
YMR233W	532.0207	454.8666	465.3748	522.9317	482.7984	487.2605	0.039903	447.4624	351.2004
YDL160C-/	392.6819	305.5517	304.3215	307.1328	389.4651	312.1207	0.008842	339.5318	254.6203
YBL046W	693.2391	711.9651	671.8786	716.3448	662.5515	572.3843	-0.09014	725.159	716.4489
YIL155C	360.4383	486.5095	383.366	224.7532	235.0617	218.1909	-0.85966	294.5607	367.8825

YJR109C	997.2509	1067.948	1005.842	858.7184	857.284	1088.019	-0.13123	1039.957	1056.235
YDR184C	1045.616	970.0525	1131.325	1163.165	1230.617	1083.127	0.143831	934.2746	981.6052
YPL064C	1098.588	1105.524	952.4868	968.8564	864.1976	779.8125	-0.27274	902.7948	780.543
YNL240C	994.9478	1172.765	1002.878	1239.277	1132.675	978.4347	0.079576	1003.98	889.4151
YHR113W	1736.552	1633.564	1651.043	1664.606	1633.91	1511.682	-0.06192	1915.769	1748.978
YDL035C	731.2406	718.887	772.6604	806.7834	792.7573	883.5265	0.159753	584.6243	749.8129
YLR058C	12063.74	15173.76	12551.29	15894.8	16008.4	14713.7	0.228491	11102.24	10214.66
YIL132C	200.3714	202.7123	205.5158	206.8446	280	182.9673	0.138263	121.422	157.1622
YOR161C-(	11.5156	21.75449	15.80891	11.6406	3.45679	8.805912	-1.0379	2.248555	7.024009
YIL069C	15941.04	14717.9	14617.31	16315.65	16254.98	13853.66	0.036125	15730.89	14472.97
YNL250W	309.7696	318.4066	366.5691	346.5318	351.4404	475.5193	0.238408	363.1416	412.6605
YNR070W	66.79048	158.2145	110.6624	85.06595	116.3786	125.2396	-0.03914	148.4046	194.0382
YKL036C	0	0	0	0	0	0	#DIV/0!	1.124277	2.634003
YFL010C	1483.209	1405.142	1529.512	1654.757	1562.469	1281.749	0.026248	1579.61	1602.352
YNL076W	1247.139	1041.249	1171.835	1185.551	1200.659	1284.685	0.085266	937.6474	1042.187
YDR214W	3493.833	3454.02	3578.742	3237.879	3315.062	3021.406	-0.13679	2988.33	3078.272
YHR032W-/	140.4903	134.4823	137.3399	162.073	135.9671	139.9162	0.087049	154.026	86.04411
YPR046W	230.312	269.9534	248.9903	266.8384	283.4568	201.5576	0.004992	205.7428	196.6722
YIL071W-/	11.5156	15.82145	10.86863	17.90862	5.761317	3.913739	-0.46997	3.372832	4.390005
YBR070C	632.2064	498.3756	528.6104	607.9977	535.8025	540.096	0.021322	490.185	494.3146
YDR179C	234.9182	162.1698	198.5994	245.3481	221.2346	150.6789	0.051326	182.133	191.4042
YPR060C	1812.555	1774.969	1839.762	1972.635	2023.375	1809.126	0.097099	1659.434	1711.224
YBR256C	1126.226	1127.278	1108.6	1027.059	1031.276	1203.475	-0.04369	1459.312	1213.397
YPL050C	4363.261	4363.753	4219.003	4921.289	4871.77	4814.877	0.174244	4046.275	4375.079
YCR065W	1280.535	1129.256	1288.426	1372.696	1470.288	1614.417	0.269373	985.9913	1071.161
YJR147W	518.202	618.0253	520.7059	540.8403	504.6914	518.5704	-0.08318	527.2861	437.2445
YBL109W	0	0	0	0	5.761317	0	#DIV/0!	0	0
YER065C	485.9583	512.2193	474.2673	421.748	369.8766	404.0935	-0.30034	472.1965	492.5586
YGR198W	1485.512	1636.531	1714.279	1520.442	1602.798	1584.086	-0.039	1520.023	1812.194
YJR137C	1447.511	1882.752	1659.935	1712.064	1841.317	2238.659	0.214974	1458.188	1561.086
YHR079C	646.0251	546.8288	749.9351	630.3834	623.3745	814.0577	0.089978	674.5665	813.029
YDL045C	684.0266	702.0767	676.8189	648.2921	725.926	663.3787	-0.01782	834.2139	776.1529
YNL101W	959.2494	1063.992	911.9765	936.6209	909.1359	1231.849	0.068341	996.1098	1106.281
YLR211C	195.7652	163.1587	182.7905	153.1187	206.2552	148.7221	-0.09243	230.4769	238.8163
YOR237W	39.15304	47.46434	39.52227	55.51672	29.95885	33.26678	-0.08719	31.47977	15.80402
YBR174C	0	2.966521	1.976114	0.895431	0	0	-2.46463	4.49711	1.756002
YBR091C	634.5095	682.2999	673.8548	751.2666	691.3581	618.3707	0.050091	721.7861	524.1666
YDR393W	840.6388	825.6818	732.1501	727.09	727.0782	704.473	-0.15199	656.578	720.8389
YBR023C	1675.52	1972.737	2109.501	2070.237	2168.56	2812.021	0.292284	2083.286	2482.987
YLR107W	722.0281	609.1257	593.8222	640.2332	610.6996	522.4841	-0.11831	512.6705	532.9467
YML104C	456.0177	452.8889	524.6582	467.415	478.1893	579.2334	0.089049	521.6648	552.2627
YDL115C	610.3268	510.2417	472.2912	508.6048	535.8025	472.584	-0.07041	626.2226	618.9908
YLR194C	1760.735	1932.194	1668.828	1841.006	1646.584	1385.464	-0.13788	1961.864	1775.318
YIL146C	194.6136	231.3887	211.4442	211.3217	176.2963	159.4849	-0.22049	245.0925	290.6184
YKR067W	680.5719	584.4047	588.8819	419.9572	452.8395	520.5273	-0.412	619.4769	640.9408
YLR256W	1744.613	1658.285	1845.69	1561.632	1515.226	1868.81	-0.08576	1656.061	1890.336
YGL124C	392.6819	316.4289	400.163	396.6759	434.4033	414.8563	0.167614	341.7804	333.6404
YLR200W	2003.714	1478.316	1667.84	1868.765	1896.626	1684.865	0.081787	1702.156	1477.676
YLR355C	17286.07	19615.63	17944.1	19198.04	18458.11	18419.03	0.031981	19271.24	18739.18
YOR195W	746.2108	478.5988	737.0904	718.1357	815.8025	1038.119	0.390671	711.6676	975.4592
YCL061C	713.9672	555.7283	775.6246	691.2728	751.2758	989.1975	0.249666	724.0347	892.9271
YPL008W	484.8067	540.8957	619.5116	510.3957	487.4074	614.457	-0.02919	546.3989	640.0628
YCR020C-/	298.254	249.1878	284.5604	349.2181	290.3704	276.897	0.139525	351.8989	325.7384
YLL038C	253.3432	321.3731	220.3367	299.074	342.2223	437.3603	0.440113	284.4422	422.3185

YJL091C	528.566	538.918	592.8341	667.9915	683.2922	672.1846	0.285371	684.685	631.2828
YKR063C	647.1767	701.0879	656.0697	610.684	674.0741	644.7885	-0.05486	793.7399	759.4709
YOR384W	225.7057	232.3775	280.6081	252.5115	256.9548	342.4522	0.205744	286.6908	414.4165
YER097W	2.30312	5.933043	1.976114	0	1.152263	0	-3.14776	2.248555	1.756002
YDR232W	4099.553	5576.071	4186.397	4958.002	4595.227	3776.758	-0.05646	3919.231	3787.697
YOR251C	1456.723	1464.473	1503.822	1415.676	1449.547	1211.302	-0.11834	1427.832	1270.468
YNL300W	3266.976	3096.059	3112.379	3399.056	3725.268	3141.754	0.115624	3035.549	2734.973
YGR197C	749.6655	1012.573	827.0036	732.4626	758.1894	714.2573	-0.23181	762.2601	726.9849
YCR008W	1925.408	1789.801	1894.105	2004.87	2038.354	1935.344	0.091976	1948.373	1912.286
YNL093W	154.309	189.8574	120.5429	190.7268	138.2716	120.3475	-0.0485	166.3931	143.1142
YER121W	178.4918	180.9578	167.9697	52.83043	109.465	93.92973	-1.04154	119.1734	129.9442
YBR106W	14102	12003.53	13729.05	14684.17	13692.35	12677.58	0.043505	14561.64	11541.32
YGL097W	2967.57	3224.609	3065.94	3257.578	3275.885	3102.616	0.057727	2987.205	2816.627
YGL230C	34.5468	39.55362	23.71336	44.77155	36.87243	20.54713	0.06316	20.23699	19.31602
YLR312C	36.84992	33.62057	21.73725	26.86293	4.609054	36.20208	-0.44628	8.99422	20.19402
YPR137W	2314.635	2286.199	2233.008	2692.561	2778.107	3063.479	0.32055	2575.72	2108.081
YNL010W	4911.403	3845.6	4431.435	4473.573	4461.564	4274.781	0.002348	5485.35	4791.252
YGR218W	2893.87	2646.137	2769.523	2770.464	2908.313	3193.611	0.094555	2934.364	3332.892
YOL095C	262.5557	247.2101	254.9187	234.6029	276.5432	283.7461	0.055895	335.0347	333.6404
YDL051W	2402.154	2594.717	2797.189	2765.986	2797.696	2903.016	0.119424	3028.804	2949.206
YOR026W	669.0563	735.6973	638.2847	634.8606	710.9466	583.1471	-0.0829	601.4885	539.0927
YBR283C	5891.381	6439.329	5646.745	6106.84	6077.038	5090.796	-0.05753	5950.801	5319.809
YBL005W	502.0801	534.9627	545.4074	459.3561	485.1029	620.3276	-0.01619	427.2254	375.7845
YHR090C	625.297	559.6837	661.01	510.3957	555.391	498.0233	-0.23933	522.789	487.2906
YER032W	846.3966	698.1213	1000.902	840.8097	1049.712	1308.167	0.329577	916.2861	1106.281
YHR214C-I	0	0	0	0	0	0	#DIV/0!	0	0
YHR138C	655.2376	584.4047	549.3596	513.082	502.3869	401.1582	-0.33669	764.5087	715.5709
YGR166W	557.355	605.1703	607.6549	615.1611	587.6544	700.5593	0.104663	703.7977	612.8447
YOR147W	434.1381	461.7885	508.8493	428.9115	525.4321	433.4466	-0.01755	596.9913	586.5047
YDL111C	1521.211	1906.484	1582.867	1827.575	1680	1406.011	-0.0282	1482.922	1362.658
YER048W-/	1642.124	1503.037	1579.903	1418.363	1603.951	1402.097	-0.09485	1581.858	1453.97
YMR066W	355.832	313.4624	386.3302	392.1988	335.3087	463.7781	0.174422	384.5029	496.0706
YHR186C	629.9033	732.7308	891.2273	833.6463	752.428	990.1759	0.192874	915.1619	890.2931
YGR042W	720.8765	612.0922	573.073	669.7824	648.7243	675.12	0.064816	598.1156	514.5086
YAL027W	203.8261	196.7792	221.3247	159.3867	200.4938	176.1182	-0.21452	268.7023	238.8163
YDR465C	2082.02	2144.795	2215.223	2427.514	2288.395	2347.265	0.132799	2113.642	2163.395
YDR182W-/	8.06092	10.87724	13.8328	9.849741	5.761317	9.784347	-0.36785	23.60983	27.21803
YMR091C	1298.96	1378.444	1330.913	1363.741	1274.403	1333.607	-0.01322	1213.095	1552.306
YAL014C	945.4307	890.9452	787.4813	935.7254	931.0289	751.4379	-0.00312	943.2688	1079.941
YDL081C	38455.19	28197.77	31893.49	34712.28	34675.06	33100.45	0.056576	39249.65	31082.99
YKR007W	1042.162	862.2689	840.8364	883.7904	755.8848	702.5161	-0.22909	1081.555	991.2632
YNL168C	696.6938	864.2465	867.5139	821.1103	808.889	838.5186	0.023607	1011.85	986.8732
YJR069C	1797.585	1692.895	1714.279	2026.36	1828.642	1691.714	0.091803	1694.286	1558.452
YJL187C	979.9775	763.3848	1054.257	882.895	1010.535	1344.369	0.210814	973.6243	1123.841
YEL022W	1194.168	1205.396	1464.3	1383.441	1456.461	1642.792	0.214322	1503.159	1635.716
YMR283C	498.6255	547.8176	469.327	584.7165	450.535	510.7429	0.028484	403.6156	426.7085
YPL138C	503.2317	543.8622	450.5539	464.7287	505.8437	455.9506	-0.0702	417.1069	404.7585
YNL190W	4302.228	4877.95	4590.512	5057.394	4936.297	4949.901	0.117926	5456.119	5505.067
YPR087W	6.90936	2.966521	4.940284	4.477155	10.37037	1.956869	0.181667	12.36705	18.43802
YMR141C	117.4591	69.21883	81.02066	78.79793	84.11523	77.29634	-0.15632	114.6763	98.33612
YMR306C-/	19.57652	5.933043	9.880568	9.849741	2.304527	2.935304	-1.2298	4.49711	0.878001
YGL220W	1765.341	1627.631	1525.56	1690.574	1867.819	1508.746	0.042944	1759.494	1391.632
YDR458C	806.092	856.3358	955.451	1004.674	898.7655	1123.243	0.209339	1030.962	1050.089
YLL027W	1931.166	2060.743	1835.81	1894.732	1699.589	1393.291	-0.22458	1548.13	1424.996

YDL156W	131.2778	119.6497	185.7547	128.0466	165.9259	234.8243	0.27613	130.4162	180.8682
YMR096W	282.1322	369.8263	289.5006	276.6882	246.5844	280.8108	-0.22755	249.5896	258.1323
YCR024C	346.6195	446.9559	401.1511	385.0353	334.1564	389.417	-0.10793	338.4075	316.0804
YCR079W	412.2585	499.3644	400.163	372.4993	269.6297	240.6949	-0.57133	442.9653	405.6365
YNL184C	0	1.977681	0	0	2.304527	0.978435	0.731188	0	0
YFR019W	644.8736	496.3979	640.2608	582.0302	505.8437	718.1711	0.019715	616.1041	665.5248
YDL217C	900.5199	947.3091	915.9287	763.8027	689.0536	733.826	-0.33789	675.6908	706.7909
YGR204W	8850.89	10395.68	9091.111	9719.904	9280.33	9534.846	0.010015	8908.775	9051.313
YMR157C	494.0192	472.6657	456.4823	397.5714	485.1029	354.1934	-0.20241	456.4567	420.5625
YIL159W	339.7102	348.0718	381.3899	367.1267	428.642	484.3252	0.259756	295.685	328.3724
YBL100W-(	10.36404	3.955362	1.976114	4.477155	9.218108	10.76278	0.585834	0	6.146008
YLR461W	0	0	0	7.163448	2.304527	0.978435	#DIV/0!	0	0
YOR351C	11.5156	19.77681	25.68948	18.80405	16.13169	15.65496	-0.17163	29.23121	22.82803
YPR032W	492.8677	672.4115	647.1772	565.9124	583.0453	614.457	-0.03957	622.8497	656.7448
YDR341C	7615.266	6644.019	7411.414	7578.033	7946.009	8023.165	0.11981	8460.188	8161.02
YDR420W	968.4619	897.8671	1024.615	940.2026	1138.436	1402.097	0.267852	638.5896	959.6552
YDR082W	460.624	490.4649	405.1033	372.4993	402.14	451.0584	-0.14596	335.0347	337.1524
YPL153C	358.1351	357.9602	500.9448	422.6434	435.5556	496.0664	0.154134	485.6879	448.6585
YCR013C	57.578	41.5313	14.82085	31.34009	61.06996	35.22365	0.16386	101.185	50.92406
YPR094W	153.1575	247.2101	191.683	208.6354	228.1482	166.3339	0.026719	159.6474	136.0902
YMR191W	1050.223	1089.702	1065.125	963.4838	835.391	882.5481	-0.25735	883.6821	771.7629
YBL055C	1288.596	1423.93	1281.51	1290.316	1221.399	1067.472	-0.15821	1186.113	1185.301
YGL226C-/	978.8259	937.4207	939.642	1003.778	1037.037	862.001	0.023514	974.7486	947.3632
YOR314W-/	2.30312	0.98884	0	1.790862	3.45679	2.935304	1.313675	0	0
YNL158W	374.257	532.985	463.3987	479.0556	413.6626	495.088	0.017956	512.6705	575.0907
YPR162C	583.8409	663.5119	578.0132	524.7226	571.5227	672.1846	-0.04572	465.4509	498.7046
YGR017W	598.8112	733.7196	781.5529	749.4758	675.2264	702.5161	0.008936	882.5578	789.323
YPL118W	2388.335	2179.404	2164.833	2086.354	2057.943	1868.81	-0.16305	2182.223	2243.293
YDL132W	2202.934	1972.737	2008.72	2061.282	2099.424	2312.041	0.065747	2112.517	2423.283
YMR166C	436.4412	687.2441	549.3596	612.4748	542.7161	486.2821	-0.02749	435.0954	451.2926
YPL122C	725.4828	686.2553	588.8819	610.684	672.9219	767.0928	0.035668	494.6821	608.4547
YMR057C	49.51708	12.85493	26.67753	39.39897	28.80659	13.69809	-0.12068	20.23699	14.92602
YNL313C	2120.022	2331.686	2357.504	2372.892	2524.609	2821.806	0.180984	2669.035	2850.869
YJL084C	572.3253	632.8579	769.6963	681.423	709.7943	816.0146	0.160474	580.1272	586.5047
YOL076W	1138.893	1162.876	1270.641	1151.524	1259.424	1457.868	0.114994	1373.867	1416.216
YPL157W	773.8483	859.3023	755.8635	764.6981	867.6544	753.3947	-0.00197	765.633	696.2549
YDR515W	416.8647	450.9112	558.2521	566.8078	449.3828	600.7589	0.181272	614.9798	578.6027
YHR070W	929.3089	1139.144	1013.746	1220.472	1207.572	1199.561	0.235057	861.1965	989.5072
YLR186W	3384.435	3298.772	3296.158	3654.254	3612.346	3311.023	0.083996	3509.994	2986.082
YGR075C	368.4992	396.525	385.3422	368.0222	358.3539	298.4226	-0.16675	346.2775	278.3263
YLR370C	2130.386	1877.808	2114.442	1964.576	1809.054	1867.832	-0.11808	2199.087	2194.125
YKR048C	5254.568	5839.103	5311.793	6605.595	6029.795	5655.353	0.156937	6136.306	5811.489
YOL136C	951.1885	1024.439	979.1643	802.3062	864.1976	916.7933	-0.19384	907.2919	1018.481
YBR251W	872.8824	864.2465	791.4335	883.7904	750.1235	780.7909	-0.06647	1092.798	963.1672
YJL142C	44.91084	33.62057	26.67753	44.77155	34.5679	32.28835	0.085439	20.23699	32.48604
YOR072W-/	2.30312	7.910723	7.904455	8.95431	2.304527	17.61182	0.672157	5.621387	0.878001
YHR091C	589.5987	736.6861	693.6159	611.5794	700.5762	651.6375	-0.04064	507.0491	626.8928
YNL130C-/	27.63744	11.86609	9.880568	18.80405	17.28395	6.849043	-0.20182	11.24277	10.53601
YLR169W	186.5527	183.9243	171.9219	200.5765	172.8395	167.3123	-0.00445	173.1387	128.1882
YMR158W	829.1232	764.3737	827.9916	649.1875	785.8437	653.5944	-0.21334	726.2833	719.9609
YDR300C	1563.818	1550.502	1517.655	1557.155	1517.531	1444.17	-0.03567	1737.009	1702.444
YML016C	768.0905	824.6929	962.3673	945.5752	987.4898	1224.022	0.305186	738.6503	811.273
YAL044W-/	11.5156	6.921883	6.916398	3.581724	3.45679	6.849043	-0.86841	2.248555	2.634003
YHR065C	2086.627	2188.304	2062.075	2501.834	2422.058	2659.386	0.25902	2097.902	2051.889

YLR035C	411.1069	380.7036	439.6853	331.3095	328.3951	346.3659	-0.29168	323.7919	268.6683
YPR086W	2259.361	2298.065	2450.381	2561.828	2316.05	2301.278	0.034851	2617.318	2596.249
YNL307C	2663.558	2339.596	2486.939	2376.474	2654.815	2891.275	0.080984	2796.078	2697.219
YDR240C	426.0772	445.967	371.5094	389.5125	396.3786	399.2014	-0.06947	477.8179	416.1725
YFR038W	869.4278	766.3513	944.5823	946.4706	917.2017	1056.709	0.178584	991.6127	990.3852
YJL094C	447.9568	542.8734	456.4823	430.7023	418.2716	399.2014	-0.21356	554.2688	521.5326
YPR051W	24.18276	22.74333	31.61782	35.81724	36.87243	32.28835	0.418516	23.60983	22.82803
YMR270C	741.6046	753.4964	638.2847	703.8088	631.4404	628.1551	-0.11979	809.4798	806.005
YDR007W	667.9048	725.8089	692.6278	667.9915	595.7202	513.6782	-0.23122	609.3584	507.4846
YML029W	914.3386	921.5993	839.8483	818.424	893.0042	1008.766	0.023747	882.5578	1026.383
YOL022C	1687.035	1818.478	1539.393	1862.497	1722.634	1800.32	0.09424	2034.942	1925.456
YCR036W	1082.466	1243.961	1126.385	1061.981	1037.037	919.7286	-0.19382	1245.699	1084.331
YMR262W	604.569	577.4828	571.0968	461.147	464.3622	454.9721	-0.34478	456.4567	449.5365
YHR127W	1059.435	904.789	989.0449	984.0787	1025.514	944.1895	0.000251	1036.584	1033.407
YOR269W	316.679	277.8642	312.226	236.3938	336.4609	328.7541	-0.00823	359.7688	404.7585
YLR271W	246.4338	183.9243	174.8861	195.204	185.5144	126.2181	-0.25571	214.737	267.7903
YJL200C	1596.062	1788.812	1719.219	1890.255	2046.42	1998.942	0.217743	1671.801	1869.264
YGR262C	734.6952	829.6371	724.2457	787.9793	840.0001	774.9203	0.070325	1023.093	896.4391
YBR031W	53007.46	48934.75	48769.5	54438.62	50928.89	46158.64	0.007775	68140.21	55607.32
YER185W	25.33432	11.86609	12.84474	18.80405	13.82716	17.61182	0.005694	4.49711	7.024009
YAR003W	610.3268	734.7084	804.2783	710.0768	695.9671	678.0553	-0.04445	572.2572	618.9908
YPL168W	268.3135	280.8307	265.7873	205.9491	233.9095	308.2069	-0.12351	275.448	295.0084
YPL063W	3766.753	3630.033	3583.682	3556.652	3538.601	3319.829	-0.07627	3610.055	3245.092
YNL099C	958.0979	908.7444	1101.683	1113.021	1002.469	1070.408	0.101953	1154.633	1029.017
YDL130W-/	1727.34	1482.272	1573.975	1318.074	1352.757	1190.755	-0.3089	1560.497	1470.652
YDL014W	9057.019	13216.84	9072.338	11212.59	10683.79	8120.03	-0.06254	8625.457	8985.463
YGR174C	955.7947	935.4431	745.9829	831.8554	737.4486	709.3652	-0.21083	961.2572	811.273
YMR040W	93.27636	124.5939	96.82957	118.1969	94.4856	78.27478	-0.11317	80.94798	80.7761
YER163C	1194.168	1178.698	1115.516	1059.295	1159.177	1060.623	-0.08926	1216.468	948.2412
YGL018C	598.8112	572.5386	541.4551	651.8738	554.2387	468.6702	-0.03239	761.1359	698.8889
YMR172C-/	0	5.933043	3.952227	1.790862	8.065844	6.849043	0.756992	0	0
YMR277W	1675.52	1902.529	1834.822	2004.87	1960	2120.268	0.168896	1937.13	2191.491
YOL120C	45164.18	35529.04	40584.43	42360.16	41626.67	39548.33	0.026608	46762.07	38240.46
YNL225C	360.4383	340.1611	332.9752	317.878	354.8971	414.8563	0.073547	408.1127	400.3685
YBL039W-I	165.8246	155.2479	170.9338	125.3603	146.3375	102.7356	-0.39397	148.4046	105.3601
YNR025C	62.18424	62.29695	66.19981	53.72586	53.00412	62.61982	-0.17115	44.9711	55.31407
YHR144C	451.4115	412.3465	418.9361	443.2384	524.2799	402.1367	0.094635	623.974	456.5606
YLR198C	39.15304	25.70985	14.82085	42.98069	33.41564	15.65496	0.208152	17.98844	19.31602
YIL046W-/	2.30312	3.955362	3.952227	1.790862	6.913581	12.71965	1.069151	0	5.268006
YDR311W	846.3966	720.8647	903.0839	793.3519	762.7984	1017.572	0.059144	888.1792	927.1691
YGR068C	517.0504	799.9719	571.0968	625.9063	670.6173	641.8532	0.037899	546.3989	500.4606
YJR067C	528.566	368.8375	450.5539	440.5521	475.8848	374.7405	-0.06209	412.6098	320.4704
YGR101W	622.9939	629.8914	515.7657	499.6505	421.7284	482.3683	-0.33337	655.4538	594.4067
YGR193C	1516.604	1413.053	1489.99	1519.546	1514.074	1556.69	0.05466	1495.289	1643.618
YFL021C-/	12.66716	4.944202	22.72531	11.6406	6.913581	9.784347	-0.50933	5.621387	6.146008
YGL064C	453.7146	537.9292	466.3628	522.9317	486.2552	526.3979	0.074791	438.4682	405.6365
YDR064W	33627.85	26786.7	30036.93	31336.5	31482.14	28187.73	0.008823	35490.07	27779.08
YHR020W	7884.731	8079.815	7958.798	8600.615	8499.095	9301.979	0.142211	9754.231	9174.233
YMR017W	43.75928	44.49782	33.59393	27.75836	29.95885	32.28835	-0.43703	52.84104	34.24204
YER019C-/	3309.583	2730.188	2909.827	2981.785	3116.873	2744.509	-0.01726	3336.856	2967.644
YOR268C	0	0	1.976114	7.163448	4.609054	1.956869	2.796528	20.23699	7.024009
YOR070C	614.933	641.7574	697.5681	528.3043	521.9754	635.0041	-0.21363	604.8613	735.7649
YDR057W	836.0325	742.6192	715.3531	663.5144	774.3211	763.1791	-0.0597	602.6127	639.1848
YGL007C-/	9.21248	8.899564	9.880568	6.268017	3.45679	0.978435	-1.387	10.1185	16.68202

YER140W	627.6002	580.4493	688.6756	658.1418	637.2017	665.3356	0.047843	582.3757	586.5047
YCR050C	1.15156	0	0	0	1.152263	0	0.000881	3.372832	1.756002
YDR174W	6742.383	6708.294	6685.192	7383.724	6607.079	6093.691	-0.00369	6335.304	5889.631
YAL029C	1766.493	1637.52	2113.454	1875.928	2131.687	2870.727	0.318055	2230.567	2482.987
YBL037W	672.511	669.445	703.4965	586.5073	627.9836	674.1415	-0.11508	688.0578	703.2789
YDR331W	687.4813	915.6662	818.1111	902.5945	963.2923	915.8149	0.200211	989.3642	993.0192
YCR015C	358.1351	321.3731	423.8764	357.277	315.7202	308.2069	-0.16931	342.9046	273.9363
YLR218C	172.734	129.5381	159.0771	170.1319	130.2058	142.8515	-0.05794	159.6474	92.19011
YDR150W	2631.314	1803.645	2798.177	2126.649	2390.947	3006.73	0.056941	2720.751	3143.244
YCR081W	232.6151	214.5784	274.6798	222.0669	222.3868	352.2365	0.142273	240.5954	319.5924
YOL078W	419.1678	409.3799	501.9329	510.3957	465.5144	651.6375	0.290752	491.3093	575.9687
YKL090W	529.7176	463.7662	496.0045	444.1338	553.0865	507.8076	0.014973	411.4856	406.5145
YIL121W	229.1604	293.6856	213.4203	282.9562	229.3004	261.2421	0.071171	214.737	208.0863
YCL058C	0	1.977681	0	1.790862	3.45679	0.978435	1.654516	0	0
YNL246W	1737.704	1975.703	1650.055	1797.13	1824.033	1559.625	-0.04999	1536.887	1559.33
YDR056C	1977.228	1698.828	1922.759	1860.706	2071.77	1828.694	0.04124	1921.39	1672.592
YDL046W	5709.434	5631.446	5498.536	5656.438	5470.947	4810.963	-0.07934	5610.145	5049.384
YPR120C	457.1693	476.6211	451.542	434.284	474.7325	538.1391	0.062988	512.6705	529.4346
YBR123C	682.875	684.2776	650.1414	662.619	653.3334	646.7453	-0.03958	568.8844	554.0187
YNL041C	572.3253	575.5051	688.6756	616.0565	646.4198	1009.745	0.30714	826.3439	753.3249
YML049C	801.4857	634.8356	925.8092	773.6524	843.4569	966.6935	0.129407	833.0896	902.5851
YHR172W	325.8915	329.2839	430.7928	347.4272	364.1153	586.0824	0.256892	393.4971	496.0706
YBR125C	777.303	757.4518	754.8754	727.9854	781.2346	700.5593	-0.05121	646.4596	624.2588
YAR020C	67.94204	66.25231	43.4745	62.68017	76.04939	33.26678	-0.04681	39.34971	31.60804
YMR030W	214.1901	155.2479	203.5397	177.2953	180.9054	184.9242	-0.0772	175.3873	251.9863
YBL105C	1313.93	1259.783	1566.07	1447.017	1268.642	2129.074	0.226862	1568.367	1748.978
YCL048W	32.24368	31.64289	21.73725	17.01319	13.82716	11.74122	-1.00778	17.98844	17.56002
YEL026W	7660.177	7254.133	6671.36	8661.504	8458.766	7772.685	0.205664	8724.393	7441.059
YEL063C	1771.099	2386.072	1995.875	1842.797	1917.366	1787.6	-0.14939	1699.908	1690.152
YDR363W	375.4085	487.4983	500.9448	583.821	508.1482	649.6807	0.352768	571.133	626.0148
YOR152C	228.0089	322.362	226.265	199.6811	172.8395	151.6574	-0.56718	190.0029	153.6502
YNL121C	4241.195	4591.186	4514.432	4649.973	4866.009	4970.448	0.118207	3984.439	4334.691
YBR072C-/	0	0	1.976114	3.581724	1.152263	5.870608	2.423952	1.124277	0.878001
YPL219W	248.7369	219.5226	260.847	192.5177	225.8436	183.9457	-0.27563	267.578	254.6203
YOR186W	3.45468	6.921883	1.976114	3.581724	4.609054	6.849043	0.283964	4.49711	10.53601
YHR089C	8073.587	7893.913	7989.427	8678.518	8292.84	7528.077	0.032306	7999.234	7520.079
YLR287C-/	23115.26	18665.35	20686.95	21258.43	22813.66	21690.92	0.074169	24428.3	20746.29
YPR044C	0	3.955362	0	1.790862	4.609054	0.978435	0.899489	0	0.878001
YDL216C	127.8232	192.8239	186.7427	112.8243	179.7531	171.2261	-0.12958	232.7254	182.6242
YBR102C	725.4828	683.2887	790.4455	743.2078	754.7326	1016.594	0.193301	778	894.6831
YIR040C	0	0	0	1.790862	2.304527	0	#DIV/0!	0	0
YOR343C	24.18276	50.43086	59.28341	17.01319	29.95885	20.54713	-0.98775	20.23699	16.68202
YLR457C	163.5215	106.7948	158.0891	122.6741	109.465	129.1534	-0.24581	146.1561	164.1862
YLR466C-I	2.30312	0.98884	0.988057	0	1.152263	0	-1.89315	0	0
YBR019C	120.9138	170.0806	142.2802	121.7786	84.11523	95.8866	-0.52178	71.95376	79.8981
YHR095W	5.7578	11.86609	9.880568	6.268017	9.218108	9.784347	-0.12221	6.745665	9.658012
YML093W	3073.513	2877.526	3340.62	3954.223	3790.947	4539.937	0.402902	2962.471	3492.688
YGR227W	732.3921	816.7822	731.1621	756.6392	819.2593	688.818	-0.00992	653.2052	653.2328
YER074W-/	798.031	620.003	609.6311	670.6778	770.8643	688.818	0.071278	744.2717	630.4048
YKL153W	27.63744	26.69869	34.58199	26.86293	38.02469	30.33148	0.098774	55.0896	37.75405
YCR006C	74.8514	86.02912	64.22369	62.68017	88.72429	86.10225	0.077375	58.46243	51.80206
YOR253W	1226.411	1338.89	1247.916	1370.905	1273.251	1012.68	-0.06041	1205.225	1038.675
YBR226C	8.06092	3.955362	2.96417	4.477155	1.152263	5.870608	-0.38144	12.36705	10.53601
YPR049C	451.4115	374.7705	399.175	438.7612	470.1235	498.0233	0.199326	284.4422	335.3964

YPR148C	3945.244	3688.375	3833.66	4016.008	3686.091	3665.216	-0.01263	3577.451	3102.856
YGL262W	0	13.84377	8.892511	12.53603	2.304527	15.65496	0.423601	11.24277	12.29202
YER093C-/	373.1054	413.3353	371.5094	327.7278	305.3498	305.2716	-0.30338	327.1647	329.2504
YDL165W	1081.315	1024.439	1074.018	1254.499	1121.152	1090.955	0.124601	1163.627	928.0471
YER039C	76.00296	88.0068	63.23564	35.81724	39.17696	41.09426	-0.96903	40.47399	35.12004
YHR158C	609.1752	585.3935	643.225	646.5012	595.7202	812.1008	0.160688	546.3989	780.543
YOL087C	724.3312	748.5522	901.1078	711.8677	755.8848	895.2678	-0.00668	818.474	928.0471
YLR361C-/	261.4041	238.3105	299.3812	236.3938	285.7613	227.9753	-0.09123	198.9971	251.9863
YDR147W	738.1499	769.3179	845.7766	617.8474	685.5968	678.0553	-0.24806	596.9913	602.3087
YJL002C	4360.957	4511.09	4554.942	4761.007	5116.05	4967.513	0.144799	4844.512	5083.626
YML107C	550.4457	575.5051	424.8644	522.9317	465.5144	430.5113	-0.1282	440.7168	363.4924
YJL087C	655.2376	675.378	731.1621	780.8159	799.6708	997.025	0.32209	610.4827	698.8889
YBL031W	552.7488	445.967	472.2912	512.1865	512.7572	549.8803	0.098387	463.2023	557.5307
YNL261W	528.566	506.2863	449.5659	588.2982	560	521.5057	0.169781	616.1041	560.1647
YDL137W	7785.697	6162.454	7008.287	6825.871	6918.19	6312.861	-0.06329	6841.228	5854.511
YOL089C	366.1961	295.6633	431.7808	359.9633	425.1852	533.2469	0.269645	431.7226	419.6845
YOL125W	914.3386	1084.758	977.1882	993.033	1129.218	907.009	0.025453	1199.604	1056.235
YMR204C	163.5215	155.2479	162.0413	138.7918	111.7696	106.6494	-0.42869	175.3873	157.1622
YBR237W	476.7458	531.9962	586.9058	509.5003	512.7572	561.6215	-0.01068	622.8497	604.0647
YJL125C	1486.664	1832.321	1675.744	1745.195	1768.724	1711.282	0.06508	1690.913	1428.508
YPR164W	506.6864	427.1791	548.3715	485.3236	573.8272	707.4083	0.253166	510.422	623.3808
YNL233W	710.5125	540.8957	683.7353	732.4626	722.4692	970.6072	0.325865	769.0058	806.883
YOL111C	2449.368	2413.759	2177.677	2515.266	2152.428	1793.471	-0.12395	2106.896	1884.19
YOR138C	637.9642	822.7152	749.9351	704.7042	616.461	663.3787	-0.15564	731.9046	705.9129
YLR322W	0	0.98884	0.988057	1.790862	4.609054	1.956869	2.07971	0	0
YDR132C	295.9509	344.1165	264.7992	272.211	324.9383	334.6247	0.042275	238.3468	245.8403
YOL061W	3953.305	4527.9	4321.761	4514.763	4235.721	4248.364	0.021906	3865.266	3937.835
YFR041C	510.1411	486.5095	612.5952	542.6312	556.5433	602.7158	0.080753	632.9682	629.5268
YCR060W	644.8736	561.6614	624.4519	667.9915	778.9301	608.5864	0.166874	934.2746	784.933
YFR009W	1989.896	2045.911	2191.51	2181.27	2210.041	2666.235	0.180556	2264.295	2349.531
YJR016C	5907.502	6472.949	6345.301	6860.793	7012.675	7152.358	0.167139	6577.023	6347.07
YIR007W	336.2555	333.2392	428.8167	342.9501	435.5556	384.5248	0.082602	309.1763	337.1524
YIL011W	7105.125	5567.172	6540.936	6713.942	6785.68	5768.851	0.004142	7052.593	5969.529
YLR419W	746.2108	843.4809	875.4183	978.7061	982.8807	1290.555	0.399738	935.3989	1173.009
YCL017C	1973.774	2499.789	2166.809	2207.237	2030.288	1937.301	-0.10487	2484.653	2250.317
YBR245C	1480.906	1360.644	1505.799	1636.848	1523.292	1885.444	0.214885	1579.61	1720.004
YAL034C	306.3149	364.8821	355.7005	360.8587	314.5679	292.552	-0.08525	410.3613	383.6865
YLR273C	64.48736	118.6609	85.96094	92.2294	64.52675	75.33947	-0.21347	87.69364	105.3601
YDR479C	533.1723	593.3043	566.1566	575.7621	557.6955	596.8452	0.031755	664.448	616.3568
YCL029C	877.4887	719.8758	875.4183	878.4178	893.0042	973.5425	0.150652	921.9075	986.8732
YNL255C	10064.63	9125.02	9714.575	10643.99	10159.51	9741.296	0.079646	10311.87	9481.534
YDR194C	2996.359	3036.729	3233.91	3509.194	3257.449	3663.26	0.170552	2809.569	3355.72
YOR318C	20.72808	11.86609	19.76114	19.69948	28.80659	30.33148	0.590547	20.23699	19.31602
YBR221C	5588.52	5796.583	5449.133	5332.292	5286.585	4475.36	-0.1574	6055.358	6045.037
YEL059C-/	350.0742	270.9423	261.8351	308.0283	285.7613	272.0049	-0.02815	310.3006	230.9143
YPL155C	756.5749	750.5299	755.8635	796.0382	850.3704	1067.472	0.262141	553.1445	722.5949
YMR198W	352.3773	322.362	395.2227	325.9369	367.572	458.8859	0.107075	468.8237	395.1005
YJL190C	27803.26	21301.6	24703.4	26166.29	25839.51	24848.33	0.05834	35666.58	29073.25
YML009C-/	11.5156	9.888404	13.8328	14.3269	13.82716	14.67652	0.281558	6.745665	7.90201
YNL214W	299.4056	252.1543	252.9425	298.1785	318.0247	260.2636	0.123603	392.3728	339.7864
YPL032C	2716.53	2329.708	2974.051	3008.648	3103.046	4053.655	0.341934	2313.763	2791.165
YGL015C	20.72808	17.79913	7.904455	10.74517	4.609054	14.67652	-0.62867	24.7341	27.21803
YDR199W	3.45468	1.977681	0.988057	5.372586	3.45679	3.913739	0.988979	0	3.512004
YJR011C	199.2199	206.6677	188.7189	173.7136	163.6214	142.8515	-0.30834	129.2919	151.8942

YER056C	7781.091	11066.11	9084.194	9641.106	9213.499	8524.123	-0.02883	9311.266	9450.804
YLR245C	154.309	194.8016	168.9577	140.5827	130.2058	183.9457	-0.18812	138.2861	146.6262
YGR131W	38.00148	51.4197	61.25952	38.50353	48.39507	31.30991	-0.35016	26.98266	22.82803
YKL021C	1883.952	2137.873	1945.484	2409.605	2575.309	2133.966	0.25457	2043.936	2150.225
YLR313C	244.1307	234.3552	201.5636	264.1522	293.8272	359.0855	0.431384	318.1705	222.1343
YCR066W	287.89	195.7904	250.9664	243.5572	246.5844	256.3499	0.023075	272.0751	286.2284
YJR013W	628.7517	650.657	634.3325	604.4159	534.6502	589.0177	-0.14722	558.7659	486.4126
YOL107W	231.4635	287.7526	252.9425	276.6882	276.5432	227.9753	0.016807	222.6069	196.6722
YNL129W	513.5957	443.9894	539.479	448.6109	483.9507	475.5193	-0.08841	492.4335	457.4386
YGL006W-/	5.7578	8.899564	9.880568	6.268017	14.97943	12.71965	0.469124	2.248555	7.90201
YDL199C	104.792	100.8617	106.7101	83.27509	86.41976	74.36104	-0.35602	124.7948	115.8961
YER147C-/	110.5498	116.6832	162.0413	152.2233	163.6214	173.1829	0.329129	158.5231	172.9662
YJL059W	330.4977	304.5629	262.8231	368.0222	277.6955	203.5144	-0.08037	247.341	246.7183
YLR363C	559.6581	617.0364	531.5746	551.5855	536.9548	522.4841	-0.08456	463.2023	497.8266
YGL246C	996.0993	1111.457	980.1524	964.3792	1136.132	1166.294	0.081344	1129.899	848.149
YOR364W	2.30312	1.977681	0.988057	0	0	0.978435	-2.42894	4.49711	5.268006
YPR053C	34.5468	54.38622	37.54616	38.50353	24.19753	29.35304	-0.45835	19.11272	23.70603
YGL075C	208.4323	188.8685	203.5397	200.5765	188.9712	198.6222	-0.03075	201.2457	202.8182
YNL138W	4183.617	4145.219	4231.847	3952.433	4211.523	3928.415	-0.05482	4664.627	4515.56
YBR089W	0	0.98884	0	2.686293	0	0	1.441807	0	0
YGL007W	34.5468	47.46434	40.51033	29.54922	35.72017	32.28835	-0.32871	31.47977	39.51005
YNL249C	246.4338	266.9869	268.7515	226.544	232.7572	244.6087	-0.1521	203.4942	186.1362
YER148W	1768.796	1526.77	1417.862	1649.384	1599.342	1472.544	0.002398	1567.243	1300.32
YKL225W	0	0	0	0	0	0	#DIV/0!	0	0
YLL006W-/	0	0	0	0	0	0	#DIV/0!	0	0
YCL031C	2505.794	2463.202	2310.077	2349.611	2364.445	2282.688	-0.05707	2321.633	2157.249
YIL055C	460.624	445.967	396.2108	292.8059	303.0453	304.2932	-0.53339	319.2948	367.0044
YIR023C-/	0	0	0	0	0	0	#DIV/0!	3.372832	0
YDR352W	1005.312	1216.274	1033.507	1107.648	1085.432	993.1112	-0.03087	965.7544	961.4112
YMR183C	4509.509	4309.367	4402.781	4566.698	4445.432	4548.743	0.036547	4117.104	3900.081
YJL198W	2829.383	3002.12	3161.782	3164.453	3539.753	4338.38	0.296158	2865.783	2943.938
YBR111W-/	558.5066	556.7172	506.8732	532.7815	492.0165	444.2094	-0.14302	703.7977	612.8447
YGL098W	567.719	617.0364	643.225	641.1286	623.3745	581.1902	0.013912	574.5058	565.4327
YGL186C	1036.404	1084.758	1114.528	1130.929	1072.757	1020.507	-0.00513	992.737	863.0751
YGL114W	781.9092	942.3649	939.642	937.5163	850.3704	985.2838	0.057988	898.2977	1053.601
YOL086W-/	472.1396	354.9937	424.8644	457.5653	495.4733	404.0935	0.116329	519.4162	438.1225
YBL108C-/	3.45468	5.933043	7.904455	15.22233	3.45679	3.913739	0.385747	2.248555	2.634003
YDL036C	1039.859	1041.249	1071.054	1093.321	1033.58	1076.278	0.023163	942.1445	919.2671
YML031C-/	0	0	0	0	0	0	#DIV/0!	0	0
YPL244C	1032.949	1137.166	1053.269	1169.433	1173.004	1064.537	0.079915	962.3815	974.5812
YJR097W	423.7741	426.1902	377.4377	498.7551	470.1235	330.7109	0.082448	426.1012	329.2504
YNL127W	572.3253	404.4357	532.5626	453.9835	538.107	686.8612	0.153659	589.1214	679.5728
YER009W	6530.496	5894.478	5843.368	5919.695	6383.54	6154.354	0.014868	8632.202	6602.568
YKL011C	328.1946	336.2057	331.9871	316.0872	255.8025	329.7325	-0.14418	279.9451	246.7183
YDR483W	6430.311	6586.666	6764.237	7449.986	7451.688	7385.225	0.172065	6943.538	6059.963
YIL002C	865.9731	778.2174	820.0872	763.8027	810.0412	936.362	0.026641	661.0752	698.0109
YMR117C	200.3714	189.8574	232.1934	194.3085	247.7366	274.9402	0.20405	147.2803	157.1622
YDR290W	4.60624	0	0.988057	0	0	0	#NUM!	0	0
YGR264C	4500.296	4637.662	4798.992	5217.677	5356.873	6264.917	0.272932	5520.202	5676.277
YER061C	726.6343	890.9452	677.807	583.821	626.8313	627.1767	-0.32073	746.5202	683.9628
YCL012C	515.8989	375.7594	359.6527	474.5784	472.428	384.5248	0.089646	503.6763	339.7864
YPL145C	3635.475	3884.165	3839.589	3964.073	4004.116	4396.107	0.122315	4274.503	4336.447
YFL046W	724.3312	696.1437	723.2576	780.8159	630.2881	650.6591	-0.05625	793.7399	734.0089
YLR007W	1090.527	1067.948	1024.615	1171.224	1024.362	917.7718	-0.03196	997.2341	893.8051

YDR500C	13170.39	10791.22	11387.35	12253.08	12523.95	12413.4	0.073264	14956.26	11875.84
YDR051C	1207.986	1298.347	1185.668	1280.466	1140.741	1124.221	-0.05844	1177.119	1069.405
YOR203W	3.45468	5.933043	3.952227	8.058879	5.761317	3.913739	0.410759	0	4.390005
YDL057W	232.6151	174.0359	260.847	175.5045	170.535	162.4202	-0.39263	195.6243	242.3283
YOR122C	10671.51	8520.838	9855.867	9865.859	10012.02	9288.281	0.005846	10048.79	9288.373
YNR065C	383.4695	434.101	439.6853	436.0749	485.1029	595.8667	0.270985	381.1301	410.9045
YNL004W	2183.358	2385.083	2239.925	2447.213	2392.099	2352.157	0.078978	2401.457	2474.207
YIL142W	5005.831	5099.45	5133.943	6109.526	5851.194	5754.175	0.217173	6152.046	6088.937
YPL226W	6235.697	6258.371	7032	7556.542	7956.379	9629.754	0.364737	7586.624	8313.792
YLR239C	751.9686	545.8399	595.7983	569.4941	492.0165	475.5193	-0.30099	574.5058	507.4846
YIL072W	78.30608	49.44202	68.17592	44.77155	61.06996	40.11582	-0.42475	85.44509	51.80206
YCR088W	5406.574	5078.684	5620.067	5489.888	5045.762	5250.281	-0.0289	4614.035	4890.466
YDL133C-/	5338.632	3549.937	4320.772	4831.746	4725.432	5056.551	0.145766	5955.298	5021.288
YMR211W	565.4159	600.2261	527.6223	551.5855	500.0823	620.3276	-0.01824	631.8439	577.7247
YDR522C	36.84992	55.37506	29.6417	26.86293	27.65432	29.35304	-0.53907	25.85838	11.41401
YER156C	4734.063	4958.046	4406.733	5298.265	4955.885	4351.099	0.05091	4676.994	4140.653
YJL132W	57.578	80.09608	65.21175	71.63448	71.44034	72.40417	0.086879	76.45087	83.4101
YGR121C	784.2123	1267.693	869.49	753.9529	679.8354	514.6567	-0.58433	688.0578	652.3548
YCL039W	967.3103	849.4139	983.1165	832.7509	857.284	861.0225	-0.13425	794.8642	943.8512
YGR284C	2246.693	2157.65	2277.471	2391.696	2363.292	1988.179	0.013187	2655.543	2498.791
YDR201W	529.7176	415.313	476.2434	530.9906	485.1029	489.2174	0.082877	468.8237	395.1005
YER162C	297.1025	256.1097	222.3128	248.9298	202.7984	260.2636	-0.12331	251.8382	318.7144
YLR122C	14.97028	4.944202	13.8328	18.80405	8.065844	9.784347	0.119209	13.49133	15.80402
YJL027C	27.63744	21.75449	22.72531	10.74517	16.13169	13.69809	-0.82975	5.621387	18.43802
YKL183W	1180.349	1211.33	1030.543	1159.583	1069.3	971.5857	-0.09665	1327.772	1228.324
YDR367W	1163.076	1086.736	1123.421	1145.256	1167.243	1079.213	0.007883	1206.35	1042.187
YDR337W	1081.315	1191.553	1049.316	1105.857	1030.124	812.1008	-0.17236	1007.353	972.8252
YAL018C	8.06092	1.977681	0	3.581724	0	0	-1.48683	4.49711	3.512004
YBL053W	9.21248	0.98884	1.976114	1.790862	5.761317	10.76278	0.588813	3.372832	0.878001
YDL221W	31.09212	29.66521	20.74919	17.90862	17.28395	12.71965	-0.76652	43.84682	21.95003
YER166W	2083.172	1818.478	2001.803	1892.941	1957.696	2282.688	0.05511	1625.705	1714.736
YMR037C	1616.79	1523.803	1903.986	1605.508	1735.309	2046.885	0.094936	1262.564	1634.838
YLR033W	1122.771	1028.394	1125.397	1176.596	1231.77	1332.628	0.191239	1002.856	982.4832
YGL083W	996.0993	872.1573	863.5617	806.7834	911.4404	1006.809	-0.00359	763.3844	803.371
YFR031C-/	42812.7	35644.73	41488.51	43951.34	41648.56	38899.63	0.053756	43528.65	39159.73
YKL076C	49.51708	27.68753	29.6417	55.51672	44.93828	47.9433	0.473937	42.72254	31.60804
YKL181W	6485.586	7145.361	6657.527	6923.473	6617.449	6438.1	-0.02217	7986.867	7242.631
YLR426W	434.1381	568.5832	554.2999	448.6109	425.1852	416.8132	-0.27074	614.9798	556.6527
YGR006W	99.03415	150.3037	121.531	94.91569	111.7696	176.1182	0.045695	146.1561	181.7462
YMR253C	361.5898	403.4469	436.7211	322.3552	275.391	319.9482	-0.38906	320.4191	298.5204
YER114C	1190.713	1258.794	1290.402	1429.108	1506.008	1700.52	0.309764	1131.023	1330.172
YGL181W	1176.894	1175.731	1101.683	1153.315	1025.514	789.5968	-0.2187	1070.312	1315.246
YDL130W	31054.12	22654.33	26042.21	29824.12	27824.86	25083.15	0.052951	32228.54	25441.84
YMR109W	1682.429	1685.973	1777.514	1763.999	1671.934	2058.627	0.094576	1369.37	1589.182
YFL050C	545.8394	556.7172	530.5865	651.8738	632.5926	758.2869	0.322864	374.3844	496.9486
YML094C-/	0	0	0	0	0	0	#DIV/0!	2.248555	0
YLR089C	2962.964	3161.323	2954.29	2658.535	2696.297	2897.145	-0.13773	2968.093	3224.02
YDL105W	203.8261	273.9088	257.8828	179.0862	218.9301	240.6949	-0.20379	245.0925	239.6943
YPR076W	0	0	0	0	0	0	#DIV/0!	1.124277	0.878001
YGR170W	800.3342	795.0277	871.4661	795.1428	739.7531	868.85	-0.03737	765.633	984.2392
YLR379W	199.2199	253.1432	283.5723	264.1522	280	295.4873	0.190191	239.4711	366.1264
YDR392W	813.0013	825.6818	811.1947	879.3133	815.8025	777.8556	0.013536	721.7861	701.5229
YDL083C	29657.27	24417.44	26965.06	29973.66	28406.75	27417.7	0.082316	31853.03	26032.73
YNL254C	276.3744	260.065	275.6679	269.5247	330.6996	415.8348	0.323242	321.5434	367.0044

YKL162C	128.9747	104.8171	102.7579	90.43853	94.4856	91.97286	-0.28147	68.58093	92.19011
YJR038C	1.15156	0.98884	3.952227	0	1.152263	1.956869	-0.97055	0	8.780011
YML120C	1336.961	1561.379	1672.78	1259.871	1230.617	1338.499	-0.25558	1195.107	1393.388
YGL085W	403.046	494.4202	421.9003	419.0617	420.5762	307.2285	-0.20215	391.2486	295.0084
YLR163C	984.5837	1181.664	985.0927	977.8107	970.2058	931.4698	-0.13015	1180.491	1191.447
YGL093W	582.6893	585.3935	671.8786	693.0636	733.9918	885.4834	0.329802	676.815	705.0349
YML065W	717.4218	631.869	827.0036	816.6331	786.9959	836.5617	0.16512	631.8439	658.5008
YER024W	344.3164	426.1902	388.3063	393.0942	376.7902	362.9993	-0.03265	267.578	310.8124
YPL048W	7099.367	7625.937	6816.604	7250.305	6839.836	5930.293	-0.10567	7024.486	6207.468
YER151C	2031.352	1979.659	2381.217	2434.677	2532.675	2926.498	0.30441	2240.685	2933.402
YJL076W	1848.254	1586.1	2081.836	1990.543	1972.675	2799.302	0.293889	1701.032	2581.323
YDR226W	12588.85	10713.1	12074.05	12668.56	12239.34	11654.14	0.047575	14837.09	12766.14
YPR089W	1315.081	1004.662	1273.605	1164.06	1288.231	1585.064	0.168082	1405.347	1446.946
YDR382W	43773.1	32705.9	38706.14	39413.29	39564.12	36655.1	0.005593	46031.29	36528.36
YBR126C	7774.181	7270.944	6988.526	5142.46	5050.371	4727.797	-0.5624	7596.743	7073.177
YPL143W	131.2778	207.6565	172.9099	174.6091	164.7737	121.3259	-0.15185	134.9133	117.6521
YLR032W	436.4412	413.3353	508.8493	412.7937	479.3416	691.7533	0.221323	472.1965	561.9207
YGR103W	5700.222	5813.393	5955.018	6582.313	6613.992	6779.574	0.193492	7236.974	7303.213
YBR216C	473.2911	504.3086	427.8286	536.3632	456.2963	609.5648	0.189066	603.737	584.7487
YDR143C	950.0369	961.1529	978.1763	1035.118	1090.041	1054.753	0.138234	681.3122	911.3651
YGL104C	451.4115	453.8778	487.112	401.1531	448.2305	373.7621	-0.18698	380.0058	394.2225
YDR524C	671.3594	711.9651	720.2934	808.5742	799.6708	936.362	0.27457	792.6156	771.7629
YPL232W	4208.952	4153.13	4080.675	4352.69	3864.692	3699.462	-0.0623	3714.613	3607.706
YER087W	179.6434	173.0471	208.48	208.6354	186.6667	206.4497	0.10073	230.4769	205.4523
YDR538W	81.76076	78.11839	62.24758	50.14414	62.22223	55.77078	-0.40174	93.31503	90.43411
YER164W	1422.177	1113.434	1443.551	1561.632	1438.025	1805.212	0.272032	1487.419	1708.59
YOR271C	3237.035	3265.151	3218.101	3416.965	3454.486	3056.63	0.030516	3134.486	2990.472
YOR191W	753.1202	842.492	992.0091	776.3387	859.5885	1392.313	0.226853	826.3439	1118.573
YLR443W	415.7131	566.6056	433.7569	440.5521	439.0124	456.929	-0.08345	418.2312	410.9045
YDR041W	1294.353	1322.08	1438.611	1270.617	1116.543	1173.143	-0.18772	1255.818	1287.15
YGL106W	5420.393	4615.907	5245.594	5233.794	5053.828	4664.198	-0.0315	6295.954	5447.997
YNL219C	1714.673	1995.48	1772.574	1859.81	1981.893	1656.49	0.004064	1686.416	1811.316
YLR147C	609.1752	445.967	520.7059	523.8272	571.5227	498.0233	0.015956	555.3931	449.5365
YPL270W	1167.682	1160.899	992.9971	893.6402	898.7655	752.4163	-0.3843	852.2023	835.857
YLR243W	1263.261	1361.633	1335.853	1279.571	1383.868	1174.122	-0.04558	1241.202	1164.229
YDL159W	332.8008	291.7079	322.1065	273.1065	316.8725	349.3012	-0.01122	312.5491	395.1005
YNL033W	28.789	13.84377	13.8328	9.849741	9.218108	6.849043	-1.12348	2.248555	0.878001
YLR071C	1152.711	976.9743	1373.399	1038.7	1361.975	1753.355	0.245886	1292.919	1618.156
YJR062C	505.5348	557.706	486.124	468.3104	458.6009	516.6135	-0.10208	500.3035	596.1627
YGL121C	909.7324	764.3737	702.5084	745.894	828.4774	763.1791	-0.02391	926.4046	839.369
YDR048C	0	1.977681	0	0	1.152263	0	-0.77934	4.49711	0
YER072W	3179.457	2904.224	2729.013	2368.415	2556.873	2349.222	-0.27673	2838.801	2523.375
YIL110W	1203.38	1225.173	1218.274	1401.35	1336.626	1322.844	0.155129	1437.951	1336.318
YOR091W	4660.363	4713.802	4870.132	5670.765	5266.996	4852.058	0.14861	4855.754	4611.262
YHR182W	460.624	421.246	501.9329	427.1206	518.5186	503.8939	0.06695	365.3902	467.0966
YJL023C	299.4056	259.0762	311.2379	231.9166	290.3704	206.4497	-0.25515	240.5954	228.2803
YNL102W	734.6952	691.1995	838.8602	729.7763	796.2141	1027.356	0.173035	819.5983	871.8551
YIL062C	2106.203	1935.161	2097.645	2012.929	1965.761	1764.118	-0.09625	2289.029	2108.959
YLR396C	635.6611	671.4227	582.9535	513.9774	609.5474	579.2334	-0.15054	573.3815	470.6086
YLR206W	2617.496	2348.496	2803.117	2576.155	2452.017	2578.175	-0.03055	1815.708	2319.679
YDR407C	1050.223	761.4071	888.2631	927.6665	966.749	1078.235	0.138848	824.0954	1083.453
YMR187C	201.523	268.9646	200.5755	230.1258	238.5185	213.2988	0.023203	149.5289	208.0863
YKR051W	513.5957	463.7662	462.4106	431.5978	436.7079	375.7189	-0.21083	364.2659	385.4425
YDR165W	2303.12	2462.213	2159.892	2596.75	2721.646	2487.181	0.172644	2318.26	2205.539

YMR295C	2000.26	2249.612	2217.2	2156.198	2121.317	1974.481	-0.0488	1941.627	1900.872
YCR096C	2.30312	0	0.988057	0	0	0.978435	-1.75006	0	0
YDR363W-/	2413.67	1961.859	2123.334	2097.995	2128.231	1951.977	-0.073	2528.5	2441.721
YJL081C	2040.564	1812.545	1910.902	1975.321	2099.424	2196.586	0.121699	2283.408	2112.471
YBL007C	3116.121	2739.088	3112.379	2922.687	2805.762	3519.43	0.044402	2838.801	3592.78
YGL133W	1193.016	1022.461	1071.054	1144.361	1212.181	1399.162	0.192518	970.2515	1007.067
YPR063C	3856.574	3505.439	3526.375	3462.632	3724.116	3265.037	-0.05904	3623.546	3201.192
YPL015C	595.3565	672.4115	625.44	505.0231	554.2387	585.104	-0.2033	697.052	776.1529
YDL019C	2731.5	2094.364	2764.583	2363.938	2303.375	3039.018	0.021859	2190.093	2331.093
YNL314W	476.7458	465.7438	386.3302	444.1338	426.3375	369.8483	-0.09943	353.0231	357.3464
YPL147W	87.51856	123.6051	124.4952	107.4517	108.3128	107.6278	-0.05354	127.0434	127.3102
YDR107C	333.9524	307.5294	383.366	324.146	232.7572	381.5895	-0.12699	267.578	252.8643
YLR149C-/	1.15156	0	0	0.895431	1.152263	3.913739	2.37207	0	0.878001
YIL042C	460.624	595.2819	440.6733	454.879	488.5597	326.7972	-0.23657	373.2601	386.3205
YDL031W	2984.843	2968.499	3121.272	3468.004	3402.634	4138.779	0.27883	2854.541	3272.31
YJR051W	768.0905	875.1238	925.8092	911.5488	1017.449	966.6935	0.172688	1111.91	1010.579
YLR423C	230.312	188.8685	243.062	180.8771	180.9054	177.0967	-0.2974	219.2341	201.0622
YER113C	914.3386	920.6104	1153.062	1124.661	1285.926	1550.819	0.406827	1249.072	1240.616
YBR217W	278.6775	322.362	343.8438	291.0151	241.9753	253.4146	-0.26486	290.0636	192.2822
YCL063W	1159.621	1080.803	1135.277	1081.681	1091.194	1186.841	-0.00685	947.7659	980.7272
YKR045C	514.7473	511.2305	437.7092	581.1347	520.8231	411.921	0.048643	487.9364	432.8545
YLL045C	22317.23	17404.58	18398.61	19991.39	18745.02	18233.13	-0.02885	27307.58	21956.17
YBR255W	344.3164	430.1456	359.6527	425.3297	394.0741	516.6135	0.236372	404.7399	546.1167
YER046W	487.1099	428.1679	459.4464	368.0222	394.0741	273.9617	-0.40804	504.8006	367.8825
YLR417W	401.8944	377.737	377.4377	368.0222	292.6749	301.3579	-0.26628	357.5202	359.1024
YBL006C	869.4278	747.5634	795.3857	806.7834	783.5392	676.0984	-0.09004	639.7139	654.9888
YGL122C	3331.463	3173.189	3474.996	3405.324	3349.63	3342.333	0.016907	3601.061	3608.584
YCR035C	2283.543	2057.777	2183.606	2278.872	2059.095	2063.519	-0.02755	2041.688	2057.157
YDR485C	538.9301	543.8622	671.8786	543.5266	652.1811	862.001	0.229838	657.7023	768.2509
YMR188C	1084.769	1028.394	1163.931	1206.146	1205.268	1088.019	0.094704	1178.243	1053.601
YOL139C	10854.6	10032.78	10498.1	10941.27	11034.07	11349.84	0.086516	10637.91	10128.62
YBR098W	216.4933	188.8685	210.4561	189.8314	187.8189	259.2852	0.048644	188.8786	246.7183
YCL004W	321.2852	404.4357	417.948	342.9501	361.8107	347.3443	-0.12039	389	400.3685
YER041W	452.5631	395.5362	415.9719	336.6821	351.4404	375.7189	-0.24879	495.8064	407.3925
YPL085W	1881.649	1653.341	2038.361	1844.588	2030.288	2245.508	0.135077	1975.356	2251.195
YOL046C	0	0.98884	0.988057	0.895431	1.152263	0.978435	0.614236	0	7.024009
Q0085	0	0	1.976114	0	0	0	#NUM!	0	0
YBR077C	1297.808	1134.2	1181.716	1147.943	996.7079	973.5425	-0.21278	1109.662	845.515
YPR147C	798.031	842.492	829.9677	818.424	820.4116	819.9283	-0.00686	765.633	667.2808
YDL215C	1036.404	1173.754	1104.648	856.0321	867.6544	978.4347	-0.29483	1218.717	1395.144
YMR065W	287.89	264.0204	309.2618	268.6293	230.4527	219.1694	-0.26181	256.3353	195.7942
YOR061W	2252.451	2460.235	2364.42	2390.801	2469.301	2486.203	0.053859	2543.116	2605.029
YBR104W	1987.592	2031.078	1869.404	2119.485	1980.741	1719.11	-0.01694	2242.934	2116.861
YIL019W	1759.584	1767.058	1730.088	2218.878	2027.984	1922.624	0.230985	1811.211	1878.922
YBR002C	1354.234	1504.026	1250.88	1459.553	1482.963	1274.9	0.037523	1322.15	1252.908
YOR165W	1837.89	1795.734	2133.215	1901.895	1992.264	2316.933	0.107066	1853.934	1835.9
YDR054C	1944.985	2304.987	2461.25	2080.982	2090.206	1977.417	-0.12632	2131.63	2345.141
YHR060W	925.8542	716.9093	962.3673	982.2878	857.284	829.7126	0.035097	802.7341	754.2029
YDR221W	889.0043	909.7332	903.0839	802.3062	903.3746	912.8796	-0.04516	887.0549	1004.433
YHL005C	2.30312	0.98884	2.96417	5.372586	2.304527	3.913739	0.889644	4.49711	0.878001
YJR019C	492.8677	500.3533	400.163	361.7541	338.7655	306.2501	-0.46886	338.4075	359.9804
YLR275W	528.566	611.1034	531.5746	460.2515	585.3498	473.5624	-0.13764	496.9306	390.7105
YOR300W	1.15156	2.966521	5.928341	3.581724	1.152263	2.935304	-0.38952	0	0.878001
YOR215C	1089.376	980.9297	996.9493	955.4249	857.284	874.7206	-0.19072	953.3873	964.9232

YJL071W	430.6834	443.0005	440.6733	452.1927	407.9013	403.1151	-0.05726	409.237	470.6086
YGR219W	1.15156	0	0	0.895431	0	2.935304	1.734032	4.49711	1.756002
YPL149W	388.0757	570.5609	446.6017	425.3297	364.1153	317.9913	-0.34359	342.9046	387.1985
YBR144C	8.06092	12.85493	9.880568	3.581724	8.065844	1.956869	-1.17869	6.745665	4.390005
YJL019W	295.9509	336.2057	433.7569	359.0678	346.8313	536.1822	0.220669	326.0405	476.7546
YOR298W	156.6122	135.4711	140.3041	124.4649	119.8354	129.1534	-0.21139	133.789	98.33612
YOR085W	3351.039	3304.705	3710.153	3713.352	3864.692	3839.378	0.139392	3386.324	3296.894
YML073C	31586.14	23395.96	26292.19	28935.85	28437.86	24253.44	0.00625	31691.13	25886.98
YLR389C	2163.781	2017.234	2202.379	2146.348	2182.387	2315.955	0.057878	2246.306	2472.451
YOR281C	1174.591	1040.26	1148.122	1243.754	1023.21	1112.48	0.007049	1117.532	975.4592
YPL249C	891.3074	840.5144	882.3347	750.3712	736.2964	961.8013	-0.09447	880.3093	899.9511
YBL052C	472.1396	482.5541	572.0849	552.4809	516.214	683.9259	0.199023	422.7283	421.4405
YGL217C	0	1.977681	2.96417	2.686293	2.304527	4.892174	0.999896	0	0.878001
YDR093W	991.4931	1041.249	1125.397	1092.426	1196.049	1447.105	0.242258	1160.254	1306.466
YCR086W	436.4412	381.6924	449.5659	382.349	375.6379	435.4034	-0.08715	421.6041	457.4386
YOR141C	996.0993	1021.472	1115.516	965.2746	1117.696	1438.299	0.16851	1029.838	1294.174
YKR049C	1639.821	1822.433	1662.9	1678.038	1542.881	1224.022	-0.20543	1569.491	1569.866
YJL150W	2.30312	2.966521	1.976114	0.895431	2.304527	0	-1.17908	0	0
YKL172W	5662.22	5433.678	5849.296	6562.614	6293.663	6438.1	0.187304	5694.465	5759.687
YJL003W	367.3476	352.0272	344.8318	308.0283	274.2387	275.9186	-0.31042	335.0347	259.0103
YJR089W	713.9672	517.1635	668.9145	680.5276	695.9671	792.5321	0.191014	695.9278	744.5449
YCL051W	522.8082	560.6725	655.0817	685.9002	660.247	757.3085	0.274867	649.8324	784.055
YJL149W	413.41	298.6298	288.5126	327.7278	300.7408	391.3739	0.027549	376.633	418.8065
YGL195W	3901.485	3463.908	3446.342	3423.233	3638.848	4009.625	0.034279	3606.682	3478.64
YIL134C-/	8.06092	2.966521	0.988057	0.895431	0	0.978435	-2.68081	4.49711	3.512004
YMR194C-/	10.36404	4.944202	6.916398	12.53603	6.913581	5.870608	0.18813	17.98844	14.04802
YOL006C	1421.025	1425.908	1494.93	1531.187	1458.766	1702.476	0.112021	1412.093	1730.54
YJR018W	0	0	0	0	1.152263	1.956869	#DIV/0!	0	0
YBR179C	960.401	909.7332	994.9732	820.2148	912.5927	1288.599	0.076631	632.9682	880.6351
YOR039W	1744.613	1658.285	1837.786	1932.34	1903.539	1708.347	0.081231	2157.488	2121.251
YPL158C	731.2406	765.3625	768.7082	781.7113	822.7161	942.2326	0.168897	580.1272	693.6208
YLR268W	2072.808	1788.812	2051.206	2063.969	2045.268	2087.001	0.067545	2382.344	2055.401
YDR408C	2486.218	2681.735	2269.567	2543.024	2472.757	2041.993	-0.07561	2467.789	2065.059
YGR072W	793.4248	795.0277	797.3619	763.8027	842.3046	808.1871	0.01712	858.948	894.6831
YIR013C	9.21248	2.966521	5.928341	3.581724	2.304527	1.956869	-1.20708	11.24277	10.53601
YDR238C	4068.461	3901.964	4486.766	4554.162	4621.729	5660.245	0.252137	4933.33	5009.874
YDL184C	8878.527	6325.612	7693.01	7432.078	7497.778	7622.985	-0.02186	11897.1	10470.16
YBR218C	4835.4	5099.45	4815.789	4902.485	4847.572	5171.027	0.016575	4084.5	4374.201
YBR048W	30005.05	24047.61	27147.85	28931.38	29016.3	26825.74	0.062123	36300.67	32104.11
YOL116W	739.3015	760.4183	863.5617	867.6727	820.4116	918.7502	0.141508	701.5491	698.0109
YFL002C	933.9151	1063.003	1089.827	1190.028	1162.634	1133.027	0.175357	987.1156	986.8732
YAL069W	2.30312	0.98884	0.988057	0	0	0	#NUM!	0	0
YBL009W	1165.379	1083.769	1185.668	1156.001	1269.794	1461.781	0.178639	1274.931	1280.126
YOR372C	751.9686	615.0587	775.6246	839.0189	775.4733	935.3836	0.251029	680.1879	879.7571
YGL100W	1889.71	1877.808	1963.269	1804.294	1837.86	1857.069	-0.05951	1653.812	1744.588
YJR034W	503.2317	490.4649	444.6256	444.1338	532.3457	411.921	-0.05096	690.3064	579.4807
YML100W-/	0	3.955362	0	4.477155	3.45679	1.956869	1.32228	0	2.634003
YDR236C	644.8736	663.5119	678.795	672.4687	724.7737	544.0097	-0.03374	622.8497	537.3367
YBR001C	200.3714	239.2994	191.683	182.6679	133.6626	171.2261	-0.37288	234.974	198.4282
YDR076W	170.4309	205.6788	211.4442	161.1776	193.5803	186.881	-0.11739	183.2572	228.2803
YMR125W	2047.474	1865.942	1982.042	1917.118	2181.235	2608.507	0.186034	2293.526	2330.215
YFR053C	5407.725	4785.988	6025.171	3333.69	3242.469	3909.825	-0.62921	6103.702	5885.241
YGL050W	933.9151	951.2645	906.0481	1005.569	917.2017	868.85	0.000203	929.7775	747.1789
YJR133W	846.3966	735.6973	781.5529	762.9072	715.5556	662.4003	-0.14282	920.7833	828.833

YNL257C	753.1202	674.3892	719.3054	687.691	843.4569	968.6504	0.219614	820.7226	814.785
YDR155C	14009.88	11715.78	13758.69	13532.65	13214.16	12833.15	0.003489	16002.97	14232.4
YAR002C-/	4093.796	3485.663	3905.789	4401.939	4368.231	3891.235	0.140656	5303.217	4491.853
YLR001C	827.9716	849.4139	855.6572	760.2209	845.7614	930.4914	0.001953	674.5665	771.7629
YMR036C	322.4368	356.9714	408.0675	382.349	320.3292	315.056	-0.09562	357.5202	394.2225
YJL055W	2328.454	2167.538	2115.43	2221.564	2151.276	1947.085	-0.06505	2590.335	2425.039
YOL069W	449.1084	335.2169	373.4855	391.3034	347.9836	347.3443	-0.09154	341.7804	341.5424
YMR290C	3668.87	3923.719	3683.476	4466.41	4526.091	4099.641	0.215438	3751.714	3798.233
YER190W	0	1.977681	0.988057	0.895431	4.609054	5.870608	1.939415	0	0
YOR041C	0	1.977681	0	0	0	0	#NUM!	2.248555	1.756002
YNL008C	488.2614	529.0296	455.4942	518.4546	565.7614	472.584	0.080036	614.9798	514.5086
YDR254W	279.8291	243.2547	248.0023	258.7796	223.5391	321.905	0.060705	246.2168	280.0823
YKL188C	209.5839	264.0204	195.6353	189.8314	217.7778	193.7301	-0.15434	184.3815	157.1622
YER181C	1.15156	0	6.916398	0	0	2.935304	-1.45869	1.124277	0
YPL167C	294.7993	265.9981	326.0588	223.8578	285.7613	349.3012	-0.04618	285.5665	360.8584
YGR258C	469.8365	468.7104	426.8405	428.9115	380.2469	493.1311	-0.06826	482.315	528.5566
YAL026C	673.6626	724.82	841.8244	742.3123	899.9178	1181.949	0.334135	782.4971	1105.403
YOR058C	800.3342	817.771	849.7289	797.829	875.7202	1037.141	0.135415	776.8757	1014.969
YMR207C	614.933	576.494	695.592	599.0434	638.354	687.8396	0.028927	520.5405	595.2847
YGL178W	605.7205	655.6012	677.807	690.3773	778.9301	961.8013	0.326206	671.1937	795.469
YPL051W	320.1337	365.871	308.2737	299.9694	253.498	243.6302	-0.31889	358.6445	303.7884
YGL131C	166.9762	183.9243	283.5723	218.4852	177.4486	343.4306	0.220727	221.4827	279.2043
YBR126W-I	23.0312	17.79913	40.51033	32.23552	35.72017	19.56869	0.105708	26.98266	21.07203
YEL067C	43.75928	70.20767	82.00872	66.2619	64.52675	77.29634	0.086498	97.81214	112.3841
YNL290W	1695.096	1530.725	1483.073	1689.678	1586.667	1582.129	0.045115	1838.194	1614.644
YDR178W	2075.111	2270.378	2074.919	1863.392	1783.704	1692.692	-0.26588	2276.662	1978.136
YCL028W	3486.923	3554.881	3576.766	3545.011	3294.321	3137.84	-0.08989	3495.379	3504.102
YCR004C	6197.696	5592.881	5512.369	4868.458	4834.898	4352.078	-0.29989	5327.951	4566.484
YBL072C	29268.05	23343.56	28033.15	29747.11	28426.34	26946.09	0.07791	34205.02	31001.34
YIL093C	2189.115	2130.951	1990.935	1943.085	1716.873	1692.692	-0.23762	2012.457	1857.85
YKL065C	3103.454	2414.748	2726.049	2363.938	2561.482	2199.521	-0.21051	3128.864	2867.552
YNL042W-I	9.21248	4.944202	3.952227	0	0	5.870608	-1.62512	0	0
YHR139C	41.45616	33.62057	17.78502	41.18983	20.74074	26.41774	-0.07188	13.49133	28.97404
YKL082C	1160.772	1286.481	1499.87	1560.736	1488.724	1896.206	0.325363	1444.697	1735.808
YPR132W	29053.86	21530.02	25159.88	26776.07	25765.76	25119.35	0.036067	29550.51	24221.42
YNL011C	321.2852	332.2504	295.429	243.5572	254.6502	231.889	-0.37827	202.3699	259.8883
YJL100W	778.4545	1128.267	805.2663	883.7904	902.2223	728.9339	-0.10882	585.7486	727.8629
YLR151C	119.7622	150.3037	180.8144	124.4649	123.2922	73.3826	-0.48954	148.4046	144.8702
YGR245C	3302.674	3580.591	3192.412	4221.957	4004.116	4071.267	0.28747	3346.974	3771.015
YER099C	1143.499	1467.439	1144.17	1345.833	1360.823	1119.329	0.026977	1222.09	1030.773
YDR295C	549.2941	509.2528	555.2879	599.9388	420.5762	669.2493	0.066329	519.4162	589.1387
YDL012C	1494.725	1244.95	1330.913	1550.887	1397.696	1180.971	0.020749	1680.795	1482.066
YKR030W	593.0534	606.1592	550.3477	573.0759	617.6132	472.584	-0.07297	467.6994	539.9707
YLR323C	496.3223	531.9962	586.9058	534.5723	573.8272	650.6591	0.123069	648.7081	581.2367
YHR069C-/	0	0	0	0	0	0	#DIV/0!	0	0
YDR276C	6014.598	5451.477	5356.256	4826.373	5190.947	4935.225	-0.16999	5802.396	4912.416
YJR123W	83240.51	69106.1	79440.76	82446.81	79765.44	78138.77	0.052341	100339.5	87894.93
YIL151C	561.9613	578.4717	676.8189	669.7824	730.535	810.1439	0.282589	684.685	715.5709
YLL062C	1492.422	1764.091	1402.053	1727.286	1389.63	1270.008	-0.08668	1260.315	1093.989
YBL010C	449.1084	512.2193	449.5659	472.7876	447.0782	503.8939	0.013096	500.3035	582.9927
YLR290C	733.5437	737.675	622.4758	608.8931	592.2634	681.969	-0.15292	661.0752	517.1426
YDL153C	2549.554	2361.351	2639.1	3153.708	3084.609	3437.241	0.357867	3227.801	3029.982
YHR218W	0	0.98884	0	0.895431	0	1.956869	1.528316	2.248555	1.756002
YBR058C	889.0043	815.7934	830.9558	918.7122	1057.778	1158.467	0.306031	979.2457	1060.625

YML025C	1545.393	1693.884	1582.867	1615.358	1454.156	1328.714	-0.13275	1468.306	1485.578
YBR016W	3567.533	3948.44	3505.626	3088.342	2836.873	2495.009	-0.3884	3444.786	3531.32
YOL097C	5489.486	5202.29	5454.074	6055.8	6057.449	5575.121	0.131638	6254.356	5900.167
YHR213W-/	5.7578	1.977681	7.904455	14.3269	10.37037	7.827478	1.056303	0	7.024009
YDR159W	628.7517	670.4338	740.0546	737.8352	736.2964	1088.998	0.329875	807.2312	838.491
YML095C	132.4294	205.6788	158.0891	117.3015	155.5556	109.5847	-0.37567	145.0318	194.9162
YNL201C	837.1841	972.0301	971.2599	948.2615	904.5268	1142.812	0.107514	753.2659	981.6052
YGR266W	1248.291	1311.202	1206.417	1112.125	1155.72	1162.38	-0.1347	1099.543	1050.967
YIL106W	933.9151	1147.055	1021.651	1033.327	995.5556	798.4027	-0.13407	817.3497	841.125
YDR179W-/	352.3773	274.8976	317.1662	293.7014	301.893	317.9913	-0.04792	240.5954	280.9603
YHR048W	491.7161	729.7642	566.1566	565.9124	558.8478	521.5057	-0.11886	513.7948	472.3646
YOR328W	261.4041	364.8821	263.8112	319.6689	278.8478	272.9833	-0.03046	209.1156	280.0823
YDL049C	193.4621	265.9981	306.2976	248.0344	274.2387	257.3283	0.025849	202.3699	192.2822
YML115C	2237.481	2053.822	2050.218	2317.375	2389.794	2316.933	0.147485	2291.278	2301.241
YDR014W-/	1.15156	3.955362	2.96417	3.581724	3.45679	3.913739	0.440392	12.36705	4.390005
YGL149W	1.15156	7.910723	4.940284	6.268017	9.218108	2.935304	0.395694	5.621387	27.21803
YPL131W	81793	71127.29	76071.48	83408.5	78491.04	76536.1	0.058304	81181.83	72964.52
YLR441C	41714.11	35971.05	38791.11	40976.71	39363.62	39192.18	0.037367	49141.04	43227.5
YGR107W	16.12184	23.73217	12.84474	9.849741	18.43622	15.65496	-0.2622	3.372832	7.024009
YGR096W	278.6775	271.9311	286.5365	282.9562	240.8231	242.6518	-0.12732	372.1358	287.9844
YJR106W	142.7934	224.4668	250.9664	174.6091	169.3827	218.1909	-0.1371	181.0087	275.6923
YMR024W	1485.512	1429.863	1497.894	1397.768	1464.527	1550.819	-5.1E-05	1464.934	1358.268
YDL117W	796.8795	818.7599	840.8364	928.562	1016.296	1058.666	0.290067	643.0867	765.6169
YKL154W	1408.358	1294.392	1346.721	1347.624	1371.194	1385.464	0.019396	1391.856	1188.813
YOR072W	6.90936	10.87724	15.80891	14.3269	18.43622	17.61182	0.584438	7.869942	10.53601
YBR230W-/	832.5778	501.3421	690.6517	607.1022	677.5309	716.2142	-0.01701	973.6243	835.857
YNL023C	1166.53	1186.609	1198.513	1346.728	1366.584	1589.956	0.276943	1119.78	1149.303
YDL001W	492.8677	541.8846	487.112	564.1215	546.1729	639.8963	0.201672	595.8671	612.8447
YJL172W	1615.639	1442.718	1586.819	1475.67	1396.543	1416.773	-0.1151	1294.043	1346.854
YPL073C	2.30312	1.977681	0	0	0	8.805912	1.040592	1.124277	0.878001
YHL029C	839.4872	1139.144	1200.489	984.9741	1098.107	1164.337	0.030666	1063.566	1193.203
YKL116C	816.456	918.6328	858.6214	838.1234	895.3087	891.354	0.017183	894.9249	911.3651
YNL085W	2205.237	1984.603	2036.385	2093.518	2190.453	2642.752	0.153815	2316.012	2449.623
YHR081W	1399.145	1201.441	1326.96	1549.991	1556.708	1473.523	0.221789	1378.364	1447.824
YLR362W	436.4412	384.6589	490.0762	425.3297	449.3828	493.1311	0.061042	466.5752	472.3646
YBR124W	1.15156	0	0	0	0	0	#NUM!	0	0
YOR196C	1231.018	1414.042	1295.342	1254.499	1177.613	1127.157	-0.14676	1312.032	1332.806
YLR467W	8.06092	15.82145	15.80891	9.849741	8.065844	23.48243	0.06074	2.248555	7.90201
YOR032W-/	20.72808	8.899564	7.904455	12.53603	12.6749	11.74122	-0.02247	10.1185	7.90201
YNL236W	597.6596	619.0141	645.2011	685.0047	848.0659	902.1168	0.387277	672.3179	826.199
YER059W	527.4145	417.2907	480.1956	404.7348	414.8148	416.8132	-0.20476	446.3382	424.0745
YDR206W	1852.86	1916.373	2055.158	1870.555	2153.58	2414.777	0.14471	2126.009	2187.101
YKR090W	655.2376	606.1592	690.6517	668.887	611.8519	883.5265	0.148889	634.0925	891.1711
YOR319W	986.8869	914.6774	797.3619	806.7834	840.0001	760.2438	-0.16513	802.7341	686.5968
YNL274C	1281.686	1296.37	1258.784	984.0787	1068.148	917.7718	-0.36946	1283.925	1168.619
YOR262W	720.8765	808.8715	734.1262	801.4108	786.9959	749.481	0.046412	816.2254	751.5689
YIL115W-/	4.60624	3.955362	0.988057	4.477155	5.761317	5.870608	0.754353	2.248555	3.512004
YNR033W	852.1544	1010.595	924.8212	956.3203	912.5927	907.9874	-0.00553	1025.341	974.5812
YJR092W	1068.648	1178.698	1377.351	1209.727	1366.584	1497.984	0.16869	1168.124	1432.02
YOR309C	270.6166	222.4891	247.0142	248.0344	218.9301	191.7732	-0.16805	248.4653	208.9643
YKL102C	12.66716	0.98884	1.976114	1.790862	2.304527	6.849043	-0.51432	21.36127	1.756002
YNR048W	454.8662	649.6682	469.327	455.7744	433.2511	523.4626	-0.15607	397.9942	455.6826
YLR119W	400.7429	330.2727	339.8915	332.2049	303.0453	283.7461	-0.2207	351.8989	410.0265
YPL199C	1963.41	1984.603	1916.83	1949.353	1820.576	1721.067	-0.09502	2248.555	2074.717

YBR175W	499.777	471.6769	469.327	496.9642	482.7984	422.6838	-0.03891	458.7052	408.2705
YOR361C	7493.201	7307.531	7489.471	8082.16	8411.523	8371.487	0.157717	8685.044	8636.897
YER013W	421.4709	467.7215	561.2163	485.3236	472.428	677.0768	0.17268	546.3989	589.1387
YDL044C	355.832	463.7662	500.9448	484.4282	443.6214	497.0448	0.109926	457.5809	401.2465
YGR225W	24.18276	14.83261	23.71336	5.372586	26.50206	28.37461	-0.05818	15.73988	4.390005
YLR195C	1632.912	1591.044	1699.458	1852.647	1766.42	2045.907	0.20241	1812.335	1860.484
YNL316C	762.3327	759.4295	795.3857	804.9925	766.2552	735.7829	-0.00631	743.1474	810.395
YER046W-/	5.7578	5.933043	6.916398	2.686293	2.304527	0.978435	-1.64024	3.372832	1.756002
YDR063W	1753.826	1348.778	1710.326	1499.847	1492.181	1498.962	-0.09988	1561.621	1482.944
YDR520C	253.3432	264.0204	260.847	269.5247	311.1111	345.3875	0.250888	313.6734	294.1304
YLR223C	1317.385	1422.941	1739.968	1598.344	1683.457	2118.311	0.269396	1132.147	1478.554
YER003C	4810.066	4018.648	4874.084	4769.961	4930.535	5363.779	0.136661	5142.445	4733.304
YBR060C	827.9716	650.657	790.4455	929.4574	932.1811	1146.725	0.406875	870.1908	1057.991
YLR080W	103.6404	145.3595	154.1369	102.0791	133.6626	86.10225	-0.32491	182.133	143.9922
YDL060W	2787.927	3042.662	3225.017	3567.397	3698.766	4359.905	0.36048	2860.162	3253.872
YKL023W	784.2123	785.1393	840.8364	702.9134	727.0782	681.969	-0.19056	835.3382	816.541
YNL200C	2432.095	2400.905	2304.149	2105.158	1951.934	1909.905	-0.25834	2928.743	2685.805
YEL007W	1045.616	1056.082	1101.683	1029.746	1080.823	1043.011	-0.0226	1128.775	1137.889
YPL263C	1581.092	1778.924	1764.669	2115.904	2099.424	2322.804	0.351415	2014.705	2083.497
YGR021W	846.3966	951.2645	717.3293	835.4371	664.856	584.1255	-0.27091	747.6445	775.2749
YJL163C	254.4947	291.7079	304.3215	191.6222	212.0165	207.4282	-0.47702	239.4711	239.6943
YOR083W	118.6107	202.7123	181.8025	205.0537	182.0576	169.2692	0.145154	201.2457	194.9162
YCR106W	434.1381	513.2082	606.6669	539.0495	557.6955	789.5968	0.279592	404.7399	490.8026
YOR347C	616.0846	749.541	654.0936	482.6373	394.0741	468.6702	-0.58614	731.9046	612.8447
YKR038C	2060.141	2149.739	1936.591	2226.937	2008.395	1908.926	-0.00052	2191.217	2087.887
YOR204W	2798.291	3478.741	3448.318	3322.049	3643.457	4401.978	0.225091	2773.593	3356.598
YAL002W	593.0534	446.9559	430.7928	441.4475	425.1852	560.6431	-0.04334	527.2861	529.4346
YIL003W	568.8706	564.6279	586.9058	620.5337	568.0659	486.2821	-0.03869	639.7139	663.7688
YGR132C	2565.676	2616.472	2554.127	2371.997	2386.338	2285.623	-0.13525	2960.223	2857.893
YOR136W	3127.637	2821.162	2696.407	2519.743	2584.527	2368.79	-0.2102	2847.795	2812.237
YDR464W	1022.585	905.7778	1232.107	1167.642	1094.65	1626.158	0.299056	745.396	1057.991
YNL170W	2.30312	1.977681	0	4.477155	3.45679	2.935304	1.3443	0	0
YLL054C	384.621	315.4401	328.0349	309.8191	346.8313	474.5408	0.137868	365.3902	397.7345
YMR307W	32865.52	32378.59	33073.23	35661.44	36600.5	37876.19	0.163796	34348.93	32445.65
YPR024W	1507.392	2149.739	1905.962	1710.273	1971.523	1850.22	-0.00808	1729.139	2130.031
YPR098C	1135.438	1080.803	1031.531	1107.648	1178.766	928.5345	-0.01465	1169.249	978.0932
YBR279W	1924.257	1882.752	2161.868	2017.406	2007.243	2108.527	0.039175	1987.723	2228.367
YNL285W	2.30312	0	0	0.895431	0	0	-1.36294	0	0
YDR434W	1190.713	1300.325	1262.737	1125.557	1338.93	1221.087	-0.02645	1352.506	1292.418
YJR057W	624.1455	526.0631	477.2314	464.7287	538.107	568.4706	-0.05064	516.0434	421.4405
YIL005W	1335.81	1118.379	1335.853	1397.768	1438.025	1746.506	0.273858	1396.353	1610.254
YBL077W	0	0	1.976114	0	1.152263	0	-0.7782	0	0
YDR526C	0	0	1.976114	0	1.152263	0	-0.7782	0	0
YML056C	7561.143	8419.976	7529.981	9017.886	8511.77	8427.258	0.142777	9511.387	8761.573
YJL197W	956.9463	985.8739	998.9255	900.8036	854.9795	1148.682	-0.0184	1101.792	1079.063
YHR054C	0	0	0	0	0	0	#DIV/0!	2.248555	0
YPL152W-/	2.30312	2.966521	5.928341	5.372586	2.304527	1.956869	-0.21703	6.745665	5.268006
YMR235C	4957.466	4689.081	4617.19	4771.752	4788.807	4620.169	-0.00842	4856.879	4478.683
YLR079W	801.4857	857.3247	766.7321	838.1234	827.3252	806.2302	0.027184	812.8526	672.5488
YMR178W	1632.912	1499.082	1515.679	1535.664	1358.519	1331.65	-0.13727	1788.725	1467.14
YOR248W	34.5468	23.73217	38.53422	46.56241	51.85186	49.90017	0.615384	68.58093	49.16806
YOL029C	750.8171	859.3023	658.0458	784.3976	727.0782	653.5944	-0.06711	833.0896	719.0829
YFR034W-/	0	0.98884	0	2.686293	2.304527	0	2.335467	1.124277	0
YDL247W	2.30312	3.955362	3.952227	0	0	1.956869	-2.38346	1.124277	0

YGL006W	1791.827	1676.085	1725.147	1498.056	1488.724	1775.859	-0.12482	1618.96	1518.064
YGR129W	669.0563	670.4338	673.8548	616.0565	555.391	570.4274	-0.20895	558.7659	550.5067
YOL099C	19.57652	5.933043	12.84474	8.058879	6.913581	5.870608	-0.87982	7.869942	2.634003
YLR039C	639.1158	537.9292	565.1685	600.8342	533.498	716.2142	0.08703	609.3584	660.2568
YPL003W	297.1025	328.295	309.2618	297.2831	271.9342	300.3795	-0.10409	301.3064	238.8163
YKL009W	6001.93	5819.326	5780.132	6781.099	6502.223	5928.336	0.126293	6637.734	5638.523
YJL121C	3166.79	2725.244	2810.034	2814.34	3008.56	2373.683	-0.08634	3563.96	2871.942
YGR246C	768.0905	845.4586	688.6756	756.6392	863.0453	742.6319	0.037174	821.8468	817.419
YOR320C	1761.887	1555.446	1691.553	1748.777	1757.202	1836.522	0.093025	1581.858	1604.986
YDR271C	0	0	0	0	0	0	#DIV/0!	0	1.756002
YJL182C	0	0	0	0	0	0	#DIV/0!	0	1.756002
YLR062C	0	0	0	0	0	0	#DIV/0!	0	1.756002
YBR277C	0	0	0	0	0	0	#DIV/0!	0	1.756002
YMR193C-/	0	0.98884	0	4.477155	0	3.913739	3.085015	1.124277	0
YJL039C	1211.441	1080.803	1339.805	1293.898	1207.572	1878.595	0.270169	1424.46	1651.52
YKL164C	10549.44	12421.81	11954.5	13079.56	13002.14	11985.83	0.12427	10252.29	10939.89
YML099W-/	5.7578	0.98884	0	0	1.152263	0	-2.5497	3.372832	2.634003
YKL053W	5.7578	5.933043	2.96417	1.790862	5.761317	1.956869	-0.62402	1.124277	0
YGR274C	760.0296	769.3179	837.8722	717.2403	814.6503	913.858	0.047083	728.5318	932.4371
YGR145W	2292.756	2607.572	2319.957	3060.583	2887.572	3207.309	0.342577	2758.977	2540.935
YMR185W	488.2614	450.9112	520.7059	499.6505	527.7367	703.4946	0.245659	555.3931	649.7208
YOR075W	498.6255	420.2572	441.6614	403.8394	494.321	524.441	0.064348	537.4046	477.6326
YBL008W	456.0177	496.3979	572.0849	481.7419	498.9301	592.9314	0.045735	538.5289	518.8986
YHR075C	204.9777	197.7681	198.5994	151.3278	145.1852	162.4202	-0.38991	181.0087	172.0882
YIR018C-/	88.67012	92.951	88.92511	73.42534	74.89713	75.33947	-0.27456	104.5578	86.04411
YLR208W	4237.741	4181.806	4252.597	4379.553	4270.288	4063.439	0.004676	4211.543	3861.449
YFL005W	3023.996	3032.774	3105.463	3295.186	3131.852	2942.153	0.032225	3729.228	3260.896
YLR076C	11.5156	12.85493	0.988057	11.6406	12.6749	7.827478	0.34203	6.745665	8.780011
YGR118W	24132.09	19116.26	21899.29	24281.4	23616.79	21826.92	0.097965	24667.77	21262.55
YKR074W	1381.872	1077.836	1180.728	1232.113	1201.811	1192.712	-0.00548	1512.153	1358.268
YBL057C	1690.49	1434.807	1484.061	1453.285	1625.844	1472.544	-0.01817	1567.243	1418.85
YKL128C	1083.618	1027.405	1020.663	1134.511	1126.914	1068.451	0.088529	1153.509	1151.937
YFL066C	0	3.955362	3.952227	0	0	7.827478	-0.01469	0	0
YGL185C	766.9389	743.608	699.5442	749.4758	720.1647	744.5888	0.002699	649.8324	599.6747
YMR060C	688.6328	596.2708	573.073	595.4616	591.1112	530.3116	-0.11394	604.8613	515.3866
YPL151C	533.1723	932.4765	719.3054	699.3316	720.1647	677.0768	-0.05957	685.8093	616.3568
YDL003W	1627.154	1444.696	1635.234	1730.868	1775.638	1974.481	0.219602	1399.725	1399.534
YAL031W-/	0	0	0	0	0	1.956869	#DIV/0!	0	0
YBR231C	789.9701	899.8448	701.5203	891.8493	890.6997	636.961	0.016898	672.3179	703.2789
YKL037W	47.21396	50.43086	44.46256	40.2944	27.65432	42.07269	-0.3692	44.9711	28.97404
YBL054W	2166.084	2347.507	2339.719	2664.803	2795.391	3259.166	0.347421	2404.83	2530.399
YBL029C-/	358.1351	375.7594	316.1782	277.5836	282.3045	216.2341	-0.43613	331.6619	323.9824
YKL221W	27.63744	11.86609	38.53422	11.6406	13.82716	12.71965	-1.03107	12.36705	14.04802
YNL090W	192.3105	194.8016	205.5158	183.5634	127.9012	147.7436	-0.36798	118.0491	129.0662
YAL001C	739.3015	615.0587	741.0426	669.7824	654.4856	896.2462	0.083666	688.0578	858.6851
YHR066W	3055.089	3053.539	3028.394	3518.149	3543.21	3393.212	0.194338	3232.298	3247.726
YLR262C-/	3318.796	2554.175	2684.55	2948.654	3018.93	2903.994	0.051998	3465.023	2925.5
YLR368W	444.5021	481.5653	394.2347	522.9317	434.4033	431.4897	0.072996	409.237	384.5645
YIL057C	0	1.977681	7.904455	0	0	0	#NUM!	4.49711	5.268006
YLR088W	799.1826	875.1238	782.541	871.2544	894.1565	815.0361	0.070813	825.2197	777.031
YOR242C	49.51708	60.31927	24.70142	65.36646	42.63375	56.74921	0.292262	44.9711	50.04606
YGR083C	2254.754	2521.543	2667.753	2768.673	2933.663	3288.519	0.27237	2740.988	2934.28
YPL211W	2635.921	2544.286	2456.309	2955.818	2782.716	2553.715	0.118849	3031.052	2427.673
YGR087C	34.5468	22.74333	26.67753	32.23552	19.58848	26.41774	-0.1019	17.98844	14.04802

YGR007W	1971.471	1840.232	1707.362	1830.261	1830.947	1834.565	-0.0061	1759.494	1736.686
YOR008C-/	10.36404	15.82145	15.80891	9.849741	3.45679	7.827478	-0.99063	5.621387	5.268006
YNL032W	766.9389	717.8982	668.9145	672.4687	669.4651	619.3492	-0.13505	740.8989	714.6929
YDR216W	274.0713	186.8908	250.9664	150.4324	176.2963	191.7732	-0.45738	272.0751	318.7144
YGR163W	710.5125	858.3135	600.7386	731.5671	687.9013	525.4194	-0.15772	607.1098	580.3587
YLR202C	9.21248	4.944202	2.96417	2.686293	8.065844	4.892174	-0.13012	3.372832	2.634003
YLR381W	305.1634	349.0607	359.6527	282.9562	392.9218	410.9426	0.100232	281.0694	296.7644
YDR235W	437.5928	413.3353	449.5659	361.7541	448.2305	456.929	-0.03774	534.0318	444.2685
YER145C	2553.008	3936.574	2978.003	3617.541	3371.523	2440.216	-0.00585	3995.682	3810.525
YPL197C	1.15156	0	6.916398	5.372586	4.609054	0.978435	0.441982	3.372832	0
YDL162C	0	7.910723	0.988057	5.372586	0	3.913739	0.0615	0	0.878001
YNR030W	1607.578	1613.788	1474.181	1833.843	1919.671	1481.35	0.156859	1695.41	2041.352
YML001W	2421.731	1854.076	2265.614	2191.12	2432.428	2027.317	0.023938	2670.159	2221.343
YBL068W	1757.28	1865.942	1722.183	1849.065	1882.799	1798.363	0.049039	1868.549	1630.448
YML067C	1239.078	1041.249	1242.975	1359.264	1269.794	1186.841	0.115095	1385.11	1330.172
YDR484W	381.1663	289.7302	347.796	392.1988	312.2634	403.1151	0.120689	395.7457	432.8545
YHR164C	407.6522	343.1276	368.5452	336.6821	286.9136	374.7405	-0.16503	345.1532	381.9305
YHR010W	26925.77	21621	24821.96	26740.26	27310.95	24997.05	0.107568	28873.69	24785.09
YKL049C	851.0028	784.1505	766.7321	799.6199	720.1647	814.0577	-0.04146	667.8208	677.8168
YOR206W	4273.439	4020.625	4341.522	4643.705	4894.815	5403.895	0.241921	4275.627	4324.155
YDL022C-/	1.15156	0	1.976114	0	0	4.892174	0.645386	0	0
YMR021C	681.7235	523.0966	543.4313	510.3957	547.3251	668.2709	-0.01849	612.7312	585.6267
YLR232W	25.33432	5.933043	7.904455	11.6406	10.37037	8.805912	-0.34609	10.1185	3.512004
YAL003W	58727.25	48577.78	55644.4	59479.9	56939.1	51409.9	0.042567	67185.7	60478.47
YDL136W	15282.35	11123.47	13964.21	15062.05	14528.89	13667.75	0.099705	15506.03	13856.61
YLR307W	4.60624	3.955362	3.952227	7.163448	6.913581	5.870608	0.672695	4.49711	5.268006
YFR010W-/	4.60624	10.87724	1.976114	0.895431	4.609054	1.956869	-1.22651	3.372832	0
YPR114W	2784.472	2637.237	2661.825	2939.7	3008.56	2688.739	0.095544	2874.778	2555.861
YOL005C	2877.748	2013.279	2480.023	2417.664	2576.461	2170.168	-0.04105	2687.023	2193.247
YGR184C	1948.439	1683.006	2003.779	2006.661	1866.667	2234.745	0.116244	1752.749	1831.51
YNL227C	264.8588	237.3217	310.2498	345.6364	289.2181	332.6678	0.252051	299.0578	324.8604
YDR256C	316.679	382.6812	411.0316	289.2242	344.5268	317.0128	-0.22391	285.5665	289.7404
YFR032C-I	2.30312	4.944202	0	4.477155	5.761317	6.849043	1.237423	1.124277	0.878001
YJL030W	634.5095	567.5944	579.0013	612.4748	498.9301	497.0448	-0.1471	614.9798	626.8928
YDR338C	350.0742	363.8933	428.8167	282.9562	369.8766	393.3308	-0.12744	368.763	343.2984
YDR117C	937.3698	697.1325	904.072	779.025	916.0495	952.9954	0.060923	992.737	914.8771
YIR009W	408.8038	486.5095	413.9958	421.748	445.926	400.1798	-0.04642	472.1965	477.6326
YPR117W	690.936	701.0879	798.3499	742.3123	725.926	935.3836	0.134033	786.9942	885.0251
YML119W	750.8171	709.9874	677.807	736.9397	631.4404	697.624	-0.04983	730.7804	680.4508
YIL023C	1039.859	1107.501	986.0807	1026.164	1048.56	901.1384	-0.07444	951.1387	919.2671
YBR264C	745.0593	607.148	740.0546	667.9915	631.4404	569.449	-0.16289	700.4249	597.9187
YML055W	751.9686	727.7866	650.1414	692.1682	646.4198	594.8883	-0.13959	668.9451	604.9427
YDR429C	7244.464	7263.033	7130.806	8137.677	7662.552	7073.105	0.080079	8531.018	8015.272
YPR064W	76.00296	129.5381	124.4952	136.1055	120.9877	79.25321	0.027323	83.19653	78.1421
YDR183W	345.468	369.8263	405.1033	412.7937	375.6379	354.1934	0.028341	341.7804	309.0564
YNL339C	2.30312	0.98884	0	0	1.152263	0	-1.51448	0	0
YGL030W	32813.7	26869.76	29312.68	30816.26	31172.18	28740.54	0.027821	35942.03	29535.96
YMR105W-/	12.66716	11.86609	10.86863	15.22233	18.43622	19.56869	0.588339	13.49133	25.46203
YPL004C	14905.79	13633.14	13859.47	12218.16	12310.78	12379.16	-0.20007	13881.45	12765.26
YDR339C	2556.463	2523.521	2398.014	2688.084	2787.325	2179.953	0.033819	2489.15	2295.095
YML110C	3710.326	3184.066	3982.857	3675.744	3369.218	3289.498	-0.07385	4244.147	4102.899
YML085C	6369.278	5579.038	6153.618	6071.918	5657.614	6135.764	-0.01898	5576.416	5789.539
YBR260C	817.6076	708.9986	827.0036	782.6067	948.3128	891.354	0.155943	773.5029	923.6571
YMR085W	100.1857	126.5716	79.04455	73.42534	71.44034	56.74921	-0.60099	132.6647	101.8481

YMR257C	239.5245	259.0762	233.1814	210.4263	224.6914	328.7541	0.061916	211.3642	215.1103
YOL017W	424.9256	387.6254	355.7005	417.2709	405.5967	434.425	0.10597	386.7515	398.6125
YBL034C	546.991	412.3465	649.1533	479.951	520.8231	777.8556	0.145059	580.1272	680.4508
YNL239W	3052.785	2839.95	2930.577	2781.209	2795.391	2955.851	-0.04836	3002.945	2949.206
YIL030C	1395.691	1564.346	1566.07	1817.725	1736.461	2110.484	0.323721	1679.671	1878.044
YMR163C	260.2525	266.9869	274.6798	291.9105	223.5391	319.9482	0.059006	260.8324	198.4282
YDR443C	515.8989	421.246	528.6104	432.4932	407.9013	723.0633	0.093096	410.3613	607.5767
YGR169C-/	2303.12	2231.813	2376.277	2581.528	2206.585	2017.532	-0.02221	2145.121	2306.509
YLR255C	6.90936	12.85493	12.84474	17.90862	14.97943	4.892174	0.21236	8.99422	7.90201
YJR160C	19.57652	11.86609	20.74919	13.43147	13.82716	21.52556	-0.09741	7.869942	5.268006
YLR359W	5534.397	6625.231	6051.848	6510.679	6408.889	5979.215	0.053445	5589.908	5330.345
YHR185C	0	2.966521	0	0	0	0	#NUM!	0	0
YLR374C	0	2.966521	0	0	0	0	#NUM!	0	0
YOR040W	257.9494	316.4289	256.8948	243.5572	197.0371	187.8595	-0.40352	302.4306	295.8864
YBL112C	0	0	0	0	0	0	#DIV/0!	0	0.878001
YLR217W	0	0	0	0	0	0	#DIV/0!	0	0.878001
YMR014W	1786.069	1798.701	1688.589	2305.735	2147.819	2151.578	0.324865	2011.332	2259.975
YHR141C	23393.94	17400.63	20506.13	21869.11	22548.64	21073.53	0.095399	25512.1	20935.06
YBR197C	195.7652	242.2659	258.8709	197.8903	207.4074	244.6087	-0.10072	221.4827	237.0603
YPL029W	481.3521	466.7327	415.9719	419.9572	465.5144	434.425	-0.04748	421.6041	482.0226
YMR048W	460.624	443.9894	384.3541	420.8526	395.2264	360.064	-0.13215	403.6156	333.6404
YNL024C	255.6463	317.4178	275.6679	269.5247	314.5679	234.8243	-0.05159	284.4422	255.4983
YHR057C	1406.055	1148.044	1195.549	1284.944	1258.272	1177.057	-0.01135	1621.208	1375.828
YPR054W	156.6122	253.1432	156.113	169.2365	154.4033	189.8163	-0.14023	161.896	129.9442
YJL050W	2762.592	2799.407	2779.404	3364.134	3581.235	4102.577	0.405416	3337.98	3493.566
YLR015W	594.2049	625.936	716.3412	715.4494	729.3828	709.3652	0.153712	635.2168	576.8467
YPR107C	363.8929	403.4469	440.6733	377.8719	341.07	323.8619	-0.21217	450.8353	392.4665
YER167W	989.19	879.0791	1075.006	1053.922	1231.77	1287.62	0.27984	815.1012	961.4112
YMR116C	57844.01	50486.24	54769.97	58285.4	56350.29	53621.16	0.044907	63659.96	52579.09
YPL225W	3248.551	2849.838	3008.633	3129.531	3023.539	2868.771	-0.01356	3209.812	3062.468
YCR077C	2092.384	2269.389	2271.543	2073.818	2249.218	2444.13	0.028822	2185.595	2440.843
YKL207W	2995.207	2343.552	2413.823	2877.915	2611.029	2236.702	-0.00502	2564.477	2319.679
YDR336W	322.4368	340.1611	311.2379	306.2374	345.679	250.4793	-0.10992	273.1994	248.4743
YLR233C	368.4992	308.5182	313.214	315.1917	309.9589	392.3523	0.039195	267.578	284.4723
YBR152W	142.7934	226.4445	212.4322	176.3999	182.0576	272.9833	0.118446	174.263	228.2803
YKL101W	2478.157	1892.641	2542.27	2487.507	2596.05	3554.653	0.321406	2133.879	2977.302
YGL115W	1804.494	1659.274	1626.342	1692.365	1645.432	1531.25	-0.06406	2012.457	1836.778
YDL069C	137.0356	136.46	137.3399	168.341	138.2716	128.1749	0.08175	158.5231	125.5542
YIL100W	0	0.98884	0	0	1.152263	0	0.220661	1.124277	0
YBR198C	1318.536	1645.43	1351.662	1475.67	1410.37	1511.682	0.027186	1434.578	1656.788
YOR312C	33705.01	26795.6	31723.54	34219.79	32702.39	31231.64	0.0899	35189.89	30684.38
YAL015C	579.2346	572.5386	662.9861	671.5733	591.1112	576.298	0.019129	717.289	648.8428
YIL027C	1224.108	960.1641	1080.934	1225.845	1162.634	1127.157	0.106611	1150.136	1094.867
YIR021W	313.2243	340.1611	261.8351	226.544	252.3457	278.8539	-0.27241	279.9451	226.5243
YLR249W	72638.1	70798.01	83103.48	83618.93	83195.73	106898.9	0.272904	88617.8	92477.22
YLR376C	204.9777	170.0806	173.898	160.2822	175.144	159.4849	-0.14952	182.133	194.9162
YLR409C	2367.607	2571.974	2529.425	2920.896	2876.05	3468.551	0.310952	2577.968	2694.585
YNL005C	1220.654	1345.812	1223.214	1369.114	1329.712	1195.647	0.039352	1223.214	1432.02
YEL068C	680.5719	710.9763	685.7114	696.6453	697.1194	665.3356	-0.01267	722.9104	635.6728
YFR014C	1535.029	1510.948	1523.584	1473.879	1268.642	1364.916	-0.15382	1378.364	1288.906
YJL158C	19822.95	18984.75	19001.32	20323.6	20100.08	17997.33	0.015193	19888.47	17045.51
YDR344C	14.97028	24.72101	56.31924	28.65379	21.89301	41.09426	-0.0672	42.72254	56.19207
YDR523C	50.66864	49.44202	46.43867	29.54922	53.00412	48.92174	-0.1566	26.98266	51.80206
YDL159W-/	4.60624	0	2.96417	10.74517	5.761317	6.849043	1.625321	10.1185	2.634003

YHR178W	360.4383	437.0675	385.3422	371.6039	375.6379	443.2309	0.00927	376.633	395.9785
YDR137W	670.2079	496.3979	650.1414	630.3834	667.1605	692.7318	0.131611	654.3295	744.5449
YNL020C	669.0563	603.1927	626.428	616.952	631.4404	649.6807	-0.00046	694.8035	637.4288
YOR375C	13107.06	12666.06	12710.36	12750.94	12195.56	11484.87	-0.07906	13293.46	12378.06
YHR142W	1060.587	1155.954	1076.982	1090.635	1085.432	975.4994	-0.06356	1147.887	878.0011
YPL142C	9.21248	6.921883	0.988057	3.581724	5.761317	12.71965	0.365722	16.86416	7.90201
YFR032C	25.33432	49.44202	37.54616	31.34009	46.09054	27.39617	-0.09964	33.72832	35.12004
YPR012W	64.48736	62.29695	63.23564	85.96138	57.61317	47.9433	0.011328	49.46821	47.41206
YJL062W	1084.769	1110.468	1164.919	1094.217	1353.91	1491.135	0.229397	1020.844	1116.817
YFR048W	614.933	585.3935	605.6788	630.3834	659.0947	725.9986	0.158319	510.422	711.1809
YHR132C	2387.184	2582.851	2379.241	2556.456	2481.976	2350.2	0.007705	2477.908	2386.407
YDR034C	627.6002	643.7351	767.7202	683.2139	792.7573	905.0521	0.223681	771.2544	772.6409
YNL029C	470.988	381.6924	390.2824	405.6303	511.605	459.8643	0.14785	530.659	467.9746
YMR019W	776.1514	631.869	771.6724	806.7834	805.4322	1024.421	0.274574	815.1012	907.8531
YMR228W	456.0177	551.773	413.0078	520.2454	525.4321	386.4817	0.01149	430.5983	426.7085
YHL038C	641.4189	621.9806	529.5985	573.0759	527.7367	530.3116	-0.13651	617.2283	632.1608
YFL041W	1067.496	1191.553	1161.955	1167.642	1175.309	1156.51	0.032713	1291.795	1432.898
YGL142C	603.4174	682.2999	557.264	631.2789	668.3128	666.314	0.093153	760.0116	718.2049
YDR417C	5.7578	4.944202	0	1.790862	10.37037	2.935304	0.496337	1.124277	1.756002
YBR015C	3579.048	3859.444	4003.606	4108.238	4212.675	4175.959	0.127215	3830.413	3623.51
YNR031C	939.6729	805.905	870.4781	808.5742	840.0001	951.0385	-0.0091	857.8237	870.0991
YBR153W	686.3297	759.4295	729.1859	664.4098	662.5515	639.8963	-0.14509	710.5434	568.9447
YCR093W	1682.429	1469.417	1988.958	1754.149	2038.354	2697.545	0.336235	1769.613	2348.653
YCL045C	2441.307	2474.079	2738.894	2760.614	2786.173	2927.477	0.146821	2589.211	3055.444
YIL049W	200.3714	170.0806	189.7069	168.341	175.144	132.0887	-0.23617	229.3526	167.6982
YBR257W	506.6864	574.5163	522.6821	585.6119	566.9136	487.2605	0.031937	465.4509	481.1446
YPL040C	682.875	689.2218	727.2098	629.488	640.6585	723.0633	-0.07482	512.6705	677.8168
YML002W	366.1961	369.8263	420.9122	369.813	407.9013	471.6055	0.110836	421.6041	460.0726
YKL131W	2.30312	1.977681	1.976114	1.790862	1.152263	0	-1.0881	6.745665	3.512004
YIL017C	446.8053	416.3018	480.1956	392.1988	354.8971	464.7565	-0.14857	328.289	384.5645
YNL264C	1263.261	1230.117	1364.506	1198.982	1236.379	1260.224	-0.06201	1170.373	1062.381
YMR034C	119.7622	113.7166	97.81763	83.27509	73.74486	87.08069	-0.44065	95.56359	72.87409
YHR206W	1583.395	1635.542	1789.371	1766.685	1896.626	2257.249	0.24141	1735.884	2041.352
YOR028C	571.1737	540.8957	606.6669	192.5177	186.6667	165.3555	-1.65824	433.9711	432.8545
YGR112W	559.6581	561.6614	531.5746	503.2322	490.8642	482.3683	-0.16285	546.3989	494.3146
YKR006C	1662.853	1701.794	1507.775	1604.612	1447.243	1410.903	-0.1267	1690.913	1496.992
YDR278C	0	0	0	0	2.304527	0	#DIV/0!	0	0
YGR291C	0	0	0	0	2.304527	0	#DIV/0!	0	0
YMR046W-/	0	0	0	0	2.304527	0	#DIV/0!	0	0
YJL175W	0	0	0	0	1.152263	0.978435	#DIV/0!	0	0
YNR050C	2702.711	2418.704	2541.282	2599.436	2689.383	2672.105	0.055084	2349.74	2141.445
YOR286W	2022.139	1676.085	1711.314	1704.901	1573.992	1540.056	-0.16679	1956.243	1639.228
YNR010W	337.4071	250.1766	269.7395	281.1653	282.3045	204.4929	-0.1588	319.2948	259.0103
YOR201C	727.7859	748.5522	687.6876	688.5865	639.5062	575.3196	-0.18513	621.7254	632.1608
YOR086C	4403.565	3668.598	4425.507	4344.631	4363.622	5468.472	0.181865	4051.896	4141.531
YIL130W	704.7547	960.1641	893.2034	904.3853	978.2717	1389.377	0.355103	640.8382	893.8051
YHR005C	3188.669	3532.138	3190.435	3586.201	3468.313	3148.603	0.041872	3714.613	3536.588
YNL159C	534.3238	623.9583	690.6517	436.0749	503.5391	504.8723	-0.35614	526.1619	515.3866
YGL005C	608.0236	491.4537	529.5985	580.2393	497.7778	578.2549	0.023886	607.1098	647.9648
YER178W	8252.079	8760.137	8232.489	8416.156	8100.412	7246.287	-0.08727	9594.584	9028.485
YDL090C	611.4783	619.0141	583.9416	560.5398	540.4116	554.7725	-0.13206	596.9913	546.1167
YML099C	505.5348	637.8021	634.3325	717.2403	667.1605	885.4834	0.352632	589.1214	760.3489
YPL181W	742.7562	794.0389	854.6692	778.1296	810.0412	831.6695	0.017018	806.107	831.467
YDR413C	6.90936	7.910723	4.940284	5.372586	3.45679	3.913739	-0.63289	3.372832	3.512004

YER077C	474.4427	662.5231	468.3389	578.4484	533.498	577.2765	0.073513	580.1272	625.1368
YPL154C	9461.216	8887.698	8769.004	8629.269	8917.367	8429.215	-0.06208	11286.62	9705.424
YKL040C	1324.294	1254.839	1290.402	1269.721	1326.255	1378.615	0.038646	1233.332	1129.987
YPL280W	58.72956	45.48666	27.66559	47.45784	53.00412	19.56869	-0.13584	41.59827	27.21803
YDL078C	4532.54	4434.949	4713.031	4491.482	4445.432	4238.579	-0.05427	4621.905	4267.085
YHR058C	589.5987	701.0879	628.4041	677.8413	589.9589	661.4219	0.007596	684.685	682.2068
YOR044W	489.413	557.706	562.2043	385.0353	369.8766	374.7405	-0.51058	363.1416	334.5184
YGR067C	32.24368	15.82145	41.49839	42.08526	34.5679	20.54713	0.11805	13.49133	26.34003
YGL189C	41840.78	30637.24	36735.95	39005.87	39180.41	37802.8	0.086832	44448.31	37936.67
YJL102W	330.4977	304.5629	372.4974	300.8648	244.2799	309.1854	-0.238	317.0462	367.8825
YIL177C	0	0	0.988057	1.790862	0	1.956869	1.923352	0	0
YJL146W	621.8424	671.4227	627.4161	592.7753	638.354	581.1902	-0.08378	519.4162	466.2186
YOR109W	1624.851	1547.535	1580.891	1502.533	1527.901	1954.913	0.068771	1500.91	1458.36
YMR005W	1905.832	1981.636	1943.508	2118.59	2027.984	2096.786	0.098585	1778.607	1806.926
YKR086W	586.144	624.9472	674.8428	621.4291	663.7038	818.9499	0.157912	628.4711	709.4249
YBR138C	207.2808	203.7011	182.7905	193.4131	210.8642	244.6087	0.128055	200.1214	214.2323
YPL098C	1541.939	1406.131	1443.551	1270.617	1370.041	1188.798	-0.19761	1614.462	1243.25
YBR181C	45967.97	35921.61	41595.22	45897.11	41981.57	39079.66	0.040022	52927.61	46196.03
YBR188C	477.8974	428.1679	432.7689	406.5257	419.4239	338.5384	-0.20128	405.8642	342.4204
YER054C	238.3729	216.5561	249.9784	199.6811	205.1029	163.3986	-0.31108	184.3815	253.7423
YDL166C	1308.172	1187.597	1255.82	1352.996	1359.671	1105.631	0.025428	1291.795	1109.793
YJR071W	5.7578	13.84377	11.85668	11.6406	9.218108	8.805912	-0.08469	4.49711	7.90201
YOR211C	1112.407	953.2422	1079.946	967.9609	1096.955	1259.245	0.079657	934.2746	934.1931
YNL047C	609.1752	610.1145	549.3596	655.4555	596.8725	674.1415	0.123311	738.6503	623.3808
YKR017C	276.3744	228.4221	235.1575	184.4588	241.9753	206.4497	-0.22549	225.9798	188.7702
YOR243C	2479.309	2569.996	2609.458	2995.217	3169.877	3391.255	0.319348	3077.147	3020.324
YPL023C	1412.964	1572.256	1514.691	1584.017	1459.918	1394.269	-0.01992	1459.312	1859.606
YML103C	1408.358	1364.6	1454.42	1490.893	1567.078	2019.489	0.264344	1660.558	1649.764
YGR051C	73.69984	89.98448	63.23564	77.00707	81.81071	70.4473	0.014833	80.94798	70.24009
YDR022C	495.1708	407.4023	448.5778	357.277	407.9013	298.4226	-0.34523	367.6387	396.8565
YOR129C	435.2897	602.2038	559.2402	471.8922	550.7819	578.2549	0.003786	401.3671	453.0486
YPR168W	80.6092	116.6832	149.1966	119.9878	150.9465	184.9242	0.395777	133.789	158.0402
YKL096W-/	71970.19	63183.94	65688.98	76949.76	80812.85	68950.29	0.174798	79674.17	65645.51
YLR252W	92.1248	71.19651	57.3073	47.45784	31.11111	47.9433	-0.80234	56.21387	55.31407
YAL019W	1309.324	1364.6	1618.437	1574.168	1762.963	2399.122	0.418337	1482.922	1996.574
YLR209C	962.7041	940.3873	943.5943	1044.968	950.6174	996.0465	0.071649	1034.335	907.8531
YLR444C	0	0	0	0	0	0	#DIV/0!	0	0
YGL024W	0	0	0	0	0	0	#DIV/0!	0	0
YEL010W	0	0	0	0	0	0	#DIV/0!	0	0
YEL034C-/	0	0	0	0	0	0	#DIV/0!	0	0
YHR028W-/	0	0	0	0	0	0	#DIV/0!	0	0
YGL086W	624.1455	585.3935	649.1533	690.3773	751.2758	922.6639	0.347136	706.0463	777.909
YDL187C	0	0	0	0	0	0	#DIV/0!	0	0
YDR431W	0	0	0	0	0	0	#DIV/0!	0	0
YKR032W	0	0	0	0	0	0	#DIV/0!	0	0
YOR366W	0	0	0	0	0	0	#DIV/0!	0	0
YNL072W	703.6031	708.0098	807.2424	836.3326	746.6667	721.1064	0.054391	607.1098	700.6449
YBR172C	1644.428	1825.399	2123.334	1888.464	2025.679	2206.37	0.129989	1979.853	2136.177
YBR241C	1281.686	1542.591	1148.122	1215.1	1143.045	1007.788	-0.239	1096.171	998.2872
YNL061W	4182.466	5197.345	4518.384	5367.214	5417.943	5126.019	0.195143	5448.249	5475.215
YDL032W	0	0	0	0	0	0	#DIV/0!	0	0
YIL068W-/	0	0	0	0	0	0	#DIV/0!	0	0
YOL019W-/	0	0	0	0	0	0	#DIV/0!	0	0
YPR002C-/	6.90936	2.966521	7.904455	2.686293	4.609054	2.935304	-0.79738	1.124277	3.512004

YAL026C-/	0	0	1.976114	0	1.152263	0.978435	0.10866	2.248555	1.756002
YCR037C	1700.854	1769.036	2092.704	1647.593	1865.515	2553.715	0.125183	1893.283	2324.069
YLR416C	2.30312	1.977681	8.892511	3.581724	3.45679	1.956869	-0.55036	7.869942	7.90201
YCR034W	3179.457	3223.62	3082.737	3540.534	3436.05	2879.533	0.055248	3080.52	3065.98
YMR028W	631.0548	641.7574	650.1414	666.2007	713.2511	543.0313	-0.00035	586.8728	533.8247
YBR253W	769.242	700.099	683.7353	706.4951	713.2511	534.2254	-0.13999	802.7341	834.101
YBR012C	131.2778	92.951	90.90123	74.32078	89.87655	85.12382	-0.33794	138.2861	100.0921
YKL024C	2731.5	2445.402	2657.873	2983.576	2735.473	2459.785	0.062003	3180.581	2616.443
YOL101C	419.1678	518.1524	446.6017	440.5521	532.3457	339.5168	-0.07654	315.922	310.8124
YKL069W	1128.529	973.019	1076.982	883.7904	943.7038	918.7502	-0.2109	1106.289	893.8051
YNL329C	571.1737	675.378	561.2163	612.4748	613.0042	711.322	0.099466	665.5723	692.7428
YGR279C	17537.11	18912.56	16936.28	18237.24	17288.56	16258.65	-0.04394	18257.14	16793.53
YIL089W	158.9153	127.5604	144.2563	142.3735	123.2922	136.0024	-0.10079	187.7543	166.8202
YHR123W	960.401	1131.233	1073.03	1150.629	1187.984	927.5561	0.045547	936.5231	886.7811
YLR125W	55.27488	57.35275	55.33118	59.09845	63.37449	59.68452	0.117079	52.84104	86.92211
YOL024W	18.42496	7.910723	11.85668	1.790862	14.97943	8.805912	-0.57848	0	5.268006
YAL056W	564.2644	628.9025	521.694	557.8535	485.1029	468.6702	-0.18199	505.9249	563.6767
YHR109W	286.7384	300.6075	256.8948	267.7339	226.9959	239.7165	-0.201	227.1041	211.5983
YDL227C	183.098	191.835	159.0771	175.5045	169.3827	135.024	-0.1541	150.6532	137.8462
YFR034C	1036.404	1621.698	1216.298	1618.044	1550.947	1326.757	0.214588	900.5463	1150.181
YAL058W	538.9301	603.1927	618.5236	530.9906	548.4774	529.3332	-0.13012	608.2341	563.6767
YDR318W	352.3773	354.0049	387.3183	348.3227	303.0453	305.2716	-0.19317	364.2659	403.8805
YNL080C	1336.961	1471.395	1323.996	1574.168	1390.782	1128.135	-0.01377	1209.723	1241.494
YKL160W	2054.383	1630.598	1924.735	1995.02	2055.638	1825.759	0.067009	2584.714	2224.855
YDR016C	454.8662	333.2392	358.6646	422.6434	404.4445	355.1718	0.043971	423.8526	348.5664
YLR270W	1647.882	1650.375	1573.975	1379.859	1454.156	1370.787	-0.21254	1752.749	1629.57
YER165W	15661.22	15448.65	15589.56	16217.15	16155.89	16121.67	0.054422	15139.52	15338.68
YJL179W	1032.949	953.2422	890.2392	1061.981	959.8355	902.1168	0.023631	1028.714	913.1211
YLR078C	963.8557	973.019	884.3109	942.8889	1000.165	896.2462	0.009234	1016.347	800.737
YIL112W	1867.83	1578.189	1866.439	1531.187	1443.786	1716.174	-0.17944	1290.671	1568.11
YPR059C	2.30312	0	0.988057	1.790862	1.152263	2.935304	0.836827	4.49711	7.024009
YHR122W	809.5466	795.0277	768.7082	827.3783	724.7737	712.3005	-0.06772	757.763	674.3048
YER007C-/	2156.872	1771.013	1875.332	2163.361	2120.165	1911.861	0.094342	2455.422	2259.975
YHR056C	268.3135	265.0092	401.1511	309.8191	351.4404	676.0984	0.51716	371.0116	412.6605
YKL026C	570.0222	636.8132	562.2043	488.0099	496.6256	364.9561	-0.39044	636.3411	561.9207
YGL074C	0	1.977681	0	0	3.45679	0	0.805623	2.248555	4.390005
YKL157W	3838.149	3255.263	3524.399	3373.089	3538.601	3807.089	0.013654	3548.22	3282.846
YNL078W	885.5496	1094.646	1044.376	1185.551	1101.564	1039.098	0.137149	1055.697	1115.061
YJL042W	1631.76	1419.975	1630.294	1387.023	1473.745	1820.867	-0.00012	1440.199	1828.876
YHR131W-/	2.30312	1.977681	6.916398	1.790862	1.152263	6.849043	-0.19344	0	0.878001
YBL021C	245.2823	239.2994	319.1424	275.7928	218.9301	204.4929	-0.20096	231.6012	215.1103
YOR221C	865.9731	1026.416	781.5529	899.9082	844.6091	847.3245	-0.04499	817.3497	763.8609
YDR509W	0	0.98884	0	2.686293	1.152263	0	1.956754	1.124277	0
YDR545W	2.30312	0.98884	2.96417	0	0	0.978435	-2.67672	5.621387	0.878001
YDL077C	499.777	454.8666	552.3238	453.0881	521.9754	611.5217	0.074277	548.6474	600.5527
YPR136C	0	0.98884	0	0	0	0.978435	-0.01526	0	0
YGR114C	1.15156	0	0	0.895431	0	0	-0.36294	0	0
YLR339C	1.15156	0	0	0.895431	0	0	-0.36294	0	0
YKL119C	663.2985	620.003	600.7386	638.4423	595.7202	497.0448	-0.12205	716.1648	580.3587
YLR393W	74.8514	85.04028	95.84151	118.1969	109.465	128.1749	0.476577	51.71676	129.9442
YJL061W	1019.131	972.0301	1065.125	1052.131	1009.383	1282.728	0.1299	1096.171	1204.617
YHR015W	34.5468	14.83261	19.76114	36.71267	55.30865	41.09426	0.945076	30.35549	22.82803
YNL188W	567.719	542.8734	600.7386	607.1022	603.7861	708.3867	0.165443	656.578	625.1368
YGL008C	54425.03	60949.16	63095.33	66024.61	73580.09	83316.65	0.320857	68311.1	77611.78

YPR170C	10.36404	7.910723	5.928341	7.163448	3.45679	3.913739	-0.73576	7.869942	2.634003
YGL241W	634.5095	585.3935	731.1621	878.4178	750.1235	958.866	0.407245	817.3497	820.053
YGL194C	312.0727	395.5362	285.5484	369.813	334.1564	350.2796	0.086122	347.4017	293.2524
YOL013W-I	5.7578	0.98884	1.976114	0	3.45679	0.978435	-0.97578	0	3.512004
YGR213C	192.3105	161.181	151.1727	113.7197	150.9465	172.2045	-0.20812	127.0434	186.1362
YOR255W	3.45468	2.966521	1.976114	3.581724	1.152263	0	-0.82687	0	1.756002
YNL048W	917.7933	923.577	951.4987	951.8432	1010.535	1017.572	0.09354	969.1272	790.201
YAL040C	1003.009	1130.245	1049.316	1188.237	1101.564	1109.545	0.095065	1016.347	1232.714
YAL008W	689.7844	675.378	910.9884	744.9986	801.9754	724.0417	-0.00326	758.8873	673.4268
YGR057C	443.3506	521.1189	512.8015	507.7094	496.6256	446.1662	-0.02638	465.4509	523.2886
YIL054W	16.12184	3.955362	5.928341	4.477155	6.913581	2.935304	-0.86018	13.49133	10.53601
YIL030W-A	0	0.98884	0	0.895431	0	0	-0.14316	0	1.756002
YIL021W	758.878	760.4183	805.2663	894.5356	733.9918	808.1871	0.067978	739.7746	725.2289
YBL038W	1545.393	1341.856	1339.805	1472.984	1221.399	1144.769	-0.13887	1385.11	1260.81
YMR075C-A	3.45468	0	1.976114	0.895431	1.152263	0.978435	-0.84369	4.49711	0.878001
YBR014C	1409.509	1304.281	1383.28	1359.264	1327.408	1149.661	-0.09486	1704.405	1435.532
YMR033W	1634.064	1417.008	1588.795	1564.318	1681.152	1685.843	0.087888	1480.673	1452.214
YDR014W	154.309	125.5827	147.2205	187.1451	178.6008	244.6087	0.515032	163.0202	189.6482
YFR046C	382.3179	354.0049	460.4345	360.8587	357.2017	362.0208	-0.14799	322.6676	321.3484
YMR115W	749.6655	903.8002	754.8754	653.6646	736.2964	721.1064	-0.19007	764.5087	847.271
YEL054C	4869.947	4184.773	4158.731	4786.079	4803.786	4071.267	0.04807	5059.249	4307.473
YOR270C	7127.004	7354.995	8328.331	8466.3	8696.132	10679.61	0.287578	8473.679	9011.803
YGR285C	13452.52	13894.2	12540.42	13894.4	13068.97	12992.63	0.002489	14770.76	13424.64
YMR073C	723.1796	780.1951	707.4487	748.5803	659.0947	649.6807	-0.10379	671.1937	658.5008
YBR184W	70.24516	69.21883	72.12815	83.27509	87.57202	76.31791	0.224189	38.22543	78.1421
YDR342C	2969.873	2646.137	2410.859	1541.932	1614.321	1698.563	-0.72542	2502.642	2099.301
YBR097W	444.5021	396.525	402.1391	306.2374	361.8107	557.7078	-0.02035	490.185	405.6365
YGR014W	6852.933	6336.489	8184.075	7655.04	8939.26	10452.62	0.339641	7124.546	9050.435
YGR277C	788.8186	781.1839	760.8038	664.4098	768.5597	663.3787	-0.15295	889.3035	770.0069
YNL077W	323.5883	422.2349	356.6885	298.1785	289.2181	252.4362	-0.39262	260.8324	293.2524
YBR189W	55438.4	43762.12	49384.07	53866.44	51221.57	47806.32	0.04125	59016.7	48570.14
YNR024W	245.2823	347.083	298.3932	362.6496	297.284	359.0855	0.194075	224.8555	332.7624
YJL169W	19.57652	6.921883	12.84474	9.849741	6.913581	12.71965	-0.41623	7.869942	10.53601
YDL094C	11.5156	10.87724	1.976114	1.790862	9.218108	4.892174	-0.61591	6.745665	10.53601
YER107W-A	11.5156	27.68753	21.73725	35.81724	18.43622	33.26678	0.522218	19.11272	34.24204
YPL018W	131.2778	146.3484	147.2205	130.7329	112.9218	106.6494	-0.27833	119.1734	137.8462
YKL195W	3599.776	3496.54	3334.692	2945.073	2808.066	2748.423	-0.29508	2826.434	3131.83
YLR154C-(	0	0	2.96417	0	2.304527	0	-0.36316	0	0.878001
YNL217W	1110.104	1056.082	1085.874	1188.237	1198.354	1079.213	0.091836	1155.757	917.5111
YML043C	818.7591	937.4207	1042.4	1052.131	1075.062	1119.329	0.2142	933.1503	999.1652
YLR319C	781.9092	593.3043	717.3293	748.5803	810.0412	855.1519	0.206033	771.2544	777.031
YPL071C	347.7711	387.6254	370.5213	346.5318	330.6996	265.1558	-0.23085	383.3786	300.2764
YNL203C	20.72808	11.86609	10.86863	8.058879	13.82716	17.61182	-0.13801	13.49133	7.024009
YFL023W	940.8245	1091.68	937.6659	1187.342	1221.399	1268.051	0.307902	1068.064	1263.444
YHR053C	2.30312	0	0	0	0	0	#NUM!	5.621387	0
YLR394W	278.6775	286.7637	280.6081	274.8973	293.8272	331.6894	0.089846	338.4075	336.2744
YGL109W	36.84992	19.77681	24.70142	25.9675	14.97943	30.33148	-0.19029	19.11272	19.31602
YHR035W	112.8529	132.5046	168.9577	128.9421	175.144	194.7085	0.267717	151.7775	123.7982
YBL071C-I	12.66716	0.98884	1.976114	6.268017	3.45679	1.956869	-0.42027	1.124277	1.756002
YOL161C	0	1.977681	3.952227	0	2.304527	0	-1.36354	0	1.756002
YNL283C	2848.959	2568.019	2956.266	2546.606	2673.251	3037.061	-0.02018	2663.413	2650.685
YGL239C	0	1.977681	0	1.790862	1.152263	1.956869	1.308971	2.248555	2.634003
YML037C	73.69984	127.5604	123.5071	119.0923	81.81071	141.873	0.07786	73.07804	136.0902
YPL229W	468.6849	420.2572	326.0588	353.6953	328.3951	317.0128	-0.28225	327.1647	366.1264

YNL022C	1381.872	1624.665	1378.339	1548.2	1615.473	1663.339	0.138595	1504.283	1528.6
YNL277W	710.5125	1138.155	904.072	794.2473	624.5268	600.7589	-0.44685	701.5491	705.0349
YNL328C	90.97324	96.90636	72.12815	69.84362	63.37449	87.08069	-0.23909	37.10116	86.92211
YEL023C	156.6122	178.9801	151.1727	144.1644	111.7696	189.8163	-0.12699	100.0607	147.5042
YNL273W	587.2956	474.6434	579.0013	551.5855	482.7984	712.3005	0.090096	622.8497	596.1627
YMR326C	2.30312	0	0.988057	0	2.304527	0	-0.51413	0	0
YER165C-/	0	10.87724	8.892511	4.477155	4.609054	10.76278	0.005771	0	21.95003
YGR143W	496.3223	600.2261	579.0013	541.7358	687.9013	799.3812	0.276147	482.315	551.3847
YIR021W-/	0	2.966521	0	11.6406	8.065844	2.935304	2.932142	2.248555	6.146008
YHR061C	309.7696	344.1165	319.1424	292.8059	255.8025	281.7892	-0.22868	257.4595	323.9824
YDL127W	1029.495	1060.037	871.4661	955.4249	1090.041	780.7909	-0.06719	765.633	883.2691
YER035W	688.6328	809.8603	648.1653	473.683	406.749	315.056	-0.84449	534.0318	477.6326
YPR106W	264.8588	300.6075	248.0023	250.7207	300.7408	236.7812	-0.04545	308.052	219.5003
YNL295W	515.8989	562.6502	436.7211	409.212	406.749	422.6838	-0.29081	456.4567	567.1887
YGL020C	1573.031	1433.819	1532.476	1706.692	1701.893	1550.819	0.127689	1763.991	1657.666
YER148W-/	9.21248	8.899564	2.96417	5.372586	2.304527	7.827478	-0.44292	11.24277	6.146008
YBL049W	180.7949	268.9646	200.5755	188.9359	138.2716	139.9162	-0.47738	148.4046	163.3082
YBR119W	504.3833	501.3421	454.5061	478.1602	481.6461	356.1502	-0.15009	424.9769	408.2705
YDR053W	0	0	1.976114	0.895431	2.304527	0.978435	1.080282	0	2.634003
YIR031C	154.309	207.6565	214.4083	140.5827	169.3827	162.4202	-0.28704	159.6474	135.2122
YIR017W-/	0	2.966521	3.952227	1.790862	4.609054	1.956869	0.272437	0	0
YER184C	127.8232	157.2256	153.1488	98.49741	107.1605	148.7221	-0.30628	103.4335	94.82412
YIR035C	1672.065	1453.595	1462.324	1671.77	1516.379	1389.377	-0.00329	1599.847	1495.236
YNL021W	622.9939	734.7084	778.5888	671.5733	706.3375	799.3812	0.027427	591.37	643.5748
YPL210C	3007.875	3054.528	3318.883	3429.501	3509.795	3717.073	0.183858	2902.884	3095.832
YLR399C	1544.242	1698.828	1815.06	1644.011	1784.856	2109.505	0.130858	1570.616	1871.02
YHR084W	1719.279	1618.732	1680.685	1818.62	1864.362	1943.171	0.164837	1569.491	1992.184
YLL020C	2.30312	1.977681	4.940284	3.581724	1.152263	0.978435	-0.69083	3.372832	0.878001
YHR153C	55.27488	42.52014	35.57005	38.50353	38.02469	43.05113	-0.15741	37.10116	19.31602
YKL192C	5139.412	4840.374	4571.739	4478.946	4677.037	4076.159	-0.13712	5486.474	4374.201
YER073W	1342.719	1673.118	1600.652	1482.834	1364.28	1343.391	-0.13967	1291.795	1388.12
YGL065C	982.2806	891.9341	913.9526	1003.778	1046.255	1043.011	0.149711	992.737	964.0452
YOL094C	978.8259	843.4809	825.0274	951.8432	985.1853	833.6264	0.065687	816.2254	772.6409
YER137C	110.5498	94.92868	116.5907	102.9746	102.5514	63.59826	-0.2591	164.1445	120.2861
YKL027W	1749.22	2196.215	1815.06	2016.511	2027.984	1738.678	0.005669	1852.809	1780.586
YML028W	53780.15	45797.16	49284.27	59919.56	56554.24	50587.03	0.166402	54296.98	46189.88
YIL039W	2252.451	2230.824	2383.193	2408.709	2586.831	2525.34	0.131333	2471.162	2192.369
YOL141W	126.6716	182.9355	197.6114	188.0405	192.428	338.5384	0.503398	177.6358	282.7163
YPL148C	253.3432	239.2994	178.8383	198.7857	149.7943	146.7652	-0.43891	263.0809	189.6482
YLR358C	17.2734	16.81029	13.8328	30.44465	12.6749	24.46087	0.496083	12.36705	14.92602
YDR090C	385.7726	523.0966	535.5268	341.1592	441.3169	365.9346	-0.33083	418.2312	390.7105
YBR074W	1334.658	1297.359	1324.984	1407.618	1326.255	1435.364	0.075376	1509.905	1353.878
YPR158W	886.7012	1153.977	837.8722	753.0575	517.3663	420.7269	-0.76734	701.5491	628.6488
YCL010C	344.3164	531.0073	523.6701	443.2384	519.6708	582.1687	0.14329	456.4567	513.6306
YLR127C	399.5913	475.6322	448.5778	440.5521	426.3375	420.7269	-0.03998	458.7052	505.7286
YKR057W	16664.22	13060.6	14527.4	15668.25	15436.87	14606.07	0.046798	17519.62	14748.66
YLR138W	976.5228	1155.954	1001.89	1149.733	903.3746	795.4674	-0.13793	1051.199	1204.617
YFR006W	2966.418	3018.93	3018.514	3142.963	3043.128	2917.692	0.015922	3100.757	2586.591
YOR099W	3749.479	3407.544	3545.148	3707.084	3477.531	3434.306	-0.01127	3568.457	3424.204
YHR063C	1092.83	1190.564	1134.289	1159.583	1311.276	1020.507	0.030773	1047.827	1055.357
YKL169C	0	2.966521	1.976114	0.895431	0	2.935304	-0.36766	0	0.878001
YML091C	1594.911	1567.312	1750.837	1698.633	1864.362	2050.799	0.192355	1286.173	1804.292
YDL133W	824.5169	793.05	871.4661	761.1164	730.535	738.7182	-0.1583	794.8642	661.1348
YOL045W	530.8691	489.476	547.3835	488.0099	649.8766	700.5593	0.229811	539.6532	621.6248

YOR139C	0	3.955362	3.952227	0.895431	1.152263	1.956869	-0.98159	3.372832	3.512004
YJR140C	477.8974	486.5095	514.7776	498.7551	508.1482	822.8636	0.306858	531.7832	633.9168
YMR126C	371.9539	476.6211	349.7721	388.6171	342.2223	309.1854	-0.20443	355.2717	370.5165
YKR098C	234.9182	294.6744	269.7395	230.1258	214.321	257.3283	-0.18779	240.5954	245.8403
YBR200W-/	2.30312	1.977681	1.976114	3.581724	3.45679	0.978435	0.357602	3.372832	0
YOR001W	1923.105	2118.096	2127.286	2423.932	2411.687	2801.259	0.308066	2362.107	2318.801
YMR129W	1829.829	1668.174	1817.037	1791.757	1955.391	2611.442	0.258627	1961.864	2127.397
YGL138C	0	1.977681	0	0	0	0	#NUM!	0	0
YBR211C	314.3759	319.3955	330.011	250.7207	258.107	248.5224	-0.34775	247.341	237.0603
YDR493W	601.1143	547.8176	600.7386	464.7287	517.3663	386.4817	-0.35441	634.0925	459.1946
YMR290W-/	1.15156	5.933043	5.928341	3.581724	1.152263	0	-1.45882	1.124277	0
YOR274W	825.6685	1011.584	917.9048	929.4574	935.6379	899.1815	0.004768	884.8064	764.7389
YBR128C	120.9138	164.1475	156.113	98.49741	114.0741	113.4984	-0.43617	137.1619	107.1161
YHR139C-/	9.21248	12.85493	7.904455	8.95431	11.52263	5.870608	-0.18594	5.621387	7.024009
YOL086C	160784.3	146329.6	148185.8	157816.1	151743.9	135790.1	-0.03188	180334.1	154853
YLR427W	1115.862	966.0971	1150.098	1087.053	1192.593	1303.275	0.148684	1015.223	1340.708
YMR089C	1497.028	1589.067	1706.374	1455.075	1553.251	1498.962	-0.08851	1606.593	1542.648
YOR015W	470.988	349.0607	389.2944	352.7998	414.8148	323.8619	-0.14794	381.1301	358.2244
YDR266C	1607.578	1595	1767.634	1816.83	1867.819	1924.581	0.174496	1703.28	1982.526
YPR194C	1799.888	2070.632	2007.731	1787.28	1850.535	1678.016	-0.14509	1751.624	1791.122
YLR129W	2392.942	2653.059	2736.917	2853.739	3273.581	4011.582	0.381518	2531.873	2828.919
YOR376W-/	19.57652	32.63173	40.51033	23.28121	23.04527	27.39617	-0.33075	28.10694	55.31407
YJL202C	8.06092	5.933043	12.84474	4.477155	13.82716	8.805912	0.014522	8.99422	9.658012
YBL104C	660.9954	753.4964	752.8993	670.6778	841.1523	868.85	0.135414	607.1098	707.6689
YDR315C	168.1278	167.114	130.4235	121.7786	130.2058	129.1534	-0.28898	158.5231	138.7242
YOR121C	61.03268	54.38622	44.46256	50.14414	76.04939	45.98643	0.106915	60.71098	44.77805
YML109W	741.6046	486.5095	615.5594	576.6576	674.0741	824.8205	0.170912	652.0809	718.2049
YDR211W	2520.765	2318.831	2392.086	2560.933	2741.235	2998.902	0.198966	2588.087	2804.335
YMR075W	327.043	390.592	507.8612	408.3165	516.214	669.2493	0.379086	418.2312	518.8986
YGR229C	2369.91	2576.918	2758.655	2840.307	2867.984	3155.452	0.202031	2028.197	2621.711
YDR291W	571.1737	635.8244	594.8102	561.4353	663.7038	696.6455	0.093001	645.3353	641.8188
YOR081C	742.7562	835.5702	829.9677	843.496	886.0906	822.8636	0.083871	744.2717	830.589
YNL057W	0	0.98884	0.988057	0	0	0	#NUM!	0	0
YJL010C	1575.334	1931.205	1800.24	2186.643	2124.774	2170.168	0.28851	1888.786	1855.216
YDR445C	0	0	1.976114	0	0	0	#NUM!	0	0
YFL015W-/	0	1.977681	0	0	0	0	#NUM!	0	0
YJL223C	0	1.977681	0	0	0	0	#NUM!	0	0
YMR324C	0	0	1.976114	0	0	0	#NUM!	0	0
YOR146W	0	0	1.976114	0	0	0	#NUM!	0	0
YMR001C-/	17.2734	23.73217	8.892511	20.59491	26.50206	19.56869	0.41796	19.11272	29.85204
YER086W	3691.901	4241.137	3783.27	4076.897	4079.013	3439.198	-0.015	3387.448	3690.239
YLR137W	325.8915	346.0942	337.9154	281.1653	304.1976	293.5304	-0.20045	317.0462	244.0843
YDR365C	2117.719	2437.492	2498.796	2952.236	2995.885	3046.846	0.350675	2622.939	3128.318
YOR321W	446.8053	472.6657	527.6223	524.7226	496.6256	517.592	0.088779	535.1561	489.0466
YLR448W	20006.05	17386.78	18594.24	20561.78	20269.47	18319.23	0.079296	21421.98	18606.6
YJR141W	494.0192	511.2305	644.2131	521.1409	511.605	596.8452	-0.01749	511.5463	454.8046
YHR111W	663.2985	847.4363	801.3141	847.0778	719.0124	744.5888	-0.00086	738.6503	810.395
YPR092W	6.90936	5.933043	9.880568	9.849741	12.6749	6.849043	0.370373	14.61561	14.04802
YLR230W	179.6434	269.9534	267.7634	260.5704	238.5185	268.0911	0.096868	279.9451	328.3724
YDL138W	413.41	631.869	535.5268	461.147	546.1729	456.929	-0.1105	413.7341	563.6767
YGL198W	393.8335	365.871	352.7363	353.6953	373.3334	304.2932	-0.10923	472.1965	377.5405
YPR067W	634.5095	545.8399	575.0491	537.2586	526.5844	525.4194	-0.14344	735.2775	621.6248
YIL022W	2665.861	3404.578	2717.156	3063.27	2979.753	2707.329	-0.00613	3101.882	3303.04
YDR113C	869.4278	734.7084	886.287	855.1366	780.0824	804.2733	-0.02981	753.2659	818.297

YGR206W	381.1663	332.2504	403.1272	373.3947	327.2428	278.8539	-0.18894	384.5029	313.4464
YOR042W	2744.167	2514.621	2773.476	2698.829	2491.194	2265.076	-0.10758	2707.26	2713.023
YER179W	29.94056	63.28579	43.4745	44.77155	50.69959	19.56869	-0.24889	67.45665	44.77805
YPL274W	4398.959	7283.799	4819.741	5555.254	5308.478	4676.918	-0.08664	4042.902	3878.131
YAL032C	644.8736	681.3111	719.3054	824.692	604.9383	626.1982	0.007273	754.3902	746.3009
YPL259C	684.0266	768.329	644.2131	777.2341	617.6132	665.3356	-0.02526	691.4306	753.3249
YER131W	30135.17	25078.97	27230.85	30422.27	30140.91	27767	0.099475	35605.87	32335.02
YML106W	6859.843	6598.532	6259.34	7167.925	6875.556	6097.605	0.030649	7442.717	6256.636
YIL085C	1735.401	1843.199	1697.482	1696.842	1966.914	1817.932	0.055153	1986.598	1680.494
YNL318C	0	1.977681	0	3.581724	0	3.913739	1.922208	0	1.756002
YER175W-/	17.2734	32.63173	27.66559	34.02638	8.065844	29.35304	-0.11867	10.1185	21.95003
YKL058W	1270.171	1089.702	1121.444	1210.623	1162.634	983.3269	-0.05264	1249.072	1026.383
YER087C-I	2449.368	2060.743	2292.292	2269.022	2577.613	2088.958	0.027975	2499.269	2167.785
YDL030W	727.7859	737.675	672.8667	607.9977	708.642	807.2086	-0.0098	688.0578	670.7928
YDR430C	1062.89	1033.338	1152.074	997.5102	1034.733	1065.515	-0.06846	1089.425	1248.518
YJL077W-I	10.36404	14.83261	12.84474	1.790862	19.58848	5.870608	-0.48132	10.1185	13.17002
YBR126W-/	4.60624	2.966521	1.976114	2.686293	3.45679	1.956869	-0.23742	4.49711	3.512004
YOR063W	85714.06	83343.43	83161.78	91346.5	86969.39	88476.92	0.081041	104020.4	89385.78
YDR410C	879.7918	682.2999	704.4845	829.1691	818.1071	681.969	0.039348	735.2775	689.2308
YMR203W	5201.596	5494.986	5280.176	5291.102	5522.799	5136.782	-0.00236	5998.02	5844.853
YNR027W	1107.801	1143.1	1096.743	1234.799	1122.305	981.37	-0.00396	1106.289	981.6052
YIL047C-/	1.15156	3.955362	0.988057	0.895431	6.913581	9.784347	1.529338	1.124277	3.512004
YHR162W	9700.741	8217.264	8573.369	9446.797	9170.865	8430.194	0.029992	11549.7	9182.135
YER089C	2105.052	2224.891	1903.986	2261.859	2004.938	1760.204	-0.0487	2003.462	1747.222
YGR276C	1058.284	1057.07	1011.77	970.6472	1116.543	1114.437	0.033969	1145.639	1062.381
YBL047C	4892.978	3933.607	4991.663	4378.658	4716.214	5611.323	0.089849	4954.691	5325.955
YMR223W	385.7726	400.4804	434.745	415.48	440.1646	458.8859	0.106487	487.9364	427.5865
YCR014C	333.9524	363.8933	410.0436	324.146	338.7655	417.7916	-0.03584	346.2775	308.1784
YML054C-/	41.45616	41.5313	26.67753	37.6081	16.13169	18.59026	-0.60044	32.60405	10.53601
YOR017W	573.4768	661.5342	646.1892	564.1215	642.963	727.9554	0.04071	582.3757	621.6248
YJL007C	1.15156	0.98884	5.928341	0	0	5.870608	-0.45883	0	0
YOR228C	346.6195	465.7438	450.5539	354.5907	343.3745	344.409	-0.27689	455.3324	442.5125
YMR260C	9883.839	8030.373	8723.554	9542.608	9612.182	8736.444	0.066339	11409.17	10039.94
YOL132W	62.18424	42.52014	61.25952	28.65379	29.95885	28.37461	-0.93199	56.21387	44.77805
YJL016W	441.0475	499.3644	459.4464	549.7946	456.2963	520.5273	0.125059	477.8179	403.0025
YDL140C	4568.238	4475.492	4534.193	4373.285	5150.618	5292.353	0.125918	4733.208	5178.45
YGL147C	39855.49	30176.44	37088.69	38903.79	36871.28	36376.25	0.06621	48219.14	40693.59
YDR301W	900.5199	853.3693	861.5856	815.7377	897.6132	1109.545	0.110103	854.4509	855.173
YDR535C	20.72808	24.72101	22.72531	24.17664	17.28395	27.39617	0.014368	16.86416	14.04802
YDR229W	900.5199	771.2955	841.8244	937.5163	775.4733	771.0066	-0.01712	807.2312	976.3372
YBL069W	298.254	313.4624	272.7037	311.61	312.2634	279.8323	0.031121	292.3121	279.2043
YJL072C	441.0475	415.313	456.4823	407.4211	435.5556	402.1367	-0.07642	395.7457	457.4386
YOR332W	2342.273	1768.047	1701.434	1986.961	2007.243	1497.005	-0.08185	2734.243	1904.384
YNL178W	60456.9	48054.68	53722.63	57167.9	54999.84	54193.54	0.036242	62917.94	51713.39
YHR177W	135.8841	152.2814	201.5636	158.4913	178.6008	203.5144	0.142595	156.2746	201.0622
YNL199C	1316.233	1470.406	1276.569	1433.585	1491.029	1453.954	0.10784	1334.517	1186.179
YCL005W-/	101.3373	78.11839	74.10426	88.64767	87.57202	87.08069	0.054383	91.06648	79.8981
YNL216W	1575.334	1501.06	1688.589	1567.004	1645.432	2310.084	0.212856	1425.584	1463.628
YDR003W	662.147	677.3557	649.1533	614.2657	557.6955	516.6135	-0.23599	634.0925	560.1647
YGR137W	147.3997	92.951	104.734	111.9289	99.09466	68.49043	-0.30403	143.9075	122.9202
YGL112C	1249.443	1251.872	1334.865	1331.506	1404.609	1440.256	0.12258	1242.327	1237.104
YLR154W-/	1.15156	0	0.988057	0	0	0	#NUM!	0	1.756002
YLR048W	32561.51	29915.39	32910.2	34172.33	34422.72	33378.32	0.096327	39536.34	34697.72
YOR120W	1148.105	1247.917	1320.044	1168.537	1116.543	996.0465	-0.17958	1156.882	1299.442

YDR332W	381.1663	428.1679	411.0316	461.147	386.0083	424.6407	0.059553	308.052	362.6144
YNR057C	134.7325	148.3261	183.7786	189.8314	149.7943	155.5711	0.085082	143.9075	151.0162
YDR246W-/	41.45616	35.59826	51.37895	42.98069	43.78601	37.18052	-0.05129	40.47399	40.38805
YNR021W	4559.026	3987.993	3894.92	4454.769	4486.914	3865.796	0.041775	4501.607	4029.147
YIL128W	794.5764	929.51	922.8451	1038.7	1072.757	1275.879	0.35583	966.8786	1121.207
YKL198C	1169.985	1687.951	1130.337	1124.661	1106.173	868.85	-0.36364	915.1619	969.3132
YOR345C	3.45468	0.98884	4.940284	0.895431	3.45679	4.892174	-0.02159	1.124277	1.756002
YLR244C	4441.567	4313.322	4267.417	5361.841	4846.42	4797.265	0.204509	4536.46	4314.497
YLR061W	219.9479	410.3688	275.6679	302.6557	245.4321	217.2125	-0.24346	212.4884	280.9603
YKL208W	298.254	410.3688	303.3334	326.8323	273.0864	264.1774	-0.22788	345.1532	378.4185
YNL108C	320.1337	341.1499	296.417	245.3481	382.5515	262.2205	-0.10557	355.2717	359.1024
YGR269W	16.12184	26.69869	22.72531	13.43147	9.218108	33.26678	-0.22923	13.49133	11.41401
YLR077W	719.725	979.9409	660.022	779.025	792.7573	780.7909	-0.00436	764.5087	672.5488
YBR233W	499.777	431.1344	463.3987	445.9247	473.5803	417.7916	-0.06023	376.633	388.9545
YOR023C	880.9434	1016.528	980.1524	918.7122	919.5062	1006.809	-0.01644	930.9018	1067.649
YAL056C-/	0	0	0	0.895431	4.609054	1.956869	#DIV/0!	2.248555	0.878001
YHL010C	327.043	295.6633	303.3334	291.0151	246.5844	322.8835	-0.10593	228.2283	309.0564
YLR044C	211779.9	219709.5	202810.5	223897.2	219295.3	199840.4	0.019727	237329.4	220703.1
YGL212W	172.734	228.4221	196.6233	154.9096	192.428	259.2852	0.021186	170.8902	191.4042
YBR258C	130.1263	154.2591	146.2324	147.7461	116.3786	126.2181	-0.14167	181.0087	152.7722
YJL074C	679.4204	535.9515	705.4726	725.2991	709.7943	1034.205	0.362361	661.0752	842.003
YDR383C	507.8379	389.6031	424.8644	455.7744	429.7943	370.8268	-0.07376	545.2746	445.1465
YDR118W	230.312	238.3105	280.6081	248.0344	285.7613	266.1342	0.094464	161.896	233.5483
YOL003C	412.2585	432.1233	414.9839	411.8983	445.926	444.2094	0.04807	530.659	443.3905
YBR027C	8.06092	13.84377	3.952227	3.581724	5.761317	2.935304	-1.07443	3.372832	1.756002
YJR128W	4.60624	0	0	0	4.609054	1.956869	0.511408	0	1.756002
YKL056C	40325.33	33526.63	37564.93	40873.74	39360.17	36927.1	0.072525	51270.43	41079.04
YNL017C	2.30312	7.910723	1.976114	4.477155	2.304527	2.935304	-0.32711	0	6.146008
YOL002C	1662.853	2126.007	1552.237	1960.994	1849.383	1620.288	0.023993	1716.772	1479.432
YGL130W	1159.621	1198.475	1185.668	1150.629	1151.111	1130.092	-0.0463	1087.176	981.6052
YMR056C	120.9138	229.411	165.9935	119.9878	97.94239	75.33947	-0.81603	110.1792	107.9941
YDR024W	24.18276	14.83261	16.79697	19.69948	16.13169	14.67652	-0.14408	22.48555	10.53601
YLL057C	64.48736	90.97332	97.81763	95.81112	86.41976	45.98643	-0.15032	136.0376	84.2881
YPL031C	2424.034	2099.308	2278.459	2303.944	2021.07	1898.163	-0.12827	2314.887	1777.952
YDL118W	3.45468	8.899564	1.976114	7.163448	5.761317	7.827478	0.534193	4.49711	2.634003
YJR084W	764.6358	688.2329	709.4248	735.1489	762.7984	658.4866	-0.00391	749.8931	684.8408
YPR152C	370.8023	413.3353	334.9513	419.9572	412.5103	420.7269	0.163286	372.1358	420.5625
YER093C	621.8424	625.936	692.6278	696.6453	706.3375	891.354	0.241719	643.0867	748.9349
YGL190C	1606.426	1633.564	1679.697	1532.082	1582.058	1682.908	-0.03642	1617.835	1792
YNL051W	476.7458	398.5027	496.9926	511.2911	407.9013	407.0288	-0.04921	492.4335	434.6105
YHR199C	1552.303	1979.659	1669.816	1694.156	1464.527	1233.806	-0.24397	1422.211	1447.824
YNL116W	804.9404	808.8715	852.693	717.2403	786.9959	755.3516	-0.12641	666.6965	634.7948
YML105C	2145.356	2171.494	1848.654	2163.361	2053.334	2082.109	0.030859	2434.061	2288.071
YNR051C	2395.245	2144.795	2687.515	2625.404	2401.317	2483.267	0.055303	2177.725	2791.165
YPL038W	467.5333	446.9559	432.7689	456.6698	474.7325	375.7189	-0.04363	558.7659	475.8766
YIL125W	2258.209	2111.174	2385.169	2101.577	2091.358	2331.61	-0.04998	2456.546	2382.895
YPL013C	2052.08	1464.473	1910.902	1861.601	2047.572	1837.5	0.082451	2173.228	1719.126
YLR284C	279.8291	270.9423	273.6917	236.3938	225.8436	216.2341	-0.28117	266.4538	257.2543
YDR248C	685.1782	672.4115	659.0339	665.3053	713.2511	784.7046	0.101266	636.3411	587.3827
YPL094C	4600.482	4745.445	4521.348	4952.629	4705.844	4414.697	0.021263	4949.069	4495.365
YKR066C	4112.221	5293.263	4250.62	4536.254	4190.782	3473.443	-0.16261	4199.176	3879.009
YNR054C	1456.723	1571.267	1588.795	1815.039	1909.301	1591.913	0.203521	1609.965	1559.33
YDR353W	8993.683	8211.331	8582.262	9273.979	9063.704	8502.598	0.05774	8703.032	8119.754
YLR066W	1386.478	1250.883	1341.781	1183.76	1221.399	1236.741	-0.12777	1550.379	1216.909

YNR032W	559.6581	661.5342	614.5713	511.2911	548.4774	447.1447	-0.28478	513.7948	525.0446
YGR235C	1431.389	1348.778	1407.981	1318.074	1235.226	1265.116	-0.13334	1588.604	1361.78
YBR085W	1117.013	1181.664	1011.77	1196.296	1272.099	903.0952	0.02636	1138.893	982.4832
YMR120C	4644.241	6207.94	5168.525	5318.86	5771.688	5532.07	0.05321	4747.824	4642.87
YGL069C	0	0	0	0	0	0	#DIV/0!	1.124277	0
YLR162W	0	0	0	0	0	0	#DIV/0!	1.124277	0
YNL235C	0	0	0	0	0	0	#DIV/0!	1.124277	0
YNL319W	0	0	0	0	0	0	#DIV/0!	1.124277	0
YPL038W-/	1.15156	8.899564	1.976114	5.372586	3.45679	1.956869	-0.15711	4.49711	4.390005
YOL080C	946.5823	797.0054	667.9264	929.4574	999.0124	908.9658	0.234648	953.3873	821.809
YIR026C	1718.127	1746.292	1739.968	2084.563	2027.984	1759.226	0.174068	2272.165	2029.938
YNL046W	711.664	802.9384	659.0339	657.2464	633.7449	516.6135	-0.26603	623.974	518.0206
YBL065W	0	0	0	0	0	0	#DIV/0!	0	0.878001
YHR180W-/	0	0	0	0	0	0	#DIV/0!	0	0.878001
YIL172C	0	0	0	0	0	0	#DIV/0!	0	0.878001
YOL079W	0	0	0	0	0	0	#DIV/0!	0	0.878001
YOL162W	0	0	0	0	0	0	#DIV/0!	0	0.878001
YPL281C	0	0	0	0	0	0	#DIV/0!	0	0.878001
YKL053C-/	982.2806	1017.517	937.6659	868.5681	920.6585	785.6831	-0.19005	1197.356	906.9751
YKR088C	1023.737	852.3805	902.0959	955.4249	982.8807	764.1575	-0.03988	973.6243	719.0829
YDR525W	18.42496	7.910723	5.928341	11.6406	13.82716	8.805912	0.087175	12.36705	2.634003
YLR092W	490.5645	494.4202	459.4464	478.1602	433.2511	434.425	-0.102	332.7861	367.0044
YFR056C	2.30312	5.933043	8.892511	6.268017	6.913581	7.827478	0.294599	7.869942	9.658012
YGL213C	423.7741	514.197	451.542	442.3429	456.2963	472.584	-0.01912	557.6416	440.7565
YGR173W	1666.307	1736.404	1788.383	2089.041	1994.568	1942.193	0.215115	1887.662	1714.736
YLR285W	1767.645	1521.825	1779.49	1760.417	1769.877	1721.067	0.051001	1706.653	1478.554
YDR025W	34976.33	27684.57	31054.63	34394.4	33749.8	30358.87	0.071881	36189.37	31529.02
YPL173W	1287.444	1149.033	1364.506	1130.929	1171.852	1119.329	-0.15149	1299.665	1194.959
YLR020C	1168.833	1298.347	1273.605	1283.153	1330.864	1453.954	0.120968	1241.202	1360.024
YOR077W	135.8841	154.2591	113.6265	129.8375	109.465	121.3259	-0.16302	101.185	172.0882
YBR284W	86.367	74.16303	80.0326	63.5756	89.87655	65.55513	-0.13543	65.20809	101.8481
YBR114W	707.0578	778.2174	766.7321	796.0382	579.5885	895.2678	0.012049	568.8844	677.8168
YLR016C	696.6938	708.9986	543.4313	534.5723	618.7655	531.29	-0.2104	650.9567	613.7228
YDL185C-/	0	0	0	0.895431	0	2.935304	#DIV/0!	1.124277	0
YKR089C	1827.526	1740.359	1582.867	1433.585	1489.877	1505.811	-0.21771	1495.289	1668.202
YBR232C	0	0	0	0	0	0.978435	#DIV/0!	0	0
YKL106C-/	0	0	1.976114	2.686293	3.45679	2.935304	2.19977	1.124277	1.756002
YIL073C	142.7934	155.2479	169.9458	145.0598	101.3992	158.5064	-0.20867	130.4162	102.7261
YBR135W	1585.698	1506.004	1641.162	1668.188	1686.914	1438.299	0.018336	1572.864	1690.152
YDL068W	0	0	0	1.790862	0	0	#DIV/0!	2.248555	0.878001
YBR021W	697.8453	940.3873	772.6604	664.4098	859.5885	636.961	-0.1579	612.7312	689.2308
YGL041C-I	2.30312	3.955362	4.940284	7.163448	4.609054	0.978435	0.187264	5.621387	0.878001
YDR158W	6102.116	5647.268	6037.027	5771.948	5731.358	5533.048	-0.06216	5958.671	5501.555
YHR021C	13178.45	12127.14	11674.88	11824.17	13260.25	13021.99	0.04327	17222.81	12349.09
YGR126W	46.0624	72.18535	64.22369	51.935	58.76544	91.97286	0.151485	46.09538	60.58207
YDL028C	685.1782	652.6347	776.6127	686.7956	791.605	883.5265	0.159699	692.5549	900.8291
YJL068C	1244.836	986.8628	1169.859	1077.204	1066.996	918.7502	-0.15127	1295.168	1151.059
YDR086C	5790.043	4712.814	5342.423	5450.489	5701.4	5440.097	0.066433	5982.28	5403.219
YLR316C	367.3476	325.3285	308.2737	329.5186	307.6543	239.7165	-0.1909	279.9451	268.6683
YLR283W	270.6166	294.6744	240.0978	233.7075	223.5391	241.6734	-0.20456	306.9278	263.4003
YLR269C	17.2734	10.87724	10.86863	11.6406	14.97943	15.65496	0.115617	14.61561	14.92602
YDR215C	6.90936	3.955362	2.96417	7.163448	0	3.913739	-0.32009	7.869942	14.04802
YEL037C	5779.679	5742.196	5362.184	6100.572	5831.605	5667.094	0.059854	5927.191	5757.053
YNL105W	0	0	0	0.895431	0	0	#DIV/0!	0	0

YKL149C	260.2525	362.9044	284.5604	366.2313	313.4157	311.1422	0.126335	317.0462	281.8383
YGL019W	2131.537	2238.735	2021.564	2224.251	2060.247	2014.597	-0.02109	2385.717	2282.803
YML116W-/	48.36552	41.5313	38.53422	41.18983	44.93828	35.22365	-0.0818	48.34393	31.60804
YPL156C	426.0772	421.246	303.3334	333.1003	266.1729	250.4793	-0.43734	424.9769	388.0765
YJL063C	2506.946	2502.755	2605.506	2517.057	2352.922	2159.405	-0.11548	2879.275	2389.041
YGL234W	5857.985	8177.71	6927.266	6925.264	7108.313	7137.681	0.014264	5450.497	6499.842
YBR194W	344.3164	305.5517	312.226	346.5318	315.7202	276.897	-0.03482	347.4017	291.4964
YGR242W	2.30312	0.98884	6.916398	1.790862	2.304527	3.913739	-0.35003	0	3.512004
YEL066W	639.1158	547.8176	472.2912	537.2586	578.4363	442.2525	-0.09086	568.8844	561.0427
YHR071W	3751.782	3516.317	3242.803	3067.747	3048.889	2376.618	-0.3075	3458.278	2828.919
YOL073C	785.3639	737.675	708.4367	731.5671	737.4486	561.6215	-0.13607	745.396	654.1108
YBR261C	1773.402	1823.422	1966.233	2075.609	1991.111	1634.964	0.03551	2218.199	1866.63
YDR491C	12.66716	29.66521	18.77308	20.59491	29.95885	22.504	0.257736	21.36127	16.68202
YBR182C-/	16.12184	39.55362	27.66559	21.49034	19.58848	17.61182	-0.5059	20.23699	13.17002
YHL008C	336.2555	237.3217	231.2053	247.139	299.5885	238.7381	-0.03505	332.7861	278.3263
YDR400W	1309.324	1354.711	1342.769	1366.428	1419.589	1384.485	0.057769	1258.066	1144.913
YGR267C	2498.885	2345.53	2567.96	2532.279	2506.173	2591.874	0.041809	2767.971	2597.127
YPL119C-/	36.84992	40.54246	31.61782	22.38578	24.19753	30.33148	-0.50313	43.84682	35.99804
YER028C	84.06388	65.26347	68.17592	73.42534	78.35392	67.512	0.011811	74.20231	56.19207
YDR505C	760.0296	703.0655	890.2392	732.4626	872.2634	1225.979	0.266455	751.0174	1010.579
YDR396W	11.5156	5.933043	11.85668	12.53603	9.218108	2.935304	-0.24727	14.61561	15.80402
YMR013C	207.2808	206.6677	165.9935	263.2567	192.428	193.7301	0.163232	182.133	162.4302
YLR003C	1522.362	1489.194	1586.819	1749.672	1839.012	1751.398	0.215738	1552.627	1680.494
YOL042W	436.4412	423.2237	461.4225	374.2902	425.1852	379.6327	-0.16403	415.9827	444.2685
YKL113C	1586.85	1583.134	1666.852	1918.013	1719.177	1680.951	0.136859	1553.751	1450.458
YIL046W	1378.417	1953.949	1637.21	1563.423	1533.663	1551.797	-0.09624	1281.676	1295.052
YDR169C-/	4.60624	0.98884	3.952227	1.790862	3.45679	8.805912	0.55777	7.869942	8.780011
YDR167W	1626.003	1431.841	1555.201	1560.736	1472.593	1515.595	-0.02019	1793.223	1638.35
YHL007C	2440.156	2287.188	2718.144	2635.254	2829.959	3556.61	0.277053	2097.902	2548.837
YLR281C	3.45468	11.86609	5.928341	4.477155	6.913581	3.913739	-0.47345	7.869942	11.41401
YER002W	2898.476	2897.302	2811.022	3349.807	3374.98	2987.161	0.174284	3058.035	3014.178
YOL025W	193.4621	231.3887	232.1934	207.74	176.2963	214.2772	-0.13509	161.896	244.0843
YLR226W	442.199	493.4314	490.0762	474.5784	500.0823	538.1391	0.085544	456.4567	499.5826
YPR190C	1950.743	2168.527	2071.955	2327.225	2456.626	2475.44	0.229604	2237.312	2244.171
YIL052C	24920.91	19507.84	21707.61	23462.08	23204.28	22118.5	0.056647	27547.05	22474.19
YLR384C	1977.228	1995.48	2209.295	2453.481	2314.897	2735.703	0.279601	1919.142	2315.289
YER078C	793.4248	814.8045	827.9916	762.0118	775.4733	747.5241	-0.09244	833.0896	799.859
YDL213C	2462.035	2190.282	2531.402	2903.883	2717.037	2347.265	0.14952	2920.873	2766.581
YNR059W	367.3476	472.6657	463.3987	400.2577	367.572	375.7189	-0.18878	403.6156	423.1965
YBR176W	564.2644	645.7128	564.1804	555.1672	667.1605	521.5057	-0.02487	563.263	535.5807
YMR114C	272.9197	262.0427	242.0739	221.1715	248.8889	226.9969	-0.1567	342.9046	243.2063
YJR030C	415.7131	354.0049	448.5778	393.9897	545.0206	602.7158	0.339682	441.841	517.1426
YNL114C	2.30312	0	0	0	1.152263	0.978435	-0.11226	0	0
YFL038C	9152.598	7787.118	8189.015	8401.829	8385.021	7784.427	-0.03237	8593.977	8140.826
YPR159C-/	5.7578	9.888404	1.976114	1.790862	9.218108	0	-0.67872	11.24277	0.878001
YPR091C	1178.046	1023.45	1245.94	1218.682	1133.827	1348.283	0.102311	1249.072	1280.126
YDL075W	28822.39	22147.06	25409.86	26308.66	27381.24	25875.68	0.058963	31351.6	26640.31
YCR108C	0	1.977681	0	1.790862	0	1.956869	0.922208	6.745665	0
YDR160W	522.8082	599.2373	562.2043	476.3693	595.7202	740.6751	0.106085	526.1619	589.1387
YMR213W	558.5066	510.2417	497.9806	556.9581	599.177	558.6862	0.130303	468.8237	718.2049
YML050W	234.9182	223.4779	180.8144	173.7136	197.0371	166.3339	-0.25114	170.8902	241.4503
YEL014C	23.0312	37.57594	26.67753	22.38578	24.19753	22.504	-0.33731	17.98844	19.31602
YBL014C	742.7562	773.2732	757.8396	923.1894	987.4898	1096.825	0.403418	852.2023	1070.283
YPL174C	544.6879	451.9001	567.1446	529.1997	534.6502	609.5648	0.097801	451.9595	561.9207

YGL146C	123.2169	106.7948	97.81763	69.84362	80.65844	88.05912	-0.45859	87.69364	54.43607
YOL129W	2092.384	1984.603	2135.191	1826.679	2102.881	1761.182	-0.12648	2179.974	1913.164
YDL050C	9.21248	5.933043	8.892511	11.6406	4.609054	2.935304	-0.32534	11.24277	3.512004
YFR045W	441.0475	417.2907	379.4138	325.0415	342.2223	335.6031	-0.30359	371.0116	361.7364
YER014W	447.9568	638.7909	614.5713	569.4941	564.6091	523.4626	-0.03759	428.3497	512.7526
YPL266W	2200.631	2199.181	2358.492	2752.555	2729.712	2617.313	0.261186	2640.928	2339.873
YOR156C	294.7993	213.5895	296.417	249.8253	248.8889	329.7325	0.041768	281.0694	278.3263
YCR002C	1925.408	1982.625	1760.717	1846.379	1916.214	1731.829	-0.04506	2092.28	1854.338
YPR101W	636.8126	548.8064	466.3628	448.6109	473.5803	418.7701	-0.30093	543.026	540.8487
YOR112W	867.1246	887.9787	937.6659	847.0778	979.4239	973.5425	0.056359	804.9827	806.883
YMR217W	12328.6	12277.44	12336.88	13926.64	13571.36	13850.72	0.162545	14629.1	13842.56
YIL104C	666.7532	786.1281	658.0458	742.3123	842.3046	902.1168	0.236375	847.7052	767.3729
YIL018W	68848.31	59657.73	62909.58	67687.42	64839.02	61414.39	0.018908	73101.65	61588.26
YAL022C	766.9389	866.2242	744.9948	807.6788	781.2346	670.2278	-0.07407	919.659	786.689
YPL076W	266.0103	312.4736	282.5843	265.943	266.1729	251.4577	-0.13606	256.3353	214.2323
YKL151C	2861.626	2864.671	2774.464	2479.449	2413.992	2306.171	-0.23967	3008.567	2569.909
YOR258W	206.1292	192.8239	172.9099	146.8507	133.6626	165.3555	-0.35905	167.5173	155.4062
YFR051C	4708.729	4801.809	4554.942	4490.587	4749.63	4392.193	-0.04512	4605.041	4634.09
YAL037W	69.0936	72.18535	66.19981	62.68017	62.22223	63.59826	-0.13839	79.8237	52.68006
YGR231C	2700.408	2617.461	2434.572	2713.156	2484.28	2129.074	-0.08152	2984.957	2677.025
YLR458W	2.30312	1.977681	0.988057	0.895431	0	1.956869	-0.88536	1.124277	0
YOR278W	446.8053	461.7885	465.3748	445.9247	410.2058	408.9857	-0.11908	436.2197	357.3464
YOR175C	1616.79	1783.868	1703.41	1667.293	1776.79	1826.738	0.04638	1638.072	1716.492
YGL233W	908.5808	716.9093	938.654	817.5285	898.7655	1139.876	0.155605	962.3815	1023.749
YOR105W	8.06092	18.78797	16.79697	16.11776	12.6749	14.67652	-0.00585	16.86416	14.92602
YOR118W	1348.477	1322.08	1440.587	1601.926	1527.901	1604.633	0.20366	1588.604	1602.352
YMR304C-/	44.91084	30.65405	26.67753	73.42534	53.00412	40.11582	0.703921	56.21387	51.80206
YMR255W	1429.086	1217.263	1319.056	1184.655	1144.198	1078.235	-0.21893	1443.572	1288.906
YER006W	3848.513	4152.141	4151.815	5074.408	4798.025	4862.821	0.278022	4316.101	4430.393
YLL066C	5.7578	4.944202	3.952227	5.372586	3.45679	6.849043	0.097463	1.124277	1.756002
YDR095C	2.30312	1.977681	0	0	0	0.978435	-2.12933	4.49711	0.878001
YGL123W	51907.72	42566.61	46630.35	50441.42	47858.11	44334.83	0.015556	56318.43	46167.05
YLR146W-/	138.1872	143.3819	92.87734	128.9421	156.7078	112.52	0.088625	116.9249	97.45812
YJR118C	773.8483	769.3179	928.7734	921.3985	858.4363	790.5752	0.056355	912.9133	815.663
YJL206C	193.4621	205.6788	193.6591	196.9948	218.9301	193.7301	0.040447	175.3873	186.1362
YGR181W	1032.949	821.7264	894.1914	924.9802	799.6708	845.3676	-0.09706	1263.688	1027.261
YPL237W	3372.919	2957.622	3146.961	3473.377	3484.445	3313.958	0.116107	3951.835	3350.452
YML116W	295.9509	282.8084	252.9425	276.6882	248.8889	260.2636	-0.08183	258.5838	244.0843
YCL001W-I	28.789	45.48666	18.77308	39.39897	21.89301	39.13739	0.110123	41.59827	24.58403
YOL027C	2002.563	2134.906	2136.179	2067.55	2051.029	2046.885	-0.02509	2111.393	2183.589
YBL063W	373.1054	315.4401	352.7363	342.0547	413.6626	377.6758	0.122288	237.2225	396.8565
YDR191W	198.0683	199.7458	242.0739	177.2953	216.6255	207.4282	-0.08962	166.3931	172.0882
YDR231C	456.0177	402.4581	484.1478	436.9703	342.2223	350.2796	-0.24941	458.7052	459.1946
YMR247W-/	13.81872	14.83261	13.8328	32.23552	8.065844	2.935304	0.025332	8.99422	8.780011
YGL167C	2369.91	2226.869	2564.007	2383.637	2389.794	3124.142	0.141291	2625.188	2687.561
YOR244W	482.5036	576.494	544.4193	462.9378	479.3416	457.9074	-0.19553	559.8902	604.9427
YER091C-/	0	12.85493	3.952227	5.372586	0	0.978435	-1.40401	2.248555	0.878001
YGL231C	1837.89	1755.192	1913.866	1915.327	1824.033	1885.444	0.03055	1937.13	1752.49
YBR227C	725.4828	824.6929	723.2576	757.5346	752.428	787.6399	0.015257	824.0954	803.371
YNR011C	621.8424	573.5275	579.9894	597.2525	625.6791	785.6831	0.17809	639.7139	643.5748
YGR154C	241.8276	298.6298	251.9545	222.9623	239.6708	190.7948	-0.27822	229.3526	188.7702
YBR127C	15734.92	14356.97	15805.95	16563.68	16071.77	16848.65	0.108539	17442.04	15761.88
YOR048C	1713.521	1740.359	1910.902	1897.418	1992.264	2432.389	0.236878	1924.763	2176.565
YLL005C	321.2852	264.0204	278.632	273.1065	294.9794	348.3228	0.085064	377.7572	280.9603

YCR087W	0	2.966521	0	2.686293	0	0	-0.14316	1.124277	0
YER047C	348.9227	414.3241	491.0642	386.8262	462.0577	565.5353	0.173315	356.396	542.6047
YCR042C	727.7859	861.28	786.4932	787.0839	861.8931	950.0601	0.12971	770.1301	866.5871
YKR009C	207.2808	245.2324	310.2498	211.3217	225.8436	221.1262	-0.21251	276.5723	256.3763
YLR289W	285.5869	316.4289	315.1901	246.2435	265.0206	294.5088	-0.18687	259.7081	324.8604
YOR293W	23953.6	19488.07	21638.44	23232.85	24477.53	22466.82	0.108786	26567.8	22804.32
YDL186W	104.792	139.4265	94.85346	99.39284	48.39507	67.512	-0.65524	62.95954	93.94611
YPL101W	1365.75	1450.629	1406.993	1421.944	1413.827	1471.566	0.028401	1374.991	1288.906
YOR020W-A	855.609	719.8758	792.4216	726.1946	768.5597	656.5297	-0.13841	858.948	752.4469
YDR406W-A	0	0	0	0	1.152263	0	#DIV/0!	0	0
YJR023C	0	0	0	0	1.152263	0	#DIV/0!	0	0
YEL003W	1133.135	1063.003	1159.979	1211.518	1272.099	1116.394	0.101208	1211.971	1280.126
YHR119W	1270.171	1077.836	1291.39	1188.237	1340.082	1585.064	0.176626	1234.457	1121.207
YIR020C-B	0	0	0	0	0	0.978435	#DIV/0!	0	0
YNL338W	0	0	0	0	0	0.978435	#DIV/0!	0	0
YOR072W-B	0	0	0	0	0	0.978435	#DIV/0!	0	0
YGR293C	0	0	0	0.895431	0	0	#DIV/0!	0	0
YOR346W	542.3847	389.6031	466.3628	591.8799	651.0289	593.9099	0.393483	494.6821	553.1407
YOR073W	598.8112	490.4649	568.1327	578.4484	639.5062	718.1711	0.224243	539.6532	630.4048
YOL038C-A	32.24368	23.73217	9.880568	24.17664	20.74074	19.56869	-0.03034	28.10694	29.85204
YLR065C	1462.481	1293.403	1539.393	1387.918	1481.811	1312.081	-0.03862	1586.356	1399.534
YOR144C	502.0801	475.6322	575.0491	440.5521	566.9136	604.6727	0.054139	454.2081	456.5606
YBL013W	33.39524	53.39738	55.33118	34.02638	35.72017	38.15895	-0.39738	32.60405	29.85204
YHR050W-A	34.5468	17.79913	13.8328	30.44465	24.19753	26.41774	0.292621	13.49133	28.97404
YHR118C	418.0163	469.6992	456.4823	463.8333	474.7325	579.2334	0.175236	430.5983	554.0187
YPL111W	6532.8	5891.511	6503.39	5077.989	4561.811	4540.916	-0.41657	6510.691	6050.305
YGR047C	457.1693	502.3309	592.8341	548.8992	555.391	684.9043	0.204871	476.6937	586.5047
YPR102C	27434.76	20224.75	25450.37	26897.85	25332.51	25096.85	0.08091	32590.56	27544.65
YKL042W	736.9984	640.7686	741.0426	693.0636	678.6832	801.338	0.036491	650.9567	725.2289
YDL027C	374.257	281.8195	278.632	289.2242	270.7819	228.9537	-0.24456	278.8208	358.2244
YDR073W	1083.618	981.9186	1038.448	851.5549	907.9836	676.0984	-0.34982	857.8237	810.395
YLR291C	2070.505	2071.621	2097.645	2316.48	2313.745	2044.929	0.097308	2139.5	1943.016
YGR041W	306.3149	371.804	342.8557	389.5125	331.8519	347.3443	0.065922	359.7688	357.3464
YEL021W	0	1.977681	0.988057	0	1.152263	0	-1.36392	3.372832	0
YGR259C	0	0	0.988057	0	0	0	#NUM!	1.124277	0
YDR164C	932.7636	838.5367	956.439	847.9732	1033.58	1067.472	0.112533	880.3093	777.031
YLR451W	912.0355	919.6216	871.4661	924.9802	920.6585	1090.955	0.119516	771.2544	888.5371
YBL100C	72.54828	156.2368	112.6385	80.58879	117.5309	137.9593	-0.02276	95.56359	94.82412
YJL052C-A	0	6.921883	0.988057	5.372586	2.304527	0	-0.0431	2.248555	0
YJR143C	4197.436	4587.231	4427.483	4563.117	4549.136	4633.867	0.057159	4662.379	4630.578
YGR211W	4425.445	4857.184	4817.765	5140.67	4846.42	5174.941	0.104728	5344.815	5518.237
YOR022C	337.4071	305.5517	329.0229	260.5704	278.8478	301.3579	-0.20921	242.8439	299.3984
YMR244C-A	19.57652	17.79913	21.73725	19.69948	5.761317	13.69809	-0.59413	13.49133	12.29202
YPL055C	1176.894	1439.752	1182.704	1188.237	1066.996	1029.313	-0.21006	1140.017	1286.272
YHR126C	2.30312	1.977681	3.952227	0	2.304527	0	-1.83695	2.248555	1.756002
YHL019C	158.9153	226.4445	243.062	213.1126	224.6914	206.4497	0.035896	225.9798	196.6722
YOR386W	333.9524	292.6968	440.6733	287.4334	293.8272	370.8268	-0.16483	372.1358	349.4444
YDR292C	2155.72	2030.089	2302.172	2214.401	2331.029	2826.698	0.184311	2267.668	2334.605
YJR041C	1211.441	1185.62	1362.53	1406.722	1488.724	1675.08	0.281785	1281.676	1305.588
YLR087C	817.6076	693.1771	681.7592	831.8554	819.2593	809.1655	0.166217	673.4422	820.931
YNL299W	913.187	903.8002	1025.603	975.1244	1045.103	1166.294	0.164776	822.9711	927.1691
YIL091C	2101.597	2086.453	2402.954	2459.749	2664.033	3388.319	0.369017	2210.33	2463.671
YMR001C	915.4902	1077.836	1057.221	1124.661	1277.86	1340.456	0.295118	863.4451	1062.381
YPL192C	104.792	138.4377	113.6265	132.5238	172.8395	125.2396	0.271016	87.69364	154.5282

YBR298C-/	0	0	0	0	0	0	#DIV/0!	0	0
YCR040W	0	0	0	0	0	0	#DIV/0!	0	0
YDR269C	0	0	0	0	0	0	#DIV/0!	0	0
YOR282W	0	0	0	0	0	0	#DIV/0!	0	0
YJL033W	2901.931	3086.171	3006.657	3452.782	3425.679	3998.863	0.274167	3264.902	3441.764
YGL199C	0	0	0	0	0	0	#DIV/0!	0	0
YHL019W-/	0	0	0	0	0	0	#DIV/0!	0	0
YHR218W-/	0	0	0	0	0	0	#DIV/0!	0	0
YJL032W	0	0	0	0	0	0	#DIV/0!	0	0
YJL222W-I	0	0	0	0	0	0	#DIV/0!	0	0
YKL177W	0	0	0	0	0	0	#DIV/0!	0	0
YLL065W	0	0	0	0	0	0	#DIV/0!	0	0
YOL050C	0	0	0	0	0	0	#DIV/0!	0	0
YNL151C	1706.612	1626.643	1728.111	2020.988	2106.338	1792.492	0.226026	2094.529	1922.822
YER144C	648.3282	692.1883	624.4519	603.5205	603.7861	618.3707	-0.10607	568.8844	700.6449
YKL121W	428.3803	345.1053	449.5659	380.5582	346.8313	485.3036	-0.01227	382.2543	443.3905
Q0105	2.30312	5.933043	1.976114	0	1.152263	0	-3.14776	4.49711	0
YFL004W	2912.295	2533.409	2629.219	2710.47	2897.943	3317.872	0.144611	2721.876	2501.425
YOR299W	1120.468	1009.606	1188.632	1186.446	1094.65	1392.313	0.146499	1078.182	1230.958
YLR421C	1982.986	1877.808	1902.997	1798.026	1792.922	1743.571	-0.11166	2193.465	1986.038
YKL005C	780.7576	765.3625	919.8809	819.3194	879.177	958.866	0.107822	713.9162	845.515
YLR442C	663.2985	570.5609	716.3412	612.4748	660.247	796.4459	0.085428	611.6069	702.4009
YPR081C	512.4442	564.6279	531.5746	512.1865	490.8642	472.584	-0.12451	523.9133	507.4846
YDL104C	201.523	239.2994	215.3964	215.7989	225.8436	154.5927	-0.1383	233.8497	212.4763
YCR038C	154.309	154.2591	169.9458	115.5106	142.8807	129.1534	-0.3042	155.1503	154.5282
YNL137C	1779.16	1792.768	1807.156	1890.255	1913.91	2007.748	0.111653	2027.072	1873.654
YOL135C	1069.799	1020.483	966.3196	924.9802	796.2141	769.0497	-0.29564	888.1792	849.905
YJL120W	4.60624	6.921883	16.79697	8.95431	9.218108	5.870608	-0.23646	14.61561	14.92602
YDR348C	1225.26	1301.314	1241.987	1327.029	1205.268	1454.932	0.081373	1106.289	1308.222
YBR136W	381.1663	372.7928	336.9274	363.545	346.8313	362.9993	-0.02335	373.2601	403.0025
YOR310C	5733.617	6778.501	6564.65	6701.406	7094.486	7002.657	0.124666	6895.194	7140.783
YPR123C	57.578	94.92868	93.8654	83.27509	95.63787	90.01599	0.126386	52.84104	91.31211
YNL286W	681.7235	679.3334	681.7592	730.6717	702.8807	595.8667	-0.00949	742.0231	585.6267
YDR525W-/	345.468	367.8486	309.2618	230.1258	274.2387	209.385	-0.51872	306.9278	240.5723
YLR075W	14971.43	13489.76	15660.7	15773.91	15321.65	15298.81	0.072455	17964.83	15896.21
YAL030W	1055.98	770.3067	874.4303	831.8554	781.2346	929.513	-0.08704	1196.231	1077.307
YIL134W	507.8379	515.1859	447.5897	419.0617	411.3581	415.8348	-0.23882	491.3093	440.7565
YEL001C	3721.842	3186.044	3388.047	3641.718	3411.852	3199.482	-0.00602	3476.266	3273.188
YAL055W	365.0445	319.3955	326.0588	281.1653	311.1111	244.6087	-0.27197	278.8208	327.4944
YMR071C	1531.575	1240.995	1326.96	1158.688	1349.301	1122.265	-0.17539	1292.919	1221.299
YHR024C	1289.747	1533.692	1369.447	1430.899	1471.44	1420.687	0.044098	1387.358	1304.71
YLR154W-(	2.30312	5.933043	5.928341	0.895431	3.45679	5.870608	-0.47049	6.745665	3.512004
YKL184W	1044.465	1119.367	1025.603	987.6604	979.4239	974.521	-0.1167	1072.561	1049.211
YCR075W-/	229.1604	202.7123	224.2889	208.6354	254.6502	181.0104	-0.02633	238.3468	206.3303
YLR371W	874.034	886.001	1015.722	893.6402	952.9219	1271.965	0.167983	775.7515	997.4092
YDR263C	155.4606	112.7278	139.316	86.85681	147.4897	76.31791	-0.39146	122.5462	126.4322
YOR167C	14057.09	10675.52	12264.75	12697.21	13217.61	13407.49	0.087926	15707.28	13630.09
YNL229C	1491.27	1716.627	1517.655	1453.285	1511.77	1569.409	-0.05955	1450.318	1507.528
YBR191W	18201.56	15283.52	16882.93	17945.33	17747.16	15465.14	0.022442	19156.56	16639
YNL292W	1364.599	1507.982	1355.614	1607.299	1580.905	1634.964	0.18994	1554.876	1553.184
YMR146C	9546.432	9383.107	9222.522	10010.02	9405.927	8806.891	0.003623	10423.18	10048.72
YOR024W	0	1.977681	2.96417	3.581724	0	0	-0.4644	0	7.024009
YLR234W	152.0059	190.8462	168.9577	154.9096	135.9671	156.5496	-0.19396	67.45665	145.7482
YMR242W-/	5.7578	5.933043	0.988057	4.477155	1.152263	0.978435	-0.94018	1.124277	4.390005

YCR094W	851.0028	752.5076	825.0274	890.0584	851.5227	759.2653	0.042329	930.9018	887.6591
YPL129W	3060.846	2848.849	2839.675	3110.727	2780.412	2844.31	-0.0023	3044.543	2684.049
YJL152W	4.60624	9.888404	3.952227	8.95431	10.37037	5.870608	0.449778	10.1185	2.634003
YIL013C	446.8053	338.1834	453.5181	363.545	376.7902	479.433	-0.02199	254.0867	381.9305
YPL208W	756.5749	756.4629	878.3825	766.489	844.6091	783.7262	0.002052	793.7399	753.3249
YFR001W	2428.64	2024.156	2137.167	2406.919	2659.424	2314.977	0.163608	2851.168	2351.287
YLR102C	324.7399	303.574	317.1662	296.3877	261.5638	259.2852	-0.21029	300.1821	324.8604
YMR063W	54.12332	96.90636	108.6863	112.8243	114.0741	112.52	0.38613	69.7052	135.2122
YLR331C	4.60624	1.977681	5.928341	2.686293	5.761317	6.849043	0.289873	2.248555	8.780011
YEL018W	254.4947	248.1989	209.468	221.1715	260.4115	222.1047	-0.01727	305.8035	260.7663
YPL137C	2562.221	2321.797	2714.192	2274.395	2287.243	3122.185	0.016165	2431.812	2678.781
YNL260C	367.3476	362.9044	373.4855	373.3947	354.8971	333.6462	-0.0557	445.2139	398.6125
YLR059C	2086.627	1985.592	1992.911	2127.544	2074.074	1816.953	-0.01112	2148.494	2011.5
YOR207C	1807.949	1909.451	2028.481	2333.493	2233.087	2876.598	0.373391	2166.483	2358.311
YGL004C	613.7814	567.5944	593.8222	539.9449	603.7861	529.3332	-0.08549	648.7081	536.4587
YFL003C	66.79048	67.24115	47.42673	78.79793	71.44034	53.81391	0.1693	53.96532	50.04606
YCR057C	2740.713	3169.234	3120.283	3446.514	3505.185	4165.197	0.299919	2893.89	3250.36
YDR316W	647.1767	743.608	715.3531	764.6981	689.0536	638.9179	-0.00926	744.2717	721.7169
YDR437W	32.24368	74.16303	80.0326	59.99388	63.37449	72.40417	0.070472	49.46821	48.29006
YLR310C	1469.39	1344.823	1563.106	1395.082	1682.305	2041.015	0.225646	1452.567	1732.296
YOR034C-A	23.0312	23.73217	37.54616	25.07207	27.65432	24.46087	-0.12733	42.72254	21.07203
YOL065C	396.1366	469.6992	399.175	426.2252	397.5309	391.3739	-0.05804	449.711	365.2484
YDR170C	2671.619	2149.739	2715.18	2366.624	2412.84	3252.317	0.091818	2778.09	2802.579
YHL011C	4872.25	5312.051	5287.092	5676.137	5689.877	5467.493	0.121733	5074.989	4878.174
YDR314C	90.97324	119.6497	136.3518	95.81112	122.1399	153.6143	0.098785	122.5462	119.4081
YOR084W	753.1202	834.5813	691.6398	723.5083	730.535	654.5728	-0.11232	726.2833	698.0109
YPL017C	325.8915	401.4692	362.6169	282.0608	297.284	286.6814	-0.33182	251.8382	277.4483
YGL027C	1213.744	1163.865	1258.784	1326.133	1348.148	1462.76	0.186091	1319.902	1434.654
YPL035C	65.63892	75.15187	57.3073	76.11164	61.06996	78.27478	0.121181	76.45087	66.72808
YNL155W	1678.974	1486.227	1612.509	1478.357	1554.403	1425.579	-0.09981	1434.578	1309.1
YOL147C	895.9136	859.3023	789.4574	744.9986	811.1935	698.6024	-0.17448	816.2254	713.8149
YDL230W	522.8082	588.3601	545.4074	510.3957	526.5844	427.576	-0.17774	582.3757	518.8986
YOR326W	7077.487	4552.621	7328.417	6233.991	6930.865	8887.123	0.218061	7058.214	7815.966
YOR354C	1294.353	1476.339	1604.604	1442.539	1449.547	1510.703	0.009037	1437.951	1518.942
YJL192C	2470.096	2172.482	2209.295	2453.481	2471.605	2174.082	0.051151	2527.376	2234.513
YKR004C	215.3417	296.6521	306.2976	274.0019	278.8478	243.6302	-0.03898	274.3237	236.1823
YPR018W	779.6061	709.9874	896.1675	736.9397	825.0206	960.8229	0.080567	739.7746	829.711
YDR457W	1845.951	1476.339	1522.596	1586.704	1605.103	1821.845	0.0494	1707.777	1728.784
YAL053W	1764.19	2225.88	1965.245	2199.179	2181.235	2112.441	0.124675	1801.093	2019.402
YOR369C	55575.43	42198.77	48411.82	52144.53	51546.51	49241.68	0.065092	61731.83	50089.08
YGL061C	545.8394	493.4314	557.264	598.1479	623.3745	524.441	0.129079	532.9075	514.5086
YML046W	485.9583	517.1635	512.8015	551.5855	557.6955	587.0608	0.16223	544.1503	633.9168
YKL079W	766.9389	743.608	764.756	811.2605	837.6955	934.4052	0.183191	804.9827	895.5611
YJR031C	900.5199	771.2955	873.4422	868.5681	869.9589	1054.753	0.134148	786.9942	863.9531
YHL025W	1621.396	1718.605	1387.232	1650.279	1534.815	1304.253	-0.07449	1598.723	1400.412
YLR154W-I	0	0	0	0	1.152263	0	#DIV/0!	0	0.878001
YDR062W	2972.176	3118.803	3375.202	3521.73	3464.856	3441.155	0.139572	3170.462	3283.724
YOR305W	743.9077	830.626	720.2934	736.9397	835.391	743.6104	0.013213	790.3671	664.6468
YER019W	1210.289	1362.622	1117.492	1220.472	1073.91	803.2949	-0.25259	1036.584	913.1211
YLR188W	1091.679	1224.184	1255.82	1058.399	1115.391	1264.138	-0.05506	1057.945	1072.917
YJL109C	3095.393	3165.278	3186.483	3591.574	4067.49	4743.451	0.392681	3512.243	3670.922
YJL160C	41.45616	67.24115	69.16398	57.30759	48.39507	27.39617	-0.41825	38.22543	31.60804
YLR012C	0	0	0	1.790862	0	0	#DIV/0!	0	0
YLR329W	46.0624	53.39738	25.68948	23.28121	32.26338	19.56869	-0.73651	48.34393	19.31602

YDL179W	483.6552	603.1927	496.0045	615.1611	631.4404	599.7805	0.222174	494.6821	459.1946
YKL086W	94.42792	87.01796	91.88928	129.8375	101.3992	67.512	0.128261	102.3093	79.0201
YLR337C	817.6076	882.0457	883.3228	877.5224	729.3828	810.1439	-0.09579	771.2544	791.079
YLR392C	292.4962	315.4401	329.0229	216.6943	245.4321	315.056	-0.26973	265.3295	271.3023
YDR161W	2204.086	2274.333	2348.611	2541.233	2672.099	2437.281	0.164317	2725.249	2474.207
YJL006C	355.832	363.8933	302.3454	257.8841	298.4362	330.7109	-0.20444	296.8093	328.3724
YNL083W	693.2391	730.7531	740.0546	646.5012	762.7984	793.5106	0.025614	685.8093	762.1049
YER069W	1026.04	1276.593	1071.054	950.9477	1015.144	948.1032	-0.21123	791.4913	913.9991
YMR197C	1163.076	1149.033	1305.223	1198.087	1075.062	1061.602	-0.11735	1152.384	1209.007
YOR025W	723.1796	878.0903	767.7202	833.6463	728.2305	702.5161	-0.06515	680.1879	739.2769
YJL213W	905.1261	1229.129	966.3196	860.5092	808.889	779.8125	-0.34022	722.9104	640.9408
YJL058C	198.0683	198.7569	209.468	190.7268	207.4074	231.889	0.055389	168.6416	215.1103
YLL001W	1106.649	852.3805	1154.05	921.3985	876.8725	1024.421	-0.14127	983.7428	1048.333
YLR405W	749.6655	907.7555	880.3586	881.9996	922.963	858.0872	0.069513	993.8613	725.2289
YGR022C	0	0.98884	0	0	0	0	#NUM!	0	0
YNR069C	31.09212	56.3639	38.53422	41.18983	63.37449	65.55513	0.433236	37.10116	36.87605
YML057C-/	0	1.977681	1.976114	2.686293	0	2.935304	0.507742	0	1.756002
YOL138C	679.4204	527.052	594.8102	646.5012	549.6297	704.473	0.077433	535.1561	577.7247
YDR249C	186.5527	245.2324	236.1456	210.4263	228.1482	209.385	-0.04379	248.4653	194.0382
YDR003W-/	141.6419	114.7055	136.3518	118.1969	111.7696	78.27478	-0.34936	123.6705	107.9941
YLR242C	199.2199	286.7637	220.3367	267.7339	294.9794	226.9969	0.161001	258.5838	285.3503
YPR088C	2098.142	2125.018	2211.271	2312.003	2388.642	2539.038	0.170114	2167.607	2371.481
YJR042W	1482.058	1515.892	1600.652	1670.874	1788.313	1933.387	0.229779	1762.867	1646.252
YOL090W	981.1291	742.6192	937.6659	899.0128	918.354	1230.871	0.195782	985.9913	911.3651
YMR316W	394.9851	522.1077	360.6407	354.5907	237.3663	288.6382	-0.53704	207.9913	316.0804
YOR304C-/	616.0846	577.4828	551.3357	547.1084	652.1811	524.441	-0.01761	592.4942	571.5787
YIL136W	1881.649	2019.212	1834.822	1458.657	1462.222	1188.798	-0.48094	1717.896	1880.678
YFR036W-/	8.06092	5.933043	0.988057	3.581724	1.152263	4.892174	-0.6382	2.248555	4.390005
YOR137C	239.5245	218.5337	263.8112	143.269	148.642	205.4713	-0.53738	207.9913	214.2323
YFR023W	0	0	1.976114	10.74517	2.304527	4.892174	3.182593	4.49711	0
YOR067C	859.0637	1059.048	910.9884	950.9477	1020.905	1035.184	0.087999	998.3584	941.2172
YKL189W	449.1084	424.2125	412.0197	382.349	377.9424	470.6271	-0.06242	391.2486	396.8565
YJL134W	1243.685	1357.678	1144.17	1361.951	1329.712	1144.769	0.034594	1278.303	1193.203
YKL115C	0	1.977681	5.928341	11.6406	9.218108	14.67652	2.168226	14.61561	11.41401
YHR194W	377.7117	434.101	404.1152	365.3359	308.8066	341.4737	-0.2597	421.6041	371.3945
YKR083C	404.1975	313.4624	261.8351	256.9887	300.7408	306.2501	-0.18104	344.0289	304.6664
YDL121C	1331.203	1249.894	1208.393	1306.434	1198.354	1108.567	-0.06867	1522.272	1183.545
YBR162C	14999.07	13732.03	16274.28	16476.83	16203.13	15937.72	0.111384	15186.74	15214
YBR288C	606.8721	585.3935	693.6159	693.959	754.7326	789.5968	0.247157	800.4856	719.9609
YOR235W	8.06092	8.899564	0.988057	7.163448	6.913581	0.978435	-0.25358	0	3.512004
YAL020C	224.5542	220.5114	225.277	197.8903	208.5597	167.3123	-0.22445	269.8266	193.1602
YGR271W	747.3624	659.5566	689.6637	700.2271	685.5968	859.0657	0.098605	739.7746	776.1529
YLR094C	981.1291	1234.073	963.3554	822.9011	843.4569	676.0984	-0.44035	808.3555	856.051
YNL094W	1074.405	1032.349	981.1404	915.1305	949.4651	1026.378	-0.09507	914.0376	996.5312
YML058W	9830.867	9770.732	9246.236	10444.31	10425.68	8236.463	0.012876	11320.35	9635.184
YBL020W	909.7324	1009.606	965.3315	977.8107	1075.062	1124.221	0.139302	920.7833	1158.961
YJL207C	1001.857	886.9899	1013.746	920.5031	1053.169	1270.987	0.160724	971.3757	1072.917
YBR051W	2.30312	1.977681	0.988057	0	0	1.956869	-1.42894	0	0
YHR102W	853.3059	890.9452	1058.209	954.5295	982.8807	1262.181	0.191194	617.2283	993.0192
YBL083C	1.15156	0	0	0	0	0	#NUM!	0	0
YLR154C-I	1.15156	0	0	0	0	0	#NUM!	0	0
YNL028W	0	0	0	0.895431	0	0.978435	#DIV/0!	0	3.512004
Q0143	0	0	0.988057	0	0	0	#NUM!	0	0
YBR224W	0	0.98884	0	0	0	0	#NUM!	0	0

YCL068C	0	0	0.988057	0	0	0	#NUM!	0	0
YDL151C	0	0	0.988057	0	0	0	#NUM!	0	0
YDR521W	0	0.98884	0	0	0	0	#NUM!	0	0
YDR544C	0	0.98884	0	0	0	0	#NUM!	0	0
YJL086C	0	0	0.988057	0	0	0	#NUM!	0	0
YJL220W	0	0	0.988057	0	0	0	#NUM!	0	0
YJL221C	0	0.98884	0	0	0	0	#NUM!	0	0
YMR159C	0	0	0.988057	0	0	0	#NUM!	0	0
YNR005C	0	0	0.988057	0	0	0	#NUM!	0	0
YLL059C	8.06092	31.64289	22.72531	17.90862	24.19753	33.26678	0.271827	22.48555	28.97404
YJR024C	1499.331	1443.707	1521.608	1563.423	1431.111	1387.42	-0.02697	1690.913	1495.236
YPR183W	8074.738	7029.667	7295.812	7958.591	7570.371	6802.078	-0.00446	7746.272	6737.78
YJL092W	657.5407	659.5566	782.541	787.0839	668.3128	755.3516	0.074394	659.9509	698.0109
YDR366C	1.15156	0	1.976114	0	0	0.978435	-1.67654	5.621387	0
YFL029C	380.0148	440.034	420.9122	449.5064	445.926	295.4873	-0.05938	445.2139	409.1485
YIL008W	947.7338	808.8715	874.4303	1021.687	942.5515	786.6615	0.064273	1045.578	795.469
YLL033W	302.8603	321.3731	254.9187	283.8516	268.4774	292.552	-0.05736	328.289	301.1544
YPR075C	1804.494	1689.928	1437.623	1497.161	1264.033	1154.553	-0.3329	1556	1398.656
YHL031C	315.5274	235.344	266.7753	285.6425	284.6091	266.1342	0.032691	312.5491	285.3503
YBL096C	2.30312	5.933043	1.976114	1.790862	0	2.935304	-1.11156	2.248555	7.024009
YOR224C	6041.083	5304.14	5875.974	6318.161	6593.252	6213.06	0.151235	6684.954	6037.135
YEL009C-/	3.45468	0.98884	0	0.895431	6.913581	1.956869	1.136047	0	5.268006
YOR172W	886.7012	822.7152	816.1349	792.4565	811.1935	794.489	-0.07468	772.3786	780.543
YDR257C	953.4916	968.0748	1075.006	1077.204	1007.078	1015.615	0.048907	1024.217	1055.357
YGL126W	1706.612	1953.949	1489.002	1799.816	1691.523	1292.512	-0.10628	1794.347	1511.918
YDR136C	2.30312	5.933043	0	0.895431	2.304527	0.978435	-0.97902	0	1.756002
YGR106C	4365.564	4371.664	4587.548	4598.934	4825.679	4257.169	0.038145	4832.145	4230.209
YLR261C	8.06092	3.955362	0.988057	0.895431	4.609054	0	-1.24031	2.248555	0.878001
YJL117W	1946.136	1925.272	1996.863	1948.458	1958.848	1912.84	-0.01188	1992.22	1904.384
YOL040C	44593.01	35504.32	40332.48	44583.51	42680.99	39216.64	0.07073	48650.86	42218.68
YHR071C-/	1.15156	3.955362	0	0.895431	1.152263	0	-1.31845	0	0
YJR151C	116.3076	127.5604	136.3518	93.12483	94.4856	164.377	-0.11131	79.8237	133.4562
YMR080C	1989.896	1991.525	2279.447	2316.48	2464.692	2978.355	0.309606	2159.737	2503.181
YHR214W-/	0	3.955362	0	0.895431	1.152263	1.956869	0.017836	0	0
YFR042W	316.679	248.1989	275.6679	281.1653	375.6379	288.6382	0.169662	328.289	342.4204
YLR274W	652.9345	708.9986	645.2011	763.8027	708.642	687.8396	0.106084	613.8555	720.8389
YDR310C	1973.774	1687.951	2297.232	2155.302	2188.148	2444.13	0.187838	1723.517	2074.717
YLR141W	222.2511	254.132	239.1098	344.7409	308.8066	239.7165	0.320149	216.9856	263.4003
YEL053W-/	1.15156	0	2.96417	3.581724	8.065844	0.978435	1.617178	1.124277	0.878001
YKL057C	1004.16	1013.561	1141.206	917.8168	1083.128	1457.868	0.130842	1115.283	1186.179
YDR395W	3144.91	3419.41	3453.259	3577.247	3706.832	4328.595	0.213166	3387.448	3616.486
YIL173W	0	0	4.940284	0.895431	1.152263	0	-1.27059	0	0
YJL143W	1814.858	1893.629	1662.9	1845.483	1711.111	1469.609	-0.09583	1923.639	1795.512
YDL148C	2048.625	2113.152	2385.169	2592.273	2685.926	3380.492	0.403327	2382.344	2777.995
YPL202C	611.4783	630.8802	667.9264	706.4951	738.6009	862.001	0.272291	668.9451	779.665
YNL113W	932.7636	842.492	829.9677	1024.373	933.3334	924.6208	0.145827	1051.199	900.8291
YHL022C	27.63744	25.70985	31.61782	34.02638	25.3498	27.39617	0.030365	26.98266	19.31602
YPL269W	397.2882	392.5697	371.5094	306.2374	391.7696	432.4681	-0.03889	314.7977	345.9324
YER112W	2003.714	1798.701	1755.777	1739.822	1898.93	1470.587	-0.12148	1724.642	1730.54
YML070W	2110.809	2316.853	2045.278	2196.492	1995.72	2192.672	-0.01976	2179.974	2010.622
YER014C-/	4.60624	1.977681	0.988057	2.686293	6.913581	0	0.342345	3.372832	2.634003
YPL191C	170.4309	199.7458	216.3844	133.4192	165.9259	207.4282	-0.21094	156.2746	152.7722
YKL107W	112.8529	113.7166	115.6026	103.87	82.96297	62.61982	-0.45596	75.32659	91.31211
YGR178C	4014.338	3996.893	3881.087	3932.733	3910.782	4055.612	0.000826	3886.627	3971.199

YCR020W-I	468.6849	438.0563	430.7928	496.0688	502.3869	468.6702	0.133417	608.2341	473.2426
YMR168C	380.0148	367.8486	328.0349	374.2902	383.7037	497.0448	0.22219	366.5145	396.8565
YLR325C	24243.79	19704.62	22093.94	23676.99	25019.1	23240.76	0.123339	28049.6	23713.05
YKL162C-/	3.45468	3.955362	5.928341	11.6406	9.218108	2.935304	0.835015	10.1185	3.512004
YLR210W	366.1961	372.7928	346.8079	349.2181	346.8313	442.2525	0.068129	402.4913	401.2465
YPL089C	770.3936	797.0054	828.9797	831.8554	858.4363	800.3596	0.055667	640.8382	811.273
YGR171C	356.9836	452.8889	404.1152	401.1531	421.7284	454.9721	0.073969	371.0116	403.8805
YDL160C	1062.89	1182.653	1286.45	1097.798	1176.461	1241.634	-0.00659	1216.468	1116.817
YAL016C-/	2.30312	1.977681	3.952227	3.581724	4.609054	0.978435	0.155375	0	0
YOR110W	837.1841	843.4809	829.9677	891.8493	724.7737	862.001	-0.01851	745.396	762.1049
YDR250C	12.66716	17.79913	12.84474	6.268017	18.43622	14.67652	-0.13724	7.869942	14.04802
YDR123C	598.8112	657.5789	668.9145	571.285	611.8519	716.2142	-0.01958	602.6127	590.0167
YBR272C	458.3209	400.4804	514.7776	488.0099	462.0577	470.6271	0.048657	491.3093	498.7046
YGR084C	1173.44	1183.642	1326.96	1263.453	1219.095	1035.184	-0.06664	1389.607	1403.046
YAR008W	532.0207	446.9559	448.5778	464.7287	481.6461	533.2469	0.051683	545.2746	444.2685
YDR444W	427.2287	561.6614	461.4225	405.6303	456.2963	479.433	-0.11267	450.8353	500.4606
YFL049W	815.3044	664.5008	649.1533	728.8809	743.2099	745.5673	0.058889	632.9682	723.4729
YLR428C	0	1.977681	0	1.790862	5.761317	0	1.933083	0	0
YFL015C	0	0	1.976114	8.95431	0	0	2.179916	4.49711	1.756002
YDL239C	154.309	162.1698	181.8025	168.341	184.3622	189.8163	0.122715	203.4942	191.4042
YAL054C	247.5854	245.2324	263.8112	234.6029	258.107	205.4713	-0.11598	258.5838	212.4763
YNL012W	177.3402	210.623	148.2085	144.1644	177.4486	131.1103	-0.24407	176.5116	156.2842
YJR080C	124.3685	140.4153	137.3399	134.3147	120.9877	124.2612	-0.0833	102.3093	147.5042
YDR230W	0	0	0.988057	0	1.152263	0.978435	1.10866	0	0
YBR042C	859.0637	891.9341	903.0839	880.2087	985.1853	878.6344	0.048083	856.6994	899.9511
YKR062W	1198.774	1199.463	1335.853	1150.629	1099.259	1235.763	-0.09933	1174.87	1195.837
YOR053W	2.30312	0.98884	0.988057	1.790862	1.152263	0	-0.54027	1.124277	2.634003
YER082C	2120.022	2429.581	2389.121	2808.072	2745.844	2710.264	0.252201	2557.731	2684.049
YMR147W	230.312	321.3731	281.5962	303.5511	291.5227	302.3363	0.106964	252.9624	259.0103
YKL185W	1563.818	1620.709	1568.046	1775.64	1724.938	1921.646	0.190176	1364.873	1602.352
YOR181W	1036.404	1148.044	883.3228	888.2676	905.6791	687.8396	-0.30581	821.8468	857.807
YIL126W	2676.225	2230.824	2839.675	2534.07	2796.543	3325.7	0.160166	2707.26	2958.864
YLR057W	722.0281	865.2354	860.5975	968.8564	929.8766	1118.351	0.301633	802.7341	989.5072
YGR046W	419.1678	419.2683	576.0371	410.1074	422.8807	458.8859	-0.1308	429.474	463.5846
YAL039C	1533.878	1890.663	1478.133	1609.985	1524.445	1123.243	-0.2035	1518.899	1443.434
YMR167W	375.4085	478.5988	444.6256	488.0099	353.7449	525.4194	0.074203	446.3382	458.3166
YNR017W	4717.941	4606.019	4201.218	4869.354	4641.317	3852.097	-0.01743	4624.153	4605.116
YHL003C	1982.986	1859.02	1944.496	1914.432	2051.029	1886.422	0.016209	1918.017	1770.05
YAR015W	4326.411	4972.879	4272.358	5282.148	5031.935	4615.277	0.137556	4733.208	4225.819
YLR214W	4090.341	4270.802	4266.429	3576.352	3545.515	3715.117	-0.22061	4745.575	4566.484
YDL006W	742.7562	647.6905	566.1566	537.2586	498.9301	541.0744	-0.31093	574.5058	618.1128
YOR264W	491.7161	428.1679	380.4019	436.0749	441.3169	352.2365	-0.08061	355.2717	403.0025
YNL157W	2022.139	1823.422	1717.243	1723.705	1517.531	1197.604	-0.32563	1891.035	1472.408
YPR037C	726.6343	553.7506	723.2576	693.0636	679.8354	536.1822	-0.06975	673.4422	534.7027
YHR173C	95.57947	89.98448	94.85346	101.1837	64.52675	77.29634	-0.20658	82.07226	96.58012
YML012W	4595.876	4148.186	4493.682	4587.293	4486.914	4303.156	0.015136	4916.465	4387.371
YDR414C	450.2599	485.5207	375.4616	469.2059	456.2963	493.1311	0.113568	466.5752	428.4645
YMR158C-/	41.45616	26.69869	25.68948	30.44465	20.74074	31.30991	-0.18596	26.98266	24.58403
YEL064C	230.312	217.5449	210.4561	197.8903	251.1934	263.1989	0.113676	218.1098	214.2323
YBR290W	1027.191	1145.077	962.3673	1006.464	1001.317	960.8229	-0.07851	954.5116	985.9952
YKL080W	4942.495	5018.365	5074.66	4762.798	4811.852	4376.538	-0.10799	5097.474	4842.176
YDR519W	1751.523	1419.975	1687.601	1582.227	1714.568	1518.531	-0.01306	1740.382	1444.312
YOR135C	0	1.977681	2.96417	0	0	0.978435	-2.3365	0	6.146008
YBL060W	492.8677	583.4159	511.8134	414.5846	463.2099	576.298	-0.12718	511.5463	491.6806

YHR132W-/	461.7755	360.9268	336.9274	327.7278	397.5309	303.3148	-0.17302	427.2254	350.3224
YER168C	1091.679	1041.249	1104.648	1096.903	1090.041	1324.801	0.117274	989.3642	1054.479
YPR045C	369.6507	449.9224	413.0078	492.4871	390.6173	440.2956	0.102567	417.1069	446.0245
YER060W-/	169.2793	199.7458	233.1814	197.8903	243.1276	265.1558	0.229765	227.1041	162.4302
YPL065W	680.5719	592.3154	550.3477	479.0556	506.9959	476.4977	-0.31802	561.0145	538.2147
YHR189W	40.3046	112.7278	78.05649	67.15733	84.11523	93.92973	0.085525	57.33815	93.94611
YNL315C	1491.27	1229.129	1274.593	1361.055	1251.358	1160.424	-0.08254	1367.121	1260.81
YDL191W	22925.26	17297.79	21056.48	22450.25	22265.19	20532.45	0.090526	24457.53	21396.01
YAL023C	9268.906	8867.921	9681.969	9460.229	10031.61	10903.68	0.127798	10071.28	10596.59
YJR021C	467.5333	551.773	470.315	513.9774	464.3622	340.4953	-0.17568	589.1214	498.7046
YLR321C	696.6938	683.2887	874.4303	822.9011	845.7614	990.1759	0.238044	874.6879	768.2509
YNL303W	272.9197	139.4265	128.4474	217.5897	168.2305	197.6438	0.109566	236.0983	182.6242
YHR116W	549.2941	525.0743	574.061	585.6119	437.8601	371.8052	-0.24054	623.974	523.2886
YBL017C	3258.915	2901.258	3465.115	3250.415	3210.206	3854.054	0.099797	3444.786	3381.182
YGR210C	2637.072	2801.385	2487.927	2919.105	2646.749	2343.351	-0.00313	2971.465	2581.323
YGR113W	791.1217	914.6774	970.2718	893.6402	866.5021	821.8852	-0.05161	773.5029	856.929
YIL032C	13.81872	13.84377	7.904455	22.38578	6.913581	5.870608	-0.01619	25.85838	5.268006
YIL102C-/	272.9197	254.132	199.5875	239.0801	241.9753	222.1047	-0.04739	276.5723	230.9143
YLR326W	589.5987	609.1257	507.8612	514.8728	587.6544	534.2254	-0.06028	482.315	478.5106
YDL123W	655.2376	869.1907	682.7473	599.9388	701.7285	514.6567	-0.28118	764.5087	624.2588
YIL028W	13.81872	29.66521	24.70142	30.44465	13.82716	12.71965	-0.25872	23.60983	7.90201
YNL153C	2026.745	1604.888	1733.052	1806.084	1898.93	1583.107	-0.02074	2009.084	1835.022
YNL066W	3020.542	3467.863	3167.71	3663.208	3657.284	3397.125	0.150469	3334.607	3027.348
YDR374W-/	386.9241	282.8084	387.3183	340.2638	337.6132	296.4657	-0.11754	373.2601	311.6904
YJR132W	1242.533	1517.87	1555.201	1584.017	1658.107	1743.571	0.208232	1435.702	1369.682
YHR049W	6601.893	6220.795	6199.069	6841.093	6631.276	6063.36	0.038465	6918.804	5972.163
YGL003C	548.1425	608.1369	655.0817	578.4484	504.6914	528.3547	-0.16867	504.8006	572.4567
YMR030W-/	0	4.944202	2.96417	7.163448	3.45679	0	0.425363	1.124277	7.90201
YAL037C-/	49.51708	83.0626	42.48644	42.98069	80.65844	62.61982	0.08941	121.422	70.24009
YIL120W	289.0415	345.1053	298.3932	266.8384	304.1976	284.7245	-0.12396	360.8931	273.9363
YLR111W	5.7578	14.83261	4.940284	3.581724	9.218108	6.849043	-0.37779	12.36705	3.512004
YPR095C	619.5392	635.8244	675.8309	720.822	667.1605	973.5425	0.290225	577.8786	651.4768
YHL018W	36.84992	59.33043	55.33118	14.3269	46.09054	57.72765	-0.35887	38.22543	60.58207
YDR335W	1450.966	1348.778	1522.596	1404.036	1458.766	1621.266	0.052996	1390.731	1511.918
YOR227W	1187.258	1180.675	1120.456	1200.773	1168.395	1446.127	0.129233	1269.309	1255.542
YGR011W	31.09212	29.66521	34.58199	35.81724	36.87243	45.008	0.303943	28.10694	25.46203
YMR236W	2230.572	2002.402	2038.361	2357.67	2404.774	2072.325	0.12412	2466.665	2261.731
YJL211C	0	6.921883	10.86863	2.686293	13.82716	3.913739	0.199383	7.869942	10.53601
YNL109W	0	0.98884	0	3.581724	2.304527	0.978435	2.795384	4.49711	1.756002
YKL008C	1665.156	1756.181	1675.744	1670.874	1607.408	1570.388	-0.07208	1554.876	1483.822
YCL059C	2919.204	2877.526	2809.046	3219.075	3295.474	2978.355	0.141544	3237.919	3003.642
YMR076C	930.4604	802.9384	852.693	821.1103	880.3293	1160.424	0.146181	762.2601	784.933
YDR072C	1703.157	2433.536	1901.021	1841.006	1597.037	1452.976	-0.30387	1856.182	2001.842
YMR312W	686.3297	582.427	592.8341	697.5408	667.1605	698.6024	0.14842	586.8728	731.3749
YBR270C	261.4041	349.0607	249.9784	248.9298	268.4774	270.048	-0.12788	264.2052	236.1823
YIL074C	2582.949	2791.497	2455.321	2561.828	2559.177	2915.735	0.037641	2334	2231.879
YFR027W	373.1054	390.592	316.1782	299.074	301.893	359.0855	-0.16968	319.2948	332.7624
YNL221C	1715.824	1749.259	1610.533	1805.189	1841.317	2141.794	0.189557	1542.509	1819.218
YDL114W	4.60624	8.899564	2.96417	3.581724	4.609054	0	-1.00777	4.49711	3.512004
YGL249W	147.3997	155.2479	147.2205	140.5827	129.0535	152.6358	-0.09133	148.4046	149.2602
YLR346C	183.098	170.0806	136.3518	176.3999	120.9877	80.23165	-0.37447	149.5289	124.6762
YMR302C	1772.251	1806.611	1781.466	1634.162	1519.836	1657.468	-0.15585	1614.462	1478.554
YMR062C	923.5511	789.0947	765.744	743.2078	719.0124	704.473	-0.19391	806.107	812.151
YPR003C	306.3149	302.5852	263.8112	344.7409	267.3251	244.6087	-0.02676	242.8439	385.4425

YLL064C	1.15156	1.977681	1.976114	0	0	3.913739	-0.38346	0	0
YMR008C	4739.821	5556.294	4900.762	4874.727	4868.313	4828.575	-0.06061	4371.191	4866.76
YBR249C	12041.86	10578.61	11295.47	11845.66	11228.81	10929.12	0.003723	11696.98	10513.18
YGL092W	1868.982	1927.25	1972.161	1909.059	1987.654	2505.771	0.150462	1924.763	2279.291
YPL109C	186.5527	140.4153	197.6114	139.6872	109.465	115.4553	-0.52482	110.1792	130.8222
YER182W	1075.557	1088.713	1057.221	947.366	819.2593	771.985	-0.34369	1236.705	1122.963
YPR181C	5213.112	5785.705	5670.458	5541.823	5724.445	6254.155	0.071846	5511.208	6086.303
YOR021C	2070.505	1875.83	1892.129	2135.603	1933.498	2022.425	0.061215	2118.139	1995.696
YCR105W	173.8856	194.8016	175.8741	154.0141	177.4486	176.1182	-0.10146	204.6185	199.3062
YDL076C	833.7294	776.2397	710.4129	823.7965	810.0412	921.6855	0.139256	763.3844	755.0809
YPR093C	366.1961	400.4804	268.7515	290.1197	293.8272	251.4577	-0.30968	381.1301	299.3984
YPR084W	274.0713	335.2169	270.7276	298.1785	273.0864	273.9617	-0.05819	291.1879	293.2524
YGL110C	743.9077	720.8647	717.3293	640.2332	753.5803	673.1631	-0.0782	721.7861	747.1789
YIR004W	1545.393	1380.421	1591.76	1576.854	1355.062	1612.46	0.008534	1567.243	1456.604
YNL096C	14024.85	12592.88	12292.41	13906.94	13995.39	12547.45	0.055985	16496.52	14309.66
YOR256C	879.7918	802.9384	932.7256	896.3265	918.354	985.2838	0.098346	744.2717	892.0491
YPL039W	386.9241	449.9224	428.8167	479.0556	452.8395	441.2741	0.117616	457.5809	472.3646
YOR047C	601.1143	629.8914	587.8938	626.8017	686.749	671.2062	0.125897	532.9075	574.2127
YBL048W	54.12332	49.44202	37.54616	49.24871	41.48148	44.02956	-0.06645	37.10116	62.33808
YLR378C	6223.03	5625.513	5564.736	6037.891	5844.28	5495.868	-0.00292	6752.411	5992.357
YLR287C	1275.928	1245.939	1274.593	1421.944	1319.342	1488.199	0.155827	1308.659	1368.804
YER104W	461.7755	366.8598	354.7124	333.1003	336.4609	385.5033	-0.16554	405.8642	356.4684
YOR190W	76.00296	76.14071	53.35507	55.51672	44.93828	37.18052	-0.57828	57.33815	66.72808
YLR207W	895.9136	856.3358	980.1524	928.562	1011.687	1213.259	0.206788	914.0376	937.7051
YGR199W	748.514	862.2689	916.9167	908.8625	937.9425	875.6991	0.107109	757.763	834.979
YOR302W	36.84992	60.31927	49.40284	39.39897	51.85186	58.70608	0.032938	29.23121	58.82607
YJR088C	772.6967	884.0233	973.236	891.8493	873.4157	1028.335	0.087087	957.8844	932.4371
YBR180W	42.60772	55.37506	55.33118	33.13095	58.76544	46.96487	-0.14285	44.9711	35.12004
YJL065C	84.06388	67.24115	83.98483	64.47103	74.89713	52.83547	-0.2918	95.56359	75.50809
YGL062W	5142.867	6203.985	5575.605	4293.592	4242.634	4431.331	-0.38403	4330.717	4698.184
YIL087C	1127.377	1070.914	1185.668	1143.465	1174.156	906.0305	-0.07002	1538.012	1175.643
YLR152C	409.9553	485.5207	356.6885	347.4272	345.679	318.9697	-0.30711	375.5087	372.2725
YJR053W	102.4888	117.672	175.8741	171.0273	168.2305	304.2932	0.700427	97.81214	206.3303
YPL030W	800.3342	827.6594	788.4693	848.8686	906.8313	1050.839	0.215895	752.1416	796.347
YJR102C	586.144	482.5541	446.6017	439.6566	452.8395	419.7485	-0.20757	541.9017	408.2705
YMR158W-I	0	0.98884	0	0	2.304527	0	1.220661	0	0.878001
YCL046W	19.57652	8.899564	3.952227	3.581724	5.761317	7.827478	-0.91732	8.99422	2.634003
YGL238W	1870.133	1764.091	2037.373	1960.994	2004.938	2386.402	0.163532	2009.084	2029.938
YDL064W	1576.486	1209.352	1318.068	1482.834	1620.082	1336.542	0.113386	1774.11	1481.188
YDR207C	899.3683	896.8783	998.9255	933.9346	1034.733	1127.157	0.147386	802.7341	956.1432
YHR129C	285.5869	328.295	350.7602	293.7014	299.5885	279.8323	-0.14381	351.8989	321.3484
YIL144W	578.0831	506.2863	662.9861	608.8931	663.7038	845.3676	0.277505	587.9971	712.0589
YER122C	3893.424	3575.647	3888.004	4045.557	4072.099	4759.106	0.181179	3722.483	3758.723
YLR293C	18100.22	16185.34	15917.6	17682.08	17345.02	15743.99	0.016229	19357.81	16680.26
YPL102C	0	0.98884	1.976114	0	0	0.978435	-1.59946	0	1.756002
YPR109W	467.5333	554.7395	479.2076	456.6698	451.6873	419.7485	-0.17702	519.4162	460.0726
YBR109W-A	1.15156	4.944202	12.84474	3.581724	8.065844	8.805912	0.110872	2.248555	11.41401
YCL044C	1092.83	1208.363	1233.095	1241.067	1204.115	1174.122	0.034293	1088.301	1156.327
YDR283C	725.4828	710.9763	730.174	685.9002	812.3457	873.7422	0.130642	613.8555	672.5488
YDL063C	1564.97	1529.736	1582.867	1732.659	2018.766	2053.734	0.311575	1792.098	1769.172
YGL173C	4207.8	3732.873	4019.415	4048.244	4172.346	4659.306	0.106893	4371.191	4274.987
YOR155C	749.6655	869.1907	742.0307	715.4494	672.9219	749.481	-0.14317	652.0809	734.8869
YLR081W	50.66864	62.29695	77.06843	57.30759	46.09054	54.79234	-0.2646	71.95376	69.36208
YGR222W	633.358	611.1034	633.3444	617.8474	636.0494	648.7022	0.018924	593.6185	562.7987

YML007C-/	5.7578	12.85493	3.952227	8.058879	9.218108	7.827478	0.15386	6.745665	7.90201
YIR036W-/	0	1.977681	0.988057	0	1.152263	0.978435	-0.47706	0	1.756002
YKL111C	0	0.98884	0	0.895431	1.152263	0	1.050191	2.248555	0
YCR049C	0	0	2.96417	0	0	1.956869	-0.59908	0	1.756002
YER037W	223.4026	263.0316	292.4648	282.9562	236.214	298.4226	0.069946	279.9451	285.3503
YIL095W	520.5051	465.7438	586.9058	467.415	464.3622	644.7885	0.003125	505.9249	579.4807
YDL082W	23063.44	20692.47	20949.77	22603.37	22116.55	21161.59	0.025981	24030.31	21619.02
YNL212W	1167.682	1188.586	1291.39	1203.459	1168.395	1425.579	0.058054	1274.931	1237.982
YPL238C	1.15156	0	0.988057	0	0	0.978435	-1.1288	1.124277	0
YDL047W	1567.273	1976.692	1764.669	1739.822	1706.502	1418.73	-0.12588	1666.179	1789.366
YLR299W	613.7814	622.9695	662.9861	554.2718	577.284	528.3547	-0.19469	525.0376	612.8447
YCR095C	389.2273	422.2349	452.53	447.7155	373.3334	392.3523	-0.05893	395.7457	354.7124
YLR302C	0	1.977681	1.976114	0	4.609054	0	0.221233	2.248555	0.878001
YER057C	3051.634	2554.175	2641.076	2766.882	2745.844	2552.736	-0.03209	3083.893	2762.191
YPR016W-/	69.0936	75.15187	66.19981	45.66698	51.85186	62.61982	-0.39412	83.19653	66.72808
YJL049W	400.7429	446.9559	436.7211	363.545	427.4897	420.7269	-0.08401	382.2543	379.2965
YNL241C	4593.573	5216.133	4322.749	4606.993	4161.976	3675.979	-0.18345	4380.185	3978.223
YNR071C	79.45764	63.28579	110.6624	76.11164	66.83128	74.36104	-0.22174	93.31503	86.92211
YPR169W	1898.922	2062.721	1979.078	2452.586	2564.938	2384.445	0.317271	2206.957	2152.859
YOR151C	2781.017	2505.722	3052.108	2832.248	2956.708	3753.276	0.194479	2754.48	3184.51
YBR006W	893.6105	1112.445	1046.352	893.6402	841.1523	891.354	-0.217	1178.243	1009.701
YOR123C	1985.289	1914.395	1861.499	2106.949	2251.523	2094.829	0.163672	2068.671	1964.966
YJR159W	6.90936	9.888404	11.85668	8.95431	11.52263	0	-0.48476	13.49133	10.53601
YPR198W	464.0787	501.3421	414.9839	432.4932	409.0535	324.8403	-0.24304	339.5318	381.0525
YLR090W	814.1529	1054.104	845.7766	769.1753	806.5844	744.5888	-0.2261	839.8353	757.7149
YOL088C	931.612	913.6886	908.0242	895.431	980.5762	859.0657	-0.0096	991.6127	887.6591
YLR336C	1026.04	1217.263	1276.569	1455.971	1496.79	1744.549	0.416312	1280.552	1377.584
YLR023C	1178.046	1410.086	1364.506	1072.726	1149.959	1260.224	-0.18252	1201.853	1109.793
YAL011W	535.4754	495.4091	497.9806	462.9378	515.0618	518.5704	-0.0308	455.3324	472.3646
YMR095C	73.69984	77.12955	91.88928	81.48422	86.41976	128.1749	0.286697	95.56359	126.4322
YDR105C	719.725	815.7934	714.3651	745.894	729.3828	665.3356	-0.07183	704.922	654.9888
YER038C	105.9435	179.969	241.0859	169.2365	131.358	217.2125	-0.02538	147.2803	126.4322
YMR216C	1032.949	1422.941	1242.975	1212.414	1223.704	1311.103	0.018738	1295.168	1276.614
YHR168W	287.89	353.016	388.3063	367.1267	376.7902	279.8323	-0.00768	360.8931	325.7384
YER157W	627.6002	614.0699	618.5236	639.3378	604.9383	765.1359	0.111321	625.0983	636.5508
YLR307C-/	4.60624	3.955362	6.916398	0	0	3.913739	-1.9836	6.745665	5.268006
YCR031C	35902.18	27871.46	30930.13	33221.39	32604.45	30599.57	0.025991	40966.42	31738.86
YMR308C	4048.885	4437.916	4272.358	4629.378	4950.124	5385.305	0.230041	4428.529	4477.805
YFL018C	5208.506	5411.924	5466.918	5354.678	5065.35	5382.369	-0.02578	5637.127	5347.027
YNL150W	1.15156	1.977681	0.988057	10.74517	2.304527	4.892174	2.123561	5.621387	4.390005
YIL079C	916.6417	1150.021	998.9255	1027.955	970.2058	896.2462	-0.0829	861.1965	971.0692
YCL038C	289.0415	420.2572	323.0946	316.9826	316.8725	244.6087	-0.23294	326.0405	335.3964
YKR095W-/	413.41	381.6924	416.96	373.3947	377.9424	367.8915	-0.11496	493.5578	423.1965
YBR134W	3.45468	0.98884	0	4.477155	0	0.978435	0.296032	1.124277	2.634003
YML024W	22357.54	17788.25	20389.54	21339.91	21210.87	20184.13	0.051491	25271.51	20364.36
YGL113W	588.4471	562.6502	661.9981	635.756	676.3787	718.1711	0.163242	555.3931	565.4327
YJR015W	552.7488	670.4338	578.0132	595.4616	595.7202	576.298	-0.02726	583.5	575.9687
YMR067C	870.5793	779.2063	897.1556	929.4574	904.5268	894.2893	0.099223	852.2023	942.9732
YDR296W	1472.845	1612.799	1690.565	1612.671	1546.338	1306.21	-0.09714	2007.96	1665.568
YOR249C	276.3744	329.2839	330.999	253.407	300.7408	362.9993	-0.03037	297.9335	316.9584
YML017W	580.3862	564.6279	553.3118	553.3764	622.2223	642.8316	0.09858	517.1676	652.3548
YNL262W	731.2406	604.1815	664.9622	672.4687	614.1564	862.9794	0.103794	558.7659	794.591
YLR415C	2.30312	1.977681	0.988057	3.581724	1.152263	1.956869	0.344701	2.248555	2.634003
YLR085C	579.2346	677.3557	639.2728	610.684	555.391	585.104	-0.11453	545.2746	607.5767

YNR032C-/	301.7087	338.1834	251.9545	326.8323	326.0906	297.4442	0.091689	433.9711	399.4905
YPL087W	1508.544	1557.424	1566.07	1465.821	1450.7	1331.65	-0.12481	1690.913	1482.066
YPL176C	1318.536	1291.426	1348.698	1310.911	1321.646	1611.482	0.100426	1209.723	1244.128
YBR041W	819.9107	1032.349	965.3315	950.0523	1033.58	1061.602	0.112091	853.3266	949.9972
YJR058C	625.297	539.9069	551.3357	520.2454	646.4198	524.441	-0.02154	600.3642	520.6546
YML026C	29945.16	24543.02	28464.93	28994.95	29056.63	28681.84	0.064292	33024.53	28938.92
YLL048C	2913.447	2777.653	3040.251	3101.773	3292.017	3909.825	0.238874	3219.931	3518.15
YDR180W	389.2273	288.7414	383.366	317.878	382.5515	495.088	0.171755	363.1416	417.0505
YOL023W	450.2599	630.8802	586.9058	511.2911	444.7737	513.6782	-0.1826	466.5752	569.8227
YHL013C	878.6402	746.5745	828.9797	933.9346	798.5186	939.2973	0.122536	779.1243	751.5689
YNR023W	649.4798	627.9137	718.3173	691.2728	728.2305	729.9123	0.107042	535.1561	632.1608
YLR264C-/	3309.583	2509.677	2443.465	2916.419	2995.885	2712.221	0.061827	3968.699	3057.2
YPL194W	328.1946	266.9869	246.0261	258.7796	265.0206	295.4873	-0.03809	224.8555	292.3744
YDL120W	1099.74	1031.361	1026.591	1091.53	989.7943	829.7126	-0.11734	1289.546	976.3372
YBR030W	1030.646	1182.653	959.4032	976.9152	989.7943	922.6639	-0.13496	981.4942	889.4151
YJR055W	263.7072	307.5294	252.9425	233.7075	223.5391	281.7892	-0.15731	259.7081	217.7443
YOR296W	541.2332	605.1703	625.44	607.9977	568.0659	723.0633	0.100085	635.2168	559.2867
YGR257C	819.9107	868.2019	827.9916	939.3071	743.2099	798.4027	-0.02032	935.3989	876.2451
YGR108W	853.3059	886.9899	951.4987	933.0391	921.8108	1068.451	0.11903	740.8989	835.857
YKL077W	3194.427	3029.807	3336.668	3450.096	3548.971	3504.753	0.135695	3387.448	3234.556
YNR068C	64.48736	94.92868	94.85346	55.51672	51.85186	67.512	-0.53999	70.82948	76.38609
YLR308W	4.60624	1.977681	0	3.581724	4.609054	2.935304	0.756927	0	4.390005
YOR188W	1574.182	1349.767	1673.768	1711.169	1762.963	2296.386	0.327783	1303.038	1741.954
YEL020C	610.3268	685.2664	578.0132	514.8728	535.8025	593.9099	-0.18809	573.3815	535.5807
YJR111C	614.933	682.2999	554.2999	527.4089	540.4116	557.7078	-0.18781	627.3468	537.3367
YJL056C	460.624	474.6434	446.6017	444.1338	440.1646	530.3116	0.033783	377.7572	374.9065
YMR301C	1050.223	1269.671	1141.206	1191.819	1317.037	1014.637	0.025776	1128.775	1094.867
YGL111W	2406.76	2651.081	2463.226	2945.968	2691.687	2811.043	0.167792	2651.046	2620.833
YGR045C	2.30312	8.899564	2.96417	1.790862	3.45679	1.956869	-0.97554	5.621387	2.634003
YNL163C	1183.804	1277.582	1239.023	1347.624	1403.457	1622.245	0.241046	1151.26	1410.948
YJL135W	0	1.977681	0	5.372586	1.152263	5.870608	2.64793	2.248555	5.268006
YJL148W	3818.573	3975.139	3550.088	4153.905	4057.12	4013.539	0.107879	4402.671	4066.023
YGR204C-/	1.15156	0.98884	5.928341	1.790862	1.152263	3.913739	-0.23479	2.248555	2.634003
YGR224W	168.1278	145.3595	175.8741	137.0009	131.358	157.528	-0.20043	100.0607	166.8202
YOL009C	304.0118	276.8753	245.0381	231.0212	273.0864	271.0264	-0.09157	257.4595	302.0324
YJL177W	25981.5	21180.96	23553.3	25331.74	24540.91	23242.72	0.048143	27896.7	23883.39
YDL010W	147.3997	134.4823	125.4832	135.2101	125.5967	101.7572	-0.16809	96.68786	151.8942
YJR156C	29.94056	61.30811	65.21175	55.51672	55.30865	27.39617	-0.17882	56.21387	36.87605
YDR470C	484.8067	379.7147	395.2227	428.016	442.4692	422.6838	0.03778	437.3439	493.4366
YOR350C	332.8008	363.8933	310.2498	296.3877	335.3087	257.3283	-0.17969	275.448	295.0084
YAL066W	0	5.933043	3.952227	0.895431	0	0	-3.46463	0	0
YMR156C	238.3729	192.8239	134.3757	174.6091	188.9712	161.4417	-0.10733	202.3699	178.2342
YLL036C	982.2806	1144.088	1055.245	1066.458	1140.741	1164.337	0.083648	984.8671	1122.085
YDR496C	2426.337	2641.193	2770.511	3101.773	3186.008	3772.844	0.360155	2837.676	3213.484
YLR422W	647.1767	663.5119	778.5888	620.5337	608.3951	745.5673	-0.08152	662.1994	693.6208
YGL078C	4275.742	4912.559	4662.64	5469.293	5337.284	5603.496	0.244598	4977.176	5392.683
YBL059C-/	580.3862	522.1077	540.4671	421.748	512.7572	481.3899	-0.21458	683.5607	590.0167
YDR198C	449.1084	482.5541	454.5061	499.6505	473.5803	586.0824	0.169808	508.1734	462.7066
YER034W	703.6031	673.4003	639.2728	694.8545	625.6791	592.9314	-0.07551	636.3411	657.6228
YDR203W	0	1.977681	0	0	0	0.978435	-1.01526	0	0.878001
YNL068C	1204.532	1068.937	1306.211	1312.702	1312.428	1480.372	0.197728	1007.353	1226.568
YJL162C	384.621	412.3465	450.5539	483.5328	412.5103	564.5568	0.227496	440.7168	465.3406
YOR132W	1594.911	1380.421	1492.954	1394.186	1444.938	1453.954	-0.05771	1551.503	1430.264
YNL097C	726.6343	620.9918	738.0784	659.9327	616.461	638.9179	-0.12296	652.0809	680.4508

YBL036C	1959.955	2048.877	1887.189	2056.805	2009.547	2109.505	0.066898	2289.029	1967.6
YGL120C	3811.663	4072.045	4169.6	4735.039	4922.47	5065.357	0.288629	4006.925	4457.611
YOR329W-/	0	0.98884	0	0	0	0	#NUM!	1.124277	0
YML031W	1995.653	1941.094	1790.359	2046.06	1998.025	1874.681	0.04749	1808.962	1907.896
YBR052C	4674.182	3583.558	4136.006	3641.718	3461.399	3352.117	-0.24539	4737.705	4121.337
YMR294W	620.6908	555.7283	602.7147	616.0565	607.2428	600.7589	0.035977	632.9682	594.4067
YDR463W	1417.57	1386.354	1476.157	1424.631	1590.124	1573.323	0.100251	1423.335	1518.064
YGL099W	1828.677	1827.377	1825.929	2154.407	2011.852	2036.123	0.178124	1759.494	2072.083
YHR099W	2026.745	1858.031	2199.414	1968.157	2060.247	2424.561	0.084897	1762.867	2165.151
YDR282C	183.098	213.5895	172.9099	143.269	146.3375	182.9673	-0.2694	202.3699	195.7942
YKL134C	409.9553	433.1121	504.897	413.6891	451.6873	419.7485	-0.06887	378.8815	431.9765
YGR217W	536.6269	508.264	697.5681	625.9063	608.3951	733.826	0.175699	568.8844	566.3107
YIL007C	513.5957	506.2863	502.9209	456.6698	396.3786	342.4522	-0.34911	514.9191	428.4645
YNL218W	465.2302	519.1412	511.8134	433.3886	503.5391	616.4139	0.054087	360.8931	471.4866
YBR020W	142.7934	122.6162	100.7818	100.2883	99.09466	66.53356	-0.46162	113.552	86.92211
YDR162C	743.9077	752.5076	816.1349	693.959	705.1852	705.4514	-0.13594	738.6503	743.6669
YLR279W	26.48588	34.60942	20.74919	27.75836	20.74074	27.39617	-0.10888	34.8526	21.07203
YMR215W	4407.02	4201.583	4491.706	4481.632	4925.926	4701.379	0.107008	4390.304	4266.207
YHL034W-/	31.09212	45.48666	18.77308	24.17664	23.04527	32.28835	-0.26212	32.60405	31.60804
YKL045W	1005.312	832.6036	1056.233	993.9284	950.6174	1197.604	0.118613	942.1445	921.9011
YCR054C	317.8305	325.3285	354.7124	336.6821	377.9424	407.0288	0.168701	285.5665	361.7364
YGR261C	1497.028	1308.236	1358.578	1462.239	1444.938	1540.056	0.094993	1466.058	1471.53
YAL047W-/	0	0	0	0.895431	0	0	#DIV/0!	2.248555	0
YHL048C-/	0	0	0	0.895431	0	0	#DIV/0!	2.248555	0
YER146W	2.30312	0.98884	0	0	0	0	#NUM!	0	0
YLR123C	10.36404	5.933043	13.8328	15.22233	24.19753	6.849043	0.618848	20.23699	16.68202
YER145C-/	38.00148	26.69869	23.71336	8.95431	24.19753	22.504	-0.66773	24.7341	22.82803
YER087C-/	20.72808	8.899564	14.82085	17.01319	13.82716	10.76278	-0.09544	14.61561	7.90201
YER183C	492.8677	383.6701	403.1272	556.0627	451.6873	441.2741	0.179315	525.0376	491.6806
YDR130C	431.835	529.0296	523.6701	453.9835	448.2305	461.8212	-0.12213	450.8353	479.3886
YGR160W	0	1.977681	0	0	0	0.978435	-1.01526	0	0
YKL096C-I	0	5.933043	2.96417	3.581724	1.152263	1.956869	-0.41116	0	3.512004
YGR020C	5293.721	4687.104	5179.394	5459.443	5173.663	5029.154	0.047002	6035.122	5128.404
YOL043C	242.9791	306.5405	245.0381	254.3024	218.9301	256.3499	-0.12308	251.8382	280.9603
YGL235W	0	1.977681	0.988057	0	0	0	#NUM!	0	0
YDR118W-/	3.45468	1.977681	0.988057	0.895431	2.304527	0.978435	-0.61972	1.124277	3.512004
YCR001W	25.33432	48.45318	35.57005	26.86293	35.72017	24.46087	-0.32924	58.46243	36.87605
YLR229C	1616.79	1552.479	1523.584	1438.062	1441.482	1422.644	-0.12539	1857.306	1659.422
YIR037W	4321.804	3485.663	3971	4016.008	3875.062	3427.457	-0.05747	4566.815	3818.427
YMR182W-/	16.12184	30.65405	19.76114	20.59491	16.13169	13.69809	-0.40003	17.98844	9.658012
YKR095W	2017.533	1496.116	2166.809	2034.419	2142.058	2813.978	0.299379	2016.954	2446.989
YHR100C	574.6284	482.5541	493.0404	579.3439	699.4239	462.7996	0.167911	617.2283	496.0706
YBR206W	47.21396	24.72101	32.60588	30.44465	42.63375	29.35304	-0.02941	50.59249	26.34003
YDR401W	0	0	2.96417	0	0	0	#NUM!	0	0
YER039C-/	25.33432	32.63173	47.42673	7.163448	14.97943	35.22365	-0.8775	28.10694	22.82803
YDL026W	0	1.977681	0.988057	1.790862	0	0.978435	-0.09887	0	1.756002
YJR043C	712.8156	775.2509	694.6039	820.2148	848.0659	887.4403	0.227636	847.7052	763.8609
YOR055W	0	1.977681	1.976114	0	1.152263	0.978435	-0.89191	0	1.756002
YMR315W-/	78.30608	80.09608	54.34313	37.6081	28.80659	31.30991	-1.12233	49.46821	58.82607
YOR003W	418.0163	504.3086	433.7569	431.5978	377.9424	331.6894	-0.24886	511.5463	415.2945
YOR291W	1000.706	896.8783	951.4987	937.5163	1011.687	1163.359	0.127605	937.6474	1044.821
YDR326C	1792.979	1502.049	2193.486	2152.616	2483.128	3566.395	0.579585	1927.012	2642.783
YGL164C	637.9642	600.2261	602.7147	641.1286	589.9589	635.0041	0.019605	599.2399	679.5728
YNL069C	38535.8	29928.24	34942.63	38044.18	36681.16	33840.14	0.070236	42252.6	34918.1

YCR007C	213.0386	194.8016	166.9816	155.805	108.3128	114.4769	-0.60246	204.6185	183.5022
YMR222C	1159.621	1105.524	1161.955	1156.001	1011.687	924.6208	-0.1483	1142.266	1086.965
YLR120W-/	4.60624	2.966521	0.988057	3.581724	2.304527	0.978435	-0.31855	2.248555	5.268006
YLR453C	118.6107	151.2926	150.1846	142.3735	89.87655	132.0887	-0.20541	136.0376	134.3342
YML012C-/	11.5156	7.910723	20.74919	10.74517	4.609054	5.870608	-0.92056	16.86416	12.29202
YOR012W	84.06388	101.8506	90.90123	79.69336	80.65844	84.14539	-0.17911	60.71098	93.94611
YEL035C	8.06092	6.921883	3.952227	11.6406	0	1.956869	-0.47772	5.621387	1.756002
YKL038W	1121.619	1052.126	1114.528	916.0259	1032.428	1117.372	-0.10105	924.1561	1148.425
YJL136C	18466.42	14123.61	15459.14	16675.61	17827.82	17537.46	0.115135	20512.44	18675.96
YDR303C	1178.046	1091.68	1152.074	1157.792	1175.309	1463.738	0.150044	1118.656	1243.25
YPL262W	3743.721	3937.563	3734.855	3648.881	3691.852	3461.702	-0.07972	3699.997	3484.786
YDR281C	11.5156	25.70985	26.67753	22.38578	29.95885	29.35304	0.354412	25.85838	12.29202
YNR062C	186.5527	151.2926	167.9697	168.341	223.5391	250.4793	0.344771	157.3988	219.5003
YDL109C	156.6122	259.0762	240.0978	225.6486	182.0576	311.1422	0.132462	148.4046	251.1083
YER007W	184.2496	202.7123	207.4919	195.204	208.5597	240.6949	0.116523	211.3642	189.6482
YOL041C	2824.777	2905.213	2864.377	3329.213	3631.934	3662.281	0.305786	3384.075	3418.936
YKL020C	1304.717	1417.997	1591.76	1404.931	1488.724	1861.961	0.140448	1269.309	1341.586
YJL047C	520.5051	526.0631	511.8134	477.2647	503.5391	653.5944	0.068711	490.185	578.6027
YDL246C	0	0	1.976114	0	0	0.978435	-1.01412	2.248555	1.756002
YCR069W	2205.237	2217.969	2244.865	2214.401	2207.737	1974.481	-0.05996	2120.387	2063.303
YNR028W	933.9151	930.4988	879.3706	870.359	1039.342	815.0361	-0.01005	969.1272	951.7532
YGL214W	1.15156	0.98884	0	0	2.304527	0	0.10659	0	0
YBR274W	371.9539	418.2795	428.8167	378.7673	430.9465	451.0584	0.04855	413.7341	345.9324
YFR054C	8.06092	2.966521	5.928341	7.163448	5.761317	3.913739	-0.01001	5.621387	1.756002
YDR285W	188.8558	146.3484	196.6233	195.204	163.6214	204.4929	0.082992	164.1445	221.2563
YAL013W	1247.139	1262.749	1023.627	1280.466	1266.338	954.9523	-0.01303	1224.338	1149.303
YKR041W	0	36.5871	16.79697	20.59491	13.82716	15.65496	-0.09226	6.745665	15.80402
YIL156W	416.8647	438.0563	478.2195	376.081	407.9013	564.5568	0.016569	403.6156	537.3367
YLR258W	3233.58	3411.499	3341.608	2616.449	2481.976	2313.998	-0.43006	3005.194	2993.106
YFL012W	1.15156	1.977681	0	5.372586	8.065844	0	2.10248	4.49711	2.634003
YER153C	56.42644	79.10723	97.81763	76.11164	88.72429	74.36104	0.035696	64.08382	100.0921
YCL024W	234.9182	325.3285	356.6885	334.8912	387.1605	499.9801	0.41439	254.0867	311.6904
YER180C	336.2555	437.0675	417.948	430.7023	455.1441	472.584	0.189439	489.0607	438.1225
YHR046C	475.5943	561.6614	517.7418	639.3378	619.9177	491.1742	0.170797	458.7052	505.7286
YEL041W	252.1916	319.3955	333.9632	275.7928	247.7366	324.8403	-0.0941	257.4595	331.8844
YBL008W-/	2.30312	0	0	0.895431	0	3.913739	1.062198	2.248555	0
YGL084C	1030.646	995.7623	1031.531	937.5163	974.8149	917.7718	-0.11171	1052.324	1029.895
YMR237W	1974.925	1762.114	2004.767	1805.189	1910.453	2307.149	0.068928	1919.142	1911.408
YPR167C	557.355	716.9093	662.9861	592.7753	560	559.6647	-0.17796	513.7948	460.9506
YDR319C	498.6255	403.4469	404.1152	445.0292	366.4198	344.409	-0.1764	413.7341	420.5625
YHR156C	219.9479	202.7123	192.6711	227.4395	216.6255	179.0536	0.018143	225.9798	190.5262
YGR058W	576.9315	524.0854	565.1685	550.6901	653.3334	673.1631	0.172023	544.1503	610.2107
YNL172W	284.4353	312.4736	350.7602	249.8253	276.5432	433.4466	0.018373	204.6185	410.0265
YLR139C	574.6284	662.5231	638.2847	712.7631	667.1605	742.6319	0.178576	483.4393	760.3489
YER158W-/	2.30312	0.98884	0	0	3.45679	2.935304	0.957342	0	2.634003
YDR081C	976.5228	1031.361	970.2718	1103.171	1090.041	1186.841	0.182627	943.2688	980.7272
YJL044C	1188.41	1150.021	1218.274	1189.132	1234.074	1294.469	0.063859	1136.645	1264.322
YMR316C-I	4.60624	5.933043	2.96417	3.581724	2.304527	3.913739	-0.46248	3.372832	7.024009
YOR378W	141.6419	167.114	182.7905	111.9289	184.3622	198.6222	0.009848	124.7948	144.8702
YOR080W	400.7429	413.3353	413.9958	345.6364	382.5515	500.9586	0.001259	438.4682	340.6644
YOR170W	5.7578	3.955362	6.916398	6.268017	3.45679	6.849043	-0.00484	2.248555	1.756002
YJR045C	18831.46	19554.32	19491.4	18282.91	17955.72	18163.66	-0.08933	18305.49	20335.38
YLR383W	593.0534	510.2417	567.1446	577.553	622.2223	752.4163	0.224867	508.1734	575.0907
YDR288W	523.9598	483.543	480.1956	478.1602	497.7778	499.9801	-0.01147	525.0376	562.7987

YPR057W	625.297	584.4047	472.2912	571.285	521.9754	534.2254	-0.04753	631.8439	544.3607
YDR531W	1619.093	1712.672	1659.935	1715.646	1697.284	1754.333	0.049869	1605.468	1480.31
YMR010W	865.9731	1062.015	972.2479	1035.118	1022.058	1183.906	0.160305	934.2746	913.9991
YDR510C-/	13.81872	14.83261	20.74919	17.90862	16.13169	8.805912	-0.20536	22.48555	23.70603
YLR184W	0	0	0	0.895431	2.304527	0.978435	#DIV/0!	2.248555	0
YER139C	556.2035	595.2819	529.5985	516.6637	565.7614	527.3763	-0.06251	568.8844	485.5346
YKR044W	870.5793	865.2354	740.0546	984.9741	781.2346	741.6535	0.018523	782.4971	792.835
YER111C	712.8156	538.918	615.5594	701.1225	636.0494	882.5481	0.249429	658.8266	741.9109
YOR174W	870.5793	788.1058	793.4096	832.7509	807.7367	697.624	-0.06867	939.896	785.811
YBR055C	909.7324	854.3581	920.869	898.1173	987.4898	1291.534	0.242829	935.3989	1007.945
YPL209C	149.7028	99.87288	130.4235	89.5431	126.749	127.1965	-0.14573	127.0434	104.4821
YEL061C	799.1826	605.1703	819.0991	710.9722	907.9836	1153.575	0.318402	647.5838	934.1931
YHL041W	0	2.966521	7.904455	2.686293	0	1.956869	-1.2273	1.124277	1.756002
YOL039W	35761.69	27551.07	31826.3	35033.74	35704.04	30255.16	0.086145	40581.92	32482.53
YML035C	1220.654	1397.232	1570.022	1505.22	1389.63	1743.571	0.147404	1385.11	1559.33
YEL030C-/	1.15156	2.966521	1.976114	2.686293	1.152263	0	-0.66687	0	1.756002
YLR140W	14.97028	8.899564	6.916398	3.581724	12.6749	6.849043	-0.41404	12.36705	13.17002
YBR095C	665.6016	706.0321	644.2131	755.7438	744.3622	745.5673	0.155762	693.6792	719.9609
YPR173C	3163.335	2895.325	3352.477	3273.696	3109.959	3155.452	0.019485	3135.61	3290.748
YPL062W	11.5156	23.73217	19.76114	32.23552	19.58848	14.67652	0.2737	13.49133	27.21803
YOR073W-/	1.15156	0.98884	0.988057	0	0	3.913739	0.323096	0	1.756002
YPR097W	1168.833	893.9118	1140.218	1037.805	1249.054	1544.948	0.258618	988.2399	1187.935
YNL189W	3667.718	3432.265	3873.183	3804.686	3833.581	4291.415	0.120576	3897.87	3772.771
YDR455C	1.15156	3.955362	4.940284	0.895431	4.609054	0.978435	-0.63208	2.248555	2.634003
YEL053C	726.6343	699.1102	815.1469	820.2148	762.7984	940.2758	0.171233	681.3122	722.5949
YKR037C	626.4486	625.936	582.9535	646.5012	683.2922	578.2549	0.056052	580.1272	557.5307
YMR239C	1125.074	1086.736	1098.719	1370.905	1370.041	1581.15	0.38467	1358.127	1409.192
YMR143W	21267.01	17057.5	19612.93	20526.86	20597.86	19064.8	0.055017	23742.49	19884.09
YBR221W-/	0	0.98884	1.976114	0	3.45679	0.978435	0.580998	0	0
YGR025W	13.81872	7.910723	1.976114	7.163448	5.761317	5.870608	-0.33485	4.49711	1.756002
YHR032C-/	4.60624	2.966521	0	2.686293	0	0	-1.4952	0	0.878001
YMR043W	2611.738	2434.525	2662.813	2663.012	2548.807	2591.874	0.017599	2300.272	2562.007
YBL027W	48990.81	38589.5	44408.21	48762.49	46235.72	45791.72	0.093132	53674.13	46907.21
YGR195W	1254.049	1439.752	1281.51	1641.325	1488.724	1400.14	0.188504	1590.853	1457.482
YNL193W	421.4709	418.2795	379.4138	349.2181	426.3375	412.8994	-0.03681	419.3555	446.0245
YDR267C	808.3951	871.1684	910.9884	930.3528	913.7449	859.0657	0.061389	849.9538	813.029
YKL112W	1083.618	1140.133	1232.107	1115.707	1131.523	1456.889	0.100087	800.4856	1144.913
YKL014C	1813.707	1661.252	1782.455	1997.707	2165.103	2839.418	0.413461	1988.847	2337.239
YDR099W	8215.229	6980.225	8251.263	7712.347	7965.597	7763.879	-0.0003	7681.064	8391.056
YHL012W	196.9167	185.902	186.7427	236.3938	255.8025	259.2852	0.399886	207.9913	270.4243
YGR074W	9.21248	19.77681	21.73725	8.95431	9.218108	8.805912	-0.91094	12.36705	9.658012
YDL240C-/	2.30312	0	0	0	0	0	#NUM!	0	0.878001
YDR061W	971.9166	1018.506	970.2718	848.8686	851.5227	807.2086	-0.23963	834.2139	789.323
YPL234C	3076.968	2860.715	2910.815	3129.531	3229.795	2744.509	0.041042	3520.113	3142.366
YIR032C	177.3402	170.0806	146.2324	125.3603	149.7943	131.1103	-0.28108	220.3584	137.8462
YDR034C-/	5.7578	0	0	0	0	0	#NUM!	0	1.756002
YMR298W	2209.844	2006.357	2018.6	2246.636	2274.568	2035.144	0.072549	2434.061	1972.868
YKR082W	1269.019	1149.033	1398.1	1482.834	1518.683	2096.786	0.417898	1372.743	1548.794
YDL023C	0	0	0	0.895431	0	0	#DIV/0!	1.124277	0
YMR128W	1356.538	1339.879	1433.67	1567.004	1598.189	2075.26	0.34352	1360.376	1662.934
YKL083W	2.30312	0.98884	0	0	1.152263	0	-1.51448	3.372832	0
YER147C	532.0207	577.4828	452.53	524.7226	409.0535	481.3899	-0.14245	547.5231	440.7565
YJR079W	3.45468	1.977681	0.988057	0.895431	2.304527	1.956869	-0.31618	5.621387	3.512004
YGR169C	443.3506	458.822	367.5571	357.277	442.4692	481.3899	0.012902	455.3324	355.5904

YPR099C	3.45468	0.98884	0.988057	0.895431	0	0	-2.60072	1.124277	0
YGR055W	7191.492	10728.92	7733.521	8483.314	7666.009	6244.37	-0.19609	6430.867	6422.578
YDR472W	1110.104	1074.87	1074.018	1038.7	1181.07	1113.459	0.032495	1046.702	1157.205
YNL230C	442.199	499.3644	547.3835	529.1997	464.3622	444.2094	-0.05046	471.0723	466.2186
YBR278W	277.5259	193.8127	210.4561	200.5765	197.0371	201.5576	-0.18637	245.0925	170.3322
YAR019W-/	10.36404	10.87724	11.85668	6.268017	4.609054	12.71965	-0.48816	4.49711	13.17002
YOR294W	3271.582	3207.798	3059.024	3921.092	3558.19	3247.425	0.169387	3667.393	3430.35
YDR327W	63.3358	49.44202	30.62976	37.6081	50.69959	30.33148	-0.27354	62.95954	41.26605
YDL125C	2833.989	2320.808	2710.24	2595.855	2546.502	2342.373	-0.0715	2852.292	2407.479
YFL068W	0	0.98884	0	0	0	0	#NUM!	0	0
YPR110C	4233.134	4485.38	4356.343	4966.06	5087.243	4749.322	0.179058	4799.541	4562.094
YHL002C-/	1.15156	0	0	0	0	0	#NUM!	0	0
YCL076W	0	0.98884	0	0	0	0	#NUM!	0	0
YMR052C-/	0	0	0.988057	0	0	0	#NUM!	0	0
YBL035C	652.9345	669.445	749.9351	763.8027	919.5062	988.2191	0.366422	785.87	772.6409
YGR149W	474.4427	482.5541	442.6495	411.0028	456.2963	419.7485	-0.121	384.5029	353.8344
YLR196W	3292.31	3529.172	3367.298	4229.121	3966.091	3771.866	0.23209	4055.269	4069.535
YFL042C	665.6016	616.0476	631.3683	590.9845	513.9095	608.5864	-0.15892	595.8671	530.3126
YPL026C	718.5734	961.1529	819.0991	571.285	521.9754	529.3332	-0.62295	685.8093	675.1828
YEL029C	479.0489	606.1592	517.7418	602.6251	521.9754	526.3979	0.042609	573.3815	487.2906
YDR374C	16.12184	3.955362	7.904455	8.95431	20.74074	9.784347	0.496619	6.745665	3.512004
YPL164C	231.4635	259.0762	205.5158	220.276	212.0165	225.04	-0.08258	224.8555	221.2563
YJR001W	2105.052	2478.034	2386.157	2505.416	2477.366	2433.367	0.089668	2122.636	2124.763
YMR119W	422.6225	575.5051	526.6343	419.0617	480.4939	462.7996	-0.16248	481.1908	470.6086
YMR088C	955.7947	1080.803	961.3793	1072.726	1065.844	1006.809	0.069245	1015.223	1049.211
YPL001W	695.5422	718.887	679.7831	599.0434	707.4898	630.112	-0.11285	647.5838	633.9168
YLR215C	617.2361	645.7128	643.225	621.4291	649.8766	565.5353	-0.05345	608.2341	574.2127
YGL129C	1199.925	1230.117	1383.28	1306.434	1338.93	1497.984	0.119748	1373.867	1424.996
YMR123W	1875.891	1505.015	1538.404	1640.43	1786.008	1545.927	0.015476	1957.367	1546.16
YJL173C	945.4307	666.4785	803.2902	776.3387	845.7614	786.6615	-0.00385	848.8295	803.371
YKL122C	2553.008	2229.835	2461.25	2649.58	2847.243	2279.753	0.10233	2501.517	2421.527
YOR160W	886.7012	1070.914	1029.555	1092.426	1134.98	1271.965	0.228316	1110.786	1115.061
YLR236C	19.57652	26.69869	7.904455	18.80405	20.74074	9.784347	-0.13531	7.869942	17.56002
YLR135W	333.9524	366.8598	389.2944	352.7998	361.8107	365.9346	-0.01271	293.4364	388.0765
YKL194C	480.2005	519.1412	538.491	479.951	511.605	471.6055	-0.07181	469.948	530.3126
YJR162C	0	0	0	0	2.304527	0.978435	#DIV/0!	0	0
YER006C-/	0	0	0	0	2.304527	0	#DIV/0!	0	0
YPR026W	344.3164	275.8865	355.7005	211.3217	213.1687	236.7812	-0.5615	237.2225	251.1083
YJR087W	0	0	0	0	1.152263	0.978435	#DIV/0!	0	0
YBR056W-/	3.45468	8.899564	4.940284	5.372586	16.13169	0.978435	0.3785	4.49711	3.512004
YKL075C	476.7458	547.8176	480.1956	460.2515	535.8025	558.6862	0.047141	504.8006	458.3166
YAL067W-/	0	1.977681	0	0	0	0.978435	-1.01526	1.124277	0.878001
YEL043W	2333.06	2098.319	2191.51	2252.904	2600.659	3058.587	0.256609	2057.428	2354.799
YER102W	22061.59	18121.49	22267.84	23241.81	23002.64	21812.25	0.124014	25656.01	22848.22
YLR340W	47729.86	37258.52	35291.41	41100.28	39562.97	33657.18	-0.07331	53901.24	41767.39
YGL223C	636.8126	687.2441	709.4248	703.8088	783.5392	708.3867	0.110752	749.8931	642.6968
YER005W	937.3698	1100.579	896.1675	960.7975	964.4445	987.2406	-0.01068	884.8064	962.2892
YGR018C	0	0	0	0.895431	0	0.978435	#DIV/0!	0	0
YEL062W	316.679	412.3465	339.8915	316.0872	311.1111	362.0208	-0.11179	310.3006	278.3263
YDL238C	190.0074	218.5337	222.3128	106.5563	186.6667	165.3555	-0.46014	194.5	184.3802
YGR260W	2101.597	2400.905	2141.119	2347.82	2358.683	2206.37	0.057316	2671.283	2260.853
YGL202W	5369.724	5161.747	5269.307	5269.612	5160.988	4857.928	-0.04755	5483.101	5056.408
YKL147C	0	0	0	1.790862	0	0.978435	#DIV/0!	0	0
YPR143W	1165.379	1200.452	1069.077	1411.199	1239.835	1195.647	0.163343	1387.358	1273.102

YGR240C-/	109.3982	150.3037	104.734	119.0923	106.0082	92.9513	-0.1964	103.4335	101.8481
YJL075C	11.5156	4.944202	3.952227	6.268017	6.913581	3.913739	-0.25582	5.621387	6.146008
YHR030C	1589.153	1791.779	1548.285	1689.678	1795.226	1460.803	0.004819	1611.09	1695.42
YDR059C	290.1931	390.592	304.3215	274.0019	292.6749	242.6518	-0.28355	309.1763	255.4983
YOL102C	710.5125	594.2931	595.7983	607.9977	629.1359	497.0448	-0.13221	640.8382	501.3386
YEL042W	4030.46	4162.029	4161.695	4397.462	4537.614	4537.002	0.124973	4580.306	4461.123
YMR242C	30705.19	24635.97	27773.29	30841.33	30148.97	28362.87	0.10442	34339.93	28866.04
YMR287C	559.6581	508.264	546.3954	544.4221	524.2799	705.4514	0.136206	557.6416	625.1368
YIL001W	348.9227	472.6657	397.1988	347.4272	338.7655	369.8483	-0.20678	344.0289	350.3224
YKL041W	1158.469	1158.921	1167.883	1089.74	1028.971	1133.027	-0.10006	1217.593	1195.837
YDR187C	0	4.944202	3.952227	0.895431	0	0.978435	-2.24721	3.372832	2.634003
YLR222C	1966.864	2301.032	2054.17	2421.245	2420.906	2845.288	0.282107	2245.182	2218.709
YHL016C	120.9138	140.4153	84.97289	90.43853	99.09466	70.4473	-0.41363	92.19075	75.50809
YGR275W	930.4604	746.5745	871.4661	1033.327	974.8149	761.2222	0.119906	991.6127	962.2892
YNR058W	87.51856	111.739	149.1966	131.6284	76.04939	79.25321	-0.28026	69.7052	112.3841
YER173W	343.1649	435.0898	358.6646	397.5714	371.0288	410.9426	0.053098	344.0289	375.7845
YPL243W	3529.531	3089.138	3578.742	3635.45	3597.367	4155.412	0.159341	3552.717	3386.45
YOL127W	38076.33	29659.28	35519.65	38710.38	39460.41	38930.94	0.181547	45713.12	37982.33
YMR074C	1834.435	1490.183	1707.362	1738.032	1850.535	1656.49	0.059832	1876.419	1782.342
YOR096W	33748.77	25130.39	29414.45	32309.84	31302.39	30514.44	0.092295	36323.16	30304.21
YER084W-/	1.15156	1.977681	0	0.895431	0	0	-1.80516	1.124277	0.878001
YKR080W	3306.129	3673.542	3605.419	3904.975	3803.622	3856.011	0.127683	3107.503	2906.184
YJL077C	31.09212	40.54246	34.58199	39.39897	26.50206	24.46087	-0.23322	41.59827	42.14405
YLR445W	26.48588	13.84377	9.880568	12.53603	18.43622	13.69809	-0.16866	17.98844	8.780011
YBR196C-/	23.0312	24.72101	21.73725	23.28121	38.02469	39.13739	0.531515	46.09538	23.70603
YBR064W	0	0	0.988057	2.686293	2.304527	2.935304	3.00395	0	3.512004
YLR425W	460.624	396.525	426.8405	346.5318	437.8601	498.0233	-0.00177	312.5491	489.0466
YLR320W	441.0475	411.3576	510.8254	423.5389	415.9671	492.1527	-0.03381	411.4856	388.0765
YHR195W	935.0667	918.6328	866.5258	794.2473	872.2634	833.6264	-0.12172	906.1676	811.273
YGR214W	36822.28	30729.21	35079.97	36791.47	36361.98	35110.15	0.077075	39870.25	36012.09
YJR017C	1832.132	1499.082	1526.548	1727.286	1603.951	1400.14	-0.03803	1685.292	1523.332
YCR095W-/	17.2734	33.62057	33.59393	11.6406	18.43622	13.69809	-0.94864	14.61561	23.70603
YLR436C	842.9419	919.6216	970.2718	883.7904	820.4116	1008.766	-0.01053	757.763	1000.921
YPR010C	5939.746	6824.977	6658.515	7515.353	7702.881	9119.012	0.325382	7322.419	7643.877
YNL103W-/	0	2.966521	2.96417	1.790862	6.913581	5.870608	1.297229	1.124277	3.512004
YHR049C-/	14.97028	15.82145	19.76114	6.268017	29.95885	29.35304	0.375461	19.11272	24.58403
YML076C	490.5645	554.7395	549.3596	530.9906	551.9342	621.306	0.095869	481.1908	506.6066
YGR273C	4.60624	0	1.976114	4.477155	0	1.956869	-0.03288	4.49711	0
YLR338W	1.15156	0	0	0.895431	0	0	-0.36294	1.124277	0
YPL092W	891.3074	966.0971	901.1078	881.1041	946.0083	709.3652	-0.12106	839.8353	856.051
YBL092W	49393.86	38294.82	45722.33	48785.77	48739.59	45158.68	0.096946	56292.57	47083.69
YGR028W	772.6967	846.4474	769.6963	729.7763	624.5268	595.8667	-0.29271	775.7515	822.687
YFR018C	1345.022	1413.053	1184.68	1395.977	1327.408	1018.551	-0.07542	1288.422	1218.665
YCL065W	1.15156	0	0	0	1.152263	0	0.000881	1.124277	0
YCL067C	2.30312	0	0	0	0	0	#NUM!	0	0
YHR191C	314.3759	225.4556	225.277	341.1592	252.3457	237.7596	0.119643	301.3064	252.8643
YKL099C	1961.107	2087.442	2087.764	2401.546	2501.564	2432.389	0.257523	2248.555	2320.557
YOR334W	376.5601	423.2237	372.4974	365.3359	379.0947	327.7756	-0.12874	378.8815	358.2244
YEL025C	700.1484	691.1995	746.971	675.155	645.2675	732.8476	-0.05855	630.7197	705.9129
YFR049W	626.4486	511.2305	586.9058	462.9378	479.3416	431.4897	-0.32811	559.8902	554.0187
YGL209W	187.7043	200.7346	257.8828	195.204	135.9671	154.5927	-0.412	181.0087	200.1842
YPR021C	540.0816	552.7618	579.0013	586.5073	549.6297	712.3005	0.144865	563.263	582.9927
YGL016W	718.5734	754.4853	865.5378	904.3853	791.605	1164.337	0.290537	851.0781	989.5072
YOR280C	483.6552	381.6924	418.9361	444.1338	437.8601	330.7109	-0.08274	430.5983	447.7805

YBR093C	380.0148	526.0631	371.5094	286.5379	406.749	432.4681	-0.18253	290.0636	324.8604
YOR396W	0	0.98884	0.988057	0.895431	5.761317	0	1.75158	0	0
YIL141W	1.15156	3.955362	1.976114	0	0	4.892174	-0.53389	1.124277	4.390005
YLR028C	5294.873	5606.725	5352.304	5377.959	4951.276	4926.419	-0.09144	5257.121	5273.274
YLR221C	1740.007	1493.149	1632.27	2002.184	1807.901	1761.182	0.195439	1849.436	1958.82
YDR469W	1047.92	863.2577	1007.818	930.3528	1022.058	1001.917	0.017358	969.1272	1112.427
YDR011W	6323.216	6363.188	7438.092	6285.926	6322.47	8422.366	0.063548	6222.876	7550.809
YDL119C	229.1604	217.5449	196.6233	179.9816	208.5597	149.7005	-0.2573	207.9913	201.9402
YPL171C	38.00148	63.28579	42.48644	85.96138	66.83128	41.09426	0.431416	44.9711	49.16806
YER159C	1515.453	1425.908	1436.635	1570.586	1580.905	1292.512	0.02159	1369.37	1468.018
YMR224C	309.7696	340.1611	352.7363	349.2181	314.5679	282.7676	-0.08309	288.9393	329.2504
YGR265W	4.60624	14.83261	10.86863	17.90862	17.28395	34.24522	1.196047	17.98844	10.53601
YHR167W	511.2926	461.7885	480.1956	428.016	543.8684	390.3955	-0.09329	432.8468	431.0985
YLR345W	633.358	661.5342	694.6039	582.0302	518.5186	458.8859	-0.35138	529.5347	587.3827
YKR054C	869.4278	794.0389	820.0872	840.8097	864.1976	951.0385	0.096874	811.7283	845.515
YOR002W	1573.031	1711.683	1661.912	1486.416	1605.103	1470.587	-0.11674	1554.876	1517.186
YBR178W	0	1.977681	3.952227	0	2.304527	1.956869	-0.47668	0	1.756002
YDL074C	2140.75	1931.205	2035.397	2206.342	2363.292	2954.873	0.30105	2021.451	2172.175
YPL099C	342.0133	344.1165	359.6527	352.7998	301.893	281.7892	-0.15926	383.3786	339.7864
YDL135C	1440.601	1080.803	1174.8	1164.06	1217.942	1179.014	-0.05376	1252.445	1272.224
YDL011C	0	0	0	0.895431	0	0	#DIV/0!	0	0
YJL009W	33.39524	24.72101	14.82085	23.28121	28.80659	25.4393	0.088048	31.47977	23.70603
YDR480W	710.5125	630.8802	572.0849	558.749	528.8889	516.6135	-0.2543	487.9364	439.8785
YBR121C	8674.701	8498.095	8200.872	9049.226	8670.783	8410.625	0.04241	9337.124	8606.167
YKR035C	0	0	0	0	0	0.978435	#DIV/0!	0	0
YPR073C	1283.989	1173.754	1439.599	1238.381	1273.251	1382.528	-0.00118	1504.283	1341.586
YOR079C	442.199	431.1344	463.3987	394.8851	382.5515	424.6407	-0.15318	429.474	417.0505
YIL050W	530.8691	568.5832	467.3509	429.8069	492.0165	412.8994	-0.23128	433.9711	442.5125
YDL002C	1090.527	1302.303	1219.262	1224.95	1293.992	1216.194	0.048326	1335.642	1206.373
YDR499W	447.9568	368.8375	448.5778	476.3693	470.1235	512.6998	0.205609	405.8642	457.4386
YLR083C	2686.589	2700.523	3054.084	3193.107	3374.98	3443.112	0.246095	3020.934	3026.47
YDR013W	325.8915	321.3731	345.8199	353.6953	373.3334	343.4306	0.108241	348.526	351.2004
YGL128C	308.6181	273.9088	276.6559	308.0283	286.9136	238.7381	-0.04347	286.6908	284.4723
YPL213W	451.4115	490.4649	461.4225	374.2902	440.1646	367.8915	-0.24717	405.8642	489.9246
YLL067C	8.06092	4.944202	3.952227	4.477155	3.45679	6.849043	-0.19797	0	5.268006
YLR166C	898.2168	783.1616	891.2273	825.5874	922.963	1156.51	0.175338	909.5405	864.8311
YPR153W	58.72956	89.98448	49.40284	54.62129	62.22223	60.66295	-0.15848	56.21387	63.21608
YNL091W	2672.771	2286.199	3165.734	2804.49	2562.634	3005.751	0.043408	2547.613	2994.862
YPL236C	274.0713	282.8084	255.9067	266.8384	215.4733	237.7596	-0.17474	276.5723	221.2563
YKL046C	2265.118	2377.172	2353.551	2436.468	2201.975	2131.031	-0.04745	2358.734	2309.143
YJL178C	2413.67	2259.5	2393.074	2589.587	2287.243	2425.54	0.047421	2799.451	2424.161
YPL096W	682.875	696.1437	626.428	553.3764	564.6091	557.7078	-0.25917	657.7023	549.6287
YDR110W	821.0622	921.5993	949.5226	919.6077	932.1811	985.2838	0.075626	955.6359	958.7772
YGL031C	29622.73	22892.64	27231.83	28459.48	27221.07	26296.41	0.039785	33029.02	27921.31
YHR074W	1969.167	1742.337	1731.076	1597.449	1601.646	1779.773	-0.12847	1814.584	1720.004
YPL235W	2470.096	2648.115	2386.157	2568.992	2563.786	2231.81	-0.02713	2595.957	2498.791
YIL004C	556.2035	503.3198	535.5268	573.9713	596.8725	498.0233	0.065267	654.3295	506.6066
YPL090C	18362.77	14415.32	17766.25	19869.61	17432.59	16934.75	0.101727	19696.22	18240.47
YCL011C	2890.415	3465.886	2951.326	3283.546	3189.465	2775.819	-0.00914	3252.535	3024.714
YMR097C	445.6537	503.3198	418.9361	425.3297	434.4033	420.7269	-0.09531	455.3324	432.8545
YFR022W	198.0683	272.92	232.1934	209.5309	214.321	221.1262	-0.12465	238.3468	223.8903
YBR111C	3004.42	2536.376	2695.419	2857.32	2800	2558.607	-0.00356	3316.619	2719.169
YFL024C	824.5169	819.7487	802.3021	830.96	876.8725	883.5265	0.08295	623.974	826.199
YDL142C	408.8038	409.3799	429.8047	367.1267	357.2017	312.1207	-0.26796	328.289	320.4704

YLR288C	363.8929	381.6924	285.5484	351.009	292.6749	275.9186	-0.16515	297.9335	302.0324
YNR049C	648.3282	649.6682	657.0578	643.8149	615.3087	518.5704	-0.1372	679.0636	615.4788
YJR076C	1906.983	1918.35	2009.708	2255.591	2166.255	2393.251	0.223991	2051.806	2072.083
YPR036W	15455.09	13653.91	14425.63	15496.33	15191.44	15257.71	0.07776	14620.1	13769.69
YDR446W	14.97028	14.83261	22.72531	8.058879	16.13169	16.63339	-0.36368	16.86416	14.92602
YKL205W	1479.755	1487.216	1538.404	1630.58	1701.893	2009.705	0.245781	1594.225	1716.492
YLR251W	233.7667	247.2101	234.1695	159.3867	131.358	138.9377	-0.73497	231.6012	220.3783
YIL156W-I	304.0118	230.3998	289.5006	183.5634	217.7778	251.4577	-0.33585	311.4249	258.1323
YKL081W	35280.34	34055.66	33985.2	34704.22	33873.09	31364.7	-0.04797	36252.33	34569.54
YOL075C	368.4992	395.5362	374.4735	410.1074	443.6214	549.8803	0.301996	380.0058	495.1926
YGL042C	217.6448	147.3372	151.1727	119.0923	150.9465	100.7788	-0.47709	223.7312	136.0902
YDL203C	633.358	680.3222	725.2337	681.423	732.8396	778.834	0.105169	598.1156	680.4508
YBR068C	2254.754	2809.296	2330.826	2090.831	1945.021	1765.096	-0.35024	2137.251	2087.887
YLR446W	343.1649	515.1859	401.1511	384.1399	381.3992	347.3443	-0.17855	363.1416	368.7605
YMR122C	101.3373	97.8952	95.84151	79.69336	85.2675	82.18852	-0.25569	83.19653	113.2621
YDL237W	1326.597	1399.209	1350.674	1275.989	1260.576	1244.569	-0.1085	1450.318	1251.152
YHR128W	3995.913	4092.811	3928.514	4521.031	4908.642	4140.736	0.175359	4751.197	4339.959
YBL090W	1135.438	970.0525	1052.281	1000.196	956.3787	995.0681	-0.09739	1209.723	995.6532
YJL085W	526.2629	554.7395	542.4432	548.8992	568.0659	603.6942	0.083902	541.9017	569.8227
YLR342W	11221.95	10411.5	11222.35	11503.6	11976.63	14460.29	0.207591	11205.67	11989.1
YER050C	1247.139	1145.077	1114.528	1156.897	1101.564	1025.4	-0.09474	1380.613	1101.013
YIR042C	0	0	0	0.895431	2.304527	0	#DIV/0!	0	0
YOR037W	374.257	381.6924	328.0349	372.4993	289.2181	328.7541	-0.13016	317.0462	348.5664
YKR002W	1032.949	1066.959	1019.675	1017.21	993.2511	949.0817	-0.07598	1032.087	989.5072
YOR029W	2.30312	3.955362	4.940284	1.790862	6.913581	1.956869	-0.07095	0	7.90201
YEL052W	959.2494	952.2533	966.3196	862.3001	909.1359	808.1871	-0.15782	1061.318	1007.067
YHR151C	482.5036	539.9069	491.0642	425.3297	485.1029	466.7134	-0.13618	455.3324	453.0486
YJL136W-/	2.30312	0.98884	0.988057	0.895431	0	0	-2.25696	0	0
YOR279C	305.1634	293.6856	286.5365	359.0678	307.6543	349.3012	0.198556	322.6676	278.3263
YBR058C-/	973.0681	662.5231	879.3706	848.8686	804.2799	784.7046	-0.04493	1028.714	876.2451
YOR158W	1423.328	1436.785	1465.288	1249.126	1258.272	1369.809	-0.15782	1364.873	1456.604
YKL033W	338.5586	398.5027	373.4855	307.1328	398.6832	435.4034	0.039306	330.5376	364.3704
YOL066C	1076.709	1297.359	1307.199	1299.27	1143.045	1246.526	0.002966	1303.038	1088.721
YDR131C	255.6463	220.5114	238.1217	179.9816	215.4733	199.6007	-0.26346	221.4827	203.6962
YBR007C	660.9954	519.1412	532.5626	596.3571	560	770.0281	0.169624	554.2688	597.0407
YGL201C	807.2435	825.6818	919.8809	919.6077	933.3334	1179.992	0.24863	862.3208	842.881
YDR219C	540.0816	539.9069	561.2163	565.9124	578.4363	602.7158	0.090178	441.841	579.4807
YBL067C	1111.255	1026.416	1076.982	1134.511	1228.313	1244.569	0.166293	989.3642	1223.055
YLR054C	19.57652	14.83261	24.70142	33.13095	21.89301	20.54713	0.354419	16.86416	26.34003
YLR277C	610.3268	671.4227	622.4758	592.7753	591.1112	715.2358	-0.00387	553.1445	621.6248
YFL022C	7485.14	6793.334	7191.078	7759.805	7757.038	8151.34	0.140657	7359.52	7295.311
YMR316C-/	1.15156	0.98884	2.96417	0	5.761317	2.935304	0.768665	0	1.756002
YGR212W	208.4323	247.2101	255.9067	223.8578	235.0617	252.4362	-0.00039	205.7428	271.3023
YBR165W	418.0163	526.0631	422.8883	373.3947	394.0741	362.9993	-0.27406	417.1069	446.0245
YML079W	1297.808	1222.207	1199.501	1235.695	1126.914	1043.011	-0.1272	1305.286	1272.224
YGR061C	5906.351	6738.948	6242.543	7028.238	7000.001	8023.165	0.223413	6384.772	6750.95
YML081W	1377.266	1255.827	1318.068	1429.108	1485.268	1746.506	0.238326	1371.619	1486.456
YMR175W-/	1.15156	5.933043	3.952227	1.790862	3.45679	2.935304	-0.43163	2.248555	1.756002
YDR508C	3368.313	4721.713	3939.383	3526.207	3411.852	3099.681	-0.26113	3185.078	3360.11
YER137W-/	0	2.966521	0.988057	0	2.304527	0	-0.77905	0	0
YOR376W	23.0312	11.86609	14.82085	14.3269	11.52263	15.65496	-0.2605	17.98844	21.95003
YOR008W-I	29.94056	19.77681	18.77308	19.69948	23.04527	31.30991	0.112688	19.11272	32.48604
YGR134W	650.6314	630.8802	598.7624	497.8597	548.4774	615.4354	-0.17822	648.7081	590.0167
YBL026W	978.8259	883.0345	957.4271	949.1569	927.5721	1002.896	0.03055	1161.379	1044.821

YGL197W	825.6685	770.3067	905.0601	756.6392	825.0206	1167.273	0.136346	658.8266	942.0952
YNL323W	1137.741	1134.2	1142.194	1154.211	1251.358	1319.908	0.125905	1192.858	1169.497
YBL018C	284.4353	299.6187	317.1662	311.61	300.7408	256.3499	-0.05302	311.4249	389.8325
YKL006C-/	589.5987	531.9962	523.6701	548.8992	515.0618	494.1095	-0.07856	645.3353	567.1887
YMR232W	52.97176	47.46434	75.09232	49.24871	71.44034	43.05113	-0.1003	60.71098	49.16806
YJL126W	412.2585	436.0786	347.796	351.009	330.6996	428.5544	-0.10748	357.5202	357.3464
YKL139W	550.4457	571.5498	577.0252	562.3307	651.0289	726.977	0.191604	584.6243	606.6987
YER175C	305.1634	315.4401	305.3096	239.9755	265.0206	258.3068	-0.27862	306.9278	272.1803
YPL069C	411.1069	432.1233	439.6853	393.9897	449.3828	343.4306	-0.11235	432.8468	365.2484
YER052C	2092.384	2400.905	2080.848	2104.263	1989.959	1875.659	-0.1391	1973.107	2112.471
YEL008W	17.2734	30.65405	15.80891	21.49034	25.3498	14.67652	-0.05114	25.85838	12.29202
YNL135C	12246.84	8801.669	10900.24	11469.58	10865.84	10154.2	0.024219	13520.56	11198.03
YDL218W	59.88112	79.10723	70.15203	69.84362	53.00412	84.14539	-0.01489	66.33237	70.24009
YDL146W	299.4056	373.7817	305.3096	282.9562	281.1523	306.2501	-0.16896	338.4075	292.3744
YJL045W	35.69836	32.63173	17.78502	41.18983	33.41564	10.76278	-0.01257	24.7341	22.82803
YLR447C	6209.211	6031.927	6170.415	6231.305	6045.926	6084.885	-0.00388	6614.124	5615.695
YDR108W	452.5631	492.4425	550.3477	485.3236	481.6461	516.6135	-0.0114	447.4624	475.8766
YPR036W-/	14483.17	17474.79	13043.34	7600.419	6479.177	4603.535	-1.26823	9664.289	8521
YBR160W	1523.514	1327.024	1304.235	1397.768	1312.428	1240.655	-0.07261	1294.043	1264.322
YDR083W	2024.442	1948.016	1820.001	2195.597	2378.272	2336.502	0.254587	2307.017	2155.493
YGR252W	830.2747	810.8492	780.5649	781.7113	721.3169	872.7638	-0.0276	729.6561	859.5631
YNL073W	665.6016	805.905	699.5442	595.4616	622.2223	628.1551	-0.23412	727.4075	781.421
YFR012W-/	10.36404	6.921883	10.86863	3.581724	8.065844	2.935304	-0.94909	5.621387	5.268006
YDL108W	461.7755	515.1859	471.3031	481.7419	458.6009	437.3603	-0.07206	426.1012	388.0765
YDR371W	505.5348	412.3465	408.0675	518.4546	488.5597	503.8939	0.188391	426.1012	434.6105
YGL107C	950.0369	1085.747	1053.269	1122.871	1069.3	1112.48	0.097334	1113.035	1208.129
YJL119C	0	6.921883	1.976114	0.895431	2.304527	0.978435	-1.09053	2.248555	2.634003
YHL046C	2.30312	0	0	0.895431	2.304527	0	0.474463	0	1.756002
YLR403W	990.3415	1081.791	1279.534	1113.916	1125.761	1361.003	0.103391	1054.572	1159.839
YOR150W	1425.631	1451.618	1330.913	1357.473	1230.617	1126.178	-0.18011	1603.22	1473.286
YEL020W-/	99.03415	88.99564	102.7579	127.1512	78.35392	89.03756	0.01851	110.1792	106.2381
YDL158C	62.18424	57.35275	44.46256	45.66698	41.48148	46.96487	-0.29024	57.33815	55.31407
YHR175W-/	74.8514	52.40854	55.33118	78.79793	54.15638	40.11582	-0.07726	68.58093	55.31407
YPL010W	3035.512	2549.231	2631.195	2845.68	2737.778	2558.607	-0.01303	3105.254	2503.181
YPR096C	0	5.933043	5.928341	3.581724	4.609054	20.54713	1.276683	3.372832	6.146008
YOR288C	742.7562	620.9918	688.6756	655.4555	772.0165	645.7669	0.014558	721.7861	658.5008
YDL175C	343.1649	391.5808	380.4019	371.6039	428.642	393.3308	0.098057	341.7804	336.2744
YIL098C	738.1499	719.8758	628.4041	601.7296	566.9136	544.0097	-0.2848	846.5809	655.8668
YLR029C	54594.31	46357.83	50648.78	55770.13	51751.61	48547	0.041903	59315.76	51265.6
YGL200C	8030.979	6352.311	7764.151	7433.868	7793.91	7123.005	0.013185	8329.772	7316.383
YJL181W	252.1916	261.0539	238.1217	208.6354	275.391	262.2205	-0.00987	219.2341	257.2543
YIL153W	869.4278	1096.624	835.8961	932.1437	843.4569	673.1631	-0.19438	869.0665	874.4891
YLR406C-/	5.7578	7.910723	2.96417	6.268017	1.152263	2.935304	-0.68361	2.248555	6.146008
YPL083C	513.5957	454.8666	546.3954	527.4089	518.5186	568.4706	0.091814	485.6879	595.2847
YGR283C	1091.679	1084.758	1099.707	1141.675	1144.198	1130.092	0.060294	1084.928	1047.455
YBR285W	290.1931	262.0427	341.8677	216.6943	262.7161	247.544	-0.29858	342.9046	331.0064
YNL326C	320.1337	417.2907	372.4974	440.5521	361.8107	305.2716	-0.00298	342.9046	338.0304
YHR012W	997.2509	931.4877	956.439	1043.177	955.2264	898.2031	0.005704	1062.442	952.6312
YJL209W	654.086	741.6303	715.3531	646.5012	630.2881	759.2653	-0.0522	603.737	741.0329
YGL172W	1860.921	1960.871	1730.088	1840.111	1866.667	1944.15	0.025512	1886.538	1855.216
YOR231W	610.3268	627.9137	590.858	550.6901	627.9836	672.1846	0.017062	554.2688	652.3548
YER070W	4077.674	3968.217	4665.604	4383.135	4774.98	5757.11	0.230652	3941.717	4372.445
YDR351W	598.8112	653.6235	856.6453	742.3123	693.6626	875.6991	0.132324	702.6734	755.9589
YDR313C	644.8736	672.4115	630.3803	569.4941	587.6544	468.6702	-0.26058	726.2833	561.0427

YGL171W	1334.658	1583.134	1463.312	1738.927	1813.663	1679.972	0.256223	1522.272	1691.908
YNL269W	20.72808	18.78797	8.892511	10.74517	12.6749	7.827478	-0.63152	2.248555	15.80402
YNR039C	581.5378	658.5677	649.1533	627.6971	689.0536	590.9746	0.014033	610.4827	628.6488
YKL206C	830.2747	822.7152	740.0546	777.2341	831.9342	651.6375	-0.08201	749.8931	676.0608
YBR142W	2095.839	1892.641	2241.901	2639.731	2652.511	3178.934	0.443242	2453.173	2696.341
YER030W	1971.471	1552.479	1742.932	1763.999	1684.609	1687.8	-0.03619	1995.593	1682.25
YEL059W	14.97028	23.73217	16.79697	7.163448	17.28395	15.65496	-0.46879	22.48555	3.512004
YLR037C	72.54828	106.7948	68.17592	87.75224	47.2428	40.11582	-0.49927	51.71676	75.50809
YBL040C	1561.515	1434.807	1475.169	1550.887	1704.198	1412.86	0.062031	1549.254	1453.97
YHR052W	3668.87	3384.801	3188.459	3869.157	4127.408	4125.081	0.24307	4467.879	3615.608
YBL094C	0	0	0.988057	0.895431	0	0	-0.14201	0	0.878001
YDL141W	605.7205	649.6682	674.8428	623.22	538.107	479.433	-0.23441	589.1214	569.8227
YLR002C	1652.489	1700.806	1841.738	2179.479	2299.918	2748.423	0.476428	1967.486	2108.081
YDR501W	385.7726	406.4134	406.0914	274.0019	330.6996	367.8915	-0.30105	313.6734	258.1323
YKL123W	0	0	0.988057	0	0	0.978435	-0.01412	1.124277	0
YMR138W	312.0727	309.5071	345.8199	364.4404	391.7696	284.7245	0.105695	400.2428	303.7884
YPR047W	523.9598	620.9918	629.3922	531.886	541.5638	521.5057	-0.15377	725.159	568.0667
YLR382C	687.4813	728.7754	658.0458	656.3509	626.8313	788.6184	-0.00174	548.6474	838.491
YKL030W	0	0	0	0.895431	1.152263	0.978435	#DIV/0!	0	0.878001
YOL121C	18851.04	14737.68	17216.89	18744.95	17900.41	17089.34	0.080867	20196.52	17927.03
YHR086W-/	4.60624	8.899564	5.928341	5.372586	9.218108	0	-0.41355	8.99422	7.90201
YIL084C	226.8573	301.5963	231.2053	188.0405	251.1934	307.2285	-0.02528	205.7428	300.2764
YBR088C	260.2525	203.7011	231.2053	251.6161	281.1523	226.0184	0.126352	313.6734	219.5003
YKL166C	1532.726	1446.674	1425.766	1644.907	1472.593	1519.509	0.073998	1413.217	1459.238
YER116C	342.0133	366.8598	441.6614	402.0485	399.8354	483.3467	0.159723	385.6272	403.0025
YGL041W-/	17.2734	17.79913	34.58199	15.22233	27.65432	17.61182	-0.20356	19.11272	21.95003
YNL161W	1161.924	1315.158	1380.315	1290.316	1308.971	1725.959	0.165155	992.737	1310.856
YDR495C	593.0534	406.4134	555.2879	417.2709	520.8231	545.9666	-0.06714	415.9827	486.4126
Q0130	57.578	94.92868	43.4745	41.18983	23.04527	15.65496	-1.29463	83.19653	57.94807
YER129W	1259.807	1257.805	1395.136	1470.298	1418.436	1771.945	0.252358	1327.772	1558.452
YMR100W	464.0787	536.9404	497.9806	453.9835	530.0412	542.0528	0.025828	509.2977	538.2147
YER068W	1365.75	1269.671	1416.873	1292.107	1485.268	1484.285	0.072676	1265.936	1321.392
YJR014W	5011.589	4229.271	4589.524	4821.001	4662.058	4179.873	-0.01757	5149.191	4731.548
YDR163W	827.9716	825.6818	885.2989	890.0584	708.642	709.3652	-0.13755	812.8526	865.7091
YLR272C	788.8186	644.724	748.9471	766.489	750.1235	945.1679	0.173727	638.5896	778.787
YER171W	948.8854	1002.684	966.3196	1115.707	1048.56	1184.884	0.19887	992.737	958.7772
YIL012W	23.0312	14.83261	9.880568	3.581724	14.97943	15.65496	-0.48066	16.86416	14.92602
YDR532C	295.9509	350.0495	271.7156	300.8648	394.0741	345.3875	0.180916	312.5491	344.1764
YMR111C	479.0489	384.6589	516.7537	376.081	447.0782	409.9641	-0.16283	346.2775	461.8286
YDR176W	1059.435	900.8336	1161.955	1087.053	1163.786	1236.741	0.159653	914.0376	1148.425
YMR245W	74.8514	90.97332	80.0326	88.64767	100.2469	52.83547	-0.02442	76.45087	86.92211
YER001W	3603.231	3528.183	3622.216	3927.36	4092.84	5265.936	0.305098	3527.983	3730.627
YNL131W	3953.305	3957.339	3868.242	3854.831	3703.375	3397.125	-0.10457	4456.636	3917.641
YDR362C	601.1143	625.936	698.5562	700.2271	646.4198	622.2845	0.0321	566.6358	613.7228
YPL207W	2383.729	2732.166	2606.494	2962.981	2755.062	3048.803	0.183011	2664.538	3037.884
YGL072C	1.15156	0	1.976114	0	0	0.978435	-1.67654	0	3.512004
YGR064W	3.45468	4.944202	0.988057	1.790862	1.152263	1.956869	-0.93787	3.372832	0.878001
YMR288W	460.624	540.8957	557.264	539.0495	594.5679	808.1871	0.316977	490.185	550.5067
YDR268W	469.8365	579.4605	478.2195	444.1338	488.5597	383.5464	-0.21477	591.37	460.9506
YJL022W	0	0	0.988057	1.790862	0	0.978435	1.486854	0	0
YCL005W	603.4174	668.4561	597.7744	575.7621	678.6832	505.8507	-0.08695	608.2341	603.1867
YDL061C	8434.025	6926.827	7499.351	8109.023	7963.293	7820.629	0.063746	10424.3	9001.267
YKR094C	22951.74	18074.03	20579.25	21812.7	22266.34	20297.63	0.06349	25381.69	22488.24
YNL223W	186.5527	136.46	106.7101	105.6609	137.1194	90.01599	-0.36877	134.9133	140.4802

YER063W	4040.824	3417.433	3860.338	3841.399	3524.774	3630.971	-0.04157	3670.766	3729.749
YOR322C	1169.985	1244.95	1179.74	1109.439	1054.321	1166.294	-0.11032	997.2341	1287.15
YLR131C	798.031	826.6706	799.338	889.163	783.5392	872.7638	0.070516	674.5665	879.7571
YIL124W	2328.454	2077.554	2272.531	2177.688	2344.856	2114.397	-0.00901	2363.231	2088.765
YMR184W	1058.284	839.5255	997.9374	890.0584	922.963	801.338	-0.14748	928.6532	802.493
YDL177C	459.4724	533.9738	488.1001	497.8597	441.3169	393.3308	-0.15296	423.8526	477.6326
YKR079C	1264.413	1148.044	1239.023	1320.761	1379.259	1606.59	0.238071	1346.884	1249.396
YHR025W	5672.584	5316.995	5529.166	6122.957	5859.26	5161.243	0.053554	5801.272	5728.079
YPL212C	2746.47	3233.508	2777.428	3141.172	3278.19	3406.91	0.16614	2693.769	3114.27
YPL037C	21509.99	18826.53	20631.61	21968.5	21860.74	19837.76	0.062491	24210.19	21405.67
YIL020C-/	1.15156	0.98884	2.96417	1.790862	3.45679	1.956869	0.497113	1.124277	6.146008
YDR060W	4901.039	4492.302	5512.369	5828.361	6431.935	8080.892	0.448539	5714.702	6152.154
YPL255W	498.6255	476.6211	493.0404	480.8465	473.5803	493.1311	-0.02051	424.9769	442.5125
YNL088W	1631.76	1277.582	1626.342	1565.213	1665.021	1927.516	0.185422	1425.584	1478.554
YBL029W	594.2049	637.8021	552.3238	695.7499	622.2223	570.4274	0.081781	522.789	547.8727
YHR006W	626.4486	570.5609	608.643	499.6505	566.9136	589.0177	-0.12518	517.1676	604.9427
YIL147C	989.19	892.9229	1079.946	939.3071	1019.753	1170.208	0.079225	974.7486	1103.647
YOL048C	148.5512	130.5269	139.316	77.9025	69.13581	89.03756	-0.82561	136.0376	83.4101
YOR316C	596.508	783.1616	662.9861	569.4941	585.3498	534.2254	-0.27422	637.4653	654.1108
YPR111W	297.1025	389.6031	337.9154	368.9176	329.5474	368.8699	0.058922	321.5434	320.4704
YHR213W-I	102.4888	80.09608	55.33118	113.7197	77.20165	84.14539	0.209329	100.0607	61.46008
YER123W	961.5525	1049.16	1045.364	985.8696	1162.634	1270.987	0.162101	948.8902	1132.621
YER105C	1649.034	1461.506	1846.678	1781.908	1746.831	2300.3	0.233727	1696.535	1752.49
YPL007C	358.1351	392.5697	377.4377	386.8262	362.963	432.4681	0.067595	326.0405	359.1024
YOR335C	5102.562	5748.129	5742.586	5882.086	5632.264	6236.543	0.097293	5660.737	6004.649
YPR189W	915.4902	852.3805	1062.161	871.2544	978.2717	1215.216	0.114947	923.0318	910.4871
YOL062C	1497.028	1551.491	1449.479	1451.494	1455.309	1367.852	-0.07348	1452.567	1468.896
YPR150W	2.30312	0	0	0	5.761317	0	1.322809	2.248555	1.756002
YOR341W	5906.351	6068.514	6565.638	6746.177	7327.243	9794.131	0.36437	6790.636	7543.785
YGR015C	237.2213	260.065	194.6472	222.0669	253.498	182.9673	-0.07138	204.6185	201.9402
YHR134W	538.9301	512.2193	496.0045	496.9642	367.572	416.8132	-0.27195	480.0665	467.0966
YBL080C	271.7681	318.4066	255.9067	250.7207	191.2757	343.4306	-0.10732	233.8497	302.0324
YPL165C	211.887	220.5114	159.0771	171.9228	194.7325	173.1829	-0.13179	214.737	134.3342
YOR038C	584.9924	571.5498	651.1294	574.8667	744.3622	802.3165	0.230983	652.0809	676.0608
YMR064W	768.0905	801.9496	939.642	702.9134	803.1276	878.6344	-0.07371	730.7804	922.7791
YGR098C	693.2391	582.427	756.8515	713.6585	708.642	952.017	0.224245	562.1387	726.1069
YGR099W	525.1113	508.264	529.5985	545.3175	640.6585	692.7318	0.265447	463.2023	600.5527
YPR171W	968.4619	878.0903	1024.615	852.4503	969.0536	991.1544	-0.0297	919.659	842.881
YLR385C	8.06092	17.79913	18.77308	14.3269	16.13169	35.22365	0.557388	12.36705	18.43802
YDL224C	1336.961	1323.068	1324.984	1281.362	1402.305	1462.76	0.057284	1371.619	1299.442
YDR450W	30915.93	23317.85	27290.13	29153.44	28069.14	27232.77	0.050966	34461.35	28262.85
YPR197C	2.30312	0	1.976114	0	1.152263	0.978435	-1.00603	0	1.756002
YBL012C	1.15156	13.84377	3.952227	6.268017	4.609054	5.870608	-0.17805	3.372832	6.146008
YKL214C	1038.707	871.1684	932.7256	1038.7	1017.449	902.1168	0.05754	1061.318	1058.869
YJR134C	996.0993	905.7778	1102.671	1053.027	1004.774	1315.995	0.167224	905.0434	1151.937
YIL086C	3.45468	5.933043	1.976114	2.686293	8.065844	7.827478	0.709271	7.869942	1.756002
YHR193C	27539.56	21736.69	25525.46	27580.17	25209.22	23057.79	0.020024	29902.41	26107.36
YNL087W	3759.843	3109.903	4175.528	3913.034	4070.947	5410.744	0.278236	3841.656	4095.875
YFR043C	343.1649	414.3241	324.0826	361.7541	400.9877	279.8323	-0.05298	350.7746	289.7404
YKL170W	4.60624	0.98884	1.976114	0.895431	2.304527	0	-1.24247	2.248555	0.878001
YMR101C	14.97028	25.70985	32.60588	13.43147	28.80659	31.30991	0.005148	30.35549	30.73004
YJR151W-/	0	0.98884	0.988057	0.895431	0	0	-1.14258	0	0
YDR416W	469.8365	535.9515	496.9926	468.3104	521.9754	621.306	0.100852	498.0549	480.2666
YDR304C	5778.528	4748.412	5391.826	5565.999	5340.741	4854.015	-0.01439	6291.457	5442.729

YAL047C	392.6819	340.1611	367.5571	374.2902	373.3334	408.9857	0.071873	375.5087	335.3964
YGR031W	422.6225	581.4382	434.745	377.8719	347.9836	325.8188	-0.45218	433.9711	370.5165
YBR107C	548.1425	660.5454	601.7266	527.4089	550.7819	500.9586	-0.19717	598.1156	527.6786
YOR252W	1513.15	1255.827	1339.805	1618.044	1508.313	1475.48	0.163499	1606.593	1529.478
YBL066C	370.8023	444.9782	391.2705	332.2049	379.0947	436.3819	-0.07276	346.2775	450.4146
YGR024C	1342.719	1212.318	1083.898	1118.393	1175.309	988.2191	-0.14898	1241.202	1151.937
YPL125W	890.1558	817.771	947.5465	872.1498	1017.449	1255.332	0.244059	927.5289	1027.261
YKL222C	216.4933	212.6007	229.2292	182.6679	214.321	200.5791	-0.13969	176.5116	232.6703
YPL127C	3793.238	3596.413	3612.336	3617.541	3646.914	3317.872	-0.05611	3327.861	3255.628
YKL196C	3615.898	3041.673	3335.68	3399.056	3213.663	2843.331	-0.07972	3458.278	3067.736
YDL173W	684.0266	981.9186	772.6604	679.6321	707.4898	717.1926	-0.21271	648.7081	743.6669
YGR232W	1024.888	904.789	1053.269	959.0066	812.3457	897.2246	-0.16067	1023.093	1007.067
YMR049C	3598.625	3677.498	4049.057	4515.659	5049.219	5825.6	0.442504	4411.665	4605.994
YKL033W-/	2183.358	1774.969	1973.149	1918.909	1935.803	1940.236	-0.0336	2313.763	1827.12
YMR284W	234.9182	227.4333	257.8828	194.3085	197.0371	200.5791	-0.28305	200.1214	232.6703
YMR108W	5661.069	6107.079	5569.676	6039.682	5876.544	6195.449	0.062997	5582.038	6045.915
YMR054W	1968.016	1843.199	2107.525	1891.15	1935.803	2512.62	0.099096	1925.887	1964.088
YLR067C	423.7741	494.4202	490.0762	455.7744	482.7984	625.2198	0.151125	464.3266	539.0927
YKR101W	293.6478	320.3843	265.7873	256.0933	213.1687	272.0049	-0.24721	267.578	253.7423
YKL074C	847.5481	846.4474	752.8993	816.6331	772.0165	849.2813	-0.00529	816.2254	790.201
YKR010C	743.9077	584.4047	681.7592	676.9459	730.535	755.3516	0.105675	644.211	704.1569
YJL067W	16.12184	12.85493	19.76114	14.3269	17.28395	8.805912	-0.27009	20.23699	14.04802
YBR201W	331.6493	294.6744	294.4409	276.6882	304.1976	340.4953	0.000965	314.7977	327.4944
YIR008C	639.1158	654.6124	580.9774	627.6971	583.0453	585.104	-0.062	630.7197	633.0388
YBR116C	1.15156	2.966521	0	0	1.152263	0	-1.8375	1.124277	1.756002
YAL016C-I	6.90936	3.955362	4.940284	4.477155	6.913581	5.870608	0.127163	4.49711	5.268006
YOL035C	1.15156	2.966521	0	1.790862	0	0.978435	-0.57245	2.248555	0
YJL064W	0	0	0.988057	0	1.152263	1.956869	1.653846	0	0
YJR135C	117.4591	135.4711	135.3638	180.8771	127.9012	148.7221	0.236623	133.789	134.3342
YGR035C	1879.346	2000.424	1535.44	1601.031	1134.98	851.2382	-0.59414	1615.587	1260.81
YFL010W-/	29.94056	27.68753	21.73725	23.28121	19.58848	17.61182	-0.39201	21.36127	21.95003
YMR102C	836.0325	833.5925	824.0394	825.5874	736.2964	907.009	-0.0144	758.8873	844.637
YKL141W	3021.693	3181.1	3098.546	2658.535	2737.778	2575.24	-0.22258	3454.905	3017.69
YDL080C	703.6031	818.7599	838.8602	928.562	803.1276	833.6264	0.119602	583.5	794.591
YDR128W	918.9448	919.6216	892.2153	851.5549	932.1811	957.8876	0.005717	790.3671	907.8531
YHR079C-/	4.60624	0.98884	5.928341	0	6.913581	0	-0.73706	2.248555	1.756002
YLR262C	330.4977	297.641	356.6885	305.342	298.4362	306.2501	-0.11396	330.5376	335.3964
YCR026C	1293.202	1202.43	1248.904	1176.596	1234.074	1307.189	-0.01031	1173.746	1203.739
YDL211C	147.3997	221.5003	206.5039	192.5177	268.4774	195.6869	0.19062	209.1156	179.9902
YGR153W	63.3358	62.29695	54.34313	46.56241	50.69959	61.64139	-0.17965	64.08382	57.94807
YGR069W	17.2734	8.899564	4.940284	8.95431	25.3498	10.76278	0.534539	4.49711	23.70603
YBR036C	2594.465	3074.305	2832.759	2743.601	2602.963	2394.23	-0.13524	2789.332	2700.731
YDR010C	34.5468	38.56478	17.78502	12.53603	13.82716	17.61182	-1.04754	24.7341	32.48604
YKL022C	662.147	736.6861	698.5562	664.4098	591.1112	713.2789	-0.09128	583.5	631.2828
Q0250	2.30312	0	5.928341	0	0	0.978435	-3.0726	0	1.756002
YJL195C	0	0	2.96417	0	0	0.978435	-1.59908	0	1.756002
YHR117W	1598.365	1763.102	1698.47	1685.201	1660.412	1785.643	0.020193	1475.052	1593.572
YJL093C	572.3253	649.6682	546.3954	524.7226	550.7819	565.5353	-0.10783	514.9191	571.5787
YIL047C	1425.631	2204.125	1942.52	2048.746	2018.766	2576.219	0.253727	1667.304	1981.648
YDR087C	2838.595	2865.66	2584.757	3259.369	3172.181	2692.652	0.138498	3339.104	2880.722
YDL196W	4.60624	15.82145	7.904455	7.163448	1.152263	7.827478	-0.81151	4.49711	9.658012
YGR077C	657.5407	619.0141	579.0013	641.1286	653.3334	668.2709	0.081012	612.7312	605.8207
YGR270W	1583.395	1422.941	1781.466	1606.403	1533.663	1890.336	0.07131	1564.994	1633.082
YDR152W	3083.878	2822.151	2746.798	2922.687	2796.543	2665.256	-0.04545	2974.838	2983.448

YOR082C	0	1.977681	0	0	0	0	#NUM!	0	0.878001
YOR087W	772.6967	800.9608	774.6366	779.025	808.889	723.0633	-0.02311	719.5376	739.2769
YBR043C	529.7176	629.8914	575.0491	525.618	562.3046	522.4841	-0.10723	477.8179	548.7507
YCL041C	6.90936	1.977681	1.976114	1.790862	4.609054	0	-0.76332	3.372832	2.634003
YPR066W	191.1589	217.5449	223.3008	145.0598	134.8148	147.7436	-0.56361	167.5173	147.5042
YDR058C	398.4397	337.1946	345.8199	345.6364	339.9177	331.6894	-0.08831	459.8295	341.5424
YBR024W	833.7294	699.1102	805.2663	727.9854	710.9466	560.6431	-0.22565	782.4971	790.201
YLR334C	6.90936	1.977681	0	2.686293	1.152263	0	-1.21114	6.745665	0
YPR159W	2348.031	2759.854	2957.254	2866.275	2960.165	3173.064	0.158146	2607.199	2676.147
YPR130C	3.45468	1.977681	5.928341	8.058879	2.304527	1.956869	0.116983	3.372832	2.634003
YPR002W	96.73103	144.3707	145.2444	73.42534	73.74486	91.97286	-0.69202	114.6763	91.31211
YPL179W	2295.059	2257.523	2412.835	2137.394	2318.354	2638.838	0.026509	2102.399	2281.047
YPR163C	7771.878	7674.391	7951.881	8879.989	8495.639	7921.407	0.112574	8276.931	8025.808
YMR179W	581.5378	578.4717	696.5801	733.358	799.6708	889.3972	0.383798	608.2341	743.6669
YBR131W	257.9494	222.4891	225.277	248.9298	244.2799	234.8243	0.044919	231.6012	211.5983
YNR064C	116.3076	114.7055	128.4474	143.269	118.6831	134.0456	0.139659	156.2746	92.19011
YDR222W	452.5631	592.3154	517.7418	715.4494	622.2223	574.3412	0.291125	558.7659	577.7247
YHL015W	58053.59	48951.56	55769.88	57706.95	58597.21	54525.23	0.069677	67927.72	60466.18
YER029C	829.1232	935.4431	753.8874	952.7386	928.7244	823.842	0.103253	980.37	848.149
YPL034W	127.8232	111.739	83.98483	120.8832	109.465	91.97286	-0.00548	121.422	93.94611
YGR030C	375.4085	357.9602	356.6885	306.2374	324.9383	253.4146	-0.30132	353.0231	278.3263
YHR009C	3435.103	3760.56	3481.912	3522.626	3332.346	3620.208	-0.02761	3811.301	3450.544
YMR038C	4448.476	4625.796	4661.652	5387.808	4853.334	4671.047	0.118538	4628.65	4329.423
YER010C	799.1826	790.0835	899.1317	764.6981	823.8684	806.2302	-0.05531	820.7226	820.931
YPR126C	28.789	22.74333	20.74919	31.34009	16.13169	24.46087	-0.00698	19.11272	21.95003
YDR212W	2936.478	3534.116	2839.675	3117.891	3123.786	2697.545	-0.05867	3323.364	3156.414
YBL032W	2475.854	2608.561	2597.601	2566.305	2688.231	2486.203	0.010986	2620.691	2553.227
YNL253W	511.2926	459.8108	418.9361	439.6566	419.4239	474.5408	-0.05978	482.315	359.9804
YNL248C	4008.58	3810.991	4082.651	4717.131	4771.523	4868.691	0.270558	4309.356	4483.951
YGL154C	290.1931	299.6187	303.3334	250.7207	243.1276	250.4793	-0.26296	273.1994	265.1563
YLR171W	2.30312	2.966521	1.976114	5.372586	0	0.978435	-0.19015	6.745665	1.756002
YBR187W	3531.834	3919.763	3525.387	4044.662	4095.144	3565.416	0.092671	4157.578	3459.324
YBR295W	396.1366	354.0049	327.0468	308.9237	324.9383	347.3443	-0.13464	355.2717	316.0804
YPL254W	956.9463	1053.115	954.4629	973.3335	1005.926	1009.745	0.011864	970.2515	973.7032
YLR091W	596.508	655.6012	585.9177	662.619	520.8231	560.6431	-0.07569	590.2457	561.9207
YLR154W-I	0	0	0.988057	0	0	1.956869	0.985882	2.248555	0.878001
YDR372C	2208.692	1954.938	2052.194	2062.178	2048.724	1981.33	-0.02897	1923.639	1910.53
YDR320W-I	6.90936	1.977681	0	0	2.304527	1.956869	-1.06038	2.248555	0.878001
YNL222W	491.7161	532.985	452.53	547.1084	462.0577	488.2389	0.019569	519.4162	491.6806
YCR018C	77.15452	71.19651	57.3073	39.39897	39.17696	30.33148	-0.91715	44.9711	43.02205
YLL004W	437.5928	407.4023	442.6495	427.1206	528.8889	522.4841	0.199394	464.3266	495.1926
YDR190C	3146.062	3568.725	3291.217	3498.449	3604.28	3456.81	0.077681	3294.133	3478.64
YLR282C	1.15156	0.98884	1.976114	1.790862	0	2.935304	0.199247	2.248555	0.878001
YDL007C-/	4.60624	0	0	0	0	0	#NUM!	0	0
YEL046C	5344.39	7344.118	6296.886	6551.869	6864.033	6944.93	0.100907	6283.587	6133.716
YPL082C	1183.804	1108.49	1240.999	1167.642	1314.733	1598.762	0.207958	1142.266	1350.366
YLR306W	272.9197	382.6812	288.5126	274.0019	240.8231	254.393	-0.29557	346.2775	240.5723
YIL115C	1159.621	1191.553	1582.867	1381.65	1405.761	1978.395	0.276709	1174.87	1416.216
YHR145C	20.72808	19.77681	34.58199	17.90862	14.97943	20.54713	-0.49077	21.36127	23.70603
YOR011W-/	0	1.977681	0	0	2.304527	0.978435	0.731188	3.372832	0
YDR506C	209.5839	185.902	254.9187	212.2172	221.2346	194.7085	-0.0502	222.6069	188.7702
YDR065W	302.8603	403.4469	392.2586	334.8912	270.7819	353.2149	-0.19619	312.5491	366.1264
YLR201C	921.248	906.7667	928.7734	818.424	869.9589	627.1767	-0.25163	878.0607	878.0011
YML084W	2.30312	0	0	0.895431	0	0	-1.36294	1.124277	0

YPR155C	324.7399	284.786	290.4887	198.7857	260.4115	250.4793	-0.34279	249.5896	273.9363
YBR155W	1742.31	1782.879	1905.962	2055.014	1939.259	2166.254	0.181796	2023.699	1949.162
YHR039C-/	7700.481	5891.511	6720.763	7128.526	7044.939	6515.397	0.026468	8347.76	7286.531
YDR361C	2132.689	2293.121	2081.836	2221.564	2188.148	2143.75	0.010122	2446.428	2079.985
YFL009W	810.6982	855.347	838.8602	771.8615	1023.21	913.858	0.112967	733.0289	910.4871
YBR204C	408.8038	397.5139	468.3389	385.0353	415.9671	416.8132	-0.06581	365.3902	400.3685
YLL030C	0	1.977681	1.976114	0	0	0	#NUM!	0	0
YGL219C	642.5704	604.1815	588.8819	530.0952	515.0618	529.3332	-0.22139	579.0029	514.5086
YOR284W	270.6166	250.1766	283.5723	281.1653	260.4115	226.0184	-0.06751	274.3237	240.5723
YIR010W	657.5407	790.0835	744.9948	850.6595	789.3005	856.1304	0.187015	679.0636	765.6169
YPR007C	26.48588	25.70985	30.62976	11.6406	11.52263	21.52556	-0.89016	16.86416	17.56002
YLR347C	1809.101	2041.955	2200.403	2162.466	2182.387	2643.731	0.207717	1910.147	2314.411
YBR084C-/	33322.69	26068.8	29787.94	32654.58	30617.94	30190.58	0.067686	37041.57	31960.12
YBL113C	77.15452	73.17419	76.08038	128.0466	120.9877	127.1965	0.732686	91.06648	99.21412
YML072C	9297.695	6752.791	9427.05	8708.067	9568.396	12490.7	0.272166	8805.341	9984.628
YDR477W	1784.918	1689.928	1838.774	1886.673	1992.264	2539.038	0.272423	1831.448	2154.615
YBL061C	1006.463	1121.345	1277.557	1039.595	1066.996	1435.364	0.056736	970.2515	1119.451
YDR204W	849.8512	836.559	786.4932	782.6067	652.1811	669.2493	-0.23305	930.9018	777.909
YMR094W	82.91232	62.29695	103.746	68.94819	110.6173	164.377	0.466281	75.32659	82.5321
YMR161W	1111.255	1395.254	1221.238	1147.047	1128.066	1114.437	-0.13721	1173.746	1176.521
YDR536W	184.2496	241.2771	182.7905	92.2294	125.5967	48.92174	-1.18935	213.6127	156.2842
YLR027C	6310.548	6123.889	6443.119	6198.174	6148.478	5901.918	-0.04889	6437.613	5664.863
YIR001C	1006.463	992.7958	934.7018	930.3528	1070.453	1046.925	0.054886	792.6156	1046.577
YBL023C	698.9969	738.6638	789.4574	753.0575	890.6997	1020.507	0.258559	698.1763	813.907
YGL206C	4179.011	3452.042	4298.047	3742.902	3724.116	4733.667	0.032477	4129.471	4191.577
YOL071W	900.5199	778.2174	802.3021	747.6849	704.033	585.104	-0.28462	878.0607	798.103
YNL205C	2.30312	0	0	0	0	1.956869	-0.23504	1.124277	0
YDL034W	3.45468	9.888404	5.928341	0.895431	3.45679	11.74122	-0.25999	10.1185	2.634003
YPR034W	2976.782	3217.687	3086.69	3270.114	3025.844	3007.708	0.003494	2808.445	3049.298
YNL186W	2658.952	2785.564	2804.105	3251.31	3316.214	3909.825	0.345049	2781.462	3396.986
YPR135W	582.6893	608.1369	716.3412	658.1418	565.7614	737.7398	0.040631	517.1676	641.8188
YMR315W	2301.968	2232.802	2312.053	2093.518	2130.535	2111.462	-0.11197	2403.705	2182.711
YNL210W	101.3373	68.22999	35.57005	48.35328	86.41976	36.20208	-0.2628	69.7052	51.80206
YPR134W	313.2243	255.1208	293.4529	211.3217	268.4774	210.3635	-0.32041	254.0867	219.5003
YDR438W	177.3402	189.8574	235.1575	148.6416	160.1646	207.4282	-0.22259	187.7543	190.5262
YJR095W	49.51708	53.39738	56.31924	41.18983	23.04527	17.61182	-0.96015	34.8526	36.87605
YJR099W	485.9583	508.264	527.6223	449.5064	473.5803	391.3739	-0.21135	483.4393	431.0985
YJL011C	1287.444	1119.367	1169.859	1253.603	1159.177	1079.213	-0.03457	1297.416	1132.621
YOR016C	945.4307	822.7152	855.6572	867.6727	879.177	707.4083	-0.09637	943.2688	773.5189
YHR187W	779.6061	832.6036	753.8874	767.3844	743.2099	638.9179	-0.1385	719.5376	770.0069
YOR100C	43.75928	66.25231	37.54616	21.49034	25.3498	29.35304	-0.95355	52.84104	30.73004
YOL026C	1265.564	1070.914	1205.429	1164.06	1063.539	980.3916	-0.14286	1280.552	1030.773
YER023W	2111.961	1865.942	1891.141	1926.968	1711.111	1652.576	-0.14968	2058.552	1669.08
YOR218C	6.90936	0.98884	1.976114	0	3.45679	3.913739	-0.42191	2.248555	1.756002
YNL107W	755.4233	831.6148	760.8038	712.7631	669.4651	690.7749	-0.17961	609.3584	769.1289
YCR032W	629.9033	571.5498	543.4313	579.3439	566.9136	634.0257	0.028975	595.8671	633.0388
YDL058W	1158.469	1053.115	1305.223	1218.682	1342.387	1728.894	0.286699	1242.327	1360.024
YGL079W	540.0816	485.5207	455.4942	436.0749	421.7284	419.7485	-0.21328	466.5752	507.4846
YIL070C	1435.995	1411.075	1150.098	1303.748	1235.226	1105.631	-0.13322	1666.179	1239.738
YBR103C-/	0	0	0	3.581724	1.152263	2.935304	#DIV/0!	1.124277	3.512004
YLR167W	96676.91	67592.19	82188.54	87632.25	85344.7	82674.8	0.05284	112036.5	91327.04
YBR161W	589.5987	643.7351	679.7831	591.8799	682.14	564.5568	-0.05734	587.9971	618.9908
YNL312W	1308.172	1396.243	1237.047	1216.891	1290.535	1133.027	-0.11461	1378.364	1151.059
YGR056W	739.3015	691.1995	855.6572	726.1946	775.4733	926.5777	0.086989	731.9046	848.149

YGL140C	1050.223	1158.921	1223.214	1070.936	1183.375	1306.21	0.052888	1146.763	1237.982
YGL002W	568.8706	491.4537	491.0642	554.2718	558.8478	450.08	0.010942	628.4711	507.4846
YFL039C	35912.55	32503.19	34469.35	35509.21	35273.09	32733.53	0.008818	39396.93	35458.07
YGR032W	669.0563	892.9229	677.807	592.7753	686.749	621.306	-0.23673	548.6474	651.4768
YFL047W	852.1544	1038.282	1009.794	879.3133	916.0495	1054.753	-0.02515	978.1214	923.6571
YBL019W	259.101	328.295	286.5365	289.2242	337.6132	280.8108	0.054611	323.7919	278.3263
YJR075W	2648.588	2425.626	2541.282	2835.83	2657.12	2849.202	0.131482	2646.549	2699.853
YOL072W	508.9895	614.0699	541.4551	600.8342	539.2593	563.5784	0.033546	523.9133	582.9927
YDR120C	2817.867	3121.769	2793.237	3077.596	3395.72	3330.592	0.166901	3275.02	3380.304
YHR002W	263.7072	246.2213	184.7666	143.269	192.428	201.5576	-0.37077	214.737	193.1602
YGR105W	1058.284	967.0859	1054.257	1016.314	1229.465	899.1815	0.030287	1231.084	977.2152
YAL037C-I	0	0.98884	0	2.686293	1.152263	0	1.956754	2.248555	0.878001
YBR085C-/	2189.115	2247.634	2310.077	1886.673	1892.017	1617.353	-0.32231	2312.639	2177.443
YPL079W	19895.5	15356.69	19029.97	20857.27	18778.44	17167.62	0.065497	20991.38	19812.97
YKL066W	96.73103	59.33043	69.16398	77.00707	85.2675	73.3826	0.06532	102.3093	73.75209
YER011W	16295.72	16760.85	14691.42	17537.91	16594.9	12177.6	-0.0441	14336.79	12211.24
YHL017W	590.7502	763.3848	659.0339	673.3641	753.5803	690.7749	0.073043	682.4364	666.4028
YEL051W	6584.62	5633.424	6245.507	6569.777	6458.437	6538.879	0.083749	7008.746	5947.579
YKL018W	819.9107	691.1995	738.0784	805.8879	722.4692	686.8612	-0.02196	829.7168	623.3808
YNL042W	466.3818	436.0786	439.6853	456.6698	496.6256	467.6918	0.082352	469.948	492.5586
YNL238W	1930.014	1877.808	2045.278	2064.864	2106.338	2535.124	0.196322	1847.188	2170.419
YDR405W	2918.053	2440.458	3004.681	2820.608	2510.782	2469.569	-0.1004	2870.28	3085.296
YML114C	1146.954	1120.356	1224.202	1315.388	1153.416	1231.849	0.083928	1050.075	1263.444
YFL032W	0	0	3.952227	0.895431	0	0	-2.14201	0	0
YJR086W	631.0548	574.5163	585.9177	508.6048	578.4363	566.5137	-0.11559	662.1994	614.6008
YIL002W-/	913.187	806.8938	834.908	804.9925	1040.494	884.505	0.095579	1261.439	882.3911
YGL134W	401.8944	443.9894	466.3628	405.6303	376.7902	373.7621	-0.18267	366.5145	349.4444
YMR119W-/	17.2734	15.82145	10.86863	8.95431	25.3498	6.849043	-0.0953	15.73988	10.53601
YOR257W	679.4204	614.0699	539.479	617.8474	562.3046	445.1878	-0.17344	703.7977	551.3847
YMR254C	13.81872	33.62057	27.66559	13.43147	16.13169	20.54713	-0.5838	20.23699	40.38805
YFL008W	928.1573	797.9942	994.9732	945.5752	995.5556	1311.103	0.257228	762.2601	978.0932
YKR069W	1359.992	1645.43	1351.662	1412.99	1238.683	1227.936	-0.16745	1030.962	988.6292
YDR242W	325.8915	305.5517	313.214	326.8323	343.3745	397.2445	0.176308	415.9827	359.9804
YPL178W	1761.887	1635.542	1569.034	1632.371	1570.535	1433.407	-0.09924	1861.804	1532.112
YGR125W	1378.417	1330.979	1261.749	1395.082	1244.445	1362.96	0.011341	1278.303	1363.536
YCL042W	3.45468	3.955362	2.96417	4.477155	1.152263	3.913739	-0.12046	4.49711	5.268006
YCL023C	0	1.977681	0	0	0	0	#NUM!	0	0
YGL022W	4745.579	5221.077	4963.009	4987.551	4645.926	4296.307	-0.10001	4719.717	4939.634
YDR154C	3.45468	16.81029	10.86863	7.163448	11.52263	5.870608	-0.34236	8.99422	10.53601
YOL008W	515.8989	493.4314	436.7211	486.219	486.2552	439.3172	-0.03459	468.8237	463.5846
YJR020W	1.15156	0	0.988057	0	0	0	#NUM!	0	0
YOR140W	519.3535	728.7754	718.3173	676.9459	677.5309	860.0441	0.171404	567.7601	751.5689
YCL016C	518.202	512.2193	571.0968	505.0231	517.3663	542.0528	-0.03379	499.1792	498.7046
YOL018C	787.667	716.9093	802.3021	685.9002	691.3581	669.2493	-0.17278	632.9682	641.8188
YPL126W	3524.925	3109.903	3744.735	3848.563	4220.741	5126.998	0.346388	3678.636	4044.073
YMR309C	5162.443	5141.97	5913.52	5698.523	6359.342	7600.481	0.277552	5499.965	6274.196
YKL035W	10004.75	9323.776	9668.136	8836.113	8938.108	8682.63	-0.13225	10397.32	9861.708
YDR255C	401.8944	388.6143	385.3422	322.3552	338.7655	430.5113	-0.10722	337.2832	413.5385
YBR202W	1072.102	1270.66	1239.023	1285.839	1221.399	1354.154	0.108442	1024.217	1285.394
YHR083W	1832.132	1675.096	1528.524	1448.807	1477.202	1406.989	-0.21684	1532.39	1485.578
YOL068C	1502.786	1540.613	1615.473	1572.377	1721.482	1610.504	0.074085	1463.809	1631.326
YHR133C	1926.56	1759.147	1814.072	1870.555	2204.28	1673.123	0.063676	1838.194	1731.418
YLR369W	831.4263	870.1796	865.5378	881.9996	831.9342	827.7558	-0.01438	791.4913	894.6831
YPL072W	161.2184	137.4488	146.2324	191.6222	199.3416	193.7301	0.394202	175.3873	206.3303

YMR229C	4803.157	4430.994	4748.601	5601.816	6066.667	7537.861	0.457935	5513.457	5980.065
YLR418C	1432.541	1500.071	1462.324	1416.572	1361.975	1285.663	-0.11287	1304.162	1283.638
YNL003C	977.6744	1138.155	1000.902	929.4574	939.0947	787.6399	-0.23067	903.9191	893.8051
YGL210W	1274.777	1093.658	1259.772	1269.721	1194.897	1107.588	-0.02244	1180.491	1151.937
YBR291C	544.6879	593.3043	472.2912	552.4809	558.8478	423.6622	-0.06908	576.7543	467.9746
YLR254C	611.4783	581.4382	502.9209	576.6576	572.6749	386.4817	-0.14299	540.7775	485.5346
YKL203C	879.7918	914.6774	846.7647	753.0575	925.2676	912.8796	-0.02759	864.5694	835.857
YOR103C	8.06092	17.79913	17.78502	21.49034	10.37037	13.69809	0.061911	11.24277	13.17002
YDL009C	78.30608	81.08492	78.05649	70.73905	56.46091	65.55513	-0.30084	66.33237	66.72808
YHL030W-A	3.45468	0	1.976114	0	1.152263	0	-2.23669	0	4.390005
YLR162W-A	2.30312	0	0	0	0	1.956869	-0.23504	2.248555	0
YGR233C	899.3683	942.3649	878.3825	621.4291	758.1894	834.6048	-0.29687	753.2659	827.955
YCR025C	72.54828	33.62057	57.3073	48.35328	63.37449	41.09426	-0.09723	60.71098	40.38805
YLR154W-E	0	0	0	0	2.304527	0	#DIV/0!	0	1.756002
YIL064W	761.1811	793.05	784.5171	779.025	868.8067	782.7478	0.055564	932.026	741.0329
YLR248W	3207.094	3168.245	3169.686	3255.787	3353.087	3783.607	0.122719	3103.006	3325.868
YDL183C	165.8246	173.0471	178.8383	171.9228	110.6173	128.1749	-0.33401	147.2803	133.4562
YEL055C	2384.881	2045.911	2334.778	2486.612	2764.28	2968.571	0.280833	2500.393	2633.125
YOR263C	0	1.977681	2.96417	3.581724	0	1.956869	0.164468	2.248555	1.756002
YDR447C	18422.66	14596.27	16521.3	17822.66	16549.96	15923.05	0.021834	19925.57	17513.49
YLR197W	6041.083	6323.635	7215.779	6885.865	6978.108	7232.589	0.10759	6623.119	6866.846
YGL034C	9.21248	2.966521	6.916398	3.581724	5.761317	4.892174	-0.42376	4.49711	5.268006
YOR115C	1193.016	1114.423	1151.086	1130.929	1053.169	1166.294	-0.04583	1370.494	1046.577
YDL088C	1558.061	1343.834	1467.264	1567.9	1523.292	1836.522	0.173563	1430.081	1467.14
YIR022W	1693.945	1366.577	1361.542	1485.52	1473.745	1318.93	-0.04772	1731.387	1419.728
YBL064C	3389.041	2850.827	3192.412	3235.192	3065.021	2927.477	-0.03164	3310.997	3461.958
YJR040W	674.8141	834.5813	675.8309	715.4494	744.3622	657.5081	-0.04554	756.6387	660.2568
YLR219W	1425.631	1506.004	1408.969	1172.119	1208.724	1333.607	-0.22475	1140.017	1405.68
YDL145C	4084.583	3931.63	4629.046	4015.113	4108.972	5031.111	0.057036	3774.199	4713.988
YLR311C	3.45468	6.921883	2.96417	1.790862	0	0	-2.89711	14.61561	0
YGR076C	649.4798	621.9806	531.5746	493.3825	521.9754	403.1151	-0.34609	579.0029	589.1387
YBL070C	0	1.977681	1.976114	0.895431	2.304527	0.978435	0.07971	5.621387	0.878001
YHR175W	834.881	719.8758	761.7918	806.7834	784.6914	758.2869	0.020537	896.0492	816.541
YDR387C	335.1039	405.4246	334.9513	316.9826	384.856	260.2636	-0.16072	339.5318	318.7144
YLR434C	1.15156	0.98884	1.976114	2.686293	0	2.935304	0.449557	6.745665	0
YGR221C	396.1366	325.3285	433.7569	419.9572	458.6009	514.6567	0.270247	359.7688	434.6105
YLL034C	1704.309	1977.681	1904.974	2290.513	2366.749	2665.256	0.390276	2181.098	2179.199
YGR148C	34185.21	25172.91	30502.3	31742.13	31486.75	29848.13	0.050739	37909.51	31889
YLL050C	8391.417	6208.929	7280.003	7509.98	7447.079	7332.39	0.026725	8828.951	7268.093
YOR327C	3507.652	3233.508	3074.833	3356.075	3453.334	3078.156	0.010481	3395.318	3534.832
YGR119C	2943.387	3124.736	3226.994	3412.488	3354.239	3404.953	0.130013	2805.072	3154.658
YOR013W	251.0401	216.5561	195.6353	256.0933	214.321	232.8675	0.084591	269.8266	207.2083
YHR165C	1566.122	1417.997	1381.303	1522.233	1637.366	1719.11	0.160378	1586.356	1432.898
YDL107W	322.4368	313.4624	270.7276	313.4009	268.4774	298.4226	-0.04251	308.052	312.5684
YIL083C	1343.87	1176.72	1210.37	1390.604	1283.622	1326.757	0.100808	1255.818	1210.763
YNL302C	29447.69	22579.18	26690.38	28425.46	27773.01	26378.6	0.069061	32036.29	27647.38
YOR111W	340.8617	312.4736	421.9003	346.5318	374.4856	283.7461	-0.0978	327.1647	397.7345
YLR110C	253172.8	204846.2	223276.1	238910	242945.5	213069.8	0.028578	303787.6	234362.2
YOL044W	211.887	245.2324	242.0739	283.8516	183.2099	164.377	-0.14705	220.3584	187.8922
YDR322W	1349.628	1211.33	1250.88	1232.113	1169.547	1223.043	-0.07262	1360.376	1415.338
YHL027W	1613.335	1658.285	1751.825	1554.468	1560.165	1665.296	-0.07169	1608.841	1612.01
YML014W	1038.707	1110.468	1017.699	1287.63	1200.659	966.6935	0.125619	1138.893	1110.671
YPL140C	521.6567	546.8288	503.909	570.3896	501.2346	635.9826	0.119013	539.6532	529.4346
YKL023C-A	418.0163	481.5653	476.2434	557.8535	475.8848	499.0017	0.155816	465.4509	468.8526

YPR074W-/	0	0	0.988057	0	0	0	#NUM!	0	0
YDL062W	0	0	0.988057	0	0	0	#NUM!	0	0
YCL007C	0	0	0	0.895431	0	0	#DIV/0!	1.124277	0
YHR054W-/	0	0	0	0	0	0.978435	#DIV/0!	1.124277	0
YNL132W	2838.595	2592.74	2725.061	3194.898	3301.235	3319.829	0.267198	2938.861	3121.294
YBL024W	2808.655	2836.983	3076.809	3284.441	3265.515	3912.76	0.262453	3176.084	3353.964
YIL035C	1568.425	1525.781	1614.485	1661.92	1608.56	1803.255	0.107722	1685.292	1581.28
YLR315W	46.0624	86.02912	96.82957	85.96138	59.9177	56.74921	-0.17601	58.46243	64.97208
YOR098C	2487.369	2723.267	2789.284	3192.212	3369.218	4055.612	0.408324	2462.168	3244.214
YBL087C	17713.29	14083.07	15793.1	16546.67	17434.9	16299.74	0.07938	20189.77	16571.39
YDL188C	1136.59	1280.548	1169.859	1191.819	1086.584	1138.898	-0.06992	1190.61	1103.647
YPR170W-I	43.75928	35.59826	28.65365	42.08526	36.87243	34.24522	0.06773	44.9711	35.12004
YGL229C	102.4888	76.14071	102.7579	63.5756	67.98354	67.512	-0.49927	55.0896	91.31211
YKL173W	915.4902	816.7822	822.0633	977.8107	937.9425	1107.588	0.243196	918.5347	942.9732
YPL086C	1346.174	1678.062	1223.214	1629.684	1576.296	1618.331	0.183726	1504.283	1488.212
YPR001W	103.6404	87.01796	78.05649	86.85681	132.5103	80.23165	0.156956	92.19075	98.33612
YNL234W	216.4933	197.7681	224.2889	222.0669	216.6255	221.1262	0.047269	211.3642	243.2063
YMR212C	1753.826	1647.408	1918.806	1951.144	2049.877	2540.995	0.298298	2145.121	1906.14
YKR031C	751.9686	723.8312	820.0872	616.0565	667.1605	963.7582	-0.03107	664.448	715.5709
YOR276W	746.2108	681.3111	843.8005	883.7904	808.889	750.4594	0.105203	903.9191	767.3729
YFL041W-/	34.5468	31.64289	24.70142	33.13095	25.3498	17.61182	-0.25638	21.36127	43.02205
YBR191W-/	3.45468	2.966521	0.988057	2.686293	9.218108	2.935304	1.002061	6.745665	4.390005
YPL205C	42.60772	86.02912	99.79374	92.2294	62.22223	90.99443	0.10365	57.33815	71.99609
YLR144C	633.358	650.657	610.6191	711.8677	670.6173	736.7613	0.161632	643.0867	585.6267
YDR228C	671.3594	711.9651	772.6604	885.5813	842.3046	933.4267	0.303791	609.3584	808.639
YGR034W	24077.97	19299.2	22154.21	23003.62	22964.61	21811.27	0.048663	27703.32	23245.96
YJL194W	307.4665	254.132	213.4203	261.4659	275.391	229.9322	-0.0154	252.9624	230.0363
YBL025W	362.7414	331.2615	242.0739	317.878	397.5309	296.4657	0.112332	300.1821	400.3685
YDR360W	6.90936	23.73217	12.84474	32.23552	16.13169	23.48243	0.724421	15.73988	15.80402
YDL095W	4912.555	4676.226	4675.485	4684	5054.98	4714.098	0.018971	4683.74	4936.122
YBR200W	1144.651	1108.49	1130.337	1130.929	1226.008	1267.073	0.09908	1018.595	1189.691
YAL048C	214.1901	228.4221	264.7992	241.7664	213.1687	279.8323	0.054738	156.2746	234.4263
YLR181C	996.0993	949.2868	955.451	985.8696	889.5474	852.2166	-0.08882	920.7833	1015.847
YBR008C	427.2287	536.9404	454.5061	451.2972	353.7449	396.2661	-0.23994	405.8642	460.0726
YJR149W	280.9806	379.7147	369.5333	255.1978	339.9177	289.6167	-0.21965	313.6734	308.1784
YNL146W	84.06388	67.24115	65.21175	86.85681	72.5926	72.40417	0.098735	62.95954	69.36208
YHR130C	28.789	22.74333	25.68948	26.86293	13.82716	18.59026	-0.38145	21.36127	28.09603
YIL048W	1004.16	1028.394	1278.546	1061.086	1166.091	1594.849	0.207027	1111.91	1211.641
YBR078W	22692.64	24981.08	23749.92	25892.28	27215.31	23922.73	0.109025	25208.55	24548.03
YIL025C	0	0	0	1.790862	2.304527	1.956869	#DIV/0!	2.248555	0
YDR243C	385.7726	394.5473	411.0316	364.4404	427.4897	495.088	0.111433	368.763	414.4165
YPR201W	70.24516	65.26347	115.6026	68.05276	62.22223	54.79234	-0.44028	69.7052	75.50809
YPR056W	776.1514	630.8802	769.6963	734.2534	691.3581	804.2733	0.034808	719.5376	660.2568
YBR076W	35.69836	8.899564	14.82085	14.3269	12.6749	27.39617	-0.12737	25.85838	23.70603
YPR176C	825.6685	841.5032	710.4129	768.2798	732.8396	644.7885	-0.14791	707.1705	791.957
YKL068W	2497.734	2564.063	2962.194	2968.354	3022.387	3683.807	0.269874	2556.607	2999.252
YDR008C	5.7578	0.98884	2.96417	2.686293	2.304527	3.913739	-0.12505	5.621387	1.756002
YGR128C	2220.208	2103.264	2194.474	2764.196	2708.971	3261.123	0.422273	2639.804	2536.545
YHR199C-/	104.792	151.2926	146.2324	96.70655	103.7037	99.80034	-0.42236	91.06648	105.3601
YNL308C	4427.748	4278.713	4574.703	5355.573	5116.05	5748.304	0.288386	4802.913	5110.844
YMR142C	44637.92	33928.1	39555.87	42746.98	40666.83	38191.24	0.041927	48224.76	40925.39
YMR310C	1537.333	1710.694	1618.437	1833.843	2080.988	1877.616	0.251299	1886.538	1755.124
YLL025W	57.578	96.90636	49.40284	57.30759	58.76544	52.83547	-0.27153	49.46821	49.16806
YPR165W	7930.793	7019.778	7104.129	7362.234	7217.778	6882.31	-0.03928	8605.22	7636.853

YMR106C	336.2555	274.8976	320.1304	286.5379	282.3045	296.4657	-0.10601	233.8497	278.3263
YJR047C	6339.337	5622.547	5460.002	5952.825	5924.939	5008.607	-0.04504	5647.246	4645.504
YBR086C	6524.739	4957.057	7029.036	6264.435	6640.494	7950.76	0.172071	6869.335	7318.139
YCR011C	1447.511	1373.499	1421.814	1123.766	1193.745	1411.881	-0.18608	1287.298	1259.932
YJR049C	1303.566	1290.437	1217.286	1230.322	1143.045	1169.229	-0.10547	1117.532	1205.495
YOR373W	754.2718	709.9874	812.1827	712.7631	740.9054	902.1168	0.049427	622.8497	721.7169
YCR016W	2061.292	1873.853	1892.129	2249.323	2085.597	2023.403	0.125825	2123.76	2095.789
YCR083W	373.1054	355.9826	284.5604	360.8587	311.1111	299.401	-0.06146	395.7457	386.3205
YHR086W	1104.346	1168.809	1064.137	1081.681	1022.058	1199.561	-0.01477	1028.714	1017.603
YNL026W	656.3892	698.1213	755.8635	622.3246	695.9671	608.5864	-0.13123	589.1214	676.9388
YHR013C	2075.111	1967.792	1860.511	2105.158	1995.72	1845.328	0.01042	2285.656	1985.16
YDR192C	489.413	439.0452	417.948	445.9247	496.6256	424.6407	0.022101	413.7341	395.1005
YMR055C	368.4992	315.4401	315.1901	300.8648	276.5432	237.7596	-0.29357	323.7919	269.5463
YGL087C	743.9077	708.0098	706.4606	749.4758	707.4898	710.3436	0.005957	801.6098	774.3969
YAL019W-/	0	0	0	0	0	1.956869	#DIV/0!	0	0.878001
YPL204W	2831.686	3279.984	3264.54	3082.969	3150.288	2902.037	-0.03755	2699.39	3132.708
YDR419W	602.2658	606.1592	564.1804	536.3632	495.4733	470.6271	-0.23854	552.0202	532.0687
YJR070C	6705.534	6873.43	6758.309	8683.89	7707.49	6826.539	0.191113	7205.494	7290.043
YJR120W	214.1901	315.4401	310.2498	348.3227	321.4815	372.7836	0.311914	259.7081	374.9065
YJL168C	1003.009	1014.55	999.9135	1041.386	1096.955	1241.634	0.163672	756.6387	1081.697
YBL042C	3172.548	4220.371	3631.109	3398.161	3548.971	3603.575	-0.06331	3376.205	3518.15
YER106W	3.45468	13.84377	11.85668	16.11776	4.609054	0	-0.49225	3.372832	8.780011
YML094W	479.0489	379.7147	375.4616	438.7612	447.0782	410.9426	0.07133	466.5752	433.7325
YLR437C-/	0	0.98884	0	0	0	0.978435	-0.01526	0	0
YOL021C	2335.364	2451.335	2462.238	2720.319	2545.35	3090.875	0.205137	2425.067	2684.049
YNL001W	939.6729	1076.847	954.4629	1074.517	986.3375	1036.162	0.059939	1009.601	1098.379
YDL029W	3103.454	3215.709	3125.224	3119.682	3082.305	2967.592	-0.0426	2964.72	3036.128
YGL157W	1559.212	1491.171	1621.401	1763.999	1691.523	1548.862	0.099219	1605.468	1679.616
YJR122W	466.3818	459.8108	475.2553	440.5521	375.6379	370.8268	-0.23958	382.2543	482.0226
YGR185C	8242.866	7443.991	8265.095	8487.791	8306.667	7551.559	0.023543	8516.402	8169.8
YOL097W-/	69.0936	69.21883	50.3909	59.99388	48.39507	62.61982	-0.14205	74.20231	67.60608
YHR077C	2181.055	2079.531	2195.462	2252.009	2411.687	2882.469	0.225092	2187.844	2355.677
YDR218C	29.94056	63.28579	18.77308	57.30759	25.3498	37.18052	0.097593	58.46243	36.87605
YGL258W	46.0624	43.50898	39.52227	33.13095	54.15638	33.26678	-0.09874	39.34971	35.12004
YML082W	878.6402	1194.519	1134.289	1137.197	1198.354	1146.725	0.118605	1092.798	1174.765
YNL149C	19.57652	9.888404	12.84474	21.49034	13.82716	21.52556	0.425997	16.86416	16.68202
YMR068W	333.9524	478.5988	427.8286	478.1602	354.8971	312.1207	-0.11521	444.0896	319.5924
YMR313C	629.9033	773.2732	708.4367	739.626	655.6379	628.1551	-0.06155	663.3237	671.6708
YHR093W	11.5156	9.888404	12.84474	12.53603	4.609054	3.913739	-0.70163	12.36705	6.146008
YDR009W	148.5512	156.2368	124.4952	124.4649	149.7943	80.23165	-0.27618	132.6647	125.5542
YHR154W	749.6655	679.3334	818.1111	737.8352	848.0659	1004.852	0.205301	672.3179	738.3989
YKL048C	549.2941	509.2528	496.9926	583.821	589.9589	610.5433	0.197962	418.2312	554.0187
YIL071C	122.0654	165.1364	149.1966	164.7593	95.63787	140.8946	-0.12099	97.81214	160.6742
YDR373W	905.1261	868.2019	889.2511	953.634	921.8108	786.6615	-0.00026	962.3815	880.6351
YDR125C	90.97324	139.4265	138.328	75.21621	84.11523	131.1103	-0.34431	125.9191	100.0921
YML077W	581.5378	568.5832	585.9177	680.5276	733.9918	544.0097	0.173971	625.0983	586.5047
YGL108C	221.0995	226.4445	225.277	216.6943	246.5844	217.2125	0.016354	232.7254	195.7942
YER172C	988.0384	1003.673	1058.209	1041.386	1011.687	1489.178	0.215895	1053.448	1052.723
YDL102W	633.358	564.6279	741.0426	711.8677	667.1605	883.5265	0.222619	635.2168	693.6208
YKL159C	503.2317	439.0452	396.2108	447.7155	445.926	366.913	-0.08655	455.3324	461.8286
YDL073W	961.5525	851.3916	1102.671	936.6209	1004.774	1320.887	0.162081	988.2399	1012.335
YJR138W	522.8082	500.3533	531.5746	442.3429	468.9712	706.4299	0.057314	471.0723	529.4346
YGR172C	837.1841	1010.595	910.0003	929.4574	851.5227	725.0201	-0.13812	991.6127	883.2691
YBR220C	720.8765	754.4853	705.4726	744.1032	759.3416	741.6535	0.041898	667.8208	759.4709

YLL022C	1011.07	1103.546	1025.603	1061.981	955.2264	938.3189	-0.08745	1104.04	959.6552
YJL014W	3919.91	3995.904	4000.642	4051.825	4137.778	4339.358	0.072312	4013.671	4042.317
YLL023C	3032.057	3266.14	3252.683	2842.994	3005.103	2466.634	-0.19996	3318.867	2954.474
YJL054W	1643.276	1809.578	1655.983	1630.58	1570.535	1539.078	-0.10805	1717.896	1655.91
YAL012W	21118.46	22390.31	20020.01	23408.36	21952.92	20324.05	0.048161	21971.75	19966.62
YMR133W	50.66864	45.48666	51.37895	30.44465	34.5679	31.30991	-0.61511	48.34393	51.80206
YGL102C	1.15156	0.98884	0.988057	0	0	0	#NUM!	2.248555	0
YBR073W	1773.402	1661.252	1775.538	1713.855	1916.214	2465.655	0.226461	1748.251	1723.516
YHL045W	0	0	0	0	2.304527	0	#DIV/0!	0	0
YKR040C	0	0	0	0	0	1.956869	#DIV/0!	0	0
YBL091C-/	788.8186	746.5745	709.4248	727.9854	684.4445	665.3356	-0.11157	737.526	767.3729
YHR155W	705.9062	607.148	733.1382	650.0829	769.712	870.8069	0.162785	613.8555	769.1289
YDR194W-/	0	0	0	0	0	0.978435	#DIV/0!	0	0
YFL012W-/	0	0	0	0	0	0.978435	#DIV/0!	0	0
YOR331C	0	0	0	0.895431	0	0	#DIV/0!	0	0
YPR142C	0	0	0	0	0	0.978435	#DIV/0!	0	0
YAL034C-I	0	0	0	0.895431	0	0	#DIV/0!	0	0
YPL059W	2019.836	1545.558	1716.255	1747.881	1681.152	1426.558	-0.12134	1871.922	1657.666
YGR247W	423.7741	520.1301	402.1391	436.0749	417.1194	309.1854	-0.21164	530.659	352.0784
YPR058W	444.5021	502.3309	508.8493	477.2647	436.7079	444.2094	-0.10002	538.5289	429.3425
YMR007W	82.91232	65.26347	64.22369	35.81724	67.98354	87.08069	-0.1541	78.69942	67.60608
YDR220C	16.12184	13.84377	11.85668	8.95431	13.82716	25.4393	0.205383	11.24277	15.80402
YMR026C	559.6581	515.1859	535.5268	509.5003	505.8437	525.4194	-0.06375	580.1272	490.8026
YKL174C	530.8691	807.8826	574.061	588.2982	623.3745	629.1335	-0.05536	649.8324	640.0628
YPR023C	1751.523	1785.846	1605.592	1746.986	1670.782	1628.115	-0.02749	1860.679	1566.354
Q0010	0	0	0	0	0	0	#DIV/0!	0	0
Q0017	0	0	0	0	0	0	#DIV/0!	0	0
Q0032	0	0	0	0	0	0	#DIV/0!	0	0
Q0050	0	0	0	0	0	0	#DIV/0!	0	0
Q0055	0	0	0	0	0	0	#DIV/0!	0	0
Q0060	0	0	0	0	0	0	#DIV/0!	0	0
Q0065	0	0	0	0	0	0	#DIV/0!	0	0
Q0070	0	0	0	0	0	0	#DIV/0!	0	0
Q0075	0	0	0	0	0	0	#DIV/0!	0	0
Q0080	0	0	0	0	0	0	#DIV/0!	0	0
Q0092	0	0	0	0	0	0	#DIV/0!	0	0
Q0110	0	0	0	0	0	0	#DIV/0!	0	0
Q0115	0	0	0	0	0	0	#DIV/0!	0	0
Q0120	0	0	0	0	0	0	#DIV/0!	0	0
Q0140	0	0	0	0	0	0	#DIV/0!	0	0
Q0142	0	0	0	0	0	0	#DIV/0!	0	0
Q0144	0	0	0	0	0	0	#DIV/0!	0	0
Q0160	0	0	0	0	0	0	#DIV/0!	0	0
Q0182	0	0	0	0	0	0	#DIV/0!	0	0
Q0297	0	0	0	0	0	0	#DIV/0!	0	0
YAL004W	0	0	0	0	0	0	#DIV/0!	0	0
YAL042C-/	0	0	0	0	0	0	#DIV/0!	0	0
YAL045C	0	0	0	0	0	0	#DIV/0!	0	0
YAL059C-/	0	0	0	0	0	0	#DIV/0!	0	0
YAL063C-/	0	0	0	0	0	0	#DIV/0!	0	0
YAL064W	0	0	0	0	0	0	#DIV/0!	0	0
YAL068W-/	0	0	0	0	0	0	#DIV/0!	0	0
YAR035C-/	0	0	0	0	0	0	#DIV/0!	0	0
YAR047C	0	0	0	0	0	0	#DIV/0!	0	0

YAR053W	0	0	0	0	0	0	#DIV/0!	0	0
YAR060C	0	0	0	0	0	0	#DIV/0!	0	0
YAR069C	0	0	0	0	0	0	#DIV/0!	0	0
YAR070C	0	0	0	0	0	0	#DIV/0!	0	0
YBL006W-/	0	0	0	0	0	0	#DIV/0!	0	0
YBL039C-/	0	0	0	0	0	0	#DIV/0!	0	0
YBL062W	0	0	0	0	0	0	#DIV/0!	0	0
YBL068W-/	0	0	0	0	0	0	#DIV/0!	0	0
YBL113W-/	0	0	0	0	0	0	#DIV/0!	0	0
YBR056C-I	0	0	0	0	0	0	#DIV/0!	0	0
YBR099C	0	0	0	0	0	0	#DIV/0!	0	0
YBR121C-/	0	0	0	0	0	0	#DIV/0!	0	0
YBR131C-/	0	0	0	0	0	0	#DIV/0!	0	0
YBR141W-/	0	0	0	0	0	0	#DIV/0!	0	0
YBR223W-/	0	0	0	0	0	0	#DIV/0!	0	0
YBR266C	0	0	0	0	0	0	#DIV/0!	0	0
YCL022C	0	0	0	0	0	0	#DIV/0!	0	0
YCL058W-/	0	0	0	0	0	0	#DIV/0!	0	0
YCR038W-/	0	0	0	0	0	0	#DIV/0!	0	0
YCR045W-/	0	0	0	0	0	0	#DIV/0!	0	0
YCR047W-/	0	0	0	0	0	0	#DIV/0!	0	0
YCR081C-/	0	0	0	0	0	0	#DIV/0!	0	0
YCR087C-/	0	0	0	0	0	0	#DIV/0!	0	0
YDL025W-/	0	0	0	0	0	0	#DIV/0!	0	0
YDL041W	0	0	0	0	0	0	#DIV/0!	0	0
YDL086C-/	0	0	0	0	0	0	#DIV/0!	0	0
YDL096C	0	0	0	0	0	0	#DIV/0!	0	0
YDL114W-/	0	0	0	0	0	0	#DIV/0!	0	0
YDL152W	0	0	0	0	0	0	#DIV/0!	0	0
YDL159C-I	0	0	0	0	0	0	#DIV/0!	0	0
YDL163W	0	0	0	0	0	0	#DIV/0!	0	0
YDL172C	0	0	0	0	0	0	#DIV/0!	0	0
YDL228C	0	0	0	0	0	0	#DIV/0!	0	0
YDR015C	0	0	0	0	0	0	#DIV/0!	0	0
YDR094W	0	0	0	0	0	0	#DIV/0!	0	0
YDR149C	0	0	0	0	0	0	#DIV/0!	0	0
YDR193W	0	0	0	0	0	0	#DIV/0!	0	0
YDR210W	0	0	0	0	0	0	#DIV/0!	0	0
YDR354C-/	0	0	0	0	0	0	#DIV/0!	0	0
YDR355C	0	0	0	0	0	0	#DIV/0!	0	0
YDR371C-/	0	0	0	0	0	0	#DIV/0!	0	0
YDR426C	0	0	0	0	0	0	#DIV/0!	0	0
YDR464C-/	0	0	0	0	0	0	#DIV/0!	0	0
YDR524C-/	0	0	0	0	0	0	#DIV/0!	0	0
YDR545C-/	0	0	0	0	0	0	#DIV/0!	0	0
YEL008C-/	0	0	0	0	0	0	#DIV/0!	0	0
YEL018C-/	0	0	0	0	0	0	#DIV/0!	0	0
YEL032C-/	0	0	0	0	0	0	#DIV/0!	0	0
YEL034W	0	0	0	0	0	0	#DIV/0!	0	0
YEL074W	0	0	0	0	0	0	#DIV/0!	0	0
YEL075W-/	0	0	0	0	0	0	#DIV/0!	0	0
YEL076C	0	0	0	0	0	0	#DIV/0!	0	0
YEL077W-/	0	0	0	0	0	0	#DIV/0!	0	0
YER023C-/	0	0	0	0	0	0	#DIV/0!	0	0

YER038W-/	0	0	0	0	0	0	#DIV/0!	0	0
YER067C-/	0	0	0	0	0	0	#DIV/0!	0	0
YER067W	0	0	0	0	0	0	#DIV/0!	0	0
YER079C-/	0	0	0	0	0	0	#DIV/0!	0	0
YER085C	0	0	0	0	0	0	#DIV/0!	0	0
YER088W-I	0	0	0	0	0	0	#DIV/0!	0	0
YER090C-/	0	0	0	0	0	0	#DIV/0!	0	0
YER138W-/	0	0	0	0	0	0	#DIV/0!	0	0
YER152W-/	0	0	0	0	0	0	#DIV/0!	0	0
YER172C-/	0	0	0	0	0	0	#DIV/0!	0	0
YER190C-/	0	0	0	0	0	0	#DIV/0!	0	0
YER190C-I	0	0	0	0	0	0	#DIV/0!	0	0
YFL013W-/	0	0	0	0	0	0	#DIV/0!	0	0
YFL031C-/	0	0	0	0	0	0	#DIV/0!	0	0
YFL063W	0	0	0	0	0	0	#DIV/0!	0	0
YFL065C	0	0	0	0	0	0	#DIV/0!	0	0
YFR009W-/	0	0	0	0	0	0	#DIV/0!	0	0
YGL014C-/	0	0	0	0	0	0	#DIV/0!	0	0
YGL041C	0	0	0	0	0	0	#DIV/0!	0	0
YGL063C-/	0	0	0	0	0	0	#DIV/0!	0	0
YGL123C-/	0	0	0	0	0	0	#DIV/0!	0	0
YGL132W	0	0	0	0	0	0	#DIV/0!	0	0
YGL152C	0	0	0	0	0	0	#DIV/0!	0	0
YGL177W	0	0	0	0	0	0	#DIV/0!	0	0
YGL188C-/	0	0	0	0	0	0	#DIV/0!	0	0
YGL218W	0	0	0	0	0	0	#DIV/0!	0	0
YGR031C-/	0	0	0	0	0	0	#DIV/0!	0	0
YGR068W-/	0	0	0	0	0	0	#DIV/0!	0	0
YGR073C	0	0	0	0	0	0	#DIV/0!	0	0
YGR115C	0	0	0	0	0	0	#DIV/0!	0	0
YGR151C	0	0	0	0	0	0	#DIV/0!	0	0
YGR226C	0	0	0	0	0	0	#DIV/0!	0	0
YGR270C-/	0	0	0	0	0	0	#DIV/0!	0	0
YGR296C-/	0	0	0	0	0	0	#DIV/0!	0	0
YGR296C-I	0	0	0	0	0	0	#DIV/0!	0	0
YHL050W-/	0	0	0	0	0	0	#DIV/0!	0	0
YHR052W-/	0	0	0	0	0	0	#DIV/0!	0	0
YHR055C	0	0	0	0	0	0	#DIV/0!	0	0
YHR056W-/	0	0	0	0	0	0	#DIV/0!	0	0
YHR073C-I	0	0	0	0	0	0	#DIV/0!	0	0
YHR073W-/	0	0	0	0	0	0	#DIV/0!	0	0
YHR137C-/	0	0	0	0	0	0	#DIV/0!	0	0
YHR165W-/	0	0	0	0	0	0	#DIV/0!	0	0
YHR180C-I	0	0	0	0	0	0	#DIV/0!	0	0
YHR182C-/	0	0	0	0	0	0	#DIV/0!	0	0
YHR193C-/	0	0	0	0	0	0	#DIV/0!	0	0
YHR212C	0	0	0	0	0	0	#DIV/0!	0	0
YHR212W-/	0	0	0	0	0	0	#DIV/0!	0	0
YHR219C-/	0	0	0	0	0	0	#DIV/0!	0	0
YIL021C-/	0	0	0	0	0	0	#DIV/0!	0	0
YIL029W-/	0	0	0	0	0	0	#DIV/0!	0	0
YIL059C	0	0	0	0	0	0	#DIV/0!	0	0
YIL066W-/	0	0	0	0	0	0	#DIV/0!	0	0
YIL100C-/	0	0	0	0	0	0	#DIV/0!	0	0

YIL105W-/	0	0	0	0	0	0	#DIV/0!	0	0
YIL142C-/	0	0	0	0	0	0	#DIV/0!	0	0
YIL156W-/	0	0	0	0	0	0	#DIV/0!	0	0
YIL171W-/	0	0	0	0	0	0	#DIV/0!	0	0
YIL176C	0	0	0	0	0	0	#DIV/0!	0	0
YIL177W-/	0	0	0	0	0	0	#DIV/0!	0	0
YIR017C	0	0	0	0	0	0	#DIV/0!	0	0
YIR041W	0	0	0	0	0	0	#DIV/0!	0	0
YJL015C	0	0	0	0	0	0	#DIV/0!	0	0
YJL020W-/	0	0	0	0	0	0	#DIV/0!	0	0
YJL026C-/	0	0	0	0	0	0	#DIV/0!	0	0
YJL077W-/	0	0	0	0	0	0	#DIV/0!	0	0
YJL197C-/	0	0	0	0	0	0	#DIV/0!	0	0
YJL217W	0	0	0	0	0	0	#DIV/0!	0	0
YJL222W-/	0	0	0	0	0	0	#DIV/0!	0	0
YJL225W-/	0	0	0	0	0	0	#DIV/0!	0	0
YJR022W	0	0	0	0	0	0	#DIV/0!	0	0
YJR140W-/	0	0	0	0	0	0	#DIV/0!	0	0
YKL100W-/	0	0	0	0	0	0	#DIV/0!	0	0
YKL145W-/	0	0	0	0	0	0	#DIV/0!	0	0
YKL156C-/	0	0	0	0	0	0	#DIV/0!	0	0
YKL224C	0	0	0	0	0	0	#DIV/0!	0	0
YKR033C	0	0	0	0	0	0	#DIV/0!	0	0
YKR035W-/	0	0	0	0	0	0	#DIV/0!	0	0
YKR075W-/	0	0	0	0	0	0	#DIV/0!	0	0
YLL019W-/	0	0	0	0	0	0	#DIV/0!	0	0
YLL037W	0	0	0	0	0	0	#DIV/0!	0	0
YLL044W	0	0	0	0	0	0	#DIV/0!	0	0
YLL066W-/	0	0	0	0	0	0	#DIV/0!	0	0
YLL066W-I	0	0	0	0	0	0	#DIV/0!	0	0
YLL067W-/	0	0	0	0	0	0	#DIV/0!	0	0
YLR101C	0	0	0	0	0	0	#DIV/0!	0	0
YLR155C	0	0	0	0	0	0	#DIV/0!	0	0
YLR156C-/	0	0	0	0	0	0	#DIV/0!	0	0
YLR156W	0	0	0	0	0	0	#DIV/0!	0	0
YLR157C	0	0	0	0	0	0	#DIV/0!	0	0
YLR157C-(	0	0	0	0	0	0	#DIV/0!	0	0
YLR158C	0	0	0	0	0	0	#DIV/0!	0	0
YLR159C-/	0	0	0	0	0	0	#DIV/0!	0	0
YLR159W	0	0	0	0	0	0	#DIV/0!	0	0
YLR160C	0	0	0	0	0	0	#DIV/0!	0	0
YLR161W	0	0	0	0	0	0	#DIV/0!	0	0
YLR163W-/	0	0	0	0	0	0	#DIV/0!	0	0
YLR170C	0	0	0	0	0	0	#DIV/0!	0	0
YLR235C	0	0	0	0	0	0	#DIV/0!	0	0
YLR280C	0	0	0	0	0	0	#DIV/0!	0	0
YLR286W-/	0	0	0	0	0	0	#DIV/0!	0	0
YLR299C-/	0	0	0	0	0	0	#DIV/0!	0	0
YLR317W	0	0	0	0	0	0	#DIV/0!	0	0
YLR347W-/	0	0	0	0	0	0	#DIV/0!	0	0
YLR364C-/	0	0	0	0	0	0	#DIV/0!	0	0
YLR366W	0	0	0	0	0	0	#DIV/0!	0	0
YLR399W-/	0	0	0	0	0	0	#DIV/0!	0	0
YLR462W	0	0	0	0	0	0	#DIV/0!	0	0

YLR463C	0	0	0	0	0	0	#DIV/0!	0	0
YLR465C	0	0	0	0	0	0	#DIV/0!	0	0
YLR466C-/	0	0	0	0	0	0	#DIV/0!	0	0
YLR467C-/	0	0	0	0	0	0	#DIV/0!	0	0
YML009W-I	0	0	0	0	0	0	#DIV/0!	0	0
YML101C-/	0	0	0	0	0	0	#DIV/0!	0	0
YML133W-/	0	0	0	0	0	0	#DIV/0!	0	0
YML133W-I	0	0	0	0	0	0	#DIV/0!	0	0
YMR013C-/	0	0	0	0	0	0	#DIV/0!	0	0
YMR031W-/	0	0	0	0	0	0	#DIV/0!	0	0
YMR141W-/	0	0	0	0	0	0	#DIV/0!	0	0
YMR153C-/	0	0	0	0	0	0	#DIV/0!	0	0
YMR272W-/	0	0	0	0	0	0	#DIV/0!	0	0
YMR307C-/	0	0	0	0	0	0	#DIV/0!	0	0
YNL013C	0	0	0	0	0	0	#DIV/0!	0	0
YNL097W-/	0	0	0	0	0	0	#DIV/0!	0	0
YNL120C	0	0	0	0	0	0	#DIV/0!	0	0
YNL144W-/	0	0	0	0	0	0	#DIV/0!	0	0
YNL171C	0	0	0	0	0	0	#DIV/0!	0	0
YNL174W	0	0	0	0	0	0	#DIV/0!	0	0
YNL226W	0	0	0	0	0	0	#DIV/0!	0	0
YNL228W	0	0	0	0	0	0	#DIV/0!	0	0
YNL296W	0	0	0	0	0	0	#DIV/0!	0	0
YNL339W-/	0	0	0	0	0	0	#DIV/0!	0	0
YNL339W-I	0	0	0	0	0	0	#DIV/0!	0	0
YNR003W-/	0	0	0	0	0	0	#DIV/0!	0	0
YNR042W	0	0	0	0	0	0	#DIV/0!	0	0
YOL083C-/	0	0	0	0	0	0	#DIV/0!	0	0
YOL150C	0	0	0	0	0	0	#DIV/0!	0	0
YOL163W	0	0	0	0	0	0	#DIV/0!	0	0
YOL164W	0	0	0	0	0	0	#DIV/0!	0	0
YOL164W-/	0	0	0	0	0	0	#DIV/0!	0	0
YOL166W-/	0	0	0	0	0	0	#DIV/0!	0	0
YOR068C	0	0	0	0	0	0	#DIV/0!	0	0
YOR108C-/	0	0	0	0	0	0	#DIV/0!	0	0
YOR161W-/	0	0	0	0	0	0	#DIV/0!	0	0
YOR161W-I	0	0	0	0	0	0	#DIV/0!	0	0
YOR169C	0	0	0	0	0	0	#DIV/0!	0	0
YOR186C-/	0	0	0	0	0	0	#DIV/0!	0	0
YOR200W	0	0	0	0	0	0	#DIV/0!	0	0
YOR231C-/	0	0	0	0	0	0	#DIV/0!	0	0
YOR277C	0	0	0	0	0	0	#DIV/0!	0	0
YOR325W	0	0	0	0	0	0	#DIV/0!	0	0
YOR333C	0	0	0	0	0	0	#DIV/0!	0	0
YOR335W-/	0	0	0	0	0	0	#DIV/0!	0	0
YOR379C	0	0	0	0	0	0	#DIV/0!	0	0
YOR394C-/	0	0	0	0	0	0	#DIV/0!	0	0
YOR394W	0	0	0	0	0	0	#DIV/0!	0	0
YOR396C-/	0	0	0	0	0	0	#DIV/0!	0	0
YPL044C	0	0	0	0	0	0	#DIV/0!	0	0
YPL114W	0	0	0	0	0	0	#DIV/0!	0	0
YPL135C-/	0	0	0	0	0	0	#DIV/0!	0	0
YPL136W	0	0	0	0	0	0	#DIV/0!	0	0
YPL222C-/	0	0	0	0	0	0	#DIV/0!	0	0

YPL250W-A	0	0	0	0	0	0	#DIV/0!	0	0
YPL251W	0	0	0	0	0	0	#DIV/0!	0	0
YPL282C	0	0	0	0	0	0	#DIV/0!	0	0
YPL283W-A	0	0	0	0	0	0	#DIV/0!	0	0
YPL283W-B	0	0	0	0	0	0	#DIV/0!	0	0
YPR038W	0	0	0	0	0	0	#DIV/0!	0	0
YPR039W	0	0	0	0	0	0	#DIV/0!	0	0
YPR050C	0	0	0	0	0	0	#DIV/0!	0	0
YPR077C	0	0	0	0	0	0	#DIV/0!	0	0
YPR160C-A	0	0	0	0	0	0	#DIV/0!	0	0
YPR160W-A	0	0	0	0	0	0	#DIV/0!	0	0
YPR169W-A	0	0	0	0	0	0	#DIV/0!	0	0
YPR170W-A	0	0	0	0	0	0	#DIV/0!	0	0
YPR177C	0	0	0	0	0	0	#DIV/0!	0	0
YPR203W	0	0	0	0	0	0	#DIV/0!	0	0
YPR204C-A	0	0	0	0	0	0	#DIV/0!	0	0

expressed genes					
WE_PT_2	WE_PT_3	WE_PT_4	Ratio	DESEQ p adj WA vs W	DESEQ p adj WE vs WE
6353.282	6074.573	6459.594	-1.12088	0.027170262	3.86469E-25
2488.242	2185.88	2271.966	-1.38686	0.012378121	2.01498E-22
976.5346	986.5723	896.0014	-0.04531	0.003408242	0.87466351
3461.203	3191.961	2842.054	0.47986	0.294546467	0.000316693
64.32799	72.46011	49.25388	1.118774	0.458109598	0.000210289
895.2311	847.2259	926.3921	0.286586	0.054030267	0.083362707
214.4266	211.8065	184.4401	-0.04188	0.004702869	0.922546597
10537.28	10713.88	11890.1	-0.51811	0.706392555	2.24404E-05
11.61478	7.431806	7.335684	-0.74971	0.730407446	0.168011786
3423.678	3377.756	2968.856	-0.24414	0.864617666	0.086902545
124.1888	130.9856	120.5148	0.691215	0.186913093	0.003993975
0	1.857952	2.09591	#DIV/0!	0.78798308	0.286863208
339.5088	325.1415	356.3047	-0.97525	0.73478662	4.23639E-08
1552.806	1688.878	1591.843	0.096678	0.15308256	0.638524044
3.573777	5.573855	5.239774	-0.78356	0.547164305	0.415175074
34.84433	34.3721	29.34274	-0.29944	0.546104275	0.465657685
1354.461	1280.129	1401.116	-0.57761	0.515068409	2.16222E-05
1397.347	1390.677	1444.082	-0.58819	0.732140966	5.20296E-07
52.71321	42.73289	44.01411	-0.0028	0.12999813	0.975879427
20605.5	20559.16	18642.07	-0.15409	0.059230691	0.381582724
3632.744	3802.298	3992.708	-0.1337	0.484643556	0.388037549
14043.16	13632.72	14064.6	-0.23603	0.614621507	0.241819205
1.786889	0	0	-0.91651	0.542904501	0.835596295
5946.765	5896.209	6320.216	-0.18874	0.158071272	0.19887348
37.52466	34.3721	54.49365	-0.53053	0.004616731	0.088947033
503.0091	500.7179	507.2102	0.413688	0.990604058	0.064390809
1559.954	1781.776	1722.838	-0.15924	0.649714874	0.422560599
22916.85	23307.07	20944.43	0.090238	0.637435429	0.536500997
27.69677	36.23005	47.15797	-0.15296	0.651806624	0.758356955
37.52466	87.32372	31.43865	-0.09678	0.953670495	0.908612163
134.9101	115.193	171.8646	0.078639	0.919799198	0.886786965
1327.658	1321.933	1592.891	-0.32686	0.98843748	0.089560109
44320.2	42404.96	47848.57	-0.13625	0.883628087	0.923725803
7.147554	2.786927	1.047955	-0.60495	0.98299578	0.670601121
1040.863	983.7853	785.9662	-0.64755	0.603004299	0.000188607
37.52466	29.72722	24.10296	0.323	0.662090696	0.483238043
0	0	0	#DIV/0!	0.844410461	0.990142758
684.3783	663.2887	737.7602	-0.131	0.40625521	0.493837083
0	0	0	#DIV/0!	0.189698167	0.990142758
305.5579	264.7581	292.3794	0.090345	0.990604058	0.826228143
2912.628	2674.521	2949.993	0.118727	0.690439171	0.558104763
0	0	0	#DIV/0!	0.555061446	0.990142758
3.573777	6.50283	3.143865	-0.50484	0.600008341	0.718291318
29.48366	18.57952	41.9182	-0.09505	0.094904827	0.907473183
975.6411	892.7457	1045.859	0.521982	0.125163885	0.000116053
45345.87	44798	42196.95	-0.09998	0.272911001	0.378149996
1195.428	1178.87	1147.511	-0.61162	8.72064E-09	1.71746E-11
3.573777	12.07668	1.047955	0.745807	0.172063056	0.545985077
969.387	946.6263	879.2342	-0.25524	0.226746984	0.027336701
31104.37	29714.22	33195.02	-0.14091	0.948896953	0.36518668

1778.848	1813.361	1767.9	0.258487	0.182617755	0.13251575
1.786889	0	0	-0.55981	0.614621507	0.836555519
4.467221	2.786927	6.287729	-0.41221	0.614563272	0.726171034
0.893444	2.786927	0	1.125891	0.867574132	0.656368654
1133.781	1240.183	1215.628	-0.03441	0.128482365	0.921976304
338.6154	376.2352	318.5783	-0.39499	0.07122269	0.113402813
2330.996	2231.4	2451.166	0.215802	1.87221E-20	0.129766751
9474.976	9526.646	8689.642	-0.53816	0.000676906	4.97881E-11
5.360666	7.431806	12.57546	-0.70905	3.15538E-09	0.333065318
145.6314	109.6191	136.2341	-0.39457	0.527593235	0.093593
4450.246	4602.146	4671.783	-0.24643	0.753964428	0.031785223
86.66409	107.7612	104.7955	-0.17741	8.48569E-11	0.595423496
6128.134	5849.76	5284.837	-0.55332	0.585115533	3.99971E-09
281.4349	299.1302	337.4415	0.130639	0.02597477	0.588622174
489.6075	462.6299	438.0451	-0.59796	0.120800067	0.000280828
892.5508	981.9274	1019.66	-0.34893	0.962011634	0.03084725
209.066	222.0252	214.8308	-0.03317	0.061571261	0.914772543
8709.295	9353.857	7135.525	-0.16824	0.944990108	0.355130219
121981.1	119492.3	110459.7	-0.11933	0.733488302	0.353286634
105.4264	121.6958	98.50776	-0.14402	0.92288341	0.655129754
661.1488	597.3314	520.8336	-0.08298	0.861862766	0.834332496
4276.024	4053.121	4616.241	0.098181	0.031086718	0.576591259
136.697	135.6305	180.2482	-0.3985	0.990604058	0.351487828
1057.838	938.2655	1045.859	-0.94983	0.134941849	0.026729458
253.7382	308.42	268.2765	-0.09358	0.974826797	0.684131921
0	0	0	#NUM!	0.58371648	0.342128545
695.0996	652.141	637.1566	0.274234	0.957996061	0.162717225
1668.954	1614.56	1728.078	0.05839	0.670951846	0.80030609
359.1646	458.914	498.8265	-0.7048	0.081828292	1.89846E-06
9611.673	9685.501	8642.484	-0.36509	0.004702869	0.000988656
5693.027	5872.056	5862.26	-0.0581	6.44159E-19	0.614824771
149.2052	173.7185	206.4471	-0.71161	2.1131E-07	4.14592E-05
4337.672	4624.441	4507.254	-0.07644	0.826203568	0.5847566
6375.618	6618.023	6135.776	-0.73031	1.30946E-07	9.07231E-14
1362.502	1251.33	1382.253	-0.30573	0.004149955	0.038141651
0	0	0	#DIV/0!	0.662355315	0.990142758
305.5579	290.7694	266.1805	0.09361	0.767527949	0.813492611
10307.67	10356.22	11754.91	0.289893	0.810878938	0.028626623
460.1238	437.5476	500.9224	-0.0672	0.91441696	0.811818841
638.8126	604.7632	472.6277	-0.44511	0.621093622	0.039433267
1814.585	1964.784	1927.189	0.359345	0.13734229	0.000319026
23213.47	23870.96	21212.7	-0.00159	0.193158044	0.993537704
423.4926	480.2805	625.6291	0.190867	0.070427808	0.411765294
1299.961	1224.39	1423.123	0.547537	0.504924333	7.58405E-05
29851.76	29163.34	27399.83	-0.01511	0.753964428	0.931618549
234.0824	182.0792	171.8646	-0.70945	0.192357364	0.039860461
234.0824	208.0906	314.3865	-0.15109	0.826883467	0.644875718
3842.704	3530.108	4388.835	-0.20552	0.743382516	0.172785623
530.7059	509.0787	703.1777	-0.01895	0.861070915	0.954839814
17513.29	18023.99	17273.44	-0.0756	0.189947746	0.562110601
414.5581	477.4935	506.1622	-0.04825	0.022126119	0.88945982
89.34443	90.11065	126.8025	-0.54068	0.990604058	0.27042216
17991.29	18602.74	20732.74	0.128448	0.173563763	0.338625904
23.22955	44.59084	23.05501	-0.31259	0.312335761	0.527784555

10.72133	8.360782	3.143865	-0.77742	0.072365168	0.351487828
4429.697	4220.337	4159.333	0.167971	0.036671148	0.118990473
259.9923	332.5733	358.4006	-0.75117	1.38834E-05	4.34522E-06
0	0	0	#DIV/0!	0.907095813	0.990142758
2122.824	2154.295	2206.993	0.371889	0.837998472	0.001092886
1050.69	1031.163	1161.134	0.098519	0.182335052	0.664575474
0	0	0	#NUM!	0.925713018	0.169296115
41.99188	31.58518	26.19887	-0.62731	0.963492814	0.112630735
128.656	126.3407	183.3921	0.502212	0.045192099	0.060227128
6128.134	5853.476	7306.342	-0.27914	0.527593235	0.058035556
354.6974	290.7694	475.7715	0.389238	0.80496489	0.142939621
225.148	203.4457	223.2144	-1.04371	0.165543705	0.016996857
4406.467	4400.558	5357.145	-0.26695	0.899653152	0.102776029
158.1396	169.0736	127.8505	0.023013	0.98409529	0.973438605
0	0	0	#DIV/0!	0.682184622	0.990142758
68.79521	59.45445	50.30183	-0.57153	0.362090257	0.045970851
111.6805	133.7725	154.0494	-0.26358	0.681419018	0.317745056
119.7215	163.4997	182.3442	0.142605	0.151730694	0.696921855
999.7641	1101.765	1255.45	0.656614	0.894528942	0.000266857
820.1818	853.7287	989.2694	-0.90652	0.000701205	2.13594E-10
12650.28	12398.11	14067.75	-0.06282	2.46892E-37	0.664491515
769.2555	815.6407	822.6446	-0.86253	5.99679E-07	4.24394E-08
1007.805	910.3962	1104.544	0.693165	0.029779632	2.65543E-05
194.7708	214.5934	315.4344	-0.6172	0.886112674	0.002842502
243.9103	248.0365	245.2214	-0.28914	0.585115533	0.163640813
900.5918	908.5383	953.639	0.0908	1.28599E-05	0.590570691
554.8289	654.9279	581.615	-0.31469	3.6065E-05	0.216876753
7142.193	7324.974	8612.093	0.070244	0.484084014	0.759231714
2225.57	2185.88	2169.267	-0.07136	0.826883467	0.694075461
8085.671	8211.217	7921.491	0.152784	0.416597489	0.098741638
1639.47	1732.54	1667.296	0.090979	0.478079371	0.572980704
460.1238	423.6129	439.0931	-0.04019	0.669419617	0.914120833
13648.25	12893.25	15358.83	-0.22995	0.351290569	0.083993003
9831.461	9701.294	11152.34	-0.04451	0.944990108	0.844571336
43.77877	26.9403	27.24683	-0.20686	0.028010538	0.69424407
1035.502	968.9217	1032.236	-0.34817	0.261041442	0.012748693
20.54922	20.43747	19.91114	-0.35873	0.967813477	0.556915963
2909.948	2917.913	2795.944	-0.71317	0.000217366	1.85992E-19
75.04932	78.03396	95.3639	-0.23829	0.969843993	0.477962261
7781.006	8030.995	9627.562	-0.83631	0.001290762	6.12454E-15
1628.749	1636.855	1799.339	-0.10099	0.270719269	0.564967632
173473.8	167271.4	165214.3	-0.05643	0.967813477	0.752110266
3360.244	3151.086	3625.924	-0.10638	0.614621507	0.567449963
1526.896	1482.645	1553.069	0.114212	0.455280748	0.459459722
1275.838	1219.745	1231.347	0.03651	7.43351E-21	0.847597088
0	1.857952	0	#DIV/0!	0.959739969	0.583691606
246.5906	261.0422	251.5092	0.068187	0.738405909	0.802920813
0	0	0	#DIV/0!	0.654781049	0.990142758
1405.388	1445.486	1678.824	0.101312	8.77792E-20	0.544257884
1943.241	1763.196	1672.536	0.159192	0.867574132	0.322653852
8326.007	8063.51	8577.511	0.159424	0.016500228	0.238463934
1164.158	1102.694	1314.135	0.123139	0.792868376	0.656460739
386.8614	380.8801	523.9774	-0.64029	0.995598118	0.000246256
424.386	470.9907	513.4979	0.377547	0.090626117	0.106176664

136.697	143.0623	129.9464	-0.29786	0.767300341	0.2533373
468.1648	489.5702	400.3188	0.288235	0.040355829	0.255165763
12780.72	13140.36	13653.8	0.589302	0.651961753	4.81136E-09
236.7627	203.4457	278.756	-0.23202	0.004455063	0.56192082
943.4771	1060.89	1166.374	-0.34552	0.003329096	0.037467608
1517.962	1399.966	1304.704	0.23055	0.723947258	0.232212558
12084.73	11886.24	12720.08	0.066868	0.94035236	0.724404377
0	0	0	#DIV/0!	0.207579254	0.990142758
344.8695	393.8857	376.2158	-0.02852	0.862474177	0.942591831
1032.822	1028.376	1075.202	-0.61474	0.880606478	0.000111761
771.0424	949.4132	1064.722	-0.45727	0.582750031	0.003921392
154.5659	135.6305	171.8646	0.380992	0.01405096	0.3930505
1593.905	1385.103	1912.518	-0.20055	1.01191E-18	0.383759687
0	1.857952	0	-0.50354	0.92288341	0.840718213
317.1727	310.2779	426.5176	-0.59567	0.431874361	9.85399E-05
1819.946	1777.131	2153.547	-0.43836	0.498379067	0.001931053
789.8047	842.581	869.8026	0.078987	0.735300868	0.744396459
2951.046	2872.393	3632.212	0.299981	0.722604138	0.109807224
2555.251	2550.967	3033.829	-0.19704	0.666493976	0.219522094
2320.275	2380.036	2112.677	0.075446	0.780429274	0.669238482
2656.21	2625.286	2799.088	-0.09739	0.228465218	0.584884922
0	0	0	#DIV/0!	0.975298616 NA	
2138.012	2158.011	2098.006	-0.12015	1.51549E-25	0.38666632
15.18855	18.57952	23.05501	0.138092	0.081224331	0.875604624
16004.27	16048.99	15323.2	0.074701	0.689047979	0.616511903
51.81977	31.58518	50.30183	0.208637	0.746342492	0.738359307
4967.55	4951.441	5065.814	0.113267	0.723947258	0.364490432
2141.586	1883.034	1990.066	0.13596	0.967813477	0.434126424
939.0099	786.8425	949.4471	-0.3299	0.576135666	0.036801389
4262.623	4277.933	4662.351	0.002726	0.901380807	0.990023409
229.6152	219.2383	255.701	-0.05586	0.31587804	0.90550917
1858.364	1856.094	2050.848	-0.01587	0.986807798	0.94960537
1407.175	1419.475	1490.192	0.078132	0.745645324	0.679871393
1039.076	1042.311	1205.148	-1.3243	0.430087902	1.4546E-29
8201.818	8332.913	10050.94	0.320438	0.91014027	0.047449206
3733.704	3500.381	3489.69	-0.06158	1.00576E-13	0.697416669
1543.872	1676.801	1846.497	-0.18335	6.62105E-05	0.255474802
1132.887	1096.191	1435.698	0.007816	0.365690431	0.984451848
2373.881	2512.879	3025.446	0.184628	0.577190434	0.336932642
2105.848	2175.661	1774.188	0.216343	2.22109E-08	0.420331471
4953.255	4821.384	4754.571	0.094697	0.179898822	0.477876695
2239.865	2282.493	2391.433	0.22272	0.767527949	0.202680028
2025.438	1910.903	2049.8	-0.14947	0.070750961	0.181864152
501.2222	504.4338	492.5388	0.01332	0.00756732	0.969186214
7348.579	7039.778	6934.318	0.086783	0.50823828	0.492537442
70.5821	93.82655	83.83639	0.238301	0.554725555 NA	
1440.232	1509.586	1898.894	-0.29116	0.613137272	0.080369062
134.9101	152.352	137.2821	0.03576	0.0053461	0.928448576
2343.504	2299.215	2523.475	-0.22075	0.621093622	0.102132739
293.0497	311.2069	273.5162	0.20835	0.099575114	0.56311517
262.6726	261.9712	287.1396	-0.44505	0.842190796	0.029788115
18377.25	19337.56	19535.98	0.128505	0.583613744	0.306201671
1399.134	1413.901	1731.221	-0.37319	0.917774343	0.013575411
812.1408	769.1919	925.3442	-0.18506	0.077927263	0.407575574

175.1151	141.2043	165.5769	0.10515	0.960823113	0.759033711
582.5257	598.2604	756.6234	-0.51588	0.835449534	0.000775343
23.22955	7.431806	6.287729	-0.78934	0.200422675	0.229480039
569.124	536.948	734.6164	-0.88951	0.296861073	2.55912E-09
581.6322	659.5728	712.6093	-0.32165	0.873978289	0.060855027
587.8863	563.8883	582.6629	-0.73785	0.961125122	8.75469E-05
79596.95	73564.66	69660.71	0.031841	0.218682865	0.841212475
416.345	473.7776	479.9633	0.47945	0.27470085	0.040331036
216.2135	334.4313	316.4824	-0.24528	0.46017679	0.361564535
10487.25	10676.72	11446.81	0.342666	0.264355919	0.000164663
4636.976	4420.996	4622.529	-0.00112	0.361731257	0.998953832
72.36898	68.74421	127.8505	0.149487	0.812875146	0.751258336
1785.995	1798.497	2004.738	-0.1613	0.195740155	0.389507343
41.99188	45.51981	58.68547	-0.36807	0.345374681	NA
186.7298	212.7354	254.653	-0.289	0.848681983	0.218832902
3715.835	3727.98	4037.77	0.177371	0.582558658	0.087007219
2617.792	2812.01	2594.736	-0.03544	0.897759686	0.855100406
193.8774	229.457	236.8378	0.113884	0.038561058	0.73912962
10526.56	10334.86	11890.1	0.112403	0.783200259	0.394829397
296.6235	235.9598	293.4274	-0.14687	0.243847797	0.636683257
8.934443	8.360782	2.09591	-0.15577	0.864963799	0.905168779
752.2801	749.6834	926.3921	0.03346	0.577578732	0.907473183
244.8037	226.6701	207.4951	-0.52846	0.189947746	0.162970478
1410.748	1266.194	1429.41	0.23319	0.62498123	0.205836834
7161.849	7040.707	7901.58	-0.27725	0.759069408	0.063171194
14378.2	14491.09	14920.78	0.249301	0.119079209	0.015923242
163.5003	216.4514	189.6798	-0.2726	0.309108798	0.535992604
266.2464	269.403	291.3315	0.013394	0.856763291	0.964974564
2372.095	2293.641	2686.956	-0.11171	0.000169043	0.46008868
11681.78	11889.96	11776.92	0.170582	0.482562486	0.126656023
0	5.573855	5.239774	-0.36315	0.848847805	0.742968787
687.0586	692.0869	717.8491	-0.48897	5.68875E-08	0.017241442
1066.772	1149.143	1171.614	0.127229	0.867587609	0.488462858
30.3771	12.07668	18.86319	-0.18398	0.310443697	0.819964665
1.786889	0	0	#DIV/0!	0.853331381	NA
12580.59	12533.74	13447.36	0.032571	0.779325825	0.840173173
424.386	379.0221	493.5868	-0.5717	6.11612E-05	0.017610586
4628.041	4527.828	4404.554	-0.06661	0.775670478	0.640605635
51765.27	50435.95	50967.29	-0.10383	0.941323092	0.44710318
419.0254	413.3942	442.237	-0.79069	1.16423E-14	1.25565E-11
1281.199	1265.265	1477.616	-0.09096	0.981023664	0.697416669
532.4928	625.2007	569.0395	-0.00894	0.16816951	0.975406172
195.6643	165.3577	142.5219	-0.17242	0.666493976	0.718440886
58.07388	48.30674	76.50071	0.282298	0.772432927	0.521104798
715.6489	731.1039	676.9789	0.391082	0.313396084	0.036487394
1039.969	1044.169	937.9196	-0.11517	0.585762809	0.611282744
28.59022	19.50849	27.24683	-0.15851	0.695990097	0.835596295
7.147554	14.86361	8.383639	-0.13633	0.701216566	0.919616146
1663.593	1732.54	2003.69	-0.47084	0.901380807	0.000192079
377.0335	335.3602	435.9492	-0.34137	0.139081773	0.071525598
3343.268	3372.182	3501.217	-0.23167	0.626788166	0.055974476
1584.97	1423.191	1091.969	0.161438	0.019988247	0.519913808
6216.585	5938.013	5597.127	0.153836	0.708999466	0.283557823
3372.752	3562.622	3396.422	-0.57975	0.292435632	1.78403E-15

187.6233	184.8662	171.8646	-1.65803	0.000902992	3.46599E-18
1783.315	1852.378	2063.423	-0.39159	3.02032E-05	0.004542562
1008.699	912.2542	1045.859	-0.10711	0.017639055	0.654628753
169.7544	176.5054	167.6728	0.300278	0.193158044	0.290714074
630.7716	607.5501	492.5388	-0.05518	0.012324166	0.873196229
256.4185	263.8291	291.3315	0.263003	0.854862911	0.195644716
201.025	192.298	207.4951	0.090165	0.42694305	0.840777068
1168.625	1174.225	1218.772	0.166904	0.29215357	0.379845684
27.69677	34.3721	34.58251	-0.25637	0.572336545	NA
3817.687	3849.676	4095.408	0.486927	0.189947746	1.11988E-07
17283.68	16916.65	18743.72	0.086322	0.60948547	0.628282957
2506.111	2410.692	2910.171	-0.02825	0.659640784	0.91372954
2134.438	2272.275	2433.351	-0.43192	0.429975868	0.000123311
1946.815	2192.383	2019.409	-0.15989	0.000302523	0.543070132
345.7629	321.4256	381.4556	0.097841	0.68563527	0.694987858
62.5411	45.51981	46.11002	0.227621	0.621093622	NA
720.1161	763.6181	964.1185	-0.30425	0.356652725	0.176171477
145.6314	152.352	139.378	0.674727	0.831439424	0.062400751
1848.536	1772.486	1869.552	-0.25489	0.827268091	0.076189989
126.8691	91.03962	100.6037	-0.0235	0.976681492	0.984456511
305.5579	367.8744	233.6939	0.409529	0.06089504	0.382319285
629.8782	644.7092	473.6756	-0.01307	0.95250253	0.971658868
47.35255	60.38342	50.30183	0.411496	0.399514053	NA
3.573777	1.857952	4.19182	0.155398	0.405459844	NA
47.35255	51.09367	52.39774	0.029941	0.30979442	0.951184679
393.1155	354.8687	328.0099	-0.23444	2.17828E-05	0.394829397
2647.275	2623.428	2860.917	-0.45284	0.884585407	9.27394E-06
22334.32	21785.41	23289.75	-0.20805	0.800609294	0.069345614
1563.527	1649.861	1730.174	0.152172	0.936346196	0.313958096
3778.376	3354.531	2747.738	0.290028	0.92288341	0.548866742
4543.164	4391.268	4565.939	-0.19323	0.043196135	0.117751922
2251.48	2289.925	2538.147	-0.39261	0.008454108	0.000306119
379.7138	343.721	632.9648	-0.08917	0.878081019	0.896421455
3731.917	3671.312	3679.37	-0.11983	0.358629856	0.27042216
19.65577	11.14771	12.57546	-1.31203	0.320833466	0.014483576
1455.421	1571.827	1677.776	0.042978	0.000288624	0.861226913
658.4684	585.2547	636.1086	0.152066	0.948234757	0.612263058
4649.484	4764.717	5277.501	0.155766	0.896968012	0.365253762
38.4181	22.29542	38.77433	0.376965	0.990604058	NA
536.0666	556.4565	429.6615	0.074837	0.000701205	0.765988241
11102.83	11308.42	11605.05	0.426488	0.060383326	1.33287E-07
3756.933	3814.374	3856.474	-0.1345	0.065480816	0.236801834
25077.19	25333.17	26807.73	0.257265	0.193158044	0.046834479
405.6237	311.2069	309.1467	0.292666	0.199696516	0.215394284
2884.038	2477.578	2539.195	0.110556	0.148209117	0.505302546
3.573777	7.431806	14.67137	-0.1902	0.036478363	0.832624087
34.84433	31.58518	27.24683	-0.17769	0.835449534	0.764338813
1310.683	1295.921	1301.56	-0.06047	0.09473638	0.712929046
1488.478	1869.099	1739.605	-0.43978	0.669696898	0.001362953
745.1325	834.2202	671.7391	-0.0266	0.777280242	0.923725803
499.4353	612.195	541.7927	-0.21418	5.71158E-06	0.293216205
413.6647	507.2208	581.615	0.092591	0.871252597	0.765179484
2817.923	2692.172	2825.286	-0.13519	0.039854593	0.231047495
5818.109	5754.076	6126.344	-0.05797	0.145043473	0.73912962

2505.218	2525.885	2766.601	-0.02023	0.890248597		0.912679298
5080.124	5088	6213.325	-0.04011	0.307232061		0.860333535
2166.602	2230.471	2590.544	0.010901	0.637486498		0.965436319
39.31155	19.50849	39.82229	0.384755	0.603004299		0.753881265
184.943	129.1276	199.1114	-0.75186	0.018359753		0.000429882
311.812	247.1076	318.5783	-0.30206	0.44839829		0.217256598
19068.78	19760.24	19785.39	0.388508	0.40568693		2.89678E-05
14918.73	14744.7	13986.01	0.257479	0.136870362		0.024833343
1107.871	958.703	1125.504	0.36128	0.951118826		0.138699579
578.0584	668.8625	664.4034	0.178627	0.100000333		0.552800466
1239.207	1218.816	1099.305	0.145252	0.229707909		0.359566588
5674.264	5859.979	6087.57	0.07791	0.603849055		0.575540189
12834.33	12607.13	13132.97	-0.24653	0.045913153		0.02329362
397.5827	411.5363	450.6206	0.154355	0.716063981		0.59853578
11475.4	10915.47	10762.5	-0.32815	0.865564724		0.101229705
855.0262	829.5754	944.2074	0.37592	0.335983122		0.007452176
5722.51	5712.272	5296.364	0.216525	0.724149904		0.078871289
223.3611	233.1729	263.0367	-0.59382	1.9456E-18		0.00064005
42304.59	42920.54	36314.78	-0.05726	0.653304895		0.798292995
392.222	441.2635	341.6333	0.032943	0.664286641		0.930336535
2769.677	2667.089	2850.437	0.011105	0.682715357		0.952666818
1099.83	1223.461	1304.704	-0.06325	0.001404555		0.765870575
4070.532	3945.36	3539.992	-0.24771	0.006229103		0.118990473
2909.948	2629.001	2838.91	0.28648	0.529339672		0.03394343
317.1727	258.2553	227.4062	0.049871	0.729364673		0.87466351
859.4934	861.1605	903.3371	-0.13528	0.394915233		0.42381604
4320.696	4083.777	4298.711	-0.2247	0.002515233		0.252406149
287.6891	265.6871	338.4894	-0.41664	0.720090812		0.176171477
5535.781	5537.625	5639.045	0.273031	0.29215357		0.042179555
21.44266	32.51415	25.15092	-0.09097	0.657109304		0.929846474
815.7146	797.0612	755.5755	-0.06566	0.001759401		0.835596295
7395.038	7020.27	6718.439	0.047245	0.811082292		0.775439768
4359.115	4530.615	5367.625	-0.39287	0.923281561		0.003888622
1822.626	1789.207	1976.443	0.966717	0.000288624		1.47331E-16
381.5007	429.1868	447.4767	-0.34221	0.02496009		0.069635315
162.6069	158.8549	172.9126	-0.22496	0.641408722		0.519258549
5807.388	5388.988	5874.835	-0.2608	0.758716249		0.04168446
60.75421	55.73855	58.68547	-0.61139	0.040661421		0.139776747
421.7057	372.5193	316.4824	-0.07481	0.439271214		0.851593116
1131.1	1144.498	980.8858	0.104802	0.000498713		0.680780346
589.6732	510.9367	484.1552	0.308956	0.175067813		0.149383412
3751.572	3920.278	4252.601	-0.47609	0.814155137		1.97977E-06
6480.151	6262.226	6801.227	-0.07279	2.50477E-06		0.634183341
1808.331	1772.486	1933.477	-0.20336	0.249012381		0.237811367
2092.446	2137.573	2001.594	-0.18465	0.80107822		0.328024792
52.71321	60.38342	49.25388	1.010937	0.79502649	NA	
390.4351	362.3005	366.7842	-0.04962	0.905906764		0.86814025
2595.456	2531.459	2933.226	0.030211	0.145286779		0.899731862
4301.934	4096.783	4581.659	0.070857	0.662776675		0.699983589
49.13943	54.80957	49.25388	-0.62138	0.274043459		0.060462725
737.0915	844.439	727.2807	-0.3917	5.32047E-05		0.026450935
961.346	1072.038	931.6319	0.120527	0.086711881		0.65282506
0	0.928976	0	#DIV/0!	0.509152281	NA	
870.2147	879.74	818.4528	0.171966	0.469683278		0.257842956

531.5993	491.4282	463.1961	0.204524	0.003450987	0.381794066
0	0	0	#NUM!	0.814031874	NA
309.1317	354.8687	425.4697	-0.27868	0.587282007	0.277654437
1701.118	1778.06	1829.729	-0.06809	0.897759686	0.706703794
937.223	943.8394	1160.086	-0.01348	0.310443697	0.956290296
571.8043	623.3427	518.7377	0.077393	0.392876266	0.795076358
3624.703	3870.113	3491.786	0.08758	0.730204291	0.562654945
1735.069	1742.759	1820.298	0.493793	0.310443697	9.79395E-06
5106.034	5225.489	5607.607	-0.0379	0.515985444	0.841070222
5388.362	5302.594	4926.436	-0.08557	0.189917625	0.553711578
840.731	910.3962	821.5966	0.091051	0.395079365	0.663320474
0	0	0	#DIV/0!	0.992476114	NA
1244.568	1374.884	1560.405	-0.16576	0.817609984	0.4158179
737.985	693.9449	698.9859	0.013876	0.827035335	0.948353155
495.8616	435.6896	463.1961	0.377762	0.0477642	0.144365513
1108.764	1074.825	1321.471	0.010054	0.651961753	0.975879427
926.5017	940.1235	793.3019	0.063838	0.335264059	0.872630949
58.07388	80.82089	90.12412	-0.23579	0.106548765	0.609543168
711.1816	678.1523	601.5261	0.123566	0.735300868	0.599924988
7377.169	6914.367	7509.645	-0.63323	2.40534E-08	7.36517E-16
2064.75	1897.897	1611.755	0.141137	0.897759686	0.535992604
733.5177	836.0782	675.9309	-1.26102	0.306444733	0.006017374
26.80333	37.15903	30.39069	0.372765	0.577578732	NA
178.6889	184.8662	203.3032	0.354387	0.203841572	0.307538923
1833.348	1778.989	1313.087	0.514702	0.365690431	0.009048373
422.5991	378.0931	367.8322	-0.02932	0.632040487	0.928540862
46.4591	59.45445	81.74048	-0.16539	0.585762809	0.742199342
5426.78	5160.46	5689.347	-0.14907	0.285144055	0.230502062
1587.65	1434.339	1663.104	0.107258	0.306444733	0.547627123
11.61478	7.431806	5.239774	0.447317	0.779325825	NA
118.8281	125.4117	145.6657	-0.58759	0.000892317	0.053436877
2299.726	2112.491	2517.188	0.109176	0.787792061	0.513503617
502.1157	565.7462	528.1693	-0.38778	0.403965481	0.102175745
125.9756	115.193	118.4189	-0.62566	0.682715357	0.057548545
3225.334	3296.006	3403.757	0.076255	0.817609984	0.583053599
406.5171	443.1214	444.3329	-0.28698	0.685238785	0.208176931
655.7881	741.3227	823.6925	-0.27893	0.394915233	0.096786584
600.3945	632.6325	682.2186	-0.09696	0.43802737	0.6924902
33.05744	18.57952	38.77433	0.120819	0.804360442	0.868094937
1279.412	1316.359	1151.702	0.267903	0.988569159	0.190716353
40.20499	31.58518	61.82934	-0.39003	0.973340554	NA
2231.824	2101.343	1993.21	0.148459	0.258861617	0.354829766
1240.101	1251.33	1111.88	0.197637	0.263755993	0.273845028
2245.225	2242.547	2641.894	0.129183	0.314856356	0.659338327
911.3131	925.2599	897.0494	0.080169	0.007621754	0.690106308
9768.92	9404.951	9869.639	0.101411	0.034815958	0.335921009
3321.826	3222.617	3194.167	0.061149	0.926122159	0.676039277
1983.446	2012.161	2192.322	-0.08534	0.864617666	0.583691606
2129.971	2136.644	2480.509	0.114925	0.657109304	0.602240104
3031.456	2959.717	3135.481	0.050103	0.470002734	0.745719365
17625.87	16661.18	17119.39	-0.27663	0.6744135	0.011689383
6413.143	6197.197	7406.945	-0.14713	0.606270396	0.351399771
1040.863	1065.535	1128.647	-0.18167	0.626294604	0.146138686
14600.67	13803.65	15130.37	0.249394	0.331331472	0.074687558

2344.398	2322.439	2082.286	0.376441	0.935523245		0.030371149
1971.831	1813.361	1761.612	0.210945	0.55438266		0.224439117
0.893444	0.928976	1.047955	-1.58993	0.582123698	NA	
3010.014	2760.916	3229.797	-0.3705	0.351446149		0.004740829
3508.556	3364.75	3460.347	-0.00236	0.39175021		0.993537704
1307.109	1321.004	1313.087	0.030526	0.134933146		0.884584155
1573.355	1436.197	1688.255	-0.03414	0.981023664		0.889287206
3927.581	3815.303	4287.183	0.154698	0.826883467		0.238211044
530.7059	554.5985	559.6079	-0.51896	0.000831429		0.000873642
146.5249	185.7952	164.5289	-0.45278	0.654926306		0.045330931
429.7467	452.4112	553.3202	0.186722	0.060257212		0.474497273
1059.625	949.4132	970.4062	0.086341	0.901266975		0.647117771
608.4355	559.2434	498.8265	0.049182	0.374469996		0.847133587
0	1.857952	0	#DIV/0!	0.522454877	NA	
431.5336	467.2748	486.2511	0.160083	0.787792061		0.609543168
20039.06	19451.82	22116.04	0.888502	0.028479181		2.3991E-17
68.79521	48.30674	60.78138	0.299528	0.321300373		0.502183544
1985.233	2006.588	1869.552	0.018045	0.130616009		0.941096477
404449.7	393264.2	379960.1	-0.04415	0.894528942		0.812861677
10834.8	11109.62	10758.3	-0.34818	0.31587804		0.001069433
1094.469	1074.825	1103.497	0.106997	0.465047544		0.467083166
764.7883	673.5074	634.0127	0.121921	0.01192228		0.606332686
4843.361	4671.819	4853.079	-1.00003	0.120176384		2.23384E-22
21932.27	21815.14	19586.28	-0.04458	0.728952588		0.765789524
0.893444	0.928976	3.143865	#DIV/0!	0.802471571	NA	
2034.373	1966.642	1816.106	0.295168	0.811082292		0.047449206
334.1482	397.6016	366.7842	0.095846	0.541271961		0.752110266
1713.626	1713.96	1636.906	0.10537	0.752287766		0.55088846
483.3533	541.5929	661.2595	-0.08232	0.141936131		0.811387384
2210.381	2355.883	2157.739	-0.03567	0.564427849		0.872213394
3791.777	4152.522	4182.388	0.059687	0.501025279		0.680271881
46404.6	45571.83	45071.49	-0.11786	0.28025041		0.477783776
753.1735	625.2007	698.9859	0.227687	0.948755315		0.227088314
176.0085	171.8605	219.0226	-0.31941	0.009055285		0.33000998
5468.772	5150.242	5245.014	-0.68135	0.03049807		1.28113E-16
447.6156	444.0504	495.6827	-0.11262	0.973340554		0.620526274
279.6481	282.4086	254.653	-0.17248	0.363948776		0.553486024
898.8049	920.615	1012.324	0.068428	0.120812072		0.826468162
1476.863	1177.012	1515.343	-0.17187	0.077927263		0.364490432
5220.395	4715.481	5201	0.078666	0.511831737		0.620526274
730.8374	733.8909	690.6023	0.115213	0.747302666		0.566742383
1569.782	1702.813	1832.873	-0.21409	0.813154117		0.387451456
5892.265	5729.922	6024.693	-0.02872	0.211501936		0.848456292
516.4108	525.8003	432.8054	-0.01307	0.91441696		0.974691849
6195.142	6278.018	6645.082	-0.00654	0.406883355		0.967502683
5341.01	5351.829	5730.217	0.277562	0.978804446		0.083378385
7935.572	7756.948	8273.604	0.418415	0.140032454		0.000123311
3033.243	3177.097	2990.863	-0.78218	1.52205E-34		3.02863E-27
168.861	212.7354	127.8505	-0.01887	0.000701205		0.977355784
1639.47	1564.395	1990.066	-0.47862	0.338642963		0.00037975
1103.404	1072.967	870.8505	0.128727	0.083426433		0.593714049
312.7055	306.562	420.2299	-0.32936	0.395100931		0.168886427
4175.958	4248.206	4703.222	-0.16475	0.734144448		0.229480039
1014.953	1031.163	1125.504	-0.22847	0.535948266		0.103471246

353.8039	308.42	334.2976	-0.17653	0.02453229		0.468420234
36524	36490.17	34388.64	-0.03334	0.858353901		0.805419251
1614.454	1477.071	1416.835	-0.30565	0.000678572		0.006064448
5881.544	5499.537	5568.832	0.019876	0.622402254		0.896421455
1306.216	1317.288	1380.157	0.382097	0.134933146		0.000106385
2124.61	2107.846	1794.099	0.159396	0.99759924		0.351487828
52650.67	52564.24	47837.04	-0.11084	0.759261525		0.495076977
743.3456	724.6011	791.2059	0.127016	0.600467264		0.545985077
496.755	592.6865	457.9563	0.156707	0.284902942		0.546682742
1349.101	1430.623	1453.513	0.241333	0.16272914		0.080270076
41897.18	40368.64	41215.02	-0.19403	0.494488338		0.060851524
8856.713	8609.747	8523.017	0.233473	1.10762E-25		0.141040456
58.96732	84.53679	67.06911	-0.05338	0.896968012		0.94960537
351.1236	317.7097	388.7913	-0.34013	0.770051929		0.120090071
150.9921	170.9315	187.5839	-0.45793	0.084787806		0.080567578
7.147554	13.00566	0	0.460338	0.43839941	NA	
60.75421	38.08801	54.49365	0.073795	0.444002938	NA	
4972.911	4960.731	4806.969	-0.31687	0.944992229		0.000195764
153.6724	133.7725	144.6178	-0.17725	0.619821934		0.542805648
3219.973	2960.646	2982.48	-0.01298	0.815150251		0.94960537
3476.392	3507.812	3555.711	0.438702	0.946696226		1.05479E-06
1794.93	1776.202	1603.371	0.205481	0.14394509		0.225879379
915.7804	958.703	886.5698	-0.09219	0.111397413		0.66509742
1995.061	2066.042	2046.656	0.397456	0.595320624		0.000230052
897.018	800.7771	793.3019	-0.05621	0.825633601		0.797601985
1165.051	1151.001	1058.434	0.091114	0.357373649		0.778199039
25.01644	46.44879	40.87024	0.654966	0.828016939		0.254420279
821.0753	854.6577	921.1524	-0.09689	0.011540931		0.626829627
269.8202	350.2239	311.2426	-0.02774	0.469192599		0.920188536
10176.33	10026.44	10348.55	0.002121	0.045826503		0.990142758
1461.675	1475.214	1563.549	-0.13108	0.988569159		0.493837083
3166.366	3152.944	3350.312	-0.40736	0.279564109		7.82735E-06
716.5423	717.1693	792.2539	-0.00874	0.504924333		0.975879427
570.0174	495.1441	465.292	0.191547	0.92288341		0.471932964
5660.863	5658.391	6304.497	-0.02588	0.620659412		0.913119202
2842.046	3073.981	3153.296	0.061749	0.49990935		0.751029502
1045.33	967.0638	990.3174	0.317872	0.265502165		0.015466264
41.09844	26.01132	26.19887	-0.14637	0.857865109	NA	
3692.605	3729.838	3696.137	-0.13496	0.036478363		0.160483163
1593.011	1646.145	1575.076	0.131181	0.798439173		0.361564535
73.26243	50.16469	69.16502	-0.40868	0.025915262		0.315833346
2635.661	2398.615	2465.838	-0.18375	1.02131E-12		0.13467359
105.4264	124.4828	124.7066	0.102711	0.37757303		0.847133587
7091.267	7011.909	8119.555	0.857992	0.948234757		6.13477E-15
130.4429	129.1276	96.41185	-0.01126	0.507065266		0.99232704
1269.584	1197.45	1145.415	0.219652	0.482263779		0.120651904
1131.1	1085.973	1103.497	0.02263	0.88874568		0.918453677
33136.95	31540.59	32137.63	0.289791	0.652050398		4.19262E-05
1863.725	1681.446	1584.508	0.186623	0.6333028		0.361564535
4500.279	4556.626	5598.175	-0.44444	0.298839192		0.008018267
1494.732	1484.503	1641.097	-0.56269	0.572336545		1.79298E-05
53821.08	50598.52	46258.82	0.12109	0.063499532		0.558104763
1286.56	1380.458	1630.618	-0.5013	0.559171932		5.12674E-05
1229.379	1303.353	1094.065	0.074913	0.361706648		0.758793804

105261.1	102195.7	105232.5	-0.14006	0.935100162	0.423533881
358.2711	379.9511	470.5317	-0.1029	0.861070915	0.718323278
5356.198	5589.647	5150.698	0.181799	0.936346196	0.082377833
2135.332	2099.485	2278.254	0.153156	0.221789876	0.333065318
1326.765	1111.055	1191.525	0.091255	0.769710962	0.666213081
546.7879	516.5105	603.622	0.649588	0.187385025	0.000192079
484.2468	523.0134	588.9507	0.409859	0.867574132	0.098823212
14993.78	14249.56	15814.69	-0.15769	0.654926306	0.361564535
641.493	625.2007	892.8576	-0.16489	0.965403682	0.570860906
6076.314	5946.374	5951.336	0.038942	0.670951846	0.778166558
18.76233	23.22439	24.10296	-0.13355	0.1202463	NA
176.902	234.1019	233.6939	0.166992	0.837998472	0.596499376
1318.724	1300.566	1389.588	0.041683	0.638519612	0.87057216
1090.895	1071.109	1172.662	0.067645	0.545771146	0.681228083
0	1.857952	0	#DIV/0!	0.836333106	NA
500.3288	526.7293	594.1904	-0.35308	0.436758255	0.038329459
55.39354	56.66752	74.4048	-0.07626	0.116786217	0.887223334
904.1656	902.0355	725.1848	0.143841	0.728166455	0.522228539
170.6479	92.89758	128.8985	0.304003	0.663061022	0.437636747
4337.672	3923.065	4213.827	-0.40519	0.003867233	0.000226461
8825.442	9018.497	9944.044	-0.07143	0.875368742	0.724404377
952.4116	921.544	911.7208	0.041781	0.003450987	0.846177331
1034.608	999.5779	938.9676	0.105566	0.049083937	0.593884478
7001.029	7284.099	9812.002	-0.48534	0.081224331	0.001954689
246.5906	260.1132	238.9337	-0.14307	0.759261525	0.72969638
5944.085	5881.346	5802.526	0.106601	0.97659978	0.401324593
114.3609	124.4828	97.45981	-0.32963	0.572336545	0.290794331
1046.223	1085.044	1030.14	0.102212	0.809173332	0.629323036
0	0.928976	0	-2.08851	0.998398411	NA
2579.374	2545.394	3460.347	-0.39014	0.386644751	0.024308063
3443.334	3113.927	2778.128	0.13103	0.617539326	0.476261394
1401.814	1273.626	1395.876	-0.06018	0.652050398	0.728787361
4932.706	4840.893	4580.611	0.199543	0.910983119	0.036487394
45.56566	33.44313	39.82229	-0.52849	0.188588777	0.266809788
396.6893	346.508	469.4838	0.131932	0.986125936	0.601858923
44967.94	44482.15	49865.89	0.349221	0.153154897	0.00185873
1015.846	986.5723	1085.681	0.067663	0.008324314	0.763161717
595.9273	693.9449	599.4302	0.050502	0.872688807	0.834319281
2180.897	2105.988	1920.901	0.249612	0.257821803	0.26890857
954.1985	1011.655	994.5092	0.291016	0.757469981	0.031567205
8243.81	8437.887	8117.459	-0.22168	0.182512116	0.063993489
742.4522	864.8764	834.1721	-0.0151	0.60948547	0.94960537
737.985	645.6382	763.9591	-0.12811	0.358629856	0.49776547
173.3282	152.352	154.0494	0.363351	0.784844775	0.48379367
209.066	209.0195	254.653	0.054197	0.105557831	0.903239699
1387.519	1496.58	1609.659	-0.09536	0.785492922	0.560116425
679.9111	570.3911	582.6629	0.195628	0.484084014	0.285602122
1870.872	1847.733	1808.77	0.125183	0.084784548	0.359873864
3071.661	2919.771	3074.7	-0.05246	0.425091186	0.762128192
10917	10092.39	11828.27	-0.14681	0.056527354	0.316669252
276.9677	313.9938	332.2017	0.066686	0.020880197	0.81253834
1.786889	3.715903	2.09591	0.528496	0.999122571	NA
525.3452	536.019	569.0395	-0.05944	0.928405436	0.824214258
2115.676	2173.803	2243.671	0.101436	0.660473408	0.575540189

68.79521	78.03396	76.50071	-0.40856	0.58619972	0.268588059
10409.52	10457.48	9945.092	0.034875	0.935960933	0.818645356
811.2474	757.1152	759.7673	0.171447	0.472470204	0.336190405
91.13131	110.5481	104.7955	-0.17923	0.006262634	0.723898526
594.1404	656.7859	617.2454	0.084287	0.764395772	0.723437511
633.452	598.2604	787.0141	-0.20342	0.682713002	0.321405678
591.4601	665.1466	601.5261	-0.16241	0.562820931	0.526905314
582.5257	537.877	457.9563	0.150051	0.434274199	0.604472877
612.0093	588.0417	540.7447	-0.26897	0.036478363	0.198436131
1391.986	1442.699	1492.288	0.021009	0.856505841	0.921973417
726.3702	695.8028	753.4796	-0.2229	0.048314421	0.164747869
622.7306	555.5275	664.4034	0.083765	0.398196147	0.813492611
191.1971	119.8379	144.6178	0.526686	0.767300341	0.322483845
11500.41	11340.01	12431.89	-0.1351	0.918350023	0.274204666
12336.68	12137.07	13082.67	0.080063	0.681805164	0.631303895
1618.028	1518.875	1701.879	-0.58718	0.019681562	2.89763E-07
401.1565	439.4055	428.6136	-0.25718	0.967813477	0.23616229
1433.978	1471.498	1343.478	-0.12453	0.388896402	0.344591494
11222.55	11364.16	12050.43	0.089156	0.335983122	0.656368654
867.5344	1013.513	816.3569	0.199263	0.038380374	0.317944832
2067.43	2277.849	2348.467	-0.07473	0.179898822	0.765789524
8.934443	4.644879	1.047955	0.699022	0.478079371	NA
3087.743	3256.989	3472.923	-0.4967	0.020685631	1.44405E-05
173.3282	233.1729	208.543	-0.25166	0.014990769	0.534193701
307.3448	358.5846	398.2229	-0.28142	0.026312287	0.209997967
506.5829	458.914	461.1002	-0.12721	0.037913264	0.612005134
3878.442	3847.818	3735.959	-0.05918	0.166321504	0.739592868
1698.438	1774.344	1806.674	0.080897	0.981637588	0.710256666
578.0584	548.0957	599.4302	-0.06199	0.368034878	0.785131021
814.8212	713.4534	796.4457	-0.22705	0.324651337	0.172785623
703.1406	691.158	733.5684	0.431826	0.990604058	0.069341932
493.1812	482.1384	597.3343	-0.21765	3.64832E-13	0.377059311
2884.038	3203.108	3542.088	-0.47547	0.304135256	0.000891501
1124.846	1167.723	1154.846	0.117965	0.859974619	0.551771495
715.6489	730.1749	682.2186	-0.03649	0.779325825	0.876563898
752.2801	907.6093	908.5769	-0.14336	0.739082502	0.583053599
1232.953	1224.39	1186.285	0.193274	0.837529432	0.23618111
2599.923	2623.428	2690.1	0.171135	0.128775373	0.177628135
142.0576	143.0623	126.8025	0.483655	0.498379067	0.167165088
4846.042	4656.955	4709.509	0.092119	0.795376015	0.463488413
582.5257	594.5445	539.6968	0.035376	0.025100811	0.921848549
71.47554	52.02264	79.64457	-0.4244	0.853477462	0.420106203
271.6071	212.7354	238.9337	0.081685	0.674403807	0.776926877
406.5171	434.7607	394.031	-0.30018	0.948234757	0.236644531
1324.084	1324.719	1367.581	-0.32463	0.922937786	0.013575411
50.03288	92.89758	62.87729	0.426277	0.88874568	NA
966.7067	999.5779	966.2144	-0.12296	0.934554042	0.602858252
948.8378	1063.677	1035.379	0.027099	0.865150494	0.91372954
1115.018	1189.089	920.1044	0.171171	0.840870655	0.470895778
1591.224	1321.004	1345.574	-0.17054	0.000791163	0.398655382
577.165	576.8939	525.0254	0.242284	0.720295497	0.353465216
154.5659	201.5877	229.5021	-0.35146	0.735300868	0.17742588
20.54922	32.51415	22.00705	0.084228	0.780980494	NA
3960.638	4021.536	3979.085	0.119318	0.914186189	0.29075107

4224.204	4213.834	4463.24	-0.26144	0.651759792	0.013833369
1017.633	1160.291	1045.859	0.186656	0.134073214	0.251774974
92.9182	50.16469	68.11707	0.328506	0.567589507	0.477876695
58.96732	96.61348	72.30889	-0.20514	0.883354954	0.682058194
8802.213	8021.706	8753.567	-0.20642	0.243530103	0.184072788
113.4674	139.3464	165.5769	-0.76973	0.417038094	0.005731554
2469.48	2462.715	2660.757	-0.47717	0.302288037	2.9108E-06
1461.675	1373.026	1388.54	0.177299	0.563625743	0.193734178
45.56566	46.44879	64.9732	-0.2869	0.863679003	0.55088846
551.2551	520.2264	533.409	0.053076	0.658199476	0.822050288
8015.088	8191.708	9133.975	-0.14792	0.965666941	0.382141979
5196.272	5196.69	4915.956	0.084174	0.811082292	0.511081603
1154.33	1107.339	1032.236	0.008647	0.052819596	0.9717805
5507.19	5519.045	5518.53	0.03874	0.639085319	0.818645356
776.4031	806.351	732.5205	0.060181	0.072199218	0.782219628
7665.752	7823.834	8321.81	-0.08427	0.0779973	0.686015779
607.5421	771.9789	728.3287	-0.15051	0.722921305	0.474497273
1.786889	5.573855	2.09591	1.844073	0.196002323 NA	
69.68865	57.5965	62.87729	-0.02728	0.215234152	0.961026715
82.19687	101.2584	120.5148	-0.36095	0.817609984	0.251743462
762.108	826.7884	1072.058	0.113291	0.639638255	0.712929046
4380.557	4199.899	4322.814	0.208776	0.798409569	0.028545761
8247.384	8024.493	8982.021	-0.17248	0.847232313	0.342128545
670.9766	644.7092	668.5952	0.031689	0.6333028	0.880061352
8.934443	7.431806	2.09591	-0.50741	0.888901047 NA	
1992.381	1816.148	1553.069	-0.31844	1.59339E-19	0.037131359
628.9848	687.4421	697.938	0.261131	0.185152106	0.250830779
8756.647	8606.96	8785.006	-0.07431	0.846655864	0.646288352
136.697	173.7185	177.1044	0.368865	0.495326515	0.348855854
452.0828	472.8487	571.1354	-0.76621	0.338642963	2.9108E-06
771.9358	819.3566	732.5205	-0.16451	0.901132408	0.333065318
9299.861	9783.044	9259.729	0.028298	0.211120083	0.832900164
13391.84	13317.8	13713.54	-0.05934	0.009017011	0.579998211
2753.595	2590.913	2779.176	0.049394	0.631766123	0.814086627
1301.748	1310.785	1476.568	-0.10023	0.668494236	0.583053599
1374.117	1498.438	1689.303	0.182057	0.505612976	0.34519405
201.025	176.5054	160.3371	0.498858	0.004702869	0.049157122
338.6154	356.7267	455.8604	-0.36931	0.408721528	0.079283616
168.861	164.4287	192.8237	-0.65908	0.51752002	0.013398862
2423.914	2407.905	2636.655	0.344221	0.600467264	0.004337582
4366.262	4381.05	5076.294	-0.41352	0.545771146	0.005408206
1624.282	1635.926	1713.406	0.056699	0.958214194	0.724404377
754.067	707.8795	726.2327	0.088354	0.183178382	0.748961137
1341.953	1476.142	1534.206	-0.00331	0.446999299	0.996720771
1114.125	1083.186	1072.058	-0.0365	0.002064668	0.872630949
767.4686	731.1039	695.842	-0.21388	0.88849017	0.180455525
567.3371	526.7293	741.9521	-0.71663	0.994127305	3.79149E-05
37124.4	37339.25	43754.21	-0.16523	0.847380389	0.266001442
297.5169	324.2125	292.3794	-0.06484	0.05061615	0.902408045
147.4183	180.2213	161.3851	-0.05832	0.159614653	0.8721296
305.5579	349.2949	291.3315	0.140446	0.951538779	0.638102356
461.0172	427.3289	399.2708	-0.37517	0.804360442	0.037530261
2411.406	2352.167	2285.59	0.007511	0.748299599	0.970425422
1630.536	1501.225	1535.254	0.075861	0.845528608	0.695745646

3050.219	2885.399	2910.171	0.482522	0.300372064	2.9108E-06
645.0668	594.5445	657.0677	0.31024	0.248671635	0.126956931
771.0424	855.5867	834.1721	-0.08798	0.536580245	0.744396459
6137.962	6303.101	6716.343	-0.26571	0.262179603	0.012142769
386.8614	496.0731	430.7095	-0.12785	0.020898839	0.664227919
322.5334	323.2836	424.4217	0.074726	0.324105472	0.844024501
965.8132	915.0411	909.6248	-0.42007	0.379871328	6.20365E-05
1060.518	1103.623	933.7278	0.229947	0.804687981	0.186593864
1912.864	1986.15	2022.553	0.209435	0.153105196	0.163756864
1079.281	1037.666	1054.243	0.12866	0.00628885	0.559214222
1380.371	1383.245	1349.766	0.469086	0.563223905	0.007395438
3985.655	4056.837	3660.506	0.079466	0.055914431	0.63553201
809.4605	680.0103	833.1241	-0.06276	0.441155859	0.790835118
648.6405	531.3741	576.3752	-0.1981	0.373435737	0.378650149
3124.375	2861.245	2636.655	0.13476	0.082467547	0.583053599
132.2298	155.139	136.2341	0.568892	0.837529432	0.232685912
913.1	879.74	954.6869	-0.4798	0.596065856	0.000164663
1155.223	1142.64	1137.031	0.217386	0.049693405	0.117363139
417.2385	335.3602	455.8604	0.236143	0.469895855	0.316260034
2275.603	2394.9	2537.099	0.027407	0.170982117	0.875604624
1107.871	1088.76	968.3103	0.121555	0.50957694	0.685537269
170.6479	199.7298	200.1594	-0.83277	0.054140788	2.54322E-07
1082.854	1055.316	1071.01	0.198665	0.119144516	0.33950184
1567.995	1547.674	1406.355	0.119921	0.832298202	0.56930697
14.29511	10.21873	11.5275	-0.37642	0.811082292 NA	
48.24599	63.17035	73.35684	0.443288	0.200584155	0.356994802
10781.19	10482.56	10632.55	-0.40855	7.8206E-06	3.40063E-05
656.6815	602.9053	629.8209	-0.01513	0.732140966	0.937994709
2104.061	2062.326	1858.024	-0.03829	0.686019125	0.896421455
191.1971	259.1842	211.6869	-0.31377	7.06707E-06	0.23616229
479.7796	482.1384	654.9718	0.154382	0.26155741	0.652302842
16414.36	16246.86	14866.29	0.035616	0.426866268	0.841212475
1811.905	1917.406	2111.629	-0.04786	0.173085065	0.820066816
363.6318	384.596	379.3597	0.198872	0.550217758	0.381727979
2740.194	2867.748	2727.827	-0.03243	0.730204074	0.836321366
4322.483	4180.391	4556.508	0.122902	0.670044046	0.293133991
3640.785	3516.173	3766.35	-0.01267	0.005009159	0.956290296
12.50822	28.79825	28.29478	-0.28908	0.205662453	0.710256666
619.1569	736.6778	638.2045	0.173012	0.10338322	0.427953678
54.5001	51.09367	76.50071	0.162915	0.98409529 NA	
4731.681	4746.137	4512.494	0.106873	0.680817293	0.447245603
1571.568	1563.466	1608.611	-0.0206	0.776861758	0.92306642
4215.27	4396.842	4146.758	-0.05568	0.642246351	0.775439768
1530.47	1454.776	1287.937	-0.02253	0.181584456	0.921973417
109.0002	78.96294	121.5628	-0.55252	0.810961861	0.050017331
3761.4	3828.309	3689.849	0.009424	0.837529432	0.951184679
19.65577	11.14771	19.91114	-0.10628	0.835449534	0.941511899
1241.888	1105.481	1066.818	-0.03476	0.735300868	0.897900971
4858.55	4800.018	4358.444	0.049019	0.935506037	0.756795912
126.8691	133.7725	140.426	-0.08811	0.98843748	0.861336132
1924.479	2063.255	1918.805	-0.108	0.017639055	0.383690121
536.0666	608.4791	549.1284	-0.06009	0.918350023	0.822050288
9464.255	9152.269	8745.184	0.114492	0.488813245	0.245528705
64.32799	37.15903	48.20593	0.147918	0.614621507 NA	

1073.027	1085.044	945.2553	0.133633	0.002387022		0.423533881
48.24599	40.87493	35.63047	-0.32072	0.07224611		0.563375937
9394.566	9518.286	9333.086	-0.07515	0.544035793		0.55088846
2071.004	2000.085	2306.549	-0.14903	0.002866239		0.300201673
3680.097	3638.798	3924.591	-0.25446	0.362358207		0.065190198
314.4924	292.6274	455.8604	-0.06411	0.001214989		0.918453677
1813.692	1741.83	1641.097	0.244398	0.910998143		0.109137955
1673.421	1631.281	1630.618	0.05898	0.528265463		0.713405771
3800.712	3747.488	3829.227	0.037584	0.999998226		0.818870327
1328.552	1252.259	1498.575	0.866632	0.470002734		6.76595E-09
1544.765	1635.926	1309.944	0.124366	0.98062117		0.483238043
3928.474	3857.107	3776.829	-0.1698	0.986125936		0.26142392
229.6152	262.9001	359.4485	0.193225	0.053184762		0.681439344
830.0097	746.8965	1086.729	-0.69744	0.648891486		5.71201E-05
1399.134	1376.742	1392.732	-0.04187	0.159614653		0.821688803
307.3448	302.8461	333.2497	0.180467	0.49990935		0.502183544
1046.223	1159.362	995.5571	0.199179	0.469683278		0.367817075
6025.388	5316.528	5938.76	0.536443	0.035710242		0.000232021
1322.298	1184.444	1029.092	0.244928	0.812875146		0.266907481
1527.79	1746.474	1712.358	-0.05287	0.02597477		0.797601985
1017.633	1126.848	1049.003	0.065725	0.502046091		0.764996976
1047.117	1062.748	1083.585	0.121828	0.967693355		0.519070487
2669.611	2652.226	2290.829	0.028906	0.603004299		0.889747395
300.1973	237.8178	343.7292	-0.48369	0.844410461		0.013137961
4.467221	3.715903	7.335684	0.325647	0.874036182	NA	
744.2391	743.1806	617.2454	-0.21652	0.00873722		0.301238876
6863.439	7191.201	7361.883	-0.02737	0.984233953		0.902408045
1327.658	1272.697	1505.911	-0.51498	0.740595411		0.000144734
722.7964	676.2944	687.4584	0.474591	0.002387022		0.000458578
1878.913	1916.477	1592.891	0.084409	0.761559926		0.638053826
3069.874	2995.018	2407.152	0.105979	0.115119266		0.675143077
2892.973	2699.604	2920.65	-0.09486	0.001195876		0.542805648
190.3036	186.7241	250.4612	0.086984	0.87547961		0.838059844
4058.024	4089.351	4416.082	0.16891	0.925203985		0.131902958
13.40166	15.79259	6.287729	0.863748	0.804360442	NA	
1252.609	1180.728	1336.142	0.029015	0.998398411		0.923725803
650.4274	716.2403	648.6841	0.143122	0.907472172		0.523627087
473.5255	432.9027	582.6629	-0.1509	0.536343685		0.536590083
1131.1	1006.081	816.3569	0.177125	0.985859213		0.497389249
870.2147	900.1775	913.8167	-0.39038	0.008048268		0.002309437
683.4849	672.5785	803.7814	-0.26121	0.074625654		0.319527914
25210.32	24831.52	25172.92	-0.04084	0.394915233		0.807881031
33.95088	16.72156	38.77433	0.01672	0.900789434		0.986414682
20216.86	19174.99	20309.37	0.042922	0.743382516		0.786995178
397.5827	383.667	432.8054	0.127319	0.991136341		0.566180254
495.8616	501.6469	542.8406	-0.58744	0.00112543		1.62112E-05
53.60666	77.10499	78.59662	-0.52783	0.759069408		0.168910143
123.2953	115.193	105.8434	-0.09127	0.504054705		0.818055883
4823.706	4610.507	4680.167	0.319098	0.936832996		0.090635263
284.1153	236.8888	289.2356	-0.22049	0.156929518		0.427496625
17.86889	19.50849	26.19887	0.208817	0.98843748		0.81253834
1117.699	1079.47	976.694	0.147558	0.825797061		0.465657685
472.632	464.4879	462.1481	-0.08718	0.108117004		0.773113821
1585.864	1608.057	1630.618	-0.235	0.284203413		0.169935181

212.6397	216.4514	276.6601	0.464073	0.846759683	0.190817982
407.4106	393.8857	421.2779	0.04566	0.842190796	0.880639773
701.3537	797.0612	773.3907	0.089856	0.434940845	0.680886616
8243.81	7957.606	7939.306	0.10707	0.298839192	0.493206792
1294.601	1307.069	1216.676	-0.07115	0.74446621	0.756795912
5199.846	5382.486	5292.172	-0.07973	0.620421746	0.499176113
137.5904	113.335	167.6728	-0.02617	0.811082292	0.941511899
3501.408	3583.06	4085.976	-0.01831	0.390909657	0.935612856
90.23787	81.74987	75.45275	0.036726	0.303800805	0.923725803
0	2.786927	0	-1.46492	0.509223684	NA
322.5334	261.0422	276.6601	-0.37117	0.621093622	0.092351218
556.6158	545.3088	506.1622	-0.16763	0.713210135	0.368584102
4225.098	4088.422	3601.821	0.176176	0.72031406	0.338698753
2694.628	2977.367	2804.327	0.020226	0.827268091	0.912679298
1291.027	1376.742	1360.245	-0.47417	0.023293079	7.44306E-05
4099.122	4287.223	4547.076	0.059606	0.328476882	0.703889775
333.2547	367.8744	397.1749	-0.12095	0.157741898	0.681950827
96.49198	81.74987	83.83639	0.185482	0.603004299	0.708596546
955.9854	833.2913	955.7349	0.231394	0.956452433	0.163200347
1358.929	1160.291	1339.286	-1.18668	7.0339E-18	2.98532E-19
235.8693	300.9881	233.6939	0.135517	0.970011305	0.656489824
5.360666	5.573855	11.5275	-0.52399	NA	NA
219.7873	256.3973	265.1326	-0.16517	0.795057685	0.684679019
1802.971	1591.335	1728.078	-0.33848	0.003481985	0.00133704
1740.429	1533.739	1665.2	0.28322	0.431874361	0.017912978
8.934443	4.644879	6.287729	0.455617	0.769573539	NA
1413.429	1285.702	1119.216	0.116075	0.295905234	0.588622174
620.9438	562.9593	536.5529	0.223068	0.581539609	0.31017583
1916.438	1896.04	1725.982	0.120407	0.995598118	0.437989627
2559.718	2681.024	2292.925	0.337007	0.059513177	0.051438409
265.3529	248.9655	212.7348	0.076308	0.897631797	0.832900164
239.4431	284.2666	237.8858	-0.05964	0.056337167	0.878767667
1894.102	1971.287	1818.202	0.050519	0.497122222	0.811374268
4122.352	3976.945	3648.979	0.051128	0.564427849	0.744172227
583.4191	568.5332	714.7052	-0.63701	0.924284267	0.000876631
6066.487	5610.085	5739.649	0.190991	0.517228307	0.24662663
133.1232	173.7185	134.1382	0.195677	0.986125936	0.690294956
1164.158	1196.521	1195.717	-0.20755	0.847983366	0.155780712
178.6889	154.21	193.8717	-0.09585	0.572336545	0.82871401
2196.979	2272.275	2595.784	-0.02421	0.387684972	0.897989514
8.040998	22.29542	13.62341	0.794522	0.563625743	NA
537.8534	431.9737	503.0183	-0.00347	0.542904501	0.996992829
121.5084	116.122	130.9944	-0.0738	0.324105472	0.897989514
385.9679	326.9995	338.4894	-0.10486	0.352221493	0.692421497
1983.446	2058.61	2094.862	0.160163	0.015489924	0.167499635
770.149	676.2944	901.2412	-0.62698	0.875720952	3.79149E-05
51.81977	64.09933	45.06206	0.13622	0.827268091	0.825561751
2557.931	2373.533	2566.442	0.272584	0.484625827	0.008127598
179.5823	156.9969	132.0423	-0.42298	0.256151839	0.245748269
187.6233	163.4997	162.433	-0.21567	0.740079901	0.543394748
1048.904	1041.382	1283.745	-0.27555	0.910682739	0.10904213
779.9768	743.1806	1067.866	-0.43669	0.458486853	0.016996857
432.427	541.5929	530.2652	-0.18442	0.599793836	0.402824474
2957.3	2908.623	2427.064	0.134766	0.242725862	0.622128808

1802.077	1752.977	1871.647	0.017668	0.765366348	0.922101817
1593.011	1653.577	1744.845	0.02576	0.014847555	0.904874311
3003.76	2992.231	3435.196	-0.33031	0.613451408	0.018761765
13693.82	13811.08	15188.01	-0.52539	0.000495416	3.36128E-07
30305.63	31098.39	31949	0.126294	0.121695117	0.326965278
3817.687	3986.235	4037.77	-0.12978	0.720895932	0.437989627
7534.415	7627.82	8329.145	-0.27852	0.735300868	0.059797997
2010.25	1939.701	1767.9	0.085998	0.493325129	0.63864783
700.4603	791.4873	653.9239	0.197172	0.730407446	0.357441003
59.86077	48.30674	64.9732	-0.25642	0.043196135	0.630318178
1591.224	1700.955	1913.566	-0.14504	0.915168823	0.52313639
35.73777	39.01698	52.39774	-0.59221	0.914186189	NA
56.28699	25.08235	66.02116	-0.04962	0.794232621	NA
586.0994	667.9336	668.5952	-0.04981	0.817609984	0.825078673
2207.701	2331.729	2182.89	0.04797	0.836333106	0.780791262
22.33611	34.3721	35.63047	0.25254	0.804360442	0.715956488
2236.291	2488.726	2586.353	-0.1864	0.648845683	0.241905667
1264.224	1359.092	1343.478	0.020616	0.369977802	0.918453677
1064.986	1011.655	1162.182	-0.75929	0.000820039	7.91344E-11
83.09032	109.6191	129.9464	-0.33425	0.654632751	0.365745492
856.813	756.1863	861.4189	0.119503	0.427171669	0.552800466
2103.168	2234.187	2109.533	0.07832	0.840099446	0.667557921
6419.397	6714.637	7200.498	-0.29317	0.960956671	0.049713965
30.3771	23.22439	15.71932	-0.21018	0.835664498	0.796040638
1778.848	1659.151	1869.552	0.039038	0.089121906	0.884584155
488.714	483.9964	525.0254	-0.17514	0.411467295	0.411708366
3778.376	3580.273	3489.69	-0.12934	0.175067813	0.521450212
5223.969	5881.346	5373.913	-0.22431	0.00602068	0.10377158
1119.486	1105.481	976.694	0.170666	0.923281561	0.321291302
1263.33	1102.694	959.9267	0.1954	0.907095813	0.489096476
1680.569	1653.577	1496.48	0.068457	0.813154117	0.693887308
246.5906	232.2439	337.4415	-0.6256	2.0377E-15	0.042769614
8308.138	8806.69	8491.579	-0.02842	0.985859213	0.863611229
383.2876	433.8317	399.2708	-0.03453	0.777280242	0.901028548
2428.381	2450.638	2882.924	-0.32212	0.183178382	0.025667068
4986.312	4826.029	4135.23	0.123165	0.785971011	0.631325703
90.23787	65.0283	63.92525	-0.07363	0.992511525	0.924895748
11969.47	12212.32	12146.85	-0.19758	0.938838346	0.088369497
1237.42	1191.876	1218.772	0.055471	0.019250807	0.791888862
2429.275	2368.888	2397.721	-0.07863	4.662E-05	0.596995764
305.5579	295.4143	245.2214	-0.39785	0.003603481	0.098823212
2194.299	2075.332	2178.698	-0.00865	0.759069408	0.965253376
3230.694	3286.716	3662.602	0.095806	0.248671635	0.575540189
3437.08	3263.492	3160.632	0.25696	0.270092699	0.006992399
327.894	305.633	362.5924	-0.09236	0.944990108	0.727738403
1012.272	988.4302	997.6531	-0.07674	1.41548E-17	0.673302041
452.9762	415.2522	441.189	-0.0873	0.206816173	0.727797788
3.573777	2.786927	2.09591	2.682823	0.108299464	NA
1766.339	1839.372	1862.216	-0.11594	0.434274199	0.510881602
218.0004	300.9881	299.7151	0.328722	0.39778623	0.269755378
1971.831	1969.429	1779.427	0.22344	0.263755993	0.231047495
1868.192	1922.051	1795.147	-0.07912	0.000647509	0.603217016
432.427	462.6299	414.9901	0.16319	0.852550008	0.462481429
619.1569	706.0216	604.67	0.024314	0.828016939	0.919915102

1635.896	1656.364	1667.296	-0.03099	0.819879878	0.877477421
536.0666	544.3798	441.189	0.398556	0.716063981	0.258108467
3230.694	3112.069	3856.474	-0.457	0.827268091	0.000143823
436.8942	490.4992	576.3752	-0.02958	0.652135039	0.915888123
995.2969	811.9248	930.5839	0.457451	0.707092679	0.008625676
139.3773	126.3407	106.8914	-0.09613	0.108117004	0.844024501
6741.93	6944.094	7432.096	-0.16241	0.864963799	0.306279885
194.7708	229.457	159.2891	-0.12888	0.004702869	0.778166558
1813.692	1731.611	1504.863	-0.04221	0.929188034	0.891241787
1480.437	1566.253	1632.714	-0.06902	0.881898117	0.741928459
5822.576	5752.218	5532.154	-0.18122	0.18266723	0.117033962
1295.494	1280.129	1554.117	0.077698	0.587719544	0.699804545
341.2957	338.1472	380.4076	-0.32498	0.004988115	0.167597558
1689.503	1607.128	1816.106	0.013237	0.007236622	0.9611653
22330.75	21596.83	19299.14	0.173279	0.043426903	0.198756229
3477.285	3642.514	3626.972	-0.10607	0.971689046	0.443898537
222.4676	237.8178	221.1185	-0.30349	0.46334755	0.20379563
1098.936	1004.223	914.8646	0.326692	0.826718993	0.057117557
2240.758	2583.482	2357.899	-0.08378	0.907472172	0.710256666
314.4924	256.3973	249.4133	-0.18838	0.941098554	0.497130933
560.1895	456.1271	477.8674	0.20832	0.975408215	0.545985077
2740.194	2829.66	3279.051	-0.125	0.940969382	0.431825344
1530.47	1684.233	1796.195	-0.0834	0.942498782	0.616511903
11098.36	10882.02	10058.27	-0.48777	0.004283832	2.54396E-05
1027.461	1004.223	990.3174	0.226016	0.795376015	0.158759962
1161.478	1078.541	1087.777	-0.00224	0.756107114	0.99985654
1071.24	1029.305	944.2074	0.16553	0.194542134	0.410453641
355.5908	333.5023	301.811	-0.01685	0.814880408	0.974018993
858.5999	1000.507	996.6051	-0.06786	0.230523928	0.789143179
92.9182	68.74421	80.69253	0.149418	0.816044012	0.745567347
739.7718	744.1096	811.1171	-0.15077	0.000107991	0.380264685
2111.209	2172.874	2084.382	0.049768	0.392876266	0.767705112
2000.422	2109.704	1935.573	0.096079	0.98062117	0.552800466
12.50822	10.21873	13.62341	-0.58925	0.817609984 NA	
432.427	397.6016	515.5938	-0.14805	0.996328617	0.577160388
190.3036	182.0792	146.7137	0.061612	0.58195463	0.888191125
342.1892	401.3175	403.4626	-0.08355	0.948234757	0.742276559
918.4607	838.8651	817.4048	0.16067	0.707368241	0.419308591
73.26243	45.51981	33.53456	-0.40774	0.008810437 NA	
197.4512	231.315	202.2553	0.145369	0.923471886	0.612005134
100.9592	97.54245	111.0832	0.158084	0.988049891	0.763234817
1051.584	966.1348	1033.284	0.40827	0.231538797	0.003281665
1638.577	1438.983	1756.372	0.45766	0.713312904	0.000794237
69.68865	97.54245	96.41185	-0.05999	0.69597302	0.914491385
1147.182	1069.251	1122.36	-0.02246	0.068521623	0.919258281
29941.1	30719.37	31574.88	-0.06068	0.193381894	0.697184019
54.5001	71.53113	50.30183	-0.42754	0.216003389	0.282321884
189.4102	184.8662	177.1044	0.437155	0.44116367	0.125404151
2492.709	2481.294	2590.544	-0.24522	0.122047295	0.045662236
399.3696	314.9228	401.3667	-0.12108	0.411467295	0.751339004
10736.52	11033.45	10840.05	-0.01474	0.868137167	0.915543523
1719.88	1616.418	1329.855	0.024747	0.172366864	0.941096765
15702.28	15331.82	16750.51	-0.24915	0.452120082	0.097010642
213.5332	212.7354	258.8449	0.180932	0.609236349	0.61918051

370.7794	377.1642	385.6474	0.062516	0.240910061	0.843790109
488.714	349.2949	339.5374	0.037979	0.988049891	0.914120833
1064.092	1046.027	1169.518	-0.02111	0.73037883	0.915543523
334.1482	376.2352	384.5994	0.243827	0.268149097	0.345371472
2604.39	2493.371	2844.15	0.28481	0.990604058	0.142827251
6046.831	5774.513	5710.306	0.518567	0.034815958	1.51115E-08
122.4019	104.0453	157.1932	0.228609	0.78204194	0.610358054
584.3125	667.0046	747.1918	-0.18483	0.960823113	0.383759687
1409.855	1330.293	1154.846	0.223943	0.223096937	0.341785585
1643.044	1552.318	1822.394	-0.06507	0.368085564	0.786995178
435.1074	485.8543	493.5868	-0.18762	0.803137847	0.277184815
3479.072	3444.642	3397.47	0.111796	0.830540147	0.414781698
952.4116	947.5553	866.6587	-0.12866	0.71440019	0.505493145
494.9681	476.5646	601.5261	-0.58278	0.707092679	0.001227995
2482.882	2451.567	2322.268	0.022766	0.81966368	0.893536629
950.6247	961.4899	1095.113	-0.06307	0.967813477	0.764996976
58.07388	59.45445	64.9732	-0.4845	0.636895882	0.249412887
114.3609	66.88625	71.26093	-0.10605	0.739579196	0.891241787
1216.871	1288.489	1229.251	-0.10502	0.669860929	0.437989627
55.39354	46.44879	47.15797	-0.92465	0.000902992	0.024245585
553.042	527.6582	613.0536	-0.26008	0.773823303	0.224439117
200.1315	226.6701	159.2891	0.310564	0.759261525	0.443898537
5159.641	5222.702	5006.081	-0.15132	0.043795381	0.146525829
4126.819	4237.987	4034.626	-0.44237	0.248671635	4.37599E-06
12966.56	13358.67	12647.77	0.091827	0.408488796	0.376402197
7814.063	7864.709	8680.21	0.254387	0.812613014	0.011498772
2602.603	2731.189	2745.642	-0.10866	6.59346E-21	0.37426169
4088.401	4088.422	4091.216	0.138298	0.594096059	0.422116695
177.7954	175.5764	221.1185	-0.40933	0.756571209	0.220334464
8017.769	7742.084	7254.992	0.002416	3.79424E-06	0.990142758
18074.38	18256.23	15286.52	0.348122	0.994015824	0.00675258
1192.748	1214.171	1206.196	-0.19621	0.92540314	0.167165088
2929.604	2742.336	2977.24	0.217331	0.867574132	0.035240969
369.8859	383.667	368.8801	0.115602	0.969843993	0.656258611
1099.83	1092.475	1093.017	-0.10286	0.941323092	0.639253997
670.9766	622.4138	702.1298	0.278588	0.462806034	0.169032618
5249.878	5160.46	5086.773	0.178367	0.862474177	0.0410944
3268.219	3002.45	2909.123	0.262217	0.387628548	0.090835789
39.31155	32.51415	48.20593	-0.03689	0.91441696 NA	
1735.069	1827.295	2063.423	-0.31077	0.914186189	0.055894075
43585.78	45095.27	47477.6	-0.00707	0.536033731	0.97680656
1826.2	1876.531	1663.104	0.100139	0.211120083	0.639831259
1000.658	973.5666	1018.612	-0.01558	0.107942056	0.960940117
236.7627	253.6104	253.6051	-0.76577	1.09818E-06	0.000164663
346697.5	334847.5	328628.2	-0.16742	0.69974305	0.251658635
450.2959	453.3402	366.7842	0.300285	0.477021755	0.224439117
14080.68	13406.05	12051.48	0.013759	0.075841354	0.951184679
229.6152	224.8121	275.6121	0.102183	0.547973756	0.752110266
224.2545	208.0906	217.9746	-0.06215	0.956429932	0.831655346
1774.38	1772.486	2083.334	0.423482	0.917013507	0.058175507
26.80333	22.29542	15.71932	-0.27553	0.084125911	0.745567347
1344.634	1558.821	1378.061	0.112078	0.780724949	0.536664638
1380.371	1200.237	1487.048	-0.25725	0.107676441	0.137586476
1253.502	1281.987	1391.684	-0.15604	0.885070844	0.366445441

863.0672	866.7344	927.4401	-0.02541	0.258320792	0.914949396
205.4922	185.7952	209.591	-0.27683	0.886941063	0.278856737
6282.7	5980.746	6485.793	0.095096	0.981637588	0.498608433
1423.257	1353.518	1088.825	-0.83656	2.30178E-13	2.95106E-11
503.9026	523.9423	483.1072	0.040174	0.055422007	0.884584155
746.026	711.5954	738.8082	-0.25906	0.984233953	0.070778066
453.8697	399.4596	341.6333	0.147617	0.033611173	0.679633204
1453.634	1347.015	1344.526	0.103709	0.184354392	0.515558505
1360.716	1440.841	1613.851	-0.31922	0.378784486	0.032265541
1250.822	1082.257	1165.326	0.021179	0.5124279	0.937994709
8728.95	9018.497	9403.299	-0.03825	0.708719765	0.841661214
1212.404	1235.538	1402.164	-0.49431	0.817609984	0.000330467
353.8039	375.3062	361.5444	-0.05183	0.978482639	0.861226913
0	0	1.047955	#DIV/0!	NA	NA
797.8457	780.3396	746.1439	0.014582	0.989430108	0.958055441
844.3048	715.3113	685.3625	0.138636	0.621093622	0.536590083
1453.634	1349.802	1261.738	0.236777	0.786126042	0.119008343
3315.572	3052.614	3267.523	-0.05903	0.962556121	0.764338813
835.3704	826.7884	979.8378	-0.48323	6.59346E-21	0.001092886
1427.724	1450.131	1692.447	-0.213	0.469841794	0.192883976
1685.036	1552.318	1579.268	0.295625	0.812875146	0.114779587
854.1327	840.7231	825.7885	0.175307	0.29215357	0.25282015
156.3527	125.4117	219.0226	-0.3775	0.151275206	0.238475413
571.8043	604.7632	578.4711	-0.24125	0.700017119	0.104544983
2552.57	2221.181	1829.729	0.207172	0.923364203	0.334275175
989.9362	928.0468	854.0832	0.289931	0.861070915	0.136946799
48.24599	47.37776	36.67842	0.212552	0.759261525	0.775439768
402.9434	379.9511	431.7574	-0.07254	0.38441957	0.810673588
804.9933	881.598	774.4387	0.040258	0.149692089	0.848456292
426.1729	484.9253	493.5868	-0.04434	0.281045561	0.9018207
30.3771	76.17601	39.82229	-0.51728	0.437903723	NA
105.4264	130.0566	126.8025	0.52243	0.837998472	0.329120807
945.264	967.0638	1055.291	-0.14282	0.743726011	0.362995196
13.40166	16.72156	26.19887	-1.51655	0.006057733	0.002226503
1640.364	1559.75	1510.103	-0.04868	0.356652725	0.753881265
611.1159	520.2264	482.0593	0.133718	0.880937401	0.70012042
103.6395	96.61348	105.8434	-0.21232	0.54644861	0.772286348
981.0018	920.615	931.6319	0.102945	0.158417136	0.562294649
810.3539	807.2799	890.7617	-0.01267	0.358629856	0.964000071
147.4183	170.9315	146.7137	0.189545	0.17223247	0.680886616
2596.349	2576.979	2511.948	0.087061	0.652050398	0.55088846
22.33611	23.22439	11.5275	-0.24042	0.610423065	NA
1091.789	1033.95	919.0564	0.058397	0.992476114	0.818645356
430.6401	434.7607	421.2779	-0.10175	0.973340554	0.684394991
5.360666	7.431806	24.10296	0.772271	NA	NA
573.5912	629.8456	598.3822	-0.03092	0.270092699	0.897595016
234.9758	222.0252	190.7278	-0.20451	0.523839234	0.537545497
24.12299	60.38342	68.11707	-0.39506	0.614621507	NA
2326.529	2493.371	2036.176	0.163413	0.727101377	0.361564535
973.8542	771.0499	964.1185	-0.19884	0.823340861	0.402016091
1889.635	1928.554	1934.525	0.477718	0.796419683	1.03473E-05
4299.254	4296.513	4987.217	-0.0086	0.795709704	0.974222974
4152.729	4525.041	4652.92	-0.1859	0.792383666	0.145716826
380.6073	295.4143	362.5924	-0.09383	0.88849017	0.735679134

3588.072	3363.821	3221.413	0.228723	0.814880408	0.166877585
687.9521	674.4364	622.4852	0.010816	0.310443697	0.962868426
48.24599	59.45445	71.26093	0.560007	0.067023828	0.322653852
1036.395	932.6917	858.2751	0.266428	0.843186128	0.149383412
0	0	0	#DIV/0!	NA	NA
786.2309	771.0499	797.4937	-0.17192	0.211078128	0.406583019
620.9438	669.7915	756.6234	-0.68653	0.059624319	3.25913E-05
1769.02	1877.46	1842.305	-0.04357	0.935523245	0.855436532
22.33611	25.08235	35.63047	-0.25745	0.962660515	0.730859949
2486.455	2532.388	2920.65	-0.26657	0.800508699	0.065706279
4453.82	4536.189	4599.474	-0.07087	0.504924333	0.602240104
5377.641	5427.076	5084.677	0.059182	0.174380645	0.789143179
3094.891	3278.355	3178.447	-0.12045	0.832043227	0.484163033
1862.831	1666.583	1319.375	0.122454	0.000544345	0.891918558
861.2803	883.4559	792.2539	0.356303	0.446999299	0.060851524
633.452	746.8965	763.9591	-0.233	0.603004299	0.229480039
1932.52	1734.398	1545.733	0.157333	0.976681492	0.39908158
553.9354	598.2604	660.2116	-0.25957	0.535948266	0.173157511
40.20499	64.09933	74.4048	-0.01112	0.310443697	0.973438605
2316.701	2309.434	2014.169	0.199823	0.327964932	0.27042216
370.7794	429.1868	333.2497	-0.1738	0.853477462	0.471424918
1427.724	1582.046	1863.264	-0.08663	0.269120346	0.739592868
534.2797	520.2264	442.237	-0.18953	0.037913264	0.348015857
1692.183	1754.835	1929.285	-0.09319	0.020628498	0.518372194
0	0	0	#DIV/0!	0.732140966	NA
3379.9	3463.222	3770.542	-0.2093	0.890079517	0.168811904
536.0666	475.6356	454.8124	0.404199	0.405459844	0.030614917
33.95088	81.74987	119.4669	0.407514	0.975298616	0.515376266
9344.533	9272.107	8772.43	0.049866	0.340833229	0.708273109
503.9026	487.7123	492.5388	-0.18367	0.29215357	0.327033685
1877.126	1897.897	2692.196	-0.63013	0.988049891	6.10192E-05
343.0826	385.5249	412.8942	-0.14108	0.001178787	0.609093663
1073.92	1207.668	1362.341	-0.45219	7.08114E-06	0.007516064
974.7477	964.2768	942.1114	0.143858	0.92288341	0.55088846
3372.752	3023.816	3401.662	-0.12322	0.515985444	0.399686741
33.05744	33.44313	44.01411	-0.395	0.58195463	0.474497273
6.25411	12.07668	6.287729	-0.83589	0.899176451	NA
1284.773	1283.845	1161.134	0.401135	0.269842373	0.001173192
5388.362	5237.565	6698.528	-0.3408	0.734322432	0.020171833
559.2961	548.0957	607.8138	-0.09302	0.032727278	0.70713713
69.68865	92.89758	91.17208	0.226289	0.902390281	0.681319476
398.4761	478.4225	451.6686	-0.04548	0.907095813	0.8721296
3846.278	3740.056	3747.487	-0.07195	0.483436974	0.590308649
463.6976	294.4853	488.347	0.060356	0.934867993	0.872630949
519.0911	596.4024	600.4782	-0.17675	0.00628885	0.413086202
619.1569	670.7205	610.9577	0.188441	0.430461737	0.437727687
1203.469	1161.22	1237.635	-0.09455	0.098885444	0.547356673
561.9764	530.4452	609.9097	-0.39629	0.137142422	0.010607892
644.1733	701.3767	714.7052	0.462823	0.00382129	0.07575529
496.755	426.3999	410.7983	0.378435	0.947527175	0.178134034
24219.49	22135.63	26864.32	-0.10363	0.831439424	0.681319476
1760.979	1778.06	1605.467	0.197511	0.717185827	0.373144367
132.2298	135.6305	132.0423	0.653785	0.899176451	0.055252373
6146.896	5936.155	6448.066	-0.27052	0.028028845	0.078319563

675.4439	678.1523	574.2793	0.27374	0.327634019	0.312936006
221.5742	263.8291	281.8999	0.209948	0.032727278	0.546687977
34231.42	33728.32	32413.24	-0.30527	0.779325825	0.017617207
2118.356	2088.338	2136.78	0.042917	0.981076246	0.797601985
3865.933	3811.588	3739.103	-0.26764	0.334053294	0.006455545
441.3615	428.2578	421.2779	-0.15982	0.362090257	0.517983853
527.1321	598.2604	777.5825	-0.42728	0.823319422	0.035071812
279.6481	263.8291	233.6939	-0.25932	0.116786217	0.422560599
5009.542	4982.097	4646.632	0.221261	0.72031406	0.022150533
10970.6	10255.89	11956.12	-0.19862	0.62498123	0.181864152
526.2387	589.8996	617.2454	0.051431	0.337335124	0.838085776
1245.461	1154.717	1183.141	-0.03545	0.324651337	0.876474178
1275.838	1101.765	1151.702	-0.00016	0.904997879	0.997242119
788.0178	695.8028	691.6502	0.039455	0.866874007	0.87057216
4405.574	4373.618	4539.741	-0.00111	0.344168489	0.996992829
1140.928	1063.677	965.1665	0.396822	0.900383237	0.003590571
1922.692	1950.849	1984.827	0.450239	0.572336545	9.09677E-05
3403.129	3414.915	2896.547	-0.03495	0.836327255	0.869498619
972.0674	1016.299	1138.079	0.060668	0.908829279	0.786706509
13.40166	11.14771	14.67137	-0.10592	0.337999896	NA
11662.13	11306.56	11244.56	0.023266	0.892614393	0.864252513
10.72133	15.79259	10.47955	-0.93675	0.525774075	NA
729.0505	689.3	770.2468	0.208106	0.98409529	0.390907234
747.8128	680.9392	701.0818	-0.02087	0.804093095	0.919258281
773.7227	943.8394	915.9126	-0.0832	0.022766512	0.746009092
2367.627	2438.561	2175.554	-0.0047	0.756663214	0.988370025
2952.833	3056.33	2761.361	0.200295	0.014083611	0.077779052
2346.185	2526.814	1954.436	0.243239	0.174380645	0.10698808
743.3456	795.2033	681.1707	0.070366	0.107676441	0.759084977
452.0828	458.914	389.8392	0.246612	0.485732259	0.233530606
1078.387	1122.203	947.3512	0.17579	0.901380807	0.328024792
415.4516	483.9964	505.1143	0.015665	0.358055859	0.963671998
461.9107	465.4169	471.5797	-0.24839	0.39778623	0.240259541
2742.874	2463.644	2330.652	0.030401	0.004702869	0.882531058
2483.775	2351.238	2355.803	-0.00402	0.00758806	0.983593071
352.9105	296.3433	311.2426	0.086003	0.310443697	0.80030609
953.305	907.6093	777.5825	0.12771	0.46334755	0.573023969
648.6405	545.3088	516.6418	0.178954	0.668494236	0.484163033
245.6972	228.528	186.536	0.085696	0.732140966	0.795076358
1800.29	1839.372	1912.518	-0.4258	0.363948776	2.16222E-05
366.3121	364.1585	407.6545	0.060802	0.310639346	0.872630949
12912.06	12284.78	12944.34	-0.0237	0.867587609	0.882565871
483.3533	451.4822	534.457	-0.13317	0.072132871	0.551771495
283.2218	300.0592	296.5712	-0.01702	0.54674796	0.961973803
261.7792	320.4966	317.5303	-0.51782	0.011094445	0.010211629
0	13.00566	2.09591	-0.21187	0.917641799	NA
165.2872	148.6361	160.3371	-0.0695	0.972861407	0.862412114
1875.339	1867.241	1847.544	0.320949	0.699717555	0.075363288
1043.543	950.3422	710.5134	0.145897	0.2795135	0.703203936
440.468	442.1925	481.0113	-0.0329	0.730204074	0.908612163
598.6077	628.9166	591.0466	0.060533	0.170750865	0.818645356
17392.68	17164.69	16838.54	-0.00216	0.729944386	0.993537704
857.7065	893.6747	855.1312	-0.16149	0.767024472	0.378149996
1945.922	1938.772	1673.584	0.25392	0.666493976	0.066899278

1140.928	1239.254	1162.182	-0.35842	0.787792061	0.007395438
231.4021	204.3747	280.8519	-0.43019	0.775670478	0.040486778
537.8534	575.036	466.3399	0.02113	0.826883467	0.928761588
443.1484	460.772	448.5247	-0.28082	0.220715145	0.131023807
637.9192	667.9336	694.7941	0.173402	0.853790729	0.49586958
1184.707	1191.876	1290.032	-0.16058	0.045913153	0.241819205
432.427	400.3886	353.1608	0.146145	0.172531274	0.558104763
1504.56	1481.716	1561.453	-0.00809	0.324651337	0.975879427
1478.65	1387.89	1431.506	0.000824	0.000290047	0.997242119
1818.159	1874.673	1747.989	0.102316	0.231792531	0.509540888
4850.509	4701.546	4418.178	0.141002	0.038561058	0.438784455
492.2878	536.948	468.4358	0.063635	0.579600128	0.824241705
619.1569	610.3371	704.2257	-0.21274	0.819847692	0.384859717
962.2395	998.6489	804.8294	0.13179	0.912898714	0.544534957
21.44266	17.65054	25.15092	-0.48954	0.193381894	0.439413908
5517.912	6234.356	6476.361	-0.12289	0.985442377	0.49318083
1412.535	1390.677	1235.539	0.071456	0.617661583	0.703889775
432.427	500.7179	368.8801	0.186351	0.969607454	0.563707226
2650.849	2646.652	2642.942	0.222539	0.976681492	0.0969577
1094.469	1020.944	933.7278	0.092279	0.732140966	0.681964737
746.026	723.6721	673.835	-0.01381	0.728930628	0.9611653
54.5001	56.66752	64.9732	0.754873	0.905394249	NA
556.6158	558.3144	492.5388	0.078175	0.883354954	0.82472084
603.9683	589.8996	532.3611	0.043277	0.735300868	0.875772383
1635.003	1396.251	1240.779	0.106758	0.723128445	0.692801718
688.8455	746.8965	646.5882	0.013751	0.976681492	0.948353155
87.55754	76.17601	101.6516	-0.34875	0.001504449	0.296426431
4943.427	5168.821	4912.813	-0.04151	0.0444701	0.767645401
1271.371	1230.893	1249.162	0.011266	0.752974908	0.962161209
2071.897	2008.446	2244.719	0.028662	0.95896821	0.908612163
841.6245	814.7117	863.5148	-0.07901	0.547164305	0.676538655
778.1899	837.9361	918.0085	-0.17897	0.85608007	0.391432089
419.9188	454.2691	435.9492	0.01351	0.007079262	0.974691849
258.2054	300.9881	295.5233	0.472052	0.034700814	0.181249192
898.8049	862.0895	894.9535	-0.25078	0.730407446	0.255231987
2726.792	2640.149	2734.114	0.104338	0.88874568	0.556664445
974.7477	869.5213	799.5896	0.220083	0.957987619	0.374485512
5602.789	5120.514	4710.557	0.071922	0.028657741	0.709370809
1272.265	1276.413	1263.834	-0.30432	0.60948547	0.0013197
1631.429	1568.111	1491.24	0.078064	0.892614393	0.690294956
586.9929	604.7632	592.0945	-0.01766	0.817609984	0.953867552
30.3771	31.58518	55.54161	-0.98243	0.001942358	0.016996857
418.1319	423.6129	554.3681	0.384212	0.364654282	0.116630357
150.0986	191.369	161.3851	0.104015	0.615439794	0.859505388
3648.826	3621.148	3613.348	0.048596	0.532414268	0.727797788
564.6568	497.002	601.5261	-0.52339	0.001496948	0.000330475
2031.692	1863.525	2002.642	0.15978	0.794232621	0.394044776
1953.069	1873.744	1831.825	0.139868	0.419992784	0.356202781
493.1812	544.3798	389.8392	-0.03373	0.671990676	0.929586888
1088.215	1153.788	1279.553	-0.3404	0.78204194	0.002264631
890.7639	871.3793	910.6728	0.384954	0.639085319	0.616511903
645.0668	720.8852	567.9916	0.364417	0.998398411	0.063099827
1197.215	1359.092	1415.787	-0.15018	0.541271961	0.420106203
201.9184	189.5111	194.9196	-0.6964	0.031833517	0.00276038

704.9275	757.1152	729.3766	0.149172	0.828016939	0.518514401
545.8944	614.982	746.1439	-0.11119	0.969843993	0.673302041
14729.32	14639.73	15448.95	-0.00427	0.98299578	0.985850238
515.5173	559.2434	472.6277	-0.2832	0.289670982	0.191922609
334.1482	380.8801	288.1876	0.102198	0.029154173	0.775889861
932.7558	863.9475	946.3033	-0.04733	0.036016203	0.847133587
19.65577	29.72722	13.62341	-0.08465	0.842190796	NA
545.001	582.4678	637.1566	-0.3995	0.064051876	0.021007242
1467.929	1218.816	1210.388	0.179895	0.818107632	0.345340904
2653.529	2745.123	2856.725	0.206621	0.017391628	0.247256105
11664.81	11551.81	10821.18	-0.10384	0.311697167	0.392881614
921.141	793.3453	785.9662	0.20049	0.883354954	0.40615036
15946.19	15580.78	15336.82	0.030516	0.203841572	0.835596295
339.5088	410.6073	408.7024	-0.01118	0.365953812	0.971161723
844.3048	816.5697	848.8435	-0.01272	0.972861407	0.962868426
1195.428	1051.601	1142.271	0.082284	0.769710962	0.664215073
106.3199	105.9032	111.0832	-0.09547	0.638519612	0.835596295
1344.634	1302.424	1231.347	-0.05493	0.011275313	0.810986507
680.8045	588.0417	646.5882	0.477126	0.602909819	0.147449754
850.5589	923.4019	717.8491	0.153932	0.174775082	0.519070487
232.2955	274.0478	325.914	-0.00715	0.825699238	0.977734128
578.0584	566.6752	494.6347	0.331551	0.270092699	0.050220436
715.6489	640.9933	700.0339	0.249448	0.33210787	0.269755378
1254.396	1097.12	1362.341	0.329168	0.271291894	0.02960885
4458.287	4120.936	3920.399	0.153692	0.681216827	0.519258549
952.4116	926.1888	906.481	0.265522	0.335666342	0.124605424
26028.71	25448.36	23480.48	0.101417	0.071245169	0.55088846
14744.51	13702.39	12675.01	0.042458	0.283639033	0.831580345
550.3617	528.5872	466.3399	0.171851	0.542904501	0.51210202
8722.696	8461.111	8381.543	0.133715	0.820906044	0.134131676
2112.996	1988.008	2068.663	0.204117	0.564427849	0.119008343
804.9933	754.3283	763.9591	0.050255	0.788811844	0.835596295
520.878	422.684	389.8392	0.240154	0.827268091	0.368584102
340.4023	363.2295	383.5515	-0.10012	0.090068897	0.753881265
550.3617	613.124	600.4782	0.108001	0.412464172	0.573023969
1199.896	1316.359	1234.491	-0.94598	0.000288624	2.76009E-08
605.7552	698.5898	623.5332	-0.12707	0.443017929	0.552800466
9994.961	9581.456	8552.36	0.083933	0.394573102	0.680886616
2620.472	2718.183	2760.313	-0.13569	0.055722587	0.326388465
82.19687	82.67884	74.4048	0.636587	0.851080991	0.19887348
2013.823	1977.789	2180.794	0.13078	0.986125936	0.476290126
751.3866	806.351	760.8153	0.02124	0.183923135	0.921848549
1057.838	1190.018	1204.1	-0.08958	0.333195304	0.642738974
1.786889	0	0	#DIV/0!	NA	NA
75.94276	63.17035	74.4048	-0.18634	0.827268091	0.765144842
172.4347	196.9429	203.3032	-0.06667	0.787792061	0.848456292
2117.463	2074.403	2750.882	-1.41828	7.42479E-28	3.15448E-31
381.5007	359.5136	319.6262	0.009184	0.955592311	0.97680656
2086.192	2167.3	2251.007	-0.0891	0.483419188	0.608610845
10231.72	9830.422	9600.315	0.016434	0.961125122	0.902851746
343.0826	299.1302	336.3935	-0.14046	0.701216566	0.659338327
1889.635	1771.557	2020.457	-0.09423	0.787792061	0.643988563
122.4019	89.18167	106.8914	0.012571	0.512542378	0.990444813
2676.759	3053.543	3069.46	-0.06733	0.398019504	0.727522975

1302.642	1309.856	1193.621	0.19149	0.798409569	0.178822023
854.1327	728.317	802.7334	0.5428	0.350866676	0.000188607
513.7304	471.9197	498.8265	-0.23448	0.810901942	0.267468464
3303.063	3454.861	3596.581	0.044603	0.844410461	0.784199388
650.4274	726.459	619.3413	-0.04727	0.145698843	0.875604624
4837.107	4773.077	4646.632	-0.03982	0.177527106	0.818945755
2810.776	2558.399	2909.123	-0.15815	0.035877527	0.331112797
11.61478	9.289758	18.86319	-0.63007	0.218419472	NA
334.1482	366.0165	318.5783	0.261912	0.936346196	0.437727687
521.7714	578.7519	500.9224	0.287206	0.865150494	0.099513252
1148.969	1190.018	914.8646	0.007824	0.695990097	0.98477285
17.86889	6.50283	8.383639	0.67993	0.731499952	NA
3717.622	3828.309	3923.543	-0.0766	0.004783907	0.531682752
1197.215	1039.524	1159.038	0.08349	0.774318005	0.680886616
982.7887	969.8507	935.8237	0.102652	0.907134252	0.658487105
61.64765	80.82089	82.78844	-0.26697	0.296056707	0.536500997
1071.24	1092.475	1073.106	0.10296	0.596065856	0.620471139
3980.294	3550.545	3383.846	0.115376	0.417487982	0.603754605
3383.473	3453.932	3147.009	0.311575	0.121148599	0.032632609
720.1161	689.3	708.4175	0.194206	0.365272696	0.280613898
0	0	0	#DIV/0!	NA	NA
1251.715	1243.899	1452.465	-0.15088	0.998398411	0.402824474
1893.208	1992.653	1581.364	0.094161	0.324651337	0.751339004
549.4682	554.5985	505.1143	0.051814	0.569085135	0.821432697
2096.914	1975.002	2069.711	0.006093	0.678868378	0.980970686
839.8376	783.1266	719.945	0.100746	0.57058689	0.666556565
3.573777	10.21873	8.383639	0.694824	NA	NA
1270.478	1178.87	1078.346	0.312071	0.415765665	0.29075107
3664.908	3780.002	4175.052	-0.46147	0.984089285	0.001688143
1407.175	1423.191	1445.13	-0.28384	0.992476114	0.004814724
8.934443	12.07668	16.76728	-0.27747	0.756107114	NA
91.13131	103.1163	125.7546	-0.17425	0.020548676	0.713308089
1730.602	1777.131	1891.559	0.016227	0.53298351	0.929442227
1557.273	1517.017	1636.906	-0.05661	0.262947776	0.771436828
2737.513	2821.299	3084.131	-0.01573	0.659336928	0.931542231
2867.063	2723.757	2848.341	-0.13802	0.038048538	0.33000998
363.6318	338.1472	334.2976	-0.2184	0.192384879	0.34361128
2064.75	1928.554	2105.341	-0.10561	0.660833087	0.620471139
729.0505	702.3057	772.3428	-0.29193	0.699465012	0.102776029
788.9113	753.3993	792.2539	0.159911	0.670504707	0.402824474
130.4429	140.2753	177.1044	-0.02856	0.548982934	0.952588397
271.6071	312.1359	289.2356	0.029518	0.831928191	0.948353155
797.8457	766.405	747.1918	0.018574	0.716063981	0.951973804
1157.904	1172.367	1083.585	0.281323	0.296333484	0.171950723
2577.587	2511.021	2529.763	-0.06646	0.127584376	0.658757039
3591.646	3508.741	3379.655	0.129458	0.986203512	0.303336182
475.3123	444.0504	433.8533	-0.04817	0.368034878	0.87466351
1257.97	1263.407	1307.848	-0.22331	0.46429351	0.149957196
4.467221	9.289758	6.287729	-0.72089	0.701216566	NA
293.9432	331.6443	388.7913	-0.04689	0.827268091	0.869372302
1785.102	1788.278	1615.946	0.178425	0.497319548	0.414929365
456.55	431.9737	445.3808	-0.26635	0.212716004	0.328024792
185.8364	206.2326	303.9069	0.263073	0.28287487	0.49413501
13054.11	13286.21	11841.89	0.095473	0.043196135	0.601242135

770.149	884.3849	788.0621	-0.178	0.735300868	0.439570865
3003.76	3230.049	2969.904	0.034862	0.988049891	0.818645356
2589.201	2631.788	2658.662	-0.21636	0.577926236	0.253693985
792.4851	836.0782	978.7899	-0.058	0.651388825	0.875604624
241.2299	247.1076	202.2553	0.307173	0.867587609	0.382319285
143.8445	181.1503	222.1664	0.094752	0.393551305	0.822050288
3496.047	3464.151	3366.031	0.009022	0.562962798	0.969186716
523.5583	480.2805	464.244	0.152367	0.947669058	0.422560599
806.7802	857.4446	819.5007	-0.12817	0.940969382	0.566141749
374.3531	392.9567	448.5247	-0.04237	0.112147069	0.895438079
1962.004	2095.769	2211.185	-0.15213	0.327964932	0.23616229
125.9756	149.5651	144.6178	-0.45146	0.236209282	0.128202832
12543.06	12302.43	14059.36	-0.02446	0.645124304	0.901090975
1500.986	1558.821	1601.275	-0.03992	0.769710962	0.864252513
1093.576	1061.819	1045.859	-0.07805	0.371964696	0.676897688
323.4268	315.8518	307.0508	-0.04596	0.745767703	0.872379241
244.8037	266.616	225.3103	-0.12704	0.579932002	0.702040936
1291.027	1281.987	1247.066	0.287088	0.587719544	0.087007219
75.94276	105.9032	86.98026	-0.05714	0.117208522	0.923725803
2863.489	2759.058	2609.408	-0.02525	0.033849408	0.875772383
808.5671	837.9361	823.6925	-0.0567	0.990604058	0.775439768
1098.043	1095.262	1161.134	-0.09602	0.772576047	0.672730781
3189.596	3368.466	2964.664	0.112998	0.444589889	0.41049793
1372.33	1536.526	1345.574	0.115967	0.17246068	0.639154099
1859.258	1767.841	1751.133	0.13366	0.162464449	0.44710318
952.4116	1001.436	1024.9	-0.06643	0.855484931	0.772033634
8.040998	7.431806	6.287729	0.226514	0.076086967	NA
29.48366	27.86927	52.39774	-0.02815	0.729364673	0.957070324
5934.257	6228.782	6258.387	-0.04893	0.872688807	0.797601985
2510.578	2689.385	2675.429	-0.08541	0.428038739	0.603217016
497.6485	601.9763	527.1213	0.146626	0.962660515	0.493837083
1310.683	1446.415	1486	-0.02381	0.925203985	0.908727843
301.9842	267.545	398.2229	-0.23677	0.986203512	0.402824474
1408.962	1384.174	1571.932	-0.0109	0.490813332	0.957489272
3404.023	3664.809	3641.643	-0.02275	0.45171569	0.899731862
1470.609	1432.481	1258.594	0.387293	0.025688649	0.167499635
1507.24	1526.307	1834.969	-0.3102	0.473487587	0.058966813
3202.998	3216.114	3052.693	-0.2364	0.003208611	0.018001949
1299.961	1214.171	1221.915	0.050916	0.899176451	0.826468162
1804.757	2005.659	1930.333	-0.01607	0.618280897	0.952680272
4598.558	4308.59	3740.151	0.220529	0.668230752	0.267586812
1248.142	1343.299	1433.602	-0.02093	0.429631603	0.923725803
803.2064	762.6891	729.3766	0.164523	0.62292707	0.59816002
1275.838	1307.998	1373.869	-0.38172	0.07881338	0.000782831
958.6657	977.2825	1154.846	-0.06676	0.425337875	0.763346604
263.5661	261.9712	285.0437	0.005233	0.000567925	0.985001162
123.2953	129.1276	130.9944	-0.00516	0.89036901	0.981434865
7186.866	6814.037	6205.989	0.054962	0.288546176	0.808870475
145.6314	137.4884	173.9605	-0.16087	0.020628498	0.723437511
1426.83	1485.432	1383.3	0.04363	0.410903637	0.814809717
3991.909	4072.63	3577.718	0.114677	0.485973657	0.505302546
350.2301	393.8857	341.6333	-0.15341	0.602427087	0.519258549
749.5997	778.4817	799.5896	-0.06539	0.441361968	0.751940308
488.714	473.7776	536.5529	0.306827	0.006229103	0.207278086

273.3939	281.4797	343.7292	-0.09552	0.500344775		0.726460865
2703.562	2776.709	3043.261	-0.0524	0.382918803		0.784199388
1579.609	1557.892	1892.607	0.062931	0.566548852		0.786706509
6665.988	7143.824	7270.711	-0.33593	0.378784486		0.002779203
35.73777	21.36644	26.19887	-0.40341	0.769710962	NA	
1375.011	1469.64	1279.553	0.206284	0.935611683		0.358153185
3657.761	3522.676	3670.986	0.224198	0.705615772		0.045330931
665.616	634.4904	584.7588	0.046412	0.991136341		0.884584155
789.8047	775.6948	751.3837	-0.38887	0.000710235		0.034978732
2914.415	2860.316	3386.99	-0.45904	0.83213076		8.91351E-05
92.9182	93.82655	101.6516	0.363291	0.136084466		0.356068164
173.3282	170.0026	197.0155	-0.13869	0.795709704		0.634014533
648.6405	588.0417	606.7659	-0.11975	0.934554042		0.601858923
568.2305	661.4307	666.4993	0.073802	0.087280745		0.800339699
445.8287	428.2578	377.2638	0.453394	0.226021964		0.057894023
828.2228	940.1235	777.5825	-0.14837	0.02453229		0.462978863
480.673	434.7607	478.9154	0.452857	0.985859213		0.017695039
6942.062	6839.12	6575.917	0.073468	0.139873727		0.524211956
229.6152	244.3206	291.3315	-0.3812	0.957754117		0.114544408
2178.217	2418.124	2315.98	0.042436	0.220715145		0.832900164
69.68865	101.2584	88.02821	-0.23242	0.804201321		0.660222897
747.8128	805.422	1075.202	-0.59298	0.451991382		0.000232021
1475.076	1377.671	1436.746	0.031589	0.352663598		0.872582499
1143.609	1109.197	1149.607	-0.01513	0.270719269		0.931618549
1914.651	1919.264	1960.724	-0.02484	0.847532334		0.907473183
1140.928	1111.984	933.7278	0.050447	0.893366537		0.845206982
3092.211	3122.288	3265.427	-0.13791	0.847714103		0.386225298
1420.576	1478	1341.382	0.150057	0.848694292		0.386078627
167.9675	180.2213	191.7757	0.343088	0.120606425		0.253693985
902.3787	946.6263	986.1256	-0.15127	0.909569287		0.361214706
187.6233	181.1503	176.0564	0.407054	0.812875146		0.128665301
7680.94	6811.25	7319.965	0.350349	0.700017119		0.015466264
395.7958	413.3942	429.6615	-0.29312	0.14853364		0.076809757
6150.47	6226.925	6135.776	0.008425	0.792868376		0.953867552
5.360666	7.431806	15.71932	-0.38364	0.7406684	NA	
17171.11	16925.01	16387.92	0.024012	0.432073195		0.875604624
930.9689	1103.623	842.5557	0.083871	0.507065266		0.722021255
975.6411	883.4559	740.9041	0.309151	0.68164261		0.167499635
1785.995	1680.517	1515.343	0.095155	0.861070915		0.656460739
4326.951	4655.098	4112.175	0.136437	0.868137167		0.442485492
2075.471	2048.392	2065.519	0.03379	0.469347309		0.864363898
52.71321	72.46011	97.45981	0.087053	0.603004299		0.905975684
816.6081	969.8507	1009.181	-0.3049	0.880647749		0.066700944
4540.484	4224.053	4486.295	-0.11367	0.527593235		0.394044776
1.786889	0.928976	1.047955	#DIV/0!	NA	NA	
1785.102	1733.469	1816.106	-0.038	0.455280748		0.827790925
27.69677	12.07668	2.09591	-0.56552	0.739610643		0.583691606
1689.503	1682.375	2023.601	-0.26091	0.191527971		0.067405031
425.2795	491.4282	512.4499	0.165915	0.835449534		0.420787436
332.3613	336.2892	354.2088	0.118494	0.981637588		0.685537269
3666.695	3777.215	3801.98	-0.63066	0.000195297		2.34866E-13
3483.539	3662.022	3244.468	0.009084	0.168909978		0.962321489
2743.767	2508.235	2547.578	0.091702	0.617661583		0.486646679
3067.194	3032.177	3533.704	0.331352	0.29215357		0.018058475

301.9842	243.3916	234.7419	0.174716	0.872095034	0.612005134
261.7792	292.6274	278.756	-0.14048	0.079950184	0.692421497
1126.633	1195.592	1174.757	-0.17083	0.587719544	0.280613898
1982.553	2017.735	2181.842	0.245475	0.207483701	0.10424789
1157.904	1228.106	1242.875	0.321708	0.977048368	0.046054594
77.72965	81.74987	106.8914	-0.12612	0.925116667	0.840777068
2094.233	2382.823	1968.059	0.223142	0.474981124	0.207615914
2625.833	3088.844	2742.498	0.158832	0.784378477	0.256199693
7317.308	6986.827	6442.827	0.074655	0.243530103	0.688084851
998.8707	1057.174	1034.331	0.053427	0.987989692	0.774230303
913.9935	903.8934	956.7828	0.165894	0.235856061	0.495076977
1251.715	1176.083	1064.722	0.038559	0.18279362	0.859505388
2457.865	2435.774	2439.639	-0.28142	0.427251895	0.025807779
2890.292	3054.472	2905.979	-0.18474	0.839922799	0.281445446
5240.051	4959.802	4882.422	-0.08352	0.307348283	0.531682752
329.6809	407.8204	354.2088	0.143263	0.804410815	0.658487105
1937.881	1876.531	2094.862	-0.51733	0.004455063	1.28535E-07
1233.847	1227.177	1326.711	-0.15975	0.394443433	0.27042216
1998.635	1977.789	1974.347	-0.12324	0.752974908	0.545627423
4397.533	4244.49	4067.113	0.16558	0.542904501	0.361214706
519.9846	628.9166	667.5473	-0.0403	0.07582841	0.887845961
279.6481	183.9372	219.0226	-0.07807	0.792868376	0.859505388
46.4591	31.58518	59.73343	-0.45964	0.814880408	NA
718.3292	657.7148	687.4584	0.132187	0.728166455	0.592123352
83.98376	58.52547	93.26799	0.34709	0.4069354	0.515558505
1741.323	1642.429	1780.475	0.047241	0.110895942	0.791888862
476.2058	526.7293	565.8956	-0.15441	0.73478662	0.572103667
1863.725	1707.457	1747.989	0.206826	0.223826825	0.305200742
424.386	441.2635	410.7983	0.13015	0.960773888	0.617995918
2640.128	2702.39	2913.315	-0.21627	0.310443697	0.099245982
1933.413	1963.855	1706.071	0.205395	0.825592309	0.266809788
2307.767	2376.32	2232.144	0.075017	0.27810082	0.66000496
803.2064	761.7601	725.1848	-0.04201	0.281045561	0.84551374
768.3621	842.581	819.5007	0.009838	0.27470085	0.97680656
48.24599	45.51981	38.77433	-0.08499	0.835764764	0.919055186
257.3119	250.8235	238.9337	0.039057	0.582750031	0.915422831
4918.411	5061.06	5978.583	-0.40541	0.813154117	0.00622934
632.5585	659.5728	656.0198	0.055895	0.759261525	0.775439768
684.3783	601.0473	534.457	-0.0196	0.592795512	0.945029596
146.5249	180.2213	145.6657	0.064165	0.614563272	0.904467867
2230.037	2275.062	2487.845	-0.01868	0.174775082	0.929723758
4970.23	4828.816	5382.296	0.013732	0.830173942	0.939512313
34.84433	22.29542	26.19887	-0.63016	0.828690815	NA
36.63121	14.86361	32.4866	-0.24273	0.549071806	0.741001187
672.7635	887.1719	643.4443	0.068932	0.302352413	0.814086627
727.2636	752.4704	656.0198	0.142338	0.142424892	0.577160388
598.6077	554.5985	572.1834	-0.04236	0.518546793	0.881988452
310.9186	300.9881	433.8533	0.148595	0.756303962	0.636020697
504.796	522.0844	561.7038	-0.03625	0.208221128	0.910820584
8364.425	8450.893	9111.968	-0.20453	0.535516871	0.054239832
1869.085	1772.486	1987.97	0.001894	0.973922065	0.992528568
2221.996	2270.417	1996.354	0.168394	0.709818881	0.302912952
687.0586	683.7262	733.5684	-0.11237	0.818647954	0.507147953
1703.798	1648.932	1663.104	0.148094	0.359465113	0.381794066

1614.454	1748.332	1661.009	0.091065	0.430461737	0.600511462
239.4431	270.3319	280.8519	-0.49051	0.760895174	0.092469287
16527.83	16324.89	15413.32	0.176644	0.240910061	0.095730045
436.8942	438.4766	389.8392	0.089629	0.969843993	0.753881265
1635.896	1641.5	1534.206	0.199383	0.197390524	0.265058952
2830.431	2834.305	2988.767	-0.16591	0.898465596	0.501883079
1944.135	2118.065	2187.082	-0.3123	0.004522157	0.017040072
1012.272	1050.672	1361.293	-0.27424	0.770381061	0.21158075
290.3694	250.8235	272.4683	-0.0804	0.694854991	0.81402952
1212.404	1205.811	1146.463	0.206097	0.44898062	0.323503842
461.0172	510.0077	595.2384	-0.16283	0.30299466	0.509206637
5081.911	4914.282	4336.437	0.245165	0.038021746	0.255165763
771.0424	744.1096	873.9944	-0.23405	4.67312E-09	0.38666632
22250.34	21532.73	26309.96	0.381904	0.423638846	0.017404146
708.5013	681.8682	658.1157	0.116085	0.660762726	0.544534957
3697.966	3602.568	3800.932	-0.32489	0.145034314	0.010351567
11.61478	5.573855	1.047955	1.693728	0.892240442	NA
835.3704	720.8852	629.8209	0.352733	0.730407446	0.227305931
1148.969	1026.518	884.4739	0.005087	0.550533949	0.984451848
3236.949	3023.816	3109.282	-0.13238	0.994145678	0.317595626
537.8534	655.8569	665.4514	-0.46945	0.054140788	0.004349123
679.0176	704.1636	575.3272	0.448909	0.621884159	0.14738752
25.01644	25.08235	24.10296	0.491117	0.272100977	NA
180.4757	165.3577	164.5289	-0.06005	0.312335761	0.856558496
2583.841	2562.115	2337.987	-0.34077	0.187777748	0.001137309
1349.994	1354.447	1216.676	0.081768	0.325572594	0.708273109
276.9677	284.2666	328.0099	0.042535	0.434274199	0.932794989
1.786889	4.644879	14.67137	1.526786	NA	NA
3353.99	3387.046	3302.106	-0.16571	0.066264238	0.144747451
874.6819	961.4899	958.8787	0.134989	0.671378444	0.442655501
881.8295	880.669	734.6164	-0.04712	0.886112674	0.858316687
3066.301	2908.623	3087.275	0.323628	0.000183667	0.032265541
0	2.786927	1.047955	#DIV/0!	NA	NA
2.680333	0.928976	5.239774	-0.46593	0.902516624	NA
17212.2	17108.02	18343.4	-0.19308	0.940969382	0.229480039
316.2793	308.42	334.2976	0.06018	0.936346196	0.841212475
102.7461	133.7725	138.33	0.263639	0.576828718	0.601858923
4322.483	4283.507	5038.567	0.086258	0.780212598	0.697774046
3244.99	3250.486	3803.028	-0.27478	0.368085564	0.112687221
1410.748	1454.776	1570.884	-0.31785	0.795811864	0.085100563
828.2228	837.0072	861.4189	1.16E-05	0.898465596	0.996992829
201.9184	234.1019	253.6051	-0.03827	0.210289554	0.924858067
688.8455	761.7601	803.7814	-0.02023	0.834965572	0.94960537
0	4.644879	0	-0.37096	0.868785389	NA
707.6079	668.8625	570.0875	0.322165	0.314879449	0.060851524
822.8622	827.7174	949.4471	-0.1425	0.520961145	0.627247952
646.8536	672.5785	530.2652	0.073609	0.425091186	0.781285519
1267.797	1294.992	1351.862	0.01391	0.948896953	0.948371294
179.5823	153.281	219.0226	-0.12058	0.00758806	0.79799583
2905.481	2892.831	3177.399	-0.10736	0.430461737	0.39276575
655.7881	586.1837	743	0.232143	0.730407446	0.277548691
2280.07	2253.695	2692.196	0.002198	0.558928437	0.996992829
3783.736	3953.721	4737.804	-0.2485	0.820742844	0.148566635
1060.518	1142.64	1109.784	-0.0408	0.394573102	0.871061917

927.3951	897.3906	731.4725	0.1542	0.344730624	0.477288843
382.3941	377.1642	422.3258	0.053991	0.577578732	0.84002392
13116.66	12945.28	11255.04	-0.05983	0.158538481	0.80286002
503.9026	510.9367	614.1016	-0.16068	0.596065856	0.515285838
740.6653	732.9619	757.6714	0.112403	0.443017929	0.599924988
174.2216	189.5111	259.8928	-0.35494	0.701216566	0.316260034
91.13131	127.2697	130.9944	0.001539	0.976681492	0.99985654
705.821	600.1183	602.5741	-0.09398	0.837529432	0.751464759
809.4605	750.6124	739.8562	0.005968	0.847410002	0.984451848
941.6902	1113.842	1084.633	-0.30305	0.417038094	0.017912698
1892.315	1943.417	1870.599	0.071436	0.837529432	0.679633204
2453.398	2393.971	2413.44	-0.10823	0.901380807	0.468800279
1513.495	1499.367	1484.952	0.133148	0.066028159	0.48662881
1963.79	1976.86	2124.205	-0.02389	0.986203512	0.91372954
636.1323	759.9022	788.0621	-0.27043	0.976681492	0.109765004
13278.37	12870.03	12835.35	-0.09369	0.556706854	0.468899388
1404.494	1540.242	1480.76	0.005556	0.496884574	0.978507567
2052.241	2025.167	2181.842	-0.05836	0.825592309	0.761486667
848.772	914.1121	835.2201	0.154382	0.976681492	0.457073409
2491.816	2401.402	2206.993	0.096073	0.759261525	0.571284382
6381.872	6265.013	5792.047	0.124103	0.189629036	0.472093197
588.7798	565.7462	619.3413	-0.07201	0.716063981	0.756407952
549.4682	621.4848	498.8265	0.146987	0.87773756	0.556052555
12691.38	12317.29	11774.82	-0.02354	0.017639055	0.883280306
756.7473	749.6834	648.6841	0.248732	0.875830579	0.303824089
521.7714	648.4251	498.8265	0.03383	0.853320493	0.905168779
115.2543	115.193	155.0973	0.249976	0.04017813	0.600553967
469.0582	525.8003	519.7856	-0.11494	0.010982999	0.630318178
1833.348	1786.42	1724.934	0.099532	0.549071806	0.511081603
867.5344	967.0638	1112.928	-0.32137	0.272597363	0.210822498
2191.619	2186.809	1850.688	0.215095	0.584494617	0.216876753
835.3704	745.0386	674.883	0.215361	0.74589429	0.315911601
1838.708	1903.471	1820.298	0.110073	0.985859213	0.441429894
2037.053	2154.295	2184.986	-0.00097	0.09014688	0.999675887
869.3213	856.5157	783.8703	-0.01405	0.944990108	0.9614279
2498.07	2330.8	2064.471	0.25911	0.313396084	0.267586812
1259.756	1213.242	1089.873	-0.11911	0.206702304	0.560116425
185.8364	259.1842	209.591	-0.33222	0.104651539	0.230651936
1300.855	1307.998	976.694	0.19925	0.379509905	0.419308591
824.6491	937.3365	952.591	-0.29852	0.623663103	0.024919247
1149.863	1136.137	994.5092	-0.07386	0.001833424	0.751339004
877.3623	764.5471	945.2553	-0.01492	0.730407446	0.967817837
1230.273	1195.592	1352.91	0.091984	0.842806932	0.689321642
1032.822	1072.038	1244.97	-0.1266	0.911996675	0.555053384
1148.969	1181.657	1159.038	0.157855	0.83703582	0.300201673
553.9354	521.1554	577.4231	0.077903	0.622518586	0.748344041
1090.895	1124.061	921.1524	0.120783	0.173085065	0.647261886
24244.5	23208.6	22706.04	0.147133	0.114505424	0.13004175
13778.7	13094.84	14004.87	0.180948	0.358649739	0.164117766
23.22955	17.65054	20.9591	-0.13877	0.395079365	NA
4891.607	4924.501	4783.914	-0.03189	0.854862911	0.862462136
8404.63	8198.211	8590.086	-0.24071	0.933380146	0.005720058
363.6318	362.3005	388.7913	-0.21546	0.236875869	0.250598589
610.2224	585.2547	588.9507	-0.05854	0.095377019	0.823966789

430.6401	423.6129	329.0578	0.105677	0.843507519		0.748344041
839.8376	780.3396	763.9591	0.152467	0.27810082		0.616338723
1950.389	2016.806	2003.69	-0.07601	0.843507519		0.680262772
2415.873	2241.619	2485.749	0.01228	0.175067813		0.945029596
1847.643	1958.281	1967.011	0.03442	0.732140966		0.84572993
1090.895	1114.771	1217.724	-0.11145	0.937354907		0.536500997
1028.354	1133.35	1024.9	-0.22686	0.840099446		0.233465653
1708.265	1760.409	1925.093	-0.04837	0.652135039		0.838122056
1824.413	1715.818	2023.601	-0.00278	0.787792061		0.990142758
221.5742	205.3036	215.8787	0.23445	0.614621507		0.422116695
309.1317	376.2352	343.7292	-0.02247	0.962660515		0.944445597
6306.823	6247.362	7288.526	-0.39862	0.614621507		0.004483661
124.1888	156.9969	129.9464	-0.08958	0.157331056		0.819004
2286.324	2284.351	2047.704	0.290763	0.800609294		0.102488637
285.9022	294.4853	208.543	0.073744	0.084559824		0.864363898
359.1646	342.7921	286.0917	0.287134	0.37602059		0.187388152
25.01644	13.00566	24.10296	0.011257	0.919200531	NA	
1241.888	1348.873	1284.793	-0.08188	0.141982581		0.643901257
4731.681	4721.984	4300.807	-0.09511	0.892614393		0.484464564
787.1244	709.7375	790.158	0.087906	0.993501117		0.656460739
964.9198	905.7514	798.5416	-0.45112	0.988049891		0.001957111
106.3199	176.5054	123.6587	-0.33808	0.253157581		0.367817075
286.7956	357.6557	286.0917	-0.09214	0.504054705		0.774230303
898.8049	839.7941	923.2483	0.107843	0.987989692		0.56192082
2025.438	2033.528	2463.742	-0.07564	0.476024675		0.723760266
531.5993	606.6212	702.1298	-0.11971	0.99650011		0.606456502
9613.46	9419.814	9466.177	-0.12539	8.51337E-08		0.25935495
3212.826	3116.714	3220.365	-0.09003	0.796419683		0.628282957
2825.071	2860.316	2508.804	0.270194	0.409809283		0.264692489
359.1646	334.4313	357.3526	-0.39018	0.394573102		0.036057439
1249.035	1176.083	1244.97	-0.03548	0.476024675		0.861107589
911.3131	963.3479	869.8026	-0.21635	0.010982999		0.175074667
315.3858	352.0818	256.7489	0.725288	0.855484931		0.00200924
30744.31	29738.37	29091.23	-0.03871	0.826883467		0.826468162
154.5659	152.352	148.8096	-0.13078	0.989430108		0.739592868
957.7722	982.8564	1057.386	0.031557	0.223467289		0.885571199
2204.127	2256.482	2483.653	-0.18388	0.791218214		0.219363336
419.0254	384.596	368.8801	0.132733	0.98843748		0.630318178
401.1565	362.3005	427.5656	-0.18269	0.887028326		0.359873864
1282.986	1131.492	1244.97	0.136188	0.764395772		0.598691725
11327.98	11271.26	10430.3	0.120277	0.564057468		0.34727798
2586.521	2768.348	2626.175	-0.1729	0.854862911		0.256269459
2.680333	0	0	-0.91651	0.793705768	NA	
958.6657	929.9047	950.4951	-0.04042	0.114505424		0.838059844
306.4514	310.2779	296.5712	0.17731	0.732140966		0.521104798
700.4603	517.4395	512.4499	0.040878	0.182617755		0.90980808
11283.31	11403.18	10813.85	0.054376	0.013492234		0.680886616
209.066	265.6871	250.4612	-0.57027	0.898465596		0.002158789
2132.651	2177.519	2135.732	-0.06627	0.04129035		0.677324798
1459.888	1406.469	1634.81	0.123854	0.978835583		0.61283164
1714.52	1786.42	1722.838	0.07789	0.504924333		0.679997772
797.8457	687.4421	706.3216	0.260125	0.809173332		0.20747063
1936.094	1735.327	1785.715	0.181419	0.526520418		0.159639694
1693.077	1686.091	1720.742	-0.19576	0.009061203		0.112057261

32.16399	26.9403	28.29478	0.000712		0.530486695	NA
1236.527	1151.93	1127.599	0.338045		0.057825057	0.015498519
1113.232	1029.305	969.3583	0.165383		0.06446805	0.353465216
3919.54	4099.57	4659.207	-0.22465		0.470002734	0.181446527
800.5261	761.7601	668.5952	0.286976		0.935506037	0.104692027
2007.569	1918.335	1814.01	0.10423		0.760106856	0.649685593
2196.086	2136.644	2105.341	0.002878		0.959739969	0.990023409
643.2799	708.8085	903.3371	-0.18412		0.892240442	0.402023383
1740.429	1752.048	1815.058	0.132069		0.603609787	0.389799953
4045.516	4139.516	3728.624	0.072459		0.195806907	0.669576945
1345.527	1296.85	1310.992	-0.28236		0.439053322	0.023570036
701.3537	716.2403	695.842	0.146203		0.80050738	0.471293277
710.2882	568.5332	571.1354	0.143767		0.92790798	0.531682752
885.4033	897.3906	898.0973	0.160004		0.527593235	0.365275241
3840.917	3706.613	3712.904	0.190286		0.901380807	0.060644712
4538.697	4535.26	4609.954	-0.11092		0.019077937	0.372102023
889.8705	815.6407	887.6178	0.027309		0.681253334	0.903329758
0.893444	0	0	-0.91651	NA		NA
1077.494	1075.754	1031.188	0.094443		0.827268091	0.648517537
9.827887	22.29542	23.05501	1.328467		0.696515701	NA
841.6245	782.1976	747.1918	0.139748		0.78204194	0.574986893
1897.676	2039.102	1908.326	0.042033		0.853008354	0.818055883
636.1323	727.388	703.1777	0.148927		0.926873595	0.578506971
3135.096	3134.364	2975.144	0.131083		0.760924499	0.306199402
4190.254	4033.613	3300.01	0.188768		0.045913153	0.505302546
3045.751	2894.688	2594.736	0.314572		0.02597477	0.174598931
2.680333	6.50283	4.19182	0.730208	NA		NA
3800.712	3751.204	4270.416	-0.30792		0.728952588	0.034584179
100.0658	95.6845	85.9323	0.218729		0.186661493	0.681857234
172.4347	170.9315	141.4739	-0.20481		0.233112258	0.573023969
12.50822	15.79259	28.29478	-0.1574		0.76444128	NA
286.7956	361.3716	365.7363	-0.34802		0.01992954	0.201829761
550.3617	644.7092	564.8477	-0.19607		0.148672328	0.361564535
1432.191	1391.606	1202.004	0.456683		0.68563527	0.021007242
38.4181	78.03396	45.06206	-0.38828		0.563351252	0.505493145
218.0004	221.0962	264.0846	-0.74398		0.95291647	0.000703618
99.17231	105.9032	118.4189	0.05717		0.727477466	0.908612163
240.3365	267.545	227.4062	-0.03038		0.587719544	0.936008754
969.387	1109.197	964.1185	0.003454		0.724149904	0.990142758
28343.63	28272.45	28948.71	-1.02864		0.690439171	5.57785E-29
919.3541	897.3906	1008.133	0.079099		0.462276826	0.753881265
311.812	363.2295	312.2906	0.16143		0.398196147	0.626416386
1234.74	1340.512	1248.114	-0.11496		0.207698921	0.514958257
686.1652	588.9706	600.4782	-0.01735		0.766887678	0.953132992
12457.29	12438.99	12098.64	0.466045		0.054030267	8.80507E-07
1216.871	1335.867	1198.86	0.223319		0.968417488	0.278856737
842.5179	885.3139	906.481	0.206436		0.786476399	0.192142371
33.95088	28.79825	29.34274	0.024836		0.98843748	NA
2.680333	1.857952	5.239774	0.702943		0.295061709	NA
145.6314	159.7838	141.4739	0.293276		0.831484164	0.424955236
670.0832	723.6721	732.5205	0.070091		0.741406787	0.79799583
952.4116	961.4899	880.2821	0.150975		0.644250773	0.372550259
355.5908	368.8034	407.6545	0.138122		0.735508761	0.693887308
9.827887	7.431806	11.5275	0.531522		0.834159161	NA

1483.117	1537.455	1629.57	-0.12149	0.35165001	0.495076977
3006.44	2856.6	2883.972	0.112238	0.098330299	0.379367723
452.9762	402.2465	438.0451	-0.18647	0.02867794	0.375516452
426.1729	414.3232	463.1961	-0.13444	0.872654096	0.59853578
1861.938	2147.792	1955.484	0.007901	0.577578732	0.971161723
517.3042	499.789	527.1213	0.010777	0.882395512	0.961026715
132.2298	111.4771	111.0832	0.413607	0.042480283	0.303824089
1544.765	1515.159	1732.269	-0.33431	0.005683428	0.073757535
708.5013	660.5018	756.6234	-0.13103	0.80050738	0.492537442
205.4922	143.9912	198.0635	0.158008	0.720425175	0.685537269
1813.692	1904.4	2079.143	-0.06897	0.822851767	0.789404047
737.0915	750.6124	694.7941	0.393229	0.317414996	0.004483801
1509.921	1319.146	1388.54	0.059635	0.990604058	0.732897571
26154.69	24226.76	25809.03	0.002457	0.903271991	0.990142758
808.5671	763.6181	741.9521	0.100343	0.945870306	0.596499376
1.786889	0	1.047955	-2.44032	0.458486853	NA
1309.789	1257.833	1170.566	0.04879	0.927916216	0.835596295
880.936	928.9758	913.8167	0.140736	0.002469991	0.394044776
475.3123	484.9253	501.9704	-0.54848	0.236209282	1.09507E-05
0	8.360782	4.19182	0.420378	NA	NA
630.7716	582.4678	634.0127	0.013376	0.960823113	0.976136838
436.8942	461.701	463.1961	-0.283	0.66770133	0.20379563
377.0335	391.0988	438.0451	0.048437	0.812875146	0.875604624
203.7053	191.369	257.7969	-0.05476	0.494492788	0.896670492
2193.406	2147.792	2375.714	-0.07677	0.856745942	0.66352118
364.5253	350.2239	301.811	-0.14725	0.841901206	0.594065295
2.680333	10.21873	5.239774	0.056489	NA	NA
425.2795	419.897	445.3808	-0.03201	0.037054401	0.91372954
249.2709	362.3005	285.0437	0.04247	0.037913264	0.919915102
771.0424	791.4873	795.3978	0.209625	0.373700455	0.34519405
1560.847	1508.657	1624.33	-0.24555	0.729363614	0.092021996
625.411	606.6212	684.3145	0.099147	0.016778454	0.725549438
2314.914	2421.84	2538.147	-0.07505	0.350490225	0.659338327
7478.128	7287.815	8381.543	-0.30839	2.46088E-31	0.022078636
1874.446	1842.159	1868.504	0.176298	0.412070549	0.158921436
55.39354	39.94596	62.87729	0.331067	0.952783825	NA
877.3623	837.0072	869.8026	0.033424	0.869592661	0.864363898
809.4605	810.0669	860.371	-0.03272	0.751619042	0.872582499
39.31155	18.57952	19.91114	-0.03409	0.582750031	0.964166603
899.6984	914.1121	892.8576	0.21665	0.972861407	0.162129177
59.86077	60.38342	74.4048	-0.06457	0.645010824	0.945263709
683.4849	647.4961	671.7391	0.045129	0.992511525	0.835181175
1038.182	1085.044	1017.564	-0.12126	0.484625827	0.410479572
882.7229	817.4987	929.536	0.110276	0.730407446	0.630318178
534.2797	650.283	575.3272	-0.00444	0.626568234	0.997083933
1092.682	941.0524	846.7476	0.183779	0.798030968	0.468420234
170.6479	207.1616	256.7489	-0.59608	0.851080991	0.016996857
2339.037	2160.798	2044.56	0.131585	0.249636644	0.365253762
3.573777	1.857952	2.09591	-2.14988	0.93284858	NA
583.4191	573.178	526.0734	0.026602	0.91441696	0.914949396
2112.996	2208.175	2761.361	-0.51994	0.827268091	0.001530525
1524.216	1463.137	1526.87	-0.05383	0.117208522	0.817977011
1657.339	1665.654	1671.488	0.292446	0.579600128	0.054060197
280.5415	323.2836	276.6601	-0.02649	0.885575053	0.946922147

623.6241	756.1863	556.464	0.186157	0.819879878		0.477288843
68.79521	54.80957	75.45275	-0.34298	0.981023664		0.664575474
134.9101	134.7015	127.8505	0.273458	0.000238806		0.417522611
647.7471	573.178	609.9097	0.034481	0.976681492		0.883471187
208.1725	159.7838	228.4542	-0.41174	0.049453408		0.149449076
10791.91	10891.31	9853.92	-0.02197	0.071548865		0.885423648
3325.4	3287.645	3521.128	-0.08951	0.310443697		0.480764167
326.1072	326.9995	320.6742	0.237562	0.804201321		0.550627492
1547.445	1594.122	1505.911	-0.00161	0.327341		0.996720771
377.0335	376.2352	335.3456	0.086462	0.38025264		0.748270161
109.0002	89.18167	166.6248	0.088654	0.752974908		0.877477421
2124.61	2038.173	2075.999	0.355769	0.000788705		0.000832494
645.0668	630.7745	527.1213	0.055171	0.303314321		0.818409186
577.165	445.9084	433.8533	0.323628	0.988049891		0.420331471
1638.577	1639.642	1462.945	0.12351	0.98409529		0.471293277
1823.52	1909.974	1668.344	0.209892	0.174573346		0.361564535
1348.207	1339.583	1206.196	0.008128	0.947037414		0.97680656
42.88532	53.88059	57.63752	-0.38627	0.761936154		0.459373691
209.9594	296.3433	318.5783	0.015762	0.617661583		0.974531425
1320.511	1302.424	1361.293	-0.05938	0.128466875		0.724404377
39.31155	52.95162	42.96615	0.503065	0.928104954	NA	
680.8045	761.7601	629.8209	0.088338	0.872444605		0.697184019
95.59854	75.24704	93.26799	-0.18722	0.185152106		0.689321642
76.83621	96.61348	59.73343	0.073752	0.752974908		0.908612163
519.0911	627.9876	529.2172	-0.09479	0.248593884		0.728985289
3987.442	3986.235	4312.334	0.018103	0.730407446		0.921114792
3442.441	3378.685	3523.224	0.01998	0.349551263		0.906395988
559.2961	486.7833	507.2102	-0.12422	0.126687185		0.617517934
109.0002	133.7725	115.275	-0.45194	0.922265371		0.133640012
4437.738	4556.626	4046.154	-0.03596	0.023980578		0.825078673
343.0826	446.8373	482.0593	-0.39367	0.192723763		0.110990341
1122.166	994.0041	1110.832	0.084773	0.774889037		0.679997772
39.31155	59.45445	23.05501	-0.80914	0.051957782		0.193779401
691.5259	705.0926	653.9239	0.385788	0.847532334		0.045393537
8218.794	8283.677	8518.825	-0.14528	0.861070915		0.207154991
870.2147	755.2573	820.5487	0.067419	0.878427603		0.804059337
4748.656	4774.006	4534.501	0.045726	0.744600754		0.817988115
622.7306	706.0216	670.6911	0.080624	0.476445149		0.730853476
817.5015	850.9418	919.0564	-0.53975	0.00049622		2.72524E-06
309.1317	276.8348	341.6333	-0.5112	0.192952947		0.063993489
73.26243	76.17601	80.69253	0.150512	0.829098381		0.818869867
3471.924	3567.267	4231.642	-0.11179	0.87547961		0.611282744
3978.507	3869.184	4063.969	0.502341	3.08372E-07		0.000660612
1599.265	1674.943	1511.151	0.193362	0.227706608		0.152398809
834.4769	876.0241	779.6784	0.031795	0.430461737		0.87383525
20509.91	20377.08	21716.77	-0.10404	0.92790798		0.324570054
569.124	487.7123	503.0183	0.178998	0.848509848		0.41069386
86.66409	114.264	143.5698	-0.12989	0.149675596		0.840777068
756.7473	615.9109	617.2454	-0.02185	0.274927458		0.94960537
314.4924	384.596	322.7701	0.240236	0.089121906		0.380264685
5038.132	5104.722	5675.724	-0.25114	0.709602489		0.169907253
467.2713	416.1811	448.5247	0.076319	0.981637588		0.789143179
7427.202	7248.798	6842.098	0.08231	0.072132871		0.656621892
1277.625	1321.004	1181.045	0.184911	0.991136341		0.241619328

64.32799	72.46011	84.88435	0.134304	0.035363713	0.832900164
1347.314	1350.731	1237.635	0.164608	0.614621507	0.398655382
90.23787	105.9032	50.30183	-0.12604	0.544702272	0.856558496
687.9521	684.6551	646.5882	-0.08468	0.450176533	0.701843587
2356.013	2203.531	2453.262	-0.31973	0.837998472	0.139776747
106.3199	83.60782	63.92525	-0.09061	0.716459644	0.890884082
663.8291	704.1636	698.9859	0.270843	0.140279845	0.093593
13903.78	14030.32	12770.38	-0.03859	0.136068446	0.833950744
472.632	416.1811	517.6897	0.083075	0.88874568	0.798420235
1079.281	977.2825	963.0705	0.159635	0.475821599	0.361351632
199.2381	205.3036	210.6389	0.11113	0.367802825	0.782080618
276.9677	277.7638	291.3315	-0.04667	0.223467289	0.901090975
3437.08	3239.338	3478.162	-0.29061	0.825797061	0.002978476
1363.396	1382.316	1212.484	0.209319	0.811082292	0.24233535
3052.006	3238.41	3049.549	-0.43071	0.004389074	2.63712E-07
353.8039	302.8461	308.0987	0.336718	0.215371475	0.359290838
26.80333	33.44313	63.92525	0.300852	0.960823113	NA
977.428	1040.453	981.9337	-0.36654	0.17431357	0.013172557
0.893444	4.644879	1.047955	-0.26276	0.969607454	NA
8798.639	9064.945	8188.72	0.058826	0.060765203	0.71266909
531.5993	553.6696	506.1622	-0.14819	0.30979442	0.511081603
295.73	311.2069	316.4824	-0.19065	0.992476114	0.536590083
440.468	459.843	422.3258	-0.05546	0.72676049	0.818945755
1088.215	941.0524	1013.372	0.021012	0.93585746	0.919915102
1259.756	1218.816	1020.708	-0.05479	0.78608533	0.806564543
1175.773	1127.777	1167.422	-0.02448	0.944990108	0.892441232
664.7225	752.4704	778.6305	-0.26918	0.200422675	0.09995667
59155.84	58722.42	52143.09	-0.13142	0.553033696	0.443898537
1148.969	1173.296	978.7899	0.264505	0.410367179	0.240898563
631.6651	649.3541	592.0945	-0.26906	0.434274199	0.064858857
330.5744	320.4966	362.5924	-0.04642	0.936346196	0.907473183
11.61478	8.360782	4.19182	-0.14169	NA	NA
263.5661	291.6984	215.8787	0.167306	0.842806932	0.702040936
832.69	886.2429	904.3851	-0.18272	0.226842122	0.253751822
2409.619	2246.263	2515.092	-0.54164	5.56055E-14	6.66086E-08
96.49198	75.24704	73.35684	0.036308	0.577578732	0.949335284
4570.861	4406.132	4135.23	0.128494	0.16710022	0.443898537
47.35255	52.95162	64.9732	-0.29975	0.106548765	0.712929046
1358.035	1354.447	1310.992	0.13674	0.693771149	0.442655501
875.5754	803.564	902.2892	0.239534	0.600600887	0.238605598
101.8526	139.3464	116.323	-0.11337	0.354108154	0.832306222
59.86077	71.53113	71.26093	-0.11208	0.927256678	0.844024501
3315.572	3492.949	3240.277	0.101129	0.099797905	0.402824474
340.4023	337.2182	459.0042	0.100521	0.988049891	0.774877994
4291.213	4184.107	4366.828	-0.12294	0.035589933	0.333056689
2.680333	2.786927	5.239774	0.40923	NA	NA
471.7386	450.5532	452.7165	-0.59117	0.035776598	3.1943E-06
827.3294	741.3227	624.5811	0.214195	0.260444304	0.386225205
0	0	0	#DIV/0!	NA	NA
2829.538	2681.024	2683.812	-0.17618	0.157365929	0.184672738
1310.683	1316.359	1411.595	-0.14233	0.946929815	0.417522611
0	0.928976	0	-0.86025	NA	NA
1630.536	1517.017	1269.073	0.18615	0.981160904	0.437727687
802.3129	852.7997	780.7264	0.052613	0.86616202	0.814580971

30.3771	25.08235	41.9182	-1.18197	0.018765686	0.010292932
101.8526	69.67318	56.58956	-0.23745	0.92790798	0.722021255
163.5003	215.5224	173.9605	0.490535	0.817609984	0.088412059
2685.693	2802.72	3139.673	-0.29888	0.572336545	0.012748693
2088.873	2030.741	1823.442	-0.11984	0.000661553	0.400098164
1546.552	1534.668	1408.451	-0.01836	0.731285248	0.947221243
594.1404	592.6865	589.9986	0.069122	0.258431804	0.735679134
2675.866	2701.462	2766.601	-0.15028	0.894462417	0.280613898
2001.315	1812.432	1960.724	-0.11099	0.582750031	0.515558505
1578.716	1721.392	1497.528	0.357369	0.311697167	0.075363288
1771.7	1730.682	1955.484	-0.3408	0.455280748	0.001662103
3.573777	0	3.143865	-0.79359	0.434274199	NA
986.3625	914.1121	943.1594	-0.24886	0.787792061	0.110349167
55.39354	68.74421	71.26093	-0.61408	0.043426903	0.092015479
1036.395	1050.672	1400.068	-0.12391	0.028028845	0.627660382
687.0586	743.1806	669.6432	0.305989	0.565330117	0.048489294
1319.617	1283.845	1198.86	0.308489	0.871801928	0.01955739
1034.608	1032.092	873.9944	0.232766	0.992476114	0.186995116
195.6643	209.9485	181.2962	0.072183	0.843507519	0.862412114
2056.709	2058.61	1778.379	0.141923	0.103085653	0.626606102
899.6984	943.8394	818.4528	0.240137	0.148004742	0.408058591
600.3945	564.8173	479.9633	-0.11816	0.703609106	0.639831259
697.78	600.1183	572.1834	0.168859	0.957754117	0.502156977
894.3377	1076.683	1026.996	-0.19976	0.735300868	0.25282015
1077.494	1034.879	925.3442	0.235878	0.759261525	0.375516452
1056.051	1018.157	1055.291	-0.07629	0.828690815	0.697184019
1544.765	1572.756	1698.735	-0.09349	0.859748112	0.673302041
3605.048	3456.719	3590.293	-0.08519	0.034815958	0.497130933
1148.969	888.1008	1047.955	0.055962	0.639350783	0.825748549
1297.281	1419.475	1120.264	0.164016	0.770051929	0.467088769
2999.292	2829.66	2954.185	0.121863	0.008233634	0.430182989
368.9925	398.5306	376.2158	0.118006	0.298622885	0.616511903
115.2543	116.122	130.9944	0.269166	0.229874823	0.61918051
0.893444	4.644879	2.09591	-0.74023	0.962556121	NA
460.1238	470.9907	520.8336	-0.17701	0.228243156	0.436785507
664.7225	800.7771	863.5148	-0.43664	0.787835013	0.014438677
432.427	368.8034	361.5444	-0.00739	0.915659837	0.979801623
1660.019	1645.216	1568.788	-0.02459	0.756571209	0.905975684
109.8936	98.47143	110.0353	-0.11357	0.335666342	0.829566409
1147.182	1144.498	1113.976	-0.00118	0.847714103	0.99985654
201.9184	282.4086	234.7419	0.10702	0.051273953	0.778199039
274.2874	276.8348	282.9478	0.468174	0.06343049	0.112852596
612.9028	604.7632	606.7659	-0.58722	0.639085319	3.93255E-05
390.4351	379.0221	402.4147	-0.01654	0.72742168	0.962321489
22763.17	21942.41	25660.22	-0.04305	0.000126563	0.832033519
1209.724	1262.478	1165.326	0.000913	0.397105869	0.99985654
435.1074	452.4112	379.3597	0.089737	0.626260019	0.775439768
333.2547	308.42	346.8731	-0.30484	0.455280748	0.282666674
192.0905	212.7354	300.7631	-0.4717	0.262290046	0.061045315
2593.669	2359.598	2421.824	0.06877	0.011540931	0.74730223
1128.42	1037.666	1057.386	0.11074	0.506703303	0.638725554
1282.093	1296.85	1176.853	0.279286	0.413714744	0.191769337
1376.798	1316.359	1252.306	0.069435	0.714180196	0.754367859
503.0091	425.4709	499.8745	-0.65669	0.287050142	2.9108E-06

1289.24	1436.197	1096.161	0.089799	0.527593235	0.728406381
228.7217	209.0195	217.9746	-0.06821	0.324651337	0.865246562
159.0331	210.8775	208.543	0.017203	0.235856061	0.974691849
3552.334	3470.653	3726.528	-0.04825	0.990735591	0.753881265
3019.842	3116.714	2878.732	0.118565	0.262338145	0.37942314
1329.445	1402.753	1111.88	0.348071	0.732140966	0.11799835
718.3292	732.0329	845.6996	0.113157	0.936346196	0.565559013
7640.735	7188.414	9008.22	-0.13129	0.369977802	0.456678882
300.1973	284.2666	221.1185	0.054144	0.274927458	0.887908388
712.9685	666.0756	581.615	0.189717	0.670790001	0.427452223
0	0	0	#DIV/0!	NA	NA
121.5084	141.2043	117.3709	0.900311	0.854862911	0.006858707
10228.15	10568.96	11869.14	-0.27963	0.867574132	0.064858857
368.099	365.0875	379.3597	0.028391	0.637753032	0.951624375
3402.236	3165.02	3519.033	0.107929	0.948896953	0.547918323
14982.17	14246.77	15576.8	-0.06322	0.553033696	0.74477658
1476.863	1496.58	1405.308	0.012253	0.720966293	0.941411168
2372.988	2406.976	2473.174	-0.02302	0.58004353	0.91107822
9.827887	13.00566	4.19182	0.261207	0.827268091	NA
325.2137	370.6613	380.4076	-0.38678	0.839737561	0.055523327
482.4599	470.0617	385.6474	0.065383	0.572219847	0.843531118
1291.92	1448.273	1473.425	0.074595	0.749431368	0.753881265
138.4839	125.4117	144.6178	0.015169	0.018619821	0.975406172
334.1482	287.9825	321.7222	-0.01736	0.182596799	0.96505606
2086.192	2188.667	1881.079	0.054077	0.786445338	0.86344732
2619.579	2524.956	2416.584	-0.0211	3.02032E-05	0.904467867
657.575	639.1353	576.3752	-0.10507	0.59668269	0.606467508
251.0578	280.5507	273.5162	0.036579	0.981023664	0.923725803
1146.289	1218.816	1121.312	0.122217	0.832043227	0.5847566
1000.658	950.3422	1129.695	-0.23524	0.256943324	0.124254462
0	0	20.9591	1.67032	NA	NA
1748.47	1729.753	1649.481	0.303964	0.088334441	0.018025328
407.4106	444.0504	387.7433	-0.31178	0.417038094	0.162970478
10295.16	10150.92	10361.13	-0.37322	0.434168259	1.53522E-08
3395.982	3350.816	3653.171	-0.25729	0.804201321	0.092971431
2639.234	2613.209	2609.408	0.082389	0.262015581	0.569740265
2563.292	2584.411	2240.528	-0.07295	0.434274199	0.744396459
1148.076	1164.936	1290.032	0.001108	0.648891486	0.995091298
643.2799	603.8342	559.6079	0.095056	0.98843748	0.68408594
1256.183	1402.753	1348.718	0.271528	0.252345272	0.128665301
1336.593	1319.146	1320.423	0.436921	0.467784161	0.00712557
592.3535	578.7519	504.0663	0.132089	0.832298202	0.681228083
952.4116	1033.021	937.9196	-0.2887	0.270719269	0.016996857
0.893444	0	0	#DIV/0!	NA	NA
540.5338	607.5501	652.8759	-0.08678	0.906767387	0.699804545
1669.847	1712.102	1641.097	0.202037	9.14127E-07	0.371661626
2239.865	2205.388	2058.183	-0.18593	0.152084817	0.227798107
1131.1	1112.913	1247.066	-0.12628	0.524923966	0.535066149
11676.42	11770.12	12348.05	-0.15512	0.901380807	0.176169028
1835.135	1751.119	1903.086	-0.00335	0.853331381	0.992528568
20.54922	23.22439	33.53456	0.079917	0.73478662	NA
278.7546	254.5394	253.6051	0.285703	0.763001611	0.376918607
1834.241	1964.784	1790.955	-0.12245	0.317073718	0.398890652
2760.743	2777.638	2706.867	0.076712	0.446999299	0.599924988

1203.469	955.9161	922.2003	-0.21619	0.886888038	0.318625647
1.786889	0	0	-1.55981	NA	NA
47.35255	47.37776	32.4866	0.028111	0.331331472	0.990023409
450.2959	331.6443	466.3399	-0.03908	0.924284267	0.914772543
4286.746	4358.754	4583.755	-0.39024	0.430461737	3.62177E-05
195.6643	158.8549	212.7348	-0.83854	0.868137167	0.001351995
3614.875	3623.934	3502.265	0.017251	0.981711158	0.922071247
1452.74	1422.262	1174.757	0.174925	0.33985187	0.437727687
1032.822	933.6206	1031.188	-0.06577	0.981637588	0.703203936
2336.357	2181.235	2232.144	-0.1072	0.034700814	0.515558505
1924.479	1638.713	1955.484	-0.07568	0.364654282	0.74872051
1043.543	1147.285	1043.763	0.218028	0.934554042	0.159147544
1238.314	1281.987	1241.827	0.135505	0.004283832	0.436738287
536.0666	539.7349	597.3343	0.010082	0.485402441	0.97680656
1912.864	1684.233	1782.571	-0.05627	0.517968435	0.806564543
6.25411	3.715903	10.47955	0.410404	0.84819607	NA
3905.245	3731.696	3550.471	0.171339	0.323145934	0.258978375
1165.051	1275.484	976.694	0.087675	0.947037414	0.753167659
1082.854	973.5666	1108.736	-0.14228	0.724349652	0.394044776
535.1731	575.965	499.8745	0.207937	0.308685572	0.545985077
150.9921	136.5594	181.2962	-0.05453	0.614621507	0.896421455
0	0	0	#DIV/0!	NA	NA
0	3.715903	14.67137	-0.45378	0.682713002	NA
792.4851	819.3566	778.6305	-0.15716	0.270719269	0.477876695
1243.674	1190.018	1338.238	-0.00714	0.926431363	0.983469507
1515.281	1371.168	1610.707	-0.02207	0.052845299	0.922101817
102.7461	48.30674	76.50071	-0.54014	0.637486498	0.210822498
4191.147	4002.028	3742.247	0.029602	0.00016523	0.87134989
3149.391	2841.737	3123.954	0.166339	0.668522148	0.247115917
67.00832	74.31806	77.54866	-0.45163	0.948234757	0.275261425
283.2218	282.4086	283.9958	0.347893	0.94035236	0.174836857
1308.002	1308.927	1056.339	0.147929	0.363948776	0.609093663
12.50822	5.573855	5.239774	1.824425	NA	NA
0	0	0	#NUM!	NA	NA
461.9107	475.6356	442.237	0.100533	0.387883832	0.69424407
2043.307	2079.977	2138.876	-0.12314	0.832298202	0.378819077
276.0743	256.3973	230.5501	-0.1117	0.230402467	0.719971458
1215.978	1178.87	976.694	0.133606	0.764395772	0.690294956
1163.264	1104.552	1169.518	-0.07802	0.804410815	0.71621184
1385.732	1378.6	1233.443	0.045413	0.555061446	0.80030609
33.05744	29.72722	18.86319	0.721044	0.537991135	NA
1044.436	1142.64	1037.475	0.301971	0.344558327	0.06712239
461.9107	466.3458	571.1354	-0.09798	0.635191985	0.732745416
4386.811	4385.695	4312.334	-0.26369	0.870263501	0.025807779
14595.31	14324.81	13116.2	0.100259	0.115777882	0.502754884
0.893444	0	0	-2.14477	NA	NA
1668.954	1545.816	1825.537	-0.12849	0.463993613	0.394829397
197.4512	172.7895	141.4739	0.201783	0.90469595	0.634014533
334.1482	354.8687	304.9549	0.086985	0.576613335	0.786706509
1711.839	1708.386	1780.475	0.032355	0.501719382	0.859010941
280.5415	229.457	219.0226	-0.04532	0.962556121	0.922101817
117.9346	142.1333	144.6178	0.146672	0.37602059	0.698960269
3.573777	0	13.62341	1.517496	0.998829997	NA
829.1163	707.8795	652.8759	0.138326	0.792868376	0.610317713

929.182	1029.305	952.591	0.203433	0.778141653	0.268536077
1164.158	1151.93	1314.135	-0.18237	0.668522148	0.402824474
0	0	3.143865	-1.79444	0.82365137	NA
519.0911	587.1127	566.9436	-0.41884	0.005553951	0.00095673
920.2476	917.8281	1102.449	-0.0851	0.553033696	0.729565723
641.493	648.4251	645.5402	0.048655	0.430461737	0.87466351
194.7708	217.3803	204.3512	0.023224	0.022922256	0.973438605
313.5989	279.6217	335.3456	0.279144	0.14943639	0.267468464
1308.002	1308.927	1189.429	0.285067	0.208221128	0.239236051
16.97544	5.573855	23.05501	-0.57914	0.882057909	NA
522.6649	484.9253	447.4767	0.27977	0.730407446	0.251743462
979.2149	960.5609	1238.683	-0.13187	0.988049891	0.545985077
23.22955	28.79825	35.63047	-0.0166	0.433570568	0.961378287
0.893444	0.928976	0	0.468598	NA	NA
832.69	912.2542	904.3851	-0.00452	0.404785633	0.993537704
1234.74	1176.083	912.7687	0.019823	0.596065856	0.952588397
444.9352	552.7406	444.3329	0.379197	0.314509384	0.066436334
1361.609	1172.367	1240.779	-0.06144	0.064123971	0.822050288
629.8782	593.6155	672.787	0.199696	0.42970588	0.285602122
8.934443	3.715903	2.09591	#DIV/0!	NA	NA
1593.905	1584.833	1519.535	0.209592	0.875368742	0.287569887
770.149	838.8651	707.3696	0.437726	0.73146374	0.046523177
6.25411	6.50283	8.383639	1.419723	0.996453878	NA
2410.513	2513.808	2231.096	-0.11155	0.992476114	0.437811634
1899.462	1850.52	1855.928	-0.05659	0.6744135	0.773113821
460.1238	453.3402	675.9309	-0.25115	0.947527175	0.350427789
1708.265	1805.929	1941.86	-0.02733	0.565571713	0.899731862
35.73777	35.30108	18.86319	-1.01244	0.483188815	0.060630053
637.0258	596.4024	755.5755	-0.45247	0.089410503	0.002309437
1519.749	1524.449	1453.513	0.242163	0.406835177	0.099741709
406.5171	468.2038	511.402	-0.09926	0.826883467	0.764338813
9049.697	9375.223	10473.26	-0.50907	0.7784685	0.000804398
969.387	912.2542	962.0226	-0.12059	0.900789434	0.414781698
8.934443	18.57952	14.67137	-0.25437	0.817609984	NA
463.6976	533.2321	505.1143	-0.19248	0.861070915	0.437811634
2096.02	2042.818	1954.436	0.010128	0.6212524	0.962161209
555.7223	580.6099	666.4993	-0.13752	0.854046275	0.536619543
1917.331	1939.701	1795.147	0.031047	0.469683278	0.87444105
143.8445	183.9372	171.8646	-0.30636	0.713210135	0.262359598
1095.363	1221.603	1113.976	0.203759	0.157331056	0.206562409
5039.919	4970.949	4691.694	0.170852	0.112064101	0.370304803
1978.086	2060.468	2061.327	-0.01754	0.226105917	0.93327699
4900.542	4702.475	4902.333	-0.0064	0.695990097	0.975406172
60.75421	41.80391	37.72638	0.55674	0.762033914	NA
3714.048	3907.272	3699.281	-0.01664	0.310443697	0.919915102
1068.559	1183.515	1371.773	-0.47614	0.494358204	0.001567387
0	0	0	#DIV/0!	NA	NA
1150.756	1134.279	987.1735	0.059486	0.68066159	0.828926206
1826.2	1674.014	1688.255	0.233823	0.225117575	0.183923086
3983.868	3870.113	4393.027	-0.05843	0.829098381	0.748961137
159.0331	139.3464	144.6178	0.119984	0.948234757	0.809142313
2018.291	1909.974	2087.526	-0.5137	0.007236622	1.46636E-09
365.4187	377.1642	355.2567	-0.07399	0.960773888	0.774877994
89.34443	68.74421	98.50776	-0.82914	0.687155674	0.007856475

526.2387	605.6922	771.2948	-0.73515	0.331795127	8.43329E-05
1111.445	1159.362	1251.258	0.079216	0.826883467	0.733968665
186.7298	172.7895	164.5289	0.270079	0.817060109	0.436772875
2509.685	2419.053	2724.683	0.092899	0.183178382	0.54110554
793.3785	802.6351	678.0268	0.0147	0.67324111	0.967817837
229.6152	168.1446	151.9535	0.573292	0.539220495	0.127945669
796.9523	815.6407	804.8294	-0.24676	0.00878771	0.162954416
224.2545	220.1673	192.8237	-0.27514	0.298839192	0.407575574
13.40166	26.01132	54.49365	0.377415	0.562248048	0.685537269
441.3615	334.4313	290.2835	-0.26346	0.794232621	0.410222128
868.4278	944.7683	956.7828	-0.47748	0.008720893	0.000100604
2307.767	2438.561	2337.987	-0.0315	0.780980494	0.877477421
2618.685	2626.214	2208.041	0.533092	0.205372066	0.20379563
3459.416	3357.318	3270.667	-0.06184	0.115981316	0.627713216
1910.184	2004.73	1903.086	-0.16675	3.09589E-05	0.109807224
466.3779	467.2748	545.9845	-0.38665	0.99647374	0.011552862
4560.139	4912.424	4706.365	-0.05942	0.993774385	0.74588273
1615.347	1659.151	1341.382	0.086718	0.655427675	0.775439768
14.29511	9.289758	18.86319	-0.31464	0.847096689 NA	
2281.857	2214.678	1963.867	0.124003	0.78204194	0.536590083
989.9362	876.9531	933.7278	0.141634	0.729364673	0.545985077
1373.224	1172.367	1450.37	0.073215	0.828016939	0.742127697
651.3209	707.8795	567.9916	0.268181	0.038048538	0.278856737
1616.241	1602.483	1539.446	0.029927	0.008686831	0.878502464
2045.987	2111.562	1873.743	-0.05631	0.836240002	0.803458765
11613.88	10647.92	9872.783	0.220832	0.02597477	0.384133344
913.9935	944.7683	853.0353	-0.04752	0.592978918	0.823966789
1017.633	1067.393	1021.756	-0.13873	0.045913153	0.402824474
934.5427	943.8394	760.8153	0.07973	0.666270528	0.783107724
645.9602	484.9253	495.6827	0.366196	0.910726255	0.142474764
1799.397	1735.327	1571.932	0.19938	0.310114043	0.350867993
1324.978	1252.259	1356.054	0.349397	0.13366062	0.190817982
3293.236	3306.225	4095.408	-0.64995	0.494209809	5.09008E-06
64.32799	46.44879	55.54161	0.185969	0.668508083	0.82132239
682.5914	660.5018	641.3484	0.195432	0.549290634	0.251743462
3673.843	3418.631	3239.229	0.211895	0.008454108	0.181725119
57.18043	87.32372	88.02821	-0.73457	7.08114E-06	0.020139549
1064.986	1029.305	991.3653	0.255084	0.842806932	0.219264296
1505.454	1438.983	1474.473	0.139576	0.045913153	0.41822825
444.9352	421.755	417.086	-0.0341	0.619820968	0.914120833
814.8212	798.9192	666.4993	0.369389	0.718622163	0.098479436
622.7306	704.1636	594.1904	-0.08858	0.564346914	0.690294956
3024.309	3072.123	3179.495	-0.27759	0.011304032	0.000732015
1093.576	1030.234	875.0423	0.113362	0.907472172	0.656368654
1928.053	1745.545	1751.133	0.15264	0.143062759	0.243388227
1741.323	1731.611	1651.577	0.129195	0.310443697	0.535221009
3244.99	3239.338	3458.251	-0.22884	0.434274199	0.064954005
1750.257	1781.776	1994.258	-0.0358	0.868137167	0.846177331
2534.701	2679.166	2337.987	0.136348	0.379440934	0.433632804
1031.035	1078.541	869.8026	0.143886	0.856763291	0.525178886
585.206	602.9053	570.0875	0.352374	0.500344775	0.051038786
1243.674	1157.504	1155.894	0.081386	0.113562986	0.686433182
740.6653	761.7601	737.7602	0.233352	0.638519612	0.306279885
1055.158	1079.47	902.2892	0.028166	0.396688154	0.923725803

410.9844	517.4395	511.402	-0.42717	0.798105246	0.033509815
2574.906	2326.155	2496.229	0.443743	0.376884278	0.000572062
573.5912	588.0417	668.5952	-0.11006	0.904254847	0.625382385
41.09844	52.02264	48.20593	-0.55737	0.261514221	0.205726872
1499.199	1671.227	1563.549	0.127221	0.766253365	0.450085983
627.1979	630.7745	661.2595	0.087076	0.654926306	0.66000496
305.5579	376.2352	364.6883	0.21776	0.759261525	0.437727687
1548.339	1510.515	1781.523	0.039742	0.697514819	0.889915228
2132.651	2131.999	1896.798	-0.18515	0.000532839	0.309248984
0	0	0	#DIV/0!	NA	NA
1707.372	1775.273	1769.996	-0.09908	0.002387022	0.515285838
508.3698	465.4169	515.5938	0.128891	0.573024726	0.575546698
402.9434	449.6243	418.134	-0.05181	0.626260019	0.86814025
1546.552	1739.043	1712.358	-0.06869	0.982280934	0.728253469
856.813	901.1065	979.8378	-0.36135	0.6744135	0.00861945
6377.405	5717.846	6959.468	0.029226	0.451912698	0.89451867
562.8699	493.2861	538.6488	-0.34469	0.67324111	0.066594503
2626.726	2711.68	2514.044	0.013613	0.071245169	0.947221243
400.263	394.8147	450.6206	-0.49957	0.490813332	0.007994297
410.0909	403.1755	406.6065	-0.08758	0.430461737	0.799909776
1.786889	0	2.09591	-1.51098	0.6333028	NA
1.786889	0	0	-3.27372	0.870947007	NA
46.4591	26.01132	36.67842	-0.03692	0.586117995	NA
3148.498	3014.526	2789.656	0.171246	0.246058895	0.260773478
945.264	925.2599	860.371	0.033061	0.871801928	0.89969904
6580.217	6470.316	6610.499	-0.16854	0.825797061	0.210414951
1394.666	1387.89	1406.355	0.020455	0.122329881	0.923725803
2005.782	2047.463	1755.324	0.14362	0.570003668	0.61918051
661.1488	686.5131	659.1636	0.140832	0.673772903	0.510445084
2976.063	2938.35	3219.317	-0.04592	0.352900476	0.81253834
984.5756	881.598	855.1312	-0.01645	0.776530046	0.953528509
4291.213	4336.459	3962.317	0.166065	0.651806624	0.269360139
552.1486	577.8229	659.1636	0.018232	0.957987619	0.946922147
1569.782	1405.54	1323.567	0.276116	0.247855038	0.144968034
9780.534	9633.479	9673.672	-0.23739	0.226056492	0.060802767
869.3213	881.598	701.0818	0.176056	0.563625743	0.529024216
1018.526	956.845	964.1185	0.250147	0.653304895	0.198030129
2115.676	2387.468	2204.897	0.163687	0.959859312	0.268382708
1396.453	1418.546	1381.205	-0.1329	0.48612699	0.361214706
888.977	873.2372	819.5007	0.342368	0.73478662	0.013177469
175.1151	206.2326	270.3724	-0.07301	0.842190796	0.855100406
1073.027	1025.589	1002.893	0.035268	0.831143588	0.850669379
932.7558	1064.606	1039.571	-0.14997	0.934554042	0.352934781
1014.059	1106.41	926.3921	-0.05807	0.140032454	0.822780332
968.4936	1103.623	940.0155	0.04183	0.925903677	0.864363898
487.8206	467.2748	448.5247	-0.01191	0.995526989	0.965791283
154.5659	192.298	212.7348	-0.1612	0.682715357	0.651302885
832.69	818.4276	899.1453	0.135062	0.231422023	0.470568982
386.8614	360.4426	391.9351	0.147279	0.252668387	0.608073878
963.1329	915.9701	1082.537	0.017909	0.865404007	0.941511899
1643.044	1655.435	1753.229	-0.10722	0.208221128	0.520301247
1263.33	1127.777	1219.819	-0.01889	0.803184945	0.92540057
337.7219	314.9228	314.3865	0.433633	0.602494298	0.060691739
8.934443	34.3721	19.91114	-0.24511	0.831143588	NA

75.94276	66.88625	97.45981	-0.01401	0.014661969	0.984451848
1593.905	1557.892	1826.585	0.224948	0.456240675	0.097879485
12.50822	25.08235	16.76728	-0.15411	0.249012381	NA
235.8693	235.0309	313.3385	-0.38447	0.465047544	0.12612237
313.5989	321.4256	230.5501	0.357095	0.856680478	0.355134703
35.73777	51.09367	73.35684	0.266759	0.716971101	0.68408594
327.894	352.0818	398.2229	-0.50956	0.188880644	0.005141611
167.0741	157.9259	212.7348	-0.06572	0.367802825	0.875604624
498.5419	502.5759	535.505	-0.18222	0.162901755	0.339186935
788.0178	732.0329	714.7052	0.046244	0.766887678	0.838059844
438.6811	357.6557	358.4006	0.001601	0.022968717	0.992528568
1026.567	1023.731	1076.25	0.251644	0.303915516	0.097199704
3.573777	1.857952	4.19182	0.218433	NA	NA
314.4924	330.7154	389.8392	-0.37278	0.67997963	0.06712239
13269.43	13116.21	14078.23	-0.33512	0.571061175	0.010351567
4155.409	4297.442	4347.965	-0.05828	0.417487982	0.7475811
2866.169	3115.785	3179.495	-0.02494	0.794232621	0.900914914
460.1238	424.5419	433.8533	0.105918	0.547416205	0.690294956
1069.453	1111.055	1101.401	0.226461	0.29215357	0.219723352
241.2299	211.8065	231.598	0.03262	0.791689312	0.929939946
852.3458	854.6577	920.1044	-0.07642	0.231538797	0.764793691
2240.758	2317.795	2058.183	0.147298	0.937354907	0.30760874
2322.062	2276.92	2361.042	0.031581	0.161427291	0.908612163
553.9354	561.1014	628.7729	-0.50534	0.1874606	0.001448138
6.25411	7.431806	17.81523	-0.1558	0.921503066	NA
276.0743	245.2496	292.3794	0.513646	0.872688807	0.096021406
539.6403	441.2635	440.1411	0.112526	0.380058107	0.751151284
280.5415	311.2069	310.1946	-0.40168	0.035767958	0.091639488
17892.11	17925.52	19990.79	-0.43578	0.787835013	0.001519825
3.573777	3.715903	2.09591	-1.16351	0.362417219	NA
1371.437	1502.154	1506.959	0.02995	0.757209202	0.875604624
1512.601	1740.901	1714.454	0.013665	0.696149523	0.953867552
17.86889	20.43747	25.15092	1.271165	0.327964932	NA
1534.937	1560.679	1269.073	0.295	0.178700298	0.167165088
500.3288	483.9964	537.6009	0.02551	0.835664498	0.929220289
550.3617	469.1328	508.2581	0.127415	0.662466622	0.583691606
49.13943	64.09933	57.63752	-0.4626	0.920564616	0.385354962
410.0909	369.7324	368.8801	0.212279	0.870816496	0.324099572
343.0826	419.897	369.9281	-0.17856	0.146490289	0.403397484
1494.732	1590.407	1366.533	0.161058	0.904254847	0.465657685
126.8691	143.9912	192.8237	-0.40796	0.025591463	0.162129177
902.3787	852.7997	869.8026	0.187811	0.477428994	0.194057829
641.493	570.3911	695.842	0.304224	0.986203512	0.193060221
2787.546	2920.7	2916.458	-0.04209	0.957987619	0.818945755
3731.917	3651.804	3732.815	-0.16292	0.897766774	0.317888314
469.9517	536.948	464.244	0.028906	0.460515819	0.931131803
973.8542	863.0185	1061.578	-0.08015	0.919200531	0.74872051
292.1563	329.7864	232.646	-0.48952	0.050382968	0.04967295
249.2709	268.474	205.3992	0.080303	0.462388123	0.835596295
1031.035	1093.404	1190.477	-0.00114	0.716459644	0.99985654
15001.82	14464.15	15654.35	-0.14078	0.665780513	0.178134034
620.9438	607.5501	591.0466	-0.00933	0.935506037	0.988943755
3002.866	3047.04	2713.155	0.179174	0.354108154	0.2912749
3348.629	3466.009	2757.169	0.07317	0.579415204	0.823637286

137.5904	138.4174	130.9944	-0.03026	0.476187241		0.967817837
1972.725	1903.471	1964.915	0.18152	0.759069408		0.078157207
514.6239	534.1611	462.1481	-0.03493	0.872444605		0.89969904
788.9113	842.581	696.89	0.22541	0.585115533		0.392881614
2408.726	2514.737	2978.288	0.142187	0.935523245		0.542274871
807.6736	855.5867	846.7476	-0.25962	0.001474651		0.066045956
484.2468	472.8487	429.6615	0.18076	0.98409529		0.465657685
25.01644	22.29542	22.00705	-0.12328	0.967317013	NA	
997.9772	824.0015	951.543	0.04631	0.769710962		0.864252513
352.017	317.7097	281.8999	0.047136	0.192384879		0.877477421
13.40166	7.431806	8.383639	-0.9999	0.948896953	NA	
7630.014	7651.044	8553.408	-0.43708	0.587719544		7.94614E-05
114.3609	120.7668	104.7955	-0.00215	0.871801928		0.997036378
8275.081	8069.083	7112.47	0.084535	0.026728815		0.718440886
930.9689	912.2542	900.1933	0.019745	0.572336545		0.938924382
1004.231	1197.45	1203.052	-0.17842	0.7406592		0.423533881
10.72133	21.36644	22.00705	-0.15881	0.875368742	NA	
1417.003	1495.651	1556.213	0.747378	0.509740526		1.89846E-06
46.4591	69.67318	32.4866	0.214495	0.834965572		0.757863278
1792.249	1757.622	1710.262	0.030682	0.652050398		0.872703049
962.2395	910.3962	904.3851	-0.07499	0.835449534		0.722021255
575.3781	601.0473	569.0395	-0.13597	0.870174956		0.582412098
98.27887	83.60782	96.41185	-0.06467	0.512918953		0.922101817
1425.937	1425.978	1425.219	-0.00147	0.182950095		0.99985654
296.6235	200.6588	245.2214	0.37202	0.325505488		0.271247752
2741.087	2803.649	2989.815	0.237361	0.9788673		0.156915071
291.2628	250.8235	313.3385	-0.19038	0.767697372		0.484464564
5167.682	5208.767	5099.349	0.006178	0.617661583		0.973599965
440.468	376.2352	368.8801	-0.28998	0.515985444		0.142939621
599.5011	675.3654	716.8011	0.287595	0.451912698		0.237500588
193.8774	160.7128	195.9676	-0.35433	0.307232061		0.129766751
191.1971	234.1019	348.969	-0.24175	0.917641799		0.519070487
1517.068	1542.1	1576.124	-0.03493	0.783200259		0.834432692
1431.298	1412.043	1346.622	0.211223	0.469683278		0.285769191
3697.072	3879.403	3976.989	0.194623	0.836962769		0.253693985
410.9844	418.0391	362.5924	-0.44437	0.288884373		0.017692756
520.878	471.9197	492.5388	0.072468	0.842165531		0.767228242
716.5423	742.2516	703.1777	-0.06416	0.956452433		0.737455632
15.18855	24.15337	11.5275	-0.48472	0.882494351	NA	
367.2056	382.738	311.2426	-0.28208	0.15308256		0.189524038
283.2218	254.5394	255.701	-0.1345	0.187619163		0.669189286
481.5665	577.8229	521.8815	0.165652	0.854862911		0.394829397
1059.625	1091.547	1122.36	0.01214	0.907551741		0.953867552
0	0	0	#DIV/0!	NA	NA	
76.83621	87.32372	103.7475	-0.16765	0.16816951		0.709172058
1419.683	1523.52	1549.925	-0.14811	0.622402254		0.36518668
742.4522	744.1096	750.3357	-0.03675	0.836327255		0.8721296
865.7475	821.2146	812.165	0.292712	0.886112674		0.083745142
373.4597	388.3119	399.2708	-0.07003	0.187509131		0.789143179
608.4355	629.8456	662.3075	0.126222	0.136068446		0.59372632
251.0578	258.2553	312.2906	0.040528	0.503721512		0.901090975
727.2636	728.317	825.7885	0.255045	0.228465218		0.136895784
6894.709	6798.245	5704.018	0.04943	0.094605412		0.859505388
4304.614	4226.84	4685.406	-0.12598	0.854862911		0.41069386

542.3207	482.1384	453.7645	0.226853	0.504834011	0.549888611
496.755	510.9367	430.7095	0.13975	0.363948776	0.549697898
2079.045	2081.835	1876.887	0.153622	0.231792531	0.554207814
4936.28	4916.14	4725.229	0.020324	0.842190796	0.919912874
83.09032	65.0283	30.39069	-0.11384	0.849658023	0.885571199
2554.357	2700.533	2388.289	0.170239	0.159784266	0.377946785
1370.543	1404.611	1225.059	-0.20418	0.509444319	0.244049419
4242.073	4559.413	4351.109	-0.00648	0.515985444	0.969852341
1602.839	1757.622	1790.955	0.133578	0.794232621	0.579998211
2686.587	2589.055	3002.391	-0.21446	0.90421043	0.231736184
3355.777	3313.657	3068.412	-0.02581	0.004275303	0.914120833
729.0505	774.7658	678.0268	-0.23854	0.944990108	0.200449188
1266.011	1291.276	1476.568	-0.01012	0.216768104	0.975406172
1743.11	1718.605	1605.467	0.198249	0.960823113	0.220390487
482.4599	516.5105	467.3879	0.004369	0.584647995	0.996316779
498.5419	504.4338	497.7786	0.030278	0.950721216	0.91372954
5798.453	5753.147	5704.018	-0.06558	0.937354907	0.593866078
25.01644	26.9403	16.76728	0.004701	0.730407446	NA
617.37	562.0303	726.2327	0.004315	0.44116367	0.996992829
843.4114	896.4616	853.0353	0.061699	0.394573102	0.803458765
424.386	443.1214	433.8533	-0.01091	0.901380807	0.975406172
117.0412	120.7668	106.8914	-0.08225	0.779325825	0.847133587
1465.249	1583.904	1698.735	-0.15196	0.00628885	0.542805648
2205.02	2057.681	2011.025	0.220743	0.43882432	0.140495882
34.84433	41.80391	59.73343	-0.90567	0.798439173	0.137586476
162.6069	167.2156	209.591	0.48431	0.084787806	0.232634698
1.786889	1.857952	7.335684	0.474655	0.960823113	NA
433.3205	426.3999	576.3752	0.616869	0.156929518	0.009798351
158.1396	206.2326	203.3032	0.30025	0.92288341	0.630318178
721.903	588.9706	693.7461	-0.34625	1.59768E-16	0.056918147
499.4353	434.7607	516.6418	-0.30145	0.186095147	0.238463934
244.8037	237.8178	204.3512	0.359778	0.747302666	0.253751822
50.92632	30.6562	31.43865	0.828362	0.855606042	NA
584.3125	573.178	597.3343	-0.07483	0.050113237	0.740018919
423.4926	428.2578	456.9083	0.135849	0.524923966	0.564967632
819.2884	763.6181	845.6996	0.13537	0.032527447	0.542081593
4359.115	4367.115	4955.779	0.061268	0.746491819	0.725549438
1363.396	1287.56	1328.807	-0.25799	0.276391642	0.130436078
944.3706	1007.01	904.3851	0.228995	0.662090696	0.205984307
222.4676	160.7128	230.5501	-0.0399	0.183178382	0.923910484
362.7384	389.2408	431.7574	-0.02173	0.336152763	0.925909288
460.1238	449.6243	510.354	-0.30034	0.39778623	0.13251575
4333.205	4135.8	4451.712	0.091791	0.860359707	0.656215943
897.018	879.74	1021.756	0.099741	0.864963799	0.686807001
4241.18	3869.184	3970.701	0.048541	0.429722241	0.746009092
1589.437	1757.622	1861.168	0.047284	0.732140966	0.824432784
864.854	849.0838	859.323	0.090393	0.395100931	0.730853476
1952.176	1806.858	1764.756	-0.00862	0.826883467	0.971886993
1126.633	919.686	889.7137	0.124007	0.963574889	0.616511903
955.9854	800.7771	1036.427	-0.2625	0.077927263	0.18366757
109.0002	88.2527	124.7066	-0.12749	0.410903637	0.826468162
1544.765	1588.549	1911.47	-0.01482	0.872444605	0.956290296
7282.464	7623.175	7372.363	0.008829	0.000468039	0.971658868
976.5346	1146.356	905.433	0.248342	0.637435429	0.138224336

348.4433	288.9115	325.914	0.26331	0.312218414	0.273534982
2978.743	3144.583	2701.628	0.258841	0.775422074	0.26142392
336.8285	393.8857	303.9069	0.09962	0.2795135	0.771436828
4.467221	3.715903	0	0.234721	0.365953812	NA
993.51	977.2825	1255.45	-0.19844	0.026312287	0.387451456
362.7384	343.721	334.2976	0.188987	0.490813332	0.470895778
754.067	679.0813	583.7109	0.297841	0.910345048	0.208318412
1332.125	1382.316	1288.985	0.08821	0.156349661	0.622128808
5324.928	5192.046	5306.844	-0.00303	0.569590917	0.990023409
2488.242	2458.999	2305.501	0.027175	0.206702304	0.889811144
2375.668	2196.099	2526.619	0.039736	0.310443697	0.853437613
723.6898	656.7859	580.567	-0.01117	0.206816173	0.966582892
10395.22	9891.734	8655.059	0.198273	0.084784548	0.321405678
1158.797	1248.543	1214.58	0.048708	0.004149955	0.80286002
0.893444	0	0	-0.91651	NA	NA
2870.636	3047.04	2662.853	0.170144	0.764235559	0.392881614
721.903	717.1693	765.0071	0.051358	0.843507519	0.809054023
108.1068	91.03962	121.5628	-0.43756	0.863207554	0.212919918
22332.53	21516.94	21928.46	-0.20067	0.958449073	0.237500588
537.8534	458.914	448.5247	0.566816	0.322090293	0.04830568
593.247	516.5105	601.5261	0.049752	0.944840582	0.875604624
47.35255	45.51981	31.43865	0.513224	0.817609984	0.379845684
1446.486	1456.634	1337.19	0.133685	0.907095813	0.443509781
602.1814	629.8456	572.1834	0.000187	0.32066134	0.996992829
193.8774	201.5877	253.6051	-0.43834	0.717185827	0.092469287
1379.478	1374.884	1451.418	-0.13543	0.990604058	0.484964546
295.73	313.0648	335.3456	0.154679	0.185572017	0.557712736
1108.764	1262.478	1211.436	-0.0533	0.041683941	0.795655341
337.7219	318.6387	373.0719	-0.18486	0.999199613	0.511678067
605.7552	681.8682	780.7264	-0.01889	0.660114316	0.948840677
1255.289	1198.379	1192.573	-0.02085	0.628362657	0.923910484
323.4268	322.3546	402.4147	0.0399	0.518074182	0.914120833
186.7298	224.8121	226.3583	0.197867	0.39473091	0.564516422
448.509	484.9253	514.5459	0.250663	0.089410503	0.733530443
535.1731	480.2805	483.1072	0.149233	0.094200573	0.437989627
176.902	198.8008	216.9267	-0.13471	0.098330299	0.729293105
524.4518	588.9706	658.1157	-0.22388	0.885070844	0.348491003
27.69677	42.73289	20.9591	0.331807	0.28287487	NA
3923.114	3969.513	4205.443	-0.00993	0.974211326	0.964166603
461.9107	418.0391	446.4288	-0.43366	0.287050142	0.009798351
3570.203	3530.108	3618.588	0.045308	0.82365137	0.764996976
365.4187	357.6557	429.6615	0.215957	0.847714103	0.408353147
89.34443	72.46011	102.6996	-0.00781	0.976661259	0.99985654
3.573777	0	2.09591	-0.08334	NA	NA
475.3123	497.931	599.4302	-0.27886	0.637486498	0.129766751
2013.823	1767.841	1747.989	0.251416	0.344730624	0.143052735
2347.972	2202.602	2161.931	0.045313	0.151730694	0.818645356
537.8534	547.1667	453.7645	0.200088	0.840099446	0.333065318
137.5904	137.4884	124.7066	0.554886	0.873487899	0.089996507
492.2878	488.6413	459.0042	0.351557	0.896727268	0.046180368
318.9596	267.545	208.543	0.310869	0.832043227	0.266775275
224.2545	250.8235	209.591	0.273972	0.907472172	0.394829397
1048.904	1012.584	950.4951	0.137714	0.015463986	0.56624122
1660.019	1632.21	1678.824	0.020542	0.80107822	0.904467867

358.2711	256.3973	306.0028	0.241225	0.848694292	0.421538281
4133.073	4193.397	3581.91	0.159777	0.380779612	0.422793542
921.141	1019.086	972.5021	-0.19558	0.56870053	0.247307157
1692.183	1755.764	1759.516	-0.09135	0.115119266	0.583691606
3635.425	3675.028	3618.588	-0.12744	0.219675312	0.318625647
484.2468	498.86	470.5317	0.220525	0.500344775	0.269882397
727.2636	876.0241	877.1382	0.035084	0.919200531	0.896670492
1898.569	1926.696	1470.281	0.215225	0.491320652	0.402165869
780.8703	886.2429	871.8985	-0.1126	0.484084014	0.5608115
1650.192	1703.742	1878.983	-0.27763	0.275860802	0.054657929
355.5908	412.4652	313.3385	-0.65368	0.836962769	0.001622235
749.5997	659.5728	676.9789	0.390321	0.587719544	0.014777529
1121.273	1142.64	1050.051	0.194649	0.920564616	0.342128545
509.2632	528.5872	674.883	-0.41507	0.270719269	0.026149665
2067.43	2064.184	2075.999	-0.00646	0.05082493	0.974691849
1064.092	961.4899	780.7264	0.240407	0.021160717	0.439957542
5.360666	15.79259	5.239774	-0.67902	0.935100162	NA
257.3119	299.1302	298.6671	0.34728	0.826883467	0.207300547
732.6243	573.178	725.1848	0.053899	0.783200259	0.859505388
1743.11	1692.594	1747.989	-0.25244	0.861141154	0.056859994
2608.857	2748.839	2386.193	0.073112	0.643482442	0.676538655
389.5417	348.3659	423.3738	-0.24628	0.828016939	0.320138645
551.2551	531.3741	622.4852	-0.37752	0.170962625	0.027155407
259.0988	280.5507	275.6121	0.148159	0.98409529	0.626829627
0.893444	9.289758	10.47955	-0.12607	NA	NA
1056.051	1133.35	1140.175	-0.11655	0.168770086	0.521135669
626.3044	638.2063	616.1975	0.069696	0.967934287	0.725153631
3894.524	3580.273	3330.401	0.052734	0.588304645	0.832900164
0	0	0	#DIV/0!	NA	NA
147.4183	142.1333	229.5021	-0.53425	0.441920545	0.083978449
130.4429	143.0623	114.2271	-0.08679	0.582003857	0.855100406
1131.994	1002.365	1088.825	0.009515	0.950182379	0.965909471
1113.232	1104.552	1064.722	0.225361	0.394573102	0.131442568
438.6811	436.6186	531.3131	0.077702	0.753108802	0.832900164
1011.379	846.2969	809.0212	0.385377	0.747302666	0.163640813
836.2638	784.0555	715.7532	0.233358	0.685468572	0.217256598
479.7796	520.2264	500.9224	-0.005	0.729944386	0.983593071
388.6483	379.0221	311.2426	0.251351	0.820906044	0.422560599
284.1153	319.5677	331.1537	-0.29097	0.50823828	0.181725119
655.7881	690.229	621.4373	0.158148	0.858869281	0.406611401
1336.593	1540.242	1669.392	-0.14309	0.484084014	0.377059311
27.69677	21.36644	24.10296	0.546063	0.433973714	NA
1240.101	1373.955	1121.312	0.026955	0.434274199	0.923725803
881.8295	934.5496	866.6587	-0.05674	0.265502165	0.79052565
3232.481	3027.532	3169.016	0.203386	0.156929518	0.149720105
874.6819	887.1719	940.0155	-0.42578	0.587188155	0.000186998
4253.688	4240.774	3547.327	-0.01786	0.000548797	0.93142242
427.0664	478.4225	448.5247	-0.076	0.913212741	0.811374268
570.9109	536.948	604.67	-0.06009	0.892614393	0.797980755
11850.64	12422.26	11577.81	-0.12353	0.598960113	0.395957024
329.6809	384.596	377.2638	0.109124	0.841901206	0.684679019
0	0	0	#DIV/0!	NA	NA
258.2054	155.139	232.646	0.269698	0.925116667	0.505493145
3262.858	3053.543	3029.638	0.214669	0.164920979	0.161107365

432.427	469.1328	503.0183	-0.02919	0.785492922	0.929693512
9830.567	9485.772	10490.03	-0.32434	0.673839005	0.012748693
1808.331	1845.875	1866.408	0.146381	0.769710962	0.33950184
37.52466	14.86361	25.15092	0.740292	0.985859213	NA
1275.838	1232.751	1287.937	-0.07005	0.769710962	0.724404377
542.3207	602.9053	504.0663	-0.09718	0.981637588	0.73912962
1014.059	1165.865	1162.182	0.041972	0.921802179	0.838059844
264.4595	277.7638	333.2497	-0.54558	0.396688154	0.003281665
1660.019	1552.318	1599.179	-0.08636	0.963625684	0.556915963
1173.092	1212.313	1087.777	-0.19473	0.197390524	0.229480039
1207.043	994.933	1152.75	0.041	0.465047544	0.875772383
2520.406	2260.198	2184.986	0.321408	0.335666342	0.161220563
1.786889	0	1.047955	-1.47895	NA	NA
1003.338	1063.677	1043.763	0.205368	0.999122571	0.26142392
142.0576	176.5054	155.0973	0.06946	0.798439173	0.87466351
4258.155	4123.723	5096.205	-0.38043	0.947527175	0.018979885
1.786889	0	5.239774	#DIV/0!	NA	NA
4282.278	4353.18	4647.68	-0.01763	0.881060269	0.923910484
653.1078	714.3824	528.1693	0.21629	0.934554042	0.42381604
56.28699	73.38909	105.8434	0.044562	0.735300868	0.960357548
61.64765	48.30674	52.39774	-0.82706	0.394743874	0.04089481
368.9925	367.8744	382.5035	0.292392	0.981637588	0.169257941
1559.06	1799.426	1686.159	-0.124	0.12559908	0.457073409
561.083	573.178	656.0198	0.019286	0.903964934	0.94960537
117.9346	113.335	112.1312	0.237789	0.888097311	0.563424525
1259.756	1198.379	1043.763	0.223579	0.956797855	0.223810637
944.3706	1046.027	880.2821	-0.00131	0.311074517	0.996992829
1727.028	1864.454	1487.048	0.195294	0.331098637	0.443864778
777.2965	748.7545	649.732	0.167273	0.643355604	0.620471139
2606.177	2811.081	2975.144	-0.09333	0.839737561	0.621036339
4050.876	4010.388	4604.714	-0.08827	0.861136809	0.649685593
1710.946	1800.355	1524.774	0.308006	0.134933146	0.020590928
0	0	0	#DIV/0!	NA	NA
998.8707	1006.081	783.8703	0.258386	0.247657122	0.161220563
622.7306	565.7462	643.4443	-0.06325	0.580755221	0.769874588
1036.395	1159.362	1153.798	-0.13465	0.193381894	0.407722648
1037.289	916.8991	847.7955	0.43654	0.30979442	0.054657929
2.680333	5.573855	13.62341	0.778674	0.78204194	NA
25.01644	15.79259	37.72638	0.987019	0.081926061	NA
499.4353	555.5275	610.9577	-0.09794	0.784189637	0.681942449
141.1642	190.44	130.9944	0.283624	0.592574853	0.565559013
2779.505	2662.445	2878.732	0.266295	0.584804307	0.0786728
28.59022	39.01698	32.4866	0.2576	0.92280023	NA
1540.298	1589.478	1426.267	0.037395	0.520356385	0.836904079
508.3698	452.4112	367.8322	-0.04489	0.663061022	0.899561084
735.3046	653.07	695.842	-0.03987	0.856763291	0.885866808
5440.182	5245.926	5162.226	-0.01153	0.211634187	0.950246349
409.1975	492.3572	383.5515	0.37129	0.302288037	0.122997082
1131.1	1194.663	1135.983	0.407352	0.933668895	0.001916809
985.469	1137.066	1068.914	0.000264	0.783200259	0.996720771
745.1325	666.0756	807.9732	-0.33491	0.493325129	0.054657929
12.50822	12.07668	14.67137	0.569514	0.820906044	NA
1244.568	1403.682	1456.657	-0.08094	0.66770133	0.73912962
871.1082	792.4163	807.9732	-0.0398	0.730407446	0.859505388

350.2301	401.3175	384.5994	0.281641	0.434940845	0.280613898
1080.174	1129.635	980.8858	0.110379	0.106967826	0.542805648
4653.951	4505.532	5112.972	-0.41879	0.380570392	2.68458E-06
480.673	592.6865	576.3752	0.009688	0.239965637	0.972140885
1073.92	1141.711	1025.948	-0.02928	0.108410382	0.884337789
922.9279	903.8934	1013.372	0.227473	0.812875146	0.252689545
3338.801	3702.897	3451.963	-0.00064	0.187777748	0.99985654
603.0749	680.9392	784.9182	-0.15277	0.820906044	0.605782742
5.360666	4.644879	17.81523	0.768277	NA	NA
972.9608	977.2825	893.9055	-0.09723	0.02392532	0.646200858
700.4603	617.7689	620.3893	-0.00181	0.8315774	0.997694701
515.5173	569.4621	631.9168	0.124824	0.720937669	0.667557921
545.8944	454.2691	521.8815	-0.56409	0.827268091	0.002810557
786.2309	684.6551	820.5487	-0.23964	0.708011334	0.208318412
470.8451	397.6016	517.6897	0.208863	0.760267849	0.538616173
12115.1	12010.73	11662.69	0.028034	0.412665866	0.826228143
1623.388	1559.75	1655.769	0.024358	0.092299784	0.884584155
1014.059	1013.513	1248.114	-0.0405	0.043065691	0.914553818
573.5912	653.07	805.8773	-0.42792	0.394915233	0.022699188
2036.159	1884.892	1954.436	-0.09193	0.455042454	0.665181522
0	1.857952	3.143865	-1.60143	0.950742266	NA
410.0909	491.4282	565.8956	-0.29178	0.683062991	0.210155633
5113.181	5157.673	5232.439	0.074991	0.229123467	0.567449963
256.4185	278.6927	343.7292	-0.13317	0.347941211	0.66509742
0	0	0	#DIV/0!	NA	NA
768.3621	744.1096	613.0536	0.208565	0.387883832	0.36518668
185.8364	152.352	187.5839	-0.35728	0.257353068	0.201829761
763.0014	822.1435	816.3569	0.024898	0.209444421	0.947221243
2811.669	2682.882	2571.681	0.116678	0.914164397	0.494034305
1298.175	1326.577	1360.245	-0.32036	0.208221128	0.076250172
2590.988	2601.132	2150.403	0.384387	0.211501936	0.073051917
524.4518	605.6922	540.7447	-0.00764	0.934554042	0.979820281
2797.374	2855.671	2974.096	0.011034	0.854862911	0.94960537
4603.025	4578.922	4496.774	0.248487	0.425091186	0.137779465
2285.43	2098.556	2520.332	-0.08707	0.904254847	0.649408519
1352.675	1281.987	1235.539	0.300257	0.696064091	0.064216598
0	0	0	#DIV/0!	NA	NA
153.6724	182.0792	173.9605	0.456476	0.46017679	0.110990341
426.1729	429.1868	426.5176	0.135618	0.902390281	0.575970418
2048.668	2304.789	1982.731	0.04874	0.981637588	0.79799583
647.7471	653.07	518.7377	0.054342	0.973922065	0.86344732
426.1729	445.9084	591.0466	-0.22254	0.478079371	0.333065318
469.9517	436.6186	438.0451	0.162594	0.871801928	0.420331471
5924.429	5679.758	6351.655	0.252341	0.962011634	0.027121839
1029.248	955.9161	1121.312	-0.15065	0.92790798	0.361214706
146.5249	142.1333	163.481	0.105286	0.831928191	0.764996976
823.7556	835.1492	708.4175	0.383202	0.599504319	0.150109736
302.8776	401.3175	400.3188	-0.21449	0.045014426	0.468420234
612.0093	720.8852	670.6911	0.120951	0.900383854	0.583691606
186.7298	160.7128	237.8858	0.111006	0.824111765	0.786995178
2843.833	3056.33	2788.608	0.125019	0.175845813	0.387856411
1467.035	1422.262	1303.656	0.461267	0.363948776	0.280613898
846.9852	786.8425	734.6164	0.079891	0.79861807	0.732126215
3567.523	3610.929	3273.811	0.005066	0.031159	0.975879427

0	0	0	#DIV/0!	NA	NA
41.09844	79.89192	97.45981	-0.84166	0.070750961	0.150320193
12619.01	12149.15	13863.4	-0.46284	0.670790001	0.000187867
7182.398	7166.119	7377.602	-0.03834	0.162448502	0.772252375
384.181	417.1101	373.0719	-0.1958	0.474093273	0.328075371
964.0264	809.1379	879.2342	0.193361	0.633808524	0.497467758
2655.316	2535.175	2502.516	0.12101	0.86365129	0.384595188
909.5263	810.0669	904.3851	0.024457	0.804201321	0.922828908
5.360666	4.644879	4.19182	-1.16926	NA	NA
722.7964	759.9022	741.9521	0.05486	0.43839941	0.7932398
6956.357	6736.003	7675.222	-0.20919	0.384189305	0.177690671
2569.546	2737.692	2655.518	-0.13538	0.802471571	0.487331989
38.4181	46.44879	52.39774	0.019619	0.914796531	NA
1609.093	1616.418	1456.657	0.180328	0.432618079	0.374236525
2730.366	2656.871	2988.767	-0.17747	0.267258978	0.343625666
3576.457	3672.241	3462.443	-0.04135	0.694473591	0.794422811
821.9687	866.7344	803.7814	0.113048	0.444234906	0.710509604
30.3771	27.86927	37.72638	-0.59854	0.935960933	0.318625647
6.25411	3.715903	9.431594	-0.1931	0.856745942	NA
30.3771	27.86927	17.81523	-0.47339	0.73478662	0.567139643
1328.552	1479.858	1391.684	-0.03181	0.651759792	0.896567419
229.6152	243.3916	225.3103	0.184178	0.986125936	0.525310286
1115.018	1102.694	951.543	-0.07018	0.960823113	0.796040638
1.786889	0	1.047955	-1.50799	NA	NA
340.4023	401.3175	367.8322	0.197617	0.842806932	0.483238043
12.50822	3.715903	8.383639	-1.18933	0.237605221	NA
14.29511	24.15337	29.34274	0.192348	0.716063981	NA
549.4682	678.1523	810.0691	-0.0784	0.861070915	0.81251428
88.45098	52.95162	116.323	-1.00667	0.767300341	0.002249649
1345.527	1265.265	1249.162	-0.04261	0.574512226	0.838059844
1224.912	1265.265	1258.594	0.080131	0.153105196	0.647435632
40.20499	30.6562	44.01411	-0.21242	0.010566322	0.765789524
1105.191	995.862	992.4133	0.165986	0.854862911	0.361564535
0	0	0	#DIV/0!	NA	NA
404.7302	345.579	368.8801	0.022775	0.995598118	0.929693512
51.81977	33.44313	67.06911	-0.20517	0.033849408	0.797601985
5945.872	5964.953	6230.092	0.083426	0.138269747	0.549038919
525.3452	500.7179	442.237	0.221122	0.399767691	0.36518668
278.7546	261.9712	273.5162	-0.11767	0.230402467	0.718291318
394.0089	415.2522	409.7504	0.164662	0.872444605	0.422560599
5419.633	5208.767	5561.497	-0.06558	0.859974619	0.65282506
67.00832	86.39475	82.78844	-0.713	0.522361449	0.017202731
2.680333	3.715903	7.335684	-0.05629	NA	NA
25.90988	18.57952	34.58251	-0.68225	0.746491819	NA
1.786889	0	2.09591	0.203136	NA	NA
1526.003	1472.427	1315.183	0.045687	0.940099788	0.818055883
4803.156	4302.087	4343.773	0.003891	0.276280221	0.987875073
25.90988	68.74421	44.01411	-0.06393	0.34814074	0.941511899
2.680333	2.786927	2.09591	-0.54908	NA	NA
95.59854	100.3294	67.06911	0.180311	0.992476114	0.746837826
3298.596	3253.273	3021.254	0.256899	0.072365168	0.161220563
542.3207	668.8625	519.7856	0.263274	0.412070549	0.277548691
1815.479	1664.725	1613.851	0.259626	0.539994787	0.244756568
172.4347	157.9259	180.2482	0.350596	0.981394188	0.332544931

1340.166	1249.472	1484.952	0.163668	0.250094568	0.39900629
18.76233	15.79259	17.81523	1.014539	0.734144448	NA
47.35255	32.51415	54.49365	-0.28589	0.731963541	0.656368654
18.76233	26.01132	24.10296	0.519337	0.813154117	NA
868.4278	910.3962	900.1933	0.013269	0.792137512	0.961026715
3370.965	3483.659	3331.449	0.035419	0.670790001	0.82991092
1070.346	1173.296	978.7899	0.130944	0.92288341	0.655129754
4378.77	4023.394	3833.419	0.057284	0.597827152	0.755018053
564.6568	533.2321	512.4499	0.204381	0.559595899	0.647117771
1533.15	1503.083	1404.26	-0.15324	0.024042196	0.357987739
848.772	813.7828	741.9521	0.129094	0.991136341	0.547201181
757.6407	842.581	838.3639	0.036872	0.590474301	0.875604624
1849.43	1838.443	1649.481	0.22019	0.302288037	0.0852812
3202.998	3270.924	3111.378	-0.24813	0.366033665	0.088369497
605.7552	598.2604	691.6502	0.030933	0.842190796	0.906493978
209.9594	187.6531	232.646	0.048658	0.714978777	0.908612163
25991.19	25875.69	26637.97	-0.35055	0.933496228	0.000652328
797.8457	744.1096	845.6996	-0.23361	0.805217464	0.224439117
12988.89	13304.79	13258.73	-0.18349	0.231538797	0.087860617
492.2878	507.2208	527.1213	0.045072	0.941323092	0.862606323
1.786889	3.715903	3.143865	-1.01635	0.614621507	NA
4059.811	4141.374	4781.818	-0.74159	0.947853941	1.88401E-07
712.0751	692.0869	662.3075	0.258675	0.960773888	0.162970478
15.18855	18.57952	15.71932	-0.46125	0.919006495	NA
836.2638	919.686	1011.276	-0.35965	0.84819607	0.036487394
809.4605	774.7658	714.7052	-0.16398	0.906767387	0.366628987
2795.587	2945.782	2574.825	-0.18003	0.000906247	0.124906775
8351.023	8362.64	8111.171	8.62E-05	0.972082308	0.999234423
626.3044	620.5558	570.0875	-0.00825	0.900789434	0.984451848
1806.544	1904.4	1623.282	0.154659	0.287237928	0.50961549
14403.21	13881.68	15069.59	-0.04115	2.89616E-19	0.780864543
172.4347	207.1616	190.7278	0.251597	0.918350023	0.422793542
2224.676	2001.943	2317.028	-0.44276	0.286635593	4.6512E-05
321.6399	315.8518	291.3315	0.353857	0.484865818	0.095730045
298.4104	308.42	322.7701	0.177858	0.612170686	0.562454643
984.5756	881.598	853.0353	0.006479	0.967693355	0.987413808
2585.628	2547.252	2440.687	-0.05416	0.000701205	0.712630985
2176.43	1918.335	2270.918	0.008671	0.910682739	0.969852341
192.0905	182.0792	295.5233	-0.40633	0.922937786	0.224223422
721.903	680.0103	847.7955	-0.08177	0.389882185	0.704908984
985.469	846.2969	898.0973	0.18993	0.795376015	0.39908158
857.7065	894.6037	906.481	0.380265	0.429631603	0.072863991
1335.699	1277.342	1166.374	0.155755	0.455280748	0.452447922
2221.102	2269.488	2485.749	0.21447	0.69974305	0.13004175
413.6647	410.6073	397.1749	-0.2434	0.733488302	0.201158042
1503.667	1529.094	1658.913	0.129167	0.342349862	0.423225223
106.3199	133.7725	132.0423	-0.06692	0.09014688	0.872689474
3.573777	8.360782	0	0.015758	NA	NA
6069.167	5556.204	5810.91	-0.17234	0.770381061	0.326388465
819.2884	834.2202	822.6446	0.174323	0.526872244	0.293268311
419.0254	456.1271	495.6827	0.198115	0.626106285	0.41069386
2351.545	2229.542	2083.334	0.262029	0.011540931	0.068011569
2609.751	2533.317	2320.172	0.140012	0.430461737	0.431825344
1205.256	1302.424	1288.985	-0.08907	0.560453068	0.614824771

3042.178	3122.288	2923.794	-0.09087	0.00628885	0.502754884
1484.011	1486.361	1636.906	-0.15718	0.687794385	0.385380994
227.8283	242.4627	244.1735	0.241529	0.854862911	0.554806856
1323.191	1329.364	1473.425	-0.07288	0.22234484	0.646200858
1725.241	1532.81	1637.953	0.103749	0.469683278	0.697184019
292.1563	281.4797	253.6051	0.091106	0.84337655	0.740018919
97.38542	110.5481	128.8985	-0.0676	0.812875146	0.914949396
631.6651	618.6979	680.1227	-0.10132	0.430461737	0.630402098
461.9107	413.3942	430.7095	-0.3997	0.596065856	0.012414864
210.8528	234.1019	237.8858	0.249031	0.923364203	0.535221009
695.0996	806.351	839.4119	0.036344	0.101973806	0.884380405
19.65577	15.79259	19.91114	-0.98048	0.434274199	NA
1355.355	1333.08	1262.786	0.001764	0.279741714	0.992528568
818.3949	831.4333	962.0226	-0.21058	0.324651337	0.311435996
6.25411	3.715903	7.335684	-0.41538	NA	NA
39558.14	39491.69	36040.22	-0.16836	0.521899793	0.267468464
7.147554	13.00566	12.57546	1.445878	NA	NA
993.51	1075.754	1097.209	-0.03575	0.990604058	0.885866808
1604.626	1525.378	1412.643	0.164815	0.362417219	0.302912952
465.4845	492.3572	501.9704	-0.10902	0.831439424	0.752683558
413.6647	430.1158	461.1002	-0.40229	0.189917625	0.019146595
28700.11	28763.88	26895.76	0.196539	0.530365059	0.045640789
3225.334	3277.426	3121.858	0.188402	0.009905069	0.131130356
1207.937	1211.384	1256.498	0.226815	0.368034878	0.351041753
704.0341	715.3113	707.3696	-0.09018	0.325126896	0.647117771
895.2311	968.9217	1159.038	-0.52256	0.249113533	0.001069433
2545.423	2540.749	2829.478	-0.07952	0.432073195	0.672052681
144.738	117.9799	160.3371	0.288321	0.925116667	0.539409857
581.6322	627.9876	625.6291	0.121361	0.44116367	0.579139814
369.8859	405.9624	490.4429	0.001159	0.371964696	0.996720771
2954.62	2998.734	2722.587	0.154524	0.147626297	0.422116695
1506.347	1525.378	1550.973	-0.09444	0.981762093	0.612461786
329.6809	280.5507	380.4076	-0.00813	0.827268091	0.990023409
597.7142	557.3855	471.5797	0.295548	0.262338145	0.252563103
1602.839	1562.537	1438.842	0.072568	0.950605067	0.730702561
2017.397	1857.952	1694.543	0.242371	0.0779973	0.317595626
1444.699	1207.668	1280.601	-0.07823	0.451017634	0.738713732
960.4526	1033.021	970.4062	0.11006	0.94309735	0.549936538
3518.383	3714.045	3589.246	-0.39702	0.886112674	0.017099575
205.4922	235.0309	223.2144	0.08698	0.029186964	0.81253834
76.83621	69.67318	114.2271	0.158404	0.766253365	0.783572702
10353.23	10300.48	9988.058	0.061082	0.233074614	0.643988563
3042.178	2788.785	2754.025	0.323761	0.139314666	0.002049784
5373.174	5154.887	5631.71	0.134475	0.419992784	0.207615914
454.7631	362.3005	435.9492	0.240816	0.866015481	0.298450788
442.2549	521.1554	594.1904	0.334329	0.831928191	0.153668434
475.3123	427.3289	421.2779	-0.07791	0.619820968	0.831655346
645.0668	728.317	809.0212	-0.24414	0.57058689	0.207615914
32.16399	47.37776	56.58956	-0.35653	0.794232621	0.545985077
21.44266	7.431806	10.47955	-0.32247	0.756663214	NA
641.493	589.8996	538.6488	0.127977	0.658567464	0.602733581
201.9184	126.3407	202.2553	-0.09343	0.499877044	0.834319281
374.3531	405.9624	462.1481	-0.46019	0.468943258	0.00941523
1197.215	1086.902	1086.729	0.141118	0.096837782	0.435236327

1698.438	1460.35	1449.322	0.249649	0.41286504	0.251743462
696.8865	656.7859	907.5289	-0.3162	0.553198087	0.146815781
64.32799	68.74421	64.9732	0.377177	0.681216827	0.553844877
287.6891	322.3546	322.7701	0.020092	0.907472172	0.959341496
46.4591	63.17035	61.82934	0.113028	0.828379866	0.886786965
4720.959	4727.558	4321.766	-0.00015	0.540391114	0.998959824
704.9275	643.7802	647.6361	0.320576	0.960956671	0.186593864
1027.461	930.8337	940.0155	0.335743	0.168465623	0.251817128
983.6821	1101.765	1013.372	0.018634	0.759261525	0.923910484
0	0	0	#DIV/0!	NA	NA
36.63121	39.94596	29.34274	-0.37341	0.989820945	0.539844242
351.1236	345.579	379.3597	-0.08227	0.22648195	0.786706509
808.5671	847.2259	1037.475	-0.66657	0.837292836	3.21175E-06
439.5746	409.6783	339.5374	-0.1357	0.27470085	0.588371763
1.786889	2.786927	2.09591	0.34037	NA	NA
1668.06	1572.756	1716.55	0.009471	0.43802737	0.9614279
1826.2	1776.202	1759.516	0.019885	0.485402441	0.922101817
2180.004	2312.221	1928.237	-0.03331	0.004783907	0.889879926
3720.302	3846.889	3730.719	0.11019	0.28025041	0.305200742
8058.867	7771.811	8583.799	-0.38661	0.71685777	0.001102729
796.9523	770.1209	682.2186	0.224097	0.941098554	0.374248122
5131.05	5101.006	4993.505	-0.05579	0.901342739	0.796040638
1194.535	1291.276	1350.814	-0.18128	0.254252351	0.324099572
554.8289	548.0957	562.7518	-0.06765	0.549915616	0.786706509
607.5421	638.2063	591.0466	-0.0854	0.530449366	0.703022228
3954.384	3982.519	4463.24	-0.23312	0.836588992	0.110742141
1078.387	1151.001	1006.037	0.234361	0.495904925	0.342128545
1776.167	1513.302	1564.597	0.279378	0.458486853	0.320399987
10.72133	13.00566	14.67137	-0.68663	0.901132408	NA
2073.684	2128.283	2024.649	0.043045	0.199696516	0.81253834
2490.923	2668.947	2737.258	-0.1328	0.886112674	0.315833346
18037.75	17834.48	19790.63	-0.41043	0.794679259	0.001503374
230.5086	246.1786	253.6051	-0.02145	0.274927458	0.931975702
1045.33	979.1405	892.8576	0.097933	0.79121035	0.687134752
5.360666	16.72156	24.10296	-0.16544	0.817650331	NA
1790.462	1531.881	1643.193	0.160437	0.034700814	0.37339403
1288.347	1230.893	1323.567	-0.03125	0.185152106	0.889747395
879.1491	875.0952	935.8237	0.073408	0.164920979	0.791888862
662.9356	667.0046	536.5529	0.127835	0.985859213	0.611282744
1587.65	1656.364	1893.654	0.104469	0.034700814	0.682436785
368.099	404.1045	365.7363	0.142392	0.572336545	0.65282506
3345.949	2866.819	2790.704	0.397998	0.237605221	0.410595442
7.147554	4.644879	6.287729	0.757246	0.907551741	NA
410.9844	471.9197	477.8674	-0.01415	0.081828292	0.972140885
231.4021	239.6757	176.0564	0.062608	0.962011634	0.850669379
3324.506	3405.625	3431.004	-0.09962	0.944173424	0.484163033
598.6077	476.5646	460.0522	0.116	0.884585407	0.658878607
256.4185	185.7952	203.3032	0.035004	0.37602059	0.92316895
0.893444	0.928976	6.287729	1.433123	NA	NA
578.0584	622.4138	511.402	-0.03462	0.808425694	0.914553818
523.5583	424.5419	598.3822	0.137904	0.682715357	0.636454553
1.786889	0	4.19182	0.468662	NA	NA
182.2626	135.6305	207.4951	-0.14856	0.29215357	0.739592868
67.00832	61.3124	50.30183	-0.04364	0.935960933	0.971658868

1090.895	1011.655	1134.935	-0.12838	0.240595478	0.595440724
1134.674	1265.265	1104.544	0.285201	0.33210787	0.260327022
83.98376	98.47143	84.88435	-0.32867	0.826736401	0.47562949
2134.438	2263.914	1968.059	0.238912	0.265699092	0.168910143
1152.543	1155.646	1094.065	0.136299	0.835449534	0.463413871
661.1488	638.2063	640.3004	0.10732	0.542904501	0.656368654
1731.495	1923.909	1816.106	-0.10115	0.967813477	0.589270302
1968.258	1927.625	1856.976	0.065772	0.301433669	0.672204533
1752.044	1958.281	1873.743	-0.20595	0.243873626	0.221138056
542.3207	497.002	507.2102	0.11374	0.239965637	0.61283164
874.6819	915.9701	1018.612	-0.44585	0.187415981	0.001258658
84.8772	61.3124	75.45275	-0.50707	0.592673308	0.192843722
1179.346	1117.558	1294.224	-0.31822	0.01192228	0.06712239
838.0507	797.0612	976.694	-0.18169	0.010145368	0.431825344
116.1478	164.4287	165.5769	-0.06843	0.007236622	0.879709806
2608.857	2474.791	2209.089	0.241416	0.226961756	0.285602122
645.0668	744.1096	838.3639	-0.38885	0.786445338	0.029788115
3376.326	3543.114	3684.609	-0.13607	0.324105472	0.339645561
1123.059	1011.655	1051.099	0.042211	0.114505424	0.835596295
23.22955	20.43747	26.19887	0.37428	0.542904501	NA
680.8045	656.7859	642.3963	0.0757	0.956452433	0.730853476
543.2141	600.1183	667.5473	-0.32215	0.883354954	0.049190168
442.2549	409.6783	550.1763	0.000491	0.408718189	0.999397196
355.5908	366.0165	313.3385	0.117406	0.610241564	0.677324798
970.2805	991.2171	955.7349	0.172514	0.896802518	0.372335894
18.76233	12.07668	6.287729	0.323708	0.827572328	NA
792.4851	763.6181	773.3907	-0.14937	0.388777613	0.512079093
267.1398	213.6644	247.3174	0.020436	0.666493976	0.975879427
1553.7	1573.685	1516.391	-0.12555	0.975298616	0.291835413
16.082	13.00566	17.81523	0.256867	0.967813477	NA
1.786889	0	12.57546	-1.7644	0.990064993	NA
319.853	310.2779	362.5924	-0.07749	0.982390822	0.833611802
1198.109	1111.055	1131.791	0.182736	0.791689312	0.198030129
1350.888	1550.461	1303.656	0.077241	0.34687574	0.723437511
6445.307	6329.112	6842.098	-0.06822	0.0779973	0.583691606
3.573777	3.715903	3.143865	-0.48944	0.826883467	NA
2765.21	2847.311	2591.592	-0.0583	0.755826522	0.774121067
1219.551	1293.134	1322.519	0.376369	0.642246351	0.008625676
345046.4	339485.8	323258.5	-0.25475	0.904254847	0.049333376
285.9022	287.0535	257.7969	0.195136	0.399288509	0.614824771
386.8614	449.6243	378.3117	0.058274	0.812875146	0.858146459
788.0178	883.4559	813.213	0.184746	0.028201152	0.302912952
555.7223	544.3798	552.2722	0.056756	0.998609328	0.835596295
865.7475	896.4616	911.7208	-0.19723	0.002987139	0.239315229
598.6077	615.9109	511.402	0.18945	0.072027046	0.395164375
441.3615	377.1642	248.3653	0.069797	0.469347309	0.856460907
45517.41	43678.58	41578.66	-0.05744	0.212716004	0.672730781
1791.356	2032.599	2229	0.809213	0.617661583	7.09339E-10
1450.06	1308.927	1465.041	-0.10662	0.723128445	0.495076977
220.6807	191.369	215.8787	-0.12056	0.503345543	0.745567347
4029.434	3986.235	4132.086	0.217097	0.695594764	0.011539863
734.4112	881.598	623.5332	0.223593	0.553198087	0.374236525
1122.166	1220.674	974.5981	0.236538	0.179746977	0.327516537
6388.126	6503.759	6464.834	-0.16507	0.44116367	0.064858857

621.8372	689.3	712.6093	-0.02035	0.269120346	0.929220289
352.017	354.8687	407.6545	-0.31766	0.08410248	0.10698808
8.934443	8.360782	9.431594	-0.21313	0.871801928	NA
3535.359	3725.193	3516.937	-0.09301	0.001223265	0.65282506
576.2715	516.5105	455.8604	0.085975	0.38025264	0.797601985
76.83621	75.24704	88.02821	0.322646	0.960823113	0.572969021
520.878	520.2264	528.1693	-0.04858	0.54674796	0.841070222
2120.143	1975.002	1847.544	0.10269	0.948896953	0.521095388
2723.218	2580.695	2618.839	0.01972	0.769710962	0.915543523
0	1.857952	6.287729	-0.37122	0.837998472	NA
12.50822	12.07668	27.24683	1.897833	0.986203512	NA
26.80333	13.00566	6.287729	0.297055	0.354108154	NA
437.7877	366.9454	422.3258	-0.21824	0.6333028	0.361214706
4342.139	4067.985	4107.983	0.262503	0.07224611	0.067080808
192.0905	229.457	170.8166	0.001841	0.827268091	0.990142758
1276.732	1208.597	1166.374	0.354181	0.728952588	0.137586476
2169.283	1988.937	2090.67	0.079814	0.607918252	0.589603442
10.72133	7.431806	8.383639	-0.49962	0.831317845	NA
104.533	88.2527	75.45275	0.961239	0.842806932	0.12712136
2124.61	2039.102	2133.636	0.180081	0.220715145	0.190817982
2190.725	2174.732	2101.15	-0.0673	0.506564301	0.680780346
1.786889	0.928976	3.143865	-1.46042	0.694447279	NA
841.6245	753.3993	739.8562	0.20802	0.173204878	0.394044776
5.360666	6.50283	3.143865	-0.08921	0.774045793	NA
8.040998	3.715903	6.287729	0.912204	NA	NA
29.48366	33.44313	28.29478	0.3795	0.8968711	NA
506.5829	442.1925	518.7377	-0.23788	0.007739408	0.22043651
686.1652	716.2403	698.9859	0.036603	0.828016939	0.884087376
4958.616	4841.822	4686.454	0.143203	0.86365129	0.189401758
19.65577	70.60216	58.68547	-0.40887	0.965666941	0.540191544
50.92632	56.66752	54.49365	0.232814	0.283463824	0.800895715
5.360666	8.360782	2.09591	-0.91503	0.962556121	NA
20.54922	12.07668	27.24683	-0.04577	0.471685453	NA
160.82	184.8662	132.0423	-0.35698	0.730800205	0.380365797
5.360666	7.431806	0	0.958038	NA	NA
1416.109	1425.978	1194.669	0.116357	0.392128795	0.568229673
843.4114	875.0952	849.8914	0.082448	0.684723286	0.690294956
1.786889	0.928976	1.047955	-0.48506	NA	NA
889.8705	874.1662	974.5981	-0.21385	0.208221128	0.186548284
201.9184	164.4287	185.488	-0.04885	0.875720952	0.891559992
5390.149	5051.77	4488.391	-0.10814	5.99679E-07	0.644740483
31.27055	20.43747	28.29478	-0.27328	0.393551305	NA
3330.76	3204.037	2915.411	0.272687	0.125442514	0.280613898
33.95088	31.58518	19.91114	-0.91075	0.183923135	0.097257448
1388.412	1293.134	1435.698	0.033464	0.520751048	0.855100406
585.206	610.3371	671.7391	-0.27613	0.381821718	0.073548481
594.1404	491.4282	681.1707	-0.11024	0.947527175	0.754561878
686.1652	622.4138	606.7659	0.040722	0.352157146	0.886786965
1176.666	1147.285	994.5092	-0.04295	0.962433805	0.87466351
335.935	404.1045	359.4485	-0.24744	0.285144055	0.247307157
431.5336	364.1585	418.134	-0.15978	0.362090257	0.517933072
966.7067	930.8337	1031.188	-0.06204	0.001914596	0.744172227
1542.978	1542.1	1424.171	0.185511	0.586957479	0.333065318
33221.83	33453.35	33559.71	-0.56541	0.585115533	2.31559E-10

3279.834	3214.256	3042.213	-0.05849	0.711956678	0.684131921
33.05744	15.79259	37.72638	-0.05656	0.918350023	0.93327699
1384.839	1324.719	1288.985	0.046749	0.707368241	0.859505388
547.6813	607.5501	592.0945	0.135092	0.936346196	0.511081603
386.8614	354.8687	325.914	0.201873	0.826098854	0.443275405
619.1569	620.5558	685.3625	-0.01075	0.690391809	0.957969787
1295.494	1298.708	1309.944	-0.05135	0.403965481	0.797601985
1.786889	0	6.287729	#DIV/0!	NA	NA
25.01644	12.07668	18.86319	0.581027	0.875830579	NA
3299.49	3380.543	3694.041	0.191139	0.260444304	0.173745879
15.18855	13.00566	19.91114	-0.70661	0.962011634	NA
3078.809	3118.572	3877.433	-0.69588	0.025214551	4.83481E-06
972.0674	1010.726	1068.914	0.062444	0.617789656	0.80286002
3759.613	3852.462	5105.636	-0.59292	0.969447004	0.000264275
4930.919	5452.159	5590.839	-0.24105	0.847517975	0.100672129
2532.021	2677.308	2384.097	0.261518	0.54674796	0.055185829
422.5991	317.7097	281.8999	-0.06278	0.252640265	0.878767667
1399.134	1452.918	1461.897	0.077358	0.892240442	0.658276069
58.07388	22.29542	37.72638	0.34401	0.827268091	NA
1324.084	1325.648	1125.504	0.118208	0.493325129	0.579998211
253.7382	235.0309	285.0437	-0.34419	0.784378477	0.10424789
1017.633	873.2372	889.7137	0.241285	0.988569159	0.164747869
1051.584	1033.021	1207.244	-0.16777	0.792868376	0.364490432
235.8693	171.8605	141.4739	-0.22871	0.919799198	0.553799324
1064.092	1018.157	870.8505	0.032207	0.485973657	0.912173468
1090.895	994.933	947.3512	0.180565	0.573411006	0.451963117
0.893444	0	2.09591	1.182577	NA	NA
1688.61	1727.895	1693.495	-0.30712	0.804201321	0.039022257
6431.012	6310.532	6401.956	0.163478	0.154093864	0.159141538
7.147554	3.715903	0	1.211875	NA	NA
2104.955	2278.778	2073.903	-0.06042	0.94035236	0.794119423
1376.798	1426.907	1199.908	0.199855	0.683079974	0.275261425
3946.343	3923.065	3500.169	0.107465	0.00758806	0.659338327
2919.776	2897.475	3046.405	-0.34845	0.904254847	0.003399865
3183.342	3065.62	2936.37	0.159382	0.727266789	0.149596369
308.2383	324.2125	427.5656	-0.02842	0.817060109	0.935768815
4010.671	3989.022	3186.831	0.251049	0.270624697	0.566028914
1071.24	1189.089	1330.903	-0.24398	0.600008341	0.258978375
14127.14	13938.35	14911.35	0.132413	0.897766774	0.341785585
826.4359	715.3113	577.4231	0.231279	0.324651337	0.465059088
773.7227	701.3767	686.4105	0.076529	0.133378715	0.762106199
69.68865	44.59084	83.83639	-0.30896	0.226105917	0.570089152
2303.299	2485.939	2517.188	-0.14493	0.9951976	0.33997316
1323.191	1252.259	1321.471	0.094844	0.840763345	0.637407167
5350.838	5247.784	6302.401	-0.28967	0.875368742	0.073064303
1730.602	1571.827	1583.46	0.082427	0.072027046	0.681523937
1354.461	1369.31	1425.219	0.020021	0.6212524	0.919916667
504.796	534.1611	638.2045	-0.17102	0.12376446	0.505302546
102.7461	126.3407	167.6728	0.096618	0.377049586	0.836257824
97.38542	107.7612	90.12412	0.188636	0.472470204	0.683195977
181.3692	145.8492	161.3851	-0.36513	0.843125549	0.210525115
1237.42	1159.362	1217.724	0.16964	0.833499639	0.278100425
31592.19	30848.5	33662.41	-0.59234	0.864963799	4.66632E-07
5791.306	5543.198	5914.657	0.07888	0.894528942	0.55402404

223.3611	236.8888	200.1594	0.269218	0.622518586	0.333325701
947.0509	1020.944	828.9323	0.080822	0.990735591	0.818719704
741.5587	741.3227	724.1368	0.115227	0.923281561	0.55088846
619.1569	510.9367	617.2454	0.135977	0.730407446	0.642738974
675.4439	746.8965	836.268	-0.05774	0.818107632	0.822780332
0.893444	3.715903	8.383639	1.470124	0.727337313	NA
1011.379	888.1008	1084.633	-0.01574	0.484865818	0.942357977
1746.684	1758.551	1716.55	0.217603	0.308044443	0.348829966
720.1161	617.7689	617.2454	0.439168	0.852231703	0.001782533
359.1646	401.3175	436.9972	-0.18903	0.986203512	0.425905313
591.4601	557.3855	598.3822	0.052102	0.843125549	0.823761853
653.1078	666.0756	776.5346	-0.01955	0.535057938	0.955311096
2002.209	2094.84	2330.652	-0.00878	0.921413319	0.9717805
1.786889	0	0	-1.74916	NA	NA
612.9028	616.8399	644.4923	0.087109	0.948896953	0.708596546
677.2307	750.6124	778.6305	0.157935	0.898182122	0.365235491
23.22955	24.15337	22.00705	0.713909	0.935506037	NA
181.3692	127.2697	145.6657	-0.2162	0.537749415	0.582933252
818.3949	881.598	945.2553	0.147296	0.521899793	0.462978863
13421.32	13652.23	13762.79	-0.2833	0.730204074	0.043279608
1002.444	947.5553	1119.216	0.102256	0.226105917	0.67856997
700.4603	692.0869	732.5205	-0.1163	0.935506037	0.534883449
422.5991	469.1328	495.6827	0.065108	0.429368942	0.831655346
4398.426	4177.604	4520.877	-0.11288	0.904521596	0.517983853
14.29511	2.786927	2.09591	1.388777	NA	NA
2582.054	2699.604	2426.016	-0.06085	0.848509848	0.764996976
641.493	583.3968	484.1552	0.116814	0.648624949	0.686855559
1014.953	1175.154	1341.382	-0.04034	0.354108154	0.887908388
514.6239	516.5105	506.1622	-0.14568	0.652183366	0.489096476
2287.217	2373.533	2549.674	0.098057	0.827268091	0.532286968
187.6233	205.3036	172.9126	-0.34889	0.000120934	0.307273072
199.2381	155.139	249.4133	-0.23988	0.090626117	0.490908806
62.5411	37.15903	77.54866	0.678377	0.916416444	0.201158042
560.1895	545.3088	615.1495	0.074102	0.678440947	0.768125604
1441.126	1521.662	1462.945	0.041825	0.493325129	0.79934904
1291.027	1335.867	1496.48	-0.26881	0.486409231	0.064858857
1873.553	1736.256	1758.468	0.259357	0.707092679	0.294886007
7115.39	7201.42	9050.138	-0.18801	0.976681492	0.370905349
0	2.786927	0	0.081418	NA	NA
727.2636	793.3453	766.055	0.102075	0.962433805	0.6924902
118.8281	130.0566	119.4669	-0.21564	0.107676441	0.566742383
990.8297	885.3139	855.1312	0.158856	0.214675875	0.569403364
4328.737	4447.936	4788.106	-0.04224	0.650244439	0.840777068
1.786889	9.289758	12.57546	-0.41393	0.916361438	NA
458.3369	465.4169	409.7504	0.236698	0.072365168	0.283703282
840.731	980.9984	720.993	0.036397	0.707919104	0.908612163
369.8859	358.5846	341.6333	0.039028	0.112254368	0.884584155
75.04932	75.24704	96.41185	-0.59594	0.91014027	0.088358317
251.0578	224.8121	268.2765	-0.44198	0.769710962	0.201829761
778.1899	715.3113	710.5134	0.100672	0.274927458	0.640053604
234.9758	257.3263	303.9069	0.103813	0.210289554	0.787901563
587.8863	633.5615	649.732	-0.18123	0.033611173	0.314044192
12.50822	18.57952	8.383639	-0.56472	0.61217167	NA
3598.793	3384.259	3425.765	0.205336	0.811082292	0.117115343

310.0252	336.2892	345.8251	-0.22054		0.337335124	0.402824474
252.8447	236.8888	219.0226	-0.14069		0.759261525	0.677324798
1.786889	1.857952	2.09591	#DIV/0!	NA	NA	
0	0	0	#NUM!	NA	NA	
3333.441	3271.853	3154.344	0.014862		0.212716004	0.936794868
2130.865	2027.025	1882.127	0.052822		0.44342611	0.832900164
60.75421	90.11065	82.78844	-0.37659		0.783760899	0.463947642
314.4924	248.0365	351.0649	0.283328		0.241933407	0.372335894
2435.529	2495.229	2554.914	-0.10695		0.995861369	0.487331989
1180.24	1135.208	1121.312	0.074426		0.451912698	0.728253469
3003.76	2799.933	3010.774	-0.40631		0.411467295	0.001219868
41.09844	20.43747	54.49365	-0.5625		0.904254847	NA
558.4027	516.5105	526.0734	-0.15123		0.779325825	0.490958801
1754.725	1632.21	1925.093	-0.0748		0.582750031	0.752838067
1641.257	1623.85	2026.745	0.117199		0.865150494	0.690294956
1815.479	1991.724	1754.276	0.371943		0.280466121	0.028065868
368.099	311.2069	287.1396	-0.10009		0.048326024	0.757633181
504.796	586.1837	569.0395	-0.0454		0.861070915	0.873196229
247.4841	252.6814	193.8717	0.324056		0.072773765	0.27042216
2163.922	2266.701	2476.317	0.082905		0.907095813	0.647040539
1360.716	1331.222	1477.616	-0.08146		0.04129094	0.583691606
1278.519	1484.503	1498.575	-0.34981		0.617661583	0.041646854
1945.922	1875.602	2020.457	-0.17826		0.579526371	0.167949464
559.2961	583.3968	570.0875	-0.39165		0.286938782	0.079283616
1468.822	1517.946	1374.917	0.106225		0.482562486	0.542805648
0	0	0	#DIV/0!	NA	NA	
1346.42	1239.254	1304.704	-0.09428		0.864963799	0.595423496
234.9758	264.7581	211.6869	-0.25485		0.005955217	0.403397484
421.7057	422.684	394.031	0.452269		0.757279972	0.091639488
524.4518	485.8543	517.6897	-0.14034		0.860650755	0.55088846
671.8701	676.2944	689.5543	0.203671		0.434274199	0.409178926
375.2466	380.8801	358.4006	0.058969		0.60948547	0.880061352
8.934443	5.573855	6.287729	-0.56536		0.345374681	NA
362.7384	387.3829	400.3188	0.193164		0.905579464	0.422793542
488.714	377.1642	441.189	0.069866		0.58195463	0.833848825
1056.051	1085.044	949.4471	0.261918		0.323780681	0.098609359
696.8865	599.1894	688.5064	0.144878		0.869592661	0.561367299
323.4268	404.1045	298.6671	0.203262		0.94035236	0.565559013
2136.225	2000.085	1909.374	0.275267		0.136068446	0.250258579
15399.41	16334.18	17299.64	-0.34034		0.817060109	0.005166188
469.0582	564.8173	463.1961	0.369166		0.985859213	0.046018016
959.5591	941.0524	913.8167	0.312896		0.632040487	0.081022399
209.066	215.5224	155.0973	0.01764		0.27747938	0.960357548
4421.656	4447.007	5063.718	-0.26025		0.711342547	0.083564912
746.026	827.7174	758.7193	-0.21639		0.649714874	0.314015775
7143.98	6454.524	6103.289	0.199294		0.171568235	0.280613898
2658.89	2376.32	2318.076	0.19192		0.237605221	0.700583782
5.360666	10.21873	4.19182	-0.75675		0.83213076	NA
943.4771	957.774	1195.717	-0.19453		0.785492922	0.515285838
173.3282	182.0792	211.6869	0.280542		0.785492922	0.557230622
1318.724	1351.66	1255.45	0.102387		0.856244695	0.480320232
8841.524	8497.341	8452.804	-0.02453		0.856763291	0.86814025
1518.855	1516.088	1297.368	0.0343		0.233444561	0.896421455
4.467221	0	2.09591	#DIV/0!	NA	NA	

122.4019	112.4061	121.5628	0.121526	0.183509443	0.797601985
1758.298	1753.906	1616.994	0.338193	0.811220717	0.007195336
236.7627	194.1559	206.4471	0.041899	0.952783825	0.912024085
16.97544	12.07668	23.05501	0.228548	0.716351188	NA
0	0	1.047955	-0.68638	NA	NA
1055.158	924.3309	954.6869	0.13613	0.206702304	0.658318551
966.7067	823.0725	906.481	0.010009	0.479830743	0.973662475
2186.258	2159.869	2517.188	0.008919	0.988569159	0.975879427
543.2141	551.8116	491.4908	-0.04694	0.847071887	0.889879926
5744.847	5423.36	5084.677	0.100751	0.352115003	0.666217785
407.4106	317.7097	345.8251	-0.15606	0.274301907	0.575540189
19.65577	33.44313	40.87024	-0.19101	0.848694292	NA
343.0826	341.8631	274.5642	0.067871	0.597707702	0.865063191
147.4183	139.3464	164.5289	0.447926	0.84488548	0.208318412
1303.535	1278.271	1438.842	-0.22413	0.58195463	0.194891359
776.4031	716.2403	948.3992	0.403213	0.690604569	0.028545761
545.8944	524.8713	548.0804	-0.10712	0.004522157	0.589413891
2445.357	2415.337	2167.171	0.096992	0.81803103	0.579811887
251.9513	218.3093	290.2835	-0.11242	0.049410877	0.746009092
1562.634	1583.904	1258.594	0.238332	0.391682273	0.309248984
757.6407	690.229	800.6375	-0.09922	0.80037736	0.685414212
945.264	954.9871	1229.251	0.062588	0.04935905	0.821432697
58.96732	56.66752	76.50071	-0.48284	0.592978918	0.223810637
548.5748	463.5589	383.5515	0.048345	0.251419778	0.908612163
67.00832	91.9686	101.6516	0.45793	0.365690431	0.283438968
2130.865	2127.354	2441.735	0.065643	0.835449534	0.740018919
0	0	0	#DIV/0!	NA	NA
319.853	342.7921	343.7292	0.173482	0.92288341	0.495986013
694.2062	759.9022	653.9239	-0.19526	0.652050398	0.365235491
4859.443	4643.95	5586.648	-0.10822	0.739965249	0.575067875
989.9362	1006.081	1091.969	-0.12982	0.861070915	0.462496385
152.779	124.4828	111.0832	0.241318	0.577332911	0.516190108
25572.16	24808.3	25350.03	-0.36827	0.832298202	0.00280023
1165.051	1169.58	1141.223	0.169663	0.383871116	0.281445446
1115.912	1227.177	1151.702	-0.13521	0.072296871	0.422793542
1836.921	1805.929	1701.879	-0.18833	0.770051929	0.161220563
1028.354	1098.978	928.488	0.154225	0.320915881	0.359873864
1452.74	1564.395	1781.523	0.116437	0.794679259	0.558104763
13691.14	13650.37	14468.07	-0.42244	0.728952588	0.005161963
591.4601	548.0957	509.3061	0.450136	0.136068446	0.092469287
450.2959	452.4112	429.6615	0.310824	0.93984524	0.081121312
1104.297	997.72	1016.516	0.325788	0.906368111	0.044242334
460.1238	431.0448	444.3329	-0.05419	0.510046913	0.840548247
1263.33	1353.518	1174.757	0.190902	0.484625827	0.398655382
45.56566	16.72156	36.67842	-0.13046	0.572336545	NA
176.902	131.9146	142.5219	0.120032	0.410903637	0.806564543
0	0	0	#NUM!	NA	NA
2115.676	1874.673	1734.365	0.054899	0.637091947	0.819964665
0	0	1.047955	#DIV/0!	NA	NA
0	2.786927	3.143865	#DIV/0!	NA	NA
1065.879	1252.259	985.0776	-0.05987	0.17246068	0.817988115
670.9766	646.5671	522.9295	0.276005	0.57058689	0.380264685
5187.337	5197.619	5526.914	-0.05316	0.527593235	0.755157732
766.5752	828.6464	636.1086	0.375097	0.78618046	0.065248652

280.5415	201.5877	234.7419	0.100975	0.856763291	0.798013277
322.5334	354.8687	359.4485	-0.08566	0.01003883	0.756795912
3113.653	3213.327	3199.406	-0.14598	0.498379067	0.385637803
4200.975	4326.24	4009.475	0.020937	0.064220724	0.908612163
28.59022	19.50849	16.76728	-0.65241	0.354108154	NA
1174.879	1102.694	1096.161	0.129269	0.497263297	0.50924517
654.8946	597.3314	624.5811	0.263565	0.572219847	0.098488434
387.7548	400.3886	319.6262	0.185702	0.883354954	0.598691725
1368.757	1378.6	1439.89	0.286883	0.05643267	0.009798351
1431.298	1451.989	1415.787	0.000398	0.331331472	0.999397196
1003.338	965.2058	887.6178	0.195704	0.740595411	0.303824089
611.1159	645.6382	575.3272	-0.03375	0.0779973	0.897989514
963.1329	1018.157	868.7546	0.200008	0.285144055	0.409178926
1074.813	1243.899	1026.996	0.219528	0.663047044	0.36518668
477.9927	453.3402	403.4626	-0.14994	0.955592311	0.550627492
939.0099	926.1888	1040.619	-0.41981	0.770051929	0.001123727
132.2298	156.9969	161.3851	-0.50677	0.205414362	0.073548481
1068.559	1042.311	1096.161	0.012587	0.6333028	0.952666818
1008.699	1064.606	1096.161	-0.00815	0.849390636	0.984451848
26.80333	32.51415	19.91114	-0.11195	0.968645699	NA
670.9766	672.5785	790.158	-0.24678	0.871801928	0.200030838
0	0.928976	1.047955	0.586005	NA	NA
0.893444	1.857952	1.047955	-1.85036	NA	NA
2450.718	2280.635	2750.882	-0.24632	0.465940241	0.118990473
0	0	0	#DIV/0!	NA	NA
364.5253	387.3829	386.6954	0.096205	0.716351188	0.73912962
801.4195	758.0442	903.3371	-0.20759	0.693771149	0.386225298
0	0	1.047955	-0.32968	NA	NA
401.1565	382.738	384.5994	0.752717	0.759261525	0.00133704
3.573777	0.928976	4.19182	-0.82348	NA	NA
824.6491	822.1435	810.0691	0.101236	0.601878463	0.663320474
981.0018	894.6037	943.1594	-0.07151	0.29215357	0.722021255
0	0	0	#NUM!	NA	NA
2241.652	2210.033	2515.092	1.197319	0.371493568	1.85992E-19
39.31155	37.15903	42.96615	-0.21057	0.38441957	0.727797788
0	0	0	#NUM!	NA	NA
499.4353	473.7776	578.4711	-0.26033	0.455280748	0.151699323
961.346	998.6489	970.4062	0.044361	0.792868376	0.843790109
1324.084	1228.106	1396.924	-0.04788	0.941098554	0.832900164
3790.884	3658.307	3143.865	0.210876	0.16816951	0.258978375
119.7215	120.7668	106.8914	-0.02515	0.507065266	0.965791283
1881.594	1762.267	1718.646	0.202115	0.03049807	0.365275241
3523.744	3456.719	3644.787	-0.19392	0.185152106	0.087076242
6482.832	6323.538	6678.617	-0.10105	0.914186189	0.483238043
62.5411	108.6902	134.1382	0.447863	0.598960113	0.398659502
683.4849	689.3	818.4528	-0.07727	0.32066134	0.755018053
13893.95	13035.39	16247.49	0.082043	0.827268091	0.666854583
771.9358	824.9305	795.3978	-0.19787	0.842806932	0.214649394
179.5823	134.7015	197.0155	0.068002	0.451912698	0.883280306
4402.893	4617.939	4160.381	-0.03712	0.031037731	0.859505388
6917.939	6568.788	6151.495	0.019861	0.003385807	0.921848549
2188.045	2252.766	2218.521	-0.07479	0.711479158	0.669238482
858.5999	832.3623	826.8364	-0.20415	0.733632007	0.181864152
768.3621	701.3767	761.8632	-0.07806	0.1975101	0.71014908

1051.584	1014.442	926.3921	0.051176	0.622651984	0.818409186
535.1731	471.9197	465.292	0.037809	0.617661583	0.908612163
17.86889	18.57952	23.05501	-0.47348	0.134982398	NA
544.1076	464.4879	477.8674	0.365619	0.134588876	0.25282015
9.827887	11.14771	17.81523	0.614052	0.965666941	NA
3329.867	3223.546	2913.315	0.026619	0.001792577	0.91372954
1447.38	1336.796	1234.491	0.058596	0.777280242	0.755018053
1465.249	1290.347	1150.654	0.041324	0.798439173	0.86344732
3231.588	3116.714	2473.174	0.196245	0.813397158	0.477547118
6035.216	5811.672	5657.908	-0.0245	0.000144604	0.881988452
7967.736	7768.095	7240.32	0.10354	0.014547812	0.575540189
0	3.715903	3.143865	-0.39341	NA	NA
0	0	0	#DIV/0!	NA	NA
1155.223	1190.018	1249.162	0.005104	0.14394509	0.988370025
913.1	914.1121	1044.811	-0.14758	0.172063056	0.40615036
1800.29	1915.548	1935.573	-0.00493	0.770144773	0.988996303
4255.475	4096.783	4387.787	-0.13836	0.550503835	0.351487828
1056.945	1099.907	1010.229	0.279432	0.105688589	0.178362257
1194.535	1182.586	1089.873	0.164796	0.200422675	0.356994802
311.812	317.7097	302.859	0.066269	0.941273396	0.851129209
1020.313	1030.234	993.4612	0.059419	0.406960002	0.770283785
285.0087	339.0762	293.4274	-0.09022	0.854046275	0.773113821
271.6071	310.2779	283.9958	-0.14667	0.944990108	0.551771495
1322.298	1277.342	1378.061	-0.14736	0.999122571	0.414929365
1056.051	1083.186	946.3033	0.097202	0.828379866	0.639253997
167.9675	157.9259	202.2553	-0.62048	0.086476182	0.00621807
149.2052	118.9089	102.6996	-0.25885	0.000791163	0.487331989
401.1565	415.2522	370.976	-0.3687	0.25618841	0.089560109
12.50822	5.573855	8.383639	0.231017	0.6212524	NA
2339.037	2434.845	2154.595	0.19414	0.268149097	0.321629539
0.893444	0	1.047955	-1.38183	NA	NA
600.3945	533.2321	641.3484	-0.29963	0.58195463	0.086239424
529.8124	455.1981	504.0663	-0.27391	0.005578646	0.129374309
490.5009	512.7946	504.0663	0.069129	0.960823113	0.806564543
403.8368	419.897	352.1128	0.128289	0.622518586	0.689648756
1004.231	1024.66	1079.394	0.104307	0.769710962	0.522768261
1878.02	2027.954	1649.481	0.279706	0.462231702	0.062642702
312.7055	299.1302	232.646	0.569529	0.859167103	0.081190592
663.8291	647.4961	673.835	-0.25199	0.362317042	0.127757998
832.69	793.3453	646.5882	0.224261	0.682715357	0.299903133
1048.01	974.4956	911.7208	0.180097	0.959739969	0.420331471
14.29511	17.65054	12.57546	0.168166	0.812875146	NA
781.7637	755.2573	713.6573	0.271861	0.834965572	0.258108467
749.5997	679.0813	750.3357	-0.21399	0.311697167	0.225630132
217.107	220.1673	226.3583	0.104697	0.989430108	0.769493959
968.4936	952.2002	1019.66	-0.13956	0.053161529	0.446206763
3420.998	3517.102	3940.31	0.136635	0.881060269	0.507147953
346.6564	450.5532	409.7504	-0.16357	0.953403647	0.578656471
50.92632	28.79825	57.63752	1.121818	0.365690431	NA
3044.858	3080.484	3201.502	-0.21472	0.798030968	0.163023482
7587.129	7633.394	7383.89	-0.04438	0.926756894	0.763891732
1827.987	1986.15	1703.975	0.261746	0.484196095	0.198030129
857.7065	814.7117	862.4669	0.047484	0.766887678	0.838085776
439.5746	497.931	404.5106	0.132083	0.837529432	0.691450677

336.8285	409.6783	365.7363	-0.24932	0.970055623	0.262359598
37717.64	36779.08	35759.36	0.169847	0.443017929	0.318281735
130.4429	150.4941	140.426	0.085293	0.803184945	0.875604624
1975.405	1894.182	1963.867	0.146904	0.021160717	0.38666632
288.5825	349.2949	367.8322	0.160532	0.811220717	0.726818972
752.2801	713.4534	660.2116	0.156246	0.906767387	0.487357424
356.4843	351.1528	369.9281	-0.1321	0.272100977	0.621324733
2988.571	2846.382	2952.089	-0.23231	0.071282701	0.225879379
238.5496	326.9995	342.6813	0.083463	0.246611129	0.859505388
394.9024	354.8687	332.2017	0.017284	0.714180196	0.955305678
4418.975	4639.305	5115.068	-0.13595	0.899176451	0.487331989
959.5591	922.4729	792.2539	0.350654	0.62974082	0.060691739
22700.63	23179.8	23671.21	-0.42059	0.745767703	0.012748693
356.4843	316.7807	410.7983	0.181776	0.962011634	0.520301247
2423.914	2714.467	2411.344	0.063866	0.998398411	0.708596546
4694.156	3997.383	4051.394	0.161339	0.056337167	0.505493145
4837.107	4743.35	5103.54	-0.64869	0.897759686	7.83084E-07
603.0749	600.1183	484.1552	0.097176	0.907095813	0.729565723
2465.906	2506.377	2522.427	0.163014	0.024577856	0.41069386
839.8376	811.9248	735.6643	0.152179	0.033849408	0.51433656
511.0501	432.9027	572.1834	-0.26705	0.730407446	0.229480039
1571.568	1591.335	1953.388	-0.19802	0.812875146	0.425191741
0	2.786927	0	-0.10793	NA	NA
64.32799	36.23005	62.87729	0.49169	0.792383666	0.414929365
91.13131	79.89192	55.54161	-0.48885	0.945870306	0.343544446
816.6081	955.9161	753.4796	0.298906	0.701216566	0.161220563
300.1973	320.4966	222.1664	0.02042	0.597146011	0.971161723
11361.04	11549.03	11156.53	-0.08536	0.077745036	0.357987739
3319.145	3150.157	3017.062	0.133511	0.917703668	0.342128545
2655.316	2432.988	2397.721	0.19713	0.577332911	0.147355085
248.3775	252.6814	194.9196	0.711227	0.526006956	0.007323791
221.5742	151.423	177.1044	-0.72477	0.311933404	0.0104057
546.7879	591.7576	530.2652	0.014151	0.738468335	0.964158522
559.2961	503.5049	408.7024	0.10607	0.854862911	0.729059351
80.40998	78.03396	90.12412	-0.11021	0.585762809	0.859199438
259.0988	270.3319	280.8519	-0.61828	4.64116E-13	0.060691739
234.0824	217.3803	294.4753	-0.06535	0.572336545	0.838059844
1516.175	1463.137	1254.402	0.229305	0.570003668	0.299395231
754.9604	788.7004	754.5275	-0.12848	0.311819514	0.494493869
5.360666	5.573855	11.5275	0.378245	NA	NA
739.7718	705.0926	726.2327	0.111499	0.735300868	0.616511903
1232.953	1323.79	1157.99	0.319674	0.429368942	0.098098196
823.7556	825.8595	864.5628	-0.06876	0.27470085	0.742276559
81.30343	62.24138	76.50071	0.075281	0.384189305	0.923725803
0	0	0	#NUM!	NA	NA
1104.297	1089.689	1229.251	0.099613	0.804410815	0.660222897
12493.92	12948.06	13688.39	-0.1367	0.921802179	0.489096476
285.9022	328.8574	320.6742	0.049626	0.35165001	0.875604624
139.3773	143.0623	198.0635	0.33439	0.045192099	0.382141979
4488.664	4342.033	4949.491	0.019694	0.447580006	0.948574402
16642.19	16703.91	17503.99	-0.45152	0.982305383	0.001735941
6964.398	7054.642	7275.951	-0.01915	0.730204074	0.908612163
1532.257	1491.935	1451.418	0.056527	0.547416205	0.814086627
830.0097	834.2202	816.3569	0.135344	0.232757941	0.398655382

1782.421	1849.591	1964.915	0.073327	0.234799471		0.653821279
0	2.786927	2.09591	-1.52678	NA	NA	
742.4522	871.3793	929.536	-0.18684	0.254252351		0.383759687
1721.667	1773.415	1695.591	0.121854	0.556307283		0.487357424
32351.62	31433.75	31734.17	-0.30134	0.755573717		0.007579991
825.5425	851.8708	813.213	0.262243	0.285086597		0.36518668
801.4195	751.5414	924.2962	-0.35502	0.355572646		0.027769084
393.1155	315.8518	283.9958	0.496124	0.085195908		0.088369497
1756.511	1758.551	1610.707	0.080103	0.340035461		0.675143077
936.3296	819.3566	774.4387	0.289357	0.323780681		0.200725526
457.4435	414.3232	410.7983	-0.00606	0.226105917		0.990142758
318.0662	399.4596	356.3047	-0.16757	0.296056707		0.502183544
841.6245	953.1291	1023.852	-0.00441	0.723947258		0.990142758
681.698	604.7632	726.2327	0.258625	0.648256394		0.183989501
1125.74	1083.186	1272.217	0.055394	0.556706854		0.85050204
763.8948	626.1297	669.6432	0.12852	0.623348614		0.664215073
698.6734	638.2063	570.0875	0.190964	0.642246351		0.306279885
704.0341	738.5357	828.9323	-0.13415	0.992476114		0.472093197
544.1076	532.3031	461.1002	0.129774	0.853320493		0.600875554
676.3373	740.3937	840.4598	-0.00918	0.147125509		0.973002234
0.893444	2.786927	0	-1.23132	NA	NA	
1842.282	1747.403	1565.645	0.239964	0.211449188		0.161220563
1199.896	1184.444	1085.681	0.188336	0.787792061		0.438623687
1733.282	1925.767	1863.264	-0.00974	0.596065856		0.965253376
1203.469	1094.333	1109.784	0.086411	0.6207623		0.656368654
360.058	357.6557	402.4147	-0.07279	0.728952588		0.811374268
92.9182	66.88625	88.02821	-0.13973	0.804201321		0.840718213
626.3044	661.4307	618.2934	0.192072	0.606270396		0.360674621
658.4684	857.4446	663.3554	0.210641	0.200039625		0.472093197
4514.574	4246.348	4287.183	0.008045	0.005036606		0.971658868
499.4353	459.843	499.8745	-0.04133	0.947037414		0.864252513
520.878	402.2465	505.1143	-0.15095	0.904254847		0.49318083
1798.503	1711.173	1749.037	0.204273	0.282011296		0.361564535
2530.234	2317.795	2329.604	0.208608	0.976681492		0.104998235
3273.58	3308.083	3204.646	-0.03713	0.000148829		0.819004
585.206	586.1837	543.8886	0.347223	0.394915233		0.060851524
508.3698	516.5105	403.4626	0.071876	0.675005225		0.856558496
2852.768	2676.379	2849.389	0.13255	0.905394249		0.329925488
1990.594	1916.477	1986.922	-0.05975	0.897759686		0.724404377
1619.814	1503.083	1566.693	0.075104	0.665579954		0.727738403
1083.748	1164.007	1399.02	-0.47834	0.466138921		0.001735941
2507.005	2479.436	2075.999	0.180488	0.362090257		0.38666632
9.827887	22.29542	10.47955	-0.26572	0.357841712	NA	
180.4757	174.6474	171.8646	-0.11069	0.682715357		0.774230303
172.4347	154.21	176.0564	0.053546	0.240910061		0.884087376
712.9685	619.6268	620.3893	0.303392	0.779325825		0.149286183
6431.012	6736.932	7002.435	-0.12284	0.991136341		0.365235491
3524.638	3767.926	4158.285	-0.06236	0.582003857		0.746698677
509.2632	557.3855	427.5656	0.321129	0.245394561		0.244704905
149.2052	165.3577	149.8575	-0.26403	0.926756894		0.422116695
454.7631	394.8147	446.4288	0.113452	0.885365393		0.656368654
243.0168	234.1019	275.6121	-0.24366	0.985859213		0.426226797
812.1408	847.2259	762.9112	0.163723	0.674403807		0.381727979
375.2466	353.9398	429.6615	0.22186	7.81293E-07		0.431825344

1127.527	1196.521	1191.525	0.161026	0.478308661	0.389216061
967.6001	890.8878	1078.346	0.031292	0.410903637	0.902851746
765.6817	831.4333	813.213	-0.06706	0.088789496	0.791888862
939.0099	954.0581	942.1114	-0.0025	0.732140966	0.997242119
1794.036	1752.977	1909.374	-0.01073	0.718383563	0.961026715
676.3373	729.246	678.0268	0.057891	0.384584076	0.830593197
14404.11	14090.7	15986.55	0.476241	0.093120159	0.000106385
201.9184	175.5764	208.543	0.487918	0.732140966	0.10999387
7.147554	9.289758	5.239774	0.640168	0.365953812	NA
12276.82	12330.3	13401.25	-0.25338	0.844410461	0.04168446
582.5257	474.7066	433.8533	0.35764	0.373700455	0.167165088
138.4839	143.9912	140.426	-0.28051	0.941323092	0.575546698
0	0	0	#NUM!	NA	NA
1827.987	1686.091	1812.962	0.158455	0.910682739	0.372768179
1181.133	1094.333	954.6869	0.12126	0.691366556	0.5608115
3089.53	2963.433	2963.616	-0.01327	0.179898822	0.930336535
106.3199	98.47143	105.8434	-0.2132	0.852550008	0.659046549
184.943	192.298	234.7419	0.019845	0.996328617	0.966975973
7.147554	8.360782	4.19182	0.758588	0.774889037	NA
421.7057	483.0674	486.2511	-0.08628	0.935100162	0.741928459
96.49198	150.4941	137.2821	-0.5441	0.918426622	0.129705424
1579.609	1752.048	1725.982	0.000472	0.490249604	0.999675887
1110.551	1281.987	1216.676	-0.15158	0.853195053	0.422560599
4176.852	4165.527	4244.217	-0.0052	0.036897698	0.971658868
1422.363	1386.961	1276.409	0.404984	0.042369293	0.002256075
430.6401	366.0165	489.3949	-0.16991	0.77572403	0.513869895
0	0	0	#DIV/0!	NA	NA
597.7142	571.3201	451.6686	0.163422	0.111187725	0.486822267
1888.741	1911.832	1858.024	0.178999	0.842165531	0.244742241
2598.136	2691.243	2773.937	0.832209	0.233378357	2.4117E-09
867.5344	874.1662	796.4457	0.18583	0.767300341	0.483184001
705.821	811.9248	748.2398	-0.09221	0.935960933	0.649685593
1335.699	1215.1	1273.265	0.278113	0.799313452	0.159445896
176.902	207.1616	209.591	-0.24583	0.80496489	0.422116695
35.73777	13.00566	32.4866	0.195705	0.937423565	NA
0.893444	2.786927	7.335684	0.231999	NA	NA
477.9927	461.701	622.4852	-0.25865	0.880275635	0.318625647
654.8946	645.6382	626.677	-0.10041	0.340833229	0.59853578
2879.571	2780.424	2486.797	0.250252	0.119516174	0.247256105
547.6813	554.5985	459.0042	-0.00658	0.631766123	0.988370025
630.7716	659.5728	553.3202	0.194717	0.739610643	0.381582724
601.288	588.0417	554.3681	-0.09921	0.792868376	0.684119849
2003.102	2039.102	1936.621	0.092948	0.459794863	0.669238482
243.0168	190.44	223.2144	-0.29125	0.465047544	0.315911601
595.0339	651.212	557.512	-0.06786	0.007990209	0.79699678
2110.315	2103.201	1924.045	0.206349	0.670790001	0.222710488
315.3858	358.5846	322.7701	-0.02353	0.458953645	0.941511899
1350.888	1512.373	1593.939	-0.09778	0.736846903	0.690294956
21850.97	20466.27	22424.14	0.183331	0.835682217	0.089171213
1159.691	1007.01	962.0226	0.306043	0.140612719	0.327516537
969.387	1170.509	899.1453	0.325368	0.345313334	0.22952299
730.8374	716.2403	604.67	0.205225	0.927256678	0.399254174
311.812	368.8034	428.6136	0.12601	0.639085319	0.694075461
431.5336	431.9737	440.1411	0.298299	0.107676441	0.370304803

769.2555	725.5301	720.993	0.166727	0.033849408	0.320519165
657.575	806.351	716.8011	-0.09541	0.812875146	0.659046549
377.9269	281.4797	316.4824	-0.10788	0.550503835	0.780325172
6.25411	6.50283	7.335684	1.741989	NA	NA
3948.13	3748.417	4031.482	0.02077	0.834965572	0.942591831
1178.453	1235.538	1083.585	-0.21066	0.484625827	0.193131605
2866.169	2858.458	3264.379	0.054497	0.458486853	0.778929671
574.4847	618.6979	692.6982	-0.24431	0.215286681	0.238463934
1549.232	1641.5	1665.2	-0.25406	0.470002734	0.015466264
106.3199	72.46011	82.78844	-0.82775	0.936879535	0.018151008
70.5821	67.81523	66.02116	-0.87026	0.000217366	0.011236162
10950.05	11230.39	11207.88	-0.22983	0.840099446	0.144365513
2851.874	2767.419	2883.972	-0.03395	0.677154401	0.830385228
28.59022	18.57952	25.15092	0.285657	0.947783958	NA
4.467221	8.360782	18.86319	-0.46627	0.748298735	NA
2265.775	2325.226	2221.664	-0.04442	0.004702869	0.832722668
4787.074	4883.626	4711.605	-0.10003	0.990604058	0.58052356
3314.678	3289.503	3150.152	0.053247	0.528927394	0.76917002
282.3284	278.6927	285.0437	-0.2455	0.857493953	0.273534982
3049.325	2971.793	3061.076	0.018413	0.329402028	0.914772543
550.3617	528.5872	623.5332	-0.00709	0.731963541	0.984456511
4287.639	4456.297	5500.715	-0.24711	0.812875146	0.163640813
543.2141	411.5363	451.6686	0.223574	0.962660515	0.386078627
427.0664	526.7293	439.0931	-0.12135	0.190992184	0.622775043
1432.191	1352.589	1178.949	0.385708	0.179898822	0.131512208
1.786889	2.786927	0	#DIV/0!	NA	NA
503.0091	502.5759	562.7518	-0.5014	0.051978056	0.002570942
824.6491	839.7941	645.5402	0.226056	0.682715357	0.292916323
573.5912	441.2635	523.9774	-0.20618	0.948234757	0.379367723
1410.748	1270.839	1575.076	-0.00396	0.92288341	0.990142758
1082.854	1272.697	1287.937	-0.32173	0.603004299	0.023502597
461.9107	479.3515	445.3808	0.070119	0.509454999	0.840718213
1023.887	1044.169	924.2962	0.143955	0.394915233	0.578237014
580.7388	541.5929	588.9507	0.036149	0.79077061	0.884746998
254.6316	185.7952	209.591	-0.22794	0.46334755	0.465657685
1977.192	2066.042	1899.942	-0.11034	0.231513117	0.388495658
8.040998	8.360782	9.431594	-1.56135	0.783760899	NA
1690.397	1571.827	1563.549	0.218308	0.959739969	0.178822023
997.9772	980.9984	1017.564	-0.01831	0.992476114	0.940307062
23980.94	24236.05	27071.82	-0.48672	0.824080056	0.001357562
906.8459	901.1065	935.8237	-0.18039	0.203841572	0.379845684
815.7146	909.4673	925.3442	-0.17776	0.93284858	0.335921009
1557.273	1548.603	1665.2	-0.0323	0.574512226	0.875929853
1515.281	1354.447	1261.738	0.393044	0.424996881	0.102617483
1738.643	1713.031	1450.37	0.058173	0.221695862	0.817988115
438.6811	494.2151	426.5176	0.126276	0.918350023	0.627713216
404.7302	425.4709	450.6206	0.055133	0.777781833	0.836257824
5141.772	5069.421	5114.02	-0.10146	0.258431804	0.387635019
3.573777	9.289758	4.19182	-1.43791	0.937354907	NA
99.17231	121.6958	107.9394	0.041343	0.774889037	0.93327699
4.467221	3.715903	4.19182	0.618093	0.429368942	NA
1251.715	1361.878	1451.418	-0.21757	0.867574132	0.27042216
1195.428	1134.279	1028.044	0.10522	0.218571331	0.627713216
1646.618	1521.662	1672.536	0.118307	0.179898822	0.579998211

343.976	264.7581	250.4612	0.87979	0.539046349		0.009996213
202.8118	238.7468	243.1255	-0.15356	0.367802825		0.612574138
292.1563	357.6557	301.811	-0.04494	0.713081901		0.896567419
409.1975	442.1925	448.5247	0.030295	0.00565249		0.931618549
0	0	0	#DIV/0!	NA	NA	
820.1818	756.1863	733.5684	0.264912	0.948896953		0.271247752
748.7063	673.5074	779.6784	0.086521	0.00602068		0.672998339
11956.07	11562.03	11874.38	0.393686	0.957754117		2.43041E-05
339.5088	315.8518	321.7222	-0.42909	0.337999896		0.027816164
338.6154	380.8801	329.0578	0.163687	0.228848778		0.549038919
4.467221	0.928976	2.09591	-0.29924	NA	NA	
1.786889	0	0	#DIV/0!	NA	NA	
16.082	33.44313	42.96615	0.2442	0.873415892	NA	
852.3458	814.7117	624.5811	0.25573	0.901132408		0.294052191
8297.417	8752.81	8025.239	0.008289	0.310443697		0.964000071
1028.354	936.4076	901.2412	0.257589	0.2795135		0.360674621
373.4597	434.7607	320.6742	0.163013	0.579415204		0.552800466
610.2224	546.2377	510.354	0.250101	0.58004353		0.325477034
50.03288	50.16469	96.41185	-0.21474	0.874036182		0.831655346
146.5249	164.4287	180.2482	0.147036	0.966586541		0.734906514
788.9113	948.4843	869.8026	0.070319	0.081224331		0.746662168
1105.191	1044.169	1010.229	-0.17097	0.303314321		0.311826056
654.8946	629.8456	888.6657	-0.4077	0.94035236		0.030371149
0	1.857952	0	#DIV/0!	NA	NA	
552.1486	517.4395	559.6079	-0.00216	0.960956671		0.991339835
557.5092	498.86	565.8956	0.165709	0.864963799		0.477770886
838.9442	794.2743	759.7673	-0.06762	0.980409444		0.794422811
1701.118	1723.25	1620.138	-0.39609	0.179898822		0.000129741
2529.341	2485.01	2292.925	0.103014	0.725429407		0.551050851
426.1729	433.8317	531.3131	0.065479	0.941098554		0.864290969
620.9438	613.124	564.8477	0.120553	0.903271991		0.655129754
14.29511	18.57952	8.383639	-0.35434	0.935163971	NA	
2783.972	2578.837	2641.894	-0.04876	0.172896395		0.797601985
587.8863	676.2944	574.2793	0.071186	0.482562486		0.803989183
1515.281	1471.498	1301.56	0.035149	0.556307283		0.883471187
637.9192	692.0869	696.89	-0.11352	0.98843748		0.579998211
552.1486	694.8739	602.5741	0.04695	0.482562486		0.886786965
1122.166	1018.157	1009.181	0.182088	0.113562986		0.311435996
2993.932	2969.007	3032.781	-0.11529	0.567906243		0.422793542
289.4759	315.8518	295.5233	-0.05662	0.484625827		0.872630949
2093.34	2168.229	2110.581	-0.04846	0.365953812		0.774121067
7178.825	6734.145	6572.773	0.192913	0.208221128		0.134770011
1097.15	1055.316	1083.585	0.163834	0.164920979		0.313958096
806.7802	852.7997	949.4471	-0.24127	0.785492922		0.164384321
32.16399	17.65054	20.9591	-0.16018	0.918350023	NA	
3.573777	0	6.287729	0.016542	0.761181704	NA	
528.919	601.0473	586.8547	0.01343	0.876058554		0.975879427
4.467221	4.644879	10.47955	-0.73765	0.899176451	NA	
140.2707	167.2156	127.8505	-0.05416	0.996781128		0.919055186
547.6813	583.3968	651.8279	-0.28304	0.208221128		0.104692027
1716.306	1620.134	1613.851	-0.05963	0.831317845		0.700302444
982.7887	829.5754	709.4655	0.117311	0.146572219		0.687575419
2.680333	3.715903	3.143865	0.381405	NA	NA	
2184.471	2231.4	2051.896	0.055267	0.006092421		0.722021255

343.976	347.4369	244.1735	0.07419	0.212716004	0.815439094
2611.538	2599.274	2609.408	-2.4E-05	0.830656714	0.99985654
2974.276	3085.129	2861.965	0.11463	0.639085319	0.489944774
510.1567	472.8487	463.1961	0.108975	0.795709704	0.638657543
1147.182	1097.12	1026.996	0.137945	0.2795135	0.469102863
446.7221	588.9706	485.2031	-0.08545	0.320543105	0.764793691
22.33611	22.29542	19.91114	-0.11001	0.542904501	NA
737.985	826.7884	690.6023	-0.10356	0.567589507	0.643988563
561.9764	491.4282	565.8956	-0.04902	0.235461691	0.87417937
1073.027	1123.132	1040.619	0.176823	0.925203985	0.348491003
2265.775	1932.27	1722.838	-0.0048	0.651388825	0.990142758
1216.871	1292.205	1211.436	0.09019	0.202320361	0.646200858
404.7302	435.6896	491.4908	-0.02904	0.017639055	0.907473183
1098.043	1230.893	1160.086	0.168236	0.997889853	0.294466305
435.1074	336.2892	303.9069	-0.09286	0.981762093	0.806564543
251.0578	208.0906	212.7348	-0.10736	0.478531557	0.802920813
2284.537	2352.167	2256.247	0.375976	0.053912087	0.000421029
844.3048	786.8425	863.5148	-0.20687	0.750700436	0.220273588
52669.43	50894.87	52012.1	-0.25474	0.973772216	0.058966813
14.29511	19.50849	4.19182	1.136582	0.989430108	NA
627.1979	642.8512	618.2934	0.079684	0.853477462	0.746898731
377.9269	340.9341	297.6192	0.248431	0.720425175	0.391432089
3001.973	3114.856	3153.296	-0.14956	0.565106682	0.211432205
1260.65	1203.953	1199.908	0.161916	0.580937291	0.350427789
963.1329	1048.814	1197.812	-0.50236	0.025591463	0.000192079
8080.31	7647.328	9411.683	-0.07149	0.894177375	0.876430377
574.4847	593.6155	572.1834	-0.61145	0.275308123	0.000109324
92.02476	57.5965	71.26093	-0.13522	0.839922799	0.818645356
920.2476	971.7086	870.8505	-0.233	0.668508083	0.186989586
539.6403	590.8286	720.993	-0.24229	0.926795891	0.321405678
2.680333	4.644879	2.09591	#DIV/0!	NA	NA
2190.725	2259.269	2128.396	0.087112	0.187777748	0.600823565
32144.34	31573.1	32594.54	-0.40475	0.905623996	0.001866776
400.263	434.7607	420.2299	0.04973	0.798030968	0.864363898
92.9182	114.264	100.6037	-0.30653	0.210289554	0.434560808
50.92632	39.94596	36.67842	-0.238	0.774889037	0.684679019
467.2713	553.6696	520.8336	-0.07212	0.766887678	0.832624087
11.61478	15.79259	22.00705	-0.17938	0.842190796	NA
0	3.715903	0	-1.08851	NA	NA
1032.822	1075.754	985.0776	0.184103	0.828016939	0.406655479
573.5912	529.5162	553.3202	0.077045	0.91675117	0.809991249
357.3777	366.0165	366.7842	-0.01244	0.858869281	0.984451848
562.8699	532.3031	682.2186	-0.07697	0.079395817	0.788938862
1804.757	1795.71	1635.858	0.15333	0.751065001	0.268230461
10.72133	1.857952	2.09591	-0.26638	0.766887678	NA
480.673	419.897	439.0931	0.081414	0.766887678	0.739592868
22538.92	23205.81	24363.9	-0.43687	0.974700015	0.001866776
9377.591	9312.053	8438.133	-0.06575	0.185152106	0.647426135
42.88532	47.37776	58.68547	0.189387	0.46334755	0.778166558
2046.881	2200.744	2533.955	-0.47972	0.947037414	0.000493703
1.786889	0	6.287729	-2.34034	NA	NA
800.5261	676.2944	670.6911	0.094796	0.29215357	0.726171034
662.9356	752.4704	602.5741	0.115519	0.817609984	0.62247661
3.573777	10.21873	12.57546	-0.60844	0.300155248	NA

595.9273	552.7406	504.0663	-0.08524	0.839922799	0.712929046
3.573777	0	0	-1.10614	NA	NA
6053.978	6109.874	5991.158	-0.01442	0.987610613	0.938440797
2628.513	2613.209	2303.405	0.093768	0.126687185	0.755018053
860.3868	807.2799	682.2186	0.171156	0.577548143	0.380365797
1055.158	945.6973	977.7419	0.002434	0.249113533	0.992729462
259.0988	301.9171	260.9408	-0.1708	0.542689845	0.578046344
85.77065	117.0509	120.5148	-0.22442	0.923364203	0.658878607
3149.391	3188.245	2587.401	0.021012	0.870947007	0.956290296
406.5171	364.1585	308.0987	0.360448	0.73478662	0.298450788
682.5914	731.1039	533.409	0.282432	0.237605221	0.30206153
423.4926	457.9851	474.7236	0.144436	0.964929778	0.538616173
223.3611	199.7298	204.3512	-0.01553	0.838103038	0.974467077
0	0.928976	0	#DIV/0!	NA	NA
1518.855	1608.057	1615.946	0.030288	0.812875146	0.891918558
1499.199	1627.566	1706.071	-0.15768	0.848694292	0.365235491
4648.59	4455.368	4800.681	-0.20154	0.600642134	0.112510644
545.8944	572.2491	547.0325	0.091211	0.80496489	0.69424407
645.0668	627.0586	543.8886	0.108579	0.862447485	0.577717703
824.6491	1032.092	793.3019	0.161431	0.256151839	0.638645387
1125.74	1109.197	943.1594	0.287704	0.626260019	0.209280914
624.5175	565.7462	471.5797	0.316646	0.452580134	0.362764905
756.7473	644.7092	647.6361	0.104378	0.817609984	0.599528544
36.63121	57.5965	35.63047	0.286942	0.956677576	0.705641462
246.5906	255.4683	212.7348	0.157067	0.855563032	0.686015779
2163.922	2021.451	1670.44	0.234878	0.405505718	0.419821619
14.29511	9.289758	6.287729	-0.83593	0.175067813	NA
6008.413	5877.63	6751.973	-0.37963	0.125178937	0.001988814
1582.29	1661.009	1895.75	0.015228	0.426735737	0.957059222
621.8372	675.3654	563.7997	0.051516	0.072365168	0.872630949
143.8445	145.8492	192.8237	-0.09533	0.036671148	0.836904079
4200.975	4432.143	4364.732	0.058806	0.28625807	0.686712135
0.893444	0	0	-1.74916	NA	NA
264.4595	286.1245	224.2623	-0.01565	0.263755993	0.975406172
8.040998	7.431806	12.57546	0.314804	0.892614393	NA
6817.873	6622.668	7615.488	-0.14479	0.854862911	0.292916323
15209.1	15614.22	18740.58	-0.45118	0.732718278	0.001157422
0	0	1.047955	-0.32968	NA	NA
219.7873	187.6531	204.3512	-0.02625	0.767300341	0.966975973
1086.428	1077.612	851.9873	0.265523	0.420515039	0.268588059
0	0	0	#DIV/0!	NA	NA
30.3771	13.00566	15.71932	0.093883	0.152084817	NA
188.5167	182.0792	137.2821	0.125659	0.53128891	0.779599442
0	0	0	#DIV/0!	NA	NA
125.9756	72.46011	106.8914	0.422729	0.164920979	0.357079822
1.786889	3.715903	1.047955	-1.90925	0.938838346	NA
3397.769	3139.009	3178.447	0.004832	0.000656764	0.988943755
671.8701	611.2661	741.9521	0.047384	0.967813477	0.855770814
458.3369	467.2748	624.5811	-0.41161	0.826883467	0.066899278
29.48366	46.44879	40.87024	-0.25376	0.919200531	NA
79.51654	46.44879	57.63752	0.150658	0.910682739	0.806564543
1019.42	1078.541	1089.873	-0.07839	0.813154117	0.75448001
10.72133	0.928976	7.335684	-0.85557	0.875830579	NA
346.6564	445.9084	490.4429	0.464603	0.425097125	0.038101932

3597.9	3639.727	3410.045	0.087589	0.949259474	0.563375937
29.48366	16.72156	26.19887	1.036314	0.810961861	NA
284.1153	249.8945	293.4274	-0.25092	0.095644436	0.244704905
898.8049	1018.157	880.2821	-0.16561	0.51970267	0.40615036
48.24599	26.01132	48.20593	0.111042	0.02107346	0.881988452
963.1329	812.8538	823.6925	0.385266	0.55438266	0.096729284
1142.715	981.9274	796.4457	0.157059	0.985859213	0.56984254
199.2381	158.8549	232.646	-0.19552	0.813154117	0.566141749
691.5259	634.4904	648.6841	0.134479	0.079089299	0.477547118
5151.6	5277.511	5518.53	0.098786	0.136084466	0.397066014
452.9762	405.9624	433.8533	0.099885	0.649302498	0.736452263
713.862	731.1039	651.8279	0.094348	0.050113237	0.711465473
711.1816	648.4251	599.4302	0.355577	0.695398138	0.060227128
595.9273	609.4081	621.4373	0.050117	0.337335124	0.832306222
5894.052	5718.775	6151.495	-0.10032	0.758716249	0.59488868
537.8534	587.1127	500.9224	0.348345	0.310443697	0.193779401
1073.027	1132.421	1238.683	0.025513	0.931326642	0.919915102
168.861	162.5708	150.9055	-0.04849	0.106548765	0.914120833
620.9438	642.8512	537.6009	-0.03088	0.967813477	0.914553818
1446.486	1566.253	1484.952	-0.05706	0.759261525	0.797980755
752.2801	726.459	658.1157	0.329357	0.29215357	0.178507885
974.7477	923.4019	833.1241	0.208446	0.104651539	0.395957024
1897.676	1960.139	1998.45	-0.03176	0.550503835	0.907473183
849.6655	816.5697	886.5698	0.24324	0.410827034	0.178822023
0.893444	0	1.047955	#DIV/0!	NA	NA
310.0252	232.2439	256.7489	0.137701	0.914747104	0.681152522
3975.827	3742.843	4105.887	0.014704	0.901380807	0.93327699
584.3125	743.1806	608.8618	0.032103	0.773823303	0.91372954
645.0668	614.053	766.055	-0.34866	0.465047544	0.078157207
2854.554	2604.848	2450.119	0.192664	0.235669657	0.241819205
7924.851	8018.919	7824.031	0.293983	0.052564266	0.000300707
347.5498	307.491	291.3315	-0.03482	0.812875146	0.919915102
7811.383	7383.499	8411.934	0.273276	0.98843748	0.101367547
1242.781	1202.095	1039.571	0.139813	0.02630298	0.596309029
2329.209	2311.292	2531.859	0.01413	0.572669463	0.952666818
1940.561	1938.772	1703.975	0.17386	0.155062297	0.324099572
349.3367	392.9567	386.6954	-0.07723	0.768800795	0.791888862
102.7461	135.6305	116.323	0.292632	0.690152662	0.558104763
692.4193	634.4904	613.0536	0.014016	0.897898013	0.946922147
1255.289	959.632	935.8237	0.138079	0.504924333	0.575540189
8284.909	8182.419	8944.295	-0.22449	0.552597953	0.039208875
2989.464	2668.018	3006.583	-0.09409	0.225501117	0.594065295
15.18855	6.50283	12.57546	-0.79194	0.485973657	NA
5336.543	5519.045	6189.222	-0.09068	0.249815953	0.596309029
192.0905	137.4884	266.1805	-0.44643	0.961125122	0.207300547
829.1163	805.422	747.1918	0.315714	0.18468856	0.133980325
509.2632	459.843	390.8872	0.069661	0.755224646	0.835596295
24480.37	25001.52	25946.32	-0.36451	0.792868376	0.010243246
11.61478	6.50283	8.383639	0.270426	0.826883467	NA
277.8612	337.2182	295.5233	-0.2703	0.673772903	0.280613898
2968.915	2655.942	2532.907	0.091321	0.054776927	0.741187433
11.61478	15.79259	14.67137	-0.88905	0.578452627	NA
0	1.857952	0	-1.50354	NA	NA
130.4429	171.8605	153.0014	0.110345	0.268149097	0.791888862

10524.77	10990.71	12905.56	0.29051	0.917013507	0.116048789
138.4839	118.9089	111.0832	-0.21391	0.609539912	0.579845143
41.09844	31.58518	44.01411	0.643287	0.562841483	0.303824089
1879.807	1913.69	1881.079	-0.14883	0.034945647	0.343544446
398.4761	325.1415	420.2299	0.497087	0.092640355	0.060227128
321.6399	287.9825	297.6192	0.115475	0.944990108	0.721329001
487.8206	524.8713	596.2863	0.037433	0.46017679	0.884584155
250.1644	220.1673	207.4951	0.108043	0.972453208	0.739592868
586.0994	641.9223	608.8618	0.366492	0.707092679	0.024833343
7.147554	0.928976	8.383639	0.112457	0.79543317	NA
96.49198	106.8322	100.6037	-0.24842	0.27470085	0.509553026
174.2216	202.5167	190.7278	0.190609	0.2795135	0.602240104
210.8528	209.0195	247.3174	-0.15155	0.856763291	0.708596546
420.8122	514.6526	515.5938	0.009492	0.732140966	0.984451848
906.8459	883.4559	863.5148	-0.16097	0.754651689	0.481380095
0.893444	1.857952	1.047955	-1.94685	NA	NA
27.69677	28.79825	32.4866	0.470302	0.482847539	NA
243.9103	258.2553	250.4612	0.312389	0.936346196	0.17898966
4379.664	4191.539	4487.343	-0.07656	0.666493976	0.535992604
0	0	0	#DIV/0!	NA	NA
16.97544	24.15337	19.91114	-0.80282	0.614621507	NA
237.6562	227.5991	216.9267	0.223088	0.546270318	0.370883338
1407.175	1342.37	1359.197	-0.06609	0.990064993	0.778199039
1.786889	0	2.09591	#DIV/0!	NA	NA
1.786889	0	2.09591	#DIV/0!	NA	NA
2051.348	2142.218	2167.171	-0.07891	0.701216566	0.578074656
301.0907	278.6927	259.8928	-0.29397	0.000661553	0.162162087
0	0	0	#NUM!	NA	NA
875.5754	931.7627	1028.044	-0.0279	0.897766774	0.908442874
4244.754	4238.916	4156.189	0.07185	0.779325825	0.533639004
3261.965	2903.049	2994.007	0.071762	0.045192099	0.756407952
478.8861	536.019	586.8547	-0.30217	0.518108649	0.11250302
550.3617	537.877	477.8674	-0.12674	0.962011634	0.545985077
896.1246	882.527	1129.695	0.063186	0.986203512	0.798013277
1031.035	1043.24	1134.935	0.132377	0.814880408	0.536590083
289.4759	290.7694	324.866	-0.6662	0.681216827	0.000370485
5.360666	2.786927	4.19182	0.752602	NA	NA
975.6411	1047.885	910.6728	0.071607	0.906767387	0.686859684
0	3.715903	0	#DIV/0!	NA	NA
915.7804	950.3422	1141.223	0.049732	0.710373411	0.835596295
284.1153	315.8518	381.4556	-0.20724	0.828016939	0.536664638
674.5504	614.982	617.2454	0.00283	0.614563272	0.996992829
6029.855	6285.45	6360.038	-0.2912	0.948896953	0.042689756
234.9758	303.7751	273.5162	0.040111	0.617661583	0.905897923
6684.75	6416.436	6191.318	-0.01582	0.088704246	0.924154584
800.5261	831.4333	692.6982	0.189419	0.918350023	0.341785585
185.8364	247.1076	207.4951	0.487929	0.537944371	0.089456731
0	0	0	#DIV/0!	NA	NA
5797.56	6160.967	5342.474	0.042848	0.017930836	0.826228143
812.1408	827.7174	798.5416	0.18447	0.03968658	0.359566588
262.6726	308.42	342.6813	-0.46945	0.821754986	0.102175745
4233.139	4399.629	4375.212	0.01019	0.263437821	0.952588397
863.0672	759.9022	813.213	0.088252	0.826883467	0.690294956
846.0917	962.4189	943.1594	-0.04383	0.902367847	0.864252513

8288.482	8517.779	10173.55	-0.57704	0.750700436		3.13602E-05
1038.182	1130.564	956.7828	-0.10856	0.788592496		0.578237014
3.573777	1.857952	2.09591	0.193012	NA	NA	
221.5742	228.528	291.3315	0.17458	0.171568235		0.656215943
8180.376	7830.337	8433.941	-0.24682	0.980754424		0.062514752
424.386	413.3942	373.0719	0.027425	0.198059489		0.931542231
2616.005	2865.89	2509.852	0.12795	0.585762809		0.350286088
5923.535	5832.11	5808.814	-0.06402	0.002758864		0.566742383
9189.968	8864.287	8030.478	0.129179	0.004218142		0.494493869
343.976	479.3515	459.0042	-0.33988	0.210289554		0.18366757
93.81165	66.88625	76.50071	0.204357	0.412665866		0.730853476
5166.788	4787.012	4925.388	0.061651	0.857370872		0.680262772
3014.481	3029.39	3446.724	-0.79482	0.5124279		2.89763E-07
595.0339	718.0983	701.0818	0.150762	0.957697061		0.505302546
33.95088	16.72156	27.24683	0.47891	0.466642335	NA	
4236.713	4259.354	4354.253	-0.04162	0.795376015		0.836904079
87.55754	88.2527	96.41185	0.183004	0.879019047		0.709925559
622.7306	542.5218	687.4584	-0.118	0.009557355		0.753881265
1067.666	1131.492	1085.681	0.332839	0.431874361		0.020934062
2346.185	2277.849	2558.058	-0.10634	0.953403647		0.539224916
417.2385	423.6129	428.6136	-0.02972	0.743600406		0.924895748
324.3203	287.0535	290.2835	0.075257	0.734144448		0.84358525
18.76233	17.65054	29.34274	0.58148	0.918350023	NA	
1.786889	17.65054	4.19182	-0.61099	0.471685453	NA	
1208.83	1133.35	1230.299	-0.10198	0.61781651		0.607746892
938.1165	947.5553	936.8717	-0.25695	0.972861407		0.05827215
753.1735	855.5867	926.3921	-0.22852	0.408488796		0.283703282
8.934443	1.857952	8.383639	0.674627	NA	NA	
0	3.715903	2.09591	-0.13372	NA	NA	
30.3771	26.9403	23.05501	-0.29628	0.284504666	NA	
2019.184	1998.227	1711.31	0.193212	0.80496489		0.267586812
1689.503	1572.756	1501.719	0.132445	0.717053318		0.595440724
1123.953	1190.018	1025.948	0.165465	0.177527106		0.310947968
848.772	874.1662	716.8011	0.053985	0.990604058		0.836257824
34413.69	33523.95	34225.16	-0.27985	0.767024472		0.022140132
24.12299	37.15903	20.9591	-0.43906	0.434285263	NA	
7180.612	7226.502	6780.268	-0.10863	0.896393169		0.401260914
473.5255	455.1981	636.1086	-0.16747	0.243530103		0.553711578
198.3446	155.139	140.426	0.006018	0.945439891		0.994756086
303.771	270.3319	302.859	-0.08225	0.031086718		0.784199388
1388.412	1242.97	1195.717	0.05193	0.02496009		0.826486725
1202.576	1115.7	1469.233	0.08196	0.35165001		0.797980755
20174.86	19506.63	21812.13	-0.49236	0.83986426		0.002079329
1793.143	1687.02	1611.755	0.198355	0.614621507		0.241619328
628.0913	663.2887	616.1975	0.545483	0.058039065		0.001207474
0	0.928976	0	-1.86025	NA	NA	
3219.08	3151.086	3080.987	0.025096	0.222727039		0.883471187
279.6481	270.3319	339.5374	0.29071	0.488617455		0.328024792
0.893444	0	0	-1.74916	NA	NA	
990.8297	975.4245	885.5219	0.119649	0.890335462		0.602036712
364.5253	303.7751	331.1537	0.137818	0.577332911		0.723437511
708.5013	777.5527	797.4937	0.097066	0.951118826		0.608228413
22315.56	21516.94	23629.29	-0.3641	0.657109304		0.00526791
380.6073	400.3886	352.1128	0.133695	0.185762147		0.684131921

94.70509	85.46577	54.49365	-0.03937	0.525181455	0.946922147
4.467221	8.360782	0	-0.03796	NA	NA
1192.748	1186.302	1197.812	-0.11838	0.072365168	0.539224916
317.1727	336.2892	373.0719	-0.004	0.465047544	0.999234423
1021.207	1036.737	1103.497	-0.17045	0.428811885	0.303824089
981.0018	928.9758	767.103	0.369097	0.068521623	0.054239832
726.3702	731.1039	709.4655	0.16294	0.356320255	0.409178926
314.4924	428.2578	319.6262	0.292217	0.924284267	0.266775275
5953.913	5838.613	6157.783	-0.14498	0.504339531	0.361564535
2984.104	2929.061	2537.099	0.12273	0.07224611	0.604835014
2985.891	2681.024	2470.03	0.34112	0.441155859	0.389799953
12773.57	12866.31	12351.2	-0.12414	0.795376015	0.40615036
1595.691	1581.117	1326.711	0.073968	0.465814049	0.798292995
29061.06	28556.71	30939.82	-0.48378	0.985859213	0.000831448
5884.224	5685.332	5783.663	-0.34262	1.23607E-14	0.000181416
129.5494	114.264	149.8575	0.055371	0.732140966	0.914120833
712.9685	635.4194	613.0536	0.338552	0.455280748	0.237811367
6406.889	6291.953	6407.196	-0.19098	0.006535586	0.016524168
500.3288	705.0926	545.9845	-0.02557	0.460515819	0.942267738
1085.535	872.3082	990.3174	0.303399	0.507065266	0.10424789
380.6073	388.3119	355.2567	-0.04693	0.31587804	0.864252513
1007.805	888.1008	944.2074	0.275396	0.049321046	0.070866904
3934.729	3678.744	3622.78	0.032826	0.69974305	0.852816937
229.6152	246.1786	222.1664	0.094089	0.759261525	0.764338813
55.39354	69.67318	86.98026	-0.37832	0.354108154	0.398613087
1841.389	1846.804	1541.542	0.125504	0.146940246	0.613915007
3054.686	2874.251	2997.151	-0.04163	0.852231703	0.79799583
1321.404	1310.785	1262.786	0.416943	0.652135039	0.361323147
360.058	396.6727	395.079	-0.11074	0.766887678	0.690294956
995.2969	1048.814	1089.873	-0.28348	0.161758304	0.028626623
5232.903	5169.75	5393.824	-0.15728	0.872654096	0.300201673
1584.077	1519.804	1643.193	-0.14434	0.990064993	0.390556389
388.6483	415.2522	463.1961	-0.25053	0.962011634	0.315076194
835.3704	863.9475	880.2821	0.026819	0.003450987	0.91372954
1058.731	1133.35	1239.731	-0.07229	0.826883467	0.721329001
357.3777	379.0221	377.2638	0.069244	0.97594944	0.838085776
7.147554	11.14771	19.91114	3.026206	0.2795135	NA
1749.364	1612.702	1423.123	0.132061	0.382461453	0.708596546
551.2551	603.8342	559.6079	0.06013	0.660321043	0.826008003
415.4516	481.2094	684.3145	-0.74445	0.951538779	0.000330467
0	4.644879	1.047955	-0.24481	NA	NA
2128.184	2163.585	2322.268	-0.28225	0.014948404	0.026340179
1465.249	1413.901	1412.643	0.044511	0.186095147	0.782219628
4517.254	4604.004	4498.87	-0.0602	0.183199252	0.693887308
426.1729	407.8204	381.4556	-0.03897	0.828016939	0.893102595
247.4841	187.6531	187.5839	-0.17558	0.363948776	0.620413666
1039.076	1028.376	909.6248	0.23067	0.44898062	0.316203475
2116.569	2067.9	2194.418	-0.0485	0.515985444	0.791888862
545.8944	493.2861	537.6009	0.009912	0.464099155	0.965364339
2651.743	2428.343	2420.776	0.274071	0.899176451	0.084617521
1178.453	1324.719	1132.839	0.345674	0.579526371	0.102958379
188.5167	203.4457	197.0155	0.134843	0.962433805	0.73912962
386.8614	428.2578	389.8392	0.099619	0.25875662	0.691804481
2138.906	2318.724	2314.932	-0.0028	0.152933305	0.990142758

1891.421	1937.843	2025.697	0.022652	0.771558038	0.904467867
0	0	0	#DIV/0!	NA	NA
2036.159	2114.349	2599.976	-0.14328	0.753964428	0.471231263
2318.488	2249.979	2066.567	0.00899	0.392876266	0.962321489
3767.654	3570.054	3368.127	0.15019	0.856763291	0.469528865
1156.117	1148.214	1038.523	0.172573	0.230347011	0.351487828
3097.571	2953.214	3265.427	-0.13599	0.740595411	0.370304803
556.6158	591.7576	640.3004	-0.30503	0.234941511	0.063099827
2337.25	2286.209	2065.519	-0.01988	0.941323092	0.948371294
327.0006	421.755	387.7433	0.092992	0.735300868	0.762489303
146.5249	139.3464	119.4669	0.087328	0.901380807	0.840777068
259.0988	284.2666	299.7151	0.11097	0.732140966	0.767228242
0	0	0	#NUM!	NA	NA
324.3203	334.4313	463.1961	-0.02223	0.442771688	0.956290296
3179.768	3074.91	2883.972	-0.00813	0.025591463	0.972140885
936.3296	1042.311	979.8378	-0.10586	0.295581805	0.596309029
287.6891	234.1019	225.3103	0.244934	0.254673637	0.390556389
1404.494	1411.114	1350.814	0.157882	0.012122297	0.497448576
208.1725	258.2553	241.0296	-0.03239	0.354108154	0.952588397
1093.576	978.2115	1111.88	0.138253	0.979755434	0.394829397
379.7138	393.8857	444.3329	-0.18147	0.876595319	0.505302546
19808.55	18724.44	18357.03	-0.37731	0.905623996	0.010218325
528.919	570.3911	410.7983	0.08239	0.399767691	0.82871401
452.0828	491.4282	498.8265	0.139917	0.060961265	0.648218962
429.7467	468.2038	399.2708	0.271176	0.189991943	0.205836834
746.026	649.3541	779.6784	0.163471	0.69974305	0.426846696
3701.54	3626.721	3555.711	0.009242	0.910682739	0.952588397
1894.102	1947.133	1916.71	-0.09463	0.880033865	0.517983853
766.5752	782.1976	775.4866	0.119882	0.369165716	0.710878442
1004.231	903.8934	957.8308	-0.22418	0.597867308	0.147203406
9756.411	9783.973	9807.81	-0.08595	0.37757303	0.421538281
279.6481	273.1189	268.2765	0.329502	0.900383237	0.263613142
455.6566	439.4055	404.5106	0.134304	0.617323493	0.590570691
377.9269	468.2038	444.3329	-0.07019	0.233378357	0.818645356
2303.299	2235.116	2129.444	0.072781	0.453550494	0.744172227
0	2.786927	2.09591	-1.10954	NA	NA
0	1.857952	8.383639	#DIV/0!	NA	NA
972.9608	1011.655	996.6051	0.023641	0.234629688	0.921848549
705.821	824.9305	746.1439	0.083154	0.97820156	0.678425078
1034.608	983.7853	856.1791	-0.4478	0.0444701	0.001688143
229.6152	197.8718	228.4542	-0.04813	0.310443697	0.912679298
2770.571	2816.655	2417.632	0.051861	0.758804926	0.763536617
2008.463	2124.568	2224.808	-0.04069	0.940969382	0.844024501
1648.405	1650.79	1764.756	-0.12837	0.032552764	0.36518668
1165.051	1315.43	1187.333	-0.07465	0.869438043	0.729565723
1871.766	1790.136	1676.728	-0.05205	0.565330117	0.818645356
2393.537	2329.871	2633.511	0.131681	0.454285123	0.470568982
723.6898	802.6351	798.5416	-0.00653	0.899176451	0.986413752
988.1493	991.2171	1025.948	0.083032	0.825200136	0.672998339
0	0	1.047955	-2.94368	NA	NA
315.3858	331.6443	344.7772	-0.07893	0.005435534	0.780344055
381.5007	431.0448	553.3202	0.036805	0.649714874	0.919467952
0	0.928976	4.19182	1.959111	NA	NA
748.7063	840.7231	772.3428	-0.28493	0.187714147	0.042867461

518.1977	562.9593	477.8674	0.240359	0.831470267		0.225664658
4.467221	0	2.09591	-0.51515	NA	NA	
290.3694	373.4483	330.1058	-0.13993	0.173427762		0.684119849
2.680333	1.857952	3.143865	-1.12057	0.38441957	NA	
616.4765	498.86	501.9704	0.425527	0.544702272		0.193397574
92.9182	114.264	118.4189	-0.09676	0.572219693		0.841259647
3455.842	3282.071	3427.86	0.020143	0.20047115		0.903329758
20858.35	21429.61	20953.86	-0.4496	0.98409529		0.003590571
2561.505	2350.309	2241.576	0.015257	0.74152952		0.938924382
910.4197	1039.524	992.4133	-0.09356	0.974826797		0.675143077
1165.051	1013.513	859.323	0.186017	0.732140966		0.422560599
567.3371	536.948	491.4908	0.333692	0.364476852		0.114325948
1.786889	0.928976	1.047955	1.51494	NA	NA	
1458.994	1270.839	1321.471	0.13083	0.168661373		0.558104763
447.6156	494.2151	501.9704	0.130904	0.7406592		0.58759663
1428.617	1433.41	1271.169	0.245678	0.488752278		0.307823418
1166.838	1140.782	1480.76	-0.31385	0.313396084		0.116775899
0	0	2.09591	#DIV/0!	NA	NA	
5708.215	5637.954	5340.378	-0.04215	0.025591463		0.754367859
250.1644	231.315	286.0917	-0.21523	0.15696576		0.422252477
884.5098	814.7117	759.7673	0.18656	0.365690431		0.416620055
821.9687	701.3767	697.938	0.046499	0.145617043		0.875604624
378.8204	451.4822	361.5444	-0.00159	0.936346196		0.996992829
3271.793	3324.804	2927.986	-0.15814	0.861602547		0.310467353
3.573777	8.360782	2.09591	0.223886	NA	NA	
8.040998	8.360782	13.62341	-0.6785	0.892614393	NA	
1890.528	1652.648	1730.174	0.161783	0.543812671		0.371885384
0	0	0	#DIV/0!	NA	NA	
900.5918	954.9871	885.5219	0.271806	0.826883467		0.328024792
1859.258	1895.111	1743.797	-0.2232	0.603004299		0.076637307
839.8376	776.6237	766.055	0.318444	0.344730624		0.057538814
2272.029	2201.673	2146.212	-0.00798	0.707368241		0.976485768
1934.307	2061.397	2243.671	-0.12398	0.746491819		0.565559013
927.3951	988.4302	781.7744	0.036369	0.949687139		0.875772383
7.147554	2.786927	3.143865	-1.32071	0.478308661	NA	
5391.936	5334.179	4634.057	0.042449	0.077746639		0.852939392
6173.7	6468.458	7633.303	-0.72662	0.938838346		4.23024E-07
4992.567	4769.362	4914.908	0.21007	0.914796531		0.030371149
28322.18	27223.63	26886.33	-0.31585	0.735300868		0.010917743
871.1082	778.4817	694.7941	0.159273	0.462276826		0.422560599
1065.879	991.2171	989.2694	0.041013	0.207773529		0.838059844
0	0	0	#DIV/0!	NA	NA	
1481.331	1517.946	1297.368	0.164893	0.285283589		0.373144367
1072.133	1067.393	872.9464	0.364491	0.233378357		0.081762395
1681.462	1727.895	1820.298	0.036897	0.682134131		0.828690234
486.9271	432.9027	465.292	-0.45954	0.854862911		0.011489515
5.360666	11.14771	7.335684	2.593338	NA	NA	
501.2222	565.7462	642.3963	-0.02586	0.909289996		0.93327699
232.2955	218.3093	194.9196	-0.0102	0.170982117		0.991297078
219.7873	260.1132	210.6389	0.161737	0.73478662		0.647117771
2415.873	2396.757	2255.199	0.027244	0.259725973		0.912679298
4177.745	4061.482	3434.148	-0.62345	1.48777E-07		4.43158E-06
850.5589	817.4987	848.8435	0.000841	0.997294627		0.990023409
737.985	784.0555	871.8985	-0.13261	0.451912698		0.515558505

1004.231	1065.535	789.11	0.220747	0.331795127	0.365253762
13612.52	13221.18	14506.84	-0.13364	0.988049891	0.376402197
3942.77	3948.147	4406.65	-0.25672	0.375474938	0.081215714
833.5835	796.1322	731.4725	0.122154	0.996781128	0.56192082
443.1484	412.4652	400.3188	0.155485	0.740887036	0.552800466
2129.971	2275.991	2217.473	-0.18374	0.69974305	0.163200347
268.0333	360.4426	285.0437	-0.16609	0.792868376	0.578237014
3750.679	3730.767	3895.248	0.006581	0.018706432	0.972140885
2.680333	1.857952	0	#DIV/0!	NA	NA
0	2.786927	0	-1.10793	NA	NA
564.6568	588.0417	565.8956	0.020597	0.759261525	0.929442227
340.4023	324.2125	366.7842	0.385685	0.853195053	0.118990473
189.4102	191.369	182.3442	0.136418	0.618648297	0.688746278
0	0	1.047955	-0.68638	NA	NA
402.0499	390.1698	304.9549	0.178276	0.910682739	0.620471139
503.0091	471.9197	513.4979	-0.02677	0.812875146	0.919654113
1050.69	1072.038	981.9337	0.132691	0.179898822	0.697184019
1103.404	1056.245	977.7419	0.223157	0.556307283	0.306279885
579.8453	566.6752	584.7588	0.048765	0.915659837	0.859505388
1032.822	844.439	893.9055	0.332994	0.09408214	0.117751922
348.4433	323.2836	352.1128	0.043181	0.134933146	0.885571199
353.8039	366.0165	280.8519	0.414026	0.638519612	0.32698614
11.61478	18.57952	20.9591	-0.49481	0.927238755	NA
66.11488	74.31806	64.9732	-0.61822	0.899235412	0.129766751
1826.2	1737.185	1715.502	0.027489	0.812875146	0.884584155
1433.085	1401.824	1685.111	-0.49772	0.04572454	7.52836E-05
3834.663	3676.886	4067.113	0.141186	0.485405854	0.2558776
4163.45	4453.51	4868.798	-0.13822	0.012367726	0.403397484
29235.28	29101.09	27237.4	-0.19281	0.662554511	0.196300439
1758.298	1595.051	1501.719	-0.25792	0.068720229	0.068696986
2481.988	2743.265	2700.58	-0.1825	0.173742426	0.302912952
0.893444	5.573855	1.047955	#DIV/0!	NA	NA
16.97544	16.72156	26.19887	-0.08879	0.962011634	NA
1715.413	1685.162	1837.065	0.134699	0.052828239	0.569189198
20557.26	20709.66	20198.28	-0.39205	0.878477491	0.007126765
248.3775	246.1786	239.9817	0.083175	0.053797631	0.81253834
868.4278	793.3453	837.316	0.342901	0.72089777	0.154958521
151.8855	161.6418	163.481	0.116799	0.1432612	0.812861677
3356.67	3324.804	3492.834	-0.06957	0.002670611	0.65282506
1137.355	1051.601	1175.805	-0.00622	0.928693659	0.989508176
717.4357	744.1096	664.4034	0.354464	0.83213076	0.131764306
1490.265	1370.239	1614.898	-0.08417	0.939073649	0.736598036
472.632	444.0504	562.7518	-0.03094	0.803184945	0.91372954
637.9192	666.0756	632.9648	0.070238	0.572336545	0.767705112
0	2.786927	0	#DIV/0!	NA	NA
3860.573	3950.934	5061.622	-0.32021	0.334672105	0.049072029
79833.71	79921.64	77896.58	-0.24864	0.766843138	0.0410944
836.2638	859.3026	720.993	0.202301	0.102584855	0.3265611
1361.609	1385.103	1615.946	0.304944	0.757469981	0.145716826
485.1402	546.2377	529.2172	-0.05829	0.963625684	0.813492611
595.0339	531.3741	609.9097	-0.02554	0.49329978	0.939639228
3286.088	3167.807	2959.425	0.00409	0.000215183	0.983469507
2.680333	1.857952	2.09591	0.143316	NA	NA
1113.232	1165.865	981.9337	0.091887	0.042077294	0.685362267

1354.461	1297.779	1348.718	-0.14723	0.375097568	0.359566588
3489.793	3206.824	3638.499	-0.0179	0.000641579	0.931683541
5970.888	6168.399	6151.495	0.004661	0.189082264	0.97680656
5.360666	7.431806	4.19182	0.688872	0.485973657	NA
968.4936	1075.754	813.213	0.21105	0.152569697	0.446297475
167.0741	178.3633	186.536	0.061077	0.254252351	0.912679298
952.4116	797.0612	752.4316	-0.05676	0.69974305	0.850619089
1167.732	1072.967	1082.537	0.042947	0.310443697	0.81253834
891.6574	780.3396	1023.852	0.11592	0.577332911	0.669238482
287.6891	267.545	278.756	0.092246	0.886123912	0.773113821
470.8451	449.6243	569.0395	0.009982	0.683705537	0.980251134
232.2955	258.2553	264.0846	0.040399	0.940969382	0.931131803
234.0824	270.3319	189.6798	0.229587	0.962556121	0.511081603
2594.562	2630.859	2344.275	0.135987	0.211120083	0.305420728
6.25411	3.715903	3.143865	-0.93864	NA	NA
7.147554	5.573855	4.19182	-1.54224	0.857865109	NA
69454.57	68540.76	68073.05	-0.16614	0.701216566	0.167165088
40641.89	40538.64	38433.75	-0.21205	0.826883467	0.069341932
17.86889	12.07668	15.71932	1.549978	0.821934951	NA
234.0824	402.2465	294.4753	-0.08921	0.756223951	0.835596295
316.2793	215.5224	244.1735	0.179798	0.783760899	0.713323797
1283.879	1378.6	1251.258	-0.11375	0.999122571	0.465657685
950.6247	946.6263	937.9196	0.424105	0.027170262	0.00145335
1156.117	1247.614	1230.299	-0.09114	0.92426981	0.607746892
9.827887	5.573855	14.67137	0.123339	0.613472884	NA
677.2307	620.5558	765.0071	-0.39594	0.968581517	0.110349167
1274.945	1312.643	1200.956	0.154571	0.021160717	0.333065318
616.4765	659.5728	570.0875	0.026077	0.249012381	0.918453677
1473.29	1399.037	1285.841	0.069957	0.440055258	0.695789063
0	0.928976	0	-1.69289	NA	NA
1387.519	1243.899	1212.484	0.183349	0.925203985	0.434126424
1062.305	1074.825	886.5698	0.158323	0.948896953	0.377946785
2420.34	2631.788	2296.069	0.03976	0.35165001	0.864252513
1140.928	1136.137	1355.006	-0.22303	0.117671029	0.152005617
620.0503	529.5162	472.6277	0.20388	0.840870655	0.394829397
3.573777	0.928976	1.047955	#DIV/0!	NA	NA
1314.257	1356.305	1498.575	0.071607	0.324651337	0.712630985
7.147554	16.72156	9.431594	1.129031	0.973819598	NA
4.467221	9.289758	8.383639	-0.28791	0.996384969	NA
973.8542	859.3026	775.4866	0.214802	0.03049807	0.359873864
565.5502	562.0303	494.6347	0.313231	0.296333484	0.090902558
2362.267	2269.488	2331.7	0.106112	0.364668012	0.567449963
841.6245	843.51	763.9591	0.098227	0.598960113	0.765789524
751.3866	807.2799	759.7673	0.053524	0.359347275	0.819004
1112.338	1220.674	1300.512	-0.01787	0.001685017	0.936474207
610.2224	599.1894	691.6502	-0.30689	0.837998472	0.043238479
2.680333	0	2.09591	-0.8553	NA	NA
1054.264	980.9984	1055.291	0.042959	0.981076246	0.835181175
1703.798	1680.517	1377.013	0.28781	0.453640482	0.169935181
187.6233	183.0082	168.7207	-0.34728	0.582750031	0.206277032
11.61478	9.289758	5.239774	-0.40744	0.843186128	NA
393.1155	465.4169	430.7095	0.009799	0.579415204	0.986255577
394.0089	312.1359	411.8463	-0.03178	0.38010566	0.918453677
1897.676	1947.133	1986.922	-0.15316	0.490493745	0.243675681

360.9515	422.684	450.6206	-0.07538	0.8968711	0.786706509
8435.007	8384.935	7949.786	-0.06898	0.045913153	0.533405119
634.3454	635.4194	578.4711	0.117811	0.527593235	0.708596546
368.9925	308.42	441.189	-0.20371	0.743382516	0.493206792
19.65577	10.21873	11.5275	0.455399	0.967317013	NA
1994.168	2304.789	1816.106	0.150517	0.181062142	0.394044776
930.9689	898.3196	863.5148	0.208585	0.981584314	0.162129177
1.786889	0.928976	1.047955	-1.0314	NA	NA
1147.182	1390.677	1348.718	-0.2327	0.648256394	0.208176931
354.6974	329.7864	333.2497	0.15872	0.246273306	0.533112779
1717.2	1595.051	1460.849	0.285527	0.208221128	0.223904778
5668.01	5735.496	5143.363	0.159634	0.37106799	0.323917219
1149.863	1067.393	884.4739	0.100869	0.027170262	0.728985289
120.615	129.1276	141.4739	-0.32242	0.369650796	0.418473065
3471.924	3079.555	3202.55	0.088911	0.004218142	0.66509742
823.7556	870.4503	745.0959	-0.0226	0.192952947	0.923725803
1894.995	1875.602	2422.872	-0.44338	0.078425971	0.001283062
1239.207	1162.149	1093.017	0.039501	0.904997879	0.832306222
2209.488	2267.63	2059.231	0.088531	0.000548353	0.583691606
598.6077	571.3201	618.2934	-0.35328	0.208033102	0.105321547
267.1398	327.9284	288.1876	0.297345	0.012060723	0.221138056
222.4676	205.3036	238.9337	0.165996	0.73037883	0.672730781
541.4272	579.6809	488.347	0.260776	0.29215357	0.383690121
620.9438	510.0077	554.3681	-0.25928	0.000208059	0.243388227
2226.463	2328.942	2299.213	0.094805	0.999646615	0.55088846
3461.203	3088.844	3205.694	0.085357	0.230347011	0.741928459
548.5748	619.6268	543.8886	0.111202	0.90738959	0.718291318
611.1159	617.7689	591.0466	-0.10345	0.880275635	0.639253997
2519.513	2257.411	2515.092	-0.2592	0.230347011	0.010871985
2204.127	2192.383	2204.897	-0.363	0.049453408	0.000370769
1193.642	1108.268	1058.434	0.312937	0.237372928	0.265938654
0.893444	1.857952	3.143865	#DIV/0!	NA	NA
435.1074	497.931	420.2299	0.241495	0.666951881	0.403033225
32764.39	31366.87	32452.02	-0.05292	0.032727278	0.647117771
2259.521	2262.056	2039.32	0.18064	0.97820156	0.423493651
858.5999	859.3026	964.1185	-0.26419	0.959504681	0.150320193
2200.553	2309.434	2241.576	0.094353	0.83213076	0.561102297
0	0	0	#DIV/0!	NA	NA
1367.863	1387.89	1350.814	0.049744	0.888097311	0.795108707
463.6976	462.6299	488.347	0.008642	0.868137167	0.974691849
1639.47	1697.239	1472.377	0.092664	0.100001859	0.699804545
0	0	0	#DIV/0!	NA	NA
0	0	0	#DIV/0!	NA	NA
8436.794	8900.517	8795.485	-0.06881	0.19615888	0.638648576
1209.724	1116.629	940.0155	-0.00217	0.954065397	0.997469085
0	0	0	#NUM!	NA	NA
4.467221	9.289758	5.239774	0.076113	NA	NA
4038.368	4113.505	4488.391	-0.14774	0.962660515	0.22043651
717.4357	770.1209	747.1918	0.004297	0.907772492	0.97680656
1623.388	1629.423	1534.206	-0.02888	0.412665866	0.907489673
55.39354	36.23005	49.25388	-0.32624	0.233378357	0.607944538
839.8376	850.0128	892.8576	0.149633	0.803209236	0.502996806
0	0	0	#NUM!	NA	NA
0	0.928976	1.047955	0.229302	NA	NA

1503.667	1491.935	1430.458	-0.08834	0.413714744	0.627713216
578.9519	561.1014	582.6629	0.050109	0.151730694	0.818055883
7.147554	6.50283	5.239774	0.261741	0.503345543	NA
772.8293	699.5187	649.732	0.156125	0.743382516	0.523923403
259.0988	282.4086	238.9337	-0.05396	0.729848204	0.886786965
5118.542	5432.65	5535.298	-0.19498	0.352221493	0.143013287
2847.407	3032.177	3122.906	-0.10078	0.703034676	0.656215943
933.6492	850.9418	885.5219	0.118888	0.884692467	0.56192082
1546.552	1735.327	1509.055	0.003216	0.550503835	0.990023409
0	0	0	#NUM!	NA	NA
0	0	0	#NUM!	NA	NA
0	0	0	#NUM!	NA	NA
0	0	0	#NUM!	NA	NA
0.893444	0	1.047955	0.203136	NA	NA
1620.708	1618.276	1364.437	-0.0033	0.250094568	0.992866088
11780.96	11205.31	12670.82	0.165694	0.363948776	0.236901534
5.360666	0	2.09591	-0.27305	NA	NA
3.573777	1.857952	3.143865	2.346276	NA	NA
1029.248	925.2599	903.3371	0.19794	0.867574132	0.369657389
2568.652	2405.118	2398.769	-0.10877	0.000656764	0.486031819
648.6405	679.0813	523.9774	0.034718	0.310443697	0.921848549
550.3617	531.3741	514.5459	0.06822	0.825444223	0.801363023
629.8782	651.212	582.6629	0.232689	0.873884347	0.267586812
199.2381	231.315	206.4471	0.266267	0.084077452	0.322653852
125.9756	82.67884	88.02821	0.053396	0.521522096	0.911521311
3851.638	3963.011	3854.378	-0.05345	0.978922635	0.702040936
3180.662	3091.631	3354.504	-0.12322	0.854950923	0.385996817
5.360666	6.50283	1.047955	-0.85097	0.848694292	NA
18974.08	18642.69	19590.47	-0.26821	0.572219847	0.057314358
1157.904	1265.265	1338.238	-0.19495	0.98843748	0.28127578
1185.601	1360.949	1400.068	-0.1826	0.940969382	0.300201673
1166.838	1192.805	1176.853	0.032312	0.537178644	0.858512476
1.786889	2.786927	0	#DIV/0!	NA	NA
701.3537	760.8311	684.3145	0.195664	0.988049891	0.223810637
464.591	540.6639	526.0734	-0.134	0.62498123	0.575540189
545.8944	589.8996	643.4443	-0.13462	0.863207554	0.685537269
1894.995	1694.452	1495.432	0.276206	0.153154897	0.080704627
0.893444	2.786927	4.19182	#DIV/0!	NA	NA
609.329	696.7318	832.0762	0.051334	0.965666941	0.886786965
16.97544	14.86361	45.06206	-0.52841	0.654781049	NA
2399.791	2275.991	2413.44	-0.06245	0.001564335	0.740438137
207.2791	168.1446	248.3653	-0.65682	0.057104652	0.003921392
8.934443	11.14771	14.67137	-0.18916	0.221695862	NA
127.7625	127.2697	213.7828	0.338872	0.303314321	0.41069386
934.5427	908.5383	739.8562	0.154817	0.796419683	0.596309029
3000.186	2923.487	2919.602	-0.13638	0.01483465	0.168460337
1987.913	2107.846	2586.353	-0.52059	0.843186128	0.001198985
426.1729	416.1811	387.7433	0.04696	0.787835013	0.870138542
1.786889	1.857952	2.09591	-1.35136	NA	NA
738.8784	775.6948	732.5205	-0.097	0.721504488	0.60614092
37.52466	30.6562	28.29478	-0.56299	0.669860929	0.385354962
3260.178	3179.884	2941.609	0.140194	0.030520992	0.399254174
2120.143	2323.368	2276.158	-0.28514	0.482562486	0.050220436
33.95088	11.14771	22.00705	0.481757	0.936346196	NA

1676.101	1527.236	1765.804	-0.07775		0.981637588	0.655129754
7.147554	9.289758	6.287729	0.476397		0.383871116	NA
878.2557	731.1039	708.4175	0.086174		0.496884574	0.703453769
202.8118	170.9315	195.9676	-0.63738		0.075331737	0.010292932
567.3371	650.283	580.567	0.013691		0.569085135	0.971658868
2.680333	2.786927	3.143865	-0.06537	NA		NA
300.1973	358.5846	292.3794	0.134073		0.783760899	0.690294956
526.2387	482.1384	419.182	-0.03976		0.901380807	0.91305142
3168.153	3133.435	3899.44	-0.19894		0.98843748	0.501904702
0	2.786927	1.047955	-0.39974	NA		NA
0.893444	0	4.19182	1.949066	NA		NA
1712.733	1716.747	2005.786	-0.0444		0.430461737	0.864107722
2469.48	2377.249	2372.57	-0.02338		0.928104954	0.929693512
1618.028	1642.429	1648.433	-0.09651		0.779325825	0.536657785
1270.478	1344.228	1358.15	-0.03589		0.497263297	0.872630949
367.2056	428.2578	391.9351	-0.06591		0.666493976	0.835596295
549.4682	465.4169	328.0099	0.300191		0.63463635	0.327911681
21065.63	20745.89	21550.14	-0.34517		0.501025279	0.005879945
795.1654	728.317	760.8153	0.178498		0.856547191	0.290794331
4408.254	4289.081	4238.978	0.004092		0.013199381	0.984451848
0	0	2.09591	#DIV/0!	NA		NA
737.985	673.5074	664.4034	0.207713		0.961122583	0.37977825
9.827887	1.857952	8.383639	-0.0268		0.846759683	NA
57385.92	55399.47	56731.04	-0.17589		0.826883467	0.228353073
11354.78	11464.49	11439.48	-0.36247		0.610617723	0.013575411
3.573777	1.857952	0	-1.43119	NA		NA
0	1.857952	4.19182	0.257957	NA		NA
2238.078	2461.786	2725.731	-0.13358		0.574512226	0.41892422
1779.741	2036.315	2088.574	-0.31008		0.892193515	0.131512208
1797.61	1954.565	1658.913	0.009281		0.51970267	0.972140885
365.4187	350.2239	357.3526	0.197252		0.249815953	0.436395412
288.5825	347.4369	288.1876	0.098922		0.331866416	0.752110266
2.680333	3.715903	3.143865	1.667399	NA		NA
624.5175	608.4791	623.5332	-0.00487		0.466761023	0.987875073
473.5255	429.1868	477.8674	0.370238		0.647202438	0.101359597
955.0919	1102.694	888.6657	0.042247		0.835368749	0.885788144
410.0909	510.0077	536.5529	0.031953		0.871252597	0.921848549
934.5427	826.7884	842.5557	0.054113		0.54674796	0.844024501
772.8293	803.564	883.426	0.216635		0.837998472	0.206834201
880.936	850.0128	1023.852	-0.02637		0.732140966	0.91372954
536.0666	550.8826	556.464	-0.24494		0.425091186	0.224439117
545.8944	600.1183	625.6291	-0.10911		0.475984345	0.626775052
7685.408	7598.093	7825.079	-0.10304		0.51752002	0.406585578
83.09032	114.264	92.22003	0.258879		0.973922065	0.635110879
292.1563	389.2408	345.8251	0.073418		0.935100162	0.814086627
5.360666	0.928976	4.19182	#DIV/0!	NA		NA
22906.12	22517.44	26182.11	-0.4559		0.904254847	0.000514141
16.082	33.44313	14.67137	0.135784		0.555851348	NA
11313.68	11291.7	11840.84	-0.21457		0.02013663	0.022543507
2114.783	2316.866	2355.803	-0.08038		0.881060269	0.696921855
3244.096	3840.386	3662.602	-0.22035		0.720425175	0.166478516
5681.412	5448.443	5425.262	-0.0424		0.914186189	0.791888862
1034.608	1041.382	877.1382	0.214159		0.455280748	0.304039612
104.533	68.74421	88.02821	-0.42889		0.119516174	0.348491003

211.7463	230.386	206.4471	0.019861	0.875368742	0.96505606
449.4025	560.1724	500.9224	0.358628	0.665579954	0.057717325
802.3129	683.7262	610.9577	0.149277	0.71440019	0.707584171
3146.711	3261.634	3110.33	0.092387	0.734144448	0.448426543
1945.922	1961.068	1863.264	0.112721	0.009017011	0.544356068
250.1644	273.1189	190.7278	0.051671	0.880647749	0.88945982
737.985	688.371	629.8209	0.429352	0.831439424	0.190817982
1853.897	1910.903	2208.041	-0.16088	0.930586489	0.381961888
3.573777	7.431806	11.5275	-0.16962	0.899176451	NA
17.86889	18.57952	13.62341	1.345296	0.941273396	NA
5565.264	5842.329	6492.081	0.127965	0.780724949	0.41822825
0	0	0	#DIV/0!	NA	NA
0	0	0	#DIV/0!	NA	NA
261.7792	262.9001	226.3583	-0.25699	0.072365168	0.354829766
0	0.928976	0	-0.50354	NA	NA
0	0.928976	0	-0.50354	NA	NA
2159.455	2171.016	2089.622	0.002954	0.000593396	0.992729462
16372.37	16484.67	17316.41	-0.47363	0.638519612	0.000657216
326.1072	247.1076	270.3724	0.294518	0.779325825	0.315911601
375.2466	376.2352	343.7292	-0.30755	0.856505841	0.097581147
305.5579	360.4426	402.4147	-0.04973	0.637435429	0.884584155
205.4922	230.386	235.7899	-0.27001	0.871801928	0.329468078
1242.781	1169.58	1393.78	-0.24017	0.971068458	0.178822023
134.9101	151.423	170.8166	0.062526	0.759261525	0.89838494
3652.4	3637.869	3407.949	0.062124	3.48359E-05	0.725549438
607.5421	649.3541	667.5473	0.082013	0.387628548	0.708596546
393.1155	392.9567	448.5247	-0.03504	0.31724782	0.913266339
1071.24	1103.623	1077.298	0.287388	0.077699804	0.103528732
47278.39	45781.78	46403.44	-0.32217	0.811082292	0.005454133
2439.103	2626.214	2659.71	-0.28441	0.946696226	0.010267904
2550.783	2365.172	2571.681	0.109646	0.890335462	0.515054924
2202.34	2160.798	2428.111	-0.10939	0.988569159	0.642738974
273.3939	265.6871	316.4824	0.128764	0.717185827	0.679997772
337.7219	297.2722	306.0028	0.184428	0.900088322	0.501883079
295.73	246.1786	312.2906	0.500466	0.812875146	0.154195687
3698.859	3337.81	3032.781	0.393294	0.173129151	0.10698808
1576.036	1764.125	1707.119	-0.19403	0.729802201	0.167165088
105.4264	120.7668	98.50776	-0.39213	0.84337655	0.24818395
1.786889	1.857952	2.09591	1.767279	NA	NA
1680.569	1588.549	1585.556	0.066167	0.910682739	0.765144842
26981.12	27102.87	26487.06	-0.29442	0.597867308	0.025884044
628.9848	693.9449	661.2595	-0.04651	0.941323092	0.859505388
900.5918	1020.944	995.5571	-0.20715	0.577332911	0.240893274
240.3365	238.7468	216.9267	-0.12633	0.272240073	0.718291318
101459.5	96397.96	81927.02	0.042607	0.084787806	0.869372302
193.8774	199.7298	202.2553	0.075264	0.628616407	0.835596295
2650.849	2628.072	2427.064	-0.03748	0.00628885	0.855100406
939.9034	1030.234	1189.429	-0.33407	0.87345829	0.039409822
638.8126	538.8059	602.5741	-0.19503	0.960823113	0.283832642
1332.125	1221.603	1410.547	-0.01327	0.285144055	0.952680272
17892.11	17544.64	17926.32	-0.05407	0.935506037	0.743917252
24.12299	43.66186	59.73343	-0.21851	0.952331288	NA
30.3771	33.44313	35.63047	-0.2489	0.831235563	0.722021255
11.61478	6.50283	13.62341	0.730606	0.271435677	NA

515.5173	540.6639	425.4697	0.354428	0.982541147	0.093149564
732.6243	790.5584	634.0127	0.039928	0.596065856	0.897900971
740.6653	732.9619	656.0198	0.091806	0.999122571	0.641336618
13706.33	13596.49	13424.3	0.080859	0.482847539	0.502156977
871.1082	883.4559	1060.53	-0.11033	0.811082292	0.656368654
9.827887	6.50283	10.47955	-0.47055	0.836356366	NA
23.22955	16.72156	29.34274	-0.57566	0.900088322	NA
41.09844	68.74421	51.34979	0.149547	0.983824284	0.831655346
1190.961	1098.049	1161.134	0.105661	0.182617755	0.647435632
900.5918	804.493	714.7052	0.401144	0.504924333	0.045848266
2294.365	2112.491	2169.267	-0.14996	0.968325512	0.240898563
890.7639	876.0241	813.213	0.155834	0.245108675	0.492960564
523.5583	444.0504	481.0113	-0.04831	0.554349677	0.881724648
973.8542	935.4786	860.371	0.099885	0.139818546	0.700799427
405.6237	362.3005	462.1481	-0.0641	0.981023664	0.855100406
630.7716	657.7148	575.3272	-0.00793	0.497309965	0.979493428
1085.535	1207.668	1191.525	-0.23001	0.872688807	0.089192563
691.5259	580.6099	760.8153	-0.12524	0.716459644	0.596309029
1.786889	0.928976	5.239774	0.880806	NA	NA
3813.22	3909.13	3593.437	0.017303	0.259281616	0.914553818
1046.223	1045.098	957.8308	0.234407	0.976661259	0.146815781
559.2961	698.5898	553.3202	-0.08358	0.504339531	0.764338813
2446.25	2514.737	2094.862	0.19182	0.152686951	0.519258549
2997.505	3008.024	2893.403	0.071784	0.272240073	0.684131921
162.6069	229.457	210.6389	0.017161	0.498379067	0.96505606
525.3452	552.7406	388.7913	0.046966	0.924509037	0.881268659
662.9356	667.0046	534.457	0.062194	0.794232621	0.838059844
461.9107	455.1981	436.9972	0.034057	0.66770133	0.913036427
1.786889	0	3.143865	-1.64179	NA	NA
398.4761	403.1755	387.7433	0.153588	0.543612102	0.561176106
1286.56	1183.515	1125.504	0.102437	0.7406592	0.547627123
75.94276	64.09933	90.12412	-0.1345	0.225736107	0.802920813
2239.865	2055.823	1984.827	0.148589	0.136068446	0.457772469
301.9842	292.6274	282.9478	-0.56721	2.34806E-39	0.000330467
437.7877	454.2691	429.6615	-0.24012	0.337335124	0.161220563
1257.076	1386.961	1285.841	-0.28309	0.430461737	0.043279608
0	0	0	#DIV/0!	NA	NA
0	0	0	#DIV/0!	NA	NA
0	0	0	#DIV/0!	NA	NA
0	0	0	#DIV/0!	NA	NA
2649.956	2609.493	2566.442	0.216196	0.720425175	0.051095669
1343.74	1332.151	1469.233	-0.37973	0.32159394	0.009064744
280.5415	368.8034	342.6813	0.193585	0.657109304	0.569294963
610.2224	528.5872	659.1636	-0.065	0.314709824	0.793207865
4162.557	4378.263	3703.473	-0.00539	0.301433669	0.987413808
973.8542	929.9047	879.2342	0.273772	0.159614653	0.361564535
3499.621	3638.798	3559.903	-0.02387	0.811082292	0.896421455
670.9766	645.6382	647.6361	0.330287	0.023800238	0.065123044
523.5583	603.8342	644.4923	-0.08745	0.926756894	0.732368319
8310.818	7840.555	8555.504	-0.17714	0.525162342	0.150260522
661.1488	672.5785	671.7391	0.226013	0.430461737	0.137783355
920.2476	784.0555	813.213	0.314645	0.086446082	0.181725119
978.3215	1010.726	794.3498	0.180324	0.953918397	0.352822706
1.786889	2.786927	4.19182	-0.23653	NA	NA

612.0093	660.5018	598.3822	0.049416		0.811220717	0.875604624
8549.368	8499.199	8832.164	-0.28293		0.671382026	0.005450387
1209.724	1294.992	1240.779	0.079379		0.831439424	0.622064701
26.80333	27.86927	30.39069	-0.27918		0.888097311	NA
4117.885	3878.474	4029.387	-0.14893		0.692644952	0.178937334
631.6651	774.7658	771.2948	0.086961		0.980746287	0.727522975
418.1319	429.1868	484.1552	0.347466		0.000302523	0.054060197
21.44266	18.57952	33.53456	0.29999		0.913769516	NA
29382.7	29478.26	31330.71	-0.45435		0.671382026	0.00095673
372.5663	338.1472	325.914	0.012909		0.31587804	0.975950358
0.893444	0	0	#DIV/0!	NA		NA
657.575	579.6809	658.1157	0.358393		0.690861229	0.019657367
1616.241	1413.901	1475.52	0.021535		0.764395772	0.928761588
2092.446	2003.801	1795.147	0.131459		0.473286484	0.34571639
870.2147	783.1266	741.9521	0.255274		0.476445149	0.225630132
297.5169	239.6757	238.9337	0.320466		0.703609106	0.238605598
1058.731	1075.754	1196.764	-0.36376		0.249012381	0.023203343
42936.25	41654.34	41247.5	-0.24069		0.853477462	0.118176723
333.2547	374.3772	397.1749	-0.02283		0.363948776	0.94960537
245.6972	265.6871	241.0296	0.195225		0.231792531	0.553844877
972.9608	1244.828	1113.976	-0.11266		0.927256678	0.640018073
5.360666	3.715903	13.62341	0.287491		0.960773888	NA
1078.387	1007.939	1020.708	0.14872		0.729944386	0.462496385
665.616	744.1096	621.4373	-0.00842		0.557418993	0.988370025
150.9921	213.6644	199.1114	-0.1421		0.467380763	0.712891158
3250.35	3189.174	2897.595	0.029804		0.00077072	0.865246562
1511.708	1475.214	1838.113	-0.04514		0.944173424	0.859010941
1903.93	1770.628	1581.364	0.08201		0.1202463	0.737751404
43.77877	56.66752	68.11707	-0.42802		0.986125936	0.359873864
291.2628	320.4966	367.8322	-0.22729		0.105688589	0.383494766
534.2797	510.9367	518.7377	0.287226		0.999122571	0.21207515
174.2216	191.369	157.1932	0.256128		0.30299466	0.568964645
57551.21	57116.22	68528.91	-0.2508		0.279564109	0.119487438
32.16399	45.51981	33.53456	-0.58897		0.072199218	0.280245751
2213.061	1977.789	1882.127	0.218563		0.0444701	0.423534898
878.2557	855.5867	833.1241	-0.18258		0.688569751	0.237500588
0	0	2.09591	#DIV/0!	NA		NA
0	0.928976	1.047955	#DIV/0!	NA		NA
0.893444	0.928976	0	#DIV/0!	NA		NA
0.893444	0.928976	0	#DIV/0!	NA		NA
0.893444	0.928976	0	#DIV/0!	NA		NA
838.0507	842.581	749.2877	0.126498		0.03547414	0.595440724
0	1.857952	0	#DIV/0!	NA		NA
0	1.857952	0	#DIV/0!	NA		NA
0	1.857952	0	#DIV/0!	NA		NA
0	1.857952	0	#DIV/0!	NA		NA
746.9194	650.283	759.7673	0.136951		0.828016939	0.542805648
2213.955	2300.144	2040.368	0.086263		0.484084014	0.691256188
1136.461	979.1405	1262.786	0.104795		0.233074614	0.695789063
5171.255	5327.676	5261.782	-0.05606		0.105979651	0.751824432
1.786889	0	0	#DIV/0!	NA		NA
1.786889	0	0	#DIV/0!	NA		NA
1.786889	0	0	#DIV/0!	NA		NA
1.786889	3.715903	2.09591	0.127822	NA		NA

1.786889	1.857952	1.047955	-0.35616	NA		NA
2387.283	2306.647	2020.457	0.085956		0.66770133	0.79052565
14.29511	0.928976	2.09591	-0.44989	NA		NA
2835.792	2615.996	3291.626	-0.07653		0.805217464	0.718323278
468.1648	619.6268	666.4993	0.061529		0.996781128	0.849305911
662.9356	661.4307	774.4387	-0.2263		0.544035793	0.293133991
98.27887	98.47143	103.7475	-0.25086		0.389992881	0.579811887
2900.12	2864.032	2874.54	-0.00946		0.766253365	0.97378472
393.1155	475.6356	523.9774	0.567026		0.828016939	0.01925496
746.9194	693.0159	960.9746	-0.32145		0.310443697	0.116630357
737.985	696.7318	638.2045	0.024885		0.66770133	0.923725803
16352.71	15344.82	15061.21	-0.16917		0.792297854	0.167949464
139.3773	143.9912	137.2821	-0.33843		0.812875146	0.255474802
1022.994	971.7086	1126.552	0.190608		0.862452498	0.349930843
75.04932	59.45445	70.21298	-0.03432		0.851080991	0.939380143
1.786889	13.00566	9.431594	1.616154		0.757669883	NA
641.493	626.1297	608.8618	0.225996		0.34723135	0.255165763
228.7217	251.7524	259.8928	0.170036		0.416597489	0.542805648
142.9511	143.0623	166.6248	0.064828		0.665579954	0.875604624
1493.839	1385.103	1509.055	0.512464		0.38183291	0.015498519
628.9848	627.0586	609.9097	0.086087		0.455280748	0.672747744
383.2876	376.2352	335.3456	-0.07366		0.365690431	0.786995178
1280.306	1255.046	1302.608	0.061879		0.962011634	0.806564543
1789.569	1900.684	1823.442	-0.38785		0.757469981	0.005720058
290.3694	279.6217	366.7842	-0.30664		0.901132408	0.263268062
1558.167	1436.197	1601.275	-0.14271		0.056286582	0.30760874
15393.15	14479.02	14930.21	-0.02916		0.582207349	0.80432116
840.731	808.2089	856.1791	-0.2175		0.916416444	0.200030838
830.9032	818.4276	864.5628	-0.11666		0.975298616	0.583053599
1617.134	1633.139	1535.254	0.158316		0.29215357	0.413081195
0.893444	1.857952	0	-2.65101	NA		NA
651.3209	741.3227	725.1848	-0.02048		0.769710962	0.937994709
1950.389	1981.505	2087.526	-0.23272		0.585762809	0.112687221
586.0994	563.8883	428.6136	0.425362		0.363948776	0.514122373
337.7219	332.5733	417.086	-0.72505		0.046345161	9.85399E-05
0	0.928976	2.09591	-1.71895	NA		NA
3272.686	3393.548	3084.131	-0.07162		0.947527175	0.690294956
1162.371	1215.1	1240.779	0.152131		0.429631603	0.398344249
1850.323	1909.974	1593.939	0.126836		0.999656194	0.614824771
6.25411	0.928976	1.047955	2.643818	NA		NA
221.5742	229.457	187.5839	-0.06936		0.521899793	0.8721296
846.0917	707.8795	864.5628	0.028141		0.867490475	0.919337062
0	0	0	#NUM!	NA		NA
4.467221	0	1.047955	-0.82186	NA		NA
574.4847	589.8996	536.5529	-0.01926		0.80050738	0.94960537
0	0	0	#DIV/0!	NA		NA
0	0	0	#DIV/0!	NA		NA
0	0	0	#DIV/0!	NA		NA
628.0913	700.4477	678.0268	0.045118		0.60948547	0.86814025
184.943	146.7782	147.7616	0.815268		0.274927458	0.049190168
1212.404	1267.123	1061.578	0.037109		0.515985444	0.885975149
25.01644	34.3721	18.86319	-0.02782		0.122047295	NA
668.2963	691.158	668.5952	0.077055		0.332003738	0.722021255
75038.6	71553.43	69187.03	-0.02061		0.009038328	0.923910484

5.360666	0.928976	3.143865	-0.74003	NA	NA	
897.018	765.476	887.6178	0.054189	0.01003883		0.84358525
256.4185	309.3489	313.3385	-0.12847	0.80037736		0.693112942
0	0	0	#NUM!	NA	NA	
182.2626	190.44	236.8378	0.375769	0.607918252		0.323379782
8.934443	5.573855	4.19182	2.827718	NA	NA	
951.5181	816.5697	932.6799	0.033382	0.647710343		0.880577976
1213.297	1263.407	1238.683	0.139228	0.603004299		0.431825344
598.6077	671.6495	722.0409	-0.10888	0.988569159		0.690038797
370.7794	403.1755	444.3329	-0.28377	0.923364203		0.149383412
6.25411	9.289758	6.287729	-0.72322	0.563351252	NA	
2.680333	0.928976	0	0.454465	NA	NA	
696.8865	770.1209	747.1918	0.010918	0.745767703		0.966582892
1104.297	1151.93	1217.724	-0.19215	0.476024675		0.321405678
2.680333	2.786927	3.143865	0.094945	NA	NA	
1256.183	1240.183	1483.904	-0.24283	0.655427675		0.164996552
1529.577	1480.787	1547.829	0.051178	0.567589507		0.775439768
199.2381	235.0309	215.8787	0.297492	0.035776598		0.35429586
419.0254	274.9768	334.2976	0.09013	0.625996416		0.797601985
828.2228	803.564	805.8773	0.011882	0.252404744		0.970425422
3692.605	3762.352	3916.207	-0.3052	0.825797061		0.034322372
9905.616	9835.066	8797.581	0.121777	0.035172288		0.527040487
14100.34	13774.85	14483.78	0.002246	0.988569159		0.990142758
637.9192	641.9223	664.4034	-0.03685	0.581224219		0.87433787
62.5411	83.60782	57.63752	0.223407	0.699574787		0.735169964
1937.881	2014.019	2421.824	-0.11507	2.34671E-09		0.60286387
513.7304	548.0957	427.5656	0.148478	0.967693355		0.697416669
10243.34	9222.871	9510.191	0.256152	0.051055002		0.227558056
784.4441	769.1919	737.7602	-0.11932	0.358814452		0.55088846
255.5251	324.2125	275.6121	0.041445	0.064827883		0.914120833
41168.12	41480.63	42414.93	-0.3678	0.848681983		0.007033921
250.1644	257.3263	325.914	-0.00522	0.57058689		0.979395135
0.893444	13.00566	4.19182	-0.60987	0.812875146	NA	
8.040998	9.289758	12.57546	0.20624	0.711398577	NA	
40.20499	31.58518	39.82229	0.479847	0.510420572	NA	
129.5494	175.5764	175.0085	0.316597	0.407216733		0.370646519
2972.489	2726.544	2903.883	-0.05503	0.001564335		0.727163127
1.786889	0.928976	8.383639	3.075166	NA	NA	
1039.076	999.5779	1087.777	0.007647	0.642006987		0.969852341
859.4934	942.9104	937.9196	-0.08095	0.174380645		0.702604821
954.1985	998.6489	712.6093	0.198749	0.365953812		0.420675433
275.1808	227.5991	306.0028	-0.34248	0.380570392		0.198030129
3.573777	4.644879	17.81523	-0.24127	0.935506037	NA	
1242.781	1299.637	1161.134	0.082684	0.022968717		0.696921855
0	0	0	#NUM!	NA	NA	
301.9842	344.65	283.9958	-0.12096	0.760924499		0.663320474
15.18855	11.14771	30.39069	-0.02311	0.872444605	NA	
144.738	124.4828	105.8434	-0.14027	0.50823828		0.786995178
2.680333	5.573855	0	0.933955	NA	NA	
0	0	0	#NUM!	NA	NA	
2590.988	2469.218	2554.914	-0.06592	0.92288341		0.721425275
0.893444	3.715903	3.143865	0.082194	NA	NA	
186.7298	192.298	134.1382	0.7098	0.907472172		0.119008343
274.2874	310.2779	337.4415	-0.17365	0.237605221		0.549352823

1398.24	1363.736	1384.348	-0.13382	0.330052034	0.389216061
1029.248	932.6917	1089.873	0.532508	0.033904641	0.016996857
70.5821	51.09367	73.35684	0.068142	0.71034712	0.948574402
137.5904	135.6305	143.5698	0.166554	0.798030968	0.739117108
646.8536	749.6834	565.8956	0.101968	0.785492922	0.752941037
0.893444	0	0	#DIV/0!	NA	NA
16.97544	21.36644	6.287729	0.438816	0.997889853	NA
612.9028	649.3541	500.9224	0.1854	0.250094568	0.527767428
0	1.857952	2.09591	-1.67116	NA	NA
354.6974	362.3005	358.4006	0.302202	0.294050379	0.175074667
889.8705	882.527	971.4542	0.149734	0.812875146	0.552800466
388.6483	458.914	448.5247	-0.22753	5.99679E-07	0.403397484
321.6399	337.2182	335.3456	0.329265	0.897766774	0.205984307
521.7714	487.7123	448.5247	-0.07468	0.143621832	0.790388906
1674.315	1589.478	1799.339	-0.01963	0.36585589	0.921976304
8.934443	4.644879	14.67137	0.115167	0.824080056	NA
141.1642	180.2213	146.7137	0.001634	0.108921558	0.999877981
403.8368	405.9624	438.0451	-0.00234	0.530486695	0.996180974
1.786889	3.715903	3.143865	1.129923	NA	NA
171.5413	139.3464	151.9535	0.065527	0.335983122	0.873067467
0.893444	0.928976	5.239774	#DIV/0!	NA	NA
110.7871	150.4941	101.6516	0.287363	0.424996881	0.502996806
1372.33	1242.97	1357.102	-0.22493	0.992511525	0.159540184
718.3292	752.4704	587.9027	0.152326	0.934132954	0.536590083
3339.695	3355.46	3224.557	0.140682	0.072881149	0.256269459
2164.815	2141.289	1977.491	0.283533	0.507862868	0.102175745
2117.463	1935.057	2011.025	0.182643	0.28025041	0.27042216
0.893444	0.928976	0	-1.80685	NA	NA
37.52466	40.87493	41.9182	0.507681	0.841901206	0.406585578
3704.22	3508.741	4194.963	-0.37468	0.43802737	0.010034507
1771.7	1754.835	1779.427	0.400465	0.378509673	0.001906013
1105.191	1126.848	1103.497	0.184472	0.248869679	0.167165088
805.8867	839.7941	953.639	0.125174	0.785492922	0.562726251
87.55754	82.67884	120.5148	-0.55325	0.58195463	0.168553612
1778.848	1798.497	1718.646	-0.04138	0.986125936	0.861226913
48687.35	46775.79	50854.11	-0.04287	0.24779362	0.835596295
2251.48	2131.999	2268.822	-0.07253	0.289945869	0.652302842
303.771	200.6588	298.6671	0.217874	0.215286681	0.690294956
209.9594	203.4457	222.1664	-0.09556	0.136084466	0.834332496
20.54922	15.79259	12.57546	0.256847	0.582207349	NA
340.4023	308.42	418.134	-0.18557	0.173157428	0.553486024
1463.462	1463.137	1451.418	0.027393	0.614621507	0.87433787
617.37	595.4735	579.5191	-0.15474	0.000316132	0.675625717
671.8701	619.6268	555.4161	0.343967	0.666720621	0.203537925
469.9517	463.5589	457.9563	-0.05611	0.871208906	0.818055883
12591.31	11911.33	12932.81	-0.37067	0.827268091	0.005669712
1039.969	1010.726	1123.408	-0.09226	0.576058796	0.737803127
2767.89	2998.734	2722.587	-0.00709	0.937354907	0.979395135
3185.129	3231.907	3334.592	-0.10516	0.950686887	0.402824474
1010.485	1156.575	1072.058	0.038064	0.904997879	0.875604624
0	1.857952	0	0.496456	NA	NA
1849.43	1572.756	1583.46	0.110769	0.350490225	0.673302041
761.2145	733.8909	662.3075	-0.01767	0.360714981	0.95937982
711.1816	676.2944	622.4852	0.206492	0.30299466	0.402165869

3.573777	2.786927	4.19182	0.031131	NA	NA
705.821	631.7035	647.6361	0.183097	0.243547924	0.576591259
221.5742	310.2779	358.4006	-0.2903	0.524823502	0.359566588
225.148	183.9372	215.8787	-0.22344	0.485974888	0.422560599
0.893444	0	2.09591	-0.75909	NA	NA
2628.513	2602.061	2478.413	0.134791	0.004040821	0.365253762
2242.545	2235.116	2123.157	0.105842	0.155062297	0.672204533
0	1.857952	4.19182	#DIV/0!	NA	NA
292.1563	271.2609	238.9337	0.143068	0.062616413	0.578237014
342.1892	405.0334	439.0931	-0.46715	0.108410382	0.040784938
2.680333	1.857952	2.09591	1.975961	NA	NA
810.3539	910.3962	905.433	0.085937	0.989820945	0.690294956
109.0002	81.74987	106.8914	-0.29991	0.172771811	0.457211032
8.934443	7.431806	3.143865	0.040647	0.904254847	NA
156142.8	149439.7	153508.6	-0.13115	0.862400346	0.370304803
1353.568	1318.217	1208.292	0.134826	0.483401265	0.567139643
1378.584	1348.873	1352.91	-0.21127	0.535057938	0.089456731
320.7465	333.5023	315.4344	-0.19372	0.579980685	0.472793604
1939.667	1969.429	1854.88	0.060121	0.182617755	0.746837826
2082.619	2074.403	2282.446	0.277111	0.279012259	0.025118017
2934.071	2908.623	2710.011	0.088972	0.009784878	0.711465473
28.59022	36.23005	22.00705	-0.52722	0.699040789	NA
14.29511	11.14771	17.81523	0.628658	0.993190072	NA
775.5096	718.0983	698.9859	0.152856	0.552781792	0.515558505
169.7544	154.21	159.2891	0.116154	0.327964932	0.752110266
45.56566	44.59084	33.53456	-0.35531	0.888097311	0.578237014
802.3129	770.1209	735.6643	0.167265	0.544035793	0.578896646
2964.448	2969.936	2582.161	0.074372	0.136655574	0.690106308
637.0258	600.1183	564.8477	0.358309	0.179898822	0.251815507
2803.628	2836.163	2801.183	0.275245	0.187777748	0.076809757
783.5506	712.5244	620.3893	0.132509	0.720425175	0.566028914
1041.756	963.3479	931.6319	0.314027	0.674403807	0.027833357
0	0	0	#DIV/0!	NA	NA
1782.421	1947.133	1800.387	-0.02228	0.011540931	0.914553818
0	0	0	#DIV/0!	NA	NA
0	0	0	#DIV/0!	NA	NA
0	0	0	#DIV/0!	NA	NA
0	0	0	#DIV/0!	NA	NA
0	0	0	#DIV/0!	NA	NA
31.27055	17.65054	26.19887	0.03249	0.669860929	NA
3556.802	3526.392	4018.907	0.06452	0.948223269	0.755018053
290.3694	257.3263	308.0987	0.023965	0.390161167	0.928702649
2811.669	2882.612	2890.26	-0.0071	0.001496948	0.972140885
551.2551	575.965	562.7518	0.137535	0.690152662	0.489096476
16749.4	16822.82	16589.13	-0.25942	0.606270396	0.029788115
566.4437	474.7066	484.1552	0.073517	0.957996061	0.79934904
886.2967	995.862	804.8294	0.209648	0.997568936	0.333065318
9.827887	8.360782	11.5275	-0.53294	0.803730942	NA
281.4349	222.0252	287.1396	-0.20684	0.812875146	0.532286968
623.6241	590.8286	679.0748	0.369077	0.735300868	0.161154644
337.7219	365.0875	398.2229	-0.21119	0.701861094	0.394022844
563.7633	541.5929	517.6897	-0.32658	0.462276826	0.048893739
3110.973	2887.257	3265.427	-0.05256	0.981711158	0.818645356
1053.371	945.6973	912.7687	0.304767	0.91014027	0.059410136

249.2709	238.7468	343.7292	-0.33194	0.549071806		0.256199693
2739.3	2825.015	2665.997	0.017616	0.458486853		0.924154584
51.81977	42.73289	56.58956	-0.15557	0.757469981		0.864252513
5314.206	5261.719	6107.481	0.489691	0.847983366		0.13275794
759.4276	736.6778	727.2807	-0.01783	0.977332424		0.952680272
835.3704	698.5898	803.7814	0.109328	0.936346196		0.663320474
27503.79	26573.35	28788.37	-0.29847	0.495011768		0.010351567
5624.232	5524.619	6186.078	-0.24539	0.883354954		0.084073915
1607.306	1548.603	1560.405	-0.22194	0.786928941		0.129766751
0	1.857952	0	-0.50354	NA	NA	
24.12299	10.21873	23.05501	0.254848	0.935731617	NA	
922.0345	991.2171	1043.763	-0.20698	0.837529432		0.303824089
1830.667	1823.579	2239.48	-0.24829	0.918350023		0.184072788
641.493	658.6438	607.8138	-0.09533	0.972861407		0.693303686
1141.822	1127.777	1016.516	-0.09382	0.730407446		0.612005134
15.18855	13.93464	22.00705	0.549596	0.716063981	NA	
4.467221	3.715903	7.335684	0.369341	NA	NA	
89456.11	87385.96	85233.32	-0.14661	0.506703303		0.198106267
645.0668	691.158	754.5275	-0.0314	0.888097311		0.914772543
5209.673	5383.415	5453.557	-0.14671	0.987797193		0.143013287
1010.485	1057.174	1036.427	-0.01284	0.989297532		0.964974564
3.573777	0	3.143865	-0.04998	NA	NA	
8611.016	8453.679	9950.332	-0.20305	0.901380807		0.251743462
1898.569	1841.23	2009.977	0.031389	0.836962769		0.896670492
1106.977	1257.833	1091.969	0.061713	0.881060269		0.762276725
5465.199	5380.628	4642.44	0.006282	0.705615772		0.984451848
475.3123	440.3345	468.4358	0.0113	0.64485916		0.961026715
335.935	376.2352	264.0846	-0.008	0.92288341		0.992528568
10.72133	27.86927	25.15092	-0.02176	0.526561206	NA	
615.5831	608.4791	468.4358	-0.09364	0.901132408		0.748344041
3.573777	0	2.09591	#DIV/0!	NA	NA	
363.6318	406.8914	399.2708	-0.20325	0.166831969		0.386029809
8616.376	8907.949	9259.729	-0.26451	0.724579185		0.049190168
42.88532	41.80391	48.20593	-0.18891	0.031433		0.782080618
460.1238	450.5532	473.6756	0.067329	0.572336545		0.791888862
5336.543	5143.739	4857.271	0.044907	0.37602059		0.814809717
36183.6	35168.24	34768	-0.32973	0.758097373		0.023440007
1156.117	1124.061	1014.42	0.361461	0.618280897		0.03394343
22.33611	13.93464	27.24683	0.454018	0.987797193	NA	
856.813	970.7797	898.0973	0.026892	0.960823113		0.924920797
236.7627	248.9655	299.7151	-0.12625	0.92660939		0.659308719
456.55	525.8003	478.9154	0.191327	0.759261525		0.365253762
1798.503	1904.4	2005.786	-0.2855	0.812356506		0.276791768
47022.86	46915.13	47457.69	-0.28223	0.862447485		0.043763037
264.4595	220.1673	164.5289	0.276317	0.766253365		0.494999963
1439.339	1362.807	1340.334	0.13171	0.491451873		0.399848949
70.5821	52.02264	80.69253	-0.33507	0.918350023		0.462481429
1821.733	1775.273	1459.801	0.222588	0.337335124		0.359566588
577.165	513.7236	512.4499	-0.16	0.158766689		0.43593104
103.6395	105.9032	115.275	-0.30124	0.484953599		0.503886247
1306.216	1288.489	1263.834	0.053083	0.337709829		0.749984274
1.786889	0	1.047955	0.106012	NA	NA	
32818.89	33199.74	32748.59	-0.17301	0.41136268		0.113154692
1014.059	980.0694	922.2003	-0.33731	0.246058895		0.017778808

420.8122	475.6356	347.921	0.306784	0.847410002	0.197029258
102.7461	134.7015	169.7687	-0.11951	0.856505841	0.791888862
34.84433	27.86927	33.53456	-0.33367	0.946357028	0.603217016
3543.4	3753.062	3892.104	-0.19368	0.836962769	0.207006031
1168.625	1105.481	1108.736	0.111092	0.014083611	0.614824771
1177.56	961.4899	1229.251	0.252896	0.105557831	0.361214706
0.893444	2.786927	2.09591	0.41897	NA	NA
4535.123	4632.802	4271.464	0.0176	0.035767958	0.914553818
165.2872	220.1673	282.9478	-0.14715	0.58195463	0.767583474
356.4843	290.7694	344.7772	-0.12971	0.402196969	0.690294956
301.9842	353.0108	333.2497	-0.11677	0.755224646	0.737751404
4.467221	16.72156	25.15092	0.310831	0.870174956	NA
736.1981	642.8512	767.103	-0.00633	0.988569159	0.990023409
420.8122	523.0134	374.1199	0.198689	0.842806932	0.422560599
1036.395	996.791	1072.058	0.05079	0.941466278	0.823966789
2.680333	3.715903	3.143865	1.024468	NA	NA
270.7136	290.7694	227.4062	-0.03083	0.769710962	0.924895748
181379.9	177768.8	190230	-0.32261	0.903964934	0.000452251
316.2793	265.6871	241.0296	0.59876	0.972861407	0.045848266
167.9675	126.3407	134.1382	-0.22475	0.733488302	0.551771495
964.9198	934.5496	741.9521	0.228432	0.14394509	0.453163355
394.0089	498.86	472.6277	-0.12165	0.828016939	0.694075461
294.8366	254.5394	229.5021	0.392961	0.811082292	0.168910143
417.2385	424.5419	500.9224	-0.12189	0.866564571	0.621297414
5.360666	3.715903	6.287729	0.997916	NA	NA
0	1.857952	0	-0.50354	NA	NA
36707.16	36429.78	36780.07	-0.33372	0.709468629	0.011978719
1.786889	1.857952	0	-1.33876	NA	NA
1588.544	1607.128	1904.134	0.089121	0.938838346	0.744396459
1185.601	1112.913	1134.935	0.145915	0.795376015	0.297698909
94.70509	80.82089	133.0903	-0.08462	0.023209184	0.889287206
9.827887	4.644879	19.91114	-0.52664	0.920564616	NA
94.70509	79.89192	83.83639	-0.35481	0.817609984	0.536657785
1858.364	1934.128	2127.348	-0.05251	0.536133458	0.840718213
3.573777	1.857952	8.383639	0.369112	NA	NA
891.6574	781.2686	781.7744	0.189801	0.98878626	0.292831789
425.2795	536.019	434.9013	0.231699	0.491534977	0.327516537
905.059	917.8281	707.3696	0.27714	0.256943324	0.220509545
1693.077	1776.202	1671.488	0.007319	0.835449534	0.975879427
529.8124	516.5105	491.4908	0.145209	0.879847555	0.56624122
1479.544	1478.929	1479.712	0.043939	0.178803574	0.87466351
827.3294	834.2202	748.2398	0.303779	0.428811885	0.028065868
1989.7	2162.656	2095.91	-0.18094	0.868137167	0.194519967
2696.415	2538.891	3155.392	0.170904	0.831470267	0.422560599
432.427	452.4112	467.3879	-0.19876	0.87547961	0.372138887
2442.677	2117.136	2277.206	-0.08644	0.79502649	0.615745925
1490.265	1487.29	1693.495	-0.32186	0.763001611	0.109807224
150.9921	183.0082	246.2694	-0.437	0.306599955	0.098435777
642.3864	640.0643	763.9591	0.156855	0.637016749	0.43545502
4113.417	4314.163	4179.244	-0.16829	0.899911835	0.150320193
3708.687	3579.344	4369.972	-0.05574	0.435014967	0.838085776
1619.814	1569.969	1467.137	-0.02975	0.132284706	0.889915228
8425.179	8097.882	8449.66	-0.01503	0.668522148	0.923725803
1207.043	1371.168	1415.787	-0.0556	0.497263297	0.819004

452.0828	437.5476	580.567	-0.08393	0.15584882	0.765789524
1036.395	1080.399	1153.798	-0.43631	0.371004931	0.000188607
1232.06	1357.234	1444.082	0.342025	0.935100162	0.084617521
6446.2	6423.867	8084.972	0.573031	0.833159137	9.80382E-05
0	0	0	#NUM!	NA	NA
0	0	0	#NUM!	NA	NA
0	0	0	#NUM!	NA	NA
0	0	0	#NUM!	NA	NA
8.934443	8.360782	3.143865	0.616581	NA	NA
792.4851	723.6721	748.2398	-0.23381	0.221789876	0.278856737
1664.487	2006.588	1859.072	-0.22269	0.257353068	0.159445896
523.5583	625.2007	706.3216	0.114964	0.228243156	0.696002785
0	0	0	#NUM!	NA	NA
0	0	0	#NUM!	NA	NA
0	0	0	#NUM!	NA	NA
0	0	0	#NUM!	NA	NA
0	0	0	#NUM!	NA	NA
0	0	0	#NUM!	NA	NA
736.1981	684.6551	696.89	-0.5758	0.302288037	0.000125515
810.3539	859.3026	824.7405	-0.0256	0.899176451	0.941596912
14.29511	5.573855	3.143865	0.032412	0.960823113	NA
458.3369	480.2805	554.3681	0.508243	0.671382026	0.001957111
6.25411	11.14771	9.431594	0.02941	0.872688807	NA
483.3533	526.7293	487.299	-0.00021	0.947037414	0.990142758
1689.503	1782.704	1832.873	-0.02655	0.046345161	0.886786965
1594.798	1590.407	1752.181	0.047398	0.804201321	0.818055883
27700.35	27070.35	27923.81	-0.29672	0.711342547	0.030380867
1029.248	1086.902	980.8858	-0.2729	0.311277227	0.053703999
1573.355	1459.421	1361.293	0.171403	0.46334755	0.292023088
147.4183	148.6361	151.9535	0.128218	0.730204074	0.807576186
89.34443	96.61348	80.69253	0.089653	0.804201321	0.889915228
740.6653	771.0499	727.2807	0.259774	0.969447004	0.292831789
478.8861	533.2321	470.5317	-0.35556	0.300155248	0.06712239
0	0	0	#NUM!	NA	NA
1842.282	1683.304	1713.406	0.142809	0.079835741	0.359983511
0	2.786927	1.047955	#DIV/0!	NA	NA
0.893444	0.928976	0	-1.24532	NA	NA
124.1888	131.9146	155.0973	0.233669	0.604042936	0.563375937
1737.749	1570.898	1926.141	0.096962	0.940848107	0.632121198
0	0	2.09591	-1.16196	NA	NA
627.1979	747.8255	783.8703	0.144642	0.548339498	0.627256877
1.786889	1.857952	3.143865	-0.52213	NA	NA
5482.174	5140.952	5414.783	-0.10011	0.6346531	0.41069386
8747.713	8900.517	10952.18	-0.63315	0.872444605	3.97861E-05
62.5411	58.52547	42.96615	0.035766	0.831799154	0.967817837
900.5918	909.4673	700.0339	0.070684	0.542904501	0.82871401
1005.125	1085.973	1200.956	-0.15653	0.453640482	0.463635155
4458.287	4636.518	4984.073	-0.27863	0.717935188	0.031877471
280.5415	313.9938	267.2285	0.06654	0.475821599	0.84162581
276.9677	230.386	280.8519	-0.11818	0.430461737	0.709201387
10.72133	8.360782	2.09591	-1.06514	0.935100162	NA
5.360666	2.786927	0	-2.01263	NA	NA
6514.996	5912.002	6229.044	0.090116	0.658567464	0.48354547
3.573777	0	0	#DIV/0!	NA	NA

209.066	297.2722	312.2906	-0.13403	0.734526609	0.72003423
2222.889	2023.309	2200.705	-0.11932	0.913769516	0.40615036
38.4181	26.01132	31.43865	-0.32305	0.918350023	0.658487105
316.2793	330.7154	328.0099	-0.3229	0.04906058	0.224319643
1915.544	1962.926	2231.096	-0.37123	0.504924333	0.006271401
6947.423	6582.722	7581.954	0.236056	0.960956671	0.208056675
236.7627	249.8945	309.1467	-0.26814	0.927256678	0.317595626
8.934443	7.431806	4.19182	1.964376	NA	NA
555.7223	584.3258	644.4923	0.07436	0.759261525	0.808870475
2536.488	2427.414	3096.707	-0.22649	0.082930432	0.295310061
546.7879	706.0216	579.5191	-0.1962	0.587719544	0.422560599
1911.077	2041.889	1986.922	-0.0448	0.883354954	0.855100406
13.40166	24.15337	14.67137	-0.12783	0.820929887	NA
29.48366	13.00566	14.67137	0.18991	0.579415204	NA
320.7465	238.7468	310.1946	-0.0759	0.938838346	0.859552774
1406.281	1353.518	1358.15	0.192139	0.727531507	0.112687221
2482.882	2590.913	2755.073	-0.03976	0.80037736	0.81253834
35.73777	30.6562	30.39069	-0.30738	0.40292054	NA
58.96732	71.53113	59.73343	-0.04009	0.98409529	0.962321489
1069.453	1184.444	889.7137	0.250576	0.362090257	0.433694723
6.25411	13.00566	8.383639	-0.72303	0.880489034	NA
173.3282	185.7952	225.3103	0.177308	0.641408722	0.640053604
1567.101	1522.591	1572.98	-0.05673	0.034815958	0.712929046
439.5746	460.772	462.1481	0.078458	0.377099016	0.751258336
1666.274	1746.474	1696.639	0.181202	0.29215357	0.172785623
1790.462	1571.827	1620.138	0.366346	0.668508083	0.028626623
7.147554	4.644879	0	-1.08262	NA	NA
1485.798	1527.236	1586.604	-0.16231	0.923053858	0.248501551
2878.677	2821.299	2647.134	0.260096	0.107676441	0.181593979
4.467221	8.360782	8.383639	-0.44751	0.774045793	NA
2890.292	2869.606	2723.635	-0.10252	0.100000333	0.442655501
259.0988	224.8121	290.2835	0.346324	0.730204291	0.284997366
473.5255	445.9084	398.2229	-0.12213	0.732779178	0.596761405
2146.947	2082.764	1978.539	-0.11475	0.022055853	0.3930505
18270.04	18109.45	19192.25	-0.43315	0.794232621	0.000984217
2297.939	2171.016	2117.917	0.052463	0.034700814	0.814086627
838.9442	755.2573	970.4062	0.066296	0.652050398	0.771436828
2500.75	2550.967	2653.422	-0.14692	0.325126896	0.380129121
476.2058	427.3289	469.4838	0.146757	0.368034878	0.55088846
544.1076	533.2321	540.7447	-0.02666	0.919799198	0.923725803
248.3775	256.3973	243.1255	-0.23329	0.592795512	0.414929365
597.7142	565.7462	454.8124	0.169914	0.184818229	0.605438344
0	0.928976	0	#DIV/0!	NA	NA
7202.054	7189.343	7980.177	-0.16615	0.859167103	0.224223422
0	0.928976	1.047955	-3.20111	NA	NA
1374.117	1286.631	1191.525	0.022068	0.600467264	0.923910484
20893.19	20190.36	22921.92	-0.44262	0.787835013	0.000914912
0	1.857952	2.09591	-1.35566	NA	NA
712.0751	739.4647	675.9309	0.346744	0.730441275	0.131442568
630.7716	653.9989	528.1693	0.026014	0.655321049	0.94960537
130.4429	141.2043	173.9605	-0.47302	0.444234906	0.110387265
22.33611	26.9403	20.9591	0.32789	0.66963368	NA
891.6574	1020.015	901.2412	-0.03587	0.005036606	0.882926075
499.4353	510.9367	471.5797	-0.03735	0.714180196	0.893531532

54.5001	101.2584	97.45981	0.248211	0.30299466	0.658487105
1689.503	1631.281	1847.544	-0.24847	0.434274199	0.093593
8.934443	15.79259	11.5275	0.712017	0.862400346	NA
425.2795	428.2578	397.1749	0.186398	0.068025474	0.36518668
558.4027	529.5162	563.7997	0.226582	0.901132408	0.362764905
2337.25	2242.547	2260.439	-0.12729	0.007079262	0.348491003
353.8039	330.7154	280.8519	0.202252	0.914186189	0.535992604
1947.708	1877.46	2026.745	-0.01667	0.813154117	0.936747437
444.9352	543.4508	596.2863	-0.03698	0.16161829	0.908612163
962.2395	923.4019	839.4119	0.17259	0.802750793	0.316260034
13489.22	13159.87	13041.8	-0.10568	0.025591463	0.253027668
828.2228	787.7714	915.9126	0.063658	0.18468856	0.800895715
56480.87	56184.45	56331.77	-0.25761	0.92288341	0.034382552
807.6736	771.0499	833.1241	-0.08574	0.757209202	0.719971458
252.8447	276.8348	265.1326	0.17125	0.600642134	0.510255874
2186.258	2157.082	2311.788	-0.33037	0.025591463	0.002333217
173.3282	110.5481	134.1382	-0.2126	0.241828097	0.583691606
4783.501	5010.895	5177.945	0.111508	0.745403683	0.339045793
38.4181	60.38342	55.54161	-0.36485	0.824080056	0.497130933
2495.39	2497.087	2565.394	-0.16829	0.671378444	0.317944832
0.893444	0	1.047955	0.203136	NA	NA
422.5991	433.8317	391.9351	0.068656	0.597339281	0.774877994
1893.208	2045.605	1898.894	0.214314	0.798030968	0.08210346
991.7231	966.1348	958.8787	-0.03057	0.50957694	0.920188536
9.827887	5.573855	10.47955	-0.88163	0.992476114	NA
1493.839	1613.631	1490.192	-0.05805	0.06343049	0.724404377
50.92632	57.5965	48.20593	-0.04794	0.152933305	0.953867552
1210.617	1244.828	1225.059	-0.15527	0.101166359	0.339089471
4488.664	4211.047	4036.722	-0.04278	0.001306775	0.780344055
4.467221	4.644879	6.287729	1.833671	NA	NA
1.786889	0.928976	0	-1.56985	NA	NA
40851.85	40003.55	39186.18	-0.35685	0.946929815	0.00684915
100.9592	87.32372	156.1453	0.09905	0.880647749	0.871061917
669.1897	745.9675	775.4866	-0.24319	0.826883467	0.198030129
152.779	206.2326	197.0155	0.036103	0.92288341	0.931618549
721.0095	848.1549	877.1382	-0.49031	0.71554501	0.003904411
3249.457	3327.591	3459.299	-0.12615	0.413714744	0.403397484
271.6071	353.0108	367.8322	0.396426	0.817060109	0.091639488
29.48366	68.74421	48.20593	0.560769	0.910682739	NA
2165.709	2213.749	2173.458	0.024523	0.866874007	0.876257447
465.4845	491.4282	479.9633	0.595238	0.729802201	0.012314933
219.7873	206.2326	183.3921	0.263379	0.794232621	0.370687445
395.7958	410.6073	478.9154	-0.09925	0.256151839	0.726635623
8.934443	8.360782	15.71932	0.308352	0.98299578	NA
2692.841	2989.444	2480.509	0.034642	0.47934099	0.896421455
473.5255	564.8173	448.5247	-0.23281	0.31883203	0.258091148
1.786889	0	4.19182	0.350297	NA	NA
1735.069	1634.068	1759.516	-0.10985	0.859974619	0.447363679
859.4934	803.564	753.4796	-0.01465	0.951118826	0.956290296
712.9685	702.3057	714.7052	0.146031	0.350762724	0.503879072
228.7217	215.5224	227.4062	0.098821	0.257483954	0.764162229
17388.21	17050.42	15920.53	0.015938	0.358649739	0.922703497
2305.98	2067.9	1969.107	0.044109	0.147662937	0.862092728
332.3613	318.6387	318.5783	-0.02727	0.801320748	0.953279068

0	0	1.047955	-0.68638	NA	NA
545.001	536.948	522.9295	0.251108	0.613451408	0.440703722
1013.166	892.7457	936.8717	0.211537	0.515985444	0.262359598
201.9184	274.9768	293.4274	-0.0535	0.505612976	0.896670492
311.812	358.5846	297.6192	0.142696	0.46017679	0.636454553
18758.76	18869.36	19760.24	-0.3679	0.484625827	0.002095105
75.04932	61.3124	81.74048	-0.10986	0.134933146	0.860281493
1364.289	1375.813	1384.348	0.045701	0.867574132	0.775119272
585.206	564.8173	710.5134	-0.37755	0.526676057	0.035698458
0	0	0	#DIV/0!	NA	NA
0	0	0	#DIV/0!	NA	NA
974.7477	1007.939	1006.037	-0.32279	0.515985444	0.007388645
1342.847	1182.586	1195.717	0.074652	0.368786861	0.767645401
0	0	0	#DIV/0!	NA	NA
0	0	0	#DIV/0!	NA	NA
0	0	0	#DIV/0!	NA	NA
0	0	0	#DIV/0!	NA	NA
503.0091	491.4282	466.3399	-0.10562	0.020838235	0.688662561
763.0014	648.4251	691.6502	0.260959	0.260444304	0.219522094
15.18855	22.29542	41.9182	-0.13082	0.98878626	NA
1119.486	1176.083	1327.759	-0.30581	0.866564571	0.049190168
568.2305	476.5646	497.7786	0.17522	0.867574132	0.505302546
37.52466	43.66186	34.58251	0.305373	0.485974888	0.656460739
16.97544	22.29542	16.76728	-0.18484	0.76103064	NA
526.2387	549.0247	534.457	0.124213	0.441489237	0.653404441
4191.147	4281.649	4576.419	-0.52995	3.50512E-06	3.97686E-08
734.4112	717.1693	577.4231	0.347398	0.411467295	0.139322477
24335.63	24341.02	23951.01	-0.31265	0.70781734	0.044732366
968.4936	810.9958	736.7123	0.28561	0.899176451	0.161107365
292.1563	292.6274	333.2497	-0.05781	0.382918803	0.869374053
880.0426	819.3566	867.7067	0.036872	0.019363982	0.885571199
1988.807	2092.053	2130.492	0.020486	0.496167264	0.912679298
370.7794	409.6783	390.8872	0.122927	0.817609984	0.628282957
3.573777	0	0	-0.50147	NA	NA
0	0.928976	1.047955	0.229302	NA	NA
1030.141	921.544	859.323	0.177255	0.617661583	0.394044776
882.7229	1006.081	915.9126	0.17189	0.526561206	0.365253762
106.3199	83.60782	102.6996	0.035161	0.965666941	0.961026715
2.680333	0.928976	2.09591	0.758321	NA	NA
3890.056	4202.686	4089.12	-0.19444	0.660114316	0.047449206
4930.919	4854.827	4804.873	-0.15935	0.333641544	0.131512208
280.5415	319.5677	219.0226	0.010195	0.446999299	0.984456511
15.18855	6.50283	16.76728	-0.00809	0.590474301	NA
1221.338	1215.1	1369.677	0.064606	0.214235831	0.794250417
0	5.573855	4.19182	0.701114	NA	NA
253.7382	262.9001	241.0296	0.257133	0.941323092	0.387856411
354.6974	405.0334	356.3047	0.044189	0.596065856	0.907473183
2650.849	2643.865	2459.55	0.167681	0.231422023	0.335454169
1341.953	1426.907	1256.498	0.052727	0.033849408	0.808493325
745.1325	739.4647	662.3075	-0.06224	0.367802825	0.797601985
949.7312	993.0751	1096.161	0.211148	0.349682314	0.251658635
2524.873	2372.604	2321.22	0.042118	0.014661969	0.875604624
1185.601	1251.33	1022.804	0.260221	0.083019794	0.186989586
100.9592	143.0623	77.54866	-0.17616	0.596065856	0.752110266

0	0	1.047955	#DIV/0!	NA	NA
0	0	1.047955	#DIV/0!	NA	NA
0	0	1.047955	#DIV/0!	NA	NA
0	0	1.047955	#DIV/0!	NA	NA
2992.145	3197.535	2919.602	-0.14322	0.010013524	0.321405678
0	0.928976	0	#DIV/0!	NA	NA
0	0.928976	0	#DIV/0!	NA	NA
0	0.928976	0	#DIV/0!	NA	NA
0.893444	0	0	#DIV/0!	NA	NA
0.893444	0	0	#DIV/0!	NA	NA
0.893444	0	0	#DIV/0!	NA	NA
0.893444	0	0	#DIV/0!	NA	NA
0.893444	0	0	#DIV/0!	NA	NA
1773.487	1920.193	1942.908	-0.09638	0.065480816	0.569109784
724.5833	663.2887	697.938	0.131352	0.603004299	0.536510619
436.8942	482.1384	477.8674	0.173673	0.981076246	0.556186772
0	0	0	#NUM!	NA	NA
2834.005	2955.072	2465.838	0.075329	0.396359861	0.724404377
1363.396	1412.043	1205.148	0.200663	0.46017679	0.318625647
1791.356	1692.594	1957.58	-0.20428	0.430087902	0.114544408
854.1327	783.1266	833.1241	0.078753	0.618280897	0.752110266
787.1244	787.7714	746.1439	0.235835	0.752287766	0.278856737
597.7142	552.7406	542.8406	0.13027	0.503345543	0.502183544
240.3365	239.6757	211.6869	0.047084	0.682715357	0.899275854
174.2216	140.2753	148.8096	-0.00377	0.2795135	0.996992829
1725.241	1690.736	1589.748	-0.22513	0.367802825	0.045823334
820.1818	909.4673	820.5487	-0.03185	0.033512898	0.896567419
11.61478	7.431806	6.287729	-0.80662	0.871801928	NA
1245.461	1198.379	1279.553	0.039925	0.6771199	0.869372302
437.7877	405.0334	320.6742	-0.00111	0.948234757	0.999921357
6914.365	7006.335	6902.879	-0.01587	0.301520393	0.924154584
77.72965	88.2527	66.02116	0.101582	0.844410461	0.886786965
675.4439	540.6639	613.0536	-0.12265	0.97820156	0.645108746
195.6643	259.1842	266.1805	-0.18776	0.021160717	0.576050724
15113.5	14782.79	14965.84	-0.17909	0.612715269	0.136963708
775.5096	706.0216	914.8646	-0.50904	0.768012666	0.005818131
392.222	403.1755	360.4965	-0.27446	0.1759595	0.146138686
3093.997	3043.325	3382.798	-0.08875	0.981637588	0.602240104
276.9677	235.0309	274.5642	-0.20947	0.230144662	0.420106203
1179.346	1125.919	1186.285	-0.1112	0.290962131	0.586606456
1279.412	1277.342	1488.096	0.002409	0.838656637	0.993021449
1.786889	4.644879	13.62341	0.38231	NA	NA
897.018	829.5754	958.8787	-0.24506	0.445880938	0.070104672
150.9921	186.7241	199.1114	-0.31326	0.946065985	0.309801385
938.1165	953.1291	927.4401	0.083737	0.489992231	0.787476613
147.4183	162.5708	111.0832	0.173085	0.337999896	0.74588273
9260.55	9313.911	11885.9	-0.53077	0.715641565	0.000501849
1461.675	1517.946	1381.205	-0.02491	0.739965249	0.902386847
14664.99	14505.96	15230.98	-0.27412	0.916166526	0.040829913
1704.692	1684.233	1482.856	0.063473	0.136814544	0.721425275
9428.517	9551.729	9342.518	-0.11665	0.983970277	0.30864647
1.786889	0.928976	2.09591	-1.13069	NA	NA
168.861	164.4287	177.1044	0.674409	0.66770133	0.060851524
0.893444	1.857952	1.047955	-1.12238	NA	NA

855.9196	785.9135	879.2342	-0.11373	0.847624712	0.559214222
2641.915	2720.041	2801.183	-0.07402	0.994002024	0.658318551
3.573777	8.360782	3.143865	-0.34326	0.813154117	NA
422.5991	443.1214	390.8872	0.397434	0.962011634	0.165590641
947.0509	945.6973	822.6446	0.226665	0.999122571	0.180352621
2494.496	2230.471	2382.001	-0.13492	0.324651337	0.484163033
322.5334	291.6984	315.4344	-0.0122	0.327341	0.96505606
141.1642	115.193	126.8025	0.317941	0.426622274	0.563375937
4.467221	7.431806	8.383639	0.29404	0.897766774	NA
301.9842	185.7952	238.9337	-0.22583	0.968581517	0.520301247
2943.005	2951.356	2651.326	0.156743	0.957754117	0.486229634
334.1482	385.5249	350.0169	-0.24279	0.837998472	0.237500588
2174.643	2076.261	2140.972	0.034695	0.959690198	0.848456292
2599.029	2374.462	2231.096	0.086101	0.005997911	0.696921855
626.3044	599.1894	637.1566	0.067304	0.707092679	0.765789524
53.60666	79.89192	46.11002	0.203152	0.794232621	0.748708061
3524.638	3264.421	3166.92	0.111364	0.022069615	0.559214222
544.1076	660.5018	606.7659	-0.27975	0.975298616	0.119347432
61.64765	45.51981	72.30889	0.291541	0.937806584	0.676538655
1910.184	1785.491	1687.207	0.172185	0.269120346	0.472093197
24.12299	31.58518	41.9182	0.028875	0.892240442	NA
310.9186	394.8147	389.8392	-0.15808	0.85272133	0.567139643
2995.719	2893.759	2456.406	-0.00434	0.735300868	0.990142758
5414.272	5404.781	5311.035	0.111565	0.218540286	0.317595626
170.6479	143.9912	124.7066	0.275659	0.856763291	0.522839098
744.2391	592.6865	648.6841	-0.10563	0.608976999	0.660222897
352.017	300.0592	323.8181	0.297714	0.080445087	0.1869705
1340.166	1382.316	1341.382	-0.02393	0.12999813	0.896421455
83.98376	81.74987	78.59662	0.186051	0.817712901	0.703453769
1534.937	1457.563	1607.563	0.16058	0.517968435	0.278856737
702.2472	711.5954	795.3978	-0.05498	0.310114043	0.822050288
528.919	543.4508	572.1834	-0.00644	0.352663598	0.988943755
7746.162	7840.555	6856.769	0.008524	0.590879965	0.990023409
1286.56	1458.492	1199.908	-0.16903	0.976661259	0.353465216
1944.135	2119.923	2216.425	-0.18562	0.804201321	0.241619328
248.3775	230.386	247.3174	-0.07676	0.907472172	0.838059844
927.3951	992.1461	934.7758	0.277891	0.760227016	0.162293367
1846.749	1719.534	1681.968	0.025908	0.825444223	0.91372954
2041.52	1896.969	2165.075	0.09093	0.469192599	0.656162087
41533.54	40559.08	42057.57	-0.43407	0.766887678	0.001503374
486.0337	549.9537	551.2243	0.014697	0.532750573	0.96009156
496.755	575.965	599.4302	-0.07968	0.402252256	0.726635623
882.7229	916.8991	796.4457	0.025369	0.246680945	0.915970521
814.8212	930.8337	816.3569	0.049021	0.503345543	0.854044896
1733.282	1674.943	1768.948	0.202656	0.755224646	0.284997366
0	0.928976	1.047955	0.586005	NA	NA
3118.12	3043.325	3338.784	-0.02724	0.226746984	0.872508626
674.5504	693.9449	797.4937	-0.01097	0.968325512	0.974691849
1081.961	1065.535	1131.791	0.165164	0.233378357	0.523725333
1168.625	1164.007	1085.681	0.096885	0.794232621	0.615745925
3467.457	3531.037	3176.351	-0.08265	0.001009169	0.683195977
51.81977	52.02264	50.30183	0.557328	0.470002734	0.360145579
1.786889	0	3.143865	#DIV/0!	NA	NA
23.22955	23.22439	15.71932	-0.70697	0.302724041	NA

588.7798	594.5445	615.1495	0.329936	0.201936757	0.051992282
101.8526	115.193	164.5289	0.48839	0.827474725	0.2912749
810.3539	764.5471	804.8294	0.022133	0.613734914	0.923725803
271.6071	274.0478	201.2073	-0.10805	0.327341	0.775439768
2448.931	2630.859	2354.755	-0.06908	0.175031997	0.678741853
353.8039	319.5677	417.086	0.217624	0.430461737	0.432540736
627.1979	678.1523	674.883	-0.13327	0.926742921	0.505493145
1081.961	1122.203	1063.674	0.353187	0.182855445	0.021007242
1257.076	1258.762	1189.429	0.064978	0.462276826	0.739117108
721.0095	733.8909	769.1989	0.062912	0.787792061	0.802401887
940.7968	1067.393	999.749	0.556127	0.03968658	0.000601979
243.9103	257.3263	242.0776	0.368836	0.879847555	0.148994093
1039.076	1101.765	1059.482	0.070301	0.502258444	0.786706509
679.9111	893.6747	748.2398	-0.15135	0.824114534	0.576591259
0	0	3.143865	#DIV/0!	NA	NA
61.64765	34.3721	47.15797	0.367692	0.490249604	0.596309029
0.893444	1.857952	0	0.062907	NA	NA
593.247	578.7519	624.5811	0.105992	0.766253365	0.690294956
247.4841	242.4627	263.0367	0.181967	0.898465596	0.544356068
97.38542	90.11065	98.50776	-0.28097	0.317073718	0.495283424
268.0333	241.5337	317.5303	0.019663	0.649714874	0.9717805
2551.677	2617.854	2309.693	0.135523	0.167511517	0.346094927
1713.626	1874.673	1792.003	0.073326	0.051978056	0.673302041
1180.24	1063.677	1030.14	0.202126	0.394915233	0.41069386
377.9269	347.4369	343.7292	0.443589	0.03968658	0.16141555
538.7469	471.9197	531.3131	-0.17936	0.946929815	0.39276575
1329.445	1196.521	1395.876	-0.46086	7.34828E-05	0.000739443
3.573777	0.928976	2.09591	-0.59366	NA	NA
227.8283	188.5821	233.6939	0.037702	0.025033988	0.928448576
3.573777	0	4.19182	0.203136	NA	NA
844.3048	973.5666	846.7476	-0.12677	0.686179051	0.538565977
407.4106	404.1045	358.4006	-0.01502	0.828379866	0.965364339
1265.117	998.6489	1221.915	-0.08891	0.897898013	0.71268973
8.040998	5.573855	8.383639	-0.82771	0.072756064	NA
415.4516	362.3005	388.7913	-0.02811	0.185762147	0.930744684
352.9105	325.1415	326.9619	0.04664	0.54674796	0.896421455
981.0018	1009.797	1099.305	-0.39337	0.778796446	0.01252509
15256.45	14770.71	14769.88	-0.02567	0.335983122	0.875604624
671.8701	775.6948	684.3145	-0.09733	0.141058983	0.678003973
6.25411	3.715903	6.287729	1.625798	NA	NA
229.6152	203.4457	213.7828	-0.10252	0.433690703	0.795664302
863.9606	716.2403	760.8153	0.041972	0.672813076	0.877444629
824.6491	920.615	785.9662	0.019868	0.005734939	0.948840677
952.4116	967.9927	886.5698	-0.02995	0.58195463	0.884746998
9474.976	9195.002	11331.54	-0.06726	0.962556121	0.778766112
1099.83	1067.393	1113.976	0.072854	0.446999299	0.75448001
1192.748	1214.171	1054.243	0.174692	0.44839829	0.429681987
1.786889	0.928976	0	#DIV/0!	NA	NA
1117.699	995.862	998.701	0.365718	0.499877044	0.163200347
0	0	0	#DIV/0!	NA	NA
0	0	0	#DIV/0!	NA	NA
0	0	0	#NUM!	NA	NA
0	0	0	#DIV/0!	NA	NA
0	0	0	#DIV/0!	NA	NA

0	0	0	#DIV/0!	NA		NA	
0	0	0	#DIV/0!	NA		NA	
0	0	0	#DIV/0!	NA		NA	
0	0	0	#DIV/0!	NA		NA	
0	0	0	#DIV/0!	NA		NA	
0	0	0	#DIV/0!	NA		NA	
0	0	0	#DIV/0!	NA		NA	
0	0	0	#DIV/0!	NA		NA	
0	0	0	#DIV/0!	NA		NA	
43.77877	26.01132	22.00705	0.250047		0.818823133	NA	
1527.79	1445.486	1532.11	-0.08513		0.896968012		0.622470399
7186.866	6803.818	7117.71	-0.04161		0.986125936		0.83508691
660.2553	700.4477	692.6982	0.01161		0.743726011		0.971658868
0	0.928976	0	-3.18218	NA		NA	
433.3205	452.4112	446.4288	0.055885		0.858075407		0.872630949
768.3621	742.2516	847.7955	-0.22767		0.824111765		0.324170132
217.107	238.7468	248.3653	-0.42301		0.860108299		0.045393537
1320.511	1240.183	1291.08	-0.20243		0.032629043		0.316402463
290.3694	307.491	280.8519	-0.02947		0.916416444		0.933856082
1.786889	2.786927	3.143865	-0.84976	NA		NA	
5126.583	5330.463	5845.492	-0.2272		0.232941496		0.06712239
0	0	1.047955	-2.91464	NA		NA	
802.3129	810.9958	731.4725	0.009506		0.68563527		0.964974564
1090.002	1016.299	1168.47	0.070144		0.813154117		0.723760266
1802.077	1765.983	1969.107	0.158983		0.69338056		0.527083496
1.786889	0	2.09591	0.55984	NA		NA	
3947.237	4044.76	4472.671	-0.12508		0.837998472		0.406585578
0	2.786927	3.143865	0.338688	NA		NA	
1751.151	1833.798	1863.264	-0.1014		0.944097019		0.420675433
35199.92	34772.49	35241.68	-0.3735		0.713016559		0.002890232
0	0	3.143865	#DIV/0!	NA		NA	
154.5659	174.6474	165.5769	0.629107		0.842190796		0.118018607
2675.866	2706.106	2421.824	0.15798		0.036478363		0.422498267
0	3.715903	0	#DIV/0!	NA		NA	
275.1808	341.8631	319.6262	-0.10311		0.569085135		0.754561878
854.1327	743.1806	780.7264	0.248301		0.599504319		0.155370416
2161.242	2197.028	2000.546	0.158467		0.312337571		0.46008868
247.4841	241.5337	281.8999	0.09742		0.209524948		0.80177911
2.680333	4.644879	0	1.286265	NA		NA	
1220.445	1219.745	1191.525	0.073137		0.621093622		0.813492611
3501.408	3602.568	3201.502	-0.02779		0.106967826		0.895919028
0	0.928976	2.09591	#DIV/0!	NA		NA	
1350.888	1450.131	1639.001	-0.32937		0.648624949		0.037459793
2737.513	2892.831	2697.436	0.105503		0.004142595		0.639253997
706.7144	843.51	756.6234	0.086291		0.112535001		0.733719596
742.4522	865.8054	779.6784	-0.29417		0.4069354		0.068011569
38.4181	13.00566	25.15092	0.140934		0.976681492	NA	
394.9024	405.0334	318.5783	0.174488		0.907551741		0.55088846
1584.077	1673.085	1687.207	-0.06794		0.492727322		0.752110266
2041.52	2034.457	1997.402	-0.04962		0.920564616		0.783572702
0.893444	0	2.09591	-1.59173	NA		NA	
160.82	186.7241	173.9605	0.169892		0.529229854		0.665181522
69.68865	97.54245	99.55571	0.094001		0.234629688		0.880577976
3848.064	3775.357	3615.444	-0.06867		0.994679365		0.566742383

517.3042	457.9851	427.5656	-0.2096	0.573411006	0.362995196
444.9352	461.701	434.9013	0.228469	0.310443697	0.344955641
18756.97	18882.36	20484.37	-0.41775	0.43229338	0.000594901
3.573777	0.928976	6.287729	-0.92204	0.648256394	NA
343.0826	399.4596	440.1411	-0.0277	0.826883467	0.924895748
792.4851	861.1605	788.0621	0.164776	0.817609984	0.397126214
410.0909	373.4483	402.4147	0.029018	0.798409569	0.924154584
1160.584	1021.873	1060.53	-0.11	0.976681492	0.579998211
3.573777	3.715903	3.143865	#DIV/0!	NA	NA
802.3129	828.6464	838.3639	0.126994	0.947529145	0.500602324
17.86889	26.01132	17.81523	0.908088	0.903271991	NA
688.8455	771.9789	755.5755	0.30911	0.9397169	0.072626489
535.1731	466.3458	479.9633	-0.00344	0.855606042	0.992528568
1128.42	1181.657	1143.319	-0.27858	0.744600754	0.056859994
487.8206	522.0844	597.3343	0.114788	0.856745942	0.666030043
516.4108	593.6155	427.5656	0.107744	0.709468629	0.72969638
910.4197	795.2033	819.5007	0.311565	0.812875146	0.076772392
0	0	5.239774	#DIV/0!	NA	NA
3.573777	2.786927	3.143865	0.019084	NA	NA
204.5987	150.4941	185.488	-0.13193	0.743382516	0.714941358
212.6397	210.8775	230.5501	-0.11143	0.703609106	0.739592868
83.98376	107.7612	190.7278	-0.38424	0.619720339	0.407303742
113.4674	117.9799	114.2271	-0.1164	0.84819607	0.765144842
0	0	1.047955	#DIV/0!	NA	NA
962.2395	979.1405	1046.907	0.181529	0.820929887	0.239197322
1324.978	1304.282	1285.841	0.138769	0.518546793	0.361564535
0	5.573855	0	-0.01636	NA	NA
2731.259	2688.456	2593.688	0.027396	0.010350895	0.875604624
331.4678	386.4539	357.3526	0.485603	0.733351868	0.021007242
1760.085	1724.179	1807.722	0.249733	0.134933146	0.056160428
809.4605	814.7117	970.4062	0.042374	0.114505424	0.894414367
3271.793	3070.265	2565.394	0.067688	0.458486853	0.806564543
1066.772	1010.726	1010.229	0.199818	0.064620826	0.332512918
520.878	505.3628	486.2511	0.175138	0.614621507	0.499732235
1412.535	1337.725	1379.109	-0.10577	0.338642963	0.697416669
571.8043	479.3515	456.9083	0.152296	0.828016939	0.625382385
3982.081	3878.474	4248.409	-0.19318	0.939162869	0.21731463
1802.077	1939.701	1913.566	0.031787	0.934088907	0.855100406
5084.591	5174.395	6086.522	0.282518	0.406883355	0.057034038
3528.211	3652.733	3451.963	-0.3936	0.006229103	4.66726E-07
654.0012	549.0247	681.1707	0.074854	0.09014688	0.793207865
330.5744	439.4055	429.6615	0.076849	0.817060109	0.8325893
1429.511	1437.126	1444.082	-0.22697	0.078514584	0.324099572
586.0994	595.4735	654.9718	0.019238	0.828016939	0.952588397
111.6805	87.32372	99.55571	0.155903	0.6771199	0.762128192
3741.745	3887.764	4301.855	-0.2261	0.938838346	0.071238709
365.4187	436.6186	447.4767	-0.10362	0.660114316	0.708596546
18.76233	28.79825	28.29478	-0.02815	0.84819607	NA
237.6562	209.0195	183.3921	-0.04163	0.748298735	0.919915102
995.2969	983.7853	923.2483	-0.00418	0.669860929	0.988370025
4883.566	4784.225	5342.474	0.00972	0.365690431	0.956290296
1315.15	1532.81	1596.035	-0.10426	0.962660515	0.656489824
0	0.928976	0	-3.3109	NA	NA
589.6732	580.6099	525.0254	0.171937	0.617661583	0.491917195

282.3284	301.9171	310.1946	-0.38291	0.547164305	0.129705424
1156.117	1072.038	1142.271	0.136684	0.499740672	0.452447922
536.0666	472.8487	418.134	0.14042	0.720425175	0.602036712
203.7053	209.9485	212.7348	0.100345	0.485574782	0.794150873
612.9028	587.1127	540.7447	0.078263	0.040735143	0.739592868
109.0002	113.335	96.41185	0.490188	0.919747739	0.394438057
1216.871	1205.811	1067.866	-0.17544	0.71034712	0.351487828
18311.14	18172.62	18244.89	-0.3297	0.6333028	0.02032709
9890.428	9987.418	9853.92	-0.06035	0.257606642	0.684131921
464.591	444.0504	479.9633	-0.23277	0.511726855	0.38622273
930.0755	866.7344	944.2074	0.15347	0.203841572	0.49670864
145.6314	138.4174	132.0423	-0.59406	0.831928191	0.139776747
438.6811	378.0931	366.7842	-0.54003	0.357841712	0.017241442
3600.58	3690.821	3330.401	0.05296	0.573411006	0.80030609
2750.915	2636.433	2851.485	-0.01573	0.990604058	0.948957763
820.1818	924.3309	788.0621	0.050388	0.827268091	0.839609028
8.934443	1.857952	4.19182	-1.63966	0.999199613	NA
165.2872	190.44	232.646	-0.3716	0.910726255	0.199496415
547.6813	520.2264	624.5811	0.231837	0.817609984	0.255582054
731.7308	614.982	810.0691	0.050113	0.235856061	0.884584155
14.29511	14.86361	25.15092	0.200352	0.837529432	NA
1814.585	1726.037	1892.607	-0.0858	0.936346196	0.694075461
3455.842	3225.404	3421.573	0.082253	0.22919987	0.600739068
301.9842	281.4797	297.6192	-0.22168	0.71270119	0.422793542
1609.093	1616.418	1462.945	0.155952	0.169117813	0.375904486
5753.781	6298.456	5938.76	-0.10405	0.831143588	0.499455038
553.042	535.09	520.8336	-0.00619	0.368085564	0.977548356
1.786889	4.644879	7.335684	0.024094	NA	NA
45.56566	52.95162	59.73343	-0.86132	0.92288341	0.102132739
233.189	211.8065	293.4274	-0.36688	0.71034712	0.171952931
14.29511	9.289758	8.383639	0.424564	0.817609984	NA
852.3458	718.0983	594.1904	0.231259	0.226961756	0.40615036
83.09032	36.23005	51.34979	0.203556	0.682715357	0.840777068
1623.388	1680.517	1502.767	0.142706	0.795376015	0.38666632
1266.011	1391.606	1221.915	0.034722	0.457531489	0.878767667
33.95088	24.15337	50.30183	0.432014	0.681419018	NA
1999.528	2326.155	2363.138	-0.08456	0.455280748	0.668686407
7.147554	4.644879	12.57546	-0.18015	0.948234757	NA
0.893444	0.928976	3.143865	-0.91737	NA	NA
1629.642	1712.102	1620.138	0.122472	0.610382096	0.351487828
2728.579	2857.529	2739.354	-0.16934	0.197190189	0.142939621
1003.338	1005.152	780.7264	0.265244	0.572336545	0.267586812
1795.823	1788.278	2013.121	-0.04811	0.102371821	0.870656158
657.575	719.9562	780.7264	0.126283	0.469683278	0.600553967
276.9677	249.8945	250.4612	0.050507	0.679066538	0.882926075
3094.891	3113.927	3349.264	0.480866	0.840099446	6.42867E-06
328.7875	279.6217	321.7222	-0.07253	0.497263297	0.815439094
1753.831	1926.696	1692.447	0.091556	0.220715145	0.658878607
0	5.573855	0	-1.10793	NA	NA
132.2298	86.39475	147.7616	-0.28529	0.843186128	0.44144154
167.9675	153.281	209.591	0.368058	0.38025264	0.413703728
1806.544	1607.128	1623.282	0.118575	0.226842122	0.421538281
805.8867	796.1322	783.8703	-0.02488	0.207380345	0.914120833
299.3038	333.5023	296.5712	-0.02012	0.959859312	0.953528509

1.786889	1.857952	1.047955	#DIV/0!	NA	NA	
4899.648	4619.796	5134.979	0.080729	0.72676049		0.626829627
11024.21	11028.8	10299.3	-0.04231	0.985859213		0.802920813
2261.307	2367.959	1900.99	0.050396	0.469192599		0.854337819
116.1478	161.6418	142.5219	0.217455	0.080905938		0.613026721
773.7227	842.581	951.543	-0.46299	0.017639055		0.00145335
6104.905	6022.55	5926.185	0.053512	0.62498123		0.745567347
1823.52	1833.798	1802.482	-0.1766	0.691366556		0.162970478
166.1806	168.1446	185.488	-0.22106	0.759261525		0.436291984
877.3623	750.6124	753.4796	0.064266	0.447208798		0.780325172
338.6154	366.9454	267.2285	-0.06949	0.27384839		0.87466351
328.7875	317.7097	294.4753	0.102135	0.848694292		0.720614012
694.2062	743.1806	782.8223	0.010933	0.698024229		0.9717805
1459.888	1360.021	1380.157	-0.11094	0.967813477		0.55088846
12630.62	12587.62	12392.07	-0.29705	0.74589429		0.009939038
971.1739	853.7287	877.1382	0.138631	0.6333028		0.498608433
431.5336	480.2805	479.9633	-0.00325	0.585115533		0.988966742
736.1981	605.6922	606.7659	0.230705	0.536343685		0.21731463
48.24599	52.95162	52.39774	0.042285	0.937806584		0.973573759
5744.847	5537.625	5919.897	-0.15226	0.989430108		0.253946352
1587.65	1485.432	1287.937	0.118836	0.307348283		0.505302546
454.7631	412.4652	347.921	0.087679	0.551840208		0.775439768
54.5001	66.88625	58.68547	-0.0475	0.191229725		0.936563036
1129.314	1184.444	1075.202	0.286996	0.231538797		0.095730045
851.4524	939.1945	828.9323	0.13286	0.59039068		0.505302546
50.92632	73.38909	75.45275	0.59685	0.978835583		0.333065318
971.1739	1006.081	891.8096	0.016986	0.706962639		0.946838074
41.09844	42.73289	54.49365	0.203385	0.826883467		0.767645401
70.5821	64.09933	75.45275	-0.28825	0.513499171		0.552800466
4863.911	4886.413	4723.133	0.095825	4.17743E-05		0.536510619
943.4771	976.3535	1147.511	-0.40821	0.810878938		0.033645271
300.1973	350.2239	343.7292	-0.17411	0.138269747		0.507147953
237.6562	270.3319	215.8787	0.666015	0.090068897		0.15455096
846.9852	893.6747	930.5839	0.201688	0.164920979		0.251743462
394.9024	429.1868	414.9901	-0.20195	0.373011749		0.433949395
0	0	0	#NUM!	NA	NA	
4.467221	11.14771	1.047955	-0.06595	0.594712518	NA	
2188.938	2127.354	2160.883	0.096396	0.268223584		0.599614212
1321.404	1369.31	1536.302	-0.20811	0.621093622		0.325427264
1014.059	1009.797	958.8787	0.177018	0.413905026		0.353465216
308.2383	336.2892	287.1396	-0.11628	0.555044592		0.682436785
709.3947	735.7488	710.5134	0.144591	0.203841572		0.610317713
4276.918	4185.036	3661.554	0.111502	0.187509131		0.515247485
14554.21	14600.71	15600.9	-0.27241	0.936346196		0.027333973
0.893444	2.786927	0	0.482594	NA	NA	
461.9107	480.2805	543.8886	0.016448	0.378784486		0.952761149
11.61478	5.573855	12.57546	0.538381	0.972370311	NA	
1170.412	1190.947	1153.798	0.06215	0.857370872		0.729827413
811.2474	796.1322	779.6784	0.306927	0.494492788		0.061045315
1674.315	1590.407	1615.946	-0.13028	0.003735039		0.38935837
4472.582	4388.481	4013.667	-0.01055	0.454541325		0.961026715
853.2393	860.2316	837.316	0.294045	0.412158288		0.060691739
49.13943	66.88625	56.58956	-0.29633	0.618280897		0.575540189
641.493	625.2007	615.1495	0.117522	0.935100162		0.502996806

6.25411	1.857952	9.431594	-0.32468	0.938838346	NA
0	0	0	#NUM!	NA	NA
0	0	3.143865	-0.10142	NA	NA
0	0	0	#NUM!	NA	NA
238.5496	233.1729	212.7348	-0.30901	0.835449534	0.229480039
600.3945	589.8996	517.6897	0.069098	0.991590779	0.836257824
19711.17	19597.67	20091.39	-0.20509	0.868269106	0.053731064
1340.166	1324.719	1315.183	0.078471	0.779325825	0.68795231
0	1.857952	2.09591	1.229302	NA	NA
1777.061	1724.179	1863.264	0.049569	0.504339531	0.838059844
619.1569	470.9907	558.56	-0.04998	0.323363221	0.851146657
354.6974	404.1045	450.6206	0.103512	0.84602472	0.713323797
0.893444	0	0	-2.39209	NA	NA
2355.119	2234.187	2568.537	-0.29291	0.882057909	0.033645271
64.32799	55.73855	57.63752	-0.33972	0.362841481	0.489722452
378.8204	438.4766	396.1269	0.087109	0.732718278	0.739117108
4187.573	4103.286	4433.897	0.021375	0.235856061	0.923725803
93.81165	85.46577	64.9732	-0.1465	0.675005225	0.806564543
2251.48	2153.366	2057.135	-0.01725	0.000445095	0.923725803
3363.818	3385.188	3085.179	0.142625	0.290962131	0.515558505
861.2803	866.7344	963.0705	-0.28635	0.187777748	0.088148215
2253.266	2223.039	2105.341	0.121405	0.136711904	0.348491003
10.72133	9.289758	4.19182	-0.57446	0.777234647	NA
434.2139	396.6727	444.3329	0.238544	0.265804194	0.335635384
755.8538	823.0725	779.6784	-0.02289	0.187777748	0.933856082
1003.338	928.0468	949.4471	0.031349	0.962660515	0.875604624
1327.658	1328.435	1282.697	-0.01762	0.001226703	0.941511899
1291.027	1186.302	1098.257	0.044297	0.270719269	0.847133587
534.2797	452.4112	493.5868	0.08918	0.905394249	0.708596546
101.8526	101.2584	68.11707	-0.29599	0.552891956	0.549038919
686.1652	663.2887	788.0621	0.067461	0.757279972	0.775439768
154.5659	170.0026	195.9676	0.342374	0.967813477	0.484163033
1275.838	1264.336	1393.78	0.02825	0.948234757	0.914120833
292.1563	253.6104	358.4006	-0.18791	0.982305383	0.570700534
723.6898	706.0216	715.7532	0.181017	0.573411006	0.33908301
7.147554	0	0	-1.33412	NA	NA
26236.88	25632.3	27680.68	-0.45516	0.918350023	0.001491463
4553.885	4621.654	4425.514	0.025852	0.02221007	0.881988452
5619.764	5590.576	5172.705	-0.00818	0.86365129	0.964158522
1.786889	0	3.143865	-1.60673	NA	NA
997.9772	926.1888	993.4612	0.086204	0.708719765	0.708596546
301.0907	365.0875	357.3526	0.044917	0.398426428	0.911997158
356.4843	303.7751	470.5317	-0.28224	0.720532728	0.278856737
2.680333	2.786927	3.143865	0.611166	NA	NA
16977.23	17454.53	18441.91	-0.37258	0.814318858	0.00657616
612.9028	722.7431	651.8279	0.241411	0.378784486	0.187946773
665.616	607.5501	694.7941	0.178275	0.910682739	0.358153185
945.264	869.5213	988.2215	0.057888	0.58371648	0.795108707
1364.289	1491.935	1439.89	-0.3591	0.638519612	0.020113967
394.0089	353.9398	293.4274	0.175124	0.935100162	0.579998211
628.9848	694.8739	599.4302	0.132693	0.684623494	0.575540189
857.7065	739.4647	765.0071	0.218613	0.745767703	0.43545502
0	5.573855	4.19182	0.41512	NA	NA
627.1979	765.476	651.8279	0.24158	0.601191315	0.214445591

258.2054	272.1899	257.7969	-0.66553	0.759611543	0.000220628
1397.347	1221.603	1268.025	-0.29215	0.411467295	0.027121839
1379.478	1357.234	1456.657	0.1881	0.572336545	0.250564669
960.4526	1014.442	938.9676	0.107311	0.577190434	0.613454546
427.9598	547.1667	581.615	-0.11124	0.947853941	0.708596546
24380.31	24117.14	25821.61	-0.32265	0.716063981	0.006052647
3412.064	3355.46	3035.925	-0.04401	0.084125911	0.839088125
427.9598	410.6073	404.5106	0.087053	0.584165589	0.819004
510.1567	480.2805	411.8463	-0.14876	0.477428994	0.606332686
844.3048	906.6803	865.6107	0.188543	0.489329504	0.289910731
720.1161	813.7828	653.9239	0.321337	0.63359404	0.071096214
2469.48	2531.459	2701.628	-0.45231	0.820929887	0.008985522
277.8612	255.4683	245.2214	0.005023	0.925846914	0.999397196
1033.715	907.6093	1061.578	-0.17868	0.638519612	0.446369682
1048.904	864.8764	1033.284	0.070576	0.487260716	0.764996976
206.3856	227.5991	300.7631	0.03693	0.649131648	0.925883666
746.026	722.7431	593.1425	0.202609	0.713210135	0.374485512
779.0834	868.5923	876.0903	-0.10668	0.944990108	0.61728503
975.6411	1075.754	950.4951	0.343949	0.518134411	0.022241758
3311.104	3152.944	3193.119	-0.04063	0.187777748	0.786995178
44.67221	61.3124	63.92525	-0.37812	0.187777748	0.434560808
5.360666	5.573855	4.19182	1.199806	NA	NA
1845.856	1818.006	1615.946	0.209085	0.094557838	0.394044776
583.4191	549.9537	538.6488	0.007421	0.287050142	0.974691849
675.4439	594.5445	580.567	0.083058	0.330281656	0.729293105
499.4353	535.09	486.2511	0.429768	0.899527569	0.009159478
1112.338	1048.814	1194.669	0.008778	0.928104954	0.976136838
2597.242	2595.558	2490.989	-0.04146	0.112700802	0.797601985
4.467221	4.644879	5.239774	0.212868	NA	NA
1461.675	1543.958	1550.973	0.24561	0.108117004	0.139151972
0	7.431806	2.09591	-0.2429	NA	NA
3925.794	3847.818	4014.715	-0.10781	0.352157146	0.393779503
3.573777	0	0	-1.03515	NA	NA
197.4512	168.1446	197.0155	0.49098	0.608209302	0.143013287
252.8447	313.9938	277.708	0.009097	0.785492922	0.990142758
20425.92	21359.01	21842.52	-0.28771	0.809173332	0.027121839
140.2707	140.2753	160.3371	0.241712	0.706962639	0.574986893
32.16399	29.72722	56.58956	-0.23701	0.834965572	0.792240634
461.9107	418.0391	414.9901	-0.10859	0.892614393	0.672161194
292.1563	293.5563	331.1537	0.099632	0.458486853	0.751940308
1.786889	2.786927	9.431594	#DIV/0!	NA	NA
165.2872	138.4174	148.8096	-0.33529	0.817609984	0.359873864
1232.06	1143.569	1248.114	0.19736	0.666493976	0.224223422
3211.039	3322.946	3022.302	0.074276	0.00311479	0.715956488
716.5423	749.6834	717.8491	0.102894	0.734144448	0.662690338
5454.477	5150.242	5193.664	0.022418	0.010810987	0.897900971
355.5908	339.0762	440.1411	-0.7514	0.310443697	6.15961E-06
498.5419	433.8317	450.6206	-0.07453	0.40292054	0.786000778
703.1406	630.7745	801.6855	0.137882	0.766760576	0.537032146
0	0	0	#NUM!	NA	NA
1333.019	1412.043	1264.882	0.259042	0.243847797	0.141040456
497.6485	577.8229	431.7574	0.149262	0.337428805	0.600875554
1450.06	1544.887	1567.741	0.028754	0.754774366	0.880577976
796.9523	671.6495	669.6432	0.097295	0.556307283	0.672747744

2020.077	2126.426	2001.594	-0.05453	0.683501136	0.763587743
4664.672	4491.598	4196.011	0.072621	0.00256492	0.640018073
0.893444	0	0	-0.91651	NA	NA
1823.52	2002.872	1876.887	0.032745	0.787054981	0.86393308
3370.965	3485.517	3729.671	-0.32801	0.090068897	0.025777316
646.8536	618.6979	636.1086	0.046721	0.865576104	0.82472084
1626.069	1608.986	1512.199	0.105628	0.494492788	0.495089963
1883.38	1956.423	1785.715	-0.03091	0.157100667	0.868461891
2233.611	2068.829	1817.154	0.054672	0.730407446	0.836841581
158.1396	188.5821	161.3851	-0.23319	0.351446149	0.469810501
411.8778	446.8373	421.2779	0.073652	0.791941475	0.795076358
623.6241	672.5785	528.1693	0.099497	0.478531557	0.739346002
461.0172	416.1811	519.7856	-0.01856	0.075531813	0.961142155
555.7223	557.3855	504.0663	0.373199	0.871801928	0.108399569
138.4839	100.3294	122.6107	0.265314	0.220715145	0.558104763
753.1735	801.7061	760.8153	0.058628	0.363948776	0.764338813
18.76233	16.72156	26.19887	-0.44358	0.918350023	NA
4708.451	4463.729	4308.143	0.054035	0.354108154	0.710256666
19.65577	20.43747	23.05501	-0.60906	0.770381061	NA
1027.461	1044.169	909.6248	0.09252	0.596065856	0.710256666
385.9679	343.721	308.0987	0.096036	0.517968435	0.764996976
1527.79	1518.875	1458.753	0.032067	0.505612976	0.86344732
0	0	0	#NUM!	NA	NA
0	0	0	#NUM!	NA	NA
0	0.928976	0	#DIV/0!	NA	NA
8.934443	12.07668	16.76728	-0.55176	0.603004299	NA
9.827887	25.08235	15.71932	-0.4948	0.426735737	NA
10.72133	15.79259	13.62341	0.248928	0.953403647	NA
452.0828	510.9367	418.134	-0.14301	0.453095561	0.609543168
453.8697	540.6639	563.7997	0.159391	0.601338342	0.505493145
0.893444	1.857952	2.09591	#DIV/0!	NA	NA
2.680333	0.928976	2.09591	0.115025	NA	NA
5010.435	4889.199	5438.886	-0.1266	0.80496489	0.4158179
273.3939	271.2609	254.653	0.000199	0.686653189	0.997242119
0	0	1.047955	#DIV/0!	NA	NA
1.786889	3.715903	1.047955	-0.08627	NA	NA
35.73777	31.58518	17.81523	-0.74821	0.670790001	NA
1303.535	1362.807	1486	-0.34527	0.369892369	0.002315134
3206.571	3442.784	3390.134	-0.3252	0.80037736	0.029788115
26.80333	6.50283	22.00705	0.415567	0.732140966	NA
2285.43	2435.774	2017.313	0.00915	0.184558164	0.983469507
441.3615	478.4225	559.6079	-0.1748	0.570597858	0.571802385
21.44266	31.58518	22.00705	-0.62099	0.979366541	NA
0	0	1.047955	#DIV/0!	NA	NA
20.54922	19.50849	16.76728	-0.42709	0.2768804	NA
0	0.928976	0	-1.50354	NA	NA
916.6738	788.7004	818.4528	0.062187	0.132511083	0.765887785
3.573777	0.928976	1.047955	1.075415	NA	NA
45.56566	47.37776	58.68547	-0.09937	0.004092287	0.87466351
390.4351	433.8317	405.5585	-0.1769	0.26155741	0.509930639
1165.945	1094.333	968.3103	0.118643	0.521198556	0.583691606
2624.046	2775.78	2285.59	0.165031	0.111482683	0.758294944
630.7716	638.2063	660.2116	0.008227	0.927256678	0.985850238
30428.92	30829.92	31571.74	-0.31842	0.736589995	0.029788115

126.8691	156.0679	142.5219	-0.45245	0.022050761	0.142039527
902.3787	962.4189	1081.489	-0.18261	0.410903637	0.324718738
4.467221	1.857952	2.09591	-0.42103	NA	NA
127.7625	137.4884	164.5289	0.083691	0.622518586	0.86814025
9.827887	12.07668	6.287729	-0.63346	0.430461737	NA
75.04932	88.2527	82.78844	0.085153	0.713312904	0.89773245
5.360666	4.644879	4.19182	0.359478	NA	NA
1089.109	931.7627	952.591	-0.06425	0.649302498	0.793207865
12755.7	12830.08	15606.14	-0.51303	0.557551669	0.000230052
1307.109	1317.288	1323.567	0.156195	0.384189305	0.402824474
3591.646	3467.867	3708.712	-0.0012	0.505612976	0.996992829
37.52466	10.21873	19.91114	0.241526	0.764243604	NA
183.1561	166.2867	168.7207	-0.12573	0.26155741	0.752110266
221.5742	212.7354	225.3103	0.13843	0.803497374	0.80286002
216.2135	241.5337	243.1255	0.220543	0.728952588	0.422560599
3130.629	3322.017	3267.523	-0.07015	0.000198278	0.601096936
1681.462	1623.85	1456.657	0.282051	0.503345543	0.134770011
551.2551	626.1297	559.6079	0.115654	0.817609984	0.681942449
0	0	0	#NUM!	NA	NA
2377.455	2332.658	2350.563	0.170068	0.682184622	0.146298537
797.8457	787.7714	857.2271	-0.23817	0.963492814	0.161107365
0	1.857952	0	#DIV/0!	NA	NA
414.5581	396.6727	418.134	0.109512	0.872444605	0.664227919
8.040998	5.573855	2.09591	0.505608	NA	NA
210.8528	189.5111	186.536	0.021792	0.831470267	0.972817917
1308.002	1150.072	1305.752	0.080135	0.967813477	0.765789524
11.61478	18.57952	15.71932	0.440852	0.959739969	NA
491.3943	471.9197	457.9563	0.010025	0.968417488	0.988370025
2406.045	2589.984	2662.853	-0.23238	3.36957E-07	0.038166749
3.573777	3.715903	4.19182	0.102149	NA	NA
90.23787	109.6191	67.06911	0.116235	0.96940125	0.864363898
461.0172	364.1585	321.7222	0.434469	0.162448502	0.176169028
442.2549	409.6783	451.6686	-0.09339	0.380498844	0.731268074
673.657	619.6268	705.2736	0.466242	0.43802737	0.009491168
318.9596	281.4797	361.5444	0.12194	0.80107822	0.746009092
0	1.857952	0	-0.86025	NA	NA
846.0917	1067.393	1057.386	-0.0722	0.551840208	0.738713732
1891.421	2024.238	1721.79	-0.02747	0.760924499	0.91372954
579.8453	669.7915	723.0889	0.432131	0.393551305	0.016825284
293.9432	375.3062	417.086	-0.20413	0.545771146	0.481380095
204.5987	235.0309	237.8858	0.116954	0.962011634	0.724404377
634.3454	675.3654	638.2045	0.169874	0.358629856	0.402824474
403.8368	350.2239	379.3597	0.297895	0.976681492	0.502421025
695.9931	651.212	598.3822	0.060502	0.485974888	0.86758766
6.25411	1.857952	0	1.037849	NA	NA
1094.469	1188.16	975.646	0.17504	0.218419472	0.291835413
1146.289	1203.953	1126.552	-0.05082	0.72034461	0.775439768
2.680333	0.928976	0	-2.11131	NA	NA
159.9265	160.7128	183.3921	0.317383	0.995299355	0.426846696
397.5827	411.5363	355.2567	-0.00535	0.998398411	0.999234423
8.934443	2.786927	8.383639	1.742878	NA	NA
17918.02	17135.89	16877.31	-0.15849	0.395739876	0.107848818
681.698	648.4251	494.6347	0.167357	0.335900008	0.547201181
472.632	528.5872	461.1002	-0.15817	0.968581517	0.41087768

501.2222	625.2007	574.2793	-0.05297	0.881060269	0.866936111
1769.02	1799.426	1644.241	0.17143	0.764235559	0.167165088
995.2969	1083.186	1075.202	0.185897	0.344730624	0.300952186
8.934443	18.57952	11.5275	-0.82759	0.859748112	NA
1.786889	0	2.09591	0.203136	NA	NA
469.9517	526.7293	577.4231	-0.00688	0.811082292	0.987875073
738.8784	799.8481	877.1382	0.031921	0.947037414	0.915422831
723.6898	762.6891	707.3696	0.062249	0.242151376	0.842118027
854.1327	695.8028	861.4189	-0.10231	0.799806234	0.686433182
965.8132	1003.294	765.0071	-0.09243	0.268706533	0.756407952
162.6069	148.6361	107.9394	0.271444	0.775670478	0.538616173
1026.567	1031.163	866.6587	0.301627	0.252640265	0.337040194
0	5.573855	0	0.367504	NA	NA
27262.56	27058.28	29883.48	-0.38024	0.70754392	0.016940762
1420.576	1402.753	1329.855	-0.08874	0.453550494	0.706703794
1.786889	0.928976	1.047955	0.51494	NA	NA
7.147554	2.786927	15.71932	-0.57838	0.798043362	NA
820.1818	785.9135	686.4105	0.112548	0.344738271	0.558104763
3315.572	3343.384	3163.776	0.02716	0.910726255	0.875929853
13.40166	4.644879	27.24683	-0.43102	0.837139541	NA
0.893444	0.928976	0	-0.5314	NA	NA
1277.625	1145.427	1028.044	0.080297	0.2758693	0.81253834
4126.819	4125.581	3821.891	0.069557	0.358629856	0.669238482
6.25411	1.857952	4.19182	0.748442	NA	NA
824.6491	850.9418	809.0212	0.238611	0.323780681	0.176171477
634.3454	671.6495	585.8068	0.148732	0.817609984	0.439411562
1335.699	1172.367	1306.8	-0.12182	0.000591296	0.471424918
16824.45	16543.2	18147.43	-0.34518	0.794039558	0.010218325
5.360666	2.786927	1.047955	#DIV/0!	NA	NA
3.573777	10.21873	10.47955	1.371688	0.869438043	NA
3.573777	0.928976	0	1.77355	NA	NA
2638.341	2440.419	2685.908	0.090329	0.92288341	0.554997306
40158.53	40657.55	38826.73	-0.33459	0.579191948	0.007913336
1440.232	1391.606	1457.705	-0.09216	0.184558164	0.620926433
394.9024	428.2578	387.7433	-0.10028	0.892614393	0.690294956
867.5344	871.3793	899.1453	0.080741	0.755175799	0.659046549
1400.921	1316.359	1175.805	0.415885	0.733488302	0.04967295
2287.217	2212.82	1858.024	-0.02944	0.014189422	0.921848549
8248.277	7990.121	7830.319	-0.00236	0.999122571	0.990142758
201.9184	237.8178	241.0296	-0.07607	0.072773765	0.809142313
8.040998	12.07668	12.57546	-0.01512	0.340833229	NA
1.786889	0	0	0.440193	NA	NA
909.5263	890.8878	972.5021	0.187301	0.0444701	0.193131605
2554.357	2479.436	2787.56	-0.3536	0.835283405	0.003563456
148.3117	133.7725	142.5219	-0.33962	0.410903637	0.353286634
0	1.857952	2.09591	0.586005	NA	NA
1896.782	1847.733	2158.787	-0.16321	0.732140966	0.367518225
1723.454	1717.676	1483.904	0.168444	0.017930836	0.503999921
0.893444	0	0	-0.91651	NA	NA
1707.372	1893.253	1608.611	0.19998	0.036816142	0.35622113
0	0	2.09591	-1.27135	NA	NA
552.1486	501.6469	548.0804	0.111809	0.59039068	0.688084851
0	0.928976	2.09591	-2.17923	NA	NA
389.5417	457.0561	364.6883	-0.00606	0.976830702	0.996992829

0.893444	0	1.047955	0.203136	NA		NA
8040.998	7858.206	9845.536	0.41716		0.425091186	0.055098956
1137.355	1270.839	1073.106	0.074599		0.886746968	0.713405771
623.6241	495.1441	504.0663	0.206985		0.864963799	0.379704424
200.1315	173.7185	207.4951	-0.10015		0.603586196	0.822050288
8.040998	3.715903	9.431594	-0.32275		0.756540804	NA
2926.923	3025.674	3368.127	-0.19188		0.220715145	0.192993665
47.35255	38.08801	45.06206	-0.26059		0.711398577	0.734906514
2165.709	2391.184	2494.133	-0.16213		0.740229545	0.373348081
0	1.857952	0	#DIV/0!	NA		NA
4310.869	4434.93	4643.488	-0.06872		0.051055002	0.600056926
0	1.857952	0	#DIV/0!	NA		NA
1.786889	0	0	#DIV/0!	NA		NA
1.786889	0	0	#DIV/0!	NA		NA
897.9115	872.3082	819.5007	0.147666		0.014083611	0.481884668
436.8942	455.1981	545.9845	0.376826		0.599211134	0.042179555
3657.761	3518.031	3644.787	-0.17159		0.010114821	0.118990473
670.0832	681.8682	658.1157	0.250844		0.37602059	0.13275794
671.8701	579.6809	528.1693	-0.19797		3.20007E-05	0.392697207
551.2551	496.0731	505.1143	-0.0354		0.882057909	0.91372954
12.50822	33.44313	20.9591	2.120566		0.729363614	NA
250.1644	252.6814	228.4542	0.128096		0.804018865	0.666556565
2497.177	2535.175	2571.681	0.255223		0.522361449	0.023809238
516.4108	487.7123	538.6488	0.111833		0.457830725	0.673302041
855.9196	1027.447	1013.372	-0.09628		0.74589429	0.626119342
705.821	575.965	664.4034	0.017855		0.577332911	0.945439187
647.7471	613.124	572.1834	0.047512		0.812875146	0.831655346
1278.519	1266.194	1075.202	-0.21385		0.506893267	0.20379563
1339.273	1335.867	1717.598	-0.25865		0.962011634	0.239315229
698.6734	750.6124	755.5755	-0.16866		0.992476114	0.446951468
2303.299	2455.283	2509.852	-0.02287		0.558928437	0.918341317
1163.264	1129.635	1094.065	0.020675		0.118929144	0.924858067
9.827887	18.57952	20.9591	0.37204		0.922856432	NA
448.509	405.9624	365.7363	0.255351		0.967934287	0.301903647
468.1648	446.8373	487.299	-0.09754		0.753964428	0.655129754
0.893444	0	4.19182	#DIV/0!	NA		NA
0.893444	0	0	#DIV/0!	NA		NA
317.1727	332.5733	251.5092	0.299114		0.005435534	0.266775275
0.893444	0	0	#DIV/0!	NA		NA
5.360666	9.289758	5.239774	0.727381		0.868137167	NA
588.7798	564.8173	555.4161	0.242418		0.864963799	0.208112627
0.893444	0	1.047955	-0.62951	NA		NA
2621.365	2515.666	2324.364	0.172976		0.112535001	0.383494766
21968.9	21684.15	19588.37	-0.2022		0.430087902	0.178134034
34672.78	35183.1	35914.46	-0.44015		0.783200259	0.010243246
747.8128	773.8368	754.5275	0.12388		0.57058689	0.511122414
801.4195	954.0581	1101.401	0.044219		0.973007344	0.881988452
0	0.928976	0	#DIV/0!	NA		NA
359.1646	359.5136	350.0169	0.275462		0.684723286	0.23616229
192.984	181.1503	177.1044	-0.04403		0.098114491	0.923725803
2112.102	2249.979	2302.357	-0.15069		0.769710962	0.348491003
5361.559	4993.245	5595.031	0.012771		0.759261525	0.938440797
4.467221	0	0	#DIV/0!	NA		NA
1199.896	1141.711	1257.546	-0.14898		0.283674866	0.386078627

114.3609	96.61348	118.4189	0.097244		0.642246351	0.844024501
0.893444	3.715903	9.431594	-0.33012		0.907411185	NA
1761.872	1703.742	1713.406	0.062407		0.988049891	0.764162229
259.9923	274.0478	308.0987	-0.00832		0.249012381	0.990023409
547.6813	553.6696	650.78	0.032362		0.618280897	0.915422831
4381.451	4397.771	4213.827	-0.06185		0.170750865	0.601613256
25425.64	25227.27	25754.54	-0.31131		0.525774075	0.018025328
620.0503	641.9223	505.1143	-0.00577		0.598960113	0.988943755
364.5253	419.897	323.8181	0.089571		0.389352673	0.773113821
1056.051	1082.257	1173.709	-0.12834		0.504924333	0.386225205
0	0	0	#NUM!	NA		NA
2264.881	2498.945	2221.664	0.061097		0.027170262	0.764793691
96.49198	88.2527	113.1791	0.244108		0.29215357	0.61955727
870.2147	826.7884	947.3512	-0.14841		0.617342557	0.540291582
137.5904	117.9799	143.5698	0.547286		0.596427641	0.253693985
427.0664	372.5193	343.7292	0.082565		0.863679003	0.783429367
3605.941	3715.903	3363.935	0.037895		0.249012381	0.847597088
31627.03	31527.58	31744.65	-0.40371		0.218682865	0.001662103
1571.568	1713.96	1869.552	-0.09032		0.784914078	0.658487105
26505.81	26610.51	27574.84	-0.30867		0.641408722	0.042689756
1.786889	0.928976	0	-0.14519	NA		NA
3916.86	4043.831	4580.611	0.475403		0.335666342	3.21175E-06
24.12299	39.94596	27.24683	-0.46006		0.777781833	NA
7.147554	20.43747	14.67137	0.073673		0.905394249	NA
25.90988	32.51415	28.29478	-0.27187		0.46017679	NA
0	2.786927	4.19182	0.405711	NA		NA
561.9764	470.9907	439.0931	0.291927		0.9960277	0.359566588
474.4189	506.2918	390.8872	0.193613		0.918350023	0.431825344
866.6409	879.74	1007.085	0.096026		0.484084014	0.625626406
32109.49	31834.14	31472.18	-0.2545		0.599211134	0.024919247
1501.88	1383.245	1619.09	-0.09564		0.884585407	0.680348001
27.69677	12.07668	8.383639	-0.25537		0.256151839	NA
1026.567	973.5666	962.0226	0.167189		0.973922065	0.457073409
8048.146	7760.664	6924.886	0.018152		0.00756732	0.93327699
2.680333	4.644879	2.09591	0.437968	NA		NA
20.54922	23.22439	20.9591	-0.01799		0.757209202	NA
651.3209	568.5332	693.7461	0.36904		0.695456738	0.034869884
0	0	4.19182	-0.68638	NA		NA
0	0.928976	1.047955	0.229302	NA		NA
747.8128	841.652	954.6869	0.000183		0.598960113	0.997469085
40708	39018.84	40444.77	-0.36777		0.598960113	0.006992399
687.9521	614.982	761.8632	-0.21563		0.07582841	0.27042216
1308.896	1354.447	1458.753	0.132403		0.770051929	0.566742383
1.786889	0	0	0.083489	NA		NA
0.893444	0.928976	1.047955	#DIV/0!	NA		NA
190.3036	259.1842	256.7489	-0.23514		0.767024472	0.533112779
2141.586	2261.127	2049.8	-0.08702		0.005386667	0.531391342
362.7384	427.3289	405.5585	0.112859		0.580245461	0.659046549
697.78	747.8255	594.1904	0.024859		0.817609984	0.923910484
399.3696	493.2861	431.7574	-0.33524		0.053184762	0.073272362
211.7463	180.2213	197.0155	0.042742		0.145286779	0.923725803
677.2307	614.053	624.5811	0.156104		0.477428994	0.470568982
883.6164	971.7086	824.7405	-0.04286		0.171797519	0.892893032
437.7877	404.1045	428.6136	-0.05247		0.794232621	0.871061917

482.4599	431.0448	408.7024	0.519506	0.570003668	0.051038786
0	7.431806	4.19182	#DIV/0!	NA	NA
1.786889	0	5.239774	-0.2353	NA	NA
5224.862	5474.454	5492.332	0.035728	0.413714744	0.8085461
1624.282	1837.514	1658.913	-0.15775	0.158417136	0.329120807
959.5591	837.9361	850.9394	-0.23748	0.947527175	0.174598931
7628.227	7363.062	6811.707	0.077649	0.812875146	0.764793691
162.6069	139.3464	209.591	-0.26549	0.413905026	0.426257832
76.83621	68.74421	77.54866	0.660049	0.433973714	0.217405432
1466.142	1434.339	1436.746	0.027247	0.92288341	0.904467867
370.7794	406.8914	299.7151	0.216451	0.802471571	0.422116695
13.40166	33.44313	23.05501	0.708127	0.183923135	NA
416.345	501.6469	476.8195	0.106096	0.740595411	0.715172443
483.3533	603.8342	599.4302	0.009648	0.04381621	0.984451848
928.2886	863.0185	724.1368	0.017065	0.649714874	0.94581612
1666.274	1643.358	1709.214	0.123186	0.350762724	0.365253762
0	7.431806	2.09591	1.854873	NA	NA
2482.882	2406.047	2086.478	0.149117	0.053089664	0.477876695
247.4841	299.1302	307.0508	-0.34562	0.540189768	0.122362777
976.5346	1176.083	1153.798	-0.19579	0.835449534	0.329120807
1.786889	0	0	#DIV/0!	NA	NA
14.29511	22.29542	23.05501	-0.47285	0.928104954	NA
619.1569	616.8399	703.1777	0.478571	0.148074498	0.002531629
7790.834	7994.765	8274.652	-0.16176	0.761936154	0.118990473
1.786889	0	0	#DIV/0!	NA	NA
1251.715	1247.614	1295.272	-0.16988	0.995598118	0.323922459
357.3777	428.2578	401.3667	-0.09726	0.455739606	0.690294956
403.8368	495.1441	539.6968	0.129982	0.28287487	0.644875718
1232.953	1226.248	1284.793	-0.02636	0.819879878	0.907473183
495.8616	570.3911	451.6686	0.229196	0.331098637	0.318452284
3085.063	2999.663	3018.11	0.005038	0.011540931	0.979410591
357.3777	353.9398	368.8801	0.04147	0.642006987	0.87909951
290.3694	319.5677	279.804	0.05452	0.901380807	0.865246562
410.9844	389.2408	503.0183	-0.04409	0.246611129	0.875929853
5.360666	6.50283	5.239774	1.113981	NA	NA
1033.715	1029.305	928.488	0.168602	0.400955126	0.457073409
61.64765	55.73855	54.49365	-0.05973	0.795811864	0.923910484
3013.587	2898.404	2749.834	0.059178	0.866564571	0.820867679
287.6891	314.9228	228.4542	0.154352	0.574512226	0.620471139
2255.053	2306.647	2474.222	0.00701	0.785492922	0.97417638
2314.021	2298.286	2385.145	-0.16318	0.79239225	0.247256105
604.8618	695.8028	615.1495	0.081174	0.10822328	0.714693632
877.3623	1053.459	896.0014	-0.02268	0.73037883	0.923725803
23340.34	23445.49	23397.69	-0.38146	0.853046438	0.003889306
1568.888	1639.642	1673.584	-0.119	0.371004931	0.450196624
2214.848	2235.116	2519.284	-0.13297	0.895968293	0.400713698
580.7388	491.4282	579.5191	-0.07631	0.824582133	0.798185205
17723.25	17538.13	16282.08	-0.14277	0.580611002	0.422560599
2960.874	2924.416	2900.739	-0.09988	0.968196977	0.583691606
439.5746	496.0731	443.2849	0.049654	0.684723286	0.84551374
222.4676	250.8235	225.3103	0.010874	0.685427571	0.975406172
2346.185	2563.044	2374.666	-0.31379	0.990064993	0.029788115
955.9854	810.9958	925.3442	0.307665	0.73176709	0.088358317
386.8614	400.3886	407.6545	0.296177	0.124733431	0.115345014

335.935	342.7921	276.6601	0.086243	0.577981995	0.80030609
559.2961	575.036	569.0395	-0.18901	0.469683278	0.326192074
2230.93	2344.735	2168.219	0.124612	0.023980578	0.340424273
15525.38	15185.97	14506.84	0.086568	0.517968435	0.49413501
15.18855	14.86361	6.287729	-0.39199	0.748298735	NA
1631.429	1517.946	1588.7	-0.06781	0.058460789	0.728787361
155.4593	163.4997	166.6248	-0.4815	7.59971E-05	0.033645271
174.2216	157.9259	248.3653	-0.55748	0.283463824	0.060691739
33823.12	32577.32	34546.88	-0.07363	0.716459644	0.546900844
427.0664	477.4935	427.5656	0.021086	0.168996242	0.961800697
108.1068	154.21	136.2341	-0.43748	0.209524948	0.313958096
734.4112	758.0442	701.0818	0.19377	0.600467264	0.291487478
1928.053	2105.059	2241.576	-0.01442	0.009038328	0.953867552
368.9925	408.7493	387.7433	0.086246	0.484625827	0.792240634
68.79521	73.38909	83.83639	-0.38273	0.523530764	0.313505068
1213.297	1166.794	1277.457	-0.14783	0.469347309	0.333065318
3734.597	3977.874	4095.408	-0.20775	0.152933305	0.103471246
958.6657	876.0241	883.426	-0.28338	0.62498123	0.073806318
572.6978	584.3258	602.5741	0.077484	0.679066538	0.716565058
13234.59	12666.58	11814.64	0.116407	0.127584376	0.503879072
1086.428	1053.459	1139.127	-0.18299	0.643621852	0.320138645
0	0	2.09591	#DIV/0!	NA	NA
437.7877	326.0705	362.5924	0.174067	0.67324111	0.549888611
955.0919	935.4786	1023.852	-0.05725	0.647886117	0.752110266
6.25411	1.857952	4.19182	0.053859	NA	NA
950.6247	684.6551	919.0564	-0.28052	0.442989531	0.144365513
442.2549	479.3515	504.0663	0.065311	0.484921587	0.797601985
0.893444	0	2.09591	#DIV/0!	NA	NA
282.3284	353.9398	313.3385	0.075017	0.384584076	0.798876088
726.3702	582.4678	757.6714	-0.46753	0.897766774	0.025807779
1234.74	1221.603	1343.478	-0.15548	0.216910334	0.266398936
410.9844	444.0504	414.9901	0.285	0.917013507	0.245528705
1426.83	1330.293	1196.764	0.140238	0.991590779	0.503205042
250.1644	222.9542	215.8787	0.111466	0.272065999	0.718168497
748.7063	679.0813	601.5261	0.232754	0.503914082	0.365235491
978.3215	915.9701	996.6051	0.176612	0.139873727	0.380365797
592.3535	596.4024	561.7038	0.192334	0.708011334	0.361564535
1129.314	1171.438	1062.626	0.019323	0.298839192	0.941596912
13.40166	22.29542	26.19887	-0.06629	0.709065901	NA
653.1078	616.8399	654.9718	0.127458	0.989306104	0.564967632
8372.466	8150.833	7465.631	0.12603	0.18789975	0.302912952
5.360666	1.857952	0	1.454465	NA	NA
220.6807	245.2496	291.3315	0.081704	0.994145678	0.843896683
427.9598	436.6186	380.4076	-0.05649	0.154093864	0.844024501
1028.354	1026.518	1118.168	-0.28507	0.418813799	0.034479858
7033.193	7477.326	7574.618	0.164618	0.045192099	0.229483247
1593.011	1602.483	1360.245	0.087679	0.116586762	0.683321289
5.360666	2.786927	1.047955	0.61433	NA	NA
3739.064	3697.324	3835.515	0.199262	0.092299784	0.287877942
2.680333	0.928976	0	#DIV/0!	NA	NA
13.40166	14.86361	11.5275	-0.59023	0.831439424	NA
25.01644	26.01132	23.05501	-0.06316	0.90469595	NA
541.4272	628.9166	588.9507	-0.07882	0.344730624	0.752110266
829.1163	837.0072	970.4062	-0.32789	0.892240442	0.022120531

1096.256	934.5496	992.4133	0.33222	0.673923772	0.225879379
1177.56	1183.515	1127.599	-0.02251	0.362841481	0.912679298
243.9103	275.9058	289.2356	-0.37867	0.869770641	0.088001869
458.3369	431.9737	481.0113	-0.40741	0.746342492	0.009491168
37.52466	64.09933	52.39774	-0.09774	0.881060269	0.901090975
394.9024	443.1214	440.1411	0.253365	0.716351188	0.292802751
643.2799	596.4024	535.505	-0.00956	0.327866713	0.97680656
248.3775	219.2383	294.4753	-0.18883	0.210589435	0.487331989
421.7057	470.0617	428.6136	0.141359	0.655062777	0.56820839
2163.922	2154.295	2216.425	0.092606	0.310114043	0.578074656
25.01644	27.86927	24.10296	0.427986	0.960773888	NA
9770.706	9679.927	10659.8	-0.3003	0.92790798	0.088369497
80.40998	58.52547	93.26799	0.18076	0.987280421	0.764338813
283.2218	239.6757	322.7701	-0.16201	0.542904501	0.589672784
23.22955	39.01698	20.9591	0.221905	0.995299355	NA
6054.872	6086.649	5708.21	-0.03946	0.985859213	0.817066359
510.1567	579.6809	402.4147	0.107597	0.973340554	0.709912994
7808.703	7877.714	10015.3	-0.08587	1.1083E-07	0.864107722
1257.076	1298.708	1491.24	0.076677	0.732140966	0.716565058
1995.954	2056.752	1899.942	-0.16929	0.012847448	0.192883976
862.1737	834.2202	807.9732	0.071166	0.917013507	0.759033711
609.329	627.9876	621.4373	-0.28406	0.122493092	0.068898592
16.082	2.786927	8.383639	0.738506	0.509078826	NA
482.4599	506.2918	519.7856	0.304772	0.760762822	0.071096214
512.837	614.982	488.347	0.324011	0.369977802	0.10872211
1041.756	1050.672	1017.564	-0.1629	0.545399422	0.27042216
0.893444	1.857952	5.239774	0.125807	NA	NA
3.573777	0	0	0.440193	NA	NA
1333.912	1320.075	1140.175	0.191895	0.668522148	0.372138887
1049.797	1073.896	1192.573	-0.4767	0.236875869	0.000164663
87.55754	86.39475	76.50071	-0.37424	0.962556121	0.365253762
37.52466	39.01698	42.96615	-0.49973	0.604929869	0.324099572
30.3771	52.95162	69.16502	-0.28533	0.935506037	0.690294956
2568.652	2671.734	2455.358	-0.1285	0.960956671	0.501904702
8.934443	8.360782	15.71932	1.209282	0.439271214	NA
668.2963	717.1693	762.9112	0.053316	0.962011634	0.834432692
358.2711	440.3345	423.3738	0.264784	0.728952588	0.22284642
482.4599	600.1183	609.9097	-0.41313	0.154093864	0.034322372
46913.86	46495.24	47030.12	-0.24012	0.826883467	0.063510063
6566.815	6895.787	7121.901	-0.18921	0.956608426	0.241575828
247.4841	228.528	255.701	0.033874	0.973223327	0.93327699
813.0343	706.9506	935.8237	-0.0908	0.434274199	0.770449453
1.786889	6.50283	2.09591	-0.2779	NA	NA
499.4353	558.3144	538.6488	-0.02247	0.715549426	0.923725803
1056.945	1256.904	1046.907	0.071357	0.76103064	0.708596546
201.9184	198.8008	215.8787	-0.71319	0.183923135	0.000227187
369.8859	353.0108	398.2229	0.134389	0.993005178	0.673302041
937.223	959.632	998.701	-0.06195	0.97836084	0.764338813
696.8865	713.4534	606.7659	-3.5E-05	0.846759683	0.996720771
1794.93	2056.752	1930.333	0.042895	0.8968711	0.818409186
683.4849	647.4961	608.8618	0.100002	0.956452433	0.678425078
5555.436	5196.69	4973.594	0.334521	0.149001182	0.033609232
849.6655	794.2743	716.8011	0.109661	0.62292707	0.708596546
581.6322	463.5589	515.5938	-0.30707	0.209856535	0.167165088

1623.388	1531.881	1532.11	-0.04063	0.034700814	0.835596295
11.61478	5.573855	22.00705	0.533528	0.648891486	NA
701.3537	707.8795	599.4302	0.111944	0.967813477	0.607944538
849.6655	859.3026	857.2271	0.262741	0.72031406	0.131463633
2573.119	2470.147	2332.748	-0.06656	0.000185215	0.752312486
1428.617	1643.358	1692.447	-0.21152	0.888097311	0.253027668
13.40166	19.50849	12.57546	0.222072	0.710373411	NA
107.2133	64.09933	70.21298	0.339833	0.386644751	0.620471139
1382.158	1516.088	1585.556	-0.00675	0.761936154	0.978405477
3602.367	3735.412	3589.246	-0.15011	0.051957782	0.353563667
0	0	0	#NUM!	NA	NA
598.6077	557.3855	558.56	-0.01994	0.187619163	0.946818235
2254.16	2320.581	2001.594	0.105321	8.89999E-05	0.602270106
443.1484	376.2352	375.1679	0.477909	0.167094261	0.022550727
0	0	0	#NUM!	NA	NA
368.9925	254.5394	289.2356	-0.21035	0.785971011	0.539844242
493.1812	538.8059	519.7856	-0.32202	0.465950462	0.091608354
789.8047	724.6011	656.0198	0.060904	0.997568936	0.866936111
0	0.928976	0	-0.50354	NA	NA
15827.37	15486.95	15962.45	-0.27451	0.651961753	0.044918766
1.786889	5.573855	6.287729	-0.89292	0.833722816	NA
279.6481	271.2609	247.3174	0.072643	0.956452433	0.882926075
247.4841	247.1076	224.2623	-0.15387	0.727251544	0.676897688
1507.24	1582.975	1491.24	0.088559	0.621884159	0.570700534
508.3698	399.4596	412.8942	0.158946	0.553033696	0.572103667
22.33611	21.36644	30.39069	0.266549	0.828690815	NA
1535.831	1503.083	1471.329	0.384357	0.470002734	0.053095679
509.2632	583.3968	471.5797	0.208668	0.848681983	0.487331989
28.59022	26.01132	31.43865	-1.29905	0.014948404	0.023695036
1541.191	1523.52	1390.636	0.041392	0.107676441	0.8721296
547.6813	536.948	444.3329	-0.03938	0.938838346	0.893756708
1646.618	1430.623	1470.281	0.228655	0.71685777	0.130431848
4364.475	4377.334	4569.083	-0.15505	0.935100162	0.300201673
746.9194	726.459	769.1989	-0.16703	0.477428994	0.386225298
857.7065	859.3026	681.1707	0.173754	0.464552004	0.505302546
1034.608	1129.635	1080.441	0.148522	0.120606425	0.333065318
7.147554	11.14771	22.00705	-0.24268	0.720648336	NA
356.4843	367.8744	304.9549	0.063357	0.511726855	0.855100406
506.5829	532.3031	394.031	0.241376	0.586160756	0.408058591
1280.306	1211.384	1150.654	0.235537	0.430461737	0.24175269
88.45098	75.24704	96.41185	0.085989	0.962660515	0.893701659
4687.009	4642.092	4269.368	0.320717	0.028304309	0.036381838
3437.08	3483.659	3776.829	-0.23172	0.455280748	0.057894023
671.8701	725.5301	630.8688	0.196061	0.900383237	0.302414442
2804.522	2781.353	2721.539	-0.04214	0.1433784	0.818645356
0	0	0	#NUM!	NA	NA
2.680333	0.928976	6.287729	0.634289	NA	NA
659.3619	650.283	536.5529	0.242052	0.179668524	0.380365797
416.345	458.914	468.4358	-0.23233	0.35165001	0.359873864
1.786889	0	1.047955	#DIV/0!	NA	NA
581.6322	519.2975	627.725	-0.07201	0.73478662	0.794250417
6263.044	6431.299	7516.98	-0.52776	0.759261525	2.16222E-05
18951.74	19647.84	19956.21	-0.29427	0.739965249	0.026742345
89.34443	148.6361	133.0903	-0.15476	0.390042791	0.775439768

4096.442	3772.571	3611.253	0.048496	0.827268091	0.794250417
1315.15	1058.103	1209.34	0.064237	0.61217167	0.806564543
990.8297	919.686	809.0212	0.222112	0.784425728	0.283438968
2182.684	2095.769	2179.746	-0.04828	0.965163416	0.797601985
955.9854	893.6747	901.2412	0.083214	0.444125545	0.716565058
425.2795	457.0561	495.6827	0.027257	0.501942014	0.935768815
1467.929	1396.251	1357.102	0.116272	0.081926061	0.520805117
5757.355	5547.843	5647.429	-0.02876	0.735300868	0.87134989
3327.186	3049.827	3206.742	0.137578	0.239965637	0.398613087
18531.82	18190.27	19744.52	-0.2771	0.709468629	0.016996857
1.786889	1.857952	0	-1.58112	NA	NA
6301.462	6212.99	5737.553	0.036155	0.001564335	0.891918558
459.2303	587.1127	485.2031	0.235108	0.945870306	0.245528705
1793.143	1799.426	1542.59	0.237336	0.325979266	0.225879379
638.8126	681.8682	806.9253	0.405766	0.755224646	0.02451735
709.3947	627.0586	484.1552	0.113244	0.649714874	0.698234721
1078.387	1051.601	974.5981	-0.00603	0.736581016	0.982607912
80.40998	104.0453	129.9464	-0.06623	0.010353677	0.915422831
583.4191	631.7035	613.0536	-0.08369	0.099551501	0.729827413
387.7548	457.9851	326.9619	0.284198	0.86365129	0.27042216
76.83621	100.3294	74.4048	0.05428	0.718668252	0.923602174
1250.822	1130.564	1093.017	0.154171	0.406333601	0.472093197
1883.38	1866.312	1690.351	0.072467	0.1975101	0.778199039
455.6566	431.0448	427.5656	0.354819	0.799313452	0.052658569
5779.691	6108.016	5553.113	-0.00473	0.475984345	0.982131958
1090.895	1098.049	813.213	0.126422	0.700014781	0.669238482
1375.011	1406.469	1474.473	-0.04217	0.614621507	0.80030609
0.893444	0	0	-2.74916	NA	NA
7171.677	7178.196	6050.892	-0.07582	0.028285791	0.777508737
209.066	222.9542	258.8449	0.179977	0.848301697	0.603217016
417.2385	481.2094	425.4697	-0.10183	0.173085065	0.708596546
255.5251	277.7638	237.8858	-0.05982	0.812875146	0.890721169
226.9348	203.4457	199.1114	0.265704	0.755224646	0.441429894
724.5833	742.2516	575.3272	0.035726	0.310073788	0.912679298
788.0178	873.2372	833.1241	0.008143	0.785492922	0.988370025
811.2474	829.5754	723.0889	0.290806	0.353648271	0.24818395
595.0339	599.1894	582.6629	0.155223	0.165510999	0.531462254
1026.567	969.8507	912.7687	0.137997	0.899176451	0.461673135
19.65577	27.86927	24.10296	0.632393	0.578995017	NA
1368.757	1405.54	1462.945	0.080751	0.745767703	0.620471139
24338.32	23880.25	24748.5	-0.36674	0.824080056	0.010962375
0	0.928976	1.047955	-0.41399	NA	NA
10.72133	13.00566	1.047955	0.795062	0.936832996	NA
854.1327	1053.459	881.3301	-0.18945	0.813397158	0.33275563
1207.043	1192.805	1026.996	0.15139	0.467158435	0.556864585
3.573777	7.431806	8.383639	0.425292	0.730407446	NA
23525.28	23321.94	24193.09	-0.242	0.93399229	0.119229885
4205.442	4104.215	3853.33	0.030773	0.140612719	0.917805295
350.2301	375.3062	368.8801	0.187895	0.875368742	0.526905314
2.680333	0	1.047955	-0.33102	NA	NA
12.50822	29.72722	10.47955	-0.79758	0.993312366	NA
0	1.857952	0	#DIV/0!	NA	NA
574.4847	549.9537	455.8604	0.106854	0.726598225	0.696921855
4744.189	4974.665	5214.624	-0.23713	0.951394798	0.110990341

378.8204	434.7607	326.9619	0.097029	0.811082292	0.726171034
402.9434	380.8801	485.2031	0.072618	0.029440414	0.832900164
500.3288	508.1497	608.8618	-0.06228	0.310443697	0.819964665
1363.396	1237.396	1412.643	-0.22908	0.274927458	0.130685332
424.386	370.6613	407.6545	0.009222	0.821250302	0.990142758
1103.404	1095.262	1219.819	-0.07051	0.408721528	0.757350793
1048.904	1064.606	896.0014	0.037553	0.234941511	0.904467867
226.0414	238.7468	189.6798	0.092618	0.665579954	0.811724117
3597.007	3806.943	3874.289	0.191655	0.697514819	0.07575529
3083.276	3104.637	3183.687	-0.06287	0.668641279	0.75448001
858.5999	770.1209	831.0282	0.236001	0.301324739	0.294466305
787.1244	824.9305	940.0155	-0.25489	0.36939921	0.132850703
4661.099	4276.075	4036.722	-0.06018	0.000208059	0.782083823
1683.249	1833.798	1913.566	-0.19379	0.894528942	0.296722063
225.148	234.1019	206.4471	0.03623	0.216003389	0.922546597
6056.659	6000.254	6346.415	0.077407	0.635969974	0.563375937
2315.808	2266.701	2020.457	0.17839	0.652183366	0.372138887
556.6158	522.0844	460.0522	0.031874	0.573830327	0.923725803
268.0333	264.7581	254.653	0.010045	0.29215357	0.975227629
858.5999	800.7771	876.0903	0.073434	0.98843748	0.711465473
754.067	740.3937	580.567	0.036954	0.67997963	0.901090975
12.50822	15.79259	14.67137	-0.25914	0.812875146	NA
303.771	315.8518	297.6192	-0.07089	0.995722579	0.808330041
563.7633	620.5558	648.6841	-0.04847	0.792868376	0.832431036
0	0	0	#NUM!	NA	NA
3.573777	4.644879	7.335684	0.086646	NA	NA
0.893444	0.928976	1.047955	-0.23272	NA	NA
0	3.715903	1.047955	#DIV/0!	NA	NA
140.2707	153.281	176.0564	0.223599	0.478308661	0.55088846
1399.134	1269.91	1429.41	-0.07415	0.088547132	0.896567419
14.29511	30.6562	20.9591	0.0208	0.655624618	NA
837.1573	908.5383	718.8971	0.03514	0.962903592	0.896421455
2375.668	2571.405	2946.849	-0.29856	0.090626117	0.025807779
913.1	954.9871	883.426	0.412592	0.613734914	0.023955106
941.6902	927.1178	950.4951	0.146353	0.985102475	0.386078627
3.573777	4.644879	0	0.452297	NA	NA
306.4514	330.7154	291.3315	-0.10544	0.65458858	0.691804481
1304.429	1194.663	1275.361	0.081869	0.962011634	0.626829627
226.9348	230.386	223.2144	0.221545	0.614311366	0.535992604
53.60666	51.09367	42.96615	-0.30988	0.76103064	0.56624122
10.72133	7.431806	10.47955	-0.56316	0.727251544	NA
2558.824	2810.152	2757.169	-0.01921	0.344738271	0.923725803
22.33611	12.07668	16.76728	-0.7459	0.141487454	NA
707.6079	733.8909	698.9859	0.232277	0.662554511	0.177201026
1.786889	0	2.09591	0.55984	NA	NA
1.786889	0	0	-0.55981	NA	NA
1853.897	1734.398	1822.394	0.233258	0.916233061	0.053045424
512.837	576.8939	610.9577	0.061471	0.622518586	0.822050288
2494.496	2283.422	2198.609	0.350065	0.263755993	0.12612237
2629.406	2778.567	2695.34	-0.20332	0.37602059	0.192142371
6.25411	12.07668	6.287729	0.213458	0.614621507	NA
694.2062	615.9109	727.2807	0.156597	0.702415456	0.400713698
1908.397	1674.943	1577.172	0.105347	0.769710962	0.647261886
2588.308	2984.799	2768.697	-0.0995	0.80496489	0.52313639

0	0	0	#NUM!	NA	NA
746.9194	839.7941	797.4937	0.123751	0.914164397	0.486948273
565.5502	605.6922	545.9845	0.157288	0.607353168	0.451963117
1.786889	2.786927	1.047955	-0.68054	NA	NA
191.1971	179.2923	193.8717	0.256206	0.006307785	0.365253762
300.1973	263.8291	337.4415	-0.41516	0.792868376	0.068859716
638.8126	697.6608	706.3216	-0.20766	0.228848778	0.322281806
0.893444	1.857952	2.09591	-1.06174	NA	NA
3218.186	3091.631	3019.158	0.235304	0.276178144	0.098090079
11.61478	0	0	0.366322	NA	NA
78.62309	72.46011	123.6587	-0.16945	0.056337167	0.763319325
2488.242	2485.01	2287.686	0.143128	0.901380807	0.381582724
8268.827	8124.822	8462.236	0.023511	0.270977162	0.874744904
872.0016	758.0442	695.842	0.19783	0.030146682	0.400713698
281.4349	260.1132	243.1255	0.239174	0.885575053	0.333065318
167.9675	126.3407	113.1791	0.128752	0.779325825	0.775439768
566.4437	600.1183	571.1354	0.027629	0.111234411	0.923725803
50408.13	50694.21	50810.09	-0.3423	0.652135039	0.001201529
805.8867	923.4019	867.7067	-0.07879	0.641408722	0.751151284
89.34443	94.75553	124.7066	-0.06506	0.999034414	0.911521311
351.1236	296.3433	378.3117	0.115246	0.187777748	0.706876462
3473.711	3542.185	3488.642	-0.05236	0.867574132	0.741368284
4844.255	5031.333	5034.375	0.150054	0.32170196	0.225879379
836.2638	719.9562	920.1044	0.008088	0.817609984	0.980529916
31.27055	18.57952	26.19887	0.304136	0.991136341	NA
2833.112	2759.058	3245.516	-0.13724	0.798439173	0.481380095
2577.587	2595.558	2635.607	0.008872	0.951001819	0.956290296
482.4599	498.86	414.9901	0.144258	0.853477462	0.583053599
4590.517	4547.336	4300.807	0.026949	0.000548797	0.855100406
250.1644	261.9712	276.6601	-0.03387	0.184109237	0.911916486
1.786889	1.857952	1.047955	-1.44226	NA	NA
3671.162	3416.773	4012.619	-0.04161	0.621884159	0.855100406
389.5417	371.5903	303.9069	0.080802	0.618790469	0.778766112
1002.444	1061.819	1023.852	0.08277	0.960773888	0.625457131
604.8618	560.1724	625.6291	0.051182	0.770381061	0.844024501
0	0	0	#NUM!	NA	NA
2156.774	2116.207	2090.67	0.145978	0.86365129	0.240259541
5.360666	0	0	0.192875	NA	NA
479.7796	530.4452	418.134	-0.08653	0.944990108	0.755018053
51.81977	46.44879	55.54161	0.220725	0.017930836	0.719971458
427.9598	462.6299	496.7306	-0.05305	0.324651337	0.840718213
3507.662	3563.551	3532.656	0.06181	0.553033696	0.675143077
0	0.928976	5.239774	0.395441	NA	NA
0	0	4.19182	#DIV/0!	NA	NA
7072.505	6877.208	7157.532	0.180424	0.585762809	0.292538732
1362.502	1405.54	1227.155	0.095635	0.2795135	0.708596546
259.0988	287.0535	302.859	-0.05217	0.288584519	0.908612163
1727.921	1582.975	1346.622	0.261042	0.263755993	0.349930843
15.18855	24.15337	15.71932	-0.29601	0.564427849	NA
1.786889	0	0	-1.50147	NA	NA
237.6562	194.1559	202.2553	0.039212	0.897759686	0.913620457
369.8859	355.7977	326.9619	0.048263	0.463180957	0.896421455
778.1899	730.1749	835.2201	-0.16859	0.159614653	0.431825344
0	0	1.047955	-0.68638	NA	NA

255.5251	247.1076	264.0846	-0.03453	0.107676441	0.914120833
2058.496	1833.798	1931.381	-0.03321	0.136068446	0.863216533
5907.453	6029.982	6864.105	-0.31883	0.913769516	0.039022257
1986.127	2176.59	2066.567	-0.12426	0.962433805	0.422116695
913.9935	954.0581	800.6375	0.114383	0.651943512	0.659338327
414.5581	414.3232	460.0522	0.166254	0.800508699	0.466447289
1.786889	0	2.09591	#DIV/0!	NA	NA
570.9109	562.0303	556.464	0.042585	0.147626297	0.840777068
248.3775	287.9825	280.8519	0.081467	0.837529432	0.795655341
807.6736	893.6747	763.9591	0.186055	0.2795135	0.333065318
16.082	26.01132	22.00705	0.311948	0.212716004	NA
2336.357	2397.686	2146.212	0.118699	0.232941496	0.589270302
28362.39	28609.67	27392.49	-0.29496	0.730204074	0.030614917
106.3199	107.7612	98.50776	0.131175	0.004400836	0.764338813
9768.026	9518.286	8299.803	-0.03098	0.208221128	0.923725803
1995.061	2226.755	1751.133	-0.00148	0.139314666	0.996720771
1162.371	1281.058	1242.875	0.233914	0.84819607	0.317595626
640.5995	747.8255	706.3216	-0.29118	0.185572017	0.104841824
103.6395	122.6248	111.0832	0.510639	0.333641544	0.328024792
1167.732	1183.515	1263.834	0.03624	0.390042791	0.872630949
96.49198	92.89758	83.83639	-1.02199	0.000205284	0.006124694
6240.708	6185.121	6128.44	0.031486	0.732718278	0.835181175
1129.314	968.9217	1022.804	0.177991	0.832298202	0.39921965
773.7227	950.3422	837.316	0.175422	0.189947746	0.467083166
4192.04	4201.757	3621.732	-0.0549	0.901380807	0.831655346
634.3454	622.4138	714.7052	-0.35086	0.095897541	0.047027405
1.786889	0	0	0.083489	NA	NA
6.25411	4.644879	8.383639	0.011559	0.903271991	NA
3241.416	3181.742	3149.104	0.123553	0.985859213	0.333065318
3363.818	3271.853	3078.891	0.067942	0.005621011	0.748344041
658.4684	701.3767	698.9859	0.243999	0.892614393	0.287132004
2058.496	2029.812	2133.636	-0.14496	0.331331472	0.225630132
58.07388	54.80957	88.02821	0.140556	0.752287766	0.875604624
232.2955	277.7638	318.5783	0.222149	0.214140629	0.477547118
195.6643	175.5764	188.6319	-0.01932	0.478079371	0.965791283
30.3771	37.15903	56.58956	0.206218	0.078454607	0.814120559
427.9598	536.019	498.8265	0.092661	0.29215357	0.724404377
1060.518	1143.569	1309.944	-0.05281	0.896802518	0.831655346
752.2801	814.7117	871.8985	-0.07845	0.698024229	0.765988241
730.8374	844.439	718.8971	0.038141	0.470002734	0.882565871
25.01644	22.29542	49.25388	-0.37645	0.114505424	NA
963.1329	1072.967	1163.23	-0.11591	0.494209809	0.625382385
1764.552	1885.821	1753.229	-0.0493	0.378784486	0.835293664
2.680333	2.786927	3.143865	0.519596	NA	NA
830.0097	653.9989	756.6234	0.115857	0.367802825	0.644740483
561.083	593.6155	580.567	-0.08718	0.899761215	0.682436785
1464.355	1399.966	1173.709	0.048874	0.161272124	0.875604624
419.0254	427.3289	471.5797	-0.14877	0.242503197	0.477876695
1061.412	1090.618	1173.709	-0.39028	0.57058689	0.036067988
0	1.857952	1.047955	-1.25894	NA	NA
73678.77	72584.59	72839.15	-0.47742	0.83213076	0.00164999
637.0258	614.982	629.8209	0.055762	0.80050738	0.821432697
1205.256	1176.083	1309.944	-0.03965	0.520356385	0.869696905
855.9196	744.1096	705.2736	-0.03998	0.755573717	0.891918558

1135.568	1119.416	1129.695	-0.07978	0.816234041	0.693887308
488.714	444.9794	551.2243	-0.19849	0.974771919	0.422793542
32273.89	32041.3	33430.81	-0.20002	0.960823113	0.060851524
777.2965	741.3227	682.2186	0.289907	0.248671635	0.184454481
904.1656	1038.595	892.8576	-0.00865	0.92288341	0.979395135
317.1727	295.4143	293.4274	0.004525	0.879847555	0.983593071
2642.808	2724.686	2742.498	0.01617	0.220796999	0.922101817
529.8124	596.4024	616.1975	0.069591	0.901380807	0.795076358
2962.661	3017.313	2936.37	-0.16302	0.135397929	0.181725119
195.6643	220.1673	195.9676	-0.00011	0.187777748	0.996992829
921.141	925.2599	1058.434	-0.18944	0.923364203	0.411765294
0.893444	0.928976	0	-1.36368	NA	NA
1569.782	1849.591	2081.238	-0.29211	0.023293079	0.071238709
18109.22	17444.31	16846.92	-0.22411	0.764235559	0.177201026
76.83621	65.95728	71.26093	-0.30306	0.907589146	0.55088846
16149.9	16152.1	20022.23	0.393912	0.880647749	0.040358902
705.821	723.6721	717.8491	0.085871	0.769573539	0.708596546
6054.872	6357.91	6119.009	-0.06862	0.587123529	0.691450677
694.2062	827.7174	798.5416	0.09032	0.948234757	0.734906514
444.0418	438.4766	432.8054	-0.13441	0.71270119	0.49670864
2327.422	2261.127	2061.327	0.142029	0.214235831	0.449939346
2482.882	2419.053	2388.289	-0.29324	0.568895197	0.034322372
1289.24	1267.123	1214.58	0.119874	0.655321049	0.521104798
0.893444	1.857952	2.09591	#DIV/0!	NA	NA
451.1894	484.9253	664.4034	-0.25896	0.681419018	0.278856737
649.534	754.3283	909.6248	-0.47508	0.775017701	0.035238922
485.1402	454.2691	386.6954	0.304284	0.38441957	0.127757998
4.467221	15.79259	20.9591	0.064604	0.944990108	NA
437.7877	531.3741	587.9027	-0.27403	0.549290634	0.321405678
10.72133	16.72156	15.71932	-1.07511	0.503345543	NA
1215.084	1222.532	987.1735	0.391673	0.268223584	0.086195548
1756.511	1689.807	1586.604	0.73237	0.311074517	4.02321E-09
312.7055	353.9398	252.5571	-0.34057	0.504924333	0.162970478
1513.495	1439.912	1662.056	-0.14144	0.608976999	0.447154499
1371.437	1360.021	1224.011	-0.00266	0.953363514	0.990142758
3.573777	1.857952	2.09591	-0.9604	NA	NA
0	0	2.09591	#DIV/0!	NA	NA
4665.566	4694.115	5076.294	-0.00529	0.466743878	0.977528539
7.147554	11.14771	9.431594	-0.07939	0.812875146	NA
444.0418	498.86	483.1072	0.027987	0.901096965	0.91817406
0	0.928976	1.047955	#DIV/0!	NA	NA
798.7392	665.1466	785.9662	0.185063	0.554725555	0.545985077
511.0501	582.4678	532.3611	0.119314	0.8892252	0.566141749
810.3539	779.4107	805.8773	0.325194	0.230144662	0.013575411
4284.065	4253.78	3722.336	0.081839	0.020689285	0.731333029
6363.11	6422.009	5830.821	0.075952	0.066189238	0.741368284
8474.319	8744.449	8501.01	-0.24065	0.134073214	0.00280023
427.0664	373.4483	347.921	0.028165	0.730407446	0.939380143
1320.511	1307.069	1279.553	0.173498	0.581539609	0.359873864
1573.355	1535.597	1549.925	0.041442	0.071137852	0.825886448
1680.569	1589.478	1586.604	0.064997	0.649891554	0.709370809
1792.249	1944.346	2053.992	0.11298	0.783760899	0.578896646
779.9768	862.0895	897.0494	0.00561	0.952783825	0.992528568
159.0331	161.6418	146.7137	-0.29285	0.09408214	0.291835413

5710.896	5843.258	4874.038	-0.06961	0.000616166	0.768568541
1533.15	1416.688	1482.856	0.191496	0.393551305	0.126868093
933.6492	864.8764	1137.031	0.1225	0.212716004	0.611282744
1242.781	1249.472	1229.251	0.08909	0.922856432	0.626527921
544.1076	494.2151	560.6559	0.029059	0.826883467	0.923725803
552.1486	549.0247	587.9027	0.133802	0.614621507	0.658878607
911.3131	916.8991	795.3978	0.040694	0.907095813	0.865063191
18.76233	13.93464	17.81523	0.464032	0.962433805	NA
71.47554	75.24704	81.74048	0.194917	0.478184092	0.69424407
0	0	0	#NUM!	NA	NA
0	0.928976	0	-1.86025	NA	NA
804.9933	928.9758	691.6502	0.032352	0.122553551	0.915543523
61.64765	52.95162	52.39774	0.139091	0.901380807	0.840777068
0	0	0	#NUM!	NA	NA
751.3866	774.7658	804.8294	-0.10651	0.820906044	0.625457131
3621.13	3663.88	3131.289	0.111242	0.405505718	0.499756226
188.5167	163.4997	144.6178	0.238005	0.302724041	0.518234816
2490.029	2700.533	2117.917	-0.07534	0.05699652	0.740018919
1.786889	0.928976	2.09591	-0.32004	NA	NA
15291.3	14914.71	15745.52	-0.28939	0.919799198	0.033123987
6657.947	7025.844	7070.552	0.036564	0.415980367	0.835596295
4.467221	5.573855	8.383639	0.330971	0.825200136	NA
1037.289	1214.171	1021.756	-0.14751	0.859611064	0.498498627
1620.708	1774.344	1492.288	0.169417	0.285839486	0.352934781
1333.912	1251.33	1416.835	-0.24008	0.853331381	0.204865574
2811.669	2774.851	3014.966	-0.24016	0.868785389	0.05825118
650.4274	712.5244	775.4866	0.008862	0.861862766	0.975406172
1366.97	1360.021	1332.999	0.088453	0.110845457	0.664215073
4612.853	4354.109	4275.656	0.056697	0.817609984	0.819964665
0	0	0	#NUM!	NA	NA
478.8861	497.002	452.7165	-0.29457	0.044964859	0.144968034
0.893444	0	0	-3.44782	NA	NA
663.8291	704.1636	769.1989	-0.26543	0.92790798	0.093149564
334.1482	354.8687	357.3526	0.083731	0.559366656	0.802920813
0	2.786927	0	-1.86025	NA	NA
467.2713	456.1271	453.7645	0.208837	0.207579254	0.408058591
2092.446	2225.826	2077.047	-0.03237	0.000217366	0.87466351
26729.17	27426.15	27145.18	-0.36489	0.826883467	0.012748693
6486.405	6776.878	7091.511	-0.24639	0.91441696	0.151699323
3119.014	2960.646	3273.811	-0.15235	0.959739969	0.270288837
3624.703	3474.369	3295.818	0.217593	0.283964296	0.060344767
209.9594	202.5167	265.1326	-0.0786	0.818107632	0.845206982
1663.593	1662.867	1458.753	0.079429	0.285144055	0.678741853
301.0907	282.4086	266.1805	-0.13175	0.903964934	0.628282957
1429.511	1380.458	1256.498	0.1363	0.526561206	0.391432089
24679.61	24524.96	24449.84	-0.28153	0.730407446	0.049157122
373.4597	328.8574	307.0508	-0.10736	0.772432927	0.748961137
193922.1	186558.8	218181.1	-0.43123	0.911468001	0.004352618
226.9348	248.9655	242.0776	0.229522	0.728952588	0.536664638
1081.068	1148.214	1111.88	-0.31747	0.660321043	0.004593992
1754.725	1635.926	1581.364	0.041426	0.640224868	0.812861677
1034.608	1019.086	1195.717	-0.05443	0.550503835	0.8325893
599.5011	570.3911	532.3611	0.086103	0.573411006	0.718291318
522.6649	496.0731	512.4499	0.127726	0.416166733	0.55841527

0	0.928976	0	#DIV/0!	NA	NA
0.893444	0	0	#DIV/0!	NA	NA
0	0	0	#NUM!	NA	NA
0	0	0	#NUM!	NA	NA
3052.899	3101.85	2770.793	-0.02638	0.005035345	0.877477421
3254.817	3251.415	3105.09	-0.02732	0.024420121	0.891411326
1619.814	1701.884	1557.261	-0.00617	0.43802737	0.982230296
92.02476	84.53679	56.58956	0.332557	0.798409569	0.58759663
3528.211	3340.597	3079.939	0.216976	0.006208168	0.283557823
14354.08	14320.16	15547.46	-0.31839	0.68563527	0.024919247
1125.74	1233.68	1147.511	0.027219	0.708696414	0.890884082
32.16399	34.3721	40.87024	-0.1616	0.92444453	NA
72.36898	99.40041	63.92525	0.102024	0.242725862	0.883471187
998.8707	931.7627	910.6728	0.02512	0.081224331	0.91372954
1593.905	1515.159	1466.089	0.027509	0.286526774	0.91372954
92.02476	79.89192	105.8434	-0.04112	0.779325825	0.937994709
192.0905	220.1673	220.0705	-0.10879	0.88874568	0.713405771
2045.094	2139.431	1751.133	-0.03393	0.071245169	0.905168779
908.6328	795.2033	702.1298	0.216966	0.935100162	0.439570865
708.5013	784.9845	746.1439	-0.16267	0.638519612	0.443275405
27.69677	26.01132	14.67137	-0.49809	0.798030968	NA
0	3.715903	4.19182	-1.07882	NA	NA
82.19687	95.6845	95.3639	0.49413	0.881060269	0.360674621
674.5504	777.5527	733.5684	0.245966	0.324610791	0.12530366
928.2886	863.9475	869.8026	0.323714	0.059452673	0.069345614
19397.57	19201.93	20800.86	-0.36355	0.814880408	0.004688416
234.0824	308.42	254.653	0.137879	0.976681492	0.6924902
325.2137	339.0762	265.1326	-0.17712	0.780429274	0.624554774
19.65577	14.86361	33.53456	0.524355	0.439271214	NA
4767.419	4577.993	4890.805	-0.01948	0.904254847	0.897989514
1254.396	1252.259	1195.717	0.16056	0.55061661	0.316157156
297.5169	277.7638	258.8449	0.509238	0.894061214	0.063275758
874.6819	954.9871	912.7687	-0.08305	0.632040487	0.660222897
409.1975	430.1158	465.292	0.006317	0.269120346	0.992866088
318.9596	326.9995	320.6742	0.051435	0.368085564	0.882926075
81.30343	72.46011	90.12412	0.297205	0.842806932	0.531682752
19.65577	14.86361	29.34274	-0.21619	0.690588843	NA
1246.355	1242.97	1213.532	0.087344	0.377099016	0.775439768
23927.33	23735.33	26307.86	-0.0129	0.380779612	0.941511899
0	1.857952	2.09591	0.229302	NA	NA
426.1729	443.1214	440.1411	0.156567	0.677154401	0.542805648
70.5821	63.17035	78.59662	-0.0367	0.324651337	0.952535643
781.7637	739.4647	784.9182	0.156068	0.886112674	0.434560808
8.934443	23.22439	13.62341	-0.69948	0.938838346	NA
729.944	720.8852	806.9253	0.005803	0.44898062	0.990444813
3242.309	3299.722	2901.787	0.180398	0.083350036	0.354829766
3.573777	2.786927	2.09591	-0.38799	NA	NA
2619.579	2616.925	2364.186	-0.03077	5.38978E-05	0.883471187
131.3363	110.5481	142.5219	0.383679	0.185931343	0.311435996
5059.575	5008.108	4787.058	-0.00154	0.000886762	0.992729462
36983.23	36378.69	36587.25	-0.28243	0.848681983	0.060870852
1851.217	1642.429	1800.387	-0.04519	0.036638284	0.82132239
79.51654	78.96294	58.68547	0.553639	0.637091947	0.287569887
6675.815	6516.765	6689.096	-0.29326	0.817609984	0.005150683

407.4106	307.491	292.3794	0.390792	0.759261525	0.123535461
6115.626	5938.942	6437.587	0.260322	0.837998472	0.092351218
6518.569	6060.638	6027.837	-0.19373	0.396423722	0.368062441
1377.691	1419.475	1274.313	0.091662	0.221789876	0.642738974
1223.125	1330.293	1258.594	0.129584	0.475821599	0.388495658
905.9525	816.5697	858.2751	0.355713	0.848681983	0.043133767
2091.553	1891.395	1970.155	-0.08841	0.334053294	0.572969021
235.8693	329.7864	294.4753	-0.4477	0.868914561	0.060644712
1168.625	1211.384	1165.326	0.20793	0.948896953	0.149449076
645.9602	721.8142	710.5134	0.130087	0.4878075	0.54849911
1755.618	1956.423	1876.887	-0.1969	0.962011634	0.190192716
466.3779	509.0787	493.5868	0.275998	0.938838346	0.142189757
296.6235	303.7751	290.2835	0.001093	0.185391819	0.990023409
695.9931	570.3911	754.5275	-0.22623	0.981762093	0.225238011
0.893444	0	0	-0.55981	NA	NA
3216.399	3098.134	3378.607	0.147987	0.846759683	0.351487828
509.2632	529.5162	565.8956	-0.01916	0.131849064	0.936794868
7473.661	6889.284	7641.687	0.017236	0.147626297	0.931798896
293.9432	284.2666	360.4965	-0.02017	0.285144055	0.952666818
1267.797	1211.384	1128.647	0.387768	0.443341372	0.036086837
3812.327	3557.048	3733.863	0.10253	0.759069408	0.588371763
8.934443	11.14771	10.47955	0.745468	0.813397158	NA
335.935	435.6896	436.9972	-0.16009	0.817609984	0.558104763
0	0	2.09591	#DIV/0!	NA	NA
2604.39	2764.632	2616.743	0.059395	0.107676441	0.753881265
924.7148	1014.442	1017.564	-0.09683	0.769573539	0.596309029
3268.219	3052.614	3184.735	0.078644	0.759261525	0.529445162
1522.429	1601.554	1679.872	-0.0367	0.505612976	0.841070222
448.509	412.4652	402.4147	-0.03723	0.234225111	0.889954225
7722.932	7815.473	7856.518	-0.09742	0.887028326	0.468374832
40.20499	48.30674	66.02116	-0.46099	0.831870041	0.357441003
2512.365	2400.473	2346.371	0.091039	0.106548765	0.639253997
33.95088	20.43747	45.06206	-0.52404	0.926756894	0.565559013
62.5411	34.3721	36.67842	0.258141	0.897759686	0.723437511
1023.887	1097.12	1213.532	-0.02862	0.596065856	0.91372954
16.97544	13.93464	20.9591	0.043766	0.652050398	NA
384.181	396.6727	455.8604	0.110506	0.774889037	0.778199039
761.2145	710.6665	641.3484	0.077653	0.812111557	0.751339004
10.72133	1.857952	14.67137	-0.02722	0.658567464	NA
104.533	119.8379	163.481	0.001948	0.523530764	0.999675887
929.182	936.4076	834.1721	0.351441	0.310443697	0.065668139
681.698	599.1894	575.3272	0.348002	0.324651337	0.070299477
125.9756	157.9259	151.9535	0.168798	0.828016939	0.760432859
886.2967	776.6237	914.8646	-0.1009	0.999825784	0.640018073
97.38542	98.47143	113.1791	-0.13358	0.427509427	0.818869867
558.4027	584.3258	749.2877	0.058045	0.475984345	0.857210686
270.7136	215.5224	215.8787	0.127383	0.973922065	0.680886616
1268.691	1082.257	1082.537	0.120088	0.325451376	0.665181522
725.4767	711.5954	767.103	0.145112	0.285144055	0.572718815
362.7384	401.3175	465.292	-0.16232	0.780980494	0.542274871
1121.273	1045.098	1030.14	0.09112	0.486116151	0.748270161
605.7552	555.5275	496.7306	0.143762	0.872688807	0.665181522
769.2555	837.9361	848.8435	-0.19543	0.503345543	0.345371472
791.5916	758.0442	725.1848	0.08751	0.844410461	0.660222897

1069.453	932.6917	999.749	-0.04432	0.660114316	0.852939392
4253.688	4143.232	4026.243	0.039937	0.517968435	0.764996976
2718.751	2663.374	3031.734	-0.16143	0.157741898	0.331112797
1596.585	1590.407	1612.803	-0.07637	0.395079365	0.612861816
22560.36	21568.03	22332.97	0.079282	0.770051929	0.598969752
23.22955	42.73289	29.34274	-0.65644	0.233378357	0.235273873
0	0	0	#NUM!	NA	NA
2228.25	2038.173	1790.955	0.218061	0.243850473	0.316669252
1.786889	0	0	#DIV/0!	NA	NA
0	1.857952	0	#DIV/0!	NA	NA
726.3702	667.0046	678.0268	-0.12402	0.525774075	0.489096476
754.9604	782.1976	714.7052	0.118372	0.484625827	0.669238482
0	0	1.047955	#DIV/0!	NA	NA
0	0	1.047955	#DIV/0!	NA	NA
0	0.928976	0	#DIV/0!	NA	NA
0.893444	0	0	#DIV/0!	NA	NA
0.893444	0	0	#DIV/0!	NA	NA
1557.273	1555.105	1747.989	-0.1234	0.577179112	0.58501253
345.7629	382.738	460.0522	-0.15581	0.517968435	0.686015779
456.55	445.9084	455.8604	-0.09603	0.681216827	0.718291318
50.92632	61.3124	73.35684	-0.24178	0.825797061	0.713405771
11.61478	25.08235	12.57546	0.280365	0.872654096	NA
560.1895	504.4338	485.2031	-0.05172	0.80107822	0.854326626
519.0911	593.6155	720.993	-0.07746	0.871801928	0.822780332
1593.011	1738.114	1739.605	-0.01973	0.899176451	0.929586888
0	0	0	#DIV/0!	NA	NA
0	0	0	#DIV/0!	NA	NA
0	0	0	#DIV/0!	NA	NA
0	0	0	#DIV/0!	NA	NA
0	0	0	#DIV/0!	NA	NA
0	0	0	#DIV/0!	NA	NA
0	0	0	#DIV/0!	NA	NA
0	0	0	#DIV/0!	NA	NA
0	0	0	#DIV/0!	NA	NA
0	0	0	#DIV/0!	NA	NA
0	0	0	#DIV/0!	NA	NA
0	0	0	#DIV/0!	NA	NA
0	0	0	#DIV/0!	NA	NA
0	0	0	#DIV/0!	NA	NA
0	0	0	#DIV/0!	NA	NA
0	0	0	#DIV/0!	NA	NA
0	0	0	#DIV/0!	NA	NA
0	0	0	#DIV/0!	NA	NA
0	0	0	#DIV/0!	NA	NA
0	0	0	#DIV/0!	NA	NA
0	0	0	#DIV/0!	NA	NA
0	0	0	#DIV/0!	NA	NA
0	0	0	#DIV/0!	NA	NA
0	0	0	#DIV/0!	NA	NA
0	0	0	#DIV/0!	NA	NA
0	0	0	#DIV/0!	NA	NA
0	0	0	#DIV/0!	NA	NA
0	0	0	#DIV/0!	NA	NA
0	0	0	#DIV/0!	NA	NA

0	0	0	#DIV/0!	NA	NA
0	0	0	#DIV/0!	NA	NA
0	0	0	#DIV/0!	NA	NA
0	0	0	#DIV/0!	NA	NA
0	0	0	#DIV/0!	NA	NA
0	0	0	#DIV/0!	NA	NA
0	0	0	#DIV/0!	NA	NA
0	0	0	#DIV/0!	NA	NA
0	0	0	#DIV/0!	NA	NA
0	0	0	#DIV/0!	NA	NA
0	0	0	#DIV/0!	NA	NA
0	0	0	#DIV/0!	NA	NA
0	0	0	#DIV/0!	NA	NA
0	0	0	#DIV/0!	NA	NA
0	0	0	#DIV/0!	NA	NA
0	0	0	#DIV/0!	NA	NA
0	0	0	#DIV/0!	NA	NA
0	0	0	#DIV/0!	NA	NA
0	0	0	#DIV/0!	NA	NA
0	0	0	#DIV/0!	NA	NA
0	0	0	#DIV/0!	NA	NA
0	0	0	#DIV/0!	NA	NA
0	0	0	#DIV/0!	NA	NA
0	0	0	#DIV/0!	NA	NA
0	0	0	#DIV/0!	NA	NA
0	0	0	#DIV/0!	NA	NA
0	0	0	#DIV/0!	NA	NA
0	0	0	#DIV/0!	NA	NA
0	0	0	#DIV/0!	NA	NA
0	0	0	#DIV/0!	NA	NA
0	0	0	#DIV/0!	NA	NA
0	0	0	#DIV/0!	NA	NA
0	0	0	#DIV/0!	NA	NA
0	0	0	#DIV/0!	NA	NA
0	0	0	#DIV/0!	NA	NA
0	0	0	#DIV/0!	NA	NA
0	0	0	#DIV/0!	NA	NA
0	0	0	#DIV/0!	NA	NA
0	0	0	#DIV/0!	NA	NA
0	0	0	#DIV/0!	NA	NA
0	0	0	#DIV/0!	NA	NA
0	0	0	#DIV/0!	NA	NA
0	0	0	#DIV/0!	NA	NA
0	0	0	#DIV/0!	NA	NA
0	0	0	#DIV/0!	NA	NA
0	0	0	#DIV/0!	NA	NA
0	0	0	#DIV/0!	NA	NA
0	0	0	#DIV/0!	NA	NA
0	0	0	#DIV/0!	NA	NA
0	0	0	#DIV/0!	NA	NA
0	0	0	#DIV/0!	NA	NA
0	0	0	#DIV/0!	NA	NA
0	0	0	#DIV/0!	NA	NA
0	0	0	#DIV/0!	NA	NA

0	0	0	#DIV/0!	NA	NA
0	0	0	#DIV/0!	NA	NA
0	0	0	#DIV/0!	NA	NA
0	0	0	#DIV/0!	NA	NA
0	0	0	#DIV/0!	NA	NA
0	0	0	#DIV/0!	NA	NA
0	0	0	#DIV/0!	NA	NA
0	0	0	#DIV/0!	NA	NA
0	0	0	#DIV/0!	NA	NA
0	0	0	#DIV/0!	NA	NA
0	0	0	#DIV/0!	NA	NA
0	0	0	#DIV/0!	NA	NA
0	0	0	#DIV/0!	NA	NA
0	0	0	#DIV/0!	NA	NA
0	0	0	#DIV/0!	NA	NA
0	0	0	#DIV/0!	NA	NA
0	0	0	#DIV/0!	NA	NA
0	0	0	#DIV/0!	NA	NA
0	0	0	#DIV/0!	NA	NA
0	0	0	#DIV/0!	NA	NA
0	0	0	#DIV/0!	NA	NA
0	0	0	#DIV/0!	NA	NA
0	0	0	#DIV/0!	NA	NA
0	0	0	#DIV/0!	NA	NA
0	0	0	#DIV/0!	NA	NA
0	0	0	#DIV/0!	NA	NA
0	0	0	#DIV/0!	NA	NA
0	0	0	#DIV/0!	NA	NA
0	0	0	#DIV/0!	NA	NA
0	0	0	#DIV/0!	NA	NA
0	0	0	#DIV/0!	NA	NA
0	0	0	#DIV/0!	NA	NA
0	0	0	#DIV/0!	NA	NA
0	0	0	#DIV/0!	NA	NA
0	0	0	#DIV/0!	NA	NA
0	0	0	#DIV/0!	NA	NA
0	0	0	#DIV/0!	NA	NA
0	0	0	#DIV/0!	NA	NA
0	0	0	#DIV/0!	NA	NA
0	0	0	#DIV/0!	NA	NA
0	0	0	#DIV/0!	NA	NA
0	0	0	#DIV/0!	NA	NA
0	0	0	#DIV/0!	NA	NA
0	0	0	#DIV/0!	NA	NA
0	0	0	#DIV/0!	NA	NA
0	0	0	#DIV/0!	NA	NA
0	0	0	#DIV/0!	NA	NA
0	0	0	#DIV/0!	NA	NA
0	0	0	#DIV/0!	NA	NA
0	0	0	#DIV/0!	NA	NA
0	0	0	#DIV/0!	NA	NA
0	0	0	#DIV/0!	NA	NA
0	0	0	#DIV/0!	NA	NA
0	0	0	#DIV/0!	NA	NA

0	0	0	#DIV/0!	NA	NA
0	0	0	#DIV/0!	NA	NA
0	0	0	#DIV/0!	NA	NA
0	0	0	#DIV/0!	NA	NA
0	0	0	#DIV/0!	NA	NA
0	0	0	#DIV/0!	NA	NA
0	0	0	#DIV/0!	NA	NA
0	0	0	#DIV/0!	NA	NA
0	0	0	#DIV/0!	NA	NA
0	0	0	#DIV/0!	NA	NA
0	0	0	#DIV/0!	NA	NA
0	0	0	#DIV/0!	NA	NA
0	0	0	#DIV/0!	NA	NA
0	0	0	#DIV/0!	NA	NA
0	0	0	#DIV/0!	NA	NA
0	0	0	#DIV/0!	NA	NA
0	0	0	#DIV/0!	NA	NA
0	0	0	#DIV/0!	NA	NA
0	0	0	#DIV/0!	NA	NA
0	0	0	#DIV/0!	NA	NA
0	0	0	#DIV/0!	NA	NA
0	0	0	#DIV/0!	NA	NA
0	0	0	#DIV/0!	NA	NA
0	0	0	#DIV/0!	NA	NA
0	0	0	#DIV/0!	NA	NA
0	0	0	#DIV/0!	NA	NA
0	0	0	#DIV/0!	NA	NA
0	0	0	#DIV/0!	NA	NA
0	0	0	#DIV/0!	NA	NA
0	0	0	#DIV/0!	NA	NA
0	0	0	#DIV/0!	NA	NA
0	0	0	#DIV/0!	NA	NA
0	0	0	#DIV/0!	NA	NA
0	0	0	#DIV/0!	NA	NA
0	0	0	#DIV/0!	NA	NA
0	0	0	#DIV/0!	NA	NA
0	0	0	#DIV/0!	NA	NA
0	0	0	#DIV/0!	NA	NA
0	0	0	#DIV/0!	NA	NA
0	0	0	#DIV/0!	NA	NA
0	0	0	#DIV/0!	NA	NA
0	0	0	#DIV/0!	NA	NA
0	0	0	#DIV/0!	NA	NA
0	0	0	#DIV/0!	NA	NA
0	0	0	#DIV/0!	NA	NA
0	0	0	#DIV/0!	NA	NA
0	0	0	#DIV/0!	NA	NA
0	0	0	#DIV/0!	NA	NA
0	0	0	#DIV/0!	NA	NA
0	0	0	#DIV/0!	NA	NA
0	0	0	#DIV/0!	NA	NA
0	0	0	#DIV/0!	NA	NA
0	0	0	#DIV/0!	NA	NA
0	0	0	#DIV/0!	NA	NA

0	0	0	#DIV/0!	NA	NA
0	0	0	#DIV/0!	NA	NA
0	0	0	#DIV/0!	NA	NA
0	0	0	#DIV/0!	NA	NA
0	0	0	#DIV/0!	NA	NA
0	0	0	#DIV/0!	NA	NA
0	0	0	#DIV/0!	NA	NA
0	0	0	#DIV/0!	NA	NA
0	0	0	#DIV/0!	NA	NA
0	0	0	#DIV/0!	NA	NA
0	0	0	#DIV/0!	NA	NA
0	0	0	#DIV/0!	NA	NA
0	0	0	#DIV/0!	NA	NA
0	0	0	#DIV/0!	NA	NA
0	0	0	#DIV/0!	NA	NA
0	0	0	#DIV/0!	NA	NA
0	0	0	#DIV/0!	NA	NA
0	0	0	#DIV/0!	NA	NA
0	0	0	#DIV/0!	NA	NA
0	0	0	#DIV/0!	NA	NA
0	0	0	#DIV/0!	NA	NA
0	0	0	#DIV/0!	NA	NA
0	0	0	#DIV/0!	NA	NA
0	0	0	#DIV/0!	NA	NA
0	0	0	#DIV/0!	NA	NA
0	0	0	#DIV/0!	NA	NA
0	0	0	#DIV/0!	NA	NA
0	0	0	#DIV/0!	NA	NA
0	0	0	#DIV/0!	NA	NA
0	0	0	#DIV/0!	NA	NA
0	0	0	#DIV/0!	NA	NA
0	0	0	#DIV/0!	NA	NA
0	0	0	#DIV/0!	NA	NA
0	0	0	#DIV/0!	NA	NA
0	0	0	#DIV/0!	NA	NA
0	0	0	#DIV/0!	NA	NA
0	0	0	#DIV/0!	NA	NA
0	0	0	#DIV/0!	NA	NA
0	0	0	#DIV/0!	NA	NA
0	0	0	#DIV/0!	NA	NA
0	0	0	#DIV/0!	NA	NA
0	0	0	#DIV/0!	NA	NA
0	0	0	#DIV/0!	NA	NA
0	0	0	#DIV/0!	NA	NA
0	0	0	#DIV/0!	NA	NA
0	0	0	#DIV/0!	NA	NA
0	0	0	#DIV/0!	NA	NA
0	0	0	#DIV/0!	NA	NA
0	0	0	#DIV/0!	NA	NA
0	0	0	#DIV/0!	NA	NA
0	0	0	#DIV/0!	NA	NA
0	0	0	#DIV/0!	NA	NA
0	0	0	#DIV/0!	NA	NA
0	0	0	#DIV/0!	NA	NA

0	0	0	#DIV/0!	NA		NA
0	0	0	#DIV/0!	NA		NA
0	0	0	#DIV/0!	NA		NA
0	0	0	#DIV/0!	NA		NA
0	0	0	#DIV/0!	NA		NA
0	0	0	#DIV/0!	NA		NA
0	0	0	#DIV/0!	NA		NA
0	0	0	#DIV/0!	NA		NA
0	0	0	#DIV/0!	NA		NA
0	0	0	#DIV/0!	NA		NA
0	0	0	#DIV/0!	NA		NA
0	0	0	#DIV/0!	NA		NA
0	0	0	#DIV/0!	NA		NA
0	0	0	#DIV/0!	NA		NA
0	0	0	#DIV/0!	NA		NA
0	0	0	#DIV/0!	NA		NA

EdgeR FDR_G	EdgeR FDR_E	EdgeR FDR_GE	logFC_G	logFC_E	logFC_GE	Gene Symb
0	2.11162E-22	4.7478E-05	4.710321	0.76902	0.776607	FIT3
0	7.76736E-23	0.000588078	4.343715	0.99971	0.884277	FIT2
0	0.569517503	0.000805631	8.513084	0.12302	-5.3745	EHD3
0	0.341849588	0.000827407	-4.26702	-0.10851	-0.64448	FLO11
0	0.019464433	0.00883753	-5.14165	-0.28888	-0.9461	YAL065C
0	0.765135358	0.009006324	-3.29564	0.043076	-0.55074	FDH1
0	0.027370663	0.142407082	-4.10047	-0.26103	0.4455	STE6
0	0.00012681	0.168520344	6.720575	0.379575	0.403278	MF(ALPHA)
0	0.936093775	0.197874004	-9.79029	-0.02303	0.885199	MFA2
0	0.407136042	0.235407237	-2.97628	0.086681	0.277229	EFT2
0	0.003277486	0.241562819	-4.94446	-0.35856	-0.4436	FLO9
0	0.806307841	0.26370665	-11.7039	0.058727	-3.56345	FLO10
0	4.85742E-10	0.293969078	7.736513	0.93065	1.561877	ARN2
0	0.231604832	0.430239299	5.356079	-0.17614	0.390751	COS5
0	0.628397894	0.449495496	-8.17047	-0.09236	0.842899	AGA2
0	0.746926462	0.459099096	-8.34015	-0.04966	0.384737	STE2
0	1.03382E-05	0.509608089	11.81727	0.583668	-2.13892	MAL11
0	1.24275E-06	0.600468279	10.58723	0.576502	1.422408	ENB1
0	0.14551117	0.69956533	-5.66481	-0.19482	0.25552	BAR1
0	0.025326351	0.807027851	2.921372	0.214437	-0.11111	YHB1
0	0.230929934	0.87592683	6.832219	0.15659	-0.14515	STE3
0	0.152200112	0.889079564	5.49858	0.18567	0.104638	SAG1
0	0.492734305	0.907281575	-9.58468	-0.13405	0.689822	YIL014C-A
0	0.031070961	0.953514172	4.593255	0.213242	-0.05808	AFB1
0	0.000179933	0.972259946	-5.01889	0.494491	0.085768	PCK1
0	0.025415061	1	11.7986	-0.41279	-0.41345	YOL159C-A
0	0.289051446	1	3.817915	0.149975	0.02189	COS4
0	0.391096287	1	3.410634	-0.08135	-0.01697	KRS1
0	0.505793014	1	-4.78527	0.112582	0.043552	YEL073C
0	0.805302069	1	-6.19957	0.056616	0.048756	SNZ3
0	0.871135401	1	-4.29733	-0.03739	-0.02208	YCL049C
1.0437E-306	0.295659	0.29930175	-3.64625	0.139076	0.331968	THI7
1.9773E-304	0.657453737	1	10.54456	0.176744	-0.09462	MF(ALPHA)
2.2786E-304	0.986843508	0.795907717	-7.71244	0.019957	0.511901	MFA1
4.4381E-296	0.015182854	0.014857079	4.233924	0.362358	0.847374	IMA1
1.3774E-286	0.411661642	0.802035501	-4.54558	-0.1408	-0.23789	YAL064W-B
3.905E-283	0.836107259	1	-12.1829	0.070568	-0.07069	HMRA1
7.1133E-278	0.883064096	0.32087275	-2.63279	-0.02526	0.261878	ARO10
2.4326E-262	0.109990533	1	-11.4933	0.322338	-0.32282	YJL218W
3.2259E-258	0.768264114	1	11.13426	-0.09143	-0.09163	MAL13
3.6492E-252	0.398327474	1	2.742094	-0.10826	-0.02247	HBN1
3.71E-252	0.451996377	1	-11.4954	0.180015	-0.1803	PGU1
4.1417E-247	0.446288659	0.933469902	-6.29698	0.16556	0.256237	SNZ2
3.4684E-245	0.006455564	0.420929331	-5.75273	0.517992	-0.55346	AQY1
1.4829E-234	0.515760255	5.95983E-05	-2.48685	-0.07781	-0.73848	UBX6
1.211E-223	0.905759808	0.267503245	1.686172	-0.01626	0.211219	EFT2
7.1769E-221	2.96228E-17	0.533418515	-1.89839	0.515573	0.161894	CTR3
5.5138E-217	0.188573634	0.417425061	-6.36675	0.286456	-1.05374	COS6
4.9557E-214	0.017246708	0.711541955	-1.88434	0.184122	0.119368	IRC7
5.014E-204	0.622972832	0.626633158	1.970577	0.055888	0.154591	ADH2

8.4133E-200	0.98408428	0.042142302	2.553187	-0.01206	-0.5179	FDC1
1.2677E-199	0.496224832	0.979797115	-8.37765	0.181371	0.370788	VTH2
3.5155E-199	0.47612507	0.952440864	-6.31778	0.183913	0.235966	YEL077C
4.3634E-197	0.795533688	0.777291545	-8.09189	-0.085	-1.10381	RDT1
1.3942E-196	0.256874534	0.404345893	3.560844	0.202772	-0.39261	MAL33
5.3956E-192	0.005227243	0.643429399	4.053539	0.47642	-0.36618	ENA5
1.8139E-191	8.00081E-06	4.96566E-13	2.131557	0.377994	-1.1814	FMP48
3.0324E-190	1.90606E-11	0.204980083	1.633876	0.406106	0.244118	HSP104
4.2932E-188	1.50591E-09	0.744376434	-5.73596	1.07095	-0.44099	PHO89
2.2501E-187	0.089587852	0.575130318	-2.91873	0.221794	0.256881	SMX3
4.3563E-187	0.084977816	0.446640364	1.764429	0.145842	0.190481	SFA1
1.4905E-186	4.75894E-09	0.024892656	-3.3792	0.693619	-0.74812	FMP45
1.1155E-183	0.005293865	2.07221E-05	1.727811	0.215622	0.63596	FET3
1.1154E-179	0.20626184	0.085923561	-2.53808	0.156562	-0.4617	CYB2
4.6238E-178	8.1361E-06	0.798608504	2.944726	0.535197	0.174056	TIS11
1.1197E-175	0.137864205	0.243549962	2.468677	0.186686	0.365776	DOG2
1.7382E-174	0.099278382	0.50705979	-2.67016	0.206687	-0.26799	GND2
1.2183E-172	0.612847593	0.592065914	-2.14189	0.064864	0.185147	TEF1
7.7813E-167	0.840784015	0.474290885	1.546551	0.024766	0.170976	TEF1
8.9396E-167	0.719429107	0.890182184	-2.89158	0.066905	0.120572	FLO1
5.5953E-166	0.762436308	1	4.213966	0.089288	-0.02466	MAL31
2.2916E-162	0.03246642	0.615820553	-1.83841	-0.18793	0.160193	MRH1
2.3353E-162	0.157263376	1	10.45614	0.401856	0.403209	CSS3
5.5309E-162	1.16401E-07	0.766734697	3.939187	0.83854	0.247961	MAL32
2.5731E-161	0.793839626	0.894445004	-2.10382	0.039427	0.087287	COA2
2.1945E-154	0.483584986	0.264879577	-8.47087	-0.19563	3.379724	SNO3
2.1083E-152	0.304258036	0.493450262	2.571731	-0.15702	-0.2966	PRM5
1.4304E-151	0.569517503	0.996250082	2.11618	-0.07941	0.042247	IMD2
2.088E-150	7.60973E-07	0.08281148	-2.18966	0.449394	0.435479	FMP16
3.0679E-149	3.87209E-06	0.919926509	1.617483	0.331709	0.060991	HSP82
9.92E-143	2.42059E-08	0.000222678	1.336175	0.329779	-0.50215	GSY1
2.7957E-142	6.72684E-13	0.992505689	-2.37435	0.685903	0.049238	PNS1
2.9334E-142	0.934743268	0.720083939	-1.45147	0.013806	0.110437	TIF1
3.5982E-142	3.8513E-20	0.340583382	1.591633	0.607864	0.226221	HSP42
5.1014E-140	0.000118538	0.844390643	1.944626	0.35873	-0.10794	FRE4
8.7716E-139	0.445190402	1	-10.0499	0.206968	-0.20739	DDI3
1.9763E-137	0.977693292	0.747048811	3.52605	-0.02141	-0.28725	YNL034W
1.8377E-136	0.096099628	0.328463464	-1.69856	-0.15438	-0.24133	ERG5
1.4322E-135	0.995096172	0.89826709	-2.17073	0.009305	0.094479	EEB1
1.0601E-134	0.302105614	0.096995994	2.889456	0.186022	0.622583	HSP30
8.9229E-134	0.000649548	0.378603211	-1.4604	-0.24439	-0.20023	YGR079W
5.9506E-133	0.384932157	0.589775129	1.350242	-0.07606	0.141176	KAR2
2.3103E-132	0.454754335	0.069148732	-2.25503	0.106363	-0.49387	COX26
1.8915E-129	0.001389186	0.059232365	-1.87061	-0.29968	-0.42922	TIR4
4.4572E-127	0.758162333	0.978193638	1.40695	0.035518	-0.03708	HXK2
5.0685E-125	0.004004613	0.800614102	5.428371	0.77035	-0.44992	MAL12
2.2271E-124	0.976915818	0.711317454	-2.83196	0.017414	0.229349	NCA3
6.2552E-124	0.196724669	0.689558052	-1.62588	0.125774	0.14263	PET10
3.4313E-123	0.860955797	1	-2.13901	0.034742	-0.02914	CSS2
8.2006E-122	0.686382379	0.28514767	1.267981	-0.04035	0.211402	TIF1
1.9619E-121	0.051484274	0.324301066	-2.63364	0.279271	-0.3906	GAP1
2.0401E-121	0.080620238	1	9.971244	0.540375	0.542685	YER188W
3.8869E-121	0.072884259	0.996250082	1.359458	-0.14508	0.030284	HXT4
1.8027E-120	0.140485465	1	-3.47968	0.284426	0.038457	HPA2

2.1131E-120	0.008068108	0.949031652	-4.40986	0.534606	0.182787	ECM12
1.3436E-119	0.012650856	1	-1.29617	-0.17982	0.020383	PRY2
1.4797E-118	3.43381E-12	0.770752057	-2.13406	0.661481	0.153966	NQM1
9.9562E-118	0.892744236	1	-10.1342	0.06782	-0.06802	YKR104W
1.0128E-117	0.097493121	0.042142302	1.551111	-0.15857	-0.41362	YHR112C
1.7952E-117	0.505136536	0.251462984	-1.82771	0.083955	-0.3147	HMX1
1.3027E-115	0.78818634	0.172556429	-7.44257	0.098694	3.631728	YFR057W
4.0538E-115	0.410849697	0.281242785	-3.3154	0.174611	0.585879	YMR321C
4.1624E-115	0.001963602	0.921286576	-2.67811	-0.43504	-0.1121	AQY2
4.8029E-115	0.05078776	0.617215067	-1.63505	0.184719	0.169671	YGP1
5.1394E-114	0.214770818	0.555225439	-2.68474	-0.20349	-0.30207	PRM7
6.0083E-114	4.7614E-06	0.545272974	-3.89835	0.797195	0.416351	HSP12
1.2265E-110	0.386354047	0.26370665	-1.71037	0.100989	0.297477	CYC1
1.6975E-109	0.98811108	1	4.81374	-0.02421	0.011565	YHR219W
4.6057E-108	0.569289414	1	-10.2652	-0.22041	0.221207	NFT1
7.6746E-108	0.030221222	0.484474484	-2.58776	0.32032	0.356186	ATG34
2.5292E-107	0.276167395	0.791580335	-2.26823	0.163335	0.166841	YER186C
2.1269E-106	0.109495914	0.806662883	-2.77442	-0.26906	0.191332	YLL053C
6.5979E-105	0.009754686	0.057460658	2.290509	-0.36328	-0.60707	TIR2
2.2323E-104	2.8142E-14	0.283510967	1.953227	0.734937	0.352034	SIT1
6.476E-104	7.31945E-19	4.99598E-11	1.236154	0.537252	-0.85117	HXT2
2.219E-101	5.38755E-16	0.742563863	-1.99492	0.767446	0.165765	PHO84
6.7172E-101	0.655308895	1.2293E-06	-1.99453	-0.07258	-1.05108	STR3
4.1954E-100	0.124795835	0.031266694	-2.30067	0.223691	0.669141	YRO2
4.3517E-99	0.138509482	0.712613882	-1.83822	0.177541	0.17326	YLR046C
8.1555E-99	0.017747826	0.006151828	-1.3432	0.196207	-0.48258	ARG1
2.57122E-97	4.8916E-05	0.139514641	2.513445	0.579198	-0.58274	HXT6
3.81453E-97	0.3855976	0.951428598	-1.63472	-0.1038	0.060212	CYB5
5.72999E-97	0.980110212	0.768691181	1.412497	0.010155	0.118106	MNN5
5.76612E-97	0.118813668	0.859769494	1.08197	-0.11575	-0.06852	URA4
7.06149E-97	0.907732085	0.457137216	-1.32324	0.018756	-0.19319	EHT1
1.20637E-95	0.673042136	0.905065494	2.612438	0.097228	-0.13657	COS1
1.62446E-95	0.040742569	0.808567391	-1.34597	0.175149	0.098364	None
2.68206E-93	0.860003201	1	-1.52434	0.029188	0.027479	ERG3
1.13098E-92	0.01637518	0.715570823	-3.1491	0.449141	-0.31877	YHR022C
1.40053E-90	0.669617672	0.00883753	-1.42604	0.05329	0.51663	TAT1
5.29487E-90	0.758588991	0.7225564	-2.90734	0.076799	0.318904	COS7
5.68573E-90	2.09773E-17	0.017068248	1.122894	0.505491	0.391053	HSP78
1.30635E-89	0.749122844	0.678833479	-2.29764	0.067764	0.25288	YER187W
2.04655E-89	2.5751E-16	0.017074244	1.339124	0.582476	0.465965	EGO4
1.46519E-88	0.726657504	0.28514767	-1.31128	-0.04297	0.251454	PHO3
9.13185E-88	0.895841581	0.907544049	1.223777	0.020313	0.065098	TDH2
2.77032E-85	0.36814865	1	-1.37651	0.097385	0.015471	COX5A
8.09642E-85	0.234261318	1	-1.23068	-0.10993	-0.0104	CHO2
1.30465E-84	6.97719E-09	2.41586E-09	1.356068	0.451967	-0.96516	NRG2
1.70527E-83	0.915720176	0.545272974	-8.21445	-0.07578	-2.57166	AAD15
2.99044E-82	0.929429208	0.769965504	-1.88584	0.024481	-0.16747	GYP8
1.74885E-81	0.533524502	1	-9.86133	0.270348	-0.27169	None
2.84623E-81	1.28038E-08	4.65146E-09	-1.24437	0.404191	-0.86875	CTT1
7.07661E-81	0.655801161	0.533418515	1.335321	-0.05702	-0.19585	PRY3
6.37824E-80	0.007154715	0.817868751	-1.15537	-0.21009	0.090983	HMG1
3.71987E-79	0.972383608	0.69956533	1.892966	-0.01659	-0.20962	YML131W
1.94295E-78	0.028520862	0.017068248	-1.79245	0.270112	0.645558	ARO9
2.88176E-78	0.828465678	0.017297064	-2.00128	0.04774	-0.71305	PIR3

1.27252E-77	0.290897532	0.69828171	-1.94945	0.162764	0.218537	ATG36
1.27252E-77	0.010256893	0.708495578	2.097917	-0.38716	0.235285	YPL277C
3.42832E-76	6.38852E-05	0.002316113	-1.17745	-0.29848	-0.51595	SAM2
6.28909E-76	0.0122332	0.125375403	3.002929	0.536875	-0.83986	YLR036C
4.40537E-75	6.17915E-05	0.860187522	1.54479	0.403468	-0.11072	YER079W
1.5724E-74	0.689168252	0.306543708	1.553411	-0.0633	-0.32106	ECM22
2.0671E-74	0.762146426	0.956492205	-1.22064	-0.03868	-0.05065	ERG10
2.77744E-74	0.039495936	1	-9.26512	-0.67206	0.674695	YLR460C
1.20224E-73	0.981817797	0.938399843	2.08459	-0.0168	0.099272	SMA2
7.86369E-73	0.021107766	0.010751875	1.630001	0.278635	0.66058	ARN1
1.5416E-72	0.0122332	0.220954671	-1.47863	0.262859	0.343672	YPR010C-A
2.12134E-72	0.93529408	0.001799415	3.242428	0.04314	-1.73625	YHL044W
3.3356E-72	4.11288E-13	1.01919E-05	1.734332	0.753714	-1.03896	ICY1
4.28187E-72	0.914594311	0.924605282	-6.35434	-0.06286	0.624094	VBA3
4.44359E-72	0.001129793	0.077372954	-1.51166	0.331402	0.451631	TSA2
5.07593E-72	0.005971008	0.223381301	-1.35397	0.261114	0.314725	RPS22B
9.47158E-72	1	0.721271027	1.564822	0.004829	-0.17202	ADH5
1.97493E-71	0.134487173	0.598611712	-1.55408	-0.18018	-0.212	IZH1
2.01824E-71	0.184854161	0.777291545	-1.25189	0.132109	0.116778	CYT1
2.7161E-71	1	0.717705244	1.173396	-0.00615	-0.13076	YKR018C
6.83856E-71	0.145608335	0.904680493	-1.20251	0.137865	-0.07112	FAA1
2.57078E-70	0.997292987	1	-8.8701	-0.02244	0.022524	VBA5
3.97296E-70	6.54753E-15	3.88711E-07	1.105561	0.523625	-0.75714	AIM17
1.97019E-69	0.032400162	0.352843224	-2.89008	0.41609	-0.65957	SUE1
5.52108E-69	0.477410911	1	1.012677	-0.06731	-0.01329	INA1
6.65801E-69	0.567411892	1	-2.88309	-0.15044	-0.05338	YMR244W
1.18608E-68	0.362927385	0.907281575	0.986766	-0.08141	-0.05968	NPT1
1.19412E-68	0.653568266	0.711247098	1.255641	-0.05841	-0.14617	MLF3
2.07468E-68	0.044230009	0.484106841	-1.32672	0.202072	0.22281	None
3.24528E-68	0.94100612	1	-1.02749	-0.01333	0.018657	IDI1
1.01382E-67	0.345281679	0.764622115	-2.24542	0.180665	-0.2176	YLL056C
1.57686E-67	0.98685381	1	-1.27651	0.009364	0.011027	ERG24
3.20797E-67	1	0.704009942	1.207592	-0.00238	-0.14463	SUT2
3.22881E-66	1.7725E-18	3.36001E-11	1.327309	0.717612	1.174806	SSA4
3.46464E-66	0.157263376	0.298558417	-1.35615	-0.15658	-0.29193	SCM4
5.70188E-66	9.11881E-08	0.000400404	1.026722	0.361449	-0.5519	SDS24
6.69692E-66	4.8916E-05	0.32467013	-1.18463	0.325044	-0.24969	CYC7
8.49802E-66	0.477410911	0.681411478	-1.6225	0.106111	-0.19909	PRY1
1.39945E-65	0.946937461	0.289206404	-1.35113	-0.01691	-0.29665	ELO1
2.53929E-64	0.003275972	1.58678E-05	1.900437	0.420781	-1.17654	CIT2
6.55909E-64	0.122926465	0.919516515	0.95767	-0.12516	0.056111	GFA1
7.41937E-64	0.225195997	0.697639077	-1.28258	-0.13404	-0.15727	YEH1
1.82895E-63	0.030015186	1	-0.93339	0.159352	-0.01855	LSC1
2.31891E-63	0.04701393	0.223381301	-1.2489	-0.19737	0.314859	SLP1
1.47432E-62	0.332602552	1	-0.92176	-0.08229	-0.00871	MVD1
1.86308E-62	0.374998623	0.7990478	4.134283	-0.30803	0.5052	PAU8
1.08677E-61	0.077481906	0.601496564	-1.28059	0.184633	0.189306	QCR9
4.24037E-61	0.040357896	0.13805534	-2.21027	0.353558	-0.63085	RSB1
8.55251E-61	0.111956571	0.669483248	-1.05642	0.14146	0.141018	COX4
9.34809E-61	0.650518689	0.070441681	2.24575	0.112387	-0.76973	FLO5
4.8842E-60	0.103071132	0.099020366	1.609602	0.226865	0.522537	FRE2
1.20624E-59	0.914995243	0.341937853	0.918668	-0.01624	-0.20281	RNR4
3.86727E-59	0.086223435	0.166524458	-1.2312	0.177019	0.347151	COX12
1.22626E-58	0.035394486	0.777291545	1.496683	0.264311	-0.1575	APT2

1.58053E-58	0.820966378	0.944544569	-1.68486	-0.0484	-0.08599	COS12
1.67181E-58	0.019540108	0.058656331	-1.33168	0.249336	0.465427	COX7
2.72171E-57	0.488044682	0.05005618	-3.25389	-0.21083	1.280358	FEX2
3.02613E-57	2.68527E-07	0.00384257	-1.40608	0.497414	0.690571	INH1
3.40339E-57	0.164194878	0.349940473	-1.29003	0.158299	0.283316	YPR127W
3.88726E-57	0.011080935	0.016422539	1.726341	0.363283	0.758739	VMR1
1.16413E-56	0.507360916	0.484106841	0.886199	0.061197	-0.16722	SED1
1.96557E-56	0.010256893	0.865801776	1.892383	-0.41136	-0.15503	YCR102C
2.75872E-56	0.159515372	0.978338942	-1.69536	0.209026	0.065613	BNA5
7.99277E-56	0.001613442	0.283481217	0.877498	-0.22228	-0.21786	PLB2
4.32787E-55	0.48908547	0.698712509	1.007048	-0.07325	0.135915	PGA3
4.44207E-55	1	0.786137812	-2.44673	0.008879	-0.25311	TEN1
9.97321E-55	0.067063438	0.985804511	-1.17737	0.185884	-0.04241	DLD1
3.76819E-54	0.196968379	0.56739618	4.701004	0.461926	-1.1641	None
3.8357E-54	0.312265984	0.611449773	-1.53506	0.149555	0.24117	YOR238W
5.11738E-54	0.139167921	0.711541955	-0.85061	-0.11469	-0.10995	ITR1
8.31477E-54	1	0.920405239	0.974123	0.002136	0.061334	SCH9
8.70112E-54	0.199012093	0.152940749	-1.79859	0.211096	-0.54062	AAD10
1.15795E-53	0.413008972	0.888371569	0.884057	-0.07516	-0.06716	UTR2
1.7914E-53	0.152890044	0.904680493	-1.70184	0.21859	-0.11519	HBT1
1.92429E-53	0.760915703	1	-3.55742	0.115046	0.037296	SIP18
1.94371E-53	0.733844269	0.752830626	-1.25403	0.051906	-0.14393	ERG29
2.82169E-53	0.021955716	1	2.817188	0.557681	-0.06847	RCK1
3.06771E-53	0.156840624	0.822329423	1.263213	-0.16918	-0.12272	YGL039W
4.43708E-53	0.10288549	0.482770834	1.079522	0.161184	0.211502	RIB4
5.21417E-53	0.005839442	0.818523526	-0.87813	-0.20185	-0.08504	ERG13
5.58959E-53	0.144550842	0.867310986	2.596904	0.354662	-0.22002	YPL278C
1.00306E-52	0.929795254	0.971609311	-1.30046	0.02138	-0.0576	NEJ1
1.56134E-52	0.000827676	0.255041627	-0.95041	0.253177	-0.24936	TMA17
6.57983E-52	0.812862656	0.169547502	0.849663	-0.02775	-0.25869	RNR2
2.40715E-51	0.651526803	0.91703897	-4.01608	0.171104	0.335375	ENA1
3.30685E-51	9.00139E-10	0.227690315	1.600451	0.718113	-0.44762	YNR014W
6.59839E-51	0.516700017	0.85743687	-1.08117	-0.07593	-0.09413	YGL101W
7.87148E-51	0.194243001	0.89915479	-2.89599	0.336656	-0.22289	YHR140W
1.08382E-50	0.69190085	0.564416885	-6.59808	-0.19057	-2.45276	YFL067W
1.09896E-50	0.713873054	1	-0.85523	-0.03888	0.011189	ERG6
1.42369E-50	6.02083E-07	0.577042274	1.940592	0.736056	-0.33563	YNL194C
2.60408E-50	0.574872729	1	0.834961	0.054207	0.021663	GLR1
3.8308E-50	0.721368967	0.711257523	0.856566	0.038976	0.117108	PGK1
7.9988E-50	1.05046E-24	0.958971364	-1.0756	0.759043	0.054722	OM14
1.30418E-49	0.776818407	0.906168502	-1.18742	0.043692	0.083667	RPL22B
2.59601E-49	0.310202666	0.522217116	-1.08608	-0.11306	0.207084	MSH1
6.16377E-49	0.434822927	1	2.371535	0.203032	-0.0824	AYT1
8.30072E-49	1	0.492136803	-1.88071	-0.00434	-0.39097	YLR030W
1.24213E-48	0.650085956	0.029197458	1.398636	-0.07873	-0.61755	HMS1
1.74471E-48	0.371648425	1	1.245376	0.120743	-0.00991	GYP7
1.85213E-48	0.542558974	1	-2.68013	0.173682	-0.03877	YOL118C
4.49124E-48	0.525158576	1	-3.17955	0.202575	-0.12459	DCG1
6.22396E-48	0.013676019	0.029970437	-1.01573	0.22214	0.443219	COX8
9.34907E-48	0.009383928	0.918382276	-1.26966	0.290167	0.085251	ODC1
9.46849E-48	0.08182657	0.593717896	0.883644	0.147711	0.154634	CPR6
2.15951E-47	0.303874634	0.041755209	1.407668	0.15422	-0.59334	YAK1
5.68774E-47	0.267371286	0.839890676	0.896129	-0.10545	-0.08815	RPN1
6.33995E-47	0.002804181	5.06852E-08	-0.76664	0.199501	0.683365	HPF1

6.98821E-47	5.56603E-23	0.019048423	1.773623	1.276551	0.849991	FIT1
8.00571E-47	3.93115E-08	0.803816091	1.058096	0.453136	-0.11553	YIR016W
8.46152E-47	0.028520862	0.405149238	1.232201	0.253077	-0.28324	BNA6
1.14139E-46	0.759694936	0.094839053	-1.61863	0.065259	-0.58732	TKL2
2.38763E-46	0.053274021	0.215066762	1.640084	0.303457	-0.49286	GAC1
2.56424E-46	0.615223063	0.328463464	-1.18358	-0.0717	-0.30321	ARA2
4.27994E-46	0.378159684	0.74594446	1.924464	-0.19046	0.252847	SPO21
1.23355E-45	0.928674902	0.188377816	1.118064	0.020647	-0.3528	SNX4
1.33337E-45	0.720422379	0.379383422	6.242347	0.190062	2.915094	DDI3
2.09615E-45	0.102540414	1.10658E-05	0.835774	-0.13615	-0.64054	HIS4
4.03532E-45	0.406769713	1	-0.93551	-0.08621	-0.00017	ERG25
5.2429E-45	0.637757348	0.931005601	1.109485	0.066307	-0.07031	DIF1
9.56594E-45	0.001225541	0.121458281	-0.9012	0.254187	0.316248	MCR1
1.11176E-44	0.00098634	0.175291985	1.392872	0.405362	-0.45094	CMK2
2.85588E-44	0.967457599	0.669524096	-1.17661	0.014673	-0.1861	YFH7
3.01137E-44	0.529464272	0.807027851	4.475536	-0.28267	0.671536	YML133C
7.16911E-44	0.058256084	0.908303081	-1.3645	0.246598	0.100262	QCR10
9.4438E-44	0.016099167	0.420929331	2.118966	-0.49014	-0.52329	YCR100C
1.26832E-43	0.156939743	0.449106932	-0.9408	0.135683	0.209314	COX6
1.59684E-43	0.998303878	1	3.036698	0.021165	-0.02839	MST27
3.58372E-43	0.678241823	0.049980756	3.006434	0.164449	-1.27444	MAN2
1.2255E-42	0.970013423	1	1.585874	0.019956	-0.01047	KIN82
2.47759E-42	0.16612431	0.672010939	4.118743	-0.52033	0.898616	YNR075C-A
2.55463E-42	0.187412702	0.817868751	-4.0796	0.447773	-0.60725	THI5
2.67392E-42	0.284831522	0.597863762	-1.77238	0.201818	-0.33841	YLR031W
9.72866E-42	8.72349E-05	0.067121145	1.593843	0.551982	-0.65753	YLR149C
3.01429E-41	0.005726133	0.015213313	-0.82403	0.214852	0.426696	NDE1
3.85891E-41	0.455741185	0.213690034	-0.78598	0.069499	0.249867	RPS4A
4.55991E-41	0.598147339	0.592391518	0.894589	-0.06163	-0.16916	GAL80
8.25007E-41	0.425297662	0.74594446	1.71626	-0.16284	-0.23144	JSN1
1.28574E-40	0.007698559	1	0.796111	0.20735	-0.02703	GLO1
2.02939E-40	2.78205E-06	0.793455902	0.833232	0.337457	0.101654	YDL124W
2.07646E-40	0.98811108	0.856077623	-2.00757	-0.01788	0.191343	YER053C-A
2.21877E-40	0.162783034	1	0.718289	0.108797	0.020114	APD1
2.80032E-40	0.01626178	0.361862491	-3.24366	0.70803	0.831502	DAL1
2.91821E-40	0.02375103	0.022570781	1.018443	0.23204	-0.51013	YPL247C
3.32629E-40	0.788431804	0.804576171	1.513058	-0.06129	-0.17794	ECM34
3.77714E-40	0.684890005	0.562048326	-0.94271	-0.05192	-0.1855	ERG26
4.60089E-40	0.500936572	1	7.894569	-0.36524	-0.3687	PAU24
8.64957E-40	0.032210521	0.031200361	-1.13195	0.240906	-0.53913	VID24
9.09338E-40	5.66115E-06	0.151369301	-0.71894	-0.28299	-0.25576	EXG1
9.09338E-40	0.034194294	0.972764042	-0.72757	0.154755	-0.0364	RAS2
1.0531E-39	0.012654875	0.84918567	-0.85354	-0.20919	-0.08628	OLE1
1.9575E-39	0.036556402	1	-1.27594	-0.26791	-0.03576	RGS2
2.13381E-39	0.086190501	0.859769494	0.853496	-0.15618	0.0855	KTI12
3.46389E-39	0.003427909	0.609220601	-3.11142	0.774761	-0.65757	UGA4
3.95567E-39	1	0.786584362	-2.40762	-0.01244	0.30523	HXT8
6.03041E-39	0.134427143	0.664126703	0.837526	0.13738	-0.1444	GUT1
9.16072E-39	0.010917159	0.133200553	-0.95598	0.240762	0.35378	YMR090W
2.13268E-38	0.758146893	0.98198617	1.228695	0.057453	-0.05742	FUS1
2.20953E-38	2.00864E-05	0.096973919	1.157373	0.447326	-0.46569	MRK1
2.56534E-38	0.97665513	0.843533194	1.379655	-0.01693	-0.14908	YER134C
2.57598E-38	0.026262642	0.931568877	-0.71462	0.161953	-0.04819	LSC2
3.08236E-38	0.46286428	0.290162005	-0.79661	-0.0723	0.233824	RCF2

3.08236E-38	0.870423644	1	-0.77379	0.022617	-0.00401	HEM12
4.40502E-38	0.323714622	0.771931968	-0.93413	0.108489	-0.12189	ZRT1
6.11244E-38	0.737659987	0.839890676	-0.90293	0.044378	-0.09748	YNL134C
6.37598E-38	0.276237448	1	-3.79431	-0.4395	0.136153	BSC1
6.61282E-38	4.62271E-06	0.58793448	-1.47024	0.582091	0.285687	YNL195C
1.45483E-37	0.100174653	0.87592683	-1.29135	0.225324	0.120511	GRE2
1.47264E-37	0.001475204	0.116060268	-0.74167	-0.2278	-0.28706	ERG1
1.52646E-37	0.006732239	0.840202254	-0.77652	-0.21069	-0.08407	ACS2
1.52711E-37	0.286101355	0.469965664	1.41587	-0.18157	-0.33462	FRK1
1.60686E-37	0.538147246	0.12136599	1.487834	0.120099	-0.57536	MGA1
1.80755E-37	0.885106299	0.164318185	0.899239	0.025217	-0.32778	DDI1
3.29227E-37	0.413618808	1	-0.72622	-0.07306	-0.00857	ERG9
4.18883E-37	1	0.005175755	-0.74167	-0.00248	0.449853	ERG2
4.58447E-37	0.983012168	0.590394506	1.440912	-0.0163	-0.28837	YHL042W
4.69683E-37	0.402010292	0.243765908	1.161215	0.12096	0.376717	RPL1B
5.68372E-37	0.464159202	0.013997445	0.981398	-0.09265	-0.54829	SRL3
6.66958E-37	0.133335918	0.587251805	0.774509	-0.13083	-0.156	RPT1
8.60244E-37	1.3186E-20	0.08281148	-1.18765	0.889771	-0.49245	YHR033W
1.53536E-36	0.894801769	0.712488585	0.91968	-0.02404	0.146851	HSC82
2.02662E-36	0.653029567	0.931005601	-1.50807	-0.09354	0.104187	FPK1
2.02662E-36	0.713530484	0.931568877	-0.77408	-0.04187	0.053731	ERG8
2.32716E-36	0.012133386	0.163693857	0.951409	0.25043	-0.35201	YKR011C
3.28078E-36	0.000508482	0.818523526	0.904683	0.306314	-0.10808	UBI4
3.63063E-36	0.039547582	0.561567789	0.846316	-0.1884	-0.17932	RQC2
5.11359E-36	0.652629347	1	-1.32097	-0.08317	0.046779	YNL040W
5.25839E-36	0.940255249	0.289206404	-0.8715	-0.01594	0.265015	RRP46
5.53087E-36	0.000648063	0.563376575	1.031332	0.344641	-0.21766	SIS1
5.66415E-36	0.570336862	0.255041627	1.771444	0.137645	0.57593	DOG1
8.49326E-36	0.019432143	0.738189833	-0.83311	-0.20408	-0.12267	MSC7
2.80058E-35	0.518133314	0.93147427	-2.14105	0.172334	-0.1598	YKL065W-[illegible]
2.95002E-35	0.013695435	0.118862393	1.269151	0.332443	-0.51026	YAP5
3.03024E-35	1	0.817868751	0.734029	0.001686	-0.08959	APA1
3.51715E-35	0.048237469	0.099783852	0.880453	0.19103	0.369989	GRX1
3.68468E-35	1.4131E-17	0.008362627	-0.90467	-0.65615	-0.53894	DAN1
6.69203E-35	0.713873054	0.003795849	-1.07156	-0.05883	0.687928	PDC5
7.64415E-35	0.860071452	0.362845506	1.504832	0.049479	0.433739	GIP1
8.01649E-35	0.09555214	0.420929331	-0.78905	0.146953	0.20416	QCR6
8.6083E-35	0.005966489	1	-2.23631	0.608546	-0.01483	GRE1
9.06296E-35	0.431938847	0.836674365	1.618579	0.166396	-0.18761	AQY3
1.12771E-34	0.050189688	0.011211089	1.117141	0.243129	-0.65375	YLR177W
1.24811E-34	1	0.040783448	1.22555	-0.00019	-0.61471	VHS3
1.43977E-34	0.001843378	0.011781836	-0.74712	0.234452	0.434501	QCR7
2.92229E-34	0.000286932	0.06526418	-0.72444	0.256564	-0.32754	GPP2
4.43322E-34	0.051527774	1	-0.89209	0.190257	0.022259	RIP1
9.32878E-34	0.324679283	0.7990478	0.932487	0.117551	0.122231	TOH1
1.02316E-33	0.024386386	0.239034519	3.345272	-0.76022	-1.41951	YAR066W
1.1284E-33	0.834204	1	-1.16371	0.042552	0.017211	PSR1
1.55136E-33	0.335596279	0.360013074	0.89605	0.111197	-0.25928	FMP52
2.38871E-33	0.968276508	0.661759663	-0.79278	0.011363	-0.1466	SCS7
4.29404E-33	0.008822013	0.635840497	-1.55059	0.423312	0.307458	YIL029C
4.55652E-33	6.60621E-07	0.607000533	1.069811	0.504416	-0.22032	GPM2
5.06911E-33	0.427585494	0.147719526	1.148332	0.122114	-0.4557	YAP6
9.60121E-33	0.177946472	0.653725725	-6.3108	0.560845	-2.42287	None
2.1892E-32	0.977602377	0.235142919	-0.79921	-0.0103	-0.27954	PKH1

2.85093E-32	0.002104078	0.532461006	-1.07995	-0.34757	0.251098	ATF2
3.01168E-32	0.413242794	0.119718988	-5.06715	0.368755	3.838316	YNL146C-[illegible]
4.10039E-32	0.177324846	0.862693719	-1.23895	0.199058	0.133647	APQ12
4.13714E-32	0.709900242	0.972387712	-0.7478	0.043527	0.041899	RHO3
4.21636E-32	0.391890423	0.711247098	-0.89018	0.100168	-0.15053	PIC2
4.68605E-32	0.713873054	0.511755541	1.171777	0.06893	-0.28421	UBP9
5.36659E-32	0.986843508	0.620580042	0.71274	-0.0079	-0.14636	RPN3
7.48002E-32	0.138242749	0.000270263	0.80647	-0.14587	-0.64316	LYS1
1.41027E-31	0.759529423	0.686727083	-0.73522	-0.03705	0.133671	ERG28
1.4866E-31	0.144030831	0.891813186	0.682735	0.121892	-0.06622	STI1
1.52707E-31	0.770087587	0.444473065	-0.87132	0.041693	-0.23075	GTR1
1.61371E-31	1	1	-7.5702	-0.0201	0.020245	SNO4
1.88534E-31	0.402601173	0.846846052	-0.93963	0.10458	0.108893	GCV3
2.0882E-31	0.919717643	0.938399843	-0.82779	0.019363	-0.05956	AIF1
2.31058E-31	0.813711396	0.013045788	1.413136	0.059703	-0.85921	SUT1
3.70403E-31	0.73093128	0.851392759	-0.98616	0.054573	-0.11231	CIR2
3.79063E-31	0.363817999	0.764340274	1.47202	-0.18107	0.221333	COS111
3.83177E-31	0.060073338	0.807027851	-1.75539	0.371254	-0.23118	ZPS1
4.14955E-31	0.461111981	1	1.03403	-0.10714	-0.03152	ATF1
4.20395E-31	4.61671E-19	0.284571567	0.638194	0.515816	0.213835	EMI2
5.43468E-31	0.563145324	0.87592683	0.988813	-0.08444	-0.10354	HRK1
5.599E-31	3.51833E-06	0.221074524	1.948368	0.884328	0.708731	BTN2
6.14682E-31	0.677097244	0.507578381	4.433115	-0.23034	-1.59389	YHR213W
7.48511E-31	1	0.043873822	1.560111	0.001057	-0.82724	AAD4
8.46187E-31	0.489296691	0.011931128	1.187287	-0.11632	-0.73138	MSN4
9.50002E-31	0.624879898	0.920011392	-1.25356	0.091275	-0.10309	CAD1
1.37806E-30	0.4171016	1	-1.81511	0.194685	-0.03955	MLS1
1.57616E-30	0.089587852	1	-0.66675	0.135262	0.02458	MIX17
1.86085E-30	0.76984172	0.294796767	-0.76443	0.037382	-0.25033	MOT3
1.86794E-30	0.882548805	0.568492423	-2.99536	0.083253	-0.79323	DAL4
2.07108E-30	6.47181E-07	0.467354309	1.543726	0.759363	-0.4163	YJL144W
2.3831E-30	0.463310994	0.930420244	-0.74903	-0.0766	-0.05824	ZRT2
3.15246E-30	0.045727816	0.844430869	1.295979	0.307304	0.157264	AFR1
3.97586E-30	0.194770307	0.058656331	1.677346	0.278624	0.855103	THI72
4.06415E-30	0.934679773	0.714788215	0.649814	-0.01414	-0.11371	PHD1
4.73723E-30	0.743416838	0.243549962	-1.08996	0.059114	0.388613	SIR2
4.76508E-30	0.048981072	0.77583136	-0.89082	0.205105	0.131067	UPS2
5.81185E-30	0.328875125	0.941464384	1.013263	0.135457	-0.07416	TAD2
5.98725E-30	0.905759808	0.853297291	-2.32749	0.058769	-0.27597	YPR195C
6.0124E-30	0.397478501	0.47679402	1.029063	-0.1217	-0.27185	RTK1
7.39641E-30	0.624111878	0.894068915	4.080825	0.29104	0.476314	MATALPHA[illegible]
7.86415E-30	0.899541977	0.16782387	0.767879	0.021646	-0.31193	GPA2
8.84244E-30	0.97988247	0.12136599	0.897233	0.011883	-0.39253	PRM15
1.06463E-29	0.70026044	0.357766808	-1.18901	0.073006	-0.35839	COX15
1.30932E-29	0.171035344	0.053709945	0.833031	0.145914	-0.42883	CTS1
1.43772E-29	0.045989617	0.857272986	-0.58685	-0.13915	0.067987	NCP1
1.95986E-29	0.737846426	0.950592115	0.640382	-0.03661	-0.04535	UBP6
2.41564E-29	0.604770313	0.92806956	-0.69841	0.055341	0.056419	GDS1
2.52387E-29	1	0.554211132	-0.90486	0.004342	-0.21209	DAP1
2.76692E-29	0.818564873	0.603045192	0.64053	0.027538	-0.14152	FRA1
3.59016E-29	0.338970995	0.056523979	-0.67067	0.087648	0.341244	RPL8A
3.92578E-29	0.92384638	0.339657587	-0.73663	0.017434	0.233035	HHF1
4.00112E-29	0.737846426	0.238946444	-0.68707	0.038856	0.253075	YBL081W
4.62772E-29	0.033709464	0.7990478	-0.75798	-0.19043	-0.10566	SAM1

5.32505E-29	0.132624237	0.243549962	0.957615	-0.18358	-0.35142	ASK10
5.56225E-29	0.839560652	0.218100263	0.849288	-0.03361	-0.32444	TYE7
5.61898E-29	0.488044682	0.264879577	-4.05999	-0.30859	1.978557	YJL225C
6.40932E-29	0.440961232	0.007546041	-0.77304	0.084249	0.514822	RPS14B
7.7401E-29	0.497819776	0.717477893	0.768833	0.076597	-0.13613	GLC8
9.56219E-29	0.332333267	0.373162993	-0.76057	0.101383	-0.23221	PUT2
1.76737E-28	0.980772539	0.990208543	-0.82711	0.01078	0.040638	IES6
1.87271E-28	0.33526319	0.717705244	-0.66344	-0.08864	-0.11687	ANP1
2.29734E-28	1.62768E-07	1	0.969209	0.516849	0.001289	OPI10
2.32382E-28	0.068421358	0.420929331	-1.14421	0.252191	0.33096	None
2.39536E-28	0.511462308	0.062663543	1.165387	0.114754	-0.59291	YDL241W
2.55183E-28	0.663621022	0.947945497	0.801627	-0.05732	-0.05884	PEP8
2.58783E-28	0.652629347	0.545272974	0.999436	0.073523	-0.24619	HAL1
3.64343E-28	0.20100005	0.417425061	-5.4446	0.550509	-3.2247	THI12
4.40164E-28	0.505136536	1	1.304157	-0.13062	-0.05697	YMR265C
6.05666E-28	1.4333E-13	0.00034954	-0.75131	-0.53866	-0.62103	MET6
6.24764E-28	0.63816324	0.201505014	-1.57825	0.115519	-0.64211	IRC4
6.92889E-28	0.203402724	0.509579361	-0.79704	-0.13349	0.206134	BDF2
7.0598E-28	1	0.893930829	0.680045	0.005296	0.07002	ENO2
7.17047E-28	0.002804181	0.331627583	0.659134	0.227651	0.218723	HSP60
7.46753E-28	0.97665513	0.410354533	-0.67987	0.009657	-0.20157	PDE2
7.6377E-28	0.227967015	0.043168161	0.984895	0.161566	-0.54403	PPZ2
8.14435E-28	3.22957E-16	5.89829E-07	0.721506	0.569423	0.791254	HSP26
9.82498E-28	0.979039665	0.770001732	0.605363	-0.00839	0.095693	SSE1
9.86954E-28	0.781865517	0.16670075	-4.54118	0.155034	-4.05988	PRM9
1.0178E-27	0.368966584	0.152940749	0.808326	-0.10503	-0.3497	YPS1
1.0178E-27	0.815832814	0.602319742	-1.17672	0.05108	-0.26339	ALD2
1.15169E-27	0.970013423	0.599915058	-0.72715	-0.01123	-0.16467	YOR1
1.29843E-27	0.170043734	0.614405454	1.264886	0.229617	-0.28251	ECM10
1.59274E-27	0.768264114	0.712529354	0.753797	-0.03975	0.139202	CDC37
1.97014E-27	0.887609886	0.582585088	0.6119	0.019536	-0.14471	PRE4
2.04931E-27	0.815832814	0.240672347	0.706679	-0.03162	0.270586	GPM1
2.13988E-27	0.511462308	0.466577874	-0.87476	-0.0858	-0.24345	ERT1
2.17069E-27	0.004061065	0.710318897	-1.45959	0.476125	-0.26621	MEP2
2.34723E-27	3.9889E-12	0.001723265	-0.61552	0.418261	0.474166	GPH1
2.34723E-27	0.760989947	0.83080918	0.92819	0.051004	0.124679	CTL1
2.69824E-27	0.196724669	1	-1.08685	0.184599	-0.03694	PFA5
2.76087E-27	0.140700887	0.609220601	-1.06321	-0.20171	0.237755	GFD2
3.1838E-27	0.030221222	0.854094663	0.853563	0.22911	-0.10515	YHR097C
3.54189E-27	0.922329251	0.517080711	0.658665	0.016601	-0.17301	GPD2
4.17515E-27	0.462711274	0.977659028	-0.84774	-0.09191	-0.04921	DSD1
5.00142E-27	0.377674449	0.842613421	1.071703	0.138312	0.138796	YOL014W
7.05572E-27	0.320534014	0.74313321	0.587221	0.083856	-0.10053	PRE10
7.54327E-27	0.936226276	1	1.204678	0.027309	-0.02889	YPK2
1.04762E-26	0.55847489	0.800614102	0.579579	0.053791	-0.08544	GSF2
1.05659E-26	0.295242295	0.290162005	0.797301	-0.11963	-0.28446	DIG1
1.2327E-26	0.423652434	0.00020131	0.681768	-0.08149	-0.60462	DLD3
1.57652E-26	2.87517E-46	1	-0.57917	0.778378	0.00629	SUC2
2.76605E-26	0.017283264	0.020433989	1.739183	0.517074	-1.10839	MAN2
3.2323E-26	0.000779302	0.197911929	-0.78206	0.299427	0.321151	QCR8
3.43787E-26	0.45440874	0.124159404	0.952631	0.10872	-0.44275	GGA1
3.57318E-26	0.067793298	0.868996053	-1.11541	0.256884	0.130492	ATP23
4.57157E-26	0.253536732	0.764340274	-0.65425	0.104733	0.107085	ERG4
4.98278E-26	0.094724157	0.720195138	-0.70896	0.154014	0.1305	GTT1

5.21184E-26	0.01494163	0.689558052	-1.00527	0.297646	-0.20018	ECM4
6.73563E-26	0.783235602	1	0.549233	0.028245	0.009072	PGI1
8.66875E-26	5.40673E-06	1	-0.643	0.315378	-0.01827	YMR196W
1.07183E-25	0.824878272	0.812668751	0.57699	0.025926	-0.08393	SCL1
1.35541E-25	0.482993634	0.000560624	-0.63347	-0.06836	-0.54185	SER3
1.73709E-25	0.541155731	0.669483248	0.74735	-0.07332	-0.15846	AIM21
1.77209E-25	0.427585494	0.938683904	0.666995	0.080876	0.053984	SSA2
2.01523E-25	0.346709681	1	0.841656	-0.11902	-0.01122	YPR174C
2.07568E-25	0.799128108	0.255041627	1.054729	0.052359	-0.40833	YGR127W
2.86973E-25	1	0.032675489	0.716566	-0.00545	-0.43577	RAD52
2.88085E-25	0.677097244	0.114153044	0.568811	0.041299	0.276502	RPS4A
2.92012E-25	2.0891E-19	1.79499E-06	0.737194	-0.67922	0.819337	PDR12
3.89547E-25	1	0.946767444	2.118238	-0.01277	0.173103	COS3
4.67484E-25	0.552705567	0.202387133	-1.0143	0.095963	0.4231	KTI11
6.49925E-25	0.005890111	0.983661882	-1.24355	0.414557	0.070368	YDR381C-A
7.30252E-25	0.267599743	0.385959394	-2.79438	0.394868	-1.06286	YCL048W-A
7.3047E-25	0.51846587	0.595381214	2.686576	-0.27073	0.715716	YPR078C
7.40093E-25	0.067354761	0.043168161	-0.56095	0.134615	0.327837	COR1
7.84055E-25	0.98811108	0.427445927	-0.9737	-0.01165	0.303151	MSS1
8.74598E-25	0.914594311	0.930835922	0.618317	-0.01728	0.053873	PCM1
9.6175E-25	0.0122332	0.004646745	0.594579	-0.19099	-0.45129	BIO2
1.38838E-24	0.864526529	0.056128421	0.770602	0.029696	-0.43212	UBX5
1.51249E-24	0.102673469	0.69956533	0.80684	0.179899	-0.164	PSK1
1.78077E-24	0.148485947	0.008483052	0.664441	-0.13466	-0.48204	VHS2
1.78077E-24	1	0.837234982	-0.71632	-0.00279	0.098761	GTT3
1.93145E-24	0.31098292	0.853367097	1.081033	-0.16513	0.141463	STP4
2.1024E-24	0.320262782	0.58876642	-2.06216	-0.29556	-0.52264	ASG7
2.39838E-24	0.024098511	0.404345893	0.771405	0.227504	-0.2487	PEP12
2.55442E-24	0.623266105	0.711247098	-0.91738	-0.07649	0.180677	YPR071W
2.78271E-24	0.232185134	0.44250063	0.528521	-0.09206	0.163299	SCW10
3.39197E-24	0.670263285	0.737474906	-0.74431	0.055363	0.135335	DUR1,2
3.4012E-24	0.000278456	0.117748886	-0.58279	0.248192	0.284765	ATP4
4.82633E-24	0.585440433	0.798608504	0.922829	0.085855	-0.14473	MMM1
6.74661E-24	0.712618958	0.689558052	1.077236	-0.0742	-0.22714	LUC7
8.02802E-24	0.769795979	0.781154036	-0.71965	-0.04004	0.117762	RPS9A
9.23374E-24	0.816009058	0.57834141	0.670958	0.032346	-0.17177	PRE1
1.27086E-23	0.011964436	0.625394004	-0.68682	-0.22358	-0.16247	RRM3
1.29781E-23	0.754750696	1	3.095821	0.172224	-0.16839	YOR387C
1.72629E-23	0.746350726	0.056128421	-0.52253	-0.03237	0.299345	HMT1
1.72884E-23	0.78296105	0.530625906	0.637767	-0.03468	-0.17702	DOA1
1.73877E-23	0.003719396	0.853162234	-1.46851	0.521924	-0.19494	DAL2
1.9846E-23	8.21754E-10	0.020319939	0.598442	0.402799	-0.40256	CIT1
2.03856E-23	0.568538677	0.573905565	-1.3399	0.126382	-0.34486	YHR180W
2.04105E-23	8.28909E-06	1.11734E-07	-0.71005	-0.36243	-0.87318	MET17
2.16782E-23	0.596380201	0.69956533	1.716461	0.158853	-0.36111	YCR099C
2.50031E-23	0.767459399	0.135324685	0.697222	-0.0403	-0.33889	MPE1
2.51677E-23	0.87366229	1	0.703488	-0.02672	0.008842	SNU71
2.89599E-23	0.16612431	0.021354889	0.504339	-0.1009	-0.33874	GLN1
3.78096E-23	0.250933629	0.927365945	0.828851	-0.14432	-0.07762	GCR1
5.8578E-23	0.004542745	0.50705979	-0.85753	0.308085	0.245745	PET9
5.98143E-23	0.000483252	0.035718466	-0.72666	0.30747	0.455425	MRPS12
6.42409E-23	0.044769919	0.706171263	0.755298	-0.20785	0.156745	CDC19
6.49119E-23	0.001052654	0.054756353	-0.68769	0.277773	0.399622	COX9
6.55517E-23	0.31098292	0.871227843	0.820761	-0.12979	0.10315	TAH18

6.64487E-23	0.528966778	0.771481707	0.694059	0.07326	0.120501	TPI1
6.78862E-23	0.667005173	0.990208543	-0.98202	0.075431	0.054204	TAZ1
6.82875E-23	0.295461185	0.46824606	0.549599	-0.08921	-0.16899	RPT2
9.19522E-23	0.843151545	0.132346701	-0.65589	0.028342	-0.32083	PDR16
1.33784E-22	1	0.702041373	0.752858	-0.00601	-0.15873	AVT3
1.3989E-22	0.298712865	0.000241139	0.977278	-0.15999	-0.93702	RNR3
1.49995E-22	0.436108065	0.235733788	-1.12371	-0.14004	-0.46848	YPC1
1.70869E-22	0.89418929	0.368515913	-0.69423	0.023078	0.242623	SOD1
1.8483E-22	0.759694936	0.771481707	-1.05959	0.062965	0.182121	RFU1
1.94868E-22	0.940875632	0.768691181	0.523115	0.01232	-0.09248	RPN2
2.02796E-22	0.384508616	0.100874715	3.590254	0.42751	-2.81177	None
2.08205E-22	0.934155569	0.697639077	-1.12172	-0.02791	-0.23877	YOR389W
2.13461E-22	0.835487572	0.75114195	0.785116	0.036249	-0.1443	SDS22
2.33085E-22	0.435407492	1	-0.60666	-0.07686	0.016737	PAN6
2.59523E-22	0.799456203	0.492130336	-5.3712	0.186838	-2.87657	YHR125W
3.83439E-22	0.030776241	0.599665071	-0.80652	0.235496	0.205374	RCF1
4.0623E-22	0.121546688	0.584277027	-1.59703	0.345607	-0.4174	NDE2
4.18821E-22	0.402684486	0.983370642	0.850467	-0.11669	-0.05238	SHE4
4.75403E-22	0.333037056	0.930420244	-1.48296	-0.22328	-0.13563	YIL166C
6.05072E-22	6.61603E-07	0.822329423	0.608187	0.353959	0.093299	MSC1
6.81531E-22	0.690335313	0.990712634	-0.67414	0.050293	0.037719	HTA1
7.76988E-22	0.079856981	0.060772124	0.698513	0.176175	-0.40953	YMR181C
8.0871E-22	0.385272897	0.099020366	0.744374	0.105891	-0.39968	FMS1
8.16854E-22	0.000138851	0.628060129	-0.81102	0.37567	0.197941	HOR7
8.45188E-22	1	0.712529354	1.311653	0.002125	0.27376	FEX1
8.95481E-22	0.663252335	0.715570823	0.548598	-0.04486	-0.11298	RPT4
9.02237E-22	0.156630548	0.847404354	-1.11155	0.228368	0.156439	YDR286C
1.32847E-21	0.956605945	0.705630037	0.743546	-0.01534	-0.15897	YGL036W
1.71982E-21	0.892842541	0.545272974	-4.66846	0.11726	1.848102	None
1.77037E-21	0.582123297	0.022570781	-0.81586	0.079368	0.561221	PMP2
1.99237E-21	0.305193244	1	0.680278	-0.11208	-0.03557	TFG1
2.20452E-21	0.514232347	1	-0.59643	0.066436	-0.01065	WHI2
2.21839E-21	0.215871633	0.388478515	0.518278	-0.09918	-0.18267	RPT3
2.39664E-21	0.026560847	1	-1.64426	0.489004	0.049641	YDR461C-A
2.53114E-21	0.733704736	0.842612183	-0.84591	-0.05682	-0.12299	PRP31
2.6209E-21	0.633340819	0.00166885	-0.60581	-0.05313	-0.52751	ALD6
2.77325E-21	0.023687856	0.318881364	-0.73826	-0.23102	0.284809	GPX2
2.80115E-21	0.775390182	1	-0.76259	-0.04412	-0.01434	NPY1
2.94482E-21	0.059742746	1	0.897432	-0.24308	-0.00984	SOK2
3.3285E-21	0.363817999	0.119718988	0.674551	-0.10182	-0.35445	LSB1
3.76383E-21	0.968013408	0.031200361	-0.57023	0.010462	0.380737	RPL9B
3.80303E-21	0.72655191	0.808567391	-0.77964	-0.05389	0.125557	EAR1
3.85143E-21	0.186602961	1	-0.70789	0.140916	-0.01893	DMA1
4.09679E-21	0.36891213	0.94082376	1.845118	-0.27823	-0.16403	YJR154W
4.69678E-21	0.265919992	0.35208106	-1.26852	0.219619	-0.4635	YOR338W
4.78772E-21	0.497819776	0.964236348	-0.59808	0.069268	0.045777	YIM1
4.80488E-21	0.98685381	0.23260457	-0.73866	-0.00996	-0.32443	RAD57
4.80579E-21	0.052367663	0.890182184	0.580564	-0.16245	0.070251	CLN2
6.63166E-21	0.753511485	0.546473621	0.579429	-0.03763	0.167005	RSN1
7.26334E-21	0.607278799	0.049893598	0.585833	-0.05599	0.369221	SRL1
8.0676E-21	0.134575272	0.139610149	-0.84157	0.186537	-0.42772	ERS1
8.73595E-21	0.98474138	0.955327449	-3.28363	-0.03806	-0.34707	None
9.13784E-21	0.985451395	0.91703897	-0.82438	0.011684	0.086367	FEN2
1.02467E-20	0.402148664	1	-0.63798	-0.08908	-0.02175	ERG27

1.08771E-20	0.15351117	0.810575835	-1.29828	0.274037	0.211298	CSI1
1.08999E-20	0.979904046	0.933146024	0.515	-0.0082	-0.04804	CDC48
1.23556E-20	0.169066016	1	0.723848	-0.15405	-0.03652	BUD13
1.55794E-20	0.006930015	0.077555297	1.421075	0.532398	-0.83714	LEE1
1.56759E-20	1	0.726816534	0.767408	0.000152	-0.1579	NUR1
1.63161E-20	0.849454954	0.341891426	-0.78342	0.034795	0.297229	MIX23
1.70223E-20	0.297563912	1	0.91523	0.156632	0.0097	SOL1
1.7967E-20	0.887204289	0.403591361	1.006145	0.037696	-0.35583	PTP2
1.93727E-20	0.00609714	0.931568877	-0.83476	0.310924	-0.07777	RSM7
2.44348E-20	0.836107259	1	-0.63954	-0.03049	0.015705	CPT1
2.80825E-20	0.009421675	1	-0.6753	0.243203	-0.03607	MIC27
3.07864E-20	0.37200931	0.856109565	1.027953	-0.15674	0.147096	RGM1
4.78145E-20	0.663621022	0.384202197	2.038868	-0.17541	-0.74837	DAK2
5.14823E-20	0.440243906	0.69956533	-0.5244	0.069679	0.11739	POR1
5.90477E-20	0.463310994	1	-0.57679	-0.07358	-0.01171	HHT1
6.26164E-20	1.9093E-08	0.224875404	0.633259	0.429478	0.291792	SOL4
7.75261E-20	0.413532829	0.550781538	-0.82345	0.114326	0.239958	BOL3
8.19653E-20	1	0.27811547	-0.53259	-0.00452	0.226604	PPT1
8.25453E-20	0.734964949	0.373398241	0.650296	0.04595	-0.24335	YNL208W
9.16093E-20	0.53992737	0.032675489	0.782041	0.087708	-0.53937	HSV2
1.03254E-19	0.164194878	0.711421266	0.759279	0.165968	-0.16731	PUN1
1.06022E-19	0.404471627	0.36347688	-2.99025	0.3748	-1.34038	YOL085C
1.18817E-19	4.35944E-07	0.385959394	-0.60514	0.373389	0.221524	GAD1
1.24395E-19	0.013475412	0.403591361	1.388173	0.494439	-0.50101	YAR023C
1.61294E-19	0.004691149	0.90453709	-0.86641	0.337927	-0.1011	YIL077C
1.83999E-19	0.040760459	0.539379949	0.857161	0.260864	-0.25811	YOR385W
1.92332E-19	0.487247805	0.313527933	-0.56221	-0.06938	0.229068	NSA2
1.9802E-19	0.790107901	0.914989908	-0.67418	-0.03889	-0.07433	ERG12
1.98329E-19	0.340662441	0.893360707	-0.66821	0.107576	-0.08243	YKR070W
1.99705E-19	0.062448847	0.92745323	-0.6763	0.186736	0.068632	DOT5
2.09834E-19	0.235853165	0.313687377	1.041065	-0.20385	-0.42625	NRT1
2.10899E-19	1.27641E-09	0.000885507	0.96031	0.706272	-0.92048	YJR115W
2.16376E-19	0.00097274	0.276169893	-0.69739	0.3089	0.29922	RDL1
2.22493E-19	0.853333651	0.699858425	0.696605	-0.03204	-0.15977	MSB4
2.31515E-19	0.714551955	1	-0.71628	0.05352	-0.0257	YPL068C
2.5337E-19	0.446288659	1	0.871276	0.117884	0.045245	COS9
2.55229E-19	0.321761523	0.688473731	-0.63968	-0.10699	-0.14987	YLR407W
2.84714E-19	0.891627765	0.055048108	0.54548	0.020379	-0.34893	CUP9
4.05803E-19	0.024386386	0.846570403	1.194537	-0.40458	-0.1872	IME2
4.08912E-19	0.885372644	0.631857671	0.500763	-0.01969	-0.13199	RPN6
4.65529E-19	0.137864205	0.16733912	0.925436	0.218097	-0.47395	MTH1
4.74856E-19	0.237237286	0.818523526	1.626141	0.317979	0.277322	None
4.88913E-19	0.529464272	1	-0.90752	-0.10375	0.023722	JLP2
5.17114E-19	0.462711274	0.493450262	0.988653	0.131423	0.323539	LIF1
5.40519E-19	0.098340256	0.179756965	-0.60395	0.153351	0.302587	ATP17
5.48534E-19	0.581221243	1	3.269033	-0.34579	-0.19105	YCR101C
6.28834E-19	0.662458676	0.888371569	-0.75331	0.065327	0.096749	TIM21
6.34562E-19	0.836107259	1	0.743457	-0.03739	0.015805	VPS9
6.69808E-19	0.46143745	0.87592683	0.829918	-0.11099	-0.11254	ECM29
6.86153E-19	0.107981181	0.004134287	-0.71866	-0.17911	0.629561	AGA1
7.09198E-19	0.290286754	0.889649609	0.9641	-0.1759	-0.12371	PFK26
7.96083E-19	0.672577856	0.2432512	-1.00348	0.084098	0.459268	TCA17
8.69956E-19	0.979039665	0.809711663	3.044639	0.047049	-0.60742	None
8.72057E-19	0.649028901	0.598759914	0.485724	-0.04451	-0.1364	LYS12

9.05971E-19	0.051638227	0.385959394	-0.5202	0.152401	0.195794	QCR2
1.00293E-18	0.83092898	0.063075209	0.653747	0.033757	-0.41263	NPL4
1.01889E-18	0.97733147	0.35748349	-1.46812	0.024911	-0.57783	AAD14
1.01889E-18	0.66730791	0.949031652	-1.51016	0.126913	0.134518	SHU2
1.10735E-18	0.041753901	1	0.609262	0.188085	0.032854	ILV6
1.10942E-18	0.001315508	0.41145986	1.232444	0.555225	0.454591	HXT1
1.2262E-18	7.17105E-05	0.063185034	-0.54533	0.285252	0.343326	ATP5
1.24292E-18	0.813026602	0.241434728	0.580277	-0.03219	-0.27005	SSL1
1.49311E-18	0.885106299	0.623756562	-1.4145	0.054814	0.383075	BNS1
1.54744E-18	0.877996534	0.744376434	-0.6565	0.027138	-0.13478	AEP2
1.58405E-18	0.616558301	0.648751155	-0.61203	0.059949	0.158001	HHF1
2.01993E-18	0.525275245	0.937179795	0.490132	-0.05857	-0.04763	RPT6
2.09109E-18	0.157263376	0.340307283	0.642403	-0.14833	0.261736	MRX12
2.156E-18	0.885106299	0.742563863	0.517978	0.020857	-0.10825	PRE5
2.17462E-18	0.338017134	0.210048952	0.674651	0.114329	-0.33047	IML2
2.48635E-18	0.374954065	0.163898132	0.634322	-0.10096	0.336971	HXT3
2.69695E-18	0.940898947	0.50705979	-0.71169	0.018355	0.231368	RPC25
3.00568E-18	0.007771785	0.919271701	-3.42449	-1.15337	-0.47117	YPR108W-A
3.07361E-18	0.227774912	0.614405454	-1.37366	0.27079	-0.38055	PHO11
3.13242E-18	0.272944905	0.647883595	-1.07134	0.198111	0.280846	MGT1
3.21477E-18	0.936093775	0.637177071	-0.91301	0.02485	-0.24038	GCV1
3.56217E-18	0.398255447	0.180992827	0.477741	-0.07299	-0.24614	SHP1
3.77377E-18	0.697408284	0.489219563	-0.64839	0.052445	0.2162	YDR524W-C
4.10068E-18	0.814509078	0.785906893	-0.61315	0.033787	-0.11515	SEN2
4.29128E-18	0.687268707	0.975984512	-2.60981	0.200176	0.205201	YIG1
4.68116E-18	2.26709E-17	0.001009254	0.662274	0.67776	-0.65543	TSL1
4.87767E-18	0.910686173	0.068733809	0.869849	0.029638	-0.55074	DIC1
5.21401E-18	0.622910776	0.97458978	0.524027	0.05207	0.039701	ADH3
5.41148E-18	0.887439198	0.179756965	1.298267	-0.05233	-0.67759	YML083C
5.4879E-18	3.15242E-05	0.040245891	-0.81054	0.445399	0.568072	MRPL33
5.66584E-18	0.663460514	0.55065098	-0.6172	0.054994	0.190109	GNA1
6.08062E-18	0.343957891	0.784759141	-0.45207	-0.07594	0.085546	VMA1
6.62388E-18	0.058697807	0.600468279	-0.44028	0.129256	-0.12522	PIL1
6.69408E-18	0.863721096	0.712488585	-0.60865	0.02736	-0.13909	OPI3
8.64521E-18	0.412595975	1	-0.5966	0.088544	0.022038	RPB7
8.90797E-18	0.999036524	0.261124045	-0.67339	-0.00738	-0.30785	DUG3
9.10465E-18	0.427585494	0.000344374	-0.97451	0.139721	-1.04022	FAT3
9.41591E-18	0.040742569	0.725989422	-0.84253	0.263335	0.185652	PAI3
9.82326E-18	0.007154715	0.438570787	1.13279	0.45745	0.415149	SDD1
1.08663E-17	0.019255559	0.27982387	0.567011	-0.20469	-0.25653	MET22
1.13188E-17	0.0122332	0.28514767	-0.66604	0.249598	0.295472	MIR1
1.19256E-17	0.897246027	0.89043028	-0.51427	-0.01912	-0.06737	TSC10
1.23108E-17	0.499874449	0.288830675	0.820436	0.105476	-0.36436	GID8
1.23962E-17	0.55262733	0.733663876	0.814852	-0.09502	0.179124	HSP31
1.27516E-17	0.04568163	0.058656331	-0.6473	-0.20137	0.426709	SAP185
1.52345E-17	0.527119409	0.38020077	-0.61185	0.073669	0.240681	IES3
1.56107E-17	0.011900388	0.007546041	-0.83337	0.314509	0.720836	ATP19
1.60566E-17	0.639535568	0.438612707	-0.55457	0.053139	0.2023	YDR524C-B
1.63639E-17	0.127610291	0.368395884	1.454303	0.364874	-0.58113	MAG1
1.7224E-17	0.152125016	0.62830703	-0.87605	0.204859	-0.24251	EDS1
1.91053E-17	0.834779949	0.804181925	-0.87156	-0.04515	-0.15732	SRO9
1.95887E-17	0.080143076	0.392148505	0.7685	0.218055	0.300874	INM2
2.3441E-17	0.842054391	0.909621954	0.521797	0.026632	-0.06322	TPD3
2.37941E-17	0.968967353	0.782726705	0.598024	-0.0122	-0.11637	TGL1

2.44696E-17	0.118813668	8.86376E-05	0.538218	-0.13939	-0.62259	ARG4
2.47026E-17	0.969492509	0.051669961	0.804276	-0.01633	-0.55056	SKM1
2.47913E-17	0.413532829	0.976378097	0.770782	0.11736	-0.05843	YAR028W
2.65796E-17	0.011575654	0.659038749	0.496053	0.192797	0.132315	BAT2
2.69938E-17	0.025415054	0.488692236	-0.8828	0.302309	-0.30086	PDR15
2.774E-17	0.554757324	0.524992421	-1.06903	0.122333	-0.34526	COQ2
2.93628E-17	0.001445517	0.136710348	-0.53812	0.247714	0.304259	MRPL11
3.05434E-17	0.589760072	0.321715999	0.671565	-0.07386	-0.29004	FAR8
3.07404E-17	0.909821229	0.043873822	-0.5784	0.02003	-0.40552	ADI1
3.23891E-17	0.155051744	0.032675489	-0.70921	0.167256	-0.52024	SSA3
3.24402E-17	0.018000801	0.29383174	-0.75936	-0.27621	-0.33537	MNN4
3.25368E-17	0.063687056	0.648898447	0.528898	-0.15807	0.143638	SAC1
3.32275E-17	0.382143759	0.919926509	-0.66676	0.106551	-0.07587	TAF13
3.36555E-17	0.96027393	0.223381301	-0.75502	-0.01666	0.373716	YML108W
3.74652E-17	0.055084413	0.711247098	0.753258	-0.23243	0.179704	CHS1
4.02473E-17	0.199012093	0.690551186	1.626261	-0.36532	-0.4106	YIR014W
4.77586E-17	0.003630141	0.081519515	-0.60966	0.26324	0.38401	MRPL20
4.80295E-17	0.008969822	1	-0.55937	-0.22367	0.012878	EFM1
5.16161E-17	1	0.229037932	-0.80853	-0.0046	-0.39833	GAT1
5.25897E-17	0.41585352	0.449106932	-0.50478	0.076232	-0.18409	NSG2
5.25897E-17	0.358333717	0.997024491	0.875687	-0.14965	0.05325	RSF2
5.26519E-17	8.2412E-08	0.064361732	-0.77171	0.536923	0.508228	YOR289W
5.45024E-17	0.037345625	0.92578246	0.719731	-0.24014	0.080236	SIP1
5.74203E-17	0.529154357	0.937363635	0.668442	-0.08294	-0.06755	ROT2
5.7588E-17	0.509499029	0.967394603	4.485026	0.438228	-0.43038	None
5.78928E-17	0.652939653	0.088859612	-1.46159	0.135023	-0.9141	ECM13
6.22853E-17	5.31206E-10	1	0.498058	0.401184	0.01324	GLK1
6.44498E-17	0.843256522	0.886876142	-0.58856	-0.02978	0.081314	RSA1
6.80473E-17	0.642716671	0.962684605	0.76284	0.074879	-0.06648	RPO41
7.5051E-17	4.7614E-06	0.254952703	-0.94042	0.574194	-0.4459	TPK1
7.5056E-17	0.637757348	0.312925981	-1.02189	0.099426	-0.44355	YKL068W-A
9.50914E-17	0.43007847	0.876200558	0.504974	-0.07605	0.072977	PFK1
1.12592E-16	0.186363168	0.688473731	-0.57908	0.13109	-0.14653	CPA1
1.14301E-16	1	0.323254295	-0.73363	-0.00537	-0.32018	THI20
1.22767E-16	0.670717765	1	0.472245	0.043443	-0.01999	SNF7
1.43854E-16	0.851320842	0.411002948	0.462154	-0.02322	-0.18107	RPT5
1.55439E-16	0.03938265	0.155227311	0.554614	0.184931	-0.31305	ORM2
1.72236E-16	0.103941245	0.845425423	-1.94609	0.504363	-0.32253	YAR068W
1.75975E-16	0.616539813	0.086540485	0.74132	0.078638	-0.47153	HUL5
1.87067E-16	0.8316826	0.997335542	1.833017	-0.1012	-0.1167	None
1.89718E-16	0.953675948	0.50705979	0.496662	-0.01225	-0.17219	STE4
1.95734E-16	0.761809122	0.992021238	0.565983	0.040222	-0.03696	BRO1
1.98826E-16	0.518133314	1	0.548462	0.070367	-0.0266	ARC35
2.00377E-16	0.261791204	0.616944383	-0.56287	0.113626	-0.16343	HFD1
2.04484E-16	0.102434008	0.314344681	-1.05688	0.283473	0.470031	MIX14
2.0854E-16	0.865084571	1	0.461785	-0.02188	0.021464	PRO2
2.14053E-16	0.750533048	1	-1.86237	0.131411	-0.10143	MND1
2.1643E-16	0.887439198	0.840006224	0.675176	-0.02835	0.11467	BZZ1
2.17751E-16	0.795533688	0.984748505	0.482831	-0.0304	-0.0344	RPN5
2.20722E-16	0.881859811	0.992021238	1.120096	0.049554	0.073838	RMA1
2.5065E-16	0.412644964	0.056128421	-0.46118	-0.07195	0.32055	LTV1
2.68658E-16	0.805302069	1	0.73076	0.044695	0.02917	MIF2
2.84558E-16	0.236477875	0.940676267	0.425104	-0.0908	-0.04306	THR4
3.07012E-16	0.893574975	0.61504221	2.383414	0.09548	-0.72826	ENA2

3.19585E-16	0.160949853	0.017177219	-0.57938	0.139575	-0.47934	UGA1
3.6352E-16	0.017770743	0.744381844	-1.36195	0.49904	-0.30262	SHC1
3.84297E-16	0.385471859	1	0.442646	0.073553	0.002416	ASN2
4.27378E-16	0.002352769	0.593717896	-0.51499	0.237545	-0.15664	MDG1
4.42821E-16	0.767459399	0.049980756	-0.54617	0.037777	0.39031	RLP24
4.55716E-16	0.000264138	0.017177219	-1.23913	0.646957	-1.00302	PUT4
4.82381E-16	0.248612302	0.471735065	0.591845	-0.12543	-0.21842	PBS2
5.04583E-16	0.87305028	0.617460449	-0.48547	0.022205	-0.14261	RHO5
5.11532E-16	0.911657957	0.975984512	0.476745	-0.01715	-0.038	YPR1
5.20676E-16	0.018089723	1.71379E-06	-0.73756	-0.27901	-1.01805	GCV2
5.22344E-16	0.630857184	0.785906893	0.58498	-0.06095	-0.11818	MVP1
5.24246E-16	0.483640078	0.558625562	0.530876	0.073996	0.173302	SPE3
5.27591E-16	0.362927385	0.125375403	1.444897	0.252345	-0.86112	YBR201C-[illegible]
5.38354E-16	0.001445517	0.0420421	-0.78586	0.374493	0.579272	ATP15
5.53311E-16	0.427585494	0.373751943	-0.5156	-0.07919	0.215389	RTT10
5.76775E-16	1	0.322647589	0.813028	0.00466	-0.36597	YMR317W
5.97709E-16	1	0.26370665	0.738476	-0.00352	-0.36016	SLM1
6.34623E-16	6.9967E-06	0.546473621	0.651164	-0.4164	-0.216	MAE1
7.24158E-16	0.295461185	0.737036218	0.758244	-0.14965	-0.17417	JIP4
7.39342E-16	0.177522395	0.133656507	-0.55591	-0.13161	0.330441	DBP8
7.57332E-16	0.418025408	0.950921142	0.626979	-0.09948	0.061354	YNL058C
8.3899E-16	0.72085728	0.75616091	0.618145	-0.05148	-0.13534	CNA1
8.81302E-16	0.783246578	0.755222158	0.536743	0.035839	-0.11783	FLC1
9.50485E-16	0.217407387	0.063736572	-0.78937	0.173562	0.542251	MRX14
1.02938E-15	0.903919288	0.818523526	-2.43296	0.081217	-0.52169	None
1.03014E-15	0.003904221	0.635840497	0.714822	0.325134	-0.20718	XKS1
1.1583E-15	0.985451395	1	0.552019	0.009263	0.032314	YLR179C
1.19161E-15	0.004332188	0.045957184	-0.60175	0.267441	0.4431	MIC10
1.20989E-15	8.58497E-07	0.847404354	-0.6067	-0.41703	-0.1022	ISU2
1.22892E-15	0.518133314	1	-0.53038	-0.06921	-0.02784	ARO1
1.31822E-15	0.09134229	0.702041373	0.700388	-0.20483	0.179739	TAF12
1.6369E-15	0.111813466	0.019048423	-0.49539	-0.13658	0.417595	SPB1
1.64713E-15	0.782040669	1	-1.10991	-0.07365	-0.02414	GRX8
1.6539E-15	0.464405882	0.411752857	0.455617	-0.06716	-0.18461	PRE2
1.69253E-15	0.995096172	0.186752472	-1.96084	-0.02465	-1.23541	None
1.72522E-15	0.983426723	1	-0.71428	-0.01239	-0.02685	TPO1
1.88523E-15	0.570094142	0.869100567	0.695323	-0.08438	-0.10857	INP52
2.20564E-15	0.293605247	1	-0.72924	0.144014	0.01323	GPI15
2.26696E-15	0.670089723	0.742768539	0.803123	-0.07741	-0.18439	PUT3
2.42444E-15	1.80782E-05	0.90423793	-0.56149	0.345743	0.074989	MRPS18
2.44048E-15	0.021955716	0.878872633	0.863986	0.328922	-0.12926	ALG13
2.44501E-15	0.702542191	0.58793448	0.464064	-0.04116	0.148038	PDI1
2.44734E-15	0.942240663	1	-1.92007	0.052069	-0.11018	YGR039W
2.52301E-15	0.644005887	1	-0.45862	-0.04679	0.007037	SAH1
2.5961E-15	0.720356067	0.812812583	-0.65389	-0.05462	-0.12412	SNF8
2.68263E-15	8.26747E-09	0.664126703	-0.62231	0.488214	0.173117	ALD3
2.76646E-15	0.177885721	0.598011569	-1.21492	0.287402	0.377969	None
2.98424E-15	0.39604593	0.937179795	-1.0022	0.164813	-0.10738	PRM1
3.04718E-15	0.236294203	0.378557332	-0.70176	-0.15314	-0.29603	ARE2
3.39511E-15	0.051373477	0.868427687	0.88756	0.295817	-0.14074	FYV10
3.65894E-15	0.914594311	0.911465377	-1.54645	-0.05319	-0.20829	None
3.76699E-15	0.448109038	0.89043028	0.640759	-0.09857	-0.09174	SOG2
3.83475E-15	0.126331092	0.593717896	0.829603	0.226103	-0.26257	VID30
3.86984E-15	1	0.068733809	-0.59211	0.004775	0.410544	REI1

4.25585E-15	0.482993634	0.325581139	1.222123	-0.17803	-0.56356	ATG1
4.47938E-15	0.995919167	0.894068915	0.782847	0.010668	-0.11105	ECL1
4.82776E-15	0.795210933	0.483346657	-0.62344	0.040647	-0.2325	AIM14
5.31921E-15	0.824457464	0.274285823	0.49445	0.029507	-0.24597	PRB1
5.35854E-15	0.587156933	1	0.617556	0.073296	-0.00505	ABD1
5.58208E-15	0.418773131	1	-0.42256	0.068059	0.021075	ACO1
5.67314E-15	0.781328521	1	-1.00997	0.068654	-0.0599	FMP40
6.63428E-15	0.446288659	0.764340274	0.555416	0.086106	-0.12318	PRE3
6.95275E-15	0.381873848	0.538130573	-1.04853	0.179231	-0.36616	SIP2
7.01964E-15	0.098839893	0.89915479	-3.42219	0.799766	0.533035	PAU8
8.18795E-15	0.094389373	0.615253714	-0.77934	0.22759	0.239879	RSM27
8.8633E-15	0.321967781	0.883095126	-0.59831	0.115053	0.089513	SCP1
1.00119E-14	0.281429987	0.860528802	0.582731	-0.12245	-0.09702	PIM1
1.00119E-14	0.954916262	0.91703897	0.480319	0.01231	-0.06105	GET2
1.02661E-14	2.6848E-06	0.560238155	0.543491	0.373032	0.184813	GRE3
1.03652E-14	0.719972823	0.461806117	0.458671	0.039669	-0.17982	PUP1
1.03652E-14	0.119397766	0.77583136	-0.78406	0.216561	-0.1681	RMI1
1.03652E-14	0.386071027	1	-1.09889	-0.18786	0.022932	PMA2
1.05404E-14	0.443115783	0.404345893	-0.58487	-0.09107	-0.24455	PSR2
1.12655E-14	2.72727E-39	0.680777882	-0.64231	1.087463	0.175933	RTC3
1.13055E-14	0.826578265	0.834791774	-0.82395	-0.04857	-0.15022	MRX11
1.16638E-14	0.490597664	1	6.205693	0.542241	0.553428	YPS5
1.17741E-14	0.986058626	0.712223429	1.124451	0.019304	0.289926	BNA1
1.18706E-14	6.75421E-06	0.931568877	-0.47088	0.309417	0.052737	GDB1
1.32787E-14	0.026368793	0.530625906	-0.48977	-0.18506	-0.17366	PST1
1.344E-14	0.759733432	0.739589142	-2.40373	0.167034	-0.62459	PAU10
1.507E-14	0.752831669	0.298179325	0.628678	0.049701	-0.30565	SRC1
1.5781E-14	0.197930958	0.89826709	-0.6897	-0.16503	-0.09744	MNT3
1.58857E-14	0.644005887	0.740020932	0.51128	-0.05429	-0.12191	PEX3
1.58976E-14	0.001922433	1	0.634496	-0.31971	-0.03099	MGA2
1.68543E-14	0.792192617	1	-0.84627	-0.05658	-0.02918	REV7
1.88991E-14	0.095680569	0.326601143	0.867001	0.260975	-0.40789	SNF3
2.07912E-14	0.446288659	0.92745323	0.537871	-0.08516	0.064715	ITR2
2.17541E-14	0.47929582	1	-0.44841	-0.06623	0.026483	ETT1
2.23646E-14	0.054666041	0.023996351	-0.78982	0.262295	0.667697	ATP18
2.4342E-14	0.139798632	0.898464555	0.542221	-0.14793	-0.0776	NPL3
2.44718E-14	0.760989947	0.863626849	1.190774	-0.09229	-0.19906	YHR210C
2.4565E-14	0.526283651	0.322465729	-0.52271	0.070353	0.247659	CSS1
2.47874E-14	0.455132842	0.91199009	0.983495	0.15444	-0.13013	SPO77
2.61551E-14	0.437604832	0.804919045	0.486374	0.07862	-0.09818	RPN12
2.62154E-14	0.454673425	0.281435365	4.662053	-0.57336	-3.51215	PAU18
2.67414E-14	0.619431177	0.822329423	-0.64774	0.072489	-0.12357	ARH1
2.79564E-14	0.363192071	0.781154036	-1.2713	0.228722	-0.27183	AGX1
2.80605E-14	0.260412922	0.929344228	-0.69755	0.151738	-0.08153	PEX21
3.05158E-14	0.008253001	0.868127068	0.445466	-0.20005	0.073523	HIS5
3.25154E-14	0.005414104	0.016422539	-0.65266	0.298979	0.586255	TOM5
3.28609E-14	0.97665513	0.812668751	-1.23972	0.024761	-0.24767	IDP2
3.46964E-14	0.026560847	0.457306305	0.447303	-0.17343	-0.17977	RSC6
3.66543E-14	0.033770868	1	1.13802	0.423436	-0.00573	CSR2
3.91396E-14	0.979904046	0.570313772	-0.96294	0.018324	0.325776	YBL071C
4.15538E-14	0.389706992	0.281242785	-0.59939	0.104831	0.303178	YLR050C
4.34561E-14	0.017099317	0.466577874	-0.69721	0.28272	0.275603	ATP20
4.49006E-14	0.266200922	0.959977162	-0.66656	0.144796	0.065747	ORM1
4.88341E-14	0.153332607	0.87592683	0.740109	-0.19889	0.117766	INO80

4.98058E-14	0.889053342	0.89043028	-0.46894	0.02085	-0.06957	YJU3
5.07939E-14	0.060882746	0.28605422	-0.48583	-0.16161	0.244768	ELP2
5.41916E-14	0.386071027	0.043873822	-0.53651	0.095008	0.423273	RPS27A
5.58708E-14	1.90848E-10	0.53398283	0.472222	0.430349	0.171929	PNC1
5.63838E-14	0.055888135	0.969243029	-0.43768	-0.14852	0.040066	ERG11
5.97127E-14	0.358012927	0.931005601	0.510128	0.096605	0.059787	PUP2
6.25665E-14	0.440961232	0.123887547	-0.53677	0.08625	0.347106	ARF1
6.74212E-14	0.343034258	1	0.51824	-0.10109	0.02614	EXG2
6.95227E-14	0.301220993	0.88137054	0.669571	-0.14056	-0.10518	SKI7
6.96123E-14	0.019167121	0.620580042	-1.21471	0.481333	-0.38432	FBP1
7.50321E-14	0.770116444	0.699343867	-0.63788	0.047201	0.174216	RBD2
7.74442E-14	0.398327474	0.499488496	1.819906	0.319602	0.715316	YGR174W-A
8.66007E-14	0.818575144	0.931005601	2.464151	0.156925	-0.29488	YOL155W-A
8.79912E-14	0.998178363	0.848126811	-0.55762	-0.00714	0.099855	YMR209C
9.63305E-14	1	0.844390643	0.458659	-0.00159	-0.08433	PRD1
9.71601E-14	0.995096172	0.668242189	-1.41916	0.020221	-0.42823	ULI1
1.06411E-13	0.788431804	0.265706256	-0.51358	0.035926	0.269341	PNO1
1.12454E-13	0.443553864	0.795907717	-0.4798	-0.07772	0.101819	TCD1
1.20161E-13	1.24175E-13	0.099133414	-0.53394	0.555449	0.365219	BDH1
1.25984E-13	0.263065203	0.888371569	-1.07272	0.235818	0.163829	YOR019W
1.28294E-13	0.882194755	0.380020625	-0.57717	0.027607	-0.25949	SHE2
1.29739E-13	0.954188947	0.752502722	0.493778	-0.01334	-0.11787	DST1
1.4207E-13	0.24298702	0.217741418	-0.53838	0.123772	0.305302	SUI1
1.44192E-13	0.97156603	0.757681998	-1.4705	-0.03149	0.354574	None
1.45707E-13	0.2730437	0.283481217	0.620836	0.137903	-0.32086	MDH2
1.47684E-13	0.168010422	1	0.659817	0.175009	-0.00054	ALT2
1.5059E-13	0.644005887	0.16670075	0.564571	-0.06215	0.350373	MDN1
1.53632E-13	0.00064536	0.855930058	0.486026	0.271554	-0.08609	YLR257W
1.61117E-13	0.6123064	0.568492423	0.551608	-0.066	-0.19333	PAH1
1.65837E-13	0.536021145	0.837446458	0.807264	-0.11161	-0.15298	HSF1
1.66655E-13	0.997705487	0.760963293	0.496017	-0.0066	-0.11695	UBX7
1.76164E-13	2.51892E-13	0.005759542	1.210265	1.267967	-1.22503	MPC3
1.87041E-13	0.928554724	1	0.418982	0.014607	0.024586	SGT2
2.22392E-13	0.905759808	0.871227843	-0.64879	-0.0263	0.107668	KTR2
2.28099E-13	0.005024141	0.767199943	-0.53004	0.253531	0.122728	ATP7
2.47583E-13	0.984954183	0.674519217	0.676186	-0.01246	-0.19949	YGR130C
2.50532E-13	0.977906393	1	1.822488	0.037827	0.09305	HXT17
2.55924E-13	0.374954065	0.328122933	0.429086	0.080829	0.211347	UBC4
2.59017E-13	0.176877685	0.115159129	0.542982	0.143014	-0.36726	PEX30
2.72418E-13	0.036997746	0.008610678	-0.43805	-0.16484	0.438028	UTP9
2.75175E-13	0.000145646	0.721271027	0.819529	0.502718	-0.21543	YMR103C
2.77437E-13	0.770350536	0.962371769	-0.48236	0.036572	-0.04779	PPN1
2.98391E-13	0.707280138	0.274807381	0.483841	0.045942	-0.25699	FRD1
3.06893E-13	0.01281546	0.617266112	0.413533	-0.18313	-0.13571	LYS21
3.16089E-13	0.904919797	0.867310986	-0.66979	0.027588	0.11458	RMP1
3.16135E-13	6.24419E-10	2.0367E-05	0.509698	0.467134	-0.72368	PRM10
3.17752E-13	0.519987007	0.330481176	-0.60618	-0.08616	0.296615	MSI1
3.24515E-13	0.023022877	0.032004077	-3.0143	1.007266	-3.68766	YBR296C-A
3.29526E-13	0.22826373	1	-0.49922	0.119454	-0.00697	UBC1
3.41117E-13	1	0.17444567	-0.91834	0.000501	-0.56018	CRR1
3.43345E-13	0.055168494	1	0.590609	-0.20977	-0.02437	AVL9
3.43509E-13	0.085590336	0.023505165	-0.4323	-0.13881	0.387289	GCD10
3.43942E-13	0.816346001	0.607571263	-0.63599	-0.04129	-0.2116	YNL035C
3.46528E-13	0.950105874	0.91703897	0.60371	0.017324	-0.08131	MAD3

3.89852E-13	0.762473638	1	0.504387	0.040254	-0.01526	AIM9
3.91972E-13	0.241759995	0.818523526	1.136351	-0.27151	-0.23164	ECM2
4.24113E-13	0.008487526	0.040783448	-0.49571	0.228406	0.412378	COX13
4.26564E-13	0.645668107	0.92711763	0.695517	0.077937	-0.08835	ERV15
4.33564E-13	0.030231646	0.273681355	0.661909	-0.26307	-0.35498	ACA1
4.60021E-13	0.101132655	0.373398241	1.025863	0.321186	-0.4809	ZNF1
5.11441E-13	0.379997978	0.727497356	0.485843	0.091876	0.127429	MMF1
6.1343E-13	0.014673884	0.155227311	1.049015	0.46162	-0.67404	HFM1
6.44663E-13	0.881859811	1	0.698625	0.034911	0.013633	KSP1
6.56112E-13	0.714551955	1	0.529297	0.049906	0.034891	RAD6
6.58202E-13	0.033976678	0.98500242	-0.42007	0.162134	0.033278	ARA1
6.70455E-13	0.910686173	0.602319742	-0.51987	0.020896	-0.17581	DPP1
6.7091E-13	0.000703715	0.688444366	0.783066	0.447226	-0.22932	MHO1
7.59769E-13	0.046513917	0.182165955	-0.48649	-0.17919	0.300048	NRP1
7.94828E-13	0.0122332	0.940676267	-0.44968	-0.20192	0.051365	FAS1
8.57777E-13	0.616558301	0.781154036	0.426085	0.051571	0.098269	PAA1
8.86702E-13	0.100684361	0.863302781	-0.7305	0.226944	0.128769	YLF2
9.08018E-13	0.369301019	0.167974771	0.605691	-0.11845	-0.3847	VAC7
9.54837E-13	0.915872899	0.825106502	0.554056	0.02171	0.112952	HMG2
9.79207E-13	0.598332204	0.801754205	-0.75287	0.093678	0.162365	YHR202W
9.83794E-13	0.724774474	0.777667569	0.942237	-0.08743	-0.21964	SIP5
9.96216E-13	0.693233428	0.67702211	-0.4707	0.046605	0.141016	KRE9
9.96216E-13	0.696300915	0.898244609	-0.45737	0.045022	0.069573	YOR342C
9.96646E-13	5.75249E-08	0.625394004	0.478314	0.399919	0.158676	SSA1
1.02388E-12	0.217407387	0.664126703	0.533969	-0.13473	-0.1657	KSS1
1.03866E-12	0.812803114	0.929199554	0.52049	-0.03549	0.065907	GLE2
1.0697E-12	0.080149997	0.954808558	0.593499	-0.20019	0.06384	LCB5
1.11368E-12	0.842349593	0.980342156	1.262167	0.076097	-0.10701	ZRG8
1.15419E-12	0.227918099	0.744376434	0.607352	0.150684	-0.15367	TRR2
1.30373E-12	0.590801428	1	-0.99553	-0.12616	-0.05295	SNO2
1.31783E-12	0.000364043	0.218691212	0.535052	0.321419	-0.31648	GAT2
1.32304E-12	0.73191055	0.553808105	0.431868	0.039201	-0.16091	CUB1
1.38647E-12	0.743338017	0.795907717	0.458962	-0.04043	-0.10204	SHE3
1.40043E-12	0.311249398	0.721271027	-1.6088	0.328519	0.447711	YJL047C-[illegible]
1.51774E-12	0.730753693	0.805275919	-0.69743	0.062844	0.149791	NNF1
1.56168E-12	0.767359823	0.679082788	0.934207	0.075403	-0.28338	STB2
1.67999E-12	0.782040669	0.955306351	-0.61674	0.046361	0.066254	COX18
1.71147E-12	0.336320447	0.933469902	0.580177	-0.12144	-0.07068	IRC20
1.8034E-12	0.007240661	0.146124605	1.94677	0.945454	-1.3248	HXT11
1.84164E-12	0.846530385	0.748466881	-0.71782	-0.04216	-0.18128	SNU23
2.09511E-12	0.884663483	0.908303081	-1.02893	-0.05062	-0.14945	MMS21
2.39565E-12	0.528966778	0.004089016	0.547335	-0.08068	-0.60719	YLR108C
2.40075E-12	0.0122332	0.099020366	-0.52604	-0.24124	-0.37978	TDA4
2.45007E-12	0.641634198	0.966118712	-1.10568	0.125605	-0.11155	POT1
2.47566E-12	0.157263376	0.477818318	-0.47501	0.133856	-0.19891	GLO2
2.49915E-12	0.196724669	0.815112247	0.414327	0.109717	-0.08838	GPP1
2.60178E-12	0.729652252	0.04122541	-0.95117	-0.08669	0.826464	YKR005C
2.60369E-12	0.632634392	0.041689887	0.808764	-0.09732	-0.70051	TOS8
2.6054E-12	0.007561283	0.930835922	0.439581	0.214311	0.055498	AIM2
2.61841E-12	0.758162333	0.502003374	0.916691	-0.07782	0.374915	RPI1
2.63326E-12	0.995214622	0.967394603	0.367072	-0.00565	0.036678	YRA1
2.73403E-12	0.254444296	0.549318354	0.716101	-0.17351	0.273463	IMH1
2.87135E-12	0.753511485	0.080581045	-0.49632	0.042275	0.374135	RPL11B
2.93651E-12	1	0.488692236	0.895337	0.008841	-0.37299	TDP1

2.96314E-12	0.466138178	0.446640364	-0.69185	0.113609	-0.30181	MRS4
3.05279E-12	0.962059221	1	-0.76531	-0.01995	-0.03249	YER158C
3.10606E-12	0.71920823	0.979084259	-0.47145	0.044658	-0.04091	PTC7
3.20449E-12	0.934332746	0.128116449	0.744193	0.026247	-0.51561	PRP21
3.39207E-12	0.360478488	0.385959394	-0.61105	-0.12259	-0.2877	HNM1
3.42418E-12	0.238567362	1.68413E-07	-0.42256	-0.10423	-0.73242	HEM13
3.55005E-12	0.995214622	0.664126703	-1.12841	-0.01704	-0.35427	MRX3
3.59653E-12	0.629304116	0.635840497	-0.59954	0.07153	0.198687	KSH1
3.72042E-12	0.065477864	1	0.671555	-0.24127	0.033002	BUL2
3.75779E-12	0.315306627	0.870558299	-0.56241	0.121661	-0.09836	OXA1
3.76245E-12	0.325985364	0.744381844	-0.52438	0.111575	0.135118	PDP3
3.77044E-12	0.463827899	0.868996053	0.417884	-0.07035	-0.0748	RPN8
3.90231E-12	0.395197397	0.979069474	0.528433	0.100893	0.046412	LST8
3.90747E-12	0.011628293	0.113089839	-0.67556	0.312548	0.479856	RTC6
3.95518E-12	0.787133068	1	0.410586	-0.03132	0.015457	FPS1
3.98597E-12	0.905759808	0.911946622	-0.51452	0.022119	0.074117	FMP42
4.02265E-12	0.159515372	0.767199943	-1.13429	0.315471	0.277082	SEO1
4.04871E-12	0.663400253	0.968819743	1.645293	0.184386	-0.16425	YIR020C
4.063E-12	0.963282012	0.52788087	-0.41939	0.01095	0.165753	LOA1
4.23613E-12	2.87766E-09	0.57156263	1.201889	1.11246	-0.45457	LSO1
4.35687E-12	0.230911149	0.733389979	0.652472	0.165227	0.175603	PPM1
4.38395E-12	0.342998394	0.921286576	1.113998	-0.23632	-0.15245	YKR075C
4.84702E-12	0.770350536	0.041614907	0.378685	-0.03059	0.331587	FPR4
4.89562E-12	7.32313E-05	0.106067898	0.413602	0.276421	0.302214	MDH1
4.96457E-12	0.267136118	1	-0.3614	-0.08599	-0.01077	ERG20
5.07155E-12	0.089965595	0.29406496	0.398706	-0.1344	-0.21609	CRH1
5.09523E-12	6.35933E-12	7.79184E-05	0.396592	0.418417	-0.5615	GLC3
5.11391E-12	0.276167395	0.978193638	-0.48678	-0.11397	-0.04315	ERG7
5.74925E-12	0.596380201	0.279528124	0.988063	0.13048	0.553896	TDA6
6.1339E-12	0.004247119	0.027182603	-0.38983	-0.20274	0.36152	NOG1
6.35406E-12	0.102121722	0.103916575	0.472514	-0.15487	-0.34647	URA2
6.35406E-12	0.339193543	0.546473621	-0.46088	0.097029	0.177958	MRPL4
6.43878E-12	0.166941663	0.385856136	0.394753	-0.11267	-0.19108	TRP3
6.6198E-12	0.824457464	0.837344851	-0.63633	-0.04253	-0.12886	YGK3
6.92565E-12	0.692438881	0.908303081	0.551138	0.057499	0.082458	YOR131C
7.09032E-12	0.889353732	0.131426625	-0.62365	-0.03021	-0.43311	MET13
7.11733E-12	0.231864508	0.482770834	0.363765	-0.09273	-0.15565	TUB3
7.18667E-12	0.803994881	0.060744575	0.500917	-0.03642	-0.4065	MIG1
7.37393E-12	0.914451528	1	1.677329	0.06942	-0.09938	RPL39
7.69108E-12	0.28734684	0.188318842	-0.53011	0.122455	0.33767	RPA14
8.02048E-12	0.649493592	0.785906893	0.477651	-0.05595	0.113744	ZEO1
8.1105E-12	0.150647646	0.842759938	0.533787	-0.15849	0.107346	SST2
8.26028E-12	0.211239403	0.457306305	0.546835	0.144484	-0.24234	UFD1
8.4279E-12	1.49926E-11	0.993440716	-0.77263	0.795772	-0.05867	YJL133C-A
8.84528E-12	0.762436308	0.318346243	0.439605	0.037169	0.235395	FBA1
8.92357E-12	0.884827129	0.171413013	0.76389	-0.03951	-0.50341	MND2
9.10741E-12	0.162783034	0.412531018	0.464897	-0.13463	0.218867	VAS1
9.35076E-12	0.819541577	0.605911675	-0.69173	0.047256	-0.24591	SAY1
9.39673E-12	0.964932829	0.929344228	-0.68971	0.01786	0.089821	GEP7
9.61405E-12	0.311249398	0.201505014	-0.73907	-0.16391	-0.45947	MMP1
1.00047E-11	0.014121187	0.560238155	-1.33222	0.591602	-0.52094	HXT13
1.00348E-11	0.471162403	0.961511492	0.486896	-0.08271	-0.05274	AMN1
1.0558E-11	0.011369315	1	-0.53335	0.253284	0.006885	MRP4
1.10588E-11	0.442611824	0.759373966	-0.48902	0.086275	0.124296	AIM45

1.13761E-11	0.344342695	0.689558052	0.514876	0.109961	-0.15859	CAT5
1.19417E-11	0.369954042	0.659101564	-0.69143	0.13921	0.226455	HER2
1.20622E-11	0.715818525	0.770752057	0.401005	-0.03983	-0.09992	DSK2
1.22615E-11	6.6889E-25	1	0.533527	0.830158	0.008245	PGM2
1.25289E-11	0.227967015	0.224206217	0.673975	0.175372	-0.41296	OXR1
1.26641E-11	0.274269052	0.328463464	-0.48128	0.114804	0.253067	PPA2
1.29028E-11	0.290286754	0.095167231	0.907469	0.213729	-0.68365	ROM1
1.32373E-11	0.666045601	0.222458653	-0.44024	0.049344	-0.27016	CMP2
1.34372E-11	0.639878051	0.02895691	-0.49984	0.05981	0.466825	CGR1
1.3469E-11	0.668103802	0.735602795	0.552646	0.062187	-0.15115	TRS23
1.41694E-11	0.902912733	0.786584362	-0.43644	-0.01968	0.103844	HTB2
1.45307E-11	0.010609484	0.049893598	-0.51607	0.248889	0.441712	TOM6
1.50194E-11	0.950105874	0.972387712	0.685417	0.021273	0.066752	SAS2
1.50573E-11	0.317595659	0.795458324	-4.76718	-0.70495	-1.15956	YEL075C
1.54003E-11	0.995469417	1	0.617529	-0.00979	-0.01181	SAP30
1.55735E-11	0.347850933	1	0.59566	-0.12762	-0.02461	YGR117C
1.58655E-11	0.527787705	0.246071819	0.49775	-0.07629	-0.29718	CMR3
1.65235E-11	0.814756057	0.96675302	0.456131	0.032469	0.048017	SUB1
1.78382E-11	9.74244E-22	0.015213313	-0.55409	0.800162	-0.559	DDR2
1.88159E-11	0.885106299	0.151369301	-0.48714	0.025034	0.336727	RPA12
2.00961E-11	0.48573866	0.23260457	0.586306	-0.09804	-0.35935	RIB1
2.092E-11	0.085529276	1	0.475505	-0.16747	-0.01605	YPS3
2.1675E-11	0.032251576	1	-0.94217	0.387813	-0.01138	UPS3
2.26141E-11	0.144889995	0.624539387	-0.47848	0.144715	0.16785	VHC1
2.48962E-11	0.437046259	0.703292774	0.589539	-0.10847	-0.17869	PTK2
2.57643E-11	0.22826373	0.546331004	0.606118	-0.1604	-0.24221	PEX5
2.68833E-11	0.597836233	1	-1.55443	-0.20379	-0.01047	YHL050C
2.69702E-11	0.347244143	0.87592683	-0.6307	0.13436	-0.11222	HRT1
2.91524E-11	0.199012093	0.659101564	-0.48105	-0.13233	0.160616	EFM2
3.14977E-11	0.320232732	0.721271027	-0.71389	0.159573	-0.20422	YNG1
3.18134E-11	0.15590479	0.990712634	1.922371	0.578618	-0.15337	FRM2
3.22872E-11	0.600450086	0.415187473	1.391579	-0.19038	-0.66948	YFL051C
3.22872E-11	0.343957891	0.888371569	0.464297	0.101388	0.080071	SFT2
3.24478E-11	7.02775E-07	0.781154036	-1.58233	1.260158	0.383798	SPL2
3.28624E-11	0.366527041	0.912865696	-0.38568	0.080392	-0.05781	VPS13
3.32393E-11	0.935821617	0.803017727	0.835005	-0.03035	-0.1938	KKQ8
3.3514E-11	0.424274634	0.937363635	1.600113	0.302905	-0.20139	PHO12
3.39859E-11	0.115959902	0.837262612	0.466597	-0.15416	0.098533	CDC20
3.55262E-11	0.437604832	0.731592804	-0.53515	0.097729	-0.14993	CWC21
3.63326E-11	0.528101039	0.158941638	0.953805	0.149026	-0.6637	YNL144C
4.1599E-11	0.413130887	1	0.396028	-0.07678	-0.01888	GSH2
4.22422E-11	0.989192764	0.52960144	2.372841	-0.04164	1.071179	COS3
4.29524E-11	0.890100171	0.971847034	0.568451	-0.02889	-0.05702	MAF1
4.30154E-11	0.840784015	0.861631716	-0.5731	0.037001	0.11031	FMP30
4.31165E-11	0.621834431	0.546331004	5.069464	-0.53328	-2.63512	YRF1-4
4.34265E-11	0.335509445	0.735465999	-0.48543	0.106962	-0.13545	MGL2
4.41285E-11	0.240168063	1	0.772297	0.202433	-0.01117	LCL1
4.54216E-11	0.37490782	0.868996053	3.112588	0.639125	-0.56782	IRC18
4.5654E-11	0.302233138	0.876579012	0.486723	-0.11501	-0.08769	VIP1
4.66556E-11	0.767459399	0.526082458	0.634063	0.054469	0.265785	DPB4
4.75346E-11	0.004332188	0.028177898	0.442357	-0.24213	-0.42687	GSH1
4.84887E-11	0.867220499	0.929303164	-0.47494	-0.02734	0.064235	VRG4
5.14217E-11	0.219561957	0.650488242	0.403617	0.109227	0.139018	ARC40
5.42368E-11	0.713873054	0.98500242	-0.60365	0.061423	0.051911	SPO7

5.45361E-11	0.558898164	0.284855193	0.487309	-0.07253	-0.28192	CRP1
5.46717E-11	0.482685637	0.612949583	0.539446	0.092722	-0.19751	PFS2
5.595E-11	0.46143745	0.041614907	-1.34729	0.233266	-1.21985	THI2
5.79552E-11	0.497819776	0.30230998	0.562335	-0.09396	-0.31767	YPL150W
5.95648E-11	0.712206218	1	-5.90782	-0.36377	0.371058	GEX1
6.01281E-11	0.08182657	0.987710835	-0.54847	0.195916	-0.0458	ARG8
6.23312E-11	2.5607E-06	0.283881682	-0.61145	0.487018	0.352838	GSP2
6.35729E-11	0.856203563	1	0.49475	0.030474	0.022106	UBC13
6.49368E-11	0.885748557	0.828700369	-1.22086	0.062301	0.271916	ATG10
6.63569E-11	0.412238661	0.163539503	-0.45758	0.088771	0.319108	SHR3
6.68083E-11	0.382001694	1	0.37041	0.076834	-0.01156	CLC1
6.90197E-11	0.174275989	0.702041373	0.476819	-0.141	0.148969	HRP1
7.02413E-11	0.473870551	0.89043028	0.444783	0.077877	0.076372	PDX3
7.04692E-11	0.00019709	0.000278421	1.317875	0.883443	-1.7677	RTS3
7.11198E-11	0.039037511	0.708809273	0.602924	-0.25444	-0.18511	SET3
7.13716E-11	0.163690013	0.822329423	-0.54873	0.163371	0.121918	RPL28
7.53062E-11	0.560250329	0.721271027	0.504665	-0.07552	-0.14851	MED2
7.5583E-11	0.813330588	0.165466155	-0.55285	0.040348	0.385115	SVP26
7.87767E-11	0.302532438	0.666296417	-1.09158	0.253009	-0.36699	YGL193C
8.07554E-11	0.082198263	1	0.502371	-0.18347	-0.02919	LHS1
8.47479E-11	0.484349463	0.855617443	-0.61711	0.104915	0.123222	DNL4
9.32231E-11	0.213168946	0.830042218	-0.57005	0.155957	-0.1245	CHO1
9.34237E-11	0.476345337	0.041798259	-0.52897	-0.09198	0.488285	PZF1
9.46051E-11	0.332333267	0.069148732	-0.38915	-0.08826	0.325517	HGH1
9.98004E-11	0.482993634	1	-6.38717	0.725753	-0.74842	None
1.00606E-10	0.289188979	0.562048326	-0.4483	0.109151	0.179117	SAR1
1.012E-10	0.019432143	0.648751155	0.613613	-0.2911	-0.21552	IRS4
1.012E-10	0.678241823	0.7990478	-1.57542	-0.17432	-0.36928	SPS4
1.07338E-10	0.353430988	0.927489937	0.350384	-0.07731	0.049456	ARO2
1.08451E-10	0.096769268	1	-0.5144	0.179119	0.010334	ATP12
1.09123E-10	0.012654875	0.00883753	-0.56975	0.279253	0.635722	PBI2
1.12578E-10	0.004018431	0.201440577	0.682771	0.381782	-0.45393	CAT2
1.16298E-10	7.18555E-09	0.726816534	-0.56785	0.545247	-0.16449	ISU1
1.20121E-10	0.781683071	0.727497356	0.587163	-0.04916	-0.17155	STB3
1.2053E-10	0.241267716	1	0.405448	0.108048	0.027082	UMP1
1.27068E-10	0.205442701	0.967394603	-1.16694	0.320123	0.125393	YIL165C
1.29483E-10	0.297448581	0.716456431	3.112661	0.656481	1.159203	YOR192C-(
1.31066E-10	0.417419311	0.003494785	0.463661	-0.0915	-0.56698	YIL108W
1.33405E-10	0.311290484	0.060238728	-0.47841	0.112399	0.412918	RPL33B
1.356E-10	0.044199429	0.522788552	-0.54732	0.224635	-0.23378	MTC2
1.37812E-10	0.679829215	0.931568877	-1.11766	-0.12555	-0.14987	YNR066C
1.42411E-10	0.840784015	1	-0.55682	0.036956	0.013469	SNM1
1.43884E-10	0.363817999	1	0.362641	0.079017	-0.01327	LYS20
1.44549E-10	0.867434951	1	-0.76642	-0.04482	-0.02904	OGG1
1.48946E-10	0.005645605	0.488692236	0.583591	0.318439	-0.26294	RNY1
1.50109E-10	0.184156664	1	0.639438	-0.18979	-0.00035	BET4
1.63688E-10	0.525579538	0.176431816	-0.41424	-0.06637	0.288523	TRM11
1.64004E-10	0.001985179	0.712529354	-0.51947	0.30553	0.158652	MRPL44
1.65049E-10	0.488044682	0.000805631	0.822306	0.142806	-1.10006	MUP3
1.66548E-10	0.335205662	0.539256261	0.824835	-0.19093	-0.34687	YBR225W
1.67083E-10	0.610223632	0.994517386	-0.57479	0.077994	0.046191	UTH1
1.75008E-10	0.302233138	0.89043028	0.579137	-0.14101	-0.10139	NAB6
1.75755E-10	0.046513917	0.234678175	0.899457	-0.37966	-0.58603	YPR015C
1.84326E-10	0.00147838	1	-0.46986	0.283448	-0.02364	HAC1

1.92977E-10	0.094685918	1	0.723129	-0.26159	-0.01872	AZF1
1.96709E-10	0.346584881	0.046842154	0.853348	0.193561	-0.7817	SDP1
2.05111E-10	0.286485784	0.072829578	-0.4274	0.106341	0.359857	RPL1B
2.10242E-10	0.877072549	0.977577446	0.378635	-0.0218	-0.03701	SPE2
2.20108E-10	0.533524502	0.021269734	-0.36482	0.057873	0.377428	NOP15
2.30186E-10	0.180943206	1	-0.60309	0.178166	-0.0304	OMA1
2.42547E-10	0.092479893	0.315733589	-0.63188	0.22605	0.360487	TOM7
2.53475E-10	0.045945353	0.840006224	0.832967	0.349671	-0.18094	TDA10
2.55688E-10	0.099660293	0.45277276	0.355719	-0.12702	-0.17076	TRP5
2.55688E-10	0.15183452	0.770752057	-0.4301	0.135385	0.113847	CCW14
2.64011E-10	0.347850933	0.795907717	0.552889	-0.12618	0.137677	RNH202
2.68425E-10	0.360478488	0.781704944	-0.47077	0.103698	-0.12105	BPT1
2.71044E-10	0.948230011	1	-0.4543	-0.0151	0.025184	AFG2
2.75649E-10	0.797483311	1	-0.49792	-0.03963	-0.00142	CCM1
2.80507E-10	0.524017731	0.735465999	0.353705	0.057939	-0.10346	HOG1
2.97073E-10	0.121199858	0.058656331	0.483023	-0.16536	-0.42869	YBR071W
2.97073E-10	0.003117739	0.091291814	0.432276	-0.25411	-0.35353	NBA1
3.00159E-10	0.995096172	0.889649609	0.423046	-0.00732	0.075736	AAP1
3.00226E-10	0.977851004	0.883095126	-0.486	-0.01147	-0.08882	MXR1
3.08386E-10	0.688094672	0.293015953	2.728107	-0.28937	1.9884	None
3.13658E-10	0.858991565	1	0.332904	-0.02098	-0.00452	WTM1
3.18959E-10	0.045349065	0.646635061	-1.45659	0.586227	0.531008	YPR145C-A
3.21717E-10	0.594731542	0.674519217	0.625576	-0.09044	-0.21383	RTG3
3.28904E-10	0.782318684	1	-0.44089	0.037087	-0.0272	GEP3
3.32624E-10	0.037277785	0.420929331	-0.54053	0.233325	-0.26866	CCC1
3.34746E-10	0.826169604	0.920011392	0.410121	-0.03006	0.06205	BLM10
3.42087E-10	0.002571956	0.983370642	-0.37058	-0.21945	0.034521	DRE2
3.48751E-10	1	0.032905196	0.451341	0.002084	-0.44481	VHR2
3.56166E-10	0.119807855	0.661759663	-0.50803	-0.17306	0.178429	YDR514C
3.60172E-10	0.880612135	0.1771952	0.578621	-0.03332	-0.41114	IKS1
3.67201E-10	0.427585494	0.731592804	0.486948	-0.0969	-0.14488	APL1
3.73022E-10	0.492643755	0.66765157	-0.58159	0.100529	-0.2014	SPC25
3.7395E-10	0.095680569	0.937363635	-0.57355	0.205827	0.076065	NHP6B
3.77563E-10	0.02963386	0.202387133	-0.41583	-0.1883	0.283701	UTP4
3.79904E-10	0.080001202	0.186880865	-0.37911	-0.14308	0.264483	UBP1
3.93181E-10	0.611875648	0.427445927	0.729488	0.102351	-0.36493	YJR039W
3.96249E-10	0.914616118	0.406901206	0.543622	0.024449	-0.27943	YGR237C
3.97714E-10	0.967317412	0.477818318	0.63892	-0.01794	-0.30015	MSB3
4.0388E-10	0.942225336	0.750952753	-0.73415	0.025768	-0.20572	MSH5
4.24448E-10	0.000459643	0.099020366	-0.38129	0.253165	0.308789	MRPS9
4.37974E-10	0.519603006	0.533816941	-0.71705	0.117789	-0.30801	IME4
4.53708E-10	0.836107259	1	-0.35328	0.024694	-0.00199	ADO1
4.63896E-10	0.052546303	0.702435074	0.553726	0.229436	-0.17929	MPM1
4.66527E-10	0.589846261	0.826782727	0.741071	0.109043	-0.17115	KDX1
4.69303E-10	4.27196E-05	0.978799772	-0.64697	0.482302	0.062568	UIP4
4.74868E-10	0.820917338	1	-2.68888	0.187052	0.12499	SPO19
4.81574E-10	0.96826199	0.962371769	-0.65541	0.017829	0.076263	None
4.92874E-10	0.099021983	0.559860815	0.536934	-0.19559	-0.22494	SND1
5.18236E-10	0.220446608	0.856077623	0.839047	-0.24036	0.175624	ULP2
5.45088E-10	0.692438881	0.971609311	0.589395	0.067804	-0.0629	BEM4
5.80596E-10	0.407136042	0.345404463	-0.54429	0.11184	-0.30351	TFB3
5.84636E-10	0.782318684	0.909621954	0.425772	-0.03661	0.06982	TPM1
6.05112E-10	0.360876565	0.861402377	-0.47061	0.105629	0.096275	DDP1
6.25895E-10	0.098839893	0.58793448	0.431677	-0.15817	-0.17413	UFD4

6.48722E-10	0.214175707	0.049422293	-0.44155	0.126509	0.41293	TSR2
6.67133E-10	0.090833226	0.358747602	-0.63641	0.234296	0.351098	YKL018C-A
6.70291E-10	0.93895154	0.919926509	0.539297	0.020417	-0.08304	SLU7
6.7943E-10	0.029490195	0.952440864	-0.52773	0.240934	0.065619	CBP6
6.89583E-10	0.830118162	0.629134428	-0.60357	-0.04358	-0.22611	SPT8
6.93638E-10	0.642716671	0.043873822	-0.39529	-0.05152	0.379048	SLX9
7.15168E-10	0.630979654	0.169011865	0.597381	0.080992	-0.43593	JHD1
7.16238E-10	0.439485588	0.69956533	0.453241	0.089462	-0.14996	SOL2
7.18641E-10	0.010710362	0.041260007	0.540792	0.28771	-0.52465	RCN2
7.19523E-10	0.149548529	0.940414551	0.400056	-0.13141	0.053357	SHS1
7.30988E-10	0.021955716	0.715570823	0.451637	-0.21968	0.143071	NUP2
7.79302E-10	0.827566727	0.688403077	0.62749	0.046641	-0.21422	FBP26
8.01225E-10	0.386354047	0.821665755	-0.60801	0.130413	0.143226	MLC2
8.10723E-10	0.788431804	0.721271027	0.519171	-0.04424	-0.16183	CLF1
8.28549E-10	0.018092653	0.994517386	-1.26317	0.604935	-0.10851	UBC11
8.42398E-10	0.637757348	0.771481707	-0.43341	0.05732	0.117714	ACB1
8.52691E-10	0.469895523	1	0.429955	-0.08088	0.01677	PRP40
8.53122E-10	0.666318943	0.846502628	0.764115	-0.09536	-0.16804	ATG20
9.0961E-10	0.347067432	0.339480893	-0.40238	-0.09358	-0.22899	OPT1
9.1366E-10	0.516700017	1	0.498864	-0.08565	-0.01177	CAB3
9.14256E-10	0.738978817	0.94443883	-0.488	0.049399	-0.06377	HEM3
9.36513E-10	0.080514417	0.441720189	1.234364	-0.48663	-0.64187	PAU3
1.00024E-09	1	0.889649609	0.71734	-0.00682	-0.13231	TDA11
1.0034E-09	0.676672695	0.996250082	0.540953	-0.06599	0.044814	CDC27
1.00415E-09	0.504911953	1	0.586193	-0.10307	-0.00807	RUD3
1.007E-09	1	1	0.474236	-0.00304	-0.02418	OTU1
1.02908E-09	3.15242E-05	0.480231621	-0.73822	0.564723	-0.3526	BLS1
1.1121E-09	0.339079516	0.256097342	-0.33592	-0.07973	0.217339	IMD3
1.21606E-09	0.848272786	0.90453709	0.432885	0.029556	-0.07477	TAH11
1.22566E-09	0.921385339	1	-0.46248	-0.02023	-0.0145	PAC10
1.25242E-09	0.402991749	1	-0.42769	0.090334	-0.01939	DEG1
1.25516E-09	0.432562651	0.7990478	-0.51836	0.10374	0.131675	YBR096W
1.3337E-09	0.050780234	0.136710348	-0.75003	0.316089	-0.57652	YCT1
1.3378E-09	0.006969693	0.951201607	-0.96391	-0.53778	0.122254	DDR48
1.35149E-09	0.227967015	0.786058196	0.574772	0.166004	0.153445	ERV1
1.36496E-09	0.833831544	0.712613882	0.41301	-0.03025	-0.13352	URA8
1.37015E-09	0.501017244	0.713282569	0.617265	-0.11022	-0.1994	CFT2
1.39433E-09	0.044176684	0.479096709	0.416225	-0.18362	0.202131	GGA2
1.41217E-09	0.024098511	0.241434728	-0.35587	0.171184	0.23688	MRPS35
1.4313E-09	0.687268707	0.971609311	0.433871	-0.05185	-0.04731	IOC2
1.44847E-09	0.820787111	1	-0.48398	0.037036	-0.03847	LAM6
1.46197E-09	1.11028E-06	1	-1.1525	0.99877	-0.02778	SPG4
1.48161E-09	0.905759808	0.066622967	-0.67397	-0.03294	-0.60062	MET32
1.58993E-09	0.827341893	0.709422392	1.066212	0.080877	-0.351	ISF1
1.60937E-09	0.479033885	1	0.335963	-0.06288	0.026593	MTC1
1.6753E-09	1.70583E-07	0.876200558	-0.49638	0.467289	0.096606	DCS2
1.6961E-09	0.379077824	0.862693719	0.462327	-0.10454	-0.09719	PIB2
1.77315E-09	1	0.264879577	0.399039	0.002165	-0.25817	PAM1
1.78495E-09	0.672399752	0.927845118	0.690957	0.086189	-0.10353	YGL117W
1.80543E-09	0.035632723	0.168268201	-0.39089	0.177674	0.291891	MRPL1
1.82008E-09	0.986843508	0.283724832	1.187418	0.025068	-0.73861	YJL107C
1.83561E-09	0.235614716	0.28514767	0.582289	-0.16822	-0.36624	SLD2
2.05621E-09	0.24298702	1	0.463357	0.131943	0.032552	OCA2
2.1333E-09	1.09363E-05	0.854094663	0.739738	0.614873	0.161095	JEN1

2.16258E-09	0.858991565	0.634900178	0.543546	-0.03605	-0.20991	VPS34
2.19409E-09	0.75339767	0.90086308	-0.55209	0.054596	0.098221	YDL085C-A
2.19409E-09	1	1	-0.37495	-0.00081	0.008941	HHT1
2.21098E-09	1	0.080581045	-0.56945	0.001337	0.498587	PUF3
2.25008E-09	0.209233788	0.108272796	0.73979	0.223409	-0.61339	AIM39
2.25683E-09	0.08182657	0.385959394	0.489869	0.193332	-0.26983	HUA1
2.27799E-09	0.842773453	0.924605282	-1.27726	-0.08771	0.201934	YAR029W
2.28789E-09	0.001052654	0.897313673	-0.52558	0.344671	0.094978	COX14
2.34593E-09	0.736287769	0.478036572	0.483208	-0.05115	-0.23719	PGD1
2.34713E-09	0.428360818	0.012916478	-0.46465	0.095353	-0.53572	PTR2
2.35578E-09	0.924898235	0.280851196	0.334437	-0.01465	0.214325	GUS1
2.35578E-09	0.717419826	0.589139583	0.591662	-0.06616	-0.24602	UBX2
2.47062E-09	0.302233138	0.745371404	0.328017	-0.08502	0.098266	OLA1
2.55443E-09	0.443908887	0.735465999	-0.5296	0.105535	-0.16284	ERF2
2.55573E-09	0.98474138	1	-0.44219	0.009923	0.00304	OST6
2.68034E-09	0.557203364	1	0.425735	-0.06987	-0.02236	SIF2
2.73082E-09	0.831957837	0.721271027	-0.92873	-0.06849	0.296476	YKL071W
2.74976E-09	0.120506262	0.093444318	-0.44673	-0.16004	0.383863	DBP7
2.85659E-09	0.125608279	0.754736247	0.915614	-0.32845	-0.26868	IES1
2.93581E-09	0.091520513	0.871412074	0.550408	-0.21451	0.112643	FYV8
2.93865E-09	0.842773453	0.976922401	0.723991	0.051874	-0.07586	LOT6
2.96513E-09	0.795210933	0.029558283	0.512608	-0.04438	-0.54298	TUL1
3.13899E-09	0.999192444	0.13719416	-0.57762	0.009033	-0.45644	YCR061W
3.25505E-09	0.013423142	0.708167385	-0.45278	-0.24138	-0.15188	VTS1
3.25505E-09	0.367878735	0.990110569	0.53663	-0.12586	-0.04919	SSD1
3.31466E-09	0.900055704	0.073409423	0.483213	-0.02547	-0.43641	AIM3
3.36999E-09	0.054646422	0.733389979	0.390961	-0.16942	0.122791	SSB1
3.41888E-09	0.302395299	0.782519814	0.390209	-0.10198	0.107612	PFK2
3.48216E-09	1	0.410952354	0.60394	0.005832	-0.32636	SKG3
3.48216E-09	0.339643562	0.702041373	0.306891	-0.07547	-0.1051	SIM1
3.64462E-09	0.112919922	0.750952753	0.379879	-0.14093	-0.11339	TMA108
3.65275E-09	0.690335313	1	-0.46185	-0.05545	0.011129	YHR078W
3.65442E-09	0.737054862	0.50705979	0.664384	-0.07124	-0.31673	YMR160W
3.66671E-09	0.095644016	0.62830703	-0.63649	0.24185	-0.24893	EDC1
3.81171E-09	0.855703098	0.400503761	-0.44588	0.030091	-0.24402	KIP3
3.82877E-09	7.48719E-13	0.405149238	0.601073	0.765721	0.329023	STF2
3.86034E-09	0.259238926	1	0.485294	0.137477	-0.02483	NTR2
3.88918E-09	0.286101355	0.949031652	0.425893	-0.11542	0.056884	SCP160
3.89214E-09	0.027559605	0.881648602	-0.36278	0.175679	-0.07115	IDH1
3.97216E-09	0.095680569	0.523868802	1.067422	-0.42011	-0.50853	YMR320W
4.0461E-09	0.666883176	0.726816534	-0.42386	-0.05426	-0.13445	CDS1
4.19413E-09	0.387479358	0.451887664	0.449646	0.101887	-0.23151	YDR306C
4.43992E-09	0.748196133	0.384874041	-0.42411	-0.04365	0.238237	YOR283W
4.54492E-09	0.570311092	0.620580042	-4.11654	-0.50424	-2.21509	CSM4
4.59324E-09	0.545224417	1	1.034963	0.175891	0.021368	PBI1
4.70769E-09	0.683010493	1	-0.6789	0.083077	-0.02081	IST3
4.82752E-09	3.9953E-62	0.46824606	-0.4627	1.289295	0.233155	NCE103
4.86084E-09	1	1	0.612796	0.006288	-0.0321	RIM8
4.90413E-09	0.899092334	0.46663268	-0.38604	-0.0205	0.195901	NOC4
5.15328E-09	0.945012036	1	0.307351	-0.01143	-0.00939	OYE2
5.21797E-09	1	0.625394004	0.674144	-0.0012	0.270597	XRS2
5.62183E-09	0.989163332	0.688444366	-0.42746	0.008736	0.152633	YPQ1
5.74787E-09	0.575831175	0.733389979	1.124121	-0.18256	0.358824	YOR365C
5.89856E-09	0.321712563	0.927963214	-0.3963	0.100935	-0.06078	YHM2

5.94154E-09	0.257727165	0.69828171	0.399863	-0.11506	-0.14094	VPS53
6.05102E-09	0.001017688	0.158941638	-0.4785	-0.32573	-0.37527	DSE4
6.10417E-09	0.684566541	0.38020077	-0.51671	0.063723	0.293964	PEX13
6.12039E-09	0.734964949	1	0.344307	-0.03751	-0.01173	STE24
6.26839E-09	0.346709681	0.361142562	-0.501	-0.12247	0.292795	UME1
6.72725E-09	0.464383989	0.397744731	-0.35096	-0.06957	0.195931	NOG2
7.02853E-09	0.013662562	0.795907717	0.401123	0.218797	-0.10817	SDH1
7.38611E-09	0.062193215	0.522788552	2.166887	0.896345	-1.13581	YMR082C
7.40742E-09	0.682492975	0.67652728	0.803758	-0.1018	-0.2965	RPR2
7.66432E-09	0.192500342	0.493450262	0.492743	-0.15926	-0.24446	ACM1
7.90418E-09	0.762704996	0.876200558	0.618332	0.061369	-0.12649	RGC1
8.08845E-09	0.989192764	0.372850575	-1.76902	0.035293	-1.09227	YGR121W-A
8.29411E-09	0.253113882	0.061449807	-0.31712	-0.0918	0.303102	UTP5
8.44752E-09	1	0.715024856	0.433464	-0.00411	-0.14626	VAM3
8.69115E-09	0.669969608	0.937363635	0.486109	-0.06368	-0.0705	ESL2
8.8146E-09	0.652629347	0.174129588	-0.7733	-0.1046	0.601336	YHR007C-A
8.93887E-09	0.391476898	1	0.443723	-0.10217	0.003766	COG8
8.93958E-09	0.252400336	0.997577123	0.470424	-0.13774	0.040882	SYP1
9.00261E-09	0.003618928	0.883095126	0.418605	-0.26493	-0.08409	PHO91
9.21586E-09	0.107317686	1	0.45634	-0.17612	-0.03074	SLI15
9.23083E-09	0.717511292	1	-5.47215	-0.38508	0.394223	YIL060W
9.2689E-09	0.598147339	0.698640889	-0.42739	0.065899	0.151254	RML2
9.32174E-09	0.271844334	0.859769494	0.582002	-0.16541	0.129697	OSH3
9.49289E-09	0.498519639	0.967394603	-0.44712	-0.08357	0.054246	PRP11
9.57672E-09	0.753511485	0.870558299	-0.42328	0.043579	-0.08931	ROX3
9.65578E-09	0.389906374	1	0.501917	-0.11623	0.024048	UBX3
1.01999E-08	0.595901981	0.543984117	4.373209	-0.52948	-2.6209	YGL258W-A
1.02715E-08	0.119925707	0.984628847	0.72053	-0.27103	-0.07263	IRR1
1.05343E-08	0.052519697	0.044385872	-0.45643	0.202652	0.467328	RPL26A
1.06956E-08	0.127179683	0.125525495	-0.33992	0.124114	0.285233	PAM16
1.07991E-08	0.843062252	0.706865475	-1.53986	-0.1092	0.549083	YBR032W
1.10415E-08	0.015182854	0.244196805	0.873885	0.477308	-0.61239	YER084W
1.10415E-08	0.587398198	0.88087787	-0.35217	-0.05595	0.071396	YPS7
1.10789E-08	0.257480037	0.816819753	-0.38382	0.111012	-0.09856	UTR4
1.11469E-08	0.676672695	0.92942427	0.358622	0.04639	-0.05546	GRH1
1.1179E-08	0.524017731	0.04678456	-0.35598	-0.06372	0.362901	RPA43
1.13954E-08	0.058914883	1	-0.53398	0.231967	-0.03103	HNT2
1.15837E-08	0.413532829	1	0.444048	0.098486	0.011676	SNA3
1.17535E-08	0.546851455	0.170622414	0.488425	0.084725	0.384325	CAF16
1.17911E-08	0.91441715	0.433015628	0.460393	-0.02284	-0.25049	YML020W
1.18791E-08	0.544905056	0.798716083	0.780058	0.136224	-0.21235	XBP1
1.18849E-08	0.920697634	0.935686072	-0.61385	0.028579	-0.09024	FRT1
1.19241E-08	0.813026602	0.863626849	0.559356	0.047036	-0.12299	RTT103
1.19418E-08	0.047950909	0.966197359	0.529539	-0.24475	-0.06553	ALY1
1.20085E-08	0.139315233	0.764340274	0.337239	0.12141	-0.10086	YMR027W
1.2016E-08	0.568084758	0.647440092	0.335629	-0.05614	-0.13289	RPN7
1.20163E-08	0.388201329	0.789249242	0.521816	0.121669	-0.14592	UBC8
1.20781E-08	0.085529276	0.828722881	-0.41161	0.166553	0.102565	MEF1
1.26533E-08	0.302233138	0.915496392	-1.85885	0.473841	0.322548	IME1
1.26937E-08	0.752643009	1	-0.57294	0.05975	-0.01179	MRM2
1.2834E-08	0.207905497	1	0.509692	-0.162	-0.03049	ARP5
1.29953E-08	0.058441959	1	-0.63269	0.275987	-0.02472	YER152C
1.30642E-08	0.770768828	0.255041627	-0.86529	0.082629	-0.5915	ARG3
1.30746E-08	0.04217695	0.625394004	0.39196	-0.18482	0.161505	FRS1

1.30746E-08	0.363817999	0.917145138	-0.52326	0.126437	0.088418	RNH203
1.3194E-08	0.931940862	0.971609311	0.322049	-0.01402	-0.03756	KGD2
1.33061E-08	0.15218416	0.868996053	0.457474	0.160876	0.09915	YET3
1.36113E-08	0.596380201	0.966118712	-0.65194	0.101631	-0.08006	SRB2
1.41772E-08	0.393339454	0.786584362	0.813872	-0.19048	-0.23104	YMR279C
1.41898E-08	0.363817999	0.87592683	-0.81654	-0.19756	0.169938	RAD28
1.43757E-08	0.613821587	0.848611069	0.404053	-0.06169	0.094303	SEC28
1.48338E-08	0.690379299	0.671237406	-0.44543	-0.05559	-0.16894	ECM25
1.48382E-08	0.639535568	0.868996053	0.484438	0.06965	0.105569	YCR023C
1.48437E-08	0.173197183	0.498203091	0.616947	0.209094	-0.30869	BOR1
1.49083E-08	1	0.164318185	-0.34132	0.000713	0.272855	BUD21
1.49364E-08	0.026191089	0.919926509	-0.75515	0.379349	0.12495	SEN15
1.525E-08	0.632899025	0.946857728	-0.36413	0.052656	-0.05072	HTB1
1.61122E-08	0.934743268	0.835951015	-0.42206	-0.0178	0.103581	RRP4
1.6519E-08	0.775390182	0.444473065	0.402507	-0.03893	0.218258	RFC5
1.65727E-08	0.671463457	1	-0.55615	0.072869	-0.03852	COX10
1.66099E-08	0.412262997	1	-0.61678	0.137027	-0.02914	YSF3
1.66139E-08	0.083183964	0.670601582	0.456668	-0.1905	-0.17446	NUP53
1.66723E-08	0.139315233	0.493774737	-0.87476	0.31138	-0.43895	PEX18
1.68468E-08	0.125494548	0.443073262	0.321883	0.120517	-0.17445	NTH1
1.71574E-08	0.874248567	0.953041258	-0.38781	0.025078	0.052342	YEA6
1.75583E-08	0.541996931	1	-0.46176	0.080786	0.02848	ICE2
1.76556E-08	0.999822551	0.332306908	0.343642	0.005558	-0.21519	GDI1
1.77073E-08	0.119522468	0.793556588	0.504887	-0.19309	0.141535	RGA1
1.83782E-08	0.079535843	0.91085821	0.407703	-0.17245	0.072858	VPS70
1.84223E-08	1	0.838466151	-0.42926	0.005094	0.104765	ANT1
1.89807E-08	0.015064016	0.171413013	-1.67786	0.859664	-1.42565	YHL015W-A
2.00633E-08	0.715720978	0.824055384	-1.00724	-0.11728	0.261488	LOH1
2.02015E-08	1	0.870558299	0.36495	0.004782	0.079411	DYS1
2.07122E-08	0.28690473	1	0.359805	0.101872	-0.02944	RPN11
2.08449E-08	0.716376728	0.721271027	-0.47492	-0.0559	-0.16038	ABZ2
2.08449E-08	0.896694852	1	0.389898	0.022091	0.003701	REH1
2.11433E-08	0.576536639	0.705630037	-0.64446	0.105853	0.228383	POF1
2.14037E-08	0.644005887	0.756960007	-0.36907	-0.05256	0.113803	POM34
2.15076E-08	0.500936572	0.859694751	0.33477	0.064251	-0.07647	PRE8
2.1736E-08	0.004018431	0.902973992	0.712912	-0.46002	0.135096	SIR4
2.19271E-08	0.642750802	0.053817845	-0.44071	0.062862	0.445011	YBL028C
2.22035E-08	0.729652252	0.000827407	-0.32058	-0.03667	0.49231	RPS28B
2.26364E-08	1	0.903248727	0.437726	-0.00605	-0.08273	MOB2
2.2704E-08	0.73107058	0.8119342	-0.42474	-0.04817	0.112957	YBR242W
2.30646E-08	0.204817452	0.828700369	0.471646	-0.15326	-0.12054	CYC8
2.40998E-08	0.472617197	0.812322751	-0.40174	0.081291	-0.10663	DFG5
2.4482E-08	1	0.533231335	0.644407	0.007999	-0.3116	MSS11
2.45998E-08	0.00020445	0.602672839	0.375152	0.292492	0.163116	YNK1
2.46821E-08	0.36016606	0.924605282	-0.42742	0.106261	-0.07057	AVT7
2.49364E-08	0.0122332	0.056523979	-0.56426	0.320257	-0.56391	FRE7
2.49364E-08	0.938970104	0.98500242	-0.65821	-0.02679	0.066818	JHD2
2.49714E-08	0.280027698	0.793455902	0.415646	-0.11993	0.117757	PAN1
2.55749E-08	0.023545099	0.294666115	0.834368	0.441154	-0.55343	AGP2
2.66417E-08	0.709900242	0.596430291	0.374353	0.045749	-0.16481	YPF1
2.66775E-08	0.259482058	1	0.379005	-0.11341	-0.00229	NAT1
2.73404E-08	1	0.466394013	-0.50085	-0.00203	0.267638	POP5
2.82426E-08	0.759733432	0.553808105	-0.41364	-0.04289	0.194611	DRN1
2.83787E-08	0.336744763	0.005410787	-0.5912	0.152915	-0.792	NRG1

2.86355E-08	0.379859709	0.98500242	-0.54953	0.13223	-0.05525	RDR1
2.88034E-08	0.349775738	0.889649609	-0.3677	0.093384	-0.07356	HEM2
2.92114E-08	0.826578265	0.646591934	-0.4252	0.034569	-0.17189	DLD2
2.92537E-08	0.387774178	0.00883753	-0.35257	0.083565	0.456478	YOP1
2.9478E-08	0.387479358	1	1.539496	0.367167	0.046663	None
2.97332E-08	0.468941828	0.713702664	0.513445	-0.10657	-0.18044	RQC1
2.98552E-08	0.10529773	0.523868802	0.334524	-0.1337	-0.16556	CSR1
2.99557E-08	0.942088826	1	-0.56867	-0.02255	-0.0436	LAM4
3.00029E-08	8.48925E-06	0.856077623	-0.50719	0.452868	-0.11708	IGD1
3.00263E-08	0.007294234	0.033474166	-0.37624	0.228056	0.416896	TIM10
3.00263E-08	0.564303748	0.049893598	-0.77113	0.130917	-0.80267	HEF3
3.03509E-08	0.501310332	0.976568193	-0.55938	0.106984	0.062737	SWM2
3.15347E-08	0.663621022	0.888371569	-0.46729	0.063916	0.094998	YCH1
3.15944E-08	0.32591766	0.224206217	0.551164	0.147812	-0.40994	YDR262W
3.15948E-08	0.879819172	0.024114512	0.654509	-0.04274	-0.75753	RAD59
3.18245E-08	0.340284679	0.045896858	-0.44125	-0.11451	0.465687	RPC53
3.207E-08	0.119316051	0.169011865	0.545917	-0.21353	-0.44305	NMA2
3.44289E-08	0.153838133	0.888371569	0.297207	-0.10738	0.061095	CCT4
3.46433E-08	0.286853393	0.398392914	-0.61825	0.175421	0.361092	YNL162W-A
3.46433E-08	0.475062233	0.41940157	-0.37537	0.076325	-0.21263	YPT52
3.46679E-08	0.99746658	0.703954346	-0.98438	0.017018	0.354908	YPL080C
3.49962E-08	0.001146851	0.122856134	-0.49855	0.351279	0.43428	YJR085C
3.57884E-08	0.399548813	0.822329423	-0.35201	-0.08232	0.092121	SAP190
3.65684E-08	0.480986795	0.602769533	-0.36104	-0.07287	0.158304	RIO1
3.66703E-08	0.818744368	1	0.36098	0.030774	-0.01326	UBC6
3.66842E-08	0.799128108	1	0.485507	-0.04441	-0.0141	CRZ1
3.76459E-08	0.430072037	0.811835676	-0.36984	0.081982	0.099981	ADH6
3.80657E-08	0.75567778	0.553808105	-0.39552	-0.04218	-0.18721	RPH1
3.85524E-08	0.84140879	0.017068248	0.625574	0.048757	-0.76894	YSW1
3.88019E-08	0.47409746	0.743105899	-0.3942	0.080547	0.128044	ESBP6
3.8878E-08	0.173422489	0.49370206	-0.63874	-0.22044	-0.33251	HOS1
3.98917E-08	0.332602552	0.049893598	0.411712	-0.11006	-0.43006	ROX1
4.0349E-08	0.0122332	0.937363635	-0.44517	0.257052	0.067028	RHB1
4.0349E-08	0.833160213	0.995105187	0.300317	-0.02424	0.028077	HTS1
4.05281E-08	0.899637841	0.546331004	-1.55096	-0.08415	0.766512	YAR064W
4.11877E-08	0.491008005	0.875200775	0.307088	-0.06124	0.066853	URA1
4.17257E-08	0.386354047	0.978338942	0.462207	-0.11246	0.05139	DPH6
4.2166E-08	0.156939743	0.710425231	-0.54966	-0.19672	-0.19653	RIM15
4.37734E-08	0.926213546	0.754404021	0.427158	-0.02011	-0.1358	SNF2
4.43198E-08	0.479751445	0.837446458	0.3961	-0.08122	-0.10003	CCT8
4.46208E-08	0.703189392	0.66258019	0.367435	0.046352	-0.14723	ODC2
4.47843E-08	0.713873054	0.745115043	-0.93961	0.112868	-0.30384	AIM33
4.49464E-08	0.16750316	0.378557332	-0.44089	0.154193	0.267113	VPS55
4.61528E-08	0.266207105	0.998341694	-0.32677	0.097341	0.029357	SHM1
4.74694E-08	0.919480877	0.28677728	-3.30167	-0.1315	-3.59845	SRL4
4.79378E-08	0.740566016	0.647440092	0.342653	-0.03898	0.141733	HOL1
4.79582E-08	0.414293185	0.967394603	-1.78218	0.393556	0.222663	YDR042C
4.80273E-08	0.958689082	0.024114512	-0.37969	0.012956	0.446129	UTP23
4.80448E-08	0.446288659	0.840202254	-0.4596	-0.09964	-0.11474	TRM12
4.96765E-08	0.803360108	0.865708429	0.555776	-0.05061	-0.12679	RFX1
5.04455E-08	2.70033E-14	0.099020366	0.321905	0.466125	0.298968	TFS1
5.33573E-08	0.310202666	0.585629945	0.327341	-0.09151	0.150297	GCD1
5.38074E-08	1	0.52788087	-0.31734	-0.00304	-0.15806	PCT1
5.38625E-08	0.681507345	0.014857079	-0.39601	-0.05265	-0.49348	BAP3

5.52779E-08	0.613821587	0.927619939	-0.7008	-0.10995	-0.11553	SPO71
5.61673E-08	0.078320718	0.668242189	-0.65918	0.283876	-0.26017	PEX2
5.81642E-08	0.886858489	0.289121329	-0.38022	0.023641	0.261139	TSR3
5.85881E-08	0.021842714	0.935686072	-0.38642	-0.21078	-0.05965	LEU9
5.86996E-08	0.196724669	0.27107384	0.444193	-0.15099	-0.31515	LEU2
5.97485E-08	0.97156603	0.904680493	0.951026	0.028684	0.183643	YGR016W
6.01512E-08	0.102962713	0.889079564	0.425108	-0.17475	-0.0884	FAA3
6.10154E-08	0.706333829	0.385959394	0.345006	-0.04372	-0.20916	CWH43
6.22892E-08	0.196724669	0.848126811	0.36439	-0.12389	0.089299	SPF1
6.27069E-08	0.891370788	0.937179795	0.377014	-0.02298	-0.05824	STP22
6.32803E-08	0.111981557	0.961003401	-0.50988	-0.20385	0.068572	AQR1
6.42324E-08	0.231230763	0.680395281	-0.39377	-0.12529	0.153482	AAT1
6.47929E-08	0.582123297	0.037301937	-0.35483	0.060128	0.397164	TRM112
6.68296E-08	0.330092657	0.721271027	0.39359	0.107236	0.138971	RIB3
6.71077E-08	0.830118162	0.350046527	0.30835	-0.02559	0.197406	MAP2
6.72873E-08	0.974667192	0.709738269	0.628488	-0.01831	-0.23046	CDC7
6.81882E-08	3.97272E-09	0.318881364	0.337078	0.394393	0.225247	YJR096W
6.83001E-08	0.131339384	0.978193638	-0.35842	0.13715	0.040502	MRP1
7.06094E-08	0.431639664	0.971609311	0.413921	0.094093	0.05056	MRI1
7.30371E-08	0.981637103	0.292120686	0.403913	-0.0106	-0.27849	PDR5
7.43984E-08	0.122042485	0.471735065	-0.52225	0.20389	-0.28314	IES5
7.51288E-08	0.707673932	1	-0.67996	0.085097	-0.02613	YJR012C
7.8464E-08	0.372381056	0.932413507	1.418337	0.362214	0.227782	YCL021W-[illegible]
7.89967E-08	0.446288659	1	-0.49556	-0.10889	-0.04166	SSY5
8.12836E-08	0.842370046	0.300568939	-1.01582	0.078481	-0.68995	AGP3
8.40626E-08	0.398465567	0.295087655	0.350779	0.084915	-0.24183	PGC1
8.61088E-08	0.975398981	0.595362002	0.562819	0.016371	0.258168	SAS5
8.6572E-08	0.059385215	1	0.453812	-0.21287	0.01428	SWH1
8.68879E-08	0.806027991	0.800614102	-0.50353	-0.0455	-0.14379	ECM5
8.81268E-08	0.03959407	0.873533661	0.343734	0.173252	0.077031	ARC19
8.90354E-08	0.65996974	0.400211383	0.429751	-0.0622	-0.25925	AKR1
8.99845E-08	0.686381253	0.356832107	0.349273	0.047049	-0.22323	LAP2
9.00258E-08	0.323293401	0.759373966	0.400518	0.111377	-0.12865	FRE6
9.27476E-08	0.382001694	0.661089241	-0.43601	-0.10886	0.179034	BAS1
9.40611E-08	0.758146893	1	-0.91196	0.097893	-0.05645	None
9.46433E-08	0.57019157	0.906628341	-0.55881	-0.09783	0.107191	AIM34
9.47573E-08	0.166666159	0.043168161	-0.41856	0.150404	0.461314	RPL36A
9.93915E-08	0.98570968	0.776410885	-0.39068	0.009499	-0.12043	THI6
1.02576E-07	0.731503094	0.786584362	0.477112	-0.05723	0.143848	CAF4
1.03295E-07	0.76388804	0.719999469	0.78118	0.083739	-0.28079	ARG82
1.04902E-07	0.261791204	0.57885291	0.372789	0.115614	-0.17586	LSB3
1.08847E-07	0.937038952	0.937363635	0.323553	0.014259	-0.05051	SHB17
1.10514E-07	0.472617197	0.327762587	1.280754	0.273389	0.869641	None
1.13049E-07	0.300891646	1	-1.14271	0.322188	-0.09515	YJR146W
1.14438E-07	0.294101962	0.789517744	0.528865	-0.1567	0.158752	CBF2
1.14883E-07	0.554757324	0.169547502	0.509521	0.093553	-0.42806	MYO3
1.20321E-07	0.732631743	0.69828171	0.450437	-0.05418	0.173871	IRC25
1.22231E-07	0.500337208	1	-0.58072	-0.11673	-0.04653	YLR173W
1.22344E-07	0.267877158	0.656171223	-0.48664	0.14853	-0.20248	PIH1
1.23312E-07	0.080426839	0.667670933	0.304249	0.135367	0.125011	SBA1
1.25926E-07	1	1	-0.35547	0.003217	-0.00995	RAM2
1.27596E-07	0.263680126	0.845203377	0.371757	-0.11608	-0.09477	EAP1
1.29172E-07	0.886323024	0.646591934	-0.36831	0.023557	0.156797	RIM2
1.2921E-07	0.147750837	1	0.380608	-0.14549	-0.00209	SCD6

1.32007E-07	0.295659	1	0.360707	-0.10662	0.029783	PPS1
1.33164E-07	0.124795835	0.569177865	0.745919	0.298836	0.360759	SRY1
1.33164E-07	0.047118728	0.924605282	0.297259	-0.14783	-0.05187	ASN1
1.34219E-07	0.884827129	0.906168502	0.52383	-0.03467	-0.10236	YKR078W
1.34841E-07	0.048588309	1	0.409892	-0.20338	0.010136	YOL036W
1.36042E-07	0.767694173	0.66907663	0.499219	0.052838	0.205196	YCR051W
1.36496E-07	3.93083E-05	1	0.362317	0.322975	-0.02039	MRP8
1.37002E-07	0.224489795	0.703292774	-0.51013	0.167247	0.1926	YSY6
1.37797E-07	0.57661888	1	-0.53835	0.093977	-0.03452	RCE1
1.39205E-07	0.143500097	0.990110569	0.463268	-0.17931	-0.04747	BUL1
1.41368E-07	0.148042869	0.996250082	-0.50213	0.189491	-0.04797	OSW5
1.42295E-07	0.009076575	0.868996053	0.485898	-0.30751	0.11333	ABP140
1.52684E-07	1.98697E-07	0.018502313	0.597525	0.646583	-0.74997	YGR161W-(
1.56623E-07	0.016892634	0.512491836	0.432965	-0.25438	-0.22893	TIP1
1.57338E-07	1	0.540028729	-0.40002	-0.0024	-0.2022	FIP1
1.58292E-07	0.769449587	0.004134287	-0.35507	0.037277	0.51989	ALB1
1.60245E-07	0.36194188	0.439260184	-2.07956	-0.51832	-1.40359	YOR316C-/
1.6043E-07	0.216106098	0.757681998	0.676503	-0.22981	-0.22265	GLN3
1.68975E-07	0.61447221	0.834791774	-0.44479	-0.07258	0.118044	MCM3
1.70897E-07	0.539290348	0.669483248	0.315574	0.060154	0.130431	CAJ1
1.7092E-07	0.000175552	0.744376434	-0.46418	0.383754	0.156815	UPS1
1.72545E-07	0.128488441	0.737474906	0.752206	-0.30317	-0.2615	PUF2
1.75709E-07	0.032733021	0.96218812	-1.05313	-0.55205	0.154003	YBR219C
1.81986E-07	0.276607865	0.772424036	-0.56342	0.171423	-0.1785	PUS5
1.83846E-07	0.500936572	0.002071764	-0.32029	0.065295	0.495787	RPL7B
1.83863E-07	0.266554604	0.89043028	0.403004	-0.12686	0.085585	BDP1
1.88645E-07	0.613304251	0.680777882	0.774753	0.128725	-0.31232	MTL1
1.91179E-07	0.246099917	1	5.274454	-1.3534	-1.43204	None
1.95342E-07	0.821809527	0.040783448	-0.30209	-0.02668	0.346465	ARX1
2.02084E-07	0.343957891	0.970494097	0.392254	-0.10847	-0.05114	DBF2
2.06382E-07	1	0.913392788	-0.41379	-0.00022	0.078341	MDV1
2.09341E-07	0.312265984	1.74933E-05	0.398893	0.115891	-0.78727	AGP1
2.09567E-07	0.374954065	0.409382088	-3.17335	-0.68558	-2.9986	None
2.09567E-07	0.615339573	0.919516515	-1.98819	0.295631	0.383942	YKR073C
2.10888E-07	0.496469376	0.46824606	-0.37078	0.076579	0.210256	BMH1
2.13358E-07	0.990169077	0.931005601	-0.50763	-0.01127	-0.08479	YMR155W
2.1586E-07	0.99746658	0.411752857	0.900168	0.016952	-0.5501	BNA2
2.18372E-07	1	0.715570823	-0.41023	-0.00176	-0.15142	SUR7
2.30939E-07	0.850649627	0.063736572	-0.41665	0.032563	0.437976	BUD20
2.36706E-07	0.132495235	0.483628979	-0.4487	0.178168	0.249855	IMG1
2.46549E-07	0.94036767	1	0.41251	-0.01803	0.031179	EAF6
2.51226E-07	0.286080627	0.649502951	-0.69	0.208704	-0.29619	GDH3
2.55275E-07	0.836107259	1	0.464092	0.039286	-0.03885	DTD1
2.5776E-07	0.715594168	1	-2.58903	0.292467	0.007048	YJL028W
2.58501E-07	0.027086984	0.76280131	-0.43472	-0.24095	-0.1436	YLR049C
2.61839E-07	0.912368353	0.482770834	-0.55373	-0.03026	0.309542	BUD23
2.63503E-07	0.730492063	0.537223328	0.47854	0.059501	-0.2483	YBL086C
2.65685E-07	0.954188947	1	-0.32918	-0.01248	-0.00048	FOL1
2.6573E-07	0.027690349	0.308492125	-0.8385	0.456463	-0.58232	PUT1
2.66517E-07	1	0.338433854	-0.29802	-0.00481	0.201423	TSC13
2.69284E-07	0.752831669	0.340307283	0.480549	-0.05597	-0.32457	ATE1
2.75719E-07	0.629304116	0.818523526	-0.47224	-0.07554	0.13303	YML018C
2.86681E-07	0.515859901	0.264418035	-0.40241	0.080862	0.30248	PMP1
2.98357E-07	0.624893229	0.608460014	-0.39314	-0.06379	0.183043	DUS1

2.98452E-07	0.884827129	0.264879577	0.452576	0.030693	-0.3409	ECM21
3.02438E-07	0.788420725	0.710318897	-0.42207	0.042341	-0.16191	CWC25
3.05967E-07	0.413008972	0.306543708	0.40473	-0.09999	0.289436	BFR1
3.05967E-07	1	0.447610866	-0.48341	-0.00152	0.284822	GOT1
3.06029E-07	0.885340254	0.419798397	0.430373	0.028971	-0.26323	STR2
3.06235E-07	0.272539197	0.846605423	-0.7819	0.242604	0.201657	MTC3
3.06235E-07	1	1	-0.81525	0.009722	-0.01694	PCH2
3.0753E-07	1	0.7990478	0.51507	0.008243	0.155462	BNA7
3.0753E-07	0.885961871	0.996590888	0.396046	-0.02643	0.038732	YML053C
3.13185E-07	0.023226764	0.545272974	-0.35333	0.200911	0.181903	RSM26
3.21481E-07	0.967392794	0.768691181	0.336906	-0.01138	-0.11064	RTG2
3.24408E-07	0.549614536	0.845425423	0.320063	0.061367	0.084183	GRX3
3.26264E-07	0.477410911	0.085125736	0.392231	0.086232	-0.39557	SUR1
3.27066E-07	0.989216982	1	-0.35267	0.008098	0.028062	SFH5
3.32705E-07	0.296901865	0.385959394	-0.40947	0.123044	0.262079	LSM7
3.4481E-07	1	0.467794221	0.290592	0.00016	0.169001	ARC1
3.50062E-07	0.575429137	0.810360586	-0.36183	-0.06577	0.10567	RRP45
3.51415E-07	0.669617672	1	-0.33746	0.04921	0.014218	PHO13
3.5621E-07	0.61337272	0.752502722	-0.41968	-0.07054	-0.14272	KRE5
3.66098E-07	0.47437713	0.98500242	0.341064	-0.07579	-0.03798	BUG1
3.68358E-07	0.092674979	0.927845118	0.353044	-0.15835	0.062324	PUB1
3.69948E-07	0.564303748	1	-0.41711	0.077514	-0.00862	ARG80
3.69959E-07	0.564106215	0.904680493	0.491623	-0.09273	-0.10011	HDA3
3.71708E-07	0.169066016	0.206688593	0.289137	-0.10939	0.240727	RPG1
3.72182E-07	0.374953133	0.715570823	0.520004	-0.1396	-0.19713	MUK1
3.78959E-07	0.813026602	1	0.469364	-0.04421	0.018041	YBP2
3.90151E-07	0.318191941	0.041643157	0.843869	0.249593	-0.98502	YKR015C
3.9706E-07	0.019464433	0.392456706	0.461757	0.273513	-0.29516	MFG1
3.99562E-07	0.327016784	1	-0.31624	-0.09141	-0.01278	NRD1
4.16312E-07	0.049774859	1	0.575828	0.294098	0.048872	URA10
4.21906E-07	0.146749371	0.834791774	0.391013	-0.15592	-0.10771	APL4
4.30674E-07	0.288190795	0.832278865	0.46098	-0.1445	-0.12808	RAV1
4.31216E-07	0.633674911	0.769266218	0.321198	-0.05213	-0.10651	TWF1
4.33044E-07	0.249550151	0.443140468	0.307665	0.10208	-0.18514	SEC17
4.33044E-07	0.953568694	1	0.454909	0.017985	-0.0062	ZDS1
4.40329E-07	0.074432949	1	0.5026	-0.23867	-0.03548	PIN4
4.40661E-07	0.654858264	0.223381301	-0.40016	-0.06125	0.325007	TRK1
4.45434E-07	0.01270424	0.967394603	0.598137	0.375702	-0.08163	TOS3
4.52586E-07	0.152035774	1	0.50836	-0.20137	0.003916	BUD3
4.55181E-07	0.439204391	0.063736572	-0.35649	0.084485	0.384197	SUA5
4.62338E-07	0.010823015	0.192674436	0.413912	0.266414	-0.35241	ATG19
4.67588E-07	0.746430113	0.93147427	0.49469	0.059737	-0.08444	FUB1
4.69007E-07	0.63232324	0.990110569	-0.41218	-0.06678	-0.0437	SMF1
4.69491E-07	0.806089523	0.721655021	-0.41453	0.039653	0.154614	RRP40
4.71546E-07	0.690374119	0.492151956	0.348703	-0.04919	-0.19707	POL31
4.71976E-07	0.485953152	1	0.437309	-0.09621	0.033313	WSC3
4.7619E-07	0.122862341	0.786892522	0.476678	-0.2012	0.151116	AFT1
4.78236E-07	0.040876372	1	0.270041	-0.14362	-0.00626	TKL1
4.78535E-07	0.078709799	0.917145138	-0.3156	-0.1475	-0.05997	CYS4
4.80677E-07	0.607770092	0.439260184	1.807491	-0.31397	1.152993	YHR022C-A
4.83353E-07	1	0.904680493	0.312305	-0.00366	0.064144	GET3
4.84808E-07	0.191649817	0.143826946	-0.27476	0.100088	0.25344	PRC1
4.85057E-07	0.05617572	1	-0.41602	0.206427	0.018957	PSF3
4.91739E-07	0.124795835	0.577843626	-0.44059	0.182681	-0.21822	HAP5

4.92764E-07	1	0.801134504	-0.5598	-0.00823	-0.16905	ROD1
5.09071E-07	0.174071202	0.904680493	0.561674	-0.21393	0.115915	SWI1
5.1163E-07	0.962059221	0.760914809	-0.33831	0.012172	0.114785	NAF1
5.15532E-07	0.384508616	0.522788552	-0.30125	0.079219	-0.16319	SUR2
5.20801E-07	0.92822386	0.817868751	0.318261	0.016071	-0.09265	YGL082W
5.21315E-07	0.630029525	0.848379499	-0.36153	0.059112	0.0945	DGK1
5.23093E-07	0.298926458	0.674519217	-0.41566	0.126568	0.175426	RPL15B
5.25353E-07	0.566626373	0.984628847	-0.3884	0.07289	-0.04417	SLC1
5.25643E-07	0.830118162	0.962684605	-0.32248	0.02852	-0.04561	ZRT3
5.26988E-07	0.996753756	0.373930465	-0.58206	-0.01153	-0.38508	YTP1
5.30263E-07	0.958689082	1	-0.56362	0.020794	0.008513	AVT5
5.33652E-07	0.251929013	0.019048423	-0.37738	0.12465	0.496031	RPL43A
5.59614E-07	0.146319766	0.668242189	-0.68178	0.269769	-0.29156	MRX5
5.60704E-07	0.156469754	0.529536924	0.41833	-0.1654	-0.22601	RFA1
5.64346E-07	0.286485784	0.244196805	0.711183	0.225216	-0.56377	FRT2
5.88884E-07	0.072758646	0.90423793	-0.4776	-0.22811	-0.09998	PRP24
6.04955E-07	0.985451395	0.984769539	1.710705	-0.04483	0.20103	None
6.05986E-07	0.52090053	0.243549962	-0.33443	-0.0685	0.267237	TRM7
6.05986E-07	0.571586601	0.868996053	0.294377	0.055271	0.071933	BCY1
6.08335E-07	0.795674553	0.868996053	-0.37123	-0.03728	-0.09026	YPK3
6.29812E-07	0.05078776	0.063075209	0.406176	0.209991	0.446331	RIE1
6.34398E-07	0.088292462	1	-0.7853	0.355853	-0.02524	DAL5
6.60254E-07	0.409635054	0.962371769	-0.50988	0.128713	-0.0732	IMP1
6.65041E-07	0.700423267	0.835951015	0.371066	-0.05177	-0.10347	CCL1
6.66242E-07	0.803085031	0.526333033	-0.37329	-0.03671	0.203175	RBG1
6.92509E-07	0.743943626	0.839890676	-0.43213	0.052819	0.118388	MTQ2
6.93938E-07	0.029527505	0.000225015	-0.2755	-0.15619	0.510181	DBP2
7.12454E-07	0.534664597	0.992350824	0.346691	0.069961	0.036696	APE1
7.2071E-07	0.102237849	0.967394603	0.512719	-0.23008	-0.07116	BOI1
7.3706E-07	0.02375103	0.623756562	-0.4588	0.267354	0.21464	None
7.3706E-07	0.712618958	0.673355127	-0.34896	-0.04696	0.149729	SOF1
7.38952E-07	0.002578481	0.841416921	-0.36571	0.270181	-0.09959	MRPL6
7.41562E-07	0.084847041	0.027801813	0.628548	-0.29761	-0.79794	YDR124W
7.53035E-07	1	0.862828907	0.3047	-0.00422	0.077283	IPP1
7.55894E-07	0.794730765	0.889649609	0.626154	0.064616	0.14056	DBP1
7.72395E-07	0.285975278	0.699105653	-0.37132	-0.11814	0.151825	RRP36
7.80756E-07	0.652629347	0.335356848	-0.33362	0.052481	0.235199	ERV14
7.85176E-07	0.761075278	0.843716147	0.499502	-0.05825	-0.13658	YBR259W
7.86199E-07	0.439669949	0.744376434	-0.41763	0.100752	0.149724	YIL161W
8.03891E-07	0.479437499	0.994376352	0.47019	-0.10664	-0.04908	ESC1
8.16417E-07	0.261791204	0.957585746	-0.29034	-0.09635	-0.04354	TAL1
8.18742E-07	0.622910776	0.427445927	-0.33247	0.056214	0.208573	RKI1
8.33175E-07	0.473677353	1	-2.80525	0.546064	0.065693	ECM23
8.39181E-07	0.179859102	0.635840497	-0.33678	0.127499	-0.15545	COQ6
8.68767E-07	0.37490782	0.974952294	0.502284	-0.13949	-0.06519	LMO1
8.73935E-07	0.37662751	0.411976149	0.556203	0.153021	-0.3562	YGR122W
8.77383E-07	0.056847963	0.515564407	0.269854	-0.13824	0.151787	DPS1
8.78295E-07	0.038009621	0.073721053	-0.49113	0.267327	0.527702	MNP1
8.8251E-07	0.327426468	0.152148466	-0.29971	-0.08916	0.279616	LCP5
8.88793E-07	0.770350536	0.812812583	-0.44042	-0.04888	-0.13139	DAT1
9.03354E-07	0.37662751	1	0.342875	-0.09457	0.031229	SEC9
9.04695E-07	0.611791537	0.328203537	0.457779	-0.08096	-0.32865	RGL1
9.04817E-07	0.133951567	0.834791774	0.316486	-0.13337	-0.08974	MET7
9.10495E-07	0.57019157	0.006535145	-0.30294	-0.05762	0.454632	RIX1

9.24671E-07	0.553921426	0.672010939	1.231819	0.243051	-0.54418	YFL019C
9.30187E-07	0.001137492	0.911946622	0.368706	-0.29623	-0.07461	YOX1
9.36135E-07	0.028455376	0.849591414	0.376196	-0.21906	0.10061	JEM1
9.38716E-07	0.091928673	0.859769494	-0.37192	0.171119	0.095781	PST2
9.46254E-07	0.252528913	0.435519854	0.42399	-0.1449	-0.26607	DPB2
9.53735E-07	0.507001261	1	0.405701	-0.08758	-0.02968	VNX1
9.72568E-07	0.995096172	1	0.28146	-0.00615	0.006438	SCT1
1.01597E-06	0.715720978	0.586189737	-0.44487	0.05982	0.224133	SME1
1.01765E-06	0.96052846	0.401097318	0.33133	-0.01249	-0.21742	SMF3
1.02471E-06	0.166697083	0.822329423	0.312583	-0.12358	0.092181	VPS1
1.0316E-06	0.028520862	0.564685666	-0.32344	0.186297	0.168653	MRPL7
1.04377E-06	0.877848777	0.571875715	-0.39963	-0.02922	-0.20563	WTM2
1.07335E-06	0.761207443	0.721913447	0.443134	-0.05216	-0.17117	PTR3
1.09231E-06	0.213721853	0.98500242	0.381215	-0.13908	-0.04434	NUS1
1.11433E-06	0.393739673	0.293969078	0.277218	-0.07438	-0.20981	SLA2
1.12369E-06	0.428998997	0.052172481	-0.28057	-0.07018	0.326439	MAK16
1.12761E-06	0.659855933	0.960144684	0.384237	-0.0609	0.058163	SET5
1.15343E-06	0.69190085	1	-3.4988	0.384195	0.114582	None
1.18309E-06	0.954188947	0.726816534	0.381511	-0.01546	-0.14613	TEA1
1.18367E-06	0.361349048	0.204980083	2.188985	-0.62843	-2.07072	YHL049C
1.18367E-06	0.946172816	0.635167376	0.463472	-0.0204	-0.21818	YKR023W
1.18937E-06	0.753616142	1	0.315543	-0.03847	-0.00794	TFG2
1.19535E-06	0.799759576	0.749124796	0.503019	-0.05175	-0.18132	YBR062C
1.21597E-06	0.34403671	0.836379632	0.288587	-0.08498	-0.08225	CDC3
1.21738E-06	0.030231646	0.699999763	0.535619	-0.31149	0.222936	BEM2
1.24687E-06	0.003590694	0.89915479	0.502547	-0.37528	0.110953	YBR238C
1.24958E-06	0.52090053	1	4.864721	-0.76727	-0.80742	YHR214C-I
1.27421E-06	0.380967903	0.081519515	-0.35465	0.097362	0.379784	HPT1
1.27787E-06	0.521735671	0.116106268	0.773288	0.164507	-0.77789	DOC1
1.27787E-06	0.122450158	0.91703897	-0.61761	0.265498	-0.12194	RCF3
1.28402E-06	0.888561341	0.780208648	-1.33567	-0.08951	0.450221	None
1.31117E-06	0.003365866	0.863509016	-0.5874	0.432576	-0.14967	SDH8
1.32775E-06	0.049766072	0.939670905	0.437666	0.233822	-0.0737	COQ3
1.33103E-06	0.045727816	0.358747602	0.489532	-0.2669	-0.34309	CLN1
1.34135E-06	0.279341459	1	-1.05059	0.338462	0.076944	RPL43A
1.35618E-06	0.030940804	0.041756428	-0.58819	0.335667	0.716341	LDB18
1.3792E-06	0.818564873	0.814212332	-0.64129	0.061182	-0.19537	ERI1
1.42351E-06	0.595637107	0.868996053	0.592299	0.11009	-0.14931	YIA6
1.43361E-06	0.767359823	0.919926509	0.368957	-0.04277	0.072559	MFT1
1.50967E-06	1.07863E-14	4.68727E-12	0.303686	0.502153	0.962463	TDH1
1.52288E-06	0.773803168	0.545272974	0.430183	0.048751	-0.23524	POR2
1.53319E-06	0.795674553	0.405149238	-0.5823	0.060325	-0.3827	CAX4
1.56685E-06	0.119099734	0.927845118	-0.34145	0.150376	-0.0632	NAT2
1.56735E-06	0.765569062	0.967394603	0.461164	0.054104	-0.06618	GIS1
1.56989E-06	2.4054E-06	0.184572953	-0.29297	-0.31849	-0.26349	FAA4
1.57277E-06	0.51219348	0.545896138	0.428253	-0.09353	-0.23442	NPR1
1.57782E-06	0.628086387	0.326601143	0.355079	-0.06161	-0.26144	RSR1
1.57782E-06	1	1	1.228048	-0.00194	-0.03637	DCV1
1.60362E-06	0.029770467	0.90453709	-2.00115	-1.10425	0.557122	None
1.62317E-06	0.815168804	0.544181296	-0.72586	-0.07041	-0.39729	CRF1
1.63383E-06	0.614870993	1	0.434048	-0.07772	0.01816	BST1
1.64627E-06	0.929614127	0.400503761	-0.34771	-0.01794	-0.23177	NRM1
1.67729E-06	0.526283651	1	0.587461	-0.12548	-0.0131	NNK1
1.68807E-06	0.98811108	0.637650576	-1.38781	0.033621	-0.6971	YBR292C

1.69839E-06	0.867434951	0.284855193	-0.33818	-0.02635	0.264108	RIO2
1.70076E-06	0.060866598	0.904680493	0.280398	-0.1454	0.060569	KTR6
1.70161E-06	0.014286971	0.708809273	0.426628	0.27783	-0.17533	PDR8
1.72938E-06	0.884827129	0.721271027	-0.45611	0.032629	0.179565	DAD3
1.73869E-06	0.692438881	0.804181925	0.334769	0.049231	-0.10577	CAP1
1.80094E-06	0.894801769	1	-0.37505	-0.02488	0.02129	YUR1
1.85477E-06	0.458468521	0.029665642	-0.80409	0.192288	-1.03434	SHH4
1.85503E-06	0.00020564	0.889649609	0.431797	0.390349	-0.10083	PIN3
1.86597E-06	0.887609886	0.599955367	-0.36552	0.025503	0.18486	ARF3
1.88167E-06	0.489250999	1	-0.60749	-0.13768	-0.02188	YEA4
1.91436E-06	0.672129195	1	0.411314	0.064018	0.009442	YIH1
1.91519E-06	0.472617197	0.008726249	0.373506	-0.08897	-0.56233	UGA3
1.97225E-06	0.839560652	0.931005601	0.316779	-0.02849	-0.05743	PTC5
2.046E-06	0.944196518	1	0.288005	-0.01313	0.019168	HSP150
2.07574E-06	0.677097244	0.889649609	0.354143	-0.05476	-0.08299	CTK3
2.09215E-06	0.034320207	0.565513961	-2.29811	1.206342	1.32595	YGL118C
2.09215E-06	0.833783236	1	0.389976	-0.03613	-0.02724	NMA1
2.11435E-06	0.00396909	0.497309894	-0.33573	-0.25136	0.199666	REF2
2.17077E-06	7.09452E-05	0.107069871	-0.35694	0.339682	0.370908	MBA1
2.19036E-06	0.774791949	1	4.901927	-0.42701	-0.45145	None
2.20592E-06	1	1	-0.42948	0.004489	-0.02447	MCH5
2.23499E-06	0.171047044	0.752502722	-0.46505	0.185429	0.169618	MRPL31
2.25714E-06	0.957720524	0.87272328	-0.45624	0.018044	-0.11456	YNL320W
2.26366E-06	0.478701236	0.844390643	0.632859	0.149821	-0.17944	SHH3
2.27121E-06	0.939749928	0.764340274	-0.30812	0.014639	0.109781	NIT3
2.31105E-06	0.912049034	0.69956533	-0.47123	0.028052	0.200201	POA1
2.33477E-06	0.84140879	0.274285823	3.274234	0.257164	-3.34297	PAU12
2.34131E-06	0.097134775	0.322929135	0.472347	0.224219	-0.35466	ATG3
2.38182E-06	0.165995342	0.207271346	0.658432	0.26939	-0.58242	PIG2
2.4747E-06	0.122042485	1	-0.43244	-0.19279	-0.02783	BUD8
2.47701E-06	0.125578299	0.571709772	-0.33095	0.145946	0.176646	PAM18
2.48863E-06	0.157055527	0.078502393	-0.45366	0.186998	-0.49841	ASK1
2.49808E-06	0.755774382	0.412531018	-0.30626	-0.0379	0.204186	DPH2
2.50702E-06	3.1416E-25	2.17479E-06	0.33597	0.746123	-0.77927	ALD4
2.54294E-06	0.899105948	0.159522721	0.296052	-0.01948	-0.28484	PCS60
2.56507E-06	0.631437679	0.888371569	1.152891	-0.204	-0.27783	MST28
2.56507E-06	1	0.917387015	-0.32842	0.002833	-0.06665	APS3
2.56618E-06	0.70191393	1	-0.32587	0.047292	-0.0255	CBP3
2.60861E-06	0.506601401	0.847404354	-1.1502	0.250917	-0.32336	YFL040W
2.66421E-06	0.413242794	0.447610866	0.347479	-0.09334	-0.2233	RAX1
2.69624E-06	0.634951173	0.916204152	-0.97971	0.166309	-0.20028	DON1
2.70218E-06	0.914039764	0.982010893	-0.3339	-0.01981	-0.04142	GIS4
2.72796E-06	0.25588535	1	-0.30814	0.107959	0.022225	COQ1
2.76486E-06	1	0.643231512	0.40308	-0.00404	-0.19375	ALG1
2.76934E-06	0.689168252	0.868031723	-0.43282	0.065007	-0.11252	SDT1
2.78236E-06	0.417307855	0.904680493	0.474012	-0.12644	-0.10474	SEC8
2.79756E-06	0.080426839	0.224910533	-0.60548	0.298278	0.524983	DAD4
2.84551E-06	0.103376814	1	0.300831	-0.14182	0.018143	NIC96
2.85734E-06	0.333861939	0.036660041	-1.56119	0.452493	2.165809	YDR157W
2.85734E-06	1	0.969393262	0.438356	0.003293	-0.06343	RAV2
2.87383E-06	0.094508839	0.021144549	-0.41853	0.199788	0.578989	NOP10
2.89693E-06	0.138285381	0.611594186	-0.35752	0.154456	-0.18007	STP3
2.90825E-06	0.062446983	0.593717896	0.357448	-0.18881	-0.18635	CLA4
2.92463E-06	0.884320839	1	-0.47412	0.03489	-0.02194	AIM22

2.93646E-06	0.444672436	0.897848413	0.488939	-0.12461	-0.11244	YLL032C
2.96963E-06	0.763546663	0.844390643	1.324703	0.160843	0.381728	IMA2
3.12854E-06	0.022548679	0.000704396	-0.55327	0.342493	-1.02217	USV1
3.16692E-06	0.981817797	0.985468267	-0.3861	-0.01148	-0.04565	ATP25
3.17434E-06	0.006764926	1	-0.62267	0.445847	-0.0531	YPR027C
3.1796E-06	0.319484149	0.35748349	0.25977	-0.0829	0.189701	SES1
3.19917E-06	0.20840105	1	0.268159	0.10258	-0.02297	PTC3
3.23099E-06	0.486326234	0.952954023	0.716151	-0.16979	-0.11696	GPB1
3.25618E-06	0.488031192	0.69579659	0.310487	-0.07307	0.13631	KTR3
3.25618E-06	0.311879301	0.913392788	-0.42365	-0.13594	0.089339	FIG2
3.27748E-06	0.676672695	1	-0.77263	-0.12001	0.066882	YGR050C
3.31718E-06	1.25272E-06	1	0.295698	-0.34121	-0.02373	SFG1
3.31751E-06	0.300891646	0.812649115	-0.39325	-0.12876	0.124604	SAS4
3.32264E-06	0.632810857	0.713395097	0.800511	-0.1414	-0.33211	ATG15
3.3306E-06	0.672577856	0.737914729	0.336528	-0.05355	-0.13051	SED5
3.45468E-06	0.054556292	0.976378097	0.442182	-0.24203	0.059837	SEC3
3.45468E-06	1	1	0.352797	0.005698	-0.02395	DBF4
3.4578E-06	0.349747954	0.876200558	-0.85948	0.255662	0.215061	YNL143C
3.46065E-06	0.663621022	0.868031723	0.613104	-0.10082	0.162261	HOT13
3.53291E-06	0.479437499	0.28677728	0.301171	-0.07241	0.242419	IES2
3.53318E-06	0.384002013	0.753347689	1.049325	-0.30506	-0.39881	YPL257W
3.62537E-06	0.94100612	0.796342002	0.398086	-0.0191	-0.13273	KEL2
3.66967E-06	0.091562555	0.771782988	-0.65603	0.316523	-0.2334	THI73
3.72351E-06	0.653413831	0.962585601	0.819736	-0.1392	0.126995	YBR196C-I
3.7494E-06	0.203043786	0.82890057	-0.4285	0.165145	-0.12998	UIP3
3.7494E-06	0.866762221	0.869100567	0.285361	0.023266	-0.07472	RPN10
3.82463E-06	0.434023315	0.789517744	0.278784	-0.07317	0.095296	NPA3
3.86838E-06	0.094415043	0.744376434	0.428795	0.20959	-0.16423	EST3
3.94468E-06	0.39604593	0.267742408	-0.5887	0.163295	0.487456	MRPL37
3.95789E-06	0.21006721	0.192811777	-0.27136	-0.10397	0.251589	URA7
3.99888E-06	0.01837786	0.953041258	-0.53456	0.344908	0.086979	MIC12
4.01571E-06	1	0.712529354	0.357091	-0.00427	-0.15	MCY1
4.02265E-06	0.001239292	0.831370236	1.136312	0.955114	-0.34386	SPG1
4.07076E-06	0.11971678	0.354608469	0.442962	-0.20532	-0.32718	CAF120
4.09208E-06	0.604739673	0.451184565	0.264155	0.050081	0.172379	YDJ1
4.09208E-06	1	0.871227843	0.410785	-0.00682	-0.10696	TAF3
4.19911E-06	0.638263859	1	0.30849	-0.05418	0.015312	SEC21
4.20565E-06	0.37662751	0.971609311	-0.37189	-0.10827	0.052989	PBY1
4.20924E-06	1.40389E-09	0.711247098	0.333731	0.46555	0.141463	SSE2
4.22134E-06	0.0122332	0.87592683	-0.6233	0.423325	0.157483	COX19
4.26141E-06	0.630769708	1	0.7864	-0.14198	0.004309	SGA1
4.27783E-06	0.603345299	0.927619939	-0.37601	0.071066	0.072766	TIF6
4.35438E-06	0.254018093	6.38095E-10	0.387455	0.139734	-1.1364	DIP5
4.39209E-06	0.04685584	0.996250082	0.309111	-0.17602	-0.0337	EUG1
4.42867E-06	0.447017626	0.863509016	-0.33418	-0.08538	0.090237	KAR4
4.43604E-06	0.675114365	0.661089241	0.245687	0.03929	0.11704	APE3
4.46524E-06	0.758077955	0.573905565	0.423794	-0.054	-0.23206	HCS1
4.49387E-06	0.167141351	0.565702828	0.983877	0.412222	-0.54288	YAT1
4.49961E-06	0.350666026	0.674877638	-0.50257	0.152426	-0.22988	ACH1
4.56473E-06	0.61766781	0.058656331	0.521237	0.096592	-0.62501	SAF1
4.58102E-06	0.653413831	0.224910533	-0.38028	0.063995	0.337618	TRX2
4.58659E-06	0.905551788	0.929199554	-0.43157	-0.02778	-0.08291	VPS30
4.60066E-06	0.07176975	0.665743457	0.313013	-0.16398	0.148092	ILS1
4.65672E-06	0.454673425	0.574102224	0.335629	-0.08564	-0.18396	TYR1

4.70425E-06	0.148485947	0.05005618	0.853262	0.372803	-1.06221	ABM1
4.7163E-06	0.270560696	0.962684605	0.373549	-0.13214	-0.05811	SWI5
4.7577E-06	0.736287769	0.689558052	-0.86949	-0.1167	0.388842	YML122C
4.81258E-06	0.801762915	0.517082803	-0.35393	-0.03788	0.211837	NCS2
4.88369E-06	0.473710488	0.264879577	0.450074	0.110653	0.3806	LSM3
4.88369E-06	0.838648058	0.807027851	-0.78906	-0.07289	0.257752	SPG3
4.98588E-06	1	0.026784925	0.369422	0.004435	-0.50877	YLR352W
5.00939E-06	0.39100906	0.339657587	0.287181	-0.08229	0.218503	THS1
5.05333E-06	1	0.871227843	-0.49379	-0.00947	-0.12901	DIB1
5.18583E-06	1	0.285732192	0.350869	-0.00371	-0.28685	VPS45
5.18795E-06	0.283359769	0.82890057	0.559579	-0.19578	0.173783	RIM13
5.30145E-06	0.267877158	0.621769539	0.50105	0.178691	-0.25567	MPO1
5.39289E-06	0.052546303	0.185487468	-0.26804	0.148677	0.2547	ATP3
5.44052E-06	0.288468769	0.721271027	0.36675	-0.12689	-0.15231	IOC3
5.5266E-06	1.16401E-07	0.384202197	-0.26047	0.326433	0.187115	CTR1
5.53415E-06	0.074432949	1	0.702501	-0.37093	0.072707	RTG1
5.55963E-06	0.927637885	0.931005601	2.050581	-0.11546	-0.39013	HSP33
5.673E-06	0.00332216	0.708167385	-0.35412	0.278942	0.153204	SWS2
5.706E-06	1	0.915496392	-2.00031	0.014925	0.463051	None
5.75676E-06	0.094685918	0.623419714	0.275271	-0.13694	0.141144	GCD11
5.86812E-06	0.835487572	0.2597044	0.410086	-0.03945	0.352345	LRS4
5.96959E-06	0.680755208	0.781154036	0.537257	0.086102	0.191852	YHI9
5.96959E-06	0.613747706	1	0.375608	0.071185	-0.0287	VPS62
6.05556E-06	1	0.986791239	-0.31206	0.000444	-0.03751	HSL7
6.06689E-06	0.665960377	1	0.343878	0.057257	-0.00622	VMS1
6.07929E-06	1	0.979069474	0.302483	0.003957	0.04004	PEX19
6.12789E-06	1	0.039570187	-0.35721	0.005236	0.471349	BMT5
6.13972E-06	1	0.377923962	0.31966	0.001832	0.234175	STM1
6.22969E-06	0.095697648	0.951428598	0.445881	-0.22249	-0.07555	SMC4
6.24846E-06	0.050398477	0.711541955	-0.33393	0.18822	0.143094	BCS1
6.28321E-06	0.899105948	1	-0.55044	0.037197	0.014156	ALF1
6.31583E-06	0.731503094	0.373162993	2.21972	-0.2955	1.889067	None
6.32578E-06	0.636133939	1	0.71675	-0.12948	-0.07097	BRN1
6.38699E-06	0.062076605	1	0.335438	0.182859	0.000793	MCP2
6.44507E-06	1.76887E-20	0.771051473	0.289331	0.60059	-0.10602	RTN2
6.47703E-06	0.529154357	0.795907717	0.723296	-0.16334	0.249978	None
6.47785E-06	0.076368853	0.919516515	0.313828	-0.16502	0.065985	GLT1
6.48127E-06	0.072476199	0.535695352	1.211837	0.642714	-0.71249	YMR206W
6.49243E-06	0.352588994	0.954183232	0.338402	-0.10548	-0.05641	SEC2
6.56354E-06	0.167183262	0.835951015	-0.39959	-0.16904	-0.12187	PIF1
6.77302E-06	0.335509445	0.844390643	-0.77338	0.243705	-0.22775	RIM4
6.77302E-06	0.762268437	1	0.77922	0.098815	0.068267	SPO74
7.01966E-06	0.079535843	0.89826709	0.258825	-0.13541	0.061556	SPN1
7.02173E-06	0.855463498	0.945585595	-0.52321	-0.04581	-0.0914	YJL193W
7.02792E-06	0.533524502	0.056523979	-0.26492	-0.0586	0.327658	MIS1
7.0555E-06	0.759694936	1	4.657841	-0.44815	-0.47352	YOL160W
7.05857E-06	5.83747E-07	0.236584307	-0.34641	0.417091	0.308951	COA1
7.09286E-06	0.09236487	1	0.463862	-0.23519	0.037266	SMC5
7.17391E-06	0.795533688	1	-4.77804	-0.35682	0.369785	None
7.2555E-06	0.920697634	0.058987073	-0.28271	-0.01666	0.346351	YTM1
7.38014E-06	0.542350614	0.752502722	-0.33216	0.072398	0.127706	ALO1
7.40801E-06	1	0.976568193	-3.30433	-0.00482	0.542369	None
7.44769E-06	0.641082995	0.703292774	0.438165	-0.07848	-0.19427	UBP2
7.60456E-06	1	0.87592683	0.346603	-0.00296	-0.09094	CUS1

8.25478E-06	3.01165E-05	0.90423793	-1.01289	1.043139	0.231923	GTO3
8.5495E-06	0.864979821	0.822329423	0.956702	0.082043	0.308863	ATG29
8.60998E-06	0.460298273	0.167222206	-0.57707	-0.14815	-0.57727	SET4
8.61632E-06	0.393339454	0.040274266	-0.29009	0.084186	0.387164	RPO26
8.68922E-06	0.132253729	0.008726249	-0.27774	-0.1281	0.446422	TRM2
8.9485E-06	0.826578265	0.871227843	-0.3663	-0.03688	0.098267	DAP2
9.0386E-06	0.595637107	0.39760733	-0.34483	0.068561	-0.24841	OPY1
9.0386E-06	0.397930773	0.708809273	0.280684	0.081458	0.124419	ERP2
9.12225E-06	0.321967781	1	-0.30193	0.099573	0.019571	PTM1
9.16072E-06	0.026572164	0.817835617	0.430724	-0.27879	-0.1415	ALK1
9.39869E-06	0.007955686	0.280831637	-0.28041	0.209054	0.237498	ATP14
9.51821E-06	0.056847963	1	-1.83254	0.93566	-0.18662	None
9.59468E-06	0.167400799	0.585629945	0.339641	0.146816	0.189425	MID2
9.61749E-06	0.684917892	0.001551953	-0.66456	-0.10627	1.233012	SPS18
9.6356E-06	0.030221222	0.522788552	0.421468	0.266116	-0.25832	YGR201C
9.82979E-06	0.055483193	0.600468279	0.352291	-0.20142	-0.19194	ACF4
1.01374E-05	0.263590333	0.119906082	-0.3132	-0.11517	-0.33933	OCH1
1.02301E-05	0.402991749	0.485259038	-0.35492	-0.10205	-0.22895	AXL1
1.02591E-05	0.99746658	0.889079564	-0.63296	0.013701	-0.15878	YPR196W
1.03037E-05	0.076342495	0.716456431	0.471496	-0.25369	0.204609	HAL5
1.03037E-05	0.055765166	0.915545807	0.526503	-0.30148	0.115876	RAD9
1.03968E-05	0.460638341	1	0.405122	0.105898	0.016354	CRT10
1.04514E-05	0.604770313	0.804576171	0.439532	-0.08722	-0.14991	YRR1
1.04724E-05	0.735543796	0.328203537	-0.34116	0.047885	0.27094	YPD1
1.05E-05	0.33363674	0.865605555	0.487676	-0.16078	-0.13625	SGF73
1.07118E-05	0.637757348	1	-0.31749	0.057755	0.031239	SNL1
1.0758E-05	0.938970104	0.755878175	-0.34841	0.017846	0.135408	RCL1
1.07656E-05	0.440961232	0.116009386	-0.25035	-0.06741	0.275064	PXR1
1.07731E-05	0.868862376	0.721655021	0.371123	0.03136	-0.15806	SWC4
1.08181E-05	0.399977002	0.918382276	0.399024	-0.11654	-0.0863	DSL1
1.08841E-05	0.009676844	0.567844831	-0.28735	-0.21256	0.164948	DRS1
1.09407E-05	0.194184425	0.935686072	-0.3862	-0.15953	0.0732	YSR3
1.10228E-05	0.113344883	0.812812583	0.802573	-0.39737	0.270071	SAE2
1.10533E-05	0.944196518	0.803017727	-2.06149	0.093646	0.738043	None
1.1076E-05	0.096641035	0.983370642	-0.40746	0.205199	-0.05306	SPC1
1.11083E-05	0.051155386	0.246416963	-0.40563	0.233398	0.362919	MRP10
1.11252E-05	0.980380105	1	0.510406	-0.0166	0.050267	CDC15
1.11713E-05	0.897637238	0.868427687	0.304378	-0.02158	0.0846	SOK1
1.13463E-05	0.261791204	0.740020932	0.731315	0.273291	-0.29666	RTC2
1.16463E-05	0.905759808	0.992792463	-0.28987	0.019459	-0.03416	GZF3
1.17318E-05	0.250649618	0.187679572	-0.60696	0.227155	-0.58902	YOR062C
1.17723E-05	0.847371083	0.011005734	0.61658	0.057769	-0.97401	USB1
1.18131E-05	0.001677606	0.043873822	-0.36667	0.314039	0.485874	YDR391C
1.18706E-05	0.736287769	0.94082376	-0.39523	0.055609	-0.07233	POP7
1.19491E-05	0.00332216	0.069148732	0.300596	0.246872	-0.36493	CWP1
1.21215E-05	0.533524502	0.741851259	-0.29763	-0.06747	0.120647	SEN1
1.21246E-05	0.898885595	0.681411478	0.518125	0.036723	-0.24647	VPS71
1.24963E-05	0.128591907	0.944495296	-0.52734	0.247026	0.094917	PET117
1.2517E-05	0.019167121	0.804576171	-0.54505	0.368171	0.186023	SDH6
1.25312E-05	0.026262642	0.336989777	-0.3219	-0.21006	0.254723	UTP22
1.26207E-05	0.335509445	1	0.37992	-0.12573	0.028881	ECM3
1.27242E-05	0.080514417	0.907733056	0.419484	-0.22508	-0.09699	APL2
1.29085E-05	0.558891569	1	0.343393	-0.07547	0.011125	TAO3
1.29106E-05	3.06854E-05	0.054247253	-0.36692	0.392312	0.469189	CMC1

1.30395E-05	0.397361658	0.98500242	0.397389	-0.11794	0.051131	SWI3
1.32776E-05	0.305163905	0.726305499	0.504599	0.175646	-0.2147	ATG7
1.33319E-05	0.387305876	0.526082458	0.746296	0.225189	-0.46081	ICL2
1.34754E-05	0.860955797	0.94748087	0.257628	0.022809	0.04632	LSM1
1.37021E-05	0.125297164	1	0.264824	-0.12743	0.016242	CDC12
1.39631E-05	0.140156874	0.616202953	0.453758	-0.21215	-0.24481	MBP1
1.4055E-05	0.799671207	0.758049402	-0.33783	-0.0383	-0.13228	MSG5
1.42441E-05	0.16730854	1	-0.31408	0.137307	-0.01054	NCE102
1.43328E-05	0.486326234	0.476983582	0.540486	0.136556	-0.36004	GAL4
1.43441E-05	0.312951757	0.95424274	0.431706	-0.14937	-0.07463	KAR3
1.4356E-05	0.782040669	1	-4.8099	-0.40849	0.426145	None
1.45037E-05	0.046903645	0.096995994	-0.68173	-0.40569	-0.79258	AAR2
1.45037E-05	0.345520596	0.192775793	-0.32232	0.104384	0.3167	RPL14B
1.45514E-05	0.758146893	0.818523526	0.51723	0.069329	-0.17247	YJL070C
1.47378E-05	0.64265761	0.84419011	-0.30552	-0.05587	-0.09387	FUS3
1.50102E-05	0.860071452	0.614405454	-0.29873	-0.02653	0.161794	SOD2
1.5117E-05	0.838648058	0.882626496	-0.27865	0.027306	-0.07329	RBA50
1.52154E-05	0.591426516	0.909303521	0.283878	0.058756	-0.06564	MTR2
1.54705E-05	0.613678519	1	-1.3892	-0.26533	0.053379	DIT2
1.55156E-05	0.130061341	0.367293941	-0.42689	0.201844	0.328513	YAH1
1.55546E-05	0.795196386	0.697639077	0.473461	0.055343	-0.22163	OKP1
1.5577E-05	0.583819577	1	-0.34849	-0.07287	0.000252	NNF2
1.59071E-05	0.049421861	0.154422599	-0.52708	0.309409	-0.5523	CUP2
1.61493E-05	0.276167395	0.5216494	0.469089	0.173808	-0.29553	ATG2
1.69179E-05	0.69799932	1	0.426666	-0.06857	0.02701	MNR2
1.70305E-05	0.0122332	0.021809306	0.26055	-0.19274	0.391688	ERO1
1.71715E-05	0.384508616	1	-0.34201	0.104126	0.004112	RSM22
1.71774E-05	1	1	-0.45321	-0.00818	-0.04132	DIA4
1.73506E-05	0.542275289	0.948505707	0.365439	-0.08376	-0.06617	YLR278C
1.73579E-05	0.037207475	0.927845118	-0.31772	0.198008	0.065309	UCC1
1.74719E-05	0.416507101	1	5.231342	-1.36866	-1.51321	None
1.74748E-05	0.002244225	0.853367097	-0.29396	-0.25306	-0.08781	SKG6
1.74751E-05	0.070677749	0.889649609	0.445344	0.248143	0.114423	RMR1
1.7576E-05	0.000684897	0.044540629	-0.22276	0.206871	0.300733	ATP2
1.78444E-05	0.455815867	0.197874004	-0.31522	0.084462	0.31094	APT1
1.78877E-05	0.196724669	0.939670905	-0.26158	-0.11019	0.049202	MRS6
1.80114E-05	0.340284679	0.929303164	0.331199	0.110217	-0.06794	TPS3
1.8032E-05	0.711405843	0.875200775	0.354909	-0.05546	0.097646	YPL088W
1.83513E-05	0.820407495	0.875200775	-0.36734	-0.03925	-0.10064	COM2
1.87946E-05	0.032155503	0.943528578	0.360947	-0.2342	-0.06707	TOS4
1.90724E-05	0.0122332	0.378603211	0.372791	-0.27767	-0.28641	VTC5
1.90724E-05	0.968013408	0.743726892	0.49034	-0.01947	-0.20268	YHR131C
1.91168E-05	0.692723579	0.021370619	-0.28709	0.04659	0.433225	TAN1
1.9547E-05	0.995919167	0.710318897	-3.66073	0.065447	-2.00911	YFL064C
2.01204E-05	0.712206218	0.962684605	-0.35108	0.054507	0.058021	COX11
2.04816E-05	0.0122332	9.20876E-05	0.41268	0.305969	-0.91038	TMC1
2.06179E-05	0.877996534	0.05801516	0.293825	-0.02463	0.382164	NOP13
2.06179E-05	0.981817797	0.419798397	-0.34099	-0.01104	0.247944	BCD1
2.07646E-05	0.355434025	0.617460449	-0.24685	0.080183	0.134852	SEC53
2.09882E-05	0.896286063	0.996250082	-0.28416	0.020823	-0.03316	GCN3
2.11389E-05	0.700418476	0.904680493	1.753098	-0.2857	0.427664	NDJ1
2.14545E-05	0.795533688	0.438612707	0.582564	-0.06913	-0.41656	CLB6
2.14885E-05	0.910359943	0.241434728	-0.27018	-0.01807	0.252455	MIC60
2.14885E-05	0.321967781	1	0.255888	-0.08869	0.021844	KAP104

2.17739E-05	0.627561248	0.501759351	0.391683	0.076218	0.257946	HAP4
2.18896E-05	0.001674292	0.990299674	-2.39008	1.884231	-0.51878	DAL80
2.19224E-05	0.347668023	0.641769073	-0.90672	-0.29702	0.48124	None
2.21978E-05	1	0.973674102	-0.53807	-0.00265	0.080404	YPL108W
2.22479E-05	0.001307365	0.112076435	-0.25397	0.22888	0.291207	TUF1
2.25755E-05	0.02963386	0.075616863	-0.62245	0.405063	0.764134	ATX1
2.27047E-05	1	1	0.264636	-0.00523	-0.02296	HRI1
2.30511E-05	0.143341906	1	0.391521	-0.18643	0.021132	SWI6
2.31355E-05	0.806893372	0.924605282	-0.28217	0.031824	0.060559	RRT2
2.32217E-05	0.370406831	0.068005273	-0.28202	-0.08992	0.355723	NOP53
2.32876E-05	0.296901865	0.889079564	0.34526	0.125118	-0.09072	YDR476C
2.34369E-05	0.460638341	0.390677789	-0.30931	-0.08354	-0.23513	MET4
2.34843E-05	0.209965516	0.020319939	0.314032	0.131331	-0.48074	YAP1801
2.38658E-05	0.613304251	0.744307428	0.400771	0.081162	-0.16724	YIL152W
2.3883E-05	0.439851325	0.937984736	-0.32122	0.089993	-0.06162	HTZ1
2.42298E-05	0.968013408	0.737036218	-1.47324	0.056066	-0.66432	None
2.49031E-05	0.086191139	1	0.285723	-0.15564	-0.02751	SYH1
2.56448E-05	0.910392982	0.886876142	0.411698	-0.02797	-0.11014	PAN3
2.57026E-05	0.885372644	0.46824606	-0.30442	0.024377	0.211028	IPI1
2.57109E-05	0.170043734	0.967394603	0.560507	-0.25447	0.090918	RAD26
2.59273E-05	0.776193997	0.711541955	0.555302	-0.07116	0.256194	SLO1
2.64E-05	0.202764822	1	-4.54766	1.316302	-1.37551	YLR157W-[illegible]
2.64628E-05	0.265919992	1	-1.50244	0.550031	0.030266	ERR3
2.65461E-05	0.125719569	0.97750026	0.372853	0.18464	-0.05404	STE50
2.65596E-05	0.954336914	1	0.320287	0.01481	-0.01625	UBC7
2.68074E-05	0.126331092	0.385959394	0.323064	0.159789	-0.24896	BXI1
2.71949E-05	0.154663593	0.759373966	-0.78381	0.359394	0.314239	OAR1
2.73581E-05	0.006993128	0.079491698	-0.262	-0.20928	0.323674	TPA1
2.75314E-05	0.222579714	0.817868751	-0.27522	-0.11327	-0.09463	SVS1
2.77157E-05	0.388076484	0.562048326	-0.69282	0.212537	0.418989	SUL1
2.78395E-05	0.28254384	0.491045055	0.466548	-0.17674	-0.31539	SMP1
2.87066E-05	0.223628637	0.972387712	-0.45412	-0.18663	0.069501	NTE1
2.88608E-05	0.138144089	0.702041373	2.73577	-1.28058	-1.69526	YRF1-7
2.90117E-05	0.000190923	1	4.581553	4.640587	5.609004	COX3
2.90583E-05	0.762964507	0.439177977	0.395445	0.053359	-0.28673	SLD5
2.90583E-05	0.767459399	0.558164588	-0.25876	0.033998	0.158402	MEU1
2.90583E-05	0.159373293	0.761176355	0.432154	0.200758	-0.17457	PEP7
2.90583E-05	0.54489641	1	0.500313	-0.11717	-0.0277	SEG2
2.94013E-05	1	0.749124796	-0.32981	0.003474	0.136627	PUF4
2.95316E-05	0.834640482	0.689558052	0.280515	0.029192	-0.13752	AGE2
2.96512E-05	0.893976085	0.178503269	1.412894	-0.10957	-1.50954	None
3.00846E-05	0.031788614	0.786892522	0.34458	-0.22949	-0.13044	DUG2
3.00984E-05	0.47612507	1	0.425862	0.113796	-0.02994	MED11
3.09264E-05	0.269333208	0.143940462	0.264598	0.102084	0.293143	MGE1
3.14137E-05	0.079535843	0.847404354	-0.26075	-0.14571	0.081827	ACC1
3.1645E-05	0.398707313	0.708493888	-2.61621	0.664009	1.368774	None
3.16695E-05	0.206911234	1	-0.27411	0.116334	0.021556	MGM101
3.21091E-05	0.841225558	0.742768539	0.639885	-0.06516	-0.27246	HXT5
3.24551E-05	0.832102442	0.659843375	-0.4318	0.045144	-0.22506	YDL180W
3.26122E-05	0.501017244	0.915496392	-0.26188	-0.06639	0.060744	UTP15
3.26122E-05	0.940649911	1	-0.47887	0.025323	0.017569	LIH1
3.27283E-05	0.203402724	0.922669797	-0.48377	-0.20712	0.10747	RRP17
3.28102E-05	0.619450596	0.522217116	-1.98141	-0.37921	-1.41082	SPR2
3.31779E-05	0.509317322	0.98500242	0.422563	-0.10656	-0.05665	SGS1

3.38946E-05	0.709900242	0.376455642	0.339509	-0.05497	-0.26927	VPS5
3.40678E-05	0.295390559	0.927963214	-0.37922	0.139567	0.080481	CLG1
3.46228E-05	0.295659	0.563376575	-2.24172	0.758612	1.444533	None
3.48836E-05	5.76932E-06	0.868996053	-0.30727	0.368827	0.089272	MRP49
3.51256E-05	0.865084571	0.592065914	-0.35756	-0.03237	0.20937	ASF1
3.57078E-05	0.420761548	0.8483833	-0.39268	-0.11623	0.123304	HLR1
3.57765E-05	0.097990915	0.169011865	0.631579	0.339427	-0.67127	YNL018C
3.58281E-05	0.773263368	0.062663543	-0.44283	0.057462	-0.57686	RAD17
3.59719E-05	0.905759808	0.073415554	-0.44555	0.031296	-0.55989	TPO4
3.60591E-05	0.820966378	0.568843524	-1.17837	0.126904	0.717462	ARR2
3.61621E-05	0.231604832	0.786137812	0.444076	-0.18449	-0.17058	YPT11
3.63174E-05	0.678331025	0.801754205	-0.35164	0.061156	0.128217	RER1
3.65929E-05	0.382913033	0.56134476	-0.79292	-0.25083	0.497046	SPS19
3.67235E-05	0.111956571	0.891157181	-2.89532	-1.31562	1.164243	YAR066W
3.6872E-05	0.526984154	0.72738114	-0.31686	-0.07699	0.141249	TMA23
3.69754E-05	0.622084537	0.87592683	0.435474	-0.08824	0.123218	IRC8
3.71394E-05	0.806085641	0.041689887	0.427225	-0.05008	-0.60715	RRD2
3.71835E-05	0.266929182	0.202387133	0.37655	-0.14798	0.384987	CLB2
3.81195E-05	0.133002584	0.967394603	-0.3379	-0.16734	-0.05594	BMT6
3.85213E-05	0.003939254	1	4.435893	-4.1407	-5.37821	YPR202W
3.85785E-05	0.36891213	0.705564376	0.350389	-0.11585	-0.16777	COG4
3.86282E-05	0.080514417	0.446640364	0.449985	-0.25559	-0.32785	ATG13
3.88883E-05	0.621279638	0.499488496	-1.71965	-0.334	-1.2548	YCR085W
3.92882E-05	0.647503232	0.736753793	0.259101	0.049515	0.113631	TOA1
3.95346E-05	0.929392974	0.713395097	-0.28288	-0.01695	0.131805	BRE4
3.95481E-05	0.601349273	0.581522652	-0.46039	0.096932	0.275291	OST4
3.97452E-05	0.564303748	0.905370228	0.293	0.067277	-0.07269	HIS3
3.98037E-05	0.030733392	0.570313772	-0.98194	0.646874	0.593282	PRM2
3.9997E-05	0.00028014	0.630792731	-0.3422	0.347819	0.189075	MRPL27
4.00701E-05	0.058451856	0.8119342	0.300008	-0.18166	-0.10759	TDA7
4.01763E-05	0.665960377	1	0.470429	0.085897	0.021771	TDA2
4.02253E-05	0.091562555	0.013045788	-0.34864	0.19008	0.578961	RPL37A
4.02327E-05	0.746350726	0.659101564	-0.27604	0.040227	0.146175	SKG1
4.13519E-05	0.591976174	1	-1.12992	0.238024	0.030361	YLR285C-A
4.16111E-05	0.468941828	0.834791774	-0.40962	0.111907	0.13741	YJR112W-A
4.19566E-05	0.676177191	0.900882736	0.263036	-0.04683	0.067605	GPI16
4.2135E-05	0.855524783	0.670242692	-0.36328	0.034745	0.187981	YCR090C
4.21662E-05	0.700788611	0.677620076	0.274303	0.045824	-0.14014	YRB2
4.25831E-05	0.760915703	0.262505363	-0.45072	0.062087	0.422613	CNL1
4.26996E-05	0.897122859	0.060300641	0.310073	0.023628	-0.41232	HSE1
4.32961E-05	0.037161407	0.910496008	0.323517	-0.21381	0.078438	SEC24
4.34195E-05	0.407136042	0.507578381	0.292576	-0.09052	0.198049	DUS3
4.42111E-05	0.75339767	0.92806956	0.245206	0.035274	-0.05295	PRE7
4.4425E-05	0.740307204	0.378557332	1.052907	-0.16009	-0.85311	None
4.44336E-05	0.552553606	0.643429399	-0.23933	-0.05617	0.130657	SWP1
4.45394E-05	0.004780956	0.21536472	-0.3398	0.286222	0.341968	TIM11
4.49244E-05	0.269333208	1	-4.47193	1.205944	-1.26202	GEX2
4.59193E-05	0.583819577	1	0.368236	-0.08207	0.042701	EPO1
4.60285E-05	0.038719477	1	0.328485	-0.21662	-0.02921	FLC3
4.64264E-05	1	0.803025975	-0.26907	0.003346	0.098999	KRE1
4.695E-05	0.954188947	0.915496392	-0.58542	-0.02772	-0.13862	THI22
4.7166E-05	4.53297E-09	0.136494495	-0.23895	0.362123	0.272746	ADH4
4.73763E-05	0.920697634	0.908854398	-0.35439	0.022887	0.08678	TAD1
4.74811E-05	0.021955716	0.203504846	-0.64128	0.453448	0.656083	XYL2

4.80109E-05	0.000213631	0.119906082	-0.43459	0.451616	0.509081	COX17
4.8685E-05	0.646727695	1	-0.32819	-0.06317	-0.02551	PER33
4.86934E-05	0.837624198	0.530280555	-0.54649	-0.0569	-0.35835	FIG4
4.87031E-05	0.660417863	0.226565606	0.255578	0.047845	-0.2545	LSB5
4.87923E-05	0.700788611	0.907320483	0.385502	-0.06507	0.095642	SQS1
4.88924E-05	0.699326009	0.106061312	0.699939	-0.11928	-0.84306	MCM10
4.93604E-05	0.466557694	0.005401573	-0.33158	-0.09213	0.607338	DHR2
4.93787E-05	0.101928042	1	0.545298	0.295082	-0.04849	GSM1
4.93787E-05	0.342526061	1	-1.15291	-0.39585	0.063833	TEP1
4.95313E-05	0.386627735	0.878688605	0.544051	0.17563	0.154816	ITT1
4.96098E-05	1.05959E-06	0.652552516	-0.29703	0.386337	0.160363	MRPL10
4.96098E-05	0.725013125	1	0.270313	0.042736	-0.02231	RPN9
5.01349E-05	0.001731932	1	0.544917	-0.51054	-0.039	SPT5
5.01611E-05	0.557460822	0.28665746	-0.22616	-0.05304	0.206088	MNN10
5.04407E-05	0.193877369	0.000749239	-0.23053	-0.10306	0.486167	RPF1
5.08932E-05	0.125946646	0.125525495	-0.3348	0.170144	0.390316	YNL122C
5.18825E-05	0.843151545	0.929344228	0.265691	0.027192	0.057206	PRE9
5.23403E-05	0.516700017	1	0.422307	-0.10745	0.037569	SWR1
5.24976E-05	0.528882079	0.951213999	-0.99614	0.239457	0.188274	ADY2
5.27321E-05	0.450549647	0.947971364	0.315904	-0.09118	-0.0607	SEC18
5.28806E-05	0.416010682	0.98500242	0.365777	-0.11243	-0.05045	TIP20
5.36566E-05	0.658210054	1	-0.31629	-0.05958	-0.02336	PHS1
5.40915E-05	0.010256893	0.863490318	-0.42491	-0.33894	0.129632	SDD4
5.41159E-05	0.113100688	0.131907273	0.274155	0.145339	-0.31766	YAP1802
5.41209E-05	0.709900242	1	0.30375	0.050349	0.008814	END3
5.4426E-05	0.013199729	0.715152314	0.428476	-0.33311	0.203314	TIF4631
5.51692E-05	0.842773453	0.711541955	0.296103	-0.03063	0.141974	YML096W
5.56263E-05	0.029490195	0.771051473	0.295702	0.204997	-0.12068	RIM11
5.58165E-05	0.529464272	1	0.391108	-0.09745	0.035417	SAP155
5.68967E-05	0.274405007	0.509579361	0.476564	-0.18966	-0.32536	PEA2
5.79844E-05	0.099649048	1	0.360119	-0.19827	-0.00058	CHS2
5.81692E-05	0.84140879	0.043538983	-0.49811	0.051417	-0.70799	ICT1
5.88414E-05	0.044150726	0.000115519	-0.33742	0.217531	0.786369	RPS30A
6.13673E-05	0.85453023	0.719739519	-0.9056	0.087951	-0.42273	NAT4
6.15911E-05	0.877653097	0.244196805	0.314918	-0.0281	-0.30933	HRD1
6.17877E-05	0.001527386	0.786584362	-0.29143	-0.27551	0.114398	ERC1
6.20711E-05	1.55085E-09	0.616944383	-0.60124	0.935835	-0.346	YGR066C
6.22593E-05	0.289155055	0.665743457	0.37461	-0.14603	-0.20088	DUN1
6.22593E-05	0.027086984	0.772424036	-0.28985	-0.20444	0.118311	NUP170
6.24986E-05	0.762964507	0.781154036	-0.43706	-0.06085	0.17469	CWC22
6.38197E-05	0.11971678	0.594195804	0.428454	-0.22707	-0.25898	PHO2
6.38455E-05	0.408733697	1	0.332596	0.104686	-0.02974	VPS51
6.45957E-05	0.940918832	0.000551855	-0.2205	0.012101	0.47944	NMD3
6.49236E-05	0.882399123	0.786584362	0.374597	-0.03269	-0.14787	TCO89
6.51112E-05	0.048714737	1	0.252794	-0.16298	0.019122	ENT1
6.5846E-05	0.166666159	0.978193638	0.322461	-0.15467	0.048773	SED4
6.61633E-05	0.049774859	0.723935587	0.255177	0.163312	0.118704	SMT3
6.661E-05	1	0.90423793	-0.26062	-0.0011	0.067295	ROT1
6.74029E-05	0.162783034	1	0.288855	-0.13967	0.005381	EDC3
6.79262E-05	0.519321422	0.897848413	0.357855	-0.09181	-0.09568	VPS54
6.79486E-05	0.640053214	0.059418296	-0.35603	-0.07084	-0.4855	FDO1
6.87588E-05	0.444456702	0.217184527	0.303414	0.089719	-0.31249	TRP4
6.87911E-05	0.21659995	0.883095126	0.389199	-0.17127	-0.11106	YTA6
6.89393E-05	0.440961232	0.771931968	0.388086	-0.11599	0.160332	SRB4

6.91573E-05	0.08272612	0.328203537	-0.40037	0.229755	0.349982	SRB7
6.96161E-05	0.001784516	0.223097491	0.291737	-0.27655	-0.29847	YOL019W
7.04584E-05	0.652629347	0.919926509	-0.34009	0.065765	0.079293	PPH3
7.04802E-05	0.013695435	0.962355326	-0.62463	0.477858	0.110998	GAS2
7.11529E-05	0.890649696	0.537223328	-0.2832	-0.02305	-0.18725	PSD1
7.1268E-05	0.996753756	0.622534051	0.306834	0.007662	-0.17826	RME1
7.20563E-05	0.867488635	0.506928274	0.473147	-0.04447	-0.32934	YOR292C
7.33221E-05	0.851320842	0.803017727	0.369106	0.037356	-0.13979	IMP2'
7.33434E-05	0.127578663	0.00166885	-0.30614	-0.15892	0.62403	UTP20
7.3502E-05	0.512975922	1	-4.49391	-0.84859	0.889266	YLR464W
7.44518E-05	0.156469754	0.025085508	-0.25009	-0.12256	0.397219	IMP4
7.49667E-05	0.346555952	1	-0.34845	-0.1231	-0.00743	PMT7
7.56141E-05	0.571690974	0.962371769	-0.37841	0.087762	-0.06747	SGF11
7.6269E-05	0.860071452	0.90453709	0.287823	0.028063	0.075019	RFC2
7.67491E-05	0.032544156	0.282780148	-0.29755	0.204895	0.27986	MRPL50
7.78857E-05	0.464410394	0.847404354	-0.27982	-0.07999	0.092158	SRP40
7.80678E-05	0.086191139	0.538039737	-0.36469	0.208819	0.242299	CUE4
7.90971E-05	0.171035344	0.427445927	0.252563	-0.12114	0.19674	MKC7
7.91859E-05	0.384327883	0.017068248	-0.39284	0.129803	0.657845	ECM1
7.97635E-05	0.371129486	0.903248727	-0.46455	0.15721	-0.12133	MTO1
8.02776E-05	0.688973799	0.214464369	-1.89408	-0.30995	2.164303	None
8.08008E-05	0.138509482	0.069129419	-1.71672	0.802196	2.724821	YBL044W
8.08496E-05	0.631219113	0.769354841	1.240344	0.257564	-0.52018	PAU5
8.09892E-05	0.062184314	1	0.269204	-0.16721	-0.00793	NUP60
8.09892E-05	0.829983598	1	0.339828	-0.03815	0.008505	RIM20
8.19297E-05	0.617873557	0.378557332	0.250192	0.053311	0.208445	RPL18A
8.19297E-05	0.206402515	0.532461006	-0.28906	-0.12969	0.194785	RSA4
8.20013E-05	0.35578661	1	0.422644	-0.14904	0.010819	SNT1
8.26336E-05	0.985451395	0.513475116	0.342725	-0.0113	-0.23787	ISY1
8.31295E-05	0.640183171	0.529437087	-0.26404	-0.05316	0.179032	SQT1
8.36843E-05	0.776818407	0.976568193	-0.30612	0.041188	-0.04753	NCR1
8.51021E-05	0.219208972	0.848379499	0.265345	-0.11742	-0.0878	SHE10
8.64995E-05	1	1	-0.3382	0.000546	-0.03349	NSE5
8.72545E-05	0.033215596	0.967394603	0.346125	-0.24238	-0.06026	LCB4
8.8611E-05	0.901660388	0.032905196	-0.2548	0.019495	0.393833	NSR1
8.89548E-05	0.298926458	1	0.428954	-0.16736	-0.01746	PEP5
8.9589E-05	0.738978817	0.237658563	0.350693	-0.05498	-0.35639	CKI1
8.98364E-05	0.535888712	0.539256261	-0.26123	-0.06553	-0.1744	DPL1
9.01982E-05	1	0.318881364	-0.25384	0.003536	0.229353	MRN1
9.06588E-05	0.301940839	0.063075209	0.299867	-0.1165	-0.41291	SLX5
9.08012E-05	0.759733432	1	-0.54594	0.078594	-0.00215	CRG1
9.09616E-05	0.99746658	0.885798515	-0.27158	0.006649	-0.07783	MRS3
9.13664E-05	0.490088889	0.723053672	-0.27718	0.075849	0.131096	YBR053C
9.22611E-05	0.375026386	0.304859152	-0.34312	-0.11682	0.315701	TRM732
9.22662E-05	1	0.939670905	0.322338	-0.00594	-0.06645	TIP41
9.35818E-05	1	1	0.348237	0.00679	0.010888	YIL067C
9.5975E-05	0.444779801	1	-0.50204	0.149444	0.022504	YBL059W
9.68441E-05	0.753616142	0.215961573	-0.29782	0.044366	-0.31232	YNL115C
9.83649E-05	0.661521318	0.283510967	-0.42581	0.082002	-0.40338	FYV6
9.87571E-05	0.963895677	0.962145993	-0.29674	0.013309	-0.05412	YMD8
9.88388E-05	0.697921391	0.221570136	-0.26169	-0.04594	0.273795	TRM8
9.91647E-05	0.920741029	0.915496392	0.269345	-0.01826	0.066807	PBR1
0.000100663	0.587866346	0.081922572	-0.43175	-0.09875	-0.5677	NSL1
0.000101657	1	0.74594446	-0.97612	-0.01915	0.439352	ARP10

0.00010229	0.055381621	0.123468015	0.948382	0.609673	-1.14493	BAG7
0.000103679	0.781305378	0.099020366	-0.26496	-0.03571	-0.33693	FET4
0.000105383	0.179113779	0.384604173	1.248383	0.595764	-1.06788	YNL277W-A
0.000106814	0.070226111	0.781154036	-0.45871	0.278689	0.18844	ELC1
0.000107153	0.703189392	0.530625906	0.647579	-0.114	-0.44409	RRI2
0.000108204	0.963749362	0.569177865	-0.70249	0.031475	-0.45611	RRG8
0.000108377	0.001489983	0.554003774	-0.3797	0.368126	0.250732	MIM2
0.000109776	0.361924883	0.804939823	-0.49689	0.173856	-0.19037	YJL118W
0.000110669	0.054782399	0.996250082	-0.31639	0.203014	-0.04036	AIM25
0.000112341	0.675724591	1	-0.30638	-0.05733	0.017844	RRN7
0.000112369	0.101132655	0.214508513	0.483883	0.274614	-0.51345	GLG1
0.000112866	0.039547582	0.878688605	0.29246	-0.20202	-0.08722	CDC9
0.000113657	0.98811108	0.636714322	3.458176	0.0879	-2.03015	YER189W
0.000115303	0.088953553	0.533231335	-0.38729	0.225255	0.265253	BSC2
0.000115432	0.360240483	0.029665642	-0.27179	0.096139	0.432722	RPL27B
0.000115973	0.75340855	0.535269963	-0.25103	-0.03792	0.17177	CBR1
0.000117625	0.758146893	1	0.251424	0.037583	-0.02397	CAP2
0.000118088	0.88355725	0.555522959	-0.38647	0.034368	-0.25586	ADA2
0.000118248	0.062193215	1	0.324209	-0.20613	-0.03834	CPR7
0.000118312	0.890649696	0.902712809	-0.48264	0.040292	-0.12966	PEX32
0.000118591	0.209498092	0.781875866	-0.33844	0.153658	-0.13939	SFK1
0.000118822	0.598532156	0.571875715	0.251551	-0.0573	-0.16293	YOL057W
0.000119907	0.214175707	0.567844831	0.321842	-0.14656	0.210739	MMR1
0.000120336	0.000616542	0.428151931	-0.34126	0.353283	0.270299	IMG2
0.000120672	0.826578265	1	-1.20636	0.135615	0.032228	YOR314W
0.000120931	0.386354047	0.243549962	0.576208	-0.19708	-0.58942	SNX41
0.000121294	0.713873054	0.482770834	0.470907	0.080873	-0.34882	GIP4
0.000121294	0.002518787	1	-0.44121	0.414171	-0.02588	TVP15
0.000121473	0.114955379	0.020319939	-0.29558	0.162245	0.496025	RPL40B
0.000122901	0.022170199	1	-1.37548	0.976304	0.163012	None
0.000123548	0.715720978	1	-0.24716	-0.04199	0.02343	SLY41
0.000125647	0.707673932	0.929633058	-0.27808	-0.0484	0.062563	YKL063C
0.000126056	0.003877357	1	1.247972	-1.20719	-0.07331	None
0.000127573	0.024218523	1	0.375026	-0.28247	-0.02354	SFB2
0.000127993	0.939749928	0.929035521	-0.36172	0.020944	-0.0824	APC11
0.000128684	1	0.558528737	-0.34526	0.002164	-0.22921	GPI1
0.000128791	0.321712563	0.670294084	0.713577	0.273826	0.394777	YOR293C-A
0.000128931	0.402010292	0.726816534	-0.35431	-0.11686	-0.17019	BIG1
0.000134368	0.056603162	0.931918778	-0.34169	0.220253	-0.07485	PEX4
0.000134696	0.521582459	0.822329423	0.340784	-0.09057	-0.12681	SPT7
0.000135193	0.002342	0.930039869	-0.5013	0.474952	-0.1126	CMC4
0.00013732	0.900344125	0.197874004	-0.26764	-0.02117	-0.29494	HIS2
0.000137815	0.381873848	0.498203091	0.404816	-0.14086	-0.29557	MCH1
0.000137815	0.953675948	0.937179795	0.242997	0.012476	0.052294	ASP1
0.000144372	0.407136042	0.714876394	0.267039	0.088284	0.13411	HCH1
0.000144632	0.628092366	0.69956533	0.408184	0.088162	-0.21366	PEX28
0.000145012	0.75339767	0.978193638	-0.33708	0.051555	0.053053	GON7
0.000145526	0.001730034	1	0.47931	0.472753	0.023994	FRE3
0.000146949	0.712618958	0.592141711	0.528453	0.092321	-0.336	TDA1
0.000146949	0.767194808	0.911946622	-0.28931	-0.04177	0.074365	GET4
0.000147691	0.753399338	0.321715999	-0.21117	0.032373	0.195524	BGL2
0.000148837	0.98811108	1	-0.32814	-0.01037	0.027119	FOL3
0.000150058	0.096921872	1	0.278956	-0.16341	-0.02884	POB3
0.000150124	0.492928432	0.960632458	0.412456	-0.11653	0.078572	CYR1

0.000150171	0.531626008	0.772424036	0.742762	0.195272	-0.31891	IRC10
0.000151727	0.194950123	0.599983173	-0.21943	-0.10438	-0.13793	RAS1
0.000152041	0.997790364	0.929303164	-0.3525	-0.00871	0.080559	DAS2
0.000153683	0.240878476	0.971609311	0.419	-0.18604	-0.07284	MSH3
0.00015444	0.767134842	0.722642147	-0.3407	-0.04936	-0.16582	ADE4
0.000155018	0.000137589	0.834537655	-0.27574	0.314823	-0.09975	YSC84
0.00015634	0.612454623	0.757681998	0.381557	-0.08627	-0.1712	MSA2
0.000156427	0.853297263	0.994517386	-0.74157	0.076289	0.09986	None
0.000157312	0.938475636	0.834791774	0.340331	0.020552	-0.12343	HIS6
0.000157583	0.230929934	0.62113714	0.408318	-0.18482	0.250252	PNT1
0.000158309	0.451996377	0.268615319	-1.07272	0.318853	1.082158	None
0.00015998	0.091562555	0.002316113	-0.25071	0.148278	0.523444	RPS10B
0.000160558	0.897738563	0.996250082	-0.54693	0.044183	-0.07192	BUD31
0.000162623	0.039001566	0.449106932	0.311688	-0.22059	0.246627	FUN12
0.000163227	0.612377616	0.889649609	-0.27813	-0.06253	0.080952	LST4
0.000163311	0.355396524	0.912157913	0.347331	0.127344	0.089937	YBR137W
0.000164145	0.819842906	1	-1.28049	0.151293	-0.03581	DIT1
0.000164987	0.047600922	9.24605E-05	0.359775	-0.24649	-0.89329	MET10
0.000165607	0.622100216	1	-0.76714	-0.16686	-0.07395	NIT1
0.000165723	0.622910776	0.971609311	0.253418	-0.0558	0.044499	GAL83
0.000165765	0.991616462	0.786892522	0.295157	0.008807	0.123065	CTH1
0.000166789	0.871347596	0.711247098	-0.36472	0.034993	0.186518	KNS1
0.000167036	0.663621022	0.670601582	0.769621	-0.15444	0.433922	SLZ1
0.000170054	0.35545331	0.576969329	-0.24175	-0.0885	0.15897	YPL245W
0.000170597	0.074432949	1	0.546865	-0.34553	-0.04292	CDC13
0.000172814	0.388489658	0.399715437	-0.29097	-0.10006	-0.24433	OPI1
0.000174794	0.412262997	0.919926509	-0.40613	0.133495	0.099917	SLX1
0.000175132	0.754750696	0.862693719	0.219329	0.033929	-0.07233	SAC6
0.000175177	0.091928673	0.774318648	-0.34216	0.202811	0.147699	SYC1
0.000176352	0.958672582	0.167222206	-0.40269	-0.0197	-0.47013	YLL058W
0.000176445	0.028113606	0.871950707	-0.39118	0.289443	0.122612	BRR6
0.000176625	0.589666034	0.84746497	-0.56595	0.132901	0.195046	POC4
0.000177879	0.976830326	0.853162234	-0.2274	-0.00903	0.077486	EMP47
0.000178537	0.136830998	0.952970302	0.325027	-0.17599	-0.06506	CSC1
0.000179511	0.632634392	0.393421909	0.282177	-0.061	-0.24025	LYP1
0.00017987	0.008041053	0.659234929	0.370661	0.325828	0.21438	YKL091C
0.000180538	0.693075842	1	-0.32048	-0.05894	-0.02312	IBD2
0.000180685	0.900786402	0.891751427	-0.27104	0.021735	0.078184	YMR130W
0.000181307	0.440961232	0.781875866	-0.84378	0.259394	0.360024	YKL183C-A
0.000182084	0.936226276	0.043101043	0.364102	-0.02256	0.566602	YBR063C
0.00018347	0.134186983	0.812812583	-0.40325	0.216917	-0.15609	RAD33
0.000183682	0.446288659	0.807027851	-0.31362	-0.09753	-0.12388	SRB5
0.000184243	0.914594311	1	0.294886	-0.02175	0.01381	MNT2
0.000184306	0.693801455	1	-4.29032	-0.57424	0.603042	None
0.000185561	0.077645949	0.721271027	-0.51308	0.315624	-0.25609	MRX7
0.000188902	0.918983013	0.377923962	0.26828	0.019166	0.235371	SEC66
0.000190915	0.770350536	1	0.286766	0.041669	-0.00908	RIM21
0.000194673	0.38127191	0.251662308	0.314596	-0.11186	-0.32814	PLB3
0.000195116	0.189863208	0.731592804	-0.32397	0.15723	-0.15756	YOR097C
0.000198062	0.530999534	0.178503269	0.344945	0.092229	-0.40092	GPM3
0.000199789	0.669969608	0.702041373	-0.3756	0.073647	-0.19882	HTD2
0.000200194	1	0.054904024	0.307111	-0.0033	-0.45627	NEM1
0.00020207	0.134575272	0.524155808	0.334583	-0.18322	0.242496	CLU1
0.000202825	0.46096079	0.834202861	-0.24683	0.075198	0.091117	SUI2

0.000203696	0.294576128	1	0.559935	-0.23248	0.010376	SNU66
0.000208056	0.826578265	0.326434921	-0.33759	0.040277	-0.31642	CLP1
0.000210186	0.124795835	0.91085821	0.368382	-0.20687	0.097851	RTS1
0.000214773	0.823461891	1	0.248664	-0.03039	0.017938	SBP1
0.000221016	0.977693292	0.897942454	-0.75714	-0.02964	0.216264	None
0.00022126	0.051317862	0.95255919	0.296057	-0.20321	0.060321	SDD3
0.000224598	0.135026933	0.893930829	-0.28742	0.156699	0.083353	IRA2
0.000225626	0.642716671	0.86530488	0.209363	0.04476	-0.0692	ARP3
0.000229364	0.949363945	0.641899279	-0.34037	-0.01876	-0.20455	TAT2
0.000231071	0.337499055	0.613403067	-0.29112	0.11161	0.18402	FCY1
0.000232547	0.050446555	0.076212737	-0.28435	-0.19545	0.400566	BMS1
0.000232831	0.361545268	0.549098089	-0.30897	0.113819	0.216105	RPB9
0.000233624	0.321712563	0.580281763	0.337585	0.134015	-0.22517	YPI1
0.000234579	0.483079932	0.491045055	0.274872	-0.0816	-0.20961	VPS35
0.0002352	0.658725803	0.847404354	-0.31944	-0.06567	0.112953	YAP3
0.000235306	1	0.986945047	0.335501	-0.00402	-0.04909	MAK32
0.000239298	0.846530385	0.830189162	-0.20448	0.022518	0.077126	SAM4
0.000240766	0.649286461	0.909554543	-0.91696	-0.19096	0.249897	REC104
0.000243255	0.533678741	0.750071687	-0.33279	-0.08894	0.155819	GAB1
0.000243255	0.358263416	0.879698394	-0.31312	-0.11667	0.097158	URK1
0.000246304	0.968504857	0.997335542	-0.40749	-0.01837	0.05412	PEX31
0.000247707	0.652629347	1	-0.50888	0.10581	-0.02818	None
0.000247881	0.008136572	0.411976149	0.35819	0.320421	-0.30323	MDJ1
0.000248044	0.846530385	0.113089839	0.262766	-0.0292	-0.34493	YAP1
0.000248536	0.395417127	0.17936463	0.966571	0.33914	1.143791	YGL088W
0.000249799	0.744117095	0.029232	0.665001	0.109584	-1.10508	YDR274C
0.000250641	1	0.945866293	-1.57865	0.012036	-0.35925	FIG1
0.000250805	0.813026602	0.003519847	-0.46837	-0.05937	-0.96414	MET8
0.000250805	0.854733623	0.798608504	0.888879	-0.09606	-0.36778	YPT35
0.000250849	1.04691E-14	0.00395857	0.340424	0.728423	-0.69828	BDH2
0.000250866	0.037225892	1	0.418027	0.305162	-0.01074	EMI1
0.000253444	0.261134686	0.750952753	-0.51722	-0.22778	-0.24178	INO1
0.000254911	0.369301019	0.236053315	0.931176	-0.34901	-1.0355	YDL242W
0.000255287	0.284831522	0.165301907	-0.30646	-0.13005	0.371336	NOP19
0.000258098	0.971367697	0.439260184	0.333431	0.014604	-0.2744	FAR10
0.000259705	0.34403671	0.063736572	0.314951	0.121187	-0.46033	NGR1
0.000261163	1	0.731592804	0.237147	0.004151	-0.11784	PRE6
0.000261412	0.034121869	0.919926509	-0.2927	0.215666	0.074278	MRPL17
0.00026305	0.744117095	0.252787222	0.30757	-0.05082	-0.3264	LPL1
0.000265566	0.261791204	0.567844831	0.598277	0.265685	-0.40915	BIO5
0.00026843	0.397361658	0.743894449	-0.32116	0.111712	-0.1538	DSS4
0.000270671	0.058379077	0.840202254	-0.34172	0.228967	0.125059	YNR040W
0.000278806	0.6215647	0.964698121	-0.27452	-0.0623	-0.05223	CCT6
0.000281094	0.652115007	0.976568193	-0.32138	-0.06792	-0.05381	YLH47
0.000281392	0.779569301	0.586097635	0.214307	0.031058	-0.14364	YCK1
0.000283142	0.937223959	0.783364287	0.268917	0.016973	-0.11726	YLR042C
0.000283598	0.311884893	0.916266829	0.334457	-0.13696	0.088089	TUB4
0.00028505	0.850784129	0.993440716	0.247306	0.02727	-0.0348	TFA1
0.000288315	0.788420725	0.810360586	0.337964	-0.04761	-0.13674	SPT20
0.000288315	0.011848972	1	-0.32794	0.282911	-0.03611	MRPL28
0.00028935	0.339164188	0.82890057	0.682252	0.26732	-0.26157	GIT1
0.000291244	0.881033345	1	-0.28601	0.027332	-0.02358	GGC1
0.000291405	0.015182854	0.046985988	0.262957	0.220643	-0.4114	MBF1
0.000292964	0.693741522	0.181792015	-0.29181	-0.05525	-0.34481	OSH6

0.000294111	0.980772539	0.129926108	-0.37282	0.014281	-0.47929	SLM3
0.000294619	0.266011692	0.745371404	0.362392	-0.16128	-0.17407	NUP116
0.000296219	0.596511642	0.402187252	0.43303	0.105103	-0.37677	TBS1
0.000298817	0.126086357	0.46663268	-1.68834	0.887358	-1.47313	YLR296W
0.000298817	0.0122332	0.715570823	-0.34911	0.299801	-0.18129	COX5B
0.000301684	1	0.330481176	0.389231	0.008405	-0.37221	YOR114W
0.000301717	0.328528692	0.591339962	0.387978	-0.15565	-0.25921	ANR2
0.000302899	0.562298059	0.239874247	0.259134	0.067507	-0.28305	HAT2
0.000302995	0.7088986	0.848611069	0.207952	-0.0384	0.0744	CCT7
0.000307289	0.27837215	0.677517465	-0.24047	-0.10445	0.138287	NOB1
0.000308542	0.330237847	0.778615611	0.256605	-0.1026	0.114605	SCJ1
0.000309124	0.36194188	0.525203768	-0.30546	-0.11487	0.226312	CDC40
0.000309782	0.02447321	0.915496392	0.306378	-0.24343	0.081684	SUP35
0.000310272	0.025326351	0.259414235	-0.24835	-0.19543	0.264191	DBP6
0.000310483	0.596380201	1	-2.85436	0.55902	-0.08065	YNL067W-I
0.000311305	0.345281679	0.871227843	0.29722	-0.11574	-0.09757	SIN3
0.000311589	1	0.862693719	-0.26453	0.000525	-0.09021	SAD1
0.000312194	0.27669168	0.585629945	-0.52708	0.22773	0.355077	YFR035C
0.000318535	0.394221434	0.577042274	-0.2737	0.09691	0.187109	RPL4B
0.000318535	0.023258179	0.750952753	0.525728	-0.42454	-0.25094	VPS64
0.000320112	0.884663483	1	0.34497	-0.03283	-0.02322	SIZ1
0.000321111	0.279341459	0.711247098	-0.69249	-0.29994	-0.37777	YER135C
0.00032171	0.529464272	0.818523526	0.27063	-0.07497	-0.10754	SPT2
0.000322346	0.4447765	0.650488242	0.371	-0.12104	0.22682	PLC1
0.000324656	0.473710488	0.13719416	0.444136	0.137271	0.571261	TFB5
0.000331802	0.639700807	0.76367822	0.281317	0.062212	0.131079	DFR1
0.00033291	0.606249543	0.202387133	-0.35802	0.085047	-0.41439	GLG2
0.000334773	0.24689862	0.185142339	-0.25911	-0.11895	0.30886	UTP6
0.000335011	0.672577856	0.764340274	-0.39748	0.080058	0.183986	CIN1
0.000338473	0.765667428	0.837234982	-0.3316	-0.05085	0.125082	LCL2
0.000339526	0.652629347	0.922539567	0.25829	0.055507	-0.06566	STS1
0.000343897	0.691034132	0.711541955	0.388959	0.075648	-0.2084	ADK2
0.000345215	0.8635571	0.28677728	0.447593	0.047366	-0.45848	FAA2
0.000346698	0.15183452	0.041756428	-0.68857	0.370974	-1.08733	ATG41
0.000347392	0.550286053	0.101364046	-0.29314	0.07812	-0.40144	TEM1
0.000347871	0.08272612	0.584732535	0.469654	0.301111	-0.31951	YLR456W
0.000348545	0.413512161	0.734750569	-0.35749	0.122646	0.179115	GPI11
0.000348662	0.607278799	0.226898765	1.242093	0.29915	-1.39865	YIL163C
0.000354851	0.986467664	1	-0.23355	0.008181	0.002397	SOL3
0.000354912	0.586093634	0.008874818	-0.32085	0.079822	0.631032	FYV7
0.000354912	1	0.834791774	-0.21331	-0.00043	-0.08131	SEC14
0.000358618	0.753846794	0.56134476	0.392963	-0.06421	-0.27852	RHO4
0.000359108	1	1	-0.63241	-0.01176	0.018056	RPL36B
0.000359112	1	1	4.05403	0.046564	0.050249	YNR077C
0.000361889	0.639747185	0.171413013	0.318246	0.070855	0.390377	BNA4
0.000365636	0.051508812	0.901225243	0.287602	-0.20398	-0.08408	CDC24
0.00036572	0.179113779	0.669483248	-0.24043	-0.12436	0.142313	YCF1
0.000371395	0.695223553	0.489652715	0.328524	-0.06361	-0.25904	MDM36
0.000375613	0.083385991	0.321663363	0.466473	-0.3038	-0.46478	COS10
0.00037748	0.156939743	0.30954724	-0.31826	-0.17249	-0.31874	DCR2
0.000377531	0.665960377	0.39760733	-0.44203	-0.09165	-0.39268	ROG1
0.000380885	0.649636464	0.569177865	0.452133	-0.09921	-0.31755	YPL077C
0.000381414	0.236287265	0.046419466	0.330224	0.156001	-0.52771	KIN1
0.000382512	0.94036767	0.907281575	-0.21426	0.013269	-0.06006	SIS2

0.000390237	0.432562651	0.838437232	0.440816	-0.14958	-0.16776	FZF1
0.000393288	0.152799039	1	0.292177	-0.16103	0.002418	SPT16
0.000394542	0.166873945	0.87106839	-0.27235	0.14454	0.090703	BET3
0.000394665	0.515760255	0.184826991	-0.23362	-0.06704	0.281763	FCF2
0.000395973	0.887970391	0.16670075	-0.21256	-0.01964	0.264339	RPB5
0.00039939	0.950105874	0.224095016	-0.31145	-0.01758	-0.35369	PEX1
0.000406659	1	0.961737929	-0.2935	7.55E-05	-0.05877	FCY21
0.000408041	0.198842787	1	0.381663	-0.19376	-0.04035	COY1
0.000408734	0.295565061	1	-0.26904	0.115236	-0.00864	TLG1
0.000408772	0.018092653	0.786892522	-0.25597	0.214022	0.112882	GRX2
0.000411669	0.098340256	0.02398692	-0.40382	0.248671	0.717043	MRP2
0.000411669	0.030231646	0.399062539	0.304456	-0.23939	-0.27309	MMT1
0.00041254	0.446288659	0.715570823	0.308709	-0.10202	-0.16513	GRR1
0.000412894	0.009676844	0.688473731	-0.33857	0.306153	0.194381	SDH7
0.000413198	0.184976433	0.327821109	-0.2242	-0.11587	0.220216	IPI3
0.000416437	0.014034771	0.634326206	0.458294	-0.40201	0.293812	SPC110
0.000416928	0.397177764	0.860528802	1.388031	0.494291	0.506673	YOL131W
0.000418695	0.279574972	0.656171223	-0.43686	-0.19328	-0.2681	SWF1
0.000426	0.951095533	0.837344851	0.355548	0.020118	-0.13644	YPL162C
0.000426402	0.347850933	0.201440577	0.240844	0.095153	0.286291	SSP120
0.000427652	0.461309575	1	0.233408	-0.07527	0.003743	SFB3
0.000427655	0.350598953	0.752906449	-0.37127	0.144878	0.179009	HSK3
0.000432116	0.005227243	0.790185414	-0.31209	0.300266	0.136902	MRPL19
0.000434539	0.781865517	0.830832007	-0.42395	-0.06234	-0.16609	FAR7
0.000435661	0.918983013	1	2.022039	0.149709	0.118705	YRF1-7
0.000436129	0.100703333	0.876200558	0.263783	0.163568	-0.08622	ZIM17
0.000436813	0.801294446	0.935685096	-0.26652	-0.03671	-0.06236	MNS1
0.000437277	0.812133286	0.697639077	0.288521	0.038509	-0.16411	EIS1
0.000438064	0.658315538	1	-4.16282	0.660634	-0.70056	YNL067W-I
0.000445779	0.051638227	0.72917091	-0.53793	0.379926	0.277011	PEX34
0.000447194	0.496224832	0.939670905	-0.51078	0.152404	-0.11603	THI21
0.000452229	0.967392794	1	-0.25221	-0.01211	0.004023	KEX1
0.000452554	0.876137537	0.107763832	0.263901	-0.02681	-0.36475	YLR225C
0.000452836	0.938970104	0.795458324	-0.42515	0.026977	-0.18425	ORT1
0.00045493	0.184282947	0.677737049	0.45007	-0.23643	-0.26604	PKH3
0.000459716	0.193803735	0.780208648	0.307863	-0.15918	-0.14091	GYL1
0.000459716	0.759241781	0.929303164	-0.30497	0.049276	-0.0754	YCL002C
0.00046534	0.427585494	0.856262311	-0.45512	-0.15584	-0.16296	YPL216W
0.000466445	0.107317686	0.83080918	-0.35365	0.215232	0.139175	SPP2
0.000466571	0.460638341	0.817868751	-0.28795	-0.09309	-0.11771	YDR415C
0.000467911	1	0.292035407	-0.24555	0.001635	0.254853	CSL4
0.000468215	0.775055908	0.110596652	-0.7429	0.110609	-1.05873	PEX9
0.000477402	0.467087012	0.804181925	-0.32364	-0.10351	0.137161	FMP27
0.000481774	0.586124484	0.438960859	0.260693	-0.06688	0.224989	MID1
0.000490312	0.024098511	1	0.235678	-0.19388	-0.01536	IXR1
0.000491982	0.005039572	0.108272796	-0.24342	0.237443	0.337881	MRPL24
0.000495015	0.021955716	0.04443004	0.239324	-0.19981	0.395863	MPP10
0.000501975	0.788895187	1	-0.35544	0.051306	0.038303	ASA1
0.00050356	0.765667428	1	-0.28474	0.044887	0.027332	COG2
0.000505307	0.969164701	0.394712497	0.222938	0.010561	0.204074	YRB1
0.000506431	0.612454623	0.993440716	0.334928	-0.08196	-0.04901	MDM10
0.000512511	0.66530676	1	-4.18987	0.672751	-0.71673	YMR272W-I
0.000513306	0.767313943	0.708495578	-0.55916	-0.08737	-0.31002	PSO2
0.000515221	0.023687856	1	0.248359	-0.20558	-0.01562	CCR4

0.000519542	0.764865689	0.986912078	0.387491	0.061893	-0.05994	NPP2
0.000522915	0.314228532	0.045166127	-0.24338	0.102668	0.401118	RPL31B
0.000532694	0.346392845	0.84419011	0.235521	-0.09506	-0.0899	BUD14
0.000537784	0.500936572	0.66765157	1.191689	-0.36728	-0.745	YPR204W
0.000538359	1	0.740653672	0.254899	-0.00131	0.129766	SPO12
0.000545955	0.860071452	0.90453709	0.345518	0.038213	0.10221	HRT3
0.00054602	0.997425542	0.927619939	0.25452	-0.00708	-0.06481	PBN1
0.000559068	0.005327849	0.324708204	-0.35497	0.346569	0.357009	ECM19
0.000559765	0.692852222	0.859694751	-0.21414	0.042569	0.077251	PPH21
0.000563948	0.04701393	1	-0.25586	0.18864	0.006325	YBR056W
0.000564329	0.458242569	0.86336133	0.297757	-0.09866	0.105895	MCM4
0.000566285	0.051155386	0.918987226	0.362468	-0.26597	-0.09852	NAB3
0.000575546	0.333861939	1	4.014777	1.368806	1.48921	None
0.00058138	0.477410911	0.565847555	-0.28096	-0.08934	-0.20359	MNL1
0.000582431	0.950105874	0.820868642	0.431717	0.025344	-0.17823	VHS1
0.000583435	0.105961984	0.179756965	-0.28997	0.180065	0.362167	[illegible]
0.000583435	0.292826036	0.290004916	-2.50059	-1.04306	-3.69384	None
0.000596704	0.989216982	0.962684605	0.219617	-0.00742	0.044753	WBP1
0.000600525	0.52244313	0.772090669	0.38964	-0.11448	-0.18445	UPC2
0.000604312	0.783881329	0.874343567	-0.60957	0.090214	-0.20673	YPL264C
0.000609378	0.01281546	0.571875715	-0.61764	0.548852	0.443159	FMP23
0.000616129	0.335664802	0.467794221	0.334414	-0.13937	-0.28309	RTT109
0.000619014	0.615223063	0.12209407	-0.23936	-0.05832	0.329475	RRT14
0.000620467	0.990169077	0.981181758	0.328312	0.010927	-0.05551	YNL165W
0.000622251	0.833793818	0.669483248	-0.49034	-0.0612	-0.30203	RRT13
0.000626652	0.480485132	0.481039634	0.28779	-0.09206	-0.23879	VPS27
0.000630203	0.440243906	0.664126703	-0.27532	-0.09439	0.171526	TBF1
0.000641741	0.140485465	1	0.358955	-0.21034	0.027971	KCS1
0.000641982	0.407136042	1	0.434133	-0.15942	-0.01204	LRG1
0.000642465	0.92392503	0.715570823	0.245012	0.018333	0.135623	YAR1
0.000645939	0.620913491	0.96218812	-0.24716	0.059572	0.050988	HOM6
0.000646125	0.004400122	0.82890057	0.247587	-0.25227	-0.10086	SIL1
0.000649216	0.496469376	1	-4.0235	0.915324	-0.97209	HXT16
0.000652936	0.038593301	0.983370642	0.295829	-0.23109	-0.04932	HOF1
0.000656468	0.478236611	0.985066576	0.271248	0.087368	-0.04346	MED8
0.000656788	0.091928673	0.976568193	-0.24379	0.158552	-0.04337	MRPL22
0.000662824	0.020403094	0.768164938	0.386019	-0.33358	-0.18678	SEC6
0.000662824	0.407136042	0.931568877	-1.51067	-0.53843	-0.37898	HOP2
0.0006642	0.472617197	0.009452184	-0.96442	0.306431	-1.99136	SIP4
0.000668968	0.564303748	1	-0.30171	0.082167	0.030055	ARR1
0.000669332	0.32171509	1	0.620113	-0.26744	-0.01442	YNR063W
0.000675441	0.077481906	0.636815206	-0.254	-0.17288	-0.16613	LYS4
0.000687026	0.893928423	0.7990478	0.874362	-0.08192	-0.39104	None
0.000693342	0.507001261	0.929303164	0.227658	-0.06952	0.058007	PEX25
0.000693866	0.825517831	0.791580335	0.403965	-0.05336	0.183736	VPS41
0.000696278	0.995919167	0.915496392	-0.29447	-0.0088	0.083044	NAT3
0.000702513	0.430250055	0.566767977	0.201735	-0.07132	0.149002	CCT5
0.000702513	0.038222175	0.888371569	-0.38085	-0.29732	-0.12393	SPP382
0.000702605	0.099346217	0.048861894	-0.25623	-0.16482	-0.42404	ATG26
0.000702605	0.835126825	0.936440675	0.286097	0.036173	-0.06905	DCP1
0.000708429	0.041913401	0.652552516	0.298493	0.229417	0.191996	MXR2
0.000713012	0.38101428	1	1.465588	-0.57832	-0.06915	[illegible]
0.000713031	0.950105874	0.677620076	0.289594	-0.01718	0.177577	AIM20
0.000719287	0.911433046	0.806965865	0.270995	-0.02245	0.118187	SKO1

0.000720842	0.125172898	0.955327449	-0.38056	-0.23087	-0.08249	POP3
0.00072089	0.076965336	0.79124327	0.254825	-0.17569	0.116414	STE5
0.000723378	0.000332944	0.065961241	-0.2013	0.244206	0.315533	ATP1
0.00072346	0.384327883	0.561585828	-0.31703	0.121356	-0.23496	YPL107W
0.000723655	0.332602552	0.36347688	-0.23473	-0.09883	0.229312	SSF2
0.000726464	0.660417863	0.378965865	0.301267	-0.06723	-0.28857	MRL1
0.000730395	0.346188985	0.559860815	0.215234	-0.08891	0.161378	DBP5
0.000734177	0.533678741	0.963980547	-0.38168	0.109975	0.078082	YJR124C
0.000738267	0.537835584	0.931568877	1.9028	-0.55286	-0.52226	YMR230W-A
0.000743111	0.032880551	0.525280623	-0.26572	0.212178	-0.20868	PET20
0.000743111	0.884827129	0.976568193	0.270278	0.027187	-0.04847	YHC1
0.000743111	0.486616397	1	-0.35268	-0.11194	-0.02041	TPO3
0.000752712	0.028417401	0.11348234	-0.34978	0.286297	0.495675	TIM8
0.000752946	0.195663525	0.743371659	-0.2933	0.154979	0.151406	APN1
0.000756418	0.951086794	0.586097635	-0.45534	-0.02663	-0.32523	SHR5
0.000759745	0.499768153	0.916400787	0.180085	-0.05615	0.050803	DED81
0.00076801	0.207851396	0.567844831	0.211063	-0.11026	0.156637	SHO1
0.000770238	0.099561277	0.35208106	0.407486	0.264227	-0.40483	RCR1
0.000775281	0.015544148	0.558625562	-0.32648	0.291133	0.244947	RSM19
0.000775281	0.308388389	0.98500242	0.261455	0.11533	-0.04313	IDP1
0.00077834	0.713873054	0.429538302	-1.69093	0.307238	1.585854	None
0.000780762	0.176877685	0.770752057	0.358997	0.197951	0.174553	YLR126C
0.000784504	0.199496245	0.92566928	0.194638	-0.10322	0.05184	YNL247W
0.000784504	0.240592911	0.934511234	-0.37865	0.185717	-0.09263	YMR252C
0.000794412	0.892198191	1	-3.97077	0.277021	-0.29333	YLR157W-I
0.000794553	0.136629201	1	0.332525	-0.20014	-0.0184	YJR061W
0.00080546	0.04046433	0.990208543	-0.42337	0.327378	0.065696	YER076C
0.000809559	0.367959138	0.496426949	-0.38676	0.153891	-0.3171	MEP3
0.000819366	0.564430341	0.839890676	0.232229	-0.06473	-0.09276	DCP2
0.000819558	0.014428866	0.904680493	0.297933	0.271269	0.090024	FES1
0.000820249	0.014447394	0.89043028	0.350368	-0.32109	-0.11313	GIN4
0.000821365	0.99176478	1	-0.30658	-0.01009	0.03226	NTO1
0.000829239	0.868504082	1	0.212402	-0.02329	0.020526	TPM2
0.000840379	0.062985577	0.838617816	0.262217	-0.18977	-0.10532	UBA1
0.000845934	0.885106299	0.781154036	-0.23886	0.02397	0.113417	HIP1
0.000850902	0.095680569	0.540033807	0.274708	-0.18236	-0.2139	ARE1
0.000856639	0.233652909	1	-3.99943	-1.63763	1.754137	None
0.000857831	0.041913401	0.717705244	-0.4043	-0.31517	-0.22822	GUP2
0.000868595	0.603345299	0.901225243	0.311708	-0.08085	-0.09704	YDR541C
0.000873341	0.868862376	0.971609311	-0.22144	-0.02418	-0.04257	ENT5
0.000874136	0.963472115	0.971609311	0.339345	-0.01789	-0.06533	CUL3
0.000874869	0.157035658	0.971609311	-0.33112	0.189846	0.063332	SYS1
0.000874872	0.759733432	0.597863762	-0.29037	-0.04898	-0.20738	VPS38
0.000875244	0.231636316	0.203404731	0.210668	-0.10674	-0.26169	ADE2
0.000875666	0.488592146	0.733341705	-0.24472	0.07826	0.132122	NUC1
0.000879168	0.628843645	1	-0.37412	-0.09078	-0.03392	PET18
0.000885214	0.118813668	0.744376434	0.413054	-0.26133	-0.21585	YAP7
0.000885539	0.025661604	0.729715118	-0.3981	0.334677	-0.21546	GPI18
0.000885539	0.615223063	0.908303081	0.287593	-0.07258	-0.08566	UNG1
0.000886649	1	0.811835676	-0.46391	0.004536	-0.20218	SCW11
0.000887025	0.07978505	0.968819743	0.214025	-0.14861	0.043025	RTF1
0.000889428	0.016503521	0.972387712	0.452725	-0.41205	-0.08639	RFC1
0.000899972	0.619450596	1	0.283334	-0.07092	-0.01654	KCH1
0.000915918	0.126912899	0.318881364	-0.20949	-0.12901	0.221533	DBP9

0.000923197	0.518133314	1	-4.39431	-1.11181	1.205969	None
0.000923521	0.010032448	0.994376352	0.954193	0.920282	-0.14348	PHM7
0.000929891	0.084180469	0.004134287	-0.23996	0.163565	0.543027	RPL34A
0.000943378	0.533678741	0.431105692	0.182538	-0.05411	0.167154	TIF5
0.000947636	0.161855773	0.971609311	-0.28973	0.165661	0.056203	TIM18
0.000948099	0.325985364	1	0.373527	-0.16339	-0.05184	NPR3
0.000948827	0.464413458	0.815112247	0.209101	-0.07112	-0.09107	POP2
0.000950263	0.919230327	0.904680493	0.273925	0.021918	-0.08407	NBP35
0.0009509	0.308388389	0.438612707	1.580747	0.687459	1.508782	COX1
0.000953038	0.74444644	0.584732535	0.247837	0.044856	-0.18208	TAF7
0.000960435	0.929795254	0.113089839	-0.23907	0.017506	0.346455	NHP2
0.0009681	0.436731198	0.907281575	-0.25676	0.09149	0.077308	RPB4
0.000970632	0.953371228	1	0.672166	0.040027	-0.08657	None
0.000989074	0.15633472	1	-0.27924	-0.1629	-0.02805	NMA111
0.00099683	0.902616953	0.13719416	-0.26023	-0.02335	0.362384	AAH1
0.001010586	0.938960046	0.765014016	0.196956	-0.01345	0.099103	GLC7
0.001018332	0.329305093	0.953128828	0.354993	-0.15546	0.080259	CMR2
0.001024445	0.380470847	0.577843626	-0.72156	0.283423	0.536212	SSP1
0.001024623	0.95461314	0.847404354	-1.2908	-0.07315	0.514259	None
0.001031619	0.358276703	0.971561168	-0.80364	0.32733	0.159853	HUR1
0.001033649	0.61985593	0.949031652	-0.26277	0.06587	-0.06076	ERP5
0.001036637	0.653468553	0.770752057	-0.34948	-0.08079	-0.17351	YJR098C
0.001038279	0.927882032	0.919926509	0.297338	0.022487	0.083613	DOS2
0.001044019	0.428998997	1	3.869997	1.174661	1.282869	None
0.001045304	0.814509078	0.616015902	0.384172	-0.05438	-0.27044	IST1
0.001045807	0.842331341	0.011004386	1.242998	0.155772	2.772061	YOR381W-A
0.001045807	0.518133314	0.927131674	0.923424	-0.28811	0.25207	HXT15
0.001045807	0.738978817	1	-0.3606	0.066128	0.021412	EMC6
0.001045968	0.020884935	0.120010897	-0.60097	0.524809	0.859505	NCE101
0.001049596	0.803202217	0.711421266	-0.23982	-0.03503	0.139674	RRN3
0.001051596	0.139798632	0.789517744	0.23019	-0.14031	0.108947	YHR045W
0.001059248	0.007076411	0.255950109	-0.70107	0.692387	-0.81253	REG2
0.001059248	0.414021553	0.789517744	0.260575	-0.09794	-0.1233	MED1
0.001059248	0.78141125	1	-3.92806	-0.47352	0.502829	None
0.001059248	0.990169077	1	0.31703	-0.01106	-0.0275	PRP4
0.001065815	0.030733392	0.264879577	0.833087	0.697252	-0.95438	ALP1
0.0010727	0.118813668	0.853367097	0.195544	-0.12514	0.075876	LCB1
0.001086154	1	0.229407061	0.343498	0.008026	-0.41798	POG1
0.001087953	0.17746989	0.786584362	0.394163	0.222789	-0.18755	ATG9
0.001090917	0.479437499	0.812812583	-0.28061	-0.09335	-0.12415	DYN3
0.00109483	0.661068682	0.972782671	-0.1949	0.044547	0.037542	HCR1
0.001109158	0.397738268	0.008957438	-0.44319	0.17033	0.94851	YGL194C-A
0.001109158	0.775312326	0.465799976	2.034528	-0.31585	2.066515	YHL037C
0.001116519	0.27789823	0.931005601	1.117896	0.537757	0.291769	THI13
0.001117436	0.582123297	0.816819753	-2.00538	0.490659	-1.00964	None
0.001117436	0.96919526	0.92711763	0.235184	-0.01183	-0.0639	RTT106
0.001123441	0.418773131	0.673402119	-0.2055	-0.07651	0.13108	YGR054W
0.001123441	0.402477551	0.744376434	-0.92286	0.348208	-0.48426	YNL019C
0.001145908	1	0.378557332	2.710867	0.022872	3.109034	YLR464W
0.001148246	0.76967246	0.90015429	0.548514	-0.09187	-0.17605	YBR013C
0.001151124	0.008862509	1	0.272905	-0.27277	0.030088	RSC8
0.001151156	0.110870889	0.978193638	0.347977	-0.22872	-0.06368	VIK1
0.001161678	0.140829707	0.883095126	0.321777	-0.19738	-0.11112	YFR016C
0.001171297	0.487676602	0.55529834	0.471156	-0.15676	-0.36893	FAR3

0.001173387	0.823755279	0.175838148	-0.26552	0.036455	-0.35085	MAM3
0.001173387	0.482993634	0.179756965	1.063146	-0.3591	-1.47364	None
0.001175156	0.443467125	1	-0.72586	0.256296	0.079004	YDR034W-B
0.001185603	0.885106299	0.516122403	1.082446	-0.11416	-0.91184	YCL001W-A
0.001186742	0.796833215	0.976568193	0.270978	-0.04113	0.050642	ALG3
0.001189271	0.616266876	1	0.19849	-0.05105	0.027268	KTR4
0.001189714	0.676923904	0.922684393	0.348251	-0.07717	-0.09725	CET1
0.001196141	0.479887942	1	0.218144	-0.07339	0.028608	CHS5
0.001199197	0.382001694	1	0.541744	-0.21973	0.034659	PKH2
0.001216226	0.0122332	0.737956609	-0.23431	0.224373	-0.12728	VTC3
0.001223321	0.709900242	0.790203342	0.283909	-0.05811	-0.13524	ATG18
0.001224607	0.792133624	0.740431023	0.24794	0.038377	-0.13435	AOS1
0.001228193	0.041731521	0.86530488	0.218261	-0.17628	-0.08177	HBS1
0.001237386	0.810491252	0.058656331	-0.23136	0.033398	0.387294	RPF2
0.001237386	0.97067567	0.919271701	-0.27976	0.013816	-0.0804	SNX3
0.001249532	0.662458676	0.971609311	-0.37684	-0.08669	0.075718	AST2
0.001268409	0.066428344	0.039714977	-0.20164	0.14808	0.366376	RPL17A
0.001268409	0.267877158	0.69956533	-0.27906	0.136614	0.170135	YJL127C-B
0.001270465	0.957430726	0.062061839	-0.18814	0.010772	0.312016	RTN1
0.001276125	0.995958449	0.949939733	-0.29235	-0.00911	-0.06855	UAF30
0.001292588	0.113532921	0.917327363	-1.33688	0.829721	0.386089	YLR365W
0.001299535	0.001473853	0.00034954	-0.27925	0.32369	0.759841	RPL29
0.001322471	0.413532829	0.411752857	0.282165	-0.10824	-0.2714	STB1
0.001326018	0.842370046	0.677620076	-0.8392	0.105339	0.54075	None
0.001332707	0.295242295	0.127873691	-0.28127	0.132132	0.407305	RUB1
0.001341058	0.470923648	0.747048811	-0.2534	0.087178	0.135341	BER1
0.001350494	0.349410964	0.002316113	-0.19479	-0.08363	0.475414	NOP4
0.001350947	1	1	0.171521	0.00277	-0.0054	TUB2
0.001354151	0.962083212	1	0.292374	-0.01621	0.041968	IAH1
0.001356237	0.139798632	0.976568193	0.29795	-0.18553	0.056601	MSS4
0.001360708	1.74071E-09	2.07221E-05	0.187935	0.366765	-0.5811	GPD1
0.001371611	0.491008005	0.750952753	-0.38628	-0.12751	-0.20565	EST2
0.001374066	0.000249464	0.148150244	-0.21291	0.276389	0.299498	ATP16
0.001383838	0.753332864	0.077306681	-0.31616	-0.05706	-0.50716	SWT21
0.00139251	0.360876565	1	-0.39335	-0.16606	-0.02898	YLR297W
0.001394854	0.995214622	1	-0.24944	-0.00802	0.005595	VAC8
0.001412436	0.082198263	0.043168161	-0.18982	-0.1352	0.341524	UTP18
0.001412608	0.985451395	1	0.215344	0.008619	-0.03119	SKP1
0.001413184	0.368353535	0.599598606	-0.48823	0.202448	0.360197	SMX2
0.001415433	0.304299791	0.935962035	-0.26198	0.121286	-0.06678	NHX1
0.001418794	0.819842906	0.496483031	-0.30692	-0.04341	-0.26418	MOG1
0.001420057	0.040357896	0.728625888	0.33132	-0.27315	-0.18823	HPR1
0.001423903	0.207956416	1	0.275042	-0.15109	-0.00755	RSC4
0.001425037	0.628871924	0.207271346	0.220843	-0.05609	-0.28382	HIS7
0.00142646	0.209597822	0.720195138	-0.2792	0.151403	0.162198	MRF1
0.00142646	0.157263376	1	-0.22519	-0.13496	0.00917	TRM10
0.001442013	0.082785052	0.451184565	-0.40292	0.284426	-0.36977	BOP2
0.001442013	1	1	3.098652	-0.07391	-0.14211	YBL111C
0.001446974	0.310057499	0.8119342	-0.2414	0.111133	0.109519	SCS2
0.001446974	0.185561714	0.926181253	-0.24472	-0.13929	-0.06811	SEC39
0.001452648	0.970974534	0.391001548	-0.31514	-0.01572	-0.3129	YDR109C
0.001455494	0.000577638	0.972461195	-0.22886	-0.28676	0.045288	GIC2
0.001479579	0.184156664	1	0.23737	-0.13639	-0.00635	CTR9
0.00149104	0.954188947	0.539917165	-0.22313	-0.01344	0.181795	ALG5

0.001491594	0.250649618	0.046235167	-0.1913	-0.098	0.341617	ENP1
0.001492241	0.892744236	0.406901206	-0.24293	0.024022	0.237393	DUT1
0.001494531	0.529751676	0.91703897	0.493523	-0.15423	-0.1464	SLD7
0.001497366	0.198089285	0.87592683	-0.209	0.116435	-0.07538	OSH7
0.00150176	0.339193543	0.976378097	0.30317	-0.13461	0.058694	BNI1
0.001502187	1	0.789517744	-0.32326	-0.00869	-0.157	SRO77
0.001509149	0.753332864	1	0.528482	0.096999	-0.0765	YLR053C
0.001509483	0.838806875	0.440092898	-0.25791	-0.03388	0.242048	SPE4
0.00151699	0.028455376	0.350046527	-0.27267	0.236574	0.287253	MIC19
0.001519476	0.762436308	0.714114751	0.496743	-0.08745	-0.29591	SSH4
0.00152807	0.157412865	0.531220921	-0.27034	-0.16286	0.224113	FAL1
0.001533885	0.925288466	0.058656331	-0.89358	0.069637	1.517402	None
0.001549458	0.441943453	0.673402119	0.212229	-0.07838	0.139604	IOC4
0.001550845	0.093143688	1	-0.28599	0.198647	0.022517	FTH1
0.001555824	1	0.598011569	1.592643	-0.00201	1.262373	FYV12
0.001561455	0.912368353	0.23906305	0.216909	0.018991	0.270081	RPL7A
0.00156893	0.051484274	0.92578246	1.501794	-1.24129	-0.51434	HXT10
0.001590924	0.964932829	1	0.256818	0.014023	0.037593	SGT1
0.001593899	0.121271795	1	0.229973	-0.15122	-0.02486	CDC1
0.001594499	1	0.795907717	0.387137	0.00723	0.18592	DOA4
0.001606664	0.004742556	0.721271027	-0.28853	0.310201	0.168385	AIM18
0.00161356	0.08826227	0.661089241	-0.18113	-0.12847	-0.12234	FAS2
0.001614868	0.002518787	0.881658423	-0.19879	-0.22734	0.070763	FAR1
0.001617795	0.11971678	1	0.322325	-0.21332	-0.0211	SNF5
0.00161907	0.244363463	0.937179795	-0.23796	0.12355	-0.06108	MSD1
0.001628615	0.000743475	0.331627583	-0.2842	0.351011	0.308241	COA3
0.001642926	0.818575144	0.466577874	0.218675	-0.03166	0.201009	YGR026W
0.001652945	0.740319156	0.699384165	0.519228	-0.10009	-0.32619	MMO1
0.001675285	0.880383419	0.406901206	-0.26708	0.029325	-0.26295	ROY1
0.00167562	0.479887942	0.702041373	-0.3228	0.11083	-0.19964	CGI121
0.001679533	0.055168494	0.910058751	0.249987	-0.19689	0.077921	NSP1
0.001686611	0.729751469	0.862693719	-0.22779	0.044962	0.088523	VCX1
0.001692024	0.888561341	0.962684605	0.411329	-0.04276	0.091324	PHO80
0.001692024	0.058914883	1	0.363319	-0.28347	-0.02153	PMS1
0.001694459	0.775312326	0.953606441	0.241283	-0.04067	-0.05673	UFD2
0.001699948	0.022170199	0.904680493	0.325171	-0.29958	0.104784	SBE22
0.001707274	0.76984172	0.524702337	-0.25839	-0.0441	0.218457	RPP1
0.001707274	0.779493671	0.755422986	-0.23151	-0.03839	-0.12407	FKH1
0.001717577	0.187466722	0.088199	-0.27285	0.156435	0.434592	RPL12A
0.001722487	0.151510275	0.083080335	0.35729	0.221427	-0.57432	YDL206W
0.001732071	0.571273445	1	-0.505	-0.14626	0.005673	COS8
0.00174176	0.240597184	0.897313673	0.178018	-0.0942	0.059885	SSZ1
0.00174648	0.001264075	0.592391518	0.197318	-0.23994	-0.15068	TEC1
0.001757132	0.957259373	0.241377894	0.178503	-0.01058	-0.22291	SER1
0.001757738	0.374954065	0.711247098	-0.31117	-0.13093	-0.18972	VFA1
0.001763044	0.531968668	0.27982387	-0.33799	-0.10561	-0.39702	MCH4
0.001777813	0.53992737	0.972387712	0.373262	0.115698	-0.07548	CDC23
0.001786258	0.138242749	0.828935693	-0.27656	0.175271	0.121737	MRP17
0.001786258	0.408819304	0.91703897	-0.65888	0.257131	0.196804	YNL097C-I
0.001786258	0.413008972	1	-0.80766	0.309583	0.023525	SPO23
0.001791993	0.408819304	1	-0.29304	-0.11578	-0.02067	TEL1
0.001793005	0.440961232	0.919516515	-0.45336	0.167799	-0.13378	SHU1
0.001798211	0.013379255	0.388159122	-0.29875	0.291268	0.303491	PET100
0.001807256	0.585153927	0.101545647	0.243873	0.069536	-0.38015	RPN4

0.001818954	0.100361569	0.955327449	0.301771	-0.21067	-0.0708	GPI13
0.00184571	0.748281711	0.139610149	-0.3175	0.059565	0.463743	YLR363W-A
0.001883706	0.265931927	1	0.636512	-0.33072	-0.08036	POX1
0.001894393	0.986058626	0.990110569	-0.30989	0.012481	-0.05213	TTI2
0.001912501	0.947996628	0.875200775	-0.62174	0.040589	-0.23089	YGL081W
0.001916819	0.666318943	0.837446458	0.1863	-0.04413	0.080621	SEC27
0.0019364	0.36194188	0.50572517	-0.34597	-0.15004	-0.30275	CAC2
0.00193781	0.035674243	1	0.402214	-0.34808	0.027922	RSC2
0.001947108	0.754399867	0.983370642	0.22398	-0.04165	0.040879	LNP1
0.001960702	0.75339767	1	-3.8096	0.546366	-0.58595	None
0.00196616	0.653029567	0.67221425	-0.53713	0.130295	0.361229	MRPL51
0.001970272	0.213420104	0.76367822	-0.33272	0.184137	-0.17703	CWC2
0.001971791	0.001118198	0.0086529	0.270246	0.333144	0.614324	MRPL39
0.001972581	0.165679639	0.915545807	0.299243	0.182219	-0.09162	TFB6
0.001982677	0.567338142	0.79041392	-1.75501	0.488611	-0.92943	YPL261C
0.00198368	0.615920906	0.735602795	0.204388	0.054937	-0.11741	NYV1
0.002015236	0.516700017	0.845430426	0.228125	-0.07474	0.096531	CAF40
0.002028184	0.059509508	0.071682931	0.241823	-0.19056	0.408649	TRM3
0.002046916	0.155051744	1	0.172995	-0.10812	-0.00373	ARO3
0.00205893	0.138509482	0.016158803	-0.21219	0.136171	0.453373	RPS25B
0.002073277	0.492734305	0.715570823	-0.32635	-0.1114	-0.19767	DNF3
0.002106968	0.767194808	1	-0.22747	0.040045	0.027207	GVP36
0.002129353	0.078795222	1	0.247905	0.185372	-0.00829	MPC1
0.002139284	0.455132842	0.966197359	0.28587	0.105966	-0.0634	CCC2
0.002156828	0.397361658	1	-0.25534	0.104565	-0.03432	TCM62
0.002182081	0.19339556	0.52021616	-0.21876	0.127004	0.190375	VMA16
0.002185703	0.166697083	0.97620447	0.320449	-0.19714	-0.06429	HPC2
0.002187141	0.146050112	0.978193638	0.371459	-0.23852	-0.07174	WHI3
0.002192863	0.261791204	0.740875651	0.945746	0.492157	0.54413	LDS2
0.002193175	0.236124298	0.733389979	-0.19915	-0.10787	0.115992	VBA4
0.002202087	0.66730791	0.554003774	-0.19059	0.045279	0.157421	IFA38
0.002221072	0.116008771	0.021809306	-0.22583	0.153498	0.465703	RPL23A
0.002226366	0.322263381	0.680777882	-0.31773	0.148579	-0.21161	PEX7
0.002231021	1	0.719704683	0.276254	-0.00582	-0.16773	MSL5
0.002246098	0.732764313	0.978377916	1.41772	0.286243	-0.27021	None
0.002253026	0.448302145	0.042142302	0.239585	0.090021	-0.44944	VHR1
0.002286987	0.257848317	0.659101564	-0.22627	0.118798	-0.15809	CIR1
0.002310738	0.163898203	0.69574725	-0.28445	-0.17565	0.185666	FHL1
0.002324401	0.765828853	0.822329423	-0.29646	-0.05297	-0.13576	SFI1
0.002338441	0.259983558	0.097506324	0.286587	0.151105	-0.45995	RAD51
0.00235513	0.38127191	1	-0.35733	-0.15251	0.017492	MUS81
0.002356088	0.011580663	0.966082547	0.435162	-0.45144	0.097961	GAL11
0.002360047	0.925411578	0.600528962	-1.16247	-0.09195	-0.96165	None
0.002378113	0.166610832	0.406901206	-0.31083	0.190557	-0.31517	SPR6
0.002382604	0.880864802	1	-0.37147	-0.04192	-0.05212	RTR2
0.002389458	0.73982331	0.726816534	0.189084	0.03745	0.11293	RVS161
0.00239308	0.649028901	0.947945497	-0.29406	-0.07416	-0.07381	ASF2
0.002394728	0.551291677	0.648751155	-0.45185	0.139685	-0.32143	HUG1
0.002394728	0.754826631	0.689558052	-1.67012	-0.29466	-1.25205	None
0.002394728	0.767134842	1	-0.29131	0.051931	-0.03615	DXO1
0.002404297	1	0.601496564	-0.33826	0.00613	-0.26029	LPP1
0.002447746	0.936093775	0.994517386	-1.71769	-0.12084	-0.29943	None
0.002447985	0.25429907	0.863302781	-0.50847	0.268245	-0.20229	MBB1
0.002449263	0.996960041	1	0.678878	-0.02174	0.096345	YPR014C

0.002453326	0.147016798	0.894445004	0.282039	0.181706	-0.09849	FIS1
0.002477014	0.080149997	1	0.338631	-0.257	-0.04854	PTP3
0.002477413	0.837441872	0.559860815	-0.54131	0.074283	0.447544	DCI1
0.002478313	0.040340762	0.975062924	0.24454	-0.2115	-0.04998	CSI2
0.00248634	0.47499087	0.874977083	-0.23295	-0.084	-0.08885	TAF11
0.002499272	0.370508284	1	-0.26461	-0.11588	0.02088	EFM3
0.002517712	0.690362072	0.860187522	0.221141	0.050416	0.090253	ARL1
0.002517712	0.266929182	0.910199402	0.191613	-0.10077	0.06178	MTR3
0.002518682	0.987715638	0.078502393	-0.22879	-0.00906	0.385809	RPB10
0.002545222	0.157752978	0.897848413	-0.26669	-0.16808	0.092472	SPT14
0.002546803	0.000646782	0.428049321	-0.24083	0.31262	0.239928	MRPL15
0.002562667	0.090430839	0.737036218	-0.46836	0.340715	0.272798	YKL133C
0.002568522	0.000483252	0.967394603	0.263761	0.350691	-0.05912	YBR287W
0.002570465	0.007166364	0.565513961	-0.2922	0.315776	-0.23928	SCO1
0.002573603	0.02375103	0.154422599	0.476377	0.448083	-0.69886	IDP3
0.002573986	0.052551709	1	0.295379	-0.24403	0.005584	REB1
0.00258561	0.320232732	0.086874857	-0.27772	0.132469	0.460015	YCR043C
0.002586581	0.968013408	0.206688593	-0.19492	-0.01069	0.264875	RLP7
0.002589416	0.172225804	0.621769539	-0.21414	-0.13155	0.161475	UTP30
0.002589416	0.295659	0.883095126	1.055813	-0.53824	0.395389	YCR022C
0.002593107	0.781247106	0.952606365	0.246953	-0.04258	-0.06084	INN1
0.00259383	0.148485947	0.333300106	-0.25404	0.163022	0.286945	GTF1
0.002618145	0.502826515	0.722814204	-0.32375	-0.11078	0.196337	BSC6
0.002619671	0.955475747	0.568872092	0.309614	0.019364	-0.25402	SPC29
0.002630781	0.686388856	0.577843626	0.253615	-0.05886	-0.20547	DOP1
0.00264794	1	0.714788215	1.23756	-0.02436	-0.79019	YGL260W
0.002661312	0.196724669	1	-0.27122	0.15924	-0.02198	YPR172W
0.002675733	0.752831669	0.851392759	0.424665	0.082049	-0.17915	SSN8
0.002678071	0.528480393	0.689558052	-0.17947	0.058672	0.119833	RPC37
0.002678389	0.879819172	0.924605282	0.950309	-0.11028	-0.28745	None
0.002696186	0.36016606	0.317858436	-1.57482	0.681979	1.798854	None
0.002700381	0.931613602	0.962684605	0.368596	0.029469	0.085477	VAB2
0.002701287	0.659284357	0.348820609	-0.20935	-0.05213	-0.23267	SEC20
0.00270658	0.280697279	0.902712809	-0.2371	-0.12218	0.080785	RNA14
0.002723808	0.509434188	0.223097491	-0.1703	-0.05793	0.227461	ZRC1
0.002727604	0.936226276	0.725591402	-1.36196	-0.10024	0.847709	YKL097C
0.002732237	0.98811108	0.75616091	0.211304	-0.00834	0.118552	NIF3
0.002739957	0.030006572	0.306618286	0.239058	-0.21952	-0.28241	UFO1
0.002757181	0.163671009	0.333575772	-0.20285	0.127156	0.230531	TDH3
0.002764387	0.83092898	0.390228331	0.457804	0.066275	-0.4827	RMD6
0.002772855	0.726198088	1	-0.31024	-0.06479	0.01474	EXO5
0.002778025	0.490597664	0.029656197	0.240921	0.085289	-0.48684	SSK1
0.002792885	0.914039764	0.975984512	0.288584	-0.02645	-0.05938	STB6
0.002792907	0.295659	0.005410787	-0.23105	-0.11607	0.564443	EFG1
0.002816682	0.030231646	0.820956	0.28737	-0.26448	0.135876	BUD2
0.002822252	0.443199053	0.889079564	-0.40142	-0.15509	0.145878	YIL092W
0.002828937	0.644005887	0.37953299	0.170011	-0.04417	0.183414	SSB2
0.002832792	0.004058934	1.88117E-06	0.254341	-0.29607	-0.91304	MET14
0.002854721	0.953016479	0.56134476	-0.20126	0.013079	0.16868	EFM5
0.002857114	0.385272897	0.974194652	-0.3854	0.165054	-0.08017	YKL070W
0.002860928	0.093744452	0.433186531	0.16704	-0.12367	-0.16783	BAT1
0.002861208	0.210176827	0.972387712	0.31944	-0.1862	-0.06764	STE13
0.002866552	0.049421861	1	0.308496	-0.26164	0.047246	RIF1
0.002881489	0.759733432	0.158209764	-0.16325	0.030574	0.242326	SUP45

0.002882456	0.358333717	0.688473731	0.252521	0.114967	-0.17062	YJR142W
0.002884125	0.00758047	1	-0.28914	0.314289	0.013133	MSY1
0.002889586	0.716376728	1	-0.90454	0.189544	0.040079	None
0.002890681	0.069457617	0.018502313	-0.22957	-0.18068	0.496927	RRP12
0.002931575	0.670263285	0.518188337	0.352587	0.08597	-0.31618	YMR1
0.002934206	0.67815339	0.717477893	0.531146	-0.12812	-0.33355	None
0.002937336	0.496585998	0.92748894	0.253245	0.089275	-0.07445	YNL095C
0.002940258	0.755185708	0.741851259	-0.20011	-0.03834	-0.11666	AIP1
0.00296719	0.758880003	0.998440996	0.185222	-0.03522	0.029717	DOT6
0.002967643	0.504279413	1	-1.3914	0.455244	0.16378	None
0.002974155	0.233215762	0.084446467	-0.99067	-0.55014	-1.81091	YOL013W-/
0.002981125	0.630246179	0.30954724	1.355819	0.367476	-1.63013	GAL7
0.003015403	0.250649618	0.915496392	-0.29757	0.161875	0.09481	BMT2
0.00303553	0.004247119	1	0.213999	-0.24949	-0.02258	EGT2
0.003046891	0.847132765	0.976159573	-0.33654	-0.04523	0.069657	MFM1
0.003063786	0.152125016	0.652552516	0.333023	-0.21801	-0.24256	PDR1
0.003073259	0.410813266	1	-0.18541	-0.07687	-0.00655	YIF1
0.003078442	0.514774579	0.976568193	-0.94186	0.312974	0.192086	None
0.003089132	0.176258659	0.522788552	0.848603	-0.53668	-0.76127	YDR029W
0.003092299	0.986843508	0.080581045	0.199095	0.00825	-0.34054	PHO8
0.003101406	0.394478226	1	0.187054	0.080132	-0.02565	ENT3
0.003122282	0.921788255	0.190795777	-1.40077	-0.11673	2.072944	None
0.003127467	0.060253788	0.949031652	0.302347	-0.24814	0.077566	OAF3
0.003136008	0.68290437	0.847404354	-1.12535	-0.25896	0.510374	YGR146C-/
0.003154256	0.568378999	0.845726915	1.740235	-0.53392	-0.85262	None
0.003154256	0.63816324	0.937984736	-0.77153	-0.20347	-0.2091	SPT4
0.003158959	0.002099596	0.746737164	-0.26274	0.321658	-0.1514	ZTA1
0.003222836	0.972383608	0.89719832	-0.24251	0.012613	-0.08615	KIN3
0.003222911	0.608133376	0.457578646	-0.17121	-0.04899	-0.1681	CRN1
0.00326275	0.738785777	0.744376434	-0.77784	0.156702	0.452505	YGL204C
0.00326347	0.527787705	0.313527933	0.778065	0.261738	-0.92328	None
0.003267287	0.572927078	0.689558052	-1.0971	0.326984	0.752777	None
0.00326966	0.295242295	0.731592804	-0.70316	0.352989	-0.43127	None
0.003269877	0.301040651	0.89043028	0.503051	0.255317	0.183779	None
0.003283584	0.304299791	0.516090954	2.497504	-1.25013	2.330526	None
0.003285547	0.231636316	1	0.241907	-0.1379	0.036665	SEC12
0.003315621	0.995096172	0.666296417	0.236658	0.008493	-0.16962	RNA15
0.003326212	1.70069E-06	0.053817845	-1.69797	3.027731	-4.31052	YIL102C
0.003326212	0.04046433	1	-0.22585	0.198842	0.024465	RSM24
0.003364098	0.805737746	1	-0.33335	0.053393	-0.00408	MTW1
0.003379353	2.58336E-05	0.027182603	0.258504	0.408258	-0.53658	TPS2
0.00339323	0.120736136	0.89826709	-0.5631	0.38882	-0.20079	YBR209W
0.003425529	0.0293299	0.98500242	0.323938	-0.30555	0.060171	SPA2
0.003432279	0.004064999	0.901390836	-0.67501	0.780201	0.234013	DIA3
0.003439547	0.767134842	0.716456431	-0.19201	0.035486	-0.12203	SMF2
0.003443243	0.041921745	0.761176355	-0.23108	0.202608	0.130597	MRPL9
0.003449986	0.781988292	0.969781978	0.362153	0.063793	0.081602	CDC26
0.003456234	0.382143759	0.786584362	-0.26034	-0.1152	0.137682	EAF1
0.003476677	0.985620143	0.971609311	-0.25969	0.011186	0.056483	IRA1
0.00348977	0.065404264	1	-0.27943	0.225512	0.03601	AIM23
0.003490267	0.182884497	1	0.292601	0.182087	-0.0415	VPS20
0.003493631	0.082198263	0.046842154	-0.20899	-0.16194	0.403057	YNR029C
0.003511019	0.212379348	0.92578246	0.243036	-0.1439	-0.0737	SKI2
0.003511179	8.12485E-06	0.001631856	0.17834	0.295812	0.489296	ENO1

0.003521583	0.557155923	1	0.17382	0.055814	0.004621	YCK2
0.00352835	1	0.972387712	-0.89095	-0.02285	0.191653	PAU2
0.003545362	0.629241061	0.995247182	0.250069	-0.06908	0.042381	RKR1
0.003597663	0.759288226	0.719999469	-0.24342	-0.04687	-0.15432	SNP1
0.003614293	0.804679845	0.561585828	0.318297	-0.05216	-0.27309	TAX4
0.0036321	0.795533688	0.864764354	0.226961	-0.03834	0.094175	PSH1
0.003637858	0.38298392	0.89826709	-0.21005	0.09312	-0.07489	VPS60
0.003652591	0.050780234	1	3.531928	-3.24876	-4.44	None
0.003652922	0.888343159	0.567844831	-0.77797	-0.0853	-0.67465	None
0.003658085	1	0.081519515	0.198274	0.000438	-0.34385	LEU4
0.003659919	0.505136536	0.673355127	-0.93448	0.323166	0.66036	MEI4
0.003662237	9.11881E-08	0.283481217	-0.26579	0.51122	0.33375	SPI1
0.003698799	0.867434951	0.69828171	0.261367	0.032909	-0.17749	PBA1
0.003714358	0.027086984	0.018502313	-0.27317	0.259632	0.605122	RPS29A
0.003714358	0.186602961	0.489652715	0.209435	0.13006	0.200626	CMD1
0.003714358	0.057147327	0.615987044	0.203797	-0.1717	-0.161	LYS2
0.003725098	0.300891646	0.708809273	0.380723	0.195742	-0.25113	SRL2
0.003755925	0.673663394	0.959222058	0.20199	-0.05006	-0.05003	RSC9
0.003778649	0.601019912	1	0.95988	-0.28775	-0.06429	PTH4
0.003791652	0.298926458	1	0.243696	-0.12619	0.016006	SAC7
0.003810214	0.147800185	0.529437087	-0.29295	0.195419	0.264992	MTQ1
0.003824947	0.379077824	0.464867211	-0.23924	-0.10773	-0.23742	HOS3
0.003825338	0.782318684	0.475256698	-0.23175	0.040818	0.226937	LSM6
0.003826564	0.582144103	0.867310986	0.429051	0.132452	0.177602	PRR2
0.003834321	0.496585998	0.848379499	0.275982	-0.10008	0.122145	BCK1
0.003866421	0.269333208	1	0.28888	-0.15804	-0.04167	PAN2
0.003866855	0.916242282	0.920011392	-2.375	-0.19886	-0.79564	None
0.003869096	0.336744763	0.113134528	-0.21963	0.106094	0.360919	SRP14
0.003905378	0.039464542	1	-0.17466	-0.15788	-0.01085	GAS5
0.003905609	0.145608335	0.910199402	-1.35775	-0.90144	-0.50379	None
0.003941274	0.805302069	0.990208543	0.223652	0.036754	0.040233	PPX1
0.00396013	0.230450293	0.834791774	0.23915	-0.13941	-0.11217	UBA2
0.003967245	0.013622527	0.341937853	0.257229	-0.27323	0.301142	GLN4
0.003972848	0.051155386	0.113089839	-0.1963	0.168816	0.323635	TOM20
0.003974097	0.231604832	0.723698581	0.171448	-0.09957	-0.10834	MEX67
0.003994259	0.801071005	1	0.364241	0.061008	-0.05634	YPL067C
0.004007109	0.03630245	0.875200775	0.356687	-0.32998	0.14294	SPT6
0.004017789	0.167905851	0.862828907	0.276361	0.178967	0.116946	YDL157C
0.004032413	0.456859829	0.746636456	-0.18373	-0.07193	-0.10872	GND1
0.00405013	0.145620859	1	0.372835	-0.25476	0.045162	PEP3
0.00414983	0.135026933	0.681411478	-0.25293	-0.17571	0.179385	PMD1
0.00414983	0.065875072	0.836800595	0.538764	0.445301	-0.25059	YGR053C
0.004167688	0.552448072	0.651238461	-0.19399	0.063531	0.145972	RIM1
0.004167688	0.599853194	0.971609311	0.221125	-0.06638	-0.04936	SSL2
0.004174785	0.352413161	0.207271346	-0.22954	0.108788	0.326552	TRX1
0.004180083	0.373168526	0.16733912	0.216616	0.099691	-0.32772	TPK2
0.004180083	0.778192419	0.803017727	0.206858	0.037637	-0.10644	BIM1
0.00422965	0.062448847	0.82890057	0.297883	0.248834	-0.14272	INO4
0.004246304	0.597836233	0.532886511	-0.45087	0.134641	-0.40889	YLR112W
0.004246304	0.267158526	0.989003553	-0.43744	-0.24091	0.080684	IMA5
0.004283544	0.207400802	0.614405454	0.35821	0.218036	0.287912	YIL024C
0.00428833	0.3567311	0.735885155	-0.20977	-0.09935	-0.12946	PCL1
0.004315745	0.000630711	0.003119282	0.207686	0.286259	0.556094	AHP1
0.00431816	0.853297263	0.750071687	0.169678	-0.02325	-0.1008	RVS167

0.004322357	0.90728766	0.287597168	-0.33359	-0.03299	-0.42193	GTT2
0.004368159	0.888561341	0.971609311	0.333975	-0.03793	-0.07478	RAD1
0.004380662	0.619870553	0.872146172	0.228652	-0.06597	-0.09324	RAD7
0.004384158	0.479322493	1	-0.30635	-0.11586	-0.03213	BRL1
0.004396353	0.707431312	1	-0.25486	0.058696	-0.00061	RBL2
0.004403025	0.968013408	0.320515456	-1.59421	0.089988	-2.09289	MEI5
0.004439245	0.533524502	0.828700369	0.237047	0.081103	-0.11462	EAF7
0.004470153	0.0969018	1	0.279722	-0.21505	-0.00132	RAX2
0.00451116	0.125172898	0.033854211	-0.23139	-0.16572	-0.48222	MIL1
0.004522093	0.631878695	0.702041373	-0.2823	0.078728	0.193089	YGL159W
0.004528628	0.743899621	1	-0.23413	-0.04875	-0.00724	AKR2
0.004532687	0.658725803	0.758181494	-0.3166	-0.08303	0.185101	YHL026C
0.004535696	0.938960046	1	0.201234	0.015757	-0.01304	DID4
0.004551154	0.413532829	1	3.493383	1.314767	1.470591	Q0255
0.004556404	0.761222633	0.935686072	-0.23722	-0.04619	-0.06775	RNH1
0.004567626	0.468207431	0.757681998	-0.21816	-0.08489	-0.12806	OSW7
0.00458294	0.582169769	0.786892522	-1.04258	-0.3219	-0.56485	SPO13
0.004583412	0.276561027	1	0.405972	0.222971	-0.02006	YNL092W
0.004613444	0.260412922	1	-0.23657	-0.13312	-0.02381	POS5
0.004621717	0.080149997	0.439227871	-0.20541	0.164203	0.214782	GCN4
0.004650878	0.587398198	0.295505757	-0.25863	0.079916	-0.32416	DFM1
0.004665423	0.773263368	0.736753793	-0.21644	0.040276	0.134534	THI80
0.004671274	0.690335313	0.585232178	0.277478	0.067487	-0.23566	CHS6
0.004705468	0.571690974	0.860381997	-0.20038	0.063978	0.086728	YMR226C
0.004705468	0.252400336	0.98500242	2.031096	-1.19247	-0.53794	PRM6
0.004709416	1	0.822329423	0.20548	0.005103	0.101383	RSP5
0.00471956	0.447812948	1	-0.30126	-0.12157	0.006688	SWT1
0.004731296	0.562258463	0.575774896	-0.27052	-0.08843	0.232894	IMP3
0.004764599	0.347850933	0.98500242	-0.24351	0.117767	0.047489	PMT5
0.004771004	0.516700017	0.92538789	0.186196	-0.06634	-0.05843	SER2
0.004789651	6.58261E-05	0.236599821	0.420694	0.660266	-0.57981	ATO2
0.004833271	0.041496611	0.769965504	0.39541	0.362091	-0.22646	SNN1
0.004846412	0.276167395	0.450558485	-0.5765	-0.31675	-0.60278	YPS6
0.004869447	0.930068125	0.712488585	-0.24958	0.021195	-0.1666	MSC2
0.004881597	0.788215883	0.670294084	-0.1795	0.031918	-0.13216	RPD3
0.004996805	0.051155386	0.676735966	-0.20871	0.183745	0.152169	MRPL36
0.00502125	0.246099917	0.832740675	0.303503	-0.17689	-0.14673	BBC1
0.005046731	0.52090053	0.690321499	-0.25196	0.088861	0.178803	VMA3
0.005053477	0.644005887	0.959977162	-1.95332	0.485208	-0.57499	GAT3
0.005073602	0.854733623	0.859694751	-0.27186	-0.03737	-0.11919	VHT1
0.005084077	0.0438626	0.758383649	-0.3832	0.34599	-0.22598	NGL3
0.005088091	0.122091532	0.886876142	0.301967	-0.22184	0.118706	VID22
0.005088091	0.576536639	0.981558389	0.201466	0.064558	-0.041	ERV46
0.005108256	0.706490906	0.978193638	-1.07367	0.24578	0.224605	SMA1
0.005120153	0.019363269	0.930420244	-0.27158	-0.28218	0.08209	PPR1
0.00512338	0.666928884	0.966118712	0.28649	-0.07471	0.069591	VAC14
0.005127661	0.300021637	0.294796767	0.276661	0.147298	-0.35211	DSC3
0.005136861	0.16612431	0.318881364	-0.43853	0.28974	0.542169	None
0.005140355	0.2344813	0.705630037	-0.45281	0.265565	0.309937	MTC7
0.005163442	0.676672695	0.326601143	-0.23524	0.059476	-0.28714	GRX4
0.005189032	0.482993634	0.368959414	-0.36756	0.140117	-0.42413	DSE2
0.005189032	0.014121187	0.7990478	0.229166	0.247753	-0.12178	AMS1
0.005192395	0.914594311	0.24850108	-0.69304	-0.06582	0.960264	None
0.005207932	0.520733173	0.263024501	0.186064	-0.06636	-0.2497	SDS23

0.00520841	0.129372156	1	0.321927	0.232235	-0.01635	SCM3
0.005303665	0.500337208	1	0.32565	0.121179	0.027551	PEX14
0.005305592	0.039547582	0.486872846	-1.7974	-1.68466	-2.64533	RNP1
0.005378158	0.995096172	0.516783348	-2.78255	-0.0855	2.711823	None
0.005378158	0.407136042	0.529437087	-0.18362	0.080963	-0.17195	VTC4
0.00538068	0.690274157	0.591440527	0.234103	0.057834	-0.19961	AKL1
0.005381119	0.321761523	0.89987755	0.525462	0.271539	0.194747	YDR018C
0.005395821	0.826578265	0.160504258	-0.38783	0.060648	-0.6067	SEI1
0.005395821	0.675205495	0.764894106	-0.18269	0.046622	0.107322	YMR099C
0.005395821	0.360876565	0.951201607	0.221968	-0.10754	0.059531	PAP2
0.005427812	0.0031883	0.251662308	-0.20101	0.252935	0.275859	CPR3
0.005437789	0.601019912	0.522217116	0.711266	0.219562	0.679364	THI4
0.005467597	0.426356042	0.929303164	0.253045	0.108694	0.078047	OCA6
0.005536392	0.447812948	1	0.23431	0.096514	-0.03944	AHC2
0.005552286	0.650079178	0.978377916	0.296438	-0.08164	-0.06258	TPO2
0.005556503	0.011617566	0.700382929	0.244804	-0.27603	-0.17208	AXL2
0.00560617	0.067063438	0.488692236	0.327771	0.278399	-0.32659	PET494
0.005677663	1	0.907281575	-0.25191	-0.00641	0.089503	GRC3
0.005680503	0.661428602	0.025659714	0.353743	0.095061	-0.78317	PKP2
0.005680503	0.884827129	0.786584362	-0.19149	-0.02307	-0.10649	ENV9
0.005741195	0.060253788	0.68343153	-0.18047	0.156078	-0.13171	SDH2
0.005741219	0.04968337	0.488692236	-0.24051	0.216373	0.240609	PAM17
0.005760942	0.145608335	0.795907717	-0.17897	0.125689	0.097168	MSS51
0.005781844	0.023154413	0.834791774	-0.30126	0.307068	0.146453	IRC24
0.005792374	0.293343157	1	-0.19593	-0.10689	0.004384	RBS1
0.005818971	0.527039178	1	-3.49198	-1.04233	1.133291	YER188C-A
0.005848981	0.892236467	0.733681433	-0.19559	0.022245	0.125632	SDO1
0.005850843	0.003850882	0.482222092	0.363065	0.455148	-0.36709	ATG8
0.005884143	0.504240033	0.182165955	0.370353	-0.13871	-0.56633	GDE1
0.005896743	0.847132765	0.689558052	0.258276	0.037425	0.187876	YSC83
0.005896743	0.17265682	1	0.287612	-0.19342	-0.01635	DFG16
0.005900171	0.571093193	0.93741782	-0.32188	-0.10553	0.093036	DPB11
0.00596838	0.49382675	0.273681355	-1.09943	-0.41255	1.495541	PRM8
0.005970086	0.729652252	0.625394004	-0.27833	-0.06245	-0.22671	YJR056C
0.005981428	0.799968632	0.69844865	-0.30606	0.053274	-0.21763	YDL176W
0.005981428	0.040357896	0.863302781	0.220572	-0.20893	-0.0971	VPS16
0.005991991	0.858991565	0.699025071	-0.27206	-0.03742	-0.19339	CDC21
0.006006905	0.780949762	0.758049402	0.387043	-0.07292	-0.23414	UBR2
0.006059863	0.02681959	1	-0.29196	-0.2945	0.035666	SEG1
0.00607525	0.170043734	0.047279785	-0.18949	0.127166	0.385881	RPL16A
0.006096639	0.196724669	0.2597044	0.263701	-0.17086	-0.36266	CHA4
0.006134238	0.585153927	0.120110561	0.245749	-0.07927	-0.41773	MIP1
0.006144732	0.402148664	0.511547888	0.342605	0.155642	-0.33455	AEP3
0.006150967	0.514826882	0.143940462	-0.20446	0.074784	0.334914	GUK1
0.006156573	0.227918099	0.872290708	-0.25448	0.154807	0.107219	CAB5
0.006181822	0.039547582	1	0.222779	-0.21233	0.023946	TUP1
0.006185554	0.053398368	0.714197164	0.363444	-0.3285	0.249346	MLP2
0.006185554	0.422477621	0.87592683	-1.03869	0.441199	0.430882	None
0.006185554	0.440961232	0.930722711	-0.32879	0.138061	0.100804	NHP6A
0.00621544	0.94982823	0.643429399	-0.52837	-0.03825	-0.41773	PUS2
0.006216911	0.830118162	0.680777882	0.18252	-0.02881	-0.13553	CLB3
0.006231539	0.825517831	1	0.15483	0.024936	-0.00105	FPR3
0.006233116	0.292656551	0.680777882	0.230165	-0.12745	0.171485	IQG1
0.006237798	0.066877727	1	3.348143	-3.07076	-4.23721	PAU19

0.00632651	0.39761828	0.298558417	-0.43268	0.196597	-0.55925	CLD1
0.006333646	0.055862382	0.223191433	0.197831	-0.17735	-0.28828	LEU1
0.006351286	1	0.980342156	0.351675	-0.00739	-0.0743	FKS3
0.006353079	0.83092898	0.667588729	-0.74694	0.115376	-0.58038	None
0.006387195	0.740566016	1	-2.50787	0.475449	0.018812	None
0.006413998	0.145012799	0.822329423	0.307707	-0.22107	0.157237	BEM3
0.00642135	0.551239517	0.793455902	0.245034	-0.08492	0.136448	MPH1
0.006439087	1	1	0.232486	-0.00172	-0.01304	CUE1
0.006460132	0.987136522	0.906628341	0.302305	-0.01341	0.109838	BCH2
0.006460132	0.240168063	0.929303164	0.233596	-0.14114	0.073443	SEC31
0.00649255	0.157469667	0.934548631	-0.29292	0.202554	-0.08729	ADY4
0.00650408	0.662481265	1	0.772227	0.210435	0.013189	None
0.00651182	0.788431804	0.730473287	-0.34579	0.063084	-0.22629	ATP22
0.006512366	0.271844334	0.605911675	-0.41861	-0.23945	-0.3571	YKL050C
0.006512366	0.137340618	0.807027851	-0.21904	0.158567	0.116726	CNB1
0.00652136	0.057890074	0.385959394	-0.26055	-0.23174	-0.30205	OAC1
0.006711373	0.010883358	0.385511932	-0.21985	0.25218	-0.25585	AVT6
0.006734904	0.527787705	0.957379336	0.191596	-0.06944	-0.05087	YPK1
0.006750708	0.063507884	0.537924573	-0.34113	0.296411	-0.32217	MBR1
0.006784515	0.113397822	1	0.276077	-0.21368	-0.04461	ISW2
0.006790143	0.582123297	1	-0.24567	0.080151	0.033402	IES4
0.006805526	0.221413078	0.186752472	-0.25704	0.159641	-0.39522	ATG33
0.006805526	0.083223738	0.74594446	-0.41414	0.339963	0.25967	YGR035W-A
0.006870646	0.307144719	0.69331605	-0.36403	-0.19668	0.266527	ENV11
0.006879736	0.986843508	0.083171731	-0.44591	0.020019	-0.82765	YPL113C
0.006893007	1	0.795458324	-0.19378	-0.00521	-0.10762	GCS1
0.006921336	0.897124331	1	-3.44278	-0.31733	0.344516	None
0.006929538	0.757145555	0.689558052	-0.2705	-0.05646	-0.19936	CIN2
0.006949271	0.266200922	0.911946622	-0.25172	0.146186	0.089149	RRG9
0.006954954	0.447812948	0.990573422	-0.20597	0.086658	0.039121	ELO3
0.006978243	0.795533688	0.635310634	-0.20375	0.036673	0.166828	YNL050C
0.00700888	0.999282889	0.36188423	-0.37539	0.01233	-0.45425	DSE1
0.007021546	0.130529586	0.040053356	-0.19129	0.141573	0.410578	RPS24A
0.007037723	0.125195943	0.998341694	0.197884	-0.14955	-0.03488	NSI1
0.007055467	0.041496611	0.817868751	0.203211	0.194036	-0.1063	YBR139W
0.007073331	0.613186069	0.285413202	-0.17816	0.054835	0.239249	RSM28
0.007077005	0.125506798	1	0.205325	-0.15515	0.000317	RPC34
0.007092106	0.484784326	0.954183232	-0.20871	-0.0824	-0.05636	CDC14
0.007148339	0.199012093	0.029197458	-0.22389	0.145411	0.502389	RPL42A
0.007191221	0.011927951	1	0.358417	-0.41635	-0.0624	OAF1
0.007207781	0.341978326	0.263985261	-0.23595	-0.12133	-0.32822	RDS2
0.007267641	0.155051744	0.314660368	0.226991	-0.16192	-0.29428	SCD5
0.007269392	0.747684444	0.668242189	-0.23299	-0.05052	0.180739	YDL129W
0.007276794	0.19327492	1	0.258212	-0.17147	-0.03423	NUT1
0.007340384	0.715818525	0.668242189	0.876229	-0.21121	0.685241	YJL043W
0.007348323	0.679403914	0.501759351	0.439995	0.115836	-0.44466	CAT8
0.007455572	0.027559605	1	3.386363	3.4421	4.389389	MATALPHA1
0.007564325	0.533678741	0.98500242	0.222612	-0.08072	0.045169	STT4
0.007594683	0.628843645	0.609220601	-2.69629	0.666623	-2.80838	None
0.007618704	0.158415825	0.484474484	-2.28652	-1.57305	-2.90107	YGR164W
0.007697826	0.428998997	0.33267248	0.23268	-0.10345	0.296573	NUP84
0.007719014	0.227967015	0.971609311	0.357431	-0.22558	-0.08656	LTE1
0.007748332	0.446288659	0.978377916	0.171949	0.073796	-0.03767	HIS1
0.007768445	0.119099734	0.465091963	0.273366	-0.21259	-0.29394	HOT1

0.007829981	1	0.84918567	-0.34546	-0.00375	-0.16362	RED1
0.007845928	0.024498149	0.328122933	-0.26696	0.279037	-0.34031	ETR1
0.007854579	0.199496245	1	0.193099	0.127223	0.032525	YPT31
0.007897844	0.15988669	0.475525924	0.166181	-0.11805	0.176724	SVF1
0.007907449	0.048569622	1	-0.65342	0.604184	0.059635	None
0.007915217	0.27087925	1	-0.21101	-0.12372	-0.00603	LRO1
0.007923313	0.089289873	0.710774648	0.215442	-0.18037	-0.15414	YHP1
0.007951997	0.613304251	0.919271701	-0.36778	-0.1145	-0.12681	SPC98
0.007961855	0.979904046	0.004134287	-0.17678	-0.00923	-0.48975	BNA3
0.007981175	0.490597664	0.711541955	-0.17601	0.069408	-0.12472	SMM1
0.007989264	0.288253124	0.812812583	-0.22616	-0.12922	-0.12124	KIN4
0.007997937	0.145012799	0.463082388	0.237262	0.174045	-0.25614	HAP2
0.008037971	0.109032775	1	0.271463	-0.21662	0.032133	SEC5
0.008048937	0.267599743	0.909621954	0.273596	-0.16268	-0.1001	MYO1
0.008066961	0.758146893	0.748790103	-0.25598	0.054073	0.163153	ARO80
0.008103884	0.017926511	0.120010897	0.203097	0.224419	0.358033	QRI5
0.008104593	0.006718369	0.908303081	0.349962	0.433308	0.128708	UGX2
0.008107084	0.682492975	0.91199009	0.185717	-0.04898	0.067072	APL5
0.008119871	0.885372644	0.998804008	0.217317	0.027096	-0.03871	PER1
0.008122244	1	1	1.118029	0.020307	0.1773	YMR084W
0.008163738	0.298926458	0.612533403	-0.23566	0.131613	0.203719	TVP23
0.008177699	0.955261579	1	-2.11122	-0.13195	-0.21035	None
0.008192152	0.299260429	0.522788552	2.152798	1.132516	2.471309	YER078W-[illegible]
0.008221422	0.77416706	0.096205883	-0.20189	0.039912	0.37254	RPC10
0.008272996	0.867220499	1	-3.36609	0.373486	-0.40669	TDA8
0.008319201	0.984860432	0.702041373	0.284801	0.013888	-0.20837	OAZ1
0.008319201	0.295353255	0.911946622	0.270444	0.153561	0.098274	SOH1
0.008319201	0.972056407	0.960632458	-2.46273	-0.12779	0.716661	YCR041W
0.008332062	0.018092653	0.119906082	0.356564	-0.39872	-0.62973	PAC11
0.008462477	0.094486252	0.179756965	1.704873	1.379048	-3.58869	None
0.008477195	0.948914258	0.57885291	-0.22843	0.017356	-0.20858	COQ8
0.00851729	0.652939653	0.411976149	-0.19704	-0.0563	0.228173	DPH1
0.008527356	0.85824178	0.47055007	-2.6737	-0.32514	3.027661	YBR090C
0.0085709	2.42817E-11	2.5162E-07	0.237374	-0.61678	-1.02364	MET3
0.008641551	0.142434746	0.888371569	-0.44834	0.328722	-0.1852	MPC54
0.008661695	0.030733392	1	3.3479	3.403447	4.356244	None
0.008684985	0.083045355	0.837234982	-0.23177	0.197072	0.117175	DGA1
0.00871431	0.703189392	1	0.213539	-0.05398	0.015235	EXO1
0.008722701	0.976677186	0.931005601	0.206091	0.011525	0.065552	LSM12
0.008722701	0.034194294	1	0.218238	-0.22166	0.019231	NOT5
0.008774025	0.549432695	0.795907717	0.530722	0.191389	-0.30293	HUL4
0.008869673	0.012515571	0.737748659	0.248772	-0.29238	0.166484	KIN2
0.008892394	1	0.048861894	-0.16067	0.005061	0.340801	BRX1
0.00894557	0.582169769	0.846846052	0.1651	0.055807	0.081121	DUG1
0.008987209	0.990965259	0.363479149	-0.57799	-0.02417	-0.71193	HIM1
0.008987209	0.27789823	0.863302781	-0.23615	0.138663	-0.10836	ALG7
0.009057458	0.600450086	0.990712634	0.188266	-0.0615	-0.0369	YMR122W-[illegible]
0.009071339	0.310049773	0.673402408	-0.19705	0.10946	0.154721	YGR111W
0.009096991	0.64082321	0.649502951	-0.39241	0.115692	-0.32203	RRT12
0.009114272	0.1765456	0.176808405	0.182863	-0.12816	0.299114	SEC63
0.009143104	0.030015186	0.157913758	0.177649	-0.18534	0.300143	KAP123
0.009150325	0.500936572	1	-0.1742	0.068477	0.009558	VPS21
0.009183374	0.21537104	0.718286937	-0.19348	0.125782	0.136513	YLR118C
0.009202801	0.177242556	0.7990478	-0.2079	0.144501	-0.11792	PDE1

0.009268399	0.867986502	0.719999469	0.201904	0.028053	-0.14241	TOR1
0.009326449	0.603570842	0.926181253	-0.25132	-0.08152	0.08452	YGL176C
0.00941736	0.011022568	0.781875866	-0.59341	0.696362	-0.35655	HXT9
0.009521175	0.031370962	1	0.380311	-0.39787	0.060023	SPT10
0.009522344	0.607278799	0.723053672	-0.75063	-0.23953	-0.54375	YDR102C
0.009526733	0.016262327	0.111100599	-0.20482	-0.23363	0.374234	MRD1
0.009550325	1	0.77583136	0.187341	0.003831	-0.11455	SGV1
0.009616044	0.720422379	1	0.211734	-0.05165	0.017915	SWA2
0.009668549	0.441287224	0.895750007	0.292457	-0.13053	-0.1181	REG1
0.009742832	0.026368793	0.041389982	-0.15639	-0.16733	0.346807	RRB1
0.009742832	0.018357262	0.493774737	0.190189	-0.215	0.201556	CDC60
0.009742832	0.713873054	1	-1.31213	0.307814	0.11759	NCB2
0.009784763	0.479561477	1	-3.28898	1.121493	-1.23345	None
0.009792379	0.290286754	0.529437087	-0.18193	-0.10612	0.183379	NCS6
0.009824991	0.073128976	0.954183232	-0.20398	0.181025	-0.05721	CYT2
0.009994176	0.809230565	0.950921142	0.181354	0.032617	-0.05227	SEC72
0.010054233	0.223168514	0.960632458	-0.17247	0.112087	0.047071	ABF2
0.010059135	0.015064016	1	0.249549	-0.29089	0.022428	MSH6
0.0100897	0.071910894	1	0.203755	-0.18378	0.034768	MSA1
0.01011029	0.855703098	1	0.309661	-0.04626	-0.03859	CIP1
0.010148846	0.730766871	0.546032589	0.193588	0.046112	-0.19063	MDR1
0.010161087	0.693233428	1	0.281415	0.07409	0.028916	DCN1
0.010167473	0.763546663	0.735885155	-0.24757	0.05224	0.168738	INA22
0.010195521	0.607278799	0.711541955	-0.2009	0.065314	0.146642	PBP4
0.010251857	0.586124484	0.978338942	0.206726	-0.07068	-0.04703	GLE1
0.010252513	0.000304987	0.711541955	-0.3134	0.492178	0.227969	BLI1
0.010273895	0.000571682	0.264879577	-0.38036	0.575999	-0.54506	ICS2
0.010365681	0.023260849	0.898464555	0.278743	0.306087	0.112013	PEX10
0.010439544	0.565975794	0.647440092	-0.87418	0.302914	-0.75058	SPR3
0.010465096	0.073236167	1	0.217199	-0.19593	0.002407	SLY1
0.010498869	0.604490537	1	3.236749	0.963321	1.094998	None
0.010502169	0.080547625	0.659843375	-0.22539	0.19745	0.185679	FYV4
0.010502169	0.000649548	0.789517744	0.229606	0.349362	-0.13614	RRF1
0.010518879	0.939477921	0.92745323	-0.23689	-0.02016	-0.08013	TRK2
0.010567334	0.954336914	0.608460014	0.299051	0.022046	-0.26884	SKP2
0.010605283	0.475062233	0.962145993	0.181177	-0.0769	-0.04928	MUM2
0.010614167	0.045989617	0.69579659	0.197803	-0.19587	-0.1523	BNI5
0.010669516	0.177202487	0.446640364	0.422645	-0.3032	-0.48189	WSC4
0.010669516	0.055657167	0.867548	-0.208	0.196721	0.096227	MRPL32
0.010706249	0.828653187	0.324708204	-0.24217	-0.04081	-0.32294	CBF1
0.010794282	0.656000455	0.672563227	0.244494	-0.07175	-0.19673	UBP15
0.01089916	0.614870993	1	-0.69112	-0.22118	0.090867	None
0.010953877	0.636888736	0.394712497	0.2735	-0.08428	-0.33408	RAD54
0.010982818	1	0.119718988	-0.20783	0.00038	0.380348	RPC11
0.011015956	0.861419309	0.919926509	0.32795	-0.04817	-0.11707	YMR087W
0.011089377	0.469985444	0.070538739	-0.20007	-0.08599	0.405259	NOP8
0.011101894	0.800876337	0.664126703	-0.21171	-0.03966	-0.17351	ADE12
0.011137456	0.663621022	0.857790511	-0.28737	0.082292	0.1388	TRS20
0.011199976	0.023222947	0.907281575	0.816003	-0.93234	-0.32359	None
0.011221162	0.209863945	0.635840497	-0.18589	0.12502	0.160703	DPH5
0.01149768	0.802618659	0.993440716	-0.14957	0.027917	0.029371	SUB2
0.011659633	0.101421422	0.869100567	0.236434	-0.20046	-0.1095	PIK1
0.011805949	0.672577856	1	0.212559	-0.06018	0.020552	CDC43
0.01183444	0.625526211	0.995015134	-0.31936	-0.10126	-0.06203	YDR209C

0.011995036	0.498387456	0.525280623	-0.2501	0.102076	0.259634	CBS2
0.012042959	0.143756666	0.983370642	0.189301	-0.1466	-0.04189	PSA1
0.012066239	0.726146888	1	-0.42839	-0.10521	0.041028	PIN2
0.012070344	0.0122332	0.69956533	-0.18439	-0.22493	0.142356	RPO31
0.01209083	1	0.768691181	0.420438	-0.00738	-0.26935	FRE8
0.012128415	0.542558974	0.838974643	0.238588	-0.09049	-0.12509	SSK22
0.012454478	0.175100138	0.911946622	-0.26141	0.18924	-0.09909	YPR011C
0.012474915	0.0122332	0.967394603	-0.21714	0.263864	-0.05781	CAR2
0.012738294	0.483066965	0.47679402	-0.40605	0.1717	-0.45159	VBA2
0.012840985	0.805907992	0.865255114	0.266655	0.050082	-0.12749	YGR168C
0.012873348	0.526984154	0.840202254	0.198504	0.077818	0.104326	PIS1
0.012902617	0.53361689	0.094839053	0.238184	-0.09234	-0.46453	SSN3
0.012905033	0.261791204	0.05005618	-0.22798	0.143755	0.502159	RPL14A
0.012953436	0.633674911	0.807027851	-0.27378	-0.08607	-0.15826	ATG23
0.012962906	0.829832371	0.907281575	0.166129	-0.02868	-0.06528	MCA1
0.012976419	0.033800973	0.721655021	-0.24336	-0.25888	0.176979	DEF1
0.012984196	0.000756119	0.002606678	0.200303	0.31009	0.616283	HSP10
0.013016135	0.934332746	0.840393339	0.257622	-0.02401	-0.13523	ATG21
0.013025872	0.0122332	0.675851417	0.20677	-0.25552	0.169912	NOT3
0.013068995	0.025326351	0.643429399	0.234487	-0.26345	0.2052	SMC2
0.013089961	0.709900242	0.287639061	-0.25001	0.064895	0.359729	DYN2
0.013150175	0.812797636	0.531220921	-0.26075	0.04783	0.271213	AUR1
0.013205289	1	1	3.137478	0.041502	0.047483	ERR2
0.013263527	0.838286416	0.417425061	-0.54782	-0.0904	-0.66641	None
0.013422562	0.598147339	0.565847555	-0.53126	0.180918	0.523633	YLR342W-A
0.013422562	0.159515372	0.69828171	-0.24842	-0.18792	-0.19533	CTF18
0.013488947	0.648018557	0.87592683	-0.39233	-0.11997	0.178963	BUB1
0.013516115	0.712297953	0.202387133	-0.13095	-0.03404	0.215415	ARB1
0.013536156	0.475222943	0.73632566	0.16165	-0.07063	-0.11423	TRP2
0.013607941	0.125195943	0.769811029	0.16741	-0.13699	-0.10876	PGM1
0.013653271	0.015940778	0.301188608	-0.35295	-0.42301	-0.5026	AUS1
0.013669191	0.005632515	0.557096755	0.384169	0.514973	0.387102	FMP33
0.013746267	0.754471255	0.943062457	0.237138	-0.05455	0.074078	ULP1
0.013800064	0.669654465	1	0.279271	-0.08143	-0.04623	DOT1
0.013868744	0.455661382	0.907281575	0.446266	0.203269	-0.17669	YEL028W
0.013906756	2.94967E-11	0.027801813	0.444738	1.208026	-1.06911	TMA10
0.014027031	0.488044682	0.94443883	-0.27388	0.116191	-0.08495	FMO1
0.014042421	0.177075653	0.909554543	0.239061	-0.1769	-0.09389	STU2
0.014049137	0.836107259	0.278240376	-0.19355	-0.03263	0.286918	YDL086W
0.014049137	0.650518689	1	1.163632	-0.35924	0.12921	None
0.014061038	1	0.639357258	0.21928	-0.00613	-0.19421	SWM1
0.014113073	0.050780234	0.77583136	0.233487	-0.23579	-0.15005	RGA2
0.014113073	0.253255251	0.846846052	-0.19038	0.123162	-0.09849	APA2
0.014137091	0.384508616	0.710200613	0.541912	-0.28398	0.419304	YML003W
0.014175316	0.207250248	0.234678175	-2.59628	-1.69745	5.029321	YDR114C
0.014175316	0.985763775	0.712529354	0.216305	-0.01098	-0.16433	YNR061C
0.014419003	0.744889837	0.688444366	-0.19846	0.047176	0.161291	HTA2
0.014621853	0.563914752	0.567463342	-0.26395	0.098117	-0.26286	IMP2
0.014663401	0.427585494	0.041614907	-0.37736	0.180359	-0.87127	JID1
0.014678059	0.592871104	0.702435074	0.233646	0.082301	-0.1832	CHA1
0.014700076	0.055046053	0.04678456	-0.20126	0.19883	0.457926	RPS25A
0.014720913	0.726770499	0.98500242	0.148848	0.037648	-0.03279	LAT1
0.014766137	0.472847077	0.949130483	0.20152	-0.0896	0.061647	MCD4
0.01478099	0.82837317	0.191196271	0.179518	0.031738	-0.30355	CST6

0.01481138	0.649493592	0.335694492	-0.16006	0.049445	-0.21885	HEM15
0.01481138	0.513831581	0.384202197	1.878679	0.732102	2.681374	None
0.014840235	0.089795085	1	-0.22604	0.202112	-0.02705	YDL144C
0.014909469	0.295761003	1	0.180272	-0.11001	-0.0196	HAA1
0.014965949	0.246344291	0.053017966	-0.15932	0.105	0.355282	RPS1B
0.014967855	0.095680569	1	0.301641	-0.26893	0.017281	THO2
0.015023977	0.017064023	0.635840497	-0.20877	0.249682	0.187534	MRPL49
0.015144831	0.006732239	1	0.352261	-0.47729	-0.0248	YJU2
0.015169276	0.263785093	0.924605282	0.179684	-0.11636	0.06522	QRI1
0.015257055	0.059385215	0.969393262	0.253511	-0.25025	-0.06807	DAL81
0.015307178	0.347850933	0.574102224	0.271677	0.152005	-0.26965	GPI12
0.015321863	0.146023005	0.993440716	-0.24691	0.194866	-0.05035	MSE1
0.015389665	0.762436308	0.920384135	-0.1976	0.044233	-0.07284	YIP3
0.015503397	0.746430113	0.223381301	-0.22158	-0.053	-0.35898	PTI1
0.015567561	0.765667428	0.69579659	-0.23533	0.052087	-0.18961	ATO3
0.015621037	0.4171016	1	0.26515	-0.13153	0.008167	YDR239C
0.015696972	0.236124298	0.875100022	-0.2043	-0.13805	-0.09611	YCG1
0.01569767	0.663939309	0.744154405	-0.19349	0.057924	0.136198	YGL242C
0.015715538	0.896286063	0.713888298	0.239353	-0.03007	-0.18366	PRP3
0.015717267	0.261134686	0.507578381	0.209268	0.136556	-0.2308	MMT2
0.015717267	0.97563827	0.535437422	-1.33262	0.076394	1.46811	YNL140C
0.015731164	0.031881825	1	0.197768	-0.21924	-0.03547	ECM32
0.015751067	0.397361658	0.892047539	0.248182	-0.12798	-0.10793	MON2
0.015946965	0.730690788	0.834202861	-0.15598	-0.03933	0.086423	MNN11
0.016005234	0.434111391	1	0.185218	-0.08955	0.004402	NGL2
0.01609467	0.937908022	0.761668786	-0.26085	-0.02394	0.176654	SMP3
0.016148906	0.979425666	0.846846052	-0.58866	-0.03379	0.309898	NDT80
0.016247381	0.939931827	0.335694492	-0.22104	-0.0198	-0.30611	RAD14
0.016248198	0.090325697	0.929303164	0.289988	-0.26527	0.102223	TRS130
0.016348135	0.048588309	0.167222206	0.159185	-0.16496	0.285085	RLI1
0.016434373	0.881859811	1	-0.20208	0.028305	0.024869	AAD3
0.016451012	0.549922765	0.840006224	-0.21807	0.084512	0.118227	ERP3
0.016568181	0.09236487	1	0.229999	-0.20945	0.012318	ALR1
0.016629126	0.358812314	0.368189	0.159817	-0.08908	-0.21414	PTA1
0.016649953	0.032364503	0.031200361	0.146471	-0.16293	0.363319	BFR2
0.016681358	0.729751469	0.05005618	0.228005	-0.05836	-0.51937	PIP2
0.016694205	0.86979785	0.767199943	0.327407	0.048679	-0.21993	OXP1
0.016712562	0.460638341	0.738191837	0.152497	-0.07056	-0.11002	SLG1
0.016712562	0.730766871	0.985483713	0.156241	0.0397	0.034591	YIP5
0.016731517	0.950105874	0.672010939	-0.18839	0.015395	-0.16028	AFG3
0.016737327	0.004542745	0.241988403	-0.20941	0.292737	0.334581	ECM15
0.016778566	0.127610291	1	0.213324	-0.17874	-0.00363	TIF4632
0.016864135	0.231948367	0.617215067	0.835878	0.574713	-0.7896	None
0.016864135	0.48908547	1	0.288519	0.126347	-0.04012	BTT1
0.01691988	0.386354047	0.404345893	-0.27026	0.142165	-0.34534	PEX27
0.01691988	0.186795666	0.634900178	0.241753	-0.18057	-0.22197	CDC45
0.017126994	0.598147339	0.689558052	-0.15089	0.053508	0.12469	CBF5
0.017191696	0.456434148	1	0.1692	0.079033	-0.03042	PUP3
0.017216957	0.054834295	1	0.30008	-0.30705	-0.02015	RCY1
0.017226663	0.676672695	0.609183674	-0.30753	0.090055	0.294356	None
0.017226663	0.649028901	0.945408772	-0.23837	-0.07539	-0.07587	TRI1
0.017250798	0.634501741	0.702041373	-0.3095	0.100758	0.248465	MHF2
0.017326009	0.900344125	0.411752857	0.200009	-0.02475	-0.25401	PSY4
0.017341593	0.0104994	0.000151825	0.289753	0.378225	-1.07846	GUT2

0.017350746	1	0.293969078	0.19686	0.000532	-0.29161	CPA2
0.017415134	0.440961232	0.818523526	-0.19366	-0.09302	0.113375	ATC1
0.017473263	0.112620905	0.589139583	-0.20902	0.180999	-0.20615	CWC27
0.017473263	0.781328521	0.915496392	-0.20559	-0.04384	0.080479	NAR1
0.017519747	0.748333252	0.942108564	0.158658	0.038503	-0.0514	APE4
0.017588855	0.371129486	0.859694751	-0.20657	-0.11321	0.105877	GPR1
0.017618421	3.15242E-05	0.335356848	-0.17705	-0.33787	-0.24792	SHM2
0.017693098	0.12956062	0.563376575	-0.33584	-0.28118	-0.34699	CSM2
0.017720611	0.368160688	0.201505014	-0.87253	0.465831	-1.55614	YOR161C-(
0.017777919	0.332602552	0.164318185	-0.15914	0.093212	0.289495	RPS24A
0.017816753	0.065898417	0.910199402	0.294317	-0.2918	-0.11907	RAD50
0.017862887	0.639674212	0.870558299	0.481253	0.157018	0.235541	PDR18
0.017862887	0.052484563	1	3.080651	3.135003	4.070729	None
0.017872118	0.469895523	0.745956748	0.185377	-0.08534	-0.13189	WWM1
0.017914222	0.410849697	1	-0.19895	-0.10146	-0.03499	MKS1
0.01791653	0.335205662	0.664126703	-0.1411	0.082346	-0.12364	AHA1
0.01791653	0.94036767	0.715570823	-0.37054	0.033393	0.287448	None
0.017968665	1	1	-0.30494	-0.00887	-0.0164	MCM16
0.017970678	0.98811108	0.494558503	-0.97086	0.046478	-1.16164	None
0.017983157	0.919717643	0.868996053	-0.22811	0.024629	0.111984	ALG14
0.018101898	0.408733697	0.314080237	-0.41267	0.210017	0.600068	CSN9
0.018144815	0.615223063	0.7990478	-0.15588	-0.05379	0.09696	ARO7
0.018151076	0.454006913	0.840006224	0.193139	0.091455	0.106681	RIB5
0.018178482	0.229832807	0.38020077	-0.13492	-0.09415	0.180038	MNN9
0.018235032	0.000136614	0.736753793	-0.18349	-0.32881	-0.13487	HCM1
0.018442093	0.434023315	0.931568877	-0.24649	0.120707	0.085258	HMS2
0.018503202	0.07270256	1	-3.18933	-3.14539	3.888102	None
0.018510137	0.567411892	0.113089839	0.233745	0.089852	-0.46316	ICL1
0.018551113	0.630109602	0.41314825	0.170053	-0.05706	-0.21742	YPP1
0.018677381	2.21536E-07	0.007546041	0.209888	-0.48586	-0.61738	MET5
0.018776417	0.404461177	0.926181253	0.254062	-0.13266	-0.09395	IRE1
0.01881943	0.704007409	0.919926509	0.18665	0.052012	0.071618	FAD1
0.018821014	0.206402515	0.626092028	0.221131	-0.16192	-0.20845	AVT4
0.018821014	0.363817999	0.864591739	0.298181	0.167536	0.150619	ATG38
0.018853201	0.977693292	0.901225243	-0.68501	-0.04107	-0.29519	HES1
0.018855399	0.748550885	0.433972501	1.644419	0.390655	-2.24895	None
0.018983642	0.641977934	0.489652715	-0.2644	0.085909	0.307051	TIM12
0.019051475	0.230836023	0.971609311	-0.18518	0.129499	-0.04949	SHE9
0.019136287	0.022992267	1	0.227191	-0.27304	0.042047	CHS3
0.01916826	0.682008236	0.87592683	-0.23159	0.068029	-0.11087	REX3
0.019221359	0.330719721	0.885657987	0.228028	-0.13661	-0.10615	MDM1
0.019291652	0.571956214	1	0.215603	0.082362	0.029372	IWR1
0.019344016	0.833783236	0.46824606	0.192466	0.034551	-0.23132	NCW2
0.019344016	0.123442499	0.976568193	0.294808	0.254	0.075058	ATG32
0.019428189	0.027303176	0.268923553	0.219443	0.255977	-0.3433	GPT2
0.019448665	0.754604732	0.259904873	0.183121	-0.04416	-0.29098	HAP1
0.01964179	0.608514335	0.710913844	-0.23674	-0.08402	0.190223	MON1
0.01969883	1	0.683800732	-0.2104	-0.00261	0.1798	YKE2
0.019756958	0.230929934	0.545272974	0.140253	-0.09908	-0.15139	ILV5
0.019812664	0.041913401	0.971609311	0.321289	-0.35238	0.086571	SLK19
0.01981356	0.075962526	0.976378097	0.289207	-0.28325	-0.07433	MRC1
0.019884495	0.667862767	0.623228732	0.24489	-0.07544	-0.23386	CHL1
0.019919844	0.451996377	1	0.278823	-0.13479	0.017698	MAK31
0.020010277	0.04046433	0.90423793	0.337064	-0.37303	0.145257	ENT4

0.020182527	0.015713327	0.7990478	0.181855	-0.23168	0.115264	GWT1
0.020219918	0.579364291	1	0.194675	0.073918	0.038327	LAS1
0.020241427	0.836107259	0.637177071	0.336894	-0.06019	0.31641	FRE5
0.02034587	0.935433283	0.00883753	1.296001	-0.12593	-4.66333	None
0.020361893	0.929795254	0.931005601	-0.21913	0.02232	-0.07743	HEM1
0.020390194	0.113957872	0.868996053	-0.17973	0.15876	0.09	TUM1
0.020390194	0.392504595	0.927845118	-0.1647	-0.08845	0.060634	TOS6
0.020390194	0.044199429	1	-0.2211	0.238454	0.01145	SNG1
0.020444371	0.51219348	0.046942988	-0.14518	0.062324	0.346248	SAT4
0.020509834	0.0969018	0.165466155	-0.42105	0.385219	0.777221	YPT53
0.020541724	2.64271E-08	0.888649502	-0.40074	0.974414	-0.18371	YER121W
0.020607074	0.492341773	0.283481217	-0.17612	0.078435	0.273183	PHO88
0.020607074	0.905759808	0.790203342	-0.14002	-0.017	0.090961	SRM1
0.020638696	0.729751469	0.92806956	-0.5904	-0.15427	-0.21873	YGL230C
0.020693798	0.51219348	1	-1.08603	0.456585	0.072388	ATG39
0.020719036	0.079546525	0.067818165	-0.16324	-0.1584	0.363527	RRP9
0.020807665	0.738978817	0.815666048	0.168275	0.043014	0.102275	YNL010W
0.020909908	0.448302145	0.971609311	0.155603	-0.07589	0.042091	CRM1
0.020920975	0.587680144	0.406901206	0.227337	0.085303	0.299236	HMI1
0.020938524	0.428439515	0.771051473	0.145733	-0.07392	0.100327	LHP1
0.021026516	0.762220096	0.929303164	-0.21441	0.050316	-0.07825	BUB3
0.021048707	0.209974178	0.614405454	-0.19384	0.142088	0.189558	SSH1
0.021104554	0.625111025	0.639357258	-0.25947	-0.08936	-0.24295	PDR3
0.021134791	0.130061341	0.846591233	-0.21927	0.187385	-0.12064	YNG2
0.021297843	0.020785019	1	0.285491	-0.35411	-0.05592	FIR1
0.021316996	0.129927096	1	2.927348	-2.66199	-3.78039	YHR214C-I
0.021337728	0.000101322	0.739091337	0.221914	0.411151	0.165761	YHR138C
0.021397583	0.2339396	0.846845309	0.225305	-0.1607	-0.12451	TRS65
0.021426318	0.49382675	0.721271027	0.234567	0.105316	0.184447	MDM32
0.021569876	0.95467013	1	-0.21857	0.017594	-0.02459	RRP42
0.021631803	0.041877885	0.439260184	-0.17833	0.195999	0.22616	ISD11
0.021694122	0.467087012	0.911946622	0.265647	-0.12669	0.109813	SOV1
0.021824231	0.228288339	1	0.236543	-0.17086	0.047734	KOG1
0.021824231	0.732997545	1	-0.2084	-0.05453	0.028549	MTE1
0.02184039	0.185910487	1	0.286086	0.221425	0.004479	SAW1
0.02190314	0.842356676	0.218256111	-0.14095	-0.02465	0.243186	RMT2
0.02190314	0.004542745	0.220954671	0.693444	1.015857	1.209635	YDR182W-/
0.021930009	0.443553864	0.438612707	0.179828	-0.08935	-0.2291	NPL6
0.021959505	0.987928469	1	0.202585	0.010389	0.017258	SYN8
0.021998468	0.095680569	0.021504559	-0.20081	0.187435	0.543297	RPP1A
0.022090529	0.07270256	0.986945047	0.20595	0.205433	-0.04685	MEH1
0.02218095	0.613747706	0.578113103	0.191284	0.06856	0.200113	FMP41
0.02218095	0.769795979	0.733681433	-0.16296	-0.03741	0.124391	HAM1
0.02218095	0.051723227	0.786137812	0.272226	-0.29191	-0.18158	SWE1
0.022189532	0.228288339	0.723935587	0.19862	-0.14377	0.15554	GEA2
0.022209707	0.663621022	0.91199009	-0.22928	-0.07255	-0.09452	RIT1
0.022238272	0.958961218	0.822329423	-0.21065	0.016308	-0.12625	SPP1
0.022539508	0.884663483	0.259295923	0.134054	-0.01945	0.219205	YNL190W
0.02255029	0.149952856	0.241434728	0.926451	0.768695	1.574494	None
0.02255029	0.871135401	0.860187522	0.367352	0.056984	-0.19441	YMR141C
0.022592102	0.428520424	0.299842252	-1.12824	0.553741	-1.82136	None
0.022592102	0.6123064	0.43642005	-0.20191	0.072581	0.259199	BOL2
0.022695579	0.152035774	0.859769494	0.19513	-0.16275	0.103897	HEH2
0.022759533	0.587866346	0.203404731	-0.19481	0.073582	-0.3431	ISA1

0.022849453	0.025661604	0.393651782	0.444543	-0.54363	-0.60199	CMR1
0.022851533	0.216269794	0.974692539	-0.26745	0.196169	-0.07128	SNZ1
0.022865235	0.663252335	0.976568193	-0.25061	0.079618	-0.06469	SLM5
0.022880825	0.058914883	0.099133414	0.286667	0.299488	-0.59929	PTC6
0.022898653	0.863630398	1	-3.01248	-0.42292	0.469931	YNL184C
0.0229361	0.413532829	0.621769539	0.247455	-0.13074	-0.24229	FAB1
0.022983827	0.138242749	0.053709945	-0.17883	0.152526	-0.42176	TIM22
0.022984576	0.019248218	0.022248686	0.141887	-0.1793	-0.38368	ADE3
0.022988835	0.015182854	0.643429399	-0.2352	0.303047	0.221949	AIM36
0.022988835	0.124190791	0.920656396	-0.24432	-0.21574	0.095716	BNR1
0.023082402	0.762436308	0.716456431	-1.20112	-0.27924	0.997828	YBL100W-(
0.023082402	0.002243865	1	-2.17389	-4.32427	2.306079	PAU4
0.023088473	0.995958449	0.80413244	0.661216	-0.02897	-0.4214	MEK1
0.023113826	0.603384401	0.488692236	0.248415	-0.09186	-0.29794	SRO7
0.023164027	0.444779801	0.721655021	0.140432	-0.07012	0.11143	YDR341C
0.023336499	0.091928673	1	-0.27417	-0.26198	0.012481	HKR1
0.023339168	0.995958449	0.471735065	-0.25284	0.010957	-0.3106	STN1
0.023467074	0.199012093	0.924605282	0.256787	-0.19742	-0.09946	RAD53
0.023632692	1	0.889649609	0.793858	0.01304	0.374817	None
0.023808167	0.788420725	0.931005601	-0.35148	-0.07627	-0.12742	RDS3
0.023909673	0.343034258	0.213690034	-0.18712	0.112408	-0.3282	SPG5
0.024024493	0.098230855	1	-0.17415	0.163695	0.012158	YBL055C
0.024038334	0.204118763	0.154422599	-0.22401	0.169395	0.432553	OST5
0.024058872	0.24298702	0.753751911	-2.09572	-1.42924	-1.34952	None
0.024081366	1	1	0.238693	-0.00763	0.021224	PGA1
0.02426732	0.799968632	0.630792731	-0.21686	-0.0455	-0.21059	ORC4
0.024368719	0.905759808	0.931005601	0.202138	0.025264	0.073741	YGR017W
0.024403937	0.000250252	0.286953753	-0.14791	0.266581	0.233416	MRP51
0.024458994	0.431584062	0.990208543	0.159268	-0.08239	-0.03605	CDC53
0.024466454	0.996753756	0.949031652	-0.28121	-0.01186	-0.09248	MME1
0.024682543	0.663202067	0.931005601	-0.22391	-0.07219	-0.08184	TFB2
0.024849432	0.774584831	0.976568193	-0.87448	0.197911	0.228797	None
0.025001961	0.451993361	0.338005227	0.154731	-0.07728	0.229492	EMW1
0.025009521	0.411661642	0.924605282	-0.22468	-0.12039	0.08753	ALY2
0.025193186	0.533404723	0.909621954	0.184319	-0.07888	0.07886	MDM20
0.025270828	0.717701684	0.837446458	-0.18579	0.051867	0.10856	TGS1
0.025663582	0.473710488	0.863011842	0.25109	-0.12114	0.134372	SLF1
0.025838254	0.035269944	0.967394603	-0.18004	-0.21046	0.05341	TRM5
0.025927745	1	0.404345893	-0.14532	0.004294	0.198408	EMG1
0.02593313	0.459480897	0.882817665	-0.2373	0.116925	-0.11616	PRP38
0.026057743	0.35708254	0.889649609	0.144578	0.086362	-0.0687	ARC18
0.026113038	0.035269944	0.984628847	0.147413	-0.17309	-0.03621	NAP1
0.026159097	0.805737746	0.104188468	0.167959	0.034989	-0.3569	PFK27
0.026186457	0.207215871	0.661759663	0.188201	0.144248	0.176252	MRPS5
0.02626568	1	0.868996053	-0.53142	0.009896	0.277684	None
0.02627928	0.628092366	0.910199402	-1.32818	-0.46146	0.593634	None
0.026282466	0.944196518	0.984628847	-0.21655	0.020029	-0.05307	MSR1
0.026312964	0.490597664	0.796888121	-0.86676	0.39395	0.572354	DGR1
0.026312964	0.97665513	1	-0.29455	0.019101	0.041416	None
0.026315064	0.022529204	0.937944825	-0.19627	0.24576	0.068921	MRPS8
0.026315064	0.665829866	1	0.142249	0.046067	0.023706	PRO1
0.026477867	0.114955379	0.737036218	-0.24048	-0.22165	0.186936	PPZ1
0.026526934	0.497910598	0.600124333	-1.06819	0.468683	-1.1726	BOL1
0.026533651	0.029711358	0.358747602	-0.13961	-0.16875	0.203376	RRP3

0.026566684	0.467895636	0.385959394	-0.26634	0.129734	-0.37208	MLH2
0.026588421	0.891627765	0.981181758	0.13841	-0.01941	0.0351	SUA7
0.026588421	0.343957891	0.996250082	0.15673	-0.09621	-0.03393	MCK1
0.026886012	0.977693292	0.69828171	0.207994	-0.01337	-0.18176	SNU56
0.026886012	0.136029049	0.992955891	0.181631	-0.16086	0.040712	IRC5
0.026957387	0.280215048	0.846605423	0.226774	0.155507	-0.12986	KHA1
0.026968925	0.530999534	0.589775129	-0.47953	-0.20693	0.5136	MAK3
0.026968926	0.38101428	1	0.192749	0.111159	-0.01531	RRN9
0.027040454	0.272944905	0.708495578	-0.21743	0.15003	-0.18429	TRP1
0.027088419	0.463310994	0.719999469	0.184745	-0.09165	-0.15152	USA1
0.02716158	0.724702775	0.860909654	0.180752	-0.05051	0.098381	TSR4
0.027225234	0.601388298	0.276492603	0.174948	0.066438	-0.28635	RBK1
0.027225234	0.058595183	0.282780148	-0.19381	0.207343	-0.31352	YMR262W
0.027360301	0.509434188	0.624068032	0.166979	-0.07583	-0.16748	YHR127W
0.027511742	0.867434951	0.974554555	0.295497	0.047977	0.081683	PAC1
0.027567565	0.412238661	0.91703897	0.34319	0.187226	-0.14295	CMG1
0.027685665	9.33296E-05	0.583172047	0.147129	-0.28726	-0.15822	ACO2
0.027694545	0.680755208	0.320515456	0.177263	0.055609	0.274325	BUD32
0.027717549	0.265919992	0.259414235	0.155351	0.109529	0.262484	RPL4A
0.027753346	0.424705765	0.439260184	-0.77936	-0.41277	-1.07886	PUG1
0.027771205	0.996753756	0.816819753	-0.19722	-0.00852	-0.12472	SWD1
0.027809876	0.87305028	0.434655336	0.280467	-0.04463	-0.37282	MRX4
0.027809876	0.186602961	0.863626849	-0.13563	0.108516	0.07285	TIM50
0.027809876	0.231864508	0.94082376	0.174331	-0.12988	-0.06101	OCA1
0.028071102	4.508E-06	0.578113103	-0.17959	0.39502	0.194276	STF1
0.028131895	0.742522958	1	-0.25188	0.066599	0.008965	NOP1
0.02818802	0.00018969	0.168268201	-0.20718	0.38718	0.400316	CBP4
0.028194949	0.682492975	1	-0.38894	0.120742	0.020518	YET2
0.028354411	0.168234936	0.74594446	-0.18328	0.151823	0.13998	GCG1
0.028414872	0.425297662	0.690809523	0.237185	0.126917	0.212123	JAC1
0.028421572	0.124795835	0.167974771	-1.29575	-1.18512	-4.10221	None
0.028469314	0.140485465	0.859694751	0.14878	-0.13156	0.08232	FCP1
0.028491219	0.082985083	0.039795632	-0.16572	0.166712	0.431333	RPL18A
0.02858112	0.716284575	1	0.217815	-0.06277	0.02371	CNM67
0.028622167	0.054541794	0.961437745	-0.3248	0.354203	-0.10206	YBL039W-[illegible]
0.028622167	0.413532829	1	-0.36922	0.199663	0.071149	None
0.028686449	0.959405766	0.803017727	0.244422	-0.01975	0.161703	DCD1
0.02884946	0.965705414	0.848379499	-0.72437	-0.05462	0.414732	None
0.028868641	0.767194808	0.372850575	-1.40952	-0.33405	2.11556	YIL046W-[illegible]
0.029214969	0.413532829	0.843716147	0.210422	-0.1156	-0.1238	TFB1
0.02928008	0.792133624	1	-0.24488	-0.0544	-0.0425	ART5
0.029396839	0.889489616	1	-0.26627	0.038236	-0.05252	YAE1
0.029438447	0.100736221	0.553808105	0.223555	0.216472	-0.25345	PCP1
0.02947077	0.301448204	0.803025975	0.147335	-0.09881	-0.09767	PDX1
0.02947077	0.58766051	0.98500242	-1.03275	0.397019	-0.25936	None
0.029508502	0.593217132	1	-0.20135	-0.07866	-0.00902	MRH4
0.029601088	0.059378831	0.047172169	-0.17647	0.190962	0.445649	RPS13
0.029673188	0.614870993	0.316895602	0.131825	-0.04932	0.207712	YHR020W
0.029829564	0.727504645	0.358747602	0.42917	0.121264	-0.63822	SPO20
0.029844908	0.022302219	0.037021588	-0.175	0.224195	0.462705	SBH2
0.029861042	0.56995474	0.001905297	1.228654	0.50431	4.861566	YOR268C
0.029915416	0.637757348	0.384604173	0.213893	0.07597	-0.30669	GYP1
0.029929041	0.954188947	0.702041373	-0.20022	-0.01712	-0.17543	YOS9
0.030001544	0.047847499	0.733389979	0.822614	0.935515	-0.66896	YGL007C-[illegible]

0.030072327	0.987488335	0.781875866	-0.18689	0.010121	0.132104	EMP65
0.030085581	0.629304116	0.953041258	2.070361	0.704302	0.866817	YCR050C
0.030185655	0.98474138	1	-0.14113	0.008234	0.010664	HMO1
0.030215904	0.122042485	0.661976013	0.233486	-0.21732	0.224232	MYO4
0.030259102	0.971037758	0.343265986	0.190249	-0.01364	-0.28799	APL3
0.030281411	0.38101428	0.609183674	0.185941	-0.10962	0.195183	GPI8
0.030298659	0.318264058	1	-0.25736	0.167268	-0.00229	CTO1
0.030302955	0.678241823	0.917145138	-0.38764	0.123266	0.162955	COA4
0.030398044	0.880612135	1	0.262595	-0.04094	0.036156	NUM1
0.030475182	0.284831522	0.825508264	0.343185	-0.23989	-0.21534	SRB8
0.030635284	0.061867898	1	0.262699	-0.28695	0.009368	AVO1
0.03068606	0.653029567	0.821197283	-0.20541	-0.07034	-0.13047	CUE2
0.030899887	0.903919288	0.957379336	-0.26595	-0.03516	0.087064	QDR2
0.030949991	0.319484149	0.87592683	-2.35178	-1.44037	-0.44584	FYV5
0.030949991	0.948375626	0.888371569	-0.16293	0.01494	-0.08043	VPS75
0.03101692	0.723141015	0.523868802	-0.16497	0.04707	0.19799	EMC10
0.03101692	0.118958546	0.712529354	-0.14262	0.133563	0.121966	NPC2
0.031030393	0.596380201	1	0.190655	-0.07506	-0.02856	CLB5
0.031077046	0.885372644	0.714823939	-0.17639	-0.02655	-0.14993	TFC1
0.031097951	0.170739874	0.883095126	0.28587	-0.24188	0.144722	COG6
0.031248702	0.156939743	0.794898864	0.229889	-0.2001	-0.15828	RSE1
0.031541774	0.167232142	1	0.331168	-0.28301	-0.05908	SPC97
0.031699734	0.940649911	0.69828171	-0.17635	-0.01737	-0.15802	PTC4
0.031726572	0.858991565	0.822329423	-0.54391	-0.09341	-0.34574	PAU7
0.031791679	0.945012036	0.758181494	0.297893	-0.02845	-0.22608	RSF1
0.031820143	0.167183262	1	0.269857	-0.2306	-0.00764	PKC1
0.031830862	0.003849025	0.967394603	-0.59133	0.915028	-0.18421	SPS22
0.031910428	0.622976577	0.000827407	-0.1545	0.057139	0.585256	SNU13
0.032143319	0.562772918	0.69828171	-0.18383	0.077463	-0.16499	CAN1
0.032400137	0.120736136	0.463829525	0.227377	-0.21519	0.30074	ESC2
0.032843591	0.064354475	0.406058995	-0.33616	0.362885	-0.47267	ATG40
0.032843591	0.283952102	0.904680493	-0.12994	-0.09141	0.059591	TOM70
0.032904256	0.270560696	0.278641706	-1.80402	-1.25753	3.54103	YBR072C-A
0.032904256	0.351200486	0.603045192	0.249532	0.15658	-0.26657	PCL8
0.032951283	0.736287769	1	0.88114	-0.24756	0.023223	YOR186W
0.032973723	0.666883176	0.490932171	-0.13969	0.046602	0.177165	GAR1
0.033005584	0.120790719	0.014001126	-0.17199	0.161703	0.525366	RPS30A
0.033084062	0.752831669	0.879698394	-2.00725	-0.4833	1.191967	None
0.033182914	0.774791949	0.959222058	0.352913	0.085448	-0.11616	RRI1
0.033256765	0.109495914	0.967394603	0.231309	-0.22562	-0.07159	EXO84
0.033340454	0.10961715	1	-2.88261	-2.83991	3.552773	None
0.033344185	0.176571598	0.286613943	-0.67685	0.559335	-1.11907	YOR343C
0.033344233	0.805302069	0.579562352	0.328877	0.071671	-0.36598	NBP1
0.033480239	0.383767265	1	-2.82346	1.498184	-1.72186	None
0.033480492	0.689168252	0.070387453	-0.39542	0.124511	-0.93715	GAL10
0.033589747	0.175236952	0.202387133	-0.86563	0.708184	1.696687	None
0.033965543	0.014121187	0.058656331	-0.16089	-0.22465	0.398861	UTP14
0.034044688	0.96919526	0.967394603	-0.18676	-0.01395	-0.05784	DIE2
0.034105521	0.382579933	0.167446707	-0.24164	0.143589	0.483677	YOS1
0.034105521	0.860071452	0.785906893	0.448434	0.078585	0.321405	None
0.034105521	0.746430113	0.99763155	-0.39829	-0.1076	-0.08834	YCR006C
0.03415884	0.644005887	1	-0.19094	0.067924	0.01672	NAT5
0.03425086	0.342455246	0.860899891	0.943009	0.602894	0.542505	None
0.03429036	0.028520862	0.617215067	-0.24593	-0.31295	-0.25906	ATG11

0.03429036	0.786618236	0.781154036	-0.13905	-0.03239	-0.10079	YPR148C
0.03429036	0.270551983	0.833104829	0.961511	-0.71438	-0.61405	YGL262W
0.03429036	0.017246708	0.986500461	-0.20676	0.280541	-0.05067	AIM11
0.03434086	1	0.326434921	-0.1814	0.002633	0.288218	CDC36
0.034357378	0.033709464	0.053817845	-0.4292	0.52244	-1.08297	HVG1
0.034515193	0.075412455	0.698712509	0.242519	-0.26023	-0.22073	KEL1
0.034575279	0.734744884	0.793556588	0.234314	-0.06622	-0.1645	DUF1
0.03468023	0.498387456	0.939620174	-0.29562	0.141537	0.108057	YLR361C-A
0.034762284	0.505136536	0.10494638	-0.17303	0.08198	-0.38476	EKI1
0.034816062	0.100684361	0.937363635	0.123913	-0.12388	0.046027	OST1
0.034889069	0.930422285	0.665743457	-0.23622	0.026156	-0.22962	PML39
0.035074625	0.059385215	0.559860815	-0.19688	-0.22044	0.228703	TRL1
0.035152337	0.08749735	0.523868802	0.20577	-0.21424	-0.25283	SHE1
0.035216869	0.344098597	0.817868751	0.181506	-0.11698	0.119846	ORC5
0.035393825	0.479241576	0.993463468	-0.15872	0.079524	0.036894	ARF2
0.035439004	0.057915483	0.971609311	0.268508	-0.30506	-0.08007	HAL9
0.035526202	0.905759808	1	0.18461	-0.02466	-0.0027	TRM13
0.0355747	0.119872507	0.379660556	0.255275	0.245048	-0.37923	INP1
0.035587589	0.939749928	1	0.194209	0.019844	0.017023	PRP5
0.035608125	0.977201635	0.780254446	-0.163	-0.01106	0.119723	GCD14
0.035613577	0.048237469	0.968819743	0.242885	-0.28644	-0.07486	MMS1
0.035629118	0.038719477	0.87592683	0.223545	-0.27379	0.118785	BNI4
0.035687445	0.533524502	0.889559014	-0.18318	0.082619	-0.0921	MDY2
0.035704344	0.919676161	0.127873691	0.185939	-0.02262	-0.39922	RUP1
0.035863562	0.12748662	0.822329423	-1.99535	-1.89513	-0.83317	None
0.036225078	0.693075842	0.937363635	-0.26158	-0.08285	-0.0979	YDR132C
0.036225078	0.900344125	1	-0.13367	-0.01854	0.007309	PRS5
0.036368227	0.738154396	0.995979951	0.20751	-0.05849	0.047614	ERJ5
0.036411113	0.686382379	0.068005273	0.210004	0.068166	0.513874	TAH1
0.036468684	0.050924397	1	0.159168	-0.18595	-0.01233	GCN20
0.036642779	0.000620029	0.624660139	0.120745	-0.22344	-0.12723	ILV3
0.036672941	0.92384638	0.904680493	-0.24291	-0.02862	0.113702	EGH1
0.036677165	0.293118671	0.354530822	0.174134	-0.12342	-0.26925	TIR3
0.036679511	0.026368793	0.538130573	0.215	-0.28173	0.260578	YLR419W
0.036693566	0.692447241	0.781154036	0.162423	0.051813	-0.11961	NFS1
0.037080361	0.045679861	0.98500242	0.164487	-0.19681	0.04204	ISW1
0.03712969	0.628366284	1	0.215399	0.081357	-0.00891	FUN19
0.03735232	0.995958449	0.488400105	0.383582	-0.01806	-0.49968	PIG1
0.037416129	0.897429809	1	0.171878	-0.02457	0.014955	PEX29
0.037542512	0.295649423	1	0.205436	-0.14545	0.012904	BIK1
0.037628066	0.562822273	0.085672111	-0.12955	0.056174	0.304191	GIS2
0.037822899	0.661068682	0.259414235	-0.14949	-0.05213	0.26583	MSS116
0.03790395	0.971257987	0.075701779	-0.56346	-0.04141	1.367496	None
0.038074303	0.167118139	0.910058751	0.145599	0.127837	-0.06659	PDB1
0.038108024	0.385773851	0.550781538	-0.35301	0.211657	0.420143	SOM1
0.038250666	0.02140102	0.99135025	-0.20997	-0.28445	-0.05065	KIP2
0.038600882	0.653413831	1	0.255371	-0.09141	0.033207	CIK1
0.038613276	0.215332477	0.053017966	0.162548	0.131243	0.422843	RPS22A
0.038619625	0.592871104	1	-0.67239	-0.2733	0.017914	None
0.038728208	0.782318684	0.297637142	0.232316	0.056142	0.391824	PEX17
0.038784952	0.065404264	0.536945233	-0.2055	-0.23004	0.251149	SVL3
0.038786966	0.043837937	0.888091236	0.614346	0.742096	0.320045	YGL015C
0.038791313	0.768238502	0.319274731	-1.40921	-0.33945	2.465345	None
0.038791313	0.484270861	0.346217123	-0.26065	0.131094	-0.41061	YJR011C

0.038800137	0.398444163	0.293969078	0.189264	-0.11211	-0.31938	FCY2
0.038931935	0.317033104	1	-0.29175	0.198476	0.024934	CDD1
0.038973395	0.970974534	0.099020366	-0.43378	-0.03222	-1.01594	FHN1
0.038977274	0.488031192	0.040274266	-0.14685	-0.07364	0.402862	MAK11
0.039024751	0.005319949	0.992021238	0.288383	-0.4636	-0.06985	SPH1
0.039037679	0.751354579	0.946806644	0.243011	-0.06695	-0.08809	RAD18
0.039074361	0.663939309	0.670294084	-0.18776	0.065109	-0.18422	GPI14
0.039200071	0.794120955	0.929303164	-0.24544	-0.05716	-0.09923	YOL107W
0.039261251	0.374953133	0.069729831	0.185788	-0.11608	-0.45686	NRK1
0.039276518	0.68602246	0.911367439	-0.85496	-0.27731	0.402394	YGL006W-/
0.039369704	0.058914883	0.921286576	0.265043	0.303678	-0.11269	YDL199C
0.03941765	0.135418484	0.897313673	0.273437	-0.26038	0.136154	None
0.039498016	0.663621022	0.993440716	-0.3175	0.110344	0.075477	YHC3
0.039505126	0.795674553	0.904680493	-0.19283	0.044493	-0.09127	NMD4
0.039514073	0.909690256	0.531865712	-0.19256	0.025525	0.238185	RAI1
0.039626593	0.03445693	1	1.485163	1.902681	-0.16556	None
0.039682049	0.826578265	0.204412156	-0.45525	0.093706	-0.89744	YPR053C
0.039706981	0.382001694	0.326601143	0.207512	-0.12847	-0.33933	MPS2
0.039759618	0.455871763	1	0.120602	0.064672	0.021365	SRV2
0.039939117	0.585786203	1	-2.78054	-1.09121	1.246906	None
0.039948198	0.033939999	0.605911675	-0.4279	0.534967	0.471146	BRP1
0.039985097	0.995096172	0.250804898	-0.20929	-0.01024	-0.38174	MPA43
0.039991546	0.885372644	0.931005601	-0.17034	0.026697	0.067717	SPT15
0.039991546	0.183508019	1	2.664012	-2.40762	-3.4911	None
0.039991546	0.183508019	1	2.664036	-2.40765	-3.4911	YLL006W-/
0.040020734	0.472952463	1	-0.12804	0.066432	0.021502	RRP7
0.040328877	8.30217E-05	0.580281763	-0.20695	0.428708	-0.23713	YIL055C
0.040822298	0.099966513	1	2.734613	2.786982	3.710858	None
0.041082982	0.861967024	1	-0.17043	0.030336	-0.00285	YPQ2
0.041101858	0.554264996	0.971609311	-0.11723	-0.05251	-0.03548	SSO2
0.041309418	0.059385215	0.492151956	-0.16997	-0.19599	0.224106	PHO90
0.041309418	0.076101547	0.745371404	0.194784	0.214419	0.159722	SUS1
0.041309418	0.752831669	0.76101686	-0.1743	0.047971	0.137885	USE1
0.041309418	0.904357451	0.937984736	-0.18067	-0.02506	-0.06867	TPN1
0.041372988	0.488044682	0.929303164	0.177248	-0.09015	-0.07239	YGL114W
0.041643305	0.102478613	0.006151703	-0.22696	0.231578	0.781594	MHF1
0.041734789	0.486588283	0.976378097	-1.01126	-0.5077	-0.30309	PAU9
0.041735321	0.717157234	0.962684605	-0.14805	-0.04529	-0.04909	PUS9
0.041735321	0.188337033	1	2.655084	-2.39755	-3.48602	None
0.041735321	0.619870553	1	-0.16738	-0.06558	0.031082	HUT1
0.041751701	0.88355725	0.648534119	-0.27786	0.044843	0.288546	JJJ3
0.041756162	0.679403914	0.852767171	0.252	-0.08549	0.152563	FAR11
0.041810893	0.235614716	0.199681756	0.153772	0.12208	0.305618	NTF2
0.041915679	0.766157173	0.768691181	-0.23558	0.06119	-0.18323	CCE1
0.042053089	0.302532438	0.39760733	-0.12469	-0.08854	0.187225	KRE2
0.042297196	0.452963704	0.714788215	-0.1808	-0.09852	-0.16246	INP51
0.042377824	0.062448847	0.666296417	-0.28144	-0.32345	-0.28472	SPC24
0.042611843	0.059305878	1	-2.71614	2.994852	-3.91196	None
0.04262536	0.051155386	0.339657587	0.141507	-0.16988	0.229951	MES1
0.042671771	0.483079932	0.041756428	0.18442	0.095265	-0.50783	CEM1
0.042671771	0.460298273	0.224875404	-0.2923	0.156852	0.558275	YCL012C
0.042671771	0.414293185	0.711541955	0.122912	-0.072	0.112095	KES1
0.042671771	0.985620143	0.748790103	0.175284	-0.01068	-0.14336	FMP32
0.042750416	0.829740566	1	-0.17401	0.036207	0.012021	NSE1

0.04284629	0.03264687	0.00166885	-0.17018	0.219269	0.650317	RPL37B
0.04284629	0.501367304	0.971609311	-0.16022	0.079458	0.048918	DET1
0.04284629	0.740750315	0.971609311	-1.05895	-0.29537	0.351475	None
0.043039681	0.530999534	0.223381301	0.292361	0.137901	-0.55955	YDL057W
0.043114968	0.27087925	0.276169893	-0.14185	0.106864	0.25269	PFY1
0.043114968	0.278286675	0.611223762	-0.21734	-0.16236	0.242146	YNR065C
0.043114968	0.261182253	0.942108564	0.130652	-0.10075	-0.04918	HRB1
0.04337704	0.231604832	0.074036394	0.11295	-0.09109	0.281154	CCT2
0.043434073	0.00502594	0.410354533	0.158119	-0.25929	0.235544	NEW1
0.043434073	0.032210521	1	-0.24488	0.316562	0.041228	LIP2
0.043463482	0.731503094	0.379660556	0.420387	0.125099	-0.64855	HOP1
0.043509793	0.955739843	0.7990478	-0.12542	-0.01127	-0.09006	ABP1
0.043607694	0.017246708	2.0367E-05	-0.19638	0.279038	0.940892	RPL41A
0.043654829	0.721964399	0.726816534	0.192382	-0.05893	-0.16812	DML1
0.043726661	0.808656261	0.23756346	-0.55451	0.123862	-1.05719	SPS2
0.043838946	0.977693292	0.838974643	-0.14379	-0.01	0.092224	YER156C
0.043865488	0.586204562	0.967394603	0.293039	-0.12602	-0.09585	YJL132W
0.04440229	0.024098511	0.320515456	-0.28027	0.380527	-0.4667	MEP1
0.044540233	0.562638241	0.033854211	0.161781	-0.0729	-0.46427	GID7
0.044540233	0.752538044	0.746737164	0.143974	0.040422	0.119383	ERV29
0.04467589	0.858991565	0.870558299	-0.19748	-0.03636	0.112285	SPC19
0.044694866	0.92361733	0.790203342	0.254202	0.031364	-0.18879	RAD4
0.044858825	0.438384456	0.857180248	0.63378	-0.3662	-0.38978	YLR122C
0.045197155	0.166916703	1	-0.84966	0.752913	-0.19464	YJL027C
0.045426088	0.397361658	1	0.160428	0.098313	0.005533	LOT5
0.04563209	0.288190795	0.27982387	-0.14687	0.108872	0.262794	KEI1
0.04563209	0.110870889	0.976568193	-0.19332	0.197482	0.05666	MRPS28
0.04609593	0.960977992	0.442779343	1.264279	0.109872	-1.87645	LDS1
0.046453071	0.720851511	0.904680493	-1.32659	-0.40015	0.685759	None
0.046597557	0.121882831	0.678279671	0.500785	0.503015	-0.49971	None
0.046809242	0.29598782	0.733389979	-0.15866	-0.11637	-0.13783	DNF1
0.046947616	0.436667707	1	-0.19326	-0.1108	-0.03645	MSN2
0.046951095	0.058385914	1	-0.1518	-0.18035	0.02494	RSC58
0.047096385	0.926698469	0.971609311	-0.18335	-0.02216	-0.05765	SCY1
0.047115121	0.321781322	0.085125736	-0.13599	0.095469	0.333597	RPL2A
0.047132549	0.786999262	0.201505014	-0.4489	-0.11059	0.910505	None
0.047620036	0.516999501	0.816993642	0.123046	0.060617	0.086039	PRS1
0.047702291	0.133103516	0.919516515	0.228991	0.225245	-0.10369	TDA5
0.04814447	0.98348805	1	0.385991	-0.02531	0.037723	PRP18
0.04846279	0.049421861	0.420929331	-0.20675	0.253948	-0.30691	YMR253C
0.048789287	0.056847963	0.372619727	-0.16157	-0.19427	0.258408	BOI2
0.048832414	0.633674911	0.489219563	0.218556	0.085494	-0.29838	GTS1
0.048833078	0.103875873	0.029665642	-0.18297	0.191957	0.54531	RPP1B
0.048933426	0.177075653	0.836674365	-0.155	-0.14016	-0.1024	MYO5
0.049317964	8.13719E-05	0.617215067	-0.19176	-0.4181	-0.21664	ALR2
0.049317964	0.589760072	1	2.615148	1.110465	1.342953	None
0.049535158	0.485953152	0.522217116	0.123808	0.065432	-0.16247	ALT1
0.049598644	0.986467664	0.241395092	0.259208	-0.01598	-0.49806	NSE4
0.049630789	0.591976174	1	2.607772	1.102631	1.332943	None
0.049653522	0.867220499	0.739113671	0.178196	-0.03231	-0.15405	PSD2
0.04973174	0.433255564	1	0.276093	-0.16203	0.054808	None
0.049744399	0.701086826	0.888371569	-0.15278	-0.05052	-0.08262	SPT3
0.050030931	0.248699128	0.026784925	-0.14668	0.117777	0.446395	RPS16B
0.050150864	0.146520392	0.781154036	0.242314	-0.2356	0.190083	RTC4

0.05032769	0.484345804	0.814159342	-0.33532	0.176954	-0.23737	YKL162C
0.05032769	0.759288226	0.87592683	1.537025	0.426557	-0.86415	None
0.0503378	0.051155386	0.743134392	-0.15931	0.19601	-0.13628	NDI1
0.0503378	0.560135699	0.764962215	-0.25018	0.114958	-0.20282	LCL3
0.050475627	0.140664834	0.986912078	0.152265	0.148969	0.040143	MAS1
0.050586575	0.002722567	1	0.194612	-0.34731	-0.04049	SPC105
0.050586575	0.148485947	1	-0.17025	-0.16407	0.002985	ORC1
0.050700427	0.563839533	0.484106841	-0.23313	-0.10696	-0.32359	YAT2
0.05086553	0.196724669	0.983370642	-0.14017	0.123018	0.039067	CAM1
0.051093221	0.051738183	0.681411478	0.179489	-0.22256	0.18225	UBP3
0.051382092	0.045478422	1	0.246468	-0.31461	-0.0462	NET1
0.051812909	0.826578265	0.53398283	0.131848	0.028946	0.171599	ADK1
0.051833833	0.396430227	0.916204152	0.200016	-0.12626	0.09424	YPR089W
0.051868698	0.039547582	0.033854211	-0.16517	0.213992	0.489355	RPP2B
0.05199992	8.89882E-15	0.27982387	0.120006	0.464616	-0.21985	TPS1
0.05199992	0.846530385	0.840202254	-0.32235	0.064172	-0.21201	RPL33A
0.052163548	0.111257458	0.921286576	0.257867	-0.27306	-0.1165	RAD5
0.05216609	0.857366864	0.007095665	0.107737	-0.02066	0.384428	NOP7
0.052292414	0.613747706	0.704128889	0.222605	-0.09305	0.215853	YBP1
0.052611574	0.07256669	0.726816534	-0.17961	-0.20915	-0.16223	SAN1
0.05283662	0.338327134	0.781709456	-0.18213	0.127137	-0.14289	VPS73
0.05283816	0.003897191	1	0.15749	-0.27509	-0.00165	AGE1
0.052871712	0.893928423	0.7990478	-0.12713	0.019804	-0.09504	SSO1
0.05290877	0.557678834	1	0.213472	-0.10055	0.003628	AIM10
0.053102837	0.057235409	1	0.323649	0.395001	-0.01957	PAD1
0.053578637	0.096123699	0.783364287	0.187667	-0.20663	0.147744	CHD1
0.053700553	1	0.870558299	-0.12148	0.00149	0.071636	FSF1
0.053941763	0.094415043	0.835951015	0.279689	-0.31067	-0.18927	ULS1
0.053984214	0.551240588	1	-0.20365	0.096424	0.026001	ECM7
0.054146543	0.008136572	0.831370236	-0.14193	0.230169	0.097318	RSM10
0.05425838	0.38597363	0.721271027	0.135203	0.087417	0.125624	MLC1
0.05432166	0.598532156	0.685581609	-0.14546	0.062847	0.148228	ALG9
0.054698621	0.549922765	0.563376575	-0.21182	0.100802	0.265562	SMD3
0.054725087	0.05078776	0.082586739	-0.16263	0.203911	-0.41094	MDL2
0.054725948	0.645802547	1	-0.14984	0.058134	0.025542	GPN3
0.054736225	0.943232175	0.961108582	0.213169	-0.02285	-0.07601	STE7
0.055027701	0.963282012	0.000164967	-0.73673	-0.06293	-4.06507	YNL033W
0.055082041	0.295659	0.926370396	0.255233	-0.19449	0.113896	RGR1
0.055090499	0.885106299	0.735465999	0.177391	0.030009	-0.1588	NTA1
0.055099656	0.019167121	0.029665642	-0.23694	0.347301	0.722534	GPG1
0.055099656	0.880612135	0.92745323	1.745197	0.2944	-0.74488	None
0.055406862	0.000305053	1	-0.13493	0.278974	0.005065	VTC1
0.055463617	0.246344291	0.751551414	0.127991	-0.10597	0.109678	HPM1
0.055498883	0.568378999	0.328463464	-0.12046	-0.05567	0.208618	TMA46
0.05554135	0.964966003	0.897942454	-0.20362	-0.01757	0.107439	YHR182W
0.055567182	0.223686385	0.969393262	-0.25931	0.220741	-0.0862	PET130
0.055567182	0.137396123	0.979701935	0.196597	-0.19865	-0.05786	POL1
0.055805179	0.455741185	0.955111107	0.130008	0.074538	-0.04804	ARC15
0.055820886	0.597190291	0.730786259	-0.18246	0.079562	-0.16559	VPS33
0.0559644	0.368966584	0.238709821	-0.15338	-0.10313	-0.30295	ENT2
0.056212382	0.084904846	0.69331605	0.201475	-0.2309	-0.20366	TRS120
0.056212382	0.782318684	0.93622018	-0.25698	-0.06648	-0.1057	YMR187C
0.056224523	0.619431177	0.341937853	-0.18135	0.075395	-0.30865	YKR051W
0.056224523	0.310057499	0.50705979	-0.12711	-0.09448	0.174892	TRM82

0.056485468	0.913952086	0.881648602	-0.12681	0.017544	-0.07194	YMR295C
0.05685888	0.539290348	1	-2.57821	1.161361	-1.3513	HMRA2
0.056966299	0.384350339	0.92711763	0.159021	0.104422	0.071181	SEM1
0.057103534	0.485953152	0.758065316	0.131628	-0.07174	0.1119	ARP4
0.057116586	0.477410911	0.855814602	0.164761	-0.09137	-0.10473	SLA1
0.057234256	0.079574256	1	-0.15826	-0.18416	0.020617	ITC1
0.057280243	0.341849588	0.87592683	-0.13174	0.093082	0.076418	YPR063C
0.057302447	0.017770743	0.86530488	0.167663	0.251651	0.102708	HST2
0.057345002	1	1	-0.18548	-0.00358	0.042217	OSH2
0.057398532	0.979904046	0.73520044	-0.21073	0.015015	-0.19018	DAL82
0.057463122	1	0.878688605	0.262166	-0.00962	-0.15114	PXA1
0.057504481	0.970365023	0.737748659	-0.26241	0.021326	-0.23448	TMN2
0.057777769	0.440243906	0.286613943	-2.13516	-1.19141	4.480262	None
0.057858599	0.500936572	0.790203342	-0.27472	0.14447	-0.21358	PKP1
0.058008657	0.113318165	0.223097491	-0.14065	-0.1506	0.287542	DBP10
0.058345601	0.751354579	0.319048281	0.154215	-0.04588	0.2751	OSM1
0.058366075	0.843393723	0.132432156	0.232604	0.048343	-0.54571	ATG17
0.058723063	0.011369315	0.525726142	0.182739	-0.29432	0.247791	TMN3
0.058729274	0.421043664	0.731592804	-0.26133	0.160277	-0.23863	ATG12
0.058759217	0.637510936	0.695014092	-0.14129	-0.05695	-0.14371	VAC17
0.058814392	0.801161752	0.661976013	-0.21256	0.052184	0.230708	YKR045C
0.058879105	0.069544572	0.161150878	0.153999	0.184804	0.348437	RPL8B
0.059030089	0.377409904	0.859769494	0.245805	-0.16577	0.155757	MTC4
0.059180316	0.360063532	0.127733954	0.233711	0.16194	-0.55239	SPO73
0.059182064	0.94036767	0.058656331	0.193885	0.021704	-0.5337	VPS36
0.059237888	0.928674902	0.463829525	-0.17047	-0.02127	-0.25156	LDB7
0.059438013	0.932813812	1	0.111668	-0.01357	0.007644	NAB2
0.059668717	0.576525662	0.889649609	-0.12313	0.056814	0.068163	RRP43
0.059789898	0.281582083	0.915496392	0.224802	-0.17909	0.110136	VPS72
0.059800424	0.72767405	0.185716843	-0.14609	0.046725	0.317026	MRPS17
0.059803489	0.960977992	0.39284795	-0.1065	-0.00973	0.172427	CDC33
0.059824695	0.350302215	0.656171223	0.246755	-0.17489	-0.27264	MMS4
0.059910921	1	0.530625906	0.19227	0.007102	-0.25885	PGS1
0.060106412	0.244868024	0.733954873	0.197288	0.166224	-0.18031	YEN1
0.060339837	0.354402795	0.93741782	0.151855	-0.10681	0.062725	SEC16
0.060463735	0.576193096	0.670601582	1.470722	0.665306	1.672228	None
0.060510711	0.36814865	0.05005618	1.934683	-1.38553	-7.48095	ATP6
0.060587591	0.415254519	0.507217503	-0.17092	0.106686	-0.23775	SLM4
0.060657072	0.8316826	0.868996053	-0.15176	-0.03357	-0.0929	YPR147C
0.060677511	6.75771E-05	0.717477893	0.159308	0.364179	0.152742	GDH2
0.060677511	0.340662441	0.721271027	-0.23049	0.164827	-0.21761	KAR5
0.060677511	0.621348798	1	0.12503	-0.05284	0.001219	CKA2
0.061061818	0.856930611	1	0.137803	0.02737	0.023911	YMC2
0.061065529	0.475062233	0.049893598	-0.12519	-0.07049	0.360594	FAF1
0.061261188	0.97156603	0.817868751	-0.15162	0.012192	0.111108	RER2
0.061506123	0.803249496	0.672010939	-0.14759	-0.03634	0.158378	SEY1
0.061538315	0.967392794	0.324676992	0.144646	0.012512	-0.25788	CDC34
0.061538315	0.954916262	1	-0.18158	-0.01765	0.042717	VMA22
0.061726762	0.977693292	0.745385015	0.143294	-0.01076	-0.12731	GTB1
0.061746371	1	0.119906082	-1.29744	0.00606	3.458795	YHL005C
0.062224565	0.018033455	0.255950109	-0.19672	0.298422	-0.386	TES1
0.062584235	0.780949762	0.801754205	-0.23211	0.062094	-0.17785	SMD2
0.063365129	0.995096172	0.591339962	-1.3075	-0.06657	-1.84591	None
0.063449051	0.012231886	0.847404354	-0.14405	0.231455	0.096261	AIM41

0.063571823	0.610223632	0.372619727	0.1742	-0.07662	-0.29435	ARG2
0.063844326	0.918983013	0.546331004	1.428915	-0.19234	2.031571	None
0.063844326	0.137099942	0.781154036	-0.24774	0.25531	-0.2022	ATG5
0.064452877	0.030231646	1	-0.84541	1.170739	-0.16682	YBR144C
0.064891108	0.128591907	0.839473292	0.291706	-0.3107	-0.20273	MPS3
0.064964034	0.436108065	0.924605282	-0.25568	0.156275	-0.11911	MUM3
0.065167074	0.321967781	0.697639077	-0.11468	-0.08624	0.118769	OST3
0.065230356	0.073170272	0.062057174	-0.16365	0.19813	0.455828	RPL6A
0.06526659	0.748196133	0.971561168	0.125947	-0.0387	0.04308	STE23
0.065372999	0.826578265	0.859694751	-0.1565	0.035926	0.101166	PLP2
0.065585127	0.890938834	0.471735065	0.188016	-0.03144	-0.28056	GYP5
0.065681238	0.04685584	0.838617816	-0.19412	-0.25847	-0.13544	SAS3
0.065818812	0.357604972	1	-1.38091	-0.96423	-0.27834	None
0.065826327	0.0969018	0.84918567	0.166711	-0.19148	0.111529	DNF2
0.065831294	0.98811108	0.617350547	0.17872	-0.01103	-0.21414	CSM1
0.065831294	0.149436038	0.953128828	0.197919	-0.20217	-0.07654	ARP8
0.066049005	0.035103743	0.87592683	-0.18266	0.253992	0.109217	FMP46
0.066683909	0.712618958	0.358747602	-1.7707	0.546234	-3.7938	None
0.066751523	0.295390559	0.212331256	-0.10727	-0.08504	0.229482	EBP2
0.066859659	0.057147327	0.92578246	-0.2083	0.265658	-0.09664	COX16
0.067097335	0.147631315	1	0.184249	-0.18977	0.006133	BIR1
0.067311076	0.192026738	0.585629945	0.179968	-0.17036	0.229224	LRE1
0.067381548	0.98474138	1	0.239282	-0.01642	0.034263	DAS1
0.067492409	0.662458676	0.488692236	-0.13102	0.051129	0.192196	GCN1
0.067596231	0.442611824	0.185716843	1.062622	0.645736	-2.58544	YIL134C-A
0.067596231	0.638898558	0.494558503	0.628417	0.260833	0.913067	None
0.067650469	0.213721853	0.975062924	0.146955	-0.13375	-0.04793	TOP1
0.067742745	0.17643013	1	-2.52806	-2.48727	3.179529	None
0.067742953	0.355396524	0.768691181	-0.22514	-0.16158	-0.19276	FZO1
0.068201405	0.605317388	0.130669798	0.124301	0.055848	0.303836	CKB2
0.068840693	0.02857849	0.740020932	-0.1596	-0.23317	-0.14747	AIM44
0.068840693	0.786528339	0.888371569	0.128656	-0.03456	0.074647	SEC22
0.068966911	0.438949356	0.981177966	-0.15684	0.096853	0.04777	ADE8
0.068966911	0.892744236	1	0.153013	-0.02552	-0.02094	UPF3
0.069535792	0.026063738	1	0.80851	1.205333	0.153371	GAT4
0.069606768	0.119099734	0.498203091	0.1435	-0.15837	0.209544	SEC26
0.069941151	0.000779302	0.001486144	0.164451	0.339752	0.704974	RPL41A
0.070064373	0.20100005	0.349201299	-0.10904	-0.10193	-0.19309	PYC2
0.070134774	0.273830786	0.053709945	0.128915	0.107189	0.377952	RPS11A
0.070330667	0.203402724	1	-0.16084	-0.14988	-0.01925	MSN1
0.070463494	0.261134686	0.721271027	-0.13552	-0.11538	0.132874	SPB4
0.070464402	0.097093353	1	-2.45249	2.720595	-3.60496	None
0.070753074	0.10422266	1	0.150963	-0.17263	0.013068	ALK2
0.070914313	0.019075148	0.889649609	0.19217	-0.30163	-0.11056	NDD1
0.071599307	0.907221502	0.785906893	-0.1151	0.017467	-0.09552	SEH1
0.072450666	0.058914883	0.202387133	0.182876	0.237298	0.403973	PET191
0.072450666	0.012274646	0.746636456	0.976333	-1.80284	-1.14301	YML100W-A
0.072450666	0.896286063	1	-0.18816	0.030814	-0.00986	FMN1
0.072556384	0.265592192	0.46824606	0.237011	0.201813	-0.36292	NTH2
0.072688411	1	0.621769539	0.230584	-0.00465	-0.28066	RAD55
0.072703225	0.313232327	0.688473731	0.146531	-0.11489	0.158687	STO1
0.07272872	1.81748E-14	1	0.152206	0.62638	-0.00596	HXK1
0.072787314	1	1	-0.1515	-0.00224	-0.00301	TYW3
0.073167276	0.242501074	1	0.155548	0.137279	-0.00913	XPT1

0.073317645	0.112837622	1	0.194262	-0.22035	-0.00249	SIP3
0.07337207	0.596380201	0.67702211	0.123851	0.057606	0.137129	CPR1
0.073374437	0.781328521	0.056523979	0.135715	0.037678	0.397244	ERP1
0.073856382	0.713873054	0.8119342	-0.15451	-0.05385	-0.11978	YLR001C
0.073875247	0.966757446	0.583321672	0.191754	-0.01746	-0.24939	MIH1
0.073909206	0.183157722	0.7225564	0.120579	0.117849	0.118732	YJL055W
0.073915461	0.492341773	0.967394603	-0.21647	0.122269	0.077616	NUF2
0.074210302	0.120527431	0.30954724	-0.10983	-0.12211	0.20879	HAS1
0.074210302	0.046903645	0.635840497	-1.38373	-2.03293	-2.06558	YRF1-2
0.07454466	0.340662441	0.644321253	1.813491	1.320501	-1.74882	None
0.074643599	0.746430113	0.976568193	0.16837	-0.05366	0.054615	ASI3
0.075062241	0.20204603	0.514203536	0.21809	-0.20818	-0.31669	CHL4
0.075259882	0.932146929	0.562048326	-0.22291	0.028566	-0.3004	PXA2
0.075259882	0.387128577	0.971609311	-1.81069	1.150497	-0.86511	YER181C
0.075443152	0.857968624	0.771481707	0.257051	-0.05345	-0.22428	REV3
0.075515218	0.780041137	1	0.173799	0.048859	-0.03669	RAD2
0.075976207	0.143789044	0.765014016	0.226758	-0.24225	0.202232	DRS2
0.075976207	0.177075653	0.919926509	0.173734	-0.17343	-0.08603	ASE1
0.076041809	0.839560652	1	-0.16079	-0.03607	-0.01796	HFA1
0.076485261	0.008822013	1	0.18676	-0.32804	-0.00633	MPT5
0.076602191	0.324783906	0.328463464	0.195869	0.152409	-0.36323	ARL3
0.076993217	0.261791204	0.904680493	0.351304	-0.30675	-0.19202	SNT2
0.077099586	0.767459399	0.661089241	-0.50722	0.147299	0.585867	None
0.077240761	0.282370113	0.187978416	0.305166	0.256265	0.697137	YEL067C
0.077240761	0.761207443	1	0.127201	-0.03851	0.017275	RFC3
0.077264488	4.13667E-06	0.403368253	-0.13689	0.369126	0.23164	SDH4
0.077356276	0.945664113	0.261118505	0.112421	-0.01274	-0.23068	RNQ1
0.077385365	0.009388611	0.622714429	-0.13169	0.228141	-0.16179	YCP4
0.077405767	0.731503094	0.247373218	0.128671	0.043078	0.270705	RPS8A
0.077430316	0.00430551	1	-0.13187	0.245538	0.019978	RSM25
0.07762445	0.043220269	1	0.141429	0.197807	-0.02814	YET1
0.077754019	0.746926462	0.023500528	-1.20629	0.368657	-5.93733	YNL042W-I
0.077917988	0.914594311	1	-0.54717	0.080032	0.077773	SPS100
0.07796152	0.039547582	0.667844098	0.167045	-0.23899	0.1916	RRP14
0.078248067	0.141633165	0.058656331	-0.14547	0.155742	0.428087	RPS23A
0.078457227	0.209645547	0.168205333	-0.19255	0.181121	-0.45499	YNL011C
0.078754535	0.643719368	0.218100263	-0.20599	-0.0872	-0.45084	LSB6
0.078872755	0.36891213	0.261118505	0.297327	0.216334	-0.61105	PCD1
0.078966682	0.124795835	0.043101043	-0.11417	-0.1275	0.357934	SDA1
0.078966682	0.98408428	1	-0.17142	-0.01232	0.031223	PRS2
0.079033452	0.205442701	0.551606929	0.204571	-0.19628	-0.28381	HDA2
0.079070313	0.905759808	0.868996053	0.162282	0.02556	0.105444	YDL012C
0.079070313	0.761075278	1	-0.18669	0.056652	-0.03937	GMH1
0.079136496	0.461336331	0.984628847	0.16115	-0.09919	0.049167	CWC24
0.079656611	0.27087925	1	2.32573	-2.08265	-3.11423	None
0.079927327	0.019622226	0.721271027	-0.14917	0.236899	0.150228	PMP3
0.080036578	0.393729423	0.129195117	0.118308	0.082262	0.300975	RPS5
0.080060737	0.031545749	0.927845118	0.164337	-0.24569	0.078507	ESL1
0.080146277	0.597239802	0.228471317	-0.18094	-0.08568	-0.39154	MHT1
0.080146277	0.936093775	0.931568877	0.164161	0.020653	0.074905	YBL010C
0.080336565	0.526984154	0.835951015	-0.17544	0.094835	-0.12942	COQ11
0.080364668	0.01270424	0.05506878	0.11754	-0.19917	0.353362	SAS10
0.080449837	0.730753693	0.834791774	1.445745	-0.48869	1.093455	YHR218W
0.080500048	0.052131095	0.55065098	0.152746	-0.20923	0.214163	UBP14

0.080500048	0.147880106	1	-0.13054	0.139099	0.014895	YML6
0.080541745	0.011964436	0.074765706	0.131446	0.224407	-0.37014	YBR016W
0.080605211	0.38127191	0.642194109	0.104717	-0.07499	0.126804	WRS1
0.080935269	0.119099734	0.87592683	-0.75459	-0.87206	0.51669	YHR213W-/
0.080935269	0.063115794	0.902276563	0.209169	-0.27656	0.118356	SAC3
0.081044166	0.433392057	0.479745932	0.283694	0.184604	-0.43907	RAD10
0.081287775	0.885748557	0.770752057	-0.18587	-0.03378	0.16554	PSY2
0.081628846	0.620115135	0.530625906	-0.12423	0.056081	-0.17914	YGR266W
0.081634803	0.92384638	0.553808105	-0.17958	0.025015	-0.24959	MOB1
0.081634803	0.986843508	0.848379499	-0.18987	-0.01283	-0.13379	NVJ3
0.081634803	0.758146893	0.871950707	-0.20334	0.063249	-0.13025	YHK8
0.081634803	1	0.995247182	-0.2385	0.002317	-0.06486	PDR10
0.081638445	0.630001142	0.7990478	-0.25639	-0.11323	-0.2112	KNH1
0.081726647	0.081781284	1	0.113573	-0.14266	0.011399	VAN1
0.081939743	0.686392021	0.545272974	0.856505	0.327761	1.223691	HED1
0.082025817	0.327426468	0.23260457	0.855444	0.673973	1.879548	YGL149W
0.082040876	0.690335313	0.279646275	-0.11027	0.041881	0.224438	RPL5
0.082418277	0.427826936	0.223097491	0.112406	0.074043	0.249389	RPS1A
0.082716152	0.486638529	0.055048108	-0.54051	-0.31797	-1.76551	None
0.082727509	0.659994539	1	0.260919	0.107948	-0.03943	TPC1
0.083408653	1	0.739091337	0.325365	-0.00246	-0.31543	ECM27
0.08343532	0.663724282	0.76367822	-0.12271	0.050062	0.112331	MRPL3
0.083442525	5.87959E-05	0.748420004	-0.13934	-0.34701	-0.13194	CYK3
0.083533022	0.842773453	0.786584362	-0.12717	0.028703	0.109151	SRP102
0.083533022	0.433535321	0.807027851	-0.56441	-0.36885	0.467668	YOR072W
0.083957634	0.270460422	0.385959394	0.214669	0.1875	0.379744	YBR230W-/
0.083957634	0.012515571	0.750952753	-0.12987	-0.22267	0.122311	FAP1
0.083970593	0.330237847	0.680777882	0.15428	-0.12229	0.175516	RMD1
0.084278831	0.806573971	0.525568993	-0.12589	0.03287	-0.18441	CPS1
0.084285462	0.851320842	0.598545037	-1.71935	-0.36331	2.363422	None
0.08452345	0.52364909	0.781704944	0.176034	-0.09752	-0.154	OCA5
0.084639969	0.516700017	0.728625888	0.142741	-0.08013	-0.14329	PRR1
0.085111035	0.359386016	0.795907717	0.136241	-0.1029	0.114557	MKT1
0.085664359	0.900565868	0.036431385	-0.13578	-0.02253	0.446786	LRP1
0.085681825	0.415045535	0.822329423	0.184498	-0.12577	-0.1445	STE11
0.085956547	0.363817999	0.196724326	1.966455	-1.52854	-5.94997	None
0.086021287	0.693518654	0.43642005	0.130645	0.049721	-0.21771	LIP5
0.086041434	0.480589154	0.567844831	-0.68998	-0.41459	-0.98807	YRF1-1
0.086207508	0.903977193	0.930420244	-0.61011	0.098173	0.290677	YOR032W-/
0.086275937	0.0122332	0.742563863	0.176906	-0.30799	0.172226	SIN4
0.087338893	0.894434943	0.049893598	0.166418	-0.02907	-0.5185	PCL6
0.087769753	0.209645547	0.990208543	0.130256	-0.12695	0.038052	EBS1
0.087769753	0.469895523	0.998440996	0.201685	-0.12557	0.054026	PXL1
0.088154937	0.631437679	0.533641348	-0.15093	0.067567	-0.22053	HSH49
0.088569596	0.024098511	0.119906082	0.133982	0.212973	-0.35294	GOR1
0.088569596	0.337257493	0.155888434	-0.14124	0.111103	0.352588	GPN2
0.088712033	0.832733162	0.480256366	-0.97581	-0.22881	1.595478	None
0.088829712	0.940649911	0.967394603	0.131759	-0.01615	-0.04975	ABZ1
0.088881505	0.083848492	0.861496883	0.17309	-0.22086	-0.11938	BUD4
0.089073769	0.118813668	0.795907717	-0.2081	0.241927	0.176871	None
0.089265095	0.734684065	1	1.188684	0.411485	-0.09316	YKL102C
0.089696918	0.666883176	0.817868751	-0.20552	0.08442	-0.16428	YNR048W
0.090386028	0.424705765	0.770482483	0.199226	0.135194	-0.18244	SRN2
0.09047873	0.15834968	0.919926509	0.112176	0.120615	0.057991	YPL199C

0.090512727	0.757783308	1	-0.16978	0.05437	0.036551	SWD3
0.090866044	0.500629099	0.194043747	0.095633	-0.05627	0.226533	PRT1
0.090866044	0.384327883	0.997805152	0.200705	-0.14745	0.054639	PRP22
0.091018551	0.926213546	0.536035164	-0.21238	0.029793	0.312312	MTF2
0.091166686	0.864979821	0.838466151	-0.75358	-0.15624	-0.56981	AMA1
0.091264445	0.066428344	0.964698121	0.133324	-0.17948	0.051977	NMT1
0.091612207	0.414293185	0.457578646	0.129542	-0.08978	-0.2135	PHA2
0.09182856	0.062102203	0.89987755	-1.02771	1.355565	-0.68477	None
0.091919767	0.165414541	0.781154036	-0.14931	0.158601	0.133437	AIM7
0.092050145	0.11971678	0.927619939	0.176349	-0.20846	0.087633	URC2
0.092561279	0.038719477	1	-0.18459	-0.27575	-0.01544	IFH1
0.092755084	0.121546688	1	0.125287	-0.14714	-0.02324	PMI40
0.09286394	0.04046433	0.438612707	0.180811	-0.26904	0.307681	ORC2
0.09286394	0.119522468	1	0.278646	0.328229	-0.01714	EMP46
0.092890138	0.010362697	0.291512883	-0.1324	-0.23889	0.271764	TSR1
0.093196993	0.295242295	0.630202471	0.133547	0.114889	-0.17024	YKL023W
0.09333977	8.03946E-05	0.589775129	0.136168	0.341285	0.185833	NNR1
0.093360294	1	0.919926509	0.1212	-0.00512	-0.06358	MIT1
0.093592446	0.003378115	0.229407061	0.115716	-0.23289	0.262339	KEL3
0.093670407	0.019075148	0.937363635	-0.18475	0.306836	0.084422	YGR021W
0.093856953	0.418012386	0.005535976	0.187833	0.13004	-0.7782	YJL163C
0.094156347	0.486096013	1	0.24761	-0.15206	-0.02496	WHI5
0.094369839	0.103376814	1	-0.22026	-0.26995	0.019336	RDS1
0.094539124	8.62465E-05	0.359252765	0.174612	0.437234	-0.32777	PYK2
0.094663369	0.715818525	0.8119342	0.115187	-0.04249	-0.0951	KAE1
0.094682911	0.190275098	0.786909408	-0.15829	-0.16241	0.139856	DED1
0.09481842	0.925573198	0.834202861	0.194947	-0.02797	-0.15145	VPS8
0.09515606	0.633340819	0.945866293	0.148709	0.067833	0.065614	CFD1
0.09546524	0.010389369	0.680110665	0.1053	0.19086	0.123672	PHB1
0.096226389	7.47611E-05	0.581268517	-0.10997	0.278069	0.152997	IDH2
0.097020659	0.056211261	1	-0.21597	-0.30454	-0.01184	SPP41
0.097182964	0.068517075	0.493450262	-1.21274	-1.74215	-2.70988	None
0.097713955	0.302233138	0.934721444	0.214655	-0.18498	-0.1014	YLL054C
0.097767968	0.398327474	0.21536472	-0.09278	-0.06707	0.216664	GAS1
0.097923039	0.634501741	0.689510419	0.163148	-0.07477	-0.18922	YME1
0.097942701	0.317422835	0.468849245	-0.15024	0.125401	0.248058	YPR098C
0.098249329	0.54489641	0.933469902	0.114432	-0.06328	-0.05434	PAF1
0.098302725	0.758162333	1	-2.28012	0.71376	-0.84653	None
0.098389748	0.996960041	0.883095126	0.122741	-0.00682	-0.07894	GPI17
0.098389748	0.938960046	0.977348163	-0.17788	0.022861	-0.06137	CDC8
0.098430098	0.093744452	0.669524096	0.150708	-0.19248	0.182316	EPS1
0.098438245	0.761075278	1	-2.27899	0.699453	-0.82951	None
0.098438245	0.761075278	1	-2.27899	0.699461	-0.82952	None
0.098791499	0.630213798	0.313687377	0.1039	-0.04823	0.211419	IMD4
0.099672929	0.99176478	1	0.16214	0.010351	-0.0168	UBP12
0.099889902	0.196724669	1	2.240571	2.289187	3.174056	YHR054C
0.099956566	1	0.973674102	0.781787	0.026757	-0.28541	YPL152W-A
0.100046999	0.4395426	0.612191336	-0.10401	0.07034	0.139335	RNA1
0.100081755	0.934743268	1	-0.13684	-0.01839	0.019767	SIC1
0.100336315	0.447335758	0.812812583	0.130912	0.087336	-0.10918	YMR178W
0.10044338	0.612847593	0.104201741	0.326994	-0.16049	0.924592	None
0.10044338	0.885106299	0.5927044	0.157642	-0.03068	-0.21784	YOL029C
0.100588686	0.572909529	0.318346243	-1.91658	-0.9526	4.246737	None
0.100645254	0.177522395	0.560749891	-1.4507	1.459646	-2.47615	MPH2

0.100865643	0.290286754	0.990208543	-0.12216	0.107909	-0.03678	PMC1
0.101107224	0.410728813	0.343092466	-0.13189	0.09422	-0.25765	SYF2
0.101107224	0.486326234	0.448655387	-0.67101	0.407589	-1.17609	None
0.101107224	0.413974827	0.967394603	0.167737	-0.1195	-0.0661	RIC1
0.101107224	0.661428602	0.985468267	-0.18154	0.078386	-0.05687	ULA1
0.101112345	0.91970774	0.099020366	-0.11276	0.017103	0.320978	MRT4
0.101569035	0.461663229	1	0.141631	0.0925	0.013776	RPE1
0.101770912	0.586552276	0.90453709	0.141612	-0.07312	-0.08352	BRF1
0.102046681	0.639535568	0.840282548	-0.11698	-0.0535	0.090182	GNT1
0.102046681	0.199639017	1	2.22021	2.268632	3.14987	None
0.102046681	0.199639017	1	2.220209	2.268631	3.149869	None
0.102046681	0.199639017	1	2.220242	2.268665	3.149884	None
0.102047446	0.199639017	1	2.220114	2.268534	3.149827	None
0.10205299	0.109416283	0.66765157	-1.49295	-1.95324	2.424267	None
0.102306828	0.382001694	0.584673159	0.193726	-0.14747	0.274178	NUP192
0.102357091	0.09990881	0.972387712	-0.11278	-0.14249	-0.04139	PIR1
0.102500492	0.406828448	0.506625282	1.215067	0.87152	-2.05774	None
0.103033071	0.990080396	0.182529351	-0.98196	-0.06278	-2.93529	None
0.103434452	0.422272348	0.775955771	0.159831	-0.11306	-0.1496	TAF1
0.103762762	0.102434008	0.010294461	-0.11176	-0.14082	0.450568	ENP2
0.103803484	0.368966584	0.71503018	0.191872	-0.15018	0.21166	RTP1
0.103803923	0.690335313	1	0.165581	-0.067	-0.00664	UFE1
0.103821968	0.355796735	0.691419277	0.161606	-0.12942	-0.18959	HIR1
0.104206933	0.616558301	0.024892656	0.180173	0.087665	-0.6555	PPE1
0.106302676	0.652629347	0.635167376	0.259319	0.11667	-0.34022	YIR018C-A
0.106565171	0.869328337	0.906168502	-0.09804	0.020891	0.05765	SEC13
0.106625687	0.750994992	0.579999612	0.107873	0.037088	0.15496	SEC4
0.106712016	0.933553067	0.493704454	-0.70952	0.096816	1.161562	None
0.106737957	0.567058038	0.078502393	-0.12153	0.065895	0.366163	RPS23A
0.106737957	0.46811084	0.600528962	0.137051	0.089827	0.190449	AIM29
0.10684567	0.438949356	0.667670933	-0.13233	0.091189	0.16415	PTH2
0.106870114	0.543950269	0.929303164	0.111941	-0.06349	0.056709	PMU1
0.107118748	0.648018557	0.333626467	-1.53153	-0.67272	-3.79734	YFL066C
0.107469917	0.440961232	0.553808105	-0.12954	-0.08911	-0.19389	YGL185C
0.107624386	0.378159684	1	-0.15682	0.121166	0.01936	SAM37
0.107636241	0.643653231	0.976568193	-0.20471	0.093776	0.072783	PRP46
0.107851325	0.011832562	0.95557089	-0.13252	-0.24512	-0.05708	MCD1
0.108039666	0.017598231	1	1.62694	-3.66259	-1.76097	None
0.108039666	0.91441715	1	-0.19386	-0.03118	-0.0333	SWC5
0.108087639	0.259482058	0.971609311	-0.46208	0.433777	0.173857	AIM26
0.108211705	0.065404264	0.033854211	-0.11609	-0.16364	0.409932	TOD6
0.108252258	0.000167114	0.737914729	-0.21397	0.534975	0.22236	YBL029C-A
0.108270739	0.093895807	0.55065098	-0.54441	0.696316	-0.82188	MCH2
0.108379644	0.853463958	0.195294516	-0.28295	0.065256	-0.69493	RHO2
0.108551218	0.502734634	0.974952294	0.187831	-0.11562	-0.06909	TFC3
0.108826478	0.613821587	0.03528112	-0.09391	-0.04649	0.3306	SSF1
0.109086871	0.079535843	0.020319939	-0.15118	0.204826	0.573014	TMA7
0.109086871	0.712618958	1	-0.16481	-0.06375	0.02619	MDM30
0.109357675	0.012013871	0.201505014	1.07991	2.068813	-3.52759	RGI2
0.109811683	1	0.635840497	-0.12527	0.003575	0.165612	GAA1
0.109867939	0.897457836	0.259414235	-0.38252	0.068829	0.863494	SSP2
0.109950484	0.017246708	0.721271027	0.120059	-0.21283	0.131735	GCD2
0.110688895	0.613304251	0.053817845	-0.12299	0.061221	0.403212	NIP7
0.110771731	0.766166784	0.680475561	-0.46652	-0.151	-0.57639	PDC6

0.110771731	0.760915703	0.89043028	-0.11349	0.037719	0.07253	ECT1
0.110825675	0.414006447	0.238946444	-0.59009	0.420094	-1.42519	YOR008C-[illegible]
0.111196127	0.846089519	0.539256261	0.143942	0.034661	-0.22212	SIW14
0.111459749	0.000378952	0.82890057	0.223044	0.54068	0.183298	ADR1
0.111639464	0.668689933	0.792753853	-0.18925	0.082011	-0.17207	GTR2
0.111639464	0.934743268	1	-0.82786	0.111856	-0.09411	None
0.111653925	0.568136366	1	-0.20539	-0.11266	-0.03752	CTF3
0.111653925	0.868862376	1	0.173914	0.037771	-0.0043	PRP42
0.111930779	0.645309081	0.772090669	0.199431	0.09238	0.192898	FTR1
0.112104295	0.901784303	0.929821526	-1.17618	-0.20178	0.628929	None
0.112633189	0.752831669	0.770752057	-1.40838	-0.47282	-1.45169	None
0.113660248	0.654688585	0.607199601	0.144312	-0.06569	0.202496	ALG12
0.113660248	1	0.98500242	0.139492	-0.00321	0.046405	YPT7
0.11456337	0.9338778	0.637213394	-0.10785	0.015243	0.143821	PRS4
0.114592878	0.721891429	0.689510419	0.124875	-0.04777	0.151735	ERV41
0.115133153	0.881928588	0.740020932	0.182872	-0.03789	0.193083	VPS52
0.115223659	0.887445308	0.32895061	0.224882	-0.04444	-0.46335	DNA2
0.115310991	0.353343062	0.018502313	-0.11605	0.095409	0.452693	RPL27A
0.115310991	0.665438196	0.477818318	-0.12743	-0.05651	-0.21742	CSE4
0.115845609	0.093890302	0.24850108	-0.10206	-0.1358	0.237805	NOC2
0.116129764	0.525161659	0.688444366	-1.62832	-0.94111	-2.00154	YDL022C-[illegible]
0.116350236	0.616539813	0.635840497	0.167787	-0.08427	-0.22531	MAC1
0.116520471	0.842773453	0.960632458	-0.76023	0.184962	-0.33062	None
0.116574302	0.625421668	0.37953299	0.111964	0.054963	0.218455	EFB1
0.116624977	0.347668023	0.035829221	-0.12914	0.107466	0.462203	RPL35B
0.116624977	1	0.211271753	-0.78735	0.027589	1.982568	CDA1
0.116624977	0.407136042	0.589139583	-1.04581	0.750473	-1.59859	None
0.11771097	0.997790364	0.373162993	-0.11628	0.006553	0.228948	YPR114W
0.11771097	0.230911149	0.486872846	-0.15864	0.160736	0.268832	RPB11
0.11771097	0.58018556	0.823211814	-0.12715	-0.06911	0.107609	UBR1
0.117749068	0.099649048	0.983370642	0.17199	-0.22752	0.058982	JJJ1
0.117843016	0.645773604	0.441597935	-0.1826	0.085414	-0.32779	CTA1
0.118179103	0.080514417	0.97097406	-0.90011	-1.29611	-0.38584	YFR032C-[illegible]
0.118189112	0.558914109	0.777667569	0.142859	0.081224	-0.13824	MAD2
0.118339854	0.61447221	0.143859218	0.183254	-0.09319	-0.50238	YDR338C
0.118359491	0.770350536	1	0.163087	-0.05311	0.017588	TMA64
0.118682266	0.984954183	0.942108564	0.164749	0.012934	-0.07847	MSL1
0.118713318	0.490088889	0.927131674	0.150958	-0.09765	0.080892	YPR117W
0.119268546	0.59221262	0.37055688	0.133057	-0.07104	-0.26402	YML119W
0.11928709	0.706086243	0.978193638	-0.13324	0.05373	-0.04793	YKE4
0.119374199	0.098108702	0.924605282	-0.14962	0.197613	0.081063	YPT10
0.119374199	0.313232327	1	-0.14091	0.125018	-0.02989	SPC2
0.119383592	1	0.404219832	0.096276	0.001671	0.183066	TIF35
0.119383592	0.716376728	0.862693719	-0.31917	-0.12472	-0.23756	YPR064W
0.119383592	0.818564873	1	-0.17577	-0.04858	-0.04674	PLP1
0.119428443	0.283359769	0.056523979	1.32269	-1.32038	-6.21142	YRF1-6
0.119455969	0.050780234	0.020319939	-0.12437	0.189208	0.483709	RPL30
0.119455969	0.438949356	0.772424036	0.474021	-0.34362	0.469863	YMR105W-[illegible]
0.11952422	0.002104078	1	-0.09319	0.206427	0.014471	LSP1
0.120204774	0.937034346	0.795458324	-0.12288	0.016995	0.113742	FCF1
0.120210012	0.166873945	0.699105653	0.121688	0.139215	0.146805	COQ5
0.120842025	0.801294446	1	-0.09941	0.029217	0.023876	TUB1
0.121208428	0.138242749	0.971609311	0.148139	-0.18094	-0.05796	RGD1
0.121766241	0.019725812	0.90453709	0.290613	0.517417	-0.17959	YMR085W

0.121823926	0.887204289	1	-0.21737	-0.04362	0.042104	PET111
0.12204285	0.079562974	0.536035164	0.155968	-0.22032	-0.24929	ESC8
0.122067735	0.51601516	1	0.236359	-0.14692	-0.00357	STU1
0.122157469	0.926213546	0.5888858	0.095619	-0.0146	-0.14065	LAP3
0.122181278	0.012312882	0.468714964	0.119896	-0.2285	0.211557	SSM4
0.122422575	0.82837317	1	-0.22589	-0.06048	0.002831	INP2
0.122924904	0.270400357	0.648340079	0.248817	-0.24222	-0.33276	SSN2
0.123187213	0.481758197	0.731592804	-0.12685	0.08407	0.140149	LSO2
0.123187213	0.984954183	0.901225243	-0.60034	-0.04719	0.383272	YLR255C
0.123283286	0.295659	0.112708349	-0.45997	-0.42806	-1.4555	MPH3
0.12349326	0.398327474	0.889079564	-0.11163	-0.08634	-0.07469	ADE13
0.123589632	0.164194878	1	-2.12165	2.376486	-3.2215	PFS1
0.123589632	0.164194878	1	-2.1217	2.376528	-3.22151	None
0.123767111	0.021155099	0.852710995	0.188293	0.334413	-0.14637	GLO4
0.124165198	0.909881518	1	2.073402	0.371842	0.478202	YBL112C
0.124165198	0.909881518	1	2.073412	0.371857	0.478214	None
0.124348898	0.023493222	0.077306681	0.102079	-0.17972	0.323389	BUD22
0.124416325	0.128547958	0.006666491	-0.12854	0.160275	0.568999	RPL42A
0.124544888	0.740566016	0.390228331	0.203392	-0.07558	-0.39759	YBR197C
0.124580565	0.190369044	0.472057083	-0.1485	0.164014	0.261229	SUV3
0.124692313	0.598077776	0.950592115	-0.17763	0.094463	-0.08231	CSM3
0.125041718	0.384001707	0.7232059	-0.18832	0.150186	0.212273	EFM6
0.125085808	0.356552847	0.499155534	0.134857	0.113435	0.229163	CPR2
0.125085808	0.890649696	0.848611069	-0.26187	0.051487	-0.20604	SMK1
0.125581158	0.001186688	0.071166723	0.106383	-0.25106	0.343248	MTR4
0.125700911	0.305163905	0.931005601	-0.13283	-0.12167	0.06897	BRE2
0.126143817	0.374729788	0.731592804	0.161	0.131722	-0.17882	YTH1
0.126200035	0.006994212	1	-0.13779	-0.28187	-0.00628	BCK2
0.126334341	0.184156664	0.05005618	-0.10641	0.119415	0.367036	ASC1
0.126868753	0.111467148	0.182165955	-0.1027	0.134199	0.27127	YPL225W
0.126872643	0.560120507	0.871066692	0.110481	-0.06412	-0.08052	PAT1
0.126889812	0.746926462	0.863626849	-0.13966	0.050943	0.104918	EMC3
0.126984855	1	0.659101564	-0.18218	0.007001	-0.2419	MRX8
0.127120516	0.542558974	0.845430426	-0.17457	-0.10413	-0.14133	EST1
0.127630528	0.224234386	0.622714429	0.269466	-0.28599	-0.38013	SPP381
0.127702117	0.017055604	0.976568193	0.189821	-0.35322	-0.07106	HSL1
0.1277632	0.194075919	0.711421266	0.109686	0.1218	0.129991	SNF4
0.127954576	0.570704752	0.302955543	-0.21132	0.119464	0.467479	CBS1
0.127963229	0.479033885	0.8119342	1.564697	-1.08688	-1.75037	None
0.128274617	0.759694936	0.996250082	0.125496	-0.04397	-0.03824	TAF5
0.12848951	0.437664279	0.054954576	-0.11264	0.08217	0.384296	RPL20B
0.128828587	0.991616462	0.949031652	0.136896	0.009453	0.064855	NTG1
0.128947576	0.846089519	0.240761747	-0.12917	0.032386	0.315079	EMC5
0.129967763	0.203402724	0.846605423	-0.18872	0.205985	-0.15263	MRS1
0.129990038	0.131282664	0.503789088	0.134486	-0.17005	0.230291	YEF3
0.129990038	0.829818589	0.679433873	0.171861	0.046881	-0.22041	PSY3
0.130054426	0.087597502	0.085125736	-0.11077	-0.15515	0.348361	UTP21
0.130224059	0.284831522	0.151369301	-0.13402	0.128698	0.375597	MRP7
0.130289609	0.448109038	0.637177071	-0.12945	0.09273	0.179509	YEL068C
0.131026092	0.395417127	0.702868145	-0.11475	0.090871	-0.13959	CMK1
0.131131765	0.926213546	0.886876142	-0.09988	0.015547	0.069112	CIS3
0.131239379	0.832549245	1	0.53474	0.144466	0.156222	YDR344C
0.131428483	0.51219348	0.992021238	-0.34031	0.215569	0.108838	SPS1
0.131819221	0.031130308	0.702041373	0.649785	-1.17478	0.864036	YDL159W-[illegible]

0.13189085	0.276302587	0.354608469	0.166002	-0.16352	-0.34663	STB5
0.132012838	0.575041968	0.917776548	0.158598	-0.0905	0.09312	RGP1
0.132076218	0.768288475	0.868996053	0.125815	-0.04274	-0.09416	ARK1
0.132272514	0.979425666	0.477818318	0.089945	0.008031	-0.16	GDH1
0.132798086	0.563145324	0.995137276	-0.14381	0.083804	0.044806	CHS7
0.132925903	0.98811108	0.688444366	0.642185	0.048104	0.821785	None
0.132925903	0.349120443	0.69956533	-0.37216	0.319054	0.45869	RRT5
0.132972136	0.858414089	0.970494097	-0.31334	-0.07537	-0.12993	None
0.133109268	0.129290846	0.812322751	-0.13585	-0.17338	0.123474	LAS21
0.133274899	0.04003014	0.597863762	0.160418	-0.26539	-0.23947	RMD8
0.133590398	0.515509269	0.551545293	-0.09795	0.062356	0.157081	ECM14
0.13399311	0.118712454	0.960632458	0.145891	-0.19244	0.065739	LYS14
0.134137995	0.751310355	0.712613882	0.161617	-0.05962	0.193348	KTR5
0.135057835	0.119536849	0.71733135	0.148969	-0.19623	0.176369	STB4
0.135223143	0.952836649	0.971609311	-0.18634	0.022951	0.075589	MTF1
0.135738245	0.58105513	0.804032062	0.135817	0.077164	-0.12685	CBP2
0.136004824	0.411661642	0.274807381	0.11166	0.087097	0.263467	FET5
0.136656078	1	0.598636186	0.144936	0.006105	0.217939	GPI10
0.137173238	0.587029779	0.995137276	-0.97798	-0.54338	-0.30908	None
0.137238453	0.38127191	0.726816534	-0.09456	-0.07831	0.108987	MNN2
0.137430243	0.395362483	0.411976149	0.124186	-0.10038	-0.24286	SSK2
0.13784725	0.404471627	0.962145993	-0.14759	0.116615	-0.06587	RIB7
0.137933322	0.07973457	0.861885315	0.185205	-0.27131	0.144855	CDC39
0.138615937	0.225458217	0.87710821	0.104208	-0.1127	0.075728	EMC1
0.138788951	0.619870553	0.733389979	0.226661	0.11912	-0.2582	DFG10
0.138866999	0.86684094	1	-0.16621	-0.03893	-0.01727	POP4
0.139155861	0.971546998	0.831867858	-0.15467	0.015511	-0.1347	ISM1
0.1393801	0.648020755	0.940508159	0.150997	-0.07456	0.076351	YML002W
0.139494092	0.117256154	0.937179795	1.000303	1.340486	0.595945	None
0.140549257	0.969492509	0.453670338	-0.15921	0.016513	-0.29718	VID28
0.141200858	0.97156603	0.609220601	-0.11224	-0.01126	-0.16684	PDR17
0.141252424	0.128547958	0.680777882	-0.23375	0.301916	-0.30553	RCH1
0.141252424	0.057147327	0.87592683	0.126716	-0.1993	0.09318	SKN7
0.141365419	4.14505E-37	2.04124E-07	0.14751	1.153803	-1.09136	CIN5
0.141365419	0.058548443	0.917776548	-0.12573	0.195426	0.075093	SHY1
0.14147613	0.034272578	0.661089241	-0.11407	0.195311	0.155985	MRPL13
0.141971456	0.295390559	1	-2.06102	-2.02358	2.678418	YDR278C
0.141971456	0.295390559	1	-2.06101	-2.02358	2.678392	None
0.141971456	0.295390559	1	-2.06101	-2.02358	2.678361	None
0.142144655	0.295461185	1	-2.05706	-2.01964	2.672745	None
0.142226212	0.121199858	0.527985788	-0.09555	-0.12686	-0.16184	LYS9
0.142498651	0.006330535	0.507911475	-0.12228	0.260656	0.213211	RDL2
0.143506532	1	0.545418491	0.213818	-0.00118	-0.35308	CSE2
0.143648787	0.295613321	0.836551898	-0.13639	0.132302	-0.11816	MRM1
0.143949913	0.427585494	0.622714429	-0.12788	-0.09856	0.187361	TCB1
0.144262898	0.051484274	0.971609311	-0.19701	-0.31771	0.082402	ASG1
0.144315702	0.950105874	0.89987755	0.097357	-0.01253	0.065332	GPA1
0.144490112	0.753846794	0.003688652	0.142181	0.052889	-0.69034	ASI2
0.144603083	0.915294708	0.859769494	0.146345	0.02547	0.11697	COG7
0.144616301	0.124190791	0.812322751	0.096135	0.127337	0.089767	PDA1
0.144806266	0.854733623	0.120662207	0.118282	-0.02985	-0.35915	RAM1
0.145720606	0.012654875	1	0.166225	-0.33416	0.039546	ARG81
0.146314989	0.492321556	0.702435074	0.130777	-0.08986	-0.16522	CTI6
0.146746164	0.508142283	0.967394603	-0.73657	0.475952	-0.33314	None

0.146978444	0.737846426	1	0.157501	-0.06205	0.024211	MRX1
0.147038	0.036516079	0.287597168	0.093344	0.16071	0.220605	PEP4
0.147038	0.607278799	0.974692539	-0.10389	-0.05726	-0.04194	NFU1
0.147363886	0.691399034	0.997983923	-0.431	0.190859	0.133902	HSP32
0.147370204	0.240168063	0.771910654	-0.08973	0.096222	0.094191	MDH3
0.147861933	0.802618659	0.927963214	0.137882	-0.04311	-0.07799	MED6
0.148356189	0.294183573	9.09486E-05	-0.14172	0.139445	-0.85919	IRC23
0.148356189	0.745058477	1	-0.47277	-0.1819	-0.14415	YGR067C
0.148437789	0.133602113	0.009452184	-0.11975	0.156255	0.541187	RPS26A
0.148654754	0.455569382	0.625394004	0.166842	0.123651	-0.24559	MEF2
0.148803096	0.321761523	0.906628341	-1.75202	-1.66248	-0.20367	YIL177C
0.149240389	0.379249077	0.060744575	-0.12767	-0.10916	-0.44582	IDS2
0.149581483	0.746430113	0.980898839	-0.12505	-0.04822	0.047191	INP53
0.149626449	0.198842787	0.990208543	-0.09799	-0.11344	-0.03325	TAF4
0.150606632	0.113344883	0.903248727	0.145098	-0.20065	-0.09732	PRP16
0.150806792	0.178180945	0.767199943	0.178201	-0.2155	-0.19185	YBR138C
0.153566267	0.008085082	0.69956533	-0.12716	0.271014	0.164371	MGR2
0.153652773	0.447017626	0.263985261	0.112699	0.08545	0.280722	RPS6A
0.153652773	0.43160484	0.739091337	-0.15644	0.1219	-0.18035	NTC20
0.153935751	0.682492975	0.205928769	0.185042	0.085932	-0.49982	GIP2
0.153935751	0.849224058	0.790203342	-0.13555	0.035467	0.137095	FAP7
0.153935751	1	0.937179795	-0.5579	-0.01635	-0.30213	None
0.15448157	0.376938607	0.933220016	-0.12719	-0.11031	-0.06917	MGM1
0.154614031	0.658859261	0.804181925	0.133389	-0.06627	0.130055	SLM2
0.154773324	0.335205662	0.971609311	-0.20369	0.189087	-0.08754	HEL1
0.155549456	0.015999866	0.13719416	0.0952	-0.18933	0.28873	PUS7
0.155549456	0.865630153	1	0.131742	0.032207	0.027381	MET12
0.155880413	0.113318165	0.669483248	0.130843	-0.18284	0.181885	NUP188
0.156028513	0.469895523	0.509023813	-0.24664	0.179931	0.441562	None
0.156028513	0.038719477	0.901362207	-0.17046	0.295145	-0.11584	ATG31
0.156451566	0.42227856	0.498477715	-0.15672	-0.12524	-0.28484	AFI1
0.156451566	0.162279302	0.943076061	0.258286	-0.32678	0.134156	NUT2
0.156522705	0.946592101	0.053817845	-0.1167	0.015983	0.425586	CWP2
0.157138553	0.002281388	0.904680493	-0.29288	0.695829	-0.19465	None
0.157203636	0.022294635	0.739091337	0.17141	-0.32828	0.200262	FUN30
0.157657956	0.763231981	0.317527924	-0.10866	0.039779	0.252955	PNP1
0.157657956	0.384327883	1	1.897148	-1.67735	-2.61868	None
0.157812734	0.384327883	1	1.894484	-1.67503	-2.61463	None
0.158453395	0.384508616	1	1.890515	-1.67125	-2.60974	None
0.158453395	0.384508616	1	1.890504	-1.67124	-2.60974	None
0.158453395	0.384508616	1	1.890549	-1.67129	-2.60975	None
0.158514028	0.033709464	0.587251805	0.137446	-0.24721	0.221109	MAD1
0.158514028	0.384508616	1	1.890807	-1.67131	-2.61089	None
0.158514028	0.384508616	1	1.890844	-1.67135	-2.6109	None
0.158514028	0.384508616	1	1.890854	-1.67137	-2.6109	YKR032W
0.158514028	0.384508616	1	1.890866	-1.67138	-2.6109	None
0.158563594	0.508078874	0.920656396	-0.13149	-0.08982	-0.07957	RNH201
0.158692031	0.343957891	0.98500242	0.118333	-0.10986	0.043052	SMY2
0.158697818	0.580749101	0.312126896	-0.14718	0.087711	-0.34397	YBR241C
0.158768298	0.398327474	0.255041627	0.098177	-0.08241	0.250838	NOP2
0.158768298	0.384834863	1	1.888366	-1.66895	-2.60801	None
0.158768298	0.384834863	1	1.888394	-1.66898	-2.60802	None
0.158768298	0.384834863	1	1.88843	-1.66903	-2.60804	YOL019W-[illegible]
0.159606769	0.521345246	0.786584362	-0.78421	0.506527	-0.8443	None

0.159777515	0.922137802	1	1.234829	0.20881	0.252285	None
0.160219704	0.533524502	1	0.164035	-0.10732	0.039346	PHO87
0.160219704	0.625197222	1	0.879565	0.479345	-0.13765	None
0.160552268	1	0.744376434	-0.11436	0.003602	0.132154	ELO2
0.160570212	0.928674902	0.979069474	-0.1603	-0.02618	-0.0626	TAP42
0.160941676	0.164539138	0.919926509	0.142208	0.179416	0.087822	SRB6
0.160978149	0.161492284	0.976568193	0.229736	0.291773	-0.09338	None
0.161148028	0.839560652	0.913628943	0.110214	-0.03076	0.070901	URA6
0.16194722	0.279341459	0.08318439	-0.19188	-0.20032	-0.64551	IZH4
0.162917914	0.035135362	0.879698394	-0.14543	0.259802	0.11025	YKL069W
0.162917914	0.662458676	0.93147427	0.131185	-0.06576	0.073854	PEX6
0.16307499	0.252510181	0.685438098	-0.09151	0.099664	0.125098	SCW4
0.163094747	0.203043786	0.675161254	0.173172	0.204858	0.240574	YIL089W
0.164022054	0.405829626	0.757681998	-0.12985	-0.10881	-0.14644	EPT1
0.164059742	0.948230011	0.903709617	0.245123	-0.03388	0.168847	YLR125W
0.16452862	0.908330878	0.312126896	-0.85323	-0.15956	-2.05927	YOL024W
0.165097016	1	0.143859218	0.132615	-0.00048	-0.40481	GPB2
0.165946243	0.87456095	0.313124264	-0.15454	0.037011	-0.36794	CTM1
0.16661475	0.806667431	0.737914729	-0.18327	0.058374	-0.21871	HO
0.166657766	0.013123836	0.522217116	-0.16513	-0.34446	-0.29711	PHO4
0.167010479	0.839560652	0.49313497	0.117352	0.033156	-0.22022	CNE1
0.167107006	0.321935126	0.862693719	0.14137	0.138422	-0.11695	MCM21
0.167127978	0.936093775	0.927489937	-0.12658	-0.01968	-0.07511	EOS1
0.167523055	0.189929789	0.035404019	0.112599	0.1375	0.45434	ELF1
0.167845864	0.575567686	0.466301517	-0.17975	0.11015	0.352313	DAD1
0.16869847	0.029341238	0.89043028	0.096838	0.181256	-0.07072	DCS1
0.16924132	0.887204289	0.778574825	-0.07704	-0.01721	0.083613	PAB1
0.169644786	0.500248241	0.41940157	-0.11604	0.082233	0.241377	PFD1
0.169710099	0.761526771	0.800070771	-0.12062	0.045647	0.122689	BOS1
0.169786519	0.860071452	0.201505014	-0.11939	0.030851	-0.33548	HOS4
0.169815465	0.237866407	0.093444318	0.931312	1.051827	3.346963	None
0.170267339	0.761075278	0.980898839	-0.11926	0.045309	-0.04703	CIA2
0.170286501	0.667488134	0.137694656	0.104744	0.052235	0.327508	TMA20
0.170286501	0.054666041	0.998804008	0.279644	-0.47554	0.091192	RSC30
0.170596619	3.90144E-06	0.411752857	-0.15896	0.54023	0.333826	GPX1
0.170748534	0.440961232	0.522788552	1.143192	0.899617	2.191466	None
0.170850484	0.87456095	0.853569585	-0.09855	0.023863	0.084971	APE2
0.171269438	0.181816644	1	0.11409	-0.14292	-0.01495	NIS1
0.171614525	0.746350726	0.840006224	0.138374	-0.05615	-0.1258	MHP1
0.17168991	0.663621022	0.358747602	-0.9229	-0.46103	-2.45215	None
0.172168122	0.482993634	0.903709617	-0.19114	0.140883	-0.1334	HAP3
0.172173991	0.981817797	0.937363635	-0.13316	0.012395	-0.07332	MCT1
0.172975963	0.734931499	0.358747602	-1.66161	-0.66197	3.946191	None
0.172975963	0.251205476	0.648340079	1.046852	1.167885	-1.63793	YRF1-1
0.172975963	0.892236467	0.915545807	0.143159	-0.03119	0.093367	VAM6
0.172975963	1	1	-1.89568	0.032549	-0.04003	None
0.172975963	1	1	-1.89592	0.038849	-0.04778	None
0.172975963	1	1	-1.89592	0.038843	-0.04777	None
0.173028575	0.802618659	0.733389979	0.137573	0.045394	-0.16878	VPH2
0.173712107	0.014466754	0.778620065	0.29355	-0.62082	-0.32311	ATP10
0.174232243	0.504240033	0.888617774	0.124023	-0.08847	0.093633	NUP82
0.174300678	0.101825222	0.25613335	-0.35957	-0.53802	0.958295	MIP6
0.174726266	0.281123697	0.889649609	0.117618	-0.12621	0.087792	KAR1
0.174887932	0.079277191	0.125375403	0.105834	-0.16798	0.341462	PMA1

0.174887932	0.298926458	1	-0.64874	0.654631	-0.03969	None
0.174928008	0.034320207	0.255041627	0.133686	-0.24816	0.354334	KAP114
0.174928008	1	0.726622371	-0.16984	0.007859	0.213088	HOS2
0.1750056	0.119341769	0.32576519	-1.17488	1.641486	3.07095	YOL013W-/
0.175314834	0.893776678	0.28749674	0.229609	-0.04984	-0.57431	RTA1
0.175586028	0.311884893	0.08245863	0.793667	-0.83848	-3.24344	OSW1
0.175869347	0.612169768	0.961702496	-0.11651	-0.06772	0.056874	ALG11
0.175960004	0.297901989	0.98500242	0.109203	-0.11373	-0.04175	CLN3
0.176229814	0.782040669	0.897848413	-0.14298	0.050759	0.103614	FUN14
0.176290154	0.291249532	0.498094313	-0.13641	0.143329	0.259496	LST7
0.176654124	0.145620859	1	0.557416	0.75608	-0.13171	YIL054W
0.176654124	0.942225336	1	1.444079	-0.21152	-0.37433	None
0.177117248	0.763546663	0.952970302	-0.11459	-0.04372	0.058867	RPB3
0.177234622	0.153838133	0.971609311	-0.12123	0.161409	0.054554	MRPL16
0.177410954	0.922329251	0.830189162	0.954493	0.169491	-0.92027	None
0.177831552	0.146319766	0.733341705	0.118509	0.160927	0.1476	GRX7
0.177831552	0.488044682	0.98500242	-0.09694	-0.07169	0.036693	ARP9
0.17789088	0.009381038	0.740020932	0.184613	-0.41518	0.225736	RAD61
0.179011152	0.87456095	0.69956533	-0.17568	0.043247	-0.23942	CNN1
0.17911209	0.413258346	0.573987041	0.118746	0.101294	-0.20262	MGR3
0.179217139	0.304299791	0.114649943	-0.10654	0.109685	0.353076	RPL12A
0.179629887	0.026850024	0.651381471	0.109477	-0.2135	0.165743	VPH1
0.179768123	1	1	0.08425	-0.00248	0.000197	ZUO1
0.179871587	0.55767454	0.931141702	-0.11262	0.073525	-0.06573	IRC21
0.179871587	0.446288659	1	-0.26446	-0.21285	0.02152	YBR184W
0.179960747	5.14593E-07	0.006151828	0.1221	0.455992	-0.61033	HXT7
0.179960747	0.853463958	0.868996053	0.206501	-0.05648	-0.17065	VPS15
0.180219478	0.010883358	0.927619939	0.136669	-0.30227	0.083625	MSB2
0.180514407	0.210426761	0.997983923	0.113767	0.137585	-0.03813	CAB4
0.181126929	0.199639017	0.269184303	-0.16654	0.204742	-0.43319	APJ1
0.181684979	0.157263376	0.054756353	-0.10655	0.142017	0.40904	RPS9B
0.181684979	0.670717765	0.808567391	-0.20229	-0.10243	0.206229	MPP6
0.181859746	0.442222692	0.983370642	-0.59307	0.474574	0.230964	None
0.182032911	0.758146893	0.676524689	0.561568	0.223945	-0.8155	None
0.182224176	0.156953675	1	0.357388	-0.48735	0.068084	None
0.182474891	1	0.167222206	0.191215	0.010954	-0.58546	CTF19
0.182474891	0.014428866	0.253365907	-0.08916	0.189043	-0.23895	MIA40
0.182764773	0.396462129	0.360540001	1.165595	-1.08537	-3.12064	YLR154C-(
0.182954444	0.682492975	0.900882736	-0.11262	-0.05558	0.08171	YNL217W
0.183089648	0.513831581	0.281242785	-0.11393	-0.08123	0.294888	RRN11
0.183415686	0.146749371	1	0.147656	-0.20365	0.00691	BUD6
0.183510813	0.076368853	0.924605282	-0.17299	0.279502	0.108505	YPL071C
0.183612304	0.820475284	1	-0.57193	0.179064	0.116621	None
0.18391766	0.038862304	0.463829525	0.11071	-0.20544	0.225974	BUD27
0.183987799	0.0108701	1	1.518231	3.803004	1.697432	CUP1-1
0.184387642	1	0.672010939	0.142077	0.006239	0.208515	CST9
0.186060092	0.854733623	1	-0.41528	0.113195	-0.12006	None
0.186351836	0.76984172	0.586189737	-0.23106	-0.08752	0.394024	NEL1
0.186374956	0.98685381	0.73632566	-0.94286	-0.07782	-1.22292	YBL071C-I
0.187333785	0.152505754	0.651238461	-1.38278	1.836246	1.688898	PAU20
0.187348047	0.74444644	0.967394603	-0.09634	0.040462	0.045771	WSC2
0.187693091	0.71619322	0.789517744	0.971823	-0.44985	1.087042	None
0.188622546	0.261791204	0.477818318	0.310982	-0.35605	-0.62383	YML037C
0.188767354	0.130559389	0.927131674	-0.16406	0.23424	-0.10227	YPL229W

0.189145688	0.93221185	0.241562819	-0.09798	-0.01656	0.271805	RCM1
0.189204231	0.971085707	0.000924389	0.168815	0.019023	-0.97891	MET2
0.189204231	0.773803168	0.845726915	-0.30422	0.113985	-0.28077	MDJ2
0.189408369	1	0.739091337	-0.22455	0.001354	-0.2795	YEL023C
0.189829819	0.613678519	1	0.162532	-0.09708	-0.01102	TOF1
0.189929033	0.995214622	0.681411478	-1.61089	0.109295	-2.38294	None
0.190210253	0.914594311	0.976568193	0.970733	-0.18968	-0.42027	None
0.190782975	0.126912899	0.944544569	-0.16134	-0.23456	0.089198	SKN1
0.190872635	0.304258036	0.005956703	-0.76953	-0.82306	4.42991	YIR021W-/
0.191187285	1	0.071166723	0.141003	-0.00792	-0.52283	GIC1
0.191187285	0.911433046	0.63834146	-0.13707	-0.02716	-0.21596	PCL2
0.191432285	4.62271E-06	0.060036243	-0.16259	0.573292	-0.61685	EDC2
0.191432285	0.515859901	0.39206293	0.170268	-0.12343	-0.38473	ISR1
0.191525031	0.151541511	0.661818179	0.140537	0.194292	-0.21325	MRX6
0.191802399	0.572909069	0.651238461	0.094925	-0.06177	0.147385	GET1
0.192003247	0.903977193	0.848611069	0.558812	0.117918	-0.51167	None
0.192171288	0.199326299	0.400503761	-0.21334	0.268348	-0.47504	MOH1
0.192850511	0.613301749	0.808567391	-0.14222	0.08516	-0.14805	MUD1
0.192850511	0.440243906	1	1.012344	-0.87932	-0.06612	None
0.192856478	0.515509269	0.5048946	-0.18749	0.135346	-0.36561	DAL7
0.193608428	0.405088718	0.202387133	-0.95536	-0.85561	-4.10463	None
0.195592398	0.895413787	0.276489336	-0.22537	0.050065	-0.59997	TOG1
0.195592398	0.368823237	0.454257899	-0.10569	0.10121	0.222288	YIR035C
0.1956685	0.611875648	0.845920712	-0.13501	-0.08178	-0.1264	HDA1
0.195934498	0.031947208	0.960632458	-0.08407	-0.16437	0.04326	SRP72
0.196127936	0.066428344	0.7225564	0.11539	-0.19801	-0.15236	BDF1
0.196127936	0.069544572	1	0.101212	-0.17202	-0.01576	STE12
0.196664198	0.379859709	0.855640301	-0.96979	0.880181	0.861466	None
0.196750691	0.693401532	0.340299676	-0.27736	-0.13657	-0.68691	SPO16
0.196941766	0.011612022	0.420929331	-0.10792	0.243012	0.237191	ACP1
0.197866653	0.382143759	0.006383153	0.1041	-0.09818	-0.53984	ALD5
0.198101585	0.059305878	0.992218183	0.094119	-0.1658	-0.03486	ALG2
0.198261579	0.483153849	0.965798756	-0.1183	-0.09221	-0.05862	RFC4
0.198268215	0.087597502	0.76367822	0.243491	0.394337	0.291394	YER137C
0.198581813	0.94982823	0.977896479	-0.10624	0.015449	0.045869	TCD2
0.19858726	0.505136536	0.446640364	-0.0979	-0.07303	0.209258	TSA1
0.198720179	0.715720978	0.379660556	-0.08676	-0.04058	0.20242	TED1
0.198720179	0.196081443	0.828048662	0.285364	-0.37124	0.28766	PPM2
0.199138318	0.157263376	0.600468279	0.200994	0.278315	-0.34221	PPT2
0.199285032	0.292656551	0.909621954	-0.37511	-0.41614	0.272284	YLR358C
0.199677826	0.099555926	0.868031723	-0.17199	0.269573	-0.14884	YDR090C
0.199737764	0.615223063	0.960372867	0.090827	-0.05493	0.047302	PFF1
0.199782566	0.002099596	0.146124605	-0.18896	0.497962	-0.61196	CUR1
0.200783908	0.171300695	0.765014016	0.169798	-0.23076	-0.20333	SGF29
0.201552066	0.759529423	1	0.119048	0.048957	0.016564	APC2
0.201935736	0.157469667	0.046235167	-0.10099	0.140173	0.417451	RPS21A
0.202874654	0.428998997	1	0.135143	0.117672	-0.04358	NHA1
0.202874654	0.99746658	1	-0.09113	-0.00628	0.021754	YFR006W
0.20303601	0.584594986	0.780254446	-0.08132	0.052711	0.094292	KTR1
0.203140428	0.843062252	1	-0.11089	-0.03301	-0.00876	PAN5
0.203305793	0.947727435	0.966082547	-1.21017	0.171816	-0.64907	None
0.203459034	0.239652304	0.926181253	-0.12818	-0.15516	0.082929	RPM2
0.203512955	0.44410329	0.735885155	-0.11141	0.094185	-0.14434	SRF1
0.203954408	0.114362042	1	0.141146	-0.21787	0.022826	PSK2

0.204959679	0.690335313	0.744376434	0.785403	0.395741	-1.00329	None
0.205437174	0.142943596	0.908010779	0.17246	-0.2508	0.125334	HIR3
0.205437174	0.196724669	0.971609311	-0.18757	0.243597	0.089046	DLT1
0.206289046	0.182884497	1	-0.15303	0.2041	0.035741	UBP11
0.206477486	1	0.848379499	-0.95952	-0.04736	0.923785	YBR200W-A
0.206756382	0.003986337	0.536035164	0.089855	-0.23041	0.172631	RRP6
0.207955606	0.112919922	0.750952753	0.122026	-0.19021	0.153213	POM152
0.208090983	0.605408587	0.074638157	1.367597	-0.88037	-6.75373	YGL138C
0.208158114	0.360876565	0.08251531	-0.1331	0.132394	-0.49598	AME1
0.208158114	0.003395103	0.915496392	-0.15648	0.404065	0.109326	MZM1
0.208574357	0.709302791	0.156558309	-0.86583	0.406399	-3.28996	None
0.208574357	0.794730765	0.903248727	-0.11133	-0.04062	-0.08401	MOD5
0.20883049	0.041913401	0.902712809	-0.19872	0.377357	-0.14991	ATG14
0.209350002	0.901896821	0.961702496	-0.49237	0.106607	-0.25995	YHR139C-A
0.209645973	0.440683477	0.794898864	0.08805	0.076082	0.099263	ADH1
0.210427594	0.279903897	1	0.122573	-0.1416	0.01568	MAG2
0.211024985	0.096928954	0.721655021	-0.08875	0.143565	0.12099	YTA12
0.211143874	0.288468769	1	-0.14792	0.167614	0.044201	YOR015W
0.21138804	0.184636081	0.748466881	0.090579	-0.12245	0.115201	HEL2
0.211475428	0.737846426	0.023505165	0.091057	-0.041	-0.42258	OPT2
0.211744758	0.019381107	0.333158461	-0.11506	-0.25046	0.292422	DIP2
0.212069075	0.206000284	0.93741782	0.336048	0.436788	0.200539	YOR376W-A
0.212189292	0.610223632	0.706225178	0.442315	-0.28215	-0.64936	None
0.212330005	0.293219896	1	-0.12474	-0.14103	-0.01827	SEA4
0.213465774	0.615223063	0.336989777	0.161845	0.100525	-0.40951	IPK1
0.213499159	0.812133286	0.634965185	-0.26063	0.089979	0.433499	None
0.213796223	0.319484149	1	0.156305	-0.1697	0.005446	ZDS2
0.213924645	0.122035336	0.731592804	0.092851	-0.14323	0.124812	GCD6
0.213924645	0.039547582	1	0.186809	-0.36772	0.019951	RCO1
0.214088123	0.013695435	0.916400787	-0.10231	-0.23363	-0.07204	SMI1
0.214094234	0.427585494	1	0.122976	-0.11027	-0.04291	HRQ1
0.214788241	0.063687056	0.437337853	0.103074	-0.18511	-0.23064	TGL5
0.2149707	0.281404598	1	-1.70926	1.939872	-2.71352	None
0.215167583	0.098514402	0.146638609	-0.09168	-0.1494	0.309793	NOP9
0.21537152	0.281429987	1	-1.70798	1.938817	-2.71318	None
0.21537152	0.281429987	1	-1.70818	1.938995	-2.71328	None
0.21537152	0.281429987	1	-1.70823	1.939035	-2.71329	PAU1
0.21537152	0.281429987	1	-1.70802	1.938852	-2.71319	None
0.21537152	0.281429987	1	-1.70806	1.938881	-2.7132	None
0.215558573	0.630124855	0.7990478	0.350221	-0.21319	0.394461	YMR001C-A
0.21562352	0.914594311	0.889649609	-0.09792	-0.01983	-0.07919	ILV1
0.21568593	0.546699631	0.643913419	-0.14102	0.100696	-0.23166	RKM5
0.2157726	0.030604	0.073409423	0.092027	-0.189	0.358189	ESF1
0.21614971	0.373168526	0.976378097	0.111647	-0.11126	-0.05126	PMT3
0.216172014	0.464360003	0.069188274	-0.08624	0.072183	0.338629	RPL6B
0.216293915	0.936226276	0.919926509	-0.13669	-0.02339	-0.09435	IPA1
0.216666113	0.527520896	0.629698062	0.124594	-0.09276	-0.2102	UBA4
0.216924307	0.871135401	0.505654891	0.426946	0.115888	0.873657	None
0.217622285	0.886323024	0.622714429	0.177481	0.044169	0.303997	None
0.218745502	0.629248171	0.237069015	0.162056	-0.09875	-0.47908	RGT2
0.218745502	0.308388389	0.915942439	0.138836	0.154402	0.099093	YIP4
0.219268606	0.038508481	0.661818179	0.11277	0.223873	0.181193	ISA2
0.219393989	0.877594103	0.977469861	0.102759	0.027223	0.046503	TIM44
0.219463576	0.322600041	0.222510153	0.109733	-0.11972	-0.33254	PDS1

0.219560298	0.156431496	0.888371569	-0.17451	0.251568	0.144191	MVB12
0.219574486	0.641082995	0.712223429	0.087194	0.051545	-0.12487	CUE5
0.220243608	0.632634392	1	0.345596	0.209047	-0.11349	DMC1
0.220337053	0.314829341	0.077306681	-0.15024	-0.16582	-0.57649	SAM3
0.220682273	1	1	0.122426	0.002707	0.026819	PRP45
0.221083581	0.858867801	0.817868751	0.122098	-0.03545	-0.13155	APM1
0.222072381	0.422272348	0.029665642	0.086172	0.078858	0.397963	RPS26B
0.222339983	0.379249077	0.2432512	-0.09326	0.092511	0.275881	URA5
0.223177113	0.539290348	0.246071819	-0.09321	0.068421	0.274525	KTR7
0.223177113	0.436135587	0.553808105	-1.08381	-0.97512	2.304057	HXT14
0.223606944	0.997790364	0.921286576	-0.4803	-0.03372	-0.33115	YER175W-[illegible]
0.22503297	0.347850933	0.738191837	-0.11465	0.120311	0.153799	TOA2
0.225101841	0.460638341	0.373560685	-0.11141	0.095461	0.275944	SBH1
0.225271916	0.767694173	0.915496392	-0.11585	0.047758	0.08396	PRP9
0.226439876	0.483066965	1	0.097631	0.080523	0.025867	CYM1
0.22726795	0.999497264	0.451184565	0.48106	0.032698	-1.08468	YJL077W-[illegible]
0.227282448	0.953675948	0.87973401	0.652819	-0.09712	-0.57406	MEO1
0.227379895	0.874643786	0.241434728	0.075902	0.020609	0.227627	RPL3
0.227482422	0.998303878	0.949031652	-0.12441	-0.00875	0.072257	STE14
0.227852486	0.427560059	0.545272974	0.072995	0.067029	0.144043	TOM40
0.228336944	0.999282889	1	-0.10208	0.007033	0.007892	BUD17
0.228458493	0.344852441	0.570229598	-0.77054	-0.82919	1.541829	None
0.22892807	0.554757324	0.444392343	0.102198	0.074031	0.232996	MPC2
0.229004785	0.971085707	0.904680493	-0.1041	0.012666	-0.08034	PTC2
0.229004785	0.735101186	1	0.09879	-0.04645	-0.02991	RNH70
0.229239719	0.735873521	0.911946622	0.112148	-0.05264	0.083656	EDE1
0.229760421	0.674015722	0.903248727	0.114813	-0.06372	0.090524	UBP8
0.229760421	0.958254142	1	-0.1592	0.02242	-0.03286	POL4
0.230214192	0.545862186	0.764962215	-0.46208	0.336961	-0.58286	YML054C-[illegible]
0.230316062	0.954188947	0.846846052	-0.13216	0.019446	0.131882	PET127
0.23062417	0.756256683	0.19677862	-1.0548	-0.4539	-4.46612	YJL007C
0.231028675	0.059378831	0.950592115	0.130992	0.244902	-0.07597	MCP1
0.231261928	0.434577301	0.11348234	0.089616	0.081734	0.330849	TIF11
0.232138765	0.011960104	0.206936896	0.235548	0.566525	-0.74606	GAS4
0.232816844	0.472617197	0.976568193	-0.11935	-0.10179	0.056026	TPH3
0.233348221	0.368093975	0.86530488	0.0863	-0.08952	0.08089	RPO21
0.234759384	0.311879301	0.071166723	0.097648	0.111177	0.395932	RPL9A
0.235195956	0.051068941	0.475256698	0.113743	-0.22227	-0.25174	CFT1
0.235871352	0.628871924	0.74594446	-0.31411	-0.19742	-0.42814	None
0.236771955	1	1	0.114901	-0.00285	-0.03966	IVY1
0.236997653	0.860955797	0.79509418	-0.13412	0.039403	0.159006	AST1
0.237002975	0.813026602	0.448042463	0.115538	-0.04181	-0.26544	PSF2
0.237002975	0.296770677	0.737036218	0.146908	0.172414	0.20325	VMA4
0.237160876	0.305237145	0.16004587	-0.09212	0.106182	0.318451	RPS3
0.237160876	0.413242794	0.931568877	0.208198	-0.20032	-0.1357	YHR177W
0.237160876	0.231636316	1	-0.09088	-0.11887	-0.02624	GCR2
0.237498871	0.672129195	0.550781538	-0.19905	0.111952	0.395694	VMA9
0.237498871	0.110173764	1	-0.13116	-0.21735	-0.00996	RAP1
0.237573352	0.071099424	0.920147193	-0.10987	0.200405	-0.07813	RCR2
0.238648058	0.199496245	1	0.215065	0.297592	0.002106	None
0.238802326	0.340221835	0.897848413	-0.08337	-0.09144	0.068543	TAF6
0.240234137	0.660417863	0.500377034	1.313321	0.75619	-2.68059	TAR1
0.240543399	0.84140879	0.13328662	0.07425	0.024054	0.269262	RPS0B
0.240543399	0.008129962	0.681411478	-0.0985	0.250467	0.157871	GCY1

0.242006423	0.283359769	0.628284624	-0.13793	-0.16796	-0.24464	IRC3
0.242115682	1	0.832278865	-0.19633	0.007288	0.211178	BIO4
0.2423147	0.551390968	0.795907717	-0.23436	0.174008	0.278462	YDR246W-A
0.24274964	0.582791209	0.356832107	-0.08986	0.063101	0.235427	YNR021W
0.243219161	0.020478658	0.457306305	0.106236	-0.24447	0.245136	MET18
0.243556489	0.632899025	0.068857176	-0.15053	0.094589	-0.61646	PTK1
0.244079902	0.98408428	0.975062924	-0.78143	-0.0798	-0.39309	None
0.244507972	0.131282664	0.40705101	-0.07608	-0.12154	0.186872	MAP1
0.244609044	0.483066965	0.996250082	-0.23965	0.203396	-0.09229	RPL22A
0.245563631	0.295649423	0.940701984	0.153812	0.184051	-0.09615	CBT1
0.245563631	0.576193096	1	0.158903	0.113975	0.005973	YNL108C
0.245639235	1	0.851745398	-0.51952	0.009289	-0.52198	None
0.245639235	1	1	-0.13262	0.005288	0.000297	FMP25
0.245818816	0.782040669	0.568396701	-0.13232	-0.05398	-0.2596	PBP2
0.245818816	0.97067567	0.929303164	0.096597	-0.01227	-0.06586	AHC1
0.245830781	0.059509508	0.293969078	0.874113	-1.90491	3.27465	None
0.245830781	0.748016729	0.98500242	-0.15603	0.072631	-0.06776	ETP1
0.245945477	0.080547625	0.040274266	-0.07387	0.133492	0.342335	PDC1
0.245945477	0.19339556	0.261118505	0.193063	-0.27605	-0.57961	VAM7
0.245945477	0.374974245	0.978193638	0.166933	0.174875	0.078665	SHG1
0.246664834	0.062193215	0.889649609	0.157333	-0.30232	0.135698	SMC3
0.246991131	0.614870993	1	0.140597	0.093261	0.048098	NKP1
0.247130687	0.220345944	0.617460449	-0.16188	-0.22021	-0.295	APC4
0.247340297	0.896286063	0.735602795	0.117936	0.028902	0.167004	PFA4
0.248057405	0.657516625	0.166524458	-0.56514	0.332493	-2.09055	YBR027C
0.24830257	0.947727435	0.868996053	-1.06093	-0.16918	1.032659	YJR128W
0.24874122	0.290286754	0.046985988	0.089964	0.10952	0.406211	TMA19
0.248845249	0.438716189	0.741079602	-0.73817	0.672584	1.025643	None
0.249636655	0.763573287	0.966082547	-0.12029	-0.05289	-0.06566	IZH2
0.249719239	0.752538044	0.467201862	-0.08451	-0.03924	-0.19439	CEG1
0.24993769	0.058914883	0.306543708	-0.25994	0.500596	-0.72845	AAC1
0.249948378	0.576420472	0.863509016	-0.40167	0.287249	0.388594	None
0.250812357	0.457843853	0.924605282	0.266821	0.242111	0.191557	JLP1
0.250812357	0.472617197	0.924605282	-0.10662	0.093828	-0.07614	PHO85
0.250812357	0.518133314	1	-0.56778	-0.45783	0.16199	None
0.250858673	0.512975922	0.592985751	0.102416	-0.08375	-0.19575	YJR084W
0.250890789	0.164090726	0.971609311	0.127113	-0.19324	-0.06632	URN1
0.250890789	0.049368202	1	0.126664	-0.25722	-0.03493	TSC11
0.251271183	0.915426343	0.969243029	0.080983	0.01747	-0.04306	CDC55
0.25149101	0.846838197	0.710774648	0.126244	-0.04078	-0.19387	COG5
0.25171745	0.384327883	0.385959394	-0.11284	0.117045	-0.28781	AIM46
0.252243273	0.619870553	0.042435201	-0.09394	-0.06218	-0.43356	DMA2
0.252277448	0.54489641	0.385959394	0.082681	0.063682	0.212057	SEC65
0.253972848	0.422246873	0.846605423	0.10896	-0.10622	-0.11401	BRE5
0.254188049	0.430199039	0.77583136	0.117832	0.113114	0.152407	MET31
0.254868426	0.533349611	0.985958815	0.083591	0.066025	0.0363	KGD1
0.255370101	0.504911953	0.185716843	-0.1179	0.098079	0.403802	MRPS16
0.255370101	0.027517618	0.851392759	-0.15746	0.351629	0.161163	ECI1
0.257635878	0.311249398	0.971609311	-0.10552	-0.12573	-0.05568	YDR248C
0.257702883	0.498295015	0.400503761	-0.07443	0.06305	0.189194	SEC62
0.257732931	0.398376237	0.872290708	-0.11334	0.115327	-0.10695	CCP1
0.257797539	0.340284679	0.371124917	-0.08735	-0.09924	0.231462	ESF2
0.257818219	0.828087918	0.856715553	-0.07174	-0.0255	0.072715	TRR1
0.25792259	0.440961232	0.921286576	0.10006	0.094712	-0.07355	SPC3

0.257940154	0.149345776	0.69956533	-0.12604	0.198337	-0.20053	PPG1
0.257942781	0.001335908	0.184032149	-0.08777	0.267796	0.302727	MIC26
0.258616939	0.222579714	0.371371969	0.119843	-0.16631	-0.31702	AAC3
0.259145491	0.008311419	0.030205993	0.106257	-0.28146	-0.52003	ADE17
0.259756712	0.397361658	1	1.498496	1.538385	2.320844	None
0.259756712	0.397361658	1	1.49851	1.538401	2.320809	YLR162W
0.259756712	0.397361658	1	1.498518	1.538409	2.320792	None
0.259756712	0.397361658	1	1.498512	1.538403	2.320805	None
0.25984167	0.782040669	0.755396858	0.571706	-0.24341	-0.8036	YPL038W-A
0.26042529	0.910923942	0.093444318	-0.11565	-0.0263	0.467856	REX4
0.260678949	1	0.061449807	0.090524	0.004363	0.396054	YVH1
0.260678949	0.570704752	0.306543708	-0.13245	0.09845	-0.38201	YNL046W
0.261360372	0.398327474	1	1.488601	1.528326	2.307711	None
0.261360372	0.398327474	1	1.488645	1.528372	2.307731	None
0.261360372	0.398327474	1	1.48866	1.528387	2.307738	IMA3
0.261360372	0.398327474	1	1.488752	1.528482	2.30778	None
0.261360372	0.398327474	1	1.488698	1.528427	2.307756	YOL162W
0.261360372	0.398327474	1	1.488796	1.528528	2.307801	ERR2
0.261687453	0.000288921	0.168851078	-0.10736	0.36151	0.381978	MDM35
0.261898459	0.890100171	1	-0.12252	0.032135	-0.01682	TVP38
0.261960473	0.925367028	1	-0.54213	-0.11059	-0.01733	API2
0.262364643	0.203402724	0.016772267	-0.1126	-0.16225	-0.60416	SUL2
0.262364643	0.885372644	0.969916554	0.446681	-0.12325	0.242015	None
0.264496157	0.996195213	1	0.116364	0.009563	-0.02481	SKI8
0.264524843	0.229077588	0.264879577	-0.07762	-0.10755	0.240839	RBG2
0.264578874	0.690335313	1	-0.088	-0.04997	0.002972	NNT1
0.264669641	0.383120823	0.077306681	-0.08731	0.093119	0.368589	RPS11A
0.26478544	0.026572164	0.769811029	-0.0894	0.205005	0.11974	MRPL40
0.264984343	0.152734962	0.96509354	0.089592	-0.14291	-0.05073	YEH2
0.265092125	0.938960046	0.735465999	0.187484	0.03462	-0.27466	RTS2
0.265092125	0.923429199	0.837344851	0.198452	0.041815	-0.21931	YBR284W
0.265223394	0.45545886	0.617215067	-0.12956	-0.12132	-0.24409	RAD16
0.2652515	0.023226764	0.803017727	-0.11743	0.274892	0.143	PML1
0.266046387	0.459558802	0.162721427	-1.42609	-1.33877	5.866568	None
0.266068831	0.614870993	0.071980397	0.084068	0.05785	-0.35925	TGL4
0.266297218	0.111956571	1	1.409586	-2.77622	-1.61098	None
0.266307186	0.490263266	0.286390317	-0.92693	-0.81508	3.015359	YKL106C-A
0.266545822	1	0.442027801	-0.18006	0.012428	-0.44108	SPO22
0.266926014	0.690335313	0.90428469	0.092432	-0.05277	-0.07772	CKS1
0.267501776	1	0.321715999	1.245171	0.019366	3.743153	None
0.267883307	0.928674902	0.499488496	-0.13404	0.027148	-0.30347	FUR4
0.267883307	1	0.891897251	-0.6553	0.033913	0.585348	YGL041C-B
0.268069048	0.359885231	0.971609311	-0.07035	0.078845	0.037736	HOM2
0.268321062	0.020822428	0.004134287	-0.10922	0.261295	0.676306	RPS27B
0.268321062	0.812803114	0.98500242	-0.25012	-0.09607	0.113026	YGR126W
0.268943931	0.471015153	0.931568877	0.128937	-0.11873	0.089476	MPS1
0.269151101	0.208240841	1	0.105994	0.15315	0.005589	YJL068C
0.269187264	0.387128577	0.084919982	-0.0825	0.087834	0.345089	SSS1
0.269263443	0.706697151	0.630042336	-0.13818	0.07596	-0.25721	TAD3
0.269632921	0.302395299	0.937470988	0.131938	0.163352	-0.0879	YLR283W
0.270450957	0.460298273	0.27982387	-0.37043	0.343707	1.13614	None
0.270552296	0.069544572	0.283104183	0.558261	1.094706	1.741697	YDR215C
0.270936684	0.404471627	0.992021238	0.070669	-0.07349	-0.03022	RAD23
0.271570793	0.114362042	1	1.402512	-2.76757	-1.60519	None

0.272315105	1	0.710913844	-0.1629	-0.01038	0.260286	DBR1
0.27238208	0.516700017	0.791580335	0.076805	0.064713	0.098354	CKB1
0.273152992	0.582123297	0.885798515	-0.23267	0.17316	0.215466	None
0.273152992	0.010643651	0.919926509	0.144983	0.382981	-0.11152	PRM4
0.273269904	0.012231886	0.326601143	-0.08843	0.22937	0.255337	MRPL8
0.273349816	0.378211699	0.536035164	-0.10201	-0.112	-0.22163	ADE5,7
0.273898775	0.427585494	0.680777882	-0.13701	0.13693	0.234698	AIM4
0.274071909	0.386859362	0.243765908	0.596958	-0.66551	-2.12722	None
0.274071909	0.969492509	0.781154036	0.123731	0.016853	-0.16397	HPA3
0.274071909	0.020195187	0.928019622	-0.11179	0.271436	-0.08116	PCL5
0.274071909	0.263065203	0.981181758	-0.12139	0.162031	0.058512	DSC2
0.274199719	1	0.906168502	0.094126	9.17E-05	0.079004	TAE1
0.274272676	0.892680849	0.856715553	-0.31927	-0.08437	0.335839	None
0.275844456	0.679403914	0.571709772	-0.35427	0.209619	-0.735	YBR182C-/
0.275844456	0.864979821	1	0.166628	0.052059	0.039653	YHL008C
0.27591551	0.19026538	0.669524096	-0.07761	-0.11754	-0.13658	URH1
0.276984686	0.999497264	0.840006224	0.072862	-0.00548	0.081611	FOL2
0.276984686	0.125331421	0.889649609	0.231961	0.399393	-0.21041	YPL119C-/
0.277878807	1	1	-0.18304	0.002196	0.040285	MIG3
0.277924263	0.156840624	1	0.159157	-0.2587	0.016615	PSP1
0.278327698	0.335205662	0.803554162	0.399596	0.476872	0.497338	None
0.278327698	0.399977002	1	-0.16249	-0.17237	-0.00871	SEC59
0.278350451	0.304011642	0.169244459	-0.07426	-0.09325	0.272994	CMS1
0.278616717	0.709900242	0.486249492	0.102779	0.057207	-0.24311	NGL1
0.27970107	0.066428344	0.967394603	-0.07807	-0.15715	-0.04462	RAD27
0.280957488	0.415254519	0.061449807	-0.10275	-0.10632	-0.46352	MET30
0.281001218	0.738978817	0.361142562	0.558593	0.286768	1.571573	YDR169C-/
0.281588576	0.407136042	0.663264835	0.078836	0.083135	0.142532	TAF10
0.281588576	0.014466754	1	-0.10421	-0.26916	0.017599	STE20
0.281588576	0.384327883	1	0.453328	0.49923	-0.04117	YLR281C
0.282269706	0.615223063	0.143826946	-0.07159	-0.05068	0.276388	NOP16
0.282269706	0.762648339	0.331418568	0.161862	-0.07647	-0.47089	LAG2
0.282807102	1	0.617215067	-0.10551	0.00784	0.206189	BUR2
0.2830323	0.413532829	0.05005618	-0.07219	-0.07526	0.34325	RPC82
0.28305633	0.097254638	0.017068248	-0.08786	0.163103	0.489775	RPL34B
0.28305633	0.062985577	0.411976149	-0.08693	-0.17782	0.228295	IKI3
0.283821183	0.914594311	0.702041373	0.09529	0.022045	-0.15859	ICP55
0.283837129	0.934679773	0.201505014	0.083675	-0.01668	0.296572	NOP6
0.283837129	0.843062252	0.326601143	0.114088	0.03976	-0.33571	MNT4
0.283877634	0.900565868	1	-0.10637	0.027096	-0.00238	ECM31
0.283987881	0.246040539	0.978338942	0.134685	0.188556	0.067811	YMR114C
0.284418062	0.119397766	0.837446458	0.146957	-0.2622	0.16885	RBH2
0.284882787	0.902052728	0.739235535	-1.37811	-0.33332	-1.87978	None
0.285076511	0.332342438	0.675803696	-0.07569	0.0919	0.133949	YPT1
0.285179756	0.128825994	0.469965664	-0.89698	1.530673	2.248352	YPR159C-/
0.285517801	0.614651456	0.904680493	0.093558	-0.06701	0.080822	NVJ2
0.286165394	0.096330761	0.016354225	-0.08872	0.166169	0.50158	RPL31A
0.286371638	0.802618659	0.550781538	0.90069	0.370057	2.009709	YCR108C
0.286371638	0.150555145	0.637177071	0.126706	-0.21191	-0.24121	SSY1
0.286668276	0.666883176	0.911946622	0.129679	-0.08179	0.107943	CEF1
0.287791843	0.048583022	0.765014016	-0.16391	0.354291	0.23131	AIM32
0.289257986	0.892744236	0.446640364	-0.26225	0.070861	-0.67468	YEL014C
0.289361409	0.06762385	0.077007481	0.099286	-0.20332	0.441118	RRN6
0.290534032	0.861419309	0.818523526	-0.1134	-0.03686	0.138955	NIP100

0.29064775	0.652939653	0.292035407	-0.23017	0.150706	-0.71421	RRT6
0.290810456	0.052331103	0.764340274	-0.08446	0.181053	0.120591	VPS68
0.291233786	0.728435834	0.507217503	0.432859	-0.23747	-1.04168	None
0.291378134	0.565156176	0.053817845	0.103218	0.082769	-0.49088	YFR045W
0.291848358	0.670089723	0.557079281	-0.12081	-0.07596	-0.26367	HEM14
0.291997894	0.332602552	0.023617213	-0.07121	-0.08764	0.387153	DIM1
0.291997894	0.589760072	0.863302781	0.151259	-0.11579	-0.15983	NFI1
0.292073414	0.805302069	1	0.078005	0.032125	-0.02945	CDC10
0.293061945	0.220821919	0.577843626	0.123543	0.182915	-0.26149	SNT309
0.293061945	0.346488241	0.790203342	-0.08908	-0.10716	-0.11879	CEX1
0.293115691	0.628414107	0.081519515	0.060678	-0.04262	0.268127	GUA1
0.293263457	0.19339556	0.706497696	0.101329	-0.15814	0.170713	SHQ1
0.293670621	0.259482058	0.186036369	-0.07503	0.104654	0.276491	RPL2A
0.29389959	0.568084758	1	0.098319	0.078747	0.009554	FUN26
0.296087169	1	0.411976149	-0.11709	0.000341	-0.31452	GPI2
0.296372391	9.67595E-05	0.822329423	-0.07343	0.279598	0.089981	NNR2
0.296645788	0.116008771	0.89719832	-0.15968	0.290454	-0.14643	HNT3
0.296717351	0.837878157	0.507578381	0.066053	-0.02424	-0.15667	RET2
0.297561113	0.357606716	0.855640301	-0.1949	0.230838	0.213524	YAL037W
0.297561113	0.243460814	0.868996053	0.083233	0.119832	0.086702	PHB2
0.298072898	0.795533688	0.897056392	-1.02621	0.423215	-1.02306	None
0.298823418	0.867434951	0.667670933	-0.10439	0.033496	-0.19262	HEM4
0.298943582	0.172864615	0.537924573	0.073825	-0.1205	-0.16835	ALE1
0.298943582	0.672577856	0.716456431	0.113537	-0.07203	0.186943	SEC15
0.299170258	0.389906374	0.458610066	-0.33508	0.372142	0.852905	YOR105W
0.299753508	0.371648425	0.218691212	0.073596	-0.08603	0.261881	RTC5
0.300228168	0.196724669	0.25778068	0.226041	-0.35803	0.765172	None
0.300439893	0.04046433	0.927229736	0.082585	0.189316	-0.06392	GFD1
0.300538674	0.083428889	0.058847237	-0.06741	-0.13453	0.320626	NUG1
0.300538674	0.367479128	0.437847764	-0.52921	-0.6315	-1.57228	YLL066C
0.300601699	0.20840105	0.971609311	0.930711	1.443074	-0.44171	None
0.303282925	0.116802721	0.077306681	-0.082	0.151167	0.372374	RPS2
0.303594319	0.801294446	1	-0.21542	-0.09179	-0.01056	YLR146W-A
0.303665899	0.583819577	0.328463464	-0.09814	0.077183	0.298637	ILM1
0.30560973	0.899105948	1	-0.13018	-0.03488	0.005606	YJL206C
0.305839844	0.019432143	0.18925097	0.105299	0.272895	0.394431	TIM13
0.306037814	0.987136522	0.28514767	0.074326	-0.00802	0.241859	SUI3
0.306631119	0.440961232	0.194652731	0.127465	-0.13298	-0.47433	ATR1
0.306631119	0.48908547	0.7990478	0.338865	-0.32394	-0.4529	YCL001W-B
0.306631119	1	0.930953395	0.065893	0.003286	-0.04963	MDM38
0.307571069	0.04471877	0.283510967	0.142739	-0.32843	-0.46589	KIP1
0.307910947	0.773803168	0.411752857	-0.13061	-0.0618	-0.3595	HST4
0.308474954	0.21006721	0.824212757	0.116808	0.180782	-0.14528	COX20
0.308493907	0.888561341	0.990110569	-0.52481	-0.15096	-0.24596	YMR247W-A
0.308502968	0.472617197	0.855814602	0.095086	-0.09379	0.106671	PMR1
0.308847289	0.079277191	1	0.104173	0.213531	0.03556	ESA1
0.308975119	0.596380201	0.702041373	-0.93206	0.704736	-1.64497	None
0.309304202	0.795533688	0.647387848	-0.07149	0.031396	0.139889	EMC4
0.309768212	1	1	0.085981	-0.00217	0.028111	MCX1
0.309959082	0.176205372	1	0.098928	-0.16409	0.033099	PRP2
0.311019254	0.533524502	0.352373517	-0.12925	0.113035	-0.38602	GTO1
0.311308256	0.459813614	0.787324112	0.066461	-0.0674	0.092482	VMA2
0.311319327	0.177242556	0.599665071	0.090744	-0.15023	0.192911	RAT1
0.311319327	0.889353732	0.919271701	0.129853	-0.03769	0.108375	SPO75

0.311828269	0.957701635	1	-1.13205	0.179691	0.430731	None
0.31279374	0.293605247	0.983370642	0.149516	-0.20518	-0.076	SAP1
0.313312764	0.151210483	0.911946622	0.094084	-0.16488	-0.08193	TAF2
0.313370952	0.501427533	0.871373143	0.153099	0.143228	-0.16141	FOX2
0.313527438	0.860071452	0.436621477	0.121395	0.041546	-0.32718	GUF1
0.313697952	0.289155055	0.009681125	-0.07631	0.105056	0.47664	RPS10A
0.31473832	0.135871655	0.559860815	-0.23215	0.41626	-0.52321	YDL186W
0.315516374	0.758162333	1	-0.07092	-0.03633	-0.01838	ELP4
0.315616763	0.036314393	0.540074298	-0.10227	0.244966	0.239166	YOR020W-[illegible]
0.315616763	0.486077625	1	-1.35845	-1.32854	1.884443	None
0.315616763	0.486077625	1	-1.35845	-1.32854	1.884442	None
0.315629158	0.388201329	0.029966612	-0.07727	0.089497	0.424307	GIM4
0.316287198	0.313190477	0.882626496	-0.09933	-0.13179	0.100899	SET1
0.316287198	0.486407666	1	-1.35406	-1.32422	1.87909	None
0.316287198	0.486407666	1	-1.35407	-1.32423	1.879091	None
0.316287198	0.486407666	1	-1.35408	-1.32424	1.879091	YOR072W-[illegible]
0.316764556	0.486638529	1	-1.3514	-1.3216	1.875668	None
0.318051793	0.207400802	0.076212737	-0.10709	-0.17009	0.501648	REV1
0.318051793	0.051425193	1	0.105242	-0.24011	-0.03417	SGO1
0.318547896	0.929321848	1	0.340941	0.075924	0.134107	YOL038C-[illegible]
0.318686644	0.138242749	0.350046527	-0.08747	0.158017	0.266515	ENV10
0.31886875	0.515305162	0.870558299	-0.1155	-0.10657	-0.12374	ELG1
0.319445411	0.767134842	0.274728931	-0.21171	0.104656	-0.72275	FMT1
0.320021795	0.887204289	0.702041373	-0.29997	-0.0897	0.535804	YHR050W-[illegible]
0.321330557	0.295761003	0.98500242	0.108392	-0.14997	0.05439	ORC6
0.322716309	3.35757E-13	0.733389979	-0.06944	0.466863	0.113701	CAR1
0.322738784	0.060866598	0.847404354	0.120187	-0.26728	-0.14205	TFC4
0.322902397	0.418783519	0.090019922	0.085864	0.095395	0.393561	RPL11A
0.323315124	0.281429987	0.535437422	0.10361	-0.14788	-0.24793	SPC42
0.323391198	0.390960813	0.818523526	0.137276	0.160932	-0.1784	MRX9
0.32389738	0.122042485	0.154422599	-0.0951	0.17956	-0.38801	SNF11
0.324126642	0.532413738	0.871066692	-0.07032	-0.06332	0.075967	GCD7
0.324990989	0.551240588	0.979069474	0.104739	-0.09178	-0.0565	BUD9
0.325077275	0.666900591	0.846846052	0.957582	0.641784	-1.10816	URA3
0.325604383	0.890501573	0.656171223	1.305113	0.379511	-2.21445	None
0.325968779	0.268778676	0.949031652	-0.09705	-0.14167	-0.06787	SEC1
0.325968779	0.209021864	0.976378097	-0.08852	-0.14211	-0.04987	LEU3
0.326053133	1	1	-0.22593	0.001985	-0.06575	None
0.326829307	0.879700572	0.86530488	-0.77377	-0.24464	-0.89643	YJL052C-[illegible]
0.327147388	0.539585697	0.166524458	-0.06205	0.055378	0.251429	PMT4
0.327220197	0.933247294	0.137628042	0.062358	0.01365	0.26412	ZPR1
0.327220197	0.560060607	0.731592804	-0.13117	0.113072	-0.21771	DDL1
0.327294861	0.479322493	0.737914729	-0.32685	0.325095	-0.5343	COA6
0.328094405	0.492472465	0.371343822	0.090569	0.088595	-0.27288	LGE1
0.328852529	0.729751469	0.276906403	0.690026	0.397523	-2.52267	ANS1
0.328852529	0.48908547	0.721271027	0.134968	-0.13374	-0.23109	APM2
0.329102469	0.759694936	0.758049402	0.135656	0.070878	-0.21046	PHR1
0.329683224	0.068396254	1	0.080428	-0.17689	0.016607	SRP101
0.329716205	0.068479815	0.434148825	-0.08177	-0.17946	0.228368	URB2
0.329867973	0.662458676	0.517080711	-0.09295	-0.0643	0.231999	CSF1
0.330567777	0.089587852	0.985096068	-0.08849	-0.1839	-0.04467	TRF5
0.331694619	0.041913401	0.259100427	-0.09205	-0.22256	0.327038	UTP25
0.331752063	0.013218736	1	-0.09697	-0.27822	0.034929	CDC5
0.333008236	0.885372644	0.635840497	-0.21527	-0.06675	0.447782	PRM3

0.33402172	0.568084758	1	1.254635	-1.08341	-1.8342	YBR298C-/
0.33402172	0.568084758	1	1.254637	-1.08342	-1.8342	MATALPHA1
0.33402172	0.568084758	1	1.254641	-1.08343	-1.83418	None
0.33402172	0.568084758	1	1.254644	-1.08343	-1.83417	None
0.33446962	0.367878735	0.020112784	-0.06944	-0.08702	0.417514	HCA4
0.33446962	0.568257317	1	1.251577	-1.08059	-1.83009	None
0.33446962	0.568257317	1	1.251588	-1.0806	-1.83009	None
0.33446962	0.568257317	1	1.251575	-1.08059	-1.83008	YBL112C
0.33446962	0.568257317	1	1.250445	-1.07954	-1.82858	None
0.33446962	0.568257317	1	1.250457	-1.07955	-1.82858	None
0.33446962	0.568257317	1	1.250458	-1.07955	-1.82858	None
0.33446962	0.568257317	1	1.250486	-1.07959	-1.82859	None
0.33446962	0.568257317	1	1.250504	-1.07961	-1.8286	None
0.334736106	0.431585405	0.119934904	0.072567	-0.08104	0.321607	RPC31
0.334736106	1	0.485259038	0.088972	0.001488	-0.23384	UBP5
0.334736106	0.731503094	0.781511608	0.122332	-0.07101	-0.18115	DGR2
0.336569948	0.00332216	0.737914729	-0.88534	3.294633	1.134506	COB
0.336569948	0.304757859	0.924605282	-0.08188	-0.1143	0.068778	VTC2
0.336569948	0.146651273	0.976568193	0.092453	-0.17081	-0.05265	BUD7
0.338217104	0.07307444	0.821241649	0.069518	0.15285	0.092513	RPN13
0.339221641	0.482993634	1	-0.09148	-0.09338	0.030446	BYE1
0.340867276	0.267877158	0.786584362	0.100888	-0.15195	-0.14878	SIR3
0.341019864	0.987715638	0.39284795	0.085627	0.009782	-0.25661	GRS2
0.341019864	0.82837317	0.790203342	0.132191	0.054965	-0.1925	QRI7
0.342933023	0.332602552	0.538130573	0.12541	0.169617	-0.30908	BUD5
0.343424416	0.729751469	0.058656331	-0.06505	0.038514	0.335386	NAM9
0.344272488	0.089471649	0.359924954	-0.0837	0.177623	-0.26271	MED7
0.344719889	0.286937957	0.795907717	0.359398	0.526496	0.516924	None
0.344719889	0.624879898	0.981181758	-0.0803	-0.06238	0.044171	PAL1
0.345151456	0.989216982	1	0.108506	0.011931	-0.0218	MEC1
0.345294697	0.519603006	0.621646201	0.064281	-0.06165	0.140236	NOP58
0.345388905	0.760989947	1	-0.19509	-0.10413	0.028512	None
0.3461671	0.737846426	0.876848679	-0.10071	0.058288	0.109856	CUS2
0.346923071	0.021842714	0.539256261	-0.13606	0.373472	-0.33588	SNA2
0.34693915	0.712726184	0.207294989	0.063849	0.039914	0.251435	RPL10
0.347047312	0.028907865	0.187978416	0.104661	0.27645	0.424078	SNC1
0.347492646	0.023493222	1	-0.09275	0.252474	0.033072	FLX1
0.347579309	0.725121662	0.860528802	-0.07038	0.042526	0.083215	IRC22
0.347579309	0.085297697	0.985096068	-0.11437	0.246442	-0.05939	PEX22
0.347756232	0.181661685	0.937363635	-0.08395	0.146864	-0.06496	TVP18
0.347756232	0.885372644	0.98500242	-0.07837	-0.025	0.040942	MAS2
0.348098689	0.985451395	0.750296312	0.530311	0.065031	-0.87287	TAR1
0.348099607	0.057915483	0.731592804	-0.07447	0.17416	0.128634	SPE1
0.348461573	0.420342557	0.643429399	-0.13386	0.155279	0.283025	EGO2
0.349509922	0.404694575	0.932528466	-0.10782	-0.12946	0.085951	ROM2
0.35157158	0.662458676	0.420929331	0.195881	0.141021	-0.57341	DIN7
0.351908627	0.095680569	0.007546041	-0.08996	0.190385	0.618804	RPS28A
0.353326542	0.71619322	0.992505689	-0.07141	0.044573	-0.03527	URE2
0.353470932	0.267345812	0.182165955	-0.07172	0.110088	0.296513	RPL21A
0.354313131	0.149532164	0.722897191	0.070479	-0.13346	0.125593	PUS4
0.354931695	0.591976174	0.667670933	0.058632	0.049928	0.120278	TIF34
0.355164572	0.43696685	0.875200775	0.723502	0.826541	0.839266	None
0.356040108	0.518133314	0.088859612	-0.17555	-0.17214	-0.84931	TOP3
0.356040108	0.273631754	0.984769539	-0.62197	0.931843	0.320297	YMR242W-/

0.356353398	0.880383419	0.697639077	0.080789	0.027307	0.156619	CDC50
0.356353398	0.78141125	0.886187413	-0.06647	0.033494	0.071815	TAF14
0.357092325	0.865572034	0.785906893	-0.42631	-0.15331	0.657239	None
0.35719746	0.427199949	0.406901206	-0.13678	-0.1602	-0.41322	PDR11
0.357219564	0.402273369	0.48488876	0.083259	-0.10199	-0.22807	RKM1
0.357602122	0.853333651	0.24850108	0.078648	-0.03017	0.297575	LOC1
0.359115951	0.398327474	0.688444366	0.097247	0.11997	-0.19253	APC9
0.359296449	0.225615871	1	0.206642	-0.34578	0.072441	RIM9
0.360414799	0.782318684	1	0.438894	-0.22453	0.073545	JIP3
0.360884325	0.632634392	0.805275919	0.141071	0.110844	0.203979	EAF5
0.360981817	0.592929724	0.785906893	0.091373	-0.07864	-0.14015	GIP3
0.361277469	0.294101962	0.689510419	0.093232	0.13913	0.18507	LTO1
0.362144443	0.97665513	0.961702496	0.066395	-0.00971	-0.04619	REX2
0.362569633	0.013423142	0.292698979	0.080596	-0.24446	0.287438	RET1
0.362934866	0.954916262	0.7225564	0.086852	0.016208	-0.15714	RPN14
0.363083128	0.53361689	1	-0.196	-0.1891	-0.03694	MSH4
0.363885381	0.021143437	0.554977541	-0.07528	-0.21549	0.188611	PWP2
0.364453835	0.300891646	0.408117554	-0.0881	0.130014	0.270205	OMS1
0.364879872	0.682008236	0.90423793	-0.22675	-0.15818	-0.22876	GPI19
0.36514907	0.118160579	0.984628847	0.097105	-0.20127	0.053839	CDC25
0.36518532	0.948002424	0.950592115	0.248564	0.05056	-0.18549	YOR034C-A
0.365273726	0.549658075	0.920011392	-0.10786	0.101498	0.097634	INP54
0.365538725	0.788420725	0.908303081	0.09828	-0.04918	0.096213	SEC7
0.365829248	0.121986295	1	-0.05722	-0.11717	0.009661	PRS3
0.366743749	0.488031192	0.878688605	0.165369	-0.1763	-0.18675	RAD34
0.366857236	0.412595975	1	-0.08948	0.109067	-0.00824	LPX1
0.367015439	0.748281711	0.024892656	-0.10232	0.059676	-0.63002	IRC15
0.367588755	0.36194188	0.403639619	0.067766	-0.09122	0.211059	CWH41
0.368742199	0.483066965	1	0.146701	-0.15899	-0.06523	None
0.369712337	0.938960046	0.264879577	-0.06966	-0.0155	-0.26114	CUZ1
0.370144204	0.312955729	0.807701151	-0.08308	0.121331	-0.12108	PEX11
0.370682001	0.460954134	0.704009942	0.089883	0.101274	-0.17455	PTP1
0.370948395	0.500005284	0.739091337	0.118975	-0.12449	0.209646	MYO2
0.371008039	0.579910404	0.630792731	-0.07883	0.07081	0.177197	MSC6
0.371381933	0.653413831	0.358747602	-0.07131	0.054201	0.236657	SOP4
0.371381933	0.805302069	1	-0.11951	0.056759	0.030308	ECM9
0.372104548	0.189891941	0.664126703	0.092391	-0.16752	-0.19665	RLF2
0.372347038	0.783211483	1	0.076973	-0.03961	0.024452	TOM1
0.372838178	0.317422835	1	-0.07435	-0.10878	0.034181	FLC2
0.373167906	0.121199858	0.018622221	-0.07671	0.158955	0.499144	RPS12
0.373167906	0.585153927	0.846605423	-0.08717	-0.07788	0.113981	DUO1
0.373272047	0.752831669	0.48488876	0.08672	-0.05065	0.245238	PRP39
0.374007542	0.332602552	0.689558052	0.077973	-0.11186	0.158726	SMY1
0.37482818	0.470910484	0.906168502	-0.08632	-0.09642	0.087058	GEA1
0.375334244	0.750994992	0.35748349	0.083071	-0.04903	-0.27767	SNF6
0.37547895	0.714010588	0.737914729	1.188046	-0.79814	1.578356	None
0.376220386	0.490597664	0.50572517	-0.06046	-0.06481	0.166998	LCB2
0.376220386	1	1	-0.08698	-0.00216	0.021249	RRG7
0.37622516	0.707813933	0.215364043	-0.10246	0.06841	-0.41827	ISC1
0.376599033	0.972383608	0.689558052	-0.0753	-0.01175	-0.15315	MDL1
0.377001755	0.059509508	0.019864748	-0.07297	-0.17929	0.475073	UTP10
0.377035025	1	0.13719416	-0.21253	0.00677	-0.98772	PIR5
0.377088928	0.048583022	1	1.027919	-3.24256	-1.12655	YLR012C
0.377329602	0.055084413	1	-0.288	0.711007	-0.07091	REC102

0.377623831	0.012650856	0.850731953	-0.08565	-0.26916	-0.11081	PCL9
0.378500457	0.305193244	0.723935587	0.192622	-0.29204	-0.35829	SRX1
0.378500457	0.761207443	0.798716083	-0.076	0.042989	-0.11594	VRP1
0.378500457	0.29048058	0.850738592	-0.12815	0.198018	-0.1652	ART10
0.379420701	0.557203364	0.286665818	0.062398	-0.05976	0.231822	ACL4
0.379485109	0.972056407	0.285413202	0.112959	0.017884	-0.41891	CTK2
0.379995563	0.758146893	0.711247098	-0.08175	0.047204	0.158973	SAL1
0.380147922	0.837034378	0.009538243	-0.08032	-0.03444	-0.56398	ARG5,6
0.38028707	0.794730765	0.561585828	0.07108	0.035866	-0.18152	VTI1
0.38028707	0.993185803	0.801754205	-0.08328	0.009159	-0.12616	HST3
0.38035117	0.781865517	5.21012E-05	-0.09265	-0.04879	-0.89833	YJL213W
0.38035117	0.210094783	0.490932171	0.107991	-0.19215	-0.30721	BIT61
0.38035117	0.779436383	0.602672839	0.087699	0.046694	-0.20979	DNM1
0.38107788	0.912696041	0.667670933	-0.1008	0.028352	0.216888	DUS4
0.381758996	0.729652252	0.283481217	1.180842	-0.76985	-5.0448	None
0.381930946	0.202258032	1	-0.2189	-0.39636	0.048039	BSC5
0.382345785	0.849224058	0.971609311	-0.7659	-0.30823	0.528169	None
0.383033859	0.551128078	1	-0.09376	-0.09129	-0.0235	RTC1
0.383434748	0.796926114	0.688444366	0.11392	-0.05735	-0.23663	YDR249C
0.383557233	0.094685918	0.996250082	-0.14067	0.316051	-0.07038	YDR003W-[illegible]
0.384097867	0.683010493	0.894989694	0.12878	-0.09322	0.14036	ARV1
0.385397203	0.065404264	0.98622493	0.06276	-0.15436	0.034697	SRP54
0.385449455	0.068453606	0.616944383	0.065482	-0.1596	0.155226	NUP85
0.386567145	0.15633472	1	0.100409	-0.1996	-0.00628	MSH2
0.386899036	0.658845761	0.017068248	-0.14629	0.113145	-0.97227	DIA1
0.386899036	0.516999501	0.735602795	-0.08702	0.090397	0.159677	BIL1
0.386927242	8.02366E-09	1	-0.07634	0.472656	-0.01944	OM45
0.387557075	0.487051554	1	-0.51899	0.562869	0.063686	None
0.387670868	0.106565166	0.164835808	0.126649	0.279248	-0.57246	SIA1
0.387670868	0.039547582	0.287070553	-0.59827	-1.74413	2.638145	PES4
0.388421905	0.996223442	0.539379949	-0.07911	0.008142	0.212304	ALG8
0.389083167	0.841196109	0.99763155	-0.09353	0.040026	-0.04671	HYM1
0.389530323	0.934679773	0.818523526	-0.08368	0.02024	0.122961	LCB3
0.390430297	0.386354047	0.003119282	0.337323	-0.46075	2.871864	None
0.390537503	0.267877158	0.587019533	0.093135	0.153357	-0.23298	MDM31
0.390728257	0.72990982	0.721271027	0.118877	0.076885	-0.22824	DAD2
0.391179222	0.047950909	0.251662308	-0.08085	0.212664	0.323954	YDL121C
0.391179222	0.607973187	0.614405454	-0.05703	-0.05031	0.137044	TOS1
0.391330896	0.486326234	0.226565606	0.081991	-0.09169	0.341557	APM3
0.391412308	0.681412538	0.404196228	-0.47563	-0.34992	-1.65503	IRC13
0.394071196	0.351913952	0.889079564	0.11584	0.1663	-0.13202	ATS1
0.394071196	0.622084537	0.971609311	0.086063	-0.07392	0.058242	SLH1
0.394556513	0.051638227	0.106067898	-0.09166	0.238489	-0.46106	GIS3
0.394556513	0.57699983	0.927845118	-0.07021	0.066254	-0.06396	APP1
0.394650502	0.913562569	0.91247927	0.079261	0.022804	0.080048	SML1
0.394756495	0.370720975	0.931005601	0.077048	-0.10761	0.068083	RFT1
0.395457605	0.190275098	1	0.088042	-0.16672	-0.01365	LAA1
0.396191694	0.945012036	0.252517824	-0.87879	0.187702	-4.34664	None
0.39643532	0.095680569	0.818523526	-0.11538	-0.26684	-0.1719	KIC1
0.39643532	0.472617197	1	-1.11401	1.295403	-1.92421	None
0.39643532	0.472617197	1	-1.11401	1.295406	-1.9242	YLR154C-[illegible]
0.396451884	0.498387456	0.05699962	1.016112	1.114145	6.725497	None
0.396451884	0.472617197	1	-1.11059	1.291674	-1.919	None
0.396451884	0.472617197	1	-1.11061	1.291693	-1.91903	None

0.396451884	0.472617197	1	-1.11047	1.291578	-1.91897	YCL068C
0.396451884	0.472617197	1	-1.11049	1.29159	-1.91897	None
0.396451884	0.472617197	1	-1.11063	1.291706	-1.91903	None
0.396451884	0.472617197	1	-1.11065	1.291722	-1.91904	None
0.396451884	0.472617197	1	-1.11054	1.291634	-1.91899	None
0.396451884	0.472617197	1	-1.11052	1.291613	-1.91898	None
0.396451884	0.472617197	1	-1.11067	1.291744	-1.91905	IMA3
0.396451884	0.472617197	1	-1.11057	1.291654	-1.91899	ATG16
0.396451884	0.472617197	1	-1.11058	1.291661	-1.91899	None
0.396451884	0.633671893	1	0.293958	-0.24854	9.18E-05	None
0.39736564	0.653413831	0.931005601	0.065011	0.052096	0.057602	MDE1
0.39736564	0.898322904	0.985234915	-0.06481	0.020731	0.037027	DPM1
0.397934074	0.763546663	0.946814146	-0.07987	-0.04651	0.064878	SRS2
0.398161372	0.102281008	0.893930829	0.866372	2.056152	1.236714	YDR366C
0.398775963	1	0.90453709	0.110054	0.008268	-0.11807	CAK1
0.399248532	0.708957109	0.444473065	-0.09282	0.064716	0.290921	URM1
0.399263239	0.110870889	0.30954724	-0.0981	0.21914	0.366898	IRC19
0.399486128	0.011098952	0.800614102	-0.08247	0.273958	-0.12991	OPY2
0.399486128	1	0.978193638	0.099694	-0.00761	0.062643	GOS1
0.400275733	0.220839812	1	0.524577	0.951711	-0.09652	None
0.400742201	0.909881518	0.040274266	-0.06104	0.018199	0.378375	RPB8
0.400799258	0.860071452	0.10735086	-0.69574	0.271862	3.823678	YEL009C-A
0.40115349	0.788420725	0.875100022	-0.0686	0.036742	-0.08436	YRM1
0.40115349	0.63838067	1	0.069028	-0.05795	-0.02016	RKM4
0.401411314	0.989163332	0.517082803	0.093389	-0.01154	-0.26589	SCS3
0.40296719	0.676672695	0.748790103	-0.69483	0.514662	-1.32363	None
0.403894146	0.776883749	0.573905565	-0.06195	0.034661	0.162675	VOA1
0.403894146	0.507001261	0.600468279	-0.61027	0.654348	-1.59569	VPS63
0.403894146	0.596380201	0.7990478	-0.05579	0.051725	0.089148	PHO86
0.404135059	0.184976433	0.023954838	-0.06636	0.128444	0.444227	RPS15
0.404206899	0.953675948	0.255041627	-0.87123	0.173624	-4.35305	None
0.404884374	0.460638341	0.2432512	0.175448	-0.21273	-0.72886	DAN4
0.405542876	0.017727781	0.717477893	0.075766	-0.24142	0.151894	NAM7
0.406447371	0.628871924	0.372341789	-0.81158	-0.70517	-3.53082	None
0.406447371	0.864977468	0.620580042	0.110791	-0.04431	0.272229	KEG1
0.406447371	0.12956062	0.756960007	0.077287	-0.16875	-0.13949	MCM5
0.406447371	0.132874131	1	-0.08061	-0.17441	0.030211	SUM1
0.406604994	0.202307461	0.7225564	-0.11524	-0.21838	0.227556	RRN5
0.406916571	0.125506798	1	-0.59806	-1.38954	0.211378	None
0.408096993	0.516999501	0.98500242	0.096158	-0.10461	0.057504	NUP120
0.408362401	0.295899479	0.328203537	-0.06486	-0.10539	0.2408	SXM1
0.40851922	0.958639237	0.259295923	-0.87009	0.16577	-4.34461	VTH1
0.408829096	0.052331103	0.462275118	-0.07479	0.200053	0.234048	TIM17
0.409337584	0.008063203	0.292547062	0.076335	-0.26939	0.298225	NOP14
0.409337584	0.114760758	0.667995836	0.082365	-0.18729	0.187607	AFT2
0.40954051	0.735101186	0.058656331	-0.07522	0.049563	0.439621	RPC19
0.411973009	0.874643786	0.997577123	-0.23566	-0.09051	-0.12391	SPO11
0.412754391	0.806613916	0.716456431	-0.10596	-0.05429	-0.21495	KAR9
0.414238114	0.391083591	0.962371769	-0.07058	0.09793	-0.05357	LSM4
0.414740444	0.791197208	1	-0.0596	0.032402	0.029511	DAK1
0.415754315	0.745058477	0.484106841	-0.58736	0.372649	1.81626	BUD25
0.415754315	0.884663483	0.488400105	-0.12653	0.046657	-0.38528	YPL191C
0.416125084	0.371424217	0.373162993	-0.15123	0.218407	-0.53494	YKL107W
0.416947561	0.770350536	0.859694751	-0.05128	0.030025	0.069574	PBP1

0.417423315	0.950685743	0.317145408	0.087862	0.019091	0.338023	HTL1
0.417496618	0.100174653	1	0.094398	-0.22523	-0.0035	CEP3
0.418093106	0.224402423	0.003209307	-0.06448	0.119716	0.54105	RPL38
0.419611321	0.923264493	0.281242785	-0.41045	-0.11496	1.680637	None
0.419611321	0.934743268	0.919033438	0.096219	-0.02466	0.098539	CLB4
0.420166402	0.408586762	0.846934431	-0.07472	-0.10192	-0.10646	RLM1
0.420166402	0.770350536	1	-0.08781	-0.05176	0.044373	MSM1
0.421161976	0.69919914	0.847404354	-0.07098	0.052779	0.100825	DHH1
0.421375059	0.246548074	0.082586739	-0.5406	-1.0132	-4.75195	None
0.42142764	0.740479146	0.723935587	-0.07071	-0.0469	-0.14244	TFC7
0.421733381	0.502826515	0.3133427	0.263114	-0.30554	-1.04857	None
0.421896486	0.32171509	0.2597044	0.078626	-0.12592	-0.32978	INO2
0.421896486	0.911433046	0.983364433	0.083564	-0.02575	0.052432	HSM3
0.422183363	0.103550972	0.488102089	0.068365	0.162181	0.211554	MRP13
0.423691896	0.624111878	0.972387712	0.090146	-0.08169	-0.06338	SEN34
0.423991949	0.980772539	0.69579659	0.100809	0.015461	-0.22252	YDR444W
0.424084529	0.148133144	0.47679402	0.079054	-0.17167	-0.2493	SWP82
0.424084529	0.110984851	0.659101564	-0.82981	-2.12163	-2.18878	None
0.424588452	0.398327474	0.527992521	0.632559	-0.91469	1.962694	YFL015C
0.424678133	1	0.670493385	0.107102	-0.00539	0.248892	ADY3
0.426588858	0.499790811	1	-0.09803	0.113748	-0.01206	ACS1
0.427061225	0.289514278	0.962371769	-0.18226	0.309177	0.141899	SPO1
0.427131927	0.631878695	1	-0.11624	0.103586	0.04682	AIM24
0.427397816	0.62038519	0.844058435	-1.09104	-1.00902	-1.08052	None
0.427719554	0.318028924	0.721655021	0.065121	-0.10601	-0.13387	CST26
0.428499065	0.996805372	0.333300106	0.062278	-0.00675	-0.23778	TFA2
0.42862699	0.905968424	0.987206488	0.664462	0.213766	-0.38912	None
0.429465176	0.058385914	0.290004916	0.055173	-0.15103	0.22444	UTP7
0.429564085	0.051484274	0.320515456	0.097137	-0.27355	-0.38045	YMR147W
0.429645418	0.010710362	0.931005601	-0.06072	-0.21581	-0.05735	ASH1
0.429716164	0.292882664	0.326232595	-0.08998	0.153052	-0.34732	LAS17
0.430758559	0.391096287	0.907281575	0.082752	-0.11893	0.092458	STH1
0.430870035	0.021955716	0.871950707	0.078408	-0.25463	0.10328	MNL2
0.430876441	1	0.449106932	0.092855	-0.00442	-0.30665	TAM41
0.431366783	0.2517386	0.906628341	-0.08779	0.160203	-0.09835	CYC3
0.431366783	0.565156176	0.971609311	0.105483	-0.11019	-0.07725	MLH1
0.431520716	0.367820737	0.575130318	-0.06372	0.095803	0.176017	TIM23
0.431979	0.867220499	1	-0.05624	-0.02325	-0.01657	LAG1
0.432245772	0.033587112	0.704128889	0.066348	-0.20187	-0.14516	ADE1
0.432793689	8.36301E-07	0.419798397	0.0501	0.29793	0.172602	FRE1
0.433251798	0.328303501	0.234642247	0.085699	0.138429	-0.38148	PTC1
0.433251798	0.995958449	0.845203377	-0.10394	0.011813	-0.15331	DSE3
0.433251798	0.021955716	0.90453709	-0.08677	0.280959	-0.09922	IGO1
0.433258424	0.915512205	0.931568877	-0.09625	0.029169	-0.09041	ERV2
0.43342275	0.929429208	0.621769539	0.133513	0.036784	-0.34376	YHR173C
0.43421858	0.332342438	0.278641706	-0.05753	0.092453	0.241256	ERV25
0.434862435	0.963282012	0.649011773	-0.08822	-0.01744	0.217937	ERD1
0.435003996	0.819842906	0.98500242	-0.19888	0.101716	-0.1253	YMR158C-[illegible]
0.43517854	0.860318409	0.854563991	-0.10481	-0.04501	0.149855	AVT2
0.436053855	0.731353745	0.904680493	-0.06508	0.04564	-0.07461	BSD2
0.436079267	0.567056169	0.702041373	0.053179	0.055766	-0.11787	VMA5
0.436130112	0.72983614	0.89719832	-0.07555	0.053313	0.090454	FPR2
0.436144377	0.022141152	0.930835922	0.723194	2.55392	0.969697	None
0.436221143	1	0.443825882	0.089686	-0.00602	-0.30168	YEL1

0.436409805	0.091723089	0.744376434	-0.10565	0.265613	0.207376	IGO2
0.437412793	0.249953428	1	-0.06763	-0.12577	-0.01799	CCA1
0.437577112	0.447427868	1	0.091396	-0.12033	-0.0372	THP3
0.438751901	0.398327474	0.929303164	-0.11991	-0.1728	0.117792	FCY22
0.439290495	0.265035517	0.142407082	0.076705	0.139777	-0.39708	VPS28
0.439329367	0.47767593	0.744376434	0.205268	-0.25767	-0.40552	PTH1
0.440288099	0.293118671	0.87592683	-0.0705	0.122517	0.092861	ATP11
0.440420243	0.379381608	0.048861894	-0.06525	0.097766	0.420209	RPL35B
0.440420243	0.66730791	0.397744731	0.052101	-0.04368	0.188199	PMT2
0.441964657	0.197970471	0.996250082	0.098449	0.200694	0.055177	REC107
0.442041336	0.099512037	0.924605282	0.079945	-0.20035	0.082581	SFH1
0.442771213	0.501380766	0.28514767	-0.16823	0.200284	0.707138	None
0.443142558	0.011022568	0.558164588	-0.10291	0.371808	0.298783	COX23
0.443202624	0.479322493	0.971609311	0.063112	-0.07915	0.046802	PEP1
0.444327428	0.990169077	1	0.063959	0.008343	0.012233	YGR210C
0.444607845	0.999736798	0.863302781	-0.07275	0.007427	-0.10218	DAM1
0.444839476	0.371969831	0.404093924	-0.44006	0.667856	1.583448	None
0.445299476	0.310898763	0.562048326	-0.11259	0.190815	0.325848	YIL102C-[illegible]
0.445643535	0.666045601	0.385856136	-0.07904	-0.06727	-0.29208	YLR326W
0.445643535	0.443822075	0.466577874	0.100443	0.134828	-0.33458	SNA4
0.445793341	0.979904046	0.835544417	-0.30391	0.049338	-0.47686	None
0.446698906	0.729387705	0.934548631	0.068239	0.049416	0.065049	GIM3
0.446982506	0.169156472	0.89826709	-0.05538	-0.12015	0.067281	SUN4
0.446982506	0.335596279	0.92566928	-0.09905	0.161699	0.102157	WIP1
0.447810471	0.062448847	0.971609311	-0.06601	-0.18466	0.050147	NMD5
0.450593399	0.858780357	0.645770166	-0.0555	0.024806	0.142208	FSH1
0.451974436	0.466698817	0.7225564	-0.07473	0.097368	-0.16016	CDH1
0.453158669	0.922260875	0.946857728	0.510246	-0.1559	0.459422	YMR030W-[illegible]
0.454297571	0.30849299	0.244196805	0.204062	0.35347	0.931384	YAL037C-[illegible]
0.454297571	0.167081065	0.710425231	-0.10586	0.232666	0.238331	QDR1
0.454327978	1	0.614405454	0.336557	-0.01776	-0.92199	YLR111W
0.455436806	0.084678945	0.992792463	-0.09905	-0.2643	0.059355	SYT1
0.456035036	0.885181241	0.75616091	0.287308	0.113581	-0.56749	YHL018W
0.456079281	0.389906374	0.860528802	0.061334	-0.09279	-0.08923	MSN5
0.456393088	0.461982321	0.863302781	0.065839	-0.0875	0.094792	HER1
0.456393088	0.242062719	0.984769539	-0.18031	-0.3537	-0.12019	None
0.456709929	0.836107259	0.471735065	0.061643	-0.03081	0.208693	TAF9
0.456864854	1	0.976378097	0.427924	0.042944	0.30966	None
0.457298356	0.767459399	0.181792015	0.587464	-0.38355	3.235831	None
0.45812894	0.936093775	0.404219832	-0.0529	-0.01438	-0.19524	LAC1
0.460921436	1	0.076212737	-0.05058	-0.00264	0.310207	KRR1
0.461833112	0.164247051	0.87973401	-0.08851	-0.19949	-0.11905	PDS5
0.462087533	0.139798632	0.523868802	0.080683	0.190823	-0.2553	IPT1
0.462087533	0.2946997	1	0.075854	-0.13764	0.028219	ELP6
0.462104267	0.838256028	0.782726705	-0.09834	0.049119	-0.18422	BIT2
0.462203351	0.00412351	0.015440085	0.056246	-0.23316	-0.44325	SER33
0.462283844	0.427585494	0.924605282	-0.08874	0.126485	-0.09483	ECO1
0.464872819	0.157263376	0.845425423	-0.06334	-0.14552	0.099293	POP1
0.465083745	0.177946472	1	-0.46693	1.013728	-0.00303	YDL114W
0.465274903	0.408586762	0.828700369	-0.12005	0.177941	0.199112	ZIP2
0.46571338	0.94100612	0.260184264	0.162904	0.042444	-0.73807	CIS1
0.465875951	0.788420725	0.203190491	-0.0556	0.033667	-0.27524	YME2
0.46614682	0.295659	0.656171223	0.064495	0.117123	-0.1671	ARG7
0.466719439	0.962506362	1	0.116355	0.024728	0.001743	YPR003C

0.46722467	0.690730215	0.240728867	-0.66536	-0.55375	-4.13256	PAU18
0.467282523	1	0.656171223	-0.05437	-0.00166	-0.14099	PLB1
0.467471293	0.916105608	0.952970302	-0.05138	0.016554	0.045932	ARO4
0.468011402	0.43160484	0.879698394	0.073425	-0.10555	0.100405	NUP145
0.469032978	0.36814865	0.102137973	-0.12453	0.200568	-0.72983	YPL109C
0.469314761	1.39043E-05	0.8119342	0.068916	0.396688	0.120648	FMP10
0.469945836	0.514580981	1	0.051489	-0.06342	0.018425	SEC23
0.470564194	0.686392021	0.264879577	-0.05216	0.044083	0.238384	SFM1
0.470600728	0.304258036	0.889649609	0.088323	0.1592	0.115893	ADH7
0.471026913	0.384917942	0.920656396	-0.06816	-0.1071	0.075465	RXT3
0.471110117	0.313366226	0.733389979	0.112571	0.200009	-0.24456	ASR1
0.471194063	0.979425666	0.780254446	0.083483	-0.01443	-0.16127	YPR084W
0.471771234	0.78787983	0.878688605	0.064775	0.039916	-0.08949	CUE3
0.47186612	0.749821409	0.774060671	-0.06121	0.043569	0.119907	DJP1
0.472099422	0.269605331	0.056523979	0.053072	0.102047	0.352923	RPS7B
0.472852539	0.341983981	1	-0.06764	-0.1153	-0.03809	TRE2
0.473053352	0.680755208	0.826140021	0.071397	-0.06181	0.121362	YPL039W
0.473146488	0.13817594	0.861402377	-0.07038	-0.17151	-0.10563	STD1
0.473261218	0.989216982	1	0.149685	0.021173	-0.07123	RRT1
0.47343817	0.47499087	0.600528962	0.051806	0.069339	0.149287	SEC61
0.47343817	0.167141351	0.994442854	0.060882	-0.13987	0.037289	YLR287C
0.474051984	0.83092898	0.630792731	0.092795	0.049139	-0.25441	RTT105
0.474339931	0.157263376	0.440092898	0.141104	0.329842	-0.51271	SPR1
0.474339931	0.023260849	0.915496392	0.069116	-0.24232	-0.08058	HRD3
0.474541677	0.324783906	1	-0.06644	-0.11705	-0.02647	PMT6
0.475293561	0.425809904	0.574248891	0.181204	-0.26908	-0.54745	YOR302W
0.475336639	0.709900242	0.937344825	0.068812	-0.0556	0.068381	EMC2
0.475997678	1	0.708493888	-0.15766	0.004338	-0.37469	DTR1
0.475997678	0.169835769	1	0.126028	0.288503	-0.01557	DLS1
0.476044207	0.035103743	0.004415892	-0.05288	0.173173	-0.47976	PYC1
0.476542875	0.08272612	0.328463464	0.079507	0.221852	0.336815	AIM19
0.476542875	0.069544572	0.855814602	-0.08624	0.248638	-0.13266	YLR152C
0.476731956	0.019248218	1	0.186324	-0.68085	0.039424	BFA1
0.476897326	0.04054043	1	-0.0649	-0.20876	0.01538	TRM44
0.477294277	0.157263376	1	-0.08611	0.202375	-0.00815	VPS25
0.478167064	0.984718514	0.50705979	-0.98779	0.152139	3.066841	None
0.479637407	0.526283651	0.750952753	-0.427	0.517241	-0.89587	None
0.479721748	0.170043734	0.91703897	0.057722	-0.13353	0.067233	CSE1
0.481633787	0.884827129	0.309689972	0.072967	0.030539	0.321241	UBC9
0.485085949	0.149436038	1	-0.06535	-0.15911	-0.02854	UME6
0.485243358	0.379077824	1	0.080192	0.131444	-0.03386	ARP1
0.485368901	0.128036313	0.853162234	0.084924	-0.21685	0.134738	NDC80
0.485368901	0.103376814	0.906628341	-0.05588	-0.15038	0.069618	GLO3
0.486308746	0.221596672	0.169011865	-0.0529	0.112738	0.288603	GSP1
0.487418991	0.844258179	0.721271027	0.714501	0.362957	-1.67703	None
0.487497358	0.533524502	0.66765157	0.073873	0.090629	-0.19543	YPR109W
0.487497358	0.774294935	0.929303164	0.373639	-0.25188	-0.40692	None
0.488889101	0.702542191	1	-0.05445	-0.04593	-0.02778	MGR1
0.489047626	0.052189123	0.664126703	-0.06593	-0.20724	-0.17612	GCN2
0.489718517	0.228877023	0.0166762	-0.05311	-0.11299	0.440807	SYO1
0.489718517	0.613821587	0.733341705	0.051818	-0.05472	0.117361	XRN1
0.490054863	0.729751469	0.05801516	0.063661	-0.05012	-0.43492	ISN1
0.491307807	0.231636316	1	0.131483	0.278516	0.030941	GAL2
0.491899366	0.6101658	0.837446458	-0.05907	-0.0633	-0.10036	PET54

0.491994764	0.956605945	0.855814602	-0.30744	0.071909	0.487953	YML007C-A
0.492233828	0.408627815	0.509261048	-0.82411	1.268771	2.602117	None
0.492233828	0.90728766	0.93147427	0.768235	-0.28052	0.798472	None
0.492292545	0.40987672	0.512491836	-0.82784	1.272281	2.596834	None
0.495744043	0.589846261	0.344884088	-0.08808	0.098689	0.378156	PHM8
0.496029636	0.904226858	0.980342156	0.087038	-0.0326	-0.06452	PRK1
0.496340018	0.380470847	0.241434728	-0.04623	0.077138	0.231045	RPL13A
0.497229314	0.576193096	1	0.058151	-0.06743	-0.02069	VID27
0.497509133	0.914927286	0.702041373	0.758005	-0.26458	-2.01249	None
0.497662096	0.740319156	0.650488242	0.063135	0.048651	-0.17555	SIT4
0.497912483	0.333401536	0.789728487	-0.07282	0.132071	-0.14348	ECM38
0.49792193	0.98408428	0.781709456	-0.08002	-0.01352	-0.16165	OCA4
0.498385565	0.639003464	0.661089241	-0.67111	0.661135	1.838776	YLR302C
0.49924073	0.119202402	0.28605422	-0.05561	0.148277	0.261058	HMF1
0.499530263	0.071099424	1	0.120944	0.363923	-0.06984	None
0.499575292	0.992842219	0.717477893	-0.07278	0.010157	-0.17471	CHM7
0.499747239	0.413532829	0.509146716	-0.0589	0.09267	-0.20495	ZWF1
0.499821244	0.490263266	1	0.133711	0.182791	-0.08088	YNR071C
0.500198864	0.030733392	0.05751254	-0.0475	-0.16738	0.333175	JIP5
0.501592254	0.140485465	0.976568193	0.066752	-0.17122	0.052287	RPB2
0.502204793	0.016934011	0.936440675	0.064428	0.247962	0.067786	UGA2
0.502248025	0.068716276	0.967394603	0.047415	-0.14505	0.041226	LEO1
0.502634231	0.34806237	1	0.294357	0.525107	0.012786	SOR1
0.502647828	0.896286063	0.143859218	-0.0819	0.032601	-0.47858	SGE1
0.502683774	0.276012403	0.599344132	-0.06746	0.136449	-0.20609	XDJ1
0.502987279	0.98685381	0.978193638	0.056945	-0.00904	-0.04395	MPD2
0.502992517	0.029454084	0.058656331	-0.06177	-0.22093	0.433351	SGD1
0.502992517	0.515859901	0.480256366	-0.06273	0.082158	-0.22944	IZH3
0.502992517	0.924812503	0.828700369	-0.06686	-0.02191	-0.1199	SWC3
0.503033321	0.997499519	0.372814632	0.137515	-0.01684	0.582013	SNO1
0.503728257	0.986843508	0.76367822	-0.06503	0.010433	-0.1398	TMS1
0.504130959	0.708957109	0.726816534	-0.15724	-0.13491	-0.36984	KRE29
0.504130959	0.91970774	1	0.065073	-0.02219	-0.01047	SKY1
0.504536652	0.692438881	0.834791774	-0.10066	0.0896	0.177963	MTG2
0.50668448	0.21659995	0.935686072	0.063596	-0.1431	-0.06802	COG3
0.507149555	0.037277785	0.873351716	0.430608	1.503453	-0.66383	YLR307C-A
0.507778018	0.068453606	0.034137138	-0.0616	0.189884	0.481129	RPS14A
0.508773168	0.086697129	0.360662746	-0.04703	-0.13872	0.203848	PSE1
0.509128945	0.897122859	1	0.04408	0.017722	-0.01792	LPD1
0.509315747	0.619450596	0.020112784	-0.40491	-0.44243	3.642459	None
0.510653352	0.99176478	0.688444366	-0.0632	0.009134	-0.1682	AIR1
0.511144976	0.593217132	0.642194109	0.096224	0.110802	-0.27918	ATG22
0.511729057	0.244947265	0.8119342	0.087968	0.190457	0.16796	PCC1
0.512392925	0.659855933	1	0.488995	-0.49624	-0.03803	None
0.512700749	0.173085722	0.04678456	-0.05662	0.138534	0.42403	RPS17A
0.513472573	0.078816177	0.919926509	-0.06459	-0.19715	-0.0786	SLD3
0.513759795	0.659755235	0.578113103	0.063832	-0.06356	-0.2065	YJR015W
0.513983749	0.498387456	0.981444893	0.05764	-0.08008	0.043942	UBX4
0.514316181	0.037225892	0.384202197	0.061121	0.214418	0.260327	MHR1
0.514316181	0.801762915	0.759037071	0.094278	-0.06039	-0.20895	APC5
0.514316181	0.413008972	1	0.068583	-0.11206	-0.03157	PSP2
0.51455276	0.368093975	0.914525622	0.086812	-0.15461	-0.11037	POL2
0.514757974	0.735101186	1	0.453755	-0.3755	0.042549	YLR415C
0.516365716	0.801762915	0.229173368	0.066995	-0.04305	-0.35439	ARP6

0.516396896	0.056645029	0.005737743	0.07773	0.253588	0.755192	HUB1
0.516396896	0.026675581	0.611594186	-0.05353	0.198902	0.165398	YDC1
0.518455414	0.173735431	0.875200775	-0.05606	-0.13916	-0.08677	TRE1
0.518583784	0.373168526	1	-0.06107	-0.10835	0.004002	FAT1
0.519707704	0.752103902	0.932413507	-0.07946	0.061947	0.088261	APS2
0.520243555	0.244947265	0.04122541	-0.04952	0.109001	0.386928	RPS18A
0.520560166	0.291582782	0.251754292	0.054597	-0.11203	0.282999	YBT1
0.520666803	0.502826515	0.967394603	0.095406	-0.13329	0.086615	SCC2
0.520912403	0.294101962	1	-0.0827	0.168377	-0.0332	IFM1
0.521779325	0.159515372	0.939670905	-0.05974	-0.15347	-0.06394	OTU2
0.522657547	0.086191139	0.589775129	-0.06583	-0.20038	-0.21311	SNF12
0.522845521	0.177112688	0.049980756	0.068023	0.168951	0.51406	YLR264C-A
0.523526599	0.970013423	1	-0.08828	0.019265	-0.03554	DDC1
0.524229461	0.301940839	0.974194652	0.071055	0.143561	0.060153	YFH1
0.524333434	0.782040669	0.581268517	-0.06295	0.04387	-0.20688	RKM3
0.524421535	0.770350536	0.8103699	-0.09851	0.0713	-0.19364	HIT1
0.524421535	0.302395299	0.891706446	0.072629	-0.14714	-0.10552	YOR296W
0.524421535	0.686382379	0.894445004	0.061157	0.05786	0.087975	MTM1
0.524714069	0.032364503	0.488692236	-0.05908	-0.21754	-0.22357	CLB1
0.525601275	0.532413738	0.430673089	-0.04288	-0.05712	0.175799	YKL077W
0.527038068	0.026560847	0.919516515	-0.12517	0.473546	-0.15704	YNR068C
0.527038068	0.244648183	1	0.382046	-0.89753	-0.23796	CDA2
0.527174331	0.034211322	0.871066692	-0.07488	-0.2745	0.120085	MSB1
0.527375747	0.439543123	0.616944383	-0.06326	0.100922	-0.19787	PXP1
0.527395953	0.672577856	0.427445927	0.066638	0.065587	-0.2745	PXP2
0.527471593	0.076685215	0.163693857	-0.06576	-0.20813	-0.39581	ZAP1
0.527471593	0.950105874	1	-0.06295	-0.01721	0.015419	ATM1
0.527707188	0.411661642	0.326601143	-0.04429	-0.07456	0.209159	NSA1
0.527998669	0.666318943	0.550542595	0.358464	0.359983	-1.26754	YGR045C
0.528193277	0.014044723	1	0.057837	-0.24189	-0.00275	RIA1
0.528321512	0.347703031	0.189220839	0.428972	-0.84115	2.719505	None
0.528411916	0.953371228	0.291391691	0.043469	-0.01157	0.215469	RPA34
0.52925952	0.649028901	0.901225243	-0.47496	0.492613	0.662839	YGR204C-A
0.529482281	0.690335313	0.134903876	0.107317	-0.10213	-0.67528	AZR1
0.530536581	0.836107259	0.929344228	0.082628	0.048092	-0.09719	MDM12
0.530707539	0.305821205	0.104201741	-0.05024	0.102089	0.335844	RPL17B
0.530707539	1	0.522788552	0.107193	-0.01133	-0.39231	GRX6
0.530707539	0.664002974	1	-0.20596	0.208282	0.047582	THI11
0.530930288	0.905759808	0.786584362	0.070037	0.027768	0.149955	UGO1
0.530957435	0.761254812	0.533231335	-0.07754	0.059379	-0.27909	MNE1
0.531338476	0.953675948	0.001009254	0.493774	-0.13381	-8.18439	None
0.531338476	0.352451342	0.795907717	-0.10928	0.205818	0.227646	TPP1
0.531338476	0.203043786	0.7990478	0.054644	-0.13275	-0.11308	PRP19
0.532916128	0.011080935	0.243549962	0.053043	-0.23168	0.286017	PUF6
0.533240644	1	0.670294084	0.063669	-0.00035	-0.184	DCK1
0.533575185	0.066848063	0.287597168	0.044061	-0.14498	0.22223	DBP3
0.534464761	3.50087E-05	0.060744575	-0.07201	0.456519	0.535709	CMC2
0.534464761	0.699522343	0.531076067	-0.06641	-0.06221	0.242537	RKM2
0.534464761	0.938960046	0.595547517	0.063702	-0.01974	-0.21006	YER034W
0.534464761	0.488044682	0.737914729	-0.87988	1.275108	1.474398	None
0.534464761	0.033587112	0.960632458	-0.0599	-0.22428	-0.05936	FKH2
0.534519899	0.206109048	0.960632458	0.079139	-0.193	0.078684	JJJ2
0.53461092	0.914594311	0.853662564	0.049422	0.018617	-0.0866	VPS17
0.534681612	0.913339726	0.553808105	0.062194	0.023786	-0.21908	PHO23

0.534681612	0.947996628	0.721271027	0.046803	-0.01332	0.120151	YBL036C
0.535474954	0.012929362	0.324676992	-0.04472	-0.19186	0.215997	PRP43
0.536166342	0.64001431	0.763322262	0.903065	0.986962	-1.42851	None
0.536258728	0.72522855	1	-0.04659	-0.04093	0.015725	NDC1
0.536270845	0.001731932	0.889649609	0.054687	0.281855	0.082848	RFS1
0.536893486	0.767134842	1	0.055084	-0.04202	-0.0111	JNM1
0.537486022	0.292062644	1	0.0478	-0.10199	-0.00585	STP1
0.537947655	0.393729423	0.390918	-0.04739	-0.0844	0.210651	LSG1
0.537947655	0.62447311	1	-0.06239	-0.07117	0.030856	TRA1
0.538245334	0.120557166	1	0.0871	0.252223	-0.03779	MRX10
0.538655118	0.998764461	0.795907717	-0.06731	0.008605	-0.14257	OCT1
0.538831903	0.368966584	0.96218812	-0.07592	-0.14211	0.074795	CCH1
0.539284195	0.157263376	0.392456706	0.074645	0.200142	-0.33068	NAS2
0.539480082	0.209645547	0.46824606	-0.07863	-0.19225	-0.31694	MGS1
0.539709969	0.644005887	0.169547502	0.120354	0.131657	-0.73174	GAL1
0.540817618	0.697051685	0.516783348	0.051667	0.049399	-0.19529	NBP2
0.541865468	0.509639659	0.826681282	-0.16773	0.241122	0.327661	None
0.542299611	0.338644692	0.927131674	-0.04203	-0.08342	0.052367	GAS3
0.542656145	0.210530389	0.804576171	-0.16941	0.412105	0.350723	None
0.543010303	0.432562651	1	-0.06424	-0.10789	0.026216	PRI2
0.544414711	0.413242794	0.967394603	-0.0769	-0.13403	0.073827	CTR86
0.544972843	0.516700017	0.915496392	0.047016	-0.06752	0.063785	APL6
0.546328707	0.649028901	0.244196805	0.883782	0.966338	5.049419	None
0.546328707	0.649028901	0.244196805	0.883793	0.96635	5.049409	YHL048C-/
0.547617971	0.492472465	0.283481217	-0.85642	1.267248	-5.06077	LSM5
0.547617971	0.995362426	0.314397572	0.226519	-0.03342	1.141258	None
0.547617971	0.123945381	0.971609311	-0.20543	0.596651	-0.18899	YER145C-/
0.547714567	0.914594311	0.874977083	-0.20917	-0.08085	-0.35033	SBH1
0.547949735	0.879819172	0.417425061	0.07403	-0.03685	0.32456	FAU1
0.547949735	1	0.449106932	0.066799	-0.00134	-0.28066	FIN1
0.548934097	0.551291677	0.260671162	0.67862	-0.96929	-4.62219	None
0.549085447	0.840784015	0.98198617	-0.437	0.255565	-0.36486	YKL096C-I
0.54932565	0.821809527	0.545272974	0.04697	0.030257	0.173478	VMA7
0.549860136	0.730766871	0.888371569	0.076045	0.067708	-0.1201	NTG2
0.549926445	0.495058536	0.283481217	-0.85011	1.260824	-5.05603	YGL235W
0.550075979	0.76984172	0.960632458	0.453638	0.3507	-0.45962	None
0.550341201	0.10529773	0.793556588	0.169731	0.520834	0.371248	YCR001W
0.550531973	0.006875897	0.376271085	0.047107	0.223783	0.21974	CDC42
0.551333047	0.077765835	0.322465729	-0.05331	0.176739	0.267534	HYR1
0.551333047	0.980604983	0.571709772	-0.23513	0.047087	-0.83793	YMR182W-/
0.551662896	0.292344093	0.53398283	0.076866	-0.16933	0.290812	MLP1
0.551989592	0.986843508	0.474290885	-0.08264	-0.01467	0.340834	GEP4
0.552339526	0.461111981	0.620580042	-0.17167	0.277807	0.564978	None
0.553467571	0.497819776	0.283510967	-0.84556	1.258023	-5.05581	None
0.5536759	0.120709475	0.818523526	-0.22883	0.678553	-0.46708	YER039C-/
0.55375023	0.767459399	0.82890057	-0.65251	0.500616	1.270882	None
0.554602969	0.147942384	0.67702211	0.054166	-0.15402	0.162732	POL32
0.556017272	1	0.643429399	0.540394	-0.02295	-1.80008	None
0.556154439	0.00286728	0.053817845	0.124074	0.64186	-0.99235	YMR315W-/
0.55617172	0.129797843	0.961702496	0.072918	0.214987	-0.0749	YSP3
0.556341515	0.317595659	1	0.058109	-0.12359	0.009645	YPK9
0.556416854	0.019388398	0.409896537	0.091599	-0.39325	0.414947	YSP2
0.557411158	0.958689082	1	0.053499	-0.01425	0.016357	YRB30
0.557716924	0.351406863	0.077555297	-0.05179	0.103823	0.388635	RPL16B

0.557726174	0.000922362	0.89915479	0.093692	0.527855	-0.14202	YCR007C
0.558003108	0.134186983	1	-0.05563	0.163206	0.035729	FSH2
0.558040147	0.72990982	1	0.380599	0.346861	0.199594	None
0.559434356	0.795533688	0.715024856	0.10167	0.073477	-0.28063	RIF2
0.559789109	0.095354954	0.930835922	0.22946	0.739051	-0.28355	None
0.560377698	0.795674553	0.76367822	-0.10137	0.073135	-0.24712	YOR012W
0.560470993	0.996753756	0.803017727	-0.35514	0.051813	-0.78673	UTR5
0.560681063	0.527104557	1	-0.05884	0.085673	-0.03545	RGT1
0.561486378	0.117458174	0.002316113	-0.05451	0.167535	0.628261	RPS21B
0.561769545	0.158486798	1	0.054042	-0.15258	-0.00441	RSC3
0.561803908	0.650079178	0.835951015	-0.03963	0.044857	-0.07897	FUM1
0.561841785	0.582123297	1	-0.23218	-0.30758	0.066058	PHM6
0.562636301	0.57019157	0.354608469	-0.09639	-0.13069	0.475822	YNR062C
0.562636301	0.681412538	1	-0.12634	-0.13203	-0.00073	YDL109C
0.563136508	0.302233138	0.906628341	0.073395	-0.16293	-0.10881	PAC2
0.563420111	0.072550807	0.019841876	0.039338	-0.13657	0.375647	NOP12
0.563420111	0.083210455	0.778620065	-0.06019	-0.20282	-0.14212	SPT23
0.563420111	0.591976174	1	0.068189	-0.08882	-0.04449	RTT101
0.564009704	0.109990533	0.467794221	0.682283	2.286737	3.244976	SOR2
0.564056024	0.690335313	0.262959237	0.041091	-0.04217	-0.23054	CPR4
0.56547394	0.341978326	0.488692236	-0.05449	0.113154	0.22662	CPR8
0.565629959	0.753846794	0.606574034	-0.72552	-0.62414	-2.65226	None
0.566374356	0.642965493	0.962371769	-0.06484	-0.07554	-0.06762	CHK1
0.566882449	0.75339767	0.847404354	-0.29597	-0.25641	-0.58611	YFR054C
0.567720361	0.84041532	0.985483713	0.088528	-0.05515	0.071657	ZIP1
0.571231463	0.899105948	0.908303081	0.062932	-0.02868	-0.09337	DEP1
0.571621418	0.937034346	0.910199402	-0.35425	-0.12099	-0.5184	YKR041W
0.571675279	0.996223442	1	0.079945	-0.01201	0.008977	UBP7
0.571736929	2.46025E-07	0.392569813	-0.04145	0.342508	-0.19736	GSY2
0.572296378	0.248973593	0.417425061	0.36162	-0.93558	1.811508	YFL012W
0.57314424	0.887970391	1	0.128681	-0.06372	-0.08264	PET122
0.573493176	0.027221239	1	-0.09924	-0.42117	-0.02152	KCC4
0.573678225	0.727504645	0.469733538	0.06375	-0.0607	0.277865	ISC10
0.573878524	0.012650856	0.420929331	0.063716	-0.30017	-0.29459	INM1
0.574155754	1	0.768164938	0.084991	0.000251	-0.21116	YEF1
0.574239333	0.776534414	0.721655021	-0.57991	-0.46345	1.683126	YBL008W-A
0.575798985	0.430199039	0.995979951	0.052103	0.094507	-0.03917	GUP1
0.576109932	0.886858489	0.865649648	-0.05228	-0.02626	0.096177	BCH1
0.576460228	0.569517503	0.0204694	-0.06315	-0.08838	-0.61176	MET16
0.577089404	0.271203734	1	-0.07826	0.187834	0.033297	YFT2
0.577906824	0.759288226	0.927963214	0.07522	-0.06523	-0.10025	LIN1
0.577906824	0.147268515	1	0.056462	-0.16958	0.003113	PEF1
0.577960652	0.6500985	0.795907717	0.118784	-0.14	-0.27475	APC1
0.577960652	0.458954168	0.885657987	-0.07153	-0.12403	0.121879	SLS1
0.577960652	0.418014035	1	0.454995	-0.88691	-0.02135	None
0.578248926	0.066144657	1	-0.0491	-0.1794	0.00734	PDC2
0.578508786	0.963282012	0.756233899	-0.04483	-0.01204	0.115755	GYP6
0.579385264	0.139339573	0.4591411	-0.3406	1.033021	1.563476	None
0.579934419	0.601019912	0.709422392	-0.10473	-0.13883	-0.30983	AMF1
0.579934419	1	1	-0.07634	-0.00014	0.001599	DIA2
0.580022237	0.342998394	0.327518381	-0.28279	-0.61973	-1.66977	None
0.580511194	0.102364988	0.859694751	-0.03621	0.120169	0.069256	SSC1
0.580511194	0.177202487	0.986500461	-0.07017	-0.1996	0.057992	SMC6
0.580599599	0.562441257	0.736378884	0.05407	0.077735	0.148048	NSE3

0.581230398	0.803401992	1	0.064906	0.047985	0.006141	BRR1
0.581506743	0.247677793	0.706865475	-0.04121	-0.10432	-0.12316	CAB1
0.581752889	0.105961984	1	-0.05167	-0.17119	-0.02638	ANY1
0.582846811	0.174275989	0.62830703	0.176525	0.505344	0.61309	None
0.582874661	0.334055071	0.374918009	0.577659	-1.37441	3.233588	None
0.583976802	0.839064323	0.971609311	-0.05852	0.037948	-0.05826	RTR1
0.584030791	0.916930597	1	-0.06084	-0.02518	-0.0104	UIP5
0.584324089	0.261323518	0.721655021	0.06642	-0.16632	0.190762	SWI4
0.585232386	0.576536639	1	0.058677	0.08265	0.032677	MED4
0.58645242	0.582169769	0.38020077	-0.06653	-0.09297	0.334935	PRP6
0.586588517	0.923003688	0.563376575	0.109174	-0.04383	-0.42622	IPL1
0.589712541	0.080514417	1	0.085812	-0.31014	0.018366	CIN8
0.590175767	0.672129195	0.715052519	-0.48928	0.547816	-1.47205	YHL041W
0.59039372	0.308657113	0.05005618	-0.05304	0.123062	0.466358	RPP2A
0.590441377	0.801071005	0.498286841	-0.05444	-0.04148	0.236481	AMD1
0.590594263	0.885748557	0.868031723	-0.47849	0.246495	-0.93094	None
0.590610744	0.374954065	1	0.249358	0.518303	0.169555	None
0.592039119	0.210094783	0.985468267	0.04962	-0.13668	0.042538	RXT2
0.593209897	0.864979821	1	0.038986	-0.02298	-0.00725	VPS4
0.593255291	0.981445908	0.711541955	-0.24297	0.052574	0.733635	YPL062W
0.594467072	1	0.908303081	-0.55167	0.003317	0.865215	None
0.594978226	0.267599743	0.780776603	-0.07072	-0.17889	0.17903	YPR097W
0.595860186	0.288638452	0.939670905	0.040001	-0.09803	0.05075	SRP1
0.595921787	1	0.538130573	0.342633	0.022509	-1.45958	None
0.597075672	0.060869744	0.940027863	-0.05191	-0.20112	-0.06595	MAK10
0.597895363	0.440961232	0.87712194	-0.05199	-0.09747	-0.09502	SPC34
0.598046417	0.102498598	0.00882836	0.044999	-0.15599	0.507417	RNT1
0.599589804	0.219208972	0.054532226	-0.0452	0.12424	0.400161	RPS16B
0.600621868	0.136055875	0.214654991	0.458373	-1.64912	-4.38778	YBR221W-/
0.600621868	0.630291514	0.290004916	-0.2682	-0.3503	-1.6664	YGR025W
0.600621868	0.887970391	0.392569813	-0.47875	0.249101	-2.6682	None
0.600621868	0.640183171	0.87973401	-0.03917	-0.04962	-0.07135	MCM1
0.60121504	0.333037056	0.027801813	-0.04305	0.098581	0.427719	RPL19A
0.60121504	0.600998428	0.255041627	0.045091	-0.06283	0.2799	SKI6
0.602072258	0.678241823	0.969393262	0.058015	0.066927	0.062192	YNL193W
0.602072258	0.542558974	1	-0.04465	-0.06968	-0.0208	CIA1
0.602206972	0.087715023	0.442948909	-0.06543	-0.23833	-0.31374	ABF1
0.602669182	0.089703965	0.132432156	0.059012	-0.21432	0.443287	URB1
0.603255153	1	1	0.039198	0.001263	0.002279	BMH2
0.604550161	0.266387962	0.174129588	0.068534	-0.17841	0.484191	YHL012W
0.604550161	0.216242824	0.449495496	-0.1905	0.528408	-0.91807	SMD1
0.604936031	0.681411432	0.484106841	0.68473	0.79145	-3.02237	None
0.60508287	0.672129195	0.031420916	-0.04362	0.051445	-0.4277	YDR061W
0.605614275	0.141633165	0.043873822	-0.0421	0.13577	0.394325	VMA11
0.605614275	0.113318165	1	0.088399	0.303696	0.046088	DAL3
0.605812555	0.418773131	0.186752472	0.543978	1.088264	-4.27239	YDR034C-/
0.606022823	0.836107259	0.415327459	-0.04675	0.032294	0.235137	LIP1
0.606022823	0.013981458	0.564685666	0.061029	-0.3061	0.249918	NUP133
0.606251566	0.899541977	0.659038749	0.766815	-0.38146	2.36718	None
0.606279745	0.013622527	0.780208648	0.055387	-0.27892	0.144641	ECM16
0.606317327	0.392406899	1	0.569568	1.200001	-0.2569	None
0.606814858	0.925411578	0.580281763	0.063738	0.026288	-0.25432	SCC4
0.606881211	0.178518827	0.516090954	0.390216	1.186999	1.786496	YJR079W
0.608773858	1	1	-0.07251	-0.00584	0.01394	PUS6

0.608862465	0.447812948	0.629134428	-0.57622	1.07888	-2.32254	None
0.609201776	0.713873054	0.050546852	-0.06752	-0.07252	-0.61323	MUP1
0.609513534	0.703379547	0.984628847	0.045684	-0.05049	-0.04223	TRS31
0.609687446	0.71920823	0.545272974	0.060905	-0.06456	-0.2589	ELA1
0.609736539	0.506234402	0.971609311	-0.08423	0.14312	-0.08871	DPB3
0.609935071	0.418773131	1	-0.2192	0.435856	-0.074	None
0.611275552	0.990425307	0.069148732	-0.04156	-0.00776	0.361648	RRS1
0.611904371	0.440961232	1	0.134926	0.26133	-0.02714	None
0.612281358	0.317595659	0.870558299	-0.04611	0.11142	0.090422	HNT1
0.612845486	0.905759808	0.343265986	0.75524	-0.36779	-4.53019	YFL068W
0.613224957	0.434712398	0.179756965	-0.03459	-0.06821	0.247419	RPC40
0.613224957	0.905759808	0.343092466	0.753475	-0.36594	-4.53493	None
0.613224957	0.905759808	0.343265986	0.753661	-0.36628	-4.52763	None
0.613224957	0.905759808	0.343265986	0.75365	-0.36625	-4.52759	None
0.613992445	0.012650856	0.563376575	0.051549	-0.26744	0.216105	POL12
0.615720635	0.515000941	0.068005273	-0.05668	-0.09653	-0.4989	GPC1
0.618802039	0.601388298	0.011257238	0.034921	-0.05072	0.40372	PWP1
0.618802039	0.895557583	0.094839053	0.049111	-0.02572	-0.40939	LAM5
0.618802039	6.96957E-05	0.166524458	-0.05808	0.434569	-0.42682	SKS1
0.619880672	0.996805372	0.943092469	-0.0566	-0.00929	0.074862	BUD16
0.621748226	0.025132758	0.254956962	0.237224	-1.17717	-1.63669	PHO92
0.6225016	0.984860432	0.688444366	0.061256	-0.01357	-0.21149	MLH3
0.622792818	0.044150726	0.52960144	-0.03702	-0.16254	-0.16611	AVT1
0.622813356	0.83092898	0.477655526	0.057524	0.042493	-0.27817	ASI1
0.622813356	1	0.661089241	-0.04557	0.005467	0.166102	VBA1
0.624562771	0.712206218	0.785906893	-0.04986	0.055827	-0.13319	HAT1
0.62528464	0.995096172	0.846934431	-0.04715	0.008446	-0.10464	CDC123
0.626062012	0.858991565	0.181954717	-0.04533	0.029698	0.332566	RSM23
0.626062012	0.472952463	0.483628979	-0.05656	0.106735	0.273959	PKR1
0.629659097	0.615223063	0.733341705	-0.05329	0.076733	0.165522	RFA3
0.630441947	0.717419826	0.744376434	-0.04164	-0.04641	0.125029	SRP21
0.630514216	0.207905497	0.522217116	0.044355	-0.13454	0.206051	MTR10
0.631111413	0.963551031	0.84419011	-0.20263	-0.06123	-0.46384	YLR236C
0.631295727	0.533524502	0.563376575	0.061071	-0.10412	-0.26546	SLX4
0.631295727	0.514937661	1	-0.04883	0.086041	0.026657	MST1
0.631439544	0.030006572	1	0.565663	-3.471	-0.61013	None
0.632080012	0.199387279	1	-0.73053	-2.3737	0.848841	None
0.632837052	0.299260429	0.006151828	0.068592	0.179533	-0.85998	ATH1
0.633560024	0.199496245	1	-0.72615	-2.3703	0.843439	None
0.634377185	0.53992737	0.92745323	-0.27703	-0.47335	-0.43433	YBR056W-A
0.634872148	0.304299791	0.636815206	0.051227	-0.13389	-0.2	YKL075C
0.635896584	0.703189392	1	0.573386	0.673475	-0.28618	YAL067W-A
0.636176874	0.037207475	0.90423793	-0.0469	-0.21916	0.084118	YEL043W
0.636397143	0.895141735	0.133400488	0.040082	0.02192	0.326138	RPS8A
0.636462725	0.042062145	0.228471317	0.052137	0.23755	0.366826	RPP0
0.636462725	0.317595659	1	0.045316	-0.11653	-0.01826	COG1
0.636474096	0.982105623	0.981786205	-0.05262	-0.01257	-0.05309	YND1
0.636474096	0.199728633	1	-0.71893	-2.36577	0.835232	YGR018C
0.636748222	0.759694936	0.272261717	-0.05943	-0.05903	-0.39126	NPR2
0.637053807	0.152890044	0.440092898	0.081624	0.278108	-0.42757	GUD1
0.637164266	0.782318684	0.441720189	0.039262	0.035645	0.206698	TNA1
0.637164266	0.883064096	0.906020326	0.034132	0.020589	-0.06043	ARO8
0.638389463	0.031788614	1	0.561223	-3.45247	-0.60643	None
0.638964313	0.892236467	0.198116982	0.042196	-0.02378	0.312773	RRP15

0.639181218	0.813293705	0.694076825	-0.08255	0.067757	-0.2931	YGR240C-A
0.639181218	0.703643853	1	-0.2482	0.292186	0.166149	None
0.639658527	0.846089519	0.941464384	0.041486	-0.02982	-0.05809	SLT2
0.640042343	0.361894148	0.608460014	-0.06775	0.162656	-0.28046	UBC5
0.640042343	0.762623465	0.781154036	-0.059	0.058136	-0.16607	TPT1
0.640503519	0.659755235	0.343265986	0.030929	-0.04142	0.186313	GDA1
0.641642152	0.443553864	0.038262982	-0.03948	0.081859	0.415703	RPL20A
0.641768476	0.684241481	0.830189162	0.058161	-0.07313	0.14341	DSS1
0.641887249	0.690335313	0.509146716	-0.0617	0.076223	-0.29876	YIL001W
0.642056276	0.24298702	1	0.038151	0.11253	0.029501	VPS24
0.642073918	0.006135462	0.451184565	-0.4227	2.781715	2.606405	None
0.642443253	0.04859996	0.421368193	-0.04035	-0.18336	0.220504	UTP13
0.642610648	0.665438196	0.244196805	-0.09541	0.125996	-0.6628	DUR3
0.642610648	1	0.480798039	0.052977	0.000221	0.269375	RTT102
0.643939627	0.853297263	0.238946444	0.115755	-0.08213	-0.81337	BIO3
0.645490471	0.72385964	1	-0.05751	-0.06584	-0.02986	RAD24
0.645885018	0.28690473	0.737748659	-0.03793	-0.10556	0.121326	SRP68
0.647111174	0.462711274	0.001629245	-0.04013	0.081585	0.58523	RPL25
0.647111174	0.996960041	0.699105653	0.041936	0.007324	0.150559	SDD2
0.648350878	0.459813614	0.074275376	-0.04253	0.087314	0.400962	RPS7A
0.648350878	0.703189392	0.818523526	0.508335	0.619026	-1.30086	None
0.649507877	0.000147156	0.061449807	-0.03575	-0.28082	-0.34799	MTD1
0.650096201	0.261791204	0.879698394	0.113776	0.33281	0.234265	ICS3
0.650096201	0.980772539	0.949025836	-0.18609	0.047062	-0.25976	GMC2
0.650346433	0.690335313	0.37055688	0.126171	-0.16071	0.75757	YBR196C-A
0.650573409	0.270323907	0.411752857	0.373222	-1.15091	2.385165	None
0.651145464	0.55907882	0.656737385	0.073578	-0.12675	-0.29016	TUS1
0.651445687	0.714551955	0.644371882	-0.05705	-0.06796	-0.22984	MMS22
0.651691784	0.891627765	0.484106841	0.042432	0.024822	-0.21896	NVJ1
0.653987549	0.454132404	0.065524422	-0.03388	0.07126	0.331553	RPS0A
0.653987549	0.660417863	0.963910538	-0.04562	0.062977	0.058268	ESS1
0.657006231	0.128547958	0.637213394	-0.17477	0.652666	-0.71555	YCR095W-A
0.657347347	0.666045601	0.711247098	0.049131	-0.06738	-0.17483	ECM30
0.657991891	0.04219607	0.186752472	0.038814	-0.18779	0.307034	RPA135
0.659274066	0.324764976	0.835951015	-0.2906	-0.805	0.778952	None
0.659278291	0.768238502	0.871066692	0.16763	-0.17087	0.370502	None
0.659291058	0.066170367	0.430383427	0.04826	-0.21568	-0.27258	WAR1
0.660843362	0.925860071	0.919926509	-0.4133	0.195079	0.712332	YGR273C
0.662344476	0.986070744	1	0.59214	-0.14033	-0.34179	None
0.663882981	0.667040818	0.847051661	-0.04954	0.06854	-0.12115	SSU1
0.664786199	0.298926458	0.024501628	-0.03923	0.111511	0.464696	RPL32
0.665302304	0.019388398	0.931005601	0.04659	0.258375	-0.07421	MSP1
0.666523626	0.956181442	0.616944383	0.04753	-0.01693	-0.20777	YFR018C
0.666523626	0.986465263	1	0.585189	-0.14006	-0.32945	None
0.667930234	0.989216982	0.15492901	0.583892	-0.12911	-5.83846	MATALPHA2
0.668121988	0.93221185	0.5927044	-0.07849	0.035739	0.358972	CTF8
0.668445385	0.224093879	0.045896858	-0.03155	-0.10275	0.343882	UTP11
0.668881356	0.938960046	0.550781538	0.049901	0.021428	-0.24397	MRS2
0.668881356	0.92847479	0.915894177	-0.0454	0.02141	-0.08248	YEL025C
0.669441699	0.003108642	1	-0.04869	0.330883	0.007574	YMR31
0.670188597	0.295242295	0.364904247	0.071238	0.207466	-0.44868	MIG2
0.670987196	0.231779764	1	0.046336	-0.15021	-0.01027	AGC1
0.671747817	0.367445036	0.38629944	0.054177	-0.14058	0.334544	KAP122
0.673189357	0.717157234	1	0.055075	0.068715	-0.0283	FSH3

0.673264191	0.584277189	0.072446037	-0.07042	-0.12171	-0.70404	PHO5
0.673742761	0.03264687	0.400503761	0.407734	-2.4779	-3.57367	YRF1-1
0.677031058	0.70751687	1	0.333807	0.432108	-0.14585	None
0.678217591	0.737846426	0.642912812	0.029343	0.034933	-0.12692	ADE16
0.679475883	0.779766213	0.09223367	0.036145	-0.03713	0.354672	RSA3
0.680722956	0.43696685	0.410236148	-0.04181	0.097143	0.25592	SDC1
0.680785037	0.649577741	1	0.045853	-0.06972	-0.01391	SNQ2
0.681447698	0.156630548	1	-0.06957	0.263389	0.009113	HEM25
0.682334688	0.051155386	0.89043028	0.105313	-0.52958	-0.22703	OYE3
0.684054727	0.888898048	1	-0.03689	-0.02381	-0.00501	BUR6
0.684795906	0.826578265	0.526996356	0.056612	-0.05002	-0.29899	MRE11
0.686905711	0.027146402	0.848126811	0.167426	-0.96181	0.443529	None
0.687476385	1	0.708809273	-0.05207	0.006654	-0.20144	THP2
0.687541673	0.138242749	0.278240376	-0.0482	0.192686	-0.35789	YLR345W
0.687541673	0.665960377	0.91703897	-0.04186	-0.06201	0.079476	DYN1
0.688959977	0.967392794	0.264879577	0.031741	0.010792	-0.24131	ALG6
0.688959977	0.758077955	0.471735065	0.349588	-0.4114	-2.15906	None
0.688959977	0.028113606	0.72853715	-0.04151	-0.23341	0.152172	BRE1
0.688959977	0.080514417	0.736753793	-0.05236	0.241257	0.187396	INA17
0.688959977	0.363817999	0.764689351	-0.04304	0.116883	0.143618	RDI1
0.688959977	0.230929934	1	0.622005	-2.2335	-0.73235	None
0.689474832	0.724774474	0.609220601	-0.11953	0.152521	0.561916	None
0.689829264	0.663252335	0.001354067	-0.04465	-0.06734	-0.73166	DIG2
0.689829264	0.604770313	0.299842252	-0.02812	0.04862	0.204146	GRS1
0.689829264	0.230911149	1	0.619436	-2.23495	-0.72931	None
0.689852181	0.516700017	0.641078964	0.03732	0.076421	0.16761	LTP1
0.690279029	0.333121659	0.976378097	-0.04442	0.127877	-0.0552	ATX2
0.690472783	0.679403914	0.32087275	-0.05105	0.073605	-0.36076	PCL7
0.690472783	0.950105874	0.907281575	0.035712	-0.01449	0.072173	NHP10
0.691851595	0.098514402	1	0.048443	-0.21635	-0.02106	LCD1
0.692221997	0.088550918	0.243308748	-0.03015	-0.13808	0.240531	EMP70
0.69238084	0.591976174	0.951201607	0.042705	-0.07682	0.065712	PSF1
0.692775459	1	0.919926509	0.051423	-0.00219	-0.09671	CWC23
0.692802831	0.289155055	0.709422392	0.051106	0.16013	-0.20092	LEA1
0.693429421	0.727504645	0.605911675	-0.21636	-0.28029	-1.08515	YLL067C
0.693429421	0.177202487	1	0.045617	-0.17286	0.005958	SEC10
0.693445904	0.702176983	0.981181758	-0.08709	0.119688	-0.10275	MAY24
0.694234071	0.752831669	1	0.042116	-0.05035	-0.01504	NST1
0.694362921	0.955475747	0.508217414	0.059578	0.023081	-0.33264	ENV7
0.694362921	0.868862376	0.930835922	0.030877	0.023109	-0.05398	DCW1
0.695224793	0.682031984	0.379660556	0.031615	0.046006	0.210509	ATG27
0.697224996	0.408969916	0.223381301	0.041385	0.106192	-0.34369	PNG1
0.698973374	0.853333651	0.862693719	0.03805	-0.03112	0.097072	FOB1
0.699277539	0.128591907	0.043873822	-0.03524	0.148983	0.421234	RPL24A
0.699537071	0.185421497	1	-0.03268	0.123651	-0.00886	QNS1
0.700436878	0.479033885	0.789517744	-0.03274	0.074524	0.105832	RVB2
0.701745425	1	0.822329423	0.046823	-0.00314	0.138087	BET1
0.702051801	0.996655297	0.373162993	0.035697	0.007417	0.244529	RPS6A
0.703253553	0.689168252	0.855492746	-0.03392	0.049564	0.09002	GBP2
0.703827043	0.884827129	0.767199943	0.042453	0.030241	-0.14719	MTG1
0.70602754	0.753511485	0.855617443	0.052936	0.064929	-0.14132	ROG3
0.706631199	0.157568347	0.199257919	-0.03492	0.141734	0.309656	YSA1
0.708197992	0.125172898	0.559875531	-0.04112	-0.18045	-0.22095	EPL1
0.708976318	0.938960046	0.023505165	-0.04208	0.020109	-0.56453	CRD1

0.709092029	0.830118162	0.668635304	-0.0556	0.051694	-0.24988	MEC3
0.709249634	0.161545506	0.978193638	-0.03927	0.159543	0.05017	MSO1
0.709257835	0.019381107	0.771051473	0.028534	-0.17973	0.099462	CDC11
0.709424754	0.335205662	1	-0.02692	-0.0818	-0.00884	VMA13
0.709424754	0.383120823	1	-0.13578	0.37649	-0.03362	ECM11
0.710381928	0.318028924	0.169244459	-0.03349	-0.10498	0.314111	LOS1
0.710557606	2.3107E-08	0.598011569	0.048988	0.615883	-0.24878	SYM1
0.711162215	0.020478658	0.803017727	-0.06974	0.435151	0.222709	YIL156W-[illegible]
0.711400069	0.48908547	1	0.025831	0.059364	0.025649	TEF4
0.711998904	0.266200922	0.536035164	0.050176	-0.17336	0.284041	YOL075C
0.7137144	0.057110214	1	0.086086	0.45686	-0.04586	None
0.715636546	0.206991704	0.889649609	-0.0373	-0.14274	-0.08826	ACK1
0.716831187	0.045036915	0.181792015	-0.03604	0.20211	-0.33575	BAP2
0.716848816	0.760915703	0.597863762	-0.05141	0.063046	-0.26688	YLR446W
0.717306642	0.076398079	0.90086308	-0.06341	0.320881	0.143589	YMR122C
0.717326089	0.186602961	0.98500242	-0.03158	0.125496	0.037747	AIM6
0.717942856	1	0.036953396	-0.02938	-0.00355	0.382546	FUR1
0.718336966	0.086191139	0.611594186	-0.03609	0.17864	0.1846	MRP21
0.718454963	0.509499029	1	0.035228	-0.07979	0.007276	EXO70
0.718586345	0.069544572	0.847404354	0.032067	-0.16697	0.091256	FKS1
0.718586345	0.237544743	0.889649609	0.036251	0.132857	0.087127	RSM18
0.71897404	0.084977816	1	-0.50925	-2.96925	0.563427	YIR042C
0.71917657	1	0.530625906	0.051509	-0.00699	-0.29957	CYC2
0.71917657	0.533524502	1	-0.03128	0.067582	-0.01874	PAP1
0.71961551	0.939281122	1	0.235919	0.116057	-0.09766	YOR029W
0.720423195	0.069544572	0.837234982	0.04079	0.212999	0.121928	AFG1
0.721265554	0.760915703	0.608460014	-0.03916	0.048777	-0.20314	MTC6
0.721345399	0.934155569	0.206936896	-0.43819	0.227122	-4.8469	YJL136W-[illegible]
0.721431252	0.335509445	0.868996053	-0.0465	-0.14669	0.123447	RFM1
0.721770493	0.099512037	0.238946444	-0.04825	0.233406	0.423289	TSC3
0.722757993	0.073267239	1	-0.03023	0.157397	-0.00094	PET123
0.723659867	0.371129486	0.625394004	0.048721	-0.14585	-0.24756	TTI1
0.723659867	0.658171258	0.770752057	0.038324	-0.06487	-0.13964	RIB2
0.723975267	0.590778718	0.306543708	0.047834	0.094549	-0.38136	YDR131C
0.724136468	0.184130852	0.98500242	0.048485	-0.19915	-0.06068	DSF2
0.724378287	0.050831616	0.931568877	-0.03825	-0.21644	0.071698	MCM6
0.724969532	0.299931985	0.888371569	-0.03934	-0.13323	-0.09771	MFB1
0.726214237	0.387128577	0.709738269	-0.0342	-0.09964	0.148946	UBP13
0.726997677	0.734964949	0.726816534	-0.11324	-0.15774	0.468361	OSW2
0.727568416	0.731503094	0.800001697	-0.03801	-0.05339	-0.12907	YSH1
0.727966785	0.083848492	1	0.02602	-0.13431	0.014604	FRS2
0.728239451	0.407136042	0.920011392	-0.29463	-0.85824	-0.6506	YMR316C-[illegible]
0.728933041	0.925594807	0.97620447	0.054343	-0.03115	-0.07624	SLI1
0.729278209	0.185910487	0.632443786	0.042987	0.178008	-0.21971	UBS1
0.729818674	0.031089948	0.644487558	-0.03168	0.197253	0.159276	YML079W
0.730780656	0.014495134	0.921286576	0.028217	-0.19707	0.058926	ADE6
0.731357276	0.103376814	0.712223429	0.034505	-0.17111	0.151116	TDA9
0.731444934	1	0.697869118	-0.24151	0.029203	-1.13551	YMR175W-[illegible]
0.734459248	0.675411275	0.047172169	-0.03538	0.058879	-0.46054	GNP1
0.734459248	0.895413787	0.238946444	-0.39058	-0.28145	-4.40453	None
0.734459248	0.304011642	0.830848068	0.115997	0.39962	0.367016	YOR376W
0.737638875	0.993946665	0.919926509	0.099755	-0.02514	0.214442	None
0.737809583	0.295649423	0.876200558	0.037647	0.133251	-0.10079	CAF130
0.740157404	0.228288339	0.129195117	0.032749	0.130291	0.359118	LSM2

0.740266656	0.19026538	0.781875866	0.052101	-0.22228	-0.19365	MDS3
0.740939486	0.591976174	0.652552516	-0.02869	-0.05957	0.147231	LEM3
0.741145139	0.168293937	0.425590725	0.046021	0.20364	0.330468	POP8
0.741555782	0.039817045	0.255041627	-0.03622	0.224273	0.329145	SFT1
0.742167299	0.794730765	1	-0.0888	0.104275	-0.02033	FUS2
0.743370873	0.78818634	0.339657587	0.044366	-0.0536	-0.35817	NIT2
0.743636844	0.470034541	0.648751155	-0.03816	-0.10118	0.199059	CTK1
0.74364414	0.088550918	0.929344228	-0.04496	0.239559	-0.09177	TMT1
0.744488753	1	0.538133644	0.041896	-0.0005	-0.26029	BTS1
0.74565969	0.766166784	0.326601143	0.027891	0.036646	-0.23108	HOM3
0.745781249	0.712206218	0.688473731	0.109211	-0.1732	-0.53614	YEL008W
0.746401665	0.334248187	0.261450758	0.035459	0.120896	0.324552	FPR1
0.74720737	0.842773453	0.89719832	0.072783	-0.07219	-0.18557	YDL218W
0.747319408	0.322712789	1	-0.04772	0.165947	-0.00879	LDB17
0.747880376	0.904357451	0.961702496	-0.13545	-0.09593	-0.23219	YJL045W
0.748659099	0.892678302	0.98198617	-0.02523	0.019356	0.035014	VMA6
0.749710528	0.853333651	0.87592683	-0.04319	-0.04127	-0.12081	TRS85
0.749782945	5.66833E-09	4.09193E-05	-0.05125	0.757619	-1.1823	SPO24
0.751036919	1	0.711421266	-0.03165	0.005994	-0.14754	CDC28
0.751275079	0.511816481	0.014757925	-0.02584	-0.06465	0.422947	RRP8
0.751521267	0.94982823	0.871950707	0.033217	-0.0164	-0.09516	GCN5
0.75209573	0.009536972	0.97620447	0.032862	0.257997	0.049539	MSK1
0.752582212	0.829832371	0.214567398	0.167704	0.180294	-1.71672	YFR012W-
0.752893877	0.472617197	0.160504258	-0.03475	-0.09463	-0.37878	KIN28
0.752953266	0.046144661	0.834791774	0.039645	-0.25063	-0.133	CTS2
0.754518851	0.905759808	0.278240376	0.028363	0.020358	0.26064	RMD9
0.75478555	0.601216089	0.715024856	0.253278	0.538473	-1.19149	None
0.755492371	0.825517831	1	0.348351	-0.39377	0.127758	PAU13
0.755773713	0.280013981	0.910579658	0.036691	-0.14228	-0.08889	SFP1
0.756294057	0.000280237	0.238946444	-0.03034	0.312566	0.297219	MRPL23
0.757840918	0.53056287	0.529437087	-0.06067	0.14921	0.400393	TIM9
0.758437357	0.085297697	0.841416921	-0.06716	0.378216	0.221483	None
0.759002377	0.69190085	0.920011392	-0.09604	0.165439	0.220662	YHR175W-
0.759639957	0.612922906	0.795907717	-0.03011	0.063662	0.114804	RET3
0.76023114	0.091928673	1	0.204939	-1.18577	0.0632	YPR096C
0.760670399	0.871347596	1	0.034769	-0.03165	-0.04017	MPD1
0.761744913	0.212423016	0.744376434	-0.03852	-0.16951	-0.16967	AIR2
0.762707677	0.004542745	0.848379499	0.039149	0.340963	0.127714	FMC1
0.762819944	0.397361658	0.187817207	-0.02617	0.084141	0.282019	RPL15A
0.764017002	0.490597664	0.492012834	-0.02845	0.077062	0.202284	EMP24
0.765274449	1	1	-0.04369	-0.00506	-0.04307	RBH1
0.765509221	0.358263416	0.91703897	-0.04268	0.148823	-0.10292	RRD1
0.765710977	0.530999534	0.98500242	-0.18981	0.475811	-0.26852	YLR406C-
0.766925771	0.83604285	0.847404354	0.035669	-0.03913	0.118807	SEN54
0.767321073	0.582169769	1	-0.02835	-0.06558	-0.01201	YGR283C
0.767408387	6.38852E-05	0.265410494	-0.04231	0.487256	0.410841	YBR285W
0.767522081	0.795533688	0.871412074	-0.04518	-0.05758	-0.13701	PFA3
0.77050198	0.892758485	0.920656396	0.028424	0.023409	0.067674	VPS29
0.77050198	0.889553139	0.98500242	-0.03554	0.029917	-0.05053	CBP1
0.770574734	0.788431804	1	0.025272	-0.03329	-0.01738	NUP49
0.770831351	0.741539213	0.917519739	0.033472	-0.05262	-0.08203	MKK1
0.771693932	0.004611469	0.847404354	0.030622	-0.27657	-0.1038	RNR1
0.772245682	0.446288659	1	0.040014	-0.12183	0.022101	SBE2
0.772443982	0.029454084	1	-0.03873	0.280137	0.042121	PIB1

0.772507866	0.207863495	0.186880865	-0.02655	-0.12223	0.296506	ROK1
0.773075834	0.861384572	0.557079281	-0.16193	0.160355	-1.07649	BSC4
0.773075834	0.709758226	0.870558299	0.032479	-0.05665	-0.10118	ZRG17
0.775002391	0.614981392	0.211271753	0.032007	-0.07107	-0.34695	ADD66
0.775376924	0.023022877	0.011257238	0.027988	-0.21374	0.510243	MAK5
0.775717756	0.343862699	0.659101564	-0.03066	0.113667	0.175599	CHZ1
0.776189469	0.836107259	0.669483248	-0.13885	0.156251	-0.77765	None
0.776753567	0.788820512	0.358747602	0.093953	0.126404	-0.82583	PAU23
0.778244578	0.836389864	0.919926509	-0.02747	-0.03117	0.068245	ERD2
0.778324119	0.542581881	0.048861894	0.025897	-0.06712	0.392437	CIC1
0.778454087	0.698820282	0.659791573	-0.46981	0.845766	2.265832	None
0.778868164	0.281429987	0.588105746	-0.03316	0.138439	-0.21479	BPL1
0.779091319	0.00054349	0.111100599	0.028537	-0.30961	0.370851	NOC3
0.780300524	0.87305028	0.007546041	-0.04097	-0.03976	-0.78633	PLM2
0.78094865	0.69799932	0.658909952	-0.46455	0.848245	2.279675	None
0.782005521	0.920869756	0.611594186	-0.0489	0.034594	0.30973	CIN4
0.782005521	0.055168494	0.739590174	0.034081	0.228801	0.163054	MSF1
0.782318078	0.938960046	0.989809599	0.041059	-0.0251	-0.05899	NAM2
0.78323441	0.482993634	0.415890779	-0.40884	-1.2782	3.656117	None
0.783421666	0.468265571	0.084446467	-0.02563	0.078154	0.355398	RPS19A
0.783421666	0.27087925	0.908854398	0.156571	0.675448	0.421587	YHR086W-A
0.784660676	0.996805372	0.974707572	0.056265	-0.01507	-0.09649	SDS3
0.784687031	1	0.661089241	0.046202	-0.00349	0.272687	POL30
0.785741087	0.413008972	1	-0.02382	-0.08111	-0.01296	TPK3
0.788089786	0.32591793	1	0.03956	-0.15888	-0.00063	SLX8
0.790169822	1	0.717477893	0.092624	0.015703	-0.48708	YGL041W-A
0.790489629	0.039069167	0.643429399	-0.03522	-0.26109	-0.21766	CBK1
0.790800921	0.821207297	0.62113714	-0.0428	-0.05428	-0.27446	VPS3
0.791584853	2.03535E-05	1	0.097612	1.285038	0.011553	OLI1
0.792375753	0.156840624	0.542977891	0.02893	-0.15755	0.212031	SAK1
0.79310432	1	0.971609311	0.033371	0.004521	0.061831	MUB1
0.793817233	0.161181868	0.667670933	0.026071	-0.14151	-0.15659	MOT2
0.793817233	0.439851325	0.681411478	0.023576	0.078674	0.137563	TMA22
0.793817233	0.192026738	1	-0.02939	0.150148	0.032477	CWC15
0.793817233	0.23043753	1	-0.03621	-0.17392	0.00222	YCS4
0.795069256	0.05095414	0.960632458	-0.02458	-0.17688	0.050361	RAD3
0.796337389	0.514232347	0.974952294	0.130869	0.38184	-0.2325	YIL012W
0.79710358	0.473307985	0.906628341	0.039516	-0.12577	0.114025	KRE28
0.798462811	0.98811108	0.364904247	0.042437	-0.01468	-0.40292	YMR111C
0.800166016	0.104019631	0.932413507	0.030637	-0.19254	-0.07419	NGG1
0.800166016	0.995096172	0.971609311	0.059707	-0.01823	-0.11201	None
0.800166016	0.00058878	1	0.026752	-0.31204	-0.01547	MNN1
0.800273415	0.050831616	0.69956533	0.022021	0.161127	0.126883	TOM22
0.800273415	0.407136042	0.703759167	-0.02872	-0.10433	-0.16333	TFC6
0.800736945	0.408586762	0.333893948	0.022528	-0.08185	0.225803	TYW1
0.800736945	0.073350804	0.541508341	0.331884	2.452195	2.71354	None
0.801592433	0.93456048	0.570229598	0.203097	0.140162	-1.51136	None
0.802180753	0.059197475	0.971609311	-0.03937	-0.28278	0.074162	HSH155
0.802936411	0.11876651	1	0.036241	0.222738	0.012427	MSW1
0.803140792	0.267371286	0.733341705	-0.34208	-1.77951	-1.86449	None
0.804928369	0.603567849	1	-0.03224	0.08206	-0.01686	LDB16
0.805511123	0.030015186	0.00166885	0.024749	0.202318	0.591655	RPS29B
0.805511123	0.347850933	0.080581045	0.023468	0.096668	0.357754	RPL40B
0.805511123	0.328528692	0.886876142	0.064253	0.273942	-0.2118	ATG4

0.805799559	1	0.836718664	0.022259	0.001152	-0.08964	THO1
0.806103544	0.855981392	0.697639077	0.028942	0.033564	-0.17202	LDB19
0.806569528	0.295390559	0.76828009	0.030176	-0.13743	-0.14832	ACE2
0.806569528	0.850282712	0.981177966	-0.02198	0.026178	0.038105	AYR1
0.808260144	0.782318684	0.509608089	-0.02842	0.044148	-0.23232	ADD37
0.808260144	0.652698925	0.727908382	-0.0323	0.074366	-0.17665	YDL177C
0.808260144	0.056645029	0.772879577	0.024791	-0.18432	0.121042	TRZ1
0.808260144	0.914039764	0.840006224	0.020475	-0.01696	0.082387	THR1
0.808260144	0.097739705	1	-0.02311	-0.15286	0.028959	PUS1
0.808539875	0.342508719	0.066288571	-0.02118	0.089468	0.339557	EGD1
0.810772189	0.604770313	0.417425061	0.206208	0.537698	1.941711	None
0.811262405	0.013981458	0.113134528	0.028127	-0.26352	0.412381	MAK21
0.811356683	0.513831581	0.488400105	-0.0297	-0.09268	-0.25427	BBP1
0.811866319	0.066877727	0.978377916	-0.0286	-0.20877	-0.05169	TOP2
0.812174707	0.065585799	0.333626467	0.030557	-0.22469	-0.32088	YBL029W
0.813702505	0.958689082	0.629718524	0.034383	0.019409	-0.23878	STP2
0.815519128	0.805302069	0.904680493	0.027383	-0.04059	0.085823	SLN1
0.815904531	0.029468425	0.167222206	0.056191	0.480874	-0.76086	RRT8
0.815904531	0.105580188	0.656171223	0.028596	0.190656	-0.19204	COT1
0.816064317	0.349120443	0.69956533	0.036924	-0.15946	-0.2274	DBF20
0.816295035	0.665829866	0.952440864	-0.06578	-0.15189	0.153733	YHR213W-[illegible]
0.81743139	0.189863208	1	0.027715	-0.15728	0.007982	YCK3
0.819104307	0.182752817	0.726816534	-0.02804	-0.16196	0.161436	NUP157
0.819177318	0.113397822	0.411752857	0.029088	-0.19459	-0.28459	TFC8
0.819177318	0.621834431	0.771782988	0.019903	-0.05169	0.102024	ALA1
0.819537047	0.472617197	1	-0.03434	-0.12029	-0.01287	SKI3
0.819978239	0.564303748	0.998341694	-0.02033	0.059873	-0.0309	APM4
0.819983682	0.892744236	0.254952703	-0.27014	0.266652	3.403657	None
0.82029634	0.193372823	0.119718988	-0.02918	-0.16688	0.440159	RPA190
0.82029634	0.904357451	0.702041373	-0.04042	-0.03771	-0.25191	YGR015C
0.82029634	0.143407346	0.752502722	-0.03089	0.193475	-0.16628	WSS1
0.82029634	0.758162333	1	-0.04757	0.085876	-0.04219	PET112
0.820593683	0.886323024	0.491465578	0.046076	-0.04876	-0.40853	SET6
0.820593683	0.358012927	0.7225564	0.032805	-0.14286	0.192736	HIR2
0.82149107	0.84140879	0.930722711	0.02963	0.039175	-0.08069	AEP1
0.822994405	0.0810865	0.981177966	-0.0343	-0.2532	-0.06387	ESP1
0.823381987	0.088550918	0.869100567	-0.02949	-0.21383	0.113178	TEL2
0.823583991	0.759694936	0.661759663	-0.02472	-0.04504	-0.17038	BSP1
0.823646359	0.16750316	1	0.092313	-0.56887	-0.06991	SWC7
0.824645602	0.52090053	1	0.020749	-0.06761	-0.02433	WHI4
0.825498282	0.201173553	0.059232365	-0.02371	0.136205	0.417694	RPS18A
0.826124989	0.602717812	1	-0.27567	0.764887	-0.39328	None
0.826314738	0.826578265	0.735602795	0.164723	-0.24018	-0.96698	None
0.826351497	0.72990982	0.457306305	0.025849	0.052703	0.24764	YRA2
0.826351497	0.261636207	1	0.030324	-0.15981	0.017626	SGM1
0.826975391	0.398157956	1	0.13535	-0.5751	0.156432	YIL086C
0.828165816	0.384001707	0.318919245	0.022616	0.097021	0.262044	EGD2
0.828165816	0.202307461	0.537891256	-0.02875	-0.16768	0.247608	TCB2
0.828165816	0.825517831	0.680761083	-0.03638	-0.05343	-0.24642	IRC6
0.829058241	0.504911953	0.846846052	-0.22205	0.754778	-0.97463	MRPL38
0.829395869	0.45341927	0.586097635	-0.09592	0.362523	0.768959	SRT1
0.830471847	0.942368879	0.484106841	-0.33572	-0.2406	-3.44844	YJR151W-[illegible]
0.830471847	0.512778293	1	-0.03041	-0.10371	-0.00939	SYF1
0.830532264	0.27087925	0.411976149	0.021581	0.113694	0.222734	CPR5

0.830532264	0.617350035	1	-0.03087	-0.08572	-0.02771	SPC72
0.831532169	0.172011278	0.154422599	0.035256	0.219925	-0.52606	IMO32
0.831532169	0.303874634	0.803816091	-0.02766	0.138104	-0.13722	IML3
0.832095654	0.970365023	0.06522464	-0.02203	0.011922	0.39377	TMA16
0.834321911	0.879819172	0.960655888	0.032997	0.039013	-0.07976	SEF1
0.834434629	0.343957891	0.913908205	0.024032	0.113741	-0.07838	THG1
0.834739623	0.290286754	0.672010939	0.028975	-0.15127	0.206357	KAP120
0.837324998	0.885372644	0.705630037	0.034522	0.039661	-0.2304	YKL222C
0.837747075	0.582123297	0.201440577	-0.01733	-0.05353	-0.24768	HHO1
0.838641409	0.518133314	1	-0.02062	0.071756	-0.01691	YKT6
0.840379492	0.9690209	0.135324685	0.028098	0.016084	-0.44871	PAR32
0.841243895	0.055657167	0.870558299	-0.0235	0.202116	0.096974	NAS6
0.841822223	0.022302219	0.014857079	-0.02142	-0.21656	0.502353	ERB1
0.842287266	0.38127191	0.69579659	-0.02271	0.104321	0.159649	YKL033W-A
0.842399319	0.354272098	0.417425061	0.029359	0.141664	-0.31638	YKU70
0.842399319	0.43696685	1	0.01666	-0.06924	-0.01397	ILV2
0.842767051	0.265350931	0.91703897	0.023831	-0.13451	-0.07935	STV1
0.843656281	0.58105513	0.886952303	0.030301	-0.09667	0.119062	PET309
0.843679477	0.414293185	0.58876642	-0.02991	0.129764	-0.25522	SIR1
0.843679477	0.843393723	0.902712809	0.021224	-0.03072	-0.07752	MUD2
0.843679477	0.643758676	0.960632458	-0.0273	-0.07557	0.069073	TOF2
0.843943698	0.54996536	1	0.077139	0.261075	-0.04723	None
0.844982905	0.892236467	0.952440864	0.027488	0.031175	0.073267	DER1
0.845364165	0.691732797	1	0.022609	0.055724	-0.01105	PRI1
0.845826143	0.080426839	0.654230288	-0.27416	2.403737	1.969816	None
0.845872011	0.94606055	1	-0.11883	-0.08926	0.075558	None
0.846291919	0.887970391	1	-0.2321	0.27228	-0.37703	None
0.847406303	0.150742703	0.652552516	0.257608	-2.09362	-2.52563	None
0.847406303	0.206110478	1	0.036332	-0.23159	0.020605	MCM22
0.84785635	0.035632723	0.256670588	-0.0378	0.36478	-0.5198	YGR035C
0.84879128	0.610223632	0.76828009	-0.06591	0.202654	-0.39368	AUA1
0.849892707	0.997630214	0.98500242	-0.0239	-0.00823	-0.04791	YMR102C
0.850016452	0.002281388	0.892830092	0.019298	0.256584	0.075611	SDH3
0.850230548	0.04046433	0.41680372	-0.02572	-0.24734	-0.28931	THI3
0.850972575	0.57792812	0.715570823	-0.02005	-0.06674	-0.13882	MTC5
0.851487269	0.839560652	0.698289037	-0.18098	0.274319	-1.33029	SAE3
0.851835576	0.447812948	1	0.025679	0.109453	-0.00847	YPT6
0.852305008	0.823461891	0.837446458	-0.01841	-0.03059	-0.09163	NPP1
0.853027215	0.332602552	1	0.037763	-0.20078	-0.04314	YDL211C
0.854759479	0.321781322	0.951428598	-0.04286	0.232033	0.120159	YGR153W
0.855144132	1	0.546032589	-0.11644	-0.01359	1.124225	None
0.855144132	0.413242794	0.750416368	0.018156	0.08383	-0.11639	CSG2
0.855151195	0.004742556	0.889649609	0.07049	0.90228	-0.28757	YDR010C
0.856247191	0.724702775	0.238946444	-0.02159	-0.05176	-0.3212	CDC16
0.85709318	0.304299791	0.324585433	-0.20846	1.172614	-3.00081	COX2
0.857618886	0.551390968	0.930835922	0.253586	0.922568	-0.80037	None
0.85848676	0.205804692	0.368959414	-0.01696	-0.11399	-0.21238	TOM71
0.858514673	0.855524783	0.71503018	-0.02318	0.034491	-0.16699	TOK1
0.860163333	0.030733392	0.924605282	0.027941	-0.29592	-0.09643	SYG1
0.860206122	0.950685743	0.118862393	-0.01862	0.014261	0.341197	RRP1
0.860887449	0.624729213	0.616944383	0.105163	0.331963	-0.94021	None
0.861085725	0.333037056	0.919935222	0.020802	-0.115	-0.07413	PEX8
0.861220657	0.514749074	1	0.021934	-0.08673	-0.03388	YTA7
0.861256332	0.490088889	0.93147427	0.016768	0.069303	0.054667	GIR2

0.861393903	0.136763555	1	-0.32647	2.527707	-0.4049	None
0.861550343	0.763573287	0.699858425	-0.01917	-0.04127	-0.14702	YVC1
0.862094348	0.999736798	0.420929331	-0.02222	-0.00772	-0.26455	QDR3
0.862332079	0.443918989	1	-0.15331	0.691204	-0.15059	None
0.863158217	0.191795718	0.00394694	0.027856	0.196746	-0.82411	UBA3
0.864258618	0.122042485	0.467852646	-0.02854	0.232518	0.325303	TGL2
0.865036236	0.066004473	1	0.02304	0.217583	-0.01689	SCO2
0.865036236	0.363817999	1	0.18303	0.972316	-0.12178	None
0.865214184	0.031947208	0.88808385	0.01771	-0.19202	-0.07783	KRE6
0.865214184	0.882360286	1	-0.18308	-0.25044	-0.25993	None
0.865822744	0.054047303	0.484474484	-0.04703	0.462474	-0.52406	PDH1
0.866012927	0.471162403	0.76367822	-0.01759	-0.07799	-0.11653	PPQ1
0.866497291	0.387128577	0.783364287	0.014115	-0.07292	0.08905	TIF3
0.867393206	0.014780962	0.713888298	0.024389	-0.29923	0.18635	SPT21
0.86935396	0.374954065	0.710774648	0.02507	-0.13537	-0.19614	CCZ1
0.86935396	0.602459029	1	0.040714	-0.14276	-0.00212	YNR064C
0.87033159	0.184130852	0.492136803	-0.02309	-0.1725	0.2631	YDR222W
0.870936285	0.182567214	0.01735184	0.015337	0.114961	0.411955	RPS20
0.870936285	0.922137802	0.67221425	0.021038	-0.0223	0.180369	SMB1
0.870936285	0.966122236	1	-0.03976	0.027821	0.062625	YPL034W
0.872863779	0.517152851	0.281435365	0.027598	0.115474	-0.42089	POP6
0.874667838	0.726146888	1	0.014538	0.038312	0.024257	TDA3
0.875357573	0.099936663	0.990712634	-0.01453	-0.13273	-0.03163	CCS1
0.876973071	0.885372644	0.949939733	0.019826	0.028091	-0.06354	YER010C
0.877722989	0.736287769	0.883018242	-0.0558	-0.14538	-0.2684	None
0.88015479	0.427585494	0.906628341	0.018228	0.093914	0.078375	TCP1
0.880488741	0.967469415	1	0.013494	-0.00975	0.001326	HEK2
0.880707838	0.889053342	0.702041373	-0.02462	-0.03446	-0.20786	TEX1
0.880913595	0.024098511	0.179756965	0.013088	-0.16154	0.243692	RPA49
0.880913595	0.209597822	0.570988724	-0.02136	0.160831	-0.22836	LYS5
0.880913595	0.496585998	0.69828171	0.140842	0.638042	1.202501	None
0.880913595	0.826578265	0.733389979	-0.01718	-0.03288	0.133572	GDT1
0.882005661	0.885106299	0.677620076	0.024319	0.035709	-0.2187	PCA1
0.882754207	0.730740342	0.901225243	0.015788	-0.04302	-0.07159	HFI1
0.883115881	0.950105874	0.824664851	-0.02037	0.017839	-0.12486	GEP5
0.883678378	0.514232347	0.226565606	0.239923	1.080655	4.446068	None
0.883678378	0.633340819	0.47679402	-0.01391	-0.04904	-0.17456	VPS74
0.883678378	0.759529423	0.769266218	-0.18066	0.442499	-1.29659	None
0.883678378	0.915872899	0.888371569	-0.02168	0.025692	0.105626	SSU72
0.883768911	0.064038126	0.016020217	-0.03859	0.405947	-1.13124	SRD1
0.88388589	0.583819577	0.514203536	0.021111	-0.08354	0.252757	ORC3
0.888160932	0.438384456	1	0.013175	-0.07017	0.015746	RVB1
0.888352736	0.849224058	1	0.158587	-0.28965	-0.10389	None
0.888540912	0.888648886	0.051772428	0.225316	0.334894	-7.15141	YDL007C-A
0.888540912	0.189863208	0.894445004	0.016581	-0.13602	-0.07991	GLY1
0.889158621	0.214629898	0.859769494	0.019942	-0.15726	0.112993	MOT1
0.889415532	0.346188985	0.719999469	0.02926	0.185292	-0.24803	UBC12
0.889451054	0.084573821	1	-0.02595	-0.26939	0.015818	NUP159
0.889478682	0.255932116	0.947945497	-0.05451	0.401678	-0.19035	None
0.890274194	0.884320839	0.739335611	0.194558	0.303991	1.569355	YOR011W-A
0.890925144	1	0.943056599	-0.02673	0.009002	-0.09665	GMC1
0.891275448	0.657364999	0.67221425	0.025126	0.087854	-0.24204	RRG1
0.892492737	0.066370363	0.919926509	-0.01947	0.215484	-0.08357	COQ9
0.893863043	0.731106025	1	-0.23811	0.691171	-0.35962	None

0.89401981	0.154494068	0.47497166	-0.02301	0.208396	-0.30811	NCA2
0.894387718	0.378211699	0.365762079	0.013817	-0.08583	0.214635	CNS1
0.894549227	0.247677793	0.164360131	0.01656	0.128031	0.345432	VMA10
0.895627954	0.663621022	0.69579659	-0.014	0.049349	0.133071	BCP1
0.895627954	0.436731198	1	-0.01987	-0.11167	-0.00195	CDC4
0.895983078	0.862941666	0.565513961	-0.01936	-0.0347	-0.23046	LDH1
0.896269289	0.893976085	0.045166127	0.20195	0.302212	-7.08879	RRT7
0.896739779	0.386672121	0.348820609	-0.0165	0.101988	-0.26545	MDM34
0.896739779	1	0.822329423	0.022287	-0.00076	-0.15044	HUA2
0.896739779	0.092327558	1	-0.01737	-0.18569	0.001697	DSN1
0.897065667	0.321967781	0.114649943	-0.05111	0.354016	-1.18356	REC8
0.897673972	0.134923757	0.891215531	0.017197	-0.16743	0.089441	KAP95
0.899013343	0.372622288	0.082586739	0.014615	0.094641	0.36264	RPL19A
0.899013343	0.001867712	0.103916575	-0.02519	-0.45905	0.606271	YBL113C
0.899013343	0.39094198	0.478124319	-0.02185	-0.13658	0.303245	TCB3
0.899117306	0.252920299	0.440858646	0.018788	-0.14964	0.274366	SNF1
0.899117306	0.36634644	0.737914729	-0.02043	-0.13443	-0.17642	SKT5
0.89914586	0.018092653	0.967406184	0.017789	0.25702	0.058321	COQ4
0.899155126	0.098340256	1	-0.04482	-0.48319	-0.04461	CTF13
0.89997777	0.629816198	0.615253714	0.01531	0.060826	-0.17391	HLJ1
0.900034837	3.96256E-07	0.927845118	-0.04201	1.115715	-0.17968	STL1
0.900092856	0.939477921	0.82890057	-0.01186	0.012971	-0.08089	AAT2
0.900618802	0.41577527	0.831867858	-0.01782	-0.10799	-0.12095	SGN1
0.901939512	0.084342343	0.929303164	-0.01939	-0.21997	0.083609	MCM2
0.902538934	1	0.902712809	0.016936	0.006266	0.087458	CHC1
0.903118513	0.003765202	0.952295244	-0.01783	0.313456	0.06712	SDH5
0.903118513	0.999736798	1	-0.20122	-0.08859	-0.34313	None
0.903139988	0.925367028	0.962371769	0.097071	0.123234	-0.34167	None
0.90350216	0.561621643	0.69956533	-0.01197	-0.0566	-0.11942	ARP7
0.903515303	0.017246708	0.269021848	0.01457	-0.2206	0.277767	UBP10
0.903515303	0.381024561	0.689558052	-0.0195	-0.12967	-0.19946	CTF4
0.903515303	0.112287674	0.985958815	-0.01181	0.126382	0.032209	YMR315W
0.904475408	0.907732085	0.830189162	0.058051	0.084401	-0.40677	MER1
0.904564564	0.720356067	0.178594088	0.024912	0.081443	-0.54559	MSS18
0.904564564	0.508655752	0.781154036	0.025331	0.133047	-0.20529	THI74
0.904689069	0.172568227	0.103916575	0.045678	0.433829	-1.13422	SFC1
0.904906216	0.635232059	0.385959394	0.018766	0.076602	-0.30722	YUH1
0.90496132	0.788431804	1	0.016182	0.041955	0.018941	RPC17
0.905103014	0.547312091	1	-0.0178	0.087557	-0.01911	ERP4
0.906191739	0.678988713	0.672010939	0.016315	0.060192	-0.1762	IKI1
0.906225165	0.042568476	0.649787892	0.051815	0.692164	-0.58216	CRC1
0.906406561	0.317033104	1	-0.01714	0.129803	-0.02714	MIM1
0.906505816	0.362954426	0.844390643	-0.01479	0.103811	-0.10097	PRO3
0.906754139	1	0.795907717	-0.11301	0.013114	-0.90911	None
0.908531175	0.76724126	0.385959394	-0.01736	0.049911	-0.2922	YAF9
0.908531175	0.912049034	0.807027851	0.015429	0.022621	0.119555	BPH1
0.911326942	0.199127168	0.60682346	0.018838	-0.18019	0.237644	USO1
0.911326942	0.118712454	0.967394603	0.016469	0.184492	-0.05927	KXD1
0.911779228	0.039495936	0.509579361	-0.01748	0.247243	0.256177	MAM33
0.912591184	0.541557467	0.027182603	0.13635	-0.74145	5.739338	None
0.912704282	0.098003967	0.024892656	0.015633	0.185341	0.530257	RPS31
0.914352481	0.99176478	0.831761374	-0.01533	0.009271	-0.11548	CSH1
0.914917671	0.500260684	0.907281575	-0.01407	0.081016	-0.07701	RFA2
0.915727695	0.897457836	0.845425423	-0.01751	-0.02973	0.127318	RSC1

0.916124711	0.996753756	0.74594446	-0.01377	0.007505	0.132195	YGL140C
0.916895739	0.637757348	0.689893247	0.018471	0.081831	0.208391	ERP6
0.917115959	0.32591766	0.308976846	0.010468	0.08439	0.208812	ACT1
0.917682362	1	0.068005273	-0.01785	0.006864	-0.52724	GSC2
0.917682362	0.945788608	1	-0.01529	0.018017	-0.0186	RGD2
0.918013407	0.905759808	1	0.018958	-0.03118	0.040848	APN2
0.919551984	0.361237604	0.702041373	0.010338	-0.08026	0.116336	HOC1
0.919551984	0.767694173	1	0.015551	-0.04834	-0.03313	THP1
0.919731181	0.899105948	0.061432357	0.010684	-0.01854	0.329452	TRM1
0.91982216	0.302233138	0.506627176	0.023935	0.205124	-0.37442	LEU5
0.92018239	0.680755208	0.62830703	-0.01668	0.068103	0.217312	VMA21
0.921264085	0.931981036	0.444392343	0.153222	-0.21449	2.713385	None
0.922353756	0.000900424	1	0.013644	0.309209	-0.03006	YBR085C-
0.922393144	0.615223063	0.265639548	0.013093	0.063935	0.289643	RPL21B
0.922513127	0.759694936	0.647440092	0.02816	0.093254	0.36203	None
0.924747826	0.29991394	0.156558309	-0.01659	-0.14867	-0.43805	TIR1
0.925770676	0.57641366	1	0.014165	-0.07725	-0.01612	YHL017W
0.928289758	0.928554724	0.612191336	-0.0107	-0.01611	0.152108	VMA8
0.929977838	0.896286063	0.873533661	0.015728	-0.03012	-0.11348	SWD2
0.930225666	0.953675948	0.523868802	-0.0131	0.015758	0.217013	BOP3
0.930225666	0.091342198	0.960632458	0.012382	-0.17181	0.055075	KEX2
0.93335548	0.040834333	0.513019288	-0.01126	0.18636	0.193752	MRP20
0.933926177	0.37662751	1	0.011794	-0.09957	-0.03316	TAF8
0.933931712	0.935433283	0.108233883	0.140582	-0.2103	-5.50831	None
0.934098267	0.217407387	0.831867858	0.016436	0.18075	0.144531	STE18
0.93420116	0.335509445	0.093444318	0.017807	0.161515	0.56891	YIL002W-
0.934249309	0.883064096	0.08251531	-0.01505	-0.03312	-0.4903	PCL10
0.934318618	0.995958449	1	-0.06294	0.040654	-0.1838	None
0.93478579	0.177324846	0.930420244	-0.01825	0.217183	0.10082	CDC31
0.937136435	0.020434856	0.741079602	0.043651	0.82928	0.499983	YMR254C
0.937310107	0.023687856	0.867399954	0.016733	-0.31644	-0.13291	SMC1
0.938488981	0.028520862	6.51059E-07	-0.01209	-0.22411	-0.90028	MET1
0.939155046	0.797072613	0.139633449	0.016706	0.054904	0.512453	AMD2
0.939223782	0.295242295	0.985958815	0.011517	0.117281	0.041679	CBC2
0.939234136	1	1	-0.0105	-0.00583	0.015549	YGR125W
0.939360856	0.572433652	0.75226958	0.07614	0.466688	0.864266	YCL042W
0.939912227	0.940255249	0.218100263	0.175099	0.259113	-5.33176	None
0.940346212	0.57019157	0.802035501	0.009313	0.058112	-0.09438	STT3
0.940346212	0.666900591	0.927845118	0.051723	0.258284	-0.30755	None
0.941042698	1	0.962684605	-0.01283	0.006258	-0.06132	COQ10
0.942095126	0.939281122	0.217184527	0.170065	0.262226	-5.33202	None
0.94265266	0.304258036	1	0.016924	-0.17562	-0.01239	SFL1
0.942982775	0.843062252	0.721271027	-0.01187	-0.03391	-0.15381	DCC1
0.943044287	0.72085728	0.013045788	0.010556	-0.04719	-0.49732	TLG2
0.943671092	0.032709474	0.400211383	0.011939	-0.22801	0.264636	NAN1
0.944948639	0.076965336	0.566604088	0.011352	-0.18735	0.201634	NIP1
0.945622151	0.006785237	0.69828171	0.007603	0.180489	0.108302	UGP1
0.945622151	0.835126825	0.868996053	0.015508	0.047422	-0.13269	RMD5
0.946016792	0.230450293	0.942694497	-0.0112	-0.1361	-0.06327	MCM7
0.946570874	0.303946937	0.263985261	-0.00944	0.10182	-0.25806	SAM35
0.946570874	0.501310332	1	-0.00913	-0.06898	0.009051	HST1
0.94705238	0.484602574	0.972387712	-0.01084	-0.08523	-0.05006	NSG1
0.94705238	1	1	-0.01042	0.00664	-0.01694	SSQ1
0.947175874	0.665960377	0.021809306	-0.01491	-0.08053	0.692761	UBP16

0.947881443	0.034785673	0.020319939	0.011158	-0.22082	0.527583	RRP5
0.948254707	0.885372644	0.131133458	-0.00889	-0.02195	-0.30465	CDC73
0.948254707	0.621372579	0.251462984	-0.01237	0.075468	-0.35297	PET8
0.948254707	0.858991565	0.794208498	0.009801	-0.02787	-0.11098	YPT32
0.948254707	0.936226276	0.91703897	0.014471	0.024586	-0.10099	CTP1
0.948922756	0.963282012	0.571709772	0.015065	0.01973	-0.27752	NDL1
0.948971244	1	0.929303164	-0.01106	-0.00247	-0.0713	TOR2
0.950188921	0.621834431	0.841416921	-0.03737	-0.23471	-0.38079	OST2
0.950475148	0.773888085	0.335356848	0.01857	0.075062	-0.48294	YDL009C
0.950728639	0.019107411	0.591346544	0.114722	2.912122	2.337239	None
0.950728639	0.737846426	0.847404354	-0.13213	0.612538	1.288229	RRT15
0.951630564	0.289240166	0.352630353	-0.01287	0.151732	-0.33026	PHO81
0.95180053	1	0.87592683	0.028093	-0.01574	-0.25001	YCR025C
0.951817275	0.946594159	0.125375403	0.149946	0.241251	5.828699	None
0.951861514	0.949363945	0.710353715	0.010584	0.016607	0.157713	EFM4
0.951918369	0.213761989	1	0.008756	-0.11763	0.011543	RCK2
0.953519621	0.775390182	0.215066762	0.017797	0.074099	-0.56951	YDL183C
0.953822131	0.290500262	0.152940749	-0.01001	-0.12128	0.356	POL5
0.953890223	1	0.960632458	-0.08531	0.0251	0.483376	None
0.9540342	0.237677178	0.165340833	0.008885	0.117305	0.311271	RPS17B
0.954066589	0.425297662	0.88087787	0.007898	-0.07576	0.070987	NOP56
0.954801274	0.942368879	0.764689351	0.052315	0.090167	-0.70029	YGL034C
0.955724012	0.501748806	0.875200775	0.01063	0.089566	0.099829	TRS33
0.955724012	0.09702616	1	-0.00968	-0.17229	0.004857	ASM4
0.956373628	0.284831522	0.635840497	-0.01053	0.132761	0.192165	SEC11
0.956597089	0.164247051	0.393317291	-0.00806	0.124776	0.209538	PRX1
0.956821269	0.933553067	0.976159573	-0.01117	0.021743	-0.05665	GEF1
0.956882569	0.448302145	0.205616642	-0.00919	0.087076	-0.31116	MSC3
0.95772276	0.693571609	1	0.009952	-0.05664	0.000847	COP1
0.95778516	0.000356469	0.358747602	0.110091	3.651899	3.281657	None
0.958588904	0.005377068	0.98500242	-0.01139	0.323022	-0.05214	MRPL25
0.958727913	0.277433134	0.336735207	0.091994	1.212702	2.780033	None
0.959147177	0.36194188	0.291391691	-0.00934	0.106944	0.286996	CTR2
0.959208535	0.813026602	0.660358366	0.013726	0.054083	-0.24945	CIN10
0.960783566	0.712618958	0.480443903	0.084832	0.46931	2.042264	None
0.961286479	0.080514417	0.976378097	0.012086	-0.24126	0.064499	TOS2
0.962559904	0.019388398	0.026915259	-0.00774	-0.20016	0.421851	RIX7
0.962641118	0.21006721	0.064361732	-0.00885	0.135515	0.415622	RPL24B
0.962832897	0.430199039	0.335356848	-0.00897	0.095164	0.273109	COF1
0.962832897	0.539290348	0.55529834	-0.0072	0.062351	0.1634	SNC2
0.962832897	0.032604415	0.822329423	-0.00699	-0.16853	-0.08722	NUP57
0.962832897	0.98811108	0.848611069	0.014523	-0.01533	0.16541	None
0.962832897	0.223628637	0.889649609	0.008215	-0.12514	0.079714	PRP8
0.962832897	0.633674911	0.919516515	-0.01122	0.079562	0.092632	MSS2
0.962832897	0.243955921	0.996250082	0.008001	-0.11794	-0.0351	CAB2
0.964087023	0.422329315	0.110596652	-0.00803	0.08783	0.350575	RPS19B
0.964477636	0.604782612	1	0.013359	0.103956	0.009098	YOR111W
0.967075253	0.104382267	0.053017966	-0.00885	0.177689	0.459803	CCW12
0.967930544	0.974684047	0.556682748	0.015691	-0.02257	-0.37536	PEX15
0.967988867	0.032152547	0.283510967	-0.00706	0.18199	0.246713	MRPL35
0.967988867	0.887204289	0.739091337	0.006819	0.02131	-0.11382	RIM101
0.968043822	0.782040669	0.675803696	-0.00913	-0.04491	0.180517	TRM9
0.969377574	0.428439515	1	0.009125	-0.10591	0.033536	MKK2
0.969591971	0.241759995	1	0.008902	-0.14343	0.028672	YKL023C-[illegible]

0.970565528	0.97156603	0.469423001	0.134171	0.207395	-3.7493	None
0.970682439	0.97156603	0.469423001	0.133478	0.208033	-3.74798	None
0.971558403	0.972056407	0.357626604	0.134184	0.207073	4.196363	None
0.97201705	0.972383608	0.35748349	0.132601	0.205518	4.199306	None
0.972448143	0.095674919	0.115693287	-0.00609	-0.13575	0.293803	KRE33
0.973379178	0.146334441	0.179756965	0.006582	-0.13261	0.289916	NCL1
0.973379178	0.592522963	0.7456439	-0.0064	-0.05754	0.113262	CKA1
0.973821113	0.942225336	0.611494739	-0.02134	-0.04743	-0.51149	NKP2
0.973821113	0.001891522	0.644321253	-0.00842	-0.32238	0.191927	NUP1
0.974766905	0.368160688	0.060851274	-0.00705	0.098799	0.397708	RPL23A
0.974766905	0.868862376	0.831867858	-0.00681	0.026054	-0.09791	PPH22
0.974919173	0.984533249	0.859769494	0.018127	0.025137	0.236075	YPR170W-I
0.977396327	0.384508616	0.379660556	-0.01683	0.236482	-0.58712	SAP4
0.977396327	0.162566905	0.477260495	0.007077	-0.1464	0.219604	SNU114
0.978812858	0.345520596	0.712529354	-0.00739	-0.11401	0.15569	ELP3
0.978907715	0.865003017	0.87163978	-0.01504	-0.06226	0.196353	CIT3
0.981132555	0.929321848	0.782427464	0.009037	0.025302	0.163263	YNL234W
0.981544486	0.21659995	0.261118505	0.007535	-0.14987	0.331153	EFR3
0.982601562	0.700306657	0.662992545	-0.00995	-0.07881	-0.2481	SPO14
0.982872124	0.97156603	0.428049321	-0.00745	0.01376	0.266334	CAF20
0.98300135	0.332602552	0.90086308	-0.02173	0.36427	0.265405	YFL041W-A
0.983080155	1	0.255950109	0.043248	0.026181	1.981355	None
0.984376987	0.387232715	0.721913447	-0.01726	-0.27038	-0.38553	None
0.986161918	0.050398477	0.892830092	0.006386	-0.20035	-0.08491	ACF2
0.986193588	0.00355233	1	-0.00706	-0.31081	-0.01647	PCF11
0.987511239	0.159387719	0.043101043	-0.00562	0.136048	0.412194	RPL26B
0.987850736	0.835898334	0.868996053	-0.01004	-0.05468	-0.15319	CDC6
0.98805317	0.982681213	0.676572746	0.010909	0.018948	0.290842	RRN10
0.988352753	0.151076423	0.971609311	-0.02439	-0.62352	0.207133	None
0.988687602	1	0.968138917	-0.00443	-0.00161	0.038665	PMT1
0.990673232	0.208186424	0.931568877	0.005329	-0.12542	-0.06	BEM1
0.991003557	0.167354676	0.353690868	-0.00984	-0.25177	-0.44593	GEM1
0.991003557	0.446288659	1	0.005487	0.086211	-0.00289	VTA1
0.991268244	0.397361658	0.60698347	0.007558	0.130831	-0.24136	FLR1
0.992853766	0.584277189	0.586097635	0.008187	0.10269	-0.27821	YJR149W
0.992853766	0.389906374	0.868427687	-0.01048	-0.18997	-0.1777	YNL146W
0.99337691	0.412262997	1	0.017244	0.301221	-0.10325	YHR130C
0.993398638	0.321710164	0.879698394	0.007391	-0.15313	0.11963	NEO1
0.99461064	0.583819577	0.697639077	-0.00419	-0.05463	0.121863	ECM33
0.996134332	0.17643013	0.276489336	0.055863	-1.71563	3.77198	None
0.996775025	0.384709115	1	0.00656	-0.12944	-0.04433	PRP28
0.997450618	0.319000663	0.639357258	0.011755	0.26293	-0.40049	ARR3
0.997897089	0.4952565	0.821241649	-0.00549	-0.0902	-0.12067	TFB4
0.998182093	0.518133314	0.775460909	0.023615	0.370901	0.592368	ECM8
0.998191836	0.563914752	0.740905504	0.005477	0.080144	-0.15065	BET2
0.99920119	0.026595764	0.888649502	0.005086	-0.22968	0.089764	NUP100
0.99920119	0.904357451	1	0.033763	0.159174	0.214195	None
1	0.010158362	0.016453992	0.001019	-0.21918	0.45282	UTP8
1	0.781683071	0.048861894	0.00237	0.074314	-0.80515	NBL1
1	0.036499632	0.103916575	0.002335	-0.15863	0.290276	KRI1
1	0.354644058	0.179756965	-0.00024	0.103161	0.324357	RPL13B
1	0.21877703	0.180992827	0.001805	-0.11797	0.294541	YMR310C
1	0.820787111	0.189220839	0.01104	-0.08933	-0.83308	PAU17
1	0.054181243	0.207271346	0.003171	0.152607	0.253893	RHO1

1	0.558891569	0.209370481	0.006746	-0.11351	-0.49348	YKU80
1	0.435407492	0.222234824	-0.00368	-0.09008	-0.30568	ANB1
1	0.999194287	0.223414237	-0.00076	-0.00822	0.365912	IST2
1	0.623266105	0.287493512	0.00412	0.062627	-0.27825	ADP1
1	1	0.334558308	0.002317	0.001062	-0.23289	UTR1
1	0.124795835	0.336735207	-0.00097	-0.18404	-0.30418	NUD1
1	0.805737746	0.354608469	0.000343	-0.03056	0.214865	YCR016W
1	0.146651273	0.373162993	-0.00111	0.233901	0.389716	TRX3
1	0.439313465	0.411534311	0.003806	-0.08453	-0.2222	NAM8
1	0.944968744	0.41415419	-0.00494	0.017888	-0.26088	SAM50
1	0.442222692	0.448510963	0.002735	0.081598	0.206947	ARD1
1	0.302233138	0.481039634	-0.00536	-0.13488	-0.2549	NUP42
1	0.308657113	0.498094313	-0.00493	0.158852	-0.29634	BUB2
1	0.452827892	0.50705979	0.002164	0.09792	0.233767	MMS2
1	0.667040818	0.507578381	0.066585	-0.8973	3.148006	None
1	0.722806559	0.519000409	0.003712	-0.04392	-0.18511	HRR25
1	0.220446608	0.524876758	-0.00425	0.140175	-0.21776	RAD30
1	0.239357669	0.553808105	-0.0001	-0.11353	0.17406	LIA1
1	0.454132404	0.587251805	-0.00487	-0.15889	0.335959	YJR120W
1	0.027985534	0.600468279	-0.00216	-0.2615	-0.22112	SET2
1	0.984004572	0.62830703	0.000188	-0.0098	-0.16624	FUI1
1	1	0.648620695	0.018465	-0.03941	-1.16703	MAM1
1	0.903977193	0.651764436	0.001567	0.031571	0.233673	GIM5
1	0.66730791	0.655315289	0.053147	-0.8982	-2.65804	None
1	0.124374834	0.65746379	-0.00086	-0.1399	0.146596	DIS3
1	0.977693292	0.664126703	0.002374	0.010858	0.158559	DOM34
1	0.953675948	0.667844098	-0.00167	-0.01113	-0.12115	ARP2
1	0.758162333	0.684923409	0.001227	-0.03812	0.136994	ARI1
1	0.289051446	0.689558052	0.000895	0.149824	-0.19664	IBA57
1	0.788420725	0.689558052	-0.00363	0.030313	0.120947	TYS1
1	0.254444296	0.710200613	-0.00674	0.283193	0.335571	YOL097W-A
1	0.083612522	0.721271027	0.003836	-0.16511	0.134392	NMD2
1	0.77606377	0.721271027	0.00088	0.177577	0.623497	SPR28
1	0.918227238	0.741851259	0.006077	-0.05801	-0.38199	VEL1
1	0.75339767	0.764340274	0.002312	-0.05123	0.146721	YML082W
1	0.518133314	0.772281426	0.015276	-0.26745	0.428963	PGA2
1	0.996753756	0.775856108	0.007653	0.013945	-0.22798	AVO2
1	1	0.781154036	0.000681	-0.0006	-0.14032	TGL3
1	0.583819577	0.785474844	0.010322	0.365988	-0.64421	AHT1
1	0.598147339	0.786892522	0.005742	0.15576	-0.27888	GAL3
1	0.024106319	0.789517744	-0.0032	-0.26954	-0.146	RTT107
1	0.028292556	0.791580335	-0.00499	-0.26325	-0.14512	ELM1
1	1	0.794208498	-0.00974	-0.00549	-0.27533	PCI8
1	0.765667428	0.862693719	-0.0023	0.044818	0.100174	FRQ1
1	0.354576312	0.868996053	-0.01076	0.251076	-0.2177	ECM18
1	0.443819937	0.886187413	0.000718	-0.12119	0.115775	BET5
1	0.743945088	0.89043028	-0.00289	-0.0673	-0.12126	YGL108C
1	0.224402423	0.917145138	-0.00279	-0.17351	0.095752	BRR2
1	0.162566905	0.93741782	-0.00393	-0.18774	0.079563	POL3
1	0.456532916	0.95255919	-0.00216	0.120613	0.07916	RCN1
1	0.380565951	0.960655888	0.002158	-0.13043	0.070883	AHK1
1	0.640053214	0.96218812	-0.00181	-0.09581	-0.08554	IML1
1	0.176418414	0.972387712	-0.00482	0.163561	0.056025	YIP1
1	0.627135065	0.979005513	0.00049	-0.06119	-0.04422	YBR220C

1	0.600387037	0.981710293	0.001967	0.067082	-0.04462	HIF1
1	0.50670267	0.981786205	0.002205	-0.05787	0.032175	CCT3
1	0.048103529	0.98500242	-0.00038	0.18302	-0.03935	POM33
1	0.304074798	0.992699715	0.003389	0.093812	-0.03215	TIM54
1	0.524017731	0.994442854	0.003071	-0.06204	-0.03119	CYS3
1	0.005227243	1	0.010642	0.63626	0.040982	REC114
1	0.040742569	1	-0.04825	3.19909	-0.05546	None
1	0.063730754	1	-0.0051	-0.22294	0.007916	RDH54
1	0.138242749	1	-0.06139	-2.64884	0.069338	None
1	0.138634396	1	-0.05054	-2.6442	0.05709	None
1	0.289051446	1	0.001723	0.116396	0.014305	SCS22
1	0.332602552	1	-0.00309	-0.14169	0.046204	LAM1
1	0.33527643	1	-0.03714	-1.85985	0.04526	YDR194W-A
1	0.33527643	1	-0.03715	-1.85985	0.045266	None
1	0.335509445	1	-0.03764	-1.85649	0.045878	None
1	0.335509445	1	-0.04127	-1.85771	0.050289	None
1	0.335511479	1	-0.03871	-1.85587	0.047185	None
1	0.340284679	1	0.002144	0.121662	0.002482	GRX5
1	0.373617533	1	0.004708	0.185979	-0.05882	CPD1
1	0.500508325	1	-0.00489	0.096407	-0.00855	YMC1
1	0.581422067	1	0.000707	0.195239	0.084663	YMR007W
1	0.672577856	1	0.015007	-0.23254	-0.05885	None
1	0.716190064	1	0.00248	0.055836	-0.01557	PEX12
1	0.759570065	1	0.003043	0.06663	0.021698	TPO5
1	0.887439198	1	0.002732	0.023226	-0.00867	EAF3
1	1	1	4.21E-17	-3.3E-17	0	
1	1	1	4.21E-17	-3.3E-17	0	
1	1	1	4.21E-17	-3.3E-17	0	
1	1	1	4.21E-17	-3.3E-17	0	
1	1	1	4.21E-17	-3.3E-17	0	
1	1	1	4.21E-17	-3.3E-17	0	
1	1	1	4.21E-17	-3.3E-17	0	
1	1	1	4.21E-17	-3.3E-17	0	
1	1	1	4.21E-17	-3.3E-17	0	
1	1	1	4.21E-17	-3.3E-17	0	
1	1	1	4.21E-17	-3.3E-17	0	
1	1	1	4.21E-17	-3.3E-17	0	
1	1	1	4.21E-17	-3.3E-17	0	
1	1	1	4.21E-17	-3.3E-17	0	
1	1	1	4.21E-17	-3.3E-17	0	
1	1	1	4.21E-17	-3.3E-17	0	
1	1	1	4.21E-17	-3.3E-17	0	
1	1	1	4.21E-17	-3.3E-17	0	
1	1	1	4.21E-17	-3.3E-17	0	
1	1	1	4.21E-17	-3.3E-17	0	
1	1	1	4.21E-17	-3.3E-17	0	
1	1	1	4.21E-17	-3.3E-17	0	
1	1	1	4.21E-17	-3.3E-17	0	
1	1	1	4.21E-17	-3.3E-17	0	
1	1	1	4.21E-17	-3.3E-17	0	
1	1	1	4.21E-17	-3.3E-17	0	
1	1	1	4.21E-17	-3.3E-17	0	
1	1	1	4.21E-17	-3.3E-17	0	
1	1	1	4.21E-17	-3.3E-17	0	
1	1	1	4.21E-17	-3.3E-17	0	

1	1	1	4.21E-17	-3.3E-17	0
1	1	1	4.21E-17	-3.3E-17	0
1	1	1	4.21E-17	-3.3E-17	0
1	1	1	4.21E-17	-3.3E-17	0
1	1	1	4.21E-17	-3.3E-17	0
1	1	1	4.21E-17	-3.3E-17	0
1	1	1	4.21E-17	-3.3E-17	0
1	1	1	4.21E-17	-3.3E-17	0
1	1	1	4.21E-17	-3.3E-17	0
1	1	1	4.21E-17	-3.3E-17	0
1	1	1	4.21E-17	-3.3E-17	0
1	1	1	4.21E-17	-3.3E-17	0
1	1	1	4.21E-17	-3.3E-17	0
1	1	1	4.21E-17	-3.3E-17	0
1	1	1	4.21E-17	-3.3E-17	0
1	1	1	4.21E-17	-3.3E-17	0
1	1	1	4.21E-17	-3.3E-17	0
1	1	1	4.21E-17	-3.3E-17	0
1	1	1	4.21E-17	-3.3E-17	0
1	1	1	4.21E-17	-3.3E-17	0
1	1	1	4.21E-17	-3.3E-17	0
1	1	1	4.21E-17	-3.3E-17	0
1	1	1	4.21E-17	-3.3E-17	0
1	1	1	4.21E-17	-3.3E-17	0
1	1	1	4.21E-17	-3.3E-17	0
1	1	1	4.21E-17	-3.3E-17	0
1	1	1	4.21E-17	-3.3E-17	0
1	1	1	4.21E-17	-3.3E-17	0
1	1	1	4.21E-17	-3.3E-17	0
1	1	1	4.21E-17	-3.3E-17	0
1	1	1	4.21E-17	-3.3E-17	0
1	1	1	4.21E-17	-3.3E-17	0
1	1	1	4.21E-17	-3.3E-17	0
1	1	1	4.21E-17	-3.3E-17	0
1	1	1	4.21E-17	-3.3E-17	0
1	1	1	4.21E-17	-3.3E-17	0
1	1	1	4.21E-17	-3.3E-17	0
1	1	1	4.21E-17	-3.3E-17	0
1	1	1	4.21E-17	-3.3E-17	0
1	1	1	4.21E-17	-3.3E-17	0
1	1	1	4.21E-17	-3.3E-17	0
1	1	1	4.21E-17	-3.3E-17	0
1	1	1	4.21E-17	-3.3E-17	0
1	1	1	4.21E-17	-3.3E-17	0
1	1	1	4.21E-17	-3.3E-17	0
1	1	1	4.21E-17	-3.3E-17	0
1	1	1	4.21E-17	-3.3E-17	0
1	1	1	4.21E-17	-3.3E-17	0
1	1	1	4.21E-17	-3.3E-17	0
1	1	1	4.21E-17	-3.3E-17	0
1	1	1	4.21E-17	-3.3E-17	0
1	1	1	4.21E-17	-3.3E-17	0
1	1	1	4.21E-17	-3.3E-17	0
1	1	1	4.21E-17	-3.3E-17	0

1	1	1	4.21E-17	-3.3E-17	0
1	1	1	4.21E-17	-3.3E-17	0
1	1	1	4.21E-17	-3.3E-17	0
1	1	1	4.21E-17	-3.3E-17	0
1	1	1	4.21E-17	-3.3E-17	0
1	1	1	4.21E-17	-3.3E-17	0
1	1	1	4.21E-17	-3.3E-17	0
1	1	1	4.21E-17	-3.3E-17	0
1	1	1	4.21E-17	-3.3E-17	0
1	1	1	4.21E-17	-3.3E-17	0
1	1	1	4.21E-17	-3.3E-17	0
1	1	1	4.21E-17	-3.3E-17	0
1	1	1	4.21E-17	-3.3E-17	0
1	1	1	4.21E-17	-3.3E-17	0
1	1	1	4.21E-17	-3.3E-17	0
1	1	1	4.21E-17	-3.3E-17	0
1	1	1	4.21E-17	-3.3E-17	0
1	1	1	4.21E-17	-3.3E-17	0
1	1	1	4.21E-17	-3.3E-17	0
1	1	1	4.21E-17	-3.3E-17	0
1	1	1	4.21E-17	-3.3E-17	0
1	1	1	4.21E-17	-3.3E-17	0
1	1	1	4.21E-17	-3.3E-17	0
1	1	1	4.21E-17	-3.3E-17	0
1	1	1	4.21E-17	-3.3E-17	0
1	1	1	4.21E-17	-3.3E-17	0
1	1	1	4.21E-17	-3.3E-17	0
1	1	1	4.21E-17	-3.3E-17	0
1	1	1	4.21E-17	-3.3E-17	0
1	1	1	4.21E-17	-3.3E-17	0
1	1	1	4.21E-17	-3.3E-17	0
1	1	1	4.21E-17	-3.3E-17	0
1	1	1	4.21E-17	-3.3E-17	0
1	1	1	4.21E-17	-3.3E-17	0
1	1	1	4.21E-17	-3.3E-17	0
1	1	1	4.21E-17	-3.3E-17	0
1	1	1	4.21E-17	-3.3E-17	0
1	1	1	4.21E-17	-3.3E-17	0
1	1	1	4.21E-17	-3.3E-17	0
1	1	1	4.21E-17	-3.3E-17	0
1	1	1	4.21E-17	-3.3E-17	0
1	1	1	4.21E-17	-3.3E-17	0
1	1	1	4.21E-17	-3.3E-17	0
1	1	1	4.21E-17	-3.3E-17	0
1	1	1	4.21E-17	-3.3E-17	0
1	1	1	4.21E-17	-3.3E-17	0
1	1	1	4.21E-17	-3.3E-17	0
1	1	1	4.21E-17	-3.3E-17	0
1	1	1	4.21E-17	-3.3E-17	0
1	1	1	4.21E-17	-3.3E-17	0
1	1	1	4.21E-17	-3.3E-17	0
1	1	1	4.21E-17	-3.3E-17	0
1	1	1	4.21E-17	-3.3E-17	0
1	1	1	4.21E-17	-3.3E-17	0

1	1	1	4.21E-17	-3.3E-17	0
1	1	1	4.21E-17	-3.3E-17	0
1	1	1	4.21E-17	-3.3E-17	0
1	1	1	4.21E-17	-3.3E-17	0
1	1	1	4.21E-17	-3.3E-17	0
1	1	1	4.21E-17	-3.3E-17	0
1	1	1	4.21E-17	-3.3E-17	0
1	1	1	4.21E-17	-3.3E-17	0
1	1	1	4.21E-17	-3.3E-17	0
1	1	1	4.21E-17	-3.3E-17	0
1	1	1	4.21E-17	-3.3E-17	0
1	1	1	4.21E-17	-3.3E-17	0
1	1	1	4.21E-17	-3.3E-17	0
1	1	1	4.21E-17	-3.3E-17	0
1	1	1	4.21E-17	-3.3E-17	0
1	1	1	4.21E-17	-3.3E-17	0
1	1	1	4.21E-17	-3.3E-17	0
1	1	1	4.21E-17	-3.3E-17	0
1	1	1	4.21E-17	-3.3E-17	0
1	1	1	4.21E-17	-3.3E-17	0
1	1	1	4.21E-17	-3.3E-17	0
1	1	1	4.21E-17	-3.3E-17	0
1	1	1	4.21E-17	-3.3E-17	0
1	1	1	4.21E-17	-3.3E-17	0
1	1	1	4.21E-17	-3.3E-17	0
1	1	1	4.21E-17	-3.3E-17	0
1	1	1	4.21E-17	-3.3E-17	0
1	1	1	4.21E-17	-3.3E-17	0
1	1	1	4.21E-17	-3.3E-17	0
1	1	1	4.21E-17	-3.3E-17	0
1	1	1	4.21E-17	-3.3E-17	0
1	1	1	4.21E-17	-3.3E-17	0
1	1	1	4.21E-17	-3.3E-17	0
1	1	1	4.21E-17	-3.3E-17	0
1	1	1	4.21E-17	-3.3E-17	0
1	1	1	4.21E-17	-3.3E-17	0
1	1	1	4.21E-17	-3.3E-17	0
1	1	1	4.21E-17	-3.3E-17	0
1	1	1	4.21E-17	-3.3E-17	0
1	1	1	4.21E-17	-3.3E-17	0
1	1	1	4.21E-17	-3.3E-17	0
1	1	1	4.21E-17	-3.3E-17	0
1	1	1	4.21E-17	-3.3E-17	0
1	1	1	4.21E-17	-3.3E-17	0
1	1	1	4.21E-17	-3.3E-17	0
1	1	1	4.21E-17	-3.3E-17	0
1	1	1	4.21E-17	-3.3E-17	0
1	1	1	4.21E-17	-3.3E-17	0
1	1	1	4.21E-17	-3.3E-17	0
1	1	1	4.21E-17	-3.3E-17	0
1	1	1	4.21E-17	-3.3E-17	0
1	1	1	4.21E-17	-3.3E-17	0
1	1	1	4.21E-17	-3.3E-17	0
1	1	1	4.21E-17	-3.3E-17	0

1	1	1	4.21E-17	-3.3E-17	0
1	1	1	4.21E-17	-3.3E-17	0
1	1	1	4.21E-17	-3.3E-17	0
1	1	1	4.21E-17	-3.3E-17	0
1	1	1	4.21E-17	-3.3E-17	0
1	1	1	4.21E-17	-3.3E-17	0
1	1	1	4.21E-17	-3.3E-17	0
1	1	1	4.21E-17	-3.3E-17	0
1	1	1	4.21E-17	-3.3E-17	0
1	1	1	4.21E-17	-3.3E-17	0
1	1	1	4.21E-17	-3.3E-17	0
1	1	1	4.21E-17	-3.3E-17	0
1	1	1	4.21E-17	-3.3E-17	0
1	1	1	4.21E-17	-3.3E-17	0
1	1	1	4.21E-17	-3.3E-17	0
1	1	1	4.21E-17	-3.3E-17	0
1	1	1	4.21E-17	-3.3E-17	0
1	1	1	4.21E-17	-3.3E-17	0
1	1	1	4.21E-17	-3.3E-17	0
1	1	1	4.21E-17	-3.3E-17	0
1	1	1	4.21E-17	-3.3E-17	0
1	1	1	4.21E-17	-3.3E-17	0
1	1	1	4.21E-17	-3.3E-17	0
1	1	1	4.21E-17	-3.3E-17	0
1	1	1	4.21E-17	-3.3E-17	0
1	1	1	4.21E-17	-3.3E-17	0
1	1	1	4.21E-17	-3.3E-17	0
1	1	1	4.21E-17	-3.3E-17	0
1	1	1	4.21E-17	-3.3E-17	0
1	1	1	4.21E-17	-3.3E-17	0
1	1	1	4.21E-17	-3.3E-17	0
1	1	1	4.21E-17	-3.3E-17	0
1	1	1	4.21E-17	-3.3E-17	0
1	1	1	4.21E-17	-3.3E-17	0
1	1	1	4.21E-17	-3.3E-17	0
1	1	1	4.21E-17	-3.3E-17	0
1	1	1	4.21E-17	-3.3E-17	0
1	1	1	4.21E-17	-3.3E-17	0
1	1	1	4.21E-17	-3.3E-17	0
1	1	1	4.21E-17	-3.3E-17	0
1	1	1	4.21E-17	-3.3E-17	0
1	1	1	4.21E-17	-3.3E-17	0
1	1	1	4.21E-17	-3.3E-17	0
1	1	1	4.21E-17	-3.3E-17	0
1	1	1	4.21E-17	-3.3E-17	0
1	1	1	4.21E-17	-3.3E-17	0
1	1	1	4.21E-17	-3.3E-17	0
1	1	1	4.21E-17	-3.3E-17	0
1	1	1	4.21E-17	-3.3E-17	0
1	1	1	4.21E-17	-3.3E-17	0
1	1	1	4.21E-17	-3.3E-17	0
1	1	1	4.21E-17	-3.3E-17	0
1	1	1	4.21E-17	-3.3E-17	0
1	1	1	4.21E-17	-3.3E-17	0

1	1	1	4.21E-17	-3.3E-17	0
1	1	1	4.21E-17	-3.3E-17	0
1	1	1	4.21E-17	-3.3E-17	0
1	1	1	4.21E-17	-3.3E-17	0
1	1	1	4.21E-17	-3.3E-17	0
1	1	1	4.21E-17	-3.3E-17	0
1	1	1	4.21E-17	-3.3E-17	0
1	1	1	4.21E-17	-3.3E-17	0
1	1	1	4.21E-17	-3.3E-17	0
1	1	1	4.21E-17	-3.3E-17	0
1	1	1	4.21E-17	-3.3E-17	0
1	1	1	4.21E-17	-3.3E-17	0
1	1	1	4.21E-17	-3.3E-17	0
1	1	1	4.21E-17	-3.3E-17	0
1	1	1	4.21E-17	-3.3E-17	0
1	1	1	4.21E-17	-3.3E-17	0

Descriptio	Gene Sum	Biological	Cellular Cc	Molecular	KEGG Pathway
Fit3p	Mannoprot	GO:00158	GO:00092	GO:0003674	molecular_function
Fit2p	Mannoprot	GO:00158	GO:00092	GO:0003674	molecular_function
Ehd3p	3-hydroxy	GO:00090	GO:00057	GO:00038	(sce00410)beta-Alanine metabolism; (sce00640)
Flo11p	GPI-anch	GO:00440	GO:00059	GO:00036	(sce04011)MAPK signaling pathway - yeast
hypotheti	Putative	GO:00081	GO:00160	GO:0003674	molecular_function
formate (	NAD(+)-d	GO:00421	GO:00058	GO:00088	(sce00630)Glyoxylate and dicarboxylate metab
ATP-bindi	Plasma m	GO:00007	GO:00433	GO:00154	(sce02010)ABC transporters
Mf(alpha)	Mating p	GO:00007	GO:00003	GO:00007	(sce04011)MAPK signaling pathway - yeast
mating p	Mating p	GO:00007	GO:00057	GO:00007	(sce04011)MAPK signaling pathway - yeast
elongati	Elongati	GO:00459	GO:19909	GO:0003746	translation elongation factor activity;GO:
floccul	Lectin-l	GO:00005	GO:00092	GO:0005537	mannose binding;GO:0048029 monosaccharide
Flo10p	Member o	GO:00005	GO:00092	GO:0005537	mannose binding;GO:0048029 monosaccharide
sideroph	Transpor	GO:00447	GO:00003	GO:0015343	siderophore transmembrane transporter acti
Cos5p	Endosoma	GO:00433	GO:00003	GO:0003674	molecular_function
Aga2p	Adhesion	GO:00007	GO:00092	GO:0050839	cell adhesion molecule binding;GO:0005515
alpha-fa	Receptor	GO:00007	GO:00380	GO:00049	(sce04011)MAPK signaling pathway - yeast
alpha-gl	High-aff	GO:00157	GO:00058	GO:0005364	maltose:proton symporter activity;GO:00053
Enb1p	Endosoma	GO:00156	GO:00331	GO:0015620	ferric-enterobactin transmembrane transpor
aspartyl	Aspartyl	GO:00007	GO:00092	GO:00041	(sce04011)MAPK signaling pathway - yeast
flavohem	Nitric o	GO:00462	GO:00104	GO:0016966	nitric oxide reductase activity;GO:0008941
Ste3p	Receptor	GO:00007	GO:00058	GO:00049	(sce04011)MAPK signaling pathway - yeast
Sag1p	Alpha-ag	GO:00007	GO:00092	GO:0050839	cell adhesion molecule binding;GO:0005515
hypotheti	Putative	GO:00081	GO:00055	GO:0003674	molecular_function
Afb1p	MATalpha-	GO:00007	GO:00092	GO:0003674	molecular_function
phosphoe	Phosphoe	GO:00060	GO:00058	GO:00046	(M00003)Gluconeogenesis, oxaloacetate => fru
hypotheti	Protein (	GO:00081	GO:00056	GO:0003674	molecular_function
Cos4p	Endosoma	GO:00433	GO:00003	GO:0003674	molecular_function
lysine--	Lysyl-tR	GO:00064	GO:00058	GO:00048	(sce00970)Aminoacyl-tRNA biosynthesis
hypotheti	Putative	GO:00081	GO:00055	GO:0003674	molecular_function
pyridoxi	Member o	GO:00086	GO:00055	GO:00363	(sce00750)Vitamin B6 metabolism
hypotheti	Protein (	GO:00081	GO:00160	GO:0003674	molecular_function
thiamine	Plasma m	GO:19030	GO:00058	GO:1903089	5-amino-1-ribofuranosylimidazole-4-carboxa
Mf(alpha)	Mating p	GO:00007	GO:00055	GO:00007	(sce04011)MAPK signaling pathway - yeast
mating p	Mating p	GO:00007	GO:00057	GO:00007	(sce04011)MAPK signaling pathway - yeast
oligo-1,(	Major is	GO:00000	GO:00058	GO:00045	(sce00052)Galactose metabolism; (sce00500)St
hypotheti	Fungal-s	GO:00081	GO:00057	GO:0003674	molecular_function
Hmra1p	Silenced	GO:00075	GO:00056	GO:00037	(sce04113)Meiosis - yeast
phenylpy	Phenylpy	GO:00000	GO:00058	GO:00505	(sce00360)Phenylalanine metabolism; (sce0110
acetyltr	Putative	GO:00081	GO:00055	GO:0008870	galactoside O-acetyltransferase activity;G
transcri	MAL-acti	GO:00000	GO:00056	GO:0000981	DNA-binding transcription factor activity,
putative	Protein (	GO:00345	GO:00056	GO:0016657	oxidoreductase activity, acting on NAD(P)H
Pgu1p	Endo-pol	GO:00454	GO:00055	GO:00046	(sce00040)Pentose and glucuronate interconve
pyridoxi	Member o	GO:00086	GO:00055	GO:00363	(sce00750)Vitamin B6 metabolism
Aqy1p	Spore-sp	GO:00304	GO:00057	GO:0015250	water channel activity;GO:0005372 water tr
Ubx6p	UBX (ubi	GO:00304	GO:00056	GO:0003674	molecular_function
elongati	Elongati	GO:00459	GO:19909	GO:0003746	translation elongation factor activity;GO:
high-aff	High-aff	GO:00156	GO:00306	GO:0005375	copper ion transmembrane transporter activ
Cos6p	Endosoma	GO:00433	GO:00003	GO:0003674	molecular_function
cysteine-	Beta-lya	GO:00194	GO:00055	GO:00041	(sce00450)Selenocompound metabolism; (sce002
alcohol (	Glucose-	GO:00009	GO:00057	GO:00040	(sce00350)Tyrosine metabolism; (sce00071)Fat

putative Ferulic a GO:00462 GO:00057 GO:0016831 carboxy-lyase activity;GO:0016830 carbon-c
signal se Putative GO:00066 GO:00003 GO:0005048 signal sequence binding;GO:0042277 peptide
Y' elemen Helicase- GO:00081 GO:00055 GO:0004386 helicase activity;GO:0017111 nucleoside-tr
Rdt1p Short ope GO:00081 GO:00055 GO:0003674 molecular_function
transcrip MAL-activ GO:00000 GO:00056 GO:0000981 DNA-binding transcription factor activity,
putative Protein v GO:00991 GO:00058 GO:0008553 proton-exporting ATPase activity, phosphor
protein k Putative GO:00064 GO:00057 GO:0004674 protein serine/threonine kinase activity;G
chaperone Disaggreg GO:00013 GO:00723 GO:00435 (sce04213)Longevity regulating pathway - mul
Pho89p Plasma me GO:00354 GO:00058 GO:0015319 sodium:inorganic phosphate symporter activ
mRNA spli Core Sm p GO:00003 GO:00465 GO:19909 (sce03040)Spliceosome
bifunctio Bifunctio GO:00009 GO:00057 GO:00338 (sce00350)Tyrosine metabolism; (sce00071)Fat
Fmp45p Integral GO:00304 GO:00451 GO:0003674 molecular_function
ferroxida Ferro-O2- GO:00332 GO:00335 GO:0005381 iron ion transmembrane transporter activit
Cyb2p Cytochrom GO:00060 GO:00057 GO:00044 (sce00620)Pyruvate metabolism; (sce01100)Met
Tis11p mRNA-bind GO:00611 GO:00009 GO:0003730 mRNA 3'-UTR binding;GO:0003729 mRNA bindin
2-deoxygl 2-deoxygl GO:00060 GO:00056 GO:0003850 2-deoxyglucose-6-phosphatase activity;GO:0
phosphogl 6-phospho GO:00090 GO:00058 GO:00046 (M00006)Pentose phosphate pathway, oxidative
translati Translati GO:00064 GO:00058 GO:00190 (sce03013)RNA transport
translati Translati GO:00064 GO:00058 GO:00190 (sce03013)RNA transport
flocculin Lectin-li GO:00362 GO:00092 GO:0005537 mannose binding;GO:0048029 monosaccharide
maltose p Maltose p GO:00000 GO:00058 GO:0005352 alpha-glucoside:proton symporter activity;
Mrh1p Protein t GO:00342 GO:00059 GO:0005216 ion channel activity;GO:0022838 substrate-
Css3p Protein o GO:00081 GO:00055 GO:0003674 molecular_function
alpha-glu Maltase GO:00000 GO:00058 GO:00045 (sce00052)Galactose metabolism; (sce00500)St
Coa2p Cytochrom GO:00336 GO:00057 GO:0003674 molecular_function
putative Protein o GO:00092 GO:19036 GO:00363 (sce00750)Vitamin B6 metabolism
pheromone Pheromone GO:00081 GO:00059 GO:0003674 molecular_function
IMP dehyd Inosine m GO:00061 GO:00007 GO:00039 (sce)Guanine ribonucleotide biosynthesis IMP
Fmp16p Protein o GO:00081 GO:00057 GO:0003674 molecular_function
Hsp90 fam Hsp90 cha GO:00322 GO:00092 GO:00426 (sce04141)Protein processing in endoplasmic
glycogen Glycogen GO:00059 GO:00057 GO:00043 (sce00500)Starch and sucrose metabolism; (sc
Pns1p Protein o GO:00158 GO:00058 GO:0015220 choline transmembrane transporter activity
translati Translati GO:00064 GO:00162 GO:00040 (sce03013)RNA transport
heat shoc Small hea GO:00070 GO:00057 GO:00510 (sce04141)Protein processing in endoplasmic
ferric-ch Ferric re GO:00158 GO:00058 GO:0052851 ferric-chelate reductase (NADPH) activity;
cyanamide Cyanamide GO:00188 GO:00055 GO:00188 (sce00791)Atrazine degradation
hypotheti Putative GO:00081 GO:00055 GO:0003674 molecular_function
C-22 ster C-22 ster GO:00066 GO:00057 GO:00002 (M00102)Ergocalciferol biosynthesis; (sce)Er
medium-ch Acyl-coen GO:00517 GO:00055 GO:0034321 alcohol O-octanoyltransferase activity;GO:
Hsp30p Negative GO:00327 GO:00057 GO:0005216 ion channel activity;GO:0022838 substrate-
hypotheti Putative GO:00081 GO:00055 GO:0003674 molecular_function
Hsp70 fam ATPase in GO:00708 GO:00340 GO:00441 (sce03060)Protein export; (sce04141)Protein
Cox26p Stabilize GO:00081 GO:00057 GO:0003674 molecular_function
Tir4p Cell wall GO:00315 GO:00092 GO:0005199 structural constituent of cell wall;GO:000
hexokinas Hexokinas GO:00324 GO:00057 GO:00191 (M00549)Nucleotide sugar biosynthesis, gluco
alpha-glu Maltase GO:00000 GO:00058 GO:00045 (sce00052)Galactose metabolism; (sce00500)St
SUN famil Protein i GO:00315 GO:00092 GO:0003674 molecular_function
Pet10p Protein of unknown function that localizes to lipid particles; localization sugg
Css2p Protein o GO:00081 GO:00057 GO:0003674 molecular_function
translati Translati GO:00064 GO:00162 GO:00040 (sce03013)RNA transport
amino aci General a GO:00158 GO:00003 GO:0001761 beta-alanine transmembrane transporter act
hypotheti Dubious o GO:00081 GO:00055 GO:0003674 molecular_function
hexose tr High-affi GO:00157 GO:00058 GO:00151 (sce04113)Meiosis - yeast
histone a Tetrameri GO:00165 GO:00057 GO:00044 (sce00360)Phenylalanine metabolism

Ecm12p	Putative	GO:007155	GO:001602	GO:0003674	molecular_function
sterol-bi	Sterol bi	GO:001591	GO:000032	GO:0032934	sterol binding;GO:0005496 steroid binding;
sedohept	Transaldo	GO:000090	GO:000563	GO:00048	(M00007)Pentose phosphate pathway, non-oxida
hypotheti	Putative	GO:005508	GO:000032	GO:0042626	ATPase activity, coupled to transmembrane
putative	Protein c	GO:001934	GO:000563	GO:0004121	cystathionine beta-lyase activity;GO:00168
Hmx1p	ER locali	GO:000678	GO:000564	GO:0004392	heme oxygenase (decyclizing) activity;GO:0
hypotheti	Putative	GO:000815	GO:000557	GO:0003674	molecular_function
hypotheti	Putative	GO:003352	GO:000557	GO:0008898	S-adenosylmethionine-homocysteine S-methyl
Aqy2p	Water cha	GO:000683	GO:000578	GO:0015250	water channel activity;GO:0005372 water tr
Ygp1p	Cell wall	GO:000707	GO:000557	GO:0003674	molecular_function
pheromone	Pheromone	GO:000815	GO:001602	GO:0003674	molecular_function
lipid-bin	Plasma me	GO:007147	GO:000576	GO:0008289	lipid binding;GO:0005488 binding;GO:000367
cytochron	Cytochron	GO:000612	GO:000575	GO:00090	(sce00920)Sulfur metabolism; (sce01100)Metab
hypotheti	Putative	GO:000815	GO:000557	GO:0003674	molecular_function
putative	Putative	GO:001623	GO:000032	GO:0042626	ATPase activity, coupled to transmembrane
Atg34p	Receptor	GO:007121	GO:003427	GO:0030674	protein binding, bridging;GO:0060090 molec
hypotheti	Putative	GO:000815	GO:000557	GO:0003674	molecular_function
hypotheti	Putative	GO:000683	GO:000588	GO:0015250	water channel activity;GO:0005372 water tr
putative	Putative	GO:007149	GO:000927	GO:0005199	structural constituent of cell wall;GO:000
siderophe	Ferrioxan	GO:004471	GO:000032	GO:0015344	siderophore uptake transmembrane transport
hexose tr	High-affi	GO:001575	GO:000032	GO:00151	(sce04113)Meiosis - yeast
phosphate	High-affi	GO:190472	GO:000032	GO:0097079	selenite:proton symporter activity;GO:0005
cystathi	Peroxison	GO:007126	GO:000577	GO:00041	(sce00450)Selenocompound metabolism; (sce002
Yro2p	Protein w	GO:003422	GO:000593	GO:0005216	ion channel activity;GO:0022838 substrate-
hypotheti	Putative	GO:000815	GO:000032	GO:0003674	molecular_function
argininos	Arginosuc	GO:000005	GO:000582	GO:00040	(sce00220)Arginine biosynthesis; (sce00250)A
hexose tr	High-affi	GO:001576	GO:000588	GO:00155	(sce04113)Meiosis - yeast
Cyb5p	Cytochron	GO:001612	GO:000578	GO:0009055	electron transfer activity;GO:0020037 heme
alpha-1,2	Alpha-1,2	GO:004635	GO:000579	GO:00002	(sce00513)Various types of N-glycan biosynth
dihydroor	Dihydroor	GO:004420	GO:000563	GO:00041	(sce)Uridine monophosphate biosynthesis, glu
medium-ch	Acyl-coen	GO:005179	GO:000574	GO:0034319	alcohol O-butanoyltransferase activity;GO:
Cos1p	Endosomal	GO:004332	GO:000032	GO:0003674	molecular_function
None	None	None	None	None	None
C-5 stero	C-5 stero	GO:000669	GO:000578	GO:00002	(M00102)Ergocalciferol biosynthesis; (sce)Er
hypotheti	Putative	GO:003449	GO:000579	GO:0003924	GTPase activity;GO:0005525 GTP binding;GO:
amino aci	Amino aci	GO:001582	GO:000578	GO:0022893	low-affinity tryptophan transmembrane tran
Cos7p	Endosomal	GO:004332	GO:000032	GO:0038024	cargo receptor activity;GO:0003674 molecul
chaperone	Oligomeri	GO:004333	GO:000575	GO:0051787	misfolded protein binding;GO:0016887 ATPas
hypotheti	Putative	GO:000815	GO:000557	GO:0003674	molecular_function
Ego4p	Protein c	GO:000815	GO:000582	GO:0003674	molecular_function
acid phos	Constitut	GO:004272	GO:003028	GO:00039	(sce00740)Riboflavin metabolism; (sce00730)T
glyceral	Glyceral	GO:000609	GO:000927	GO:00043	(M00002)Glycolysis, core module involving th
cytochron	Subunit V	GO:000612	GO:000575	GO:00041	(sce00190)Oxidative phosphorylation; (sce011
phosphati	Phosphati	GO:000665	GO:000578	GO:00046	(M00091)Phosphatidylcholine (PC) biosynthesi
Nrg2p	Transcrip	GO:190046	GO:003151	GO:0000980	RNA polymerase II distal enhancer sequence
putative	Putative	GO:000608	GO:000557	GO:0018456	aryl-alcohol dehydrogenase (NAD+) activity
Gyp8p	GTPase-ac	GO:000688	GO:000578	GO:0017137	Rab GTPase binding;GO:0005096 GTPase activ
None	None	None	None	None	None
catalase	Cytosolic	GO:007147	GO:000577	GO:00040	(sce00380)Tryptophan metabolism; (sce00630)G
Pry3p	Cell wall	GO:001591	GO:000927	GO:0003674	molecular_function
hydroxyme	HMG-CoA r	GO:001928	GO:000577	GO:00044	(sce)C5 isoprenoid biosynthesis, mevalonate
hypotheti	Protein c	GO:005511	GO:000582	GO:0016491	oxidoreductase activity;GO:0003824 catalyt
aromatic-	Aromatic	GO:001950	GO:000563	GO:00801	(sce00360)Phenylalanine metabolism; (sce0035
beta-1,3-	O-glycosy	GO:003150	GO:000927	GO:0005199	structural constituent of cell wall;GO:000

Atg36p	Pex3p int	GO:00302	GO:00057	GO:0003674	molecular_function
hypotheti	Putative	GO:00081	GO:00160	GO:0003674	molecular_function
methionin	S-adenosy	GO:00065	GO:00104	GO:00044	(sce)Methionine salvage pathway; (M00034)Met
hypotheti	Putative	GO:00081	GO:00160	GO:0005524	ATP binding;GO:0032559 adenyl ribonucleoti
hypotheti	Putative	GO:00081	GO:00056	GO:0003674	molecular_function
Ecm22p	Sterol re	GO:00359	GO:00484	GO:0000978	RNA polymerase II proximal promoter sequen
acetyl-Co	Acetyl-Co	GO:00066	GO:00057	GO:00039	(sce00072)Synthesis and degradation of keton
hypotheti	Member of	GO:00551	GO:00055	GO:0016491	oxidoreductase activity;GO:0003824 catalyt
Sma2p	Meiosis-s	GO:00705	GO:00056	GO:0003674	molecular_function
sideropho	ARN famil	GO:00447	GO:00003	GO:0015343	siderophore transmembrane transporter acti
hypotheti	Putative protein of unknown function; SWAT-GFP, seamless-GFP and mCherry fusion				
DUP240 fa	Putative	GO:00160	GO:00057	GO:0003674	molecular_function
Icy1p	Protein c	GO:00081	GO:00003	GO:0003674	molecular_function
basic ami	Permease	GO:19908	GO:00003	GO:0015174	basic amino acid transmembrane transporter
thioredox	Stress in	GO:00427	GO:00058	GO:0008379	thioredoxin peroxidase activity;GO:0051920
ribosomal	Protein c	GO:00021	GO:00226	GO:00037	(sce03010)Ribosome
alcohol c	Alcohol c	GO:00434	GO:00056	GO:00040	(sce00350)Tyrosine metabolism; (sce00071)Fat
PAQR-type	Membrane	GO:00068	GO:00057	GO:0038023	signaling receptor activity;GO:0060089 mol
ubiquinol	Cytochrom	GO:00061	GO:00057	GO:00451	(sce00190)Oxidative phosphorylation; (sce011
hypotheti	Protein c	GO:00081	GO:00056	GO:0003674	molecular_function
long-chai	Long chai	GO:19053	GO:00057	GO:01023	(sce)beta-Oxidation, acyl-CoA synthesis; (MO
basic ami	Plasma me	GO:00158	GO:00057	GO:0003674	molecular_function
Aim17p	Protein c	GO:00453	GO:00057	GO:0016702	oxidoreductase activity, acting on single
Sue1p	Protein r	GO:00301	GO:00057	GO:0003674	molecular_function
Ina1p	Protein c	GO:00315	GO:00512	GO:0003674	molecular_function
hypotheti	Putative	GO:00081	GO:00003	GO:0003674	molecular_function
nicotinat	Nicotinat	GO:00343	GO:00007	GO:00045	(sce00760)Nicotinate and nicotinamide metabo
Mlf3p	Serine-ri	GO:00309	GO:00058	GO:0003674	molecular_function
None	None	None	None	None	None
isopenten	Isopenten	GO:00509	GO:00058	GO:00044	(sce)C5 isoprenoid biosynthesis, mevalonate
hypotheti	Putative	GO:00081	GO:00055	GO:0050662	coenzyme binding;GO:0048037 cofactor bindi
delta(14)	C-14 ster	GO:00066	GO:00301	GO:00506	(sce00100)Steroid biosynthesis; (sce01130)Bi
Sut2p	Zn2Cys6 t	GO:19023	GO:00056	GO:0001103	RNA polymerase II repressing transcription
Hsp70 fam	Heat shoc	GO:00066	GO:00058	GO:00441	(sce04213)Longevity regulating pathway - mul
Scm4p	Mitochonc	GO:00004	GO:00057	GO:0003674	molecular_function
Sds24p	Protein i	GO:00300	GO:00058	GO:0003674	molecular_function
cytochrom	Cytochrom	GO:00061	GO:00057	GO:00090	(sce00920)Sulfur metabolism; (sce01100)Metab
sterol-bi	Sterol bi	GO:00159	GO:00003	GO:0015485	cholesterol binding;GO:0032934 sterol bind
fatty aci	Elongase	GO:00346	GO:00301	GO:01027	(sce)Fatty acid biosynthesis, elongation, en
citrate (	Citrate s	GO:00065	GO:00057	GO:00041	(M00010)Citrate cycle, first carbon oxidatio
glutamine	Glutamine	GO:00342	GO:00055	GO:00043	(sce00250)Alanine, aspartate and glutamate m
sterol es	Steryl es	GO:00161	GO:00058	GO:0004771	sterol esterase activity;GO:0016298 lipase
succinate	Alpha sub	GO:00061	GO:00426	GO:00047	(sce00640)Propanoate metabolism; (sce)Citrat
Slp1p	Glycosyla	GO:00349	GO:00301	GO:0003674	molecular_function
diphospho	Mevalonat	GO:00192	GO:00058	GO:00041	(sce)C5 isoprenoid biosynthesis, mevalonate
seripaupe	Protein c	GO:00304	GO:00092	GO:0005199	structural constituent of cell wall;GO:000
ubiquinol	Subunit 9	GO:00061	GO:00057	GO:00081	(sce00190)Oxidative phosphorylation; (sce011
phosphol	Putative	GO:19053	GO:00003	GO:0004012	phospholipid-translocating ATPase activity
cytochrom	Subunit I	GO:00061	GO:00057	GO:00041	(sce00190)Oxidative phosphorylation; (sce011
floccul	Lectin-li	GO:00362	GO:00092	GO:0005537	mannose binding;GO:0048029 monosaccharide
ferric/cu	Ferric re	GO:00156	GO:00003	GO:0052851	ferric-chelate reductase (NADPH) activity;
ribonucle	Ribonucle	GO:00092	GO:00059	GO:00047	(sce00480)Glutathione metabolism; (sce00240)
cytochrom	Subunit V	GO:00336	GO:00057	GO:00041	(sce00190)Oxidative phosphorylation; (sce011
adenine p	Potential	GO:00442	GO:00057	GO:00020	(sce00230)Purine metabolism; (sce01100)Metab

Cos12p	Endosomal	GO:004332	GO:000032	GO:0003674 molecular_function	
cytochron	Subunit V	GO:000612	GO:000575	GO:000412	(sce00190)Oxidative phosphorylation; (sce011(
fluoride	Protein i	GO:190342	GO:000588	GO:1903425 fluoride transmembrane transporter activit	
ATPase in	Protein t	GO:003278	GO:000573	GO:0042030 ATPase inhibitor activity;GO:0060590 ATPas	
pyridoxin	Putative	GO:004282	GO:000563	GO:005023	(sce00750)Vitamin B6 metabolism; (sce01100)M
putative	Vacuolar	GO:001589	GO:000032	GO:0042626 ATPase activity, coupled to transmembrane r	
Sed1p	Major str	GO:003150	GO:000927	GO:0005199 structural constituent of cell wall;GO:000	
hypotheti	Putative	GO:004668	GO:000557	GO:0016491 oxidoreductase activity;GO:0003824 catalyt	
kynurenin	Kynurenin	GO:003435	GO:000563	GO:003042	(sce00380)Tryptophan metabolism; (sce01100)M
lysophosp	Phospholi	GO:004647	GO:000927	GO:000462	(sce00564)Glycerophospholipid metabolism
cytochron	Putative	GO:000130	GO:000578	GO:000412	(sce00520)Amino sugar and nucleotide sugar m
Ten1p	Protein t	GO:003220	GO:199087	GO:0043047 single-stranded telomeric DNA binding;GO:0	
D-lactate	Major mit	GO:190345	GO:000574	GO:000872	(sce00620)Pyruvate metabolism
None	None	None	None	None	None
hypotheti	Putative	GO:000815	GO:000573	GO:0003674 molecular_function	
myo-inosi	Myo-inosi	GO:001579	GO:000032	GO:0005366 myo-inositol:proton symporter activity;GO:	
serine/th	AGC famil	GO:000132	GO:000032	GO:000467	(sce04213)Longevity regulating pathway - mul
putative	Putative	GO:000608	GO:000557	GO:0018456 aryl-alcohol dehydrogenase (NAD+) activity	
Utr2p	Chitin tr	GO:000603	GO:000014	GO:0004553 hydrolase activity, hydrolyzing O-glycosyl	
Hbt1p	Shmoo tip	GO:000075	GO:000593	GO:0003674 molecular_function	
Sip18p	Phospholi	GO:004263	GO:000573	GO:0005543 phospholipid binding;GO:0008289 lipid bind	
Erg29p	Protein o	GO:000669	GO:000563	GO:0003674 molecular_function	
putative	Protein k	GO:004477	GO:000563	GO:0005516 calmodulin binding;GO:0004674 protein seri	
carbonyl	Aldehyde	GO:190142	GO:000573	GO:0004090 carbonyl reductase (NADPH) activity;GO:000	
lumazine	Lumazine	GO:000923	GO:000934	GO:000090	(sce00740)Riboflavin metabolism; (sce01110)B
hydroxyme	3-hydroxy	GO:001014	GO:000563	GO:000442	(sce00072)Synthesis and degradation of keton
hypotheti	Putative	GO:000815	GO:000557	GO:0003674 molecular_function	
Nej1p	Protein i	GO:004500	GO:007041	GO:004502	(sce03450)Non-homologous end-joining
Tma17p	ATPase de	GO:007068	GO:000563	GO:0043023 ribosomal large subunit binding;GO:0043024	
ribonucle	Ribonucle	GO:000926	GO:000597	GO:000474	(sce00480)Glutathione metabolism; (sce00240)I
Na(+)/Li	P-type AT	GO:004214	GO:000032	GO:0008554 sodium-exporting ATPase activity, phosphor	
hypotheti	Putative	GO:000815	GO:000573	GO:0003674 molecular_function	
hypotheti	Protein of unknown function; non-essential gene; interacts with the DNA helicase				
hypotheti	Putative	GO:000815	GO:000578	GO:0003674 molecular_function	
hypotheti	Protein o	GO:000815	GO:001602	GO:0003674 molecular_function	
sterol 24	Delta(24)	GO:000669	GO:000574	GO:000383	(M00102)Ergocalciferol biosynthesis; (sce)Er
hypotheti	Integral	GO:003043	GO:004512	GO:0003674 molecular_function	
glutathic	Cytosolic	GO:001073	GO:000573	GO:000436	(sce00480)Glutathione metabolism
phosphog	3-phospho	GO:190386	GO:000573	GO:000461	(M00002)Glycolysis, core module involving th
Om14p	Mitochond	GO:000662	GO:003130	GO:1990593 nascent polypeptide-associated complex bin	
ribosomal	Ribosomal	GO:000218	GO:002262	GO:000373	(sce03010)Ribosome
mismatch	DNA-bindi	GO:004350	GO:003230	GO:0032139 dinucleotide insertion or deletion binding	
acetyltra	Acetyltra	GO:004338	GO:000557	GO:0045462 trichothecene 3-O-acetyltransferase activi	
hypotheti	Putative	GO:000815	GO:000557	GO:0003674 molecular_function	
Hms1p	bHLH prot	GO:000712	GO:000563	GO:0046983 protein dimerization activity;GO:0003700 D	
Gyp7p	GTPase-ac	GO:003288	GO:000582	GO:0017137 Rab GTPase binding;GO:0005096 GTPase activ	
hypotheti	Dubious o	GO:000815	GO:000557	GO:0003674 molecular_function	
Dcg1p	Protein o	GO:000680	GO:000557	GO:0003674 molecular_function	
cytochron	Subunit V	GO:000612	GO:000574	GO:000412	(sce00190)Oxidative phosphorylation; (sce011(
mitochond	Mitochond	GO:007077	GO:000574	GO:0015183 L-aspartate transmembrane transporter acti	
peptidylp	Peptidyl-	GO:000041	GO:000582	GO:0016018 cyclosporin A binding;GO:0003755 peptidyl-	
serine/th	Serine-th	GO:006140	GO:000563	GO:0004712 protein serine/threonine/tyrosine kinase a	
proteasom	Non-ATPas	GO:004316	GO:003451	GO:003067	(sce03050)Proteasome
mannoprot	Haze-prot	GO:003150	GO:000927	GO:0015926 glucosidase activity;GO:0004553 hydrolase	

Fit1p	Mannoprot	GO:00158(	GO:00092'	GO:0005524 ATP binding;GO:0032559 adenyl ribonucleoti
hypotheti	Putative	GO:00081[	GO:00055'	GO:0005524 ATP binding;GO:0032559 adenyl ribonucleoti
nicotina	Quinolina	GO:00343[	GO:00056;	GO:00045] (sce00760)Nicotinate and nicotinamide metabo
transket	Transketo	GO:00060(	GO:00058;	GO:00048((M00007)Pentose phosphate pathway, non-oxida
protein p	Regulator	GO:00070(	GO:00001(	GO:0019888 protein phosphatase regulator activity;GO:
D-arabin	NAD-deper	GO:00704{	GO:00058;	GO:0047816 D-arabinose 1-dehydrogenase (NAD) activity
Spo21p	Componen	GO:00321;	GO:00359'	GO:0005198 structural molecule activity;GO:0003674 mo
Snx4p	Sorting r	GO:00344(	GO:00004(	GO:00322((sce04144)Endocytosis
cyanamide	Cyanamide	GO:00188(	GO:00055'	GO:00188; (sce00791)Atrazine degradation
trifuncti	Multifunc	GO:00001(	GO:00057;	GO:00046; (sce)Histidine biosynthesis, PRPP => histidi
methylste	C-4 methy	GO:00066(	GO:00057{	GO:00025[(sce00100)Steroid biosynthesis; (sce01130)Bi
Dif1p	Protein t	GO:19051]	GO:00056;	GO:1990846 ribonucleoside-diphosphate reductase inhib
cytochron	Mitochonc	GO:00066(	GO:00313(	GO:00041; (sce00520)Amino sugar and nucleotide sugar m
calmoduli	Calmoduli	GO:00345(	GO:00057;	GO:00046{ (Human)(hsa04925)Aldosterone synthesis and s
Yfh7p	Putative	GO:00163]	GO:00055'	GO:0016887 ATPase activity;GO:0017111 nucleoside-trip
Y' elemer	Putative	GO:00081[	GO:00057;	GO:0004386 helicase activity;GO:0017111 nucleoside-tr
ubiquinol	Subunit c	GO:00061;	GO:00057[	GO:00081; (sce00190)Oxidative phosphorylation; (sce011
hypotheti	Putative protein of unknown function			
cytochron	Subunit V	GO:00061;	GO:00057[	GO:00041; (sce00190)Oxidative phosphorylation; (sce011
DUP240 fa	Putative	GO:00160[	GO:00306(	GO:0003674 molecular_function
putative	Mannitol	GO:00195(	GO:00055'	GO:00460; (sce00051)Fructose and mannose metabolism
putative	Putative	GO:00007/	GO:00058{	GO:0004674 protein serine/threonine kinase activity;G
hypotheti	Protein c	GO:00081[	GO:00055'	GO:0003674 molecular_function
4-amino-[	Protein i	GO:00092;	GO:00055'	GO:00036' (sce00730)Thiamine metabolism; (sce01100)Met
hypotheti	Putative	GO:00081[	GO:00055'	GO:0003674 molecular_function
hypotheti	Protein c	GO:00081[	GO:00055'	GO:0003674 molecular_function
NADH-ubic	Mitochonc	GO:00196[	GO:00057[	GO:0003954 NADH dehydrogenase activity;GO:0016651 oxi
ribosoma	Protein c	GO:00021{	GO:00226;	GO:00198/ (sce03010)Ribosome
transcrip	Transcrip	GO:00060]	GO:00056;	GO:0001102 RNA polymerase II activating transcription
Jsn1p	Member of	GO:00516[	GO:00324'	GO:0003729 mRNA binding;GO:0003723 RNA binding;GO:000
lactoylg	Monomeric	GO:00192/	GO:00056;	GO:00044((sce00620)Pyruvate metabolism
aldo-keto	NADPH-dep	GO:00345(	GO:00058;	GO:0051268 alpha-keto amide reductase activity;GO:005
hypotheti	Protein c	GO:00081[	GO:00057{	GO:0003674 molecular_function
Apd1p	Protein c	GO:00081[	GO:00056;	GO:0003674 molecular_function
allantoir	Allantoir	GO:00094/	GO:00057;	GO:00040; (sce00230)Purine metabolism; (sce01100)Metab
hypotheti	Putative	GO:00081[	GO:00056;	GO:0003674 molecular_function
Ecm34p	Protein c	GO:00715[	GO:00057(	GO:0003674 molecular_function
sterol-4-	C-3 stero	GO:00066(	GO:00057{	GO:00025[(sce00100)Steroid biosynthesis; (sce01130)Bi
seripaupe	Cell wall	GO:00315(	GO:00092'	GO:0005199 structural constituent of cell wall;GO:000
glucose-i	GID Compl	GO:00457;	GO:00346[	GO:0003674 molecular_function
glucan 1,	Major exo	GO:00092[	GO:00092'	GO:00043; (sce00500)Starch and sucrose metabolism
Ras fami	GTP-bindi	GO:20002;	GO:00057{	GO:00039; (sce04933)AGE-RAGE signaling pathway in diab
stearoyl-	Delta(9)	GO:00066;	GO:00301'	GO:00047((sce01040)Biosynthesis of unsaturated fatty
GTPase-ac	Negative	GO:00071{	GO:00056;	GO:0005096 GTPase activator activity;GO:0030695 GTPas
Kti12p	Protein t	GO:00020(	GO:00335{	GO:0003682 chromatin binding;GO:0005524 ATP binding;G
Uga4p	GABA (gar	GO:00158/	GO:00003;	GO:0015495 gamma-aminobutyric acid:proton symporter a
hexose tr	Protein c	GO:00157(	GO:00058{	GO:00155' (sce04113)Meiosis - yeast
glycerol	Glycerol	GO:00195(	GO:00057;	GO:00043' (sce00561)Glycerolipid metabolism; (sce01100
hypotheti	Putative	GO:00081[	GO:00057;	GO:0003674 molecular_function
Fus1p	Membrane	GO:00313{	GO:00433;	GO:00036' (sce04011)MAPK signaling pathway - yeast
putative	Glycogen	GO:00714'	GO:00055'	GO:0004674 protein serine/threonine kinase activity;G
Mg-depenc	Magnesiur	GO:00353;	GO:00056;	GO:0030946 protein tyrosine phosphatase activity, met
succinate	Beta subu	GO:00061(	GO:00057;	GO:00047' (sce00640)Propanoate metabolism; (sce)Citrat
Rcf2p	Cytochron	GO:00336]	GO:00313(	GO:0003674 molecular_function

uroporphy	Uroporphy	GO:000678	GO:000582	GO:000485	(sce00860)Porphyrin and chlorophyll metaboli
high-affi	High-affi	GO:007157	GO:000588	GO:0000006	high-affinity zinc transmembrane transport
hypotheti	NADH-depe	GO:190142	GO:000563	GO:0004029	aldehyde dehydrogenase (NAD) activity;GO:0
Bsc1p	Protein c	GO:000815	GO:000557	GO:0003674	molecular_function
hypotheti	Protein c	GO:000815	GO:000573	GO:0003674	molecular_function
methylgly	3-methylb	GO:000820	GO:000563	GO:004656	(sce00640)Propanoate metabolism; (sce00620)P
squalene	Squalene	GO:000669	GO:000581	GO:000450	(sce00909)Sesquiterpenoid and triterpenoid b
acetate--	Acetyl-co	GO:001942	GO:000573	GO:000398	(sce00640)Propanoate metabolism; (sce00680)M
protein k	Protein k	GO:000646	GO:000593	GO:0004674	protein serine/threonine kinase activity;G
Mga1p	Protein s	GO:006140	GO:000079	GO:0000978	RNA polymerase II proximal promoter sequen
Ddi1p	DNA damag	GO:004332	GO:000588	GO:0004190	aspartic-type endopeptidase activity;GO:00
bifunctio	Farnesyl-	GO:000669	GO:000574	GO:000431	(sce00909)Sesquiterpenoid and triterpenoid b
C-8 stero	C-8 stero	GO:000669	GO:000578	GO:000024	(M00102)Ergocalciferol biosynthesis; (sce)Erg
hypotheti	Protein c	GO:000815	GO:000032	GO:0003674	molecular_function
ribosomal	Ribosomal	GO:000047	GO:003068	GO:000373	(sce03010)Ribosome
DNA-bindi	GTB motif	GO:007193	GO:003330	GO:0001135	RNA polymerase II transcription regulator r
proteasom	ATPase of	GO:004589	GO:000854	GO:003640	(sce03050)Proteasome
putative	Putative	GO:000656	GO:000582	GO:000434	(M00015)Proline biosynthesis, glutamate => p
Hsp90 fam	Cytoplasm	GO:000049	GO:000927	GO:004262	(sce04141)Protein processing in endoplasmic r
serine/th	Ser/Thr p	GO:006109	GO:004333	GO:0004674	protein serine/threonine kinase activity;G
phosphome	Phosphome	GO:003138	GO:000577	GO:000463	(sce)C5 isoprenoid biosynthesis, mevalonate p
hypotheti	Protein c	GO:000815	GO:000563	GO:0003674	molecular_function
ubiquitin	Ubiquitin	GO:003043	GO:199042	GO:0043008	ATP-dependent protein binding;GO:0031386 p
Rqc2p	Component	GO:199011	GO:199011	GO:0043023	ribosomal large subunit binding;GO:0043022
putative	Protein c	GO:010607	GO:000573	GO:0002196	Ser-tRNA(Ala) hydrolase activity;GO:000216
exosome n	Exosome n	GO:007103	GO:000017	GO:000372	(sce03018)RNA degradation
type II H	Type II H	GO:003571	GO:002262	GO:005178	(Human)(hsa04141)Protein processing in endop
2-deoxygl	2-deoxygl	GO:000600	GO:000557	GO:0003850	2-deoxyglucose-6-phosphatase activity;GO:0
meiotic r	Protein c	GO:000713	GO:000578	GO:0004029	aldehyde dehydrogenase (NAD) activity;GO:0
hypotheti	Putative protein of unknown function; SWAT-GFP fusion protein localizes to the e				
Yap5p	Basic leu	GO:001072	GO:000079	GO:0000981	DNA-binding transcription factor activity,
bifunctio	AP4A phos	GO:000916	GO:000563	GO:000478	(sce00920)Sulfur metabolism; (sce00230)Purine
dithiol g	Glutathio	GO:001073	GO:000563	GO:0004362	glutathione-disulfide reductase activity;G
Dan1p	Cell wall	GO:003150	GO:000927	GO:0005199	structural constituent of cell wall;GO:000
indolepyr	Minor iso	GO:000094	GO:000582	GO:005017	(sce00010)Glycolysis / Gluconeogenesis; (sce0
protein p	Meiosis-s	GO:003047	GO:000562	GO:000815	(sce04113)Meiosis - yeast
ubiquinol	Subunit 6	GO:000612	GO:000575	GO:000812	(sce00190)Oxidative phosphorylation; (sce0110
Gre1p	Hydrophil	GO:000815	GO:000573	GO:0003674	molecular_function
Aqy3p	Putative	GO:007191	GO:007194	GO:001516	(Human)(hsa04962)Vasopressin-regulated water
hypotheti	Putative	GO:000815	GO:000573	GO:0003674	molecular_function
phosphopa	Negative	GO:001593	GO:007151	GO:0004633	phosphopantothenoylcysteine decarboxylase a
ubiquinol	Subunit 7	GO:000612	GO:000575	GO:000812	(sce00190)Oxidative phosphorylation; (sce0110
glycerol-	DL-glycer	GO:000611	GO:000563	GO:000012	(sce00561)Glycerolipid metabolism; (sce01100)
ubiquinol	Ubiquinol	GO:000612	GO:000575	GO:000812	(sce00190)Oxidative phosphorylation; (sce0110
Toh1p	GPI-ancho	GO:000815	GO:000927	GO:0003674	molecular_function
hypotheti	Putative	GO:000815	GO:003122	GO:0003674	molecular_function
phosphata	Plasma me	GO:007147	GO:000582	GO:0004721	phosphoprotein phosphatase activity;GO:001
Fmp52p	Protein c	GO:005111	GO:000574	GO:0003674	molecular_function
fatty aci	Sphingoli	GO:000667	GO:000578	GO:0080132	fatty acid alpha-hydroxylase activity;GO:0
hypotheti	Putative protein of unknown function; deletion confers sensitivity to 4-(N-(S-gl				
phosphogl	Homolog o	GO:000609	GO:000573	GO:000461	(M00002)Glycolysis, core module involving thr
Yap6p	Basic leu	GO:000013	GO:000563	GO:0001010	RNA polymerase II sequence-specific DNA-bi
None	None	None	None	None	None
serine/th	Serine/th	GO:006021	GO:003212	GO:000467	(sce04011)MAPK signaling pathway - yeast

alcohol (Alcohol aGO:00342(GO:003017GO:0004026 alcohol O-acetyltransferase activity;GO:00
hypotheti Putative GO:00815 GO:00055 GO:0003674 molecular_function
Apq12p Nuclear eGO:00069 GO:00319 GO:0003674 molecular_function
Rho famil Non-essenGO:00903 GO:00059 GO:0003924 GTPase activity;GO:0005525 GTP binding;GO:
Cu/Pi car Mitochon GO:00068 GO:00313 GO:0005375 copper ion transmembrane transporter activ
putative Ubiquitin GO:00165 GO:00057 GO:0004843 thiol-dependent ubiquitin-specific proteas
proteasom Essential GO:00065 GO:00085 GO:00302 (sce03050)Proteasome
saccharop Saccharop GO:00198 GO:00057 GO:00047 (M00030)Lysine biosynthesis, AAA pathway, 2-
Erg28p Endoplasm GO:00066 GO:00057 GO:0030674 protein binding, bridging;GO:0060090 molec
Hsp90 coc Hsp90 coc GO:00066 GO:00057 GO:00420 (Human)(hsa05020)Prion diseases
Rag GTPas Subunit c GO:19037 GO:00344 GO:0003924 GTPase activity;GO:0005525 GTP binding;GO:
glutathic Possible GO:00617 GO:00009 GO:0019172 glyoxalase III activity;GO:0016836 hydro-l
glycine c H subunit GO:00194 GO:00059 GO:00043 (sce00630)Glyoxylate and dicarboxylate metab
Aif1p Mitochon GO:00003 GO:00057 GO:0003955 NAD(P)H dehydrogenase (quinone) activity;G
Sut1p Zn(II)2Cy GO:19023 GO:00056 GO:0001103 RNA polymerase II repressing transcription
putative Putative GO:00229 GO:00313 GO:0004174 electron-transferring-flavoprotein dehydro
Cos111p Protein r GO:00071 GO:00057 GO:0003674 molecular_function
Zps1p Putative GO:00065 GO:00092 GO:0005178 integrin binding;GO:0008237 metallopeptida
alcohol (Alcohol a GO:19010 GO:00058 GO:0004026 alcohol O-acetyltransferase activity;GO:00
putative Non-essen GO:00060 GO:00058 GO:00191 (M00549)Nucleotide sugar biosynthesis, gluco
putative Protein k GO:00068 GO:00056 GO:0004674 protein serine/threonine kinase activity;G
Btn2p v-SNARE b GO:00068 GO:00343 GO:0051087 chaperone binding;GO:0000149 SNARE binding
hypotheti Pseudogen GO:00001 GO:00055 GO:0003674 molecular_function
putative Putative GO:00060 GO:00055 GO:0018456 aryl-alcohol dehydrogenase (NAD+) activity
stress-re Stress-re GO:00972 GO:00058 GO:00009 (sce04213)Longevity regulating pathway - mul
Cad1p AP-1-like GO:00360 GO:00056 GO:0001228 DNA-binding transcription activator activi
malate sy Malate sy GO:00060 GO:00095 GO:00044 (M00012)Glyoxylate cycle; (sce)Glyoxylate cy
Mix17p Mitochon GO:00090 GO:00057 GO:0003674 molecular_function
Mot3p Transcrip GO:00108 GO:00007 GO:0001103 RNA polymerase II repressing transcription
allantoin Allantoin GO:00157 GO:00003 GO:0005274 allantoin:proton symporter activity;GO:001
hypotheti Cytoplasmic hydrophilin essential in desiccation-rehydration process; expression
low-affin Low-affin GO:00715 GO:00057 GO:0000007 low-affinity zinc ion transmembrane transp
Afr1p Protein r GO:00007 GO:00014 GO:0003674 molecular_function
thiamine Transport GO:19030 GO:00003 GO:1903089 5-amino-1-ribofuranosylimidazole-4-carboxa
Phd1p Transcrip GO:20002 GO:00056 GO:0043565 sequence-specific DNA binding;GO:0003700 D
NAD-depen Conserved GO:19045 GO:00308 GO:00469 (sce00760)Nicotinate and nicotinamide metabo
Ups2p Mitochon GO:20012 GO:00313 GO:1990050 phosphatidic acid transporter activity;GO:
tRNA(ader Subunit c GO:00021 GO:00527 GO:0052717 tRNA-specific adenosine-34 deaminase activ
hypotheti Dubious c GO:00081 GO:00055 GO:0003674 molecular_function
putative Putative GO:00064 GO:00059 GO:0004674 protein serine/threonine kinase activity;G
homeodoma Homeobox- GO:00011 GO:00056 GO:00083 (sce04113)Meiosis - yeast
guanine r Nucleotic GO:00102 GO:00058 GO:00316 (sce04113)Meiosis - yeast
phosphori Phosphori GO:00461 GO:00056 GO:00089 (M00549)Nucleotide sugar biosynthesis, gluco
Cox15p Protein r GO:00067 GO:00057 GO:00041 (sce00860)Porphyrin and chlorophyll metaboli
Cts1p Endochiti GO:00060 GO:00092 GO:00088 (sce00520)Amino sugar and nucleotide sugar m
Ncp1p NADP-cyto GO:00066 GO:00057 GO:0003958 NADPH-hemoprotein reductase activity;GO:00
ubiquitin Ubiquitin GO:00726 (Human)G GO:0004843 thiol-dependent ubiquitin-specific proteas
Gds1p Protein c GO:00090 GO:00057 GO:0003674 molecular_function
Dap1p Heme-binc GO:00324 GO:00057 GO:0020037 heme binding;GO:0046906 tetrapyrrole bindi
Fra1p Protein i GO:00343 GO:00058 GO:0070006 metalloaminopeptidase activity;GO:0004177
ribosomal Ribosomal GO:00004 GO:00226 GO:00037 (sce03010)Ribosome
histone H Histone H GO:00615 GO:00312 GO:00469 (Human)(hsa05322)Systemic lupus erythematosu
hypotheti Non-essen GO:00081 GO:00055 GO:0003674 molecular_function
methionir S-adenosy GO:00065 GO:00104 GO:00044 (sce)Methionine salvage pathway; (M00034)Met

Ask10p	Regulatoı	GO:00903	GO:00057	GO:0016247	channel regulator activity;GO:0098772 mole
Tye7p	Serine-r	GO:00458	GO:00007	GO:0043565	sequence-specific DNA binding;GO:0046983 p
Y' elemer	Putative	GO:00081	GO:00055	GO:0004386	helicase activity;GO:0017111 nucleoside-tr
ribosomal	Protein (	GO:00002	GO:00226	GO:007018	(sce03010)Ribosome
Glc8p	Regulatoı	GO:00325	GO:00056	GO:0004864	protein phosphatase inhibitor activity;GO:
1-pyrroli	Delta-1-p	GO:00101	GO:00057	GO:00038	(sce00330)Arginine and proline metabolism; (
Ies6p	Componen	GO:00519	GO:00310	GO:0003674	molecular_function
Anp1p	Subunit (	GO:00000	GO:00001	GO:00000	(sce)N-glycan biosynthesis, high-mannose typ
Opi10p	Protein w	GO:00060	GO:00056	GO:0003674	molecular_function
None	None	None	None	None	None
hypotheti	Putative	GO:00081	GO:00057	GO:0003674	molecular_function
retromer	Vacuolar	GO:00450	GO:00309	GO:00085	(sce04144)Endocytosis
Hal1p	Cytoplasr	GO:00096	GO:00057	GO:0003674	molecular_function
4-amino-5	Protein i	GO:00092	GO:00055	GO:00036	(sce00730)Thiamine metabolism; (sce01100)Met
hypotheti	Putative	GO:00081	GO:00055	GO:0003674	molecular_function
5-methyl	Cobalamir	GO:00090	GO:00058	GO:00038	(sce00450)Selenocompound metabolism; (sce002
Irc4p	Protein (	GO:19904	GO:00056	GO:0003674	molecular_function
Bdf2p	Protein i	GO:00314	GO:00719	GO:0001094	TFIID-class transcription factor complex b
phosphopy	Enolase I	GO:00328	GO:00000	GO:00046	(M00002)Glycolysis, core module involving th
chaperone	Tetradeca	GO:00450	GO:00426	GO:00036	(sce03018)RNA degradation
3',5'-cy	High-affi	GO:00199	GO:00056	GO:00041	(sce00230)Purine metabolism
salt home	Serine/th	GO:00068	GO:00056	GO:00047	(sce03015)mRNA surveillance pathway; (sce041
chaperone	Small hea	GO:00346	GO:00104	GO:00510	(sce04141)Protein processing in endoplasmic
adenyl-nu	ATPase co	GO:00104	GO:00058	GO:00007	(sce04141)Protein processing in endoplasmic
pheromone	Pheromone	GO:00160	GO:00057	GO:0003674	molecular_function
aspartyl	Aspartic	GO:00714	GO:00092	GO:00041	(sce04011)MAPK signaling pathway - yeast
aldehyde	Cytoplasr	GO:00194	GO:00057	GO:00438	(sce00360)Phenylalanine metabolism; (sce004
ATP-bindi	Plasma me	GO:00429	GO:00058	GO:0008559	xenobiotic transmembrane transporting ATPa
Hsp70 far	Heat shoc	GO:00510	GO:00426	GO:0044183	protein binding involved in protein foldin
Cdc37p	Essential	GO:00001	GO:00057	GO:0051087	chaperone binding;GO:0031072 heat shock pr
proteasor	Beta 7 su	GO:00104	GO:00345	GO:00042	(sce03050)Proteasome
phosphogl	Tetrameri	GO:00060	GO:00057	GO:00046	(M00002)Glycolysis, core module involving th
Ert1p	Transcrip	GO:00614	GO:00056	GO:0001227	DNA-binding transcription repressor activi
ammonium	Ammonium	GO:00071	GO:00058	GO:0008519	ammonium transmembrane transporter activit
glycogen	Glycogen	GO:00059	GO:00057	GO:00081	(sce00500)Starch and sucrose metabolism; (sc
polynucle	RNA 5'-tı	GO:00985	GO:00315	GO:0004651	polynucleotide 5'-phosphatase activity;GO:
palmitoyl	Palmitoyl	GO:00182	GO:00057	GO:0019706	protein-cysteine S-palmitoyltransferase ac
Gfd2p	Protein (	GO:00081	GO:00055	GO:0003676	nucleic acid binding;GO:1901363 heterocycl
hypotheti	Putative	GO:00081	GO:00343	GO:0003674	molecular_function
glycerol-	NAD-deper	GO:00061	GO:00093	GO:00479	(sce00564)Glycerophospholipid metabolism; (s
D-serine	D-serine	GO:00360	GO:00055	GO:00087	(sce00260)Glycine, serine and threonine meta
hypotheti	Putative	GO:00081	GO:00055	GO:0003674	molecular_function
proteasor	Alpha 7 s	GO:00104	GO:00345	GO:00042	(sce03050)Proteasome
putative	Protein k	GO:00602	GO:00056	GO:00046	(Human)(hsa04068)FoxO signaling pathway; (hs
Gsf2p	Endoplasr	GO:00343	GO:00301	GO:0051082	unfolded protein binding;GO:0005515 protei
Dig1p	MAP kinas	GO:20002	GO:19905	GO:00081	(sce04011)MAPK signaling pathway - yeast
D-lactate	2-hydroxy	GO:00195	GO:00312	GO:00996	(sce00620)Pyruvate metabolism
beta-fru	Invertase	GO:19029	GO:00003	GO:00516	(sce00052)Galactose metabolism; (sce00500)St
putative	Mannitol	GO:00195	GO:00055	GO:00460	(sce00051)Fructose and mannose metabolism
ubiquinol	Subunit 8	GO:00061	GO:00057	GO:00081	(sce00190)Oxidative phosphorylation; (sce011
ubiquitir	Golgi-loc	GO:00433	GO:00058	GO:0043130	ubiquitin binding;GO:0032182 ubiquitin-lik
putative	Putative	GO:00336	GO:00313	GO:0004222	metalloendopeptidase activity;GO:0008237 m
delta(24	C-24(28)	GO:00066	GO:00301	GO:00002	(M00102)Ergocalciferol biosynthesis; (sce)Er
bifunctic	ER associ	GO:00107	GO:00057	GO:00046	(sce00480)Glutathione metabolism

S-glutat	S-glutat	GO:00988	GO:00057	GO:0045174 glutathione dehydrogenase (ascorbate) acti
glucose-	Glycolyt	GO:00060	GO:00057	GO:00043 (M00004)Pentose phosphate pathway (Pentose p
hypothet	Putative	GO:00093	GO:00057	GO:0004573 mannosyl-oligosaccharide glucosidase activ
proteaso	Alpha 1	GO:00104	GO:00345	GO:00042 (sce03050)Proteasome
phosphog	3-phosph	GO:00065	GO:00057	GO:00617 (M00020)Serine biosynthesis, glycerate-3P =>
Aim21p	Protein	GO:00346	GO:00304	GO:0043022 ribosome binding;GO:0051015 actin filament
Hsp70 fa	HSP70 fa	GO:00357	GO:00092	GO:00441 (sce04213)Longevity regulating pathway - mul
hypothet	Protein	GO:00081	GO:00058	GO:0003674 molecular_function
hypothet	Putative	GO:00070	GO:00057	GO:0003674 molecular_function
recombin	Protein	GO:00007	GO:00002	GO:19908 (sce03440)Homologous recombination
ribosoma	Protein	GO:00021	GO:00226	GO:00198 (sce03010)Ribosome
ATP-bind	Plasma m	GO:00353	GO:00058	GO:0042626 ATPase activity, coupled to transmembrane
Cos3p	Endosoma	GO:00433	GO:00003	GO:0003674 molecular_function
Kti11p	Zn-ribbo	GO:00171	GO:00058	GO:0009055 electron transfer activity;GO:0005506 iron
hypothet	Protein of unknown function; localized to the mitochondrial outer membrane			
hypothet	Putative	GO:00081	GO:00003	GO:0003674 molecular_function
hypothet	Putative	GO:00081	GO:00055	GO:0003674 molecular_function
ubiquino	Core sub	GO:00090	GO:00057	GO:00081 (sce00190)Oxidative phosphorylation; (sce011
Mss1p	Mitochon	GO:00708	GO:00057	GO:0003924 GTPase activity;GO:0005525 GTP binding;GO:
phosphoa	Essentia	GO:00342	GO:00056	GO:00046 (sce00520)Amino sugar and nucleotide sugar m
biotin s	Biotin s	GO:00091	GO:00057	GO:00040 (sce00780)Biotin metabolism; (sce01100)Metab
Ubx5p	UBX doma	GO:00431	GO:00056	GO:0003674 molecular_function
serine/t	PAS doma	GO:00609	GO:00058	GO:0004674 protein serine/threonine kinase activity;G
Vhs2p	Regulato	GO:00321	GO:00057	GO:0003674 molecular_function
Gtt3p	Protein	GO:00081	GO:00343	GO:0003674 molecular_function
Stp4p	Protein	GO:00081	GO:00057	GO:0043565 sequence-specific DNA binding;GO:0003677 D
Asg7p	Protein	GO:00007	GO:00125	GO:0003674 molecular_function
SNAP rec	Target m	GO:00000	GO:00003	GO:0005484 SNAP receptor activity;GO:0000149 SNARE bi
hypothet	Putative	GO:00081	GO:00057	GO:0003674 molecular_function
putative	Cell wal	GO:00007	GO:00092	GO:0042973 glucan endo-1,3-beta-D-glucosidase activit
bifuncti	Urea ami	GO:00434	GO:00057	GO:00040 (sce00791)Atrazine degradation; (sce00220)Ar
F1F0 ATP	Subunit	GO:00159	GO:00002	GO:00469 (sce00190)Oxidative phosphorylation; (sce011
ERMES co	ER integ	GO:00450	GO:00328	GO:01200 (sce04139)Mitophagy - yeast
Luc7p	Essentia	GO:00063	GO:00056	GO:0003729 mRNA binding;GO:0003723 RNA binding;GO:000
ribosoma	Protein	GO:00459	GO:00226	GO:00198 (sce03010)Ribosome
proteaso	Beta 4 s	GO:00104	GO:00345	GO:00611 (sce03050)Proteasome
Rrm3p	DNA heli	GO:00970	GO:00057	GO:0043141 ATP-dependent 5'-3' DNA helicase activity;
hypothet	Putative	GO:00081	GO:00058	GO:0003674 molecular_function
protein-	Nuclear	GO:00605	GO:00058	GO:00352 (Human)(hsa04922)Glucagon signaling pathway;
Doa1p	WD repea	GO:00345	GO:00057	GO:00431 (sce04141)Protein processing in endoplasmic
allantoi	Allantoi	GO:00002	GO:00055	GO:00040 (sce00230)Purine metabolism; (sce01100)Metab
citrate	Citrate	GO:00463	GO:00057	GO:00041 (M00010)Citrate cycle, first carbon oxidatio
hypothet	Putative	GO:00081	GO:00160	GO:0003674 molecular_function
bifuncti	O-acetyl	GO:00712	GO:00058	GO:00039 (sce00920)Sulfur metabolism; (sce00270)Cyste
hypothet	Putative	GO:00081	GO:00160	GO:0003674 molecular_function
cleavage	Essentia	GO:00987	GO:00058	GO:00360 (sce03015)mRNA surveillance pathway
Snu71p	Componen	GO:00083	GO:00056	GO:0003723 RNA binding;GO:0003676 nucleic acid bindin
glutamat	Glutamin	GO:00065	GO:00343	GO:00043 (sce00910)Nitrogen metabolism; (sce00220)Arg
transcri	Transcri	GO:00723	GO:00056	GO:0000978 RNA polymerase II proximal promoter sequen
ADP/ATP	Major AD	GO:00158	GO:00057	GO:0005471 ATP:ADP antiporter activity;GO:0015217 ADP
putative	Mitochon	GO:00325	GO:00057	GO:00430 (sce03010)Ribosome
pyruvate	Pyruvate	GO:00060	GO:00058	GO:00047 (sce)Guanine ribonucleotide biosynthesis IMP
cytochro	Subunit	GO:00061	GO:00057	GO:00041 (sce00190)Oxidative phosphorylation; (sce011
NAPDH-de	Conserve	GO:19013	GO:00057	GO:0003958 NADPH-hemoprotein reductase activity;GO:00

triose-pł Triose pł GO:00195(GO:0057: GO:00048((M00002)Glycolysis, core module involving th
lysophosp Lyso-phos GO:00359(GO:00313(GO:00471((sce00564)Glycerophospholipid metabolism
proteason ATPase oi GO:00706! GO:00085! GO:00364((sce03050)Proteasome
phosphati Phosphati GO:00439! GO:00715(GO:0008526 phosphatidylinositol transporter activity;
Avt3p Vacuolar GO:00329' GO:00003: GO:00151((sce04138)Autophagy - yeast
ribonucl(Minor is(GO:00092(GO:00059' GO:00047! (sce00480)Glutathione metabolism; (sce00240)
phytocer: Alkaline GO:00465] GO:00325! GO:00707' (sce00600)Sphingolipid metabolism; (sce01100)
superoxi(Cytosoli(GO:00013: GO:00057! GO:00047((sce04213)Longevity regulating pathway - mul
Rfu1p Protein (GO:00109! GO:00057(GO:0061578 Lys63-specific deubiquitinase activity;GO:
proteason Subunit (GO:00432! GO:00345] GO:00316: (sce03050)Proteasome
None None None None None None
hypotheti Putative GO:00081! GO:00055' GO:0003674 molecular_function
type 1 pı Regulato: GO:00514! GO:00001(GO:0004865 protein serine/threonine phosphatase inhib
pantoate- Pantother GO:00333] GO:00056! GO:00045! (sce00410)beta-Alanine metabolism; (sce00770)
hypotheti Putative GO:00081! GO:00055' GO:0003674 molecular_function
Rcf1p Cytochror GO:00972! GO:00313(GO:0003674 molecular_function
NADH-ubi(Mitochon(GO:00196! GO:00057! GO:0003954 NADH dehydrogenase activity;GO:0016651 oxi
She4p Protein (GO:00075! GO:00057: GO:0017022 myosin binding;GO:0008092 cytoskeletal pro
hypotheti Putative protein with similarity to allantoate permease; similar to the allantoa
Msc1p Protein (GO:00071! GO:00057(GO:0003674 molecular_function
histone l Histone l GO:00615(GO:00312! GO:0046982 protein heterodimerization activity;GO:004
hypotheti Protein (GO:00081! GO:00055' GO:0003674 molecular_function
polyamin(Polyamin(GO:00462(GO:00057: GO:00529((sce00410)beta-Alanine metabolism; (sce00330)
Hor7p Protein (GO:00070(GO:00003: GO:0003674 molecular_function
fluoride Protein i GO:19034: GO:00058(GO:1903425 fluoride transmembrane transporter activit
proteason ATPase oi GO:00458! GO:00085! GO:00316: (sce03050)Proteasome
hypotheti Putative protein of unknown function; predicted to have thiol-disulfide oxidored
hypotheti Putative GO:00081! GO:00057: GO:0003674 molecular_function
None None None None None None
proteoli| Proteoli| GO:00327(GO:00003: GO:0030234 enzyme regulator activity;GO:0098772 molec
transcri| TFIIF (Tı GO:00011] GO:00056' GO:00011: (sce03022)Basal transcription factors
Whi2p Protein ı GO:00004: GO:00719! GO:0019211 phosphatase activator activity;GO:0019208
proteason ATPase oi GO:00458! GO:00085! GO:00364((sce03050)Proteasome
hypotheti Putative protein of unknown function
U4/U6-U5 Splicing GO:00002! GO:00056(GO:00037: (sce03040)Spliceosome
aldehyde Cytosoli(GO:00067! GO:00057: GO:00337: (sce00410)beta-Alanine metabolism; (sce00280)
glutathi(Phosphol: GO:00988(GO:00313] GO:00470((sce00590)Arachidonic acid metabolism; (sce0(
NAD(+) di NADH dipł GO:00196' GO:00057' GO:00002] (sce00760)Nicotinate and nicotinamide metabo
Sok2p Nuclear ı GO:00071: GO:00058: GO:00435((sce04113)Meiosis - yeast
Lsb1p Negative GO:00343] GO:00304' GO:0051015 actin filament binding;GO:0035091 phosphat
ribosoma] Ribosoma] GO:00021(GO:00226: GO:00198! (sce03010)Ribosome
Ear1p Specific] GO:00433: GO:00100(GO:0003674 molecular_function
ubiquiti: Ubiquiti: GO:00315' GO:00057: GO:0004842 ubiquitin-protein transferase activity;GO:
hypotheti Putative GO:00081! GO:00057: GO:0003674 molecular_function
hypotheti Putative GO:00350(GO:00704(GO:0004402 histone acetyltransferase activity;GO:0061
Yim1p Protein (GO:00069' GO:00058] GO:0003674 molecular_function
putative Protein (GO:00007(GO:00330(GO:00001! (sce03440)Homologous recombination
cyclin CI G1 cycli: GO:00003: GO:00003(GO:00165: (sce04011)MAPK signaling pathway - yeast; (s
Rsn1p Membrane GO:00068! GO:00003: GO:0005227 calcium activated cation channel activity;
Srl1p Mannopro(GO:00315(GO:00059: GO:0003674 molecular_function
cystinos: Protein i GO:00158] GO:00003: GO:0015184 L-cystine transmembrane transporter activi
None None None None None None
Fen2p Plasma m(GO:00987] GO:00003: GO:0015233 pantothenate transmembrane transporter act
3-keto-s1 3-keto s1 GO:00066! GO:00057! GO:00002! (sce00100)Steroid biosynthesis; (sce01130)Bi

Csi1p	Subunit (	GO:00075	GO:00818	GO:0003674 molecular_function	
AAA fami	AAA ATPas	GO:199017	GO:00083	GO:003159	(sce04141)Protein processing in endoplasmic
Bud13p	Subunit (	GO:00028	GO:007027	GO:0003674 molecular_function	
Lee1p	Zinc-fing	GO:00815	GO:00557	GO:0046872 metal ion binding;GO:0043169 cation bindin	
Nur1p	Protein i	GO:00430	GO:00343	GO:0003674 molecular_function	
Mix23p	Mitochond	GO:00815	GO:00575	GO:0003674 molecular_function	
Sol1p	Protein v	GO:00090	GO:00563	GO:00170	(M00006)Pentose phosphate pathway, oxidative
tyrosine	Nuclear p	GO:00018	GO:00563	GO:00472	(sce04011)MAPK signaling pathway - yeast
mitochond	Mitochond	GO:00002	GO:00576	GO:00198	(sce03010)Ribosome
diacylgly	Cholineph	GO:00665	GO:00574	GO:00414	(sce)Phosphatidylcholine (PC) biosynthesis,
Mic27p	Component	GO:00324	GO:00616	GO:0003674 molecular_function	
Rgm1p	Putative	GO:00459	GO:00079	GO:0000981 DNA-binding transcription factor activity,	
dihydroxy	Dihydroxy	GO:00195	GO:00582	GO:00503	(sce00051)Fructose and mannose metabolism; (
porin POR	Mitochond	GO:00510	GO:00313	GO:00152	(Human)(hsa04216)Ferroptosis; (hsa05012)Park
histone H	Histone H	GO:00709	GO:00435	GO:00314	(Human)(hsa05322)Systemic lupus erythematosus
6-phospho	6-phospho	GO:00090	GO:00582	GO:00170	(M00006)Pentose phosphate pathway, oxidative
Bol3p	Protein i	GO:01060	GO:00575	GO:0003674 molecular_function	
protein s	Protein s	GO:00064	GO:00582	GO:00472	(Human)(hsa04010)MAPK signaling pathway
hypotheti	Protein (	GO:00815	GO:00198	GO:0043022 ribosome binding;GO:0043021 ribonucleoprot	
Hsv2p	Phosphati	GO:00344	GO:00340	GO:0010314 phosphatidylinositol-5-phosphate binding;G	
Pun1p	Plasma me	GO:00315	GO:00512	GO:0003674 molecular_function	
hypotheti	Putative	GO:00815	GO:00557	GO:0003674 molecular_function	
glutamate	Glutamate	GO:00065	GO:00582	GO:00043	(sce00430)Taurine and hypotaurine metabolism
DUP240 fa	Putative	GO:00160	GO:00579	GO:0003674 molecular_function	
hypotheti	Putative protein of unknown function; the authentic, non-tagged protein is detec				
hypotheti	Putative	GO:00815	GO:00573	GO:0003674 molecular_function	
rRNA-proc	Protein (	GO:00004	GO:00306	GO:0003674 molecular_function	
mevalonat	Mevalonat	GO:00101	GO:00582	GO:00044	(sce)C5 isoprenoid biosynthesis, mevalonate
hypotheti	Putative	GO:00464	GO:00573	GO:0003674 molecular_function	
thioredox	Nuclear t	GO:00988	GO:00007	GO:0008379 thioredoxin peroxidase activity;GO:0051920	
nicotinam	High-affi	GO:00342	GO:00588	GO:0034257 nicotinamide riboside transmembrane transp	
hypotheti	Putative	GO:00815	GO:00557	GO:0003674 molecular_function	
thiosulfa	Thiosulfa	GO:00815	GO:00574	GO:0004792 thiosulfate sulfurtransferase activity;GO:	
Rab GTPas	GTPase-ac	GO:00706	GO:00001	GO:0017137 Rab GTPase binding;GO:0005096 GTPase activ	
hypotheti	Protein (	GO:00815	GO:00563	GO:0003674 molecular_function	
Cos9p	Endosomal	GO:00433	GO:00576	GO:0003674 molecular_function	
hypotheti	Putative	GO:00815	GO:00593	GO:0003674 molecular_function	
Cup9p	Homeodoma	GO:00359	GO:00563	GO:0001103 RNA polymerase II repressing transcription	
protein k	Serine/th	GO:19025	GO:00563	GO:00046	(sce04113)Meiosis - yeast
proteasom	Essential	GO:00432	GO:00345	GO:00051	(sce03050)Proteasome
Mth1p	Negative	GO:19046	GO:00557	GO:0003674 molecular_function	
None	None	None	None	None	None
Jlp2p	Protein (	GO:00815	GO:00573	GO:0003674 molecular_function	
Lif1p	Component	GO:00063	GO:00328	GO:00036	(sce03450)Non-homologous end-joining
F1F0 ATP	Subunit t	GO:00159	GO:00002	GO:00469	(sce00190)Oxidative phosphorylation; (sce011
hypotheti	Putative	GO:00815	GO:00003	GO:0003674 molecular_function	
Tim21p	Nonessent	GO:00301	GO:00574	GO:0003674 molecular_function	
guanine n	Guanine n	GO:00000	GO:00582	GO:0043130 ubiquitin binding;GO:0032182 ubiquitin-lik	
Ecm29p	Scaffold	GO:00432	GO:00005	GO:0032947 protein-containing complex scaffold activi	
Aga1p	Anchorage	GO:00007	GO:00092	GO:0050839 cell adhesion molecule binding;GO:0005515	
Pfk26p	6-phospho	GO:00060	GO:00582	GO:00038	(sce00051)Fructose and mannose metabolism
Tca17p	Component	GO:00344	GO:19900	GO:0003674 molecular_function	
None	None	None	None	None	None
homoisoci	Homo-isoc	GO:00198	GO:00573	GO:00470	(sce)Lysine biosynthesis, 2-oxoglutarate =>

ubiquinol	Subunit 2	GO:00061	GO:00300	GO:00081	(sce00190)Oxidative phosphorylation; (sce011
Npl4p	Substrate	GO:00726	GO:00008	GO:00431	(sce04141)Protein processing in endoplasmic
putative	Putative	GO:00060	GO:00055	GO:0018456 aryl-alcohol dehydrogenase (NAD+) activity	
Shu2p	Component	GO:00007	GO:00971	GO:0003674 molecular_function	
acetolact	Regulator	GO:00090	GO:00059	GO:00039	(sce00660)C5-Branched dibasic acid metabolis
hexose t	Low-affin	GO:00157	GO:00058	GO:00053	(sce04113)Meiosis - yeast
F1F0 ATP	Subunit 5	GO:00159	GO:00002	GO:00469	(sce00190)Oxidative phosphorylation; (sce011
TFIIH/NEF	Subunit c	GO:00708	GO:00056	GO:00616	(sce03022)Basal transcription factors; (sce0
Bns1p	Protein c	GO:00513	GO:00055	GO:0003674 molecular_function	
Aep2p	Mitochond	GO:00701	GO:00057	GO:0003723 RNA binding;GO:0003676 nucleic acid bindin	
histone H	Histone H	GO:00615	GO:00312	GO:00469	(Human)(hsa05322)Systemic lupus erythematosu
proteasom	ATPase of	GO:00706	GO:00345	GO:00364	(sce03050)Proteasome
Mrx12p	Protein t	GO:00081	GO:00057	GO:0003674 molecular_function	
proteasom	Alpha 6 s	GO:00104	GO:00345	GO:00042	(sce03050)Proteasome
Iml2p	Protein i	GO:00712	GO:00057	GO:0003674 molecular_function	
hexose t	Low affin	GO:00157	GO:00058	GO:00155	(sce04113)Meiosis - yeast
DNA-direc	RNA polym	GO:00427	GO:00056	GO:00010	(sce03020)RNA polymerase; (sce00240)Pyrimidi
hypothet	Putative	GO:00081	GO:00055	GO:0003674 molecular_function	
acid phos	One of th	GO:00163	GO:00092	GO:00039	(sce00740)Riboflavin metabolism; (sce00730)T
methylate	DNA repai	GO:00063	GO:00056	GO:0003908 methylated-DNA-[protein]-cysteine S-methyl	
glycine c	T subunit	GO:00194	GO:00059	GO:00043	(sce00670)One carbon pool by folate; (sce006
protein p	UBX domai	GO:00311	GO:00058	GO:00198	(sce04141)Protein processing in endoplasmic
hypothet	Putative	GO:00081	GO:00055	GO:0003674 molecular_function	
tRNA spli	Subunit c	GO:00003	GO:00002	GO:0000213 tRNA-intron endonuclease activity;GO:0016	
Yig1p	Protein t	GO:00061	GO:00058	GO:0003674 molecular_function	
trehalose	Large sub	GO:00704	GO:00059	GO:00038	(sce00500)Starch and sucrose metabolism; (sc
Dic1p	Mitochond	GO:19023	GO:00057	GO:00151	(Human)(hsa04964)Proximal tubule bicarbonate
alcohol d	Mitochond	GO:00009	GO:00057	GO:00040	(sce00350)Tyrosine metabolism; (sce00071)Fat
hypothet	Protein c	GO:00081	GO:00055	GO:0003674 molecular_function	
mitochond	Mitochond	GO:00325	GO:00057	GO:0003735 structural constituent of ribosome;GO:0005	
glucosami	Glucosami	GO:00060	GO:00057	GO:00043	(sce00520)Amino sugar and nucleotide sugar m
H(+)-tran	Subunit A	GO:00070	GO:00002	GO:00469	(sce04145)Phagosome; (sce00190)Oxidative pho
lipid-bin	Eisosome	GO:00974	GO:00362	GO:0008289 lipid binding;GO:0005488 binding;GO:00036	
bifunctio	Methylene	GO:00066	GO:00319	GO:00046	(M00091)Phosphatidylcholine (PC) biosynthesi
DNA-direc	RNA polym	GO:00602	GO:00056	GO:00039	(sce03020)RNA polymerase; (sce00240)Pyrimidi
glutamine	Component	GO:00065	GO:00616	GO:0004044 amidophosphoribosyltransferase activity;GO	
Fat3p	Protein i	GO:00159	GO:00512	GO:0003674 molecular_function	
Pai3p	Cytoplasm	GO:00070	GO:00057	GO:0019828 aspartic-type endopeptidase inhibitor acti	
Sdd1p	Protein c	GO:00081	GO:00055	GO:0003674 molecular_function	
3'(2'),5'	Bisphosph	GO:00090	GO:00056	GO:00084	(sce00920)Sulfur metabolism; (sce01130)Biosy
Mir1p	Mitochond	GO:00354	GO:00313	GO:0005315 inorganic phosphate transmembrane transpor	
3-dehydro	3-ketosph	GO:00066	GO:00057	GO:00475	(sce00600)Sphingolipid metabolism; (sce01100
glucose-i	Subunit c	GO:00070	GO:00346	GO:0003674 molecular_function	
glutathio	Methylgly	GO:00192	GO:00104	GO:0019172 glyoxalase III activity;GO:0016836 hydro-l	
Sap185p	Protein t	GO:00020	GO:0005739 mitochondrion;GO:0044444 cytoplasmic part;GO:00057		
Ies3p	Subunit c	GO:00427	GO:00310	GO:0003674 molecular_function	
F1F0 ATP	Subunit h	GO:00159	GO:00002	GO:00469	(sce00190)Oxidative phosphorylation; (sce011
hypothet	Putative	GO:00081	GO:00312	GO:0003674 molecular_function	
Mag1p	3-methyl-	GO:00063	GO:00329	GO:00528	(sce03410)Base excision repair
Eds1p	Putative	GO:00063	GO:00056	GO:0000981 DNA-binding transcription factor activity,	
Sro9p	Cytoplasmic RNA-b	GO:00104	GO:0003729 mRNA binding;GO:0003723 RNA binding;GO:000		
inositol	Inositol	GO:00060	GO:00055	GO:00528	(sce00562)Inositol phosphate metabolism; (sc
protein p	Regulator	GO:00070	GO:00001	GO:00047	(sce03015)mRNA surveillance pathway; (sce041
Tgl1p	Steryl es	GO:00161	GO:00058	GO:00047	(sce00100)Steroid biosynthesis

argininos	Argininos	GO:004245	GO:000582	GO:000405	(sce00220)Arginine biosynthesis; (sce00250)A
putative	Member of	GO:199087	GO:000588	GO:004836	(sce04011)MAPK signaling pathway - yeast
DUP240 fa	Putative	GO:001605	GO:000032	GO:0003674 molecular_function	
branched-	Cytosolic	GO:001950	GO:000573	GO:005265	(sce)Valine/isoleucine biosynthesis, pyruvate
ATP-bindi	Plasma me	GO:004661	GO:000588	GO:0042626 ATPase activity, coupled to transmembrane m	
4-hydroxy	Para hydr	GO:000674	GO:003130	GO:000841	(M00128)Ubiquinone biosynthesis, eukaryotes,
mitochond	Mitochond	GO:003254	GO:000576	GO:000373	(sce03010)Ribosome
Far8p	Protein i	GO:000032	GO:000578	GO:0003674 molecular_function	
acireduct	Acireduct	GO:001950	GO:000563	GO:001030	(sce)Methionine salvage pathway; (M00034)Meth
Hsp70 fam	ATPase in	GO:000661	GO:000582	GO:004418	(sce04213)Longevity regulating pathway - mul
Mnn4p	Putative	GO:000649	GO:001602	GO:0008047 enzyme activator activity;GO:0030234 enzym	
phosphati	Phosphati	GO:003609	GO:003533	GO:0043813 phosphatidylinositol-3,5-bisphosphate 5-ph	
Taf13p	TFIID sub	GO:005112	GO:000566	GO:001625	(sce03022)Basal transcription factors
hypotheti	Protein o	GO:000815	GO:000563	GO:0003674 molecular_function	
chitin sy	Chitin sy	GO:000091	GO:004500	GO:000410	(sce00520)Amino sugar and nucleotide sugar me
hypotheti	Putative protein of unknown function; green fluorescent protein (GFP)-fusion pro				
mitochond	Mitochond	GO:003254	GO:000576	GO:0003735 structural constituent of ribosome;GO:0005	
protein-l	Lysine me	GO:001802	GO:000573	GO:0016279 protein-lysine N-methyltransferase activit	
Gat1p	Transcrip	GO:000108	GO:000563	GO:0000978 RNA polymerase II proximal promoter sequen	
Nsg2p	Protein i	GO:001612	GO:000578	GO:0051082 unfolded protein binding;GO:0005515 protei	
Rsf2p	Zinc-fing	GO:000012	GO:000079	GO:0000981 DNA-binding transcription factor activity,	
hypotheti	Putative	GO:000815	GO:000563	GO:0003674 molecular_function	
Sip1p	Alternate	GO:200022	GO:003158	GO:0004679 AMP-activated protein kinase activity;GO:0	
glucan 1,	Glucosida	GO:007088	GO:001717	GO:003391	(sce)N-glycan precursor trimming; (M00073)N-g
None	None	None	None	None	None
Ecm13p	Non-essen	GO:007155	GO:000557	GO:0003674 molecular_function	
glucokina	Glucokina	GO:004632	GO:000582	GO:001915	(M00549)Nucleotide sugar biosynthesis, glucos
Rsa1p	Protein i	GO:000049	GO:000565	GO:0003674 molecular_function	
DNA-direc	Mitochond	GO:000626	GO:003424	GO:0003896 DNA primase activity;GO:0001018 mitochondr	
cAMP-depe	cAMP-depe	GO:001060	GO:000595	GO:000469	(sce04213)Longevity regulating pathway - mul
hypotheti	Putative	GO:000815	GO:000557	GO:0003674 molecular_function	
6-phospho	Alpha sub	GO:006162	GO:000594	GO:000387	(sce00051)Fructose and mannose metabolism; (s
carbamoyl	Small sub	GO:000652	GO:000595	GO:000408	(sce)Uridine monophosphate biosynthesis, glu
trifuncti	Trifuncti	GO:000923	GO:000582	GO:005033	(sce00730)Thiamine metabolism; (sce01100)Meta
ESCRT-II	One of fo	GO:190466	GO:000081	GO:000551	(sce04144)Endocytosis
proteasom	ATPase of	GO:004589	GO:000854	GO:003640	(sce03050)Proteasome
sphingoli	Protein t	GO:190006	GO:003533	GO:0003674 molecular_function	
hypotheti	Fungal-sp	GO:000815	GO:000557	GO:0003674 molecular_function	
ubiquitin	Multiubiq	GO:001099	GO:000050	GO:003445	(sce04120)Ubiquitin mediated proteolysis
None	None	None	None	None	None
G protein	G protein	GO:000075	GO:012017	GO:003168	(sce04011)MAPK signaling pathway - yeast
Bro1p	Cytoplasm	GO:190466	GO:001000	GO:0035800 deubiquitinase activator activity;GO:00165	
Arc35p	Subunit o	GO:003431	GO:003047	GO:000520	(sce04144)Endocytosis
hexadecen	Dehydroge	GO:003218	GO:003130	GO:001848	(sce00410)beta-Alanine metabolism; (sce00280)
Mix14p	Mitochond	GO:000906	GO:000575	GO:0003674 molecular_function	
glutamate	Gamma-glu	GO:005512	GO:000563	GO:000435	(M00015)Proline biosynthesis, glutamate => pr
Mnd1p	Protein r	GO:000713	GO:000079	GO:0003690 double-stranded DNA binding;GO:0003677 DNA	
Bzz1p	SH3 domai	GO:004501	GO:003047	GO:0008047 enzyme activator activity;GO:0030234 enzym	
proteasom	Subunit o	GO:000033	GO:003451	GO:000551	(sce03050)Proteasome
putative	Putative	GO:000676	GO:000575	GO:000884	(M00126)Tetrahydrofolate biosynthesis, GTP =>
Ltv1p	Componen	GO:190466	GO:003444	GO:0003674 molecular_function	
Mif2p	Protein r	GO:005145	GO:000077	GO:0019237 centromeric DNA binding;GO:1990837 sequenc	
threonine	Threonine	GO:000908	GO:000563	GO:000479	(M00018)Threonine biosynthesis, aspartate =>
Na(+)-exp	P-type AT	GO:009913	GO:000573	GO:0008554 sodium-exporting ATPase activity, phosphor	

4-aminobu	Gamma-ami	GO:00094	GO:00057	GO:00343	(sce)GABA (gamma-Aminobutyrate) shunt; (M000
Shc1p	Sporulati	GO:00060	GO:00056	GO:0008047	enzyme activator activity;GO:0030234 enzym
asparagin	Asparagin	GO:00709	GO:00058	GO:00040	(sce00250)Alanine, aspartate and glutamate m
Mdg1p	Plasma me	GO:00007	GO:00451	GO:0003674	molecular_function
ATPase-ac	Essential	GO:19026	GO:00306	GO:00016	(sce03010)Ribosome
proline p	Proline p	GO:00355	GO:00058	GO:0015193	L-proline transmembrane transporter activi
mitogen-a	MAP kinas	GO:00001	GO:00059	GO:00050	(sce04139)Mitophagy - yeast; (sce04011)MAPK
Rho famil	Non-esser	GO:00436	GO:00003	GO:0003924	GTPase activity;GO:0005525 GTP binding;GO:
trifuncti	NADPH-dep	GO:00195	GO:00058	GO:0004032	alditol:NADP+ 1-oxidoreductase activity;GO
glycine c	P subunit	GO:00194	GO:00059	GO:00043	(sce00630)Glyoxylate and dicarboxylate metab
Mvp1p	Protein r	GO:00066	GO:00057	GO:0032266	phosphatidylinositol-3-phosphate binding;G
spermidin	Spermidin	GO:00082	GO:00056	GO:00047	(sce)Methionine salvage pathway; (M00034)Met
hypotheti	Putative protein of unknown function				
F1F0 ATP	Epsilon s	GO:00159	GO:00057	GO:00469	(sce00190)Oxidative phosphorylation; (sce011
Rtt10p	WD40 doma	GO:00324	GO:00057	GO:0003674	molecular_function
hypotheti	Putative	GO:00081	GO:00055	GO:0003674	molecular_function
phosphati	Phosphoin	GO:00709	GO:00321	GO:0005546	phosphatidylinositol-4,5-bisphosphate bind
malate de	Mitochonc	GO:00061	GO:00057	GO:00044	(sce00620)Pyruvate metabolism; (sce01200)Car
Jip4p	Protein c	GO:00081	GO:00055	GO:0003674	molecular_function
ATP-depen	ATPase, p	GO:00004	GO:00306	GO:0004004	ATP-dependent RNA helicase activity;GO:000
hypotheti	Putative	GO:00081	GO:00059	GO:0003674	molecular_function
calcineur	Calcineur	GO:00007	GO:00059	GO:00331	(Human)(hsa05014)Amyotrophic lateral scleros
flavin ad	Flavin ad	GO:00158	GO:00059	GO:0015230	FAD transmembrane transporter activity;GO:
putative	Putative	GO:00325	GO:00057	GO:00037	(sce03010)Ribosome
None	None	None	None	None	None
xylulokin	Xylulokin	GO:00059	GO:00057	GO:00048	(sce00040)Pentose and glucuronate interconve
hypotheti	Protein c	GO:00104	GO:00003	GO:0030414	peptidase inhibitor activity;GO:0061134 pe
Mic10p	Conservec	GO:00424	GO:00616	GO:0003674	molecular_function
putative	Mitochonc	GO:00020	GO:00057	GO:0008198	ferrous iron binding;GO:0051537 2 iron, 2
pentafunc	Pentafunc	GO:00094	GO:00057	GO:00038	(M00022)Shikimate pathway, phosphoenolpyruva
Taf12p	Subunit	GO:00439	GO:00466	GO:00044	(sce03022)Basal transcription factors
27S pre-r	AdoMet-de	GO:00004	GO:00306	GO:0008650	rRNA (uridine-2'-O-)-methyltransferase act
glutathio	Glutaredo	GO:00988	GO:00057	GO:0004362	glutathione-disulfide reductase activity;G
proteasom	Beta 5 su	GO:00801	GO:00345	GO:00042	(sce03050)Proteasome
None	None	None	None	None	None
Tpo1p	Polyamine	GO:19037	GO:00331	GO:0015606	spermidine transmembrane transporter activ
phosphati	Polyphosp	GO:00468	GO:00304	GO:00438	(sce00562)Inositol phosphate metabolism; (sc
phosphati	Protein i	GO:00065	GO:00005	GO:00171	(sce00563)Glycosylphosphatidylinositol (GPI)
Put3p	Transcrip	GO:20011	GO:00056	GO:0000978	RNA polymerase II proximal promoter sequen
mitochonc	Mitochonc	GO:00000	GO:00057	GO:00701	(sce03010)Ribosome
N-acetylg	Catalytic	GO:00064	GO:00435	GO:00045	(sce)N-glycan precursor biosynthesis; (M0005
protein c	Protein c	GO:19043	GO:00057	GO:00037	(sce04141)Protein processing in endoplasmic
hypotheti	Putative	GO:00081	GO:00055	GO:0003674	molecular_function
adenosylh	S-adenosy	GO:00195	GO:00058	GO:00040	(sce00270)Cysteine and methionine metabolism
ESCRT-II	Component	GO:00004	GO:00008	GO:00036	(sce04144)Endocytosis
aldehyde	Cytoplasm	GO:00194	GO:00057	GO:00438	(sce00360)Phenylalanine metabolism; (sce0041
None	None	None	None	None	None
pheromone	Pheromone	GO:00032	GO:00433	GO:0003674	molecular_function
sterol ac	Acyl-CoA:	GO:00082	GO:00057	GO:00347	(sce00100)Steroid biosynthesis
glucose-i	Subunit c	GO:00457	GO:00346	GO:0004842	ubiquitin-protein transferase activity;GO:
None	None	None	None	None	None
Sog2p	Key compo	GO:00071	GO:00433	GO:0003674	molecular_function
glucose-i	Central c	GO:00457	GO:00346	GO:0003674	molecular_function
Rei1p	Cytoplasm	GO:00071	GO:00306	GO:0008270	zinc ion binding;GO:0043565 sequence-speci

serine/tl	Protein s	GO:00617(	GO:01200(	GO:00046	(sce04136)Autophagy - other; (sce04139)Mitopl
Ecl1p	Protein (	GO:00081	GO:00055	GO:0003674	molecular_function
putative	NADPH oxi	GO:00329	GO:00970	GO:0000293	ferric-chelate reductase activity;GO:00161
proteina	Vacuolar	GO:00070	GO:00003	GO:00042	(sce04138)Autophagy - yeast
mRNA (gu	Methyltra	GO:01060(	GO:00058	GO:00044	(sce03015)mRNA surveillance pathway
aconitat	Aconitase	GO:00060	GO:00426	GO:00039	(M00010)Citrate cycle, first carbon oxidatio
Fmp40p	Putative	GO:00081	GO:00057	GO:0003674	molecular_function
proteaso	Beta 1 su	GO:00104	GO:00345	GO:00042	(sce03050)Proteasome
Sip2p	One of th	GO:20002	GO:00315	GO:00046	(Human)(hsa04710)Circadian rhythm; (hsa04213)
seripaup	Protein (	GO:00304	GO:00092	GO:0005199	structural constituent of cell wall;GO:000
mitochon	Mitochon	GO:00325	GO:00057	GO:0003735	structural constituent of ribosome;GO:0005
Scp1p	Componen	GO:00013(	GO:00304	GO:0030674	protein binding, bridging;GO:0051015 actin
ATP-depe	ATP-depe	GO:00511	GO:00057	GO:0004176	ATP-dependent peptidase activity;GO:000425
GET comp	Subunit (	GO:00450	GO:00435	GO:0043495	protein membrane anchor;GO:0005515 protein
trifuncti	Aldose re	GO:00195	GO:00058	GO:00328	(sce00040)Pentose and glucuronate interconve
proteaso	Beta 2 su	GO:00104	GO:00345	GO:00042	(sce03050)Proteasome
Rmi1p	Subunit (	GO:00070	GO:00314	GO:0000400	four-way junction DNA binding;GO:0000217 D
H(+)-exp	Plasma me	GO:01200	GO:00057	GO:00085	(sce00190)Oxidative phosphorylation
putative	Plasma me	GO:00714	GO:00058	GO:0004721	phosphoprotein phosphatase activity;GO:001
Rtc3p	Protein (	GO:00063	GO:00056	GO:0003674	molecular_function
Mrx11p	Protein t	GO:00081	GO:00057	GO:0003674	molecular_function
Yps5p	Protein v	GO:00315	GO:00092	GO:0004190	aspartic-type endopeptidase activity;GO:00
3-hydrox	3-hydrox	GO:00343	GO:00056	GO:00003	(sce00380)Tryptophan metabolism; (sce01100)M
bifuncti	Glycogen	GO:00059	GO:00057	GO:00041	(sce00500)Starch and sucrose metabolism; (sc
Pst1p	Cell wal	GO:00315	GO:00092	GO:0003674	molecular_function
seripaup	Protein (	GO:00315	GO:00092	GO:0005199	structural constituent of cell wall;GO:000
Src1p	Inner nu	GO:00430	GO:00056	GO:0003674	molecular_function
alpha-1,	Alpha-1,	GO:00064	GO:00003	GO:00000	(sce00514)Other types of O-glycan biosynthes
Pex3p	Peroxiso	GO:00450	GO:00057	GO:00306	(sce04146)Peroxisome
Mga2p	ER membr	GO:00360	GO:00301	GO:0003674	molecular_function
Rev7p	Accessor	GO:00709	GO:00160	GO:00038	(sce01100)Metabolic pathways
glucose	Plasma me	GO:00515	GO:00058	GO:00053	(sce04113)Meiosis - yeast
myo-inos	Myo-inos	GO:00157	GO:00003	GO:0005366	myo-inositol:proton symporter activity;GO:
Ett1p	Nuclear p	GO:20006	GO:00056	GO:0003674	molecular_function
F1F0 ATP	Subunit (	GO:00336	GO:00002	GO:00469	(sce00190)Oxidative phosphorylation; (sce011
mRNA-bin	RNA-bind	GO:20008	GO:00104	GO:00313	(sce03022)Basal transcription factors
aldose 1-	Putative	GO:00334	GO:00055	GO:0004034	aldose 1-epimerase activity;GO:0016857 rac
Css1p	Protein (	GO:00071	GO:00055	GO:0003674	molecular_function
Spo77p	Meiosis-	GO:00321	GO:00057	GO:0003674	molecular_function
proteaso	Subunit (	GO:00431	GO:00345	GO:00036	(sce03050)Proteasome
seripaup	Protein (	GO:00315	GO:00092	GO:0005199	structural constituent of cell wall;GO:000
NADPH-ad	Oxidored	GO:00068	GO:00057	GO:0015039	NADPH-adrenodoxin reductase activity;GO:00
alanine--	Alanine:	GO:00192	GO:00057	GO:00047	(sce00630)Glyoxylate and dicarboxylate metab
Pex21p	Peroxin	GO:00165	GO:00057	GO:0005515	protein binding;GO:0005488 binding;GO:0003
histidin	Histidin	GO:00001	GO:00057	GO:00044	(sce)Histidine biosynthesis, PRPP => histidi
Tom5p	Componen	GO:00700	GO:00057	GO:0008320	protein transmembrane transporter activity
isocitra	Cytosoli	GO:00060	GO:00058	GO:00044	(M00010)Citrate cycle, first carbon oxidatio
Rsc6p	Componen	GO:00063	GO:00165	GO:0015616	DNA translocase activity;GO:0008094 DNA-de
Csr2p	Nuclear u	GO:00700	GO:00058	GO:0031625	ubiquitin protein ligase binding;GO:004438
hypothet	Putative	GO:00081	GO:00160	GO:0003674	molecular_function
hypothet	Putative protein of unknown function; green fluorescent protein (GFP)-fusion pro				
F1F0 ATP	Subunit g	GO:00159	GO:00002	GO:00469	(sce00190)Oxidative phosphorylation; (sce011
sphingol	Protein t	GO:19000	GO:00353	GO:0003674	molecular_function
chromati	ATPase a	GO:00427	GO:00310	GO:0043140	ATP-dependent 3'-5' DNA helicase activity;

acylglyc	Monoglyc	GO:00194	GO:00057	GO:00473	(sce)Acylglycerol degradation; (M00098)Acylg
Elongato	Subunit	GO:00324	GO:00335	GO:0008017	microtubule binding;GO:0015631 tubulin bin
ribosoma	Protein	GO:00000	GO:00226	GO:00037	(sce03010)Ribosome
nicotina	Nicotina	GO:19045	GO:00007	GO:00089	(sce00760)Nicotinate and nicotinamide metabo
sterol 1	Lanoster	GO:00066	GO:00057	GO:00083	(sce00100)Steroid biosynthesis; (sce01130)Bi
proteaso	Alpha 5	GO:00104	GO:00345	GO:00042	(sce03050)Proteasome
Arf fami	ADP-ribo	GO:00068	GO:00057	GO:00039	(sce04144)Endocytosis
glucan e	Exo-1,3-	GO:00092	GO:00092	GO:00043	(sce00500)Starch and sucrose metabolism
Ski7p	GTP-bind	GO:00704	GO:00058	GO:00037	(sce03015)mRNA surveillance pathway; (sce030
fructose	Fructose-	GO:00059	GO:00425	GO:00421	(M00003)Gluconeogenesis, oxaloacetate => fru
putative	Possible	GO:00065	GO:00301	GO:0004252	serine-type endopeptidase activity;GO:0008
hypothet	Putative	GO:00081	GO:00055	GO:0003674	molecular_function
hypothet	Putative	GO:00081	GO:00055	GO:0003674	molecular_function
hypothet	Putative	GO:00322	GO:00160	GO:0008757	S-adenosylmethionine-dependent methyltrans
metalloe	Zinc met	GO:00065	GO:00057	GO:00042	(Human)(hsa04614)Renin-angiotensin system
Uli1p	Protein	GO:00309	GO:00057	GO:0003674	molecular_function
Pno1p	Essentia	GO:00004	GO:00306	GO:0051082	unfolded protein binding;GO:0003723 RNA bi
tRNA thr	tRNA thr	GO:00615	GO:00057	GO:0061503	tRNA threonylcarbamoyladenosine dehydratas
(R,R)-bu	NAD-depe	GO:00340	GO:00057	GO:00007	(sce00650)Butanoate metabolism
hypothet	Protein	GO:00004	GO:00058	GO:0043022	ribosome binding;GO:0043021 ribonucleoprot
She2p	RNA-bind	GO:00075	GO:00059	GO:1990825	sequence-specific mRNA binding;GO:0003729
Dst1p	General	GO:00315	GO:00056	GO:0001139	RNA polymerase II complex recruiting activ
translat	Translat	GO:19901	GO:00436	GO:00430	(sce03013)RNA transport
None	None	None	None	None	None
malate d	Cytoplas	GO:00165	GO:00343	GO:00300	(M00012)Glyoxylate cycle; (sce)Glyoxylate cy
alanine	Catalyti	GO:00428	GO:00056	GO:00040	(sce00220)Arginine biosynthesis; (sce00250)A
AAA fami	Huge dyn	GO:00000	GO:00306	GO:00168	(sce03008)Ribosome biogenesis in eukaryotes
hypothet	Protein	GO:00081	GO:00057	GO:0003674	molecular_function
phosphat	Mg2+-dep	GO:00062	GO:00058	GO:00081	(sce00561)Glycerolipid metabolism; (sce00564
stress-r	Trimeric	GO:00614	GO:00057	GO:0000978	RNA polymerase II proximal promoter sequen
Ubx7p	UBX (ubi	GO:00304	GO:00056	GO:0003674	molecular_function
mitochon	Highly c	GO:00068	GO:00313	GO:0050833	pyruvate transmembrane transporter activit
Sgt2p	Glutamin	GO:19036	GO:00723	GO:0032947	protein-containing complex scaffold activi
mannosyl	Mannosyl	GO:00000	GO:00001	GO:0000026	alpha-1,2-mannosyltransferase activity;GO:
F1F0 ATP	Subunit	GO:00159	GO:00002	GO:00469	(sce00190)Oxidative phosphorylation; (sce011
hypothet	Componen	GO:00081	GO:00321	GO:0003674	molecular_function
hexose t	Putative	GO:00157	GO:00003	GO:00155	(sce04113)Meiosis - yeast
E2 ubiqu	Ubiquiti	GO:00716	GO:00001	GO:00706	(sce04120)Ubiquitin mediated proteolysis; (s
peroxiso	ER-resid	GO:00325	GO:00057	GO:0003674	molecular_function
Utp9p	Nucleola	GO:00459	GO:0034455 t-UTP		(sce03008)Ribosome biogenesis in eukaryotes
hypothet	Putative	GO:00081	GO:00055	GO:0003674	molecular_function
endopoly	Dual end	GO:00067	GO:00003	GO:0000298	endopolyphosphatase activity;GO:0004309 ex
fumarate	Soluble	GO:00714	GO:00058	GO:0016156	fumarate reductase (NADH) activity;GO:0000
homocitr	Homocitr	GO:00198	GO:00057	GO:00044	(sce)Lysine biosynthesis, 2-oxoglutarate =>
Rmp1p	Subunit	GO:00002	GO:00001	GO:00421	(sce03008)Ribosome biogenesis in eukaryotes
pheromon	Pheromon	GO:00081	GO:00057	GO:0003674	molecular_function
Msi1p	Subunit	GO:00063	GO:00331	GO:0042393	histone binding;GO:0005515 protein binding
hypothet	Putative protein of unknown function; identified by gene-trapping, microarray-ba				
E2 ubiqu	Ubiquiti	GO:00304	GO:00058	GO:00706	(sce04120)Ubiquitin mediated proteolysis
putative	Putative	GO:00304	GO:00056	GO:0004553	hydrolase activity, hydrolyzing O-glycosyl
Avl9p	Conserve	GO:00068	GO:00059	GO:0003674	molecular_function
tRNA 1-m	Subunit	GO:00304	GO:00315	GO:0016429	tRNA (adenine-N1-)-methyltransferase activ
hypothet	Nuclear	GO:00081	GO:00058	GO:0003674	molecular_function
Mad3p	Subunit	GO:00070	GO:00007	GO:00046	(sce04111)Cell cycle - yeast

Aim9p	Protein (	GO:00081[illegible]	GO:00057[illegible]	GO:0003674 molecular_function	
Ecm2p	Pre-mRNA	GO:00003[illegible]	GO:00710[illegible]	GO:00170[illegible]	(sce03040)Spliceosome
cytochron	Subunit \	GO:00061[illegible]	GO:00057[illegible]	GO:00041[illegible]	(sce00190)Oxidative phosphorylation; (sce011[illegible]
Erv15p	Protein i	GO:00071[illegible]	GO:00301[illegible]	GO:0003674 molecular_function	
Aca1p	ATF/CREB	GO:00459[illegible]	GO:00056[illegible]	GO:0001228 DNA-binding transcription activator activi	
DNA-bindi	Zinc clus	GO:00614[illegible]	GO:00056[illegible]	GO:0001228 DNA-binding transcription activator activi	
isoleucir	Mitochonc	GO:00090[illegible]	GO:00057[illegible]	GO:0019239 deaminase activity;GO:0016787 hydrolase ac	
Hfm1p	Meiosis s	GO:19901[illegible]	GO:00056[illegible]	GO:0000404 heteroduplex DNA loop binding;GO:0032406 M	
putative	Serine/tl	GO:20002[illegible]	GO:00104[illegible]	GO:0004712 protein serine/threonine/tyrosine kinase a	
E2 ubiqui	Ubiquitir	GO:01201[illegible]	GO:19903[illegible]	GO:00431[illegible]	(sce04120)Ubiquitin mediated proteolysis
D-arabinc	NADP+ dep	GO:00442[illegible]	GO:00058[illegible]	GO:0045290 D-arabinose 1-dehydrogenase [NAD(P)+] acti	
bifuncti(	Diacylgly	GO:00066[illegible]	GO:00003[illegible]	GO:00008[illegible]	(sce00561)Glycerolipid metabolism; (sce00564[illegible]
Mho1p	Protein (	GO:00081[illegible]	GO:00056[illegible]	GO:0003674 molecular_function	
Nrp1p	Putative	GO:00081[illegible]	GO:00104[illegible]	GO:0003723 RNA binding;GO:0046872 metal ion binding;G[illegible]	
tetrafunc	Beta subu	GO:00427[illegible]	GO:00058[illegible]	GO:00043[illegible]	(sce)Fatty acid biosynthesis, elongation; (M[illegible]
polyamine	Polyamine	GO:00063[illegible]	GO:00057[illegible]	GO:0004059 aralkylamine N-acetyltransferase activity;[illegible]	
Ylf2p	Protein (	GO:00081[illegible]	GO:00057[illegible]	GO:0005525 GTP binding;GO:0032550 purine ribonucleosi	
Vac7p	Integral	GO:00105[illegible]	GO:00707[illegible]	GO:0003674 molecular_function	
hydroxyme	HMG-CoA r	GO:00066[illegible]	GO:00057[illegible]	GO:00044[illegible]	(sce)C5 isoprenoid biosynthesis, mevalonate [illegible]
hypotheti	Putative	GO:00091[illegible]	GO:00003[illegible]	GO:0016787 hydrolase activity;GO:0003824 catalytic ac	
Sip5p	Protein (	GO:00421[illegible]	GO:00057[illegible]	GO:0003674 molecular_function	
Kre9p	Glycoprot	GO:00060[illegible]	GO:00003[illegible]	GO:0003674 molecular_function	
hypotheti	Protein (	GO:00081[illegible]	GO:00056[illegible]	GO:0003674 molecular_function	
Hsp70 fan	ATPase ir	GO:00066[illegible]	GO:00092[illegible]	GO:00441[illegible]	(sce04213)Longevity regulating pathway - mul[illegible]
mitogen-a	Mitogen-a	GO:00109[illegible]	GO:00425[illegible]	GO:00047[illegible]	(sce04933)AGE-RAGE signaling pathway in diab[illegible]
RNA expor	RNA expor	GO:20007[illegible]	GO:00056[illegible]	GO:00431[illegible]	(sce03013)RNA transport
sphingani	Minor spł	GO:00468[illegible]	GO:00001[illegible]	GO:00084[illegible]	(sce)Sphingosine degradation; (M00100)Sphing[illegible]
Zrg8p	Protein (	GO:00315[illegible]	GO:00059[illegible]	GO:0003674 molecular_function	
thioredo:	Mitochonc	GO:00194[illegible]	GO:00057[illegible]	GO:00047[illegible]	(sce00450)Selenocompound metabolism; (sce002[illegible]
putative	Protein (	GO:00092[illegible]	GO:19036[illegible]	GO:00363[illegible]	(sce00750)Vitamin B6 metabolism
Gat2p	Protein (	GO:00063[illegible]	GO:00056[illegible]	GO:0000981 DNA-binding transcription factor activity,	
hypotheti	Conservec	GO:00081[illegible]	GO:00056[illegible]	GO:0003674 molecular_function	
She3p	Protein a	GO:00075[illegible]	GO:00059[illegible]	GO:1990825 sequence-specific mRNA binding;GO:0003729 [illegible]	
hypotheti	Putative	GO:00081[illegible]	GO:00055[illegible]	GO:0003674 molecular_function	
MIND comp	Essential	GO:00070[illegible]	GO:00008[illegible]	GO:0003674 molecular_function	
Stb2p	Protein t	GO:00081[illegible]	GO:00708[illegible]	GO:0003674 molecular_function	
membrane	Protein i	GO:00329[illegible]	GO:00313[illegible]	GO:0032977 membrane insertase activity;GO:0008565 pro	
E3 ubiqui	E3 ubiqui	GO:00450[illegible]	GO:00057[illegible]	GO:0004386 helicase activity;GO:0004842 ubiquitin-pro	
hexose tı	Hexose tı	GO:19023[illegible]	GO:00058[illegible]	GO:00053[illegible]	(sce04113)Meiosis - yeast
U4/U6-U5	Componen1	GO:00003[illegible]	GO:00465[illegible]	GO:00468[illegible]	(sce03040)Spliceosome
SUMO liga	Highly cc	GO:19906[illegible]	GO:00309[illegible]	GO:0030291 protein serine/threonine kinase inhibitor [illegible]	
hypotheti	Protein (	GO:00081[illegible]	GO:00056[illegible]	GO:0003674 molecular_function	
Tda4p	Putative	GO:00081[illegible]	GO:00057[illegible]	GO:0003674 molecular_function	
acetyl-Cc	3-ketoacy	GO:00101[illegible]	GO:00057[illegible]	GO:00039[illegible]	(sce00592)alpha-Linolenic acid metabolism; ([illegible]
hydroxyac	Cytoplasn	GO:00192[illegible]	GO:00057[illegible]	GO:00044[illegible]	(sce00620)Pyruvate metabolism
glycerol-	Constitut	GO:00061[illegible]	GO:00056[illegible]	GO:00001[illegible]	(sce00561)Glycerolipid metabolism; (sce01100[illegible]
hypotheti	Putative	GO:00081[illegible]	GO:00057[illegible]	GO:0003674 molecular_function	
Tos8p	Homeodoma	GO:00063[illegible]	GO:00007[illegible]	GO:0003682 chromatin binding;GO:0043565 sequence-spec	
protein /	Cytoplasn	GO:00081[illegible]	GO:00057[illegible]	GO:0016787 hydrolase activity;GO:0003824 catalytic ac	
Rpi1p	Transcrip	GO:00092[illegible]	GO:00056[illegible]	GO:0030695 GTPase regulator activity;GO:0060589 nucle[illegible]	
RNA-bindi	Nuclear p	GO:19022[illegible]	GO:00003[illegible]	GO:19901[illegible]	(sce03015)mRNA surveillance pathway; (sce030[illegible]
Imh1p	Protein i	GO:00430[illegible]	GO:00001[illegible]	GO:0003674 molecular_function	
ribosomal	Ribosomal	GO:00000[illegible]	GO:00226[illegible]	GO:00198[illegible]	(sce03010)Ribosome
tyrosyl-I	Tyrosyl-I	GO:00000[illegible]	GO:00057[illegible]	GO:0070260 5'-tyrosyl-DNA phosphodiesterase activity;[illegible]	

Fe(2+) tr	Iron tran	GO:004825	GO:000574	GO:0005381 iron ion transmembrane transporter activit
hypotheti	Protein o	GO:000815	GO:000557	GO:0003674 molecular_function
type 2C p	Type 2C s	GO:000647	GO:000563	GO:0004722 protein serine/threonine phosphatase activ
Prp21p	Subunit o	GO:000039	GO:000568	GO:0003723 RNA binding;GO:0003676 nucleic acid bindin
Hnm1p	Plasma me	GO:190227	GO:000578	GO:1901235 (R)-carnitine transmembrane transporter ac
coproporp	Coproporp	GO:001935	GO:000582	GO:00410((sce00860)Porphyrin and chlorophyll metaboli
Mrx3p	Protein t	GO:000815	GO:000574	GO:0003674 molecular_function
Ksh1p	Essential	GO:000930	GO:000013	GO:0003674 molecular_function
ubiquitin	Component	GO:000651	GO:000015	GO:0034450 ubiquitin-ubiquitin ligase activity;GO:006
membrane	Mitochond	GO:003297	GO:009700	GO:009717 (sce03060)Protein export
Pdp3p	Component	GO:003296	GO:199046	GO:0003682 chromatin binding;GO:0005488 binding;GO:00
proteasom	Essential	GO:004316	GO:003451	GO:000551 (sce03050)Proteasome
TOR compl	Protein i	GO:003193	GO:003193	GO:003029 (sce04136)Autophagy - other; (sce04138)Autoph
putative	Protein i	GO:003254	GO:000576	GO:000373 (sce03010)Ribosome
Fps1p	Aquaglyce	GO:000684	GO:000032	GO:0015254 glycerol channel activity;GO:0015168 glyce
Fmp42p	Putative	GO:005508	GO:000032	GO:0003674 molecular_function
putative	Putative	GO:005508	GO:007194	GO:0022857 transmembrane transporter activity;GO:0005
hypotheti	Protein o	GO:000815	GO:000557	GO:0003674 molecular_function
lysophosp	Lysophosp	GO:003535	GO:000581	GO:0003841 1-acylglycerol-3-phosphate O-acyltransfera
Lso1p	Protein w	GO:005507	GO:000563	GO:0003674 molecular_function
leucine c	Carboxyl	GO:000648	GO:000557	GO:0018423 protein C-terminal leucine carboxyl O-meth
hypotheti	Protein o	GO:000703	GO:000577	GO:0003674 molecular_function
peptidylp	Peptidyl-	GO:000041	GO:000078	GO:0005527 macrolide binding;GO:0003755 peptidyl-prol
malate de	Mitochond	GO:000130	GO:000575	GO:003006 (M00012)Glyoxylate cycle; (sce)Glyoxylate cyc
bifunctio	Farnesyl	GO:003338	GO:000578	GO:000416 (sce00900)Terpenoid backbone biosynthesis; (
transglyc	Chitin tr	GO:000603	GO:000013	GO:0004553 hydrolase activity, hydrolyzing O-glycosyl
1,4-alpha	Glycogen	GO:000597	GO:000573	GO:010275 (sce00500)Starch and sucrose metabolism; (sce
lanostero	Lanostero	GO:000669	GO:000581	GO:000025 (sce00100)Steroid biosynthesis; (sce01130)Bi
Tda6p	Putative	GO:001503	GO:000032	GO:0003674 molecular_function
putative	Putative	GO:000005	GO:003068	GO:000552 (sce03008)Ribosome biogenesis in eukaryotes
bifunctio	Bifunctio	GO:004598	GO:000573	GO:000407 (sce)Uridine monophosphate biosynthesis, glu
mitochond	Mitochond	GO:003254	GO:000576	GO:0003735 structural constituent of ribosome;GO:0005
bifunctio	Indole-3-	GO:000016	GO:000573	GO:000442 (sce)Tryptophan biosynthesis, chorismate =>
serine/th	Protein k	GO:007147	GO:000563	GO:0004693 cyclin-dependent protein serine/threonine
putative	Putative	GO:001631	GO:000563	GO:0033883 pyridoxal phosphatase activity;GO:0016791
methylene	Major iso	GO:000908	GO:000573	GO:000448 (sce00670)One carbon pool by folate; (sce012
alpha-tub	Alpha-tub	GO:009886	GO:000588	GO:0005200 structural constituent of cytoskeleton;GO:
transcrip	Transcrip	GO:000043	GO:000564	GO:0001227 DNA-binding transcription repressor activi
ribosomal	Ribosomal	GO:000218	GO:002262	GO:000373 (sce03010)Ribosome
DNA-direc	RNA polym	GO:004279	GO:000573	GO:000105 (sce03020)RNA polymerase; (sce00240)Pyrimidin
Zeo1p	Periphera	GO:003150	GO:000574	GO:0003674 molecular_function
GTPase-ac	GTPase-ac	GO:000075	GO:000588	GO:000509 (sce04011)MAPK signaling pathway - yeast
polyubiqu	Substrate	GO:007162	GO:000083	GO:003159 (sce04141)Protein processing in endoplasmic
hypotheti	Putative protein of unknown function; the authentic, non-tagged protein is detec			
fructose-	Fructose	GO:000609	GO:000927	GO:000433 (M00003)Gluconeogenesis, oxaloacetate => fru
Mnd2p	Subunit o	GO:190242	GO:000568	GO:199094 (sce04113)Meiosis - yeast
valine--t	Mitochond	GO:000643	GO:000573	GO:000483 (sce00970)Aminoacyl-tRNA biosynthesis
steryl de	Sterol de	GO:003421	GO:000581	GO:0034084 steryl deacetylase activity;GO:0019213 dea
Gep7p	Protein o	GO:000815	GO:003196	GO:0003674 molecular_function
S-methylm	High-affi	GO:001580	GO:000578	GO:0000100 S-methylmethionine transmembrane transport
hexose tr	Putative	GO:001579	GO:000588	GO:001557 (sce04113)Meiosis - yeast
Amn1p	Protein r	GO:200104	GO:001900	GO:0031267 small GTPase binding;GO:0004842 ubiquitin-
mitochond	Mitochond	GO:003254	GO:000576	GO:000373 (sce03010)Ribosome
Aim45p	Putative	GO:003353	GO:000575	GO:0050660 flavin adenine dinucleotide binding;GO:000

putative	Protein i	GO:00067	GO:00057	GO:00086	(M00128)Ubiquinone biosynthesis, eukaryotes,
glutamyl-	Subunit c	GO:00706	GO:00309	GO:00505	(sce00970)Aminoacyl-tRNA biosynthesis; (sce0
Dsk2p	Nuclear-e	GO:00304	GO:00058	GO:00315	(sce04141)Protein processing in endoplasmic
phosphogl	Phosphogl	GO:00059	GO:00058	GO:00046	(M00549)Nucleotide sugar biosynthesis, gluco
Oxr1p	Protein c	GO:00345	GO:00057	GO:0003674	molecular_function
inorganic	Mitochond	GO:00090	GO:00057	GO:00044	(sce00190)Oxidative phosphorylation
Rho famil	Guanine n	GO:0090630 activa		GO:00050	(sce04011)MAPK signaling pathway - yeast
calcineur	Calcineur	GO:00007	GO:00059	GO:00331	(Human)(hsa05014)Amyotrophic lateral scleros
Cgr1p	Protein i	GO:00063	GO:00306	GO:0003674	molecular_function
TRAPP sub	Core comp	GO:00068	GO:19900	GO:0017112	Rab guanyl-nucleotide exchange factor acti
histone H	Histone H	GO:00063	GO:00312	GO:00469	(Human)(hsa05322)Systemic lupus erythematosu
Tom6p	Component	GO:00700	GO:00057	GO:0008320	protein transmembrane transporter activity
Sas2p	Histone a	GO:00439	GO:00332	GO:0046972	histone acetyltransferase activity (H4-K16
hypotheti	Putative	GO:00081	GO:00055	GO:0003674	molecular_function
Sap30p	Component	GO:20002	GO:00336	GO:0004407	histone deacetylase activity;GO:0033558 pr
hypotheti	Putative	GO:00081	GO:00057	GO:0003674	molecular_function
Cmr3p	Putative	GO:00081	GO:00055	GO:0043565	sequence-specific DNA binding;GO:0003677 D
chromatin	Transcrip	GO:00752	GO:00056	GO:0003713	transcription coactivator activity;GO:0003
Ddr2p	Multi-str	GO:00703	GO:00003	GO:0003674	molecular_function
DNA-direc	RNA polym	GO:00427	GO:00057	GO:00010	(sce03020)RNA polymerase; (sce00240)Pyrimidi
GTP cyclo	GTP cyclo	GO:00092	GO:00056	GO:00039	(sce00740)Riboflavin metabolism; (sce01110)B
aspartyl	Aspartic	GO:00315	GO:00092	GO:0004190	aspartic-type endopeptidase activity;GO:00
Ups3p	Mitochond	GO:00705	GO:00313	GO:1990050	phosphatidic acid transporter activity;GO:
Vhc1p	Vacuolar	GO:19905	GO:00311	GO:0015379	potassium:chloride symporter activity;GO:0
protein k	Serine/th	GO:00158	GO:00058	GO:0004674	protein serine/threonine kinase activity;G
Pex5p	Peroxisom	GO:00165	GO:19904	GO:00050	(sce04146)Peroxisome
hypotheti	Putative	GO:00081	GO:00160	GO:0004386	helicase activity;GO:0017111 nucleoside-tr
SCF ubiqu	RING-H2 d	GO:00311	GO:00353	GO:00197	(sce03420)Nucleotide excision repair; (sce04
S-adenosy	S-adenosy	GO:00459	GO:00057	GO:0016279	protein-lysine N-methyltransferase activit
Yng1p	Subunit c	GO:00329	GO:19904	GO:0004402	histone acetyltransferase activity;GO:0061
type II n	Type II n	GO:00459	GO:00056	GO:0016657	oxidoreductase activity, acting on NAD(P)H
hypotheti	Putative	GO:00081	GO:00058	GO:0003674	molecular_function
Sft2p	Tetra-spa	GO:00421	GO:00001	GO:0003674	molecular_function
Spl2p	Protein w	GO:00066	GO:00057	GO:0004860	protein kinase inhibitor activity;GO:00192
membrane	Protein i	GO:00321	GO:19908	GO:0003674	molecular_function
putative	Putative	GO:00300	GO:00056	GO:0004674	protein serine/threonine kinase activity;G
acid phos	One of th	GO:00163	GO:00092	GO:00039	(sce00740)Riboflavin metabolism; (sce00730)T
ubiquitin	Activator	GO:19057	GO:00335	GO:19907	(sce04120)Ubiquitin mediated proteolysis; (s
U2-type s	Protein i	GO:00003	GO:00056	GO:0003674	molecular_function
hypotheti	Putative	GO:00081	GO:00057	GO:0003674	molecular_function
glutathio	Glutathio	GO:00067	GO:00058	GO:00043	(sce00480)Glutathione metabolism; (sce00270)
Cos3p	Endosomal	GO:00433	GO:00003	GO:0003674	molecular_function
RNA polym	Highly co	GO:00615	GO:00057	GO:0000994	RNA polymerase III core binding;GO:0001030
N-acetylp	Protein w	GO:00702	GO:00313	GO:0102200	N-acetylphosphatidylethanolamine-hydrolysi
Y' elemen	Helicase	GO:00007	GO:00056	GO:0003678	DNA helicase activity;GO:0004386 helicase
putative	Monoacylg	GO:00517	GO:00055	GO:0034338	short-chain carboxylesterase activity;GO:0
Lcl1p	Putative	GO:00081	GO:00312	GO:0003674	molecular_function
Irc18p	Protein i	GO:00304	GO:00003	GO:0003674	molecular_function
inositol	Inositol	GO:00060	GO:00058	GO:0000830	inositol hexakisphosphate 4-kinase activit
DNA polym	Subunit c	GO:00708	GO:00086	GO:00083	(sce03410)Base excision repair; (sce03030)DN
glutamate	Gamma glu	GO:00067	(Human)GO	GO:00043	(sce00480)Glutathione metabolism; (sce00270)
GDP-manno	Golgi GDP	GO:19905	GO:00306	GO:0005458	GDP-mannose transmembrane transporter acti
Arc40p	Subunit c	GO:00343	GO:00304	GO:00431	(sce04144)Endocytosis
Nem1-Spo7	Putative	GO:19037	GO:00715	GO:0004721	phosphoprotein phosphatase activity;GO:001

Crp1p	Protein t	GO:00081	GO:00056	GO:0003677	DNA binding;GO:0003676 nucleic acid bindin
cleavage	Integral	GO:00987	GO:00058	GO:00037	(sce03015)mRNA surveillance pathway
Thi2p	Transcri	GO:00901	GO:00056	GO:0000978	RNA polymerase II proximal promoter sequen
non-speci	Protein k	GO:00064	GO:00364	GO:0004674	protein serine/threonine kinase activity;G
glutathio	Proton:g	GO:00347	GO:00057	GO:0015343	siderophore transmembrane transporter acti
acetylorn	Acetylorn	GO:00065	GO:00057	GO:00039	(M00028)Ornithine biosynthesis, glutamate =>
Ran GTPas	GTP bindi	GO:00066	GO:00160	GO:00039	(sce03008)Ribosome biogenesis in eukaryotes;
E2 ubiqui	E2 ubiqui	GO:00109	GO:00313	GO:00616	(sce04120)Ubiquitin mediated proteolysis
E2-like (	Conserved	GO:00448	GO:00340	GO:00197	(sce04138)Autophagy - yeast
Shr3p	Endoplasm	GO:00068	GO:00433	GO:0051082	unfolded protein binding;GO:0005515 protei
Clc1p	Clathrin	GO:00482	GO:00301	GO:0032050	clathrin heavy chain binding;GO:0030276 cl
Hrp1p	Subunit o	GO:00724	GO:00104	GO:00037	(sce03015)mRNA surveillance pathway
pyridoxan	Pyridoxin	GO:00094	GO:00057	GO:00047	(sce00750)Vitamin B6 metabolism; (sce01100)M
Rts3p	Putative	GO:00081	GO:00056	GO:0003674	molecular_function
histone-b	Defining	GO:00458	GO:00349	GO:0035064	methylated histone binding;GO:0140034 meth
ribosomal	Ribosomal	GO:00021	GO:00306	GO:00037	(sce03010)Ribosome
Med2p	Subunit o	GO:00511	GO:00708	GO:0001135	RNA polymerase II transcription regulator
Svp26p	Integral	GO:00450	GO:00301	GO:0097020	COPII adaptor activity;GO:0030674 protein
hypotheti	Haploid-s	GO:00081	GO:00055	GO:0003674	molecular_function
Hsp70 fam	Molecular	GO:00312	GO:00058	GO:00007	(sce04141)Protein processing in endoplasmic
DNA ligas	DNA ligas	GO:00511	GO:00328	GO:00039	(sce03450)Non-homologous end-joining
CDP-diacy	Phosphati	GO:00066	GO:00057	GO:00038	(M00093)Phosphatidylethanolamine (PE) biosyn
Pzf1p	Transcri	GO:00427	GO:00056	GO:0001002	RNA polymerase III type 1 promoter sequenc
Hgh1p	Nonessent	GO:00081	GO:00056	GO:0003674	molecular_function
None	None	None	None	None	None
Arf famil	ARF famil	GO:00034	GO:00301	GO:00039	(sce04141)Protein processing in endoplasmic
Irs4p	EH domain	GO:00001	GO:00004	GO:0003674	molecular_function
Sps4p	Protein w	GO:00304	GO:00058	GO:00036	(sce04113)Meiosis - yeast
bifunctio	Bifunctio	GO:00094	GO:00058	GO:00041	(M00022)Shikimate pathway, phosphoenolpyruva
ATP synth	Assembly	GO:00336	GO:00057	GO:0019904	protein domain specific binding;GO:0005515
Pbi2p	Cytosolic	GO:00421	GO:00058	GO:0004867	serine-type endopeptidase inhibitor activi
carnitine	Carnitine	GO:00094	GO:19904	GO:00040	(sce04146)Peroxisome
iron-bind	Conserved	GO:00020	GO:00057	GO:0008198	ferrous iron binding;GO:0051537 2 iron, 2
Stb3p	Ribosomal	GO:00004	GO:00056	GO:0001228	DNA-binding transcription activator activi
Ump1p	Chaperone	GO:00432	GO:00056	GO:00036	(sce03050)Proteasome
hypotheti	Putative	GO:00004	GO:00055	GO:0003674	molecular_function
hypotheti	Putative	GO:00081	GO:00055	GO:0003674	molecular_function
putative	Putative	GO:00065	GO:00057	GO:0008237	metallopeptidase activity;GO:0070011 pepti
ribosomal	Ribosomal	GO:00422	GO:00226	GO:00037	(sce03010)Ribosome
Mtc2p	Protein o	GO:00081	GO:00055	GO:0003674	molecular_function
hypotheti	Putative	GO:00081	GO:00003	GO:0003674	molecular_function
Snm1p	Ribonucle	GO:00305	GO:00056	GO:00421	(sce03008)Ribosome biogenesis in eukaryotes
homocitra	Homocitra	GO:00198	GO:00057	GO:00044	(sce)Lysine biosynthesis, 2-oxoglutarate =>
8-oxoguan	Nuclear a	GO:00709	GO:00315	GO:00340	(sce03410)Base excision repair
ribonucle	Vacuolar	GO:00905	GO:00057	GO:0033897	ribonuclease T2 activity;GO:0016892 endori
Rab geran	Alpha sub	GO:00183	GO:00059	GO:0004663	Rab geranylgeranyltransferase activity;GO:
tRNA (gua	Catalytic	GO:00304	GO:00435	GO:0004809	tRNA (guanine-N2-)-methyltransferase activ
mitochond	Mitochond	GO:00325	GO:00057	GO:0003735	structural constituent of ribosome;GO:0005
Mup3p	Low affin	GO:19036	GO:00003	GO:0015191	L-methionine transmembrane transporter act
hypotheti	Putative	GO:00081	GO:00055	GO:0003674	molecular_function
SUN famil	Mitochond	GO:00013	GO:00313	GO:0016798	hydrolase activity, acting on glycosyl bon
Nab6p	Putative	GO:00081	GO:00104	GO:0003729	mRNA binding;GO:0003723 RNA binding;GO:000
hypotheti	Putative	GO:00081	GO:00055	GO:0043565	sequence-specific DNA binding;GO:0003677 D
transcrip	Basic leu	GO:00106	GO:00056	GO:0000981	DNA-binding transcription factor activity,

Azf1p Zinc-fing GO:00602 GO:00058 GO:0000978 RNA polymerase II proximal promoter sequen
mitogen-a Stress-in GO:00109 GO:00056 GO:00335 (sce04011)MAPK signaling pathway - yeast
ribosomal Ribosomal GO:00004 GO:00306 GO:00037 (sce03010)Ribosome
adenosylm S-adenosy GO:00065 GO:00058 GO:00040 (sce)Methionine salvage pathway; (M00034)Met
rRNA-bind Constitue GO:00009 GO:00306 GO:0019843 rRNA binding;GO:0003723 RNA binding;GO:000
metalloen Metalloen GO:00356 GO:00057 GO:0004222 metalloendopeptidase activity;GO:0008237 m
Tom7p Component GO:00450 GO:00057 GO:0008320 protein transmembrane transporter activity
putative ATP-bind GO:00163 GO:00056 GO:00088 (sce00561)Glycerolipid metabolism; (sce00630)
tryptopha Tryptopha GO:00001 GO:00056 GO:00048 (sce)Tryptophan biosynthesis, chorismate =>
Ccw14p Covalent GO:00315 GO:0009277 fungal-type cell wall;GO:0005618 cell wall;GO:00312
Rnh202p Ribonucle GO:00431 GO:00322 GO:0004523 RNA-DNA hybrid ribonuclease activity;GO:00
ATP-bind ABC type GO:00157 GO:00003 GO:0015127 bilirubin transmembrane transporter activi
AAA famil ATPase of GO:00422 GO:00306 GO:00168 (sce03008)Ribosome biogenesis in eukaryotes
Ccm1p Mitochon GO:00009 GO:00057 GO:0019843 rRNA binding;GO:0003723 RNA binding;GO:000
mitogen-a Mitogen-a GO:00613 GO:00056 GO:00047 (sce04933)AGE-RAGE signaling pathway in diab
hypotheti Protein GO:00081 GO:00059 GO:0003674 molecular_function
Nba1p Protein GO:00081 GO:00059 GO:0043022 ribosome binding;GO:0043021 ribonucleoprot
arginine/ Arginine/ GO:00059 GO:00056 GO:0070006 metalloaminopeptidase activity;GO:0004177
peptide-m Methionin GO:00345 GO:00056 GO:0036456 L-methionine-(S)-S-oxide reductase activit
None None None None None None
transcrip Transcrip GO:00611 GO:00056 GO:0003714 transcription corepressor activity;GO:0003
hypotheti Putative GO:00081 GO:00055 GO:0003674 molecular_function
Rtg3p bHLH/Zip GO:00714 GO:00056 GO:0001085 RNA polymerase II transcription factor bin
Gep3p Protein GO:00304 GO:00057 GO:0005525 GTP binding;GO:0032550 purine ribonucleosi
Ccc1p Vacuolar GO:00068 GO:00003 GO:0015093 ferrous iron transmembrane transporter act
Blm10p Proteasom GO:19902 GO:00345 GO:00705 (sce03050)Proteasome
electron Component GO:19012 GO:00057 GO:0051537 2 iron, 2 sulfur cluster binding;GO:005153
Vhr2p Non-essen GO:00063 GO:00056 GO:0003677 DNA binding;GO:0003676 nucleic acid bindin
hypotheti Protein GO:00081 GO:00057 GO:0003676 nucleic acid binding;GO:1901363 heterocycl
protein k Protein k GO:00109 GO:00055 GO:0004694 eukaryotic translation initiation factor 2
Apl1p Beta-adap GO:00068 GO:00301 GO:0030276 clathrin binding;GO:0005515 protein bindin
kinetoch Component GO:00070 GO:0031262 Ndc80 complex;GO:0000778 condensed nuclear chromoso
high-mob High-mobi GO:00011 GO:00007 GO:0008301 DNA binding, bending;GO:0031491 nucleosome
Utp4p Subunit GO:00459 GO:00344 GO:00036 (sce03008)Ribosome biogenesis in eukaryotes
ubiquitin Ubiquitin GO:00165 GO:00057 GO:0004843 thiol-dependent ubiquitin-specific proteas
hypotheti Putative protein of unknown function; the authentic, non-tagged protein is detec
hypotheti Putative GO:00081 GO:00057 GO:0003674 molecular_function
Rab GTPa Rab GTPa GO:00706 GO:00001 GO:0017137 Rab GTPase binding;GO:0005096 GTPase activ
MutS fam Protein GO:00510 GO:00323 GO:0032143 single thymine insertion binding;GO:003213
mitochon Mitochon GO:00004 GO:00057 GO:00037 (sce03010)Ribosome
mRNA (N6- mRNA N6-a GO:20002 GO:00363 GO:0001734 mRNA (N6-adenosine)-methyltransferase acti
adenosine Adenosine GO:00442 GO:00058 GO:00040 (sce00230)Purine metabolism; (sce01100)Metab
Mpm1p Mitochon GO:00081 GO:00057 GO:0003674 molecular_function
putative Protein k GO:00517 GO:00056 GO:00046 (sce04011)MAPK signaling pathway - yeast
Uip4p Protein GO:00081 GO:00057 GO:0003674 molecular_function
Spo19p Meiosis-s GO:00705 GO:00056 GO:0003674 molecular_function
None None None None None None
hypotheti Protein GO:00150 GO:00058 GO:0043022 ribosome binding;GO:0043021 ribonucleoprot
SUMO prot Peptidase GO:00070 GO:00056 GO:0070140 SUMO-specific isopeptidase activity;GO:007
Bem4p Protein GO:00192 GO:00058 GO:0005096 GTPase activator activity;GO:0030695 GTPas
TFIIH/NEF Subunit GO:00457 GO:00709 GO:00615 (sce03022)Basal transcription factors; (sce0
tropomyo Major iso GO:00071 GO:00001 GO:0003786 actin lateral binding;GO:0051015 actin fil
polyphosp Polyphosp GO:19019 GO:00056 GO:1990174 phosphodiesterase decapping endonuclease a
putative Ubiquitin GO:00109 GO:00057 GO:19048 (sce04120)Ubiquitin mediated proteolysis

Tsr2p	Protein v	GO:00004(	GO:0005€	GO:0003674	molecular_function
hypotheti	Putative protein of unknown function; identified by homology; green fluorescent				
mRNA spli	RNA splic	GO:00003{	GO:00710	GO:003062	(sce03040)Spliceosome
Cbp6p	Mitochonc	GO:00345{	GO:00616	GO:0043022	ribosome binding;GO:0043021 ribonucleoprot
SAGA comp	Subunit c	GO:00165	GO:00001	GO:0017025	TBP-class protein binding;GO:0003712 trans
Slx9p	Protein i	GO:00000{	GO:00306	GO:0003674	molecular_function
[Histone	JmjC doma	GO:00705	GO:00056	GO:0051864	histone demethylase activity (H3-K36 speci
Sol2p	Protein v	GO:00090	GO:00058	GO:00170	(M00006)Pentose phosphate pathway, oxidative
Rcn2p	Protein c	GO:00081	GO:00057	GO:0003674	molecular_function
septin SI	Componen	GO:00321	GO:00001	GO:0032947	protein-containing complex scaffold activi
nucleopor	Nucleopor	GO:00353	GO:00446	GO:0008536	Ran GTPase binding;GO:0017056 structural c
fructose-	Fructose-	GO:00060	GO:00058	GO:00043	(sce00051)Fructose and mannose metabolism
Mlc2p	Regulato	GO:00009	GO:00001	GO:0032038	myosin II heavy chain binding;GO:0045159 m
Clf1p	Member o	GO:00003	GO:00710	GO:00003	(sce03040)Spliceosome
putative	Ubiquitin	GO:00311	GO:00056	GO:00616	(sce04120)Ubiquitin mediated proteolysis
long-cha	Acyl-CoA-	GO:00427	GO:00055	GO:00360	(Human)(hsa03320)PPAR signaling pathway
Prp40p	U1 snRNP	GO:00452	GO:00056	GO:00037	(sce03040)Spliceosome
Atg20p	Sorting n	GO:00344	GO:00340	GO:0032266	phosphatidylinositol-3-phosphate binding;G
oligopept	Proton-co	GO:00356	GO:00058	GO:0005427	proton-dependent oligopeptide secondary ac
phosphopa	Subunit c	GO:00159	GO:00715	GO:00046	(sce)Coenzyme A biosynthesis, pantothenate =
hydroxyme	Porphobil	GO:00181	GO:00056	GO:00044	(sce00860)Porphyrin and chlorophyll metaboli
seripaupe	Member o	GO:00315	GO:00092	GO:0005199	structural constituent of cell wall;GO:000
Tda11p	Putative	GO:00081	GO:00057	GO:0003674	molecular_function
anaphase	Subunit c	GO:00311	GO:00056	GO:00616	(sce04120)Ubiquitin mediated proteolysis; (s
Rud3p	Golgi ma	GO:00068	GO:00057	GO:0051020	GTPase binding;GO:0019899 enzyme binding;G
ubiquitin	Deubiqui	GO:19041	GO:00058	GO:19042	(sce04141)Protein processing in endoplasmic
Bls1p	Subunit c	GO:00070	GO:00310	GO:0003674	molecular_function
IMP dehy	Inosine n	GO:00061	GO:00058	GO:00039	(sce)Guanine ribonucleotide biosynthesis IMP
Tah11p	DNA repli	GO:00062	GO:00056	GO:00036	(sce04111)Cell cycle - yeast; (sce04113)Meio
tubulin-b	Part of t	GO:00070	GO:00162	GO:0015631	tubulin binding;GO:0008092 cytoskeletal pr
pseudouri	tRNA:pseu	GO:00311	GO:00056	GO:0106029	tRNA pseudouridine synthase activity;GO:00
hypotheti	Putative	GO:00081	GO:00057	GO:0003674	molecular_function
Yct1p	High-affi	GO:19037	GO:00057	GO:0033229	cysteine transmembrane transporter activit
DNA damag	DNA damag	GO:00062	GO:00058	GO:0003924	GTPase activity;GO:0016887 ATPase activity
flavin-li	Flavin-li	GO:00450	GO:00057	GO:0016971	flavin-linked sulfhydryl oxidase activity;
CTP synth	Minor CTF	GO:00442	GO:00972	GO:00038	(sce)Pyrimidine ribonucleotide biosynthesis,
cleavage	Subunit c	GO:00987	GO:0005847 mRNA c	(sce03015)mRNA surveillance pathway	
phosphati	Protein t	GO:00433	GO:00058	GO:0070273	phosphatidylinositol-4-phosphate binding;G
mitochonc	Mitochonc	GO:00325	GO:00057	GO:0003735	structural constituent of ribosome;GO:0005
Ioc2p	Subunit c	GO:00063	GO:00364	GO:0031491	nucleosome binding;GO:0016887 ATPase activ
Lam6p	Sterol t	GO:00323	GO:19908	GO:0015248	sterol transporter activity;GO:0005319 lip
Spg4p	Protein i	GO:00081	GO:00055	GO:0003674	molecular_function
Met32p	Zinc-fing	GO:00313	GO:00056	GO:0001102	RNA polymerase II activating transcription
Isf1p	Serine-ri	GO:00090	GO:00055	GO:0003674	molecular_function
Mtc1p	Protein c	GO:00081	GO:00301	GO:0043022	ribosome binding;GO:0043021 ribonucleoprot
5'-(N(7)-	m(7)Gppp	GO:00002	GO:00009	GO:0050072	m7G(5')pppN diphosphatase activity;GO:0000
Pib2p	Protein c	GO:00081	GO:00057	GO:0046872	metal ion binding;GO:0043169 cation bindin
Pam1p	Essentia	GO:00071	GO:00059	GO:0003674	molecular_function
hypotheti	Putative	GO:00081	GO:00055	GO:0003674	molecular_function
mitochonc	Mitochonc	GO:00325	GO:00057	GO:0003735	structural constituent of ribosome;GO:0005
hypotheti	Putative	GO:00081	GO:00055	GO:0003674	molecular_function
Sld2p	Single-s	GO:19029	GO:00312	GO:0003688	DNA replication origin binding;GO:0003697
Oca2p	Protein c	GO:00353	GO:00057	GO:0004725	protein tyrosine phosphatase activity;GO:0
Jen1p	Monocarbo	GO:00068	GO:00003	GO:0097079	selenite:proton symporter activity;GO:0015

phosphati	Phosphati	GO:200115	GO:003427	GO:001630	(sce00562)Inositol phosphate metabolism; (sc
hypotheti	Putative	GO:000815	GO:000563	GO:0003674	molecular_function
histone H	Histone H	GO:007091	GO:004350	GO:003149	(Human)(hsa05322)Systemic lupus erythematosu
mRNA-bind	Protein c	GO:001079	GO:003247	GO:0003729	mRNA binding;GO:0003723 RNA binding;GO:000
Aim39p	Protein c	GO:000815	GO:003196	GO:0003674	molecular_function
Hua1p	Cytoplasm	GO:000815	GO:000573	GO:0003674	molecular_function
DUP240 fa	Member of	GO:001605	GO:000579	GO:0003674	molecular_function
Cox14p	Mitochond	GO:007013	GO:000574	GO:0003674	molecular_function
Pgd1p	Subunit c	GO:005112	GO:007084	GO:0001135	RNA polymerase II transcription regulator r
Ptr2p	Integral	GO:003544	GO:000032	GO:004293	(Human)(hsa04974)Protein digestion and absorp
glutamate	Glutamyl-	GO:000642	GO:001710	GO:000481	(sce00860)Porphyrin and chlorophyll metaboli
Ubx2p	Bridging	GO:003043	GO:000083	GO:003067	(sce04141)Protein processing in endoplasmic r
Obg-like	P-loop AT	GO:000815	GO:000573	GO:0005525	GTP binding;GO:0032550 purine ribonucleosi
palmitoyl	Subunit c	GO:001823	GO:003017	GO:0019706	protein-cysteine S-palmitoyltransferase ac
dolichyl-	Subunit c	GO:001827	GO:000825	GO:000457	(M00072)N-glycosylation by oligosaccharyltran
Sif2p	WD40 repe	GO:003193	GO:003496	GO:0003714	transcription corepressor activity;GO:0003
hypotheti	Putative	GO:011009	GO:001602	GO:0004022	alcohol dehydrogenase (NAD) activity;GO:00
putative	Putative	GO:000046	GO:000573	GO:0004004	ATP-dependent RNA helicase activity;GO:000
Ies1p	Subunit c	GO:004276	GO:003101	GO:0003674	molecular_function
Fyv8p	Protein c	GO:000815	GO:000563	GO:0003674	molecular_function
flavin-de	FMN-depen	GO:004299	GO:000582	GO:0052874	FMN reductase (NADH) activity;GO:0052873 F
ubiquitin	Subunit c	GO:004316	GO:004469	GO:0061630	ubiquitin protein ligase activity;GO:00616
hypotheti	Protein c	GO:000836	GO:001602	GO:0003674	molecular_function
Vts1p	Flap-stru	GO:003207	GO:000093	GO:0070336	flap-structured DNA binding;GO:0030371 tra
mRNA-bind	Translati	GO:006023	GO:000017	GO:0000175	3’-5’-exoribonuclease activity;GO:0000900
Aim3p	Protein t	GO:005101	GO:003047	GO:0003674	molecular_function
Hsp70 fam	Cytoplasm	GO:000005	GO:000584	GO:0044183	protein binding involved in protein foldin
6-phospho	Beta subu	GO:006162	GO:000594	GO:000387	(sce00051)Fructose and mannose metabolism; (
Skg3p	Protein c	GO:003214	GO:000593	GO:000467	(sce03018)RNA degradation
putative	Protein c	GO:003150	GO:000927	GO:0016798	hydrolase activity, acting on glycosyl bon
Tma108p	Ribosome-	GO:200076	GO:000585	GO:1990593	nascent polypeptide-associated complex bin
hypotheti	High osmo	GO:000815	GO:001602	GO:0003674	molecular_function
hypotheti	Protein c	GO:000815	GO:000032	GO:0003674	molecular_function
Edc1p	RNA-bindi	GO:003205	GO:009874	GO:0003729	mRNA binding;GO:0003723 RNA binding;GO:000
tubulin-d	Kinesin-r	GO:007046	GO:006167	GO:0070463	tubulin-dependent ATPase activity;GO:00085
ATPase-st	Protein i	GO:007146	GO:000573	GO:0003674	molecular_function
Ntr2p	Essential	GO:000039	GO:007100	GO:0003674	molecular_function
Scp160p	Essential	GO:000075	GO:000032	GO:0001965	G-protein alpha-subunit binding;GO:0003729
isocitrat	Subunit c	GO:000653	GO:000596	GO:000444	(M00010)Citrate cycle, first carbon oxidation
hypotheti	Putative	GO:000815	GO:000557	GO:0003674	molecular_function
phosphati	Phosphati	GO:001602	GO:007031	GO:000460	(M00093)Phosphatidylethanolamine (PE) biosyn
hypotheti	F-box pro	GO:003114	GO:001900	GO:0030674	protein binding, bridging;GO:0004842 ubiqu
phosphogl	Phosphata	GO:001631	GO:000563	GO:001679	(M00002)Glycolysis, core module involving thr
Csm4p	Protein r	GO:004514	GO:000078	GO:0003674	molecular_function
Pbi1p	Putative	GO:004249	GO:000557	GO:0008080	N-acetyltransferase activity;GO:0016410 N-a
U2 snRNP	Component	GO:000034	GO:007027	GO:0000384	first spliceosomal transesterification act
carbonate	Carbonic	GO:007124	GO:000575	GO:000408	(sce00910)Nitrogen metabolism
Rim8p	Protein i	GO:000140	GO:000989	GO:0003674	molecular_function
ribosome	Nucleolar	GO:000047	GO:003069	GO:0003674	molecular_function
NADPH deh	Conserved	GO:000691	GO:000573	GO:0018548	pentaerythritol trinitrate reductase activ
Xrs2p	Protein r	GO:009755	GO:000563	GO:000369	(sce03450)Non-homologous end-joining; (sce034
cationic	Putative	GO:003448	GO:007162	GO:0015174	basic amino acid transmembrane transporter
putative	Putative	GO:000927	GO:001602	GO:0003674	molecular_function
Yhm2p	Citrate a	GO:000684	GO:004264	GO:0005371	tricarboxylate secondary active transmembra

Vps53p	Componen	GO:00901	GO:00009	GO:0003674	molecular_function
endo-1,3	Daughter	GO:00009	GO:00304	GO:0052862	glucan endo-1,4-beta-glucanase activity, C
peroxin I	Peroxisor	GO:00165	GO:19904	GO:00306	(sce04146)Peroxisome
zinc meta	Highly co	GO:00714	GO:00056	GO:00042	(sce00900)Terpenoid backbone biosynthesis; (
Ume1p	Componen	GO:00319	GO:00336	GO:0003714	transcription corepressor activity;GO:0003
putative	Putative	GO:00000	GO:00306	GO:00039	(sce03008)Ribosome biogenesis in eukaryotes
succinate	Flavoprot	GO:00061	GO:00452	GO:00081	(sce)Citrate cycle, second carbon oxidation,
hypotheti	Putative	GO:00081	GO:00055	GO:0003674	molecular_function
ribonucle	Subunit c	GO:00349	GO:00056	GO:00045	(sce03013)RNA transport
Acm1p	Pseudosub	GO:19046	GO:00056	GO:0055105	ubiquitin-protein transferase inhibitor ac
Rgc1p	Putative	GO:00903	GO:00057	GO:0016247	channel regulator activity;GO:0098772 mole
hypotheti	Putative	GO:00081	GO:00055	GO:0003674	molecular_function
Utp5p	Subunit c	GO:00459	GO:00344	GO:00036	(sce03008)Ribosome biogenesis in eukaryotes
SNAP rece	Syntaxin-	GO:00514	GO:00003	GO:00054	(sce04130)SNARE interactions in vesicular tra
Esl2p	hEST1A/B	GO:00081	GO:19904	GO:0043022	ribosome binding;GO:0043021 ribonucleoprot
hypotheti	Putative	GO:00081	GO:00160	GO:0003674	molecular_function
Golgi tra	Componen	GO:00322	GO:00171	GO:0003674	molecular_function
Syp1p	Negative	GO:00001	GO:00001	GO:0004857	enzyme inhibitor activity;GO:0030234 enzym
Pho91p	Low-affir	GO:20001	GO:19908	GO:0015169	glycerol-3-phosphate transmembrane transpo
Sli15p	Subunit c	GO:00321	GO:00058	GO:0030295	protein kinase activator activity;GO:0019
hypotheti	Mitochon	GO:00081	GO:00057	GO:0003674	molecular_function
mitochon	Mitochon	GO:00325	GO:00057	GO:0003735	structural constituent of ribosome;GO:0005
oxysterol	Member of	GO:00014	GO:00325	GO:0008142	oxysterol binding;GO:0005545 1-phosphatidy
Prp11p	Subunit c	GO:00002	GO:00056	GO:00082	(sce03040)Spliceosome
Rox3p	Subunit c	GO:00011	GO:00708	GO:0001135	RNA polymerase II transcription regulator
Ubx3p	Clathrin-	GO:00335	GO:00446	GO:0030276	clathrin binding;GO:0005515 protein bindin
hypotheti	Putative	GO:00081	GO:00055	GO:0003674	molecular_function
Irr1p	Subunit c	GO:00070	GO:00349	GO:00036	(sce04111)Cell cycle - yeast; (sce04113)Meio
ribosoma	Ribosoma	GO:00422	GO:00226	GO:00037	(sce03010)Ribosome
import mo	Subunit c	GO:00301	GO:00014	GO:0019904	protein domain specific binding;GO:0005515
hypotheti	Putative	GO:00081	GO:00055	GO:0003674	molecular_function
hypotheti	Protein c	GO:00081	GO:00056	GO:0003674	molecular_function
putative	Putative	GO:00315	GO:00092	GO:0004190	aspartic-type endopeptidase activity;GO:00
putative	Protein w	GO:00195	GO:00056	GO:00438	(sce)Methionine salvage pathway; (M00034)Met
Grh1p	Acetylate	GO:00070	GO:00058	GO:0044877	protein-containing complex binding;GO:0005
DNA-direc	RNA polym	GO:00427	GO:00057	GO:00010	(sce03020)RNA polymerase; (sce00240)Pyrimidi
bis(5'-ac	Dinucleos	GO:00091	GO:00057	GO:00477	(sce00230)Purine metabolism
Sna3p	Protein i	GO:00325	GO:00003	GO:0031625	ubiquitin protein ligase binding;GO:004438
putative	Part of e	GO:00063	GO:00300	GO:00168	(sce03018)RNA degradation
hypotheti	Putative	GO:00081	GO:00055	GO:0003674	molecular_function
Xbp1p	Transcrip	GO:00310	GO:00056	GO:0000981	DNA-binding transcription factor activity,
Frt1p	Tail-anch	GO:00714	GO:00057	GO:0003674	molecular_function
Rtt103p	Protein i	GO:00105	GO:00165	GO:0000993	RNA polymerase II complex binding;GO:00431
Aly1p	Alpha arr	GO:20003	GO:00057	GO:0031625	ubiquitin protein ligase binding;GO:004438
putative	A metal-c	GO:00064	GO:00056	GO:0051998	protein carboxyl O-methyltransferase activ
proteasor	Essential	GO:00431	GO:00085	GO:00302	(sce03050)Proteasome
E2 ubiqui	Ubiquitir	GO:00457	GO:00057	GO:00616	(sce04120)Ubiquitin mediated proteolysis
Mef1p	Mitochon	GO:00701	GO:00057	GO:0003746	translation elongation factor activity;GO:
transcrip	Master re	GO:00071	GO:00056	GO:00081	(sce04113)Meiosis - yeast
21S rRNA	Mitochon	GO:00311	GO:00057	GO:0008650	rRNA (uridine-2'-O-)-methyltransferase act
Arp5p	Nuclear a	GO:00427	GO:00310	GO:0043140	ATP-dependent 3'-5' DNA helicase activity;
2-aminoad	Protein w	GO:00090	GO:00056	GO:0047536	2-aminoadipate transaminase activity;GO:00
ornithine	Ornithine	GO:00424	GO:00057	GO:00045	(sce00220)Arginine biosynthesis; (sce01230)B
phenylala	Beta subu	GO:00064	GO:00093	GO:00048	(sce00970)Aminoacyl-tRNA biosynthesis

Rnh203p	Ribonucle	GO:00431	GO:00322	GO:0004523	RNA-DNA hybrid ribonuclease activity;GO:00
alpha-ke	Dihydrol	GO:00335	GO:00093	GO:00041	(sce00310)Lysine degradation; (sce)Citrate c
Yet3p	Protein	GO:00709	GO:00057	GO:00036	(sce04141)Protein processing in endoplasmic
Srb2p	Subunit	GO:00106	GO:00708	GO:0001094	TFIID-class transcription factor complex b
hypothet	Putative	GO:00354	GO:00058	GO:0080139	borate efflux transmembrane transporter ac
Rad28p	Protein	GO:00062	GO:00314	GO:00048	(sce03420)Nucleotide excision repair; (sce04
coatomer	Epsilon-(	GO:00325	GO:00301	GO:1990841	promoter-specific chromatin binding;GO:000
Ecm25p	Non-esse	GO:00715	GO:00057	GO:0003674	molecular_function
hypothet	Vacuolar	GO:00550	GO:00003	GO:0003674	molecular_function
Bor1p	Boron ef	GO:00354	GO:00003	GO:00801	(Human)(hsa04964)Proximal tubule bicarbonate
Bud21p	Componen	GO:00004	GO:00306	GO:0030515	snoRNA binding;GO:0003723 RNA binding;GO:0
Sen15p	Subunit	GO:00003	GO:00002	GO:0000213	tRNA-intron endonuclease activity;GO:0016
histone	Histone	GO:00063	GO:00312	GO:00469	(Human)(hsa05322)Systemic lupus erythematosu
exosome	Exosome	GO:00710	GO:00001	GO:00037	(sce03018)RNA degradation
replicat	Subunit	GO:00062	GO:00313	GO:00036	(sce03430)Mismatch repair; (sce03030)DNA rep
protohem	Heme A:f	GO:00480	GO:00319	GO:00084	(sce00860)Porphyrin and chlorophyll metaboli
U2 snRNP	Componen	GO:00002	GO:00056	GO:00036	(sce03040)Spliceosome
FG-nucle	FG-nucle	GO:00601	GO:00446	GO:00170	(sce03013)RNA transport
Pex18p	Peroxin;	GO:00165	GO:00057	GO:0005515	protein binding;GO:0005488 binding;GO:0003
alpha,al	Neutral	GO:00714	GO:00057	GO:00045	(sce00500)Starch and sucrose metabolism; (sc
NAD+ tra	Putative	GO:00353	GO:00057	GO:0051724	NAD transmembrane transporter activity;GO:
Ice2p	Integral	GO:00362	GO:00325	GO:0003674	molecular_function
Gdi1p	GDP diss	GO:00435	GO:00057	GO:0005093	Rab GDP-dissociation inhibitor activity;GO
Rga1p	GTPase-a	GO:00007	GO:00321	GO:00050	(sce04011)MAPK signaling pathway - yeast
putative	Protein	GO:00066	GO:00057	GO:0004177	aminopeptidase activity;GO:0008238 exopept
Ant1p	Peroxiso	GO:00158	GO:00057	GO:0015217	ADP transmembrane transporter activity;GO:
hypothet	Putative	GO:00081	GO:00055	GO:0003674	molecular_function
Loh1p	Protein	GO:00304	GO:00058	GO:0003674	molecular_function
deoxyhyp	Deoxyhyp	GO:00086	GO:00057	GO:0034038	deoxyhypusine synthase activity;GO:0016765
proteaso	Metallop	GO:19029	GO:00345	GO:00048	(sce03050)Proteasome
aminodeo	Aminodeo	GO:00466	GO:00057	GO:0008696	4-amino-4-deoxychorismate lyase activity;G
Reh1p	Cytoplas	GO:00071	GO:00306	GO:0046872	metal ion binding;GO:0043169 cation bindin
nicotina	Nicotina	GO:00343	GO:00056	GO:00003	(sce00760)Nicotinate and nicotinamide metabo
Pom34p	Subunit	GO:00304	GO:00313	GO:0017056	structural constituent of nuclear pore;GO:
proteaso	Alpha 2	GO:00104	GO:00345	GO:00042	(sce03050)Proteasome
chromati	SIR prot	GO:00013	GO:00991	GO:0031491	nucleosome binding;GO:0032947 protein-cont
hypothet	Protein	GO:00081	GO:00306	GO:0043022	ribosome binding;GO:0043021 ribonucleoprot
ribosoma	Protein	GO:00064	GO:00226	GO:00037	(sce03010)Ribosome
Mob2p	Activato	GO:00071	GO:00059	GO:0030295	protein kinase activator activity;GO:0019
hypothet	Putative	GO:00101	GO:00056	GO:0050484	GMP 5'-nucleotidase activity;GO:0002953 5'
transcri	General	GO:00359	GO:00905	GO:00428	(sce04011)MAPK signaling pathway - yeast; (s
putative	Putative	GO:00071	GO:00466	GO:0008496	mannan endo-1,6-alpha-mannosidase activity
Mss11p	Transcri	GO:00360	GO:00056	GO:00036	(sce04011)MAPK signaling pathway - yeast
nucleosi	Nucleosi	GO:00061	GO:00057	GO:00045	(sce)Pyrimidine ribonucleotide biosynthesis,
Avt7p	Putative	GO:19034	GO:00003	GO:0061459	L-arginine transmembrane transporter activ
putative	Putative	GO:00332	GO:00058	GO:0052851	ferric-chelate reductase (NADPH) activity;
histone	JmjC dom	GO:19043	GO:00350	GO:0034649	histone demethylase activity (H3-monomethy
Pan1p	Part of	GO:20006	GO:00304	GO:0003779	actin binding;GO:0005509 calcium ion bindi
Agp2p	Plasma m	GO:19022	GO:00003	GO:0015203	polyamine transmembrane transporter activi
aspartic	Intramem	GO:00064	GO:00714	GO:0042500	aspartic endopeptidase activity, intramemb
peptide	Subunit	GO:00171	GO:00314	GO:0004596	peptide alpha-N-acetyltransferase activity
RNA-bind	Subunit	GO:00349	GO:00056	GO:00045	(sce03008)Ribosome biogenesis in eukaryotes;
Drn1p	Splicing	GO:00003	GO:00058	GO:0061632	RNA lariat debranching enzyme activator ac
transcri	Transcri	GO:19004	GO:00315	GO:0000980	RNA polymerase II distal enhancer sequence-

Rdr1p	Transcrip	GO:00094	GO:00056	GO:0000981 DNA-binding transcription factor activity,	
porphobil	Aminolevu	GO:00067	GO:00058	GO:00046	(sce00860)Porphyrin and chlorophyll metaboli
D-lactate	D-2-hydro	GO:19034	GO:00312	GO:00044	(sce00620)Pyruvate metabolism
Yop1p	Reticulon	GO:00325	GO:00717	GO:0005515 protein binding;GO:0005488 binding;GO:0003	
None	None	None	None	None	None
Rqc1p	Component	GO:19901	GO:19901	GO:0003674 molecular_function	
Csr1p	Phosphati	GO:19013	GO:00056	GO:0008526 phosphatidylinositol transporter activity;	
Lam4p	Sterol-bi	GO:01200	GO:00325	GO:0120015 intermembrane sterol transfer activity;GO:	
Igd1p	Cytoplasm	GO:00458	GO:00057	GO:0004857 enzyme inhibitor activity;GO:0030234 enzym	
protein t	Essential	GO:00450	GO:00427	GO:0008565 protein transporter activity;GO:0051082 un	
translati	Translati	GO:0006414 transla		GO:0003746 translation elongation factor activity;GO:	
Swm2p	Protein w	GO:00362	GO:00057	GO:0003674 molecular_function	
phosphata	Phosphata	GO:00353	GO:00056	GO:0004792 thiosulfate sulfurtransferase activity;GO:	
hypotheti	Putative	GO:00081	GO:00003	GO:0003674 molecular_function	
Rad59p	Protein i	GO:00007	GO:00056	GO:00055	(sce03440)Homologous recombination
DNA-direc	RNA polym	GO:00427	GO:00056	GO:00010	(sce03020)RNA polymerase; (sce00240)Pyrimidi
nicotinam	Nicotinic	GO:00346	GO:00056	GO:00003	(sce00760)Nicotinate and nicotinamide metabo
chaperoni	Subunit o	GO:00064	GO:00058	GO:0051082 unfolded protein binding;GO:0005524 ATP bi	
hypotheti	Putative	GO:00081	GO:00056	GO:0003674 molecular_function	
Rab famil	Endosomal	GO:00362	GO:00003	GO:00039	(sce04145)Phagosome; (sce04144)Endocytosis
hypotheti	Dubious o	GO:00081	GO:00055	GO:0003674 molecular_function	
hypotheti	Protein of unknown function; GFP-fusion protein is induced in response to the DN				
Sap190p	Protein t	GO:00020	GO:00057	GO:0003674 molecular_function	
protein k	Serine ki	GO:20002	GO:00308	GO:00046	(sce03008)Ribosome biogenesis in eukaryotes
E2 ubiqui	Ubiquitin	GO:00304	GO:00057	GO:00616	(sce04120)Ubiquitin mediated proteolysis; (s
DNA-bindi	Transcrip	GO:00973	GO:00056	GO:0000987 proximal promoter sequence-specific DNA bi	
NADP-depe	NADPH-dep	GO:00338	GO:00055	GO:00338	(sce00040)Pentose and glucuronate interconve
Rph1p	JmjC doma	GO:00705	GO:00350	GO:0051864 histone demethylase activity (H3-K36 speci	
Ysw1p	Protein r	GO:00321	GO:00056	GO:0003674 molecular_function	
Esbp6p	Protein w	GO:00157	GO:00057	GO:0008028 monocarboxylic acid transmembrane transpor	
histone c	Class I h	GO:00615	GO:00001	GO:0032041 NAD-dependent histone deacetylase activity	
Rox1p	Heme-depe	GO:00972	GO:00007	GO:0001135 RNA polymerase II transcription regulator	
putative	Putative	GO:00158	GO:00198	GO:00039	(Human)(hsa04211)Longevity regulating pathwa
histidine	Cytoplasm	GO:00064	GO:00057	GO:00048	(sce00970)Aminoacyl-tRNA biosynthesis
hypotheti	Pseudogen	GO:00081	GO:00160	GO:0003674 molecular_function	
dihydroor	Dihydroor	GO:00442	GO:00198	GO:19906	(sce)Uridine monophosphate biosynthesis, glu
diphthine	Diphtham	GO:00171	GO:00057	GO:0017178 diphthine-ammonia ligase activity;GO:0016	
protein k	Protein k	GO:00614	GO:00056	GO:00046	(sce04213)Longevity regulating pathway - mul
SWI/SNF c	Catalytic	GO:19001	GO:00165	GO:0015616 DNA translocase activity;GO:0070577 lysine	
chaperoni	Subunit o	GO:00064	GO:00058	GO:0051082 unfolded protein binding;GO:0005524 ATP bi	
mitochond	Mitochond	GO:00707	GO:00057	GO:0015183 L-aspartate transmembrane transporter acti	
cytochrom	Protein o	GO:00551	GO:00058	GO:0016491 oxidoreductase activity;GO:0003824 catalyt	
Vps55p	Late endo	GO:20000	GO:00344	GO:0003674 molecular_function	
glycine h	Mitochond	GO:00192	GO:00057	GO:00709	(sce)C1-unit interconversion, eukaryotes; (M
Srl4p	Protein o	GO:00092	GO:00057	GO:0003674 molecular_function	
Hol1p	Putative	GO:00068	GO:00003	GO:0015665 alcohol transmembrane transporter activity	
hypotheti	Putative	GO:00081	GO:00055	GO:0003674 molecular_function	
rRNA-bind	Component	GO:00004	GO:00320	GO:0070181 small ribosomal subunit rRNA binding;GO:00	
Trm12p	S-adenosy	GO:00315	GO:00057	GO:0102522 tRNA 4-demethylwyosine alpha-amino-alpha-c	
Rfx1p	Major tra	GO:00001	GO:00056	GO:00009	(sce04111)Cell cycle - yeast
Tfs1p	Inhibitor	GO:00109	GO:00003	GO:0004867 serine-type endopeptidase inhibitor activi	
translati	Gamma sub	GO:19035	GO:00320	GO:00037	(sce03013)RNA transport
choline-p	Cholineph	GO:00066	GO:00425	GO:00041	(sce)Phosphatidylcholine (PC) biosynthesis,
amino aci	Amino aci	GO:00033	GO:00057	GO:0015171 amino acid transmembrane transporter activ	

Spo71p	Meiosis-s	GO:00321	GO:00056	GO:0003674	molecular_function
ubiquitin	RING-fing	GO:00165	GO:19904	GO:00048	(sce04146)Peroxisome
Tsr3p	Protein	GO:00304	GO:00056	GO:0016740	transferase activity;GO:0003824 catalytic
2-isopro	Alpha-is	GO:00090	GO:00057	GO:00038	(sce)Leucine biosynthesis, 2-oxoisovalerate
3-isopro	Beta-iso	GO:00090	GO:00058	GO:00038	(sce00660)C5-Branched dibasic acid metabolis
hypothet	Putative	GO:00081	GO:00057	GO:0003674	molecular_function
long-cha	Long cha	GO:00016	GO:00055	GO:01023	(sce)beta-Oxidation, acyl-CoA synthesis; (M0
Cwh43p	GPI lipi	GO:00065	GO:00059	GO:0003674	molecular_function
ion-tran	P-type A	GO:00300	GO:00058	GO:0015662	ATPase activity, coupled to transmembrane
ubiquiti	Componen	GO:19046	GO:00008	GO:00431	(sce04144)Endocytosis
Aqr1p	Plasma m	GO:00329	GO:00003	GO:0008028	monocarboxylic acid transmembrane transpor
aspartat	Mitochon	GO:00065	GO:00057	GO:00040	(sce00360)Phenylalanine metabolism; (sce0035
RNA meth	Protein	GO:00704	GO:00435	GO:0004809	tRNA (guanine-N2-)-methyltransferase activ
3,4-dihy	3,4-dihy	GO:00092	GO:00057	GO:00086	(sce00740)Riboflavin metabolism; (sce01110)B
methioni	Methioni	GO:00355	GO:00056	GO:0070006	metalloaminopeptidase activity;GO:0004177
serine/t	DDK (Dbf	GO:19049	GO:00314	GO:00046	(sce04111)Cell cycle - yeast; (sce04113)Meio
aldo-ket	Xylose a	GO:00195	GO:00058	GO:0004032	alditol:NADP+ 1-oxidoreductase activity;GO
mitochon	Mitochon	GO:00194	GO:00057	GO:00047	(sce04213)Longevity regulating pathway - mul
S-methyl-	5'-methyl	GO:00195	GO:00056	GO:00465	(sce)Methionine salvage pathway; (M00034)Met
ATP-bind	Plasma m	GO:00468	GO:00057	GO:00085	(sce02010)ABC transporters
Ies5p	Non-esse	GO:00007	GO:00310	GO:0003674	molecular_function
hypothet	Essentia	GO:00081	GO:00160	GO:0003674	molecular_function
hypothet	Putative	GO:00081	GO:00003	GO:0003674	molecular_function
Ssy5p	Serine p	GO:00165	GO:00198	GO:0004252	serine-type endopeptidase activity;GO:0008
Agp3p	Low-affi	GO:00033	GO:00057	GO:0015171	amino acid transmembrane transporter activ
phosphat	Phosphat	GO:00344	GO:00058	GO:00344	(sce00564)Glycerophospholipid metabolism
Sas5p	Subunit	GO:00063	GO:00332	GO:0004402	histone acetyltransferase activity;GO:0061
oxystero	Protein	GO:00347	GO:00001	GO:0008142	oxysterol binding;GO:0005545 1-phosphatidy
Ecm5p	Subunit	GO:00347	GO:00702	GO:0034647	histone demethylase activity (H3-trimethyl
Arc19p	Subunit	GO:00343	GO:00304	GO:00037	(sce04144)Endocytosis
palmitoy	Palmitoy	GO:00900	GO:00319	GO:0019706	protein-cysteine S-palmitoyltransferase ac
bifuncti	Leucyl a	GO:00301	GO:00056	GO:00043	(sce00590)Arachidonic acid metabolism; (sce0
putative	Putative	GO:00068	GO:00003	GO:0052851	ferric-chelate reductase (NADPH) activity;
Bas1p	Myb-rela	GO:00511	GO:00056	GO:0001135	RNA polymerase II transcription regulator
None	None	None	None	None	None
Aim34p	Protein	GO:00081	GO:00319	GO:0003674	molecular_function
ribosoma	Ribosoma	GO:00021	GO:00226	GO:00037	(sce03010)Ribosome
bifuncti	Thiamine-	GO:00092	GO:00058	GO:00047	(sce00730)Thiamine metabolism; (sce01100)Met
Caf4p	WD40 rep	GO:00165	GO:00705	GO:0003674	molecular_function
inositol	Inositol	GO:00008	GO:00056	GO:00008	(sce00562)Inositol phosphate metabolism; (sc
Lsb3p	Protein	GO:19000	GO:00304	GO:0051015	actin filament binding;GO:0035091 phosphat
sedohept	Sedohept	GO:00463	GO:00056	GO:00502	(M00002)Glycolysis, core module involving th
None	None	None	None	None	None
hypothet	Protein	GO:00081	GO:00056	GO:0003674	molecular_function
Cbf2p	Essentia	GO:00009	GO:00315	GO:0019237	centromeric DNA binding;GO:0008301 DNA bin
myosin 3	One of t	GO:20006	GO:00304	GO:0000146	microfilament motor activity;GO:0003774 mo
Irc25p	Componen	GO:00704	GO:00056	GO:0003674	molecular_function
hypothet	Putative	GO:00081	GO:00003	GO:0003674	molecular_function
Pih1p	Componen	GO:00004	GO:00972	GO:0005515	protein binding;GO:0005488 binding;GO:0003
Hsp90 co	Co-chape	GO:00322	GO:00058	GO:0051879	Hsp90 protein binding;GO:0051087 chaperone
bifuncti	Alpha su	GO:00183	GO:00059	GO:00046	(sce00900)Terpenoid backbone biosynthesis; (
Eap1p	eIF4E-as	GO:00002	GO:00104	GO:0008190	eukaryotic initiation factor 4E binding;GO
Rim2p	Mitochon	GO:19905	GO:00057	GO:0015218	pyrimidine nucleotide transmembrane transp
Scd6p	Represso	GO:00459	GO:00104	GO:0031370	eukaryotic initiation factor 4G binding;GO

tyrosine/Protein GO:0001GO:0005GO:0008138 protein tyrosine/serine/threonine phosphat
threo-3-3-hydroxGO:0070GO:0005GO:0018(Human)(hsa00260)Glycine, serine and threoni
asparagiAsparagiGO:0070GO:0005GO:0040(sce00250)Alanine, aspartate and glutamate m
hypothetCytoplasGO:0008GO:0005GO:0032266 phosphatidylinositol-3-phosphate binding;G
hypothetProtein GO:0008GO:0005GO:0003674 molecular_function
hypothetPutative GO:0008GO:0005GO:0003674 molecular_function
Mrp8p Protein GO:0008GO:0005GO:0003674 molecular_function
Ysy6p Protein GO:0030GO:0005GO:0003674 molecular_function
CAAX preType II GO:0071GO:0030GO:0004(sce00900)Terpenoid backbone biosynthesis; (
ubiquitiUbiquitiGO:1904GO:0000GO:0034450 ubiquitin-ubiquitin ligase activity;GO:006
Osw5p Protein of unknown function with possible role in spore wall assembly; predicted
Abp140p AdoMet-dGO:0030GO:0030GO:0052735 tRNA (cytosine-3-)-methyltransferase activ
hypothetPutative GO:0008GO:0005GO:0003674 molecular_function
putative Major ceGO:0031GO:0009GO:0005199 structural constituent of cell wall;GO:001
cleavage Subunit GO:0098GO:0005GO:0030(sce03015)mRNA surveillance pathway
Alb1p ShuttlinGO:0042GO:0005GO:0043022 ribosome binding;GO:0043021 ribonucleoprot
hypothetPutative GO:0008GO:0005GO:0003674 molecular_function
nitrogenTranscriGO:0001GO:0005GO:0000978 RNA polymerase II proximal promoter sequen
MCM DNA Protein GO:1902GO:0071GO:0017(sce03030)DNA replication; (sce04111)Cell cy
Caj1p Nuclear GO:0016GO:0005GO:0003674 molecular_function
Ups1p PhosphatGO:2001GO:0031GO:1990050 phosphatidic acid transporter activity;GO:
Puf2p PUF famiGO:0000GO:0005GO:0003729 mRNA binding;GO:0003723 RNA binding;GO:000
hypothetPutative GO:0015GO:0016GO:0008521 acetyl-CoA transmembrane transporter activ
pseudouriPseudouriGO:0001GO:0005GO:0120159 rRNA pseudouridine synthase activity;GO:00
ribosomaRibosomaGO:0000GO:0022GO:0003(sce03010)Ribosome
transcriEssentiaGO:0070GO:0000GO:0001156 TFIIIC-class transcription factor complex
Mtl1p Putative GO:0042GO:0005GO:0003(sce04011)MAPK signaling pathway - yeast
None None None None None None
putative Nuclear GO:0000GO:0030GO:0008237 metallopeptidase activity;GO:0070011 pepti
serine/thSer/Thr GO:1901GO:0005GO:1901(sce04392)Hippo signaling pathway -multiple
Mdv1p PeripherGO:0016GO:0005GO:0043130 ubiquitin binding;GO:0032182 ubiquitin-lik
amino acLow-affiGO:0015GO:0005GO:0015193 L-proline transmembrane transporter activi
None None None None None None
hypothetPutative GO:0008GO:0005GO:0003674 molecular_function
14-3-3 f14-3-3 pGO:0031GO:0010GO:0050(sce04011)MAPK signaling pathway - yeast
hypothetPutative GO:0008GO:0000GO:0003674 molecular_function
dioxygenTryptophGO:0034GO:0005GO:0033(sce00380)Tryptophan metabolism; (sce01100)M
Sur7p Plasma mGO:0097GO:0030GO:0003674 molecular_function
Bud20p C2H2-typGO:0000GO:0030GO:0043023 ribosomal large subunit binding;GO:0043021
mitochonMitochonGO:0032GO:0005GO:0003735 structural constituent of ribosome;GO:0005
Eaf6p Subunit GO:0043GO:1990GO:0043994 histone acetyltransferase activity (H3-K23
glutamatNADP(+)-GO:0006GO:0005GO:0004(sce00910)Nitrogen metabolism; (sce00220)Arg
D-tyrosyD-Tyr-tRGO:1900GO:0005GO:0097358 D-leucyl-tRNA(Leu) deacylase activity;GO:0
hypothetProtein GO:0008GO:0005GO:0043022 ribosome binding;GO:0043021 ribonucleoprot
hypothetPutative protein of unknown function
18S rRNA MethyltrGO:0000GO:0005GO:0016435 rRNA (guanine) methyltransferase activity;
hypothetProtein GO:0008GO:0005GO:0003674 molecular_function
trifunctiMultifunGO:0046GO:0031GO:0004(M00126)Tetrahydrofolate biosynthesis, GTP =
proline Proline GO:0010GO:0005GO:0004(sce00330)Arginine and proline metabolism; (
trans-2-Enoyl reGO:0042GO:0005GO:0102(sce)Fatty acid biosynthesis, elongation, en
arginyltArginyl-GO:0016GO:0005GO:0004057 arginyltransferase activity;GO:0016755 tra
hypothetProtein GO:0008GO:0000GO:0003674 molecular_function
proteoliRegulatoGO:0032GO:0005GO:0030234 enzyme regulator activity;GO:0098772 molec
tRNA dihDihydrouGO:0002GO:0005GO:0102263 tRNA-dihydrouridine17 synthase activity;GO

Ecm21p	Protein i	GO:007008	GO:000582	GO:0031625 ubiquitin protein ligase binding;GO:004438
U2-type s	Splicing	GO:000039	GO:000568	GO:0003674 molecular_function
Bfr1p	Component	GO:000829	GO:000584	GO:0003729 mRNA binding;GO:0003723 RNA binding;GO:000
Got1p	Homodimer	GO:004214	GO:000013	GO:0003674 molecular_function
cystathio	Cystathio	GO:001934	GO:000563	GO:000396 (sce00450)Selenocompound metabolism; (sce009
Mtc3p	Protein c	GO:000612	GO:000575	GO:0004129 cytochrome-c oxidase activity;GO:0016676 o
Pch2p	Hexameric	GO:005159	GO:000573	GO:0016887 ATPase activity;GO:0017111 nucleoside-trip
arylforma	Formylkyn	GO:001944	GO:000557	GO:000406 (sce00380)Tryptophan metabolism; (sce00630)G
hypotheti	Putative	GO:000815	GO:000563	GO:0003674 molecular_function
mitochond	Mitochond	GO:001943	GO:000576	GO:000478 (sce04213)Longevity regulating pathway - mul
Rtg2p	Sensor of	GO:190000	GO:004669	GO:0051219 phosphoprotein binding;GO:0005515 protein
monothiol	Glutathio	GO:003106	GO:000582	GO:0015038 glutathione disulfide oxidoreductase activ
mannosyli	Mannosyli	GO:005199	GO:000032	GO:0000030 mannosyltransferase activity;GO:0016758 tr
Sfh5p	Non-class	GO:004300	GO:003254	GO:0008526 phosphatidylinositol transporter activity;
Sm-like p	Lsm (Like	GO:003049	GO:000568	GO:000372 (sce03018)RNA degradation; (sce03040)Spliceo
Arc1p	Protein t	GO:005135	GO:001710	GO:0004831 tyrosine-tRNA ligase activity;GO:0080025 p
exosome r	Exosome r	GO:007103	GO:000017	GO:001709 (sce03018)RNA degradation
4-nitroph	Conserved	GO:004683	GO:000563	GO:000896 (Human)(hsa00630)Glyoxylate and dicarboxylat
Kre5p	Protein r	GO:007088	GO:000578	GO:000398 (sce04141)Protein processing in endoplasmic
Bug1p	Cis-golgi	GO:000688	GO:000580	GO:0003674 molecular_function
Pub1p	Poly (A)-	GO:003406	GO:001049	GO:0008266 poly(U) RNA binding;GO:0008187 poly-pyrimi
Arg80p	Transcrip	GO:190007	GO:000563	GO:0000987 proximal promoter sequence-specific DNA bi
Hda3p	Subunit o	GO:001097	GO:007082	GO:0004407 histone deacetylase activity;GO:0033558 pr
translati	eIF3a sub	GO:000218	GO:007154	GO:000374 (sce03013)RNA transport
guanine n	Guanine n	GO:000689	GO:000582	GO:0005096 GTPase activator activity;GO:0030695 GTPas
Ybp2p	Central k	GO:000705	GO:004350	GO:0003674 molecular_function
hypotheti	Putative	GO:000815	GO:000557	GO:0003674 molecular_function
Mfg1p	Regulator	GO:000815	GO:000563	GO:0003674 molecular_function
Nrd1 comp	RNA-bindi	GO:007104	GO:003564	GO:0001068 transcription regulatory region RNA bindin
orotate p	Minor oro	GO:004420	GO:000573	GO:000458 (sce)Uridine monophosphate biosynthesis, glu
Apl4p	Gamma-ada	GO:000689	GO:003012	GO:003027 (Human)(hsa04142)Lysosome
Rav1p	Subunit o	GO:000703	GO:004329	GO:0003674 molecular_function
Twf1p	Twinfilin	GO:005101	GO:003047	GO:0003785 actin monomer binding;GO:0051015 actin fil
Sec17p	Alpha-SNA	GO:003549	GO:000032	GO:000548 (sce04138)Autophagy - yeast
Zds1p	Protein w	GO:000640	GO:000593	GO:0004864 protein phosphatase inhibitor activity;GO:
Pin4p	Protein i	GO:200081	GO:007101	GO:0003729 mRNA binding;GO:0003723 RNA binding;GO:000
Trk1p	Component	GO:003000	GO:004512	GO:0140107 high-affinity potassium ion transmembrane
serine/th	Protein k	GO:190018	GO:000563	GO:000467 (sce04138)Autophagy - yeast
Bud3p	Guanine n	GO:000712	GO:000014	GO:0005089 Rho guanyl-nucleotide exchange factor acti
threonylc	Protein i	GO:000294	GO:000078	GO:0061710 L-threonylcarbamoyladenylate synthase;GO:0
Atg19p	Receptor	GO:003043	GO:003427	GO:0030674 protein binding, bridging;GO:0060090 molec
Fub1p	Proteasom	GO:000632	GO:000557	GO:0070628 proteasome binding;GO:0044877 protein-cont
divalent	Divalent	GO:000687	GO:000032	GO:0015086 cadmium ion transmembrane transporter acti
exosome r	Exosome r	GO:007104	GO:000017	GO:003014 (sce03018)RNA degradation
DNA-direc	Subunit o	GO:004313	GO:004362	GO:000831 (sce03410)Base excision repair; (sce03440)Ho
Wsc3p	Sensor-tr	GO:003024	GO:004333	GO:000488 (sce04139)Mitophagy - yeast; (sce04011)MAPK
DNA-bindi	Transcrip	GO:003608	GO:000077	GO:0000987 proximal promoter sequence-specific DNA bi
transketo	Transketo	GO:000609	GO:000582	GO:000480 (M00007)Pentose phosphate pathway, non-oxida
cystathio	Cystathio	GO:001934	GO:001049	GO:000412 (sce)Cysteine biosynthesis, homocysteine + s
hypotheti	Putative	GO:000815	GO:000557	GO:0003674 molecular_function
guanine n	Guanine n	GO:199050	GO:004352	GO:0005085 guanyl-nucleotide exchange factor activity
carboxype	Vacuolar	GO:004693	GO:000032	GO:000418 (Human)(hsa04614)Renin-angiotensin system; (
DNA repli	Subunit o	GO:000072	GO:007116	GO:0003674 molecular_function
Hap5p	Subunit o	GO:000043	GO:001660	GO:0000978 RNA polymerase II proximal promoter sequen

Rod1p	Alpha-arr	GO:000075	GO:000582	GO:0031625	ubiquitin protein ligase binding;GO:004438
Swi1p	Subunit o	GO:003149	GO:001651	GO:0015616	DNA translocase activity;GO:0001102 RNA po
RNA-bindi	RNA-bindi	GO:000049	GO:000573	GO:0003723	RNA binding;GO:0003676 nucleic acid bindin
sphingosi	Sphingani	GO:003014	GO:000578	GO:000017	(sce00600)Sphingolipid metabolism; (sce01100)
hypotheti	Putative	GO:007110	GO:000582	GO:0016807	cysteine-type carboxypeptidase activity;GO
diacylgly	Diacylgly	GO:000665	GO:003017	GO:000414	(sce00564)Glycerophospholipid metabolism; (s
ribosomal	Ribosomal	GO:000218	GO:002262	GO:000373	(sce03010)Ribosome
1-acylgly	1-acyl-sn	GO:001602	GO:000581	GO:000384	(sce00561)Glycerolipid metabolism; (sce00564)
Zn(2+) tr	Vacuolar	GO:000688	GO:000032	GO:0005385	zinc ion transmembrane transporter activit
Ytp1p	Probable	GO:000815	GO:001602	GO:0003674	molecular_function
amino aci	Putative	GO:190340	GO:000032	GO:0061459	L-arginine transmembrane transporter activ
ribosomal	Ribosomal	GO:000218	GO:002262	GO:000373	(sce03010)Ribosome
Mrx5p	Protein that associates with mitochondrial ribosome; homozygous diploid deletion				
replicati	Subunit o	GO:000626	GO:000566	GO:004304	(sce03440)Homologous recombination; (sce0343
Frt2p	Tail-anch	GO:007146	GO:000578	GO:0003674	molecular_function
U6 snRNP	Splicing	GO:000024	GO:000568	GO:0017070	U6 snRNA binding;GO:0017069 snRNA binding;
None	None	None	None	None	None
tRNA meth	2'-O-ribo	GO:003048	GO:000573	GO:0008175	tRNA methyltransferase activity;GO:0008173
cAMP-depe	Regulator	GO:006140	GO:000595	GO:000486	(Human)(hsa04910)Insulin signaling pathway
putative	AGC kinas	GO:001810	GO:000563	GO:0004674	protein serine/threonine kinase activity;G
Rie1p	Putative	GO:000637	GO:001049	GO:0003729	mRNA binding;GO:0003723 RNA binding;GO:000
allantoat	Allantoat	GO:001571	GO:000032	GO:0015124	allantoate transmembrane transporter activ
endopepti	Catalytic	GO:000662	GO:004272	GO:000823	(sce03060)Protein export
TFIIH com	Cyclin as	GO:007081	GO:007098	GO:000835	(sce03022)Basal transcription factors; (sce0
GTP-bindi	Member of	GO:190383	GO:001049	GO:0005525	GTP binding;GO:0032550 purine ribonucleosi
S-adenosy	S-adenosy	GO:001836	GO:003565	GO:0008276	protein methyltransferase activity;GO:0008
DEAD-box	ATP-depen	GO:007104	GO:000573	GO:000400	(sce03040)Spliceosome
metalloam	Vacuolar	GO:003225	GO:003427	GO:0070006	metalloaminopeptidase activity;GO:0004177
Boi1p	Protein i	GO:000711	GO:004333	GO:0005543	phospholipid binding;GO:0008289 lipid bind
None	None	None	None	None	None
rRNA-proc	Protein r	GO:000046	GO:008000	GO:0003729	mRNA binding;GO:0003723 RNA binding;GO:000
mitochond	Mitochond	GO:003254	GO:000576	GO:001984	(sce03010)Ribosome
hypotheti	Putative	GO:000815	GO:000557	GO:0003674	molecular_function
inorganic	Cytoplasm	GO:000679	GO:000557	GO:000442	(sce00190)Oxidative phosphorylation
putative	Putative	GO:000641	GO:000573	GO:0003724	RNA helicase activity;GO:0003743 translati
rRNA-proc	Component	GO:000046	GO:003068	GO:0003674	molecular_function
cornichon	COPII-coa	GO:000712	GO:003013	GO:0038024	cargo receptor activity;GO:0003674 molecul
hypotheti	Protein o	GO:000815	GO:000557	GO:0003674	molecular_function
hypotheti	Putative	GO:000815	GO:000573	GO:0003674	molecular_function
Esc1p	Protein i	GO:003439	GO:000078	GO:0003674	molecular_function
sedoheptu	Transaldo	GO:000905	GO:000563	GO:000480	(M00007)Pentose phosphate pathway, non-oxida
ribose-5-	Ribose-5-	GO:000905	GO:000563	GO:000475	(M00007)Pentose phosphate pathway, non-oxida
Ecm23p	Non-esser	GO:000712	GO:000557	GO:0008270	zinc ion binding;GO:0043565 sequence-speci
putative	Flavin-de	GO:000674	GO:000574	GO:000868	(M00128)Ubiquinone biosynthesis, eukaryotes,
Lmo1p	Homolog o	GO:000042	GO:000573	GO:0003674	molecular_function
hypotheti	Protein t	GO:007048	GO:000588	GO:0003674	molecular_function
aspartate	Aspartyl-	GO:000642	GO:001710	GO:000481	(sce00970)Aminoacyl-tRNA biosynthesis
mitochond	Mitochond	GO:003254	GO:000576	GO:000373	(sce03010)Ribosome
Lcp5p	Essential	GO:000048	GO:003204	GO:0003674	molecular_function
Dat1p	DNA bindi	GO:000012	GO:000582	GO:0003680	AT DNA binding;GO:1990837 sequence-specifi
Sec9p	t-SNARE p	GO:000690	GO:000593	GO:0019905	syntaxin binding;GO:0005484 SNAP receptor
Rgl1p	Regulator	GO:000091	GO:000093	GO:0003674	molecular_function
tetrahydr	Folylpoly	GO:004690	GO:000574	GO:000432	(M00126)Tetrahydrofolate biosynthesis, GTP =>
Rix1p	Component	GO:000626	GO:009734	GO:0003682	chromatin binding;GO:0005488 binding;GO:00

hypotheti	Dubious (	GO:00081	GO:00055	GO:0003674 molecular_function	
Yox1p	Homeobox	GO:00719	GO:00007	GO:00009	(sce04111)Cell cycle - yeast
Jem1p	DnaJ-like	GO:00007	GO:00319	GO:00517	(sce04141)Protein processing in endoplasmic
flavodox	Protein v	GO:00551	GO:00451	GO:0003955 NAD(P)H dehydrogenase (quinone) activity;G	
DNA polyn	Second la	GO:00708	GO:00086	GO:00303	(sce03410)Base excision repair; (sce03030)DN
Vnx1p	Calcium/H	GO:00357	GO:19908	GO:0015369 calcium:proton antiporter activity;GO:0051	
bifuncti	Glycerol	GO:00160	GO:00301	GO:00162	(sce00561)Glycerolipid metabolism; (sce00564)
mRNA spl	Core Sm p	GO:00003	GO:00056	GO:19909	(sce03040)Spliceosome
putative	Putative	GO:00300	GO:00003	GO:0015086 cadmium ion transmembrane transporter acti	
dynamin-l	Dynamin-l	GO:00609	GO:00304	GO:00039	(sce04139)Mitophagy - yeast
mitochon	Mitochon	GO:00000	GO:00057	GO:00037	(sce03010)Ribosome
transcri	Transcri	GO:00611	GO:00058	GO:0003714 transcription corepressor activity;GO:0003	
Ptr3p	Componen	GO:00432	GO:00198	GO:0003674 molecular_function	
ditrans,	Forms deh	GO:00194	GO:19044	GO:00455	(sce00900)Terpenoid backbone biosynthesis; (
Sla2p	Adaptor p	GO:00071	GO:00301	GO:0035615 clathrin adaptor activity;GO:0043325 phosp	
ribosome	Essential	GO:00004	GO:00306	GO:0003674 molecular_function	
S-adenosy	Methyltra	GO:00349	GO:00007	GO:0008757 S-adenosylmethionine-dependent methyltrans	
None	None	None	None	None	None
Tea1p	Ty1 enhar	GO:00063	GO:00512	GO:0000981 DNA-binding transcription factor activity,	
hypotheti	Putative	GO:00081	GO:00055	GO:0003674 molecular_function	
hypotheti	Putative protein of unknown function; the authentic, non-tagged protein is detec				
transcri	TFIIF (T	GO:00011	GO:00056	GO:00011	(sce03022)Basal transcription factors
hypotheti	Protein (	GO:00081	GO:00055	GO:0046872 metal ion binding;GO:0043169 cation bindin	
septin CI	Componen	GO:00311	GO:00001	GO:0010314 phosphatidylinositol-5-phosphate binding;G	
Bem2p	Rho GTPas	GO:00350	GO:00056	GO:00050	(sce04011)MAPK signaling pathway - yeast
hypotheti	Mitochon	GO:00090	GO:00325	GO:0003729 mRNA binding;GO:0003723 RNA binding;GO:000	
hypotheti	Putative	GO:00081	GO:00055	GO:0003674 molecular_function	
hypoxant	Dimeric h	GO:00322	GO:00056	GO:00526	(sce00230)Purine metabolism; (sce01110)Biosy
anaphase	Processiv	GO:19046	GO:00056	GO:00616	(sce04120)Ubiquitin mediated proteolysis; (s
hypotheti	Putative	GO:00081	GO:00160	GO:0003674 molecular_function	
None	None	None	None	None	None
Sdh8p	Protein i	GO:00345	GO:00057	GO:0044183 protein binding involved in protein foldin	
hexapreny	O-methylt	GO:00067	GO:00057	GO:00615	(M00128)Ubiquinone biosynthesis, eukaryotes,
cyclin CI	G1 cyclin	GO:00070	GO:00003	GO:00165	(sce04011)MAPK signaling pathway - yeast; (s
ribosomal	Ribosomal	GO:00021	GO:00226	GO:00037	(sce03010)Ribosome
Ldb18p	Componen	GO:00001	GO:00002	GO:0003674 molecular_function	
Eri1p	Endoplasm	GO:00065	GO:00005	GO:00050	(sce00563)Glycosylphosphatidylinositol (GPI)-
NAD+ tran	Mitochon	GO:00068	GO:00057	GO:0005477 pyruvate secondary active transmembrane tr	
Mft1p	Subunit (	GO:00064	GO:00004	GO:0032947 protein-containing complex scaffold activi	
glyceral	Glyceral	GO:00060	GO:00092	GO:00043	(M00002)Glycolysis, core module involving th
putative	Putative	GO:00510	GO:00469	GO:0015288 porin activity;GO:0008308 voltage-gated an	
Cax4p	Dolichyl	GO:00064	GO:00301	GO:00478	(sce00510)N-Glycan biosynthesis
Nat2p	Protein (	GO:00171	GO:00057	GO:0016746 transferase activity, transferring acyl gr	
histone (	Histone (	GO:00602	GO:00350	GO:00518	(sce04213)Longevity regulating pathway - mul
long-cha	Long cha	GO:19053	GO:00104	GO:01023	(sce)beta-Oxidation, acyl-CoA synthesis; (MO
serine/t	Protein k	GO:00901	GO:00057	GO:0004674 protein serine/threonine kinase activity;G	
Ras fami	GTP-bind	GO:00071	GO:00059	GO:00039	(Human)(hsa05211)Renal cell carcinoma; (hsa0
Dcv1p	Protein (	GO:00081	GO:00358	GO:0003674 molecular_function	
None	None	None	None	None	None
Crf1p	Transcri	GO:00106	GO:00007	GO:0003714 transcription corepressor activity;GO:0003	
Bst1p	GPI inos	GO:00066	GO:00057	GO:00501	(sce00563)Glycosylphosphatidylinositol (GPI)-
Nrm1p	Transcri	GO:00719	GO:00309	GO:0070491 repressing transcription factor binding;GO	
protein	Protein k	GO:00181	GO:00005	GO:0009931 calcium-dependent protein serine/threonine	
hypotheti	Dubious (	GO:00081	GO:00160	GO:0003674 molecular_function	

protein k	Essential	GO:004683	GO:003068	GO:000467	(sce03008)Ribosome biogenesis in eukaryotes
putative	Probable	GO:000003	GO:000032	GO:000003	(sce00513)Various types of N-glycan biosynthe
Pdr8p	Transcrip	GO:003600	GO:000563	GO:0000978 RNA polymerase II proximal promoter sequen	
Dad3p	Essential	GO:005198	GO:004272	GO:0051010 microtubule plus-end binding;GO:0008017 mi	
Cap1p	Alpha sub	GO:005101	GO:000829	GO:005101	(sce04144)Endocytosis
mannosylt	Mannosylt	GO:000003	GO:000013	GO:0000026 alpha-1,2-mannosyltransferase activity;GO:	
protein S	Putative	GO:000612	GO:000574	GO:000817	(sce)Citrate cycle, second carbon oxidation,
Pin3p	Negative	GO:003431	GO:003047	GO:0003674 molecular_function	
Arf famil	Glucose-r	GO:001051	GO:000593	GO:000392	(sce04144)Endocytosis
Yea4p	Uridine d	GO:003422	GO:003017	GO:0005462 UDP-N-acetylglucosamine transmembrane tran	
Yih1p	Negative	GO:000646	GO:000584	GO:1905538 polysome binding;GO:0004860 protein kinase	
Uga3p	Transcrip	GO:000108	GO:000563	GO:0001010 RNA polymerase II sequence-specific DNA-bi	
type 2C p	Mitochond	GO:190418	GO:000575	GO:0004741 [pyruvate dehydrogenase (lipoamide)] phosp	
heat shoc	O-mannosy	GO:003150	GO:000562	GO:0005199 structural constituent of cell wall;GO:001	
Ctk3p	Gamma sub	GO:007161	GO:0070692 CTDK-1 complex;GO:0008024 cyclin/CDK positive trans		
hypotheti	Putative	GO:000815	GO:000557	GO:0003674 molecular_function	
nicotinam	Nicotinic	GO:003462	GO:000563	GO:000030	(sce00760)Nicotinate and nicotinamide metabo
RNA-proce	RNA-bindi	GO:190350	GO:000584	GO:000368	(sce03015)mRNA surveillance pathway
Mba1p	Membrane-	GO:003297	GO:000574	GO:0043022 ribosome binding;GO:0043021 ribonucleoprot	
None	None	None	None	None	None
Mch5p	Plasma me	GO:003221	GO:000588	GO:0032217 riboflavin transmembrane transporter activ	
mitochond	Mitochond	GO:003254	GO:000576	GO:0003735 structural constituent of ribosome;GO:0005	
hypotheti	Putative	GO:000815	GO:000032	GO:0003674 molecular_function	
protein S	Putative	GO:000612	GO:000574	GO:000817	(sce)Citrate cycle, second carbon oxidation,
putative	Nit prote	GO:000652	GO:000573	GO:005015	(Human)(hsa00250)Alanine, aspartate and gluta
ADP-ribos	Phosphata	GO:000638	GO:000557	GO:0047407 ADP-ribosyl-[dinitrogen reductase] hydrola	
seripaupe	Protein c	GO:003150	GO:000927	GO:0005199 structural constituent of cell wall;GO:000	
Atg3p	E2-like e	GO:004480	GO:006190	GO:001977	(sce04136)Autophagy - other; (sce04138)Autoph
putative	Putative	GO:000597	GO:000573	GO:0019888 protein phosphatase regulator activity;GO:	
Bud8p	Protein i	GO:000028	GO:000593	GO:0003674 molecular_function	
Pam18p	Subunit o	GO:003015	GO:000140	GO:0001671 ATPase activator activity;GO:0060590 ATPas	
Ask1p	Essential	GO:005198	GO:004272	GO:0051010 microtubule plus-end binding;GO:0008017 mi	
Dph2p	Protein r	GO:001718	GO:000573	GO:0016740 transferase activity;GO:0003824 catalytic a	
aldehyde	Mitochond	GO:000674	GO:004264	GO:000403	(sce00410)beta-Alanine metabolism; (sce00280)
Pcs60p	Oxalyl-Co	GO:003361	GO:000578	GO:0050203 oxalate-CoA ligase activity;GO:0016405 CoA-	
DUP240 fa	Putative	GO:001605	GO:003066	GO:0003674 molecular_function	
Aps3p	Small sub	GO:000662	GO:003012	GO:000856	(Human)(hsa04142)Lysosome
Cbp3p	Mitochond	GO:003455	GO:006167	GO:0043022 ribosome binding;GO:0043021 ribonucleoprot	
hypotheti	Putative	GO:003421	GO:000562	GO:0005351 carbohydrate:proton symporter activity;GO:	
Rax1p	Protein i	GO:000028	GO:000593	GO:0003674 molecular_function	
Don1p	Meiosis-s	GO:003043	GO:007005	GO:0031624 ubiquitin conjugating enzyme binding;GO:00	
Gis4p	CAAX box	GO:000687	GO:000588	GO:0003674 molecular_function	
trans-hex	Hexapreny	GO:000674	GO:000574	GO:000001	(sce00900)Terpenoid backbone biosynthesis; (
chitobios	Mannosylt	GO:000649	GO:000578	GO:000457	(sce)N-glycan precursor biosynthesis; (M00055
nucleotid	Pyrimidin	GO:000620	GO:000557	GO:000825	(sce00760)Nicotinate and nicotinamide metabo
exocyst s	Essential	GO:009052	GO:000562	GO:0003674 molecular_function	
Dad4p	Essential	GO:005198	GO:004272	GO:0051010 microtubule plus-end binding;GO:0008017 mi	
linker nu	Linker nu	GO:000005	GO:004461	GO:001705	(sce03013)RNA transport
hypotheti	Putative	GO:000815	GO:000557	GO:0003674 molecular_function	
Rav2p	Subunit o	GO:000703	GO:004329	GO:0003674 molecular_function	
snoRNP co	Subunit o	GO:003111	GO:009060	GO:003451	(sce03008)Ribosome biogenesis in eukaryotes
Stp3p	Zinc-fing	GO:000815	GO:000563	GO:0043565 sequence-specific DNA binding;GO:0003677 D	
serine/th	Cdc42p-ac	GO:199087	GO:000593	GO:004836	(sce04011)MAPK signaling pathway - yeast
putative	Putative	GO:000924	GO:000573	GO:001697	(sce00785)Lipoic acid metabolism; (sce01100)M

hypotheti	Protein (	GO:00081	GO:00058	GO:0043022 ribosome binding;GO:0043021 ribonucleoprot
oligo-1,6	Isomalta	GO:00000	GO:00055	GO:00045(sce00052)Galactose metabolism; (sce00500)St
Usv1p	Putative	GO:00614	GO:00056	GO:0043565 sequence-specific DNA binding;GO:0003677 D
Atp25p	Mitochon	GO:00336	GO:00057	GO:0003674 molecular_function
hypotheti	Putative	GO:00081	GO:00057	GO:0003674 molecular_function
serine--t	Cytosoli	GO:00064	GO:00104	GO:00048(sce00970)Aminoacyl-tRNA biosynthesis
type 2C p	Type 2C p	GO:00001	GO:00056	GO:1990439 MAP kinase serine/threonine phosphatase ac
Gpb1p	Multistep	GO:00102	GO:00058	GO:0004862 cAMP-dependent protein kinase inhibitor ac
mannosylt	Putative	GO:00000	GO:00003	GO:00000(sce00514)Other types of O-glycan biosynthes
Fig2p	Cell wall	GO:00007	GO:00092	GO:0003674 molecular_function
hypotheti	Protein (	GO:00081	GO:00055	GO:0003674 molecular_function
Sfg1p	Nuclear p	GO:00071	GO:00056	GO:0003674 molecular_function
Sas4p	Subunit (	GO:00063	GO:00332	GO:0004402 histone acetyltransferase activity;GO:0061
triglycer	Phosphol	GO:00344	GO:00325	GO:00048(sce)Acylglycerol degradation; (M00098)Acylg
t-SNARE s	cis-Golgi	GO:00482	GO:00058	GO:00054(sce04130)SNARE interactions in vesicular tr
GTP-Rho b	Subunit (	GO:00905	GO:00056	GO:0017049 GTP-Rho binding;GO:0005546 phosphatidylino
protein s	Regulato	GO:19034	GO:00314	GO:00435(sce04111)Cell cycle - yeast; (sce04113)Meio
hypotheti	Protein (	GO:00081	GO:00160	GO:0003674 molecular_function
Hot13p	Zinc-bin	GO:00450	GO:00057	GO:0008270 zinc ion binding;GO:0046914 transition met
Ies2p	Protein t	GO:00063	GO:00310	GO:0003677 DNA binding;GO:0003676 nucleic acid bindin
hypotheti	Putative	GO:00070	GO:00160	GO:0003674 molecular_function
Kel2p	Protein t	GO:00903	GO:19906	GO:0003674 molecular_function
Thi73p	Putative	GO:00550	GO:00057	GO:0022857 transmembrane transporter activity;GO:0005
hypotheti	Putative	GO:00081	GO:00055	GO:0003674 molecular_function
DUP240 fa	Putative	GO:00160	GO:00003	GO:0003674 molecular_function
proteasom	Non-ATPa	GO:00432	GO:00085	GO:00315(sce03050)Proteasome
GTPase NE	Member of	GO:00070	GO:00058	GO:0003924 GTPase activity;GO:0005525 GTP binding;GO:
telomeras	Componen	GO:00070	GO:00056	GO:0033680 ATP-dependent DNA/RNA helicase activity;GO
mitochon	Mitochon	GO:00304	GO:00057	GO:0003735 structural constituent of ribosome;GO:0005
CTP synth	Major CTP	GO:00442	GO:00972	GO:00038(sce)Pyrimidine ribonucleotide biosynthesis,
Mic12p	Componen	GO:00424	GO:00616	GO:0003674 molecular_function
putative	Putative	GO:00065	GO:00057	GO:00801(sce00920)Sulfur metabolism; (sce00270)Cyste
Spg1p	Protein r	GO:00081	GO:00319	GO:0003674 molecular_function
Caf120p	Part of t	GO:00321	GO:00059	GO:00046(sce03018)RNA degradation
type I HS	Type I HS	GO:00357	GO:00723	GO:00016(sce04141)Protein processing in endoplasmic
Taf3p	TFIID sub	GO:00511	GO:00056	GO:00162(sce03022)Basal transcription factors
coatomer	Gamma sub	GO:00068	GO:00301	GO:0005198 structural molecule activity;GO:0003674 mo
putative	Putative	GO:00009	GO:00009	GO:0004835 tubulin-tyrosine ligase activity;GO:001688
adenyl-nu	Member of	GO:00104	GO:00058	GO:00007(sce04141)Protein processing in endoplasmic
Cox19p	Protein r	GO:00336	GO:00057	GO:0005507 copper ion binding;GO:0046914 transition m
glucan 1,	Intracel	GO:00059	GO:00056	GO:00043(sce00500)Starch and sucrose metabolism; (sc
translati	Constitu	GO:00000	GO:00306	GO:00430(sce03008)Ribosome biogenesis in eukaryotes
Dip5p	Dicarbox	GO:00707	GO:00003	GO:0015183 L-aspartate transmembrane transporter acti
protein (	Protein (	GO:00454	GO:00057	GO:00191(sce04141)Protein processing in endoplasmic
Kar4p	Transcrip	GO:00073	GO:00058	GO:0003700 DNA-binding transcription factor activity;
Ape3p	Vacuolar	GO:00070	GO:00003	GO:0004177 aminopeptidase activity;GO:0008238 exopept
ATP-depen	Hexameri	GO:00325	GO:00332	GO:0043141 ATP-dependent 5'-3' DNA helicase activity;
carnitine	Outer mit	GO:00009	GO:00057	GO:00040(sce04146)Peroxisome
acetyl-Co	Protein w	GO:00196	GO:00057	GO:00087(sce00620)Pyruvate metabolism
SCF ubiqu	F-Box pro	GO:00311	GO:00190	GO:0004842 ubiquitin-protein transferase activity;GO:
thioredox	Cytoplasm	GO:00800	GO:00001	GO:0015035 protein disulfide oxidoreductase activity;
beclin 1	Subunit (	GO:00069	GO:00342	GO:00036(sce04136)Autophagy - other; (sce04138)Autop
isoleucin	Cytoplasm	GO:00064	GO:00058	GO:00048(sce00970)Aminoacyl-tRNA biosynthesis
prephenat	Prephenat	GO:00065	GO:00057	GO:00089(sce00400)Phenylalanine, tyrosine and trypto

Abm1p	Protein (	GO:000022	GO:000557	GO:0003674	molecular_function
DNA-bindi	Transcrip	GO:000707	GO:000563	GO:003603	(sce04111)Cell cycle - yeast
hypotheti	Putative	GO:000815	GO:000557	GO:0003674	molecular_function
Ncs2p	Protein i	GO:000214	GO:000582	GO:001678	(sce04122)Sulfur relay system
U4/U6-U5	Lsm (Like	GO:003390	GO:000568	GO:000372	(sce03018)RNA degradation; (sce03040)Spliceos
Spg3p	Protein i	GO:000815	GO:000578	GO:0003674	molecular_function
hypotheti	Putative protein of unknown function with similarity to F-box proteins; interact				
threonine	Threonyl-	GO:000643	GO:000573	GO:000482	(sce00970)Aminoacyl-tRNA biosynthesis
U4/U6-U5	17-kDa co	GO:000039	GO:004654	GO:000367	(sce03040)Spliceosome
Vps45p	Protein (	GO:003554	GO:003120	GO:000014	(sce04144)Endocytosis; (sce04138)Autophagy -
Rim13p	Calpain-l	GO:001648	GO:000557	GO:0004198	calcium-dependent cysteine-type endopeptida
Mpo1p	Protein i	GO:004652	GO:000578	GO:0003674	molecular_function
F1F0 ATP	Gamma sub	GO:001598	GO:000575	GO:004696	(sce00190)Oxidative phosphorylation; (sce0110
Ioc3p	Subunit o	GO:000706	GO:003643	GO:0031491	nucleosome binding;GO:0016887 ATPase activ
high-affi	High-affi	GO:001567	GO:000588	GO:0005375	copper ion transmembrane transporter activ
Rtg1p	Transcrip	GO:007140	GO:000563	GO:0000981	DNA-binding transcription factor activity,
glutathio	Possible	GO:006172	GO:000093	GO:0019172	glyoxalase III activity;GO:0016836 hydro-l
putative	Putative	GO:003254	GO:000576	GO:000373	(sce03010)Ribosome
None	None	None	None	None	None
translati	Gamma sub	GO:004590	GO:004361	GO:003130	(sce03013)RNA transport
Lrs4p	Nucleolar	GO:003450	GO:003355	GO:000367	(sce04113)Meiosis - yeast
Yhi9p	Protein o	GO:003090	GO:000557	GO:0016853	isomerase activity;GO:0003824 catalytic ac
Vps62p	Vacuolar	GO:000662	GO:000032	GO:0003674	molecular_function
protein a	Protein a	GO:001991	GO:003217	GO:003524	(sce03013)RNA transport; (sce04011)MAPK signa
Vms1p	Component	GO:007267	GO:003626	GO:0004045	aminoacyl-tRNA hydrolase activity;GO:00048
Pex19p	Chaperone	GO:004504	GO:199041	GO:003332	(sce04146)Peroxisome
25S rRNA	Methyltra	GO:007047	GO:000573	GO:0070042	rRNA (uridine-N3-)-methyltransferase activ
Stm1p	Protein i	GO:004355	GO:000584	GO:0045142	triplex DNA binding;GO:0042162 telomeric D
condensin	Subunit o	GO:000713	GO:000079	GO:001688	(sce04111)Cell cycle - yeast
bifunctio	Protein t	GO:003297	GO:000574	GO:0008320	protein transmembrane transporter activity
Alf1p	Alpha-tub	GO:000702	GO:000587	GO:0043014	alpha-tubulin binding;GO:0015631 tubulin b
None	None	None	None	None	None
condensin	Subunit o	GO:200037	GO:000079	GO:007258	(sce04111)Cell cycle - yeast
Mcp2p	Mitochond	GO:005508	GO:003259	GO:0003674	molecular_function
Rtn2p	Reticulon	GO:007178	GO:003254	GO:0003674	molecular_function
None	None	None	None	None	None
glutamate	NAD(+)-de	GO:009705	GO:000573	GO:001604	(sce00910)Nitrogen metabolism; (sce00250)Ala
hypotheti	Putative	GO:000815	GO:000557	GO:0003674	molecular_function
guanine n	Guanyl-nu	GO:000688	GO:000593	GO:0070273	phosphatidylinositol-4-phosphate binding;G
DNA helic	DNA helic	GO:007193	GO:004355	GO:0043141	ATP-dependent 5'-3' DNA helicase activity;
Rim4p	Putative	GO:000627	GO:001660	GO:0003723	RNA binding;GO:0003676 nucleic acid bindin
Spo74p	Component	GO:003043	GO:003597	GO:0005198	structural molecule activity;GO:0003674 mo
Spn1p	Protein i	GO:200125	GO:000563	GO:0000993	RNA polymerase II complex binding;GO:00431
hypotheti	Putative	GO:199053	GO:000579	GO:0089721	phosphoenolpyruvate transmembrane transpor
trifuncti	Mitochond	GO:000925	GO:000573	GO:000447	(sce)C1-unit interconversion, eukaryotes; (M
hypotheti	Dubious o	GO:000815	GO:000557	GO:0003674	molecular_function
Coa1p	Mitochond	GO:003361	GO:003130	GO:0003674	molecular_function
DNA repai	Subunit o	GO:007113	GO:003091	GO:0019789	SUMO transferase activity;GO:0003684 damag
None	None	None	None	None	None
Ytm1p	Constitue	GO:004227	GO:007054	GO:0003674	molecular_function
D-arabino	D-Arabino	GO:007048	GO:003130	GO:0003885	D-arabinono-1,4-lactone oxidase activity;G
None	None	None	None	None	None
ubiquitin	Ubiquitin	GO:001063	GO:000573	GO:0004843	thiol-dependent ubiquitin-specific proteas
U2 snRNP	Protein i	GO:000024	GO:000568	GO:000372	(sce03040)Spliceosome

omega-cla	Omega cla	GO:00067	GO:00057	GO:0004364 glutathione transferase activity;GO:001676	
Atg29p	Autophagy	GO:00344	GO:199031	GO:00001	(sce04138)Autophagy - yeast
Set4p	Protein (	GO:00063	GO:00055	GO:0046872 metal ion binding;GO:0043169 cation bindin	
DNA-direc	RNA polym	GO:00427	GO:00056	GO:00039	(sce03020)RNA polymerase; (sce00240)Pyrimidi
Trm2p	tRNA meth	GO:00304	GO:00055	GO:0030697 S-adenosylmethionine-dependent tRNA (m5U54	
Dap2p	Dipeptidy	GO:00164	GO:00003	GO:00082	(Human)(hsa04974)Protein digestion and absor
Opy1p	Protein (	GO:190264	GO:00057	GO:0005546 phosphatidylinositol-4,5-bisphosphate bind	
Erp2p	Member of	GO:00066	GO:00301	GO:0003674 molecular_function	
Ptm1p	Protein (	GO:00081	GO:00319	GO:0003674 molecular_function	
protein k	Protein k	GO:00723	GO:00056	GO:0072354 histone kinase activity (H3-T3 specific);G	
F1FO ATP	Subunit k	GO:00336	GO:00002	GO:00469	(sce00190)Oxidative phosphorylation; (sce011
None	None	None	None	None	None
Mid2p	O-glycosy	GO:00007	GO:00433	GO:00048	(sce04011)MAPK signaling pathway - yeast
Sps18p	Protein (	GO:00320	GO:00001	GO:0005096 GTPase activator activity;GO:0030695 GTPas	
hypotheti	Putative	GO:00064	GO:00056	GO:0003674 molecular_function	
Acf4p	Protein (	GO:00081	GO:00055	GO:0003674 molecular_function	
Och1p	Mannosylt	GO:00064	GO:00001	GO:00331	(sce)N-glycan biosynthesis, high-mannose typ
Axl1p	Haploid s	GO:00714	GO:00433	GO:0004222 metalloendopeptidase activity;GO:0008237 m	
putative	Putative	GO:00063	GO:00056	GO:0000981 DNA-binding transcription factor activity,	
protein k	Putative	GO:00300	GO:00056	GO:0004674 protein serine/threonine kinase activity;G	
chromatin	DNA damag	GO:00315	GO:00007	GO:00423	(sce04111)Cell cycle - yeast; (sce04113)Meio
Crt10p	Protein i	GO:00063	GO:00055	GO:0003674 molecular_function	
Yrr1p	Zn2-Cys6	GO:00436	GO:00056	GO:0000978 RNA polymerase II proximal promoter sequen	
Ypd1p	Osmotic s	GO:00072	GO:00056	GO:00099	(sce04011)MAPK signaling pathway - yeast
deubiquit	Subunit (	GO:00458	GO:00466	GO:0004402 histone acetyltransferase activity;GO:0061	
Snl1p	Ribosome-	GO:00069	GO:00319	GO:00430	(sce04141)Protein processing in endoplasmic
rRNA-proc	Endonucle	GO:00004	GO:00306	GO:00045	(sce03008)Ribosome biogenesis in eukaryotes
telomeras	Essential	GO:00004	GO:00057	GO:0010521 telomerase inhibitor activity;GO:0004857 e	
Swc4p	Component	GO:00439	GO:00352	GO:0003714 transcription corepressor activity;GO:0003	
Dsl1p	Periphera	GO:00070	GO:19904	GO:0003674 molecular_function	
putative	Nucleolar	GO:00000	GO:00306	GO:0004004 ATP-dependent RNA helicase activity;GO:000	
sphingani	Dihydrosp	GO:00468	GO:00057	GO:00423	(sce00600)Sphingolipid metabolism
ssDNA enc	Endonucle	GO:00312	GO:00308	GO:0000014 single-stranded DNA endodeoxyribonuclease	
None	None	None	None	None	None
signal pe	Subunit (	GO:00064	GO:00057	GO:00082	(sce03060)Protein export
mitochond	Mitochond	GO:00325	GO:00057	GO:0003735 structural constituent of ribosome;GO:0005	
serine/th	Protein k	GO:00512	GO:00058	GO:00046	(sce04392)Hippo signaling pathway -multiple
Sok1p	Protein (	GO:00199	GO:00056	GO:0003674 molecular_function	
cationic	Putative	GO:00344	GO:00716	GO:0015174 basic amino acid transmembrane transporter	
Gzf3p	GATA zinc	GO:00902	GO:00056	GO:0000978 RNA polymerase II proximal promoter sequen	
hypotheti	Protein (	GO:00070	GO:00057	GO:0003674 molecular_function	
phosphori	Putative	GO:00344	GO:00057	GO:1990838 poly(U)-specific exoribonuclease activity,	
hypotheti	Putative	GO:00081	GO:00056	GO:0003674 molecular_function	
ribonucle	Subunit (	GO:00349	GO:00056	GO:00045	(sce03008)Ribosome biogenesis in eukaryotes;
Cwp1p	Cell wall	GO:00321	GO:00715	GO:0005199 structural constituent of cell wall;GO:000	
putative	ATP-deper	GO:19902	GO:00356	GO:0033682 ATP-dependent 5'-3' DNA/RNA helicase activ	
Vps71p	Nucleosor	GO:00310	GO:00008	GO:0031491 nucleosome binding;GO:0042826 histone deac	
Pet117p	Protein r	GO:00324	GO:00996	GO:0003674 molecular_function	
Sdh6p	Mitochond	GO:00345	GO:00057	GO:0003674 molecular_function	
rRNA-proc	Component	GO:00064	GO:00344	GO:00037	(sce03008)Ribosome biogenesis in eukaryotes
putative	Non-esser	GO:00715	GO:00057	GO:0016887 ATPase activity;GO:0017111 nucleoside-trip	
Apl2p	Beta-adap	GO:00068	GO:00301	GO:0030276 clathrin binding;GO:0005515 protein bindin	
Tao3p	Component	GO:00071	GO:00433	GO:0003674 molecular_function	
Cmc1p	Copper-bi	GO:00336	GO:00313	GO:0005507 copper ion binding;GO:0046914 transition m	

Swi3p	Subunit (	GO:00314	GO:00165	GO:0015616 DNA translocase activity;GO:0008094 DNA-de	
Atg7p	Autophagy	GO:00448	GO:00004	GO:00197	(sce04136)Autophagy - other; (sce04138)Autop
methylis	2-methyli	GO:00196	GO:00057	GO:00464	(M00012)Glyoxylate cycle; (sce)Glyoxylate cy
Lsm1p	Lsm (Like	GO:00002	GO:19907	GO:00003	(sce03018)RNA degradation
septin CI	Componen	GO:00009	GO:00056	GO:00103	(Human)(hsa04215)Apoptosis - multiple specie
transcri	Transcri	GO:00719	GO:00309	GO:00012	(sce04111)Cell cycle - yeast
tyrosine/	Dual-spec	GO:00109	GO:00056	GO:00170	(sce04011)MAPK signaling pathway - yeast
Nce102p	Protein (	GO:00610	GO:00702	GO:0003674 molecular_function	
galactos	DNA-bindi	GO:00004	GO:00056	GO:0001085 RNA polymerase II transcription factor bin	
Kar3p	Minus-end	GO:00007	GO:00058	GO:0008569 ATP-dependent microtubule motor activity,	
None	None	None	None	None	None
U5 snRNP	Componen	GO:00002	GO:00056	GO:0003674 molecular_function	
ribosoma	Ribosoma	GO:00422	GO:00306	GO:00037	(sce03010)Ribosome
metallo-(	Putative	GO:00081	GO:00057	GO:0003876 AMP deaminase activity;GO:0047623 adenosin	
mitogen-a	Mitogen-a	GO:00715	GO:00425	GO:00047	(sce04933)AGE-RAGE signaling pathway in diab
superoxi	Mitochon	GO:00013	GO:00057	GO:00047	(sce04213)Longevity regulating pathway - mul
Rba50p	Protein i	GO:00063	GO:00057	GO:0003674 molecular_function	
Mtr2p	mRNA tra	GO:00000	GO:00422	GO:00085	(sce03015)mRNA surveillance pathway; (sce030
putative	N-formyl	GO:00304	GO:00055	GO:00044	(sce00380)Tryptophan metabolism; (sce00071)F
adrenodo	Ferredox	GO:00067	GO:00057	GO:0016653 oxidoreductase activity, acting on NAD(P)H	
Okp1p	Outer kin	GO:00086	GO:00008	GO:0003674 molecular_function	
Nnf2p	Protein t	GO:00081	GO:00057	GO:0003674 molecular_function	
Cup2p	Copper-bi	GO:00068	GO:00056	GO:0005507 copper ion binding;GO:0000978 RNA polymera	
Atg2p	Periphera	GO:00617	GO:00619	GO:00322	(sce04136)Autophagy - other; (sce04138)Autop
putative	Vacuolar	GO:00109	GO:19908	GO:0015095 magnesium ion transmembrane transporter ac	
ER oxido	Thiol ox	GO:00349	GO:00057	GO:00169	(Human)(hsa05110)Vibrio cholerae infection;
mitochon	Mitochon	GO:00325	GO:00057	GO:0008168 methyltransferase activity;GO:0016741 tran	
putative	Probable	GO:00701	GO:00057	GO:00048	(sce00970)Aminoacyl-tRNA biosynthesis
hypothet	Zinc-clus	GO:00063	GO:00056	GO:0000981 DNA-binding transcription factor activity,	
Ucc1p	F-box pro	GO:00714	GO:00190	GO:0030674 protein binding, bridging;GO:0004842 ubiqu	
None	None	None	None	None	None
Skg6p	Integral	GO:19024	GO:00059	GO:0003674 molecular_function	
Rmr1p	Protein r	GO:00063	GO:00056	GO:0003674 molecular_function	
F1F0 ATP	Beta sub	GO:00159	GO:00057	GO:00469	(sce00190)Oxidative phosphorylation; (sce011
adenine	Adenine	GO:00442	GO:00056	GO:00020	(sce00230)Purine metabolism; (sce01100)Metab
GTPase-a	Rab esco	GO:00183	GO:00059	GO:0004663 Rab geranylgeranyltransferase activity;GO:	
trehalos	Regulato	GO:00704	GO:00059	GO:00038	(sce00500)Starch and sucrose metabolism; (sc
aldo-ket	Putative	GO:00060	GO:00055	GO:0018456 aryl-alcohol dehydrogenase (NAD+) activity	
Com2p	Transcri	GO:00081	GO:00056	GO:0043565 sequence-specific DNA binding;GO:0003677 D	
Tos4p	Putative	GO:00069	GO:00702	GO:0003682 chromatin binding;GO:0005488 binding;GO:00	
hypothet	Novel sub	GO:00067	GO:00716	GO:0003674 molecular_function	
hypothet	Putative	GO:00081	GO:00058	GO:0003674 molecular_function	
putative	Putative	GO:00064	GO:00056	GO:0016746 transferase activity, transferring acyl gr	
hypothet	Putative	GO:00081	GO:00055	GO:0003674 molecular_function	
Cox11p	Protein r	GO:00090	GO:00057	GO:00055	(sce00190)Oxidative phosphorylation; (sce011
Tmc1p	AN1-type	GO:00712	GO:00056	GO:0008270 zinc ion binding;GO:0046914 transition met	
Nop13p	Nucleola	GO:00422	GO:00306	GO:0019843 rRNA binding;GO:0003723 RNA binding;GO:000	
Bcd1p	Essentia	GO:00004	GO:00707	GO:0046872 metal ion binding;GO:0043169 cation bindin	
phosphom	Phosphom	GO:00092	GO:00104	GO:00046	(sce00051)Fructose and mannose metabolism; (
translat	Alpha sub	GO:19038	GO:00320	GO:00037	(sce03013)RNA transport
Ndj1p	Protein t	GO:20007	GO:00359	GO:0042162 telomeric DNA binding;GO:0043565 sequence-	
B-type c	B-type c	GO:00062	GO:00003	GO:00165	(sce04011)MAPK signaling pathway - yeast; (s
Mic60p	Componen	GO:00450	GO:00616	GO:0003674 molecular_function	
Kap104p	Transpor	GO:00066	GO:00059	GO:0008139 nuclear localization sequence binding;GO:0	

transcri[illegible]	Transcri[illegible]	GO:00004[illegible]	GO:00166[illegible]	GO:0001102 RNA polymerase II activating transcription	
Dal80p	Negative	GO:00902[illegible]	GO:00056[illegible]	GO:0000978 RNA polymerase II proximal promoter sequen	
None	None	None	None	None	None
hypotheti	Cytoplasm	GO:00081[illegible]	GO:00057[illegible]	GO:0003674 molecular_function	
translati	Mitochon[illegible]	GO:00701[illegible]	GO:00057[illegible]	GO:0003746 translation elongation factor activity;GO:[illegible]	
copper m[illegible]	Cytosoli[illegible]	GO:00068[illegible]	GO:00058[illegible]	GO:0016531 copper chaperone activity;GO:0016530 metal	
Hri1p	Protein of unknown		GO:00058[illegible]	GO:0003674 molecular_function	
transcri[illegible]	Transcri[illegible]	GO:00108[illegible]	GO:00309[illegible]	GO:00037[illegible] (sce04011)MAPK signaling pathway - yeast; (s[illegible]	
diphtham[illegible]	Methyles[illegible]	GO:00171[illegible]	GO:00057[illegible]	GO:0061685 diphthine methylesterase activity;GO:0052[illegible]	
Nop53p	Nucleola[illegible]	GO:00000[illegible]	GO:00057[illegible]	GO:00080[illegible] (Human)(hsa05168)Herpes simplex infection	
hypotheti	Putative	GO:00081[illegible]	GO:00057[illegible]	GO:0003674 molecular_function	
Met4p	Leucine-[illegible]	GO:00193[illegible]	GO:00897[illegible]	GO:0003713 transcription coactivator activity;GO:0003	
Yap1801p	Protein [illegible]	GO:00482[illegible]	GO:00304[illegible]	GO:0005545 1-phosphatidylinositol binding;GO:0032050 [illegible]	
hypotheti	Putative	GO:00081[illegible]	GO:00055[illegible]	GO:0003674 molecular_function	
histone [illegible]	Histone [illegible]	GO:00704[illegible]	GO:00007[illegible]	GO:00469[illegible] (Human)(hsa05322)Systemic lupus erythematosu[illegible]	
None	None	None	None	None	None
Syh1p	Protein [illegible]	GO:00310[illegible]	GO:00009[illegible]	GO:0043022 ribosome binding;GO:0043021 ribonucleoprot	
Pan3p	Essentia[illegible]	GO:00106[illegible]	GO:00312[illegible]	GO:00045[illegible] (sce03018)RNA degradation	
Ipi1p	Componen[illegible]	GO:00062[illegible]	GO:00713[illegible]	GO:0003682 chromatin binding;GO:0005488 binding;GO:00[illegible]	
DNA-depe[illegible]	Protein [illegible]	GO:00012[illegible]	GO:00056[illegible]	GO:00080[illegible] (sce03420)Nucleotide excision repair	
Slo1p	Protein [illegible]	GO:00068[illegible]	GO:00056[illegible]	GO:0003674 molecular_function	
hypotheti	Putative	GO:00081[illegible]	GO:00055[illegible]	GO:0003674 molecular_function	
phosphop[illegible]	Enolase,	GO:00060[illegible]	GO:00000[illegible]	GO:00046[illegible] (M00002)Glycolysis, core module involving th[illegible]	
Ste50p	Adaptor [illegible]	GO:00007[illegible]	GO:00057[illegible]	GO:00320[illegible] (sce04011)MAPK signaling pathway - yeast	
E2 ubiqu[illegible]	Ubiquiti[illegible]	GO:00304[illegible]	GO:00008[illegible]	GO:00616[illegible] (sce04120)Ubiquitin mediated proteolysis; (s[illegible]	
Bxi1p	Protein [illegible]	GO:00309[illegible]	GO:00003[illegible]	GO:0003674 molecular_function	
3-oxoacy[illegible]	Mitochon[illegible]	GO:00066[illegible]	GO:00057[illegible]	GO:00043[illegible] (sce)Fatty acid biosynthesis, elongation; (M[illegible]	
oxidativ[illegible]	Fe(II)/2-	GO:00355[illegible]	GO:00056[illegible]	GO:0035516 oxidative DNA demethylase activity;GO:0035	
Svs1p	Cell wal[illegible]	GO:00081[illegible]	GO:00092[illegible]	GO:0003674 molecular_function	
sulfate [illegible]	High aff[illegible]	GO:19023[illegible]	GO:00058[illegible]	GO:0008271 secondary active sulfate transmembrane tra	
Smp1p	MADS-box	GO:00613[illegible]	GO:00056[illegible]	GO:00083[illegible] (sce04011)MAPK signaling pathway - yeast	
lysophos[illegible]	Serine e[illegible]	GO:00346[illegible]	GO:00057[illegible]	GO:00046[illegible] (sce00564)Glycerophospholipid metabolism	
Y' eleme[illegible]	Helicase	GO:00007[illegible]	GO:00057[illegible]	GO:0003678 DNA helicase activity;GO:0004386 helicase [illegible]	
cytochro[illegible]	Subunit [illegible]	GO:00061[illegible]	GO:00057[illegible]	GO:00154[illegible] (sce00190)Oxidative phosphorylation; (sce011[illegible]	
DNA repl[illegible]	Subunit [illegible]	GO:00007[illegible]	GO:00711[illegible]	GO:0043138 3'-5' DNA helicase activity;GO:0003678 DNA	
S-methyl-	Methylth[illegible]	GO:00195[illegible]	GO:00058[illegible]	GO:00170[illegible] (sce)Methionine salvage pathway; (M00034)Met[illegible]	
Pep7p	Adaptor [illegible]	GO:00000[illegible]	GO:00100[illegible]	GO:00322[illegible] (sce04144)Endocytosis	
Seg2p	Eisosome	GO:00081[illegible]	GO:00058[illegible]	GO:0003674 molecular_function	
Puf4p	Member o[illegible]	GO:00013[illegible]	GO:00057[illegible]	GO:0003729 mRNA binding;GO:0003723 RNA binding;GO:000	
Age2p	ADP-ribo[illegible]	GO:00068[illegible]	GO:00301[illegible]	GO:00050[illegible] (sce04144)Endocytosis	
None	None	None	None	None	None
glutamin[illegible]	Componen[illegible]	GO:00067[illegible]	GO:00616[illegible]	GO:0036374 glutathione hydrolase activity;GO:0016805 [illegible]	
Med11p	Subunit [illegible]	GO:00511[illegible]	GO:00708[illegible]	GO:0001097 TFIIH-class transcription factor complex b	
Mge1p	Mitochon[illegible]	GO:00301[illegible]	GO:00014[illegible]	GO:0000774 adenyl-nucleotide exchange factor activity	
acetyl-C[illegible]	Acetyl-C[illegible]	GO:20012[illegible]	GO:00057[illegible]	GO:00039[illegible] (sce)Fatty acid biosynthesis, initiation; (M[illegible]	
None	None	None	None	None	None
Mgm101p	Protein [illegible]	GO:00007[illegible]	GO:00002[illegible]	GO:0003697 single-stranded DNA binding;GO:0003677 DNA	
hexose t[illegible]	Hexose t[illegible]	GO:00157[illegible]	GO:00058[illegible]	GO:00155[illegible] (sce04113)Meiosis - yeast	
hypotheti	Putative	GO:00081[illegible]	GO:00003[illegible]	GO:0003674 molecular_function	
snoRNA-b[illegible]	Nucleola[illegible]	GO:00459[illegible]	GO:00344[illegible]	(Human)G[illegible] (sce03008)Ribosome biogenesis in eukaryotes	
putative	Putative	GO:00160[illegible]	GO:00055[illegible]	GO:0016298 lipase activity;GO:0016788 hydrolase activ	
Rrp17p	Componen[illegible]	GO:00070[illegible]	GO:00057[illegible]	GO:0019843 rRNA binding;GO:0003697 single-stranded DN[illegible]	
Spr2p	Putative	GO:00081[illegible]	GO:00092[illegible]	GO:0003674 molecular_function	
ATP-depe[illegible]	RecQ fam[illegible]	GO:19032[illegible]	GO:00314[illegible]	GO:00431[illegible] (sce03440)Homologous recombination	

sorting r	Nexin-1 h	GO:00450	GO:00309	GO:00322	(sce04144)Endocytosis
Clg1p	Cyclin-li	GO:00162	GO:00003	GO:0016538 cyclin-dependent protein serine/threonine	
None	None	None	None	None	None
mitochond	Mitochond	GO:00325	GO:00057	GO:0003735 structural constituent of ribosome;GO:0005	
nucleosom	Nucleosom	GO:00350	GO:00007	GO:0042393 histone binding;GO:0005515 protein binding	
Hlr1p	Protein i	GO:00315	GO:00057	GO:0003674 molecular_function	
hypotheti	Putative	GO:00081	GO:00056	GO:0003674 molecular_function	
Rad17p	Checkpoin	GO:00071	GO:00308	GO:00036	(sce04111)Cell cycle - yeast; (sce04113)Meio
Tpo4p	Polyamine	GO:19037	GO:00003	GO:0015606 spermidine transmembrane transporter activ	
Arr2p	Arsenate	GO:00466	GO:00056	GO:0030611 arsenate reductase activity;GO:0004725 pro	
Rab fami	Rab GTPas	GO:00483	GO:00059	GO:0003924 GTPase activity;GO:0005525 GTP binding;GO:	
protein i	Protein i	GO:00066	GO:00301	GO:0003674 molecular_function	
Sps19p	Peroxisom	GO:00304	GO:00057	GO:00086	(sce04146)Peroxisome
hypotheti	Putative	GO:00081	GO:00312	GO:0003674 molecular_function	
Tma23p	Nucleolar	GO:00422	GO:00058	GO:0043022 ribosome binding;GO:0043021 ribonucleoprot	
Irc8p	Bud tip l	GO:00063	GO:00059	GO:0003674 molecular_function	
peptidylp	Peptidyl-	GO:00436	GO:00001	GO:00081	(Human)(hsa04931)Insulin resistance
B-type cy	B-type cy	GO:00106	GO:00003	GO:00165	(sce04011)MAPK signaling pathway - yeast; (s
25S rRNA	Methyltra	GO:00704	GO:00056	GO:0070042 rRNA (uridine-N3-)-methyltransferase activ	
hypotheti	Putative	GO:00081	GO:00055	GO:0003674 molecular_function	
Golgi tra	Essential	GO:00003	GO:00171	GO:0003674 molecular_function	
serine/th	Regulator	GO:00712	GO:01200	GO:00198	(sce04136)Autophagy - other; (sce04138)Autoph
hypotheti	Putative	GO:00081	GO:00055	GO:0003674 molecular_function	
transcrip	TFIIA lar	GO:00511	GO:00056	GO:00170	(sce03022)Basal transcription factors
Bre4p	Zinc fing	GO:00068	GO:00719	GO:0003674 molecular_function	
olichyl-d	Subunit o	GO:00064	GO:00057	GO:00045	(M00072)N-glycosylation by oligosaccharyltran
imidazole	Imidazole	GO:00001	GO:00055	GO:00044	(sce)Histidine biosynthesis, PRPP => histidin
pheromone	Pheromone	GO:00007	GO:00160	GO:0003674 molecular_function	
mitochond	Mitochond	GO:00325	GO:00057	GO:0003735 structural constituent of ribosome;GO:0005	
Tda7p	Cell cycl	GO:00081	GO:00003	GO:0003674 molecular_function	
Tda2p	Protein c	GO:00308	GO:00304	GO:0051015 actin filament binding;GO:0003779 actin bi	
ribosomal	Ribosomal	GO:00004	GO:00306	GO:00198	(sce03010)Ribosome
Skg1p	Transmemb	GO:00315	GO:00331	GO:0003674 molecular_function	
hypotheti	Putative	GO:00081	GO:00055	GO:0003674 molecular_function	
hypotheti	Putative	GO:00081	GO:00057	GO:0003674 molecular_function	
GPI-ancho	Subunit o	GO:00162	GO:00427	GO:00039	(sce00563)Glycosylphosphatidylinositol (GPI)-
hypotheti	Putative	GO:00081	GO:00056	GO:0046872 metal ion binding;GO:0043169 cation bindin	
Yrb2p	Protein c	GO:00000	GO:00056	GO:0008536 Ran GTPase binding;GO:0005096 GTPase activ	
Cnl1p	Subunit o	GO:00070	GO:00310	GO:0003674 molecular_function	
ESCRT-0 s	Subunit o	GO:19046	GO:00335	GO:00431	(sce04144)Endocytosis
COPII sub	Component	GO:00901	GO:00301	GO:00050	(sce04141)Protein processing in endoplasmic r
tRNA dihy	Dihydrour	GO:00029	GO:00343	GO:0102265 tRNA-dihydrouridine47 synthase activity;GO	
proteasom	Beta 6 su	GO:00104	GO:00345	GO:00042	(sce03050)Proteasome
None	None	None	None	None	None
dolichyl-	Delta sub	GO:00064	GO:00082	GO:00045	(M00072)N-glycosylation by oligosaccharyltran
F1F0 ATP	Subunit e	GO:00159	GO:00002	GO:00469	(sce00190)Oxidative phosphorylation
glutathio	Proton:gl	GO:00347	GO:00057	GO:0015343 siderophore transmembrane transporter acti	
Epo1p	Protein i	GO:00611	GO:00059	GO:0003674 molecular_function	
putative	Putative	GO:00158	GO:00057	GO:0015230 FAD transmembrane transporter activity;GO:	
Kre1p	Cell wall	GO:00315	GO:00092	GO:0005199 structural constituent of cell wall;GO:000	
putative	Protein w	GO:00092	GO:00055	GO:0050334 thiaminase activity;GO:0008972 phosphometh	
alcohol d	Alcohol d	GO:00009	GO:00057	GO:00040	(sce00350)Tyrosine metabolism; (sce00071)Fat
tRNA-spec	tRNA-spec	GO:00064	GO:00056	GO:0043829 tRNA-specific adenosine-37 deaminase activ	
D-xylulos	Xylitol d	GO:00195	GO:00055	GO:00465	(sce00040)Pentose and glucuronate interconve

copper m Copper m GO:00156 GO:00057 GO:00165 (sce00190)Oxidative phosphorylation; (sce011
Per33p Protein GO:00510 GO:00056 GO:0003674 molecular_function
phosphati Phosphat GO:00360 GO:00707 GO:0043813 phosphatidylinositol-3,5-bisphosphate 5-ph
Lsb5p Protein GO:00516 GO:00304 GO:0003674 molecular_function
Sqs1p Protein GO:00510 GO:00306 GO:0003676 nucleic acid binding;GO:1901363 heterocycl
Mcm10p Essentia GO:00304 GO:00312 GO:0003688 DNA replication origin binding;GO:0003697
RNA heli Predomina GO:00459 GO:00057 GO:0034459 ATP-dependent 3'-5' RNA helicase activity;
Gsm1p Putative GO:00061 GO:00056 GO:0000981 DNA-binding transcription factor activity,
putative PTEN hom GO:00304 GO:00057 GO:0016314 phosphatidylinositol-3,4,5-trisphosphate 3
Itt1p Protein GO:00064 GO:00001 GO:0031624 ubiquitin conjugating enzyme binding;GO:00
mitochon Mitochon GO:00325 GO:00057 GO:00037 (sce03010)Ribosome
proteaso Non-ATPa GO:00432 GO:00345 GO:00051 (sce03050)Proteasome
transcri Spt4p/5p GO:20012 GO:00320 GO:0001179 RNA polymerase I transcription factor bind
alpha-1, Subunit GO:00000 GO:00001 GO:00000 (sce)N-glycan biosynthesis, high-mannose typ
rRNA-bin Protein GO:00000 GO:00306 GO:0019843 rRNA binding;GO:0003723 RNA binding;GO:000
hypothet Mitochon GO:00325 GO:00057 GO:0003735 structural constituent of ribosome;GO:0005
proteaso Alpha 3 GO:00801 GO:00345 GO:00042 (sce03050)Proteasome
chromati Swi2/Snf GO:00434 GO:00008 GO:0004386 helicase activity;GO:0042393 histone bindi
Ady2p Acetate GO:00068 GO:00057 GO:0015123 acetate transmembrane transporter activity
AAA fami AAA ATPa GO:00482 GO:00433 GO:00703 (sce04138)Autophagy - yeast
Tip20p Periphera GO:19025 GO:00709 GO:0005515 protein binding;GO:0005488 binding;GO:0003
enoyl-Co Essentia GO:00304 GO:00003 GO:01021 (sce)Fatty acid biosynthesis, elongation, en
Sdd4p Putative GO:00063 GO:00056 GO:0008270 zinc ion binding;GO:0043565 sequence-speci
Yap1802p Protein GO:00482 GO:00304 GO:0005545 1-phosphatidylinositol binding;GO:0032050
End3p EH domai GO:00071 GO:00304 GO:0030674 protein binding, bridging;GO:0003779 actin
translat Translat GO:00340 GO:00162 GO:00037 (sce03013)RNA transport
putative Putative GO:00065 GO:00057 GO:0004066 asparagine synthase (glutamine-hydrolyzing
serine/t Protein GO:00304 GO:00057 GO:00046 (sce04113)Meiosis - yeast
Sap155p Protein GO:00000 GO:0005737 cytoplasm;GO:0005622 intracellular;GO:0044424 intra
Pea2p Coiled-c GO:00313 GO:0000133 polarisome;GO:0005934 cellular bud tip;GO:0043332 m
chitin s Chitin s GO:00000 GO:00304 GO:00041 (sce00520)Amino sugar and nucleotide sugar m
lysophos Lysophos GO:00359 GO:00057 GO:0003841 1-acylglycerol-3-phosphate O-acyltransfera
ribosoma Protein GO:00021 GO:00104 GO:00037 (sce03010)Ribosome
Nat4p N alpha- GO:00611 GO:00056 GO:0043998 H2A histone acetyltransferase activity;GO:
E3 ubiqu Ubiquiti GO:00309 GO:00008 GO:00616 (sce04120)Ubiquitin mediated proteolysis; (s
Erc1p Member o GO:00065 GO:00160 GO:0015297 antiporter activity;GO:0015238 drug transm
hypothet Putative GO:00457 GO:00346 GO:0003674 molecular_function
serine/t Cell-cyc GO:00484 GO:00056 GO:00046 (sce04111)Cell cycle - yeast
Nup170p Subunit GO:00708 GO:00446 GO:19908 (sce03013)RNA transport
U2-type Spliceos GO:00003 GO:00710 GO:0003723 RNA binding;GO:0003676 nucleic acid bindin
Pho2p Homeobox GO:00001 GO:00058 GO:00009 (sce04111)Cell cycle - yeast
Vps51p Componen GO:00071 GO:00100 GO:0005515 protein binding;GO:0005488 binding;GO:0003
ribosome Protein GO:00000 GO:00306 GO:00430 (sce03008)Ribosome biogenesis in eukaryotes;
Tco89p Subunit GO:00060 GO:00003 GO:0003674 molecular_function
epsin Epsin-li GO:00001 GO:00059 GO:00431 (sce04144)Endocytosis
Sed4p Integral GO:00034 GO:00301 GO:00050 (sce04141)Protein processing in endoplasmic
SUMO fam Ubiquiti GO:00169 GO:00059 GO:00313 (sce03013)RNA transport
Rot1p Molecula GO:00071 GO:00057 GO:0051082 unfolded protein binding;GO:0005515 protei
Edc3p Non-esse GO:19001 GO:00009 GO:00037 (sce03018)RNA degradation
Vps54p Componen GO:00304 GO:00009 GO:0019905 syntaxin binding;GO:0000149 SNARE binding;
Fdo1p Protein GO:00075 GO:00056 GO:0003674 molecular_function
anthrani Anthrani GO:00001 GO:00058 GO:00040 (sce)Tryptophan biosynthesis, chorismate =>
putative Putative GO:00510 GO:00059 GO:0008568 microtubule-severing ATPase activity;GO:00
Srb4p Subunit GO:00511 GO:00708 GO:0001139 RNA polymerase II complex recruiting activ

Srb7p	Subunit (	GO:00001:	GO:00708	GO:0001103	RNA polymerase II repressing transcription
hypotheti	Protein (	GO:00081!	GO:00358:	GO:0003674	molecular_function
phosphop	Catalyti	GO:19026(	GO:00302	GO:00047	(Human)(hsa04922)Glucagon signaling pathway
1,3-beta-	1,3-beta-	GO:00719	GO:00092	GO:0042124	1,3-beta-glucanosyltransferase activity;GO
phosphati	Phosphat	GO:00106:	GO:00313(	GO:00046(	(M00093)Phosphatidylethanolamine (PE) biosyn
Rme1p	Zinc fing	GO:00360	GO:00056:	GO:00009	(sce04113)Meiosis - yeast
hypotheti	Putative	GO:00081!	GO:00003:	GO:0003674	molecular_function
Imp2'p	Transcri	GO:00004:	GO:00057:	GO:0003713	transcription coactivator activity;GO:0003
Utp20p	Componen	GO:00004	GO:00306	GO:0003729	mRNA binding;GO:0003723 RNA binding;GO:000
hypotheti	Putative	GO:00081!	GO:00055	GO:0003674	molecular_function
snoRNA-b	Componen	GO:00422	GO:00344!	GO:00430	(sce03008)Ribosome biogenesis in eukaryotes
putative	Putative	GO:00352(	GO:00975	GO:00041(	(sce00515)Mannose type O-glycan biosynthesis
SAGA hist	Integral	GO:00165	GO:00001:	GO:0008047	enzyme activator activity;GO:0030234 enzym
replicati	Subunit (	GO:00062	GO:00313	GO:00036	(sce03430)Mismatch repair; (sce03030)DNA rep
mitochon	Mitochon	GO:00325	GO:00057(	GO:0003735	structural constituent of ribosome;GO:0005
Srp40p	Nucleola	GO:00069	GO:00057:	GO:0003674	molecular_function
Cue4p	Protein (	GO:00081!	GO:00057	GO:0003674	molecular_function
aspartyl	GPI-anch	GO:00014(	GO:00092	GO:00041	(sce04011)MAPK signaling pathway - yeast
Ecm1p	Pre-ribo	GO:00000!	GO:00306	GO:0003674	molecular_function
tRNA modi	Mitochon	GO:00708	GO:00057:	GO:0050660	flavin adenine dinucleotide binding;GO:005
None	None	None	None	None	None
hypotheti	Putative	GO:00081!	GO:00055	GO:0003674	molecular_function
seripaup	Member o	GO:00315(	GO:00092	GO:0005199	structural constituent of cell wall;GO:000
FG-nucle	FG-nucle	GO:00319	GO:00446	GO:0017056	structural constituent of nuclear pore;GO:
Rim20p	Protein i	GO:00014(	GO:00058	GO:00036	(sce04144)Endocytosis
ribosoma	Ribosoma	GO:00021	GO:00226	GO:00037:	(sce03010)Ribosome
Rsa4p	WD-repea	GO:00000:	GO:00058	GO:0043022	ribosome binding;GO:0043021 ribonucleoprot
Snt1p	Subunit (	GO:00328	GO:00349(	GO:0042826	histone deacetylase binding;GO:0003714 tra
Isy1p	Member o	GO:00003	GO:00710	GO:00003	(sce03040)Spliceosome
Sqt1p	Specific	GO:00000:	GO:00306	GO:0051082	unfolded protein binding;GO:0005515 protei
sphingoli	Vacuolar	GO:00066(	GO:00003:	GO:00466	(Human)(hsa04142)Lysosome
She10p	Protein i	GO:00304	GO:00057	GO:0003674	molecular_function
Smc5-Smc	Componen	GO:00169:	GO:00309	GO:0019789	SUMO transferase activity;GO:0019787 ubiqu
sphingan	Sphingoi	GO:00468:	GO:00325	GO:00084	(sce)Sphingosine degradation; (M00100)Sphing
Nsr1p	Nucleola	GO:00000:	GO:00056:	GO:0043047	single-stranded telomeric DNA binding;GO:0
tethering	Histone	GO:00362(	GO:00332(	GO:00306	(sce04138)Autophagy - yeast
bifuncti	Choline	GO:00066!	GO:00057:	GO:00043(	(sce)Phosphatidylcholine (PC) biosynthesis,
sphingan	Dihydros	GO:00301	GO:00325	GO:00081	(sce)Sphingosine degradation; (M00100)Sphing
Mrn1p	RNA-bind	GO:00003	GO:00104	GO:0003729	mRNA binding;GO:0003723 RNA binding;GO:000
SUMO-targ	Subunit (	GO:00169:	GO:00337(	GO:0032183	SUMO binding;GO:0032182 ubiquitin-like pro
S-adenos	S-AdoMet-	GO:00550	GO:00057:	GO:0008757	S-adenosylmethionine-dependent methyltrans
Fe(2+) t	Iron tra	GO:00482!	GO:00057	GO:0005381	iron ion transmembrane transporter activit
hypotheti	Putative	GO:00081!	GO:00055	GO:00036	(Human)(hsa00053)Ascorbate and aldarate meta
tRNA met	Protein i	GO:00304	GO:00058:	GO:0003674	molecular_function
Tip41p	Protein	GO:00319:	GO:00058:	GO:00036	(sce04138)Autophagy - yeast
hypotheti	Uncharac	GO:00081!	GO:00003:	GO:0003674	molecular_function
hypotheti	Putative protein of unknown function; the authentic, non-tagged protein is detec				
hypotheti	Putative	GO:00081!	GO:00003:	GO:0003674	molecular_function
Fyv6p	Protein (	GO:00063(	GO:00007	GO:0003674	molecular_function
Ymd8p	Putative	GO:00157	GO:00306(	GO:0005338	nucleotide-sugar transmembrane transporter
tRNA (gu	Noncatal	GO:01060(	GO:01061	GO:0008176	tRNA (guanine-N7-)-methyltransferase activ
putative	Putative	GO:00551	GO:00056:	GO:0016726	oxidoreductase activity, acting on CH or C
MIND com	Essentia	GO:00000	GO:00008	GO:0003674	molecular_function
Arp10p	Componen	GO:00002	GO:00058(	GO:0003774	motor activity;GO:0005200 structural const

Bag7p	Rho GTPa	GO:00350	GO:00055	GO:0005096	GTPase activator activity;GO:0030695 GTPas
Fet4p	Low-affi	GO:00156	GO:00003	GO:0005375	copper ion transmembrane transporter activ
hypothet	Putative	GO:00081	GO:00055	GO:0003674	molecular_function
elongin (	Elongin (	GO:00709	GO:00001	GO:00048	(sce04120)Ubiquitin mediated proteolysis
Rri2p	Subunit	GO:00007	GO:00081	GO:0003674	molecular_function
Rrg8p	Protein	GO:00000	GO:00057	GO:0003674	molecular_function
Mim2p	Mitochon	GO:00450	GO:00313	GO:0003674	molecular_function
hypothet	Protein	GO:00081	GO:00058	GO:0043022	ribosome binding;GO:0043021 ribonucleoprot
Aim25p	Mitochon	GO:19031	GO:00057	GO:0017128	phospholipid scramblase activity;GO:000554
Rrn7p	Componen	GO:00011	GO:00056	GO:0001164	RNA polymerase I CORE element sequence-spe
glycogen	Glycogen	GO:00059	GO:00055	GO:00084	(sce00500)Starch and sucrose metabolism; (sc
DNA liga	DNA liga	GO:19034	GO:00056	GO:00039	(sce03410)Base excision repair; (sce03430)Mi
hypothet	Putative	GO:00081	GO:00055	GO:0003674	molecular_function
Bsc2p	Protein	GO:00081	GO:00058	GO:0003674	molecular_function
ribosoma	Ribosoma	GO:00021	GO:00306	GO:00037	(sce03010)Ribosome
Cbr1p	Cytochro	GO:00551	GO:00057	GO:00041	(sce00520)Amino sugar and nucleotide sugar m
F-actin-	Beta sub	GO:00510	GO:00082	GO:00510	(sce04144)Endocytosis
chromati	Transcri	GO:00350	GO:00466	GO:0004402	histone acetyltransferase activity;GO:0061
peptidyl	Peptidyl-	GO:00004	GO:00058	GO:0016018	cyclosporin A binding;GO:0003755 peptidyl-
Pex32p	Peroxiso	GO:00070	GO:00057	GO:0003674	molecular_function
Sfk1p	Plasma m	GO:00610	GO:00057	GO:0003674	molecular_function
dipeptid	Dipeptid	GO:00065	GO:00056	GO:0008239	dipeptidyl-peptidase activity;GO:0004177 a
Mmr1p	Phosphor	GO:00483	GO:00059	GO:0003674	molecular_function
mitochon	Mitochon	GO:00325	GO:00057	GO:0003735	structural constituent of ribosome;GO:0005
hypothet	Dubious	GO:00081	GO:00055	GO:0003674	molecular_function
Snx41p	Sorting	GO:00421	GO:00100	GO:0032266	phosphatidylinositol-3-phosphate binding;G
protein	Cytoplas	GO:00436	GO:00057	GO:0008157	protein phosphatase 1 binding;GO:0019888 p
Tvp15p	Integral	GO:00161	GO:00301	GO:0003674	molecular_function
ubiquiti	Ubiquiti	GO:00000	GO:00226	GO:00313	(sce03010)Ribosome
None	None	None	None	None	None
Sly41p	Protein	GO:19905	GO:00057	GO:0089721	phosphoenolpyruvate transmembrane transpor
hypothet	Putative	GO:00081	GO:00057	GO:0003674	molecular_function
None	None	None	None	None	None
COPII su	Componen	GO:00901	GO:00301	GO:00050	(sce04141)Protein processing in endoplasmic
anaphase	Catalyti	GO:00458	GO:00056	GO:00976	(sce04120)Ubiquitin mediated proteolysis; (s
phosphat	Membrane	GO:00065	GO:00005	GO:00171	(sce00563)Glycosylphosphatidylinositol (GPI)-
hypothet	Putative	GO:00081	GO:00055	GO:0003674	molecular_function
Big1p	Integral	GO:00060	GO:00301	GO:0003674	molecular_function
E2 ubiqu	Peroxiso	GO:00165	GO:00057	GO:0061631	ubiquitin conjugating enzyme activity;GO:0
SAGA his	Subunit	GO:00007	GO:00466	GO:0046982	protein heterodimerization activity;GO:000
Cmc4p	Protein	GO:00081	GO:00057	GO:0003674	molecular_function
histidin	Histidin	GO:00001	GO:00057	GO:00044	(sce)Histidine biosynthesis, PRPP => histidi
Mch1p	Protein	GO:00157	GO:00003	GO:0008028	monocarboxylic acid transmembrane transpor
asparagi	Cytosoli	GO:00065	GO:00425	GO:00040	(sce00460)Cyanoamino acid metabolism; (sce00
Hch1p	Heat sho	GO:00327	GO:00056	GO:0001671	ATPase activator activity;GO:0060590 ATPas
Pex28p	Peroxiso	GO:00070	GO:00057	GO:0003674	molecular_function
chromati	Componen	GO:00000	GO:00004	GO:0031490	chromatin DNA binding;GO:0003682 chromatin
ferric-c	Ferric r	GO:00158	GO:00003	GO:0052851	ferric-chelate reductase (NADPH) activity;
protein	Protein	GO:00181	GO:00056	GO:0004683	calmodulin-dependent protein kinase activi
Get4p	Protein	GO:00450	GO:00718	GO:0003674	molecular_function
glucan 1,	Endo-bet	GO:00315	GO:00092	GO:00043	(sce00500)Starch and sucrose metabolism
dihydrof	Dihydrof	GO:00067	GO:00057	GO:00088	(M00126)Tetrahydrofolate biosynthesis, GTP =
FACT com	Subunit	GO:00458	GO:00056	GO:0031491	nucleosome binding;GO:0042393 histone bind
adenylat	Adenylat	GO:00061	GO:00057	GO:00040	(sce04213)Longevity regulating pathway - mul

Irc10p	Protein	GO:00081	GO:00700	GO:0003674	molecular_function
Ras fami	GTPase i	GO:00457	GO:00058	GO:00039	(sce04933)AGE-RAGE signaling pathway in diab
putative	Putative	GO:00442	GO:00056	GO:0004849	uridine kinase activity;GO:0019206 nucleos
mismatch	Mismatch	GO:00431	GO:00323	GO:00004	(sce03430)Mismatch repair
amidopho	Phosphor	GO:00061	GO:00057	GO:00040	(M00048)Inosine monophosphate biosynthesis,
Ysc84p	Actin-bi	GO:19000	GO:00304	GO:0051015	actin filament binding;GO:0035091 phosphat
Msa2p	Putative	GO:00000	GO:00056	GO:0003674	molecular_function
None	None	None	None	None	None
1-(5-pho	Enzyme t	GO:00001	GO:00056	GO:00039	(sce)Histidine biosynthesis, PRPP => histidi
Pnt1p	Mitochon	GO:00329	GO:00313	GO:0003674	molecular_function
None	None	None	None	None	None
ribosoma	Protein	GO:00064	GO:00226	GO:00037	(sce03010)Ribosome
U2 snRNP	Componen	GO:00002	GO:00056	GO:00036	(sce03040)Spliceosome
translat	Translat	GO:00017	GO:00332	GO:00313	(sce03013)RNA transport
Lst4p	Subunit	GO:19042	GO:19908	GO:0005096	GTPase activator activity;GO:0030695 GTPas
hypothet	Protein	GO:00066	GO:00723	GO:0003674	molecular_function
Dit1p	Sporulat	GO:00304	GO:00055	GO:0003824	catalytic activity;GO:0003674 molecular_fu
sulfite	Subunit	GO:00193	GO:00093	GO:00047	(M00176)Assimilatory sulfate reduction, sulf
Nit1p	Nitrilas	GO:00068	GO:00055	GO:0000257	nitrilase activity;GO:0016815 hydrolase ac
Gal83p	One of t	GO:20002	GO:00056	GO:0004679	AMP-activated protein kinase activity;GO:0
putative	Member o	GO:00611	GO:00058	GO:0003730	mRNA 3'-UTR binding;GO:0003729 mRNA bindin
serine/t	Protein	GO:00164	GO:00056	GO:0004712	protein serine/threonine/tyrosine kinase a
Slz1p	Sporulat	GO:20002	GO:00363	GO:0005515	protein binding;GO:0005488 binding;GO:0003
hypothet	Putative	GO:00081	GO:00056	GO:0003674	molecular_function
telomere-	Single s	GO:00519	GO:19908	GO:0098505	G-rich strand telomeric DNA binding;GO:001
transcri	Transcri	GO:00710	GO:00343	GO:0070300	phosphatidic acid binding;GO:0003714 trans
endonucl	Endonucl	GO:00062	GO:00335	GO:0017108	5'-flap endonuclease activity;GO:0048256 f
fimbrin	Fimbrin,	GO:00706	GO:00304	GO:0030674	protein binding, bridging;GO:0051015 actin
cleavage	Subunit	GO:00063	GO:00058	GO:00037	(sce03015)mRNA surveillance pathway
cystathi	Putative	GO:00193	GO:00055	GO:00039	(sce00450)Selenocompound metabolism; (sce009
Brr6p	Essentia	GO:00069	GO:00319	GO:0003674	molecular_function
Poc4p	Componen	GO:00511	GO:00056	GO:0003674	molecular_function
Emp47p	Integral	GO:00070	GO:00301	GO:00055	(sce04141)Protein processing in endoplasmic
Csc1p	Calcium	GO:00342	GO:00003	GO:0005227	calcium activated cation channel activity;
lysine p	Lysine p	GO:19038	GO:00321	GO:0015181	arginine transmembrane transporter activit
phosphat	Putative	GO:00159	GO:00056	GO:0008526	phosphatidylinositol transporter activity;
Ibd2p	Componen	GO:00070	GO:00009	GO:0003674	molecular_function
hypothet	Putative protein of unknown function; YMR130W is not an essential gene				
hypothet	Putative	GO:00081	GO:00160	GO:0003674	molecular_function
hypothet	Putative	GO:00081	GO:00160	GO:0003674	molecular_function
Rad33p	Protein	GO:00062	GO:00056	GO:0003674	molecular_function
Srb5p	Subunit	GO:00011	GO:00708	GO:0001135	RNA polymerase II transcription regulator
alpha-1,	Mannosyl	GO:00064	GO:00003	GO:00000	(sce00514)Other types of O-glycan biosynthes
None	None	None	None	None	None
Mrx7p	Protein	GO:00081	GO:00057	GO:0003674	molecular_function
Sec63 co	Non-esse	GO:00312	GO:00312	GO:0008565	protein transporter activity;GO:0005215 tr
Rim21p	pH senso	GO:00014	GO:00057	GO:0003674	molecular_function
lysophos	Phosphol	GO:00066	GO:00003	GO:00046	(sce00564)Glycerophospholipid metabolism
hypothet	Putative	GO:00081	GO:00160	GO:0003674	molecular_function
phosphog	Homolog	GO:00060	GO:00057	GO:00046	(M00002)Glycolysis, core module involving th
hydroxya	Mitochon	GO:00066	GO:00057	GO:0019171	3-hydroxyacyl-[acyl-carrier-protein] dehyd
Nem1-Spo	Probable	GO:19037	GO:00715	GO:0004721	phosphoprotein phosphatase activity;GO:001
translat	Subunit	GO:00483	GO:00104	GO:0003743	translation initiation factor activity;GO:
translat	Alpha su	GO:00017	GO:00436	GO:00430	(sce04138)Autophagy - yeast; (sce04141)Prote

U4/U6-U5	Componen	GO:00004	GO:0465	GO:00036	(sce03040)Spliceosome
cleavage	Componen	GO:00987	GO:00058	GO:00055	(sce03015)mRNA surveillance pathway
protein	B-type r	GO:00315	GO:00007	GO:00725	(sce03015)mRNA surveillance pathway; (sce041
Sbp1p	Protein	GO:00320	GO:00104	GO:0031370	eukaryotic initiation factor 4G binding;GO
None	None	None	None	None	None
Sdd3p	Putative	GO:00065	GO:00057	GO:0004222	metalloendopeptidase activity;GO:0008237 m
Ras GTPa	GTPase-a	GO:00704	GO:00057	GO:0005096	GTPase activator activity;GO:0030695 GTPas
actin-re	Essentia	GO:00343	GO:00304	GO:00037	(sce04138)Autophagy - yeast
aromatic	High aff	GO:00158	GO:00057	GO:0005300	high-affinity tryptophan transmembrane tra
cytosine	Cytosine	GO:00442	GO:00056	GO:01024	(sce00330)Arginine and proline metabolism; (
GTPase B	GTPase r	GO:00004	GO:00306	GO:00345	(sce03008)Ribosome biogenesis in eukaryotes
DNA-dire	RNA poly	GO:00011	GO:00056	GO:00039	(sce03020)RNA polymerase; (sce00240)Pyrimidi
type 1 p	Regulato	GO:00070	GO:00001	GO:0004865	protein serine/threonine phosphatase inhib
retromer	Endosoma	GO:00450	GO:00309	GO:00085	(sce04144)Endocytosis
Yap3p	Basic le	GO:00063	GO:00056	GO:0043565	sequence-specific DNA binding;GO:0003700 D
Mak32p	Protein	GO:00444	GO:00055	GO:0003674	molecular_function
S-adenos	S-adenos	GO:00192	GO:00056	GO:00088	(sce00270)Cysteine and methionine metabolism
Rec104p	Protein	GO:00421	GO:00007	GO:0003674	molecular_function
GPI-anch	GPI tran	GO:00162	GO:00427	GO:00039	(sce00563)Glycosylphosphatidylinositol (GPI)
uridine	Uridine/	GO:00442	GO:00056	GO:00048	(sce00240)Pyrimidine metabolism; (sce01100)M
peroxiso	Peroxiso	GO:00070	GO:00057	GO:0003674	molecular_function
None	None	None	None	None	None
Mdj1p	Co-chape	GO:00065	GO:00313	GO:00016	(Human)(hsa05203)Viral carcinogenesis
DNA-bind	Basic le	GO:00613	GO:00056	GO:0043565	sequence-specific DNA binding;GO:0003700 D
hypothet	Putative	GO:00081	GO:00160	GO:0003674	molecular_function
hypothet	Putative	GO:00081	GO:00055	GO:0003674	molecular_function
Fig1p	Integral	GO:00322	GO:00433	GO:0003674	molecular_function
bifuncti	Bifuncti	GO:00193	GO:00055	GO:00431	(sce00860)Porphyrin and chlorophyll metaboli
Ypt35p	Endosoma	GO:00081	GO:00100	GO:0032266	phosphatidylinositol-3-phosphate binding;G
putative	Putative	GO:00551	GO:00056	GO:00525	(sce00650)Butanoate metabolism
Emi1p	Non-esse	GO:00304	GO:00055	GO:0003674	molecular_function
inositol-	Inositol-	GO:00060	GO:00057	GO:00045	(sce00562)Inositol phosphate metabolism; (sc
hypothet	Putative	GO:00081	GO:00055	GO:0003674	molecular_function
Nop19p	Ribosome	GO:00004	GO:00306	GO:0003674	molecular_function
Far10p	Protein	GO:00003	GO:00057	GO:0003674	molecular_function
Ngr1p	RNA bind	GO:00611	GO:00104	GO:1990446	U1 snRNP binding;GO:0070990 snRNP binding;
proteaso	Alpha 4	GO:00104	GO:00345	GO:00042	(sce03050)Proteasome
mitochon	Mitochon	GO:00325	GO:00057	GO:0003735	structural constituent of ribosome;GO:0005
putative	Phosphol	GO:00550	GO:00058	GO:0004622	lysophospholipase activity;GO:0004620 phos
Bio5p	Putative	GO:00091	GO:00057	GO:0022857	transmembrane transporter activity;GO:0005
guanine	Guanine	GO:00068	GO:00058	GO:0005085	guanyl-nucleotide exchange factor activity
hypothet	Putative protein of unknown function; the authentic, non-tagged protein is detec				
chaperon	Subunit	GO:00064	GO:00058	GO:0051082	unfolded protein binding;GO:0005524 ATP bi
Ylh47p	Mitochon	GO:00512	GO:00057	GO:0043022	ribosome binding;GO:0043021 ribonucleoprot
serine/t	Palmitoy	GO:00102	GO:00319	GO:00046	(sce04392)Hippo signaling pathway -multiple
hypothet	Cell wal	GO:00081	GO:00092	GO:0003674	molecular_function
gamma-tu	Gamma-tu	GO:20007	GO:00009	GO:0005200	structural constituent of cytoskeleton;GO:
transcri	TFIIE la	GO:00011	GO:00056	GO:00010	(sce03022)Basal transcription factors
Spt20p	Subunit	GO:00364	GO:00466	GO:0003712	transcription coregulator activity;GO:0140
mitochon	Mitochon	GO:00325	GO:00057	GO:0003735	structural constituent of ribosome;GO:0005
Git1p	Plasma m	GO:00014	GO:00003	GO:0001406	glycerophosphodiester transmembrane transp
Ggc1p	Mitochon	GO:19037	GO:00057	GO:0001409	guanine nucleotide transmembrane transport
Mbf1p	Transcri	GO:00459	GO:00057	GO:0003713	transcription coactivator activity;GO:0003
oxystero	Member o	GO:00347	GO:00325	GO:0008142	oxysterol binding;GO:0043325 phosphatidyli

tRNA-5-ta tRNA-spec GO:19907 GO:00057 GO:0061708 tRNA-5-taurinomethyluridine 2-sulfurtransf
FG-nucle FG-nucle GO:00000 GO:00446 GO:00081 (sce03013)RNA transport
Tbs1p Protein GO:00063 GO:00319 GO:0000981 DNA-binding transcription factor activity,
hypotheti Dubious GO:00081 GO:00160 GO:0003674 molecular_function
cytochrom Subunit GO:00061 GO:00057 GO:00504 (sce00190)Oxidative phosphorylation; (sce011
hypotheti Putative protein of unknown function; null mutant is viable
Anr2p Protein GO:00081 GO:00058 GO:0003674 molecular_function
Hat2p Subunit GO:00063 GO:00001 GO:0004402 histone acetyltransferase activity;GO:0061
chaperon Subunit GO:00064 GO:00058 GO:0051082 unfolded protein binding;GO:0005524 ATP bi
rRNA-bin Protein GO:00004 GO:00306 GO:00701 (sce03008)Ribosome biogenesis in eukaryotes
Scj1p One of s GO:00304 GO:00057 GO:00510 (sce04141)Protein processing in endoplasmic
Cdc40p Pre-mRNA GO:00003 GO:00710 GO:00003 (sce03040)Spliceosome
translat Translat GO:00021 GO:00184 GO:00037 (sce03015)mRNA surveillance pathway
putative Essentia GO:00004 GO:00306 GO:0004004 ATP-dependent RNA helicase activity;GO:000
hypotheti Dubious GO:00081 GO:00055 GO:0003674 molecular_function
transcri Componen GO:00611 GO:00336 GO:00044 (sce04139)Mitophagy - yeast
mRNA spl Conserve GO:00002 GO:00056 GO:00364 (sce03040)Spliceosome
hypotheti Putative GO:00081 GO:00160 GO:0003674 molecular_function
ribosoma Ribosoma GO:00021 GO:00226 GO:00037 (sce03010)Ribosome
Vps64p Protein GO:00003 GO:00057 GO:0003674 molecular_function
SUMO lig SUMO E3 GO:19906 GO:00059 GO:00197 (sce04120)Ubiquitin mediated proteolysis
hypotheti Putative GO:00081 GO:00055 GO:0003674 molecular_function
Spt2p Protein GO:00063 GO:00058 GO:0000217 DNA secondary structure binding;GO:0003677
phosphat Phosphol GO:00014 GO:00007 GO:00044 (sce04933)AGE-RAGE signaling pathway in diab
TFIIH co Componen GO:00062 GO:00056 GO:00009 (sce03022)Basal transcription factors; (sce0
dihydrof Dihydrof GO:00464 GO:00057 GO:00041 (sce)Tetrahydrofolate biosynthesis, GTP => T
glycogen Glycogen GO:00059 GO:00057 GO:00084 (sce00500)Starch and sucrose metabolism; (sc
snoRNA-b Nucleola GO:00004 GO:00343 GO:00305 (sce03008)Ribosome biogenesis in eukaryotes
Cin1p Tubulin GO:00070 GO:00055 GO:0048487 beta-tubulin binding;GO:0005096 GTPase act
Lcl2p Putative GO:00304 GO:00003 GO:0003674 molecular_function
Sts1p Protein GO:19909 GO:00319 GO:1990919 nuclear membrane proteasome anchor;GO:0043
adenylat Mitochon GO:00469 GO:00057 GO:00468 (sce)Adenine ribonucleotide biosynthesis, IM
medium-c Medium c GO:00016 GO:00057 GO:01023 (sce)beta-Oxidation, acyl-CoA synthesis; (M0
Atg41p Protein GO:00322 GO:00340 GO:0003674 molecular_function
Ras fami GTP-bind GO:00315 GO:00058 GO:00039 (sce04111)Cell cycle - yeast
pyridoxa Protein GO:00428 GO:00056 GO:0004733 pyridoxamine-phosphate oxidase activity;GO
mannose- ER membr GO:00065 GO:00057 GO:00513 (sce)GPI-anchor biosynthesis, core oligosacc
hypotheti Protein GO:00081 GO:00055 GO:0003674 molecular_function
6-phosph 6-phosph GO:00090 GO:00056 GO:00170 (M00006)Pentose phosphate pathway, oxidative
Fyv7p Essentia GO:00004 GO:00057 GO:0003674 molecular_function
phosphat Phosphat GO:19013 GO:00001 GO:0008526 phosphatidylinositol transporter activity;
Rho fami Non-esse GO:00903 GO:00001 GO:0003924 GTPase activity;GO:0005525 GTP binding;GO:
ribosoma Ribosoma GO:00021 GO:00226 GO:00037 (sce03010)Ribosome
hypotheti Protein GO:00081 GO:00055 GO:0003674 molecular_function
kynureni Kynureni GO:00343 GO:00057 GO:00045 (sce00380)Tryptophan metabolism; (sce01100)M
Rho fami Guanine GO:00435 GO:01201 GO:00050 (sce04011)MAPK signaling pathway - yeast
ATP-bind Vacuolar GO:00157 GO:00003 GO:00154 (Human)(hsa01523)Antifolate resistance; (hsa
Mdm36p Mitochon GO:00483 GO:00057 GO:0003674 molecular_function
Cos10p Endosoma GO:00433 GO:00056 GO:0003674 molecular_function
phospho Protein GO:00070 GO:00003 GO:0004721 phosphoprotein phosphatase activity;GO:001
putative Lipase w GO:00160 GO:00056 GO:0047372 acylglycerol lipase activity;GO:0016298 li
hypotheti Putative GO:00081 GO:00055 GO:0003674 molecular_function
serine/t Serine/t GO:00459 GO:00098 GO:0004674 protein serine/threonine kinase activity;G
phosphop Negative GO:00159 GO:00715 GO:0004633 phosphopantothenoylcysteine decarboxylase

Fzf1p	Transcrip	GO:00614	GO:00056	GO:0000978 RNA polymerase II proximal promoter sequen
chromatin	Subunit	GO:00458	GO:00056	GO:0031491 nucleosome binding;GO:0042393 histone bind
TRAPP cor	Core comp	GO:00322	GO:19900	GO:0017112 Rab guanyl-nucleotide exchange factor acti
Fcf2p	Nucleolar	GO:00004	GO:00057	GO:0003674 molecular_function
DNA-direc	RNA polym	GO:00427	GO:00056	GO:00039 (sce03020)RNA polymerase; (sce00240)Pyrimidin
AAA famil	AAA-perox	GO:00165	GO:00057	GO:00426 (sce04146)Peroxisome
purine-cy	Putative	GO:00158	GO:00058	GO:0015205 nucleobase transmembrane transporter activ
Coy1p	Golgi mem	GO:00068	GO:00301	GO:0003674 molecular_function
Tlg1p	Essential	GO:00069	GO:00319	GO:00054 (sce04130)SNARE interactions in vesicular tra
dithiol g	Cytoplasm	GO:00988	GO:00057	GO:0004362 glutathione-disulfide reductase activity;G
mitochond	Mitochond	GO:00325	GO:00057	GO:00037 (sce03010)Ribosome
Mmt1p	Putative	GO:00068	GO:00319	GO:0008324 cation transmembrane transporter activity;
SCF ubiqu	F-box pro	GO:00007	GO:00001	GO:00306 (sce04120)Ubiquitin mediated proteolysis; (s
Sdh7p	Mitochond	GO:00345	GO:00057	GO:0003674 molecular_function
chromatin	Component	GO:00062	GO:00056	GO:0003682 chromatin binding;GO:0005488 binding;GO:00
Spc110p	Inner pla	GO:19024	GO:0005823 central plaque of spindle pole body;GO:0005822 inne	
hypotheti	Putative	GO:00081	GO:00055	GO:0003674 molecular_function
palmitoyl	Palmitoyl	GO:00304	GO:00304	GO:0019706 protein-cysteine S-palmitoyltransferase ac
hypotheti	Putative	GO:00081	GO:00003	GO:0003674 molecular_function
Ssp120p	Protein	GO:00081	GO:00003	GO:0005509 calcium ion binding;GO:0046872 metal ion b
Sfb3p	Component	GO:00901	GO:00301	GO:00050 (sce04141)Protein processing in endoplasmic
Hsk3p	Essential	GO:00519	GO:00427	GO:0051010 microtubule plus-end binding;GO:0008017 mi
mitochond	Mitochond	GO:00000	GO:00057	GO:00701 (sce03010)Ribosome
Far7p	Protein	GO:00003	GO:00057	GO:0003674 molecular_function
Y' elemen	Helicase	GO:00007	GO:00057	GO:0003678 DNA helicase activity;GO:0004386 helicase
Zim17p	Protein	GO:00301	GO:00057	GO:0051087 chaperone binding;GO:0008270 zinc ion bind
mannosyl-	Alpha-1,2	GO:00359	GO:00057	GO:00045 (sce)N-glycan precursor trimming; (M00073)N-
Eis1p	Component	GO:00709	GO:00321	GO:0003674 molecular_function
hypotheti	Dubious	GO:00081	GO:00055	GO:0003674 molecular_function
Pex34p	Protein	GO:00070	GO:00057	GO:0003674 molecular_function
bifunctio	Hydroxyme	GO:00092	GO:00058	GO:00503 (sce00730)Thiamine metabolism; (sce01100)Meta
serine-ty	Cell deat	GO:00516	GO:00003	GO:0004185 serine-type carboxypeptidase activity;GO:0
hypotheti	Putative	GO:00345	GO:00057	GO:0003674 molecular_function
Ort1p	Ornithine	GO:19905	GO:00057	GO:0000064 L-ornithine transmembrane transporter acti
protein k	Protein k	GO:00001	GO:00055	GO:00046 (sce04011)MAPK signaling pathway - yeast
Gyl1p	Putative	GO:00171	GO:00059	GO:0005096 GTPase activator activity;GO:0030695 GTPas
hypotheti	Putative	GO:00081	GO:00058	GO:0003674 molecular_function
hypotheti	Putative	GO:00460	GO:00007	GO:0003674 molecular_function
Spp2p	Essential	GO:00327	GO:00056	GO:0001671 ATPase activator activity;GO:0060590 ATPas
putative	Putative	GO:00065	GO:00003	GO:0004177 aminopeptidase activity;GO:0008238 exopept
exosome n	Exosome n	GO:00710	GO:00001	GO:00037 (sce03018)RNA degradation
hypotheti	Putative	GO:00165	GO:00057	GO:0005052 peroxisome matrix targeting signal-1 bindi
Fmp27p	Putative	GO:00081	GO:00057	GO:0003674 molecular_function
Mid1p	N-glycosy	GO:00987	GO:00057	GO:0015275 stretch-activated, cation-selective, calci
DNA-bindi	Transcrip	GO:20008	GO:00007	GO:0003684 damaged DNA binding;GO:0043565 sequence-sp
mitochond	Mitochond	GO:00325	GO:00057	GO:00037 (sce03010)Ribosome
rRNA-proc	Component	GO:00004	GO:00344	GO:00036 (sce03008)Ribosome biogenesis in eukaryotes
Asa1p	Subunit	GO:00063	GO:00702	GO:0003674 molecular_function
Golgi tra	Essential	GO:00003	GO:00171	GO:0003674 molecular_function
Ran GTPas	Ran GTPas	GO:00066	GO:00056	GO:00085 (Human)(hsa05203)Viral carcinogenesis; (hsa0
Mdm10p	Subunit	GO:00450	GO:00328	GO:00036 (sce04139)Mitophagy - yeast
hypotheti	Protein	GO:00081	GO:00003	GO:0003674 molecular_function
Pso2p	Nuclease	GO:00318	GO:00007	GO:0035312 5'-3' exodeoxyribonuclease activity;GO:001
CCR4-NOT	Component	GO:00070	GO:00165	GO:00045 (sce03018)RNA degradation

nucleoti	Nucleoti	GO:00091	GO:00057	GO:0035529 NADH pyrophosphatase activity;GO:0004528 p	
ribosoma	Ribosoma	GO:00064	GO:00226	GO:00037	(sce03010)Ribosome
protein	Protein	GO:00903	GO:19906	GO:0019888 protein phosphatase regulator activity;GO:	
Y' elemen	DNA heli	GO:00325	GO:00055	GO:0003678 DNA helicase activity;GO:0004386 helicase	
Spo12p	Nucleola	GO:00315	GO:00057	GO:00036	(sce04111)Cell cycle - yeast; (sce04113)Meio
SCF ubiq	Putative	GO:00714	GO:00190	GO:0030674 protein binding, bridging;GO:0004842 ubiqu	
Pbn1p	Componen	GO:00065	GO:19905	GO:00000	(sce00563)Glycosylphosphatidylinositol (GPI)
Ecm19p	Protein	GO:00715	GO:00319	GO:0003674 molecular_function	
phosphop	Catalyti	GO:20007	GO:00001	GO:00047	(sce04136)Autophagy - other; (sce03015)mRNA
17-beta-	Putative	GO:00092	GO:00057	GO:0046557 glucan endo-1,6-beta-glucosidase activity;	
MCM DNA	Essentia	GO:00062	GO:00711	GO:19901	(sce03030)DNA replication; (sce04111)Cell cy
Nab3p	RNA-bind	GO:00710	GO:00356	GO:0001068 transcription regulatory region RNA bindin	
None	None	None	None	None	None
alpha-1,	Alpha-1,	GO:19043	GO:00443	GO:0004571 mannosyl-oligosaccharide 1,2-alpha-mannosi	
putative	Cytoplas	GO:00000	GO:00056	GO:0004674 protein serine/threonine kinase activity;G	
hypothet	Putative	GO:00081	GO:00160	GO:0003674 molecular_function	
None	None	None	None	None	None
dolichyl-	Beta sub	GO:00182	GO:00082	GO:00045	(M00072)N-glycosylation by oligosaccharyltra
Upc2p	Sterol r	GO:00359	GO:00484	GO:0000978 RNA polymerase II proximal promoter sequen	
hypothet	Putative	GO:00081	GO:00160	GO:0003674 molecular_function	
Fmp23p	Putative	GO:00081	GO:00057	GO:0003674 molecular_function	
H3 histo	Histone	GO:00970	GO:00007	GO:0032931 histone acetyltransferase activity (H3-K56	
Rrt14p	Putative	GO:00081	GO:00057	GO:0003674 molecular_function	
hypothet	Putative	GO:00081	GO:00055	GO:0003674 molecular_function	
Rrt13p	Putative	GO:00081	GO:00055	GO:0003674 molecular_function	
ESCRT-0	Endosoma	GO:19046	GO:00335	GO:00322	(sce04145)Phagosome; (sce04144)Endocytosis
Tbf1p	Telobox-	GO:00010	GO:00007	GO:0043035 chromatin insulator sequence binding;GO:00	
inositol	Inositol	GO:00109	GO:00057	GO:00332	(sce04070)Phosphatidylinositol signaling sys
Lrg1p	GTPase-a	GO:00903	GO:00059	GO:0005096 GTPase activator activity;GO:0030695 GTPas	
Yar1p	Ankyrin-	GO:00000	GO:00056	GO:0051082 unfolded protein binding;GO:0005515 protei	
homoseri	Homoseri	GO:00090	GO:00056	GO:00044	(M00018)Threonine biosynthesis, aspartate =>
Sil1p	Nucleoti	GO:00066	GO:00057	GO:00007	(sce04141)Protein processing in endoplasmic
hexose t	Putative	GO:00157	GO:00058	GO:00155	(sce04113)Meiosis - yeast
formin-b	Protein	GO:00903	GO:00001	GO:0008092 cytoskeletal protein binding;GO:0005515 pr	
Med8p	Subunit	GO:00001	GO:00708	GO:0017025 TBP-class protein binding;GO:0000978 RNA p	
mitochon	Mitochon	GO:00422	GO:00057	GO:0003735 structural constituent of ribosome;GO:0005	
SNARE-bi	Essentia	GO:00355	GO:00056	GO:0000149 SNARE binding;GO:0042803 protein homodimer	
Hop2p	Meiosis-	GO:00071	GO:00007	GO:0003690 double-stranded DNA binding;GO:0003677 DNA	
Sip4p	C6 zinc	GO:00614	GO:00056	GO:0000978 RNA polymerase II proximal promoter sequen	
Arr1p	Transcri	GO:00613	GO:00056	GO:0000978 RNA polymerase II proximal promoter sequen	
hypothet	Putative zinc-cluster protein of unknown function				
homoacon	Homoacon	GO:00198	GO:00057	GO:00044	(sce)Lysine biosynthesis, 2-oxoglutarate =>
None	None	None	None	None	None
Pex25p	Peripher	GO:00165	GO:00057	GO:0003674 molecular_function	
Vps41p	Subunit	GO:00328	GO:00308	GO:00171	(sce04138)Autophagy - yeast
peptide	Catalyti	GO:00171	GO:00314	GO:0004596 peptide alpha-N-acetyltransferase activity	
chaperon	Subunit	GO:00064	GO:00058	GO:0051082 unfolded protein binding;GO:0005524 ATP bi	
mRNA spl	Essentia	GO:00003	GO:00710	GO:0004004 ATP-dependent RNA helicase activity;GO:000	
Atg26p	UDP-gluc	GO:00321	GO:00160	GO:0016906 sterol 3-beta-glucosyltransferase activity	
Dcp1p	Subunit	GO:00310	GO:00987	GO:00080	(sce03018)RNA degradation
Mxr2p	Methioni	GO:00300	GO:00057	GO:0033743 peptide-methionine (R)-S-oxide reductase a	
hypothet	Putative	GO:00081	GO:00055	GO:0003674 molecular_function	
Aim20p	Protein	GO:00070	GO:00003	GO:0003674 molecular_function	
Sko1p	Basic le	GO:00613	GO:00058	GO:00510	(sce04011)MAPK signaling pathway - yeast

Pop3p	Subunit	GO:00349	GO:00056	GO:00045	(sce03008)Ribosome biogenesis in eukaryotes;
Ste5p	Pheromone	GO:00007	GO:00708	GO:00050	(sce04011)MAPK signaling pathway - yeast
F1F0 ATP	Alpha sul	GO:00159	GO:00057	GO:00469	(sce00190)Oxidative phosphorylation; (sce011
hypotheti	Putative protein of unknown function; green fluorescent protein (GFP)-fusion pro				
rRNA-bin	Protein	GO:00000	GO:00306	(Human)GO:0003723 RNA binding;GO:0003676 nucleic acid	
Mrl1p	Membrane	GO:00070	GO:00100	GO:00050	(sce04145)Phagosome
ATP-depe	Cytoplasm	GO:00169	GO:00446	GO:0000822 inositol hexakisphosphate binding;GO:00040	
hypotheti	Putative	GO:00550	GO:00003	GO:0005524 ATP binding;GO:0032559 adenyl ribonucleoti	
hypotheti	Putative	GO:00081	GO:00055	GO:0003674 molecular_function	
Pet20p	Mitochon	GO:00090	GO:00057	GO:0003674 molecular_function	
Yhc1p	Component	GO:00003	GO:00056	GO:00306	(sce03040)Spliceosome
spermine	Polyamine	GO:19037	GO:00003	GO:0000297 spermine transmembrane transporter activit	
protein	Mitochon	GO:00450	GO:00427	GO:0008565 protein transporter activity;GO:0005215 tr	
DNA-(apu	Major ap	GO:00062	GO:00057	GO:00083	(sce03410)Base excision repair
Shr5p	Palmitoy	GO:00182	GO:00424	GO:0019706 protein-cysteine S-palmitoyltransferase ac	
asparagi	Cytosoli	GO:00064	GO:00058	GO:00048	(sce00970)Aminoacyl-tRNA biosynthesis
osmosens	Transmem	GO:00014	GO:00446	GO:00050	(sce04011)MAPK signaling pathway - yeast
Rcr1p	Protein	GO:00060	GO:00301	GO:0003674 molecular_function	
mitochon	Mitochon	GO:00000	GO:00057	GO:0003735 structural constituent of ribosome;GO:0005	
isocitra	Mitochon	GO:00065	GO:00426	GO:00044	(M00010)Citrate cycle, first carbon oxidatio
None	None	None	None	None	None
putative	Putative	GO:00068	GO:00343	GO:0016740 transferase activity;GO:0003824 catalytic	
cysteine-	Cysteiny	GO:00064	GO:00058	GO:00048	(sce00970)Aminoacyl-tRNA biosynthesis
hypotheti	Putative protein of unknown function; green fluorescent protein (GFP)-fusion pro				
hypotheti	Putative	GO:00081	GO:00055	GO:0003674 molecular_function	
hypotheti	Putative protein of unknown function; non-essential gene; transcription represse				
hypotheti	Putative	GO:00081	GO:00057	GO:0003674 molecular_function	
ammonium	Ammonium	GO:00724	GO:00058	GO:0008519 ammonium transmembrane transporter activit	
decappin	Catalytic	GO:00310	GO:00987	GO:00500	(sce03018)RNA degradation
Fes1p	Hsp70 (S	GO:00716	GO:00057	GO:0000774 adenyl-nucleotide exchange factor activity	
protein	Protein	GO:00448	GO:00059	GO:00046	(sce04111)Cell cycle - yeast
Nto1p	Subunit	GO:00165	GO:19904	GO:0035064 methylated histone binding;GO:0140034 meth	
tropomyo	Minor is	GO:00071	GO:00001	GO:0003786 actin lateral binding;GO:0051015 actin fil	
E1 ubiqu	Ubiquiti	GO:00165	GO:00056	GO:00048	(sce04120)Ubiquitin mediated proteolysis
histidin	High-aff	GO:00897	GO:00057	GO:0005291 high-affinity L-histidine transmembrane tr	
sterol a	Acyl-CoA	GO:00082	GO:00057	GO:00347	(sce00100)Steroid biosynthesis
None	None	None	None	None	None
putative	Probable	GO:00157	GO:00057	GO:0008374 O-acyltransferase activity;GO:0015293 symp	
carbonyl	Aldehyde	GO:19014	GO:00055	GO:0033721 aldehyde dehydrogenase (NADP+) activity;GO	
Ent5p	Protein	GO:00344	GO:00301	GO:0080025 phosphatidylinositol-3,5-bisphosphate bind	
cullin C	Ubiquiti	GO:00311	GO:00314	GO:00616	(sce04120)Ubiquitin mediated proteolysis
Sys1p	Integral	GO:00430	GO:00301	GO:0003674 molecular_function	
Vps38p	Part of	GO:00354	GO:00342	GO:0000149 SNARE binding;GO:0005515 protein binding;G	
phosphor	Phosphor	GO:00061	GO:00057	GO:00437	(M00048)Inosine monophosphate biosynthesis,
ribonucl	Major mi	GO:00063	GO:00057	GO:00000	(Human)(hsa04210)Apoptosis
Pet18p	Protein	GO:00067	GO:00058	GO:0003674 molecular_function	
Yap7p	Putative	GO:00010	GO:00007	GO:0000978 RNA polymerase II proximal promoter sequen	
GPI-anch	Function	GO:00065	GO:00315	GO:00045	(sce)GPI-anchor biosynthesis, core oligosacc
uracil-D	Uracil-D	GO:00975	GO:00057	GO:00048	(sce03410)Base excision repair
putative	Cell wal	GO:00715	GO:00092	GO:0042973 glucan endo-1,3-beta-D-glucosidase activit	
Rtf1p	Subunit	GO:20011	GO:00165	GO:1990269 RNA polymerase II C-terminal domain phosph	
replicat	Subunit	GO:00062	GO:00056	GO:00036	(sce03430)Mismatch repair; (sce03030)DNA rep
Kch1p	Potassiu	GO:00718	GO:00059	GO:0015079 potassium ion transmembrane transporter ac	
ATP-depe	DEAD-box	GO:00004	GO:00057	GO:0033680 ATP-dependent DNA/RNA helicase activity;GO	

None	None	None	None	None	None
Phm7p	Protein (	GO:00342	GO:00003	GO:0005227 calcium activated cation channel activity;	
ribosoma	Ribosoma	GO:00021	GO:00306	GO:00037	(sce03010)Ribosome
translat	Translat	GO:00422	GO:00436	GO:00050	(sce03013)RNA transport
Tim18p	Componen	GO:00450	GO:00427	GO:0008177 succinate dehydrogenase (ubiquinone) activ	
Npr3p	Subunit (	GO:19038	GO:00970	GO:0003674 molecular_function	
CCR4-NOT	RNase of	GO:00439	GO:00300	GO:00045	(sce03018)RNA degradation
Fe-S clu	Essentia	GO:00020	GO:19045	GO:0051539 4 iron, 4 sulfur cluster binding;GO:00515	
cytochro	Subunit	GO:00061	GO:00057	GO:00041	(sce00190)Oxidative phosphorylation; (sce011
Taf7p	TFIID su	GO:00511	GO:00056	GO:00162	(sce03022)Basal transcription factors
snoRNA-b	Protein	GO:00311	GO:00314	GO:00345	(sce03008)Ribosome biogenesis in eukaryotes
DNA-dire	RNA polym	GO:00319	GO:00056	GO:00039	(sce03020)RNA polymerase; (sce00240)Pyrimidi
None	None	None	None	None	None
Nma111p	Serine p	GO:01201	GO:00056	GO:0004252 serine-type endopeptidase activity;GO:0008	
adenine	Adenine	GO:00061	GO:00056	GO:00000	(sce00230)Purine metabolism; (sce01100)Metab
type 1 s	Type 1 S	GO:19035	GO:00014	GO:00047	(sce03015)mRNA surveillance pathway; (sce041
Cmr2p	Putative	GO:00081	GO:00059	GO:0003824 catalytic activity;GO:0003674 molecular_fu	
Ssp1p	Protein	GO:00321	GO:00700	GO:00036	(sce04113)Meiosis - yeast
None	None	None	None	None	None
Hur1p	Protein (	GO:00162	GO:00160	GO:0003674 molecular_function	
Erp5p	Protein	GO:00070	GO:00301	GO:0003674 molecular_function	
hypothet	Putative	GO:00066	GO:00057	GO:0008374 O-acyltransferase activity;GO:0016747 tran	
Dos2p	Protein (	GO:00081	GO:00057	GO:0003674 molecular_function	
None	None	None	None	None	None
Ist1p	Protein	GO:00325	GO:00057	GO:00036	(sce04144)Endocytosis
hypothet	Putative	GO:00081	GO:00055	GO:0003674 molecular_function	
hexose t	Putative	GO:19023	GO:00058	GO:00155	(sce04113)Meiosis - yeast
Emc6p	Member o	GO:00349	GO:00725	GO:0003674 molecular_function	
Nce101p	Protein (	GO:00093	GO:00058	GO:0003674 molecular_function	
rDNA-bin	Protein	GO:00063	GO:00057	GO:0001179 RNA polymerase I transcription factor bind	
hypothet	Putative	GO:00081	GO:00057	GO:0003674 molecular_function	
protein	Regulato	GO:0043666 regula	GO:0019888 protein phosphatase regulator activity;GO:		
Med1p	Subunit (	GO:00001	GO:00708	GO:0003712 transcription coregulator activity;GO:0140	
None	None	None	None	None	None
U4/U6-U5	Splicing	GO:00342	GO:00465	GO:00306	(sce03040)Spliceosome
arginine	Arginine	GO:19038	GO:00057	GO:0015181 arginine transmembrane transporter activit	
serine C-	Componen	GO:00301	GO:00353	GO:00047	(sce00600)Sphingolipid metabolism; (sce04138)
Pog1p	Nuclear (	GO:00003	GO:00007	GO:0000978 RNA polymerase II proximal promoter sequen	
autophag	Transmem	GO:00617	GO:00619	GO:00036	(sce04136)Autophagy - other; (sce04138)Autop
dynein l	Dynein l	GO:00304	GO:00058	GO:0003774 motor activity;GO:0017111 nucleoside-triph	
translat	eIF3j co	GO:00004	GO:00104	GO:00037	(sce03013)RNA transport
hypothet	Putative	GO:00081	GO:00057	GO:0003674 molecular_function	
hypothet	Putative	GO:00081	GO:00055	GO:0003674 molecular_function	
4-amino-	Protein	GO:00092	GO:00055	GO:00036	(sce00730)Thiamine metabolism; (sce01100)Met
None	None	None	None	None	None
Rtt106p	Histone (	GO:00708	GO:00056	GO:0042393 histone binding;GO:0003690 double-stranded	
hypothet	Eukaryot	GO:00064	GO:00226	GO:0043022 ribosome binding;GO:0003743 translation in	
hypothet	Putative	GO:00081	GO:00056	GO:0003674 molecular_function	
hypothet	Putative	GO:00081	GO:00055	GO:0003674 molecular_function	
hypothet	Putative	GO:00081	GO:00057	GO:0003674 molecular_function	
Rsc8p	Componen	GO:00063	GO:00165	GO:0015616 DNA translocase activity;GO:0008094 DNA-de	
Vik1p	Protein	GO:00070	GO:00058	GO:0008017 microtubule binding;GO:0015631 tubulin bin	
hypothet	Protein (	GO:00454	GO:00059	GO:0015035 protein disulfide oxidoreductase activity;	
Far3p	Protein (	GO:00003	GO:00001	GO:0003674 molecular_function	

Mam3p	Protein	GO:00300	GO:00003	GO:0003674 molecular_function	
None	None	None	None	None	None
hypotheti	Predicte	GO:00081	GO:00059	GO:0003674 molecular_function	
hypotheti	Putative	GO:00709	GO:00057	GO:0043022 ribosome binding;GO:0043021 ribonucleoprot	
dolichyl-	Dolichol-	GO:00064	GO:00301	GO:00529	(sce)N-glycan precursor biosynthesis; (M0005
putative	Glycosyl	GO:00000	GO:00003	GO:0000026 alpha-1,2-mannosyltransferase activity;GO:	
polynucle	RNA 5'-t	GO:00985	GO:00315	GO:0004651 polynucleotide 5'-phosphatase activity;GO:	
Chs5p	Componen	GO:00060	GO:00340	GO:0031267 small GTPase binding;GO:0051020 GTPase bin	
serine/t	Serine/t	GO:00602	GO:00321	GO:00046	(sce04011)MAPK signaling pathway - yeast
vacuolar	Regulato	GO:00421	GO:00332	GO:0005516 calmodulin binding;GO:0005515 protein bind	
phosphoi	Phosphoi	GO:00344	GO:00707	GO:00800	(sce04136)Autophagy - other; (sce04138)Autop
E1 ubiqu	Subunit	GO:00169	GO:00315	GO:00199	(sce04120)Ubiquitin mediated proteolysis
ribosome	GTPase w	GO:00709	GO:19905	GO:00037	(sce03015)mRNA surveillance pathway
rRNA-bin	Essentia	GO:19026	GO:00306	GO:0008097 5S rRNA binding;GO:0019843 rRNA binding;GO	
Snx3p	Sorting	GO:00344	GO:00319	GO:00322	(sce04144)Endocytosis
Ast2p	Lipid ra	GO:00066	GO:00057	GO:0016491 oxidoreductase activity;GO:0003824 catalyt	
ribosoma	Ribosoma	GO:00004	GO:00306	GO:00037	(sce03010)Ribosome
hypotheti	Putative protein of unknown function; identified based on homology to the filame				
Rtn1p	Reticulo	GO:00717	GO:00325	GO:0003674 molecular_function	
Uaf30p	Subunit	GO:00427	GO:00005	GO:0001165 RNA polymerase I upstream control element	
hypotheti	Putative	GO:00081	GO:00055	GO:0003674 molecular_function	
ribosoma	Ribosoma	GO:00021	GO:00226	GO:00037	(sce03010)Ribosome
Stb1p	Protein	GO:19004	GO:00309	GO:0008134 transcription factor binding;GO:0005515 pr	
None	None	None	None	None	None
NEDD8 fa	Ubiquiti	GO:00451	GO:00056	GO:0031386 protein tag;GO:0031625 ubiquitin protein l	
Ber1p	Protein	GO:00076	GO:00058	GO:0003674 molecular_function	
mRNA-bin	Nucleola	GO:00004	GO:00306	GO:00037	(sce03008)Ribosome biogenesis in eukaryotes
beta-tub	Beta-tub	GO:00304	GO:00058	GO:00052	(sce04145)Phagosome
isoamyl	Isoamyl	GO:00060	GO:00055	GO:0016788 hydrolase activity, acting on ester bonds;	
1-phosph	Phosphat	GO:00313	GO:00058	GO:00163	(sce00562)Inositol phosphate metabolism; (sc
glycerol-	NAD-depe	GO:00069	GO:00093	GO:00479	(sce00564)Glycerophospholipid metabolism; (s
Est2p	Reverse	GO:00013	GO:00003	GO:0003721 telomerase RNA reverse transcriptase activ	
F1F0 ATP	Delta su	GO:00159	GO:00057	GO:00469	(sce00190)Oxidative phosphorylation; (sce011
Swt21p	Protein	GO:00003	GO:00305	GO:0003674 molecular_function	
hypotheti	Protein	GO:00081	GO:00003	GO:0003674 molecular_function	
protein	Phosphor	GO:00715	GO:00715	GO:0043495 protein membrane anchor;GO:0005515 protein	
Utp18p	Small-su	GO:00004	GO:00343	GO:00036	(sce03008)Ribosome biogenesis in eukaryotes
SCF ubiq	Evolutio	GO:00070	GO:00315	GO:00616	(sce04120)Ubiquitin mediated proteolysis; (s
mRNA spl	Core Sm	GO:00003	GO:00056	GO:00037	(sce03040)Spliceosome
bifuncti	Na+/H+ a	GO:00070	GO:00319	GO:0015386 potassium:proton antiporter activity;GO:00	
Ran GTPa	Conserve	GO:00066	GO:0005634 nucleus;GO:0043231 intracellular membrane-bounded o		
Hpr1p	Subunit	GO:20012	GO:00004	GO:0032947 protein-containing complex scaffold activi	
Rsc4p	Componen	GO:00063	GO:00165	GO:0015616 DNA translocase activity;GO:0070577 lysine	
imidazol	Imidazol	GO:0000105 histid	GO:00001	(sce)Histidine biosynthesis, PRPP => histidi	
Mrf1p	Mitochon	GO:00701	GO:00057	GO:0016149 translation release factor activity, codon	
tRNA (gu	tRNA met	GO:00304	GO:00056	GO:0052905 tRNA (guanine(9)-N(1))-methyltransferase a	
Bop2p	Protein	GO:00081	GO:00055	GO:0003674 molecular_function	
hypotheti	Helicase-	GO:00081	GO:00057	GO:0005524 ATP binding;GO:0032559 adenyl ribonucleoti	
phosphat	Integral	GO:00423	GO:00715	GO:0033149 FFAT motif binding;GO:0035091 phosphatidyl	
Sec39p	Componen	GO:00325	GO:00709	GO:0003674 molecular_function	
putative	Putative	GO:00193	GO:00055	GO:0019150 D-ribulokinase activity;GO:0019200 carbohy	
Gic2p	Redundan	GO:00903	GO:00059	GO:0005546 phosphatidylinositol-4,5-bisphosphate bind	
Ctr9p	Componen	GO:20012	GO:00165	GO:1990269 RNA polymerase II C-terminal domain phosph	
dolichyl-	UDP-gluc	GO:00064	GO:00057	GO:00045	(sce)N-glycan precursor biosynthesis; (M0005

snoRNA-bi	Protein a	GO:00169	GO:00306	GO:0030515 snoRNA binding;GO:0003723 RNA binding;GO:0	
bifuncti	Deoxyuri	GO:00358	GO:00056	GO:00358	(sce00240)Pyrimidine metabolism; (sce01100)M
Sld7p	Protein v	GO:00301	GO:00312	GO:0003674 molecular_function	
oxystero	Oxystero	GO:00347	GO:00325	GO:0008142 oxysterol binding;GO:0015248 sterol transp	
formin BN	Formin; p	GO:00510	GO:00001	GO:00055	(sce04011)MAPK signaling pathway - yeast
putative	Protein v	GO:00068	GO:00059	GO:0019905 syntaxin binding;GO:0017137 Rab GTPase bin	
hypotheti	Putative	GO:00081	GO:00055	GO:0003674 molecular_function	
spermine	Spermine	GO:00065	GO:00057	GO:00167	(sce00410)beta-Alanine metabolism; (sce00330)
Mic19p	Componen	GO:00424	GO:00616	GO:0003674 molecular_function	
Ssh4p	Specific	GO:00433	GO:00003	GO:0003674 molecular_function	
ATP-depen	Nucleola	GO:00004	GO:00970	GO:00040	(sce03015)mRNA surveillance pathway; (sce030
None	None	None	None	None	None
Ioc4p	Member o	GO:00070	GO:00364	GO:0016887 ATPase activity;GO:0017111 nucleoside-trip	
Fth1p	Putative	GO:00347	GO:00335	GO:0015093 ferrous iron transmembrane transporter act	
Fyv12p	Protein	GO:00081	GO:00160	GO:0003674 molecular_function	
ribosoma	Ribosoma	GO:00004	GO:00306	GO:00037	(sce03010)Ribosome
hexose tr	Putative	GO:00157	GO:00003	GO:00053	(sce04113)Meiosis - yeast
co-chaper	Cochaper	GO:00513	GO:00001	GO:0051087 chaperone binding;GO:0030674 protein bindi	
putative	Putative	GO:00065	GO:00057	GO:0046872 metal ion binding;GO:0016787 hydrolase act	
ubiquiti	Ubiquiti	GO:19046	GO:00319	GO:00048	(sce04144)Endocytosis
Aim18p	Protein	GO:00081	GO:00057	GO:0016872 intramolecular lyase activity;GO:0016853 i	
trifuncti	Alpha su	GO:00427	GO:00058	GO:00043	(sce)Fatty acid biosynthesis, elongation; (M
cyclin-de	CDK inhi	GO:00007	GO:01201	GO:00048	(sce04011)MAPK signaling pathway - yeast; (s
Snf5p	Subunit	GO:20002	GO:00165	GO:0015616 DNA translocase activity;GO:0001102 RNA po	
aspartate	Mitochon	GO:00701	GO:00057	GO:00048	(sce00970)Aminoacyl-tRNA biosynthesis
Coa3p	Mitochon	GO:00701	GO:00313	GO:0003674 molecular_function	
hypotheti	Putative	GO:00081	GO:00056	GO:0003674 molecular_function	
Mmo1p	Protein	GO:00081	GO:00057	GO:0003674 molecular_function	
Roy1p	GTPase in	GO:00066	GO:00306	GO:0005095 GTPase inhibitor activity;GO:0030695 GTPas	
Cgi121p	Componen	GO:00007	GO:00004	GO:0003674 molecular_function	
FG-nucle	FG-nucle	GO:00000	GO:00446	GO:00170	(sce03013)RNA transport
Vcx1p	Vacuolar	GO:00068	GO:00003	GO:0015369 calcium:proton antiporter activity;GO:0051	
Pho80p	Cyclin;	GO:00162	GO:19908	GO:00165	(sce04111)Cell cycle - yeast
ATP-bindi	ATP-bindi	GO:00007	GO:00323	GO:00004	(sce03430)Mismatch repair
ubiquiti	Ubiquiti	GO:00304	GO:00001	GO:00344	(sce04120)Ubiquitin mediated proteolysis; (s
Sbe22p	Protein	GO:00315	GO:00057	GO:0003674 molecular_function	
RNA-bindi	Subunit	GO:00349	GO:00056	GO:00045	(sce03008)Ribosome biogenesis in eukaryotes;
forkhead	Forkhead	GO:00900	GO:00058	GO:0019237 centromeric DNA binding;GO:0003688 DNA rep	
ribosoma	Ribosoma	GO:00000	GO:00226	GO:00701	(sce03010)Ribosome
hypotheti	Putative	GO:00068	GO:00003	GO:0003674 molecular_function	
Cos8p	Endosoma	GO:00433	GO:00003	GO:0003674 molecular_function	
Ssz1p	Hsp70 pr	GO:00510	GO:00058	GO:0044183 protein binding involved in protein foldin	
Tec1p	Transcri	GO:00105	GO:19905	GO:00011	(sce04392)Hippo signaling pathway -multiple
O-phosph	3-phosph	GO:00065	GO:00057	GO:00046	(M00020)Serine biosynthesis, glycerate-3P =>
Vfa1p	Protein	GO:00327	GO:00057	GO:0001671 ATPase activator activity;GO:0060590 ATPas	
Mch4p	Protein v	GO:00157	GO:00003	GO:0008028 monocarboxylic acid transmembrane transpor	
anaphase	Subunit	GO:00300	GO:00056	GO:00303	(sce04120)Ubiquitin mediated proteolysis; (s
mitochon	Mitochon	GO:00325	GO:00057	GO:00701	(sce03010)Ribosome
hypotheti	Putative	GO:00081	GO:00055	GO:0003674 molecular_function	
Spo23p	Protein	GO:00304	GO:00055	GO:0003674 molecular_function	
DNA-bindi	Protein	GO:19042	GO:00007	GO:00421	(sce03440)Homologous recombination
Shu1p	Componen	GO:00007	GO:00971	GO:0003674 molecular_function	
Pet100p	Chaperon	GO:00336	GO:00313	GO:0051082 unfolded protein binding;GO:0005515 protei	
stress-re	Transcri	GO:00613	GO:00056	GO:0000978 RNA polymerase II proximal promoter sequen	

mannose-	ER membr	GO:00065	GO:00057	GO:00513	(sce00563)Glycosylphosphatidylinositol (GPI)-
hypothet	Protein	GO:00081	GO:00056	GO:0003674	molecular_function
acyl-CoA	Fatty-ac	GO:00335	GO:00057	GO:00039	(sce00592)alpha-Linolenic acid metabolism; (
Tti2p	Subunit	GO:00714	GO:00702	GO:0003674	molecular_function
hypothet	Putative	GO:00081	GO:00055	GO:0003674	molecular_function
coatomer	Essentia	GO:00325	GO:00301	GO:0043130	ubiquitin binding;GO:0032182 ubiquitin-lik
Cac2p	Subunit	GO:00063	GO:00331	GO:0042393	histone binding;GO:0005515 protein binding
Rsc2p	Componen	GO:00709	GO:00165	GO:0015616	DNA translocase activity;GO:0008094 DNA-de
Lnp1p	Lunapark	GO:00617	GO:00717	GO:0046872	metal ion binding;GO:0043169 cation bindin
None	None	None	None	None	None
mitochon	Mitochon	GO:00325	GO:00057	GO:0003735	structural constituent of ribosome;GO:0005
Cwc2p	Member o	GO:00003	GO:00710	GO:0000384	first spliceosomal transesterification act
mitochon	Mitochon	GO:00325	GO:00057	GO:0003735	structural constituent of ribosome;GO:0005
TFIIH co	Subunit	GO:00081	GO:00056	GO:0003674	molecular_function
hypothet	Putative	GO:00081	GO:00055	GO:0003674	molecular_function
Nyv1p	v-SNARE	GO:00421	GO:00003	GO:0005484	SNAP receptor activity;GO:0000149 SNARE bi
CCR4-NOT	Componen	GO:00329	GO:00300	GO:00036	(sce03018)RNA degradation
tRNA (gu	2'-O-rib	GO:00304	GO:00057	GO:0009020	tRNA (guanosine-2'-O-)-methyltransferase a
3-deoxy-	3-deoxy-I	GO:00094	GO:00057	GO:00038	(M00022)Shikimate pathway, phosphoenolpyruva
ribosoma	Protein	GO:00021	GO:00226	GO:00037	(sce03010)Ribosome
aminopho	Trans-go	GO:00007	GO:00708	GO:0004012	phospholipid-translocating ATPase activity
Gvp36p	BAR doma	GO:00309	GO:00301	GO:0003674	molecular_function
pyruvate	Highly c	GO:00068	GO:00313	GO:0050833	pyruvate transmembrane transporter activit
Cu(2+)-t	Cu(+2)-t	GO:00600	GO:00125	GO:00436	(Human)(hsa04978)Mineral absorption; (hsa015
Tcm62p	Protein	GO:00345	GO:00057	GO:0051082	unfolded protein binding;GO:0005524 ATP bi
H(+)-tra	Subunit	GO:00070	GO:00002	GO:00469	(sce04145)Phagosome; (sce00190)Oxidative pho
Hpc2p	Subunit	GO:00063	GO:00004	GO:0031491	nucleosome binding;GO:0003682 chromatin bi
mRNA-bin	RNA bind	GO:00070	GO:00056	GO:0035614	snRNA stem-loop binding;GO:0030619 U1 snRN
Lds2p	Protein	GO:00304	GO:00056	GO:0003674	molecular_function
Vba4p	Protein	GO:19908	GO:00003	GO:0015174	basic amino acid transmembrane transporter
ketoredu	Microsom	GO:00427	GO:00057	GO:01023	(sce)Fatty acid biosynthesis, elongation, en
ribosoma	Ribosoma	GO:00021	GO:00226	GO:00701	(sce03010)Ribosome
Pex7p	Peroxiso	GO:00165	GO:00057	GO:00050	(sce04146)Peroxisome
mRNA spl	Componen	GO:00003	GO:00002	GO:0045131	pre-mRNA branch point binding;GO:0036002 p
None	None	None	None	None	None
Vhr1p	Transcri	GO:00614	GO:00056	GO:0000977	RNA polymerase II regulatory region sequen
Cir1p	Mitochon	GO:00229	GO:00057	GO:0009055	electron transfer activity;GO:0016491 oxid
Fhl1p	Regulato	GO:00106	GO:00325	GO:0001226	RNA polymerase II transcription corepresso
Sfi1p	Centrin	GO:00304	GO:00058	GO:0003674	molecular_function
recombin	Strand e	GO:19904	GO:00007	GO:00001	(sce03440)Homologous recombination
Mus81p	Subunit	GO:00315	GO:00484	GO:00482	(sce03440)Homologous recombination
Gal11p	Subunit	GO:20002	GO:00708	GO:0001095	TFIIE-class transcription factor complex b
None	None	None	None	None	None
Spr6p	Protein	GO:00304	GO:00057	GO:0003674	molecular_function
putative	Protein	GO:00709	GO:00056	GO:0008420	RNA polymerase II CTD heptapeptide repeat
amphiphy	Amphiphy	GO:00609	GO:19905	GO:0008289	lipid binding;GO:0008092 cytoskeletal prot
Asf2p	Anti-sil	GO:00304	GO:00007	GO:0003674	molecular_function
Hug1p	Ribonucl	GO:19051	GO:00056	GO:1990846	ribonucleoside-diphosphate reductase inhib
None	None	None	None	None	None
Dxo1p	mRNA 5'-	GO:00002	GO:00057	GO:0008409	5'-3' exonuclease activity;GO:0004527 exon
phosphat	Lipid ph	GO:00066	GO:00001	GO:0000810	diacylglycerol diphosphate phosphatase act
None	None	None	None	None	None
Mbb1p	Putative	GO:00081	GO:00055	GO:0003674	molecular_function
hypothet	Dubious	GO:00081	GO:00055	GO:0003674	molecular_function

Fis1p	Protein i	GO:00436	GO:00057	GO:00036	(sce04139)Mitophagy - yeast
tyrosine	Phosphoty	GO:00001	GO:00056	GO:00047	(sce04011)MAPK signaling pathway - yeast
putative	Peroxison	GO:00066	GO:00057	GO:0004165	dodecenoyl-CoA delta-isomerase activity;GO
Csi2p	Protein c	GO:00081	GO:00059	GO:0003674	molecular_function
Taf11p	TFIID sub	GO:00511	GO:00056	GO:00162	(sce03022)Basal transcription factors
protein-l	S-adenosy	GO:00180	GO:00057	GO:0016279	protein-lysine N-methyltransferase activit
Arf famil	Soluble (	GO:00344	GO:00058	GO:0003924	GTPase activity;GO:0005525 GTP binding;GO:
exosome r	Exosome r	GO:00710	GO:00001	GO:00037	(sce03018)RNA degradation
DNA-direc	RNA polym	GO:00427	GO:00056	GO:00039	(sce03020)RNA polymerase; (sce00240)Pyrimidi
phosphati	UDP-glyco	GO:00065	GO:00005	GO:00171	(sce)GPI-anchor biosynthesis, core oligosacc
mitochond	Mitochond	GO:00905	GO:00057	GO:0004525	ribonuclease III activity;GO:0032296 doubl
hypotheti	Putative protein of unknown function; not required for growth of cells lacking t				
hypotheti	Protein c	GO:00550	GO:00057	GO:0003674	molecular_function
Cu-bindin	Copper-bi	GO:00068	GO:00057	GO:0008379	thioredoxin peroxidase activity;GO:0051920
isocitrat	Peroxison	GO:00067	GO:19904	GO:00044	(M00010)Citrate cycle, first carbon oxidatio
DNA-bindi	RNA polym	GO:00011	GO:00056	GO:0043565	sequence-specific DNA binding;GO:0003677 D
hypotheti	Putative	GO:00081	GO:00433	GO:0003674	molecular_function
Rlp7p	Nucleolar	GO:00004	GO:00306	GO:00198	(sce03010)Ribosome
Utp30p	Putative	GO:00004	GO:00306	GO:0003723	RNA binding;GO:0003676 nucleic acid bindin
hypotheti	Putative	GO:00081	GO:00055	GO:0003674	molecular_function
Inn1p	Essential	GO:19034	GO:00001	GO:0005543	phospholipid binding;GO:0008289 lipid bind
glutamyl-	Subunit c	GO:00706	GO:00309	GO:0050567	glutaminyl-tRNA synthase (glutamine-hydrol
Bsc6p	Protein c	GO:00550	GO:00301	GO:0003674	molecular_function
Spc29p	Inner pla	GO:00304	GO:00058	GO:0005200	structural constituent of cytoskeleton;GO:
Dop1p	Golgi-loc	GO:00068	GO:00058	GO:0003674	molecular_function
hypotheti	Putative	GO:00081	GO:00055	GO:0003674	molecular_function
pyridoxal	Protein c	GO:00428	GO:00056	GO:0004733	pyridoxamine-phosphate oxidase activity;GO
cyclin-de	Cyclin-li	GO:00004	GO:00165	GO:0000979	RNA polymerase II core promoter sequence-s
DNA-direc	RNA polym	GO:00427	GO:00056	GO:00010	(sce03020)RNA polymerase; (sce00240)Pyrimidi
None	None	None	None	None	None
None	None	None	None	None	None
Vab2p	Subunit c	GO:00070	GO:00310	GO:0003674	molecular_function
Sec20p	Membrane	GO:00482	GO:00312	GO:00054	(sce04130)SNARE interactions in vesicular tr
cleavage	Component	GO:00724	GO:00057	GO:00037	(sce03015)mRNA surveillance pathway
Zn(2+) tr	Vacuolar	GO:00068	GO:00003	GO:00053	(Human)(hsa04978)Mineral absorption
hypotheti	Putative	GO:00081	GO:00055	GO:0003674	molecular_function
hypotheti	Protein c	GO:00081	GO:00057	GO:0003674	molecular_function
SCF ubiqu	F-box rec	GO:00714	GO:00190	GO:0030674	protein binding, bridging;GO:0004842 ubiqu
glyceralc	Glyceralc	GO:00060	GO:00092	GO:00043	(M00002)Glycolysis, core module involving th
Rmd6p	Protein r	GO:00304	GO:00055	GO:0003674	molecular_function
Exo5p	Mitochond	GO:00362	GO:00057	GO:0045145	single-stranded DNA 5'-3' exodeoxyribonucl
mitogen-a	Cytoplasm	GO:00072	GO:19903	GO:00001	(sce04139)Mitophagy - yeast; (sce04011)MAPK
Stb6p	Protein t	GO:00081	GO:00708	GO:0003674	molecular_function
Efg1p	Essential	GO:00003	GO:00306	GO:0003674	molecular_function
Bud2p	GTPase ac	GO:00315	GO:00056	GO:0005096	GTPase activator activity;GO:0030695 GTPas
hypotheti	Putative	GO:00081	GO:00160	GO:0003674	molecular_function
Hsp70 fam	Cytoplasm	GO:00000	GO:00104	GO:00441	(sce04213)Longevity regulating pathway - mul
adenylyl-	Adenylyls	GO:00193	GO:00057	GO:00040	(M00176)Assimilatory sulfate reduction, sulf
protein-l	S-adenosy	GO:00180	GO:00057	GO:0016279	protein-lysine N-methyltransferase activit
hypotheti	Putative	GO:00081	GO:00057	GO:0048037	cofactor binding;GO:0005488 binding;GO:000
branched-	Mitochond	GO:00195	GO:00057	GO:00526	(sce)Valine/isoleucine biosynthesis, pyruvat
Ste13p	Dipeptidy	GO:00073	GO:00058	GO:0004177	aminopeptidase activity;GO:0008238 exopept
DNA-bindi	Protein t	GO:00304	GO:00701	GO:0042162	telomeric DNA binding;GO:0043565 sequence-
translati	Polypepti	GO:00021	GO:00184	GO:00161	(sce03015)mRNA surveillance pathway

hypotheti	8-oxo-dG	GO:00081	GO:00055	GO:0044715	8-oxo-dGDP phosphatase activity;GO:0017110
tyrosine-	Mitochon	GO:00701	GO:00057	GO:00048	(sce00970)Aminoacyl-tRNA biosynthesis
None	None	None	None	None	None
mRNA-bin	Protein	GO:00004	GO:00306	GO:0003729	mRNA binding;GO:0003723 RNA binding;GO:000
phosphati	Phosphat	GO:00468	GO:00057	GO:00044	(sce00562)Inositol phosphate metabolism; (sc
None	None	None	None	None	None
hypotheti	Putative	GO:00550	GO:00057	GO:0003674	molecular_function
Aip1p	Actin co	GO:00510	GO:00426	GO:0051015	actin filament binding;GO:0003779 actin bi
Dot6p	Protein	GO:00615	GO:00702	GO:0043565	sequence-specific DNA binding;GO:0003677 D
None	None	None	None	None	None
hypotheti	Dubious	GO:00081	GO:00160	GO:0003674	molecular_function
UDP-gluc	Galactos	GO:00334	GO:00057	GO:00081	(sce)Nucleotide sugar biosynthesis, galactos
25S rRNA	Nucleola	GO:00311	GO:00057	GO:0016433	rRNA (adenine) methyltransferase activity;
Egt2p	Glycosyl	GO:00009	GO:00304	GO:0008810	cellulase activity;GO:0004553 hydrolase ac
Mfm1p	Mitochon	GO:00450	GO:00057	GO:0015095	magnesium ion transmembrane transporter ac
drug-res	Transcri	GO:20010	GO:00058	GO:0001228	DNA-binding transcription activator activi
Yif1p	Integral	GO:00150	GO:00301	GO:0003674	molecular_function
None	None	None	None	None	None
hypotheti	Putative	GO:00081	GO:00160	GO:0003674	molecular_function
alkaline	Repressi	GO:00464	GO:00003	GO:00473	(sce)Tetrahydrofolate biosynthesis, GTP => T
Ent3p	Protein	GO:00344	GO:00301	GO:00080	(sce04144)Endocytosis
None	None	None	None	None	None
Oaf3p	Putative	GO:00001	GO:00057	GO:0000981	DNA-binding transcription factor activity,
hypotheti	Putative	GO:00081	GO:00160	GO:0003674	molecular_function
None	None	None	None	None	None
transcri	Spt4p/5p	GO:20012	GO:00057	GO:0001181	RNA polymerase I general transcription ini
NADPH:qu	NADPH-de	GO:00345	GO:00056	GO:0032440	2-alkenal reductase [NAD(P)] activity;GO:0
serine/t	Nonessen	GO:19031	GO:00719	GO:0004674	protein serine/threonine kinase activity;G
Crn1p	Coronin;	GO:00343	GO:00304	GO:0071933	Arp2/3 complex binding;GO:0030674 protein
hypotheti	Protein	GO:00081	GO:00057	GO:0003674	molecular_function
None	None	None	None	None	None
None	None	None	None	None	None
None	None	None	None	None	None
None	None	None	None	None	None
None	None	None	None	None	None
Sar fami	Guanine	GO:00034	GO:00301	GO:00050	(sce04141)Protein processing in endoplasmic
Rna15p	Componen	GO:00724	GO:00058	GO:00037	(sce03015)mRNA surveillance pathway
hypotheti	Putative	GO:00081	GO:00055	GO:0003674	molecular_function
mitochon	Mitochon	GO:00325	GO:00057	GO:0003735	structural constituent of ribosome;GO:0005
MIND com	Essentia	GO:00345	GO:00008	GO:0003674	molecular_function
trehalos	Phosphat	GO:00304	GO:00059	GO:00038	(sce00500)Starch and sucrose metabolism; (sc
hypotheti	Dubious	GO:00081	GO:00160	GO:0003674	molecular_function
Spa2p	Componen	GO:00313	GO:19027	GO:00050	(sce04011)MAPK signaling pathway - yeast
putative	Protein	GO:00014	GO:00092	GO:00039	(sce00740)Riboflavin metabolism; (sce00730)T
divalent	Divalent	GO:00068	GO:00057	GO:0015086	cadmium ion transmembrane transporter acti
mitochon	Mitochon	GO:00325	GO:00057	GO:00037	(sce03010)Ribosome
anaphase	Subunit	GO:00300	GO:00056	GO:00616	(sce04120)Ubiquitin mediated proteolysis; (s
Eaf1p	Componen	GO:00062	GO:00352	GO:0003677	DNA binding;GO:0003676 nucleic acid bindin
GTPase-a	GTPase-a	GO:00457	GO:00057	GO:0005096	GTPase activator activity;GO:0030695 GTPas
Aim23p	Mitochon	GO:00701	GO:00057	GO:0043022	ribosome binding;GO:0003743 translation in
ESCRT-II	Myristoy	GO:19046	GO:00008	GO:00055	(sce04144)Endocytosis
hypotheti	Putative	GO:00081	GO:00057	GO:0005524	ATP binding;GO:0032559 adenyl ribonucleoti
SKI comp	Ski comp	GO:00704	GO:00550	GO:00040	(sce03018)RNA degradation
phosphop	Enolase	GO:00328	GO:00000	GO:00046	(M00002)Glycolysis, core module involving th

serine/t	Palmitoy	GO:00102	GO:00059	GO:00046	(sce04392)Hippo signaling pathway -multiple
seripaup	Member o	GO:00315	GO:00092	GO:0005199	structural constituent of cell wall;GO:000
ubiquiti	RING doma	GO:19901	GO:19901	GO:0043023	ribosomal large subunit binding;GO:0043022
U1 snRNP	Componen	GO:00003	GO:00056	GO:00306	(sce03040)Spliceosome
Tax4p	EH domai	GO:00322	GO:00004	GO:0003674	molecular_function
ubiquiti	E3 ubiqu	GO:00065	GO:00007	GO:0004842	ubiquitin-protein transferase activity;GO:
Vps60p	Protein	GO:00325	GO:00003	GO:00036	(sce04144)Endocytosis
None	None	None	None	None	None
None	None	None	None	None	None
2-isopro	Alpha-is	GO:00090	GO:00057	GO:00038	(sce)Leucine biosynthesis, 2-oxoisovalerate
Mei4p	Meiosis-	GO:00421	GO:00007	GO:0003674	molecular_function
Spi1p	GPI-anch	GO:00104	GO:00092	GO:0005199	structural constituent of cell wall;GO:000
Pba1p	Protein	GO:00432	GO:00057	GO:0003674	molecular_function
ribosoma	Protein	GO:00021	GO:00226	GO:00037	(sce03010)Ribosome
calmodul	Calmodul	GO:19035	GO:00058	GO:00510	(sce04070)Phosphatidylinositol signaling sys
L-aminoa	Alpha am	GO:00198	GO:00057	GO:00040	(M00030)Lysine biosynthesis, AAA pathway, 2-
Srl2p	Protein	GO:00061	GO:00056	GO:0003674	molecular_function
Rsc9p	Componen	GO:00615	GO:00165	GO:0015616	DNA translocase activity;GO:0008094 DNA-de
Pth4p	Protein	GO:00701	GO:00057	GO:0003747	translation release factor activity;GO:000
Sac7p	GTPase a	GO:00900	GO:00059	GO:00050	(sce04011)MAPK signaling pathway - yeast
S-adenos	S-adenos	GO:00064	GO:00057	GO:0008276	protein methyltransferase activity;GO:0008
histone	Trichost	GO:00709	GO:00056	GO:0032041	NAD-dependent histone deacetylase activity
U4/U6-U5	Lsm (Lik	GO:00304	GO:00056	GO:00037	(sce03018)RNA degradation; (sce03040)Spliceo
serine/t	Serine/t	GO:00460	GO:00056	GO:0004674	protein serine/threonine kinase activity;G
mitogen-	MAPKKK a	GO:00001	GO:00433	GO:00047	(sce04139)Mitophagy - yeast; (sce04011)MAPK
poly(A)-	Catalyti	GO:00002	GO:00312	GO:00045	(sce03018)RNA degradation
None	None	None	None	None	None
RNA-bind	Signal r	GO:00066	GO:00057	GO:00309	(sce03060)Protein export
1,3-beta-	1,3-beta-	GO:00719	GO:00092	GO:0042124	1,3-beta-glucanosyltransferase activity;GO
None	None	None	None	None	None
exopolyp	Exopolyp	GO:00067	GO:00057	GO:00043	(sce00230)Purine metabolism
E1 ubiqu	Subunit	GO:00169	GO:00315	GO:00199	(sce04120)Ubiquitin mediated proteolysis
glutamin	Glutamin	GO:00973	GO:00057	GO:00048	(sce00970)Aminoacyl-tRNA biosynthesis; (sce0
Tom20p	Componen	GO:00160	GO:00057	GO:0030943	mitochondrion targeting sequence binding;G
Mex67p	Poly(A)R	GO:00000	GO:00422	GO:00037	(sce03015)mRNA surveillance pathway; (sce030
hypothet	Putative protein of unknown function; green fluorescent protein (GFP)-fusion pro				
chromati	Nucleoso	GO:00004	GO:00353	GO:0001073	transcription antitermination factor activ
hypothet	Putative	GO:00081	GO:00057	GO:0003674	molecular_function
phosphog	6-phosph	GO:00090	GO:00057	GO:00046	(M00006)Pentose phosphate pathway, oxidative
tetherin	Componen	GO:00328	GO:00332	GO:00306	(sce04138)Autophagy - yeast
Pmd1p	Protein	GO:00304	GO:00057	GO:0003674	molecular_function
hypothet	Putative protein of unknown function				
Rim1p	ssDNA-bi	GO:00902	GO:00426	GO:0003697	single-stranded DNA binding;GO:0003677 DNA
TFIIH/NE	Componen	GO:00169	GO:00056	GO:00156	(sce03022)Basal transcription factors; (sce0
thioredo	Cytoplas	GO:00800	GO:00057	GO:0015035	protein disulfide oxidoreductase activity;
cAMP-dep	cAMP-dep	GO:00106	GO:00059	GO:00046	(sce04213)Longevity regulating pathway - mul
microtub	Microtub	GO:00315	GO:00353	GO:0051010	microtubule plus-end binding;GO:0008017 mi
Ino4p	Transcri	GO:00086	GO:00905	GO:0000978	RNA polymerase II proximal promoter sequen
hypothet	Dubious	GO:00081	GO:00055	GO:0003674	molecular_function
oligo-1,	Alpha-gl	GO:00000	GO:00055	GO:00045	(sce00052)Galactose metabolism; (sce00500)St
hypothet	Putative	GO:00081	GO:00055	GO:0003674	molecular_function
Pcl1p	Cyclin,	GO:00311	GO:00003	GO:00165	(sce04111)Cell cycle - yeast
thioredo	Thiol-sp	GO:00427	GO:00057	GO:00083	(sce04122)Sulfur relay system
amphiphy	Calmodul	GO:00609	GO:19905	GO:00055	(sce04144)Endocytosis

glutathi(	Glutathi(	GO:00067	GO:0057	GO:0043295 glutathione binding;GO:1900750 oligopeptid	
ssDNA en(	Single-s1	GO:00007	GO:00001	GO:00000	(sce03420)Nucleotide excision repair
UV-damage	Nucleoti(	GO:00007	GO:00001	GO:0008094 DNA-dependent ATPase activity;GO:0003684 d	
Brl1p	Essentia	GO:00069	GO:00319	GO:0003674 molecular_function	
Rbl2p	Protein	GO:00070	GO:00058	GO:0048487 beta-tubulin binding;GO:0015631 tubulin bi	
Mei5p	Meiosis-s	GO:00007	GO:00007	GO:0003674 molecular_function	
Eaf7p	Subunit (	GO:00165	GO:00352	GO:0003674 molecular_function	
Rax2p	N-glycosy	GO:00611	GO:19029	GO:0003674 molecular_function	
Mil1p	Predicte(	GO:00081	GO:00160	GO:0003674 molecular_function	
hypothet	Putative	GO:00551	GO:0057	GO:0016639 oxidoreductase activity, acting on the CH-	
putative	Ankyrin	GO:00183	GO:00003	GO:0019706 protein-cysteine S-palmitoyltransferase ac	
hypothet	Putative	GO:00081	GO:00719	GO:0003674 molecular_function	
ESCRT-II	Class E	GO:19046	GO:00008	GO:00036	(sce04144)Endocytosis
maturase-	Maturase-	GO:00003	GO:0057	GO:0004536 deoxyribonuclease activity;GO:0004519 endo	
RNA-DNA	Ribonucl(	GO:00431	GO:00056	GO:00045	(sce03030)DNA replication
Osw7p	Protein	GO:00304	GO:00057	GO:0003674 molecular_function	
Spo13p	Meiotic	GO:00511	GO:00726	GO:0003674 molecular_function	
S-adenosy	S-adenosy	GO:00064	GO:00055	GO:00307	(sce00340)Histidine metabolism
NADH kin	Mitochon(	GO:00067	GO:00057	GO:0042736 NADH kinase activity;GO:0003951 NAD+ kinas	
amino ac	bZIP tra	GO:00106	GO:00007	GO:00011	(sce04138)Autophagy - yeast
Dfm1p	Endoplas	GO:00304	GO:00008	GO:00036	(sce04141)Protein processing in endoplasmic
thiamine	Thiamine	GO:00092	GO:0057	GO:00047	(sce00730)Thiamine metabolism; (sce01100)Met
Chs6p	Member o	GO:00342	GO:00340	GO:0003674 molecular_function	
oxidored	NADP(+)-(	GO:00551	GO:00058	GO:00311	(sce00260)Glycine, serine and threonine metal
pheromone	Potassiu	GO:00718	GO:00059	GO:0015079 potassium ion transmembrane transporter ac	
NEDD4 fa	NEDD4 fa	GO:20002	GO:00304	GO:00431	(sce04120)Ubiquitin mediated proteolysis; (s
mRNA-pro(	RNA endo	GO:00710	GO:00056	GO:0004521 endoribonuclease activity;GO:0004540 ribon	
snoRNA-b	Componen	GO:00422	GO:00344	GO:00305	(sce03008)Ribosome biogenesis in eukaryotes
putative	Protein (	GO:00352	GO:00975	GO:00041	(sce00515)Mannose type O-glycan biosynthesis
phospho	Phospho	GO:00065	GO:00056	GO:00046	(M00020)Serine biosynthesis, glycerate-3P =>
putative	Putative	GO:00068	GO:00003	GO:0015123 acetate transmembrane transporter activity	
Snn1p	Subunit (	GO:00070	GO:00310	GO:0003674 molecular_function	
aspartyl	Putative	GO:00315	GO:00092	GO:0004190 aspartic-type endopeptidase activity;GO:00	
metal ca	Endoplas	GO:00068	GO:00319	GO:0005385 zinc ion transmembrane transporter activit	
histone (	Histone (	GO:00611	GO:00702	GO:00320	(sce04213)Longevity regulating pathway - mul
mitochon(	Mitochon(	GO:00325	GO:00057	GO:0003735 structural constituent of ribosome;GO:0005	
Bbc1p	Protein	GO:00343	GO:00304	GO:0017024 myosin I binding;GO:0017022 myosin binding	
H(+)-tra	Proteoli	GO:00070	GO:00002	GO:00469	(sce04145)Phagosome; (sce00190)Oxidative pho
Gat3p	Protein (	GO:00063	GO:00056	GO:0008270 zinc ion binding;GO:0043565 sequence-speci	
Vht1p	High-aff	GO:00158	GO:00058	GO:0015225 biotin transmembrane transporter activity;	
3'-5' po	3'-5' ex(	GO:00436	GO:00055	GO:0004535 poly(A)-specific ribonuclease activity;GO:	
Vid22p	Glycosyl	GO:00070	GO:00057	GO:0003677 DNA binding;GO:0046872 metal ion binding;G	
Erv46p	Protein	GO:00068	GO:00301	GO:0003674 molecular_function	
Sma1p	Protein (	GO:00321	GO:00056	GO:0003674 molecular_function	
Ppr1p	Zinc fin	GO:19003	GO:00056	GO:0000981 DNA-binding transcription factor activity,	
Vac14p	Enzyme r	GO:19037	GO:00707	GO:00329	(Human)(hsa05203)Viral carcinogenesis; (hsa0
Dsc3p	Subunit (	GO:00068	GO:00446	GO:0003674 molecular_function	
None	None	None	None	None	None
Mtc7p	Protein (	GO:00081	GO:00160	GO:0003674 molecular_function	
monothio	Glutathi(	GO:00310	GO:00058	GO:0015038 glutathione disulfide oxidoreductase activ	
Dse2p	Daughter	GO:00071	GO:00092	GO:0015926 glucosidase activity;GO:0004553 hydrolase	
alpha-ma	Vacuolar	GO:00193	GO:00342	GO:00045	(sce00511)Other glycan degradation
None	None	None	None	None	None
Sds23p	Protein	GO:00300	GO:00056	GO:0003674 molecular_function	

Scm3p	Nonhistor	GO:20000	GO:00007	GO:0097030 CENP-A containing nucleosome binding;GO:00	
Pex14p	Central	GO:00165	GO:19904	GO:00306	(sce04146)Peroxisome
Rnp1p	Ribonucle	GO:00422	GO:00104	GO:0008143 poly(A) binding;GO:0008266 poly(U) RNA bin	
None	None	None	None	None	None
Vtc4p	Vacuolar	GO:00067	GO:00332	GO:0008976 polyphosphate kinase activity;GO:0000822 i	
serine/t	Ser-Thr	GO:19001	GO:00056	GO:0004674 protein serine/threonine kinase activity;G	
putative	Probable	GO:00361	GO:00057	GO:0016746 transferase activity, transferring acyl gr	
seipin	Seipin i	GO:00343	GO:00325	GO:0003674 molecular_function	
glucose-(	Glucose-(	GO:00442	GO:00056	GO:00479	(sce00010)Glycolysis / Gluconeogenesis; (sce
non-canon	Non-canon	GO:00710	GO:00314	GO:00344	(sce03018)RNA degradation
peptidylp	Mitochon	GO:00004	GO:00057	GO:0016018 cyclosporin A binding;GO:0003755 peptidyl-	
thiamine	Thiazole	GO:00528	GO:00058	GO:00081	(sce00730)Thiamine metabolism; (sce01100)Met
protein-t	Cytoplasm	GO:00353	GO:00057	GO:0004725 protein tyrosine phosphatase activity;GO:0	
Ahc2p	Component	GO:00165	GO:0005671 Ada2/Gcn5/Ada3 transcription activator complex;GO:1		
spermine	Polyamine	GO:19037	GO:00003	GO:0000297 spermine transmembrane transporter activit	
Axl2p	Integral	GO:00071	GO:00001	GO:0005509 calcium ion binding;GO:0046872 metal ion b	
Pet494p	Mitochon	GO:00701	GO:00057	GO:0045182 translation regulator activity;GO:0003674	
polynucl	Polynucle	GO:00004	GO:00308	GO:0051731 polynucleotide 5'-hydroxyl-kinase activity	
protein	Mitochon	GO:19041	GO:00057	GO:0004740 pyruvate dehydrogenase (acetyl-transferrin	
Env9p	Protein	GO:00066	GO:00058	GO:00164	(Human)(hsa00830)Retinol metabolism; (hsa011
succinat	Iron-sul	GO:00060	GO:00057	GO:00515	(sce)Citrate cycle, second carbon oxidation,
Pam17p	Constitu	GO:00301	GO:00014	GO:0003674 molecular_function	
Mss51p	Specific	GO:00701	GO:00313	GO:0045182 translation regulator activity;GO:0003674	
sepiapte	Putative	GO:00551	GO:00160	GO:0102306 benzil reductase [(S)-benzoin-forming] act	
Rbs1p	Protein	GO:19901	GO:00056	GO:0003723 RNA binding;GO:0003676 nucleic acid bindin	
hypothet	Putative	GO:00081	GO:00055	GO:0003674 molecular_function	
guanine	Guanine	GO:00422	GO:00306	GO:00050	(sce03008)Ribosome biogenesis in eukaryotes
ubiquiti	Component	GO:00712	GO:00331	GO:00084	(sce04136)Autophagy - other; (sce04139)Mitop
glycerop	Glycerop	GO:00464	GO:00058	GO:00473	(sce00564)Glycerophospholipid metabolism
Ysc83p	Non-esse	GO:00081	GO:00057	GO:0003674 molecular_function	
Dfg16p	Probable	GO:00071	GO:00058	GO:0003674 molecular_function	
protein	DNA repl	GO:19025	GO:00312	GO:0070182 DNA polymerase binding;GO:0030295 protein	
pheromon	Pheromon	GO:00160	GO:00057	GO:0003674 molecular_function	
hypothet	Protein	GO:00081	GO:00056	GO:0003674 molecular_function	
hypothet	Protein	GO:00081	GO:00055	GO:0003674 molecular_function	
tethering	Subunit	GO:00328	GO:00332	GO:00350	(sce04138)Autophagy - yeast
thymidyl	Thymidyl	GO:00062	GO:00343	GO:00047	(sce00670)One carbon pool by folate; (sce002
putative	Cytoplasm	GO:00716	GO:19903	GO:0061630 ubiquitin protein ligase activity;GO:00616	
Seg1p	Component	GO:00709	GO:00321	GO:0043022 ribosome binding;GO:0043021 ribonucleoprot	
ribosoma	Ribosoma	GO:00171	GO:00226	GO:00037	(sce03010)Ribosome
Cha4p	DNA bind	GO:00090	GO:00056	GO:0000981 DNA-binding transcription factor activity,	
DNA-dire	Mitochon	GO:00320	GO:00057	GO:00038	(sce04139)Mitophagy - yeast; (sce01100)Metab
Aep3p	Periphera	GO:00701	GO:00057	GO:0003674 molecular_function	
guanylat	Guanylat	GO:00467	GO:00058	GO:00043	(sce)Guanine ribonucleotide biosynthesis IMP
putative	Subunit	GO:00159	GO:19901	GO:00041	(sce)Coenzyme A biosynthesis, pantothenate =
chromati	General	GO:00359	GO:00170	GO:00800	(sce04011)MAPK signaling pathway - yeast; (s
Mlp2p	Myosin-l	GO:19019	GO:00446	GO:00170	(sce03013)RNA transport
None	None	None	None	None	None
high-mob	High-mob	GO:00011	GO:00007	GO:0032407 MutSalpha complex binding;GO:0032404 misma	
pseudour	Mitochon	GO:00311	GO:00057	GO:0106029 tRNA pseudouridine synthase activity;GO:00	
B-type c	B-type c	GO:00106	GO:00003	GO:00165	(sce04111)Cell cycle - yeast; (sce04113)Meio
peptidylp	Nucleola	GO:00515	GO:00057	GO:0005527 macrolide binding;GO:0003755 peptidyl-prol	
Iqg1p	Essentia	GO:19034	GO:00001	GO:0051015 actin filament binding;GO:0005516 calmodul	
seripaup	Protein	GO:00315	GO:00092	GO:0005199 structural constituent of cell wall;GO:000	

carboxyli	Mitochon	GO:00359	GO:00057	GO:00046	(sce00564)Glycerophospholipid metabolism
3-isopro	Isopropy	GO:00090	GO:00093	GO:00038	(sce)Leucine biosynthesis, 2-oxoisovalerate =
putative	Protein i	GO:00304	GO:00001	GO:00038	(sce00500)Starch and sucrose metabolism; (sc
None	None	None	None	None	None
None	None	None	None	None	None
Bem3p	Rho GTPa	GO:00350	GO:00059	GO:00322	(sce04011)MAPK signaling pathway - yeast
3'-5' DN	3'-5' DN	GO:00605	GO:00007	GO:0043140 ATP-dependent 3'-5' DNA helicase activity;	
Cue1p	Ubiquiti	GO:00970	GO:00008	GO:00970	(sce04141)Protein processing in endoplasmic
Bch2p	Member o	GO:00342	GO:00340	GO:0003674 molecular_function	
Sec31p	Componen	GO:00901	GO:00301	GO:00051	(sce04141)Protein processing in endoplasmic
Ady4p	Structur	GO:00304	GO:00359	GO:0005198 structural molecule activity;GO:0003674 mo	
None	None	None	None	None	None
Atp22p	Specific	GO:00701	GO:00057	GO:0045182 translation regulator activity;GO:0003674	
hypothet	Protein	GO:00709	GO:00321	GO:0003674 molecular_function	
calcineu	Calcineu	GO:00007	GO:00059	GO:00047	(Human)(hsa05014)Amyotrophic lateral scleros
Oac1p	Mitochon	GO:19015	GO:00057	GO:0034658 isopropylmalate transmembrane transporter	
aspartat	Vacuolar	GO:19034	GO:00003	GO:0061459 L-arginine transmembrane transporter activ	
serine/t	S/T prot	GO:00610	GO:00059	GO:00046	(Human)(hsa04068)FoxO signaling pathway; (hs
Mbr1p	Protein i	GO:00090	GO:00057	GO:0003674 molecular_function	
DNA tran	ATP-depe	GO:00601	GO:00086	GO:0015616 DNA translocase activity;GO:0019237 centro	
Ies4p	Componen	GO:00007	GO:00310	GO:0003674 molecular_function	
Atg33p	Mitochon	GO:00004	GO:00319	GO:00036	(sce04139)Mitophagy - yeast
hypothet	Putative	GO:00081	GO:00055	GO:0003674 molecular_function	
Env11p	Protein	GO:00066	GO:00057	GO:0003677 DNA binding;GO:0046872 metal ion binding;	
glyoxyla	Glyoxyla	GO:00551	GO:00058	GO:0016618 hydroxypyruvate reductase activity;GO:0030	
Gcs1p	ADP-ribo	GO:00300	GO:00058	GO:00050	(sce04144)Endocytosis
None	None	None	None	None	None
GTPase-a	GTPase-a	GO:00070	GO:00058	GO:0005096 GTPase activator activity;GO:0030695 GTPas	
Rrg9p	Protein	GO:00000	GO:00057	GO:0003674 molecular_function	
fatty ac	Elongase	GO:00346	GO:00301	GO:01027	(sce)Fatty acid biosynthesis, elongation, en
hypothet	Putative	GO:00081	GO:00055	GO:0003674 molecular_function	
Dse1p	Daughter	GO:00109	GO:00059	GO:0031435 mitogen-activated protein kinase kinase ki	
ribosoma	Protein	GO:00004	GO:00226	GO:00037	(sce03010)Ribosome
Nsi1p	RNA poly	GO:19000	GO:00335	GO:0043022 ribosome binding;GO:0043021 ribonucleoprot	
carboxyp	Putative serine type carboxypeptidase; role in phytochelatin synthesis; green fl				
mitochon	Mitochon	GO:00701	GO:00057	GO:0003735 structural constituent of ribosome;GO:0005	
DNA-dire	RNA poly	GO:00427	GO:00056	GO:00010	(sce03020)RNA polymerase; (sce00240)Pyrimidi
phospho	Protein	GO:00512	GO:00308	GO:00081	(sce04111)Cell cycle - yeast; (sce04113)Meio
ribosoma	Ribosoma	GO:00468	GO:00226	GO:00037	(sce03010)Ribosome
oleate-a	Oleate-a	GO:00614	GO:00897	GO:0000978 RNA polymerase II proximal promoter sequen	
Rds2p	Transcri	GO:00614	GO:00056	GO:0000978 RNA polymerase II proximal promoter sequen	
Scd5p	Protein	GO:20003	GO:00304	GO:0008157 protein phosphatase 1 binding;GO:0019903 p	
hypothet	Protein	GO:00081	GO:00056	GO:0003674 molecular_function	
Nut1p	Componen	GO:00063	GO:00165	GO:0003712 transcription coregulator activity;GO:0140	
hypothet	Putative	GO:00081	GO:00057	GO:0003674 molecular_function	
DNA-bind	Zinc clu	GO:00359	GO:00056	GO:0000978 RNA polymerase II proximal promoter sequen	
transcri	Transcri	GO:00458	GO:00056	GO:0008301 DNA binding, bending;GO:0003713 transcript	
1-phosph	Phosphat	GO:00468	GO:00057	GO:00044	(sce00562)Inositol phosphate metabolism; (sc
None	None	None	None	None	None
hypothet	Dubious	GO:00081	GO:00055	GO:0003674 molecular_function	
Nup84p	Subunit	GO:00353	GO:00310	GO:00170	(sce03013)RNA transport
mitotic	Protein	GO:00615	GO:00059	GO:00050	(sce04111)Cell cycle - yeast
ATP phos	ATP phos	GO:00001	GO:00057	GO:00038	(sce)Histidine biosynthesis, PRPP => histidi
Hot1p	Transcri	GO:00723	GO:00056	GO:00162	(sce04011)MAPK signaling pathway - yeast

Red1p	Protein (	GO:00515	GO:00008	GO:00314	(sce04113)Meiosis - yeast
Etr1p	2-enoyl	GO:00066	GO:00057	GO:00166	(sce00062)Fatty acid elongation; (sce01212)F
Rab fami	Rab fami	GO:00344	GO:00550	GO:00039	(sce04144)Endocytosis
Svf1p	Protein v	GO:00345	GO:00056	GO:0003674 molecular_function	
None	None	None	None	None	None
phosphol	Acyltrans	GO:00194	GO:00970	GO:00460	(sce00561)Glycerolipid metabolism
Yhp1p	Homeobox	GO:00719	GO:00007	GO:00009	(sce04111)Cell cycle - yeast
Spc98p	Componen	GO:00514	GO:00009	GO:0051011 microtubule minus-end binding;GO:0043015 g	
kynureni	Kynureni	GO:00970	GO:00057	GO:00162	(sce00380)Tryptophan metabolism; (sce01100)M
Smm1p	Dihydrou	GO:00029	GO:00056	GO:0102264 tRNA-dihydrouridine20 synthase activity;GO	
putative	Serine/t	GO:00315	GO:00058	GO:0004674 protein serine/threonine kinase activity;G	
Hap2p	Subunit (	GO:00004	GO:00166	GO:0000978 RNA polymerase II proximal promoter sequen	
exocyst	Essentia	GO:00905	GO:00056	GO:0003674 molecular_function	
myosin 1	Type II n	GO:00009	GO:00001	GO:00001	(Human)(hsa05416)Viral myocarditis; (hsa0426
Aro80p	Zinc fin	GO:00065	GO:00056	GO:0000981 DNA-binding transcription factor activity,	
Qri5p	Mitochon	GO:00325	GO:00313	GO:0003735 structural constituent of ribosome;GO:0005	
Ugx2p	Protein (	GO:00081	GO:00055	GO:0003674 molecular_function	
Apl5p	Delta ad	GO:00066	GO:00301	GO:00055	(Human)(hsa04142)Lysosome
Per1p	Protein (	GO:00300	GO:00003	GO:0016788 hydrolase activity, acting on ester bonds;	
putative	Putative	GO:00342	GO:00055	GO:0004360 glutamine-fructose-6-phosphate transaminas	
Tvp23p	Integral	GO:00093	GO:00301	GO:0003674 molecular_function	
None	None	None	None	None	None
hypothet	Putative	GO:00081	GO:00055	GO:0003674 molecular_function	
DNA-dire	RNA poly	GO:00427	GO:00056	GO:00039	(sce03020)RNA polymerase; (sce00240)Pyrimidi
Tda8p	Putative	GO:00081	GO:00055	GO:0003674 molecular_function	
Oaz1p	Regulato	GO:20011	GO:00057	GO:0008073 ornithine decarboxylase inhibitor activity	
Soh1p	Subunit (	GO:00063	GO:00708	GO:0003713 transcription coactivator activity;GO:0003	
hypothet	Protein (	GO:00070	GO:00160	GO:0003674 molecular_function	
dynein i	Dynein i	GO:20005	GO:00058	GO:00085	(sce04145)Phagosome
None	None	None	None	None	None
protein	ATPase r	GO:19010	GO:00313	GO:0016887 ATPase activity;GO:0004672 protein kinase	
Dph1p	Protein	GO:00171	GO:00057	GO:0016740 transferase activity;GO:0003824 catalytic	
hypothet	Putative	GO:00081	GO:00160	GO:0003674 molecular_function	
sulfate	ATP sulf	GO:00101	GO:00057	GO:00047	(sce00261)Monobactam biosynthesis; (M00176)A
Mpc54p	Componen	GO:00321	GO:00359	GO:0005198 structural molecule activity;GO:0003674 mo	
None	None	None	None	None	None
diacylgl	Diacylgl	GO:00353	GO:00058	GO:00038	(sce00561)Glycerolipid metabolism
Rad2 fam	5'-3' ex	GO:00312	GO:00056	GO:00353	(sce03430)Mismatch repair
Lsm12p	Protein (	GO:00160	GO:00104	GO:0003723 RNA binding;GO:0003676 nucleic acid bindin	
CCR4-NOT	Componen	GO:00310	GO:00300	GO:00048	(sce03018)RNA degradation
putative	Protein v	GO:00165	GO:00314	GO:0004842 ubiquitin-protein transferase activity;GO:	
serine/t	Serine/t	GO:00459	GO:00058	GO:0004674 protein serine/threonine kinase activity;G	
Brx1p	Nucleola	GO:00004	GO:00306	(Human)GO:0003723 RNA binding;GO:0003676 nucleic acid	
metallod	Cys-Gly r	GO:00067	GO:00058	GO:00705	(sce00480)Glutathione metabolism; (sce01100)
Him1p	Protein (	GO:00062	GO:00055	GO:0003674 molecular_function	
UDP-N-ac	UDP-N-ac	GO:00064	GO:00435	GO:00039	(sce)N-glycan precursor biosynthesis; (M0005
hypothet	Protein of unknown function; green fluorescent protein (GFP)-fusion protein loca				
hypothet	Putative	GO:00083	GO:00056	GO:0003674 molecular_function	
Rrt12p	Probable	GO:00304	GO:00056	GO:0004252 serine-type endopeptidase activity;GO:0008	
protein-	Essentia	GO:00312	GO:00312	GO:00085	(sce03060)Protein export; (sce04141)Protein
Kap123p	Karyophe	GO:20002	GO:00104	GO:0008139 nuclear localization sequence binding;GO:0	
Rab fami	Endosoma	GO:00617	GO:00057	GO:00039	(sce04145)Phagosome; (sce04144)Endocytosis
palmitoy	Acyl-pro	(Human)G	(Human)G	(Human)G	(Human)(hsa00564)Glycerophospholipid metabol
3',5'-cy	Low-affi	GO:00460	GO:00055	GO:00041	(sce00230)Purine metabolism

phosphati	PIK-relat	GO:190535	GO:00319	GO:00046	(sce04136)Autophagy - other; (sce04213)Longe
hypotheti	Putative	GO:000815	GO:000557	GO:0003674	molecular_function
hexose tı	Putative	GO:001575	GO:00003	GO:00053	(sce04113)Meiosis - yeast
Spt10p	Histone I	GO:00970	GO:00007	GO:0032931	histone acetyltransferase activity (H3-K56
hypotheti	Putative	GO:000815	GO:000557	GO:0003674	molecular_function
RNA-bindi	Essential	GO:00004	GO:003068	GO:0042134	rRNA primary transcript binding;GO:0019843
cyclin-de	Cyclin (I	GO:005157	GO:00080	GO:0008353	RNA polymerase II CTD heptapeptide repeat
Swa2p	Auxilin-l	GO:007231	GO:000578	GO:0043130	ubiquitin binding;GO:0030276 clathrin bind
protein p	Regulato	GO:006158	GO:000016	GO:0019888	protein phosphatase regulator activity;GO:
ribosome	Specific	GO:000636	GO:00057	GO:0051082	unfolded protein binding;GO:0005515 protei
leucine--	Cytosolic	GO:00064	GO:00057	GO:00048	(sce00970)Aminoacyl-tRNA biosynthesis
negative	Subunit c	GO:001648	GO:00170	GO:0017025	TBP-class protein binding;GO:0001047 core
None	None	None	None	None	None
Ncs6p	Protein ı	GO:00021	GO:00021	GO:00000	(sce04122)Sulfur relay system
cytochron	Cytochron	GO:001806	GO:000575	GO:00044	(sce00860)Porphyrin and chlorophyll metaboli
Sec63 con	Non-esser	GO:003120	GO:003120	GO:0008565	protein transporter activity;GO:0005215 tr
DNA-bindi	Mitochonc	GO:00901	GO:000026	GO:0008301	DNA binding, bending;GO:0008134 transcript
mismatch	Protein ı	GO:003287	GO:003230	GO:00321	(sce03430)Mismatch repair
Msa1p	Activato	GO:000708	GO:00056	GO:0003674	molecular_function
Cip1p	Cyclin-de	GO:00457	GO:00056	GO:0004861	cyclin-dependent protein serine/threonine
Mdr1p	Cytoplasm	GO:00906	GO:00057	GO:0017137	Rab GTPase binding;GO:0005096 GTPase activ
NEDD8 lig	Scaffold-	GO:00514	GO:000015	GO:0097602	cullin family protein binding;GO:0031624 u
Ina22p	F1F0 ATP	GO:003361	GO:19905	GO:0003674	molecular_function
Pbp4p	Pbp1p bir	GO:000815	GO:00104	GO:0003674	molecular_function
nucleopoı	Cytoplasm	GO:001697	GO:004461	GO:0000822	inositol hexakisphosphate binding;GO:00313
Bli1p	Subunit c	GO:00070	GO:003108	GO:0003674	molecular_function
Ics2p	Protein c	GO:000815	GO:000557	GO:0003674	molecular_function
ubiquitir	Peroxisom	GO:001655	GO:000577	GO:00048	(sce04146)Peroxisome
septin SF	Sporulati	GO:00304	GO:000561	GO:00329	(sce04113)Meiosis - yeast
syntaxin-	Hydrophil	GO:00355	GO:00301	GO:0019905	syntaxin binding;GO:0000149 SNARE binding;
None	None	None	None	None	None
Fyv4p	Protein c	GO:00325	GO:000576	GO:0003735	structural constituent of ribosome;GO:0005
Rrf1p	Mitochonc	GO:000641	GO:00057	GO:0003747	translation release factor activity;GO:000
Trk2p	Component	GO:003000	GO:000588	GO:0140107	high-affinity potassium ion transmembrane
putative	F-box prc	GO:00313	GO:001900	GO:0043022	ribosome binding;GO:0030674 protein bindin
Mum2p	Protein e	GO:20002	GO:00363	GO:0005515	protein binding;GO:0005488 binding;GO:0003
Bni5p	Linker pı	GO:00009	GO:00001	GO:0030674	protein binding, bridging;GO:0060090 molec
Wsc4p	Endoplasm	GO:00450	GO:000578	GO:0008565	protein transporter activity;GO:0005215 tr
mitochonc	Mitochonc	GO:00325	GO:000576	GO:00037	(sce03010)Ribosome
Cbf1p	Basic hel	GO:00614	GO:008971	GO:0019237	centromeric DNA binding;GO:0001103 RNA pol
ubiquitir	Ubiquitir	GO:00109	GO:000577	GO:00048	(Human)(hsa04068)FoxO signaling pathway; (hs
None	None	None	None	None	None
DNA-deper	DNA-deper	GO:003207	GO:00056	GO:001561	(sce03440)Homologous recombination
DNA-direc	RNA polym	GO:004277	GO:000566	GO:00010	(sce03020)RNA polymerase; (sce00240)Pyrimidi
putative	Putative ADP-ribose-1''-monophosphatase; converts ADP-ribose-1''-monophosphate t				
Nop8p	Nucleolar	GO:000046	GO:00057	GO:0003723	RNA binding;GO:0003676 nucleic acid bindin
adenylosı	Adenylosı	GO:00442	GO:00057	GO:000401	(sce)Adenine ribonucleotide biosynthesis, IM
TRAPP sul	Core comp	GO:000688	GO:199007	GO:0003674	molecular_function
None	None	None	None	None	None
diphthine	Methyltra	GO:001718	GO:00057	GO:0004164	diphthine synthase activity;GO:0008757 S-a
ATP-deper	Component	GO:000640	GO:00003	GO:00040	(sce03015)mRNA surveillance pathway; (sce030
1-phospha	Phosphati	GO:004685	GO:000580	GO:00044	(sce00562)Inositol phosphate metabolism; (sc
protein g	Beta subı	GO:00183	GO:000595	GO:0004662	CAAX-protein geranylgeranyltransferase act
hypotheti	Putative	GO:000815	GO:00160	GO:0003674	molecular_function

Cbs2p	Mitochon	GO:00333	GO:00313	GO:0008677	2-dehydropantoate 2-reductase activity;GO:
mannose-	GDP-mann	GO:00000	GO:00057	GO:00044	(sce00051)Fructose and mannose metabolism; (
Pin2p	Exomer-d	GO:00081	GO:00003	GO:0003674	molecular_function
DNA-dire	RNA polym	GO:00427	GO:00056	GO:00010	(sce03020)RNA polymerase; (sce00240)Pyrimidi
putative	Protein	GO:00550	GO:00003	GO:0000293	ferric-chelate reductase activity;GO:00167
mitogen-	MAP kina	GO:00001	GO:00057	GO:00047	(sce04011)MAPK signaling pathway - yeast
hypothet	Mitochon	GO:19025	GO:00057	GO:1902557	5'-adenylyl sulfate transmembrane transpor
ornithin	L-ornith	GO:00101	GO:00058	GO:00045	(sce00330)Arginine and proline metabolism; (
Vba2p	Permease	GO:19908	GO:00003	GO:0015174	basic amino acid transmembrane transporter
hypothet	Putative protein of unknown function; YGR168C is not an essential gene				
CDP-diac	Phosphat	GO:00066	GO:00057	GO:00038	(sce00562)Inositol phosphate metabolism; (sc
cyclin-d	Cyclin-d	GO:00004	GO:00165	GO:0008353	RNA polymerase II CTD heptapeptide repeat
ribosoma	Ribosoma	GO:00422	GO:00226	GO:00037	(sce03010)Ribosome
Atg23p	Periphera	GO:00344	GO:00340	GO:00036	(sce04138)Autophagy - yeast
Ca(2+)-d	Ca2+-dep	GO:00065	GO:00058	GO:0004198	calcium-dependent cysteine-type endopeptid
DNA dama	RNAPII d	GO:00062	GO:00007	GO:0003677	DNA binding;GO:0003676 nucleic acid bindin
Hsp10p	Mitochon	GO:00450	GO:00057	GO:0051087	chaperone binding;GO:0051082 unfolded prot
Atg21p	Phosphoi	GO:00344	GO:00340	GO:0080025	phosphatidylinositol-3,5-bisphosphate bind
CCR4-NOT	Componen	GO:00002	GO:00300	GO:00048	(sce03018)RNA degradation
condensi	Subunit	GO:19033	GO:00007	GO:00036	(sce04111)Cell cycle - yeast
dynein l	Cytoplas	GO:20005	GO:19904	GO:0008574	ATP-dependent microtubule motor activity,
inositol	Phosphat	GO:00066	GO:00709	GO:0045140	inositol phosphoceramide synthase activity
phosphop	Enolase,	GO:00060	GO:00000	GO:00046	(M00002)Glycolysis, core module involving th
None	None	None	None	None	None
hypothet	Putative	GO:00081	GO:00160	GO:0003674	molecular_function
Ctf18p	Subunit	GO:00357	GO:00313	GO:0005524	ATP binding;GO:0032559 adenyl ribonucleoti
protein	Protein	GO:00070	GO:00007	GO:00046	(sce04111)Cell cycle - yeast; (sce04113)Meio
ATP-bind	ATPase o	GO:00000	GO:00306	GO:0042624	ATPase activity, uncoupled;GO:0043022 ribo
anthrani	Anthrani	GO:00001	GO:00057	GO:00040	(sce)Tryptophan biosynthesis, chorismate =>
phosphog	Phosphog	GO:00059	GO:00058	GO:00046	(M00549)Nucleotide sugar biosynthesis, gluco
ATP-bind	Plasma m	GO:00353	GO:00057	GO:0034041	sterol-transporting ATPase activity;GO:003
Fmp33p	Putative	GO:00081	GO:00319	GO:0003674	molecular_function
SUMO pro	Protease	GO:00169	GO:00056	GO:0070140	SUMO-specific isopeptidase activity;GO:007
histone	Nucleoso	GO:00314	GO:00991	GO:00311	(sce00310)Lysine degradation
hypothet	Putative	GO:00081	GO:00055	GO:0003674	molecular_function
Tma10p	Protein	GO:00081	GO:00057	GO:0043023	ribosomal large subunit binding;GO:0043024
N,N-dime	Flavin-c	GO:00064	GO:00057	GO:0004499	N,N-dimethylaniline monooxygenase activity
Stu2p	Microtub	GO:00309	GO:00353	GO:0051010	microtubule plus-end binding;GO:0008017 mi
carboxym	Putative	GO:00081	GO:00057	GO:00088	(sce01130)Biosynthesis of antibiotics; (sce0
None	None	None	None	None	None
Swm1p	Subunit	GO:19048	GO:00056	GO:00616	(sce04120)Ubiquitin mediated proteolysis; (s
Rga2p	GTPase-a	GO:00311	GO:00321	GO:00050	(sce04011)MAPK signaling pathway - yeast
bifuncti	Diadenos	GO:00091	GO:00056	GO:00047	(sce00230)Purine metabolism
hypothet	Putative	GO:00081	GO:00055	GO:0003674	molecular_function
hypothet	Putative	GO:00081	GO:00055	GO:0003674	molecular_function
hypothet	Protein	GO:00081	GO:00003	GO:0003674	molecular_function
histone	Histone	GO:00063	GO:00312	GO:0046982	protein heterodimerization activity;GO:004
endopept	Catalyti	GO:00066	GO:00427	GO:00082	(sce03060)Protein export
Jid1p	Probable	GO:00081	GO:00319	GO:0003674	molecular_function
L-serine	Cataboli	GO:00065	GO:00426	GO:00047	(sce)Isoleucine biosynthesis, threonine => 2-
ribosoma	Protein	GO:00021	GO:00226	GO:00037	(sce03010)Ribosome
dihydrol	Dihydrol	GO:00060	GO:00059	GO:00047	(M00307)Pyruvate oxidation, pyruvate => acet
mannose-	Protein	GO:00158	GO:00092	GO:00513	(sce)GPI-anchor biosynthesis, core oligosacc
Cst6p	Basic le	GO:00614	GO:00058	GO:0001228	DNA-binding transcription activator activi

ferrochel	Ferrochel	GO:00067	GO:00057	GO:00043	(sce00860)Porphyrin and chlorophyll metaboli
None	None	None	None	None	None
hypotheti	Protein (	GO:00551	GO:00056	GO:0016491 oxidoreductase activity;GO:0003824 catalyt	
Haa1p	Transcrip	GO:00614	GO:00056	GO:0005507 copper ion binding;GO:0000978 RNA polymera	
ribosomal	Ribosomal	GO:00004	GO:00226	GO:00037	(sce03010)Ribosome
Tho2p	Subunit c	GO:20012	GO:00004	GO:00037	(sce03040)Spliceosome; (sce03013)RNA transpo
mitochon	Mitochon	GO:00325	GO:00057	GO:0003735 structural constituent of ribosome;GO:0005	
mRNA spl	Essential	GO:00003	GO:00710	GO:0000384 first spliceosomal transesterification act	
UDP-N-ac	UDP-N-ac	GO:00060	GO:00056	GO:00039	(sce00520)Amino sugar and nucleotide sugar m
Dal81p	Positive	GO:19017	GO:00056	GO:0001135 RNA polymerase II transcription regulator	
N-acetylg	ER membra	GO:00065	GO:00057	GO:00002	(sce)GPI-anchor biosynthesis, core oligosacc
glutamat	Mitochon	GO:00701	GO:00057	GO:00505	(sce00860)Porphyrin and chlorophyll metaboli
Yip3p	Protein localized	GO:00301	GO:0003674 molecular_function		
cleavage	Essential	GO:00308	GO:00058	GO:00037	(sce03015)mRNA surveillance pathway
putative	Plasma me	GO:00068	GO:00057	GO:0015123 acetate transmembrane transporter activity	
hypotheti	Protein (	GO:00081	GO:00058	GO:0043022 ribosome binding;GO:0043021 ribonucleoprot	
condensin	Subunit c	GO:19033	GO:00007	GO:00036	(sce04111)Cell cycle - yeast
hypotheti	Protein (	GO:00081	GO:00055	GO:0003674 molecular_function	
U4/U6-U5	Splicing	GO:00003	GO:00465	GO:00036	(sce03040)Spliceosome
Mmt2p	Putative	GO:00068	GO:00319	GO:0008324 cation transmembrane transporter activity;	
hypotheti	Protein (	GO:00081	GO:00055	GO:0003674 molecular_function	
Ecm32p	DNA deper	GO:00064	GO:00104	GO:0004004 ATP-dependent RNA helicase activity;GO:000	
Mon2p	Protein v	GO:00068	GO:00319	GO:0005085 guanyl-nucleotide exchange factor activity	
alpha-1,6	Subunit c	GO:00064	GO:00001	GO:00000	(sce)N-glycan biosynthesis, high-mannose typ
RNA exon	Protein i	GO:00905	GO:00056	GO:0004521 endoribonuclease activity;GO:0004527 exonu	
glycosyl	Alpha 1,2	GO:00062	GO:00057	GO:00045	(sce00563)Glycosylphosphatidylinositol (GPI)-
transcri	Meiosis-s	GO:00459	GO:00002	GO:00435	(sce04113)Meiosis - yeast
Rad14p	Protein t	GO:19012	GO:00001	GO:00036	(sce03420)Nucleotide excision repair
transpor	Componen	GO:00344	GO:19900	GO:0017112 Rab guanyl-nucleotide exchange factor acti	
Fe-S clu	Essential	GO:00000	GO:00306	GO:0043024 ribosomal small subunit binding;GO:0003743	
putative	Putative	GO:00060	GO:00055	GO:0018456 aryl-alcohol dehydrogenase (NAD+) activity	
Erp3p	Protein v	GO:00066	GO:00301	GO:0003674 molecular_function	
Mg(2+) t	Plasma me	GO:00109	GO:00058	GO:0015095 magnesium ion transmembrane transporter ac	
RNA-proc	Subunit c	GO:00987	GO:00058	GO:00037	(sce03015)mRNA surveillance pathway
rRNA-pro	Componen	GO:00004	GO:00306	GO:0003674 molecular_function	
oleate-a	Autoregul	GO:00614	GO:00897	GO:0000978 RNA polymerase II proximal promoter sequen	
5-oxopro	5-oxopro	GO:00067	GO:00058	GO:00171	(sce00480)Glutathione metabolism
Slg1p	Sensor-t	GO:00302	GO:00433	GO:00048	(sce04139)Mitophagy - yeast; (sce04011)MAPK
Yip5p	Protein t	GO:00161	GO:00057	GO:0017137 Rab GTPase binding;GO:0017016 Ras GTPase b	
AAA fami	Mitochon	GO:00301	GO:00057	GO:0004222 metalloendopeptidase activity;GO:0008237 m	
Ecm15p	Non-esser	GO:00081	GO:00058	GO:0003674 molecular_function	
translat	Translat	GO:00340	GO:00162	GO:00313	(sce03013)RNA transport
None	None	None	None	None	None
CCR4-NOT	Heterotr	GO:00066	GO:00058	GO:0051082 unfolded protein binding;GO:0005515 protei	
Pex27p	Periphera	GO:00070	GO:00057	GO:0003674 molecular_function	
DNA repl	DNA repl	GO:19029	GO:00711	GO:00431	(sce04111)Cell cycle - yeast; (sce04113)Meio
pseudour	Pseudour	GO:00004	GO:00314	GO:00099	(sce03008)Ribosome biogenesis in eukaryotes
proteaso	Beta 3 s	GO:00104	GO:00345	GO:00611	(sce03050)Proteasome
Rcy1p	F-box pr	GO:00344	GO:00001	GO:0000149 SNARE binding;GO:0005515 protein binding;G	
None	None	None	None	None	None
Tri1p	Non-esser	GO:00081	GO:00057	GO:0003674 molecular_function	
Mhf2p	Componen	GO:00007	GO:00718	GO:0046982 protein heterodimerization activity;GO:000	
Psy4p	Regulato	GO:20000	GO:00302	GO:0019888 protein phosphatase regulator activity;GO:	
glycerol-	Mitochon	GO:00061	GO:00313	GO:00525	(sce00564)Glycerophospholipid metabolism; (s

carbamoyl	Large sul	GO:00065	GO:00059	GO:00040	(sce)Uridine monophosphate biosynthesis, glu
Atc1p	Nuclear	GO:00071	GO:00056	GO:0003674	molecular_function
putative	Componen	GO:00004	GO:00056	GO:0003755	peptidyl-prolyl cis-trans isomerase activi
Nar1p	Subunit	GO:00162	GO:00973	GO:0008901	ferredoxin hydrogenase activity;GO:0016699
aspartyl	Cytoplasm	GO:00065	GO:00003	GO:0070006	metalloaminopeptidase activity;GO:0004177
Gpr1p	Plasma m	GO:00097	GO:00058	GO:00055	(sce04213)Longevity regulating pathway - mul
glycine	Cytosoli	GO:00192	GO:00433	GO:00709	(sce)C1-unit interconversion, eukaryotes; (M
Csm2p	Componen	GO:00007	GO:00971	GO:0003674	molecular_function
hypotheti	Protein	GO:00081	GO:00160	GO:0003674	molecular_function
riboscma	Protein	GO:00004	GO:00226	GO:00037	(sce03010)Ribosome
MRX comp	Subunit	GO:00320	GO:00308	GO:00040	(sce03450)Non-homologous end-joining; (sce03
ATP-bind	Putative	GO:00353	GO:00057	GO:0008559	xenobiotic transmembrane transporting ATPa
None	None	None	None	None	None
Wwm1p	WW domai	GO:00069	GO:00057	GO:0003674	molecular_function
Mks1p	Pleiotro	GO:20002	GO:00056	GO:0003674	molecular_function
Aha1p	Co-chape	GO:00327	GO:00057	GO:0001671	ATPase activator activity;GO:0060590 ATPas
None	None	None	None	None	None
Mcm16p	Componen	GO:00340	GO:00007	GO:0003674	molecular_function
None	None	None	None	None	None
N-acetyl	Componen	GO:00064	GO:00435	GO:00045	(sce)N-glycan precursor biosynthesis; (M0005
Csn9p	Subunit	GO:00007	GO:00081	GO:0003674	molecular_function
chorisma	Chorisma	GO:00090	GO:00056	GO:00041	(M00024)Phenylalanine biosynthesis, chorisma
riboflav	Riboflav	GO:00092	GO:00055	GO:00047	(sce00740)Riboflavin metabolism; (sce01110)B
mannosyl	Subunit	GO:00000	GO:00001	GO:00000	(sce)N-glycan biosynthesis, high-mannose typ
Hcm1p	Forkhead	GO:00513	GO:00007	GO:0000981	DNA-binding transcription factor activity,
Hms2p	Protein	GO:00614	GO:00056	GO:0000978	RNA polymerase II proximal promoter sequen
None	None	None	None	None	None
isocitra	Isocitra	GO:00060	GO:00057	GO:00464	(M00012)Glyoxylate cycle; (sce)Glyoxylate cy
Ypp1p	Cargo-tr	GO:00066	GO:00304	GO:0043022	ribosome binding;GO:0043021 ribonucleoprot
sulfite	Sulfite	GO:00193	GO:00093	GO:00503	(M00176)Assimilatory sulfate reduction, sulf
bifuncti	Serine-t	GO:00362	GO:19906	GO:00045	(sce04141)Protein processing in endoplasmic
FMN aden	Flavin a	GO:00067	GO:00057	GO:00039	(sce00740)Riboflavin metabolism; (sce01110)B
Avt4p	Vacuolar	GO:00329	GO:19908	GO:00151	(sce04138)Autophagy - yeast
Atg38p	Homodime	GO:00162	GO:00342	GO:00051	(sce04138)Autophagy - yeast
oxystero	Protein	GO:00347	GO:00003	GO:0008142	oxysterol binding;GO:0015248 sterol transp
None	None	None	None	None	None
Tim12p	Essentia	GO:00450	GO:00427	GO:0008320	protein transmembrane transporter activity
She9p	Protein	GO:00070	GO:00057	GO:0003674	molecular_function
chitin s	Chitin s	GO:00342	GO:00450	GO:00041	(sce00520)Amino sugar and nucleotide sugar m
RNA exon	RNA exon	GO:00344	GO:00056	GO:0000175	3'-5'-exoribonuclease activity;GO:0016896
Mdm1p	PtdIns-3-	GO:19908	GO:00715	GO:0032266	phosphatidylinositol-3-phosphate binding;G
Iwr1p	RNA poly	GO:00066	GO:00056	GO:0005515	protein binding;GO:0005488 binding;GO:0003
Ncw2p	Structur	GO:00315	GO:00092	GO:0005199	structural constituent of cell wall;GO:000
mitophag	Mitochon	GO:00319	GO:00313	GO:00036	(sce04139)Mitophagy - yeast
bifuncti	Glycerol-	GO:00160	GO:00301	GO:00162	(sce00561)Glycerolipid metabolism; (sce00564
Hap1p	Zinc fin	GO:00614	GO:00057	GO:0000978	RNA polymerase II proximal promoter sequen
Mon1p	Subunit	GO:00443	GO:00325	GO:00103	(sce04138)Autophagy - yeast
tubulin-	Subunit	GO:00329	GO:00162	GO:0051087	chaperone binding;GO:0051082 unfolded prot
ketol-ac	Acetohyd	GO:00090	GO:00426	GO:00044	(sce)Valine/isoleucine biosynthesis, pyruvat
Slk19p	Kinetoch	GO:00986	GO:00007	GO:00036	(sce04111)Cell cycle - yeast; (sce04113)Meio
chromati	S-phase	GO:00484	GO:00312	GO:00036	(sce04111)Cell cycle - yeast
Chl1p	Probable	GO:00340	GO:00007	GO:0004003	ATP-dependent DNA helicase activity;GO:000
Mak31p	Non-cata	GO:00064	GO:00314	GO:0004596	peptide alpha-N-acetyltransferase activity
Ent4p	Protein	GO:00001	GO:00304	GO:0030276	clathrin binding;GO:0005515 protein bindin

glucosami	Protein i	GO:00065	GO:00057	GO:00322	(sce)GPI-anchor biosynthesis, core oligosacch
rRNA-pro	Endonucl	GO:00004	GO:00306	GO:0004519 endonuclease activity;GO:0004518 nuclease	
putative	Putative	GO:00156	GO:00057	GO:0052851 ferric-chelate reductase (NADPH) activity;	
None	None	None	None	None	None
5-aminol	5-aminol	GO:00067	GO:00057	GO:00038	(sce00860)Porphyrin and chlorophyll metaboli
thiosulf	Rhodanes	GO:00021	GO:00057	GO:00047	(sce04122)Sulfur relay system; (sce00920)Sul
Tos6p	Glycosyl	GO:00081	GO:00092	GO:0003674 molecular_function	
Sng1p	Protein i	GO:00610	GO:00003	GO:0003674 molecular_function	
serine/t	Ser/Thr	GO:19033	GO:00057	GO:0004674 protein serine/threonine kinase activity;G	
Rab fami	Stress-i	GO:00068	GO:00057	GO:0003924 GTPase activity;GO:0005525 GTP binding;GO:	
hypothet	Putative	GO:00081	GO:00055	GO:0003674 molecular_function	
Pho88p	Protein involved in SRP-independent targeting of substrates to the ER; component				
Ran guan	Nucleoti	GO:00064	GO:00007	GO:0005087 Ran guanyl-nucleotide exchange factor acti	
hypothet	Putative	GO:00081	GO:00057	GO:0003674 molecular_function	
Atg39p	Autophag	GO:00617	GO:00340	GO:0030674 protein binding, bridging;GO:0060090 molec	
Rrp9p	Protein i	GO:00001	GO:00314	GO:0034511 U3 snoRNA binding;GO:0030515 snoRNA bindin	
putative	Putative protein of unknown function; similar to phosphoserine phosphatases; gre				
exportin	Major ka	GO:00000	GO:00058	GO:00050	(sce03008)Ribosome biogenesis in eukaryotes;
ATP-depe	Mitochon	GO:00320	GO:00057	GO:0043140 ATP-dependent 3'-5' DNA helicase activity;	
Lhp1p	RNA bind	GO:00080	GO:00057	GO:0000049 tRNA binding;GO:0003723 RNA binding;GO:000	
Bub3p	Kinetoch	GO:00070	GO:19902	GO:00431	(sce04111)Cell cycle - yeast
Ssh1p	Subunit	GO:00066	GO:00712	GO:00083	(sce03060)Protein export; (sce04145)Phagosom
drug-res	Transcri	GO:20010	GO:00056	GO:0000978 RNA polymerase II proximal promoter sequen	
histone	Subunit	GO:00165	GO:00327	GO:0004402 histone acetyltransferase activity;GO:0061	
Fir1p	Protein i	GO:00063	GO:0005935 cellular bud neck;GO:0005933 cellular bud;GO:003042		
hypothet	Putative	GO:00081	GO:00160	GO:0003674 molecular_function	
hypothet	Protein	GO:00421	GO:00055	GO:0004866 endopeptidase inhibitor activity;GO:006113	
Trs65p	Componen	GO:00512	GO:19900	GO:0017112 Rab guanyl-nucleotide exchange factor acti	
Mdm32p	Mitochon	GO:00483	GO:00057	GO:0003674 molecular_function	
exosome	Exosome	GO:00710	GO:00001	GO:00170	(sce03018)RNA degradation
Isd11p	Cysteine	GO:00162	GO:19902	GO:0031071 cysteine desulfurase activity;GO:0016783 s	
Sov1p	Mitochon	GO:00081	GO:00057	GO:0003674 molecular_function	
ubiquiti	Subunit	GO:00719	GO:00319	GO:00431	(sce04136)Autophagy - other; (sce04213)Longe
Mte1p	Protein	GO:00510	GO:00358	GO:0003674 molecular_function	
DNA-bind	5'- and	GO:00007	GO:00056	GO:0070338 5'-flap-structured DNA binding;GO:0070337	
protein-	Arginine	GO:00464	GO:00056	GO:0019702 protein-arginine N5-methyltransferase acti	
hypothet	Putative	GO:00081	GO:00160	GO:0003674 molecular_function	
Npl6p	Componen	GO:00063	GO:00165	GO:0015616 DNA translocase activity;GO:0008094 DNA-de	
syntaxin	Endosoma	GO:00068	GO:00312	GO:00054	(sce04130)SNARE interactions in vesicular tr
ribosoma	Ribosoma	GO:00321	GO:00226	GO:00302	(sce03010)Ribosome
Meh1p	Componen	GO:00070	GO:00719	GO:0003674 molecular_function	
Fmp41p	Putative	GO:00081	GO:00057	GO:00468	(Human)(hsa00350)Tyrosine metabolism; (hsa01
nucleosi	Nucleosi	GO:00092	GO:00056	GO:00088	(sce00230)Purine metabolism; (sce01100)Metab
tyrosine	Protein	GO:00106	GO:00059	GO:00047	(sce04011)MAPK signaling pathway - yeast; (s
Arf fami	Guanine	GO:00320	GO:00001	GO:00050	(sce04144)Endocytosis
tRNA A64-	Initiato	GO:00199	GO:00057	GO:0043399 tRNA A64-2'-O-ribosylphosphate transferase	
Spp1p	Subunit	GO:19033	GO:00481	GO:0042800 histone methyltransferase activity (H3-K4	
hypothet	Hydrophi	GO:00426	GO:00092	GO:0003674 molecular_function	
None	None	None	None	None	None
hypothet	Putative	GO:00081	GO:00055	GO:0003674 molecular_function	
None	None	None	None	None	None
Bol2p	Cytosoli	GO:00343	GO:00058	GO:0003674 molecular_function	
Heh2p	Inner nu	GO:00081	GO:00056	GO:0003674 molecular_function	
Fe-bindi	Protein	GO:00091	GO:00057	GO:0051537 2 iron, 2 sulfur cluster binding;GO:00515	

Cmr1p	Nuclear p	GO:20000(	GO:00343(	GO:0003677 DNA binding;GO:0003676 nucleic acid bindin	
pyridoxir	Protein i	GO:00086	GO:19036(	GO:003638	(sce00750)Vitamin B6 metabolism
asparagir	Mitochon(	GO:007014	GO:000575	GO:00048	(sce00970)Aminoacyl-tRNA biosynthesis
type 2C p	Mitochon(	GO:190418	GO:000575	GO:000474	(sce04139)Mitophagy - yeast
hypotheti	Protein (	GO:000815	GO:000557	GO:0003674 molecular_function	
1-phospha	1-phospha	GO:004685	GO:007077	GO:000028	(sce00562)Inositol phosphate metabolism; (sc
transloca	Essential	GO:004503	GO:004272	GO:0030943 mitochondrion targeting sequence binding;G	
trifuncti	Cytoplasm	GO:000925	GO:000573	GO:000447	(sce)C1-unit interconversion, eukaryotes; (M
Aim36p	Protein (	GO:000815	GO:003190	GO:0003674 molecular_function	
formin BN	Formin; r	GO:005101	GO:004333	GO:000552	(sce04011)MAPK signaling pathway - yeast
hypotheti	Putative	GO:000815	GO:000557	GO:0003674 molecular_function	
seripaupe	Member of	GO:003150	GO:000927	GO:0005199 structural constituent of cell wall;GO:000	
serine/th	Meiosis-s	GO:005159	GO:000563	GO:000468	(sce04113)Meiosis - yeast
Rab GTPas	Effector	GO:000689	GO:004333	GO:0019905 syntaxin binding;GO:0017137 Rab GTPase bin	
arginine-	Arginyl-t	GO:000642	GO:000573	GO:00048	(sce00970)Aminoacyl-tRNA biosynthesis
Hkr1p	Mucin fam	GO:000723	GO:003042	GO:000503	(sce04011)MAPK signaling pathway - yeast
Stn1p	Telomere	GO:00322	GO:199087	GO:0043047 single-stranded telomeric DNA binding;GO:0	
serine/th	DNA damag	GO:000920	GO:000582	GO:000368	(sce04111)Cell cycle - yeast; (sce04113)Meio
None	None	None	None	None	None
U2 snRNP	Component	GO:000024	GO:000568	GO:000367	(sce03040)Spliceosome
Spg5p	Protein r	GO:004324	GO:000557	GO:0070628 proteasome binding;GO:0044877 protein-cont	
3'-5'-exo	3'-->5' e	GO:000630	GO:000573	GO:0008296 3'-5'-exodeoxyribonuclease activity;GO:001	
dolichyl-	Zeta subu	GO:000648	GO:0034998 oligosa	(M00072)N-glycosylation by oligosaccharyltra	
None	None	None	None	None	None
Pga1p	Essential	GO:000650	GO:003150	GO:0000030 mannosyltransferase activity;GO:0016758 tr	
origin re	Subunit (	GO:000626	GO:000566	GO:000368	(sce04111)Cell cycle - yeast; (sce04113)Meio
hypotheti	Putative	GO:00086	GO:000563	GO:0004733 pyridoxamine-phosphate oxidase activity;GO	
mitochon(	Mitochon(	GO:007012	GO:000576	GO:0003735 structural constituent of ribosome;GO:0005	
cullin CI	Cullin; s	GO:003114	GO:001900	GO:003067	(sce04120)Ubiquitin mediated proteolysis; (s
Mme1p	Transport	GO:199061	GO:000574	GO:0015095 magnesium ion transmembrane transporter ac	
TFIIH/NEF	Subunit (	GO:007081	GO:000567	GO:000167	(sce03022)Basal transcription factors; (sce0
None	None	None	None	None	None
tetratri(	Essential	GO:003150	GO:000563	GO:0003674 molecular_function	
Aly2p	Alpha arr	GO:200039	GO:000577	GO:0030674 protein binding, bridging;GO:0031625 ubiqu	
Mdm20p	Non-catal	GO:001719	GO:003141	GO:0004596 peptide alpha-N-acetyltransferase activity	
RNA methy	Trimethyl	GO:001712	GO:000573	GO:007116	(sce03013)RNA transport
Slf1p	RNA bindi	GO:000687	GO:001049	GO:0003729 mRNA binding;GO:0003723 RNA binding;GO:000	
tRNA (gua	tRNA(m(1)	GO:007090	GO:000575	GO:0052906 tRNA (guanine(37)-N(1))-methyltransferase	
18S rRNA	Methyltra	GO:000047	GO:000588	GO:007003	(sce03008)Ribosome biogenesis in eukaryotes
U4/U6-U5	Unique co	GO:000038	GO:004654	GO:000367	(sce03040)Spliceosome
Arc18p	Subunit (	GO:003431	GO:000588	GO:005101	(sce04144)Endocytosis
histone (	Histone (	GO:000711	GO:000593	GO:0030332 cyclin binding;GO:0042393 histone binding;	
6-phospho	6-phospho	GO:000611	GO:000582	GO:000433	(sce00051)Fructose and mannose metabolism
mitochon(	Mitochon(	GO:003254	GO:000576	GO:000373	(sce03010)Ribosome
None	None	None	None	None	None
None	None	None	None	None	None
arginine-	Mitochon(	GO:007014	GO:000575	GO:00048	(sce00970)Aminoacyl-tRNA biosynthesis
Dgr1p	Protein (	GO:000815	GO:000573	GO:0003674 molecular_function	
None	None	None	None	None	None
mitochon(	Mitochon(	GO:003254	GO:000576	GO:0003735 structural constituent of ribosome;GO:0005	
glutamate	Gamma-glu	GO:005512	GO:000582	GO:000434	(M00015)Proline biosynthesis, glutamate => p
salt home	Serine/th	GO:000688	GO:001989	GO:000472	(sce03015)mRNA surveillance pathway; (sce041
Bol1p	Mitochon(	GO:004457	GO:000575	GO:0003674 molecular_function	
RNA-deper	Protein i	GO:000046	GO:000573	GO:0004004 ATP-dependent RNA helicase activity;GO:000	

mismatch	Protein i	GO:00071	GO:00323	GO:0000404	heteroduplex DNA loop binding;GO:0000405 b
transcri	Transcri	GO:00011	GO:00975	GO:00011	(sce03022)Basal transcription factors
serine/t	Dual-spec	GO:00304	GO:00055	GO:0004712	protein serine/threonine/tyrosine kinase a
Snu56p	Componen	GO:00083	GO:00056	GO:0003723	RNA binding;GO:0003676 nucleic acid bindin
putative	Putative	GO:00063	GO:00056	GO:0004386	helicase activity;GO:0016887 ATPase activi
Kha1p	Putative	GO:00718	GO:00057	GO:0015386	potassium:proton antiporter activity;GO:00
peptide a	Catalyti	GO:00171	GO:00314	GO:0004596	peptide alpha-N-acetyltransferase activity
Rrn9p	Protein i	GO:00427	GO:00005	GO:0001181	RNA polymerase I general transcription ini
phosphor	Phosphor	GO:00001	GO:00057	GO:0046	(sce)Tryptophan biosynthesis, chorismate =>
Usa1p	Scaffold	GO:00324	GO:00008	GO:00329	(sce04141)Protein processing in endoplasmic
Tsr4p	Cytoplasm	GO:00304	GO:00057	GO:0003674	molecular_function
putative	Putative	GO:00193	GO:00056	GO:00047	(sce00030)Pentose phosphate pathway
putative	Protein c	GO:00903	GO:00055	GO:0016888	endodeoxyribonuclease activity, producing
hypothet	Protein c	GO:00000	GO:00056	GO:0003676	nucleic acid binding;GO:1901363 heterocycl
Pac1p	Involved	GO:19030	GO:00058	GO:00510	(sce00565)Ether lipid metabolism; (sce01100)
Cmg1p	Putative	GO:00081	GO:00007	GO:0003676	nucleic acid binding;GO:1901363 heterocycl
aconitat	Putative	GO:00198	GO:00057	GO:00039	(sce)Lysine biosynthesis, 2-oxoglutarate =>
serine/t	Protein k	GO:00002	GO:00004	GO:0016887	ATPase activity;GO:0004674 protein serine/
ribosoma	Ribosoma	GO:00021	GO:00226	GO:00037	(sce03010)Ribosome
Pug1p	Plasma me	GO:00353	GO:00003	GO:0003674	molecular_function
COMPASS s	Subunit c	GO:00063	GO:00481	GO:0042800	histone methyltransferase activity (H3-K4
Mrx4p	Protein t	GO:00081	GO:00319	GO:0003674	molecular_function
protein t	Essentia	GO:00301	GO:00057	GO:0030943	mitochondrion targeting sequence binding;G
putative	Putative	GO:00353	GO:00057	GO:0004725	protein tyrosine phosphatase activity;GO:0
ATPase-b	Protein i	GO:00327	GO:00057	GO:0042030	ATPase inhibitor activity;GO:0060590 ATPas
rRNA met	Histone g	GO:00004	GO:00314	GO:19902	(sce03008)Ribosome biogenesis in eukaryotes
Cbp4p	Mitochon	GO:00345	GO:00325	GO:0003674	molecular_function
Yet2p	Protein c	GO:00709	GO:00057	GO:0043022	ribosome binding;GO:0043021 ribonucleoprot
gamma-gl	Gamma-gl	GO:00067	GO:00056	GO:0061928	glutathione specific gamma-glutamylcyclotr
J-type c	Speciali	GO:00445	GO:00057	GO:0001671	ATPase activator activity;GO:0060590 ATPas
None	None	None	None	None	None
protein s	Carboxy-t	GO:00709	GO:00058	GO:0008420	RNA polymerase II CTD heptapeptide repeat
ribosoma	Ribosoma	GO:00021	GO:00226	GO:00037	(sce03010)Ribosome
Cnm67p	Componen	GO:00304	GO:0005824 outer plaque of spindle pole body;GO:0005816 spindl		
hypothet	Putative protein of unknown function; mCherry fusion protein localizes to the va				
None	None	None	None	None	None
deoxycyt	Deoxycyt	GO:00062	GO:00057	GO:00041	(sce00240)Pyrimidine metabolism; (sce01100)M
None	None	None	None	None	None
hypothet	Putative	GO:00081	GO:00160	GO:0003674	molecular_function
TFIIH/NE	Subunit c	GO:00708	GO:00056	GO:00103	(sce03022)Basal transcription factors; (sce0
Art5p	Protein p	GO:00700	GO:00058	GO:0031625	ubiquitin protein ligase binding;GO:004438
Yae1p	Protein t	GO:01060	GO:00056	GO:0030674	protein binding, bridging;GO:0060090 molec
rhomboid	Mitochon	GO:00064	GO:00057	GO:0004252	serine-type endopeptidase activity;GO:0008
Pdx1p	E3-bindi	GO:00060	GO:00059	GO:0016746	transferase activity, transferring acyl gr
None	None	None	None	None	None
ATP-depe	Mitochon	GO:19027	GO:00057	GO:1990400	mitochondrial ribosomal large subunit rRNA
ribosoma	Protein c	GO:00004	GO:00226	GO:00701	(sce03010)Ribosome
proline--	Prolyl-t	GO:00064	GO:00171	GO:00048	(sce00970)Aminoacyl-tRNA biosynthesis
Spo20p	Meiosis-s	GO:00313	GO:00056	GO:0005546	phosphatidylinositol-4,5-bisphosphate bind
Arf fami	Ssh1p-Ss	GO:00312	GO:00712	GO:00154	(sce03060)Protein export; (sce04145)Phagosom
hypothet	Putative	GO:00081	GO:00160	GO:0003674	molecular_function
Gyp1p	Cis-golg	GO:00906	GO:00057	GO:0017137	Rab GTPase binding;GO:0005096 GTPase activ
Yos9p	ER quali	GO:00974	GO:00008	GO:00704	(sce04141)Protein processing in endoplasmic
hypothet	Putative	GO:00081	GO:00055	GO:0003674	molecular_function

Emp65p	Integral	GO:00354	GO:00301	GO:0003674 molecular_function	
hypotheti	Non-esser	GO:00081	GO:00055	GO:0003674 molecular_function	
Hmo1p	Chromatir	GO:00011	GO:00335	GO:0000400 four-way junction DNA binding;GO:0008301 D	
myosin 4	Type V my	GO:00075	GO:00319	GO:0000146 microfilament motor activity;GO:0003774 mo	
Apl3p	Alpha-ada	GO:00725	GO:00301	GO:00356	(sce04144)Endocytosis
GPI-ancho	ER membra	GO:00162	GO:00427	GO:00039	(sce00563)Glycosylphosphatidylinositol (GPI)-
Cto1p	Protein i	GO:00068	GO:00055	GO:0042131 thiamine phosphate phosphatase activity;GO	
Coa4p	Twin Cx(9	GO:00336	GO:00057	GO:0003674 molecular_function	
Num1p	Protein i	GO:00309	GO:00059	GO:0005543 phospholipid binding;GO:0008289 lipid bind	
Srb8p	Subunit c	GO:00004	GO:00165	GO:0003713 transcription coactivator activity;GO:0003	
Avo1p	Componen	GO:00382	GO:00319	GO:0005546 phosphatidylinositol-4,5-bisphosphate bind	
Cue2p	Protein c	GO:00081	GO:00055	GO:0043130 ubiquitin binding;GO:0032182 ubiquitin-lik	
cation tr	Plasma me	GO:00600	GO:00058	GO:0015238 drug transmembrane transporter activity;GO	
Fyv5p	Protein i	GO:00007	GO:00056	GO:0003674 molecular_function	
Vps75p	NAP famil	GO:00350	GO:00007	GO:0010698 acetyltransferase activator activity;GO:00	
Emc10p	Putative	GO:00081	GO:00057	GO:0003674 molecular_function	
sterol tr	Sterol tr	GO:00323	GO:00003	GO:0003674 molecular_function	
B-type cy	B-type cy	GO:00106	GO:00003	GO:00165	(sce04011)MAPK signaling pathway - yeast; (s
transcrip	Subunit c	GO:00427	GO:00001	GO:0001002 RNA polymerase III type 1 promoter sequenc	
Golgi tra	Componen	GO:00322	GO:00171	GO:0003674 molecular_function	
U2 snRNP	Protein i	GO:00002	GO:00056	GO:00036	(sce03040)Spliceosome
Spc97p	Componen	GO:00514	GO:00009	GO:0051011 microtubule minus-end binding;GO:0043015 g	
type 2C p	Cytoplasm	GO:00064	GO:00057	GO:0004724 magnesium-dependent protein serine/threoni	
seripaupe	Member of	GO:00315	GO:00092	GO:0005199 structural constituent of cell wall;GO:000	
Rsf1p	Protein i	GO:00063	GO:00057	GO:0003674 molecular_function	
protein k	Protein s	GO:00321	GO:00321	GO:00046	(sce04933)AGE-RAGE signaling pathway in diab
Sps22p	Protein c	GO:00304	GO:00092	GO:0003674 molecular_function	
RNA bindi	RNA bindi	GO:00004	GO:00314	GO:00306	(sce03040)Spliceosome; (sce03008)Ribosome bi
arginine	Plasma me	GO:19038	GO:00321	GO:0015181 arginine transmembrane transporter activit	
Esc2p	Sumo-like	GO:00332	GO:00056	GO:0003674 molecular_function	
Atg40p	Autophagy	GO:00617	GO:00325	GO:0030674 protein binding, bridging;GO:0060090 molec	
protein c	Componen	GO:00450	GO:00057	GO:0030943 mitochondrion targeting sequence binding;G	
hypotheti	Putative	GO:00081	GO:00055	GO:0003674 molecular_function	
Pcl8p	Cyclin; i	GO:00457	GO:00003	GO:0016538 cyclin-dependent protein serine/threonine	
hypotheti	Putative	GO:00081	GO:00160	GO:0003674 molecular_function	
H/ACA sno	Protein c	GO:00004	GO:00314	GO:00345	(sce03008)Ribosome biogenesis in eukaryotes
ribosoma	Protein c	GO:00021	GO:00104	GO:00037	(sce03010)Ribosome
None	None	None	None	None	None
COP9 sigr	Catalytic	GO:00704	GO:00081	GO:0019784 NEDD8-specific protease activity;GO:001978	
exocyst s	Exocyst s	GO:00905	GO:00056	GO:0003674 molecular_function	
None	None	None	None	None	None
hypotheti	Dubious c	GO:00081	GO:00055	GO:0003674 molecular_function	
Nbp1p	Spindle p	GO:00717	GO:00058	GO:0008289 lipid binding;GO:0005488 binding;GO:000367	
None	None	None	None	None	None
bifuncti	UDP-gluco	GO:00334	GO:00058	GO:00039	(sce)Galactose degradation, Leloir pathway,
None	None	None	None	None	None
Utp14p	Subunit c	GO:00004	GO:00320	GO:00055	(sce03008)Ribosome biogenesis in eukaryotes
dolichyl-	Dolichyl-	GO:00064	GO:00057	GO:01060	(sce)N-glycan precursor biosynthesis; (M0005
Yos1p	Integral	GO:00068	GO:00301	GO:0003674 molecular_function	
None	None	None	None	None	None
hypotheti	Putative	GO:00081	GO:00055	GO:0003674 molecular_function	
peptide a	Subunit c	GO:00064	GO:00314	GO:0004596 peptide alpha-N-acetyltransferase activity	
None	None	None	None	None	None
autophagy	Adapter p	GO:00617	GO:19903	GO:00329	(sce04136)Autophagy - other; (sce04139)Mitop

hypotheti	Protein (	GO:00081	GO:00058	GO:0043022 ribosome binding;GO:0043021 ribonucleoprot	
hypotheti	Putative	GO:00081	GO:00055	GO:0003674 molecular_function	
Aim11p	Protein (	GO:00081	GO:00057	GO:0003674 molecular_function	
CCR4-NOT	Componen	GO:00310	GO:00300	GO:00048	(sce03018)RNA degradation
putative	Protein (	GO:19905	GO:00306	GO:0005458 GDP-mannose transmembrane transporter acti	
Kel1p	Protein	GO:00903	GO:19906	GO:0003674 molecular_function	
Duf1p	Ubiquiti	GO:19030	GO:00057	GO:0043130 ubiquitin binding;GO:0032182 ubiquitin-lik	
hypotheti	Putative	GO:00081	GO:00055	GO:0003674 molecular_function	
bifuncti	Ethanola	GO:00066	GO:00057	GO:00043	(sce)Phosphatidylethanolamine (PE) biosynthe
dolichyl-	Alpha su	GO:00182	GO:00057	GO:00045	(M00072)N-glycosylation by oligosaccharyltra
Pml39p	Protein	GO:00512	GO:00319	GO:0043022 ribosome binding;GO:0043021 ribonucleoprot	
tRNA lig	tRNA lig	GO:00320	GO:00056	GO:0051730 GTP-dependent polyribonucleotide 5'-hydrox	
She1p	Mitotic	GO:20005	GO:00058	GO:0008017 microtubule binding;GO:0015631 tubulin bin	
origin r	Subunit (	GO:00319	GO:00056	GO:00036	(sce04111)Cell cycle - yeast; (sce04113)Meio
Arf fami	ADP-ribo	GO:00068	GO:00057	GO:00039	(sce04144)Endocytosis
Hal9p	Putative	GO:00096	GO:00057	GO:0000981 DNA-binding transcription factor activity,	
tRNA:m4X	2'-O-met	GO:00304	GO:00056	GO:0106050 tRNA 2'-O-methyltransferase activity;GO:00	
Inp1p	Periphera	GO:00450	GO:00057	GO:0030674 protein binding, bridging;GO:0060090 molec	
DEAD-box	RNA heli	GO:00003	GO:00056	GO:00081	(sce03040)Spliceosome
tRNA 1-m	Subunit (	GO:00304	GO:00315	GO:0016429 tRNA (adenine-N1-)-methyltransferase activ	
Mms1p	Subunit (	GO:00706	GO:00353	GO:0003674 molecular_function	
Bni4p	Targetin	GO:00060	GO:00321	GO:0005515 protein binding;GO:0005488 binding;GO:0003	
Mdy2p	Protein	GO:00007	GO:00723	GO:0043023 ribosomal large subunit binding;GO:0043024	
Rup1p	Protein	GO:19024	GO:00056	GO:0005515 protein binding;GO:0005488 binding;GO:0003	
None	None	None	None	None	None
hypotheti	Protein (	GO:00512	GO:00057	GO:0003674 molecular_function	
ribose p	5-phosph	GO:00060	GO:00021	GO:00047	(M00005)PRPP biosynthesis, ribose 5P => PRPP
Erj5p	Type I m	GO:00064	GO:00057	GO:0003674 molecular_function	
Tah1p	Componen	GO:00004	GO:00972	GO:0051087 chaperone binding;GO:0005515 protein bindi	
putative	Positive	GO:00064	GO:00226	GO:0016887 ATPase activity;GO:0017111 nucleoside-trip	
dihydrox	Dihydrox	GO:00090	GO:00057	GO:00041	(sce)Valine/isoleucine biosynthesis, pyruvat
hydrolas	Steryl-b	GO:19044	GO:00003	GO:0050295 steryl-beta-glucosidase activity;GO:000842	
Tir3p	Cell wal	GO:00315	GO:00092	GO:0005199 structural constituent of cell wall;GO:000	
putative	Putative	GO:00081	GO:00057	GO:0034459 ATP-dependent 3'-5' RNA helicase activity;	
Nfs1p	Cysteine	GO:00709	GO:19902	GO:00310	(sce04122)Sulfur relay system; (sce00730)Thi
chromati	ATPase s	GO:00070	GO:00364	GO:0000182 rDNA binding;GO:0031491 nucleosome binding	
Fun19p	Non-esse	GO:00350	GO:00704	GO:0004402 histone acetyltransferase activity;GO:0061	
protein	Putative	GO:0005979 regula		GO:0019888 protein phosphatase regulator activity;GO:	
Pex29p	ER-resid	GO:00325	GO:00057	GO:0003674 molecular_function	
Bik1p	Microtub	GO:00007	GO:00353	GO:0008017 microtubule binding;GO:0015631 tubulin bin	
mRNA-bin	Translat	GO:20007	GO:00104	GO:0045182 translation regulator activity;GO:0003697	
ATP-depe	Mitochon	GO:00063	GO:00057	GO:0033592 RNA strand annealing activity;GO:0004004 A	
None	None	None	None	None	None
pyruvate	E1 beta	GO:00060	GO:00059	GO:00047	(M00307)Pyruvate oxidation, pyruvate => acet
Som1p	Subunit (	GO:00331	GO:00427	GO:0004175 endopeptidase activity;GO:0070011 peptidas	
Kip2p	Kinesin-	GO:00070	GO:19037	GO:0008574 ATP-dependent microtubule motor activity,	
Cik1p	Kinesin-	GO:00007	GO:00058	GO:0008569 ATP-dependent microtubule motor activity,	
ribosoma	Protein (	GO:00021	GO:00226	GO:00037	(sce03010)Ribosome
None	None	None	None	None	None
Pex17p	Membrane	GO:00165	GO:19904	GO:0005515 protein binding;GO:0005488 binding;GO:0003	
Svl3p	Protein (	GO:00068	GO:00059	GO:0016491 oxidoreductase activity;GO:0003824 catalyt	
hypotheti	Putative	GO:00081	GO:00056	GO:0003674 molecular_function	
None	None	None	None	None	None
hypotheti	Putative	GO:00081	GO:00055	GO:0003674 molecular_function	

purine-cy	Purine-cy	GO:00158	GO:00003	GO:0015212	cytidine transmembrane transporter activit
cytidine	Cytidine	GO:00062	GO:00058	GO:00041	(sce00240)Pyrimidine metabolism; (sce01100)M
Fhn1p	Protein	GO:00709	GO:00321	GO:0003674	molecular_function
Mak11p	Protein	GO:00004	GO:00319	GO:0003674	molecular_function
Sph1p	Protein	GO:00313	GO:19027	GO:0005078	MAP-kinase scaffold activity;GO:0035591 si
E3 ubiqu	E3 ubiqu	GO:00422	GO:00975	GO:0043142	single-stranded DNA-dependent ATPase activ
glycosyl	Glycosyl	GO:00065	GO:19905	GO:00000	(sce)GPI-anchor biosynthesis, core oligosacc
hypothet	Putative	GO:00068	GO:00301	GO:0003674	molecular_function
ribosyln	Nicotinam	GO:00343	GO:00003	GO:00502	(sce00760)Nicotinate and nicotinamide metabo
hypothet	Putative	GO:00081	GO:00055	GO:0003674	molecular_function
hypothet	Putative	GO:00157	GO:00003	GO:0005366	myo-inositol:proton symporter activity;GO:
None	None	None	None	None	None
amino ac	Protein	GO:00158	GO:00003	GO:00036	(Human)(hsa04142)Lysosome
Nmd4p	Protein	GO:00001	GO:00056	GO:0003674	molecular_function
decappin	Nuclear	GO:00710	GO:00058	GO:1990174	phosphodiesterase decapping endonuclease a
None	None	None	None	None	None
hypothet	Putative	GO:00081	GO:00055	GO:0003674	molecular_function
Mps2p	Essentia	GO:00719	GO:00058	GO:0005198	structural molecule activity;GO:0003674 mo
adenylat	CAP (cyc	GO:00308	GO:00304	GO:0008179	adenylate cyclase binding;GO:0003779 actin
None	None	None	None	None	None
Brp1p	Putative	GO:00081	GO:00055	GO:0003674	molecular_function
Mpa43p	Protein	GO:00059	GO:00057	GO:0016773	phosphotransferase activity, alcohol group
TATA-bin	TATA-bin	GO:00011	GO:00056	GO:00011	(sce03022)Basal transcription factors
None	None	None	None	None	None
hypothet	Putative	GO:00081	GO:00160	GO:0003674	molecular_function
Rrp7p	Essentia	GO:00000	GO:00344	GO:00036	(sce03008)Ribosome biogenesis in eukaryotes
hypothet	Putative	GO:00081	GO:00057	GO:0003674	molecular_function
None	None	None	None	None	None
Ypq2p	Putative	GO:00344	GO:00716	GO:0015174	basic amino acid transmembrane transporter
syntaxin	Plasma m	GO:00313	GO:00056	GO:00703	(sce04130)SNARE interactions in vesicular tr
SPX doma	Low-affi	GO:20001	GO:00058	GO:0005315	inorganic phosphate transmembrane transpor
Sus1p	Componen	GO:00710	GO:00001	GO:0003713	transcription coactivator activity;GO:0008
SNAP rec	Essentia	GO:00482	GO:00714	GO:00054	(sce04130)SNARE interactions in vesicular tr
Tpn1p	Plasma m	GO:00319	GO:00003	GO:0031924	vitamin B6 transmembrane transporter activ
hypothet	Putative	GO:00356	GO:00003	GO:0035673	oligopeptide transmembrane transporter act
Mhf1p	Componen	GO:00007	GO:00718	GO:0046982	protein heterodimerization activity;GO:000
seripaup	Protein	GO:00315	GO:00092	GO:0005199	structural constituent of cell wall;GO:000
pseudour	Mitochon	GO:00004	GO:00057	GO:0106029	tRNA pseudouridine synthase activity;GO:00
None	None	None	None	None	None
Hut1p	Protein	GO:00157	GO:00301	GO:0005460	UDP-glucose transmembrane transporter acti
Jjj3p	Protein	GO:00171	GO:00056	GO:0008198	ferrous iron binding;GO:0005506 iron ion b
Far11p	Protein	GO:00003	GO:00001	GO:0003674	molecular_function
Ran GTPa	Nuclear	GO:00066	GO:00446	GO:0008536	Ran GTPase binding;GO:0008565 protein tran
crucifor	Mitochon	GO:00000	GO:00057	GO:0000402	crossed form four-way junction DNA binding
alpha-1,	Alpha1,2-	GO:00000	GO:00003	GO:00000	(sce00514)Other types of O-glycan biosynthes
phosphoi	Phosphat	GO:00468	GO:00304	GO:00438	(sce00562)Inositol phosphate metabolism; (sc
kinetoch	Componen	GO:00070	GO:0031262 Ndc80 complex;GO:0000778 condensed nuclear chromoso		
None	None	None	None	None	None
methioni	Methiony	GO:00064	GO:00171	GO:00048	(sce00450)Selenocompound metabolism; (sce009
fatty ac	Mitochon	GO:00066	GO:00057	GO:00043	(sce)Fatty acid biosynthesis, elongation; (M
hypothet	Protein	GO:00081	GO:00055	GO:0003674	molecular_function
oxystero	One of s	GO:00356	GO:00001	GO:0008142	oxysterol binding;GO:0070273 phosphatidyli
Fmp32p	Putative	GO:00336	GO:00057	GO:0003674	molecular_function
Smc5-Smc	Componen	GO:00063	GO:00309	GO:0019789	SUMO transferase activity;GO:0004842 ubiqu

ribosomal	Ribosomal	GO:00004	GO:00306	GO:00198	(sce03010)Ribosome
acid phos	Acid phos	GO:00323	GO:00056	GO:0003993 acid phosphatase activity;GO:0016791 phosp	
None	None	None	None	None	None
hypotheti	Putative	GO:00081	GO:00160	GO:0047372 acylglycerol lipase activity;GO:0016298 li	
profilin	Profilin;	GO:00903	GO:00198	GO:0005546 phosphatidylinositol-4,5-bisphosphate bind	
hypotheti	Protein	GO:00081	GO:00057	GO:0003674 molecular_function	
mRNA-bind	Poly(A+)	GO:20008	GO:00710	GO:0003729 mRNA binding;GO:0003723 RNA binding;GO:000	
chaperoni	Subunit	GO:00064	GO:00058	GO:0051082 unfolded protein binding;GO:0005524 ATP bi	
New1p	ATP bindi	GO:00169	GO:00306	GO:0016887 ATPase activity;GO:0003729 mRNA binding;GO	
lipoyl(oc	Lipoyl li	GO:00092	GO:00057	GO:01025	(sce00785)Lipoic acid metabolism; (sce01100)M
Hop1p	Meiosis-s	GO:00515	GO:00008	GO:00004	(sce04113)Meiosis - yeast
Abp1p	Actin-bin	GO:20006	GO:00304	GO:0051015 actin filament binding;GO:0003779 actin bi	
ribosomal	Ribosomal	GO:00021	GO:00226	GO:00037	(sce03010)Ribosome
Dml1p	Essential	GO:00062	GO:00057	GO:0003674 molecular_function	
Sps2p	Protein	GO:00304	GO:00092	GO:0003674 molecular_function	
hypotheti	Putative	GO:00081	GO:00056	GO:0003674 molecular_function	
hypotheti	Putative	GO:00065	GO:00003	GO:00046	(sce00563)Glycosylphosphatidylinositol (GPI)-
ammonium	Ammonium	GO:00724	GO:00058	GO:0008519 ammonium transmembrane transporter activit	
glucose-i	Subunit	GO:00457	GO:00346	GO:0003674 molecular_function	
Erv29p	Protein	(Human)G(	GO:00301	GO:0097020 COPII adaptor activity;GO:0030674 protein	
Spc19p	Essential	GO:00519	GO:00427	GO:0051010 microtubule plus-end binding;GO:0008017 mi	
Rad4p	Protein	GO:00062	GO:00001	GO:19901	(sce03420)Nucleotide excision repair
hypotheti	Putative	GO:00081	GO:00160	GO:0003674 molecular_function	
hypotheti	Putative	GO:00081	GO:00055	GO:0003674 molecular_function	
Lot5p	Protein	GO:00081	GO:00056	GO:0003674 molecular_function	
Kei1p	Component	GO:00066	GO:00709	GO:0070917 inositol phosphoceramide synthase regulato	
mitochond	Mitochond	GO:00325	GO:00057	GO:00198	(sce03010)Ribosome
Lds1p	Protein	GO:00304	GO:00056	GO:0003674 molecular_function	
None	None	None	None	None	None
None	None	None	None	None	None
aminophos	Aminophos	GO:00007	GO:00708	GO:0015662 ATPase activity, coupled to transmembrane	
stress-re	Stress-re	GO:00972	GO:00058	GO:00009	(sce04213)Longevity regulating pathway - mul
Rsc58p	Component	GO:00063	GO:00165	GO:0015616 DNA translocase activity;GO:0008094 DNA-de	
Scy1p	Putative	GO:00064	GO:00301	GO:0004672 protein kinase activity;GO:0016773 phospho	
ribosomal	Ribosomal	GO:00021	GO:00306	GO:00198	(sce03010)Ribosome
None	None	None	None	None	None
ribose ph	5-phospho	GO:00060	GO:00021	GO:00047	(M00005)PRPP biosynthesis, ribose 5P => PRPP
Tda5p	Putative	GO:00424	GO:00319	GO:0016491 oxidoreductase activity;GO:0003824 catalyt	
mRNA spli	Splicing	GO:00710	GO:00710	GO:00003	(sce03040)Spliceosome
hypotheti	Putative	GO:00081	GO:00160	GO:0003674 molecular_function	
Boi2p	Protein	GO:00071	GO:00059	GO:0005543 phospholipid binding;GO:0008289 lipid bind	
Gts1p	Protein	GO:00105	GO:00304	GO:0070491 repressing transcription factor binding;GO	
ribosomal	Ribosomal	GO:00321	GO:00226	GO:00302	(sce03010)Ribosome
myosin 5	One of tw	GO:20006	GO:00304	GO:0000146 microfilament motor activity;GO:0003774 mo	
putative	Probable	GO:00109	GO:00058	GO:0015095 magnesium ion transmembrane transporter ac	
None	None	None	None	None	None
alanine t	Alanine t	GO:00065	GO:00057	GO:00040	(sce00220)Arginine biosynthesis; (sce00250)A
Smc5-Smc6	Component	GO:00169	GO:00309	GO:0019789 SUMO transferase activity;GO:0019787 ubiqu	
None	None	None	None	None	None
phosphati	Phosphati	GO:00066	GO:00100	GO:00046	(M00093)Phosphatidylethanolamine (PE) biosyn
None	None	None	None	None	None
transcrip	Subunit	GO:00014	GO:00466	GO:0046982 protein heterodimerization activity;GO:000	
ribosomal	Protein	GO:00004	GO:00226	GO:00037	(sce03010)Ribosome
Rtc4p	Protein	GO:00081	GO:00056	GO:0003674 molecular_function	

hypotheti	Putative	GO:00199	GO:00057	GO:0035243	protein-arginine omega-N symmetric methylt
None	None	None	None	None	None
NADH-ubi	NADH:ubi	GO:00061	GO:00057	GO:0008137	NADH dehydrogenase (ubiquinone) activity;G
Lcl3p	Putative	GO:00903	GO:00057	GO:0004519	endonuclease activity;GO:0004518 nuclease
Mas1p	Beta sub	GO:00066	GO:00170	GO:0004222	metalloendopeptidase activity;GO:0008237 m
Spc105p	Subunit	GO:00070	GO:00009	GO:0008017	microtubule binding;GO:0015631 tubulin bin
origin r	Largest	GO:00333	GO:00056	GO:00036	(sce04111)Cell cycle - yeast; (sce04113)Meio
carnitin	Carnitin	GO:00094	GO:00058	GO:00040	(sce04146)Peroxisome
translat	One of t	GO:00064	GO:00104	GO:0003746	translation elongation factor activity;GO:
mRNA-bin	Ubiquiti	GO:00345	GO:19908	GO:00048	(sce04139)Mitophagy - yeast
Net1p	Core sub	GO:00310	GO:00308	GO:00001	(sce04111)Cell cycle - yeast
adenylat	Adenylat	GO:00061	GO:00057	GO:00040	(sce)Adenine ribonucleotide biosynthesis, IM
hypothet	Protein	GO:00081	GO:00057	GO:0003674	molecular_function
ribosoma	Ribosoma	GO:00321	GO:00226	GO:00302	(sce03010)Ribosome
alpha,al	Synthase	GO:00904	GO:00059	GO:00038	(sce00500)Starch and sucrose metabolism; (sc
ribosoma	Ribosoma	GO:00422	GO:00226	GO:00037	(sce03010)Ribosome
DNA heli	DNA heli	GO:00422	GO:00007	GO:0009378	four-way junction helicase activity;GO:000
mRNA-bin	Componen	GO:00004	GO:00705	GO:0003729	mRNA binding;GO:0003723 RNA binding;GO:000
Ybp1p	Protein	GO:00345	GO:00057	GO:0003674	molecular_function
ubiquiti	Ubiquiti	GO:00716	GO:00056	GO:0061630	ubiquitin protein ligase activity;GO:00616
putative	Mitochon	GO:00066	GO:00003	GO:0022857	transmembrane transporter activity;GO:0005
Age1p	ADP-ribo	GO:00068	GO:00058	GO:00050	(sce04144)Endocytosis
syntaxin	Plasma m	GO:00313	GO:00056	GO:00433	(sce04130)SNARE interactions in vesicular tr
putative	Protein	GO:00064	GO:00057	GO:00048	(sce00970)Aminoacyl-tRNA biosynthesis
phenylac	Phenylac	GO:00462	GO:00057	GO:01061	(sce00130)Ubiquinone and other terpenoid-qui
chromati	Chromati	GO:00714	GO:00466	GO:0070615	nucleosome-dependent ATPase activity;GO:00
Fsf1p	Putative	GO:00550	GO:00319	GO:0015075	ion transmembrane transporter activity;GO:
transloc	Swi2/Snf	GO:20010	GO:00007	GO:0032183	SUMO binding;GO:0008094 DNA-dependent ATPa
Ecm7p	Putative	GO:00715	GO:00003	GO:0003674	molecular_function
mitochon	Mitochon	GO:00325	GO:00057	GO:00037	(sce03010)Ribosome
Mlc1p	Essentia	GO:00009	GO:01201	GO:0032038	myosin II heavy chain binding;GO:0045159 m
dolichyl-	Mannosyl	GO:00064	GO:00057	GO:00529	(sce)N-glycan precursor biosynthesis; (M0005
mRNA spl	Core Sm	GO:00003	GO:00002	GO:00037	(sce04139)Mitophagy - yeast; (sce03040)Splic
ATP-bind	Mitochon	GO:00903	GO:00057	GO:00154	(Human)(hsa02010)ABC transporters
putative	Putative	GO:00070	GO:00056	GO:0003924	GTPase activity;GO:0005525 GTP binding;GO:
mitogen-	Signal t	GO:00715	GO:00433	GO:00047	(sce04011)MAPK signaling pathway - yeast
hypothet	Putative	GO:00081	GO:00058	GO:0003674	molecular_function
Rgr1p	Subunit	GO:00001	GO:00708	GO:0001093	TFIIB-class transcription factor binding;G
amidase	Amidase;	GO:00301	GO:00057	GO:0070773	protein-N-terminal glutamine amidohydrolas
Gpg1p	Proposed	GO:00014	GO:0005737 cytoplasm;GO:0005622 intracellular;GO:0044424 intra		
None	None	None	None	None	None
Vtc1p	Regulato	GO:00067	GO:00332	GO:0003729	mRNA binding;GO:0003723 RNA binding;GO:000
Hpm1p	AdoMet-d	GO:00420	GO:00056	GO:0018064	protein-histidine N-methyltransferase acti
Tma46p	Protein	GO:00021	GO:00058	GO:1905538	polysome binding;GO:0043021 ribonucleoprot
hypothet	Protein	GO:00071	GO:00059	GO:0003674	molecular_function
Pet130p	Protein	GO:00081	GO:00057	GO:0003674	molecular_function
DNA-dire	Catalyti	GO:19029	GO:00056	GO:00038	(sce03030)DNA replication; (sce00240)Pyrimid
Arc15p	Subunit	GO:00343	GO:00313	GO:00510	(sce04144)Endocytosis
tetherin	ATP-bind	GO:00514	GO:00332	GO:00350	(sce04138)Autophagy - yeast
epsin	Epsin-li	GO:00001	GO:00433	GO:00302	(sce04144)Endocytosis
Trs120p	Componen	GO:00344	GO:19900	GO:0017112	Rab guanyl-nucleotide exchange factor acti
hypothet	Putative	GO:00081	GO:00160	GO:0003674	molecular_function
hypothet	Putative	GO:00081	GO:00057	GO:0003674	molecular_function
Trm82p	Catalyti	GO:01060	GO:01061	GO:0008176	tRNA (guanine-N7-)-methyltransferase activ

hypotheti	Protein (	GO:00081	GO:00433	GO:0043022 ribosome binding;GO:0043021 ribonucleoprot	
homeodoma	Silenced	GO:00081	GO:00056	GO:00435	(sce04113)Meiosis - yeast
proteason	19S prot	GO:00727	GO:00345	GO:00036	(sce03440)Homologous recombination; (sce0305
Arp4p	Nuclear a	GO:00513	GO:00352	GO:0043140 ATP-dependent 3'-5' DNA helicase activity;	
cytoskele	Cytoskele	GO:00343	GO:00304	GO:0098748 endocytic adaptor activity;GO:0043130 ubiq	
Itc1p	Subunit (	GO:00460	GO:00007	GO:0003674 molecular_function	
hypotheti	ER-local	GO:00081	GO:00057	GO:0003674 molecular_function	
histone (	Cytoplasm	GO:00319	GO:00056	GO:00171	(sce00760)Nicotinate and nicotinamide metabo
oxysterol	Member o	GO:00356	GO:00325	GO:0008142 oxysterol binding;GO:0005545 1-phosphatidy	
Dal82p	Positive	GO:00010	GO:00056	GO:0043565 sequence-specific DNA binding;GO:0003700 D	
ATP-bind	Subunit (	GO:00159	GO:00057	GO:00053	(sce02010)ABC transporters; (sce04146)Peroxi
Tmn2p	Protein v	GO:00014	GO:00003	GO:0003674 molecular_function	
None	None	None	None	None	None
protein	Mitochon	GO:00159	GO:00057	GO:0004740 pyruvate dehydrogenase (acetyl-transferrin	
putative	Putative	GO:19026	GO:00306	GO:0004004 ATP-dependent RNA helicase activity;GO:000	
fumarate	Fumarate	GO:00464	GO:00057	GO:0016156 fumarate reductase (NADH) activity;GO:0000	
protein	Scaffold	GO:00457	GO:01200	GO:00302	(sce04138)Autophagy - yeast
Tmn3p	Protein v	GO:00014	GO:00301	GO:0003674 molecular_function	
Atg12p	Ubiquiti	GO:00617	GO:00342	GO:00197	(sce04136)Autophagy - other; (sce04138)Autop
Vac17p	Phosphop	GO:00000	GO:00715	GO:0043495 protein membrane anchor;GO:0005515 protein	
hypotheti	Putative	GO:00081	GO:00057	GO:0003674 molecular_function	
ribosoma	Ribosoma	GO:00004	GO:00226	GO:00037	(sce03010)Ribosome
Mtc4p	Protein (	GO:00081	GO:00058	GO:0003674 molecular_function	
Spo73p	Meiosis-	GO:00321	GO:00056	GO:0003674 molecular_function	
ESCRT-II	Componen	GO:00004	GO:00008	GO:00322	(sce04144)Endocytosis
Ldb7p	Componen	GO:00063	GO:00165	GO:0015616 DNA translocase activity;GO:0008094 DNA-de	
mRNA-bin	Nuclear	GO:19001	GO:00056	GO:0033204 ribonuclease P RNA binding;GO:0008097 5S r	
exosome	Exosome	GO:00710	GO:00001	GO:00170	(sce03018)RNA degradation
Vps72p	Htz1p-bi	GO:00434	GO:00008	GO:0042393 histone binding;GO:0005515 protein binding	
mitochon	Mitochon	GO:00304	GO:00057	GO:00198	(sce03010)Ribosome
translat	mRNA cap	GO:00001	GO:00162	GO:00988	(sce03013)RNA transport
Mms4p	Subunit (	GO:00105	GO:00484	GO:0008821 crossover junction endodeoxyribonuclease a	
CDP-diac	Phosphat	GO:00320	GO:00057	GO:00084	(sce00564)Glycerophospholipid metabolism; (s
crossove	Holliday	GO:00709	GO:00056	GO:1904162 5'-3' exodeoxyribonuclease activity involv	
Sec16p	COPII ve	GO:00708	GO:00125	GO:0043495 protein membrane anchor;GO:0005515 protein	
None	None	None	None	None	None
F1F0 ATP	Subunit a	GO:00159	GO:00002	GO:00469	(sce00190)Oxidative phosphorylation; (sce011
Slm4p	Componen	GO:00162	GO:00344	GO:0003674 molecular_function	
hypotheti	Protein (	GO:00194	GO:00058	GO:0004806 triglyceride lipase activity;GO:0016298 li	
glutamat	NAD(+)-d	GO:00195	GO:00057	GO:00043	(sce00430)Taurine and hypotaurine metabolism
Kar5p	Protein	GO:00482	GO:00319	GO:0003674 molecular_function	
casein k	Alpha' c	GO:00075	GO:00344	GO:00046	(sce04139)Mitophagy - yeast; (sce03008)Ribos
organic	Putative	GO:19038	GO:00057	GO:0005342 organic acid transmembrane transporter act	
Faf1p	Protein	GO:00004	GO:00057	GO:0003674 molecular_function	
ditrans,	Forms th	GO:00194	GO:19044	GO:00455	(sce00900)Terpenoid backbone biosynthesis; (
dynamin-	Dynamin-	GO:00163	GO:00325	GO:0003924 GTPase activity;GO:0005525 GTP binding;GO:	
SCF E2 u	Ubiquiti	GO:00311	GO:00190	GO:00616	(sce04120)Ubiquitin mediated proteolysis
Vma22p	Protein	GO:00070	GO:19908	GO:0051082 unfolded protein binding;GO:0005515 protei	
Gtb1p	Glucosid	GO:00064	GO:00171	GO:0004558 alpha-1,4-glucosidase activity;GO:0090599	
hypotheti	Putative	GO:00081	GO:00160	GO:0003674 molecular_function	
Tes1p	Peroxiso	GO:00066	GO:00057	GO:00162	(sce00062)Fatty acid elongation; (sce01040)B
mRNA spl	Core Sm	GO:00003	GO:00465	GO:00037	(sce03040)Spliceosome
None	None	None	None	None	None
Aim41p	Protein (	GO:00081	GO:00057	GO:0016884 carbon-nitrogen ligase activity, with glut	

acetyl-C	Acetylgl	GO:00065	GO:0057	GO:00040	(M00028)Ornithine biosynthesis, glutamate =>
None	None	None	None	None	None
Atg5p	Conserve	GO:00448	GO:01200	GO:00197	(sce04136)Autophagy - other; (sce04213)Longe
hypotheti	Dubious	GO:00081	GO:00055	GO:0003674 molecular_function	
Mps3p	Nuclear	GO:00007	GO:00058	GO:0043495 protein membrane anchor;GO:0005524 ATP bin	
Mum3p	Protein	GO:00304	GO:00160	GO:0016746 transferase activity, transferring acyl gr	
dolichyl-	Gamma su	GO:00352	GO:00082	GO:00045	(M00072)N-glycosylation by oligosaccharyltra
ribosoma	Ribosoma	GO:00000	GO:00226	GO:00037	(sce03010)Ribosome
metalloe	Metallop	GO:00714	GO:00198	GO:00042	(Human)(hsa05010)Alzheimer's disease
Plp2p	Protein	GO:00073	GO:00057	GO:0031683 G-protein beta/gamma-subunit complex bindi	
Gyp5p	GTPase-a	GO:00906	GO:00059	GO:0017137 Rab GTPase binding;GO:0005096 GTPase activ	
Sas3p	Histone	GO:00304	GO:19904	GO:0010485 H4 histone acetyltransferase activity;GO:0	
None	None	None	None	None	None
aminopho	Aminopho	GO:00007	GO:00708	GO:0004012 phospholipid-translocating ATPase activity	
Csm1p	Nucleola	GO:00345	GO:00335	GO:1990644 microtubule site clamp;GO:0030674 protein	
Arp8p	Nuclear	GO:00325	GO:00310	GO:0043140 ATP-dependent 3'-5' DNA helicase activity;	
putative	Putative	GO:00551	GO:00057	GO:0016491 oxidoreductase activity;GO:0003824 catalyt	
None	None	None	None	None	None
Ebp2p	Required	GO:00422	GO:00306	GO:0003729 mRNA binding;GO:0003723 RNA binding;GO:000	
Cox16p	Mitochon	GO:00336	GO:00057	GO:0003674 molecular_function	
Bir1p	Subunit	GO:00070	GO:00007	GO:0046872 metal ion binding;GO:0043169 cation bindin	
Lre1p	Protein	GO:00315	GO:00059	GO:0004860 protein kinase inhibitor activity;GO:00192	
SCF ubiq	Putative	GO:00311	GO:00190	GO:0030674 protein binding, bridging;GO:0004842 ubiqu	
Gcn1p	Positive	GO:19042	GO:00226	GO:0043022 ribosome binding;GO:0019887 protein kinase	
hypotheti	Putative	GO:00081	GO:00160	GO:0003674 molecular_function	
None	None	None	None	None	None
DNA topo	Topoisom	GO:00001	GO:00312	GO:0003917 DNA topoisomerase type I activity;GO:00039	
None	None	None	None	None	None
mitofusi	Mitofusi	GO:00080	GO:00313	GO:0003924 GTPase activity;GO:0005525 GTP binding;GO:	
casein k	Beta' re	GO:00063	GO:00344	GO:00302	(sce04139)Mitophagy - yeast; (sce03008)Ribos
Aim44p	Protein	GO:19903	GO:00059	GO:0003674 molecular_function	
SNAP rec	R-SNARE	GO:00482	GO:00125	GO:00054	(sce04130)SNARE interactions in vesicular tr
phosphor	Phosphor	GO:00061	GO:00056	GO:00046	(M00048)Inosine monophosphate biosynthesis,
Upf3p	Componen	GO:00704	GO:00058	GO:00037	(sce03015)mRNA surveillance pathway; (sce030
Gat4p	Protein	GO:00063	GO:00056	GO:0008270 zinc ion binding;GO:0043565 sequence-speci	
coatomer	Essentia	GO:00068	GO:00301	GO:0005198 structural molecule activity;GO:0003674 mo	
ribosoma	Ribosoma	GO:00021	GO:00226	GO:00037	(sce03010)Ribosome
pyruvate	Pyruvate	GO:00060	GO:00058	GO:00047	(sce00020)Citrate cycle (TCA cycle); (sce006
ribosoma	Protein	GO:00000	GO:00226	GO:00198	(sce03010)Ribosome
Msn1p	Transcri	GO:00360	GO:00056	GO:0000978 RNA polymerase II proximal promoter sequen	
putative	Putative	GO:19026	GO:00306	GO:0004004 ATP-dependent RNA helicase activity;GO:000	
None	None	None	None	None	None
protein	Protein	GO:00723	GO:00056	GO:0072354 histone kinase activity (H3-T3 specific);G	
Ndd1p	Transcri	GO:00902	GO:00056	GO:0003713 transcription coactivator activity;GO:0003	
Seh1p	Subunit	GO:19042	GO:00970	GO:00170	(sce03013)RNA transport
Pet191p	Protein	GO:00336	GO:00313	GO:0003674 molecular_function	
hypotheti	Putative	GO:00081	GO:00055	GO:0003674 molecular_function	
riboflav	Riboflav	GO:00093	GO:00057	GO:00039	(sce00740)Riboflavin metabolism; (sce01110)B
alpha,al	Putative	GO:00059	GO:00057	GO:00045	(sce00500)Starch and sucrose metabolism; (sc
putative	Protein	GO:00007	GO:00330	GO:00080	(sce03440)Homologous recombination
Sto1p	Large su	GO:00310	GO:00058	GO:00003	(sce03015)mRNA surveillance pathway; (sce030
hexokina	Hexokina	GO:00324	GO:00057	GO:00191	(M00549)Nucleotide sugar biosynthesis, gluco
tRNA met	tRNA met	GO:00315	GO:00057	GO:0008175 tRNA methyltransferase activity;GO:0008173	
xanthine	Xanthine-	GO:00322	GO:00057	GO:0000310 xanthine phosphoribosyltransferase activit	

Sip3p	Putative	GO:00457[illegible]	GO:00325[illegible]	GO:0016538 cyclin-dependent protein serine/threonine [illegible]	
peptidyl[illegible]	Cytoplasm	GO:00458[illegible]	GO:00349[illegible]	GO:00160[illegible]	(Human)(hsa05145)Toxoplasmosis; (hsa05012)Pa[illegible]
Erp1p	Member o[illegible]	GO:00066[illegible]	GO:00301[illegible]	GO:0003674 molecular_function	
hypotheti[illegible]	Putative	GO:00162[illegible]	GO:00003[illegible]	GO:0003674 molecular_function	
putative	Protein [illegible]	GO:01100[illegible]	GO:00056[illegible]	GO:00047[illegible]	(sce04011)MAPK signaling pathway - yeast; (s[illegible]
hypotheti[illegible]	Putative protein of unknown function; functions together with HAM1 to mediate re[illegible]				
kinetoch[illegible]	Component	GO:00513[illegible]	GO:00312[illegible]	GO:0044877 protein-containing complex binding;GO:0005[illegible]	
ATP-deper	ATP-deper	GO:19904[illegible]	GO:00314[illegible]	GO:0004004 ATP-dependent RNA helicase activity;GO:000[illegible]	
Y' elemer	Helicase	GO:00007[illegible]	GO:00056[illegible]	GO:0003678 DNA helicase activity;GO:0004386 helicase [illegible]	
None	None	None	None	None	None
putative	Subunit [illegible]	GO:00363[illegible]	GO:00976[illegible]	GO:0004842 ubiquitin-protein transferase activity;GO:[illegible]	
Chl4p	Outer kir	GO:00340[illegible]	GO:00009[illegible]	GO:0005198 structural molecule activity;GO:0003674 mo[illegible]	
ATP-bind[illegible]	Subunit [illegible]	GO:00159[illegible]	GO:00057[illegible]	GO:00053[illegible]	(sce02010)ABC transporters; (sce04146)Peroxi[illegible]
hypotheti[illegible]	Mitochon[illegible]	GO:00081[illegible]	GO:00319[illegible]	GO:0003674 molecular_function	
Rev3p	Catalyti[illegible]	GO:00709[illegible]	GO:00160[illegible]	GO:00038[illegible]	(sce01100)Metabolic pathways
ssDNA en[illegible]	Single-s[illegible]	GO:00062[illegible]	GO:00001[illegible]	GO:00000[illegible]	(sce03420)Nucleotide excision repair
aminopho[illegible]	Trans-go[illegible]	GO:00007[illegible]	GO:19905[illegible]	GO:0004012 phospholipid-translocating ATPase activity	
Ase1p	Mitotic [illegible]	GO:00000[illegible]	GO:00058[illegible]	GO:0008017 microtubule binding;GO:0015631 tubulin bin[illegible]	
acetyl-C[illegible]	Mitochon[illegible]	GO:20012[illegible]	GO:00057[illegible]	GO:00039[illegible]	(sce)Fatty acid biosynthesis, initiation; (M[illegible]
Mpt5p	mRNA-bin[illegible]	GO:19001[illegible]	GO:00057[illegible]	GO:0030674 protein binding, bridging;GO:0003729 mRNA [illegible]	
Arf fami[illegible]	ARF-like	GO:00430[illegible]	GO:00421[illegible]	GO:0003924 GTPase activity;GO:0005525 GTP binding;GO:[illegible]	
DNA-bind[illegible]	Subunit [illegible]	GO:00362[illegible]	GO:00702[illegible]	GO:0004842 ubiquitin-protein transferase activity;GO:[illegible]	
None	None	None	None	None	None
hypotheti[illegible]	Putative	GO:00081[illegible]	GO:00057[illegible]	GO:0003674 molecular_function	
replicat[illegible]	Subunit [illegible]	GO:00062[illegible]	GO:00313[illegible]	GO:00036[illegible]	(sce03430)Mismatch repair; (sce03030)DNA rep[illegible]
succinat[illegible]	Membrane	GO:00061[illegible]	GO:00057[illegible]	GO:00081[illegible]	(sce)Citrate cycle, second carbon oxidation,
prion do[illegible]	[PIN(+)]	GO:00081[illegible]	GO:00058[illegible]	GO:0003674 molecular_function	
flavodox[illegible]	Protein [illegible]	GO:00229[illegible]	GO:00451[illegible]	GO:0003955 NAD(P)H dehydrogenase (quinone) activity;G[illegible]	
ribosoma[illegible]	Protein [illegible]	GO:00004[illegible]	GO:00226[illegible]	GO:00037[illegible]	(sce03010)Ribosome
mitochon[illegible]	Mitochon[illegible]	GO:00325[illegible]	GO:00057[illegible]	GO:0003735 structural constituent of ribosome;GO:0005	
Yet1p	Endoplasm	GO:00709[illegible]	GO:00057[illegible]	GO:0043022 ribosome binding;GO:0043021 ribonucleoprot[illegible]	
hypotheti[illegible]	Putative	GO:00081[illegible]	GO:00055[illegible]	GO:0003674 molecular_function	
Sps100p	Protein [illegible]	GO:00304[illegible]	GO:00056[illegible]	GO:0003674 molecular_function	
ribosome	Essentia[illegible]	GO:00422[illegible]	GO:00226[illegible]	GO:0003723 RNA binding;GO:0003677 DNA binding;GO:000[illegible]	
ribosoma[illegible]	Ribosoma[illegible]	GO:00004[illegible]	GO:00226[illegible]	GO:00037[illegible]	(sce03010)Ribosome
hypotheti[illegible]	Putative	GO:00081[illegible]	GO:00055[illegible]	GO:0003674 molecular_function	
1-phosph[illegible]	Type II [illegible]	GO:00468[illegible]	GO:00003[illegible]	GO:00044[illegible]	(sce00562)Inositol phosphate metabolism; (sc[illegible]
8-oxo-dG[illegible]	8-oxo-dG[illegible]	GO:00159[illegible]	GO:00057[illegible]	GO:0008413 8-oxo-7,8-dihydroguanosine triphosphate py[illegible]	
Sda1p	Protein [illegible]	GO:00000[illegible]	GO:00057[illegible]	GO:0003674 molecular_function	
ribose p[illegible]	5-phosph[illegible]	GO:00060[illegible]	GO:00021[illegible]	GO:00047[illegible]	(M00005)PRPP biosynthesis, ribose 5P => PRPP
Hda2p	Subunit [illegible]	GO:00109[illegible]	GO:00708[illegible]	GO:0004407 histone deacetylase activity;GO:0033558 pr[illegible]	
hypotheti[illegible]	Tail-anc[illegible]	GO:00081[illegible]	GO:00059[illegible]	GO:0003674 molecular_function	
Gmh1p	Golgi mem	GO:00161[illegible]	GO:00301[illegible]	GO:0003674 molecular_function	
U2-type [illegible]	General [illegible]	GO:00003[illegible]	GO:00056[illegible]	GO:0003677 DNA binding;GO:0046872 metal ion binding;G[illegible]	
None	None	None	None	None	None
Pmp3p	Small pl[illegible]	GO:00356[illegible]	GO:00058[illegible]	GO:0080025 phosphatidylinositol-3,5-bisphosphate bind	
ribosoma[illegible]	Protein [illegible]	GO:00064[illegible]	GO:00226[illegible]	GO:00198[illegible]	(sce03010)Ribosome
Est1p	hEST1A/B	GO:00081[illegible]	GO:00055[illegible]	GO:0003674 molecular_function	
S-adenos[illegible]	S-methyl[illegible]	GO:00192[illegible]	GO:00057[illegible]	GO:00088[illegible]	(sce00270)Cysteine and methionine metabolism
hypotheti[illegible]	Putative	GO:00081[illegible]	GO:00301[illegible]	GO:0003677 DNA binding;GO:0003676 nucleic acid bindin[illegible]	
ubiquino[illegible]	Putative	GO:19010[illegible]	GO:00057[illegible]	GO:0003954 NADH dehydrogenase activity;GO:0016651 oxi[illegible]	
rRNA-pro[illegible]	Subunit [illegible]	GO:00004[illegible]	GO:00320[illegible]	GO:0003674 molecular_function	
hypotheti[illegible]	Helicase-	GO:00081[illegible]	GO:00055[illegible]	GO:0005524 ATP binding;GO:0032559 adenyl ribonucleoti[illegible]	
ubiquiti[illegible]	Ubiquiti[illegible]	GO:00457[illegible]	GO:00056[illegible]	GO:0004843 thiol-dependent ubiquitin-specific proteas[illegible]	

mitochon	Mitochon	GO:00325	GO:00057	GO:00037	(sce03010)Ribosome
hypotheti	Tail-anc	GO:00081	GO:00702	GO:0003674 molecular_function	
tryptoph	Cytoplas	GO:00064	GO:00057	GO:00048	(sce00970)Aminoacyl-tRNA biosynthesis
hypotheti	Putative	GO:00081	GO:00160	GO:0003674 molecular_function	
Sac3p	mRNA exp	GO:00710	GO:00446	GO:0005515 protein binding;GO:0005488 binding;GO:0003	
Rad10p	Single-s	GO:00007	GO:00705	GO:00000	(sce03420)Nucleotide excision repair
Psy2p	Subunit	GO:19026	GO:00302	GO:0019888 protein phosphatase regulator activity;GO:	
hypotheti	Protein	GO:00081	GO:00056	GO:0003674 molecular_function	
Mob1p	Componen	GO:00070	GO:00058	GO:00192	(sce04392)Hippo signaling pathway -multiple
Nvj3p	Protein	GO:19908	GO:00715	GO:0032266 phosphatidylinositol-3-phosphate binding;G	
Yhk8p	Presumed	GO:00068	GO:00058	GO:0015297 antiporter activity;GO:0015238 drug transm	
ATP-bind	ATP-bind	GO:19904	GO:00003	GO:0042626 ATPase activity, coupled to transmembrane	
Knh1p	Protein	GO:00060	GO:00092	GO:0003674 molecular_function	
Van1p	Componen	GO:00000	GO:00001	GO:00000	(sce)N-glycan biosynthesis, high-mannose typ
Hed1p	Meiosis-	GO:19038	GO:00007	GO:0004857 enzyme inhibitor activity;GO:0030234 enzym	
hypotheti	Putative	GO:00081	GO:00160	GO:0003674 molecular_function	
ribosoma	Ribosoma	GO:00000	GO:00226	GO:00080	(sce03010)Ribosome
ribosoma	Ribosoma	GO:00004	GO:00226	GO:00037	(sce03010)Ribosome
None	None	None	None	None	None
thiamine	Mitochon	GO:19905	GO:00313	GO:0090422 thiamine pyrophosphate transmembrane trans	
Ecm27p	Protein	GO:00703	GO:00057	GO:0003674 molecular_function	
mitochon	Mitochon	GO:00310	GO:00057	GO:0004525 ribonuclease III activity;GO:0032296 doubl	
Cyk3p	SH3-doma	GO:19903	GO:00001	GO:0030234 enzyme regulator activity;GO:0098772 molec	
Signal r	Signal r	GO:00066	GO:00057	GO:00050	(sce03060)Protein export
hypotheti	Dubious	GO:00081	GO:00055	GO:0003674 molecular_function	
hypotheti	Putative protein of unknown function; YBR230W-A has a paralog, COQ8, that arose				
Fap1p	Protein	GO:00001	GO:00056	GO:0000977 RNA polymerase II regulatory region sequen	
Rmd1p	Cytoplas	GO:00304	GO:00003	GO:0003674 molecular_function	
Gly-Xaa	Vacuolar	GO:00516	GO:00003	GO:0004181 metallocarboxypeptidase activity;GO:000418	
None	None	None	None	None	None
Oca5p	Cytoplas	GO:00081	GO:00057	GO:0003674 molecular_function	
serine/t	Serine/t	GO:00460	GO:00057	GO:0004674 protein serine/threonine kinase activity;G	
Mkt1p	Protein	GO:00903	GO:00104	GO:0017108 5'-flap endonuclease activity;GO:0048256 f	
Lrp1p	Nuclear	GO:00710	GO:00001	GO:00037	(sce03018)RNA degradation
mitogen-	Signal t	GO:00715	GO:00057	GO:00320	(sce04011)MAPK signaling pathway - yeast
None	None	None	None	None	None
putative	Protein	GO:00091	GO:00057	GO:00169	(sce00785)Lipoic acid metabolism; (sce01100)
Y' eleme	Helicase	GO:00007	GO:00056	GO:0003678 DNA helicase activity;GO:0004386 helicase	
hypotheti	Protein	GO:00081	GO:00057	GO:0003674 molecular_function	
Sin4p	Subunit	GO:00702	GO:00708	GO:0001102 RNA polymerase II activating transcription	
Pcl6p	Pho85p c	GO:00059	GO:00003	GO:0016538 cyclin-dependent protein serine/threonine	
Ebs1p	Protein	GO:00322	GO:00056	GO:0070034 telomerase RNA binding;GO:0042162 telomeri	
Pxl1p	Protein	GO:00300	GO:00059	GO:0005094 Rho GDP-dissociation inhibitor activity;GO	
U2 snRNP	U2-snRNP	GO:00003	GO:00056	GO:00037	(sce03040)Spliceosome
glyoxyla	Glyoxyla	GO:00094	GO:00057	GO:0047964 glyoxylate reductase activity;GO:0016618 h	
putative	Putative	GO:00340	GO:00057	GO:0003924 GTPase activity;GO:0005525 GTP binding;GO:	
None	None	None	None	None	None
4-amino-	Para-ami	GO:00081	GO:00057	GO:00468	(sce00790)Folate biosynthesis
Bud4p	Anillin-	GO:00071	GO:00001	GO:0005525 GTP binding;GO:0032550 purine ribonucleosi	
None	None	None	None	None	None
hypotheti	Putative	GO:00081	GO:00055	GO:0003674 molecular_function	
putative	Potentia	GO:00159	GO:00058	GO:0015247 aminophospholipid transmembrane transporte	
ESCRT-I	Componen	GO:19046	GO:00008	GO:0005515 protein binding;GO:0005488 binding;GO:0003	
hypotheti	Putative	GO:00081	GO:00719	GO:0003674 molecular_function	

Swd3p	Essential	GO:190334	GO:004818	GO:0042800	histone methyltransferase activity (H3-K4
translati	eIF3b sul	GO:000218	GO:004361	GO:003136	(sce03013)RNA transport
DEAH-box	DEAH-box	GO:000038	GO:007102	GO:003445	(sce03040)Spliceosome
Mtf2p	Mitochon	GO:003254	GO:000575	GO:0003723	RNA binding;GO:0003676 nucleic acid bindin
Ama1p	Activato	GO:190578	GO:000568	GO:199075	(sce04113)Meiosis - yeast
glycylpe	N-myrist	GO:001800	GO:000582	GO:0004379	glycylpeptide N-tetradecanoyltransferase a
prephena	Prephena	GO:000909	GO:000573	GO:000466	(M00024)Phenylalanine biosynthesis, chorisma
None	None	None	None	None	None
Aim7p	Protein	GO:003431	GO:003047	GO:0071933	Arp2/3 complex binding;GO:0003779 actin bi
Urc2p	Putative	GO:000108	GO:000563	GO:0000981	DNA-binding transcription factor activity,
Ifh1p	Coactiva	GO:006096	GO:003254	GO:0003713	transcription coactivator activity;GO:0003
mannose-6	Mannose-6	GO:000003	GO:000563	GO:000447	(sce00051)Fructose and mannose metabolism; (
origin re	Subunit	GO:000626	GO:000566	GO:000368	(sce04111)Cell cycle - yeast; (sce04113)Meio
Emp46p	Integral	GO:000702	GO:003013	GO:000553	(sce04141)Protein processing in endoplasmic
Tsr1p	Protein	GO:000046	GO:003068	GO:0034511	U3 snoRNA binding;GO:0030515 snoRNA bindin
hypotheti	Putative	GO:000815	GO:000573	GO:0003674	molecular_function
NADHX ep	NADHX ep	GO:004649	GO:000573	GO:0052856	NADHX epimerase activity;GO:0052857 NADPHX
Mit1p	Transcri	GO:000815	GO:000563	GO:0003677	DNA binding;GO:0003676 nucleic acid bindin
Kel3p	Cytoplas	GO:000815	GO:000573	GO:0003674	molecular_function
hypotheti	Putative	(Human)GO	(Human)GO	(Human)GO:0005515	protein binding;GO:0005488 binding;
hypotheti	Putative	GO:005508	GO:000032	GO:0003674	molecular_function
transcri	Represso	GO:000708	GO:003330	GO:000113	(sce04111)Cell cycle - yeast
Rds1p	Putative	GO:004594	GO:000563	GO:0000978	RNA polymerase II proximal promoter sequen
pyruvate	Pyruvate	GO:000609	GO:000573	GO:000474	(sce)Guanine ribonucleotide biosynthesis IMP
tRNA N6-a	Highly c	GO:000294	GO:000040	GO:0061711	N(6)-L-threonylcarbamoyladenine synthase a
DEAD-box	ATP-depen	GO:000039	GO:001049	GO:0033592	RNA strand annealing activity;GO:0004004 A
CORVET c	Membrane-	GO:003251	GO:003326	GO:004349	(sce04138)Autophagy - yeast
iron-sul	Highly c	GO:000209	GO:190456	GO:0051539	4 iron, 4 sulfur cluster binding;GO:005153
prohibiti	Subunit	GO:000130	GO:199042	GO:0003674	molecular_function
isocitra	Subunit	GO:000653	GO:000596	GO:000444	(M00010)Citrate cycle, first carbon oxidatio
Spp41p	Protein	GO:001062	GO:000582	GO:0003674	molecular_function
None	None	None	None	None	None
hypotheti	Putative	GO:004594	GO:000563	GO:0000978	RNA polymerase II proximal promoter sequen
1,3-beta-	Beta-1,3-	GO:007197	GO:000093	GO:0042124	1,3-beta-glucanosyltransferase activity;GO
i-AAA pr	Catalyti	GO:004504	GO:003194	GO:000417	(sce04139)Mitophagy - yeast
hypotheti	Protein of unknown function; localized to the mitochondrial outer membrane				
Paf1p	Componen	GO:200125	GO:001659	GO:199026	(sce04011)MAPK signaling pathway - yeast
None	None	None	None	None	None
GPI-anch	Transmem	GO:001625	GO:004276	GO:000392	(sce00563)Glycosylphosphatidylinositol (GPI)-
bifuncti	Thymidyl	GO:000623	GO:000582	GO:000479	(sce00240)Pyrimidine metabolism; (sce01100)M
protein	ER prote	GO:000662	GO:000578	GO:001915	(sce04141)Protein processing in endoplasmic
None	None	None	None	None	None
None	None	None	None	None	None
IMP dehy	Inosine	GO:000617	GO:000573	GO:000393	(sce)Guanine ribonucleotide biosynthesis IMP
putative	Ubiquiti	GO:001063	GO:000563	GO:0004843	thiol-dependent ubiquitin-specific proteas
hypotheti	Putative	GO:000815	GO:000557	GO:0003674	molecular_function
hypotheti	Protein	GO:000815	GO:000557	GO:0003674	molecular_function
GTPase-a	GTPase a	GO:000640	GO:000078	GO:000509	(sce03013)RNA transport
cyclin-d	Cyclin-d	GO:004573	GO:000563	GO:000486	(sce04011)MAPK signaling pathway - yeast; (s
hypotheti	Protein	GO:000815	GO:000563	GO:0003674	molecular_function
None	None	None	None	None	None
hypotheti	Putative	GO:000815	GO:000557	GO:0008017	microtubule binding;GO:0015631 tubulin bin
None	None	None	None	None	None
alpha-gl	Alpha-gl	GO:001576	GO:000588	GO:0005363	maltose transmembrane transporter activity

calcium-t	Vacuolar	GO:00705	GO:00003	GO:00053	(Human)(hsa04970)Salivary secretion; (hsa049
Syf2p	Member of	GO:00003	GO:00710	GO:00003	(sce03040)Spliceosome
None	None	None	None	None	None
Ric1p	Protein i	GO:00421	GO:00340	GO:0017112	Rab guanyl-nucleotide exchange factor acti
Ula1p	Protein t	GO:00451	GO:00057	GO:0019781	NEDD8 activating enzyme activity;GO:000864
Mrt4p	Protein i	GO:00000	GO:00306	GO:0070180	large ribosomal subunit rRNA binding;GO:00
ribulose-	D-ribulos	GO:00090	GO:00058	GO:00047	(M00007)Pentose phosphate pathway, non-oxida
transcrip	TFIIIB B-	GO:00900	GO:00001	GO:0000994	RNA polymerase III core binding;GO:0001006
glucose N	N-acetylg	GO:00064	GO:00057	GO:0008375	acetylglucosaminyltransferase activity;GO:
None	None	None	None	None	None
None	None	None	None	None	None
None	None	None	None	None	None
None	None	None	None	None	None
None	None	None	None	None	None
Nup192p	Essential	GO:00069	GO:00446	GO:00170	(sce03013)RNA transport
beta-1,3-	O-glycosy	GO:00315	GO:00056	GO:0005199	structural constituent of cell wall;GO:000
None	None	None	None	None	None
None	None	None	None	None	None
Taf1p	TFIID sub	GO:00511	GO:00056	GO:00044	(sce03022)Basal transcription factors
ribosome	Component	GO:00004	GO:00306	GO:0003674	molecular_function
Rtp1p	Protein r	GO:00066	GO:00057	GO:0003674	molecular_function
Ufe1p	t-SNARE p	GO:00482	GO:00312	GO:00054	(sce04130)SNARE interactions in vesicular tr
Hir1p	Subunit o	GO:00307	GO:00004	GO:0031491	nucleosome binding;GO:0003714 transcriptio
carboxyle	Protein w	GO:19905	GO:00057	GO:0051723	protein methylesterase activity;GO:0052689
hypotheti	Putative	GO:00081	GO:00055	GO:0003674	molecular_function
GTPase-ac	Structura	GO:00310	GO:00301	GO:00051	(sce04141)Protein processing in endoplasmic
Rab famil	Rab famil	GO:00071	GO:00433	GO:00039	(sce04144)Endocytosis
None	None	None	None	None	None
ribosomal	Ribosomal	GO:00004	GO:00226	GO:00037	(sce03010)Ribosome
Aim29p	Protein o	GO:00081	GO:00057	GO:0003674	molecular_function
aminoacyl	One of tw	GO:00324	GO:00057	GO:0004045	aminoacyl-tRNA hydrolase activity;GO:01401
putative	Putative	GO:00081	GO:00056	GO:0016853	isomerase activity;GO:0003824 catalytic ac
Y' elemen	Helicase-	GO:00081	GO:00056	GO:0004386	helicase activity;GO:0017111 nucleoside-tr
putative	Putative	GO:00551	GO:00058	GO:0004617	phosphoglycerate dehydrogenase activity;GO
SAM compl	Component	GO:00450	GO:00014	GO:0003674	molecular_function
mRNA spli	Member of	GO:00003	GO:00056	GO:00036	(sce03040)Spliceosome
kleisin a	Essential	GO:00340	GO:00349	GO:00199	(sce04111)Cell cycle - yeast
None	None	None	None	None	None
Swc5p	Component	GO:00434	GO:00008	GO:0003674	molecular_function
Aim26p	Protein o	GO:00004	GO:00319	GO:0003674	molecular_function
Tod6p	PAC motif	GO:00063	GO:00702	GO:0043565	sequence-specific DNA binding;GO:0003677 D
hypotheti	Protein o	GO:00081	GO:00058	GO:0003674	molecular_function
Mch2p	Protein w	GO:00157	GO:00160	GO:0008028	monocarboxylic acid transmembrane transpor
Rho famil	Non-essen	GO:00309	GO:00321	GO:0003924	GTPase activity;GO:0005525 GTP binding;GO:
transcrip	Subunit o	GO:00427	GO:00007	GO:0001002	RNA polymerase III type 1 promoter sequenc
rRNA-bind	Constitue	GO:00004	GO:00306	(Human)	GO:0003723 RNA binding;GO:0003676 nucleic acid
Tma7p	Protein o	GO:00021	GO:00056	GO:0043024	ribosomal small subunit binding;GO:0043021
SCF ubiqu	F-box con	GO:00107	GO:00190	GO:0004842	ubiquitin-protein transferase activity;GO:
Rgi2p	Protein o	GO:00061	GO:00057	GO:0003674	molecular_function
GPI-ancho	Subunit o	GO:00162	GO:00427	GO:00039	(sce00563)Glycosylphosphatidylinositol (GPI)-
Ssp2p	Sporulati	GO:00304	GO:00056	GO:0003676	nucleic acid binding;GO:1901363 heterocycl
translati	Delta sub	GO:00064	GO:00320	GO:00037	(sce03013)RNA transport
ribosome	Nucleolar	GO:19026	GO:00306	GO:0003723	RNA binding;GO:0003676 nucleic acid bindin
indolepyr	Minor iso	GO:00009	GO:00058	GO:00501	(sce00010)Glycolysis / Gluconeogenesis; (sce

ethanolar	Ethanolar	GO:00066	GO:00056	GO:00043	(sce)Phosphatidylethanolamine (PE) biosynthe
hypotheti	Putative	GO:00081	GO:00160	GO:0003674 molecular_function	
putative	Tyrosine	GO:00353	GO:00058	GO:0004725 protein tyrosine phosphatase activity;GO:0	
DNA-bindi	Carbon so	GO:00614	GO:00058	GO:0001093 TFIIB-class transcription factor binding;G	
Gtr2p	Subunit c	GO:19037	GO:00344	GO:0003924 GTPase activity;GO:0005525 GTP binding;GO:	
None	None	None	None	None	None
Ctf3p	Outer kin	GO:00070	GO:00007	GO:0003674 molecular_function	
mRNA spli	U1 snRNP	GO:00003	GO:00002	GO:0030627 pre-mRNA 5'-splice site binding;GO:0036002	
high-affi	High affi	GO:19016	GO:00335	GO:0015093 ferrous iron transmembrane transporter act	
None	None	None	None	None	None
None	None	None	None	None	None
dolichyl-	Alpha-1,6	GO:00064	GO:00057	GO:00529	(sce)N-glycan precursor biosynthesis; (M0005
Rab fami	Rab fami	GO:00328	GO:19908	GO:00039	(sce04145)Phagosome; (sce04144)Endocytosis;
ribose ph	5-phospho	GO:00060	GO:00021	GO:00047	(M00005)PRPP biosynthesis, ribose 5P => PRPP
Erv41p	Protein	GO:00068	GO:00301	GO:0003674 molecular_function	
Vps52p	Componen	GO:00901	GO:00009	GO:0019905 syntaxin binding;GO:0017137 Rab GTPase bin	
bifunctio	Triparti	GO:00719	GO:00007	GO:00171	(sce03030)DNA replication
ribosoma	Ribosoma	GO:00021	GO:00226	GO:00037	(sce03010)Ribosome
centromer	Histone H	GO:00616	GO:00057	GO:0019237 centromeric DNA binding;GO:0031492 nucleos	
mRNA-bind	Protein i	GO:00422	GO:00306	GO:0003729 mRNA binding;GO:0003723 RNA binding;GO:000	
hypotheti	Protein c	GO:00081	GO:00058	GO:0003674 molecular_function	
Mac1p	Copper-se	GO:00068	GO:00056	GO:0005507 copper ion binding;GO:0000978 RNA polymera	
None	None	None	None	None	None
translati	Translati	GO:19901	GO:00058	GO:0003746 translation elongation factor activity;GO:	
ribosoma	Ribosoma	GO:00004	GO:00306	GO:00037	(sce03010)Ribosome
chitin de	Chitin de	GO:00304	GO:00056	GO:00040	(sce00520)Amino sugar and nucleotide sugar m
None	None	None	None	None	None
hypotheti	Putative	GO:00081	GO:00057	GO:0003674 molecular_function	
DNA-direc	RNA polym	GO:00011	GO:00056	GO:00039	(sce03020)RNA polymerase; (sce00240)Pyrimidi
E3 ubiqui	E3 ubiqui	GO:01201	GO:19903	GO:1904855 proteasome regulatory particle binding;GO:	
Jjj1p	Co-chaper	GO:00000	GO:00306	GO:0001671 ATPase activator activity;GO:0060590 ATPas	
catalase	Catalase	GO:00013	GO:00057	GO:00040	(sce00380)Tryptophan metabolism; (sce00630)G
hypotheti	Putative protein of unknown function; identified by gene-trapping, microarray-ba				
spindle c	Componen	GO:00070	GO:00007	GO:00448	(sce04111)Cell cycle - yeast; (sce04113)Meio
hypotheti	Putative	GO:00428	GO:00160	GO:0015297 antiporter activity;GO:0015238 drug transm	
Tma64p	Protein c	GO:00017	GO:00226	GO:0043024 ribosomal small subunit binding;GO:0003743	
U2 snRNP	U2B compo	GO:00003	GO:00056	GO:0030620 U2 snRNA binding;GO:0035614 snRNA stem-loo	
hypotheti	Putative	GO:00081	GO:00160	GO:0003674 molecular_function	
hypotheti	Putative	GO:00081	GO:00057	GO:0003674 molecular_function	
Zn(2+) tr	Zinc tran	GO:00068	GO:00057	GO:0005385 zinc ion transmembrane transporter activit	
Rab fami	Rab fami	GO:00066	GO:00003	GO:0003924 GTPase activity;GO:0005525 GTP binding;GO:	
signal pe	Subunit c	GO:00064	GO:00057	GO:00082	(sce03060)Protein export
translati	eIF3g sub	GO:00021	GO:00436	GO:00037	(sce03013)RNA transport
hypotheti	Putative	GO:00081	GO:00160	GO:0003674 molecular_function	
Plp1p	Protein t	GO:00073	GO:00057	GO:0031683 G-protein beta/gamma-subunit complex bindi	
Y' elemen	Helicase	GO:00007	GO:00056	GO:0003678 DNA helicase activity;GO:0004386 helicase	
ribosoma	Ribosoma	GO:00480	GO:00226	GO:00306	(sce03010)Ribosome
hypotheti	Putative	GO:00081	GO:00055	GO:0003674 molecular_function	
lipid-bin	Eisosome	GO:00709	GO:00362	GO:0008289 lipid binding;GO:0005488 binding;GO:000367	
rRNA-proc	Putative	GO:00004	GO:00320	GO:00045	(sce03008)Ribosome biogenesis in eukaryotes
2-hexapre	2-hexapre	GO:00067	GO:00057	GO:00433	(M00128)Ubiquinone biosynthesis, eukaryotes,
alpha-tub	Alpha-tub	GO:00988	GO:00058	GO:00052	(sce04145)Phagosome
Rgd1p	GTPase-ac	GO:00072	GO:00304	GO:0010314 phosphatidylinositol-5-phosphate binding;G	
hypotheti	Putative	GO:00342	GO:00055	GO:0004360 glutamine-fructose-6-phosphate transaminas	

Pet111p	Mitochon	GO:00701	GO:0057	GO:0045182 translation regulator activity;GO:0003674	
Esc8p	Protein	GO:00070	GO:0056	GO:0003674 molecular_function	
Stu1p	Componen	GO:00513	GO:0000777 condensed chromosome kinetochore;GO:0005816 spindle		
bleomyci	Cysteine	GO:00434	GO:00057	GO:0004177 aminopeptidase activity;GO:0004197 cystein	
E3 ubiqu	Membrane-	GO:00304	GO:00008	GO:00616	(sce04141)Protein processing in endoplasmic
Inp2p	Peroxiso	GO:00450	GO:00057	GO:0043495 protein membrane anchor;GO:0005515 protein	
Ssn2p	Subunit	GO:00004	GO:00165	GO:0001135 RNA polymerase II transcription regulator	
Lso2p	Protein	GO:00550	GO:00056	GO:0043022 ribosome binding;GO:0043021 ribonucleoprot	
hypotheti	Dubious	GO:00081	GO:00055	GO:0003674 molecular_function	
alpha-gl	Alpha-gl	GO:00157	GO:00003	GO:0005363 maltose transmembrane transporter activity	
adenylos	Adenylos	GO:00442	GO:00055	GO:00706	(M00048)Inosine monophosphate biosynthesis,
Pfs1p	Sporulat	GO:00304	GO:00058	GO:0003674 molecular_function	
None	None	None	None	None	None
hydroxya	Mitochon	GO:00192	GO:00057	GO:00044	(sce00620)Pyruvate metabolism
hypotheti	Putative	GO:00081	GO:00055	GO:0003674 molecular_function	
None	None	None	None	None	None
Bud22p	Protein	GO:00304	GO:00306	GO:0003674 molecular_function	
ribosoma	Ribosoma	GO:00468	GO:00226	GO:00037	(sce03010)Ribosome
hypotheti	Protein	GO:00081	GO:00056	GO:0003674 molecular_function	
ATP-depe	ATP-depe	GO:00003	GO:00002	GO:0008859 exoribonuclease II activity;GO:0004004 ATP	
Csm3p	Replicat	GO:00340	GO:00312	GO:0003677 DNA binding;GO:0003676 nucleic acid bindin	
putative	Putative	GO:00322	GO:00057	GO:0008757 S-adenosylmethionine-dependent methyltrans	
peptidyl	Peptidyl-	GO:00004	GO:00003	GO:0016018 cyclosporin A binding;GO:0003755 peptidyl-	
mitogen-	Middle s	GO:00304	GO:00057	GO:0004707 MAP kinase activity;GO:0004674 protein ser	
ATP-depe	ATP-depe	GO:00710	GO:00314	GO:00344	(sce03018)RNA degradation
Bre2p	Subunit	GO:00801	GO:00481	GO:0042800 histone methyltransferase activity (H3-K4	
cleavage	Essentia	GO:00987	GO:00058	GO:00037	(sce03015)mRNA surveillance pathway
Bck2p	Serine/t	GO:00000	GO:00056	GO:0003674 molecular_function	
guanine	G-protei	GO:19026	GO:00226	GO:00050	(Human)(hsa05162)Measles
hypotheti	Protein	GO:00081	GO:00058	GO:0043022 ribosome binding;GO:0043021 ribonucleoprot	
Pat1p	Deadenyl	GO:00002	GO:19907	GO:00037	(sce03018)RNA degradation
Emc3p	Member o	GO:00349	GO:00725	GO:0003674 molecular_function	
Mrx8p	Protein	GO:00081	GO:00057	GO:0005525 GTP binding;GO:0032550 purine ribonucleosi	
Est1p	TLC1 RNA-	GO:00719	GO:00056	GO:0070034 telomerase RNA binding;GO:0042162 telomeri	
U4/U6-U5	mRNA spl	GO:00003	GO:00465	GO:0003723 RNA binding;GO:0003676 nucleic acid bindin	
protein	Nim1p-re	GO:19029	GO:00001	GO:00046	(sce04011)MAPK signaling pathway - yeast; (s
AMP-acti	Activati	GO:00457	GO:00056	GO:0004679 AMP-activated protein kinase activity;GO:0	
Cbs1p	Mitochon	GO:00345	GO:00313	GO:0045182 translation regulator activity;GO:0003674	
None	None	None	None	None	None
chromati	Subunit	GO:00511	GO:00466	GO:00162	(sce03022)Basal transcription factors
ribosoma	Ribosoma	GO:00021	GO:00226	GO:00037	(sce03010)Ribosome
bifuncti	DNA N-gl	GO:00902	GO:00057	GO:00007	(sce03410)Base excision repair
Emc5p	Member o	GO:00349	GO:00725	GO:0003674 molecular_function	
Mrs1p	Splicing	GO:00003	GO:00057	GO:0000402 crossed form four-way junction DNA binding	
translati	Translat	GO:00064	GO:00104	GO:0003746 translation elongation factor activity;GO:	
Psy3p	Componen	GO:00007	GO:00971	GO:0003674 molecular_function	
rRNA-pro	Subunit	GO:00422	GO:00343	GO:00036	(sce03008)Ribosome biogenesis in eukaryotes
mitochon	Mitochon	GO:00325	GO:00057	GO:0000048 peptidyltransferase activity;GO:0016755 tr	
hypotheti	Protein	GO:00081	GO:00057	GO:0003674 molecular_function	
calmoduli	Calmoduli	GO:00345	(Human)G	GO:00046	(Human)(hsa04925)Aldosterone synthesis and s
Cis3p	Mannose-	GO:00315	GO:00059	GO:0005199 structural constituent of cell wall;GO:000	
hypotheti	Putative	GO:00081	GO:00055	GO:0003674 molecular_function	
putative	Putative	GO:00321	GO:00056	GO:00046	(sce04113)Meiosis - yeast
hypotheti	Putative	GO:00081	GO:00055	GO:0003674 molecular_function	

Stb5p	Transcrip	GO:004361	GO:000563	GO:0000981	DNA-binding transcription factor activity,
Rgp1p	Subunit c	GO:004214	GO:003400	GO:0017112	Rab guanyl-nucleotide exchange factor acti
serine/th	Serine/th	GO:200030	GO:003047	GO:0004674	protein serine/threonine kinase activity;G
glutamate	NADP(+)-c	GO:001967	GO:000582	GO:000435	(sce00910)Nitrogen metabolism; (sce00220)Arg
Chs7p	Protein c	GO:003422	GO:000562	GO:0051082	unfolded protein binding;GO:0005515 protei
None	None	None	None	None	None
Rrt5p	Putative	GO:000815	GO:000562	GO:0003723	RNA binding;GO:0003676 nucleic acid bindin
None	None	None	None	None	None
mannose-e	Integral	GO:000650	GO:003017	GO:005120	(sce00563)Glycosylphosphatidylinositol (GPI)-
Rmd8p	Cytosolic	GO:003043	GO:000578	GO:0003674	molecular_function
putative	Putative	GO:007155	GO:000032	GO:000418	(Human)(hsa04614)Renin-angiotensin system; (I
Lys14p	Transcrip	GO:200119	GO:000563	GO:0000978	RNA polymerase II proximal promoter sequen
putative	Putative	GO:000003	GO:000579	GO:0000026	alpha-1,2-mannosyltransferase activity;GO:
Stb4p	Putative	GO:000635	GO:000573	GO:0000981	DNA-binding transcription factor activity,
RNA polym	Mitochond	GO:000639	GO:003424	GO:0034246	mitochondrial sequence-specific DNA-bindin
Cbp2p	Required	GO:000037	GO:000573	GO:0003723	RNA binding;GO:0003676 nucleic acid bindin
ferroxida	Multicopp	GO:003321	GO:000032	GO:0005381	iron ion transmembrane transporter activit
putative	Integral	GO:000650	GO:000578	GO:000458	(sce)GPI-anchor biosynthesis, core oligosacch
None	None	None	None	None	None
alpha-1,2	Alpha-1,2	GO:004635	GO:000013	GO:000002	(sce)N-glycan biosynthesis, high-mannose type
mitogen-a	MAP kinas	GO:000723	GO:000593	GO:000470	(sce04011)MAPK signaling pathway - yeast
2,5-diami	Diaminohy	GO:000923	GO:000557	GO:000870	(sce00740)Riboflavin metabolism; (sce01100)M
CCR4-NOT	Subunit c	GO:001060	GO:003001	GO:000167	(sce03018)RNA degradation
Emc1p	Member of	GO:003497	GO:007254	GO:0003674	molecular_function
putative	Probable	GO:001940	GO:000578	GO:0102389	polyprenol reductase activity;GO:0003865 3
RNase P/M	Subunit c	GO:003496	GO:000565	GO:003320	(sce03008)Ribosome biogenesis in eukaryotes;
isoleucin	Mitochond	GO:007015	GO:000575	GO:000482	(sce00970)Aminoacyl-tRNA biosynthesis
hypotheti	Putative	GO:000815	GO:000557	GO:0003674	molecular_function
None	None	None	None	None	None
glucose-i	GID Compl	GO:004572	GO:003465	GO:0003674	molecular_function
phosphati	Phosphati	GO:190135	GO:000576	GO:0008526	phosphatidylinositol transporter activity;
Rch1p	Putative	GO:001591	GO:007194	GO:0015248	sterol transporter activity;GO:0005319 lip
kinase-re	Nuclear r	GO:006140	GO:000582	GO:0000156	phosphorelay response regulator activity;G
Cin5p	Basic leu	GO:004253	GO:000563	GO:0070491	repressing transcription factor binding;GO
Shy1p	Mitochond	GO:003361	GO:000574	GO:0051082	unfolded protein binding;GO:0005515 protei
mitochond	Mitochond	GO:003254	GO:000576	GO:0003735	structural constituent of ribosome;GO:0005
hypotheti	Putative	GO:000815	GO:001602	GO:0003674	molecular_function
None	None	None	None	None	None
None	None	None	None	None	None
None	None	None	None	None	None
saccharop	Saccharop	GO:001987	GO:007194	GO:000475	(M00030)Lysine biosynthesis, AAA pathway, 2-c
thiosulfa	Protein w	GO:000815	GO:000573	GO:0004792	thiosulfate sulfurtransferase activity;GO:
Cse2p	Subunit c	GO:005112	GO:007084	GO:0003712	transcription coregulator activity;GO:0005
Mrm1p	Ribose me	GO:003116	GO:000573	GO:0008989	rRNA (guanine-N1-)-methyltransferase activ
tricalbin	Lipid-bin	GO:006030	GO:003254	GO:0005544	calcium-dependent phospholipid binding;GO:
Asg1p	Zinc clus	GO:004594	GO:000563	GO:0000981	DNA-binding transcription factor activity,
guanine n	Subunit c	GO:007170	GO:000583	GO:003168	(sce04011)MAPK signaling pathway - yeast
Asi2p	Subunit c	GO:003630	GO:009765	GO:0003674	molecular_function
Golgi tra	Component	GO:003225	GO:001711	GO:0003674	molecular_function
pyruvate	E1 alpha	GO:000608	GO:000596	GO:000473	(M00307)Pyruvate oxidation, pyruvate => acet
protein f	Beta subu	GO:001834	GO:000596	GO:000466	(sce00900)Terpenoid backbone biosynthesis; (
Arg81p	Zinc fing	GO:000082	GO:000563	GO:0000978	RNA polymerase II proximal promoter sequen
Cti6p	Component	GO:006118	GO:003369	GO:0035064	methylated histone binding;GO:0140034 meth
None	None	None	None	None	None

Mrx1p	Protein i	GO:00081	GO:00057	GO:0003674 molecular_function	
proteina	Vacuolar	GO:00066	GO:00003	GO:00041	(sce04138)Autophagy - yeast
Nfu1p	Protein i	GO:00068	GO:00057	GO:0051539 4 iron, 4 sulfur cluster binding;GO:005153	
glutathi	Possible	GO:00617	GO:00104	GO:0019172 glyoxalase III activity;GO:0016836 hydro-l	
malate d	Peroxison	GO:00067	GO:19904	GO:00300	(M00012)Glyoxylate cycle; (sce)Glyoxylate cy
mediator	Subunit	GO:00511	GO:00708	GO:0003712 transcription coregulator activity;GO:0140	
Irc23p	Protein	GO:00081	GO:00057	GO:0003674 molecular_function	
hypothet	Putative	GO:00081	GO:00056	GO:0043565 sequence-specific DNA binding;GO:0003677 D	
ribosoma	Protein	GO:00064	GO:00226	GO:00037	(sce03010)Ribosome
Mef2p	Mitochon	GO:00327	GO:00057	GO:0003746 translation elongation factor activity;GO:	
Y' elemen	Putative	GO:00081	GO:00055	GO:0004386 helicase activity;GO:0017111 nucleoside-tr	
Ids2p	Protein i	GO:00513	GO:00056	GO:0003674 molecular_function	
phosphat	Polyphos	GO:00468	GO:00304	GO:00438	(sce00562)Inositol phosphate metabolism; (sc
Taf4p	TFIID su	GO:00511	GO:00056	GO:0016251 RNA polymerase II general transcription in	
DEAH-box	DEAH-box	GO:00003	GO:00710	GO:00344	(sce03040)Spliceosome
hypothet	Cytoplasm	GO:00081	GO:00057	GO:0003674 molecular_function	
Mgr2p	Subunit	GO:00450	GO:00057	GO:0003674 molecular_function	
ribosoma	Protein	GO:00004	GO:00226	GO:00037	(sce03010)Ribosome
Ntc20p	Member o	GO:00003	GO:00710	GO:0000384 first spliceosomal transesterification act	
Gip2p	Putative	GO:00059	GO:00001	GO:0019888 protein phosphatase regulator activity;GO:	
nucleosi	Essential	GO:00469	GO:00104	GO:00040	(sce)Adenine ribonucleotide biosynthesis, IM
None	None	None	None	None	None
dynamin-	Mitochon	GO:00080	GO:00970	GO:0003924 GTPase activity;GO:0008017 microtubule bin	
phosphat	Phosphoi	GO:00709	GO:00319	GO:0005546 phosphatidylinositol-4,5-bisphosphate bind	
E3 ubiqu	RING fing	GO:00362	GO:00001	GO:0031624 ubiquitin conjugating enzyme binding;GO:00	
pseudour	Pseudour	GO:00004	GO:00056	GO:0106029 tRNA pseudouridine synthase activity;GO:00	
methylen	Protein v	GO:00090	GO:00058	GO:00044	(sce00670)One carbon pool by folate; (sce012
Nup188p	Subunit	GO:00319	GO:00446	GO:00170	(sce03013)RNA transport
None	None	None	None	None	None
Atg31p	Autophag	GO:00448	GO:19903	GO:00036	(sce04138)Autophagy - yeast
Afi1p	Arf3p po	GO:00002	GO:00059	GO:0003674 molecular_function	
mediator	Subunit	GO:00511	GO:00708	GO:0003712 transcription coregulator activity;GO:0140	
Cwp2p	Covalent	GO:00315	GO:00092	GO:0005199 structural constituent of cell wall;GO:000	
None	None	None	None	None	None
DNA-depe	Snf2p fa	GO:00708	GO:00007	GO:0008094 DNA-dependent ATPase activity;GO:0004386 h	
purine-n	Purine n	GO:00461	GO:00057	GO:00477	(sce00760)Nicotinate and nicotinamide metabo
None	None	None	None	None	None
None	None	None	None	None	None
None	None	None	None	None	None
None	None	None	None	None	None
None	None	None	None	None	None
coiled-c	Coiled-c	GO:00902	GO:00007	GO:00448	(sce04111)Cell cycle - yeast; (sce04113)Meio
None	None	None	None	None	None
None	None	None	None	None	None
hypothet	Putative	GO:00081	GO:00055	GO:0003674 molecular_function	
None	None	None	None	None	None
ribonucl	Ribonucl	GO:00431	GO:00322	GO:00045	(sce03030)DNA replication
Smy2p	GYF doma	GO:00068	GO:00009	GO:0003674 molecular_function	
hypothet	Putative	GO:00550	GO:00003	GO:0022857 transmembrane transporter activity;GO:0005	
rRNA (cy	rRNA m5C	GO:00704	GO:00306	GO:0009383 rRNA (cytosine-C5-)-methyltransferase acti	
None	None	None	None	None	None
None	None	None	None	None	None
hypothet	Protein	GO:00081	GO:00055	GO:0003674 molecular_function	
None	None	None	None	None	None

None	None	None	None	None	None
SPX domai	Low-affir	GO:200018	GO:00058	GO:0005315 inorganic phosphate transmembrane transpor	
None	None	None	None	None	None
fatty aci	Fatty aci	GO:003462	GO:003017	GO:010275	(sce)Fatty acid biosynthesis, elongation, en
Tap42p	Essential	GO:004594	GO:001989	GO:005172	(sce04136)Autophagy - other; (sce04138)Autopl
Srb6p	Subunit (	GO:004594	GO:007084	GO:0003712 transcription coregulator activity;GO:0140	
None	None	None	None	None	None
bifunctic	Uridylate	GO:000620	GO:000563	GO:000412	(sce)Pyrimidine ribonucleotide biosynthesis,
Izh4p	Membrane	GO:000688	GO:000032	GO:0038023 signaling receptor activity;GO:0060089 mol	
L-methior	Methionir	GO:003459	GO:000582	GO:007019	(sce00270)Cysteine and methionine metabolism
AAA famil	AAA-perox	GO:001656	GO:000577	GO:001688	(sce04146)Peroxisome
putative	Cell wall	GO:000074	GO:000927	GO:0042973 glucan endo-1,3-beta-D-glucosidase activit	
hypotheti	Protein (	GO:000815	GO:000032	GO:0003674 molecular_function	
bifunctic	sn-1,2-di	GO:000665	GO:000013	GO:000414	(sce)Phosphatidylethanolamine (PE) biosynthe:
hypotheti	Putative	GO:000815	GO:000557	GO:0003674 molecular_function	
hypotheti	Putative	GO:000815	GO:000557	GO:0003674 molecular_function	
Gpb2p	Multister	GO:001025	GO:000573	GO:0004862 cAMP-dependent protein kinase inhibitor ac	
cytochror	Cytochror	GO:001802	GO:000582	GO:000027	(sce00310)Lysine degradation
Hop	Site-spec	GO:000072	GO:000563	GO:0004519 endonuclease activity;GO:0004518 nuclease	
phosphate	Basic hel	GO:001603	GO:000563	GO:000371	(sce04111)Cell cycle - yeast
calnexin	Calnexin;	GO:003043	GO:003017	GO:005108	(sce04145)Phagosome; (sce04141)Protein proce:
Mcm21p	Component	GO:190526	GO:000081	GO:0003674 molecular_function	
Eos1p	Protein i	GO:000648	GO:000578	GO:0003674 molecular_function	
Elf1p	Transcrip	GO:004809	GO:000802	GO:0000993 RNA polymerase II complex binding;GO:00431	
Dad1p	Essential	GO:005198	GO:004272	GO:0051010 microtubule plus-end binding;GO:0008017 mi	
5'-(N(7)-	Non-esser	GO:190191	GO:000093	GO:004469	(sce03018)RNA degradation
polyadeny	Poly(A) k	GO:006021	GO:001049	GO:000842	(sce03015)mRNA surveillance pathway; (sce030
prefoldir	Subunit (	GO:003296	GO:001627	GO:0051082 unfolded protein binding;GO:0005515 protei	
Bos1p	v-SNARE	GO:004828	GO:001250	GO:000548	(sce04130)SNARE interactions in vesicular tra
Hos4p	Subunit (	GO:001657	GO:003496	GO:0003674 molecular_function	
None	None	None	None	None	None
iron-sulf	Component	GO:001622	GO:009736	GO:0003674 molecular_function	
Tma20p	Protein (	GO:000218	GO:002262	GO:0043024 ribosomal small subunit binding;GO:0043021	
Rsc30p	Component	GO:000633	GO:001658	GO:0015616 DNA translocase activity;GO:0008094 DNA-de	
glutathic	Phosphol	GO:009886	GO:003131	GO:004706	(sce00590)Arachidonic acid metabolism; (sce0
None	None	None	None	None	None
metallo-a	Aminopept	GO:004317	GO:003028	GO:0070006 metalloaminopeptidase activity;GO:0004177	
Nis1p	Protein localized GO:0005935 cellular bud neck;GO:0005933 cellular bud;GO:003042				
Mhp1p	Microtubu	GO:000702	GO:000587	GO:0005200 structural constituent of cytoskeleton;GO:	
None	None	None	None	None	None
Hap3p	Subunit (	GO:000043	GO:001660	GO:000097	(Human)(hsa04612)Antigen processing and pres
[acyl-car	Predicted	GO:000663	GO:000573	GO:000431	(sce)Fatty acid biosynthesis, initiation; (M
None	None	None	None	None	None
Y' elemer	Helicase	GO:000072	GO:000563	GO:0003678 DNA helicase activity;GO:0004386 helicase	
Vam6p	Guanine r	GO:003288	GO:199081	GO:000508	(sce04138)Autophagy - yeast
None	None	None	None	None	None
None	None	None	None	None	None
None	None	None	None	None	None
Vph2p	Integral	GO:000703	GO:199087	GO:0003674 molecular_function	
Atp10p	Assembly	GO:003361	GO:003259	GO:0051082 unfolded protein binding;GO:0005515 protei	
linker nu	Linker nu	GO:000005	GO:004461	GO:0017056 structural constituent of nuclear pore;GO:	
Mip6p	Putative	GO:000640	GO:001049	GO:0008143 poly(A) binding;GO:0008266 poly(U) RNA bin	
Kar1p	Protein i	GO:003047	GO:000582	GO:0005515 protein binding;GO:0005488 binding;GO:0003	
H(+)-expo	Plasma me	GO:190290	GO:004512	GO:000855	(sce00190)Oxidative phosphorylation

None	None	None	None	None	None
Kap114p	Karyophe	GO:00066(	GO:00564	GO:0008536 Ran GTPase binding;GO:0008565 protein tran	
histone (	Histone (	GO:00709:	GO:00349(	GO:0045129 NAD-independent histone deacetylase activi	
hypotheti	Dubious (	GO:00081!	GO:00160:	GO:0003674 molecular_function	
Rta1p	Protein i	GO:00081!	GO:00003:	GO:0003674 molecular_function	
Osw1p	Protein i	GO:00304	GO:00311(	GO:0003674 molecular_function	
alpha-1,:	Alpha-1,:	GO:00064!	GO:00057!	GO:000437	(sce)N-glycan precursor biosynthesis; (M0005!
cyclin CI	G1 cycli	GO:00070!	GO:00003(	GO:00165:	(sce04111)Cell cycle - yeast; (sce04113)Meio:
Fun14p	Integral	GO:00550!	GO:00313(	GO:0003674 molecular_function	
Lst7p	Subunit (	GO:19042(	GO:19908	GO:0005096 GTPase activator activity;GO:0030695 GTPas	
hypotheti	Protein (	GO:00081!	GO:00160:	GO:0003674 molecular_function	
None	None	None	None	None	None
DNA-dire(	RNA polyn	GO:00011	GO:00056(	GO:00039(	(sce03020)RNA polymerase; (sce00240)Pyrimidi
mitochon(	Mitochon(	GO:00325	GO:00057(	GO:0019843 rRNA binding;GO:0003735 structural constit	
None	None	None	None	None	None
glutathi(	Cis-golg	GO:00988(	GO:00057!	GO:0004362 glutathione-disulfide reductase activity;G	
Arp9p	Componen	GO:00427(	GO:00165!	GO:0015616 DNA translocase activity;GO:0008094 DNA-de	
Rad61p	Subunit (	GO:00341!	GO:00007!	GO:0003674 molecular_function	
centrome	Kinetoch(	GO:19017:	GO:00007	GO:0019237 centromeric DNA binding;GO:1990837 sequenc	
Mgr3p	Subunit (	GO:00065	GO:00319	GO:0051787 misfolded protein binding;GO:0005515 prote	
ribosoma	Ribosoma	GO:00000:	GO:00226:	GO:00701!	(sce03010)Ribosome
H(+)-tra	Subunit a	GO:00070:	GO:00002:	GO:00469(	(sce04145)Phagosome; (sce00190)Oxidative pho:
zuotin	Ribosome-	GO:00000!	GO:00058	GO:0043022 ribosome binding;GO:0043021 ribonucleoprot	
Irc21p	Protein (	GO:00069	GO:00057:	GO:0020037 heme binding;GO:0046906 tetrapyrrole bindi	
hypotheti	Putative	GO:00081!	GO:00055	GO:0003674 molecular_function	
hexose t	High-aff	GO:00157!	GO:00058!	GO:00151	(sce04113)Meiosis - yeast
ubiquiti	Serine/t	GO:00450!	GO:00342	GO:00431:	(sce04136)Autophagy - other; (sce04138)Autop
Msb2p	Mucin fa	GO:00072:	GO:00304:	GO:00050:	(sce04011)MAPK signaling pathway - yeast
putative	Subunit (	GO:00159:	GO:19901	GO:00045!	(sce)Coenzyme A biosynthesis, pantothenate =
Apj1p	Chaperon(	GO:00327!	GO:00343!	GO:0001671 ATPase activator activity;GO:0060590 ATPas	
ribosoma	Protein (	GO:00459(	GO:00226:	GO:00198	(sce03010)Ribosome
Mpp6p	Nuclear (	GO:00710:	GO:00058	GO:00082(	(sce03018)RNA degradation
None	None	None	None	None	None
None	None	None	None	None	None
None	None	None	None	None	None
Ctf19p	Outer ki	GO:00070!	GO:00008	GO:0003674 molecular_function	
Mia40p	Import a	GO:00450	GO:00313(	GO:0016972 thiol oxidase activity;GO:0003756 protein	
hypotheti	Putative	GO:00081!	GO:00055	GO:0003674 molecular_function	
putative	Putative protein of unknown function; weak sequence similarity to bis (5'-nucleo				
Rrn11p	Componen	GO:00011!	GO:00708(	GO:0001164 RNA polymerase I CORE element sequence-spe	
Bud6p	Actin- a	GO:00903:	GO:00001:	GO:0005519 cytoskeletal regulatory protein binding;GO	
hypotheti	Putative	GO:00081!	GO:00056:	GO:0003674 molecular_function	
None	None	None	None	None	None
Bud27p	Unconven	GO:19901	GO:00165!	GO:0003712 transcription coregulator activity;GO:0140	
metallot	Metallot	GO:00715!	GO:00058:	GO:0046870 cadmium ion binding;GO:0004784 superoxide	
SUMO lig	SUMO E3	GO:00071:	GO:00007!	GO:0019789 SUMO transferase activity;GO:0019787 ubiqu	
None	None	None	None	None	None
Nel1p	Activato	GO:00901	GO:00301:	GO:0005096 GTPase activator activity;GO:0030695 GTPas	
hypotheti	Putative	GO:00081!	GO:00055	GO:0003674 molecular_function	
seripaup(	Protein (	GO:00315(	GO:00092	GO:0005199 structural constituent of cell wall;GO:000	
Wsc2p	Sensor-t	GO:00315(	GO:00433:	GO:00048!	(sce04139)Mitophagy - yeast; (sce04011)MAPK
None	None	None	None	None	None
hypotheti	Putative	GO:00081!	GO:00301:	GO:0003674 molecular_function	
hypotheti	Putative	GO:00081!	GO:00057:	GO:0003674 molecular_function	

rRNA (cy1	rRNA m5C	GO:00704	GO:0057	GO:0009383	rRNA (cytosine-C5-)-methyltransferase acti
homoseri	L-homose	GO:00090	GO:0057	GO:00044	(sce00270)Cysteine and methionine metabolism
Mdj2p	Constitu	GO:00301	GO:00014	GO:0001671	ATPase activator activity;GO:0060590 ATPas
hypotheti	Putative	GO:00081	GO:0055	GO:0003674	molecular_function
Tof1p	Subunit	GO:00431	GO:0312	GO:0003677	DNA binding;GO:0003676 nucleic acid bindin
None	None	None	None	None	None
None	None	None	None	None	None
beta-gluc	Protein	GO:00060	GO:0301	GO:0015926	glucosidase activity;GO:0004553 hydrolase
hypotheti	Putative	GO:00081	GO:0160	GO:0003674	molecular_function
Gic1p	Protein	GO:00708	GO:0059	GO:0017048	Rho GTPase binding;GO:0005096 GTPase activ
cyclin P	Cyclin,	GO:00311	GO:0003	GO:00165	(sce04111)Cell cycle - yeast
Edc2p	RNA-bind	GO:00320	GO:0057	GO:0003729	mRNA binding;GO:0003723 RNA binding;GO:000
putative	Predicte	GO:00109	GO:0055	GO:0004694	eukaryotic translation initiation factor 2
Mrx6p	Protein	GO:00081	GO:0057	GO:0003674	molecular_function
GET comp	Subunit	GO:00718	GO:0435	GO:0043495	protein membrane anchor;GO:0005515 protein
None	None	None	None	None	None
Moh1p	Protein	GO:00081	GO:0055	GO:0046872	metal ion binding;GO:0043169 cation bindin
Mud1p	U1 snRNP	GO:00003	GO:0056	GO:0035614	snRNA stem-loop binding;GO:0030619 U1 snRN
None	None	None	None	None	None
malate sy	Malate s	GO:00060	GO:0095	GO:00044	(M00012)Glyoxylate cycle; (sce)Glyoxylate cy
None	None	None	None	None	None
Tog1p	Transcri	GO:00459	GO:0056	GO:0000981	DNA-binding transcription factor activity,
sepiapte	Putative cytoplasmic short-chain dehydrogenase/reductase				
histone	Putative	GO:00013	GO:0708	GO:0032041	NAD-dependent histone deacetylase activity
signal re	Core comp	GO:00066	GO:0057	GO:00083	(sce03060)Protein export
chromati	Protein	GO:19000	GO:0008	GO:0001094	TFIID-class transcription factor complex b
homeodom	Transcri	GO:00073	GO:1990	GO:00435	(sce04011)MAPK signaling pathway - yeast
None	None	None	None	None	None
Spo16p	Meiosis-	GO:00105	GO:0007	GO:0003674	molecular_function
acyl car	Mitochon	GO:00091	GO:0991	GO:00000	(sce00190)Oxidative phosphorylation; (sce01
aldehyde	Mitochon	GO:00194	GO:0057	GO:00040	(sce00410)beta-Alanine metabolism; (sce00280
GDP-Man:M	Mannosyl	GO:00335	GO:0057	GO:00331	(sce)N-glycan precursor biosynthesis; (M0005
replicati	Subunit	GO:00062	GO:0313	GO:00036	(sce03430)Mismatch repair; (sce03030)DNA rep
hypotheti	Putative	GO:00081	GO:0055	GO:0008270	zinc ion binding;GO:0046914 transition met
tRNA thr	tRNA thr	GO:00615	GO:0057	GO:0061503	tRNA threonylcarbamoyladenosine dehydratas
thioredo	Thioredo	GO:00422	GO:0058	GO:0008379	thioredoxin peroxidase activity;GO:0051920
Ted1p	GPI-glyc	GO:00065	GO:0057	GO:0016787	hydrolase activity;GO:0003824 catalytic ac
tRNA met	AdoMet-d	GO:00315	GO:0057	GO:0018423	protein C-terminal leucine carboxyl O-meth
holo-[ac	Phosphop	GO:00180	GO:0057	GO:0008897	holo-[acyl-carrier-protein] synthase activ
hypotheti	Protein	GO:00081	GO:0055	GO:0003674	molecular_function
hypotheti	Putative protein of unknown function				
Pff1p	Multi-sp	GO:00065	GO:0003	GO:0008237	metallopeptidase activity;GO:0070011 pepti
Cur1p	Sorting	GO:00346	GO:0056	GO:0051087	chaperone binding;GO:0005515 protein bindi
Sgf29p	Componen	GO:00439	GO:0056	GO:0001135	RNA polymerase II transcription regulator
anaphase	Subunit	GO:00311	GO:0056	GO:00616	(sce04120)Ubiquitin mediated proteolysis; (s
ribosoma	Protein	GO:00004	GO:0226	GO:00037	(sce03010)Ribosome
Nha1p	Na+/H+ a	GO:00976	GO:0451	GO:0015385	sodium:proton antiporter activity;GO:00054
putative	Putative	GO:00065	GO:0160	GO:0004177	aminopeptidase activity;GO:0030145 mangane
alpha-1,	Alpha-1,	GO:00000	GO:0003	GO:00000	(sce00514)Other types of O-glycan biosynthes
2-dehydr	2-dehydr	GO:00333	GO:0057	GO:00086	(sce00770)Pantothenate and CoA biosynthesis;
None	None	None	None	None	None
ribonucl	Protein	GO:00016	GO:0306	GO:00045	(sce03013)RNA transport
Srf1p	Regulato	GO:00160	GO:0003	GO:0003674	molecular_function
serine/t	PAS-doma	GO:00609	GO:0057	GO:0004674	protein serine/threonine kinase activity;G

None	None	None	None	None	None
Hir3p	Subunit (	GO:00719:	GO:00004]	GO:0031491	nucleosome binding;GO:0003714 transcriptio
Dlt1p	Protein (	GO:00081!	GO:00160:	GO:0003674	molecular_function
ubiquitir	Ubiquitir	GO:00165'	GO:00055'	GO:0004843	thiol-dependent ubiquitin-specific proteas
hypotheti	Putative	GO:00081!	GO:00055'	GO:0003674	molecular_function
exosome r	Nuclear e	GO:00710:	GO:00001'	GO:00001'	(sce03018)RNA degradation
Pom152p	Glycoprot	GO:00304'	GO:00707(	GO:0043495	protein membrane anchor;GO:0017056 structu
hypotheti	Putative	GO:00081!	GO:00056:	GO:0003674	molecular_function
Ame1p	Essential	GO:00345(	GO:00008]	GO:0003674	molecular_function
Mzm1p	Protein r	GO:00345!	GO:00057!	GO:0044183	protein binding involved in protein foldin
None	None	None	None	None	None
tRNA dim(	Delta 2-i	GO:00615(	GO:00057:	GO:00523(	(sce01110)Biosynthesis of secondary metaboli
Atg14p	Autophagy	GO:00347:	GO:00342'	GO:00036'	(sce04138)Autophagy - yeast
hypotheti	Putative	GO:00081!	GO:00055'	GO:0003674	molecular_function
alcohol (	Alcohol (	GO:00434!	GO:00058(	GO:00191'	(sce00350)Tyrosine metabolism; (sce00071)Fat
Mag2p	Cytoplasn	GO:00081!	GO:00057:	GO:0046872	metal ion binding;GO:0043169 cation bindin
m-AAA prc	Mitochonc	GO:00450:	GO:00057:	GO:0004222	metalloendopeptidase activity;GO:0008237 m
hypotheti	Putative	GO:00081!	GO:00160:	GO:0003674	molecular_function
E3 ubiqui	RING fing	GO:00362(	GO:00058:	GO:0043022	ribosome binding;GO:0043021 ribonucleoprot
Opt2p	Oligopept	GO:00515]	GO:00001:	GO:0035673	oligopeptide transmembrane transporter act
snoRNA-bi	Nucleolar	GO:00004'	GO:00343(	(Human)G(	(sce03008)Ribosome biogenesis in eukaryotes
hypotheti	Putative	GO:00081!	GO:00055'	GO:0003674	molecular_function
None	None	None	None	None	None
Sea4p	Subunit (	GO:19042(	GO:00970:	GO:0003674	molecular_function
inositol	Inositol	GO:00527:	GO:00056:	GO:00352!	(sce00562)Inositol phosphate metabolism; (sc
None	None	None	None	None	None
Zds2p	Protein v	GO:00109'	GO:00059:	GO:0003674	molecular_function
translati	Catalytic	GO:00064:	GO:00320:	GO:00313(	(sce03013)RNA transport
Rco1p	Essential	GO:00601!	GO:00322:	GO:0046872	metal ion binding;GO:0043169 cation bindin
Smi1p	Protein i	GO:00329!	GO:00433:	GO:0003677	DNA binding;GO:0003676 nucleic acid bindin
ATP-deper	3'-5' DN/	GO:00007:	GO:00056:	GO:0043140	ATP-dependent 3'-5' DNA helicase activity;
Tgl5p	Bifunctic	GO:00066:	GO:00058]	GO:0042171	lysophosphatidic acid acyltransferase acti
None	None	None	None	None	None
RNA-bindi	Essential	GO:00000!	GO:00306(	GO:0003729	mRNA binding;GO:0003723 RNA binding;GO:000
None	None	None	None	None	None
None	None	None	None	None	None
seripaupe	Member of	GO:00315(	GO:00092'	GO:0005199	structural constituent of cell wall;GO:000
None	None	None	None	None	None
None	None	None	None	None	None
hypotheti	Putative	GO:00081!	GO:00055'	GO:0003674	molecular_function
threonine	Threonine	GO:00065(	GO:00057:	GO:00047!	(sce)Isoleucine biosynthesis, threonine => 2
S-adenosy	Protein l	GO:00064'	GO:00055'	GO:0008757	S-adenosylmethionine-dependent methyltrans
Esf1p	Nucleolar	GO:00063(	GO:00057:	GO:0003723	RNA binding;GO:0003676 nucleic acid bindin
dolichyl-	Protein (	GO:00717]	GO:00975(	GO:00041(	(sce00515)Mannose type O-glycan biosynthesis
ribosomal	Ribosomal	GO:00000:	GO:00226:	GO:00037:	(sce03010)Ribosome
putative	Essential	GO:00987(	GO:00058:	GO:0031624	ubiquitin conjugating enzyme binding;GO:00
Uba4p	E1-like p	GO:20002:	GO:00058:	GO:00422!	(sce04122)Sulfur relay system
None	None	None	None	None	None
None	None	None	None	None	None
glucose s	Plasma me	GO:00515!	GO:00058(	GO:00053!	(sce04113)Meiosis - yeast
Yip4p	Protein t	GO:00081!	GO:00001:	GO:0017137	Rab GTPase binding;GO:0017016 Ras GTPase b
Isa2p	Protein r	GO:00091(	GO:00057!	GO:0051537	2 iron, 2 sulfur cluster binding;GO:005153
protein t	Essential	GO:00301!	GO:00014(	GO:0051087	chaperone binding;GO:0030674 protein bindi
securin	Securin;	GO:00011(	GO:00058]	GO:00198!	(sce04111)Cell cycle - yeast; (sce04113)Meio

ubiquitir	ESCRT-I s	GO:190466	GO:00081	GO:00431	(sce04144)Endocytosis
ubiquitir	Ubiquitir	GO:00065	GO:00057	GO:0031624 ubiquitin conjugating enzyme binding;GO:00	
recombina	Meiosis-s	GO:00007(	GO:00007	GO:00420	(sce04113)Meiosis - yeast
bifunctio	High-affi	GO:190196	GO:00057	GO:0015489 putrescine transmembrane transporter activ	
mRNA spli	Protein i	GO:00003	GO:00710(	GO:00003	(sce03040)Spliceosome
Apm1p	Mu1-like	GO:00068	GO:00301	GO:00302	(Human)(hsa04142)Lysosome
ribosomal	Protein c	GO:00064(	GO:00226	GO:00037	(sce03010)Ribosome
orotate p	Major oro	GO:00442(	GO:00056	GO:00045	(sce)Uridine monophosphate biosynthesis, glu
putative	Putative	GO:00000	GO:00057	GO:0000026 alpha-1,2-mannosyltransferase activity;GO:	
Hxt14p	Protein w	GO:00157	GO:00003	GO:0005354 galactose transmembrane transporter activi	
hypotheti	Putative	GO:00081	GO:00055	GO:0003674 molecular_function	
transcrip	TFIIA sma	GO:00511	GO:00056	GO:00170	(sce03022)Basal transcription factors
Arf fami	Dubious c	GO:00312(	GO:00057	GO:00154	(sce03060)Protein export; (sce04145)Phagosom
SF3a spli	Subunit c	GO:00003	GO:00710(	GO:00082	(sce03040)Spliceosome
Cym1p	Lysine-sp	GO:00164	GO:00057	GO:0004222 metalloendopeptidase activity;GO:0008237 m	
hypotheti	Putative	GO:00081	GO:00055	GO:0003674 molecular_function	
Meo1p	Putative	GO:00081	GO:00057	GO:0003674 molecular_function	
ribosomal	Ribosomal	GO:19901	GO:00306	GO:00037	(sce03010)Ribosome
protein-S	Farnesyl	GO:00064	GO:00056	GO:00046	(sce00900)Terpenoid backbone biosynthesis; (
Tom40p	Component	GO:00301	GO:00057	GO:00152	(Human)(hsa05014)Amyotrophic lateral scleros
putative	Putative	GO:00094	GO:00058	GO:00084	(sce00750)Vitamin B6 metabolism; (sce01100)M
None	None	None	None	None	None
mitochond	Highly co	GO:00068	GO:00313(	GO:0050833 pyruvate transmembrane transporter activit	
type 2C p	Type 2C p	GO:00001	GO:00056	GO:1990439 MAP kinase serine/threonine phosphatase ac	
Rnh70p	3'-5' exo	GO:00344	GO:00056	GO:00001	(sce03008)Ribosome biogenesis in eukaryotes
Ede1p	Scaffold	GO:00324	GO:00304	GO:00431	(sce04144)Endocytosis
ubiquitir	Ubiquitir	GO:00347	GO:00466	GO:0004843 thiol-dependent ubiquitin-specific proteas	
DNA-direc	DNA polym	GO:00063(	GO:00056	GO:00038	(sce03450)Non-homologous end-joining
hypotheti	Putative	GO:00081	GO:00055	GO:0003674 molecular_function	
Pet127p	Protein w	GO:00009	GO:00057	GO:0003674 molecular_function	
hypotheti	Putative	GO:00081	GO:00160	GO:0003674 molecular_function	
Mcp1p	Mitochond	GO:00550	GO:00325	GO:0003674 molecular_function	
Tif11p	Translati	GO:00017	GO:00332	GO:00430	(sce03013)RNA transport
1,3-beta-	1,3-beta-	GO:00719	GO:00092	GO:0042124 1,3-beta-glucanosyltransferase activity;GO	
hypotheti	Putative	GO:00081	GO:00057	GO:0003674 molecular_function	
DNA-direc	RNA polym	GO:00011	GO:00056	GO:00039	(sce03020)RNA polymerase; (sce00240)Pyrimidi
ribosomal	Ribosomal	GO:00021	GO:00226	GO:00198	(sce03010)Ribosome
Cft1p	RNA-bindi	GO:00987	GO:00058	GO:00037	(sce03015)mRNA surveillance pathway
None	None	None	None	None	None
Ivy1p	Phosphol	GO:00421	GO:00003	GO:0005543 phospholipid binding;GO:0008289 lipid bind	
Ast1p	Lipid ra	GO:00325	GO:00057	GO:0016491 oxidoreductase activity;GO:0003824 catalyt	
DNA repli	Subunit c	GO:00007	GO:00711	GO:0043138 3'-5' DNA helicase activity;GO:0003678 DNA	
H(+)-tran	Subunit I	GO:00070	GO:00002	GO:00469	(sce04145)Phagosome; (sce00190)Oxidative pho
ribosomal	Protein c	GO:00000	GO:00306	GO:00085	(sce03010)Ribosome
hypotheti	Putative transcription factor containing a WOPR domain; binds DNA in vitro; simi				
Gcr2p	Transcrip	GO:00723	GO:00056	GO:0001102 RNA polymerase II activating transcription	
H(+)-tran	Vacuolar	GO:00070	GO:00002	GO:0042625 ATPase coupled ion transmembrane transport	
DNA-bindi	Essential	GO:00723	GO:00701	GO:0001094 TFIID-class transcription factor complex b	
Rcr2p	Vacuolar	GO:00161	GO:00003	GO:0003674 molecular_function	
None	None	None	None	None	None
Taf6p	Subunit	GO:00511	GO:00466	GO:00162	(sce03022)Basal transcription factors
Tar1p	Dubious c	GO:00434	GO:00057	GO:0003674 molecular_function	
ribosomal	Protein c	GO:00004	GO:00226	GO:00037	(sce03010)Ribosome
glycerol	Glycerol	GO:00195	GO:00058	GO:00479	(sce00561)Glycerolipid metabolism; (sce01100)

double-st	Double-st	GO:00320	GO:00057	GO:0033676	double-stranded DNA-dependent ATPase activ
dethiobio	Dethiobio	GO:00091	GO:00057	GO:00041	(sce00780)Biotin metabolism; (sce01100)Metab
hypotheti	Putative	GO:00081	GO:00055	GO:0003674	molecular_function
hypotheti	Putative	GO:00081	GO:00057	GO:0005509	calcium ion binding;GO:0046872 metal ion b
Met18p	Component	GO:00974	GO:00973	GO:0003674	molecular_function
putative	Putative	GO:00158	GO:00056	GO:0004674	protein serine/threonine kinase activity;G
None	None	None	None	None	None
methionin	Methionin	GO:00355	GO:00104	GO:0070006	metalloaminopeptidase activity;GO:0004177
ribosomal	Ribosomal	GO:00021	GO:00226	GO:00037	(sce03010)Ribosome
Cbt1p	Protein i	GO:00482	GO:00057	GO:0003674	molecular_function
hypotheti	Protein p	GO:00163	GO:00056	GO:0016791	phosphatase activity;GO:0042578 phosphoric
None	None	None	None	None	None
Fmp25p	Protein r	GO:00345	GO:00313	GO:0003674	molecular_function
Pbp2p	RNA bindi	GO:00007	GO:00056	GO:0003729	mRNA binding;GO:0003723 RNA binding;GO:000
Ahc1p	Subunit c	GO:19904	GO:00056	GO:0004402	histone acetyltransferase activity;GO:0061
None	None	None	None	None	None
Etp1p	Protein c	GO:00454	GO:00057	GO:00081	(Human)(hsa04014)Ras signaling pathway
indolepyr	Major of	GO:00009	GO:00058	GO:00501	(sce00010)Glycolysis / Gluconeogenesis; (sce
Vam7p	Vacuolar	GO:00619	GO:00003	GO:00322	(sce04130)SNARE interactions in vesicular tr
Shg1p	Subunit c	GO:00515	GO:00481	GO:0042800	histone methyltransferase activity (H3-K4
cohesin s	Subunit c	GO:00071	GO:00349	GO:00168	(sce04111)Cell cycle - yeast; (sce04113)Meio
Nkp1p	Central k	GO:00513	GO:00007	GO:0003674	molecular_function
anaphase	Subunit c	GO:00458	GO:00056	GO:00199	(sce04120)Ubiquitin mediated proteolysis; (s
palmitoyl	Palmitoyl	GO:00182	GO:00057	GO:0019706	protein-cysteine S-palmitoyltransferase ac
hypotheti	Putative	GO:00081	GO:00160	GO:0003674	molecular_function
hypotheti	Putative	GO:00081	GO:00055	GO:0003674	molecular_function
Tma19p	Protein t	GO:00021	GO:00104	GO:0043023	ribosomal large subunit binding;GO:0043024
None	None	None	None	None	None
PAQR-type	Plasma me	GO:00068	GO:00003	GO:00380	(Human)(hsa04920)Adipocytokine signaling pat
mRNA guan	Guanylylt	GO:00063	GO:00315	GO:0004484	mRNA guanylyltransferase activity;GO:00081
ADP/ATP c	Mitochond	GO:00158	GO:00057	GO:0005471	ATP:ADP antiporter activity;GO:0015217 ADP
None	None	None	None	None	None
sulfonate	Fe(II)-de	GO:00463	GO:00057	GO:0000907	sulfonate dioxygenase activity;GO:0000908
cyclin-de	Cyclin-de	GO:00457	GO:19908	GO:00046	(sce04138)Autophagy - yeast; (sce04111)Cell
None	None	None	None	None	None
hypotheti	Protein t	GO:00007	GO:00703	GO:0003690	double-stranded DNA binding;GO:0003723 RNA
Urn1p	Protein c	GO:00452	GO:00056	GO:0003723	RNA binding;GO:0003676 nucleic acid bindin
TORC2 com	Subunit c	GO:00309	GO:00319	GO:0005085	guanyl-nucleotide exchange factor activity
protein p	Regulator	GO:00902	GO:00001	GO:00047	(sce03015)mRNA surveillance pathway; (sce041
Golgi tra	Component	GO:00322	GO:00171	GO:0003674	molecular_function
Aim46p	Protein c	GO:00081	GO:00057	GO:0016872	intramolecular lyase activity;GO:0016853 i
ubiquitin	Ubiquitin	GO:00315	GO:00321	GO:0004842	ubiquitin-protein transferase activity;GO:
RNA-bindi	Subunit c	GO:00066	GO:00057	GO:00083	(sce03060)Protein export
Bre5p	Ubiquitin	GO:00345	GO:19908	GO:00037	(sce04139)Mitophagy - yeast
Met31p	Zinc-fing	GO:00313	GO:00056	GO:0000987	proximal promoter sequence-specific DNA bi
alpha-ket	Subunit c	GO:00061	GO:00093	GO:00045	(sce00310)Lysine degradation; (sce00380)Tryp
mitochond	Mitochond	GO:00325	GO:00057	GO:00037	(sce03010)Ribosome
dodecenoy	Peroxisom	GO:00066	GO:00057	GO:00041	(sce00071)Fatty acid degradation; (sce04146)
gluconoki	Putative	GO:00461	GO:00003	GO:00463	(sce00030)Pentose phosphate pathway; (sce012
Sec63 com	Essential	GO:00312	GO:00312	GO:00085	(sce03060)Protein export; (sce04141)Protein
cytochrom	Mitochond	GO:00427	GO:00057	GO:0004130	cytochrome-c peroxidase activity;GO:000460
RNA-bindi	Essential	GO:00004	GO:00057	GO:0001671	ATPase activator activity;GO:0060590 ATPas
thioredox	Cytoplasm	GO:00194	GO:00057	GO:00047	(sce00450)Selenocompound metabolism; (sce002
signal pe	Subunit c	GO:00064	GO:00057	GO:00082	(sce03060)Protein export

putative	Putative	GO:00059	GO:00056	GO:0004722	protein serine/threonine phosphatase activ
Mic26p	Component	GO:00424	GO:00616	GO:0003674	molecular_function
ADP/ATP	Mitochon	GO:00158	GO:00057	GO:0005471	ATP:ADP antiporter activity;GO:0015217 ADP
bifuncti	Enzyme o	GO:00061	GO:00058	GO:00039	(M00048)Inosine monophosphate biosynthesis,
None	None	None	None	None	None
hypotheti	Protein	GO:00081	GO:00160	GO:0003674	molecular_function
None	None	None	None	None	None
None	None	None	None	None	None
hypotheti	Putative	GO:00081	GO:00055	GO:0003674	molecular_function
putative	Putative	GO:00000	GO:00057	GO:0008408	3'-5' exonuclease activity;GO:0004527 exon
tyrosine	Dual spe	GO:00000	GO:00104	GO:1990275	preribosome binding;GO:0008138 protein tyr
hypotheti	Putative	GO:00081	GO:00057	GO:0003674	molecular_function
None	None	None	None	None	None
None	None	None	None	None	None
oligo-1,	Alpha-gl	GO:00000	GO:00057	GO:00045	(sce00052)Galactose metabolism; (sce00500)St
None	None	None	None	None	None
hypotheti	Putative	GO:00550	GO:00057	GO:0022857	transmembrane transporter activity;GO:0005
phosphop	Enolase,	GO:00060	GO:00000	GO:00046	(M00002)Glycolysis, core module involving th
Mdm35p	Mitochon	GO:00453	GO:00057	GO:0005515	protein binding;GO:0005488 binding;GO:0003
Tvp38p	Integral	GO:00000	GO:00301	GO:0003674	molecular_function
Api2p	Putative	GO:00081	GO:00055	GO:0003674	molecular_function
sulfate	High aff	GO:19023	GO:00058	GO:0008271	secondary active sulfate transmembrane tra
None	None	None	None	None	None
SKI comp	Ski comp	GO:00704	GO:00550	GO:00036	(sce03018)RNA degradation
Rbg2p	Protein	GO:19038	GO:00057	GO:0005525	GTP binding;GO:0032550 purine ribonucleosi
S-adenos	S-adenos	GO:00180	GO:00057	GO:0071885	N-terminal protein N-methyltransferase act
ribosoma	Protein	GO:00000	GO:00226	GO:00198	(sce03010)Ribosome
mitochon	Mitochon	GO:00325	GO:00057	GO:00037	(sce03010)Ribosome
sterol e	Steryl e	GO:00000	GO:00058	GO:0004771	sterol esterase activity;GO:0016298 lipase
Rts2p	Basic zi	GO:00062	GO:00056	GO:0008270	zinc ion binding;GO:0003690 double-strande
metallo-	Putative	GO:00081	GO:00058	GO:0003876	AMP deaminase activity;GO:0047623 adenosin
DNA repa	Nucleoti	GO:00007	GO:00001	GO:0008094	DNA-dependent ATPase activity;GO:0003684 d
Pml1p	Subunit	GO:00512	GO:00702	GO:0003729	mRNA binding;GO:0003723 RNA binding;GO:000
None	None	None	None	None	None
Tgl4p	Multifun	GO:00066	GO:00058	GO:00474	(sce00592)alpha-Linolenic acid metabolism;
None	None	None	None	None	None
hypotheti	Putative	GO:00081	GO:00055	GO:0003674	molecular_function
Spo22p	Meiosis-	GO:00901	GO:00007	GO:0003674	molecular_function
cyclin-d	Cyclin-d	GO:00603	GO:00003	GO:00615	(sce04111)Cell cycle - yeast
None	None	None	None	None	None
uracil p	Plasma m	GO:19037	GO:00003	GO:0015505	uracil:cation symporter activity;GO:00152
hypotheti	Putative	GO:00081	GO:00057	GO:0003674	molecular_function
aspartat	Aspartic	GO:00090	GO:00058	GO:00040	(sce00261)Monobactam biosynthesis; (M00018)T
ribosoma	Protein	GO:00004	GO:00226	GO:00037	(sce03010)Ribosome
hypotheti	Putative	GO:00081	GO:00056	GO:0003674	molecular_function
serine/t	Dual-spe	GO:00070	GO:00007	GO:00047	(sce04111)Cell cycle - yeast
S-formyl	Esterase	GO:00462	GO:00058	GO:00187	(sce00680)Methane metabolism; (sce01200)Carb
transloc	Subunit	GO:00312	GO:00712	GO:00154	(sce03060)Protein export; (sce04145)Phagosom
Tad3p	Subunit	GO:00021	GO:00527	GO:0052717	tRNA-specific adenosine-34 deaminase activ
hypotheti	Putative	GO:00081	GO:00319	GO:0003674	molecular_function
None	None	None	None	None	None
hypotheti	Dubious	GO:00081	GO:00160	GO:0003674	molecular_function
Rad23p	Protein	GO:00304	GO:00001	GO:00002	(sce03420)Nucleotide excision repair; (sce04
None	None	None	None	None	None

RNA lari	RNA lari	GO:00071	GO:00056	GO:0008419 RNA lariat debranching enzyme activity;GO:	
casein k	Beta regu	GO:00063	GO:00344	GO:00302	(sce04139)Mitophagy - yeast; (sce03008)Ribos
None	None	None	None	None	None
pheromon	Pheromon	GO:00454	GO:00003	GO:0015035 protein disulfide oxidoreductase activity;	
mitochon	Mitochon	GO:00325	GO:00057	GO:0003735 structural constituent of ribosome;GO:0005	
bifuncti	Enzyme o	GO:00061	GO:00058	GO:00046	(M00048)Inosine monophosphate biosynthesis,
Aim4p	Protein	GO:00081	GO:00057	GO:0003674 molecular_function	
None	None	None	None	None	None
D-amino-	D-Amino	GO:00704	GO:00056	GO:00478	(sce00360)Phenylalanine metabolism
Pcl5p	Cyclin;	GO:00162	GO:00003	GO:00165	(sce04138)Autophagy - yeast
Dsc2p	Multi-tr	GO:00081	GO:00446	GO:0003674 molecular_function	
N-termin	AdoMet-d	GO:00180	GO:00058	GO:0071885 N-terminal protein N-methyltransferase act	
None	None	None	None	None	None
hypothet	Putative	GO:00081	GO:00055	GO:0003674 molecular_function	
hypothet	Putative	GO:00157	GO:00003	GO:0015499 formate transmembrane transporter activity	
trifunct	Uridine	GO:00062	GO:00058	GO:00454	(sce00760)Nicotinate and nicotinamide metabo
GTP cycl	GTP-cycl	GO:00359	GO:00056	GO:00039	(M00126)Tetrahydrofolate biosynthesis, GTP =
hypothet	Putative	GO:00081	GO:00160	GO:0003674 molecular_function	
Mig3p	Transcri	GO:00107	GO:00007	GO:0001227 DNA-binding transcription repressor activi	
Psp1p	Asn and	GO:00081	GO:00009	GO:0003729 mRNA binding;GO:0003723 RNA binding;GO:000	
None	None	None	None	None	None
dolichol	Dolichol	GO:00430	GO:00301	GO:00041	(sce00510)N-Glycan biosynthesis; (sce01100)M
Cms1p	Putative	GO:00081	GO:00306	GO:0003674 molecular_function	
RNA exon	Putative	GO:00903	GO:00057	GO:0004527 exonuclease activity;GO:0004519 endonuclea	
multifun	5’ to 3’	GO:00007	GO:00057	GO:19041	(sce03450)Non-homologous end-joining; (sce03
ubiquiti	F-box pr	GO:00193	GO:00432	GO:00431	(sce04120)Ubiquitin mediated proteolysis; (s
hypothet	Putative	GO:00081	GO:00055	GO:0003674 molecular_function	
Taf10p	Subunit	GO:00511	GO:00466	GO:00162	(sce03022)Basal transcription factors
mitogen-	Cdc42p-a	GO:19908	GO:00433	GO:00440	(sce04392)Hippo signaling pathway -multiple
hypothet	Putative protein of unknown function; green fluorescent protein (GFP)-fusion pro				
Nop16p	Constitu	GO:00422	GO:00306	GO:0003674 molecular_function	
Lag2p	Protein	GO:00514	GO:00057	GO:0003674 molecular_function	
Bur2p	Cyclin f	GO:00515	GO:00003	GO:0016538 cyclin-dependent protein serine/threonine	
DNA-dire	RNA poly	GO:00427	GO:00056	GO:00010	(sce03020)RNA polymerase; (sce00240)Pyrimidi
ribosoma	Ribosoma	GO:00021	GO:00226	GO:00037	(sce03010)Ribosome
Elongato	Subunit	GO:00020	GO:00335	GO:0000049 tRNA binding;GO:0003723 RNA binding;GO:000	
aminopep	Mitochon	GO:00508	GO:00313	GO:0004177 aminopeptidase activity;GO:0030145 mangane	
Nop6p	rRNA-bin	GO:00422	GO:00306	GO:0030515 snoRNA binding;GO:0019843 rRNA binding;GO:	
putative	Putative	GO:00064	GO:00057	GO:0000033 alpha-1,3-mannosyltransferase activity;GO:	
3-methyl-	Ketopant	GO:00159	GO:00057	GO:00038	(sce00770)Pantothenate and CoA biosynthesis;
putative	Protein	GO:00065	GO:00058	GO:0043022 ribosome binding;GO:0043021 ribonucleoprot	
hypothet	Putative	GO:00193	GO:00055	GO:0003674 molecular_function	
None	None	None	None	None	None
Rab fami	Rab fami	GO:19902	GO:00340	GO:00001	(Human)(hsa05134)Legionellosis
hypothet	Protein	GO:00081	GO:00058	GO:0003674 molecular_function	
Nvj2p	Lipid-bi	GO:00068	GO:00715	GO:0043022 ribosome binding;GO:0043021 ribonucleoprot	
ribosoma	Ribosoma	GO:00064	GO:00226	GO:00037	(sce03010)Ribosome
hypothet	Putative	GO:00081	GO:00055	GO:0003674 molecular_function	
Ssy1p	Componen	GO:19038	GO:00057	GO:0015181 arginine transmembrane transporter activit	
Cef1p	Essentia	GO:00003	GO:00710	GO:00003	(sce03040)Spliceosome
Aim32p	Protein	GO:00081	GO:00055	GO:0003674 molecular_function	
hypothet	Putative	GO:00081	GO:00057	GO:0003674 molecular_function	
Rrn6p	Componen	GO:00011	GO:00708	GO:0001164 RNA polymerase I CORE element sequence-spe	
Nip100p	Large su	GO:00001	GO:00002	GO:0003774 motor activity;GO:0008017 microtubule bind	

Rrt6p	Putative	GO:00081[illegible]	GO:00160[illegible]	GO:0003674	molecular_function
Vps68p	Vacuolar	GO:00325[illegible]	GO:00344[illegible]	GO:0003674	molecular_function
None	None	None	None	None	None
hypotheti	Putative	GO:00068[illegible]	GO:00057[illegible]	GO:0022857	transmembrane transporter activity;GO:0005
oxygen-de	Protopor	GO:00067[illegible]	GO:00313[illegible]	GO:00047[illegible]	(sce00860)Porphyrin and chlorophyll metaboli
putative	Essentia	GO:00311[illegible]	GO:00306[illegible]	GO:0052909	18S rRNA (adenine(1779)-N(6)/adenine(1780)
SUMO liga	SUMO E3	GO:19906[illegible]	GO:00007[illegible]	GO:0019789	SUMO transferase activity;GO:0019787 ubiqu
septin CI	Componen	GO:00009[illegible]	GO:00001[illegible]	GO:00103[illegible]	(Human)(hsa05100)Bacterial invasion of epith
Snt309p	Member o	GO:00003[illegible]	GO:00710[illegible]	GO:0000384	first spliceosomal transesterification act
Cex1p	Componen	GO:00064[illegible]	GO:00056[illegible]	GO:0000049	tRNA binding;GO:0003723 RNA binding;GO:000
GMP synt	GMP synt	GO:00061[illegible]	GO:00058[illegible]	GO:00039[illegible]	(sce)Guanine ribonucleotide biosynthesis IMP
Hsp90 co	Chaperon	GO:00004[illegible]	GO:00056[illegible]	GO:0051082	unfolded protein binding;GO:0005515 protei
ribosoma	Ribosoma	GO:00021[illegible]	GO:00306[illegible]	GO:00198[illegible]	(sce03010)Ribosome
nucleosi	High aff	GO:00342[illegible]	GO:00003[illegible]	GO:00342[illegible]	(Human)(hsa05034)Alcoholism
phosphati	Protein	GO:00065[illegible]	GO:00005[illegible]	GO:00171[illegible]	(sce00563)Glycosylphosphatidylinositol (GPI)
NADHX deh	Widely-c	GO:00464[illegible]	GO:00057[illegible]	GO:0047453	ATP-dependent NAD(P)H-hydrate dehydratase
DNA 5'-a	DNA 5' A	GO:00000[illegible]	GO:00056[illegible]	GO:0033699	DNA 5'-adenosine monophosphate hydrolase a
coatomer	Delta su	GO:00483[illegible]	GO:00301[illegible]	GO:0003674	molecular_function
hypotheti	Putative	GO:00081[illegible]	GO:00055[illegible]	GO:0003674	molecular_function
prohibiti	Subunit	GO:00013[illegible]	GO:19904[illegible]	GO:0003674	molecular_function
None	None	None	None	None	None
uroporphy	Uroporph	GO:00067[illegible]	GO:00055[illegible]	GO:00048[illegible]	(sce00860)Porphyrin and chlorophyll metaboli
lysophos	Broad-sp	GO:00464[illegible]	GO:00057[illegible]	GO:00471[illegible]	(sce00565)Ether lipid metabolism; (sce00561)
Rab GTPa	Essentia	GO:00905[illegible]	GO:00056[illegible]	GO:0017137	Rab GTPase binding;GO:0017016 Ras GTPase b
hypotheti	Protein	GO:00081[illegible]	GO:00055[illegible]	GO:0003674	molecular_function
Rtc5p	Protein	GO:00081[illegible]	GO:00057[illegible]	GO:0003674	molecular_function
None	None	None	None	None	None
Gfd1p	Coiled-c	GO:00064[illegible]	GO:00056[illegible]	GO:0003674	molecular_function
RNA-bind	GTPase t	GO:00000[illegible]	GO:00306[illegible]	GO:00039[illegible]	(sce03008)Ribosome biogenesis in eukaryotes
Y' eleme	Putative	GO:00081[illegible]	GO:00055[illegible]	GO:0004386	helicase activity;GO:0017111 nucleoside-tr
None	None	None	None	None	None
ribosoma	Protein	GO:00064[illegible]	GO:00226[illegible]	GO:00701[illegible]	(sce03010)Ribosome
hypotheti	Putative	GO:00081[illegible]	GO:00055[illegible]	GO:0003674	molecular_function
Ilm1p	Protein	GO:00000[illegible]	GO:00057[illegible]	GO:0003674	molecular_function
hypotheti	Putative	GO:00459[illegible]	GO:00056[illegible]	GO:0000981	DNA-binding transcription factor activity,
protein	Mitochon	GO:00450[illegible]	GO:00427[illegible]	GO:0008565	protein transporter activity;GO:0005215 tr
translati	Beta sub	GO:00017[illegible]	GO:00436[illegible]	GO:00313[illegible]	(sce03013)RNA transport
borate t	Multidru	GO:00354[illegible]	GO:00057[illegible]	GO:0080139	borate efflux transmembrane transporter ac
hypotheti	Putative	GO:00709[illegible]	GO:00057[illegible]	GO:0043022	ribosome binding;GO:0043021 ribonucleoprot
ribosome-	Mitochon	GO:00345[illegible]	GO:00057[illegible]	GO:00430[illegible]	(sce04139)Mitophagy - yeast
Kip1p	Kinesin-	GO:00305[illegible]	GO:00058[illegible]	GO:0008574	ATP-dependent microtubule motor activity,
NAD-depe	NAD(+)-d	GO:00715[illegible]	GO:19023[illegible]	GO:00171[illegible]	(sce00760)Nicotinate and nicotinamide metabo
Cox20p	Mitochon	GO:00336[illegible]	GO:00057[illegible]	GO:0051082	unfolded protein binding;GO:0005515 protei
hypotheti	Putative	GO:00081[illegible]	GO:00003[illegible]	GO:0003674	molecular_function
Ca(2+)/M	High aff	GO:00300[illegible]	GO:00001[illegible]	GO:0015410	manganese-transporting ATPase activity;GO:
NuA4 his	Catalyti	GO:00012[illegible]	GO:00327[illegible]	GO:00104[illegible]	(Human)(hsa05166)HTLV-I infection
None	None	None	None	None	None
chaperon	Member o	GO:00349[illegible]	GO:00725[illegible]	GO:0003674	molecular_function
Mcx1p	Non-prot	GO:19042[illegible]	GO:00057[illegible]	GO:0004176	ATP-dependent peptidase activity;GO:004262
DEAH-box	RNA-depe	GO:00003[illegible]	GO:00710[illegible]	GO:00344[illegible]	(sce03040)Spliceosome
omega-cl	Omega-cl	GO:00988[illegible]	GO:00057[illegible]	GO:0045174	glutathione dehydrogenase (ascorbate) acti
H(+)-tra	Subunit	GO:19029[illegible]	GO:00002[illegible]	GO:00469[illegible]	(sce04145)Phagosome; (sce00190)Oxidative pho
ssRNA ex	Nuclear	GO:19014[illegible]	GO:00057[illegible]	GO:00045[illegible]	(sce03018)RNA degradation; (sce03008)Ribosom
Spo75p	Meiosis-	GO:00304[illegible]	GO:00058[illegible]	GO:0005227	calcium activated cation channel activity;

None	None	None	None	None	None
putative	Putative	GO:00510	GO:00056	GO:0008568 microtubule-severing ATPase activity;GO:00	
Taf2p	TFIID su	GO:00511	GO:00056	GO:0016	(sce03022)Basal transcription factors
bifuncti	3-hydrox	GO:00066	GO:00057	GO:00043	(Human)(M00104)Bile acid biosynthesis, chole
GTPase G	Mitochon	GO:00457	GO:00057	GO:0043022 ribosome binding;GO:0043021 ribonucleoprot	
ribosoma	Protein	GO:00064	GO:00226	GO:00037	(sce03010)Ribosome
hypotheti	Putative	GO:00081	GO:00055	GO:0003674 molecular_function	
Elongato	Subunit	GO:00020	GO:00335	GO:0000993 RNA polymerase II complex binding;GO:00431	
hypotheti	Putative protein of unknown function; conserved in A. gossypii; the authentic, n				
None	None	None	None	None	None
None	None	None	None	None	None
tubulin-	Subunit	GO:00070	GO:00162	GO:0044183 protein binding involved in protein foldin	
histone	Histone	GO:19033	GO:00481	GO:00428	(sce00310)Lysine degradation
None	None	None	None	None	None
None	None	None	None	None	None
hypotheti	Putative	GO:00081	GO:00055	GO:0003674 molecular_function	
None	None	None	None	None	None
deoxycyti	Deoxycyt	GO:00709	GO:00056	GO:0017125 deoxycytidyl transferase activity;GO:0003	
Sgo1p	Componen	GO:00340	GO:00007	GO:00036	(sce04113)Meiosis - yeast
hypotheti	Putative	GO:00081	GO:00055	GO:0003674 molecular_function	
Env10p	Protein involved in SRP-independent targeting of substrates to the ER; component				
Elg1p	Subunit	GO:00105	GO:00313	GO:0003682 chromatin binding;GO:0003677 DNA binding;G	
methiony	Methiony	GO:00719	GO:00057	GO:00044	(sce00670)One carbon pool by folate; (sce009
hypotheti	Protein	GO:00081	GO:00055	GO:0003674 molecular_function	
origin r	Subunit	GO:00062	GO:00056	GO:00036	(sce04111)Cell cycle - yeast; (sce04113)Meio
arginase	Arginase,	GO:00903	GO:00433	GO:00903	(sce)Polyamine biosynthesis, arginine => orn
transcri	Subunit	GO:00427	GO:00001	GO:0001002 RNA polymerase III type 1 promoter sequenc	
ribosoma	Ribosoma	GO:00000	GO:00226	GO:00198	(sce03010)Ribosome
Spc42p	Central	GO:00304	GO:0005821 intermediate layer of spindle pole body;GO:0005823		
Mrx9p	Protein	GO:00081	GO:00319	GO:0003674 molecular_function	
Snf11p	Subunit	GO:00459	GO:00165	GO:0015616 DNA translocase activity;GO:0008094 DNA-de	
translati	Beta sub	GO:00064	GO:00320	GO:00037	(sce03013)RNA transport
Bud9p	Protein	GO:00002	GO:00059	GO:0003674 molecular_function	
orotidin	Orotidin	GO:00442	GO:00058	GO:00045	(sce)Uridine monophosphate biosynthesis, glu
None	None	None	None	None	None
Sec1p	Sm-like	GO:00313	GO:00059	GO:0000149 SNARE binding;GO:0005515 protein binding;G	
leucine-	Zinc-knu	GO:20012	GO:00056	GO:0001227 DNA-binding transcription repressor activi	
None	None	None	None	None	None
hypotheti	Putative	GO:00081	GO:00055	GO:0003674 molecular_function	
dolichyl-	Protein (	GO:19001	GO:00975	GO:00041	(sce00515)Mannose type O-glycan biosynthesis
zinc fin	Essentia	GO:00000	GO:00056	GO:0061770 translation elongation factor binding;GO:0	
putative	DDHD dom	GO:00320	GO:00057	GO:0004620 phospholipase activity;GO:0016298 lipase a	
Coa6p	Protein	GO:00068	GO:00057	GO:0003674 molecular_function	
Lge1p	Protein	GO:00062	GO:00056	GO:0003674 molecular_function	
Ans1p	Putative	GO:00081	GO:00003	GO:0005199 structural constituent of cell wall;GO:000	
Apm2p	Protein	GO:00068	GO:00301	GO:0030276 clathrin binding;GO:0005515 protein bindin	
deoxyrib	DNA phot	GO:00007	GO:00057	GO:0003904 deoxyribodipyrimidine photo-lyase activity	
Signal r	Signal r	GO:00066	GO:00057	GO:00050	(sce03060)Protein export
Urb2p	Protein	GO:00063	GO:00057	GO:0003674 molecular_function	
Csf1p	Protein	GO:00061	GO:00057	GO:0003674 molecular_function	
non-cano	Non-cano	GO:00710	GO:00314	GO:00046	(sce03018)RNA degradation
rRNA-bin	Nucleola	GO:00004	GO:00320	GO:0034511 U3 snoRNA binding;GO:0030515 snoRNA bindin	
polo kin	Polo-lik	GO:01100	GO:00058	GO:00192	(sce04111)Cell cycle - yeast; (sce04113)Meio
pheromon	Protein	GO:00482	GO:00313	GO:0003674 molecular_function	

hypotheti	Putative	GO:00081	GO:00055	GO:0003674 molecular_function	
transcri	Transcri	GO:00458	GO:00056	GO:0008301 DNA binding, bending;GO:0003713 transcript	
None	None	None	None	None	None
None	None	None	None	None	None
RNA-deper	DEAD box	GO:00063	GO:00320	GO:0034512 box C/D snoRNA binding;GO:0034511 U3 snoRN	
None	None	None	None	None	None
None	None	None	None	None	None
hypotheti	Putative	GO:00081	GO:00055	GO:0003674 molecular_function	
None	None	None	None	None	None
None	None	None	None	None	None
None	None	None	None	None	None
None	None	None	None	None	None
None	None	None	None	None	None
DNA-direc	RNA polyn	GO:00427	GO:00056	GO:00010	(sce03020)RNA polymerase; (sce00240)Pyrimidi
putative	Putative	GO:00165	GO:00001	GO:00048	(sce04144)Endocytosis
Dgr2p	Protein	GO:00081	GO:00055	GO:0017137 Rab GTPase binding;GO:0017016 Ras GTPase b	
cytochror	Cytochror	GO:00061	GO:00057	GO:00451	(sce00190)Oxidative phosphorylation; (sce011
vacuolar	Regulator	GO:00421	GO:00332	GO:0005516 calmodulin binding;GO:0005515 protein bind	
exomer co	Member o	GO:00342	GO:00340	GO:0003674 molecular_function	
proteasor	Subunit	GO:00109	GO:00345	GO:00611	(sce03050)Proteasome
Bye1p	Negative	GO:00001	GO:00056	GO:0035064 methylated histone binding;GO:0140034 meth	
chromatir	Silencing	GO:00013	GO:00057	GO:0031493 nucleosomal histone binding;GO:0031491 nuc	
putative	Glycine-t	GO:00701	GO:00057	GO:00048	(sce00970)Aminoacyl-tRNA biosynthesis
putative	Protein i	GO:00726	GO:00004	GO:0061711 N(6)-L-threonylcarbamoyladenine synthase a	
Ras famil	GTP/GDP e	GO:00071	GO:00001	GO:0005088 Ras guanyl-nucleotide exchange factor acti	
mitochond	Mitochond	GO:00459	GO:00057	GO:0019843 rRNA binding;GO:0003735 structural constit	
mediator	Subunit	GO:00001	GO:00708	GO:0003712 transcription coregulator activity;GO:0140	
None	None	None	None	None	None
Pal1p	Protein	GO:00068	GO:00059	GO:0003674 molecular_function	
protein k	Genome ir	GO:20001	GO:00057	GO:00046	(sce04111)Cell cycle - yeast; (sce04113)Meio
RNA-proce	Protein i	GO:00004	GO:00314	GO:00305	(sce03008)Ribosome biogenesis in eukaryotes
None	None	None	None	None	None
U2 snRNP	Putative	GO:19032	GO:00056	GO:0030620 U2 snRNA binding;GO:0017069 snRNA binding;	
Sna2p	Protein	GO:00325	GO:00003	GO:0031625 ubiquitin protein ligase binding;GO:004438	
ribosoma	Ribosoma	GO:00000	GO:00226	GO:00037	(sce03010)Ribosome
SNAP rece	Vesicle n	GO:00068	GO:00059	GO:00054	(sce04130)SNARE interactions in vesicular tr
flavin ad	Mitochond	GO:00158	GO:00057	GO:0008517 folic acid transmembrane transporter activ	
Irc22p	Protein	GO:00081	GO:00057	GO:0003674 molecular_function	
ubiquitir	Putative	GO:00165	GO:00057	GO:0097027 ubiquitin-protein transferase activator ac	
Tvp18p	Integral	GO:00161	GO:00301	GO:0003674 molecular_function	
mitochond	Alpha sul	GO:00066	GO:00170	GO:0004222 metalloendopeptidase activity;GO:0008237 m	
Tar1p	Dubious	GO:00434	GO:00057	GO:0003674 molecular_function	
ornithine	Ornithine	GO:00333	GO:00057	GO:00045	(sce)Polyamine biosynthesis, arginine => orn
Ego2p	Componen	GO:00382	GO:00344	GO:0003674 molecular_function	
Rho famil	Guanine r	GO:19035	GO:00059	GO:00055	(sce04011)MAPK signaling pathway - yeast
exodeoxyr	Mitochond	GO:00007	GO:00057	GO:0035312 5'-3' exodeoxyribonuclease activity;GO:001	
ribosoma	Protein	GO:00064	GO:00226	GO:00037	(sce03010)Ribosome
Ure2p	Nitrogen	GO:00429	GO:00057	GO:00046	(sce00480)Glutathione metabolism
ribosoma	Ribosoma	GO:00021	GO:00226	GO:00037	(sce03010)Ribosome
pseudour	Pseudour	GO:19904	GO:00057	GO:0106029 tRNA pseudouridine synthase activity;GO:00	
translati	eIF3i sul	GO:00021	GO:00436	GO:00037	(sce03013)RNA transport
None	None	None	None	None	None
DNA topo	DNA Topo	GO:00070	GO:00314	GO:00039	(sce03440)Homologous recombination
hypotheti	Putative	GO:00081	GO:00055	GO:0003674 molecular_function	

aminophos	Endosomal	GO:00159	GO:19905	GO:0015247	aminophospholipid transmembrane transporte
TATA-bin	Subunit	GO:00011	GO:19904	GO:00011	(sce03022)Basal transcription factors
None	None	None	None	None	None
ATP-bind	ATP-bind	GO:00353	GO:00058	GO:0042626	ATPase activity, coupled to transmembrane
protein-l	SET-doma	GO:00180	GO:00056	GO:0016279	protein-lysine N-methyltransferase activit
Loc1p	Nuclear	GO:00000	GO:00306	GO:00037	(sce01110)Biosynthesis of secondary metaboli
anaphase	Subunit	GO:00314	GO:00056	GO:00616	(sce04120)Ubiquitin mediated proteolysis; (s
Rim9p	Plasma me	GO:00304	GO:00358	GO:0003674	molecular_function
Jip3p	Putative	GO:00081	GO:00055	GO:0003674	molecular_function
Eaf5p	Non-esser	GO:00062	GO:00352	GO:0003674	molecular_function
protein	Cytoplasm	GO:00436	GO:00058	GO:0008157	protein phosphatase 1 binding;GO:0019888 p
ribosome	Essential	GO:01060	GO:00056	GO:0030674	protein binding, bridging;GO:0060090 molec
Rex2p	3'-5' RN	GO:00344	GO:00057	GO:00001	(sce03008)Ribosome biogenesis in eukaryotes
DNA-dire	Second-la	GO:00427	GO:00056	GO:00010	(sce03020)RNA polymerase; (sce00240)Pyrimidi
Rpn14p	Evolution	GO:00706	GO:00005	GO:0003674	molecular_function
MutS fam	Protein i	GO:00510	GO:00007	GO:0032143	single thymine insertion binding;GO:003213
snoRNA-bi	Conserve	GO:00004	GO:00343	GO:00037	(sce03008)Ribosome biogenesis in eukaryotes
putative	Protein i	GO:00322	GO:00057	GO:0008757	S-adenosylmethionine-dependent methyltrans
phosphat	Subunit	GO:00065	GO:00005	GO:00171	(sce00563)Glycosylphosphatidylinositol (GPI)
Ras fami	Membrane	GO:00070	GO:00057	GO:0005088	Ras guanyl-nucleotide exchange factor acti
hypotheti	Putative	GO:00081	GO:00160	GO:0003674	molecular_function
phosphoi	Phosphat	GO:00468	GO:00003	GO:00044	(sce00562)Inositol phosphate metabolism; (sc
Arf fami	Guanine	GO:00320	GO:00057	GO:00050	(sce04144)Endocytosis
ribose p	5-phosph	GO:00060	GO:00021	GO:00047	(M00005)PRPP biosynthesis, ribose 5P => PRPP
Rad34p	Protein i	GO:00062	GO:00001	GO:0003684	damaged DNA binding;GO:0003697 single-stra
triglyce	Peroxiso	GO:00194	GO:00057	GO:0004806	triglyceride lipase activity;GO:0016298 li
Irc15p	Microtub	GO:00513	GO:00058	GO:00506	(M00307)Pyruvate oxidation, pyruvate => acet
Cwh41p	Processi	GO:00708	GO:00003	GO:00045	(sce)N-glycan precursor trimming; (M00073)N-
None	None	None	None	None	None
Cuz1p	Protein	GO:00712	GO:00056	GO:0070628	proteasome binding;GO:0008270 zinc ion bin
Pex11p	Peroxiso	GO:00443	GO:00057	GO:0003674	molecular_function
tyrosine	Phosphot	GO:00014	GO:00057	GO:0004725	protein tyrosine phosphatase activity;GO:0
myosin 2	Type V m	GO:00071	GO:00715	GO:0000146	microfilament motor activity;GO:0003774 mo
Msc6p	Multicop	GO:00071	GO:00057	GO:0003729	mRNA binding;GO:0003723 RNA binding;GO:000
Sop4p	ER-membr	GO:00068	GO:00057	GO:0003674	molecular_function
Ecm9p	Non-esser	GO:00715	GO:00057	GO:0003674	molecular_function
Rlf2p	Largest	GO:00063	GO:00331	GO:0042393	histone binding;GO:0005515 protein binding
E3 ubiqu	E3 ubiqu	GO:00004	GO:00057	GO:00616	(sce04120)Ubiquitin mediated proteolysis
flavin a	Putative	GO:00158	GO:00057	GO:0015230	FAD transmembrane transporter activity;GO:
ribosoma	Protein	GO:00021	GO:00226	GO:00037	(sce03010)Ribosome
Duo1p	Essential	GO:00519	GO:00427	GO:0051010	microtubule plus-end binding;GO:0008017 mi
Prp39p	U1 snRNP	GO:00003	GO:00002	GO:0030627	pre-mRNA 5'-splice site binding;GO:0036002
Smy1p	Kinesin-l	GO:00069	GO:00059	GO:00085	(sce04144)Endocytosis
Arf fami	Guanine	GO:00320	GO:00001	GO:00050	(sce04144)Endocytosis
Snf6p	Subunit	GO:00059	GO:00165	GO:0015616	DNA translocase activity;GO:0000182 rDNA b
None	None	None	None	None	None
serine C-	Componen	GO:00301	GO:00353	GO:00047	(sce00600)Sphingolipid metabolism; (sce04138
Rrg7p	Protein	GO:00081	GO:00057	GO:0003674	molecular_function
inositol	Inositol	GO:00301	GO:00313	GO:00527	(sce00600)Sphingolipid metabolism; (sce01100
ATP-bind	Mitochon	GO:00903	GO:00057	GO:0015421	oligopeptide-transporting ATPase activity;
snoRNA-bi	Nucleola	GO:00004	GO:0034455 t-UTP		(sce03008)Ribosome biogenesis in eukaryotes
beta-1,3-	Putative	GO:00315	GO:00092	GO:0005199	structural constituent of cell wall;GO:000
hypotheti	Putative	GO:00081	GO:00055	GO:0003674	molecular_function
Rec102p	Protein i	GO:00421	GO:00007	GO:0003674	molecular_function

Pcl9p	Cyclin; f	GO:000007	GO:00003(	GO:0016538	cyclin-dependent protein serine/threonine l
sulfiredo	Sulfiredo	GO:00988(	GO:00056:	GO:0032542	sulfiredoxin activity;GO:0016667 oxidoredu
Vrp1p	Verprolir	GO:20006(	GO:003047	GO:005101	(sce04144)Endocytosis
Art10p	Protein c	GO:00068	GO:00057:	GO:0003674	molecular_function
Acl4p	Specific	GO:00304:	GO:00056:	GO:0003674	molecular_function
Ctk2p	Beta subu	GO:00459(	GO:00706	GO:0061575	cyclin-dependent protein serine/threonine l
Ca(2+)-bi	ADP/ATP t	GO:00158(	GO:00057	GO:0005471	ATP:ADP antiporter activity;GO:0015217 ADP
bifunctio	Acetylglu	GO:00065	GO:00057	GO:00039	(M00028)Ornithine biosynthesis, glutamate =>
v-SNARE p	Protein i	GO:00482	GO:00125(	GO:00054	(sce04130)SNARE interactions in vesicular tra
NAD-deper	Member of	GO:199041	GO:00007	GO:00171	(sce00760)Nicotinate and nicotinamide metabo
hypotheti	Protein c	GO:00197(	GO:00058	GO:0043022	ribosome binding;GO:0043021 ribonucleoprot
Bit61p	Subunit c	GO:00382(	GO:00319	GO:0003674	molecular_function
dynamin-r	Dynamin-r	GO:00033	GO:00057	GO:00039	(sce04139)Mitophagy - yeast
tRNA dihy	Dihydrour	GO:00029	GO:00055	GO:0102266	tRNA-dihydrouridine20a synthase activity;G
None	None	None	None	None	None
Bsc5p	Protein c	GO:00065	GO:00001	GO:0034450	ubiquitin-ubiquitin ligase activity;GO:006
None	None	None	None	None	None
Rtc1p	Subunit c	GO:19042(	GO:00970	GO:0043022	ribosome binding;GO:0043021 ribonucleoprot
hypotheti	Putative	GO:00081	GO:00055	GO:0003674	molecular_function
hypotheti	Putative	GO:00081	GO:00055	GO:0003674	molecular_function
sterol ho	Cortical	GO:00970	GO:00325	GO:0003674	molecular_function
RNA-bindi	Signal re	GO:00066	GO:00057	GO:00309	(sce03060)Protein export
Nup85p	Subunit c	GO:00000	GO:00310	GO:00170	(sce03013)RNA transport
mismatch	Protein t	GO:004311	GO:00323(	GO:00004(	(sce03430)Mismatch repair
Dia1p	Protein c	GO:00014(	GO:00057:	GO:0003674	molecular_function
Bil1p	Protein t	GO:005101	GO:00059	GO:0003674	molecular_function
Om45p	Mitochonc	GO:00081	GO:00313(	GO:0003674	molecular_function
None	None	None	None	None	None
Sia1p	Protein c	GO:19026(	GO:00055	GO:0004721	phosphoprotein phosphatase activity;GO:001
Pes4p	Poly(A) b	GO:00081	GO:00104	GO:0008143	poly(A) binding;GO:0008266 poly(U) RNA bin
dolichyl-	Glucosyl	GO:00064	GO:00057	GO:00422	(sce)N-glycan precursor biosynthesis; (M0005
Hym1p	Component	GO:000711	GO:00433	GO:0004674	protein serine/threonine kinase activity;G
sphingani	Long-chai	GO:00301	GO:00057	GO:00423	(sce00600)Sphingolipid metabolism
None	None	None	None	None	None
Mdm31p	Mitochonc	GO:19002(	GO:00057	GO:0003674	molecular_function
Dad2p	Essential	GO:00519	GO:00427	GO:0051010	microtubule plus-end binding;GO:0008017 mi
hypotheti	Putative protein of unknown function; green fluorescent protein (GFP)-fusion pro				
Tos1p	Covalent	GO:00081	GO:00092	GO:0003674	molecular_function
Apm3p	Mu3-like	GO:00066	GO:00301	GO:0003674	molecular_function
Irc13p	Dubious c	GO:00081	GO:00160	GO:0003674	molecular_function
Ats1p	Protein i	GO:000711	GO:00057:	GO:0003674	molecular_function
putative	Putative	GO:00516(	GO:00427	GO:0003724	RNA helicase activity;GO:0004386 helicase a
Gis3p	Protein c	GO:00355	GO:00056:	GO:0003674	molecular_function
phosphati	Phosphati	GO:00163	GO:00304	GO:0008195	phosphatidate phosphatase activity;GO:0016
ribonucle	Ribonucle	GO:190511	GO:00056:	GO:1990846	ribonucleoside-diphosphate reductase inhib
glycolipi	Membrane	GO:00342(	GO:00057	GO:0034202	glycolipid-translocating activity;GO:00053
Laa1p	AP-1 acce	GO:00421	GO:00301	GO:0043022	ribosome binding;GO:0043021 ribonucleoprot
None	None	None	None	None	None
putative	Protein k	GO:000711	GO:00433	GO:0004674	protein serine/threonine kinase activity;G
None	None	None	None	None	None
hypotheti	Putative	GO:00081	GO:00055	GO:0003674	molecular_function
None	None	None	None	None	None
None	None	None	None	None	None
None	None	None	None	None	None

hypotheti	Putative	GO:00072(	GO:00055'	GO:0005085	guanyl-nucleotide exchange factor activity
None	None	None	None	None	None
None	None	None	None	None	None
None	None	None	None	None	None
None	None	None	None	None	None
None	None	None	None	None	None
oligo-1,(	Alpha-gl	GO:00002	GO:00057	GO:00045	(sce00052)Galactose metabolism; (sce00500)St
Atg16p	Conserve	GO:00448(	GO:01200	GO:00197	(sce04138)Autophagy - yeast
None	None	None	None	None	None
None	None	None	None	None	None
methylthi	5'-methy	GO:00195(	GO:00057	GO:00465	(sce)Methionine salvage pathway; (M00034)Met
dolichyl-	Dolichol	GO:00193	GO:00057	GO:00045	(sce00510)N-Glycan biosynthesis; (sce01100)M
DNA heli	DNA heli	GO:19909	GO:00056	GO:00431	(sce03430)Mismatch repair; (sce03420)Nucleot
hypotheti	Putative	GO:00081	GO:00160	GO:0003674	molecular_function
cyclin-d	Cyclin-d	GO:00606	GO:00709	GO:00199	(sce04111)Cell cycle - yeast
ubiquiti	Ubiquiti	GO:00021	GO:00058	GO:00313	(sce04122)Sulfur relay system
Irc19p	Protein	GO:00304	GO:00055	GO:0003674	molecular_function
Opy2p	Integral	GO:00007	GO:00003	GO:00036	(sce04011)MAPK signaling pathway - yeast
Gos1p	v-SNARE	GO:00482(	GO:00057	GO:00054	(sce04130)SNARE interactions in vesicular tr
None	None	None	None	None	None
DNA-dire	RNA poly	GO:00427	GO:00056	GO:00039	(sce03020)RNA polymerase; (sce00240)Pyrimidi
hypotheti	Putative	GO:00081	GO:00055	GO:0003674	molecular_function
Yrm1p	Zinc fin	GO:00063	GO:00056	GO:0000981	DNA-binding transcription factor activity,
ribosoma	Ribosoma	GO:00180	GO:00056	GO:0016279	protein-lysine N-methyltransferase activit
Scs3p	Protein	GO:00343	GO:00301	GO:0003674	molecular_function
None	None	None	None	None	None
Voa1p	ER prote	GO:00346	GO:00003	GO:0003674	molecular_function
Vps63p	Putative	GO:00066	GO:00056	GO:0003674	molecular_function
Pho86p	Endoplas	GO:00109	GO:00057	GO:0051082	unfolded protein binding;GO:0005515 protei
ribosoma	Protein	GO:00064(	GO:00226	GO:00037	(sce03010)Ribosome
None	None	None	None	None	None
Dan4p	Cell wal	GO:00315(	GO:00092	GO:0005199	structural constituent of cell wall;GO:000
ATP-depe	ATP-depe	GO:00704	GO:00058	GO:00040(	(sce03015)mRNA surveillance pathway; (sce030
None	None	None	None	None	None
Keg1p	Integral	GO:00060	GO:00301	GO:0003674	molecular_function
MCM DNA	Componen	GO:00319	GO:00711	GO:00171	(sce03030)DNA replication; (sce04111)Cell cy
Sum1p	Transcri	GO:00322	GO:00056	GO:00036	(sce04113)Meiosis - yeast
Rrn5p	Protein	GO:00427	GO:00005(	GO:0001181	RNA polymerase I general transcription ini
None	None	None	None	None	None
Nup120p	Subunit	GO:00353	GO:00310	GO:00170	(sce03013)RNA transport
Sxm1p	Nuclear	GO:00064(	GO:00056	GO:0008536	Ran GTPase binding;GO:0008565 protein tran
signal s	Putative	GO:00068	GO:00003	GO:0005048	signal sequence binding;GO:0042277 peptide
protein	Essentia	GO:00301	GO:00057	GO:0015450	P-P-bond-hydrolysis-driven protein transme
snoRNA-b	Nucleola	GO:00004	GO:0030692 Noc4p-Nop14p complex;GO:0030689 Noc complex;GO:0030		
Aft2p	Iron-reg	GO:00360	GO:00057	GO:0000978	RNA polymerase II proximal promoter sequen
DNA-dire	RNA poly	GO:00427	GO:00057	GO:00010	(sce03020)RNA polymerase; (sce00240)Pyrimidi
Spo11p	Meiosis-	GO:00007(	GO:00056	GO:00039	(sce04113)Meiosis - yeast
Kar9p	Spindle	GO:00315	GO:00007	GO:0003674	molecular_function
U6 snRNA	Lsm (Lik	GO:00003	GO:00056	GO:00170	(sce03018)RNA degradation; (sce03040)Spliceo
dihydrox	Dihydrox	GO:00195	GO:00058	GO:00503	(sce00051)Fructose and mannose metabolism; (
Bud25p	Protein	GO:00002	GO:00055	GO:0003674	molecular_function
hypotheti	Protein	GO:00711(	GO:00057	GO:0016807	cysteine-type carboxypeptidase activity;GO
putative	Putative	GO:00551	GO:00160	GO:0016491	oxidoreductase activity;GO:0003824 catalyt
Pbp1p	Componen	GO:19003(	GO:00104	GO:0003729	mRNA binding;GO:0003723 RNA binding;GO:000

Htl1p	Componen	GO:00063	GO:00165	GO:0015616 DNA translocase activity;GO:0008094 DNA-de	
Cep3p	Essentia	GO:00070	GO:00315	GO:0019237 centromeric DNA binding;GO:0008301 DNA bin	
ribosoma	Ribosoma	GO:00021	GO:00226	GO:00037	(sce03010)Ribosome
None	None	None	None	None	None
B-type c	B-type c	GO:00106	GO:00003	GO:00165	(sce04111)Cell cycle - yeast; (sce04113)Meio
Rlm1p	MADS-box	GO:00360	GO:00056	GO:00083	(sce04011)MAPK signaling pathway - yeast
methioni	Mitochon	GO:00064	GO:00057	GO:00048	(sce00450)Selenocompound metabolism; (sce009
DExD/H-b	Cytoplas	GO:00459	GO:00104	GO:00040	(sce03018)RNA degradation
None	None	None	None	None	None
transcri	RNA pol	GO:00427	GO:00001	GO:0001002 RNA polymerase III type 1 promoter sequenc	
None	None	None	None	None	None
Ino2p	Transcri	GO:00086	GO:00905	GO:0000978 RNA polymerase II proximal promoter sequen	
Hsm3p	Evolutio	GO:00706	GO:00085	GO:0003674 molecular_function	
mitochon	Mitochon	GO:00325	GO:00057	GO:0003735 structural constituent of ribosome;GO:0005	
tRNA spl	Subunit	GO:00003	GO:00002	GO:0000213 tRNA-intron endonuclease activity;GO:0016	
putative	Putative	GO:00160	GO:00057	GO:0047372 acylglycerol lipase activity;GO:0016298 li	
Swp82p	Member o	GO:00459	GO:00165	GO:0015616 DNA translocase activity;GO:0008094 DNA-de	
None	None	None	None	None	None
hypothet	Putative	GO:00081	GO:00160	GO:0003674 molecular_function	
Ady3p	Protein	GO:00321	GO:00700	GO:0003674 molecular_function	
acetate-	Acetyl-c	GO:00196	GO:00057	GO:00039	(sce00640)Propanoate metabolism; (sce00680)M
putative	Meiosis-	GO:00705	GO:00056	GO:0004623 phospholipase A2 activity;GO:0004620 phosp	
Aim24p	Protein	GO:00070	GO:00313	GO:0003674 molecular_function	
None	None	None	None	None	None
putative	Acyltran	GO:00361	GO:00058	GO:0016746 transferase activity, transferring acyl gr	
transcri	TFIIE sm	GO:00063	GO:00056	GO:00010	(sce03022)Basal transcription factors
None	None	None	None	None	None
Utp7p	Nucleola	GO:00004	GO:00306	GO:0003674 molecular_function	
hypothet	Putative protein of unknown function; SWAT-GFP and mCherry fusion proteins local				
DNA-bind	Componen	GO:19004	GO:00336	GO:0001227 DNA-binding transcription repressor activi	
actin-bi	Actin as	GO:20006	GO:00304	GO:00510	(sce04144)Endocytosis
RSC chro	ATPase c	GO:00615	GO:00165	GO:0015616 DNA translocase activity;GO:0070577 lysine	
putative	Putative	GO:00304	GO:00001	GO:00045	(sce)N-glycan precursor trimming; (M00073)N-
putative	Mitochon	GO:00320	GO:00313	GO:0004605 phosphatidate cytidylyltransferase activit	
holocyto	Cytochro	GO:00180	GO:00057	GO:00044	(sce00860)Porphyrin and chlorophyll metaboli
mismatch	Protein	GO:00007	GO:00323	GO:00004	(sce03430)Mismatch repair
protein	Essentia	GO:00301	GO:00057	GO:0030943 mitochondrion targeting sequence binding;G	
sphingos	Ceramide	GO:00465	GO:00615	GO:00502	(sce00600)Sphingolipid metabolism; (sce01100)
phosphor	N-succin	GO:00061	GO:00056	GO:00046	(M00048)Inosine monophosphate biosynthesis,
ferric/c	Ferric r	GO:00156	GO:00058	GO:0052851 ferric-chelate reductase (NADPH) activity;	
type 2C	Type 2C	GO:00513	GO:00058	GO:19904	(sce04011)MAPK signaling pathway - yeast
Dse3p	Daughter	GO:00070	GO:00059	GO:0003674 molecular_function	
phosphat	Protein	GO:19051	GO:00009	GO:0004865 protein serine/threonine phosphatase inhib	
flavin-l	Flavin-l	GO:00551	GO:00003	GO:0016971 flavin-linked sulfhydryl oxidase activity;	
hypothet	Protein	GO:00081	GO:00003	GO:0003674 molecular_function	
Erv25p	Member o	GO:00070	GO:00301	GO:0003674 molecular_function	
Erd1p	Predicte	GO:00066	GO:00057	GO:0003674 molecular_function	
hypothet	Putative	GO:00081	GO:00055	GO:0003674 molecular_function	
Avt2p	Putative	GO:00033	GO:00057	GO:0015171 amino acid transmembrane transporter activ	
Bsd2p	Heavy me	GO:00066	GO:00003	GO:0005515 protein binding;GO:0005488 binding;GO:0003	
H(+)-tra	Subunit	GO:00070	GO:00002	GO:00085	(sce04145)Phagosome; (sce00190)Oxidative pho
peptidyl	Membrane-	GO:00004	GO:00003	GO:0005528 FK506 binding;GO:0005527 macrolide binding	
None	None	None	None	None	None
Arf fami	Guanine	GO:00105	GO:00059	GO:0005086 ARF guanyl-nucleotide exchange factor acti	

phosphata	Protein i	GO:19001	GO:00056	GO:0004864	protein phosphatase inhibitor activity;GO:
tRNA ade	ATP (CTP)	GO:00016	GO:00057	GO:00529	(sce03013)RNA transport
Thp3p	Protein	GO:00003	GO:00353	GO:0003674	molecular_function
purine-c	Putative	GO:00158	GO:00003	GO:0015205	nucleobase transmembrane transporter activ
ESCRT-I	Componen	GO:19046	GO:00008	GO:00448	(sce04144)Endocytosis
aminoacyl	One of tv	GO:00325	GO:00057	GO:0004045	aminoacyl-tRNA hydrolase activity;GO:0140
Atp11p	Molecula	GO:00336	GO:00057	GO:0051082	unfolded protein binding;GO:0005515 protei
ribosoma	Ribosoma	GO:00004	GO:00306	GO:00037	(sce03010)Ribosome
dolichyl-	Protein (	GO:00717	GO:00975	GO:00041	(sce00515)Mannose type O-glycan biosynthesis
Rec107p	Protein i	GO:00071	GO:00007	GO:0003674	molecular_function
Sfh1p	Componen	GO:00332	GO:00165	GO:0015616	DNA translocase activity;GO:0008094 DNA-de
None	None	None	None	None	None
Cox23p	Protein	GO:00331	GO:00057	GO:0003674	molecular_function
type I s	Type I t	GO:00066	GO:00319	GO:0005048	signal sequence binding;GO:0042277 peptide
hypothet	Putative	GO:00081	GO:00057	GO:0005525	GTP binding;GO:0032550 purine ribonucleosi
Dam1p	Essentia	GO:19907	GO:19905	GO:00510	(sce04111)Cell cycle - yeast
None	None	None	None	None	None
hypothet	Putative	GO:00193	GO:00301	GO:00302	(sce00563)Glycosylphosphatidylinositol (GPI)-
hypothet	Putative	GO:00081	GO:00719	GO:0003674	molecular_function
Sna4p	Protein	GO:00081	GO:00003	GO:0003674	molecular_function
None	None	None	None	None	None
tubulin-	Subunit	GO:00716	GO:00162	GO:0051082	unfolded protein binding;GO:0015631 tubuli
putative	Cell wal	GO:00009	GO:00092	GO:0015926	glucosidase activity;GO:0004553 hydrolase
Wip1p	Kinetoch	GO:00070	GO:00007	GO:0019237	centromeric DNA binding;GO:1990837 sequenc
Nmd5p	Karyophe	GO:00066	GO:00343	GO:0008536	Ran GTPase binding;GO:0008565 protein tran
putative	Putative	GO:00081	GO:00056	GO:0052689	carboxylic ester hydrolase activity;GO:001
Cdh1p	Activato	GO:19024	GO:00056	GO:19907	(sce04120)Ubiquitin mediated proteolysis; (s
hypothet	Putative	GO:00081	GO:00160	GO:0003674	molecular_function
hypothet	Putative	GO:00081	GO:00055	GO:0003674	molecular_function
multidru	Multidru	GO:00304	GO:00058	GO:0015238	drug transmembrane transporter activity;GO
hypothet	Dubious	GO:00081	GO:00160	GO:0003674	molecular_function
Arf fami	Guanine	GO:00320	GO:00001	GO:0005086	ARF guanyl-nucleotide exchange factor acti
4a-hydro	Putative 4a-hydroxytetrahydrobiopterin dehydratase; green fluorescent protein (G				
karyophe	Karyophe	GO:00715	GO:00425	GO:00050	(sce03013)RNA transport
Her1p	Protein	GO:00070	GO:00058	GO:0043022	ribosome binding;GO:0043021 ribonucleoprot
None	None	None	None	None	None
chromati	Subunit	GO:00439	GO:00466	GO:00044	(sce03022)Basal transcription factors
None	None	None	None	None	None
None	None	None	None	None	None
sphingos	Ceramide	GO:00465	GO:00615	GO:00502	(sce00600)Sphingolipid metabolism; (sce01100
ribosome	Nucleola	GO:00004	GO:00306	GO:0003723	RNA binding;GO:0003676 nucleic acid bindin
Pds5p	Cohesion	GO:00071	GO:00349	GO:0005198	structural molecule activity;GO:0003674 mo
inositol	Inositol	GO:00066	GO:00709	GO:0045140	inositol phosphoceramide synthase activity
Elongato	Subunit	GO:00324	GO:00335	GO:0000049	tRNA binding;GO:0016887 ATPase activity;GO
Bit2p	Subunit	GO:00382	GO:00319	GO:0003674	molecular_function
phosphog	3-phosph	GO:00065	GO:00057	GO:00617	(M00020)Serine biosynthesis, glycerate-3P =>
Eco1p	Acetyltr	GO:00340	GO:00435	GO:0016407	acetyltransferase activity;GO:0016747 tran
ribonucl	Subunit	GO:00349	GO:00056	GO:00045	(sce03008)Ribosome biogenesis in eukaryotes;
short-ch	Putative	GO:00551	GO:00160	GO:0016491	oxidoreductase activity;GO:0003824 catalyt
Zip2p	Meiosis-	GO:00071	GO:00007	GO:0003677	DNA binding;GO:0000166 nucleotide binding;
Cis1p	Protein	GO:00081	GO:00057	GO:0003674	molecular_function
Yme2p	Integral	GO:00000	GO:00313	GO:0003723	RNA binding;GO:0003676 nucleic acid bindin
glutamat	Mitochon	GO:00065	GO:00057	GO:00043	(M00028)Ornithine biosynthesis, glutamate =>
hypothet	Putative	GO:19023	GO:00057	GO:0008271	secondary active sulfate transmembrane tra

seripaupe	Protein (	GO:00315(	GO:00092	GO:0005199 structural constituent of cell wall;GO:000	
lysophos	Phosphol	GO:00361	GO:00425	GO:00046	(sce00564)Glycerophospholipid metabolism
3-deoxy-	3-deoxy-I	GO:00094	GO:00056	GO:00038	(M00022)Shikimate pathway, phosphoenolpyruva
nucleocy	Essential	GO:00353	GO:00446	GO:0008139 nuclear localization sequence binding;GO:0	
hypotheti	UbiB fami	(Human)G(	(Human)G(	(Human)GO:0004674 protein serine/threonine kinase act	
Fmp10p	Putative	GO:00081	GO:00319	GO:0003674 molecular_function	
GTPase-ac	GTPase-ac	GO:00034(	GO:00301	GO:00050	(sce04141)Protein processing in endoplasmic
protein-a	SPOUT met	GO:00352	GO:00057	GO:0035241 protein-arginine omega-N monomethyltransfe	
NADP-depe	NADPH-dep	GO:00060	GO:00055	GO:00081(	(sce00040)Pentose and glucuronate interconve
Rxt3p	Component	GO:00164	GO:00336	GO:0003674 molecular_function	
ubiquitin	Ubiquitin	GO:00009	GO:00056	GO:0004842 ubiquitin-protein transferase activity;GO:	
hypotheti	Putative	GO:00071	GO:00160	GO:0005525 GTP binding;GO:0032550 purine ribonucleosi	
Cue3p	Protein (	GO:00081	GO:00057	GO:0043130 ubiquitin binding;GO:0032182 ubiquitin-lik	
Djp1p	Cytosolic	GO:00450	GO:00059	GO:0051087 chaperone binding;GO:0005515 protein bindi	
ribosomal	Protein (	GO:00422	GO:00226	GO:00037	(sce03010)Ribosome
putative	Transfer	GO:00433	GO:00160	GO:0008237 metallopeptidase activity;GO:0070011 pepti	
hypotheti	Putative	GO:00081	GO:00055	GO:0003674 molecular_function	
Std1p	Protein i	GO:00715	GO:00058	GO:0030295 protein kinase activator activity;GO:00192	
Rrt1p	Protein (	GO:00081	GO:00160	GO:0003674 molecular_function	
transloco	Conserved	GO:00312(	GO:00057	GO:00154	(sce03060)Protein export; (sce04145)Phagosom
hypotheti	Putative	GO:00517	GO:00056	GO:0003674 molecular_function	
Rtt105p	Protein v	GO:00105	GO:00056	GO:0003674 molecular_function	
glucan 1,	Sporulati	GO:00304	GO:00056	GO:00043	(sce00500)Starch and sucrose metabolism
ubiquitin	ER membra	GO:00309	GO:00008	GO:00048	(sce04141)Protein processing in endoplasmic
dolichyl-	Protein (	GO:00717	GO:00975	GO:00041	(sce00515)Mannose type O-glycan biosynthesis
hypotheti	CPA1 uORF	GO:00064	GO:00058	GO:0045182 translation regulator activity;GO:0003674	
Emc2p	Member of	GO:00349	GO:00725	GO:0003674 molecular_function	
Dtr1p	Putative	GO:00304	GO:00056	GO:0005275 amine transmembrane transporter activity;G	
Dls1p	Subunit (	GO:00708	GO:00086	GO:00469	(sce03410)Base excision repair; (sce03030)DN
pyruvate	Pyruvate	GO:00060	GO:00058	GO:00047	(sce00020)Citrate cycle (TCA cycle); (sce006
Aim19p	Protein (	GO:00081	GO:00319	GO:0003674 molecular_function	
hypotheti	Putative	GO:00550	GO:00160	GO:0003674 molecular_function	
Bfa1p	Subunit (	GO:00315	GO:19903	GO:00050	(sce04111)Cell cycle - yeast
tRNA (ura	tRNA(Ser)	GO:00304	GO:00057	GO:0016300 tRNA (uracil) methyltransferase activity;G	
ESCRT-II	Component	GO:00004	GO:00008	GO:00428	(sce04144)Endocytosis
None	None	None	None	None	None
None	None	None	None	None	None
importin-	Nuclear e	GO:00066(	GO:00056	GO:0005049 nuclear export signal receptor activity;GO	
E2 SUMO-c	SUMO-con	GO:00000	GO:00007	GO:00616	(sce04120)Ubiquitin mediated proteolysis; (s
DNA-bindi	Rpd3L his	GO:19005	GO:00336	GO:00704	(sce04139)Mitophagy - yeast; (sce04138)Autop
actin-rel	Actin-rel	GO:00315	GO:00002	GO:0003774 motor activity;GO:0017111 nucleoside-triph	
kinetocho	Component	GO:00513	GO:0031262 Ndc80 complex;GO:0000778 condensed nuclear chromoso		
ADP-ribos	ADP-ribos	GO:00482(	GO:00301	GO:00050	(sce04144)Endocytosis
Ran GTPas	Ran GTPas	GO:00319	GO:00058	GO:00039	(sce03008)Ribosome biogenesis in eukaryotes;
None	None	None	None	None	None
hypotheti	Predicted membrane protein; SWAT-GFP and mCherry fusion proteins localize to the				
None	None	None	None	None	None
Mgr1p	Subunit (	GO:00065	GO:00319	GO:0051787 misfolded protein binding;GO:0005515 prote	
serine/th	Protein k	GO:01000(	GO:00226	GO:00046	(sce04138)Autophagy - yeast; (sce04141)Prote
Syo1p	Transport	GO:00066(	GO:00056	GO:0051082 unfolded protein binding;GO:0005515 protei	
chromatin	Evolution	GO:00070	GO:00905	GO:00045	(sce03018)RNA degradation; (sce03008)Ribosom
IMP 5'-nu	Inosine 5	GO:00061	GO:00055	GO:00504	(sce00760)Nicotinate and nicotinamide metabo
galactose	Galactose	GO:00157	GO:00058	GO:00053	(sce04113)Meiosis - yeast
Pet54p	Mitochon	GO:00003	GO:00313	GO:0097157 pre-mRNA intronic binding;GO:0048027 mRNA	

hypotheti	Putative protein of unknown function; green fluorescent protein (GFP)-fusion pro				
None	None	None	None	None	None
None	None	None	None	None	None
None	None	None	None	None	None
bifuncti	Lysophos	GO:00905	GO:00056	GO:0052642 lysophosphatidic acid phosphatase activity	
serine/t	Protein	GO:01201	GO:00304	GO:0004674 protein serine/threonine kinase activity;G	
ribosoma	Ribosoma	GO:00021	GO:00226	GO:00037	(sce03010)Ribosome
Vid27p	Cytoplas	GO:00081	GO:00057	GO:0003674 molecular_function	
None	None	None	None	None	None
type 2A-	Ceramide-	GO:00020	GO:00001	GO:0004722 protein serine/threonine phosphatase activ	
gamma-gl	Gamma-gl	GO:00193	GO:00003	GO:01029	(sce00430)Taurine and hypotaurine metabolism
Oca4p	Cytoplas	GO:00353	GO:00057	GO:0004725 protein tyrosine phosphatase activity;GO:0	
hypotheti	Putative	GO:00081	GO:00160	GO:0003674 molecular_function	
putative	Member o	GO:19015	GO:00057	GO:0019239 deaminase activity;GO:0016787 hydrolase ac	
None	None	None	None	None	None
Chm7p	Yeast ho	GO:00070	GO:00055	GO:00036	(sce04144)Endocytosis
glucose-	Glucose-	GO:00450	GO:00057	GO:00043	(M00006)Pentose phosphate pathway, oxidative
aldose 1-	Putative	GO:00334	GO:00055	GO:0004034 aldose 1-epimerase activity;GO:0016857 rac	
Jip5p	Protein	GO:00422	GO:00057	GO:0003674 molecular_function	
DNA-dire	RNA poly	GO:00011	GO:00056	GO:00039	(sce03020)RNA polymerase; (sce00240)Pyrimidi
succinat	Succinat	GO:00065	GO:00057	GO:00090	(sce)GABA (gamma-Aminobutyrate) shunt; (M000
Leo1p	Componen	GO:20011	GO:00165	GO:1990269 RNA polymerase II C-terminal domain phosph	
L-iditol	Sorbitol	GO:00193	GO:00055	GO:00039	(sce00040)Pentose and glucuronate interconve
Sge1p	Plasma m	GO:00068	GO:00058	GO:0015238 drug transmembrane transporter activity;GO	
Xdj1p	Chaperon	GO:00301	GO:00313	GO:0001671 ATPase activator activity;GO:0060590 ATPas	
protein	Member o	GO:00454	GO:00057	GO:0003756 protein disulfide isomerase activity;GO:00	
Sgd1p	Essentia	GO:00069	GO:00970	GO:0003723 RNA binding;GO:0003676 nucleic acid bindin	
Izh3p	Membrane	GO:00068	GO:00057	GO:0038023 signaling receptor activity;GO:0060089 mol	
Swc3p	Protein	GO:00434	GO:00008	GO:0003674 molecular_function	
putative	Protein	GO:00086	GO:19036	GO:00363	(sce00750)Vitamin B6 metabolism
Tms1p	Vacuolar	GO:00081	GO:00003	GO:0003674 molecular_function	
Smc5-Smc	Subunit	GO:00169	GO:00309	GO:0019789 SUMO transferase activity;GO:0019787 ubiqu	
serine/t	SR prote	GO:00423	GO:00056	GO:0004674 protein serine/threonine kinase activity;G	
putative	Putative	GO:00021	GO:00057	GO:0043022 ribosome binding;GO:0043021 ribonucleoprot	
Golgi tr	Essentia	GO:00003	GO:00171	GO:0008565 protein transporter activity;GO:0005215 tr	
hypotheti	Putative protein of unknown function				
ribosoma	Protein	GO:00000	GO:00226	GO:00701	(sce03010)Ribosome
Pse1p	Karyophe	GO:00601	GO:00343	GO:0008139 nuclear localization sequence binding;GO:0	
dihydrol	Dihydrol	GO:00065	GO:00093	GO:00043	(M00307)Pyruvate oxidation, pyruvate => acet
None	None	None	None	None	None
TRAMP co	Zinc knu	GO:00710	GO:00314	GO:00046	(sce03018)RNA degradation
Atg22p	Vacuolar	GO:00329	GO:00716	GO:00036	(sce04138)Autophagy - yeast
chromati	Componen	GO:00073	GO:00004	GO:0031490 chromatin DNA binding;GO:0003682 chromatin	
None	None	None	None	None	None
ribosoma	Ribosoma	GO:00000	GO:00226	GO:00037	(sce03010)Ribosome
Sld3p	Protein	GO:00062	GO:00312	GO:0003682 chromatin binding;GO:0005488 binding;GO:00	
hypotheti	Putative	GO:00081	GO:00057	GO:0003674 molecular_function	
Ubx4p	UBX doma	GO:00304	GO:00198	GO:0044877 protein-containing complex binding;GO:0005	
Mhr1p	Mitochon	GO:00902	GO:00057	GO:0000150 recombinase activity;GO:0003697 single-str	
anaphase	Subunit	GO:00458	GO:00056	GO:00616	(sce04120)Ubiquitin mediated proteolysis; (s
Psp2p	Asn rich	GO:00081	GO:00009	GO:0003729 mRNA binding;GO:0003723 RNA binding;GO:000	
DNA poly	Catalyti	GO:00333	GO:00086	GO:00083	(sce03410)Base excision repair; (sce03030)DN
hypotheti	Putative	GO:00081	GO:00057	GO:0003674 molecular_function	
Arp6p	Actin-re	GO:00434	GO:00008	GO:0031491 nucleosome binding;GO:0003682 chromatin bi	

ubiquitir	Ubiquitir	GO:00075	GO:00056	GO:0031386 protein tag;GO:0003674 molecular_function	
alkaline	Alkaline	GO:00465	GO:00003	GO:00707	(sce00600)Sphingolipid metabolism
Tre1p	Transfer	GO:00433	GO:00003	GO:0003674 molecular_function	
long-chai	Very long	GO:00445	GO:00312	GO:0031957 very long-chain fatty acid-CoA ligase acti	
Aps2p	Small sub	GO:00068	GO:00301	GO:00085	(sce04144)Endocytosis
ribosomal	Protein c	GO:00064	GO:00226	GO:00198	(sce03010)Ribosome
bile acid	Transport	GO:00157	GO:00003	GO:0015432 bile acid-exporting ATPase activity;GO:003	
cohesin-l	Subunit c	GO:19054	GO:00057	GO:00435	(sce04111)Cell cycle - yeast
translati	Mitochonc	GO:00701	GO:00057	GO:0003743 translation initiation factor activity;GO:	
Otu2p	Protein c	GO:00165	GO:00058	GO:0004843 thiol-dependent ubiquitin-specific proteas	
Snf12p	73 kDa su	GO:00614	GO:00165	GO:0015616 DNA translocase activity;GO:0008094 DNA-de	
hypotheti	Putative	GO:00081	GO:00055	GO:0003674 molecular_function	
Ddc1p	DNA damag	GO:00515	GO:00308	GO:00084	(sce04111)Cell cycle - yeast; (sce04113)Meio
ferroxida	Mitochonc	GO:00182	GO:00057	GO:00349	(sce00860)Porphyrin and chlorophyll metaboli
protein-l	Ribosomal	GO:00180	GO:00058	GO:0016279 protein-lysine N-methyltransferase activit	
Hit1p	Protein i	GO:00004	GO:00707	GO:0046872 metal ion binding;GO:0043169 cation bindin	
hypotheti	Putative	GO:00081	GO:00057	GO:0003674 molecular_function	
Mtm1p	Mitochonc	GO:00319	GO:00057	GO:0030170 pyridoxal phosphate binding;GO:0070279 vit	
B-type cy	B-type cy	GO:00106	GO:00003	GO:00165	(sce04011)MAPK signaling pathway - yeast; (s
hypotheti	Putative protein of unknown function; green fluorescent protein (GFP)-fusion pro				
hypotheti	Putative	GO:00065	GO:00001	GO:0034450 ubiquitin-ubiquitin ligase activity;GO:006	
chitin de	Chitin de	GO:00304	GO:00056	GO:00040	(sce00520)Amino sugar and nucleotide sugar m
Msb1p	Protein c	GO:00300	GO:00059	GO:0003674 molecular_function	
putative	Peroxisom	GO:00015	GO:00057	GO:00309	(sce04146)Peroxisome
Pxp2p	Peroxisom	GO:00988	GO:00057	GO:0051920 peroxiredoxin activity;GO:0016684 oxidored	
Zap1p	Zinc-regu	GO:00972	GO:00056	GO:0000977 RNA polymerase II regulatory region sequen	
ATP-bindi	Mitochonc	GO:00482	GO:00057	GO:00426	(sce02010)ABC transporters
ribosome	Constitue	GO:00422	GO:00306	GO:0003674 molecular_function	
hypotheti	Putative	GO:00081	GO:00160	GO:0003674 molecular_function	
GTPase RI	Cytoplasm	GO:00422	GO:19909	GO:00430	(sce03008)Ribosome biogenesis in eukaryotes
None	None	None	None	None	None
DNA-direc	RNA polym	GO:00427	GO:00057	GO:00010	(sce03020)RNA polymerase; (sce00240)Pyrimidi
hypotheti	Putative	GO:00081	GO:00055	GO:0003674 molecular_function	
azole tra	Plasma me	GO:00451	GO:00058	GO:1901474 azole transmembrane transporter activity;G	
ERMES cor	Mitochonc	GO:00450	GO:00328	GO:19016	(sce04139)Mitophagy - yeast
ribosomal	Ribosomal	GO:00004	GO:00306	GO:00037	(sce03010)Ribosome
glutathic	Cis-golgi	GO:00988	GO:00057	GO:0004362 glutathione-disulfide reductase activity;G	
4-amino-5	Protein i	GO:00092	GO:00055	GO:00036	(sce00730)Thiamine metabolism; (sce01100)Met
Ugo1p	Outer mem	GO:00080	GO:00313	GO:0022857 transmembrane transporter activity;GO:0005	
Mne1p	Protein i	GO:00003	GO:00057	GO:0003674 molecular_function	
None	None	None	None	None	None
polynucle	DNA 3'-ph	GO:00985	GO:00056	GO:0046404 ATP-dependent polydeoxyribonucleotide 5'-h	
E3 ubiqui	Splicing	GO:00003	GO:00710	GO:00003	(sce04120)Ubiquitin mediated proteolysis; (s
Puf6p	Pumilio-h	GO:00171	GO:00059	GO:0000900 translation repressor activity, mRNA regul	
Dck1p	Dock fami	GO:00004	GO:00057	GO:0005085 guanyl-nucleotide exchange factor activity	
RNA-deper	RNA-Deper	GO:00004	GO:00306	GO:0004004 ATP-dependent RNA helicase activity;GO:000	
Cmc2p	Protein i	GO:00331	GO:00057	GO:0046872 metal ion binding;GO:0043169 cation bindin	
protein-l	Ribosomal	GO:00180	GO:00055	GO:0016279 protein-lysine N-methyltransferase activit	
hypotheti	Protein c	GO:00081	GO:00056	GO:0003674 molecular_function	
None	None	None	None	None	None
forkhead	Forkhead	GO:19034	GO:00058	GO:0003688 DNA replication origin binding;GO:0001085	
Jjj2p	Protein c	GO:00510	GO:00056	GO:0051082 unfolded protein binding;GO:0005515 protei	
retromer	Subunit c	GO:00421	GO:00309	GO:0032266 phosphatidylinositol-3-phosphate binding;G	
Pho23p	Componen	GO:20002	GO:00336	GO:0004402 histone acetyltransferase activity;GO:0061	

hypotheti	Putative	GO:00081	GO:00056	GO:0030170 pyridoxal phosphate binding;GO:0070279 vit	
DEAH-box	RNA helic	GO:00003	GO:00710	GO:00344	(sce03040)Spliceosome
None	None	None	None	None	None
Ndc1p	Subunit	GO:00717	GO:00707	GO:00170	(sce03013)RNA transport
flavodox	Protein	GO:00551	GO:00451	GO:0010181 FMN binding;GO:0050662 coenzyme binding;GO	
Jnm1p	Componen	GO:00001	GO:00002	GO:0003774 motor activity;GO:0017111 nucleoside-triph	
Stp1p	Transcri	GO:00080	GO:00058	GO:0000978 RNA polymerase II proximal promoter sequen	
putative	Putative	GO:00000	GO:00433	GO:00039	(sce03008)Ribosome biogenesis in eukaryotes
histone	Subunit	GO:00165	GO:00466	GO:00044	(Human)(hsa05166)HTLV-I infection
Mrx10p	Mitochon	GO:00081	GO:00057	GO:0003674 molecular_function	
metalloe	Mitochon	GO:00066	GO:00057	GO:0004222 metalloendopeptidase activity;GO:0008237 m	
Cch1p	Voltage-	GO:00705	GO:00058	GO:0005245 voltage-gated calcium channel activity;GO:	
Nas2p	Evolutio	GO:00706	GO:00085	GO:0003674 molecular_function	
ssDNA-de	Protein	GO:00335	GO:00308	GO:0043142 single-stranded DNA-dependent ATPase activ	
galactok	Galactok	GO:00004	GO:00058	GO:00043	(sce)Nucleotide sugar biosynthesis, galactos
Nbp2p	Protein	GO:00001	GO:00056	GO:0030674 protein binding, bridging;GO:0060090 molec	
None	None	None	None	None	None
putative	Putative	GO:00719	GO:00092	GO:0042124 1,3-beta-glucanosyltransferase activity;GO	
None	None	None	None	None	None
DNA prim	Subunit	GO:00062	GO:00056	GO:00038	(sce03030)DNA replication; (sce00240)Pyrimid
Ctr86p	Essentia	GO:00090	GO:00056	GO:0003674 molecular_function	
Apl6p	Beta3-li	GO:00066	GO:00301	GO:0003674 molecular_function	
None	None	None	None	None	None
hypotheti	Putative	GO:00081	GO:00055	GO:0003674 molecular_function	
RNA-bind	Lsm (Lik	GO:00009	GO:00056	GO:00037	(sce03018)RNA degradation; (sce03040)Spliceo
None	None	None	None	None	None
hypotheti	Putative	GO:00081	GO:00055	GO:0003674 molecular_function	
Arf fami	Dubious	GO:00312	GO:00057	GO:00154	(sce03060)Protein export; (sce04145)Phagosom
5-formyl	5,10-met	GO:00359	GO:00057	GO:00302	(sce00670)One carbon pool by folate; (sce011
Fin1p	Spindle	GO:19024	GO:00002	GO:0005200 structural constituent of cytoskeleton;GO:	
None	None	None	None	None	None
hypotheti	Putative	GO:00081	GO:00160	GO:0003674 molecular_function	
H(+)-tra	Subunit	GO:00070	GO:00002	GO:00469	(sce04145)Phagosome; (sce00190)Oxidative pho
bifuncti	DNA N-gl	GO:00062	GO:00056	GO:00007	(sce03410)Base excision repair
hypotheti	Putative	GO:00081	GO:00055	GO:0003674 molecular_function	
None	None	None	None	None	None
hypotheti	Putative	GO:00081	GO:00055	GO:0003674 molecular_function	
Rho fami	Small rh	GO:00313	GO:00059	GO:00039	(sce04933)AGE-RAGE signaling pathway in diab
peroxire	Thiol pe	GO:00988	GO:00057	GO:00470	(sce00590)Arachidonic acid metabolism; (sce0
hypotheti	Putative protein of unknown function				
Mlp1p	Myosin-l	GO:19019	GO:00446	GO:00170	(sce03013)RNA transport
phosphat	Mitochon	GO:00320	GO:00057	GO:00089	(sce00564)Glycerophospholipid metabolism; (s
None	None	None	None	None	None
None	None	None	None	None	None
hypotheti	Putative	GO:00081	GO:00160	GO:0003674 molecular_function	
None	None	None	None	None	None
DNA poly	Third su	GO:00431	GO:00436	GO:00083	(sce03410)Base excision repair; (sce03440)Ho
None	None	None	None	None	None
hypotheti	Putative	GO:00081	GO:00055	GO:0003674 molecular_function	
putative	Putative	GO:00301	GO:00056	GO:00042	(sce04138)Autophagy - yeast
putative	Vacuolar	GO:00991	GO:19908	GO:0015662 ATPase activity, coupled to transmembrane	
Ysp2p	Sterol-b	GO:01200	GO:00325	GO:0120015 intermembrane sterol transfer activity;GO:	
Yrb30p	RanGTP-b	GO:00435	GO:00056	GO:0005096 GTPase activator activity;GO:0030695 GTPas	
ribosoma	Ribosoma	GO:00171	GO:00226	GO:00037	(sce03010)Ribosome

DUP240 fa	Putative	GO:00160	GO:00003	GO:0003674 molecular_function	
putative	Putative	GO:00081	GO:00056	GO:0052689 carboxylic ester hydrolase activity;GO:001	
None	None	None	None	None	None
Rif2p	Protein	GO:00070	GO:00701	GO:0042162 telomeric DNA binding;GO:0043565 sequence-	
None	None	None	None	None	None
hypotheti	Putative	GO:00081	GO:00055	GO:0003674 molecular_function	
Utr5p	Protein	GO:00081	GO:00055	GO:0003674 molecular_function	
Rgt1p	Glucose-	GO:00463	GO:00056	GO:0000978 RNA polymerase II proximal promoter sequen	
ribosoma	Protein	GO:00004	GO:00226	GO:00037	(sce03010)Ribosome
Rsc3p	Componen	GO:00332	GO:00165	GO:0015616 DNA translocase activity;GO:0008094 DNA-de	
fumarase	Fumarase	GO:00061	GO:00452	GO:00043	(sce)Citrate cycle, second carbon oxidation,
Phm6p	Protein	GO:00081	GO:00003	GO:0003674 molecular_function	
hypotheti	Putative membrane protein of unknown function				
putative	Putative	GO:00160	GO:00055	GO:0047372 acylglycerol lipase activity;GO:0016298 li	
Pac2p	Microtub	GO:00070	GO:00058	GO:0043014 alpha-tubulin binding;GO:0015631 tubulin b	
Nop12p	Nucleola	GO:00004	GO:00306	GO:0003723 RNA binding;GO:0003676 nucleic acid bindin	
Spt23p	ER membr	GO:00360	GO:00301	GO:0003674 molecular_function	
cullin R	Cullin s	GO:00706	GO:00353	GO:0004842 ubiquitin-protein transferase activity;GO:	
L-iditol	Sorbitol	GO:00193	GO:00055	GO:00039	(sce00040)Pentose and glucuronate interconve
peptidyl	Peptidyl-	GO:00004	GO:00003	GO:0016018 cyclosporin A binding;GO:0003755 peptidyl-	
peptidyl	Peptidyl-	GO:00004	GO:00003	GO:0016018 cyclosporin A binding;GO:0003755 peptidyl-	
None	None	None	None	None	None
serine/t	Serine/t	GO:00333	GO:00358	GO:00046	(sce04111)Cell cycle - yeast; (sce04113)Meio
hypotheti	Putative	GO:00081	GO:00055	GO:0003674 molecular_function	
Zip1p	Transver	GO:00451	GO:00008	GO:00321	(sce04113)Meiosis - yeast
Rpd3L his	Componen	GO:19005	GO:00336	GO:0004407 histone deacetylase activity;GO:0033558 pr	
hypotheti	Protein	GO:00081	GO:00726	GO:0003674 molecular_function	
ubiquiti	Ubiquiti	GO:00165	GO:00057	GO:0004843 thiol-dependent ubiquitin-specific proteas	
glycogen	Glycogen	GO:00059	GO:00056	GO:00043	(sce00500)Starch and sucrose metabolism; (sc
hypotheti	Putative	GO:00081	GO:00055	GO:0003674 molecular_function	
Pet122p	Mitochon	GO:00701	GO:00313	GO:0003743 translation initiation factor activity;GO:	
serine/t	Protein	GO:00448	GO:00321	GO:00055	(sce04111)Cell cycle - yeast
Isc10p	Protein	GO:00304	GO:00055	GO:0003674 molecular_function	
inositol	Inositol	GO:00060	GO:00056	GO:00528	(sce00562)Inositol phosphate metabolism; (sc
NADH/NAD	ATP-NADH	GO:00067	GO:00055	GO:0042736 NADH kinase activity;GO:0003951 NAD+ kinas	
hypotheti	Putative	GO:00081	GO:00055	GO:0003674 molecular_function	
O-acyltr	Plasma m	GO:00195	GO:00057	GO:0008374 O-acyltransferase activity;GO:0015293 symp	
exomer c	Member o	GO:00342	GO:00340	GO:0043022 ribosome binding;GO:0043021 ribonucleoprot	
phosphoa	3'-phosp	GO:00193	GO:00057	GO:00046	(M00176)Assimilatory sulfate reduction, sulf
Yft2p	Protein	GO:00343	GO:00301	GO:0003674 molecular_function	
U5 snRNP	Non-esse	GO:00081	GO:00056	GO:0005515 protein binding;GO:0005488 binding;GO:0003	
Pef1p	Penta-EF-	GO:00034	GO:00059	GO:0005509 calcium ion binding;GO:0008270 zinc ion bi	
anaphase	Largest	GO:00311	GO:00056	GO:00329	(sce04120)Ubiquitin mediated proteolysis; (s
Sls1p	Mitochon	GO:00090	GO:00426	GO:0003674 molecular_function	
None	None	None	None	None	None
Pdc2p	Transcri	GO:20011	GO:00056	GO:0000978 RNA polymerase II proximal promoter sequen	
Gyp6p	GTPase-a	GO:00906	GO:00301	GO:0017137 Rab GTPase binding;GO:0005096 GTPase activ	
None	None	None	None	None	None
Amf1p	Low affi	GO:00354	GO:00058	GO:0008519 ammonium transmembrane transporter activit	
DNA-bind	Origin-b	GO:00014	GO:00435	GO:0061630 ubiquitin protein ligase activity;GO:00616	
None	None	None	None	None	None
Hsp70 fa	Hsp70 fa	GO:00320	GO:00014	GO:00441	(sce03018)RNA degradation
DNA repa	Componen	GO:19906	GO:00309	GO:0019789 SUMO transferase activity;GO:0003684 damag	
Smc5-Smc	Componen	GO:00169	GO:00309	GO:0019789 SUMO transferase activity;GO:0019787 ubiqu	

Brr1p	snRNP pr	GO:00002	GO:0030532 small nuclear ribonucleoprotein complex;GO:0120114		
pantother	Pantother	GO:00159	GO:00056	GO:00045	(sce)Coenzyme A biosynthesis, pantothenate =
hypotheti	Putative	GO:00453	GO:00058	GO:0003674 molecular_function	
None	None	None	None	None	None
None	None	None	None	None	None
RNA polyr	CTD phosp	GO:00709	GO:00056	GO:0008420 RNA polymerase II CTD heptapeptide repeat	
Uip5p	Protein	GO:00070	GO:00301	GO:0005537 mannose binding;GO:0048029 monosaccharide	
SBF compl	DNA bindi	GO:00719	GO:00309	GO:00012	(sce04011)MAPK signaling pathway - yeast; (s
Med4p	Subunit	GO:00063	GO:00708	GO:0000979 RNA polymerase II core promoter sequence-s	
U4/U6-U5	Splicing	GO:00002	GO:00710	GO:00036	(sce03040)Spliceosome
aurora ki	Aurora ki	GO:00902	GO:00058	GO:0035174 histone serine kinase activity;GO:0035173	
kinesin r	Kinesin r	GO:00000	GO:00058	GO:0008569 ATP-dependent microtubule motor activity,	
hypotheti	Putative	GO:00081	GO:00055	GO:0003674 molecular_function	
ribosomal	Ribosomal	GO:00321	GO:00226	GO:00302	(sce03010)Ribosome
AMP deami	AMP deami	GO:00061	GO:00058	GO:00038	(sce00230)Purine metabolism; (sce01130)Biosy
None	None	None	None	None	None
None	None	None	None	None	None
Rxt2p	Componen	GO:20002	GO:00336	GO:0004407 histone deacetylase activity;GO:0033558 pr	
AAA famil	AAA-ATPa	GO:00706	GO:00100	GO:00168	(sce04144)Endocytosis
hypotheti	Dubious	GO:00081	GO:00055	GO:0003674 molecular_function	
None	None	None	None	None	None
hypotheti	Protein	GO:00081	GO:00319	GO:0035091 phosphatidylinositol binding;GO:0005543 ph	
karyophe	Karyophe	GO:00311	GO:00425	GO:0061608 nuclear import signal receptor activity;GO	
None	None	None	None	None	None
Mak10p	Non-cata	GO:00171	GO:00314	GO:0004596 peptide alpha-N-acetyltransferase activity	
Spc34p	Essentia	GO:00519	GO:00427	GO:0051010 microtubule plus-end binding;GO:0008017 mi	
ribonucl	Nuclear	GO:00308	GO:00057	GO:00045	(sce03008)Ribosome biogenesis in eukaryotes
ribosomal	Protein	GO:00004	GO:00226	GO:00037	(sce03010)Ribosome
hypotheti	Putative	GO:00081	GO:00055	GO:0003674 molecular_function	
hypotheti	Putative	GO:00081	GO:00055	GO:0003674 molecular_function	
None	None	None	None	None	None
transcri	Transcri	GO:19004	GO:00007	GO:00443	(sce04011)MAPK signaling pathway - yeast; (s
ribosomal	Ribosomal	GO:00021	GO:00226	GO:00037	(sce03010)Ribosome
exosome r	Exosome r	GO:00710	GO:00001	GO:00037	(sce03018)RNA degradation
hypotheti	Putative	GO:00081	GO:00055	GO:0003674 molecular_function	
iron-sul	Componen	GO:00020	GO:00973	GO:0003674 molecular_function	
DNA-bindi	DNA bindi	GO:00304	GO:00001	GO:0044374 sequence-specific DNA binding, bending;GO:	
Urb1p	Protein	GO:00004	GO:00057	GO:0003729 mRNA binding;GO:0003723 RNA binding;GO:000	
14-3-3 fa	14-3-3 p	GO:00342	GO:00058	GO:00508	(sce04011)MAPK signaling pathway - yeast
UTP--glu	Putative	GO:00060	GO:00057	GO:00039	(M00549)Nucleotide sugar biosynthesis, gluco
mRNA spli	Core Sm	GO:00003	GO:00002	GO:00037	(sce03040)Spliceosome
None	None	None	None	None	None
hypotheti	Protein	GO:00081	GO:00057	GO:0016887 ATPase activity;GO:0017111 nucleoside-trip	
H(+)-tra	Vacuolar	GO:00070	GO:00002	GO:00469	(sce04145)Phagosome; (sce00190)Oxidative pho
ureidogl	Ureidogl	GO:00002	GO:00160	GO:00503	(sce00230)Purine metabolism; (sce01100)Metab
hypotheti	Putative	GO:00081	GO:00160	GO:0003674 molecular_function	
sphingos	Ceramide	GO:00465	GO:00615	GO:0050291 sphingosine N-acyltransferase activity;GO:	
Nup133p	Subunit	GO:00353	GO:00310	GO:00170	(sce03013)RNA transport
None	None	None	None	None	None
ATP-depen	Essentia	GO:00004	GO:00306	GO:0034459 ATP-dependent 3'-5' RNA helicase activity;	
None	None	None	None	None	None
cohesin-	Subunit	GO:00705	GO:00321	GO:00435	(sce04111)Cell cycle - yeast
hypotheti	Putative	GO:00081	GO:00055	GO:0003674 molecular_function	
pseudour	tRNA:pse	GO:00311	GO:00057	GO:0106029 tRNA pseudouridine synthase activity;GO:00	

None	None	None	None	None	None
Mup1p	High affi	GO:19036	GO:00003	GO:0000102	L-methionine secondary active transmembran
TRAPP sul	Core comp	GO:00068	GO:199007	GO:0017112	Rab guanyl-nucleotide exchange factor acti
elongin /	Elongin /	GO:000651	GO:00704	GO:0004842	ubiquitin-protein transferase activity;GO:
DNA polym	Third-lar	GO:00708	GO:00086	GO:00083	(sce03410)Base excision repair; (sce03030)DN
None	None	None	None	None	None
Rrs1p	Essential	GO:00000	GO:00306	GO:0003674	molecular_function
None	None	None	None	None	None
adenosine	Adenosine	GO:00903	GO:00056	GO:0043530	adenosine 5'-monophosphoramidase activity;
hypotheti	Putative	GO:00081	GO:00058	GO:0003674	molecular_function
DNA-direc	RNA polym	GO:00427	GO:00057	GO:00010	(sce03020)RNA polymerase; (sce00240)Pyrimidi
None	None	None	None	None	None
None	None	None	None	None	None
None	None	None	None	None	None
DNA-direc	B subunit	GO:00062	GO:00056	GO:00038	(sce03030)DNA replication; (sce00240)Pyrimid
glyceroph	Glyceroph	GO:00066	GO:00057	GO:0071617	lysophospholipid acyltransferase activity;
rRNA-proc	Protein w	GO:00063	GO:00057	GO:0003674	molecular_function
Lam5p	Putative	GO:00323	GO:00715	GO:0015248	sterol transporter activity;GO:0005319 lip
putative	Putative	GO:00071	GO:00056	GO:0004674	protein serine/threonine kinase activity;G
putative	Putative	GO:00094	GO:00058	GO:00084	(sce00750)Vitamin B6 metabolism; (sce01100)M
mRNA-bind	Posttrans	GO:00434	GO:00057	GO:0003730	mRNA 3'-UTR binding;GO:0003729 mRNA bindin
mismatch	Protein i	GO:00007	GO:00975	GO:00309	(sce03430)Mismatch repair
Avt1p	Vacuolar	GO:00158	GO:00003	GO:0015188	L-isoleucine transmembrane transporter act
putative	Subunit c	GO:00363	GO:00976	GO:0004842	ubiquitin-protein transferase activity;GO:
Vba1p	Permease	GO:19908	GO:00003	GO:0015174	basic amino acid transmembrane transporter
histone a	Catalytic	GO:00063	GO:00001	GO:00104	(Human)(hsa05034)Alcoholism
cell prol	Assembly	GO:19051	GO:00057	GO:0000287	magnesium ion binding;GO:0005524 ATP bindi
mitochond	Mitochond	GO:00325	GO:00057	GO:0005525	GTP binding;GO:0032550 purine ribonucleosi
Pkr1p	V-ATPase	GO:00700	GO:00301	GO:0003674	molecular_function
Rfa3p	Subunit c	GO:00062	GO:00056	GO:0003690	double-stranded DNA binding;GO:0043565 seq
signal re	Subunit c	GO:00066	GO:00057	GO:0003723	RNA binding;GO:0003676 nucleic acid bindin
Mtr10p	Nuclear i	GO:00064	GO:00343	GO:0008536	Ran GTPase binding;GO:0008565 protein tran
hypotheti	Dubious c	GO:00081	GO:00055	GO:0003674	molecular_function
Slx4p	Endonucle	GO:19052	GO:00335	GO:0017108	5'-flap endonuclease activity;GO:0048256 f
threonine	Mitochond	GO:00701	GO:00057	GO:00048	(sce00970)Aminoacyl-tRNA biosynthesis
None	None	None	None	None	None
None	None	None	None	None	None
alpha,alp	Acid treh	GO:00714	GO:00302	GO:00045	(sce00500)Starch and sucrose metabolism; (sc
None	None	None	None	None	None
hypotheti	Protein of unknown function; mRNA identified as translated by ribosome profiling				
hypotheti	Putative	GO:00424	GO:00057	GO:0003674	molecular_function
hypotheti	Putative	GO:00081	GO:00055	GO:0003674	molecular_function
hypotheti	Predicted	GO:00081	GO:00058	GO:0043022	ribosome binding;GO:0043021 ribonucleoprot
ribosomal	Protein c	GO:00004	GO:00226	GO:00037	(sce03010)Ribosome
ribosomal	Conserved	GO:00000	GO:00306	GO:00701	(sce03010)Ribosome
Golgi tra	Essential	GO:00003	GO:00171	GO:0030674	protein binding, bridging;GO:0060090 molec
apyrase	Apyrase w	GO:00066	GO:00301	GO:01024	(sce00240)Pyrimidine metabolism; (sce00230)P
hypotheti	Protein c	GO:00081	GO:00055	GO:0003674	molecular_function
nitrogen	Subunit c	GO:19038	GO:00970	GO:00036	(Human)(hsa04150)mTOR signaling pathway
guanine c	Guanine c	GO:00061	GO:00057	GO:00088	(sce00230)Purine metabolism; (sce01100)Metab
Tna1p	High affi	GO:19032	GO:00003	GO:0046943	carboxylic acid transmembrane transporter
bifunctio	Aromatic	GO:00195	GO:00057	GO:00801	(M00024)Phenylalanine biosynthesis, chorisma
None	None	None	None	None	None
Rrp15p	Nucleolar	GO:00004	GO:00306	GO:0003674	molecular_function

hypotheti	Putative	GO:00081	GO:00055	GO:0003674 molecular_function	
None	None	None	None	None	None
mitogen-a	Serine/th	GO:00109	GO:00059	GO:0004	(sce04139)Mitophagy - yeast; (sce04138)Autop
E2 ubiqui	Ubiquitin	GO:00346	GO:00001	GO:00706	(sce04120)Ubiquitin mediated proteolysis; (s
tRNA 2'-p	tRNA 2'-p	GO:00063	GO:00056	GO:0000215 tRNA 2'-phosphotransferase activity;GO:014	
guanosine	Guanosine	GO:00064	GO:00001	GO:00043	(Human)(hsa00240)Pyrimidine metabolism; (hsa
ribosomal	Ribosomal	GO:00021	GO:00226	GO:00037	(sce03010)Ribosome
Dss1p	3'-5' exo	GO:00009	GO:00450	GO:0008859 exoribonuclease II activity;GO:0000175 3'-	
hypotheti	Putative	GO:00081	GO:00001	GO:0003674 molecular_function	
ESCRT-III	One of fo	GO:19046	GO:00008	GO:00036	(sce04144)Endocytosis
None	None	None	None	None	None
Utp13p	Nucleolar	GO:00004	GO:00343	(Human)GO	(sce03008)Ribosome biogenesis in eukaryotes
Dur3p	Plasma me	GO:00434	GO:00057	GO:0015489 putrescine transmembrane transporter activ	
Rtt102p	Component	GO:00063	GO:00165	GO:0015616 DNA translocase activity;GO:0008094 DNA-de	
adenosylm	7,8-diami	GO:00091	GO:00057	GO:00041	(sce00780)Biotin metabolism; (sce01100)Metab
Rad24p	Checkpoin	GO:00333	GO:00313	GO:00036	(sce04111)Cell cycle - yeast; (sce04113)Meio
signal re	Core comp	GO:00066	GO:00057	GO:00309	(sce03060)Protein export
ribosomal	Ribosomal	GO:00000	GO:00306	GO:00198	(sce03010)Ribosome
Sdd2p	Protein w	GO:00104	GO:00058	GO:0003677 DNA binding;GO:0003676 nucleic acid bindin	
ribosomal	Protein c	GO:00422	GO:00226	GO:00037	(sce03010)Ribosome
None	None	None	None	None	None
methylene	NAD-depen	GO:00091	GO:00058	GO:00044	(sce00670)One carbon pool by folate; (sce011
Ics3p	Protein w	GO:00550	GO:00160	GO:0003674 molecular_function	
Gmc2p	Protein i	GO:00071	GO:00007	GO:0003674 molecular_function	
hypotheti	Putative	GO:00081	GO:00160	GO:0003674 molecular_function	
None	None	None	None	None	None
Rho famil	Guanine n	GO:19035	GO:00001	GO:00050	(sce04011)MAPK signaling pathway - yeast
Mms22p	Subunit o	GO:00451	GO:00353	GO:0003674 molecular_function	
Nvj1p	Nuclear e	GO:00715	GO:0071561 nucleus-vacuole junction;GO:0005640 nuclear outer m		
ribosomal	Ribosomal	GO:00004	GO:00104	GO:00037	(sce03010)Ribosome
peptidylp	Peptidylp	GO:20007	GO:00058	GO:00009	(Human)(hsa04622)RIG-I-like receptor signali
hypotheti	Putative	GO:00081	GO:00055	GO:0003674 molecular_function	
Ecm30p	Protein c	GO:00715	GO:00001	GO:0003674 molecular_function	
DNA-direc	RNA polym	GO:00427	GO:00057	GO:00010	(sce03020)RNA polymerase; (sce00240)Pyrimidi
None	None	None	None	None	None
None	None	None	None	None	None
War1p	Homodimer	GO:00614	GO:00057	GO:0000981 DNA-binding transcription factor activity,	
hypotheti	Putative	GO:00081	GO:00055	GO:0003674 molecular_function	
None	None	None	None	None	None
Ssu1p	Plasma me	GO:00003	GO:00057	GO:0000319 sulfite transmembrane transporter activity	
ribosomal	Ribosomal	GO:00021	GO:00226	GO:00037	(sce03010)Ribosome
Msp1p	Highly-co	GO:00066	GO:00057	GO:0016887 ATPase activity;GO:0017111 nucleoside-trip	
hypotheti	Putative	GO:00171	GO:00057	GO:0016603 glutaminyl-peptide cyclotransferase activi	
None	None	None	None	None	None
homeodoma	Homeobox-	GO:00011	GO:00056	GO:00083	(sce04113)Meiosis - yeast
Ctf8p	Subunit o	GO:00357	GO:00313	GO:0003677 DNA binding;GO:0003676 nucleic acid bindin	
rRNA-proc	Subunit o	GO:00004	GO:00320	GO:0003674 molecular_function	
Mrs2p	Mitochond	GO:00450	GO:00057	GO:0015095 magnesium ion transmembrane transporter ac	
hypotheti	Putative	GO:00081	GO:00160	GO:0003674 molecular_function	
mitochond	Subunit o	GO:00061	GO:00093	GO:0004591 oxoglutarate dehydrogenase (succinyl-trans	
Mig2p	Zinc fing	GO:00004	GO:00057	GO:0001227 DNA-binding transcription repressor activi	
amino aci	Mitochond	GO:00707	GO:19908	GO:0015183 L-aspartate transmembrane transporter acti	
Kap122p	Karyopher	GO:00066	GO:00056	GO:0008565 protein transporter activity;GO:0005215 tr	
putative	Putative	GO:00081	GO:00056	GO:0052689 carboxylic ester hydrolase activity;GO:001	

acid pho	Repressi	GO:00160	GO:00092	GO:00474	(sce00740)Riboflavin metabolism; (sce00730)T
Y' eleme	Helicase	GO:00007	GO:00056	GO:0003678	DNA helicase activity;GO:0004386 helicase
None	None	None	None	None	None
bifuncti	Enzyme o	GO:00061	GO:00058	GO:00039	(M00048)Inosine monophosphate biosynthesis,
Rsa3p	Protein	GO:00000	GO:00306	GO:0003674	molecular_function
Sdc1p	Subunit	GO:00063	GO:00481	GO:0042800	histone methyltransferase activity (H3-K4
ATP-bind	Plasma m	GO:00353	GO:00057	GO:00085	(sce02010)ABC transporters
Hem25p	Mitochon	GO:19049	GO:00057	GO:0015187	glycine transmembrane transporter activity
NADPH de	Conserve	GO:00069	GO:00055	GO:0018548	pentaerythritol trinitrate reductase activ
negative	Subunit	GO:00614	GO:00170	GO:0017025	TBP-class protein binding;GO:0001047 core
MRX comp	Nuclease	GO:00107	GO:00308	GO:00082	(sce03450)Non-homologous end-joining; (sce03
None	None	None	None	None	None
Thp2p	Subunit	GO:00064	GO:00004	GO:0003676	nucleic acid binding;GO:1901363 heterocycl
bifuncti	Similar	GO:00061	GO:00058	GO:00043	(sce00051)Fructose and mannose metabolism
dynein h	Cytoplas	GO:00723	GO:00002	GO:00085	(sce04145)Phagosome
dolichyl-	Alpha 1,	GO:00064	GO:00057	GO:00422	(sce)N-glycan precursor biosynthesis; (M0005
None	None	None	None	None	None
E3 ubiqu	E3 ubiqu	GO:00315	GO:00007	GO:0003688	DNA replication origin binding;GO:0004842
Ina17p	F1FO ATP	GO:00336	GO:19905	GO:0003674	molecular_function
Rdi1p	Rho GDP	GO:00328	GO:00059	GO:00050	(Human)(hsa04962)Vasopressin-regulated water
None	None	None	None	None	None
None	None	None	None	None	None
Dig2p	MAP kina	GO:20002	GO:19905	GO:00081	(sce04011)MAPK signaling pathway - yeast
glycine--	Cytoplas	GO:00701	GO:00057	GO:00048	(sce00970)Aminoacyl-tRNA biosynthesis
None	None	None	None	None	None
tyrosine	Protein	GO:00353	GO:00056	GO:00039	(sce00740)Riboflavin metabolism; (sce00730)T
Mn(2+) t	Golgi me	GO:00300	GO:00003	GO:0005384	manganese ion transmembrane transporter ac
Pcl7p	Pho85p c	GO:00059	GO:00003	GO:0016538	cyclin-dependent protein serine/threonine
Nhp10p	Non-esse	GO:00427	GO:00310	GO:0000404	heteroduplex DNA loop binding;GO:0045027 D
Lcd1p	Essentia	GO:00070	GO:00002	GO:00036	(sce04111)Cell cycle - yeast
Emp70p	Protein	GO:00014	GO:00003	GO:0003674	molecular_function
DNA repl	Subunit	GO:19029	GO:00711	GO:0043138	3'-5' DNA helicase activity;GO:0003678 DNA
U2-type	Componen	GO:00003	GO:00056	GO:0003674	molecular_function
U2 snRNP	Componen	GO:00003	GO:00056	GO:00306	(sce03040)Spliceosome
Y' eleme	Putative	GO:00081	GO:00055	GO:0004386	helicase activity;GO:0017111 nucleoside-tr
exocyst	Essentia	GO:00905	GO:00001	GO:0003674	molecular_function
hypothet	Protein	GO:00081	GO:00160	GO:0003674	molecular_function
Nst1p	Protein	GO:00096	GO:00057	GO:0003674	molecular_function
putative	Vacuolar	GO:00328	GO:00003	GO:0004674	protein serine/threonine kinase activity;G
putative	Putative	GO:00071	GO:00059	GO:0008496	mannan endo-1,6-alpha-mannosidase activity
Atg27p	Type I m	GO:00344	GO:00340	GO:00322	(sce04138)Autophagy - yeast
peptide-	Conserve	GO:00974	GO:00057	GO:00002	(sce04141)Protein processing in endoplasmic
replicat	Nucleola	GO:00315	GO:00335	GO:00431	(sce04111)Cell cycle - yeast
riboso	Ribosoma	GO:19026	GO:00226	GO:00037	(sce03010)Ribosome
glutamin	Glutamin	GO:00094	GO:00056	GO:00039	(sce00760)Nicotinate and nicotinamide metabo
RuvB fam	ATP-depe	GO:00004	GO:00057	GO:0043141	ATP-dependent 5'-3' DNA helicase activity;
Bet1p	Type II	GO:00069	GO:00001	GO:00054	(sce04130)SNARE interactions in vesicular tr
riboso	Protein	GO:00004	GO:00226	GO:00037	(sce03010)Ribosome
single-s	Poly(A+)	GO:20008	GO:00710	GO:0043047	single-stranded telomeric DNA binding;GO:0
putative	Putative	GO:00325	GO:00057	GO:0003924	GTPase activity;GO:0005525 GTP binding;GO:
Rog3p	Alpha-ar	GO:00007	GO:00058	GO:0031625	ubiquitin protein ligase binding;GO:004438
ADP-ribo	Nudix hy	GO:00196	GO:00057	GO:00476	(sce00230)Purine metabolism
Epl1p	Subunit	GO:00162	GO:00327	GO:0004402	histone acetyltransferase activity;GO:0061
cardioli	Cardioli	GO:00320	GO:00057	GO:00088	(sce00564)Glycerophospholipid metabolism; (s

Mec3p	DNA damag	GO:003331	GO:003089	GO:000367	(sce04111)Cell cycle - yeast; (sce04113)Meio
Mso1p	Lipid-int	GO:003212	GO:003310	GO:0003674 molecular_function	
septin CI	Componen	GO:003218	GO:000014	GO:0010314 phosphatidylinositol-5-phosphate binding;G	
H(+)-tra	Subunit I	GO:000703	GO:000022	GO:004696	(sce04145)Phagosome; (sce00190)Oxidative pho
Ecm11p	Meiosis-s	GO:199041	GO:000079	GO:0003674 molecular_function	
Ran GTPa	Nuclear p	GO:007152	GO:001630	GO:000853	(sce03013)RNA transport
ethanol n	Protein i	GO:000606	GO:000574	GO:000367	(sce04146)Peroxisome
hypotheti	Putative protein of unknown function; originally identified based on homology to				
translati	Gamma sub	GO:000641	GO:000585	GO:0003746 translation elongation factor activity;GO:	
hypotheti	Putative	GO:005508	GO:000032	GO:004262	(Human)(hsa04975)Fat digestion and absorptio
None	None	None	None	None	None
Ack1p	Protein t	GO:003150	GO:000573	GO:0008047 enzyme activator activity;GO:0030234 enzym	
branched-	High-affi	GO:001582	GO:000578	GO:0015171 amino acid transmembrane transporter activ	
hexokina	Putative	GO:000609	GO:000582	GO:00191	(M00549)Nucleotide sugar biosynthesis, gluco
hypotheti	Putative	GO:000815	GO:000557	GO:0003674 molecular_function	
Aim6p	Protein c	GO:000662	GO:000557	GO:0008081 phosphoric diester hydrolase activity;GO:0	
uracil ph	Uracil ph	GO:004420	(Human)GO	GO:000484	(sce00240)Pyrimidine metabolism; (sce01100)M
mitochon	Mitochon	GO:007012	GO:000576	GO:0003735 structural constituent of ribosome;GO:0005	
GTP-Rho b	Subunit c	GO:009052	GO:000562	GO:0017049 GTP-Rho binding;GO:0005546 phosphatidylino	
1,3-beta-	Catalytic	GO:000607	GO:000014	GO:000384	(sce00500)Starch and sucrose metabolism; (sc
mitochon	Mitochon	GO:003254	GO:000576	GO:007018	(sce03010)Ribosome
hypotheti	Putative	GO:001719	GO:000557	GO:0008999 ribosomal-protein-alanine N-acetyltransfer	
oxidored	Mitochon	GO:001806	GO:003131	GO:0016491 oxidoreductase activity;GO:0003824 catalyt	
polynucl	Poly(A) p	GO:007105	GO:000584	GO:000465	(sce03015)mRNA surveillance pathway
hypotheti	Putative	GO:000815	GO:000557	GO:0003674 molecular_function	
Afg1p	Protein t	GO:001655	GO:000574	GO:0016887 ATPase activity;GO:0017111 nucleoside-trip	
Mtc6p	Protein c	GO:000815	GO:000032	GO:0003674 molecular_function	
hypotheti	Putative	GO:000815	GO:000557	GO:0003674 molecular_function	
Rfm1p	Componen	GO:003229	GO:000563	GO:0030674 protein binding, bridging;GO:0060090 molec	
Tsc3p	Protein t	GO:009015	GO:003533	GO:0008047 enzyme activator activity;GO:0030234 enzym	
mitochon	Mitochon	GO:003254	GO:000576	GO:0003735 structural constituent of ribosome;GO:0005	
Tti1p	Subunit c	GO:000632	GO:007020	GO:0003674 molecular_function	
bifuncti	Bifuncti	GO:000045	GO:000573	GO:004372	(sce00740)Riboflavin metabolism; (sce01100)M
hypotheti	F-box pro	GO:003114	GO:001900	GO:0030674 protein binding, bridging;GO:0004842 ubiqu	
Dsf2p	Deletion	GO:004308	GO:000593	GO:0008047 enzyme activator activity;GO:0030234 enzym	
MCM DNA h	Protein i	GO:000626	GO:007116	GO:199016	(sce03030)DNA replication; (sce04111)Cell cy
Mfb1p	Mitochon	GO:003114	GO:003131	GO:0003674 molecular_function	
ubiquiti	Ubiquiti	GO:001099	GO:000562	GO:0004843 thiol-dependent ubiquitin-specific proteas	
Osw2p	Protein c	GO:003047	GO:000562	GO:0003674 molecular_function	
cleavage	Endoribon	GO:003424	GO:000584	GO:000840	(sce03015)mRNA surveillance pathway
phenylal	Alpha sub	GO:000643	GO:000932	GO:000482	(sce00970)Aminoacyl-tRNA biosynthesis
hypotheti	Protein c	GO:000815	GO:000578	GO:0003674 molecular_function	
N-acetyl	N-acetyl	GO:004249	GO:000563	GO:0008080 N-acetyltransferase activity;GO:0016410 N-	
Ubs1p	Ubiquiti	GO:000661	GO:000563	GO:0003674 molecular_function	
hypotheti	Non-esse	GO:000815	GO:000563	GO:0003674 molecular_function	
phosphor	Formylgl	GO:000618	GO:000573	GO:000464	(M00048)Inosine monophosphate biosynthesis,
Tda9p	Transcri	GO:001941	GO:000079	GO:0000981 DNA-binding transcription factor activity,	
hypotheti	Putative	GO:000815	GO:000557	GO:0003674 molecular_function	
glutamin	High-affi	GO:003552	GO:000593	GO:0015193 L-proline transmembrane transporter activi	
None	None	None	None	None	None
hypotheti	Putative	GO:000815	GO:001602	GO:0003674 molecular_function	
None	None	None	None	None	None
CCR4-NOT	Subunit c	GO:003296	GO:003001	GO:0003674 molecular_function	
Sm-like p	Lsm (Like	GO:000803	GO:000568	GO:000372	(sce03018)RNA degradation; (sce03040)Spliceo

Mds3p	Putative	GO:00752	GO:00057	GO:0003674	molecular_function
Lem3p	Membrane	GO:00159	GO:19905	GO:0015247	aminophospholipid transmembrane transporte
ribonucl	Subunit (	GO:00349	GO:00056	GO:00045	(sce03008)Ribosome biogenesis in eukaryotes;
Sft1p	Intra-Go	GO:00068	GO:00001	GO:00054	(sce04130)SNARE interactions in vesicular tr
Fus2p	Cell fus	GO:00313	GO:00433	GO:0005089	Rho guanyl-nucleotide exchange factor acti
putative	Nit prot	GO:00068	GO:00057	GO:0016810	hydrolase activity, acting on carbon-nitro
cyclin-d	Catalyti	GO:00459	GO:00706	GO:0008353	RNA polymerase II CTD heptapeptide repeat
Tmt1p	Trans-ac	GO:00322	GO:00058	GO:0046547	trans-aconitate 3-methyltransferase activi
farnesyl	Geranylg	GO:00333	GO:00057	GO:00041	(sce00900)Terpenoid backbone biosynthesis; (
aspartat	Aspartat	GO:00090	GO:00057	GO:00040	(sce00261)Monobactam biosynthesis; (M00018)T
hypothet	Putative	GO:00081	GO:00055	GO:0003674	molecular_function
peptidyl	Peptidyl-	GO:19017	GO:00057	GO:0005527	macrolide binding;GO:0003755 peptidyl-prol
hypothet	Putative	GO:00081	GO:00160	GO:0003674	molecular_function
Ldb17p	Protein	GO:00068	GO:00304	GO:0003674	molecular_function
succinat	Minor su	GO:00061	GO:00452	GO:00081	(sce)Citrate cycle, second carbon oxidation,
H(+)-tra	Subunit (	GO:00070	GO:00002	GO:00085	(sce04145)Phagosome; (sce00190)Oxidative pho
Trs85p	Componen	GO:00712	GO:19900	GO:0003674	molecular_function
Spo24p	Small (6	GO:00304	GO:00056	GO:0003674	molecular_function
cyclin-d	Cyclin-d	GO:19035	GO:00002	GO:00046	(sce04011)MAPK signaling pathway - yeast; (s
25S rRNA	Nucleola	GO:00311	GO:00306	GO:0106142	rRNA (adenine-N1-)-methyltransferase activ
histone	Catalyti	GO:00329	GO:00466	GO:00104	(Human)(hsa04330)Notch signaling pathway; (h
lysine--	Mitochon	GO:00701	GO:00057	GO:00048	(sce00970)Aminoacyl-tRNA biosynthesis
hypothet	Putative	GO:00081	GO:00055	GO:0003674	molecular_function
TFIIH co	Serine/t	GO:19000	GO:00709	GO:00199	(sce03022)Basal transcription factors; (sce0
putative	Putative	GO:00060	GO:00055	GO:00045	(sce00520)Amino sugar and nucleotide sugar m
Rmd9p	Mitochon	GO:00434	GO:00313	GO:0003729	mRNA binding;GO:0003723 RNA binding;GO:000
None	None	None	None	None	None
seripaup	Protein (	GO:00315	GO:00092	GO:0005199	structural constituent of cell wall;GO:000
zinc-coo	Regulate	GO:00609	GO:00056	GO:0003677	DNA binding;GO:0046872 metal ion binding;G
mitochon	Mitochon	GO:00171	GO:00057	GO:00037	(sce03010)Ribosome
protein	Essentia	GO:00450	GO:00427	GO:0008565	protein transporter activity;GO:0051082 un
None	None	None	None	None	None
hypothet	Putative	GO:00081	GO:00160	GO:0003674	molecular_function
coatomer	Zeta sub	GO:00068	GO:00301	GO:0003674	molecular_function
hypothet	Protein (	GO:00081	GO:00058	GO:0043022	ribosome binding;GO:0043021 ribonucleoprot
protein (	Member o	GO:00454	GO:00003	GO:00191	(sce04141)Protein processing in endoplasmic
TRAMP co	RNA-bind	GO:00710	GO:00314	GO:00344	(sce03018)RNA degradation
Fmc1p	Mitochon	GO:00336	GO:00057	GO:00036	(sce04139)Mitophagy - yeast
ribosoma	Ribosoma	GO:00021	GO:00226	GO:00037	(sce03010)Ribosome
Emp24p	Componen	GO:00066	GO:00301	GO:0003674	molecular_function
hypothet	Putative	GO:00081	GO:00055	GO:0003674	molecular_function
peptidyl	Peptidyl-	GO:00436	GO:00001	GO:0008160	protein tyrosine phosphatase activator act
hypothet	Putative	GO:00081	GO:00003	GO:0003674	molecular_function
tRNA spl	Subunit (	GO:00003	GO:00002	GO:0000213	tRNA-intron endonuclease activity;GO:0016
putative	Putative	GO:00322	GO:00058	GO:0043022	ribosome binding;GO:0043021 ribonucleoprot
hypothet	Putative	GO:00081	GO:00055	GO:0003674	molecular_function
palmitoy	Palmitoy	GO:00182	GO:00003	GO:0019706	protein-cysteine S-palmitoyltransferase ac
retromer	Subunit (	GO:00421	GO:00309	GO:00085	(sce04144)Endocytosis
Cbp1p	Mitochon	GO:00009	GO:00313	GO:0003729	mRNA binding;GO:0003723 RNA binding;GO:000
FG-nucle	FG-nucle	GO:00000	GO:00446	GO:00170	(sce03013)RNA transport
mitogen-	MAPKK in	GO:00001	GO:00059	GO:00047	(sce04139)Mitophagy - yeast; (sce04011)MAPK
ribonucl	Major is	GO:00092	GO:00059	GO:00047	(sce00480)Glutathione metabolism; (sce00240)
Sbe2p	Protein	GO:00315	GO:00057	GO:0003674	molecular_function
phosphat	RING-typ	GO:00165	GO:00003	GO:0032266	phosphatidylinositol-3-phosphate binding;G

RNA-deper	RNA-deper	GO:000047	GO:003068	GO:0004004	ATP-dependent RNA helicase activity;GO:000
Bsc4p	Protein (	GO:000815	GO:000557	GO:0003674	molecular_function
Zn(2+) tr	Endoplasm	GO:007157	GO:000578	GO:0005385	zinc ion transmembrane transporter activit
Add66p	Protein i	GO:004324	GO:000582	GO:0003674	molecular_function
putative	Essential	GO:000046	GO:000573	GO:0004004	ATP-dependent RNA helicase activity;GO:000
Chz1p	Histone (	GO:000633	GO:000563	GO:0042393	histone binding;GO:0005515 protein binding
None	None	None	None	None	None
seripaupe	Cell wall	GO:003150	GO:000927	GO:0005199	structural constituent of cell wall;GO:000
Erd2p	HDEL rece	GO:000662	GO:000580	GO:004501	(Human)(hsa05110)Vibrio cholerae infection
Cic1p	Essential	GO:000047	GO:003068	GO:0070628	proteasome binding;GO:0030674 protein bind
None	None	None	None	None	None
biotin--|	Biotin:ap	GO:000930	GO:000563	GO:007173	(sce00780)Biotin metabolism; (sce01100)Metab
Noc3p	Subunit (	GO:000626	GO:000565	GO:0003682	chromatin binding;GO:0005488 binding;GO:00
Plm2p	Putative	GO:000697	GO:000079	GO:0003682	chromatin binding;GO:0005488 binding;GO:00
None	None	None	None	None	None
Arf famil	GTP-bindi	GO:000702	GO:000573	GO:0005525	GTP binding;GO:0032550 purine ribonucleosi
phenylala	Mitochond	GO:007015	GO:000575	GO:000482	(sce00970)Aminoacyl-tRNA biosynthesis
leucine--	Mitochond	GO:000642	GO:000575	GO:000482	(sce00970)Aminoacyl-tRNA biosynthesis
None	None	None	None	None	None
ribosomal	Protein (	GO:000640	GO:002262	GO:000373	(sce03010)Ribosome
hypotheti	Putative	GO:000815	GO:000557	GO:0003674	molecular_function
Sds3p	Component	GO:004370	GO:003369	GO:0004407	histone deacetylase activity;GO:0033558 pr
prolifera	Prolifera	GO:190239	GO:004362	GO:003033	(sce03410)Base excision repair; (sce03430)Mi
cAMP-depe	cAMP-depe	GO:001060	GO:000595	GO:000469	(sce04213)Longevity regulating pathway - mul
SUMO-targ	Subunit (	GO:001692	GO:003376	GO:0004842	ubiquitin-protein transferase activity;GO:
hypotheti	Putative protein of unknown function; conserved in fungi; identified by expressi				
serine/th	Serine/th	GO:000711	GO:000562	GO:0004674	protein serine/threonine kinase activity;G
CORVET co	Component	GO:000703	GO:003326	GO:000367	(sce04138)Autophagy - yeast
F0 ATP sy	F0-ATP sy	GO:001598	GO:000027	GO:004693	(sce00190)Oxidative phosphorylation; (sce011
serine/th	Upstream	GO:000597	GO:000582	GO:000467	(sce04138)Autophagy - yeast
Mub1p	MYND doma	GO:000651	GO:199030	GO:0046872	metal ion binding;GO:0043169 cation bindin
CCR4-NOT	Ubiquitin	GO:003108	GO:003001	GO:000484	(sce03018)RNA degradation
Tma22p	Protein (	GO:000218	GO:000584	GO:0043024	ribosomal small subunit binding;GO:0003743
U2-type s	Non-esser	GO:004529	GO:000568	GO:000372	(sce03040)Spliceosome
condensin	Subunit (	GO:190334	GO:000079	GO:004239	(sce04111)Cell cycle - yeast
TFIIH/NEF	5' to 3'	GO:004595	GO:000567	GO:004314	(sce03022)Basal transcription factors; (sce0
hypotheti	Dubious (	GO:000815	GO:000557	GO:0003674	molecular_function
Kre28p	Subunit (	GO:000815	GO:000077	GO:000367	(sce03013)RNA transport
hypotheti	Protein (	GO:007236	GO:000563	GO:0000978	RNA polymerase II proximal promoter sequen
histone a	Subunit (	GO:004396	GO:004669	GO:0004402	histone acetyltransferase activity;GO:0061
None	None	None	None	None	None
alpha-1,3	Alpha-1,3	GO:000649	GO:000013	GO:000003	(sce00514)Other types of O-glycan biosynthes
Tom22p	Component	GO:004504	GO:000574	GO:0008320	protein transmembrane transporter activity
transcrip	Subunit (	GO:004279	GO:000012	GO:0001002	RNA polymerase III type 1 promoter sequenc
putative	Iron-sulf	GO:003159	GO:000578	GO:0102521	tRNA-4-demethylwyosine synthase activity;G
None	None	None	None	None	None
None	None	None	None	None	None
U2 snRNP	U2-snRNP	GO:000024	GO:000568	GO:000372	(sce03040)Spliceosome
tryptopha	Mitochond	GO:007018	GO:000575	GO:000483	(sce00970)Aminoacyl-tRNA biosynthesis
None	None	None	None	None	None
Ldb16p	Protein i	GO:003438	GO:003017	GO:0003674	molecular_function
ribosomal	Protein (	GO:000218	GO:002262	GO:000373	(sce03010)Ribosome
ubiquitin	Ubiquitin	GO:000005	GO:002262	GO:003138	(sce03010)Ribosome
cysteine	Conserved	GO:005169	GO:000582	GO:001978	(sce04136)Autophagy - other; (sce04138)Autop

Tho1p	Conserve(	GO:00169	GO:00056	GO:0003682 chromatin binding;GO:0003690 double-strand	
Ldb19p	Alpha-ar	GO:00020	GO:00301	GO:0031625 ubiquitin protein ligase binding;GO:004438	
DNA-bind	Transcri	GO:00601	GO:00058	GO:0000987 proximal promoter sequence-specific DNA bi	
acylglyc	Bifuncti	GO:00194	GO:00057	GO:01025	(sce00565)Ether lipid metabolism; (sce00564)
Add37p	Protein (	GO:00304	GO:00057	GO:0003674 molecular_function	
hypotheti	Putative	GO:00081	GO:00055	GO:0003674 molecular_function	
tRNase Z	tRNA 3'-e	GO:00726	GO:00057	GO:00427	(sce03013)RNA transport
homoseri	Homoseri	GO:00090	GO:00055	GO:00044	(M00018)Threonine biosynthesis, aspartate =>
pseudour	tRNA:pse	GO:00311	GO:00056	GO:0009982 pseudouridine synthase activity;GO:0016866	
Egd1p	Subunit	GO:00066	GO:00058	GO:0051082 unfolded protein binding;GO:0005515 protei	
None	None	None	None	None	None
RNA-bind	Constitu	GO:00000	GO:00306	GO:0003729 mRNA binding;GO:0003723 RNA binding;GO:000	
Bbp1p	Protein	GO:00719	GO:00058	GO:0005198 structural molecule activity;GO:0003674 mo	
DNA topo	Topoisom	GO:00970	GO:00007	GO:00039	(Human)(hsa01524)Platinum drug resistance
hypotheti	Non-esse	GO:00081	GO:00056	GO:0003674 molecular_function	
Stp2p	Transcri	GO:00459	GO:00058	GO:0000978 RNA polymerase II proximal promoter sequen	
Sln1p	Transmem	GO:00072	GO:00058	GO:00001	(sce04139)Mitophagy - yeast; (sce04011)MAPK
Rrt8p	Protein	GO:00304	GO:00056	GO:0003674 molecular_function	
metal ca	Vacuolar	GO:00068	GO:00003	GO:0015087 cobalt ion transmembrane transporter activ	
serine/t	Ser/Thr	GO:00009	GO:00058	GO:00046	(sce04111)Cell cycle - yeast
hypotheti	Pseudoge	GO:00081	GO:00055	GO:0003674 molecular_function	
casein k	Palmitoy	GO:00181	GO:00003	GO:0004674 protein serine/threonine kinase activity;G	
Nup157p	Subunit	GO:00362	GO:00446	GO:00170	(sce03013)RNA transport
transcri	Subunit	GO:00427	GO:00001	GO:0001002 RNA polymerase III type 1 promoter sequenc	
alanine--	Cytoplas	GO:00701	GO:00057	GO:00048	(sce00970)Aminoacyl-tRNA biosynthesis
SKI comp	Ski comp	GO:00704	GO:00550	GO:00036	(sce03018)RNA degradation
Apm4p	Cargo-bi	GO:00068	GO:00301	GO:00055	(sce04144)Endocytosis
None	None	None	None	None	None
DNA-dire	RNA poly	GO:00427	GO:00057	GO:00010	(sce03020)RNA polymerase; (sce00240)Pyrimidi
putative	Putative protein of unknown function; green fluorescent protein (GFP)-fusion pro				
metalloe	SUMO-lig	GO:19904	GO:00003	GO:0070140 SUMO-specific isopeptidase activity;GO:007	
glutamyl-	Subunit	GO:00706	GO:00309	GO:00505	(sce00970)Aminoacyl-tRNA biosynthesis; (sce0
Set6p	SET doma	GO:00322	GO:00055	GO:0008168 methyltransferase activity;GO:0016741 tran	
Hir2p	Subunit	GO:00307	GO:00004	GO:0031491 nucleosome binding;GO:0003714 transcriptio	
Aep1p	Protein	GO:00064	GO:00057	GO:0045182 translation regulator activity;GO:0003674	
separase	Separase,	GO:00328	GO:00447	GO:00041	(sce04111)Cell cycle - yeast; (sce04113)Meio
Tel2p	Subunit	GO:00070	GO:00702	GO:0042162 telomeric DNA binding;GO:0043565 sequence-	
Bsp1p	Adapter	GO:00516	GO:00304	GO:0030674 protein binding, bridging;GO:0060090 molec	
Swc7p	Protein	GO:00063	GO:00008	GO:0003674 molecular_function	
Whi4p	Putative	GO:00611	GO:00056	GO:0035614 snRNA stem-loop binding;GO:0030619 U1 snRN	
ribosoma	Protein	GO:00064	GO:00226	GO:00198	(sce03010)Ribosome
None	None	None	None	None	None
None	None	None	None	None	None
Yra2p	Member o	GO:00169	GO:00056	GO:0003723 RNA binding;GO:0003677 DNA binding;GO:0003	
Sgm1p	Protein	GO:00081	GO:00301	GO:0003674 molecular_function	
hypotheti	Dubious	GO:00081	GO:00055	GO:0003674 molecular_function	
Egd2p	Alpha su	GO:00066	GO:00058	GO:0080025 phosphatidylinositol-3,5-bisphosphate bind	
tricalbi	ER prote	GO:00603	GO:00325	GO:0008289 lipid binding;GO:0005488 binding;GO:000367	
Irc6p	Clathrin	GO:00161	GO:00055	GO:0030674 protein binding, bridging;GO:0060090 molec	
mitochon	Mitochon	GO:00325	GO:00057	GO:0070180 large ribosomal subunit rRNA binding;GO:00	
ditrans,	Forms th	GO:00194	GO:19044	GO:00455	(sce00900)Terpenoid backbone biosynthesis; (
hypotheti	Putative	GO:00081	GO:00055	GO:0003674 molecular_function	
mRNA spl	Member o	GO:00003	GO:00710	GO:00003	(sce03040)Spliceosome
peptidyl	Peptidyl-	GO:00004	GO:00057	GO:0016018 cyclosporin A binding;GO:0003755 peptidyl-	

gamma-tul	Gamma-tul	GO:00315	GO:0005824 outer plaque of spindle pole body;GO:0005816 spindl		
Imo32p	Conserve	GO:00081	GO:00057	GO:0016787 hydrolase activity;GO:0003824 catalytic ac	
Iml3p	Outer ki	GO:00340	GO:00009	GO:0003674 molecular_function	
Tma16p	Protein	GO:00081	GO:00056	GO:0003674 molecular_function	
Sef1p	Putative	GO:00304	GO:00056	GO:0000981 DNA-binding transcription factor activity,	
tRNA guar	tRNAHis	GO:00991	GO:00056	GO:0008193 tRNA guanylyltransferase activity;GO:00081	
Kap120p	Karyophe	GO:00066	GO:00056	GO:0008139 nuclear localization sequence binding;GO:0	
hypotheti	Protein	GO:00063	GO:00726	GO:0043022 ribosome binding;GO:0043021 ribonucleoprot	
histone H	Histone H	GO:00319	GO:00007	GO:0031492 nucleosomal DNA binding;GO:0031491 nucleos	
palmitoyl	Vesicle m	GO:00421	GO:00312	GO:00164	(sce04130)SNARE interactions in vesicular tra
Par32p	Protein	GO:00081	GO:00057	GO:0003674 molecular_function	
Nas6p	Evolution	GO:00706	GO:00058	GO:1904855 proteasome regulatory particle binding;GO:	
Erb1p	Constitu	GO:00004	GO:00705	GO:0070180 large ribosomal subunit rRNA binding;GO:00	
putative	Putative	GO:00163	GO:00055	GO:1990738 pseudouridine 5'-phosphatase activity;GO:0	
ATP-deper	Subunit	GO:00991	GO:00991	GO:00040	(sce03450)Non-homologous end-joining
acetolact	Acetolac	GO:00090	GO:00059	GO:00039	(sce00660)C5-Branched dibasic acid metabolis
H(+)-trar	Subunit	GO:00070	GO:00002	GO:00469	(sce04145)Phagosome; (sce00190)Oxidative pho
Pet309p	Specific	GO:00701	GO:00313	GO:0045182 translation regulator activity;GO:0003729	
Sir1p	Protein	GO:00063	GO:00056	GO:0043565 sequence-specific DNA binding;GO:0003677 D	
Mud2p	Protein	GO:00003	GO:00002	GO:00451	(sce03040)Spliceosome
Tof2p	Protein	GO:00310	GO:00057	GO:0000182 rDNA binding;GO:0019211 phosphatase activa	
None	None	None	None	None	None
derlin	ER membra	GO:00708	GO:00008	GO:00517	(sce04141)Protein processing in endoplasmic
DNA prima	Subunit	GO:00062	GO:00056	GO:00038	(sce03030)DNA replication; (sce00240)Pyrimid
None	None	None	None	None	None
None	None	None	None	None	None
None	None	None	None	None	None
None	None	None	None	None	None
Mcm22p	Outer ki	GO:00340	GO:00007	GO:0003674 molecular_function	
hypotheti	Putative	GO:00081	GO:00055	GO:0003674 molecular_function	
Aua1p	Protein	GO:00068	GO:00055	GO:0003674 molecular_function	
hypotheti	Protein	GO:00081	GO:00055	GO:0017137 Rab GTPase binding;GO:0017016 Ras GTPase b	
succinate	Subunit	GO:00061	GO:00057	GO:00081	(sce)Citrate cycle, second carbon oxidation,
branched-	Regulato	GO:00901	GO:00058	GO:00474	(sce00010)Glycolysis / Gluconeogenesis; (sce
Mtc5p	Subunit	GO:19042	GO:00970	GO:00036	(Human)(hsa04150)mTOR signaling pathway
Sae3p	Meiosis-	GO:00007	GO:00327	GO:0003674 molecular_function	
Rab famil	Rab famil	GO:00003	GO:00058	GO:0003924 GTPase activity;GO:0005525 GTP binding;GO:	
nucleotic	Nucleoti	GO:00160	GO:00160	GO:0035529 NADH pyrophosphatase activity;GO:0004528 p	
hypotheti	Protein	GO:00081	GO:00003	GO:0003674 molecular_function	
hypotheti	Putative	GO:00081	GO:00055	GO:0003674 molecular_function	
None	None	None	None	None	None
mannosyli	Endoplasm	GO:00066	GO:00301	GO:0030234 enzyme regulator activity;GO:0098772 molec	
hypotheti	Dubious	GO:00081	GO:00055	GO:0003674 molecular_function	
anaphase	Subunit	GO:00322	GO:00056	GO:00616	(sce04120)Ubiquitin mediated proteolysis; (s
cytochror	Subunit	GO:00061	GO:00704	GO:00041	(sce00190)Oxidative phosphorylation; (sce011
None	None	None	None	None	None
protein	Mitochon	GO:00066	GO:00313	GO:0008320 protein transmembrane transporter activity	
Tok1p	Outward-	GO:00300	GO:00058	GO:0022841 potassium ion leak channel activity;GO:000	
Syg1p	Plasma m	GO:00071	GO:00003	GO:0003674 molecular_function	
Rrp1p	Essentia	GO:00063	GO:00306	GO:0003674 molecular_function	
None	None	None	None	None	None
Pex8p	Intraper	GO:00165	GO:00057	GO:0030674 protein binding, bridging;GO:0060090 molec	
Yta7p	Protein	GO:20002	GO:00424	GO:0042393 histone binding;GO:0016887 ATPase activity	
Gir2p	Highly-a	GO:19038	GO:00058	GO:0005515 protein binding;GO:0005488 binding;GO:0003	

None	None	None	None	None	None
Yvc1p	Vacuolar	GO:00975	GO:19908	GO:0005227	calcium activated cation channel activity;
Qdr3p	Multidrug	GO:00105	GO:00058	GO:0015203	polyamine transmembrane transporter activi
None	None	None	None	None	None
NEDD8-act	Protein	GO:00451	GO:00056	GO:00197	(sce04120)Ubiquitin mediated proteolysis
triglycer	Triacylgl	GO:00194	GO:00057	GO:00048	(sce)Acylglycerol degradation; (M00098)Acylg
putative	Protein a	GO:00068	GO:00057	GO:0008379	thioredoxin peroxidase activity;GO:0051920
None	None	None	None	None	None
beta-gluc	Type II	GO:00060	GO:00059	GO:0015926	glucosidase activity;GO:0004553 hydrolase
None	None	None	None	None	None
putative	Putative	GO:00196	GO:00057	GO:00475	(sce00640)Propanoate metabolism
Ppq1p	Protein p	GO:00900	GO:00056	GO:00047	(sce03015)mRNA surveillance pathway; (sce041
Tif3p	Translati	GO:00017	GO:00104	GO:00340	(sce03013)RNA transport
Spt21p	Protein w	GO:00304	GO:00007	GO:0003674	molecular_function
Ccz1p	Subunit c	GO:00443	GO:00325	GO:00103	(sce04138)Autophagy - yeast
epoxide h	Epoxide h	GO:00096	GO:00055	GO:0004301	epoxide hydrolase activity;GO:0016803 ethe
hypotheti	Protein c	GO:00345	GO:00057	GO:0003674	molecular_function
ribosomal	Protein c	GO:00004	GO:00226	GO:00037	(sce03010)Ribosome
mRNA spli	Core Sm p	GO:00003	GO:00056	GO:00037	(sce03040)Spliceosome
hypotheti	Putative	GO:00081	GO:00055	GO:0003674	molecular_function
ribonucle	Subunit c	GO:00349	GO:00056	GO:00421	(sce03008)Ribosome biogenesis in eukaryotes;
Tda3p	Putative	GO:00421	GO:00057	GO:0016491	oxidoreductase activity;GO:0003824 catalyt
copper ch	Copper ch	GO:00194	GO:00057	GO:00165	(Human)(hsa05014)Amyotrophic lateral scleros
bifunctio	Bifunctio	GO:00196	GO:00055	GO:0047443	4-hydroxy-4-methyl-2-oxoglutarate aldolase
None	None	None	None	None	None
chaperoni	Alpha sub	GO:00064	GO:00058	GO:0051082	unfolded protein binding;GO:0005524 ATP bi
Hek2p	RNA bindi	GO:00482	GO:00007	GO:0003729	mRNA binding;GO:0003723 RNA binding;GO:000
Tex1p	Protein i	GO:00064	GO:00004	GO:00037	(sce03040)Spliceosome; (sce03013)RNA transpo
DNA-direc	RNA polym	GO:00427	GO:00057	GO:00010	(sce03020)RNA polymerase; (sce00240)Pyrimidi
holo-[acy	Phosphopa	GO:00198	GO:00057	GO:00088	(sce00770)Pantothenate and CoA biosynthesis
None	None	None	None	None	None
putative	Calcium t	GO:00324	GO:00057	GO:0005384	manganese ion transmembrane transporter ac
cation-tr	Cadmium t	GO:00705	GO:00058	GO:0008551	cadmium-exporting ATPase activity;GO:00436
Hfi1p	Adaptor p	GO:00439	GO:00466	GO:0004402	histone acetyltransferase activity;GO:0061
Gep5p	Protein c	GO:00000	GO:00057	GO:0003674	molecular_function
None	None	None	None	None	None
Vps74p	Golgi pho	GO:00603	GO:00057	GO:00702	(Human)(hsa05202)Transcriptional misregulati
None	None	None	None	None	None
RNA polym	Phosphata	GO:00315	GO:00058	GO:00084	(sce03015)mRNA surveillance pathway
Srd1p	Protein i	GO:00063	GO:00056	GO:0008270	zinc ion binding;GO:0043565 sequence-speci
origin re	Subunit c	GO:00062	GO:00056	GO:00036	(sce04111)Cell cycle - yeast; (sce04113)Meio
RuvB fami	ATP-depen	GO:00004	GO:00352	GO:00431	(Human)(hsa04310)Wnt signaling pathway
None	None	None	None	None	None
hypotheti	Putative	GO:00081	GO:00055	GO:0003674	molecular_function
threonine	Threonine	GO:00065	GO:00058	GO:00087	(sce00260)Glycine, serine and threonine metal
DNA-bindi	Essential	GO:00458	GO:00002	GO:0017025	TBP-class protein binding;GO:0004386 helic
NEDD8-con	Enzyme th	GO:00451	GO:00055	GO:00197	(sce04120)Ubiquitin mediated proteolysis
FG-nucleo	FG-nucleo	GO:00000	GO:00446	GO:0000774	adenyl-nucleotide exchange factor activity
None	None	None	None	None	None
hypotheti	Putative	GO:00081	GO:00055	GO:0003674	molecular_function
putative	Protein i	GO:00332	GO:00003	GO:0005381	iron ion transmembrane transporter activit
Rrg1p	Protein c	GO:00070	GO:00057	GO:0003674	molecular_function
ubiquinon	Protein r	GO:19010	GO:00057	GO:0008289	lipid binding;GO:0005488 binding;GO:00367
None	None	None	None	None	None

Nca2p	Protein i	GO:00090(	GO:00057(	GO:0003674 molecular_function	
HSP70/90	TPR-conta	GO:00420(	GO:00057(	GO:0051879 Hsp90 protein binding;GO:0030544 Hsp70 pro	
H(+)-trar	Subunit (	GO:00070(	GO:00002(	GO:00085(	(sce04145)Phagosome; (sce00190)Oxidative pho
protein-i	Essential	GO:00000(	GO:00199(	GO:0019207 kinase regulator activity;GO:0015631 tubul	
SCF ubiqu	F-box pro	GO:00311(	GO:00432(	GO:00508(	(sce04120)Ubiquitin mediated proteolysis; (s
triacylgl	Serine hy	GO:00550(	GO:00058(	GO:0004806 triglyceride lipase activity;GO:0016298 li	
Rrt7p	Putative	GO:00081(	GO:00160(	GO:0003674 molecular_function	
ERMES cor	Mitochond	GO:19904(	GO:00328(	GO:00082(	(sce04139)Mitophagy - yeast
Hua2p	Cytoplasm	GO:00081(	GO:00057(	GO:0003674 molecular_function	
MIND comp	Essential	GO:00514(	GO:00009(	GO:0003674 molecular_function	
Rec8p	Meiosis-s	GO:00071(	GO:00007(	GO:00036(	(sce04113)Meiosis - yeast
karyopher	Karyopher	GO:00601(	GO:00425(	GO:00050(	(sce03013)RNA transport
ribosomal	Ribosomal	GO:00021(	GO:00226(	GO:00037(	(sce03010)Ribosome
hypotheti	Helicase-	GO:00081(	GO:00055(	GO:0004386 helicase activity;GO:0017111 nucleoside-tr	
Tcb3p	Cortical	GO:00603(	GO:00325(	GO:0005544 calcium-dependent phospholipid binding;GO:	
AMP-activ	AMP-activ	GO:20002(	GO:00001(	GO:00046(	(sce04138)Autophagy - yeast; (sce04113)Meios
Skt5p	Activator	GO:00342(	GO:00001(	GO:0008047 enzyme activator activity;GO:0030234 enzym	
ubiquinor	Protein w	GO:00067(	GO:00057(	GO:0003674 molecular_function	
Ctf13p	Subunit (	GO:00009(	GO:00315(	GO:0019237 centromeric DNA binding;GO:0008301 DNA bin	
type I HS	Co-chaper	GO:00304(	GO:00057(	GO:00016(	(sce04141)Protein processing in endoplasmic
glucose-i	Glycerol	GO:00157(	GO:00003(	GO:0005351 carbohydrate:proton symporter activity;GO:	
aspartate	Cytosolic	GO:00192(	GO:00057(	GO:00040(	(sce00360)Phenylalanine metabolism; (sce0035
Sgn1p	Cytoplasm	GO:00160(	GO:00057(	GO:00081(	(sce03015)mRNA surveillance pathway
MCM DNA h	Protein i	GO:19024(	GO:00711(	GO:00171(	(sce03030)DNA replication; (sce04111)Cell cy
clathrin	Clathrin	GO:00068(	GO:00301(	GO:00320(	(sce04144)Endocytosis
succinate	Protein i	GO:00061(	GO:00057(	(Human)GO:0005515 protein binding;GO:0005488 binding;	
None	None	None	None	None	None
None	None	None	None	None	None
Arp7p	Component	GO:00427(	GO:00165(	GO:0015616 DNA translocase activity;GO:0008094 DNA-de	
ubiquitir	Ubiquitir	GO:00991(	GO:00991(	GO:0004843 thiol-dependent ubiquitin-specific proteas	
chromatir	Chromatir	GO:00007(	GO:00312(	GO:0003682 chromatin binding;GO:0003677 DNA binding;G	
hypotheti	Protein w	GO:00067(	GO:00056(	GO:0016491 oxidoreductase activity;GO:0003824 catalyt	
Mer1p	mRNA-binc	GO:00480(	GO:00056(	GO:1990446 U1 snRNP binding;GO:0070990 snRNP binding;	
Mss18p	Nuclear e	GO:00003(	GO:00057(	GO:0003674 molecular_function	
Thi74p	Mitochond	GO:00081(	GO:00003(	GO:0003674 molecular_function	
Sfc1p	Mitochond	GO:00157(	GO:00057(	GO:0005469 succinate:fumarate antiporter activity;GO:	
ubiquitir	Ubiquitir	GO:00109(	GO:00057(	GO:0004843 thiol-dependent ubiquitin-specific proteas	
DNA-direc	RNA polym	GO:00427(	GO:00056(	GO:0001056 RNA polymerase III activity;GO:0003899 DNA-	
Erp4p	Member of	GO:00066(	GO:00301(	GO:0003674 molecular_function	
Elongator	Subunit (	GO:00020(	GO:00335(	GO:0000049 tRNA binding;GO:0016887 ATPase activity;GO	
carnitine	Mitochond	GO:00066(	GO:00057(	GO:0005476 carnitine:acyl carnitine antiporter activi	
Mim1p	Mitochond	GO:00450(	GO:00313(	GO:0003674 molecular_function	
pyrroline	Delta 1-p	GO:00551(	GO:00057(	GO:00047(	(M00015)Proline biosynthesis, glutamate => p
None	None	None	None	None	None
Yaf9p	Subunit (	GO:00063(	GO:00352(	GO:0005515 protein binding;GO:0005488 binding;GO:0003	
Bph1p	Protein b	GO:00315(	GO:00003(	GO:0003674 molecular_function	
Uso1p	Essential	GO:00482(	GO:00125(	GO:0008565 protein transporter activity;GO:0005215 tr	
Kxd1p	Subunit (	GO:00070(	GO:00310(	GO:0003674 molecular_function	
Mam33p	Specific	GO:00701(	GO:00057(	GO:0097177 mitochondrial ribosome binding;GO:0008494	
None	None	None	None	None	None
ubiquitir	Fusion pr	GO:00021(	GO:00226(	GO:00313(	(sce03010)Ribosome
mannosyli	Mannosyli	GO:00519(	GO:00003(	GO:0000030 mannosyltransferase activity;GO:0016758 tr	
Rfa2p	Subunit (	GO:20000(	GO:00056(	GO:00985(	(sce03440)Homologous recombination; (sce0343
RSC subur	Component	GO:00421(	GO:00165(	GO:0015616 DNA translocase activity;GO:0008094 DNA-de	

hypotheti	Putative	GO:00081	GO:00057	GO:0003674	molecular_function
Erp6p	Member o	GO:00070	GO:00301	GO:0003674	molecular_function
actin	Actin; s	GO:00071	GO:00001	GO:00044	(sce04145)Phagosome
1,3-beta-	Catalyti	GO:00304	GO:00001	GO:00038	(sce00500)Starch and sucrose metabolism; (sc
Rgd2p	GTPase-a	GO:00435	GO:00009	GO:0005096	GTPase activator activity;GO:0030695 GTPas
DNA-(apu	Class II	GO:00062	GO:00056	GO:00083	(sce03410)Base excision repair
alpha-1,	Alpha-1,	GO:00060	GO:00001	GO:00000	(sce)N-glycan biosynthesis, high-mannose typ
Thp1p	Nuclear	GO:00710	GO:00703	GO:0003690	double-stranded DNA binding;GO:0044877 pro
tRNA (gu	tRNA met	GO:00029	GO:00056	GO:0004809	tRNA (guanine-N2-)-methyltransferase activ
coenzyme	Mitochon	GO:00353	GO:00057	GO:0015228	coenzyme A transmembrane transporter activ
Vma21p	Integral	GO:00700	GO:00125	GO:0003674	molecular_function
None	None	None	None	None	None
hypotheti	Protein	GO:00081	GO:00056	GO:0003674	molecular_function
ribosoma	Ribosoma	GO:00021	GO:00226	GO:00037	(sce03010)Ribosome
None	None	None	None	None	None
GPI-anch	Cell wal	GO:00315	GO:00092	GO:0005199	structural constituent of cell wall;GO:000
hypotheti	Putative	GO:00081	GO:00301	GO:0003674	molecular_function
H(+)-tra	Subunit	GO:00070	GO:00164	GO:00469	(sce04145)Phagosome; (sce00190)Oxidative pho
WD-repea	Subunit	GO:00347	GO:00481	GO:00428	(sce03015)mRNA surveillance pathway
Bop3p	Protein	GO:00714	GO:00056	GO:0003674	molecular_function
kexin KE	Kexin, a	GO:00073	GO:00301	GO:0004252	serine-type endopeptidase activity;GO:0008
mitochon	Mitochon	GO:00325	GO:00057	GO:00037	(sce03010)Ribosome
Taf8p	TFIID su	GO:00511	GO:00056	GO:00162	(sce03022)Basal transcription factors
None	None	None	None	None	None
Ste18p	G protei	GO:00007	GO:01201	GO:00316	(sce04011)MAPK signaling pathway - yeast
hypotheti	Putative protein of unknown function; identified by expression profiling and mas				
Pcl10p	Pho85p c	GO:00457	GO:00003	GO:0016538	cyclin-dependent protein serine/threonine
None	None	None	None	None	None
centrin	Calcium-	GO:00304	GO:00058	GO:0005509	calcium ion binding;GO:0046872 metal ion b
hypotheti	Dubious	GO:00081	GO:00160	GO:0003674	molecular_function
cohesin	Subunit	GO:00070	GO:00349	GO:00036	(sce04111)Cell cycle - yeast; (sce04113)Meio
uroporph	S-adenos	GO:00193	GO:00056	GO:00048	(sce00860)Porphyrin and chlorophyll metaboli
putative	Putative	GO:00081	GO:00055	GO:00438	(sce00360)Phenylalanine metabolism; (sce0038
Cbc2p	Small su	GO:00001	GO:00058	GO:00003	(sce03015)mRNA surveillance pathway; (sce030
hypotheti	Putative	GO:19023	GO:00003	GO:0008271	secondary active sulfate transmembrane tra
hypotheti	Putative	GO:00081	GO:00057	GO:0003674	molecular_function
None	None	None	None	None	None
dolichyl-	Subunit	GO:00182	GO:00082	GO:00045	(M00072)N-glycosylation by oligosaccharyltra
None	None	None	None	None	None
ubiquino	Coenzyme	GO:00067	GO:00057	GO:0048039	ubiquinone binding;GO:0048038 quinone bind
None	None	None	None	None	None
Sfl1p	Transcri	GO:19004	GO:00056	GO:0001103	RNA polymerase II repressing transcription
Dcc1p	Subunit	GO:00357	GO:00313	GO:0003689	DNA clamp loader activity;GO:0033170 prote
Tlg2p	Syntaxin-	GO:00066	GO:00313	GO:00054	(sce04130)SNARE interactions in vesicular tr
Nan1p	U3 snoRN	GO:00459	GO:00344	GO:00345	(sce03008)Ribosome biogenesis in eukaryotes
translati	eIF3c su	GO:00064	GO:00436	GO:00313	(sce03013)RNA transport
UTP gluc	UDP-gluc	GO:00060	GO:00104	GO:00039	(M00549)Nucleotide sugar biosynthesis, gluco
ubiquiti	Componen	GO:00457	GO:00346	GO:0004842	ubiquitin-protein transferase activity;GO:
mini-chr	Componen	GO:00062	GO:00711	GO:19901	(sce03030)DNA replication; (sce04111)Cell cy
SAM comp	Componen	GO:00450	GO:00014	GO:0003674	molecular_function
histone	NAD(+)-d	GO:19906	GO:00057	GO:00451	(sce00760)Nicotinate and nicotinamide metabo
Nsg1p	Protein	GO:00161	GO:00343	GO:0051082	unfolded protein binding;GO:0005515 protei
Hsp70 fa	Mitochon	GO:00068	GO:00057	GO:0044183	protein binding involved in protein foldin
putative	Deubiqui	GO:00165	GO:00057	GO:0004843	thiol-dependent ubiquitin-specific proteas

Rrp5p	RNA bindi	GO:00004	GO:00306	GO:0034512 box C/D snoRNA binding;GO:0042134 rRNA pri	
Cdc73p	Componen	GO:20012	GO:00165	GO:1990269 RNA polymerase II C-terminal domain phosph	
Pet8p	S-adenos	GO:19019	GO:00057	GO:0000095 S-adenosyl-L-methionine transmembrane tran	
Rab fami	Rab fami	GO:00344	GO:00059	GO:00039	(sce04144)Endocytosis
Ctp1p	Mitochon	GO:00068	GO:00057	GO:0005371 tricarboxylate secondary active transmembr	
Ndl1p	Homolog	GO:00304	GO:00058	GO:0051010 microtubule plus-end binding;GO:0008017 mi	
phosphat	PIK-rela	GO:19053	GO:00319	GO:00044	(sce04136)Autophagy - other; (sce04213)Longe
dolichyl-	Epsilon	GO:00064	GO:00082	GO:00045	(M00072)N-glycosylation by oligosaccharyltra
hypotheti	Protein	GO:00081	GO:00058	GO:0003674 molecular_function	
None	None	None	None	None	None
Rrt15p	Putative	GO:00081	GO:00055	GO:0003674 molecular_function	
Pho81p	Cyclin-d	GO:00457	GO:00058	GO:00048	(sce04111)Cell cycle - yeast
hypotheti	Putative	GO:00081	GO:00055	GO:0003674 molecular_function	
None	None	None	None	None	None
Efm4p	Lysine m	GO:00180	GO:00057	GO:0016279 protein-lysine N-methyltransferase activit	
serine/t	Protein	GO:00072	GO:00057	GO:0004683 calmodulin-dependent protein kinase activi	
hypotheti	Protein	GO:19026	GO:00313	GO:0003674 molecular_function	
DNA-dire	DNA Poly	GO:00093	GO:00057	GO:0000182 rDNA binding;GO:0003887 DNA-directed DNA p	
None	None	None	None	None	None
ribosoma	Ribosoma	GO:00000	GO:00226	GO:00037	(sce03010)Ribosome
snoRNP c	Essentia	GO:00001	GO:00314	GO:00305	(sce03008)Ribosome biogenesis in eukaryotes
hypotheti	Putative	GO:00081	GO:00055	GO:0005524 ATP binding;GO:0032559 adenyl ribonucleoti	
Trs33p	Core com	GO:00344	GO:19900	GO:0003674 molecular_function	
FG-nucle	FG-nucle	GO:00319	GO:00446	GO:0017056 structural constituent of nuclear pore;GO:	
signal p	18kDa ca	GO:00064	GO:00057	GO:00082	(sce03060)Protein export
thioredo	Mitochon	GO:00988	GO:00057	GO:00083	(Human)(hsa01100)Metabolic pathways
Gef1p	Voltage-	GO:00068	GO:00057	GO:0005247 voltage-gated chloride channel activity;GO	
Msc3p	Protein	GO:00071	GO:00058	GO:0003674 molecular_function	
coatomer	Alpha su	GO:00068	GO:00301	GO:0043130 ubiquitin binding;GO:0032182 ubiquitin-lik	
None	None	None	None	None	None
mitochon	Mitochon	GO:00325	GO:00057	GO:0003735 structural constituent of ribosome;GO:0005	
None	None	None	None	None	None
low-affi	Low-affi	GO:00156	GO:00003	GO:0005375 copper ion transmembrane transporter activ	
Cin10p	Putative	GO:00342	GO:00003	GO:0005351 carbohydrate:proton symporter activity;GO:	
None	None	None	None	None	None
Tos2p	Protein	GO:19024	GO:00331	GO:0003674 molecular_function	
putative	Putative	GO:00000	GO:00306	GO:19902	(sce03008)Ribosome biogenesis in eukaryotes
ribosoma	Ribosoma	GO:19026	GO:00226	GO:00037	(sce03010)Ribosome
cofilin	Cofilin,	GO:00430	GO:00304	GO:00510	(Human)(hsa05133)Pertussis; (hsa04666)Fc gam
SNAP rec	Vesicle	GO:00068	GO:00306	GO:00054	(sce04130)SNARE interactions in vesicular tr
FG-nucle	FG-nucle	GO:00362	GO:00446	GO:00170	(sce03013)RNA transport
None	None	None	None	None	None
U4/U6-U5	Componen	GO:00003	GO:00465	GO:00306	(sce03040)Spliceosome
Mss2p	Periphera	GO:00329	GO:00313	GO:0003674 molecular_function	
phosphop	Subunit	GO:00159	GO:19901	GO:00046	(sce)Coenzyme A biosynthesis, pantothenate =
ribosoma	Protein	GO:00064	GO:00226	GO:00037	(sce03010)Ribosome
hypotheti	Putative	GO:00081	GO:00057	GO:0047429 nucleoside-triphosphate diphosphatase acti	
Ccw12p	Cell wal	GO:00007	GO:00059	GO:0005199 structural constituent of cell wall;GO:000	
Pex15p	Tail-anc	GO:00165	GO:00057	GO:0043495 protein membrane anchor;GO:0005515 protein	
mitochon	Mitochon	GO:00104	GO:00057	GO:0030414 peptidase inhibitor activity;GO:0061134 pe	
alkaline-	Cys2His2	GO:00109	GO:00056	GO:0001227 DNA-binding transcription repressor activi	
tRNA (ca	tRNA met	GO:00020	GO:00056	GO:0016300 tRNA (uracil) methyltransferase activity;G	
putative	MAPKK in	GO:00001	GO:00059	GO:00047	(sce04139)Mitophagy - yeast; (sce04011)MAPK
hypotheti	Putative protein of unknown function				

None	None	None	None	None	None
None	None	None	None	None	None
None	None	None	None	None	None
None	None	None	None	None	None
ribosome	Protein i	GO:19048	GO:00306	GO:19908	(sce03008)Ribosome biogenesis in eukaryotes
tRNA (cy	S-adenos	GO:00021	GO:00057	GO:0016428	tRNA (cytosine-5-)-methyltransferase activ
casein k	Alpha ca	GO:00075	GO:00344	GO:00046	(sce04139)Mitophagy - yeast; (sce03008)Ribos
Nkp2p	Central	GO:00513	GO:00315	GO:0003674	molecular_function
FG-nucle	FG-nucle	GO:00000	GO:00446	GO:0008139	nuclear localization sequence binding;GO:0
ribosoma	Ribosoma	GO:00021	GO:00226	GO:00701	(sce03010)Ribosome
phosphop	Catalyti	GO:20001	GO:00007	GO:00047	(sce04136)Autophagy - other; (sce03015)mRNA
hypothet	Putative	GO:00905	GO:00160	GO:0004521	endoribonuclease activity;GO:0004540 ribon
Sap4p	Protein i	GO:00000	GO:00057	GO:0004722	protein serine/threonine phosphatase activ
U5 snRNP	GTPase c	GO:00003	GO:00465	GO:00306	(sce03040)Spliceosome
Elongato	Subunit	GO:00029	GO:00335	GO:0004402	histone acetyltransferase activity;GO:0061
citrate	Dual spe	GO:00196	GO:00057	GO:00504	(M00010)Citrate cycle, first carbon oxidatio
hypothet	Protein	GO:00081	GO:00003	GO:0019825	oxygen binding;GO:0020037 heme binding;GO:
Efr3p	Protein i	GO:00726	GO:00057	GO:0003674	molecular_function
phospholi	Phosphol	GO:00007	GO:00056	GO:00702	(sce00565)Ether lipid metabolism; (sce00564)
Caf20p	Phosphop	GO:00106	GO:00058	GO:0003743	translation initiation factor activity;GO:
hypothet	Putative	GO:00081	GO:00055	GO:0003674	molecular_function
None	None	None	None	None	None
None	None	None	None	None	None
Acf2p	Intracel	GO:00300	GO:00099	GO:0052862	glucan endo-1,4-beta-glucanase activity, C
Pcf11p	mRNA 3'	GO:00987	GO:00058	GO:00009	(sce03015)mRNA surveillance pathway
ribosoma	Ribosoma	GO:00422	GO:00226	GO:00037	(sce03010)Ribosome
AAA fami	Essentia	GO:00319	GO:00056	GO:00036	(sce04111)Cell cycle - yeast; (sce04113)Meio
Rrn10p	Protein i	GO:00427	GO:00005	GO:0001165	RNA polymerase I upstream control element
None	None	None	None	None	None
dolichyl-	Protein (	GO:00717	GO:00975	GO:00041	(sce00515)Mannose type O-glycan biosynthesis
phosphat	Protein	GO:00007	GO:00059	GO:00161	(sce04011)MAPK signaling pathway - yeast
ERMES co	Outer mi	GO:00550	GO:00328	GO:00039	(Human)(hsa04137)Mitophagy - animal
Vta1p	Multives	GO:00324	GO:00057	GO:00016	(sce04144)Endocytosis
Flr1p	Plasma m	GO:00159	GO:00058	GO:0015244	fluconazole transmembrane transporter acti
putative	Putative	GO:00551	GO:00057	GO:00185	(sce00910)Nitrogen metabolism
hypothet	Putative	GO:00081	GO:00057	GO:0003674	molecular_function
hypothet	Putative	GO:00081	GO:00160	GO:0003674	molecular_function
putative	Phosphol	GO:00453	GO:00301	GO:0015662	ATPase activity, coupled to transmembrane
Ecm33p	GPI-anch	GO:00315	GO:00092	GO:0003674	molecular_function
None	None	None	None	None	None
mRNA spl	RNA bind	GO:00003	GO:00056	GO:00003	(sce03040)Spliceosome
Arr3p	Plasma m	GO:00156	GO:00058	GO:0015104	antimonite transmembrane transporter activ
TFIIH/NE	Subunit	GO:00708	GO:00056	GO:00083	(sce03022)Basal transcription factors; (sce0
Ecm8p	Non-esse	GO:00715	GO:00055	GO:0003674	molecular_function
Rab gera	Beta sub	GO:00183	GO:00059	GO:0004663	Rab geranylgeranyltransferase activity;GO:
FG-nucle	FG-nucle	GO:00319	GO:00446	GO:00081	(sce03013)RNA transport
None	None	None	None	None	None
Utp8p	Nucleola	GO:00064	GO:00344	GO:00000	(sce03008)Ribosome biogenesis in eukaryotes
Nbl1p	Subunit	GO:00070	GO:00321	GO:0030674	protein binding, bridging;GO:0060090 molec
Kri1p	Essentia	GO:00004	GO:00306	GO:0003674	molecular_function
ribosoma	Ribosoma	GO:00021	GO:00226	GO:00037	(sce03010)Ribosome
putative	Putative	GO:00322	GO:00056	GO:0008168	methyltransferase activity;GO:0016741 tran
seripaup	Protein	GO:00315	GO:00092	GO:0005199	structural constituent of cell wall;GO:000
Rho fami	GTP-bind	GO:19033	GO:00001	GO:00316	(sce04144)Endocytosis; (sce04011)MAPK signal

ATP-deper	Subunit (	GO:0007533	GO:00435	GO:00700	(sce03450)Non-homologous end-joining
translati	Translati	GO:00459	GO:00226	GO:0003746	translation elongation factor activity;GO:
Ist2p	Cortical	GO:00603	GO:00331	GO:0008289	lipid binding;GO:0005488 binding;GO:000367
putative	Putative	GO:00550	GO:00003	GO:00426	(Human)(hsa01523)Antifolate resistance; (hsa
NADH/NAD	ATP-NADH	GO:00067	GO:00056	GO:00427	(sce00760)Nicotinate and nicotinamide metabo
Nud1p	Componen	GO:00000	GO:00058	GO:00199	(sce04392)Hippo signaling pathway -multiple
hypotheti	Putative	GO:00081	GO:00057	GO:0003674	molecular_function
Trx3p	Mitochon	GO:00066	GO:00057	GO:00150	(Human)(hsa05418)Fluid shear stress and athe
Nam8p	RNA bindi	GO:00480	GO:00056	GO:0003729	mRNA binding;GO:0003723 RNA binding;GO:000
SAM compl	Componen	GO:00450	GO:00014	GO:0008320	protein transmembrane transporter activity
peptide a	Subunit (	GO:00616	GO:00314	GO:1990190	peptide-glutamate-N-acetyltransferase acti
FG-nucle	FG-nucle	GO:00319	GO:00446	GO:0017056	structural constituent of nuclear pore;GO:
Bub2p	Mitotic	GO:19025	GO:19903	GO:00171	(sce04111)Cell cycle - yeast
E2 ubiqu	Ubiquitin	GO:00109	GO:00313	GO:0061631	ubiquitin conjugating enzyme activity;GO:0
None	None	None	None	None	None
serine/tl	Conserve	GO:20011	GO:00335	GO:00047	(sce03008)Ribosome biogenesis in eukaryotes
DNA-dire	DNA polyn	GO:00709	GO:00358	GO:0003887	DNA-directed DNA polymerase activity;GO:00
Lia1p	Deoxyhypu	GO:00086	GO:00056	GO:0019135	deoxyhypusine monooxygenase activity;GO:00
hypotheti	Protein (	GO:00159	GO:00055	GO:0003674	molecular_function
histone r	Histone m	GO:00714	GO:00007	GO:00469	(sce00310)Lysine degradation
uridine p	High affi	GO:00158	GO:00003	GO:0015213	uridine transmembrane transporter activity
Mam1p	Monopolin	GO:00107	GO:00335	GO:00036	(sce04113)Meiosis - yeast
Gim5p	Subunit (	GO:00329	GO:00162	GO:0051082	unfolded protein binding;GO:0015631 tubuli
None	None	None	None	None	None
exosome (	Exosome (	GO:00710	GO:00001	GO:00001	(sce03018)RNA degradation
ribosome	Protein t	GO:00709	GO:19905	GO:00045	(sce03015)mRNA surveillance pathway
actin-rel	Essential	GO:00304	GO:00304	GO:00168	(sce04138)Autophagy - yeast
carbonyl	NADPH-dep	GO:00551	GO:00056	GO:0004090	carbonyl reductase (NADPH) activity;GO:001
Iba57p	Protein i	GO:01060	GO:00057	GO:0016740	transferase activity;GO:0003824 catalytic
tyrosine-	Cytoplasm	GO:00064	GO:00104	GO:00048	(sce00970)Aminoacyl-tRNA biosynthesis
hypotheti	Putative	GO:00081	GO:00055	GO:0003674	molecular_function
Nmd2p	Protein i	GO:00704	GO:00351	GO:00037	(sce03015)mRNA surveillance pathway; (sce030
septin SI	Sporulati	GO:00321	GO:00056	GO:0032947	protein-containing complex scaffold activi
Vel1p	Protein (	GO:00081	GO:00057	GO:0003674	molecular_function
putative	Putative	GO:00193	GO:00056	GO:00039	(sce00450)Selenocompound metabolism; (sce009
Pga2p	Essential	GO:00150	GO:00319	GO:0003674	molecular_function
Avo2p	Componen	GO:00309	GO:00057	GO:0003674	molecular_function
bifuncti	Bifuncti	GO:00194	GO:00058	GO:00716	(sce)Acylglycerol degradation; (M00098)Acylg
Aht1p	Dubious (	GO:00081	GO:00055	GO:0003674	molecular_function
transcri	Transcri	GO:00004	GO:00058	GO:00043	(sce)Nucleotide sugar biosynthesis, galactos
Rtt107p	Protein i	GO:19906	GO:00353	GO:0003674	molecular_function
serine/tl	Serine/tl	GO:19029	GO:00001	GO:00046	(sce04138)Autophagy - yeast
Pci8p	Possible	GO:00003	GO:00081	GO:0003674	molecular_function
frequenin	N-myrist	GO:00431	GO:00331	GO:0008047	enzyme activator activity;GO:0005509 calci
alpha/bet	Protein (	GO:00359	GO:00057	GO:0004623	phospholipase A2 activity;GO:0004620 phosp
TRAPP sul	Core comp	GO:00068	GO:19900	GO:0017112	Rab guanyl-nucleotide exchange factor acti
hypotheti	Protein (	GO:00081	GO:00058	GO:0003674	molecular_function
ATP-deper	RNA-deper	GO:00003	GO:00465	GO:00040	(sce03040)Spliceosome
DNA-dire	Catalyti	GO:00431	GO:00436	GO:00082	(sce03410)Base excision repair; (sce03440)Ho
Rcn1p	Protein i	GO:00708	GO:00057	GO:0008597	calcium-dependent protein serine/threonine
Ahk1p	Scaffold	GO:00072	GO:00055	GO:0005078	MAP-kinase scaffold activity;GO:0035591 si
Iml1p	GTPase-a	GO:20007	GO:00970	GO:0005096	GTPase activator activity;GO:0030695 GTPas
transpor	Integral	GO:00482	GO:00301	GO:0003674	molecular_function
hypotheti	Putative	GO:00158	GO:00160	GO:00085	(Human)(hsa00604)Glycosphingolipid biosynthe

Hif1p	Non-esser	GO:00634	GO:00001	GO:0042393 histone binding;GO:0005515 protein binding	
chaperoni	Subunit (	GO:00064	GO:00058	GO:0051082 unfolded protein binding;GO:0005524 ATP bi	
nucleopor	Transmemb	GO:00310	GO:00313	GO:0043022 ribosome binding;GO:0043021 ribonucleoprot	
Tim54p	Componen	GO:00450	GO:00427	GO:0008320 protein transmembrane transporter activity	
cystathio	Cystathio	GO:00193	GO:00056	GO:00445	(sce)Cysteine biosynthesis, homocysteine + s
Rec114p	Protein i	GO:00421	GO:00007	GO:0003674 molecular_function	
None	None	None	None	None	None
DNA-deper	DNA-deper	GO:00451	GO:00056	GO:00156	(sce03440)Homologous recombination
None	None	None	None	None	None
None	None	None	None	None	None
phosphol	Protein i	GO:00603	GO:00007	GO:0003674 molecular_function	
Lam1p	Putative	GO:00457	GO:00325	GO:0016538 cyclin-dependent protein serine/threonine	
hypotheti	Putative	GO:00081	GO:00055	GO:0003674 molecular_function	
None	None	None	None	None	None
None	None	None	None	None	None
None	None	None	None	None	None
None	None	None	None	None	None
monothiol	Glutathio	GO:00162	GO:00057	GO:0015038 glutathione disulfide oxidoreductase activ	
2',3'-cyc	Cyclic nu	GO:00091	GO:00301	GO:0004113 2',3'-cyclic-nucleotide 3'-phosphodiestera	
organic a	Secondary	GO:19049	GO:00003	GO:0015187 glycine transmembrane transporter activity	
hypotheti	Putative	GO:00081	GO:00055	GO:0003674 molecular_function	
None	None	None	None	None	None
ubiquitin	C3HC4-typ	GO:00165	GO:00057	GO:00080	(sce04146)Peroxisome
Tpo5p	Protein i	GO:19020	GO:00001	GO:0015203 polyamine transmembrane transporter activi	
Eaf3p	Componen	GO:00601	GO:0032221 Rpd3S complex;GO:0035267 NuA4 histone acetyltransfe		

)Propanoate metabolism; (sce00280)Valine, leucine and isoleucine degradation

olism; (sce00680)Methane metabolism; (sce01200)Carbon metabolism

0043022 ribosome binding;GO:0019843 rRNA binding
binding;GO:0030246 carbohydrate binding
binding;GO:0030246 carbohydrate binding
vity;GO:0051184 cofactor transmembrane transporter activity;GO:0022857 transmembrane tran

protein binding;GO:0005488 binding

52 alpha-glucoside:proton symporter activity;GO:0015574 trehalose transmembrane transport
ter activity;GO:0015343 siderophore transmembrane transporter activity;GO:0051184 cofacto

nitric oxide dioxygenase activity;GO:0016708 oxidoreductase activity, acting on paired d

protein binding;GO:0005488 binding

ctose-6P; (sce)Gluconeogenesis, oxaloacetate => fructose-6P; (sce00020)Citrate cycle (TCA

mide transmembrane transporter activity;GO:0015234 thiamine transmembrane transporter act

arch and sucrose metabolism; (sce01100)Metabolic pathways

0)Metabolic pathways
O:0016413 O-acetyltransferase activity;GO:0008374 O-acyltransferase activity
RNA polymerase II-specific;GO:0008270 zinc ion binding;GO:0046914 transition metal ion b
, nitrogenous group as acceptor;GO:0016651 oxidoreductase activity, acting on NAD(P)H;GO:
rsions; (sce01100)Metabolic pathways

ansmembrane transporter activity;GO:0022838 substrate-specific channel activity

0043022 ribosome binding;GO:0019843 rRNA binding
ity;GO:0046915 transition metal ion transmembrane transporter activity;GO:0046873 metal i

70)Cysteine and methionine metabolism; (sce01230)Biosynthesis of amino acids
ty acid degradation; (sce00010)Glycolysis / Gluconeogenesis

arbon lyase activity;GO:0016829 lyase activity
binding;GO:0033218 amide binding
iphosphatase activity;GO:0016462 pyrophosphatase activity

RNA polymerase II-specific;GO:0008270 zinc ion binding;GO:0046914 transition metal ion b
ylative mechanism;GO:0036442 proton-exporting ATPase activity;GO:0015662 ATPase activity,
0:0004672 protein kinase activity;GO:0016773 phosphotransferase activity, alcohol group a
tiple species
ity;GO:0005436 sodium:phosphate symporter activity;GO:0005315 inorganic phosphate transme

ty acid degradation; (sce00680)Methane metabolism

y;GO:0004322 ferroxidase activity;GO:0016724 oxidoreductase activity, oxidizing metal ion
abolic pathways
g;GO:0003723 RNA binding
050308 sugar-phosphatase activity;GO:0019203 carbohydrate phosphatase activity
phase, glucose 6P => ribulose 5P; (sce)Pentose phosphate pathway, oxidative phase, gluco

binding;GO:0030246 carbohydrate binding
GO:0015151 alpha-glucoside transmembrane transporter activity;GO:0042947 glucoside transm
specific channel activity;GO:0015267 channel activity

arch and sucrose metabolism; (sce01100)Metabolic pathways

=> GDP,GTP; (M00050)Guanine ribonucleotide biosynthesis IMP => GDP,GTP; (sce00230)Purine

reticulum
e01100)Metabolic pathways
;GO:0015101 organic cation transmembrane transporter activity;GO:0008519 ammonium transme

reticulum
GO:0000293 ferric-chelate reductase activity;GO:0016723 oxidoreductase activity, oxidizin

gocalciferol biosynthesis; (sce00100)Steroid biosynthesis
0004026 alcohol O-acetyltransferase activity;GO:0034318 alcohol O-acyltransferase activit
specific channel activity;GO:0015267 channel activity

processing in endoplasmic reticulum

5198 structural molecule activity;GO:0003674 molecular_function
se => UDP-glucose; (sce)Nucleotide sugar biosynthesis, glucose => UDP-glucose; (sce00051)
arch and sucrose metabolism; (sce01100)Metabolic pathways

;ests a role in lipid metabolism; expression pattern suggests a role in respiratory growth

ivity;GO:0015193 L-proline transmembrane transporter activity;GO:0015175 neutral amino ac

GO:0008289 lipid binding
tive phase, fructose 6P => ribose 5P; (sce)Pentose phosphate pathway, non-oxidative phase
movement of substances;GO:0043492 ATPase activity, coupled to movement of substances;GO:0
46 carbon-sulfur lyase activity;GO:0030170 pyridoxal phosphate binding
020037 heme binding;GO:0016705 oxidoreductase activity, acting on paired donors, with inc

transferase activity;GO:0008172 S-methyltransferase activity;GO:0008757 S-adenosylmethion
ansmembrane transporter activity;GO:0022838 substrate-specific channel activity

4 molecular_function
olic pathways

movement of substances;GO:0043492 ATPase activity, coupled to movement of substances;GO:0
ular adaptor activity;GO:0005515 protein binding

ansmembrane transporter activity;GO:0022838 substrate-specific channel activity
5198 structural molecule activity;GO:0003674 molecular_function
er activity;GO:0015343 siderophore transmembrane transporter activity;GO:0051184 cofactor

384 manganese ion transmembrane transporter activity;GO:0005315 inorganic phosphate trans
70)Cysteine and methionine metabolism; (sce01230)Biosynthesis of amino acids
specific channel activity;GO:0015267 channel activity

lanine, aspartate and glutamate metabolism; (sce01230)Biosynthesis of amino acids

binding;GO:0046906 tetrapyrrole binding
esis; (sce01100)Metabolic pathways
tamine (+ PRPP) => UMP; (M00051)Uridine monophosphate biosynthesis, glutamine (+ PRPP) =>
0034323 O-butanoyltransferase activity;GO:0004026 alcohol O-acetyltransferase activity

gocalciferol biosynthesis; (sce00100)Steroid biosynthesis
0032550 purine ribonucleoside binding
sporter activity;GO:0015196 L-tryptophan transmembrane transporter activity;GO:0015173 ar
ar_function
e activity;GO:0017111 nucleoside-triphosphatase activity

hiamine metabolism; (sce01100)Metabolic pathways
ree-carbon compounds; (sce)Glycolysis, core module involving three-carbon compounds; (M00
00)Metabolic pathways
s, PE => PC; (sce)Phosphatidylcholine (PC) biosynthesis, PE => PC; (sce00564)Glycerophosp
-specific DNA binding;GO:0001158 enhancer sequence-specific DNA binding;GO:0035326 enhanc
;GO:0016616 oxidoreductase activity, acting on the CH-OH group of donors, NAD or NADP as
ator activity;GO:0030695 GTPase regulator activity

lyoxylate and dicarboxylate metabolism; (sce04213)Longevity regulating pathway - multiple

pathway; (M00095)C5 isoprenoid biosynthesis, mevalonate pathway; (sce00900)Terpenoid back
ic activity;GO:0003674 molecular_function
0)Tyrosine metabolism; (sce00400)Phenylalanine, tyrosine and tryptophan biosynthesis
5198 structural molecule activity;GO:0003674 molecular_function

hionine salvage pathway; (sce00270)Cysteine and methionine metabolism
de binding;GO:0030554 adenyl nucleotide binding

ce-specific DNA binding;GO:0000987 proximal promoter sequence-specific DNA binding;GO:000
e bodies; (sce)C5 isoprenoid biosynthesis, mevalonate pathway; (sce00650)Butanoate metabo
ic activity;GO:0003674 molecular_function

vity;GO:0051184 cofactor transmembrane transporter activity;GO:0022857 transmembrane tran
proteins localize to the cytosol; conserved among Saccharomyces sensu stricto species

activity;GO:0015171 amino acid transmembrane transporter activity;GO:0046943 carboxylic
peroxiredoxin activity;GO:0004601 peroxidase activity

ty acid degradation; (sce00010)Glycolysis / Gluconeogenesis
ecular transducer activity;GO:0003674 molecular_function
00)Metabolic pathways

0086)beta-Oxidation, acyl-CoA synthesis; (sce00061)Fatty acid biosynthesis

donors with incorporation of molecular oxygen, incorporation of two atoms of oxygen;GO:00

lism; (sce01100)Metabolic pathways

pathway; (M00095)C5 isoprenoid biosynthesis, mevalonate pathway; (sce00900)Terpenoid back
ng;GO:0003824 catalytic activity
osynthesis of antibiotics; (sce01110)Biosynthesis of secondary metabolites
factor binding;GO:0070491 repressing transcription factor binding;GO:0001085 RNA polymer
tiple species; (sce04144)Endocytosis; (sce03040)Spliceosome

olic pathways
ing;GO:0043178 alcohol binding
doplasmic reticulum; (sce00062)Fatty acid elongation; (sce01040)Biosynthesis of unsaturat
n, oxaloacetate => 2-oxoglutarate; (sce)Citrate cycle, first carbon oxidation, oxaloaceta
etabolism; (sce00520)Amino sugar and nucleotide sugar metabolism; (sce01130)Biosynthesis
activity;GO:0052689 carboxylic ester hydrolase activity
e cycle, second carbon oxidation, 2-oxoglutarate => oxaloacetate; (M00009)Citrate cycle (

pathway; (M00095)C5 isoprenoid biosynthesis, mevalonate pathway; (sce00900)Terpenoid back
5198 structural molecule activity;GO:0003674 molecular_function
00)Metabolic pathways
;GO:0043492 ATPase activity, coupled to movement of substances;GO:0005548 phospholipid tr
00)Metabolic pathways
binding;GO:0030246 carbohydrate binding
GO:0000293 ferric-chelate reductase activity;GO:0016723 oxidoreductase activity, oxidizin
Pyrimidine metabolism; (sce00230)Purine metabolism
00)Metabolic pathways
olic pathways

00)Metabolic pathways
y;GO:0015103 inorganic anion transmembrane transporter activity;GO:0008509 anion transmem
e regulator activity;GO:0060589 nucleoside-triphosphatase regulator activity
etabolic pathways
movement of substances;GO:0043492 ATPase activity, coupled to movement of substances;GO:0
5198 structural molecule activity;GO:0003674 molecular_function
ic activity;GO:0003674 molecular_function
etabolic pathways

etabolism
098847 sequence-specific single stranded DNA binding;GO:0042162 telomeric DNA binding

0005365 myo-inositol transmembrane transporter activity;GO:0015166 polyol transmembrane t
tiple species; (sce04138)Autophagy - yeast
;GO:0016616 oxidoreductase activity, acting on the CH-OH group of donors, NAD or NADP as
compounds;GO:0016798 hydrolase activity, acting on glycosyl bonds;GO:0016757 transferase

ing;GO:0043168 anion binding

ne/threonine kinase activity;GO:0004672 protein kinase activity
4029 aldehyde dehydrogenase (NAD) activity;GO:0016620 oxidoreductase activity, acting on
iosynthesis of secondary metabolites; (sce01100)Metabolic pathways
e bodies; (sce)C5 isoprenoid biosynthesis, mevalonate pathway; (sce00650)Butanoate metabo

ribosomal small subunit binding;GO:0030674 protein binding, bridging
Pyrimidine metabolism; (sce00230)Purine metabolism
ylative mechanism;GO:0008553 proton-exporting ATPase activity, phosphorylative mechanism;

Hpr5p; YGL101W has a paralog, YBR242W, that arose from the whole genome duplication

gocalciferol biosynthesis; (sce00100)Steroid biosynthesis

ree-carbon compounds; (sce)Glycolysis, core module involving three-carbon compounds; (M00
ding;GO:0044877 protein-containing complex binding;GO:0005488 binding

;GO:0032143 single thymine insertion binding;GO:0032138 single base insertion or deletion
ty;GO:0016413 O-acetyltransferase activity;GO:0008374 O-acyltransferase activity

NA-binding transcription factor activity;GO:0140110 transcription regulator activity
ator activity;GO:0030695 GTPase regulator activity

00)Metabolic pathways
vity;GO:0005313 L-glutamate transmembrane transporter activity;GO:0015172 acidic amino ac
prolyl cis-trans isomerase activity;GO:0043022 ribosome binding
ctivity;GO:0004713 protein tyrosine kinase activity;GO:0004674 protein serine/threonine k

activity, hydrolyzing O-glycosyl compounds;GO:0016798 hydrolase activity, acting on glyco

de binding;GO:0030554 adenyl nucleotide binding
de binding;GO:0030554 adenyl nucleotide binding
lism; (sce01100)Metabolic pathways
tive phase, fructose 6P => ribose 5P; (sce)Pentose phosphate pathway, non-oxidative phase
0019208 phosphatase regulator activity;GO:0031072 heat shock protein binding
;GO:0045290 D-arabinose 1-dehydrogenase [NAD(P)+] activity;GO:0016616 oxidoreductase acti
lecular_function

ne; (M00026)Histidine biosynthesis, PRPP => histidine; (sce00340)Histidine metabolism
osynthesis of antibiotics; (sce01100)Metabolic pathways
itor activity;GO:0004857 enzyme inhibitor activity;GO:0030234 enzyme regulator activity
etabolism
ecretion; (hsa04921)Oxytocin signaling pathway
hosphatase activity;GO:0016462 pyrophosphatase activity
iphosphatase activity;GO:0016462 pyrophosphatase activity
00)Metabolic pathways

00)Metabolic pathways

0:0004672 protein kinase activity;GO:0016773 phosphotransferase activity, alcohol group a

abolic pathways

doreductase activity, acting on NAD(P)H;GO:0016491 oxidoreductase activity

factor binding;GO:0019210 kinase inhibitor activity;GO:0033613 activating transcription
3676 nucleic acid binding

1269 alpha-keto ester reductase activity;GO:0004032 alditol:NADP+ 1-oxidoreductase activi

olic pathways

osynthesis of antibiotics; (sce01100)Metabolic pathways
5198 structural molecule activity;GO:0003674 molecular_function

etic complications; (sce04213)Longevity regulating pathway - multiple species; (sce04138)
acids; (sce01212)Fatty acid metabolism
e regulator activity;GO:0060589 nucleoside-triphosphatase regulator activity
0:0032559 adenyl ribonucleotide binding
ctivity;GO:0005280 amino acid:proton symporter activity;GO:0015489 putrescine transmembra

)Metabolic pathways

0:0004672 protein kinase activity;GO:0016773 phosphotransferase activity, alcohol group a
al-dependent;GO:0003993 acid phosphatase activity;GO:0004725 protein tyrosine phosphatase
e cycle, second carbon oxidation, 2-oxoglutarate => oxaloacetate; (M00009)Citrate cycle (

sm; (sce01110)Biosynthesis of secondary metabolites; (sce01100)Metabolic pathways
er activity;GO:0005385 zinc ion transmembrane transporter activity;GO:0072509 divalent in
016620 oxidoreductase activity, acting on the aldehyde or oxo group of donors, NAD or NAD

yruvate metabolism; (sce04011)MAPK signaling pathway - yeast
iosynthesis; (sce00100)Steroid biosynthesis; (sce01130)Biosynthesis of antibiotics
ethane metabolism; (sce00620)Pyruvate metabolism
0:0004672 protein kinase activity;GO:0016773 phosphotransferase activity, alcohol group a
ce-specific DNA binding;GO:0000987 proximal promoter sequence-specific DNA binding;GO:000
70001 aspartic-type peptidase activity;GO:0043130 ubiquitin binding
iosynthesis; (sce00100)Steroid biosynthesis; (sce01130)Biosynthesis of antibiotics
gocalciferol biosynthesis; (sce00100)Steroid biosynthesis

recruiting activity;GO:0001134 transcription regulator recruiting activity;GO:0000978 RNA

roline; (sce00332)Carbapenem biosynthesis; (sce)Proline biosynthesis, glutamate => prolin
reticulum
0:0004672 protein kinase activity;GO:0016773 phosphotransferase activity, alcohol group a
pathway; (M00095)C5 isoprenoid biosynthesis, mevalonate pathway; (sce00900)Terpenoid back

rotein tag;GO:0005515 protein binding
ribosome binding;GO:0000049 tRNA binding
1 aminoacyl-tRNA editing activity;GO:0004812 aminoacyl-tRNA ligase activity

lasmic reticulum; (hsa05164)Influenza A
050308 sugar-phosphatase activity;GO:0019203 carbohydrate phosphatase activity
016620 oxidoreductase activity, acting on the aldehyde or oxo group of donors, NAD or NAD
ndoplasmic reticulum (ER) while mCherry fusion localizes to both the ER and vacuole
RNA polymerase II-specific;GO:0003700 DNA-binding transcription factor activity;GO:01401
e metabolism
0:0015038 glutathione disulfide oxidoreductase activity;GO:0015037 peptide disulfide oxid
5198 structural molecule activity;GO:0003674 molecular_function
01130)Biosynthesis of antibiotics; (sce01110)Biosynthesis of secondary metabolites

00)Metabolic pathways

reabsorption

activity;GO:0004864 protein phosphatase inhibitor activity;GO:0019212 phosphatase inhibit
00)Metabolic pathways
)Metabolic pathways
00)Metabolic pathways

6791 phosphatase activity;GO:0042578 phosphoric ester hydrolase activity

020037 heme binding;GO:0005506 iron ion binding
utathionylacetyl)amino) phenylarsenoxide (GSAO); YIL029C has a paralog, YPR071W, that aro
ree-carbon compounds; (sce)Glycolysis, core module involving three-carbon compounds; (M00
nding transcription factor recruiting activity;GO:0061629 RNA polymerase II sequence-spec

34318 alcohol O-acyltransferase activity;GO:0016413 O-acetyltransferase activity

0032550 purine ribonucleoside binding
ity;GO:0005315 inorganic phosphate transmembrane transporter activity;GO:0046915 transiti
e activity;GO:0036459 thiol-dependent ubiquitinyl hydrolase activity;GO:0004197 cysteine-

oxoglutarate => 2-aminoadipate => lysine; (sce)Lysine biosynthesis, AAA pathway, 2-oxoglu
ular adaptor activity;GO:0005515 protein binding

0032550 purine ribonucleoside binding
yase activity;GO:0016835 carbon-oxygen lyase activity
olism; (sce00260)Glycine, serine and threonine metabolism; (sce01130)Biosynthesis of anti
0:0016668 oxidoreductase activity, acting on a sulfur group of donors, NAD(P) as acceptor
factor binding;GO:0070491 repressing transcription factor binding;GO:0001085 RNA polymer
genase activity;GO:0043783 oxidoreductase activity, oxidizing metal ions with flavin as a

se activity;GO:0050839 cell adhesion molecule binding
34318 alcohol O-acyltransferase activity;GO:0047617 acyl-CoA hydrolase activity
se => UDP-glucose; (sce)Nucleotide sugar biosynthesis, glucose => UDP-glucose; (sce00051)
0:0004672 protein kinase activity;GO:0016773 phosphotransferase activity, alcohol group a
;GO:0005515 protein binding

;GO:0016616 oxidoreductase activity, acting on the CH-OH group of donors, NAD or NADP as
tiple species; (sce04138)Autophagy - yeast; (sce04011)MAPK signaling pathway - yeast
ty, RNA polymerase II-specific;GO:0000981 DNA-binding transcription factor activity, RNA
cle; (sce00630)Glyoxylate and dicarboxylate metabolism

factor binding;GO:0001102 RNA polymerase II activating transcription factor binding;GO:0
5295 solute:proton symporter activity;GO:0015205 nucleobase transmembrane transporter act
induced by osmotic stress, starvation and during stationary phase; protein abundance inc
orter activity;GO:0005385 zinc ion transmembrane transporter activity;GO:0072509 divalent

mide transmembrane transporter activity;GO:1901474 azole transmembrane transporter activi
NA-binding transcription factor activity;GO:0140110 transcription regulator activity
lism; (sce04213)Longevity regulating pathway - multiple species; (sce01100)Metabolic path
0005548 phospholipid transporter activity;GO:0005319 lipid transporter activity
ity;GO:0008251 tRNA-specific adenosine deaminase activity;GO:0004000 adenosine deaminase

0:0004672 protein kinase activity;GO:0016773 phosphotransferase activity, alcohol group a

se => UDP-glucose; (sce)Nucleotide sugar biosynthesis, glucose => UDP-glucose; (sce00052)
sm; (sce00190)Oxidative phosphorylation; (sce01110)Biosynthesis of secondary metabolites
etabolism; (sce01100)Metabolic pathways
16653 oxidoreductase activity, acting on NAD(P)H, heme protein as acceptor;GO:0010181 FMN
e activity;GO:0036459 thiol-dependent ubiquitinyl hydrolase activity;GO:0019783 ubiquitin

ng;GO:0048037 cofactor binding
aminopeptidase activity;GO:0008235 metalloexopeptidase activity

s; (hsa05034)Alcoholism; (hsa05203)Viral carcinogenesis

hionine salvage pathway; (sce00270)Cysteine and methionine metabolism

cular function regulator;GO:0003674 molecular_function
rotein dimerization activity;GO:0003700 DNA-binding transcription factor activity
iphosphatase activity;GO:0016462 pyrophosphatase activity

0019212 phosphatase inhibitor activity;GO:0019888 protein phosphatase regulator activity
sce00250)Alanine, aspartate and glutamate metabolism; (sce01100)Metabolic pathways

e; (M00074)N-glycan biosynthesis, high-mannose type; (sce00513)Various types of N-glycan

abolic pathways

70)Cysteine and methionine metabolism; (sce01230)Biosynthesis of amino acids

inding;GO:0070577 lysine-acetylated histone binding;GO:0001091 RNA polymerase II basal tr
ree-carbon compounds; (sce)Glycolysis, core module involving three-carbon compounds; (M00

13)Meiosis - yeast
reticulum
reticulum

0)beta-Alanine metabolism; (sce00340)Histidine metabolism
se activity;GO:0042910 xenobiotic transmembrane transporter activity;GO:0042626 ATPase ac
g;GO:0051787 misfolded protein binding;GO:0031072 heat shock protein binding
otein binding;GO:0051082 unfolded protein binding

ree-carbon compounds; (sce)Glycolysis, core module involving three-carbon compounds; (M00
ty, RNA polymerase II-specific;GO:0000981 DNA-binding transcription factor activity, RNA
y;GO:0008324 cation transmembrane transporter activity;GO:0015075 ion transmembrane trans
e01110)Biosynthesis of secondary metabolites; (sce01100)Metabolic pathways
0098518 polynucleotide phosphatase activity;GO:0016791 phosphatase activity
tivity;GO:0019707 protein-cysteine S-acyltransferase activity;GO:0016417 S-acyltransferas
ic compound binding;GO:0097159 organic cyclic compound binding

ce04011)MAPK signaling pathway - yeast; (sce01110)Biosynthesis of secondary metabolites
bolism

a04151)PI3K-Akt signaling pathway
n binding;GO:0005488 binding

arch and sucrose metabolism; (sce01100)Metabolic pathways

00)Metabolic pathways
e protein binding;GO:0005515 protein binding
etallopeptidase activity;GO:0004175 endopeptidase activity
gocalciferol biosynthesis; (sce00100)Steroid biosynthesis

vity;GO:0015038 glutathione disulfide oxidoreductase activity;GO:0016672 oxidoreductase a
hosphate cycle); (sce)Pentose phosphate pathway (Pentose phosphate cycle); (M00001)Glycol
ity;GO:0015926 glucosidase activity;GO:0004553 hydrolase activity, hydrolyzing O-glycosyl

serine; (sce)Serine biosynthesis, glycerate-3P => serine; (sce00680)Methane metabolism
binding;GO:0043021 ribonucleoprotein complex binding
tiple species; (sce04144)Endocytosis; (sce03040)Spliceosome

movement of substances;GO:0043492 ATPase activity, coupled to movement of substances;GO:0

ion binding;GO:0008270 zinc ion binding

00)Metabolic pathways
0032550 purine ribonucleoside binding
etabolism; (sce01130)Biosynthesis of antibiotics; (sce01100)Metabolic pathways
olic pathways

O:0004672 protein kinase activity;GO:0016773 phosphotransferase activity, alcohol group a

NA binding;GO:0046872 metal ion binding

nding;GO:0005515 protein binding

y;GO:0042124 1,3-beta-glucanosyltransferase activity;GO:0042123 glucanosyltransferase act
ginine biosynthesis; (sce01100)Metabolic pathways
00)Metabolic pathways

3676 nucleic acid binding

GO:0043139 5'-3' DNA helicase activity;GO:0004003 ATP-dependent DNA helicase activity

(hsa04068)FoxO signaling pathway
reticulum
olic pathways
n, oxaloacetate => 2-oxoglutarate; (sce)Citrate cycle, first carbon oxidation, oxaloaceta

ine and methionine metabolism; (sce01200)Carbon metabolism

g;GO:1901363 heterocyclic compound binding
inine biosynthesis; (sce00630)Glyoxylate and dicarboxylate metabolism
ce-specific DNA binding;GO:0000987 proximal promoter sequence-specific DNA binding;GO:000
transmembrane transporter activity;GO:0005347 ATP transmembrane transporter activity

=> GDP,GTP; (sce)Adenine ribonucleotide biosynthesis, IMP => ADP,ATP; (M00002)Glycolysis
00)Metabolic pathways
16653 oxidoreductase activity, acting on NAD(P)H, heme protein as acceptor;GO:0010181 FMN

ree-carbon compounds; (sce)Glycolysis, core module involving three-carbon compounds; (M00

GO:0008525 phosphatidylcholine transporter activity;GO:0005548 phospholipid transporter a

Pyrimidine metabolism; (sce00230)Purine metabolism
)Metabolic pathways
tiple species; (sce04146)Peroxisome
0004869 cysteine-type endopeptidase inhibitor activity;GO:0004843 thiol-dependent ubiquit

itor activity;GO:0072542 protein phosphatase activator activity;GO:0019211 phosphatase ac
)Pantothenate and CoA biosynthesis; (sce01110)Biosynthesis of secondary metabolites

doreductase activity, acting on NAD(P)H;GO:0016491 oxidoreductase activity
tein binding;GO:0005515 protein binding
ite permease (Dal5p) subfamily of the major facilitator superfamily; mRNA expression is el

6983 protein dimerization activity;GO:0003677 DNA binding

)Arginine and proline metabolism

y;GO:0015103 inorganic anion transmembrane transporter activity;GO:0008509 anion transmem

luctase active site

ular function regulator;GO:0003674 molecular_function

phosphatase regulator activity;GO:0008047 enzyme activator activity

)Valine, leucine and isoleucine degradation; (sce00340)Histidine metabolism
0480)Glutathione metabolism
lism; (sce04146)Peroxisome; (sce01100)Metabolic pathways

idylinositol binding;GO:0005543 phospholipid binding

0019787 ubiquitin-like protein transferase activity;GO:0140096 catalytic activity, acting

733 peptide-lysine-N-acetyltransferase activity;GO:0034212 peptide N-acetyltransferase ac

ce04111)Cell cycle - yeast; (sce04113)Meiosis - yeast
GO:0005261 cation channel activity;GO:0022839 ion gated channel activity

ty;GO:0000099 sulfur amino acid transmembrane transporter activity;GO:0015179 L-amino aci

ivity;GO:0051185 coenzyme transmembrane transporter activity;GO:0008028 monocarboxylic ac
osynthesis of antibiotics; (sce01100)Metabolic pathways

reticulum

g;GO:0043167 ion binding

phase, glucose 6P => ribulose 5P; (sce)Pentose phosphate pathway, oxidative phase, gluco

choline => PC; (sce00440)Phosphonate and phosphinate metabolism; (sce00565)Ether lipid me

RNA polymerase II-specific;GO:0043565 sequence-specific DNA binding;GO:0003700 DNA-bindi
sce00561)Glycerolipid metabolism; (sce00680)Methane metabolism
inson's disease; (hsa04022)cGMP-PKG signaling pathway
s; (hsa05034)Alcoholism; (hsa05202)Transcriptional misregulation in cancer
phase, glucose 6P => ribulose 5P; (sce)Pentose phosphate pathway, oxidative phase, gluco

ein complex binding;GO:0044877 protein-containing complex binding
O:0080025 phosphatidylinositol-3,5-bisphosphate binding;GO:0070273 phosphatidylinositol-4

; (sce)GABA (gamma-Aminobutyrate) shunt; (M00027)GABA (gamma-Aminobutyrate) shunt

:ted in highly purified mitochondria in high-throughput studies; deletion confers sensitiv

pathway; (M00095)C5 isoprenoid biosynthesis, mevalonate pathway; (sce00900)Terpenoid back

peroxiredoxin activity;GO:0004601 peroxidase activity
orter activity;GO:1903089 5-amino-1-ribofuranosylimidazole-4-carboxamide transmembrane tr

0016783 sulfurtransferase activity;GO:0016782 transferase activity, transferring sulfur-c
ator activity;GO:0030695 GTPase regulator activity

factor binding;GO:0070491 repressing transcription factor binding;GO:0001085 RNA polymer

00)Metabolic pathways

e protein binding;GO:0005085 guanyl-nucleotide exchange factor activity
ty;GO:0005198 structural molecule activity;GO:0005515 protein binding
protein binding;GO:0005488 binding

2-oxoadipate; (M00433)Lysine biosynthesis, 2-oxoglutarate => 2-oxoadipate; (M00030)Lysine

00)Metabolic pathways
reticulum
;GO:0016616 oxidoreductase activity, acting on the CH-OH group of donors, NAD or NADP as

n; (sce)Valine/isoleucine biosynthesis, pyruvate => valine / 2-oxobutanoate => isoleucine

00)Metabolic pathways
3420)Nucleotide excision repair

g;GO:1901363 heterocyclic compound binding
s; (hsa05034)Alcoholism; (hsa05203)Viral carcinogenesis

ne metabolism; (sce00230)Purine metabolism

hiamine metabolism; (sce01100)Metabolic pathways
transferase activity;GO:0008172 S-methyltransferase activity;GO:0008168 methyltransferase
30)Glyoxylate and dicarboxylate metabolism; (sce00260)Glycine, serine and threonine metab
reticulum

92 endoribonuclease activity, producing 3'-phosphomonoesters;GO:0004549 tRNA-specific rib

e01100)Metabolic pathways
reclamation
ty acid degradation; (sce00010)Glycolysis / Gluconeogenesis

198 structural molecule activity;GO:0003674 molecular_function
etabolism
sphorylation; (sce01100)Metabolic pathways
4 molecular_function
s, PE => PC; (sce)Phosphatidylcholine (PC) biosynthesis, PE => PC; (sce00564)Glycerophosp
ne metabolism; (sce00230)Purine metabolism
:0036374 glutathione hydrolase activity;GO:0008242 omega peptidase activity

vity;GO:0004866 endopeptidase inhibitor activity;GO:0061135 endopeptidase regulator activ

nthesis of antibiotics; (sce01100)Metabolic pathways
ter activity;GO:0015291 secondary active transmembrane transporter activity;GO:0022804 ac
)Metabolic pathways

yase activity;GO:0016835 carbon-oxygen lyase activity
7 cytoplasm

00)Metabolic pathways

RNA polymerase II-specific;GO:0008270 zinc ion binding;GO:0043565 sequence-specific DNA
3676 nucleic acid binding
e04070)Phosphatidylinositol signaling system; (sce01100)Metabolic pathways
11)Cell cycle - yeast; (sce04113)Meiosis - yeast

lanine, aspartate and glutamate metabolism; (sce01230)Biosynthesis of amino acids

e => valine / 2-oxobutanoate => isoleucine; (M00019)Valine/isoleucine biosynthesis, pyruv
movement of substances;GO:0043492 ATPase activity, coupled to movement of substances;GO:0
4-hydroxybenzoate => ubiquinone; (sce)Ubiquinone biosynthesis, eukaryotes, 4-hydroxybenz

hionine salvage pathway; (sce00270)Cysteine and methionine metabolism
tiple species; (sce04144)Endocytosis; (sce03040)Spliceosome
e regulator activity;GO:0098772 molecular function regulator
osphatase activity;GO:0043812 phosphatidylinositol-4-phosphate phosphatase activity;GO:01

etabolism
tein localizes to the vacuole; expression directly regulated by the metabolic and meiotic
198 structural molecule activity;GO:0003674 molecular_function
y;GO:0016278 lysine N-methyltransferase activity;GO:0008276 protein methyltransferase act
ce-specific DNA binding;GO:0000987 proximal promoter sequence-specific DNA binding;GO:000
n binding;GO:0005488 binding
RNA polymerase II-specific;GO:0008270 zinc ion binding;GO:0043565 sequence-specific DNA

004674 protein serine/threonine kinase activity;GO:0004672 protein kinase activity
glycan precursor trimming; (sce)N-glycan biosynthesis, high-mannose type

se => UDP-glucose; (sce)Nucleotide sugar biosynthesis, glucose => UDP-glucose; (sce00051)

ial promoter sequence-specific DNA binding;GO:0003899 DNA-directed 5'-3' RNA polymerase a
tiple species; (sce04138)Autophagy - yeast; (sce04113)Meiosis - yeast

sce00052)Galactose metabolism; (M00001)Glycolysis (Embden-Meyerhof pathway), glucose => p
tamine (+ PRPP) => UMP; (M00051)Uridine monophosphate biosynthesis, glutamine (+ PRPP) =>
abolic pathways

04 peptidase activator activity;GO:0061134 peptidase regulator activity

)Valine, leucine and isoleucine degradation; (sce00340)Histidine metabolism

roline; (sce00332)Carbapenem biosynthesis; (sce)Proline biosynthesis, glutamate => prolin
binding;GO:0003676 nucleic acid binding
e regulator activity;GO:0098772 molecular function regulator

> THF; (sce)Tetrahydrofolate biosynthesis, GTP => THF; (sce00790)Folate biosynthesis

e-specific double-stranded DNA binding;GO:0003690 double-stranded DNA binding
homoserine => threonine; (sce)Threonine biosynthesis, aspartate => homoserine => threoni
ylative mechanism;GO:0008553 proton-exporting ATPase activity, phosphorylative mechanism;

27)GABA (gamma-Aminobutyrate) shunt; (sce00650)Butanoate metabolism
e regulator activity;GO:0098772 molecular function regulator
etabolism; (sce01110)Biosynthesis of secondary metabolites; (sce01100)Metabolic pathways

ty;GO:0015175 neutral amino acid transmembrane transporter activity;GO:0015179 L-amino ac
signaling pathway - yeast
0032550 purine ribonucleoside binding
:0004090 carbonyl reductase (NADPH) activity;GO:0008106 alcohol dehydrogenase (NADP+) act
olism; (sce00260)Glycine, serine and threonine metabolism; (sce01200)Carbon metabolism
O:1901981 phosphatidylinositol phosphate binding;GO:0035091 phosphatidylinositol binding
hionine salvage pathway; (sce00410)beta-Alanine metabolism

00)Metabolic pathways

ing;GO:0046625 sphingolipid binding;GO:1902936 phosphatidylinositol bisphosphate binding
bon metabolism

8186 RNA-dependent ATPase activity;GO:0003724 RNA helicase activity

is (ALS); (hsa04370)VEGF signaling pathway; (hsa04924)Renin secretion
0015215 nucleotide transmembrane transporter activity;GO:0051184 cofactor transmembrane t

rsions; (sce01100)Metabolic pathways
ptidase regulator activity;GO:0004857 enzyme inhibitor activity

sulfur cluster binding;GO:0051539 4 iron, 4 sulfur cluster binding
te + erythrose-4P => chorismate; (sce)Shikimate pathway, phosphoenolpyruvate + erythrose-

ivity;GO:0016436 rRNA (uridine) methyltransferase activity;GO:0016435 rRNA (guanine) meth
O:0015038 glutathione disulfide oxidoreductase activity;GO:0015037 peptide disulfide oxid

ity;GO:0000297 spermine transmembrane transporter activity;GO:0015203 polyamine transmemb
e04070)Phosphatidylinositol signaling system; (sce01100)Metabolic pathways
-anchor biosynthesis; (sce01100)Metabolic pathways
ce-specific DNA binding;GO:0000987 proximal promoter sequence-specific DNA binding;GO:000

5)N-glycan precursor biosynthesis; (sce00510)N-Glycan biosynthesis
reticulum

; (sce01100)Metabolic pathways

0)beta-Alanine metabolism; (sce00340)Histidine metabolism

0019787 ubiquitin-like protein transferase activity;GO:0140096 catalytic activity, acting

fic DNA binding;GO:0046914 transition metal ion binding

hagy - yeast; (sce04138)Autophagy - yeast

75 superoxide-generating NADPH oxidase activity;GO:0016723 oxidoreductase activity, oxidi

n, oxaloacetate => 2-oxoglutarate; (sce)Citrate cycle, first carbon oxidation, oxaloaceta

)Longevity regulating pathway - multiple species; (hsa04920)Adipocytokine signaling pathwa
5198 structural molecule activity;GO:0003674 molecular_function
198 structural molecule activity;GO:0003674 molecular_function
filament binding;GO:0003779 actin binding
2 serine-type endopeptidase activity;GO:0003697 single-stranded DNA binding
binding;GO:0005488 binding
rsions; (sce01100)Metabolic pathways

NA secondary structure binding;GO:0003697 single-stranded DNA binding

6791 phosphatase activity;GO:0042578 phosphoric ester hydrolase activity

70001 aspartic-type peptidase activity;GO:0004175 endopeptidase activity
etabolic pathways
e01110)Biosynthesis of secondary metabolites; (sce01100)Metabolic pathways

5198 structural molecule activity;GO:0003674 molecular_function

is

0005365 myo-inositol transmembrane transporter activity;GO:0015166 polyol transmembrane t

00)Metabolic pathways

emase and epimerase activity, acting on carbohydrates and derivatives;GO:0016854 racemase

5198 structural molecule activity;GO:0003674 molecular_function
16491 oxidoreductase activity;GO:0003824 catalytic activity
olism; (sce00680)Methane metabolism; (sce00250)Alanine, aspartate and glutamate metabolis
674 molecular_function
ne; (M00026)Histidine biosynthesis, PRPP => histidine; (sce00360)Phenylalanine metabolism
;GO:1904680 peptide transmembrane transporter activity;GO:0022884 macromolecule transmemb
n, oxaloacetate => 2-oxoglutarate; (sce)Citrate cycle, first carbon oxidation, oxaloaceta
pendent ATPase activity;GO:0042623 ATPase activity, coupled
9 ubiquitin-like protein ligase binding;GO:0019899 enzyme binding

tein localizes to the endoplasmic reticulum; YLR050C is not an essential gene
00)Metabolic pathways

GO:0043138 3’-5’ DNA helicase activity;GO:0004003 ATP-dependent DNA helicase activity

lycerol degradation; (sce00561)Glycerolipid metabolism
ding;GO:0008092 cytoskeletal protein binding

lism; (sce04213)Longevity regulating pathway - multiple species; (sce01100)Metabolic path
osynthesis of antibiotics; (sce01110)Biosynthesis of secondary metabolites

18)RNA degradation
ctose-6P; (sce)Gluconeogenesis, oxaloacetate => fructose-6P; (sce00051)Fructose and manno
236 serine-type peptidase activity;GO:0017171 serine hydrolase activity

ferase activity;GO:0008168 methyltransferase activity;GO:0016741 transferase activity, tr

nding;GO:0003676 nucleic acid binding
e;GO:0008641 ubiquitin-like modifier activating enzyme activity;GO:0016877 ligase activit

ein complex binding;GO:0044877 protein-containing complex binding
mRNA binding;GO:0008289 lipid binding
ity;GO:0000993 RNA polymerase II complex binding;GO:0043175 RNA polymerase core enzyme bi

cle; (sce)Citrate cycle, second carbon oxidation, 2-oxoglutarate => oxaloacetate
lanine, aspartate and glutamate metabolism; (sce01210)2-Oxocarboxylic acid metabolism

)Glycerophospholipid metabolism; (sce01110)Biosynthesis of secondary metabolites
ce-specific DNA binding;GO:0000987 proximal promoter sequence-specific DNA binding;GO:000

y;GO:0008028 monocarboxylic acid transmembrane transporter activity;GO:0046943 carboxylic
ty;GO:0005198 structural molecule activity;GO:0005515 protein binding
0000030 mannosyltransferase activity;GO:0016758 transferase activity, transferring hexosy
00)Metabolic pathways

ce04141)Protein processing in endoplasmic reticulum

opolyphosphatase activity;GO:0016462 pyrophosphatase activity
104 succinate dehydrogenase activity;GO:0016628 oxidoreductase activity, acting on the CH
2-oxoadipate; (M00433)Lysine biosynthesis, 2-oxoglutarate => 2-oxoadipate; (M00030)Lysine

;GO:0005488 binding
sed expression analysis, and genome-wide homology searching

compounds;GO:0016810 hydrolase activity, acting on carbon-nitrogen (but not peptide) bon

ity;GO:0016426 tRNA (adenine) methyltransferase activity;GO:0008175 tRNA methyltransferas

00)Metabolic pathways

ty, RNA polymerase II-specific;GO:0000981 DNA-binding transcription factor activity, RNA
ty, RNA polymerase II-specific;GO:0001012 RNA polymerase II regulatory region DNA binding
tivity;GO:0003824 catalytic activity
utLbeta complex binding;GO:0032135 DNA insertion or deletion binding
ctivity;GO:0004674 protein serine/threonine kinase activity;GO:0003729 mRNA binding

vity;GO:0004032 alditol:NADP+ 1-oxidoreductase activity;GO:0008106 alcohol dehydrogenase
)Glycerophospholipid metabolism; (sce01110)Biosynthesis of secondary metabolites

O:0043169 cation binding
00083)Fatty acid biosynthesis, elongation; (sce)Fatty acid biosynthesis, initiation
GO:0004145 diamine N-acetyltransferase activity;GO:0008080 N-acetyltransferase activity
de binding;GO:0032561 guanyl ribonucleotide binding

pathway; (M00095)C5 isoprenoid biosynthesis, mevalonate pathway; (sce00900)Terpenoid back
tivity;GO:0003674 molecular_function

tiple species; (sce04144)Endocytosis; (sce03040)Spliceosome
etic complications; (sce04011)MAPK signaling pathway - yeast

osine degradation; (sce00600)Sphingolipid metabolism

40)Pyrimidine metabolism

RNA polymerase II-specific;GO:0008270 zinc ion binding;GO:0043565 sequence-specific DNA

mRNA binding;GO:0003723 RNA binding

tein transporter activity;GO:0005215 transporter activity
tein transferase activity;GO:0019787 ubiquitin-like protein transferase activity

activity;GO:0004860 protein kinase inhibitor activity;GO:0019789 SUMO transferase activit

sce01040)Biosynthesis of unsaturated fatty acids; (sce00280)Valine, leucine and isoleucin

)Metabolic pathways

ific DNA binding;GO:0003677 DNA binding
tivity;GO:0003674 molecular_function
oside-triphosphatase regulator activity;GO:0030234 enzyme regulator activity
40)Spliceosome; (sce03013)RNA transport

GO:0017005 3'-tyrosyl-DNA phosphodiesterase activity;GO:0070259 tyrosyl-DNA phosphodieste

y;GO:0046915 transition metal ion transmembrane transporter activity;GO:0046873 metal ion

ity;GO:0004721 phosphoprotein phosphatase activity;GO:0016791 phosphatase activity
g;GO:1901363 heterocyclic compound binding
tivity;GO:0034228 ethanolamine transmembrane transporter activity;GO:0015226 carnitine tr
sm; (sce01110)Biosynthesis of secondary metabolites; (sce01100)Metabolic pathways

1630 ubiquitin protein ligase activity;GO:0061659 ubiquitin-like protein ligase activity

03674 molecular_function

hagy - yeast

rol transmembrane transporter activity;GO:0015166 polyol transmembrane transporter activi

215 transporter activity;GO:0003674 molecular_function

se activity;GO:0042171 lysophosphatidic acid acyltransferase activity;GO:0016411 acylglyc

yltransferase activity;GO:0003880 protein C-terminal carboxyl O-methyltransferase activit

yl cis-trans isomerase activity;GO:0016859 cis-trans isomerase activity
cle; (sce)Citrate cycle, second carbon oxidation, 2-oxoglutarate => oxaloacetate
sce01130)Biosynthesis of antibiotics; (sce01110)Biosynthesis of secondary metabolites
compounds;GO:0016798 hydrolase activity, acting on glycosyl bonds;GO:0016757 transferase
e01110)Biosynthesis of secondary metabolites; (sce01100)Metabolic pathways
osynthesis of antibiotics; (sce01110)Biosynthesis of secondary metabolites

tamine (+ PRPP) => UMP; (M00051)Uridine monophosphate biosynthesis, glutamine (+ PRPP) =>
198 structural molecule activity;GO:0003674 molecular_function
tryptophan; (M00023)Tryptophan biosynthesis, chorismate => tryptophan; (sce00400)Phenylal
kinase activity;GO:0097472 cyclin-dependent protein kinase activity;GO:0004674 protein se
phosphatase activity;GO:0042578 phosphoric ester hydrolase activity
00)Carbon metabolism; (sce01100)Metabolic pathways
0003924 GTPase activity;GO:0005525 GTP binding
ty, RNA polymerase II-specific;GO:0000978 RNA polymerase II proximal promoter sequence-sp

ne metabolism; (sce00230)Purine metabolism

reticulum
ted in highly purified mitochondria in high-throughput studies
ctose-6P; (sce)Gluconeogenesis, oxaloacetate => fructose-6P; (sce00051)Fructose and manno

cetylase activity;GO:0016787 hydrolase activity

er activity;GO:0072349 modified amino acid transmembrane transporter activity;GO:1901682

protein transferase activity;GO:0051020 GTPase binding

9055 electron transfer activity;GO:0050662 coenzyme binding

4-hydroxybenzoate => ubiquinone; (sce)Ubiquinone biosynthesis, eukaryotes, 4-hydroxybenz
1100)Metabolic pathways
reticulum
se => UDP-glucose; (sce)Nucleotide sugar biosynthesis, glucose => UDP-glucose; (sce00052)

is (ALS); (hsa04370)VEGF signaling pathway; (hsa04924)Renin secretion

vity;GO:0005088 Ras guanyl-nucleotide exchange factor activity;GO:0017137 Rab GTPase bind
s; (hsa05034)Alcoholism; (hsa05203)Viral carcinogenesis
;GO:1904680 peptide transmembrane transporter activity;GO:0022884 macromolecule transmemb
specific);GO:0010485 H4 histone acetyltransferase activity;GO:0004402 histone acetyltran

otein deacetylase activity;GO:0019213 deacetylase activity

NA binding;GO:0046872 metal ion binding
712 transcription coregulator activity;GO:0003682 chromatin binding

ne metabolism; (sce00230)Purine metabolism
iosynthesis of secondary metabolites; (sce01100)Metabolic pathways
70001 aspartic-type peptidase activity;GO:0004175 endopeptidase activity
0005548 phospholipid transporter activity;GO:0005319 lipid transporter activity
015377 cation:chloride symporter activity;GO:0015296 anion:cation symporter activity
0:0004672 protein kinase activity;GO:0016773 phosphotransferase activity, alcohol group a

iphosphatase activity;GO:0016462 pyrophosphatase activity
120)Ubiquitin mediated proteolysis; (sce04141)Protein processing in endoplasmic reticulum
y;GO:0016278 lysine N-methyltransferase activity;GO:0008276 protein methyltransferase act
733 peptide-lysine-N-acetyltransferase activity;GO:0034212 peptide N-acetyltransferase ac
, nitrogenous group as acceptor;GO:0016651 oxidoreductase activity, acting on NAD(P)H;GO:

10 kinase inhibitor activity;GO:0019887 protein kinase regulator activity

0:0004672 protein kinase activity;GO:0016773 phosphotransferase activity, alcohol group a
hiamine metabolism; (sce01100)Metabolic pathways
ce04111)Cell cycle - yeast; (sce04113)Meiosis - yeast

Cysteine and methionine metabolism; (sce01100)Metabolic pathways

RNA polymerase III type 1 promoter DNA binding;GO:0080084 5S rDNA binding
ng phospholipas activity;GO:0070290 N-acylphosphatidylethanolamine-specific phospholipase
activity;GO:0140097 catalytic activity, acting on DNA
047372 acylglycerol lipase activity;GO:0004806 triglyceride lipase activity

y;GO:0000831 inositol hexakisphosphate 6-kinase activity;GO:0052723 inositol hexakisphosp
A replication; (sce03420)Nucleotide excision repair
Cysteine and methionine metabolism; (sce01100)Metabolic pathways
vity;GO:0036080 purine nucleotide-sugar transmembrane transporter activity;GO:0005338 nuc

6791 phosphatase activity;GO:0042578 phosphoric ester hydrolase activity

g;GO:1901363 heterocyclic compound binding

ce-specific DNA binding;GO:0000987 proximal promoter sequence-specific DNA binding;GO:000
0:0004672 protein kinase activity;GO:0016773 phosphotransferase activity, alcohol group a
vity;GO:0015299 solute:proton antiporter activity;GO:0015298 solute:cation antiporter act
ornithine; (sce)Ornithine biosynthesis, glutamate => ornithine; (sce00220)Arginine biosy
(sce03013)RNA transport

n binding;GO:0005488 binding
athrin binding;GO:0005516 calmodulin binding

etabolic pathways

ylation-dependent protein binding;GO:0140030 modification-dependent protein binding

recruiting activity;GO:0001134 transcription regulator recruiting activity;GO:0001103 RNA
binding, bridging;GO:0060090 molecular adaptor activity

reticulum

thesis, PA => PS => PE; (sce)Phosphatidylethanolamine (PE) biosynthesis, PA => PS => PE;
e-specific DNA binding;GO:0001010 RNA polymerase II sequence-specific DNA-binding transcr

reticulum

te + erythrose-4P => chorismate; (sce)Shikimate pathway, phosphoenolpyruvate + erythrose-
protein binding;GO:0005488 binding
ty;GO:0004866 endopeptidase inhibitor activity;GO:0061135 endopeptidase regulator activit

sulfur cluster binding;GO:0051539 4 iron, 4 sulfur cluster binding
ty, RNA polymerase II-specific;GO:0000981 DNA-binding transcription factor activity, RNA

dase activity, acting on L-amino acid peptides;GO:0008233 peptidase activity

2-oxoadipate; (M00433)Lysine biosynthesis, 2-oxoglutarate => 2-oxoadipate; (M00030)Lysine

bonuclease activity, producing 3'-phosphomonoesters;GO:0016894 endonuclease activity, act
0004661 protein geranylgeranyltransferase activity;GO:0008318 protein prenyltransferase a
ity;GO:0016423 tRNA (guanine) methyltransferase activity;GO:0008175 tRNA methyltransferas
198 structural molecule activity;GO:0003674 molecular_function
ivity;GO:0043865 methionine transmembrane transporter activity;GO:0000099 sulfur amino ac

ds;GO:0016787 hydrolase activity;GO:0003824 catalytic activity
3676 nucleic acid binding
NA binding;GO:0046872 metal ion binding
RNA polymerase II-specific;GO:0043565 sequence-specific DNA binding;GO:0003700 DNA-bindi

ce-specific DNA binding;GO:0000987 proximal promoter sequence-specific DNA binding;GO:000

hionine salvage pathway; (sce00330)Arginine and proline metabolism
3676 nucleic acid binding
etallopeptidase activity;GO:0004175 endopeptidase activity
;GO:1904680 peptide transmembrane transporter activity;GO:0022884 macromolecule transmemb
)Glyoxylate and dicarboxylate metabolism; (sce00260)Glycine, serine and threonine metabol
tryptophan; (M00023)Tryptophan biosynthesis, chorismate => tryptophan; (sce00400)Phenylal
25 anchored component of membrane
16891 endoribonuclease activity, producing 5'-phosphomonoesters;GO:0016893 endonuclease a
ty;GO:0015086 cadmium ion transmembrane transporter activity;GO:0005310 dicarboxylic acid

3676 nucleic acid binding
etic complications; (sce04139)Mitophagy - yeast; (sce04011)MAPK signaling pathway - yeast

ein complex binding;GO:0044877 protein-containing complex binding
aminopeptidase activity;GO:0008235 metalloexopeptidase activity
y;GO:0033744 L-methionine:thioredoxin-disulfide S-oxidoreductase activity;GO:0008113 pept

712 transcription coregulator activity;GO:0140110 transcription regulator activity

ding;GO:0008134 transcription factor binding;GO:0000981 DNA-binding transcription factor
de binding;GO:0032561 guanyl ribonucleotide binding
ivity;GO:0005384 manganese ion transmembrane transporter activity;GO:0005381 iron ion tra

9 4 iron, 4 sulfur cluster binding;GO:0051536 iron-sulfur cluster binding
g;GO:1901363 heterocyclic compound binding
ic compound binding;GO:0097159 organic cyclic compound binding
alpha kinase activity;GO:0045182 translation regulator activity;GO:0004674 protein serine
g;GO:0005488 binding
me kinetochore;GO:0000780 condensed nuclear chromosome, centromeric region
binding;GO:0003682 chromatin binding

e activity;GO:0036459 thiol-dependent ubiquitinyl hydrolase activity;GO:0004197 cysteine-
ted in highly purified mitochondria in high-throughput studies

ator activity;GO:0030695 GTPase regulator activity
8 single base insertion or deletion binding;GO:0032137 guanine/thymine mispair binding

vity;GO:0008174 mRNA methyltransferase activity;GO:0008173 RNA methyltransferase activity
olic pathways

ein complex binding;GO:0044877 protein-containing complex binding
0138 ubiquitin-like protein-specific isopeptidase activity;GO:0016929 SUMO-specific prote
e regulator activity;GO:0060589 nucleoside-triphosphatase regulator activity
3420)Nucleotide excision repair
ament binding;GO:0003779 actin binding
ctivity;GO:0034431 bis(5'-adenosyl)-hexaphosphatase activity;GO:0052840 inositol diphosph

protein (GFP)-fusion protein localizes to the cytoplasm

ein complex binding;GO:0044877 protein-containing complex binding
cription coregulator activity;GO:0008134 transcription factor binding

fic);GO:0032452 histone demethylase activity;GO:0035064 methylated histone binding
phase, glucose 6P => ribulose 5P; (sce)Pentose phosphate pathway, oxidative phase, gluco

ty;GO:0003924 GTPase activity;GO:0005525 GTP binding
onstituent of nuclear pore;GO:0005096 GTPase activator activity

yosin II binding;GO:0032036 myosin heavy chain binding

0:1901981 phosphatidylinositol phosphate binding;GO:0035091 phosphatidylinositol binding
tive transmembrane transporter activity;GO:0015322 secondary active oligopeptide transmem
> CoA; (M00120)Coenzyme A biosynthesis, pantothenate => CoA; (sce00770)Pantothenate and C
sm; (sce01110)Biosynthesis of secondary metabolites; (sce01100)Metabolic pathways
5198 structural molecule activity;GO:0003674 molecular_function

ce04111)Cell cycle - yeast; (sce04113)Meiosis - yeast
0:0005515 protein binding
reticulum

=> GDP,GTP; (M00050)Guanine ribonucleotide biosynthesis IMP => GDP,GTP; (sce00230)Purine
sis - yeast
otein binding;GO:0005515 protein binding
09982 pseudouridine synthase activity;GO:0016866 intramolecular transferase activity

y;GO:0000099 sulfur amino acid transmembrane transporter activity;GO:0015175 neutral amin
;GO:0017111 nucleoside-triphosphatase activity
GO:0016972 thiol oxidase activity;GO:0016670 oxidoreductase activity, acting on a sulfur
UMP => UDP/UTP,CDP/CTP; (M00052)Pyrimidine ribonucleotide biosynthesis, UMP => UDP/UTP,C

0:1901981 phosphatidylinositol phosphate binding;GO:0043130 ubiquitin binding
198 structural molecule activity;GO:0003674 molecular_function
ity;GO:0003682 chromatin binding
id transporter activity;GO:0005215 transporter activity

factor binding;GO:0033613 activating transcription factor binding;GO:0001085 RNA polymer

ein complex binding;GO:0044877 protein-containing complex binding
340 RNA 7-methylguanosine cap binding;GO:0000339 RNA cap binding
g;GO:0043167 ion binding

198 structural molecule activity;GO:0003723 RNA binding

single-stranded DNA binding;GO:1990837 sequence-specific double-stranded DNA binding
004721 phosphoprotein phosphatase activity;GO:0016791 phosphatase activity
136 sialic acid transmembrane transporter activity;GO:0015355 secondary active monocarbox

e04070)Phosphatidylinositol signaling system; (sce04136)Autophagy - other

s; (hsa05034)Alcoholism; (hsa05202)Transcriptional misregulation in cancer
3676 nucleic acid binding

recruiting activity;GO:0000979 RNA polymerase II core promoter sequence-specific DNA bind
ption
sm; (sce01110)Biosynthesis of secondary metabolites; (sce00970)Aminoacyl-tRNA biosynthesi
reticulum
de binding;GO:0032561 guanyl ribonucleotide binding
tivity;GO:0019707 protein-cysteine S-acyltransferase activity;GO:0016417 S-acyltransferas
nsferase; (sce)N-glycosylation by oligosaccharyltransferase; (sce00510)N-Glycan biosynthe
712 transcription coregulator activity;GO:0140110 transcription regulator activity
16616 oxidoreductase activity, acting on the CH-OH group of donors, NAD or NADP as accept
8186 RNA-dependent ATPase activity;GO:0003724 RNA helicase activity

MN reductase (NADPH) activity;GO:0003955 NAD(P)H dehydrogenase (quinone) activity
59 ubiquitin-like protein ligase activity;GO:0004842 ubiquitin-protein transferase activi

nslation repressor activity;GO:0045182 translation regulator activity
translation repressor activity, mRNA regulatory element binding;GO:0016896 exoribonucleas

g;GO:0051787 misfolded protein binding;GO:0031072 heat shock protein binding
sce00052)Galactose metabolism; (M00001)Glycolysis (Embden-Meyerhof pathway), glucose => p

ds;GO:0016787 hydrolase activity;GO:0003824 catalytic activity
ding;GO:0070006 metalloaminopeptidase activity;GO:0043023 ribosomal large subunit binding

3676 nucleic acid binding
74 ATP-dependent microtubule motor activity, plus-end-directed;GO:1990939 ATP-dependent m

mRNA binding;GO:0003723 RNA binding
n, oxaloacetate => 2-oxoglutarate; (sce)Citrate cycle, first carbon oxidation, oxaloaceta

thesis, PA => PS => PE; (sce)Phosphatidylethanolamine (PE) biosynthesis, PA => PS => PE;
itin-protein transferase activity;GO:0060090 molecular adaptor activity
ree-carbon compounds; (sce)Glycolysis, core module involving three-carbon compounds; (M00

acyltransferase activity;GO:0016407 acetyltransferase activity
ivity;GO:0140098 catalytic activity, acting on RNA;GO:0003723 RNA binding

ity;GO:0052690 trichloro-p-hydroquinone reductive dehalogenase activity;GO:0003959 NADPH
440)Homologous recombination
activity;GO:0015171 amino acid transmembrane transporter activity;GO:0046943 carboxylic

ane transporter activity;GO:0015142 tricarboxylic acid transmembrane transporter activity

-3 substituted reducing group;GO:0052861 glucan endo-1,3-beta-glucanase activity, C-3 sub

sce01130)Biosynthesis of antibiotics
712 transcription coregulator activity;GO:0140110 transcription regulator activity

2-oxoglutarate => oxaloacetate; (M00009)Citrate cycle (TCA cycle, Krebs cycle); (M00011)

tivity;GO:0055106 ubiquitin-protein transferase regulator activity;GO:0004857 enzyme inhi
cular function regulator;GO:0003674 molecular_function

ansport; (sce04138)Autophagy - yeast
ein complex binding;GO:0044877 protein-containing complex binding

e regulator activity;GO:0008092 cytoskeletal protein binding
rter activity;GO:0005315 inorganic phosphate transmembrane transporter activity;GO:001560
09 kinase activator activity;GO:0019887 protein kinase regulator activity

198 structural molecule activity;GO:0003723 RNA binding
linositol binding;GO:0015248 sterol transporter activity

recruiting activity;GO:0001134 transcription regulator recruiting activity;GO:0003712 tra
g;GO:0005488 binding

sis - yeast

protein binding;GO:0005488 binding

70001 aspartic-type peptidase activity;GO:0004175 endopeptidase activity
hionine salvage pathway; (sce00270)Cysteine and methionine metabolism
488 binding;GO:0003674 molecular_function
ne metabolism; (sce00230)Purine metabolism

9 ubiquitin-like protein ligase binding;GO:0019899 enzyme binding

RNA polymerase II-specific;GO:0043565 sequence-specific DNA binding;GO:0003700 DNA-bindi

75 RNA polymerase core enzyme binding;GO:0001099 basal RNA polymerase II transcription ma
9 ubiquitin-like protein ligase binding;GO:0019899 enzyme binding
ity;GO:0010340 carboxyl-O-methyltransferase activity;GO:0008171 O-methyltransferase activ

0008135 translation factor activity, RNA binding;GO:0003924 GTPase activity

ivity;GO:0016436 rRNA (uridine) methyltransferase activity;GO:0008649 rRNA methyltransfer
GO:0043138 3'-5' DNA helicase activity;GO:0004003 ATP-dependent DNA helicase activity
08483 transaminase activity;GO:0030170 pyridoxal phosphate binding
iosynthesis of amino acids; (sce01130)Biosynthesis of antibiotics

16891 endoribonuclease activity, producing 5'-phosphomonoesters;GO:0016893 endonuclease a
ycle, second carbon oxidation, 2-oxoglutarate => oxaloacetate; (M00009)Citrate cycle (TCA
reticulum
inding;GO:0001135 RNA polymerase II transcription regulator recruiting activity;GO:000109
tivity;GO:0015562 efflux transmembrane transporter activity;GO:0008519 ammonium transmemb
120)Ubiquitin mediated proteolysis
3682 chromatin binding;GO:0005198 structural molecule activity

reclamation; (hsa04976)Bile secretion; (hsa04972)Pancreatic secretion
003676 nucleic acid binding
92 endoribonuclease activity, producing 3'-phosphomonoesters;GO:0004549 tRNA-specific rib
s; (hsa05034)Alcoholism; (hsa05203)Viral carcinogenesis

lication; (sce03420)Nucleotide excision repair
sm; (sce00190)Oxidative phosphorylation; (sce01110)Biosynthesis of secondary metabolites

674 molecular_function
e01100)Metabolic pathways
0051185 coenzyme transmembrane transporter activity;GO:0015215 nucleotide transmembrane t

:0005092 GDP-dissociation inhibitor activity;GO:0005096 GTPase activator activity

idase activity;GO:0008237 metallopeptidase activity
0005347 ATP transmembrane transporter activity;GO:0000295 adenine nucleotide transmembran

transferase activity, transferring alkyl or aryl (other than methyl) groups;GO:0016740 t

0:0016833 oxo-acid-lyase activity;GO:0016830 carbon-carbon lyase activity
g;GO:0043167 ion binding
lism; (sce01100)Metabolic pathways
0005198 structural molecule activity;GO:0003674 molecular_function

aining complex scaffold activity;GO:0003682 chromatin binding
ein complex binding;GO:0044877 protein-containing complex binding

09 kinase activator activity;GO:0019887 protein kinase regulator activity
-deoxynucleotidase activity;GO:0008253 5'-nucleotidase activity
ce04111)Cell cycle - yeast
;GO:0004559 alpha-mannosidase activity;GO:0015923 mannosidase activity

UMP => UDP/UTP,CDP/CTP; (M00052)Pyrimidine ribonucleotide biosynthesis, UMP => UDP/UTP,C
ity;GO:0015194 L-serine transmembrane transporter activity;GO:0005302 L-tyrosine transmem
GO:0000293 ferric-chelate reductase activity;GO:0016723 oxidoreductase activity, oxidizin
l-K4 specific);GO:0034647 histone demethylase activity (H3-trimethyl-K4 specific);GO:0032
ng;GO:0008092 cytoskeletal protein binding
ty;GO:0022857 transmembrane transporter activity;GO:0005215 transporter activity
rane cleaving;GO:0004190 aspartic-type endopeptidase activity;GO:0070001 aspartic-type pe
;GO:0034212 peptide N-acetyltransferase activity;GO:0008080 N-acetyltransferase activity
(sce03013)RNA transport
tivity;GO:0008047 enzyme activator activity;GO:0030234 enzyme regulator activity
-specific DNA binding;GO:0001158 enhancer sequence-specific DNA binding;GO:0035326 enhanc

RNA polymerase II-specific;GO:0008270 zinc ion binding;GO:0043565 sequence-specific DNA
sm; (sce01110)Biosynthesis of secondary metabolites; (sce01100)Metabolic pathways

674 molecular_function

GO:0008525 phosphatidylcholine transporter activity;GO:0005548 phospholipid transporter a
0120013 intermembrane lipid transfer activity;GO:0015248 sterol transporter activity
e regulator activity;GO:0098772 molecular function regulator
folded protein binding;GO:0005215 transporter activity
0008135 translation factor activity, RNA binding;GO:0016887 ATPase activity

0016783 sulfurtransferase activity;GO:0004725 protein tyrosine phosphatase activity

ne metabolism; (sce00230)Purine metabolism
lism; (sce01100)Metabolic pathways
nding;GO:0032559 adenyl ribonucleotide binding

A-damaging agent MMS; the authentic, non-tagged protein is detected in highly purified mi

ce04141)Protein processing in endoplasmic reticulum
nding;GO:0001228 DNA-binding transcription activator activity, RNA polymerase II-specific
rsions; (sce00561)Glycerolipid metabolism; (sce00010)Glycolysis / Gluconeogenesis
fic);GO:0032454 histone demethylase activity (H3-K9 specific);GO:0032452 histone demethyl

ter activity;GO:0046943 carboxylic acid transmembrane transporter activity;GO:0015293 sym
(H3-K14 specific);GO:0031078 histone deacetylase activity (H3-K14 specific);GO:0017136 N
recruiting activity;GO:0001134 transcription regulator recruiting activity;GO:0008301 DNA
y; (hsa05231)Choline metabolism in cancer; (hsa04919)Thyroid hormone signaling pathway

tamine (+ PRPP) => UMP; (M00051)Uridine monophosphate biosynthesis, glutamine (+ PRPP) =>
80 acid-ammonia (or amide) ligase activity;GO:0016879 ligase activity, forming carbon-nit
tiple species; (sce04138)Autophagy - yeast; (sce04113)Meiosis - yeast
-acetylated histone binding;GO:0000182 rDNA binding
nding;GO:0032559 adenyl ribonucleotide binding
vity;GO:0005313 L-glutamate transmembrane transporter activity;GO:0015172 acidic amino ac
ic activity;GO:0003674 molecular_function

00141)C1-unit interconversion, eukaryotes; (sce00460)Cyanoamino acid metabolism

;GO:1901618 organic hydroxy compound transmembrane transporter activity;GO:0015238 drug t

19843 rRNA binding;GO:0003723 RNA binding
arboxypropyltransferase activity;GO:0008175 tRNA methyltransferase activity;GO:0008173 RN

ty;GO:0004866 endopeptidase inhibitor activity;GO:0061135 endopeptidase regulator activit

choline => PC; (sce00440)Phosphonate and phosphinate metabolism; (M00090)Phosphatidylchol
ity;GO:0046943 carboxylic acid transmembrane transporter activity;GO:0008514 organic anio

activity;GO:0003674 molecular_function
=> 2-oxoisocaproate; (M00432)Leucine biosynthesis, 2-oxoisovalerate => 2-oxoisocaproate;
n; (sce)Leucine biosynthesis, 2-oxoisovalerate => 2-oxoisocaproate; (M00432)Leucine biosy

0086)beta-Oxidation, acyl-CoA synthesis; (sce00061)Fatty acid biosynthesis

movement of ions, phosphorylative mechanism;GO:0042625 ATPase coupled ion transmembrane t

ter activity;GO:0046943 carboxylic acid transmembrane transporter activity;GO:0015238 dru
0)Tyrosine metabolism; (sce00220)Arginine biosynthesis
ity;GO:0016435 rRNA (guanine) methyltransferase activity;GO:0016423 tRNA (guanine) methyl
iosynthesis of secondary metabolites; (sce01100)Metabolic pathways
aminopeptidase activity;GO:0008235 metalloexopeptidase activity
sis - yeast
:0008106 alcohol dehydrogenase (NADP+) activity;GO:0004033 aldo-keto reductase (NADP) act
tiple species; (sce04146)Peroxisome
hionine salvage pathway; (sce00270)Cysteine and methionine metabolism

236 serine-type peptidase activity;GO:0017171 serine hydrolase activity
ity;GO:0046943 carboxylic acid transmembrane transporter activity;GO:0008514 organic anio

733 peptide-lysine-N-acetyltransferase activity;GO:0034212 peptide N-acetyltransferase ac
linositol binding;GO:0015248 sterol transporter activity
-K4 specific);GO:0032453 histone demethylase activity (H3-K4 specific);GO:0032452 histone

tivity;GO:0019707 protein-cysteine S-acyltransferase activity;GO:0031683 G-protein beta/g
1100)Metabolic pathways
GO:0000293 ferric-chelate reductase activity;GO:0016723 oxidoreductase activity, oxidizin
recruiting activity;GO:0001134 transcription regulator recruiting activity;GO:0000978 RNA

abolic pathways

e04070)Phosphatidylinositol signaling system; (sce04138)Autophagy - yeast
idylinositol binding;GO:0005543 phospholipid binding
ree-carbon compounds; (sce)Glycolysis, core module involving three-carbon compounds; (M00

ding, bending;GO:1990837 sequence-specific double-stranded DNA binding
tor activity;GO:0003779 actin binding

674 molecular_function
binding;GO:0031072 heat shock protein binding
sce01130)Biosynthesis of antibiotics
:0031369 translation initiation factor binding;GO:0003729 mRNA binding
orter activity;GO:0015215 nucleotide transmembrane transporter activity;GO:0015605 organo
:0031369 translation initiation factor binding;GO:0003729 mRNA binding

ase activity;GO:0004725 protein tyrosine phosphatase activity;GO:0004721 phosphoprotein p
ne metabolism; (hsa01100)Metabolic pathways
etabolism; (sce01110)Biosynthesis of secondary metabolites; (sce01100)Metabolic pathways
O:1901981 phosphatidylinositol phosphate binding;GO:0035091 phosphatidylinositol binding

sce01130)Biosynthesis of antibiotics
1630 ubiquitin protein ligase activity;GO:0061659 ubiquitin-like protein ligase activity
l to contain an N-terminal transmembrane domain; osw5 null mutant spores exhibit increased
ity;GO:0016427 tRNA (cytosine) methyltransferase activity;GO:0008175 tRNA methyltransfera

6298 lipase activity;GO:0016788 hydrolase activity, acting on ester bonds

ein complex binding;GO:0044877 protein-containing complex binding

ce-specific DNA binding;GO:0000987 proximal promoter sequence-specific DNA binding;GO:000
cle - yeast; (sce04113)Meiosis - yeast

0005548 phospholipid transporter activity;GO:0005319 lipid transporter activity
3676 nucleic acid binding
ity;GO:0071077 adenosine 3',5'-bisphosphate transmembrane transporter activity;GO:0000295
04730 pseudouridylate synthase activity;GO:0009982 pseudouridine synthase activity

binding;GO:0001025 RNA polymerase III transcription factor binding;GO:0000995 RNA polymer

dase activity, acting on L-amino acid peptides;GO:0008233 peptidase activity
species; (sce04111)Cell cycle - yeast
e protein binding;GO:0051082 unfolded protein binding
ty;GO:0015175 neutral amino acid transmembrane transporter activity;GO:0015179 L-amino ac

etabolic pathways

ribonucleoprotein complex binding;GO:0008270 zinc ion binding
198 structural molecule activity;GO:0003674 molecular_function
specific);GO:0010484 H3 histone acetyltransferase activity;GO:0004402 histone acetyltran
inine biosynthesis; (sce00250)Alanine, aspartate and glutamate metabolism
051500 D-tyrosyl-tRNA(Tyr) deacylase activity;GO:0051499 D-aminoacyl-tRNA deacylase activ
ein complex binding;GO:0044877 protein-containing complex binding

GO:0008649 rRNA methyltransferase activity;GO:0140102 catalytic activity, acting on a rRN

> THF; (sce)Tetrahydrofolate biosynthesis, GTP => THF; (sce00790)Folate biosynthesis
sce01130)Biosynthesis of antibiotics; (sce01110)Biosynthesis of secondary metabolites
doplasmic reticulum; (sce00062)Fatty acid elongation; (sce01040)Biosynthesis of unsaturat
nsferase activity, transferring amino-acyl groups;GO:0016746 transferase activity, transf

ular function regulator;GO:0003674 molecular_function
:0017150 tRNA dihydrouridine synthase activity;GO:0016627 oxidoreductase activity, acting

9 ubiquitin-like protein ligase binding;GO:0019899 enzyme binding

3676 nucleic acid binding

20)Sulfur metabolism; (sce00270)Cysteine and methionine metabolism
xidoreductase activity, acting on a heme group of donors, oxygen as acceptor;GO:0015002 h
hosphatase activity;GO:0016462 pyrophosphatase activity
lyoxylate and dicarboxylate metabolism; (sce01100)Metabolic pathways

tiple species; (sce04146)Peroxisome
binding;GO:0005488 binding
ity;GO:0015037 peptide disulfide oxidoreductase activity;GO:0015035 protein disulfide oxi
ansferase activity, transferring hexosyl groups;GO:0016757 transferase activity, transfer
GO:0008525 phosphatidylcholine transporter activity;GO:0005548 phospholipid transporter a
some
hosphatidylinositol-3,5-bisphosphate binding;GO:0032266 phosphatidylinositol-3-phosphate

e metabolism; (hsa01200)Carbon metabolism; (hsa01100)Metabolic pathways
reticulum

dine tract binding;GO:0003727 single-stranded RNA binding
nding;GO:0000976 transcription regulatory region sequence-specific DNA binding;GO:1990837
otein deacetylase activity;GO:0019213 deacetylase activity

e regulator activity;GO:0005085 guanyl-nucleotide exchange factor activity

g;GO:0001069 regulatory region RNA binding;GO:0003729 mRNA binding
tamine (+ PRPP) => UMP; (M00051)Uridine monophosphate biosynthesis, glutamine (+ PRPP) =>

ament binding;GO:0003779 actin binding

0019212 phosphatase inhibitor activity;GO:0019888 protein phosphatase regulator activity
3676 nucleic acid binding
transporter activity;GO:0015079 potassium ion transmembrane transporter activity;GO:00468

vity;GO:0005088 Ras guanyl-nucleotide exchange factor activity;GO:0017048 Rho GTPase bind
061711 N(6)-L-threonylcarbamoyladenine synthase activity;GO:0043047 single-stranded telom
ular adaptor activity;GO:0005515 protein binding
aining complex binding;GO:0005488 binding
vity;GO:0005384 manganese ion transmembrane transporter activity;GO:0015295 solute:proton

mologous recombination; (sce03430)Mismatch repair
signaling pathway - yeast
nding;GO:0000976 transcription regulatory region sequence-specific DNA binding;GO:1990837
tive phase, fructose 6P => ribose 5P; (sce)Pentose phosphate pathway, non-oxidative phase
erine => cysteine; (M00338)Cysteine biosynthesis, homocysteine + serine => cysteine; (sce

;GO:0051082 unfolded protein binding;GO:0016887 ATPase activity
hsa04142)Lysosome

ce-specific DNA binding;GO:0000987 proximal promoter sequence-specific DNA binding;GO:000

9 ubiquitin-like protein ligase binding;GO:0019899 enzyme binding
lymerase II activating transcription factor binding;GO:0008094 DNA-dependent ATPase activ
g;GO:1901363 heterocyclic compound binding
)Metabolic pathways
:0070004 cysteine-type exopeptidase activity;GO:1990380 Lys48-specific deubiquitinase act
ce01110)Biosynthesis of secondary metabolites; (sce01100)Metabolic pathways

)Glycerophospholipid metabolism; (sce01110)Biosynthesis of secondary metabolites
y;GO:0072509 divalent inorganic cation transmembrane transporter activity;GO:0046915 tran

ity;GO:0015194 L-serine transmembrane transporter activity;GO:0005302 L-tyrosine transmem

ι strain has a sporulation defect characterized by elevated dityrosine in the soluble frac
0)Mismatch repair; (sce03030)DNA replication

GO:0003723 RNA binding

RNA methyltransferase activity;GO:0140101 catalytic activity, acting on a tRNA

0:0004672 protein kinase activity;GO:0016773 phosphotransferase activity, alcohol group a
3676 nucleic acid binding
ity;GO:0071916 dipeptide transmembrane transporter activity;GO:0035673 oligopeptide trans

3420)Nucleotide excision repair
de binding;GO:0032561 guanyl ribonucleotide binding
757 S-adenosylmethionine-dependent methyltransferase activity;GO:0008168 methyltransferas

aminopeptidase activity;GO:0008235 metalloexopeptidase activity
ing;GO:0043168 anion binding

3676 nucleic acid binding

on initiation factor activity;GO:0004386 helicase activity

ar_function

tive phase, fructose 6P => ribose 5P; (sce)Pentose phosphate pathway, non-oxidative phase
tive phase, fructose 6P => ribose 5P; (sce)Pentose phosphate pathway, non-oxidative phase
fic DNA binding;GO:0046914 transition metal ion binding
4-hydroxybenzoate => ubiquinone; (sce)Ubiquinone biosynthesis, eukaryotes, 4-hydroxybenz

c double-stranded DNA binding;GO:0003690 double-stranded DNA binding
activity;GO:0000149 SNARE binding

> THF; (sce)Tetrahydrofolate biosynthesis, GTP => THF; (sce00790)Folate biosynthesis
03674 molecular_function

reticulum
0:0010181 FMN binding;GO:0016655 oxidoreductase activity, acting on NAD(P)H, quinone or s
A replication; (sce03420)Nucleotide excision repair
139 metal ion:proton antiporter activity;GO:0015386 potassium:proton antiporter activity
)Glycerophospholipid metabolism; (sce01110)Biosynthesis of secondary metabolites

vity;GO:0005384 manganese ion transmembrane transporter activity;GO:0015295 solute:proton

712 transcription coregulator activity;GO:0000981 DNA-binding transcription factor activi

sce01110)Biosynthesis of secondary metabolites
hatidylinositol-3,4-bisphosphate binding;GO:0080025 phosphatidylinositol-3,5-bisphosphate

ferase activity;GO:0008168 methyltransferase activity;GO:0016741 transferase activity, tr

RNA polymerase II-specific;GO:0008270 zinc ion binding;GO:0043565 sequence-specific DNA

ted in highly purified mitochondria in high-throughput studies

g;GO:0043167 ion binding
0:0070273 phosphatidylinositol-4-phosphate binding;GO:1901981 phosphatidylinositol phosph

3676 nucleic acid binding

nthesis of secondary metabolites; (sce01100)Metabolic pathways
ce04111)Cell cycle - yeast; (sce04113)Meiosis - yeast

g;GO:0005515 protein binding;GO:0005488 binding
4-hydroxybenzoate => ubiquinone; (sce)Ubiquinone biosynthesis, eukaryotes, 4-hydroxybenz
ce04111)Cell cycle - yeast

-anchor biosynthesis; (sce01100)Metabolic pathways
ansporter activity;GO:0051724 NAD transmembrane transporter activity;GO:0050833 pyruvate
ty;GO:0005198 structural molecule activity;GO:0003676 nucleic acid binding
ree-carbon compounds; (sce)Glycolysis, core module involving three-carbon compounds; (M00
ion channel activity;GO:0022829 wide pore channel activity

oups;GO:0016740 transferase activity;GO:0003824 catalytic activity
tiple species
0086)beta-Oxidation, acyl-CoA synthesis; (sce00061)Fatty acid biosynthesis
0:0004672 protein kinase activity;GO:0016773 phosphotransferase activity, alcohol group a
4720)Long-term potentiation; (hsa04972)Pancreatic secretion

712 transcription coregulator activity;GO:0140110 transcription regulator activity
-anchor biosynthesis; (sce01100)Metabolic pathways
:0008134 transcription factor binding;GO:0005515 protein binding
kinase activity;GO:0010857 calcium-dependent protein kinase activity;GO:0004683 calmodul

esis; (sce01100)Metabolic pathways
ce-specific DNA binding;GO:0000987 proximal promoter sequence-specific DNA binding;GO:000
crotubule binding;GO:0015631 tubulin binding

0000030 mannosyltransferase activity;GO:0016758 transferase activity, transferring hexosy
2-oxoglutarate => oxaloacetate; (M00009)Citrate cycle (TCA cycle, Krebs cycle); (M00011)

sporter activity;GO:0015165 pyrimidine nucleotide-sugar transmembrane transporter activit
inhibitor activity;GO:0003785 actin monomer binding
nding transcription factor recruiting activity;GO:0061629 RNA polymerase II sequence-spec
hatase activity;GO:0004724 magnesium-dependent protein serine/threonine phosphatase activ
6887 ATPase activity;GO:0017111 nucleoside-triphosphatase activity
cription elongation factor complex;GO:0019908 nuclear cyclin-dependent protein kinase hol

lism; (sce01100)Metabolic pathways

ein complex binding;GO:0044877 protein-containing complex binding

ity;GO:0008028 monocarboxylic acid transmembrane transporter activity;GO:0090482 vitamin
198 structural molecule activity;GO:0003674 molecular_function

2-oxoglutarate => oxaloacetate; (M00009)Citrate cycle (TCA cycle, Krebs cycle); (M00011)
amate metabolism
se activity;GO:0016799 hydrolase activity, hydrolyzing N-glycosyl compounds;GO:0016791 ph
5198 structural molecule activity;GO:0003674 molecular_function
hagy - yeast
0019208 phosphatase regulator activity;GO:0030234 enzyme regulator activity

e regulator activity;GO:0008565 protein transporter activity
crotubule binding;GO:0015631 tubulin binding
activity;GO:0003674 molecular_function
)Valine, leucine and isoleucine degradation; (sce00340)Histidine metabolism
-ligase activity;GO:0016878 acid-thiol ligase activity

ein complex binding;GO:0003729 mRNA binding
0005402 carbohydrate:cation symporter activity;GO:0015295 solute:proton symporter activit

44390 ubiquitin-like protein conjugating enzyme binding;GO:0043130 ubiquitin binding

sce01110)Biosynthesis of secondary metabolites
5)N-glycan precursor biosynthesis; (sce00510)N-Glycan biosynthesis
lism

crotubule binding;GO:0015631 tubulin binding

NA binding;GO:0046872 metal ion binding

Metabolic pathways

ein complex binding;GO:0003729 mRNA binding
arch and sucrose metabolism; (sce01100)Metabolic pathways
NA binding;GO:0046872 metal ion binding

tivity;GO:0033549 MAP kinase phosphatase activity;GO:0004724 magnesium-dependent protein
tivity;GO:0030291 protein serine/threonine kinase inhibitor activity;GO:0004860 protein k
is

733 peptide-lysine-N-acetyltransferase activity;GO:0034212 peptide N-acetyltransferase ac
lycerol degradation; (sce00561)Glycerolipid metabolism
ansport
sitol-4,5-bisphosphate binding;GO:1902936 phosphatidylinositol bisphosphate binding
sis - yeast

al ion binding;GO:0046872 metal ion binding
g;GO:1901363 heterocyclic compound binding

215 transporter activity;GO:0003674 molecular_function

0032550 purine ribonucleoside binding
:0033677 DNA/RNA helicase activity;GO:0008026 ATP-dependent helicase activity
198 structural molecule activity;GO:0003674 molecular_function
UMP => UDP/UTP,CDP/CTP; (M00052)Pyrimidine ribonucleotide biosynthesis, UMP => UDP/UTP,C[illegible]

ine and methionine metabolism; (sce01200)Carbon metabolism

reticulum

lecular_function
1 acid-amino acid ligase activity;GO:0016879 ligase activity, forming carbon-nitrogen bon
reticulum
etal ion binding;GO:0046872 metal ion binding
e01100)Metabolic pathways

vity;GO:0005313 L-glutamate transmembrane transporter activity;GO:0015172 acidic amino ac
reticulum
GO:0140110 transcription regulator activity;GO:0003674 molecular_function
idase activity;GO:0008237 metallopeptidase activity
GO:0043139 5'-3' DNA helicase activity;GO:0004003 ATP-dependent DNA helicase activity

0019787 ubiquitin-like protein transferase activity;GO:0140096 catalytic activity, acting
GO:0015036 disulfide oxidoreductase activity;GO:0016667 oxidoreductase activity, acting o
hagy - yeast

phan biosynthesis; (sce01130)Biosynthesis of antibiotics; (sce01110)Biosynthesis of secon

some

.s with Skp1p and Cdc53p; YLR352W is not an essential gene

yeast
ase activity;GO:0004197 cysteine-type endopeptidase activity;GO:0008234 cysteine-type pep

00)Metabolic pathways
ity;GO:0003682 chromatin binding
ity;GO:0046915 transition metal ion transmembrane transporter activity;GO:0046873 metal i
RNA polymerase II-specific;GO:0046983 protein dimerization activity;GO:0003700 DNA-bindi
yase activity;GO:0016835 carbon-oxygen lyase activity

tivity;GO:0003674 molecular_function

aling pathway - yeast; (sce04111)Cell cycle - yeast
43 thiol-dependent ubiquitin-specific protease activity;GO:0036459 thiol-dependent ubiqui

ity;GO:0016436 rRNA (uridine) methyltransferase activity;GO:0008649 rRNA methyltransferas
NA binding;GO:0043022 ribosome binding

;GO:0042626 ATPase activity, coupled to transmembrane movement of substances;GO:1904680 p
inding;GO:0008092 cytoskeletal protein binding

nine, aspartate and glutamate metabolism; (sce01230)Biosynthesis of amino acids

0:1901981 phosphatidylinositol phosphate binding;GO:0005085 guanyl-nucleotide exchange fa
GO:0043139 5'-3' DNA helicase activity;GO:0010521 telomerase inhibitor activity
g;GO:1901363 heterocyclic compound binding
lecular_function
75 RNA polymerase core enzyme binding;GO:0001099 basal RNA polymerase II transcription ma
ter activity;GO:0008028 monocarboxylic acid transmembrane transporter activity;GO:0015605
00141)C1-unit interconversion, eukaryotes; (sce00670)One carbon pool by folate

ed DNA binding;GO:0003697 single-stranded DNA binding

0:0016899 oxidoreductase activity, acting on the CH-OH group of donors, oxygen as accepto

e activity;GO:0036459 thiol-dependent ubiquitinyl hydrolase activity;GO:0004197 cysteine-

5 transferase activity, transferring alkyl or aryl (other than methyl) groups;GO:0016740

g;GO:0043167 ion binding
ne metabolism; (sce00230)Purine metabolism
) methyltransferase activity;GO:0051908 double-stranded DNA 5'-3' exodeoxyribonuclease ac
ption
ing;GO:1902936 phosphatidylinositol bisphosphate binding;GO:1901981 phosphatidylinositol

O:0035184 histone threonine kinase activity;GO:0035173 histone kinase activity
00)Metabolic pathways

e regulator activity;GO:0060589 nucleoside-triphosphatase regulator activity

e; (M00074)N-glycan biosynthesis, high-mannose type; (sce00513)Various types of N-glycan
etallopeptidase activity;GO:0004175 endopeptidase activity
RNA polymerase II-specific;GO:0008270 zinc ion binding;GO:0043565 sequence-specific DNA
O:0004672 protein kinase activity;GO:0016773 phosphotransferase activity, alcohol group a
sis - yeast

ce-specific DNA binding;GO:0000987 proximal promoter sequence-specific DNA binding;GO:000

733 peptide-lysine-N-acetyltransferase activity;GO:0034212 peptide N-acetyltransferase ac
reticulum

nzyme inhibitor activity;GO:0008047 enzyme activator activity
712 transcription coregulator activity;GO:0140110 transcription regulator activity

8186 RNA-dependent ATPase activity;GO:0003724 RNA helicase activity

activity;GO:0004520 endodeoxyribonuclease activity;GO:0004536 deoxyribonuclease activity

198 structural molecule activity;GO:0003674 molecular_function
species; (sce04111)Cell cycle - yeast; (sce04113)Meiosis - yeast

activity;GO:0015171 amino acid transmembrane transporter activity;GO:0046943 carboxylic
ce-specific DNA binding;GO:0000987 proximal promoter sequence-specific DNA binding;GO:000

producing 3' uridine cyclic phosphate ends;GO:0000175 3'-5'-exoribonuclease activity;GO:

(sce03013)RNA transport
5198 structural molecule activity;GO:0003674 molecular_function
ity;GO:0032575 ATP-dependent 5'-3' RNA helicase activity;GO:0033678 5'-3' DNA/RNA helicas
etylase binding;GO:0003682 chromatin binding

hosphatase activity;GO:0016462 pyrophosphatase activity
g;GO:0005488 binding

etal ion binding;GO:0046872 metal ion binding

pendent ATPase activity;GO:0042623 ATPase activity, coupled
hagy - yeast
cle; (sce00630)Glyoxylate and dicarboxylate metabolism

s; (hsa04210)Apoptosis

ding;GO:0000978 RNA polymerase II proximal promoter sequence-specific DNA binding;GO:0000
minus-end-directed;GO:1990939 ATP-dependent microtubule motor activity;GO:0003777 microtu

e-phosphate deaminase activity;GO:0016814 hydrolase activity, acting on carbon-nitrogen (
etic complications; (sce04011)MAPK signaling pathway - yeast; (sce04111)Cell cycle - yeas
tiple species; (sce04146)Peroxisome

08)Ribosome biogenesis in eukaryotes; (sce03013)RNA transport
atty acid degradation
, heme protein as acceptor;GO:0051537 2 iron, 2 sulfur cluster binding;GO:0051536 iron-su

se II proximal promoter sequence-specific DNA binding;GO:0000987 proximal promoter sequen
hagy - yeast
tivity;GO:0046873 metal ion transmembrane transporter activity;GO:0022890 inorganic catio
(hsa04141)Protein processing in endoplasmic reticulum
sferase activity, transferring one-carbon groups;GO:0003735 structural constituent of rib

RNA polymerase II-specific;GO:0008270 zinc ion binding;GO:0043565 sequence-specific DNA
itin-protein transferase activity;GO:0060090 molecular adaptor activity

00)Metabolic pathways
olic pathways
0004661 protein geranylgeranyltransferase activity;GO:0008318 protein prenyltransferase a
e01100)Metabolic pathways
;GO:0016616 oxidoreductase activity, acting on the CH-OH group of donors, NAD or NADP as
NA binding;GO:0046872 metal ion binding
03674 molecular_function

oups;GO:0003723 RNA binding;GO:0016740 transferase activity

00)Metabolic pathways
al ion binding;GO:0046872 metal ion binding
3676 nucleic acid binding
g;GO:0003676 nucleic acid binding
sce00520)Amino sugar and nucleotide sugar metabolism; (sce01130)Biosynthesis of antibioti

specific DNA binding;GO:0003677 DNA binding
ce04111)Cell cycle - yeast; (sce04113)Meiosis - yeast

005048 signal sequence binding;GO:0008565 protein transporter activity

factor binding;GO:0033613 activating transcription factor binding;GO:0001085 RNA polymer
ce-specific DNA binding;GO:0000987 proximal promoter sequence-specific DNA binding;GO:000

0008135 translation factor activity, RNA binding;GO:0003924 GTPase activity
lochaperone activity;GO:0140104 molecular carrier activity

ce04111)Cell cycle - yeast; (sce04113)Meiosis - yeast
89 carboxylic ester hydrolase activity;GO:0016788 hydrolase activity, acting on ester bon

712 transcription coregulator activity;GO:0140110 transcription regulator activity
clathrin heavy chain binding;GO:0005546 phosphatidylinositol-4,5-bisphosphate binding

s; (hsa05034)Alcoholism

ein complex binding;GO:0044877 protein-containing complex binding

03674 molecular_function

ree-carbon compounds; (sce)Glycolysis, core module involving three-carbon compounds; (M00

ce04141)Protein processing in endoplasmic reticulum

00083)Fatty acid biosynthesis, elongation; (sce00780)Biotin metabolism
514 DNA demethylase activity;GO:0031543 peptidyl-proline dioxygenase activity

nsporter activity;GO:0015301 anion:anion antiporter activity;GO:0015116 sulfate transmemb

activity;GO:0140097 catalytic activity, acting on DNA
00)Metabolic pathways
helicase activity;GO:0004386 helicase activity
hionine salvage pathway; (sce00270)Cysteine and methionine metabolism

3676 nucleic acid binding

dipeptidase activity;GO:0008242 omega peptidase activity
inding;GO:0001135 RNA polymerase II transcription regulator recruiting activity;GO:000109
;GO:0060590 ATPase regulator activity;GO:0051087 chaperone binding
00082)Fatty acid biosynthesis, initiation; (sce00061)Fatty acid biosynthesis

binding;GO:0003676 nucleic acid binding

ity, acting on ester bonds;GO:0016787 hydrolase activity
A binding;GO:0003723 RNA binding

kinase regulator activity;GO:0019887 protein kinase regulator activity;GO:0019207 kinase

198 structural molecule activity;GO:0003674 molecular_function
;GO:0005488 binding

sis - yeast
ity;GO:0000297 spermine transmembrane transporter activity;GO:0015203 polyamine transmemb
tein tyrosine phosphatase activity;GO:0004721 phosphoprotein phosphatase activity
0032550 purine ribonucleoside binding

ein complex binding;GO:0044877 protein-containing complex binding

ce04111)Cell cycle - yeast
ity;GO:0016436 rRNA (uridine) methyltransferase activity;GO:0008649 rRNA methyltransferas

hagy - yeast

nsferase; (sce)N-glycosylation by oligosaccharyltransferase; (sce00510)N-Glycan biosynthe
ne; (M00026)Histidine biosynthesis, PRPP => histidine; (sce00340)Histidine metabolism

198 structural molecule activity;GO:0003674 molecular_function

nding;GO:0008092 cytoskeletal protein binding

-anchor biosynthesis; (sce01100)Metabolic pathways
g;GO:0043167 ion binding
ator activity;GO:0030695 GTPase regulator activity

reticulum
:0017150 tRNA dihydrouridine synthase activity;GO:0016627 oxidoreductase activity, acting

nsferase; (sce)N-glycosylation by oligosaccharyltransferase; (sce00510)N-Glycan biosynthe

vity;GO:0015299 solute:proton antiporter activity;GO:0015298 solute:cation antiporter act

0015215 nucleotide transmembrane transporter activity;GO:0051184 cofactor transmembrane t
5198 structural molecule activity;GO:0003674 molecular_function
ylpyrimidine kinase activity;GO:0008902 hydroxymethylpyrimidine kinase activity
ty acid degradation; (sce00010)Glycolysis / Gluconeogenesis
ity;GO:0008251 tRNA-specific adenosine deaminase activity;GO:0004000 adenosine deaminase
rsions; (sce01100)Metabolic pathways

00)Metabolic pathways

osphatase activity;GO:0106018 phosphatidylinositol-3,5-bisphosphate phosphatase activity;

ic compound binding;GO:0097159 organic cyclic compound binding
single-stranded DNA binding;GO:1990837 sequence-specific double-stranded DNA binding
GO:0034458 3'-5' RNA helicase activity;GO:0004004 ATP-dependent RNA helicase activity
RNA polymerase II-specific;GO:0008270 zinc ion binding;GO:0043565 sequence-specific DNA
-phosphatase activity;GO:0034594 phosphatidylinositol trisphosphate phosphatase activity;
44390 ubiquitin-like protein conjugating enzyme binding;GO:0061630 ubiquitin protein liga

ing;GO:0001042 RNA polymerase I core binding;GO:0001181 RNA polymerase I general transcri
e; (M00074)N-glycan biosynthesis, high-mannose type; (sce00513)Various types of N-glycan
3676 nucleic acid binding
198 structural molecule activity;GO:0003674 molecular_function

ng;GO:0016887 ATPase activity
;GO:0042895 antibiotic transmembrane transporter activity;GO:0008028 monocarboxylic acid

674 molecular_function
doplasmic reticulum; (sce00062)Fatty acid elongation; (sce01040)Biosynthesis of unsaturat
fic DNA binding;GO:0046914 transition metal ion binding
clathrin heavy chain binding;GO:0005546 phosphatidylinositol-4,5-bisphosphate binding
binding;GO:0060090 molecular adaptor activity

) activity;GO:0016884 carbon-nitrogen ligase activity, with glutamine as amido-N-donor;GO

cellular part
ating projection tip
etabolism
se activity;GO:0042171 lysophosphatidic acid acyltransferase activity;GO:0016411 acylglyc

1990189 peptide-serine-N-acetyltransferase activity;GO:0010485 H4 histone acetyltransfera
ce04141)Protein processing in endoplasmic reticulum
embrane transporter activity;GO:0015291 secondary active transmembrane transporter activi

g;GO:1901363 heterocyclic compound binding

674 molecular_function
(sce03013)RNA transport

reticulum

n binding;GO:0005488 binding

GO:0005515 protein binding

tryptophan; (M00023)Tryptophan biosynthesis, chorismate => tryptophan; (sce00400)Phenylala
42623 ATPase activity, coupled;GO:0016887 ATPase activity
ity;GO:0000979 RNA polymerase II core promoter sequence-specific DNA binding;GO:0001046 c

factor binding;GO:0070491 repressing transcription factor binding;GO:0001085 RNA polymer

:0042123 glucanosyltransferase activity;GO:0016740 transferase activity
thesis, PA => PS => PE; (sce)Phosphatidylethanolamine (PE) biosynthesis, PA => PS => PE;

712 transcription coregulator activity;GO:0140110 transcription regulator activity
3676 nucleic acid binding

; (sce00514)Other types of O-glycan biosynthesis; (sce01100)Metabolic pathways
e regulator activity;GO:0098772 molecular function regulator
lication; (sce03420)Nucleotide excision repair
198 structural molecule activity;GO:0003674 molecular_function

0662 coenzyme binding;GO:0048037 cofactor binding

5198 structural molecule activity;GO:0003674 molecular_function
0005543 phospholipid binding;GO:0008289 lipid binding

ein complex binding;GO:0044877 protein-containing complex binding
nscription corepressor activity;GO:0003712 transcription coregulator activity

n binding;GO:0005488 binding

itin-like protein transferase activity;GO:0140096 catalytic activity, acting on a protein
osine degradation; (sce00600)Sphingolipid metabolism
008139 nuclear localization sequence binding;GO:0098847 sequence-specific single stranded

choline => PC; (M00090)Phosphatidylcholine (PC) biosynthesis, choline => PC; (sce00564)Gl
osine degradation; (sce00600)Sphingolipid metabolism
3676 nucleic acid binding
tein binding;GO:0004842 ubiquitin-protein transferase activity
ferase activity;GO:0008168 methyltransferase activity;GO:0016741 transferase activity, tr
y;GO:0046915 transition metal ion transmembrane transporter activity;GO:0046873 metal ion
bolism; (hsa00030)Pentose phosphate pathway; (hsa01200)Carbon metabolism

ted in highly purified mitochondria in high-throughput studies; YBL059W has a paralog, YE

activity;GO:0015297 antiporter activity;GO:0015932 nucleobase-containing compound transm
ity;GO:0016423 tRNA (guanine) methyltransferase activity;GO:0008175 tRNA methyltransferas
H2 groups, NAD or NADP as acceptor;GO:0016725 oxidoreductase activity, acting on CH or CH

ituent of cytoskeleton;GO:0017111 nucleoside-triphosphatase activity

e regulator activity;GO:0060589 nucleoside-triphosphatase regulator activity
ity;GO:0005381 iron ion transmembrane transporter activity;GO:0046915 transition metal io

ein complex binding;GO:0044877 protein-containing complex binding
8 phospholipid transporter activity;GO:0005319 lipid transporter activity
cific DNA binding;GO:0001163 RNA polymerase I regulatory region sequence-specific DNA bin
e01100)Metabolic pathways
smatch repair; (sce03030)DNA replication

etabolism

733 peptide-lysine-N-acetyltransferase activity;GO:0034212 peptide N-acetyltransferase ac
prolyl cis-trans isomerase activity;GO:0016859 cis-trans isomerase activity

minopeptidase activity;GO:0008238 exopeptidase activity

198 structural molecule activity;GO:0003674 molecular_function

O:1901981 phosphatidylinositol phosphate binding;GO:0035091 phosphatidylinositol binding
rotein phosphatase regulator activity;GO:0019903 protein phosphatase binding

ter activity;GO:0008028 monocarboxylic acid transmembrane transporter activity;GO:0015605

reticulum
ce04111)Cell cycle - yeast; (sce04113)Meiosis - yeast
-anchor biosynthesis; (sce01100)Metabolic pathways

061650 ubiquitin-like protein conjugating enzyme activity;GO:0004842 ubiquitin-protein tr
5198 structural molecule activity;GO:0046983 protein dimerization activity

ne; (M00026)Histidine biosynthesis, PRPP => histidine; (sce00340)Histidine metabolism
ter activity;GO:0046943 carboxylic acid transmembrane transporter activity;GO:0008514 org
250)Alanine, aspartate and glutamate metabolism; (sce01110)Biosynthesis of secondary meta
e regulator activity;GO:0051879 Hsp90 protein binding

binding;GO:0003677 DNA binding
GO:0000293 ferric-chelate reductase activity;GO:0016723 oxidoreductase activity, oxidizin
ty;GO:0005516 calmodulin binding;GO:0004674 protein serine/threonine kinase activity

> THF; (sce)Tetrahydrofolate biosynthesis, GTP => THF; (sce00790)Folate biosynthesis
ing;GO:0003682 chromatin binding
tiple species; (sce00230)Purine metabolism; (sce04113)Meiosis - yeast

etic complications; (sce04213)Longevity regulating pathway - multiple species; (sce04138)
ide kinase activity;GO:0019205 nucleobase-containing compound kinase activity

PRPP + glutamine => IMP; (sce)Inosine monophosphate biosynthesis, PRPP + glutamine => IMP
idylinositol binding;GO:0005543 phospholipid binding

ne; (M00026)Histidine biosynthesis, PRPP => histidine; (sce00340)Histidine metabolism

e regulator activity;GO:0060589 nucleoside-triphosphatase regulator activity

nction
ate => H2S; (sce)Assimilatory sulfate reduction, sulfate => H2S; (sce00920)Sulfur metabol
tivity, acting on carbon-nitrogen (but not peptide) bonds, in nitriles;GO:0016810 hydrola
004674 protein serine/threonine kinase activity;GO:0019901 protein kinase binding
g;GO:0003723 RNA binding
ctivity;GO:0004713 protein tyrosine kinase activity;GO:0004674 protein serine/threonine k
674 molecular_function

0521 telomerase inhibitor activity;GO:0043047 single-stranded telomeric DNA binding
cription corepressor activity;GO:0005543 phospholipid binding
lap endonuclease activity;GO:0016888 endodeoxyribonuclease activity, producing 5'-phospho
filament binding;GO:0003779 actin binding

20)Sulfur metabolism; (sce00270)Cysteine and methionine metabolism

reticulum
GO:0005261 cation channel activity;GO:0022839 ion gated channel activity
y;GO:0015174 basic amino acid transmembrane transporter activity;GO:0015171 amino acid tr
GO:0008525 phosphatidylcholine transporter activity;GO:0031210 phosphatidylcholine bindin

recruiting activity;GO:0000979 RNA polymerase II core promoter sequence-specific DNA bind
is

ansporter activity;GO:0003674 molecular_function

ree-carbon compounds; (sce)Glycolysis, core module involving three-carbon compounds; (M00
ratase activity;GO:0016836 hydro-lyase activity;GO:0016835 carbon-oxygen lyase activity
6791 phosphatase activity;GO:0042578 phosphoric ester hydrolase activity
0008135 translation factor activity, RNA binding;GO:0003729 mRNA binding
in processing in endoplasmic reticulum; (sce03013)RNA transport

13)Meiosis - yeast
:0031369 translation initiation factor binding;GO:0003729 mRNA binding

etallopeptidase activity;GO:0004175 endopeptidase activity
e regulator activity;GO:0060589 nucleoside-triphosphatase regulator activity

nsporter activity;GO:0015196 L-tryptophan transmembrane transporter activity;GO:0015173 a
sce00240)Pyrimidine metabolism; (sce01100)Metabolic pathways

ne metabolism; (sce00230)Purine metabolism
itor activity;GO:0072542 protein phosphatase activator activity;GO:0019211 phosphatase ac

NA-binding transcription factor activity;GO:0140110 transcription regulator activity

; (sce01110)Biosynthesis of secondary metabolites; (sce01100)Metabolic pathways

-anchor biosynthesis; (sce01100)Metabolic pathways
etabolic pathways

NA-binding transcription factor activity;GO:0140110 transcription regulator activity

sm; (sce01110)Biosynthesis of secondary metabolites; (sce01100)Metabolic pathways
O:1901981 phosphatidylinositol phosphate binding;GO:0035091 phosphatidylinositol binding

e01130)Biosynthesis of antibiotics; (sce01100)Metabolic pathways

GO:0043021 ribonucleoprotein complex binding

198 structural molecule activity;GO:0003674 molecular_function
pholipase activity;GO:0016298 lipase activity
215 transporter activity;GO:0003674 molecular_function
;GO:0051020 GTPase binding;GO:0008270 zinc ion binding
:ted in highly purified mitochondria in high-throughput studies
nding;GO:0032559 adenyl ribonucleotide binding
ein complex binding;GO:0044877 protein-containing complex binding
species; (sce04011)MAPK signaling pathway - yeast

0003924 GTPase activity;GO:0005525 GTP binding

110 transcription regulator activity;GO:0003674 molecular_function
198 structural molecule activity;GO:0003674 molecular_function
orter activity;GO:0015169 glycerol-3-phosphate transmembrane transporter activity;GO:0015
er activity;GO:0015216 purine nucleotide transmembrane transporter activity;GO:0015215 nu
712 transcription coregulator activity;GO:0043565 sequence-specific DNA binding
nositol-3,4-bisphosphate binding;GO:0010314 phosphatidylinositol-5-phosphate binding

erase;GO:0016783 sulfurtransferase activity;GO:0000049 tRNA binding

RNA polymerase II-specific;GO:0008270 zinc ion binding;GO:0043565 sequence-specific DNA

00)Metabolic pathways

733 peptide-lysine-N-acetyltransferase activity;GO:0034212 peptide N-acetyltransferase ac
nding;GO:0032559 adenyl ribonucleotide binding

reticulum

8186 RNA-dependent ATPase activity;GO:0003724 RNA helicase activity

DNA binding;GO:0003676 nucleic acid binding
etic complications; (sce00562)Inositol phosphate metabolism; (sce04070)Phosphatidylinosit
3420)Nucleotide excision repair
HF; (M00126)Tetrahydrofolate biosynthesis, GTP => THF; (sce00790)Folate biosynthesis
e01100)Metabolic pathways

ivator activity;GO:0030695 GTPase regulator activity

495 protein membrane anchor;GO:0005515 protein binding
P => ADP,ATP; (sce00730)Thiamine metabolism; (M00049)Adenine ribonucleotide biosynthesis,
0086)beta-Oxidation, acyl-CoA synthesis; (sce00061)Fatty acid biosynthesis

:0016641 oxidoreductase activity, acting on the CH-NH2 group of donors, oxygen as accepto
haride; (M00065)GPI-anchor biosynthesis, core oligosaccharide; (sce00563)Glycosylphosphat

phase, glucose 6P => ribulose 5P; (sce)Pentose phosphate pathway, oxidative phase, gluco

GO:0008525 phosphatidylcholine transporter activity;GO:0005548 phospholipid transporter a
0032550 purine ribonucleoside binding

etabolic pathways

02010)ABC transporters; (hsa04976)Bile secretion

6791 phosphatase activity;GO:0042578 phosphoric ester hydrolase activity
pase activity;GO:0052689 carboxylic ester hydrolase activity

O:0004672 protein kinase activity;GO:0016773 phosphotransferase activity, alcohol group a
activity;GO:0004864 protein phosphatase inhibitor activity;GO:0019212 phosphatase inhibit

ce-specific DNA binding;GO:0000987 proximal promoter sequence-specific DNA binding;GO:000
ing;GO:0003682 chromatin binding
vity;GO:0005088 Ras guanyl-nucleotide exchange factor activity;GO:0017137 Rab GTPase bind

ne metabolism; (sce00230)Purine metabolism

ity;GO:0022857 transmembrane transporter activity;GO:0005215 transporter activity

ansport
0:0015038 glutathione disulfide oxidoreductase activity;GO:0015037 peptide disulfide oxid

GO:0015075 ion transmembrane transporter activity;GO:0022857 transmembrane transporter ac
ce04111)Cell cycle - yeast

03674 molecular_function
r plaque of spindle pole body;GO:0005816 spindle pole body

tivity;GO:0019707 protein-cysteine S-acyltransferase activity;GO:0016417 S-acyltransferas

inding;GO:0043169 cation binding
reticulum
crotubule binding;GO:0015631 tubulin binding

activity;GO:0140097 catalytic activity, acting on DNA
ing;GO:0046914 transition metal ion binding
glycan precursor trimming; (sce)N-glycan biosynthesis, high-mannose type

abolic pathways
070008 serine-type exopeptidase activity;GO:0004180 carboxypeptidase activity

vity;GO:0015179 L-amino acid transmembrane transporter activity;GO:0015171 amino acid tra

e regulator activity;GO:0060589 nucleoside-triphosphatase regulator activity

e regulator activity;GO:0060589 nucleoside-triphosphatase regulator activity
idase activity;GO:0070011 peptidase activity, acting on L-amino acid peptides

ng;GO:0000268 peroxisome targeting sequence binding;GO:0005048 signal sequence binding

um channel activity;GO:0140135 mechanosensitive cation channel activity;GO:0008381 mechan
ecific DNA binding;GO:0003677 DNA binding

5166)HTLV-I infection

6895 exodeoxyribonuclease activity, producing 5'-phosphomonoesters;GO:0008409 5'-3' exonu

hosphodiesterase I activity;GO:0047429 nucleoside-triphosphate diphosphatase activity

0019208 phosphatase regulator activity;GO:0030234 enzyme regulator activity
activity;GO:0140097 catalytic activity, acting on DNA
sis - yeast
itin-protein transferase activity;GO:0060090 molecular adaptor activity
-anchor biosynthesis; (sce01100)Metabolic pathways

surveillance pathway; (sce04138)Autophagy - yeast
GO:0008422 beta-glucosidase activity;GO:0015926 glucosidase activity
cle - yeast; (sce04113)Meiosis - yeast
g;GO:0001069 regulatory region RNA binding;GO:0003729 mRNA binding

dase activity;GO:0015924 mannosyl-oligosaccharide mannosidase activity;GO:0004559 alpha-m
0:0004672 protein kinase activity;GO:0016773 phosphotransferase activity, alcohol group a

nsferase; (sce)N-glycosylation by oligosaccharyltransferase; (sce00510)N-Glycan biosynthe
ce-specific DNA binding;GO:0000987 proximal promoter sequence-specific DNA binding;GO:000

specific);GO:0010484 H3 histone acetyltransferase activity;GO:0004402 histone acetyltran

03691 double-stranded telomeric DNA binding;GO:0042162 telomeric DNA binding
tem; (sce04138)Autophagy - yeast
e regulator activity;GO:0060589 nucleoside-triphosphatase regulator activity
n binding;GO:0005488 binding
homoserine => threonine; (sce)Threonine biosynthesis, aspartate => homoserine => threoni
reticulum

otein binding;GO:0005488 binding
olymerase II proximal promoter sequence-specific DNA binding;GO:0003714 transcription cor
198 structural molecule activity;GO:0003674 molecular_function
ization activity;GO:0046983 protein dimerization activity
binding;GO:0003676 nucleic acid binding
ce-specific DNA binding;GO:0000987 proximal promoter sequence-specific DNA binding;GO:000
ce-specific DNA binding;GO:0000987 proximal promoter sequence-specific DNA binding;GO:000

2-oxoadipate; (M00433)Lysine biosynthesis, 2-oxoglutarate => 2-oxoadipate; (M00030)Lysine

;GO:0034212 peptide N-acetyltransferase activity;GO:0008080 N-acetyltransferase activity
nding;GO:0032559 adenyl ribonucleotide binding
8186 RNA-dependent ATPase activity;GO:0003724 RNA helicase activity
;GO:0102202 soladodine glucosyltransferase activity;GO:0102203 brassicasterol glucosyltra

ctivity;GO:0016671 oxidoreductase activity, acting on a sulfur group of donors, disulfide

(sce03013)RNA transport

00)Metabolic pathways
›tein localizes to mitochondria; YPL107W is not an essential gene
binding;GO:1901363 heterocyclic compound binding

04 ATP-dependent RNA helicase activity;GO:0008186 RNA-dependent ATPase activity
de binding;GO:0030554 adenyl nucleotide binding

y;GO:0015203 polyamine transmembrane transporter activity;GO:0008519 ammonium transmembra
ansporter activity;GO:0046872 metal ion binding

tivity;GO:0019707 protein-cysteine S-acyltransferase activity;GO:0016417 S-acyltransferas

198 structural molecule activity;GO:0003723 RNA binding
n, oxaloacetate => 2-oxoglutarate; (sce)Citrate cycle, first carbon oxidation, oxaloaceta

activity;GO:0003674 molecular_function

›tein localizes to mitochondria; YMR252C is not an essential gene

›d by Rml01p; YJR061W has a paralog, MNN4, that arose from the whole genome duplication

y;GO:0008324 cation transmembrane transporter activity;GO:0015075 ion transmembrane trans

;GO:0060590 ATPase regulator activity;GO:0060589 nucleoside-triphosphatase regulator acti

ylation-dependent protein binding;GO:0140030 modification-dependent protein binding
ament binding;GO:0003779 actin binding

ansporter activity;GO:0005287 high-affinity basic amino acid transmembrane transporter ac

orter activity;GO:0015291 secondary active transmembrane transporter activity
:0016620 oxidoreductase activity, acting on the aldehyde or oxo group of donors, NAD or N
ing;GO:1902936 phosphatidylinositol bisphosphate binding;GO:1901981 phosphatidylinositol

O:0005488 binding
PRPP + glutamine => IMP; (sce)Inosine monophosphate biosynthesis, PRPP + glutamine => IMP

ce-specific DNA binding;GO:0000987 proximal promoter sequence-specific DNA binding;GO:000
haride; (M00065)GPI-anchor biosynthesis, core oligosaccharide; (sce00563)Glycosylphosphat

y;GO:0042124 1,3-beta-glucanosyltransferase activity;GO:0042123 glucanosyltransferase act
oserine binding;GO:0099122 RNA polymerase II C-terminal domain binding;GO:0050815 phospho
lication; (sce03420)Nucleotide excision repair
tivity;GO:0046873 metal ion transmembrane transporter activity;GO:0015077 monovalent inor
:0033677 DNA/RNA helicase activity;GO:0004003 ATP-dependent DNA helicase activity

GO:0005261 cation channel activity;GO:0022839 ion gated channel activity

ity;GO:0000104 succinate dehydrogenase activity;GO:0016635 oxidoreductase activity, actin

6 iron-sulfur cluster binding;GO:0051540 metal cluster binding
00)Metabolic pathways

ne metabolism; (sce00230)Purine metabolism

236 serine-type peptidase activity;GO:0017171 serine hydrolase activity
olic pathways
13)Meiosis - yeast
nction

sferase activity, transferring acyl groups other than amino-acyl groups;GO:0016746 transf

ing;GO:0001042 RNA polymerase I core binding;GO:0001181 RNA polymerase I general transcri

0019208 phosphatase regulator activity;GO:0030234 enzyme regulator activity
110 transcription regulator activity;GO:0003674 molecular_function

y;GO:0015174 basic amino acid transmembrane transporter activity;GO:0015171 amino acid tr
)Autophagy - yeast; (sce01100)Metabolic pathways
ce-specific DNA binding;GO:0000987 proximal promoter sequence-specific DNA binding;GO:000
hagy - yeast
osphatase activity;GO:0016462 pyrophosphatase activity

abolic pathways

DNA binding;GO:0003677 DNA binding
itiation factor activity;GO:0000049 tRNA binding

pendent ATPase activity;GO:0042623 ATPase activity, coupled
ding;GO:0008092 cytoskeletal protein binding
GO:0015036 disulfide oxidoreductase activity;GO:0016667 oxidoreductase activity, acting o

ein complex binding;GO:0044877 protein-containing complex binding
5)N-glycan precursor biosynthesis; (sce00510)N-Glycan biosynthesis
0000030 mannosyltransferase activity;GO:0016758 transferase activity, transferring hexosy
0098518 polynucleotide phosphatase activity;GO:0016791 phosphatase activity
ding;GO:0046983 protein dimerization activity

ing;GO:0005488 binding
hagy - yeast

:0003723 RNA binding

ic activity;GO:0003674 molecular_function

ntous fungus, <i>Ashbya gossypii</i>; SWAT-GFP and seamless-GFP fusion proteins localize

sequence-specific DNA binding;GO:0001163 RNA polymerase I regulatory region sequence-spec

otein binding;GO:0005488 binding

igase binding;GO:0044389 ubiquitin-like protein ligase binding

GO:0016787 hydrolase activity;GO:0003824 catalytic activity
e04070)Phosphatidylinositol signaling system; (sce04139)Mitophagy - yeast
ce04011)MAPK signaling pathway - yeast; (sce01110)Biosynthesis of secondary metabolites
ity;GO:0003720 telomerase activity;GO:0003964 RNA-directed DNA polymerase activity
00)Metabolic pathways

binding;GO:0005488 binding

ce04141)Protein processing in endoplasmic reticulum; (sce04111)Cell cycle - yeast

15385 sodium:proton antiporter activity;GO:0005451 monovalent cation:proton antiporter ac
rganelle;GO:0043227 membrane-bounded organelle
ty;GO:0005198 structural molecule activity;GO:0003676 nucleic acid binding
-acetylated histone binding;GO:0140033 acetylation-dependent protein binding
ne; (M00026)Histidine biosynthesis, PRPP => histidine; (sce00340)Histidine metabolism
specific;GO:0003747 translation release factor activity;GO:0008079 translation terminati
ctivity;GO:0009019 tRNA (guanine-N1-)-methyltransferase activity;GO:0016423 tRNA (guanine

de binding;GO:0030554 adenyl nucleotide binding
inositol binding;GO:0005543 phospholipid binding

drate kinase activity;GO:0016773 phosphotransferase activity, alcohol group as acceptor
ing;GO:1902936 phosphatidylinositol bisphosphate binding;GO:0017048 Rho GTPase binding
oserine binding;GO:0099122 RNA polymerase II C-terminal domain binding;GO:0045142 triplex
5)N-glycan precursor biosynthesis; (sce00510)N-Glycan biosynthesis

003676 nucleic acid binding
etabolic pathways

orter activity;GO:0032934 sterol binding

ding;GO:0005096 GTPase activator activity

)Arginine and proline metabolism; (sce00480)Glutathione metabolism

40)Spliceosome; (sce03013)RNA transport

hosphatase activity;GO:0016462 pyrophosphatase activity
ivity;GO:0005381 iron ion transmembrane transporter activity;GO:0072509 divalent inorgani

ng, bridging;GO:0060090 molecular adaptor activity
ivity;GO:0043169 cation binding

somerase activity;GO:0003824 catalytic activity
00083)Fatty acid biosynthesis, elongation; (sce)Fatty acid biosynthesis, initiation
ce04111)Cell cycle - yeast
lymerase II activating transcription factor binding;GO:0008094 DNA-dependent ATPase activ

e regulator activity;GO:0060589 nucleoside-triphosphatase regulator activity

139 metal ion:proton antiporter activity;GO:0015386 potassium:proton antiporter activity

ce04141)Protein processing in endoplasmic reticulum

(sce03013)RNA transport
lication origin binding;GO:0001227 DNA-binding transcription repressor activity, RNA poly

g;GO:0051787 misfolded protein binding;GO:0031072 heat shock protein binding
species; (sce04011)MAPK signaling pathway - yeast
serine; (sce)Serine biosynthesis, glycerate-3P => serine; (sce00750)Vitamin B6 metabolis
e regulator activity;GO:0060589 nucleoside-triphosphatase regulator activity
ter activity;GO:0046943 carboxylic acid transmembrane transporter activity;GO:0015293 sym
ce04111)Cell cycle - yeast; (sce04113)Meiosis - yeast

n binding;GO:0005488 binding
ce-specific DNA binding;GO:0000987 proximal promoter sequence-specific DNA binding;GO:000

-anchor biosynthesis; (sce01100)Metabolic pathways

sce01040)Biosynthesis of unsaturated fatty acids; (sce00071)Fatty acid degradation

e protein binding;GO:0005198 structural molecule activity
;GO:0005488 binding
pendent ATPase activity;GO:0042623 ATPase activity, coupled
g;GO:0043167 ion binding

198 structural molecule activity;GO:0003674 molecular_function
ivity;GO:0017070 U6 snRNA binding;GO:0000386 second spliceosomal transesterification acti
198 structural molecule activity;GO:0003674 molecular_function

nding;GO:0005515 protein binding

ctivity;GO:0106050 tRNA 2'-O-methyltransferase activity;GO:0016423 tRNA (guanine) methylt
te + erythrose-4P => chorismate; (sce)Shikimate pathway, phosphoenolpyruvate + erythrose-

;GO:0043492 ATPase activity, coupled to movement of substances;GO:0005548 phospholipid tr

y;GO:0008028 monocarboxylic acid transmembrane transporter activity;GO:0046943 carboxylic
24)Platinum drug resistance
nding;GO:0032559 adenyl ribonucleotide binding
sphorylation; (sce01100)Metabolic pathways
nding;GO:0044877 protein-containing complex binding
A binding;GO:0035613 RNA stem-loop binding

activity;GO:0015171 amino acid transmembrane transporter activity;GO:0046943 carboxylic
doplasmic reticulum; (sce00062)Fatty acid elongation; (sce01040)Biosynthesis of unsaturat

re-mRNA binding;GO:0008270 zinc ion binding

ce-specific DNA binding;GO:0000976 transcription regulatory region sequence-specific DNA
oreductase activity;GO:0003824 catalytic activity
r binding;GO:0001225 RNA polymerase II transcription coactivator binding;GO:0001224 RNA p

inding;GO:0001097 TFIIH-class transcription factor complex binding;GO:0001135 RNA polymer

phosphatase activity;GO:0043175 RNA polymerase core enzyme binding;GO:0004722 protein ser
ein binding;GO:0005515 protein binding

itor activity;GO:0004857 enzyme inhibitor activity;GO:0030234 enzyme regulator activity

uclease activity;GO:0004518 nuclease activity
ivity;GO:0008195 phosphatidate phosphatase activity;GO:0016791 phosphatase activity

:0016863 intramolecular oxidoreductase activity, transposing C=C bonds;GO:0016860 intramo

y;GO:0016278 lysine N-methyltransferase activity;GO:0008276 protein methyltransferase act
0032550 purine ribonucleoside binding

ne metabolism; (sce00230)Purine metabolism
haride; (M00065)GPI-anchor biosynthesis, core oligosaccharide; (sce00563)Glycosylphosphat
e-stranded RNA-specific ribonuclease activity;GO:0016891 endoribonuclease activity, produ
he mitochondrial genome; SWAT-GFP and mCherry fusion proteins localize to the mitochondri

peroxiredoxin activity;GO:0004601 peroxidase activity
n, oxaloacetate => 2-oxoglutarate; (sce)Citrate cycle, first carbon oxidation, oxaloaceta
NA binding;GO:0003676 nucleic acid binding

g;GO:1901363 heterocyclic compound binding

ing;GO:0030234 enzyme regulator activity
yzing) activity;GO:0016884 carbon-nitrogen ligase activity, with glutamine as amido-N-don

0005198 structural molecule activity;GO:0003674 molecular_function

:0016641 oxidoreductase activity, acting on the CH-NH2 group of donors, oxygen as accepto
pecific DNA binding;GO:0001046 core promoter sequence-specific DNA binding;GO:0016538 cyc
ne metabolism; (sce00230)Purine metabolism

ansport

itin-protein transferase activity;GO:0060090 molecular adaptor activity
ree-carbon compounds; (sce)Glycolysis, core module involving three-carbon compounds; (M00[illegible]

ease activity;GO:0008297 single-stranded DNA exodeoxyribonuclease activity;GO:0035312 5'-
signaling pathway - yeast

e regulator activity;GO:0060589 nucleoside-triphosphatase regulator activity

tiple species; (sce04144)Endocytosis; (sce03040)Spliceosome
ate => H2S; (sce)Assimilatory sulfate reduction, sulfate => H2S; (sce00920)Sulfur metabol
y;GO:0016278 lysine N-methyltransferase activity;GO:0008276 protein methyltransferase act
3674 molecular_function
e => valine / 2-oxobutanoate => isoleucine; (M00019)Valine/isoleucine biosynthesis, pyruv[illegible]
idase activity;GO:0008236 serine-type peptidase activity
specific DNA binding;GO:0003677 DNA binding

nucleoside-diphosphatase activity;GO:0016462 pyrophosphatase activity

3676 nucleic acid binding
e04070)Phosphatidylinositol signaling system; (sce04138)Autophagy - yeast

nding;GO:0008092 cytoskeletal protein binding
NA binding;GO:0003676 nucleic acid binding

e => UDP-galactose; (M00554)Nucleotide sugar biosynthesis, galactose => UDP-galactose; (s
GO:0008649 rRNA methyltransferase activity;GO:0140102 catalytic activity, acting on a rRN
tivity, hydrolyzing O-glycosyl compounds;GO:0016798 hydrolase activity, acting on glycosy
tivity;GO:0046873 metal ion transmembrane transporter activity;GO:0022890 inorganic catio
ty, RNA polymerase II-specific;GO:0000981 DNA-binding transcription factor activity, RNA

HF; (M00126)Tetrahydrofolate biosynthesis, GTP => THF; (sce00790)Folate biosynthesis

RNA polymerase II-specific;GO:0008270 zinc ion binding;GO:0046914 transition metal ion b

tiation factor activity;GO:0000182 rDNA binding;GO:0000993 RNA polymerase II complex bind
003960 NADPH:quinone reductase activity;GO:0016628 oxidoreductase activity, acting on the
0:0004672 protein kinase activity;GO:0016773 phosphotransferase activity, alcohol group a
binding, bridging;GO:0051015 actin filament binding

reticulum

198 structural molecule activity;GO:0003674 molecular_function

e01100)Metabolic pathways

hiamine metabolism; (sce01100)Metabolic pathways
vity;GO:0005384 manganese ion transmembrane transporter activity;GO:0015295 solute:proton

ce04111)Cell cycle - yeast; (sce04113)Meiosis - yeast
g;GO:1901363 heterocyclic compound binding
e regulator activity;GO:0060589 nucleoside-triphosphatase regulator activity
itiation factor activity;GO:0008135 translation factor activity, RNA binding

de binding;GO:0030554 adenyl nucleotide binding

ree-carbon compounds; (sce)Glycolysis, core module involving three-carbon compounds; (MO0

species; (sce04011)MAPK signaling pathway - yeast
5198 structural molecule activity;GO:0003674 molecular_function
ribosome binding;GO:0061630 ubiquitin protein ligase activity

0019787 ubiquitin-like protein transferase activity;GO:0140096 catalytic activity, acting

=> 2-oxoisocaproate; (M00432)Leucine biosynthesis, 2-oxoisovalerate => 2-oxoisocaproate;

5198 structural molecule activity;GO:0003674 molecular_function

tem
oxoglutarate => 2-aminoadipate => lysine; (sce)Lysine biosynthesis, AAA pathway, 2-oxoglu

pendent ATPase activity;GO:0042623 ATPase activity, coupled
4045 aminoacyl-tRNA hydrolase activity;GO:0008079 translation termination factor activity

757 S-adenosylmethionine-dependent methyltransferase activity;GO:0008168 methyltransferas
(H3-K14 specific);GO:0031078 histone deacetylase activity (H3-K14 specific);GO:0017136 N
some
0:0004672 protein kinase activity;GO:0016773 phosphotransferase activity, alcohol group a
signaling pathway - yeast

:0042123 glucanosyltransferase activity;GO:0016740 transferase activity

1100)Metabolic pathways
0:0015450 P-P-bond-hydrolysis-driven protein transmembrane transporter activity;GO:000832
08)Ribosome biogenesis in eukaryotes; (sce03013)RNA transport
otein localizes to the cytoplasm; YPL067C is not an essential gene
ity, DNA binding;GO:0031491 nucleosome binding;GO:0042393 histone binding

phase, glucose 6P => ribulose 5P; (sce)Pentose phosphate pathway, oxidative phase, gluco

binding;GO:0003676 nucleic acid binding
3420)Nucleotide excision repair
GO:0015036 disulfide oxidoreductase activity;GO:0016667 oxidoreductase activity, acting o
tiple species; (sce04138)Autophagy - yeast; (sce04113)Meiosis - yeast
crotubule binding;GO:0015631 tubulin binding
ce-specific DNA binding;GO:0000987 proximal promoter sequence-specific DNA binding;GO:000

arch and sucrose metabolism; (sce01100)Metabolic pathways

e binding;GO:0004364 glutathione transferase activity

amaged DNA binding;GO:0042623 ATPase activity, coupled

nding;GO:0008092 cytoskeletal protein binding

NH2 group of donors, NAD or NADP as acceptor;GO:0016638 oxidoreductase activity, acting o
tivity;GO:0019707 protein-cysteine S-acyltransferase activity;GO:0016417 S-acyltransferas

nuclease activity;GO:0004518 nuclease activity

e activity;GO:0016773 phosphotransferase activity, alcohol group as acceptor

reticulum
abolic pathways

bolism; (sce00240)Pyrimidine metabolism; (sce01100)Metabolic pathways
tivity;GO:0046873 metal ion transmembrane transporter activity;GO:0015077 monovalent inor
ce04144)Endocytosis; (sce04011)MAPK signaling pathway - yeast
uclease activity;GO:0004519 endonuclease activity

; (sce00514)Other types of O-glycan biosynthesis; (sce01100)Metabolic pathways
serine; (sce)Serine biosynthesis, glycerate-3P => serine; (sce00680)Methane metabolism
;GO:0042895 antibiotic transmembrane transporter activity;GO:0008028 monocarboxylic acid

70001 aspartic-type peptidase activity;GO:0004175 endopeptidase activity
y;GO:0072509 divalent inorganic cation transmembrane transporter activity;GO:0046915 tran
tiple species
198 structural molecule activity;GO:0003674 molecular_function
;GO:0008092 cytoskeletal protein binding
sphorylation; (sce01100)Metabolic pathways
fic DNA binding;GO:0046914 transition metal ion binding
GO:0051185 coenzyme transmembrane transporter activity;GO:0008028 monocarboxylic acid tra
0000175 3'-5'-exoribonuclease activity;GO:0016896 exoribonuclease activity, producing 5'-
0:0043169 cation binding

RNA polymerase II-specific;GO:0008270 zinc ion binding;GO:0043565 sequence-specific DNA
5166)HTLV-I infection

ity;GO:0015037 peptide disulfide oxidoreductase activity;GO:0015035 protein disulfide oxi
activity, hydrolyzing O-glycosyl compounds;GO:0016798 hydrolase activity, acting on glyco

31491 nucleosome binding;GO:0042393 histone binding

ding;GO:0070717 poly-purine tract binding

nositol hexakisphosphate binding;GO:0016776 phosphotransferase activity, phosphate group
0:0004672 protein kinase activity;GO:0016773 phosphotransferase activity, alcohol group a
oups;GO:0016740 transferase activity;GO:0003824 catalytic activity

01130)Biosynthesis of antibiotics; (sce01110)Biosynthesis of secondary metabolites

prolyl cis-trans isomerase activity;GO:0016859 cis-trans isomerase activity
abolic pathways
004721 phosphoprotein phosphatase activity;GO:0016791 phosphatase activity
902562 H4 histone acetyltransferase complex;GO:0000123 histone acetyltransferase complex
y;GO:0015203 polyamine transmembrane transporter activity;GO:0008519 ammonium transmembra
inding;GO:0043169 cation binding
molecular_function
;GO:0019205 nucleobase-containing compound kinase activity;GO:0016773 phosphotransferase
g) kinase activity;GO:0004672 protein kinase activity;GO:0016773 phosphotransferase activ
00)Metabolic pathways
2-oxoglutarate => oxaloacetate; (M00009)Citrate cycle (TCA cycle, Krebs cycle); (M00011)

molecular_function
ivity;GO:0050664 oxidoreductase activity, acting on NAD(P)H, oxygen as acceptor;GO:001665
g;GO:1901363 heterocyclic compound binding

hagy - yeast; (sce04138)Autophagy - yeast

kinase activator activity;GO:0019209 kinase activator activity

40)Pyrimidine metabolism; (sce01100)Metabolic pathways
59 ubiquitin-like protein ligase activity;GO:0004842 ubiquitin-protein transferase activi
ein complex binding;GO:0044877 protein-containing complex binding

RNA polymerase II-specific;GO:0008270 zinc ion binding;GO:0043565 sequence-specific DNA
olic pathways

=> GDP,GTP; (M00050)Guanine ribonucleotide biosynthesis IMP => GDP,GTP; (sce00230)Purine
> CoA; (M00120)Coenzyme A biosynthesis, pantothenate => CoA; (sce00770)Pantothenate and C
ce04111)Cell cycle - yeast

tch repair complex binding;GO:0008301 DNA binding, bending
09982 pseudouridine synthase activity;GO:0016866 intramolecular transferase activity
sis - yeast
yl cis-trans isomerase activity;GO:0016859 cis-trans isomerase activity
in binding;GO:0003779 actin binding
5198 structural molecule activity;GO:0003674 molecular_function

=> 2-oxoisocaproate; (M00432)Leucine biosynthesis, 2-oxoisovalerate => 2-oxoisocaproate;
e04011)MAPK signaling pathway - yeast

GO:0033677 DNA/RNA helicase activity;GO:0043138 3'-5' DNA helicase activity
reticulum

reticulum
lecular_function

molecular_function

is (ALS); (hsa04370)VEGF signaling pathway; (hsa04924)Renin secretion
activity;GO:1901239 malonate(1-) transmembrane transporter activity;GO:0015131 oxaloaceta
ity;GO:0015194 L-serine transmembrane transporter activity;GO:0005302 L-tyrosine transmem
a04151)PI3K-Akt signaling pathway

meric DNA binding;GO:0008094 DNA-dependent ATPase activity

0:0043169 cation binding
267 glyoxylate reductase (NADP) activity;GO:0051287 NAD binding

e regulator activity;GO:0060589 nucleoside-triphosphatase regulator activity

doplasmic reticulum; (sce00062)Fatty acid elongation; (sce01040)Biosynthesis of unsaturat

nase binding;GO:0031683 G-protein beta/gamma-subunit complex binding;GO:0019901 protein k

ein complex binding;GO:0044877 protein-containing complex binding
uorescent protein (GFP)-fusion protein localizes to the vacuole; expression induced by ni
198 structural molecule activity;GO:0003674 molecular_function
ne metabolism; (sce00230)Purine metabolism
sis - yeast

ce-specific DNA binding;GO:0000987 proximal promoter sequence-specific DNA binding;GO:000
ce-specific DNA binding;GO:0000987 proximal promoter sequence-specific DNA binding;GO:000
rotein phosphatase binding;GO:0019902 phosphatase binding

110 transcription regulator activity;GO:0003674 molecular_function

ce-specific DNA binding;GO:0000987 proximal promoter sequence-specific DNA binding;GO:000
ion coactivator activity;GO:0003712 transcription coregulator activity
e04070)Phosphatidylinositol signaling system; (sce04011)MAPK signaling pathway - yeast

ne; (M00026)Histidine biosynthesis, PRPP => histidine; (sce00340)Histidine metabolism

atty acid metabolism; (sce01100)Metabolic pathways

amma-tubulin binding;GO:0008017 microtubule binding
etabolic pathways
:0017150 tRNA dihydrouridine synthase activity;GO:0016627 oxidoreductase activity, acting
0:0004672 protein kinase activity;GO:0016773 phosphotransferase activity, alcohol group a
ce-specific DNA binding;GO:0000987 proximal promoter sequence-specific DNA binding;GO:000

0)Cardiac muscle contraction; (hsa05410)Hypertrophic cardiomyopathy (HCM)
RNA polymerase II-specific;GO:0008270 zinc ion binding;GO:0043565 sequence-specific DNA
198 structural molecule activity;GO:0003674 molecular_function

GO:0016787 hydrolase activity;GO:0003824 catalytic activity
e (isomerizing) activity;GO:0070548 L-glutamine aminotransferase activity;GO:0008483 tran

ne metabolism; (sce00230)Purine metabolism

;GO:0042979 ornithine decarboxylase regulator activity;GO:0004857 enzyme inhibitor activi
712 transcription coregulator activity;GO:0140110 transcription regulator activity

activity;GO:0017111 nucleoside-triphosphatase activity
activity;GO:0003674 molecular_function

ssimilatory sulfate reduction, sulfate => H2S; (sce)Assimilatory sulfate reduction, sulfa
lecular_function

g;GO:1901363 heterocyclic compound binding

0019787 ubiquitin-like protein transferase activity;GO:0140096 catalytic activity, acting
0:0004672 protein kinase activity;GO:0016773 phosphotransferase activity, alcohol group a
binding;GO:1901363 heterocyclic compound binding
Metabolic pathways

5)N-glycan precursor biosynthesis; (sce00510)N-Glycan biosynthesis
lizes to the cytoplasm and endoplasmic reticulum

236 serine-type peptidase activity;GO:0017171 serine hydrolase activity
processing in endoplasmic reticulum
008536 Ran GTPase binding;GO:0005048 signal sequence binding

ism; (hsa05231)Choline metabolism in cancer

vity regulating pathway - multiple species; (sce04139)Mitophagy - yeast

specific);GO:0010484 H3 histone acetyltransferase activity;GO:0004402 histone acetyltran

rRNA binding;GO:0003729 mRNA binding
kinase activity;GO:0004693 cyclin-dependent protein serine/threonine kinase activity;GO:0
ing;GO:0032182 ubiquitin-like protein binding
0019208 phosphatase regulator activity;GO:0030234 enzyme regulator activity
n binding;GO:0005488 binding

promoter binding;GO:0003714 transcription corepressor activity

sm
ansporter activity;GO:0003674 molecular_function
ion factor binding;GO:0003677 DNA binding

kinase inhibitor activity;GO:0030291 protein serine/threonine kinase inhibitor activity;G
ator activity;GO:0030695 GTPase regulator activity
biquitin conjugating enzyme binding;GO:0044390 ubiquitin-like protein conjugating enzyme

69 translation initiation factor binding;GO:0043178 alcohol binding

GO:0005515 protein binding

198 structural molecule activity;GO:0003674 molecular_function
8079 translation termination factor activity;GO:0043023 ribosomal large subunit binding
transporter activity;GO:0015079 potassium ion transmembrane transporter activity;GO:00468
g, bridging;GO:0043021 ribonucleoprotein complex binding
674 molecular_function
ular adaptor activity;GO:0005515 protein binding
ansporter activity;GO:0003674 molecular_function

ymerase II repressing transcription factor binding;GO:0001102 RNA polymerase II activatin
a05168)Herpes simplex infection; (hsa05203)Viral carcinogenesis

ne metabolism; (sce00230)Purine metabolism
o ADP-ribose; may have a role in tRNA splicing; contains an A1pp domain
g;GO:1901363 heterocyclic compound binding
P => ADP,ATP; (M00049)Adenine ribonucleotide biosynthesis, IMP => ADP,ATP; (sce00250)Alan

denosylmethionine-dependent methyltransferase activity;GO:0008168 methyltransferase activ
40)Spliceosome; (sce03013)RNA transport
e04070)Phosphatidylinositol signaling system; (sce01100)Metabolic pathways
ivity;GO:0004661 protein geranylgeranyltransferase activity;GO:0008318 protein prenyltran

0050661 NADP binding;GO:0045182 translation regulator activity
sce00520)Amino sugar and nucleotide sugar metabolism; (sce01110)Biosynthesis of secondary

ne metabolism; (sce00230)Purine metabolism
23 oxidoreductase activity, oxidizing metal ions, NAD or NADP as acceptor;GO:0016722 oxid

ter activity;GO:0046964 3'-phosphoadenosine 5'-phosphosulfate transmembrane transporter a
sce01130)Biosynthesis of antibiotics; (sce01110)Biosynthesis of secondary metabolites
activity;GO:0015171 amino acid transmembrane transporter activity;GO:0046943 carboxylic

e04070)Phosphatidylinositol signaling system; (sce00564)Glycerophospholipid metabolism
kinase activity;GO:0004693 cyclin-dependent protein serine/threonine kinase activity;GO:0

ase activity;GO:0004197 cysteine-type endopeptidase activity;GO:0008234 cysteine-type pep
g;GO:1901363 heterocyclic compound binding
ein binding;GO:0005524 ATP binding
ing;GO:0070273 phosphatidylinositol-4-phosphate binding;GO:0032266 phosphatidylinositol-3

plus-end-directed;GO:1990939 ATP-dependent microtubule motor activity;GO:0003777 microtub
;GO:0016758 transferase activity, transferring hexosyl groups;GO:0016757 transferase acti
ree-carbon compounds; (sce)Glycolysis, core module involving three-carbon compounds; (M00

de binding;GO:0030554 adenyl nucleotide binding
sis - yeast
some binding;GO:0043021 ribonucleoprotein complex binding
tryptophan; (M00023)Tryptophan biosynthesis, chorismate => tryptophan; (sce00400)Phenylal
se => UDP-glucose; (sce)Nucleotide sugar biosynthesis, glucose => UDP-glucose; (sce00052)
4040 lipid-transporting ATPase activity;GO:0015248 sterol transporter activity

0138 ubiquitin-like protein-specific isopeptidase activity;GO:0016929 SUMO-specific prote

ribosomal small subunit binding;GO:0043021 ribonucleoprotein complex binding
;GO:0050661 NADP binding;GO:0016709 oxidoreductase activity, acting on paired donors, wit
crotubule binding;GO:0015631 tubulin binding
1110)Biosynthesis of secondary metabolites; (sce01100)Metabolic pathways

ce04111)Cell cycle - yeast; (sce04113)Meiosis - yeast

6983 protein dimerization activity;GO:0003677 DNA binding

-oxobutanoate => isoleucine; (M00570)Isoleucine biosynthesis, threonine => 2-oxobutanoate

yl-CoA; (sce)Pyruvate oxidation, pyruvate => acetyl-CoA; (sce00020)Citrate cycle (TCA cyc
haride; (M00065)GPI-anchor biosynthesis, core oligosaccharide; (sce00563)Glycosylphosphat
ty, RNA polymerase II-specific;GO:0000981 DNA-binding transcription factor activity, RNA

sm; (sce01110)Biosynthesis of secondary metabolites; (sce01100)Metabolic pathways

ic activity;GO:0003674 molecular_function
se II proximal promoter sequence-specific DNA binding;GO:0000987 proximal promoter sequen

rt
198 structural molecule activity;GO:0003674 molecular_function
ivity;GO:0140098 catalytic activity, acting on RNA;GO:0003824 catalytic activity
etabolism; (sce01130)Biosynthesis of antibiotics; (sce01100)Metabolic pathways
recruiting activity;GO:0001134 transcription regulator recruiting activity;GO:0008134 tra
haride; (M00065)GPI-anchor biosynthesis, core oligosaccharide; (sce00563)Glycosylphosphat
sm; (sce01110)Biosynthesis of secondary metabolites; (sce00970)Aminoacyl-tRNA biosynthesi

;GO:0042895 antibiotic transmembrane transporter activity;GO:0008028 monocarboxylic acid
ein complex binding;GO:0044877 protein-containing complex binding

GO:0015075 ion transmembrane transporter activity;GO:0022857 transmembrane transporter ac

8186 RNA-dependent ATPase activity;GO:0003724 RNA helicase activity
;GO:0051020 GTPase binding;GO:0098772 molecular function regulator
e; (M00074)N-glycan biosynthesis, high-mannose type; (sce00513)Various types of N-glycan
clease activity;GO:0004540 ribonuclease activity
-anchor biosynthesis

vity;GO:0005088 Ras guanyl-nucleotide exchange factor activity;GO:0017137 Rab GTPase bind
translation initiation factor activity;GO:0008135 translation factor activity, RNA bindi
;GO:0016616 oxidoreductase activity, acting on the CH-OH group of donors, NAD or NADP as

tivity;GO:0046873 metal ion transmembrane transporter activity;GO:0022890 inorganic catio

ce-specific DNA binding;GO:0000987 proximal promoter sequence-specific DNA binding;GO:000

signaling pathway - yeast
inding;GO:0031267 small GTPase binding
etallopeptidase activity;GO:0016887 ATPase activity

n binding;GO:0005488 binding

sis - yeast

0:0005488 binding

3690 double-stranded DNA binding;GO:0046983 protein dimerization activity
0019208 phosphatase regulator activity;GO:0030234 enzyme regulator activity
ce01110)Biosynthesis of secondary metabolites

tamine (+ PRPP) => UMP; (M00051)Uridine monophosphate biosynthesis, glutamine (+ PRPP) =>

ty;GO:0016859 cis-trans isomerase activity;GO:0016853 isomerase activity
oxidoreductase activity, acting on hydrogen as donor, iron-sulfur protein as acceptor;GO
aminopeptidase activity;GO:0008235 metalloexopeptidase activity
tiple species; (sce04113)Meiosis - yeast
00141)C1-unit interconversion, eukaryotes; (sce00460)Cyanoamino acid metabolism

440)Homologous recombination
se activity;GO:0042910 xenobiotic transmembrane transporter activity;GO:0042626 ATPase ac

e regulator activity;GO:0051879 Hsp90 protein binding

5)N-glycan precursor biosynthesis; (sce00510)N-Glycan biosynthesis

te => phenylalanine; (sce)Phenylalanine biosynthesis, chorismate => phenylalanine; (sce00
iosynthesis of secondary metabolites; (sce01100)Metabolic pathways
e; (M00074)N-glycan biosynthesis, high-mannose type; (sce00513)Various types of N-glycan
RNA polymerase II-specific;GO:0043565 sequence-specific DNA binding;GO:0003700 DNA-bindi
ce-specific DNA binding;GO:0000987 proximal promoter sequence-specific DNA binding;GO:000

cle; (sce00630)Glyoxylate and dicarboxylate metabolism
ein complex binding;GO:0044877 protein-containing complex binding
ate => H2S; (sce)Assimilatory sulfate reduction, sulfate => H2S; (sce00920)Sulfur metabol
reticulum
iosynthesis of secondary metabolites; (sce01100)Metabolic pathways

orter activity;GO:0032934 sterol binding

;GO:1904680 peptide transmembrane transporter activity;GO:0022884 macromolecule transmemb

etabolism
exoribonuclease activity, producing 5'-phosphomonoesters;GO:0004532 exoribonuclease activ
0:1901981 phosphatidylinositol phosphate binding;GO:0035091 phosphatidylinositol binding
674 molecular_function
5198 structural molecule activity;GO:0003674 molecular_function

)Glycerophospholipid metabolism; (sce01110)Biosynthesis of secondary metabolites
ce-specific DNA binding;GO:0000987 proximal promoter sequence-specific DNA binding;GO:000

ein binding;GO:0015631 tubulin binding
e => valine / 2-oxobutanoate => isoleucine; (M00019)Valine/isoleucine biosynthesis, pyruv
sis - yeast

8026 ATP-dependent helicase activity;GO:0003678 DNA helicase activity
;GO:0034212 peptide N-acetyltransferase activity;GO:0008080 N-acetyltransferase activity
g;GO:0005488 binding

haride; (M00065)GPI-anchor biosynthesis, core oligosaccharide; (sce00563)Glycosylphosphat
activity;GO:0016788 hydrolase activity, acting on ester bonds
GO:0000293 ferric-chelate reductase activity;GO:0016723 oxidoreductase activity, oxidizin

sm; (sce00260)Glycine, serine and threonine metabolism; (sce01110)Biosynthesis of seconda
fur metabolism; (sce00270)Cysteine and methionine metabolism

O:0004672 protein kinase activity;GO:0016773 phosphotransferase activity, alcohol group a
0032550 purine ribonucleoside binding

of an alternative ER targeting pathway that has partial functional redundancy with the G
vity;GO:0008536 Ran GTPase binding;GO:0005088 Ras guanyl-nucleotide exchange factor activ

ular adaptor activity;GO:0005515 protein binding
g;GO:0003723 RNA binding
en fluorescent protein (GFP)-fusion protein localizes to the cytoplasm and nucleus; homoz
(sce03013)RNA transport
GO:0043138 3'-5' DNA helicase activity;GO:0004003 ATP-dependent DNA helicase activity
3676 nucleic acid binding

e; (sce04141)Protein processing in endoplasmic reticulum
ce-specific DNA binding;GO:0000987 proximal promoter sequence-specific DNA binding;GO:000
733 peptide-lysine-N-acetyltransferase activity;GO:0034212 peptide N-acetyltransferase ac
7 site of polarized growth

5 endopeptidase regulator activity;GO:0030414 peptidase inhibitor activity
vity;GO:0005088 Ras guanyl-nucleotide exchange factor activity;GO:0017137 Rab GTPase bind

ulfurtransferase activity;GO:0016782 transferase activity, transferring sulfur-containing

vity regulating pathway - multiple species; (sce04138)Autophagy - yeast

3'-flap-structured DNA binding;GO:0070336 flap-structured DNA binding
vity;GO:0016274 protein-arginine N-methyltransferase activity;GO:0016273 arginine N-methy

pendent ATPase activity;GO:0031490 chromatin DNA binding
ansport

100)Metabolic pathways
olic pathways
ce04111)Cell cycle - yeast; (sce04113)Meiosis - yeast

activity;GO:0016763 transferase activity, transferring pentosyl groups;GO:0140101 cataly
specific);GO:0018024 histone-lysine N-methyltransferase activity;GO:0016279 protein-lysin

6 iron-sulfur cluster binding;GO:0051540 metal cluster binding

g;GO:1901363 heterocyclic compound binding

e04070)Phosphatidylinositol signaling system; (sce04145)Phagosome
0:0008320 protein transmembrane transporter activity;GO:0005048 signal sequence binding
00141)C1-unit interconversion, eukaryotes; (sce00670)One carbon pool by folate

5198 structural molecule activity;GO:0003674 molecular_function

ding;GO:0005096 GTPase activator activity

098847 sequence-specific single stranded DNA binding;GO:0042162 telomeric DNA binding
sis - yeast

aining complex binding;GO:0005488 binding
6888 endodeoxyribonuclease activity, producing 5'-phosphomonoesters;GO:0016895 exodeoxyri
nsferase; (sce)N-glycosylation by oligosaccharyltransferase; (sce00510)N-Glycan biosynthe

ansferase activity, transferring hexosyl groups;GO:0016757 transferase activity, transfer
sis - yeast
:0016641 oxidoreductase activity, acting on the CH-NH2 group of donors, oxygen as accepto
198 structural molecule activity;GO:0003674 molecular_function
ce04141)Protein processing in endoplasmic reticulum; (sce04111)Cell cycle - yeast
tivity;GO:0046873 metal ion transmembrane transporter activity;GO:0022890 inorganic catio
3420)Nucleotide excision repair

itin protein ligase binding;GO:0044389 ubiquitin-like protein ligase binding
;GO:0034212 peptide N-acetyltransferase activity;GO:0008080 N-acetyltransferase activity

3676 nucleic acid binding
activity;GO:0009019 tRNA (guanine-N1-)-methyltransferase activity;GO:0016423 tRNA (guanin

GO:0008047 enzyme activator activity

198 structural molecule activity;GO:0003674 molecular_function
roline; (sce00332)Carbapenem biosynthesis; (sce)Proline biosynthesis, glutamate => prolin
13)Meiosis - yeast

8186 RNA-dependent ATPase activity;GO:0003724 RNA helicase activity

ubble DNA binding;GO:0032135 DNA insertion or deletion binding

ctivity;GO:0030332 cyclin binding;GO:0004857 enzyme inhibitor activity
g;GO:1901363 heterocyclic compound binding
ty;GO:0017111 nucleoside-triphosphatase activity
05451 monovalent cation:proton antiporter activity;GO:0022821 potassium ion antiporter ac
;GO:0034212 peptide N-acetyltransferase activity;GO:0008080 N-acetyltransferase activity
tiation factor activity;GO:0017025 TBP-class protein binding;GO:0140223 general transcrip
tryptophan; (M00023)Tryptophan biosynthesis, chorismate => tryptophan; (sce00400)Phenylal
reticulum

5'-phosphomonoesters;GO:0004520 endodeoxyribonuclease activity;GO:0016893 endonuclease ac
ic compound binding;GO:0097159 organic cyclic compound binding
Metabolic pathways
ic compound binding;GO:0097159 organic cyclic compound binding
2-oxoadipate; (M00433)Lysine biosynthesis, 2-oxoglutarate => 2-oxoadipate; (M00030)Lysine
threonine kinase activity;GO:0004672 protein kinase activity

specific);GO:0018024 histone-lysine N-methyltransferase activity;GO:0016279 protein-lysin

0:0008320 protein transmembrane transporter activity;GO:0005048 signal sequence binding
004721 phosphoprotein phosphatase activity;GO:0016791 phosphatase activity
e regulator activity;GO:0051117 ATPase binding

ein complex binding;GO:0044877 protein-containing complex binding
ansferase activity;GO:0003839 gamma-glutamylcyclotransferase activity;GO:0016842 amidine-
e regulator activity;GO:0051087 chaperone binding

phosphatase activity;GO:0004722 protein serine/threonine phosphatase activity;GO:0004721

e pole body;GO:0044450 microtubule organizing center part
cuole

etabolic pathways

3420)Nucleotide excision repair
9 ubiquitin-like protein ligase binding;GO:0019899 enzyme binding
ular adaptor activity;GO:0005515 protein binding
236 serine-type peptidase activity;GO:0017171 serine hydrolase activity
oups;GO:0005198 structural molecule activity;GO:0016740 transferase activity

binding;GO:0004004 ATP-dependent RNA helicase activity;GO:0070180 large ribosomal subuni

ing;GO:0070300 phosphatidic acid binding;GO:1902936 phosphatidylinositol bisphosphate bin
e; (sce04141)Protein processing in endoplasmic reticulum

ator activity;GO:0030695 GTPase regulator activity
reticulum

NA binding, bending;GO:0000217 DNA secondary structure binding
tor activity;GO:0051015 actin filament binding

-anchor biosynthesis; (sce01100)Metabolic pathways
:0003993 acid phosphatase activity;GO:0016791 phosphatase activity

ing;GO:0043168 anion binding
712 transcription coregulator activity;GO:0140110 transcription regulator activity
ing;GO:1902936 phosphatidylinositol bisphosphate binding;GO:1901981 phosphatidylinositol
e protein binding;GO:0005515 protein binding
:0008324 cation transmembrane transporter activity;GO:0015075 ion transmembrane transport

42393 histone binding;GO:0008047 enzyme activator activity

ce04111)Cell cycle - yeast; (sce04113)Meiosis - yeast
e-specific DNA binding;GO:0001003 RNA polymerase III type 2 promoter sequence-specific DN

amma-tubulin binding;GO:0008017 microtubule binding
ne phosphatase activity;GO:0004722 protein serine/threonine phosphatase activity;GO:00047
5198 structural molecule activity;GO:0003674 molecular_function

etic complications; (sce04070)Phosphatidylinositol signaling system; (sce04139)Mitophagy

ogenesis in eukaryotes
y;GO:0015174 basic amino acid transmembrane transporter activity;GO:0015171 amino acid tr

ular adaptor activity;GO:0005515 protein binding
O:0015450 P-P-bond-hydrolysis-driven protein transmembrane transporter activity;GO:000832

kinase regulator activity;GO:0019887 protein kinase regulator activity;GO:0019207 kinase

3 ubiquitin-like protein-specific protease activity;GO:0004222 metalloendopeptidase activ

4 molecular_function

galactose => alpha-D-glucose-1P; (M00632)Galactose degradation, Leloir pathway, galactose

5)N-glycan precursor biosynthesis; (sce00510)N-Glycan biosynthesis

;GO:0034212 peptide N-acetyltransferase activity;GO:0008080 N-acetyltransferase activity

hagy - yeast; (sce04138)Autophagy - yeast

ein complex binding;GO:0044877 protein-containing complex binding

vity;GO:0036080 purine nucleotide-sugar transmembrane transporter activity;GO:0005338 nuc

e protein binding;GO:0005515 protein binding

sis, ethanolamine => PE; (M00092)Phosphatidylethanolamine (PE) biosynthesis, ethanolamine
nsferase; (sce)N-glycosylation by oligosaccharyltransferase; (sce00510)N-Glycan biosynthe
ein complex binding;GO:0008270 zinc ion binding
yl-kinase activity;GO:0051735 GTP-dependent polynucleotide kinase activity;GO:0051732 pol
ding;GO:0008092 cytoskeletal protein binding
sis - yeast

RNA polymerase II-specific;GO:0008270 zinc ion binding;GO:0043565 sequence-specific DNA
08175 tRNA methyltransferase activity;GO:0008171 O-methyltransferase activity
ular adaptor activity;GO:0005515 protein binding

ity;GO:0016426 tRNA (adenine) methyltransferase activity;GO:0008175 tRNA methyltransferas

674 molecular_function
ribosomal small subunit binding;GO:0070628 proteasome binding
674 molecular_function

; (sce)PRPP biosynthesis, ribose 5P => PRPP; (sce00030)Pentose phosphate pathway

ng;GO:0005488 binding
hosphatase activity;GO:0016462 pyrophosphatase activity
e => valine / 2-oxobutanoate => isoleucine; (M00019)Valine/isoleucine biosynthesis, pyruv
2 beta-glucosidase activity;GO:0015926 glucosidase activity
5198 structural molecule activity;GO:0003674 molecular_function
GO:0034458 3'-5' RNA helicase activity;GO:0004004 ATP-dependent RNA helicase activity
amine metabolism; (sce01100)Metabolic pathways
;GO:0004386 helicase activity
733 peptide-lysine-N-acetyltransferase activity;GO:0034212 peptide N-acetyltransferase ac
0019208 phosphatase regulator activity;GO:0030234 enzyme regulator activity

ding;GO:0042803 protein homodimerization activity
single-stranded DNA binding;GO:0003729 mRNA binding
TP-dependent RNA helicase activity;GO:0008186 RNA-dependent ATPase activity

yl-CoA; (sce)Pyruvate oxidation, pyruvate => acetyl-CoA; (sce00020)Citrate cycle (TCA cyc
e activity, acting on L-amino acid peptides;GO:0008233 peptidase activity
plus-end-directed;GO:1990939 ATP-dependent microtubule motor activity;GO:0003777 microtub
minus-end-directed;GO:1990939 ATP-dependent microtubule motor activity;GO:0003777 microtu

674 molecular_function
ic activity;GO:0003674 molecular_function

y;GO:0015214 pyrimidine nucleoside transmembrane transporter activity;GO:0005337 nucleosi
etabolic pathways

gnaling adaptor activity;GO:0032947 protein-containing complex scaffold activity
ity;GO:0008094 DNA-dependent ATPase activity;GO:0003697 single-stranded DNA binding
haride; (M00065)GPI-anchor biosynthesis, core oligosaccharide; (sce00563)Glycosylphosphat

lism; (sce01100)Metabolic pathways

0005365 myo-inositol transmembrane transporter activity;GO:0015166 polyol transmembrane t

ctivity;GO:0034353 RNA pyrophosphohydrolase activity;GO:0016891 endoribonuclease activity

lecular_function
binding;GO:0008092 cytoskeletal protein binding

as acceptor;GO:0016772 transferase activity, transferring phosphorus-containing groups;G

activity;GO:0015171 amino acid transmembrane transporter activity;GO:0046943 carboxylic
ansport
ter activity;GO:0015291 secondary active transmembrane transporter activity;GO:0022804 ac
047 enzyme activator activity;GO:0003712 transcription coregulator activity
ansport
ity;GO:0090482 vitamin transmembrane transporter activity;GO:0015238 drug transmembrane t
ivity;GO:1904680 peptide transmembrane transporter activity;GO:0042887 amide transmembran
3682 chromatin binding;GO:0003690 double-stranded DNA binding
5198 structural molecule activity;GO:0003674 molecular_function
09982 pseudouridine synthase activity;GO:0016866 intramolecular transferase activity

vity;GO:0005459 UDP-galactose transmembrane transporter activity;GO:0015165 pyrimidine nu
inding;GO:0046914 transition metal ion binding

sporter activity;GO:0017016 Ras GTPase binding
;GO:0008821 crossover junction endodeoxyribonuclease activity;GO:0016889 endodeoxyribonuc
is
e04070)Phosphatidylinositol signaling system; (sce01100)Metabolic pathways
me kinetochore;GO:0000780 condensed nuclear chromosome, centromeric region

70)Aminoacyl-tRNA biosynthesis
00083)Fatty acid biosynthesis, elongation; (sce00780)Biotin metabolism

nositol-4-phosphate binding;GO:0005546 phosphatidylinositol-4,5-bisphosphate binding

itin-protein transferase activity;GO:0019787 ubiquitin-like protein transferase activity

hatase activity;GO:0042578 phosphoric ester hydrolase activity

pase activity;GO:0052689 carboxylic ester hydrolase activity
ing;GO:0070064 proline-rich region binding;GO:0003785 actin monomer binding

3676 nucleic acid binding
nding;GO:0032559 adenyl ribonucleotide binding
:0017111 nucleoside-triphosphatase activity
Metabolic pathways

nding;GO:0008092 cytoskeletal protein binding

-anchor biosynthesis
y;GO:0008324 cation transmembrane transporter activity;GO:0015075 ion transmembrane trans

binding, bridging;GO:0060090 molecular adaptor activity
crotubule binding;GO:0015631 tubulin binding

r activity;GO:0030234 enzyme regulator activity;GO:0098772 molecular function regulator

movement of ions, phosphorylative mechanism;GO:0004012 phospholipid-translocating ATPase
tiple species; (sce04138)Autophagy - yeast; (sce04011)MAPK signaling pathway - yeast
pendent ATPase activity;GO:0042623 ATPase activity, coupled
transferase activity, alcohol group as acceptor;GO:0005524 ATP binding

; (sce)PRPP biosynthesis, ribose 5P => PRPP; (sce00030)Pentose phosphate pathway
ic activity;GO:0003674 molecular_function

ing;GO:0043168 anion binding
:0005096 GTPase activator activity;GO:0030695 GTPase regulator activity

tor activity;GO:0003779 actin binding
tivity;GO:0046873 metal ion transmembrane transporter activity;GO:0022890 inorganic catio

lanine, aspartate and glutamate metabolism; (sce01210)2-Oxocarboxylic acid metabolism
itin-like protein transferase activity;GO:0140096 catalytic activity, acting on a protein

thesis, PA => PS => PE; (sce)Phosphatidylethanolamine (PE) biosynthesis, PA => PS => PE;

3712 transcription coregulator activity;GO:0046983 protein dimerization activity

ransferase activity;GO:0016274 protein-arginine N-methyltransferase activity;GO:0016273 a

O:0050136 NADH dehydrogenase (quinone) activity;GO:0003954 NADH dehydrogenase activity
activity;GO:0016788 hydrolase activity, acting on ester bonds
etallopeptidase activity;GO:0004175 endopeptidase activity
ding;GO:0008092 cytoskeletal protein binding
sis - yeast

0001047 core promoter binding;GO:0008135 translation factor activity, RNA binding

P => ADP,ATP; (sce00730)Thiamine metabolism; (M00049)Adenine ribonucleotide biosynthesis,

e01100)Metabolic pathways

0403 Y-form DNA binding;GO:0000400 four-way junction DNA binding
3676 nucleic acid binding

59 ubiquitin-like protein ligase activity;GO:0004842 ubiquitin-protein transferase activi
215 transporter activity;GO:0003674 molecular_function

ansport

none biosynthesis; (sce01110)Biosynthesis of secondary metabolites; (sce01100)Metabolic pa
00182 rDNA binding;GO:0008094 DNA-dependent ATPase activity
0022857 transmembrane transporter activity;GO:0005215 transporter activity
se activity;GO:0004386 helicase activity

yosin II binding;GO:0032036 myosin heavy chain binding
5)N-glycan precursor biosynthesis; (sce00510)N-Glycan biosynthesis
eosome

0032550 purine ribonucleoside binding

O:0001091 RNA polymerase II basal transcription factor binding;GO:0001103 RNA polymerase
e activity;GO:0008418 protein-N-terminal asparagine amidohydrolase activity;GO:0016811 hy
cellular part

3676 nucleic acid binding
vity;GO:0008170 N-methyltransferase activity;GO:0008757 S-adenosylmethionine-dependent me
ein complex binding;GO:0003729 mRNA binding

ine metabolism; (sce00230)Purine metabolism

vity;GO:0005088 Ras guanyl-nucleotide exchange factor activity;GO:0017137 Rab GTPase bind

ity;GO:0016423 tRNA (guanine) methyltransferase activity;GO:0008175 tRNA methyltransferas

ein complex binding;GO:0044877 protein-containing complex binding

0)Proteasome
GO:0031493 nucleosomal histone binding;GO:0043138 3'-5' DNA helicase activity
uitin binding;GO:0038024 cargo receptor activity

lism; (sce04213)Longevity regulating pathway - multiple species; (sce01100)Metabolic path
linositol binding;GO:0015248 sterol transporter activity
NA-binding transcription factor activity;GO:0140110 transcription regulator activity
some

g) kinase activity;GO:0004672 protein kinase activity;GO:0016773 phosphotransferase activ
8186 RNA-dependent ATPase activity;GO:0003724 RNA helicase activity
104 succinate dehydrogenase activity;GO:0016628 oxidoreductase activity, acting on the CH

hagy - yeast
binding;GO:0005488 binding

pendent ATPase activity;GO:0042623 ATPase activity, coupled
RNA binding;GO:0008143 poly(A) binding

;GO:0005488 binding

ctivity;GO:0016889 endodeoxyribonuclease activity, producing 3'-phosphomonoesters;GO:0016
ce01100)Metabolic pathways
ed in UV-damage excision repair;GO:0008821 crossover junction endodeoxyribonuclease activ
binding;GO:0005488 binding

00)Metabolic pathways

pase activity;GO:0052689 carboxylic ester hydrolase activity
; (sce00910)Nitrogen metabolism; (sce00220)Arginine biosynthesis

ome biogenesis in eukaryotes
ivity;GO:0022857 transmembrane transporter activity;GO:0005215 transporter activity

sce01110)Biosynthesis of secondary metabolites
0032550 purine ribonucleoside binding

n binding;GO:0005488 binding
alpha-glucosidase activity;GO:0015926 glucosidase activity

iosynthesis of unsaturated fatty acids; (sce01110)Biosynthesis of secondary metabolites

amine as amido-N-donor;GO:0016879 ligase activity, forming carbon-nitrogen bonds;GO:00168

ornithine; (sce)Ornithine biosynthesis, glutamate => ornithine; (sce00220)Arginine biosy

vity regulating pathway - multiple species; (sce04138)Autophagy - yeast

ding;GO:0032559 adenyl ribonucleotide binding
oups;GO:0016740 transferase activity;GO:0003824 catalytic activity
nsferase; (sce)N-glycosylation by oligosaccharyltransferase; (sce00510)N-Glycan biosynthe

ng;GO:0003779 actin binding;GO:0008092 cytoskeletal protein binding
ator activity;GO:0030695 GTPase regulator activity
004402 histone acetyltransferase activity;GO:0061733 peptide-lysine-N-acetyltransferase a

;GO:0043492 ATPase activity, coupled to movement of substances;GO:0005548 phospholipid tr
binding, bridging;GO:0060090 molecular adaptor activity
GO:0043138 3'-5' DNA helicase activity;GO:0004003 ATP-dependent DNA helicase activity
ic activity;GO:0003674 molecular_function

3676 nucleic acid binding

g;GO:0043167 ion binding
10 kinase inhibitor activity;GO:0019887 protein kinase regulator activity
itin-protein transferase activity;GO:0060090 molecular adaptor activity
regulator activity;GO:0043021 ribonucleoprotein complex binding

16 DNA topoisomerase activity;GO:0016853 isomerase activity

0032550 purine ribonucleoside binding
ome biogenesis in eukaryotes

ansport; (sce04145)Phagosome
PRPP + glutamine => IMP; (sce)Inosine monophosphate biosynthesis, PRPP + glutamine => IMP
13)RNA transport
fic DNA binding;GO:0046914 transition metal ion binding
lecular_function

20)Pyruvate metabolism; (sce01200)Carbon metabolism

ce-specific DNA binding;GO:0000987 proximal promoter sequence-specific DNA binding;GO:000
8186 RNA-dependent ATPase activity;GO:0003724 RNA helicase activity

0:0035184 histone threonine kinase activity;GO:0035173 histone kinase activity
712 transcription coregulator activity;GO:0140110 transcription regulator activity

iosynthesis of secondary metabolites; (sce01100)Metabolic pathways
e01100)Metabolic pathways

40)Spliceosome; (sce03013)RNA transport
se => UDP-glucose; (sce)Nucleotide sugar biosynthesis, glucose => UDP-glucose; (sce00051)
RNA methyltransferase activity;GO:0140101 catalytic activity, acting on a tRNA
y;GO:0004422 hypoxanthine phosphoribosyltransferase activity;GO:0004615 phosphomannomutas

kinase regulator activity;GO:0019887 protein kinase regulator activity;GO:0019207 kinase
rkinson's disease; (hsa04022)cGMP-PKG signaling pathway

ce04111)Cell cycle - yeast
sistance to 5-FU; specifically reduces the incorporation of 5-FU into RNA, without affect
488 binding;GO:0003674 molecular_function
8186 RNA-dependent ATPase activity;GO:0003724 RNA helicase activity
activity;GO:0140097 catalytic activity, acting on DNA

0019787 ubiquitin-like protein transferase activity;GO:0140096 catalytic activity, acting
lecular_function
some

;GO:0043492 ATPase activity, coupled to movement of substances;GO:0005548 phospholipid tr
ding;GO:0008092 cytoskeletal protein binding
00082)Fatty acid biosynthesis, initiation; (sce00061)Fatty acid biosynthesis
binding;GO:0060090 molecular adaptor activity
0032550 purine ribonucleoside binding
0019787 ubiquitin-like protein transferase activity;GO:0003682 chromatin binding

lication; (sce03420)Nucleotide excision repair
2-oxoglutarate => oxaloacetate; (M00009)Citrate cycle (TCA cycle, Krebs cycle); (M00011)

0:0010181 FMN binding;GO:0016655 oxidoreductase activity, acting on NAD(P)H, quinone or s

198 structural molecule activity;GO:0003674 molecular_function
ein complex binding;GO:0044877 protein-containing complex binding

676 nucleic acid binding

e04070)Phosphatidylinositol signaling system; (sce01100)Metabolic pathways
rophosphatase activity;GO:0047429 nucleoside-triphosphate diphosphatase activity;GO:00301

; (sce)PRPP biosynthesis, ribose 5P => PRPP; (sce00030)Pentose phosphate pathway
otein deacetylase activity;GO:0019213 deacetylase activity

0:0043169 cation binding

ing;GO:0032266 phosphatidylinositol-3-phosphate binding;GO:0070300 phosphatidic acid bind

; (sce01110)Biosynthesis of secondary metabolites; (sce01100)Metabolic pathways
g;GO:1901363 heterocyclic compound binding
doreductase activity, acting on NAD(P)H;GO:0050662 coenzyme binding

de binding;GO:0030554 adenyl nucleotide binding
e activity;GO:0036459 thiol-dependent ubiquitinyl hydrolase activity;GO:0004197 cysteine-

674 molecular_function

0019208 phosphatase regulator activity;GO:0030234 enzyme regulator activity

species; (sce04111)Cell cycle - yeast
0:1901981 phosphatidylinositol phosphate binding;GO:0035091 phosphatidylinositol binding
embrane transporter activity;GO:0015291 secondary active transmembrane transporter activi
movement of substances;GO:0043492 ATPase activity, coupled to movement of substances;GO:0

e; (M00074)N-glycan biosynthesis, high-mannose type; (sce00513)Various types of N-glycan
e regulator activity;GO:0098772 molecular function regulator

porter activity;GO:0015234 thiamine transmembrane transporter activity;GO:1901474 azole t

e-stranded RNA-specific ribonuclease activity;GO:0016891 endoribonuclease activity, produ
ular function regulator;GO:0003674 molecular_function

from the whole genome duplication
ce-specific DNA binding;GO:0000976 transcription regulatory region sequence-specific DNA

0 carboxypeptidase activity;GO:0008235 metalloexopeptidase activity

0:0004672 protein kinase activity;GO:0016773 phosphotransferase activity, alcohol group a
lap endonuclease activity;GO:0016888 endodeoxyribonuclease activity, producing 5'-phospho

Metabolic pathways
activity;GO:0140097 catalytic activity, acting on DNA

factor binding;GO:0033613 activating transcription factor binding;GO:0001085 RNA polymer
kinase regulator activity;GO:0019887 protein kinase regulator activity;GO:0019207 kinase
c DNA binding;GO:0043565 sequence-specific DNA binding
:0005092 GDP-dissociation inhibitor activity;GO:0030695 GTPase regulator activity

ydroxypyruvate reductase activity;GO:0030267 glyoxylate reductase (NADP) activity
0032550 purine ribonucleoside binding

de binding;GO:0032561 guanyl ribonucleotide binding

r activity;GO:0004012 phospholipid-translocating ATPase activity;GO:0015605 organophospha
674 molecular_function

specific);GO:0018024 histone-lysine N-methyltransferase activity;GO:0016279 protein-lysin

g;GO:1901363 heterocyclic compound binding

ctivity;GO:0019107 myristoyltransferase activity;GO:0016410 N-acyltransferase activity
te => phenylalanine; (sce)Phenylalanine biosynthesis, chorismate => phenylalanine; (sce00

nding;GO:0008092 cytoskeletal protein binding
RNA polymerase II-specific;GO:0008270 zinc ion binding;GO:0043565 sequence-specific DNA
712 transcription coregulator activity;GO:0140110 transcription regulator activity
sce00520)Amino sugar and nucleotide sugar metabolism; (sce01130)Biosynthesis of antibioti
sis - yeast
reticulum
g;GO:0043021 ribonucleoprotein complex binding

epimerase activity;GO:0016854 racemase and epimerase activity
g;GO:1901363 heterocyclic compound binding

GO:0003674 molecular_function

ce-specific DNA binding;GO:0000987 proximal promoter sequence-specific DNA binding;GO:000
=> GDP,GTP; (sce)Adenine ribonucleotide biosynthesis, IMP => ADP,ATP; (M00002)Glycolysis
ctivity;GO:0031490 chromatin DNA binding;GO:0016747 transferase activity, transferring ac
TP-dependent RNA helicase activity;GO:0008186 RNA-dependent ATPase activity

6 iron-sulfur cluster binding;GO:0051540 metal cluster binding

n, oxaloacetate => 2-oxoglutarate; (sce)Citrate cycle, first carbon oxidation, oxaloaceta

ce-specific DNA binding;GO:0000987 proximal promoter sequence-specific DNA binding;GO:000
:0042123 glucanosyltransferase activity;GO:0016740 transferase activity

-anchor biosynthesis; (sce01100)Metabolic pathways
etabolic pathways
reticulum

=> GDP,GTP; (M00050)Guanine ribonucleotide biosynthesis IMP => GDP,GTP; (sce00230)Purine
e activity;GO:0036459 thiol-dependent ubiquitinyl hydrolase activity;GO:0019783 ubiquitin

ce04111)Cell cycle - yeast; (sce04113)Meiosis - yeast

ding;GO:0008092 cytoskeletal protein binding

;GO:0015151 alpha-glucoside transmembrane transporter activity;GO:0042947 glucoside trans

72)Pancreatic secretion; (hsa04261)Adrenergic signaling in cardiomyocytes

vity;GO:0005088 Ras guanyl-nucleotide exchange factor activity;GO:0017137 Rab GTPase bind
1 ubiquitin-like modifier activating enzyme activity;GO:0016877 ligase activity, forming
19843 rRNA binding;GO:0003723 RNA binding
tive phase, fructose 6P => ribose 5P; (sce)Pentose phosphate pathway, non-oxidative phase
RNA polymerase III type 3 promoter sequence-specific DNA binding;GO:0001156 TFIIIC-class
0008194 UDP-glycosyltransferase activity;GO:0016758 transferase activity, transferring he

5198 structural molecule activity;GO:0003674 molecular_function

ansport; (sce04145)Phagosome
n corepressor activity;GO:0003712 transcription coregulator activity
carboxylic ester hydrolase activity;GO:0003735 structural constituent of ribosome

reticulum; (sce03013)RNA transport

01 catalytic activity, acting on a tRNA;GO:0052689 carboxylic ester hydrolase activity
tivity;GO:0003674 molecular_function
iphosphatase activity;GO:0016462 pyrophosphatase activity
:0016618 hydroxypyruvate reductase activity;GO:0030267 glyoxylate reductase (NADP) activi

NA binding;GO:0003676 nucleic acid binding

ter activity;GO:0046943 carboxylic acid transmembrane transporter activity;GO:0015293 sym
0032550 purine ribonucleoside binding
e-specific DNA binding;GO:0001003 RNA polymerase III type 2 promoter sequence-specific DN
binding;GO:1901363 heterocyclic compound binding
ribonucleoprotein complex binding;GO:0044877 protein-containing complex binding
0019787 ubiquitin-like protein transferase activity;GO:0140096 catalytic activity, acting

-anchor biosynthesis; (sce01100)Metabolic pathways
ic compound binding;GO:0097159 organic cyclic compound binding

g;GO:1901363 heterocyclic compound binding
01130)Biosynthesis of antibiotics; (sce01110)Biosynthesis of secondary metabolites

sis, ethanolamine => PE; (sce00440)Phosphonate and phosphinate metabolism; (M00092)Phospha

004721 phosphoprotein phosphatase activity;GO:0016791 phosphatase activity
0:0001094 TFIID-class transcription factor complex binding;GO:0001091 RNA polymerase II b
0032550 purine ribonucleoside binding

pre-mRNA binding;GO:0003723 RNA binding
ivity;GO:0005381 iron ion transmembrane transporter activity;GO:0072509 divalent inorgani

5)N-glycan precursor biosynthesis; (sce00510)N-Glycan biosynthesis
(sce04138)Autophagy - yeast
; (sce)PRPP biosynthesis, ribose 5P => PRPP; (sce00030)Pentose phosphate pathway

ding;GO:0000149 SNARE binding

omal DNA binding;GO:0031491 nucleosome binding
3676 nucleic acid binding

se II proximal promoter sequence-specific DNA binding;GO:0000987 proximal promoter sequen

0008135 translation factor activity, RNA binding;GO:0005085 guanyl-nucleotide exchange fa

etabolism; (sce01100)Metabolic pathways

ne metabolism; (sce00230)Purine metabolism
0061630 ubiquitin protein ligase activity;GO:0061659 ubiquitin-like protein ligase activi
e regulator activity;GO:0060589 nucleoside-triphosphatase regulator activity
lyoxylate and dicarboxylate metabolism; (sce04213)Longevity regulating pathway - multiple
ised expression analysis, and genome-wide homology searching
sis - yeast
embrane transporter activity;GO:0015291 secondary active transmembrane transporter activi
translation initiation factor activity;GO:0008135 translation factor activity, RNA bindi
p binding;GO:0030619 U1 snRNA binding

y;GO:0072509 divalent inorganic cation transmembrane transporter activity;GO:0046915 tran
0032550 purine ribonucleoside binding

ng;GO:0044877 protein-containing complex binding;GO:0005488 binding
activity;GO:0140097 catalytic activity, acting on DNA

4 molecular_function

4-hydroxybenzoate => ubiquinone; (sce)Ubiquinone biosynthesis, eukaryotes, 4-hydroxybenz

0:0080025 phosphatidylinositol-3,5-bisphosphate binding;GO:0070273 phosphatidylinositol-4
e (isomerizing) activity;GO:0070548 L-glutamine aminotransferase activity;GO:0008483 tran

molecular_function

pole body;GO:0000779 condensed chromosome, centromeric region
e-type endopeptidase activity;GO:0008238 exopeptidase activity
reticulum
binding;GO:0005488 binding
recruiting activity;GO:0001134 transcription regulator recruiting activity;GO:0003713 tra
ein complex binding;GO:0044877 protein-containing complex binding

;GO:0015151 alpha-glucoside transmembrane transporter activity;GO:0042947 glucoside trans
PRPP + glutamine => IMP; (sce)Adenine ribonucleotide biosynthesis, IMP => ADP,ATP; (sce)I

-dependent RNA helicase activity;GO:0016896 exoribonuclease activity, producing 5'-phosph
g;GO:1901363 heterocyclic compound binding
ferase activity;GO:0008168 methyltransferase activity;GO:0016741 transferase activity, tr
prolyl cis-trans isomerase activity;GO:0016859 cis-trans isomerase activity
ine/threonine kinase activity;GO:0004672 protein kinase activity

specific);GO:0018024 histone-lysine N-methyltransferase activity;GO:0016279 protein-lysin

ein complex binding;GO:0044877 protein-containing complex binding

de binding;GO:0032561 guanyl ribonucleotide binding
c DNA binding;GO:0003697 single-stranded DNA binding
g;GO:1901363 heterocyclic compound binding
ce04111)Cell cycle - yeast
043539 protein serine/threonine kinase activator activity;GO:0030295 protein kinase activ
molecular_function

;GO:0000403 Y-form DNA binding;GO:0070336 flap-structured DNA binding
0019843 rRNA binding;GO:0008135 translation factor activity, RNA binding

ansferase activity, transferring amino-acyl groups;GO:0003735 structural constituent of r

ecretion; (hsa04921)Oxytocin signaling pathway
5198 structural molecule activity;GO:0003674 molecular_function

RNA polymerase II-specific;GO:0008270 zinc ion binding;GO:0043565 sequence-specific DNA
vity;GO:0005088 Ras guanyl-nucleotide exchange factor activity;GO:0017137 Rab GTPase bind
0:0004672 protein kinase activity;GO:0016773 phosphotransferase activity, alcohol group a
inine biosynthesis; (sce00250)Alanine, aspartate and glutamate metabolism
n binding;GO:0005488 binding

g;GO:1901363 heterocyclic compound binding

-anchor biosynthesis

hsa04974)Protein digestion and absorption; (hsa04972)Pancreatic secretion
ce-specific DNA binding;GO:0000987 proximal promoter sequence-specific DNA binding;GO:000
0000030 mannosyltransferase activity;GO:0016758 transferase activity, transferring hexosy
RNA polymerase II-specific;GO:0008270 zinc ion binding;GO:0043565 sequence-specific DNA
g transcription factor activity;GO:0008168 methyltransferase activity;GO:0016741 transfer
g;GO:1901363 heterocyclic compound binding
y;GO:0004322 ferroxidase activity;GO:0016724 oxidoreductase activity, oxidizing metal ion
haride; (M00065)GPI-anchor biosynthesis, core oligosaccharide; (sce00563)Glycosylphosphat

e; (M00074)N-glycan biosynthesis, high-mannose type; (sce00513)Various types of N-glycan

etabolic pathways

-oxo-5-alpha-steroid 4-dehydrogenase activity;GO:0033765 steroid dehydrogenase activity,
(sce03013)RNA transport

GO:0008525 phosphatidylcholine transporter activity;GO:0005548 phospholipid transporter a
id transporter activity;GO:0005215 transporter activity
0:0000978 RNA polymerase II proximal promoter sequence-specific DNA binding;GO:0000987 pr
:0008134 transcription factor binding;GO:0043565 sequence-specific DNA binding
n binding;GO:0005488 binding
198 structural molecule activity;GO:0003674 molecular_function

oxoglutarate => 2-aminoadipate => lysine; (sce)Lysine biosynthesis, AAA pathway, 2-oxoglu
0016783 sulfurtransferase activity;GO:0016782 transferase activity, transferring sulfur-c
198 structural molecule activity;GO:0140110 transcription regulator activity
ity;GO:0016435 rRNA (guanine) methyltransferase activity;GO:0008649 rRNA methyltransferas
0005543 phospholipid binding;GO:0008289 lipid binding
RNA polymerase II-specific;GO:0008270 zinc ion binding;GO:0043565 sequence-specific DNA

yl-CoA; (sce)Pyruvate oxidation, pyruvate => acetyl-CoA; (sce00020)Citrate cycle (TCA cyc
sce01130)Biosynthesis of antibiotics
ce-specific DNA binding;GO:0000987 proximal promoter sequence-specific DNA binding;GO:000
ylation-dependent protein binding;GO:0140030 modification-dependent protein binding

6 iron-sulfur cluster binding;GO:0051540 metal cluster binding
yase activity;GO:0016835 carbon-oxygen lyase activity
cle; (sce)Citrate cycle, second carbon oxidation, 2-oxoglutarate => oxaloacetate
110 transcription regulator activity;GO:0003674 molecular_function

NA binding;GO:0046872 metal ion binding

0008135 translation factor activity, RNA binding;GO:0003924 GTPase activity
iphosphatase activity;GO:0016462 pyrophosphatase activity

e04070)Phosphatidylinositol signaling system; (sce01100)Metabolic pathways
itiation factor activity;GO:0001102 RNA polymerase II activating transcription factor bin

ivity;GO:0140098 catalytic activity, acting on RNA;GO:0003824 catalytic activity
0019208 phosphatase regulator activity;GO:0030234 enzyme regulator activity
P => ADP,ATP; (M00049)Adenine ribonucleotide biosynthesis, IMP => ADP,ATP; (sce03008)Ribo

ding;GO:0005525 GTP binding
ing;GO:1902936 phosphatidylinositol bisphosphate binding;GO:1901981 phosphatidylinositol
44390 ubiquitin-like protein conjugating enzyme binding;GO:0061630 ubiquitin protein liga
09982 pseudouridine synthase activity;GO:0016866 intramolecular transferase activity
00)Carbon metabolism; (sce01100)Metabolic pathways

110 transcription regulator activity;GO:0003674 molecular_function
5198 structural molecule activity;GO:0003674 molecular_function

elicase activity;GO:0042623 ATPase activity, coupled
lism; (sce00240)Pyrimidine metabolism; (sce00230)Purine metabolism

sis - yeast

215 transporter activity;GO:0003674 molecular_function
vity;GO:0016434 rRNA (cytosine) methyltransferase activity;GO:0008169 C-methyltransferase

ter activity;GO:0015291 secondary active transmembrane transporter activity;GO:0022804 ac

doplasmic reticulum; (sce00062)Fatty acid elongation; (sce01040)Biosynthesis of unsaturat
hagy - yeast
110 transcription regulator activity;GO:0003674 molecular_function

UMP => UDP/UTP,CDP/CTP; (M00052)Pyrimidine ribonucleotide biosynthesis, UMP => UDP/UTP,C
ecular transducer activity;GO:0003674 molecular_function

y;GO:0042124 1,3-beta-glucanosyltransferase activity;GO:0042123 glucanosyltransferase act

sis, ethanolamine => PE; (sce00440)Phosphonate and phosphinate metabolism; (M00092)Phosph

tivity;GO:0030291 protein serine/threonine kinase inhibitor activity;GO:0004860 protein k

activity;GO:0016788 hydrolase activity, acting on ester bonds

ssing in endoplasmic reticulum

75 RNA polymerase core enzyme binding;GO:0001099 basal RNA polymerase II transcription ma
crotubule binding;GO:0015631 tubulin binding

18)RNA degradation; (sce03013)RNA transport
n binding;GO:0005488 binding
ansport

ribonucleoprotein complex binding;GO:0044877 protein-containing complex binding
pendent ATPase activity;GO:0042623 ATPase activity, coupled
0480)Glutathione metabolism

aminopeptidase activity;GO:0008235 metalloexopeptidase activity
7 site of polarized growth
0005198 structural molecule activity;GO:0003674 molecular_function

entation; (hsa05152)Tuberculosis; (hsa05166)HTLV-I infection
00082)Fatty acid biosynthesis, initiation; (sce00061)Fatty acid biosynthesis

activity;GO:0140097 catalytic activity, acting on DNA

n binding;GO:0005488 binding
0005198 structural molecule activity;GO:0003674 molecular_function
ding;GO:0070717 poly-purine tract binding
674 molecular_function

sporter activity;GO:0017016 Ras GTPase binding
ty;GO:0032041 NAD-dependent histone deacetylase activity (H3-K14 specific);GO:0031078 his

5)N-glycan precursor biosynthesis; (sce00510)N-Glycan biosynthesis
sis - yeast

e regulator activity;GO:0060589 nucleoside-triphosphatase regulator activity

ne metabolism; (sce00230)Purine metabolism
uent of ribosome;GO:0005198 structural molecule activity

0:0015038 glutathione disulfide oxidoreductase activity;GO:0015037 peptide disulfide oxid
pendent ATPase activity;GO:0042623 ATPase activity, coupled

e-specific double-stranded DNA binding;GO:0003690 double-stranded DNA binding
in binding;GO:0005488 binding

sphorylation; (sce01100)Metabolic pathways
ein complex binding;GO:0051082 unfolded protein binding
ng;GO:0048037 cofactor binding

hagy - yeast

> CoA; (M00120)Coenzyme A biosynthesis, pantothenate => CoA; (sce00770)Pantothenate and C
e regulator activity;GO:0031072 heat shock protein binding

disulfide isomerase activity;GO:0016670 oxidoreductase activity, acting on a sulfur group

tidyl)-tetraphosphatases; (GFP)-fusion protein localizes to the vacuole; null mutant is h
cific DNA binding;GO:0001163 RNA polymerase I regulatory region sequence-specific DNA bin
:0008047 enzyme activator activity;GO:0003779 actin binding

110 transcription regulator activity;GO:0003674 molecular_function
dismutase activity;GO:0016721 oxidoreductase activity, acting on superoxide radicals as a
itin-like protein transferase activity;GO:0003682 chromatin binding

e regulator activity;GO:0060589 nucleoside-triphosphatase regulator activity

5198 structural molecule activity;GO:0003674 molecular_function
signaling pathway - yeast

vity;GO:0016434 rRNA (cytosine) methyltransferase activity;GO:0008169 C-methyltransferase
; (sce01130)Biosynthesis of antibiotics; (sce01100)Metabolic pathways
e regulator activity;GO:0008565 protein transporter activity

g;GO:1901363 heterocyclic compound binding

activity, hydrolyzing O-glycosyl compounds;GO:0016798 hydrolase activity, acting on glyco

ator activity;GO:0030695 GTPase regulator activity

3676 nucleic acid binding
alpha kinase activity;GO:0045182 translation regulator activity;GO:0004674 protein serine

binding;GO:0005488 binding

g;GO:0043167 ion binding
A binding;GO:0035613 RNA stem-loop binding

cle; (sce00630)Glyoxylate and dicarboxylate metabolism

RNA polymerase II-specific;GO:0008270 zinc ion binding;GO:0043565 sequence-specific DNA

(H3-K14 specific);GO:0031078 histone deacetylase activity (H3-K14 specific);GO:0017136 N

inding;GO:0070577 lysine-acetylated histone binding;GO:0001091 RNA polymerase II basal tr

00)Metabolic pathways
)Valine, leucine and isoleucine degradation; (sce00340)Histidine metabolism
5)N-glycan precursor biosynthesis; (sce00510)N-Glycan biosynthesis
lication; (sce03420)Nucleotide excision repair
al ion binding;GO:0046872 metal ion binding
e;GO:0008641 ubiquitin-like modifier activating enzyme activity;GO:0016877 ligase activit
peroxiredoxin activity;GO:0004601 peroxidase activity
tivity;GO:0003674 molecular_function
yltransferase activity;GO:0003880 protein C-terminal carboxyl O-methyltransferase activit
ity;GO:0016780 phosphotransferase activity, for other substituted phosphate groups;GO:000

dase activity, acting on L-amino acid peptides;GO:0008233 peptidase activity
ng;GO:0005488 binding
recruiting activity;GO:0001134 transcription regulator recruiting activity;GO:0035064 met
ce04111)Cell cycle - yeast; (sce04113)Meiosis - yeast

51 monovalent cation:proton antiporter activity;GO:0015299 solute:proton antiporter activ
se ion binding;GO:0008238 exopeptidase activity
is
(sce01110)Biosynthesis of secondary metabolites; (sce01100)Metabolic pathways

0:0004672 protein kinase activity;GO:0016773 phosphotransferase activity, alcohol group a

n corepressor activity;GO:0003712 transcription coregulator activity

e activity;GO:0036459 thiol-dependent ubiquitinyl hydrolase activity;GO:0004197 cysteine-

ral constituent of nuclear pore;GO:0005198 structural molecule activity

g;GO:0005515 protein binding;GO:0005488 binding

tes; (sce01100)Metabolic pathways

ty acid degradation; (sce00010)Glycolysis / Gluconeogenesis
g;GO:0043167 ion binding
etallopeptidase activity;GO:0016887 ATPase activity

ein complex binding;GO:0004842 ubiquitin-protein transferase activity
ivity;GO:1904680 peptide transmembrane transporter activity;GO:0042887 amide transmembran

e04070)Phosphatidylinositol signaling system; (sce01100)Metabolic pathways

g;GO:0043167 ion binding
g;GO:1901363 heterocyclic compound binding
GO:0043138 3'-5' DNA helicase activity;GO:0004003 ATP-dependent DNA helicase activity
vity;GO:0071617 lysophospholipid acyltransferase activity;GO:0004806 triglyceride lipase

3676 nucleic acid binding

5198 structural molecule activity;GO:0003674 molecular_function

-oxobutanoate => isoleucine; (M00570)Isoleucine biosynthesis, threonine => 2-oxobutanoate
ferase activity;GO:0008168 methyltransferase activity;GO:0016741 transferase activity, tr
g;GO:1901363 heterocyclic compound binding
; (sce00514)Other types of O-glycan biosynthesis; (sce01100)Metabolic pathways

44390 ubiquitin-like protein conjugating enzyme binding;GO:0019899 enzyme binding

inding;GO:0031267 small GTPase binding
9 4 iron, 4 sulfur cluster binding;GO:0051536 iron-sulfur cluster binding
ng, bridging;GO:0060090 molecular adaptor activity
sis - yeast

44390 ubiquitin-like protein conjugating enzyme binding;GO:0043130 ubiquitin binding

ity;GO:0015606 spermidine transmembrane transporter activity;GO:0000095 S-adenosyl-L-meth

tamine (+ PRPP) => UMP; (M00051)Uridine monophosphate biosynthesis, glutamine (+ PRPP) =>
0000030 mannosyltransferase activity;GO:0016758 transferase activity, transferring hexosy
ty;GO:0005351 carbohydrate:proton symporter activity;GO:0015149 hexose transmembrane tran

e; (sce04141)Protein processing in endoplasmic reticulum

etallopeptidase activity;GO:0004175 endopeptidase activity

sce01130)Biosynthesis of antibiotics
is (ALS)
etabolic pathways

y;GO:0008028 monocarboxylic acid transmembrane transporter activity;GO:0046943 carboxylic
tivity;GO:0033549 MAP kinase phosphatase activity;GO:0004724 magnesium-dependent protein

e activity;GO:0036459 thiol-dependent ubiquitinyl hydrolase activity;GO:0019783 ubiquitin

:0042123 glucanosyltransferase activity;GO:0016740 transferase activity

ne metabolism; (sce00230)Purine metabolism

ing;GO:0043168 anion binding
ic activity;GO:0003674 molecular_function
helicase activity;GO:0004386 helicase activity
sphorylation; (sce01100)Metabolic pathways

lar to C. albicans Wor1p transcription factor that regulates white-opaque switching; over
factor binding;GO:0033613 activating transcription factor binding;GO:0001085 RNA polymer
er activity;GO:0042626 ATPase activity, coupled to transmembrane movement of substances;G
inding;GO:0051880 G-quadruplex DNA binding;GO:0001091 RNA polymerase II basal transcripti

)Metabolic pathways

ity;GO:0008094 DNA-dependent ATPase activity;GO:0004386 helicase activity
olic pathways

inding;GO:0043169 cation binding

O:0004672 protein kinase activity;GO:0016773 phosphotransferase activity, alcohol group a

aminopeptidase activity;GO:0008235 metalloexopeptidase activity

ester hydrolase activity;GO:0016788 hydrolase activity, acting on ester bonds

3676 nucleic acid binding
733 peptide-lysine-N-acetyltransferase activity;GO:0034212 peptide N-acetyltransferase ac

01130)Biosynthesis of antibiotics; (sce01110)Biosynthesis of secondary metabolites
ansport; (sce04138)Autophagy - yeast
specific);GO:0018024 histone-lysine N-methyltransferase activity;GO:0016279 protein-lysin
sis - yeast

ce04111)Cell cycle - yeast; (sce04113)Meiosis - yeast
tivity;GO:0019707 protein-cysteine S-acyltransferase activity;GO:0016417 S-acyltransferas

ribosomal small subunit binding;GO:0043021 ribonucleoprotein complex binding

hway; (hsa04211)Longevity regulating pathway; (hsa04152)AMPK signaling pathway
92 RNA guanylyltransferase activity;GO:0070568 guanylyltransferase activity
transmembrane transporter activity;GO:0005347 ATP transmembrane transporter activity

taurine dioxygenase activity;GO:0016706 oxidoreductase activity, acting on paired donors,
cycle - yeast

binding;GO:0003677 DNA binding
g;GO:1901363 heterocyclic compound binding
;GO:0008047 enzyme activator activity;GO:0051020 GTPase binding
11)Cell cycle - yeast

somerase activity;GO:0003824 catalytic activity
0019787 ubiquitin-like protein transferase activity;GO:0140096 catalytic activity, acting

nding;GO:0000976 transcription regulatory region sequence-specific DNA binding;GO:1990837
tophan metabolism; (sce)Citrate cycle, second carbon oxidation, 2-oxoglutarate => oxaloac

Peroxisome
00)Carbon metabolism; (sce01130)Biosynthesis of antibiotics
processing in endoplasmic reticulum
1 peroxidase activity;GO:0016684 oxidoreductase activity, acting on peroxide as acceptor
e regulator activity;GO:0060589 nucleoside-triphosphatase regulator activity
40)Pyrimidine metabolism

ity;GO:0004721 phosphoprotein phosphatase activity;GO:0016791 phosphatase activity

transmembrane transporter activity;GO:0005347 ATP transmembrane transporter activity
PRPP + glutamine => IMP; (sce)Inosine monophosphate biosynthesis, PRPP + glutamine => IMP

uclease activity;GO:0004518 nuclease activity
osine/serine/threonine phosphatase activity;GO:0004725 protein tyrosine phosphatase activ

arch and sucrose metabolism; (sce01100)Metabolic pathways

215 transporter activity;GO:0003674 molecular_function
ree-carbon compounds; (sce)Glycolysis, core module involving three-carbon compounds; (M00
674 molecular_function

nsporter activity;GO:0015301 anion:anion antiporter activity;GO:0015116 sulfate transmemb

de binding;GO:0032561 guanyl ribonucleotide binding
ivity;GO:0016279 protein-lysine N-methyltransferase activity;GO:0016278 lysine N-methyltr

activity;GO:0052689 carboxylic ester hydrolase activity
d DNA binding;GO:0046914 transition metal ion binding
e-phosphate deaminase activity;GO:0016814 hydrolase activity, acting on carbon-nitrogen (
amaged DNA binding;GO:0004386 helicase activity
3676 nucleic acid binding

sce00590)Arachidonic acid metabolism; (sce)Acylglycerol degradation

0 uracil transmembrane transporter activity;GO:0015391 nucleobase:cation symporter activi

hreonine biosynthesis, aspartate => homoserine => threonine; (sce)Threonine biosynthesis,

on metabolism
e; (sce04141)Protein processing in endoplasmic reticulum
ity;GO:0008251 tRNA-specific adenosine deaminase activity;GO:0004000 adenosine deaminase

141)Protein processing in endoplasmic reticulum

0016891 endoribonuclease activity, producing 5'-phosphomonoesters;GO:0016893 endonuclease
ome biogenesis in eukaryotes

GO:0015036 disulfide oxidoreductase activity;GO:0016667 oxidoreductase activity, acting o
198 structural molecule activity;GO:0003674 molecular_function
PRPP + glutamine => IMP; (sce)Inosine monophosphate biosynthesis, PRPP + glutamine => IMP

ivity;GO:0008276 protein methyltransferase activity;GO:0008168 methyltransferase activity

;GO:0042895 antibiotic transmembrane transporter activity;GO:0008028 monocarboxylic acid
lism; (sce00240)Pyrimidine metabolism
> THF; (sce)Tetrahydrofolate biosynthesis, GTP => THF; (sce00790)Folate biosynthesis

ty, RNA polymerase II-specific;GO:0000978 RNA polymerase II proximal promoter sequence-sp
3676 nucleic acid binding

etabolic pathways

se activity;GO:0004518 nuclease activity
410)Base excision repair; (sce03030)DNA replication
ce04111)Cell cycle - yeast

species; (sce04011)MAPK signaling pathway - yeast
tein localizes to mitochondria; YLR281C is not an essential gene

kinase regulator activity;GO:0019887 protein kinase regulator activity;GO:0019207 kinase
ne metabolism; (sce00230)Purine metabolism

3676 nucleic acid binding
se ion binding;GO:0008238 exopeptidase activity
0003723 RNA binding
0000030 mannosyltransferase activity;GO:0016758 transferase activity, transferring hexosy
(sce01110)Biosynthesis of secondary metabolites; (sce01100)Metabolic pathways
ein complex binding;GO:0008233 peptidase activity

ein complex binding;GO:0008289 lipid binding

y;GO:0015174 basic amino acid transmembrane transporter activity;GO:0015171 amino acid tr

cific DNA binding;GO:0001179 RNA polymerase I transcription factor binding;GO:0001163 RNA
ing;GO:0015631 tubulin binding

215 transporter activity;GO:0003674 molecular_function
sm; (sce01110)Biosynthesis of secondary metabolites; (sce01100)Metabolic pathways
-N(6))-dimethyltransferase activity;GO:0008988 rRNA (adenine-N6-)-methyltransferase activ
itin-like protein transferase activity;GO:0008270 zinc ion binding
elial cells
ivity;GO:0140098 catalytic activity, acting on RNA;GO:0003824 catalytic activity
3676 nucleic acid binding
=> GDP,GTP; (M00050)Guanine ribonucleotide biosynthesis IMP => GDP,GTP; (sce00230)Purine
n binding;GO:0005488 binding

-anchor biosynthesis; (sce01100)Metabolic pathways
activity;GO:0052855 ADP-dependent NAD(P)H-hydrate dehydratase activity;GO:0016836 hydro-l
ctivity;GO:0120108 DNA-3'-diphospho-5'-guanosine diphosphatase;GO:0035312 5'-3' exodeoxyr

sm; (sce01110)Biosynthesis of secondary metabolites; (sce01100)Metabolic pathways
Glycerolipid metabolism; (sce00564)Glycerophospholipid metabolism
inding;GO:0031267 small GTPase binding

iphosphatase activity;GO:0016462 pyrophosphatase activity

RNA polymerase II-specific;GO:0008270 zinc ion binding;GO:0046914 transition metal ion b
ansporter activity;GO:0046872 metal ion binding

tivity;GO:0015562 efflux transmembrane transporter activity;GO:0008519 ammonium transmemb
ein complex binding;GO:0044877 protein-containing complex binding

plus-end-directed;GO:1990939 ATP-dependent microtubule motor activity;GO:0003777 microtub
lism; (sce04213)Longevity regulating pathway - multiple species; (sce01100)Metabolic path
n binding;GO:0005488 binding

0008553 proton-exporting ATPase activity, phosphorylative mechanism;GO:0005388 calcium-tr

3 ATPase activity, coupled;GO:0051082 unfolded protein binding

vity;GO:0015038 glutathione disulfide oxidoreductase activity;GO:0016672 oxidoreductase a
sphorylation; (sce01100)Metabolic pathways
e biogenesis in eukaryotes
GO:0005261 cation channel activity;GO:0022839 ion gated channel activity

42623 ATPase activity, coupled;GO:0016887 ATPase activity

sterol => cholate/chenodeoxycholate; (hsa)Bile acid biosynthesis, cholesterol => cholate/
ein complex binding;GO:0003924 GTPase activity

75 RNA polymerase core enzyme binding;GO:0001099 basal RNA polymerase II transcription ma
on-tagged protein is detected in highly purified mitochondria in high-throughput studies

g;GO:0051082 unfolded protein binding;GO:0015631 tubulin binding

87 DNA-directed DNA polymerase activity;GO:0034061 DNA polymerase activity

of an alternative ER targeting pathway that has partial functional redundancy with the G
0:0003676 nucleic acid binding
70)Aminoacyl-tRNA biosynthesis

sis - yeast
ithine => putrescine; (M00134)Polyamine biosynthesis, arginine => ornithine => putrescine
e-specific DNA binding;GO:0001003 RNA polymerase III type 2 promoter sequence-specific DN

central plaque of spindle pole body;GO:0005816 spindle pole body

pendent ATPase activity;GO:0042623 ATPase activity, coupled

tamine (+ PRPP) => UMP; (M00051)Uridine monophosphate biosynthesis, glutamine (+ PRPP) =>

0:0005488 binding
ty, RNA polymerase II-specific;GO:0001228 DNA-binding transcription activator activity, R

; (sce00514)Other types of O-glycan biosynthesis; (sce01100)Metabolic pathways
008270 zinc ion binding;GO:0046914 transition metal ion binding
ctivity;GO:0016788 hydrolase activity, acting on ester bonds

5198 structural molecule activity;GO:0003674 molecular_function
g;GO:0005488 binding
;GO:0003913 DNA photolyase activity;GO:0016830 carbon-carbon lyase activity

g;GO:0019843 rRNA binding
sis - yeast

ion coactivator activity;GO:0003712 transcription coregulator activity

A binding;GO:0004004 ATP-dependent RNA helicase activity

ne metabolism; (sce00230)Purine metabolism

inding;GO:0031267 small GTPase binding
00)Metabolic pathways
ing;GO:0005488 binding

ylation-dependent protein binding;GO:0140030 modification-dependent protein binding
leosome binding;GO:0003697 single-stranded DNA binding

ctivity;GO:0004222 metalloendopeptidase activity;GO:0008237 metallopeptidase activity
vity;GO:0005085 guanyl-nucleotide exchange factor activity;GO:0017016 Ras GTPase binding
uent of ribosome;GO:0005198 structural molecule activity
110 transcription regulator activity;GO:0003674 molecular_function

sis - yeast

GO:0003723 RNA binding
9 ubiquitin-like protein ligase binding;GO:0019899 enzyme binding

ansport
ity;GO:0015230 FAD transmembrane transporter activity;GO:0005310 dicarboxylic acid transm

tivity;GO:0055106 ubiquitin-protein transferase regulator activity;GO:0043495 protein mem

etallopeptidase activity;GO:0004175 endopeptidase activity

ithine => putrescine; (M00134)Polyamine biosynthesis, arginine => ornithine => putrescine

7108 5'-flap endonuclease activity;GO:0048256 flap endonuclease activity

09982 pseudouridine synthase activity;GO:0016866 intramolecular transferase activity

r activity;GO:0004012 phospholipid-translocating ATPase activity;GO:0015605 organophospha

movement of substances;GO:0043492 ATPase activity, coupled to movement of substances;GO:0
y;GO:0016278 lysine N-methyltransferase activity;GO:0008276 protein methyltransferase act
tes
ce04111)Cell cycle - yeast; (sce04113)Meiosis - yeast

rotein phosphatase regulator activity;GO:0043022 ribosome binding
ular adaptor activity;GO:0005515 protein binding

ne metabolism; (sce00230)Purine metabolism

8 single base insertion or deletion binding;GO:0032137 guanine/thymine mispair binding

ferase activity;GO:0008168 methyltransferase activity;GO:0016741 transferase activity, tr
-anchor biosynthesis; (sce01100)Metabolic pathways
vity;GO:0005085 guanyl-nucleotide exchange factor activity;GO:0017016 Ras GTPase binding

e04070)Phosphatidylinositol signaling system; (sce01100)Metabolic pathways

; (sce)PRPP biosynthesis, ribose 5P => PRPP; (sce00030)Pentose phosphate pathway
nded DNA binding;GO:0003677 DNA binding
pase activity;GO:0052689 carboxylic ester hydrolase activity
yl-CoA; (sce)Pyruvate oxidation, pyruvate => acetyl-CoA; (sce00640)Propanoate metabolism
glycan precursor trimming; (sce00510)N-Glycan biosynthesis

ding;GO:0044877 protein-containing complex binding

004721 phosphoprotein phosphatase activity;GO:0016791 phosphatase activity
tor activity;GO:0051015 actin filament binding
3676 nucleic acid binding

;GO:0005488 binding

0015215 nucleotide transmembrane transporter activity;GO:0051184 cofactor transmembrane t

crotubule binding;GO:0015631 tubulin binding
pre-mRNA binding;GO:0003723 RNA binding

inding;GO:0008094 DNA-dependent ATPase activity

)Autophagy - yeast; (sce01100)Metabolic pathways

)Metabolic pathways
GO:0015440 peptide-transporting ATPase activity;GO:0033220 amide-transporting ATPase acti

5198 structural molecule activity;GO:0003674 molecular_function

kinase regulator activity;GO:0019887 protein kinase regulator activity;GO:0019207 kinase
ctase activity, acting on a sulfur group of donors;GO:0016209 antioxidant activity

kinase activator activity;GO:0016538 cyclin-dependent protein serine/threonine kinase reg
transmembrane transporter activity;GO:0005347 ATP transmembrane transporter activity
ornithine; (sce)Ornithine biosynthesis, glutamate => ornithine; (sce00220)Arginine biosy
ansport; (sce04138)Autophagy - yeast
lism; (sce04213)Longevity regulating pathway - multiple species; (sce01100)Metabolic path
ein complex binding;GO:0016810 hydrolase activity, acting on carbon-nitrogen (but not pep

0:0017150 tRNA dihydrouridine synthase activity;GO:0016627 oxidoreductase activity, actin

1630 ubiquitin protein ligase activity;GO:0061659 ubiquitin-like protein ligase activity

ein complex binding;GO:0044877 protein-containing complex binding

6791 phosphatase activity;GO:0042578 phosphoric ester hydrolase activity
ding;GO:0070717 poly-purine tract binding
5)N-glycan precursor biosynthesis; (sce00510)N-Glycan biosynthesis
0:0004672 protein kinase activity;GO:0016773 phosphotransferase activity, alcohol group a

crotubule binding;GO:0015631 tubulin binding
tein localizes to the endoplasmic reticulum; YDL121C is not an essential protein

activity;GO:0003729 mRNA binding

791 phosphatase activity;GO:0042578 phosphoric ester hydrolase activity
itor activity;GO:0004857 enzyme inhibitor activity;GO:0030234 enzyme regulator activity
19 lipid transporter activity;GO:0005215 transporter activity
ein complex binding;GO:0044877 protein-containing complex binding

0:0004672 protein kinase activity;GO:0016773 phosphotransferase activity, alcohol group a

;GO:0051020 GTPase binding;GO:0098772 molecular function regulator

arch and sucrose metabolism; (sce01100)Metabolic pathways

hionine salvage pathway; (sce00270)Cysteine and methionine metabolism
etabolic pathways
ide excision repair

ansport

ne metabolism; (sce00230)Purine metabolism

RNA polymerase II-specific;GO:0008270 zinc ion binding;GO:0043565 sequence-specific DNA
y;GO:0016278 lysine N-methyltransferase activity;GO:0008276 protein methyltransferase act

n binding;GO:0005488 binding

5198 structural molecule activity;GO:0003674 molecular_function
13)RNA transport

cle - yeast; (sce04113)Meiosis - yeast

tiation factor activity;GO:0000182 rDNA binding;GO:0140223 general transcription initiati

sporter activity;GO:0017016 Ras GTPase binding
binding;GO:0033218 amide binding
mbrane transporter activity;GO:0008320 protein transmembrane transporter activity;GO:1904
688 preribosome, small subunit precursor
ce-specific DNA binding;GO:0000987 proximal promoter sequence-specific DNA binding;GO:000
ne metabolism; (sce00230)Purine metabolism

some
sce00561)Glycerolipid metabolism; (sce00680)Methane metabolism

:0070004 cysteine-type exopeptidase activity;GO:1990380 Lys48-specific deubiquitinase act
ic activity;GO:0003674 molecular_function
3676 nucleic acid binding

pendent ATPase activity;GO:0042623 ATPase activity, coupled
ding, bending;GO:1990837 sequence-specific double-stranded DNA binding

sis - yeast

70)Aminoacyl-tRNA biosynthesis

e-specific DNA binding;GO:0001003 RNA polymerase III type 2 promoter sequence-specific DN

ce-specific DNA binding;GO:0000987 proximal promoter sequence-specific DNA binding;GO:000

198 structural molecule activity;GO:0003674 molecular_function
92 endoribonuclease activity, producing 3'-phosphomonoesters;GO:0004549 tRNA-specific rib
pase activity;GO:0052689 carboxylic ester hydrolase activity
pendent ATPase activity;GO:0031490 chromatin DNA binding

ethane metabolism; (sce00620)Pyruvate metabolism
holipase activity;GO:0016298 lipase activity

oups;GO:0016740 transferase activity;GO:0003824 catalytic activity

ize to the cell periphery
ty, RNA polymerase II-specific;GO:0000987 proximal promoter sequence-specific DNA binding

-acetylated histone binding;GO:0140033 acetylation-dependent protein binding
glycan precursor trimming; (sce)N-glycan biosynthesis, high-mannose type
y;GO:0070567 cytidylyltransferase activity;GO:0016779 nucleotidyltransferase activity
sm

0:0015450 P-P-bond-hydrolysis-driven protein transmembrane transporter activity;GO:000832
)Metabolic pathways
PRPP + glutamine => IMP; (sce)Inosine monophosphate biosynthesis, PRPP + glutamine => IMP
GO:0000293 ferric-chelate reductase activity;GO:0016723 oxidoreductase activity, oxidizin

itor activity;GO:0004864 protein phosphatase inhibitor activity;GO:0019212 phosphatase in
GO:0016972 thiol oxidase activity;GO:0016670 oxidoreductase activity, acting on a sulfur

ity;GO:0046943 carboxylic acid transmembrane transporter activity;GO:0008514 organic anio
674 molecular_function
sphorylation; (sce01100)Metabolic pathways
;GO:0003755 peptidyl-prolyl cis-trans isomerase activity

vity;GO:0005085 guanyl-nucleotide exchange factor activity;GO:0051020 GTPase binding

0019212 phosphatase inhibitor activity;GO:0019888 protein phosphatase regulator activity

ity;GO:0022857 transmembrane transporter activity;GO:0005215 transporter activity

01 catalytic activity, acting on a tRNA;GO:0052689 carboxylic ester hydrolase activity
n binding;GO:0005488 binding

; (sce00514)Other types of O-glycan biosynthesis; (sce01100)Metabolic pathways

pendent ATPase activity;GO:0042623 ATPase activity, coupled

binding;GO:0033218 amide binding
de binding;GO:0032561 guanyl ribonucleotide binding

-anchor biosynthesis; (sce00510)N-Glycan biosynthesis; (sce01100)Metabolic pathways

n binding;GO:0008092 cytoskeletal protein binding
activity, hydrolyzing O-glycosyl compounds;GO:0016798 hydrolase activity, acting on glyco
e-specific double-stranded DNA binding;GO:0003690 double-stranded DNA binding
sporter activity;GO:0017016 Ras GTPase binding
6788 hydrolase activity, acting on ester bonds;GO:0016787 hydrolase activity
ce04111)Cell cycle - yeast

:0022857 transmembrane transporter activity;GO:0005215 transporter activity

vity;GO:0005085 guanyl-nucleotide exchange factor activity;GO:0051020 GTPase binding
FP)-fusion protein localizes to mitochondria and is induced in response to the DNA-damagi

ein complex binding;GO:0044877 protein-containing complex binding

)Metabolic pathways
g;GO:1901363 heterocyclic compound binding
lecular_function
;GO:0016758 transferase activity, transferring hexosyl groups;GO:0016757 transferase acti
:0017111 nucleoside-triphosphatase activity

serine; (sce)Serine biosynthesis, glycerate-3P => serine; (sce00680)Methane metabolism
sferase activity, transferring acyl groups other than amino-acyl groups;GO:0016746 transf
(sce03013)RNA transport
ic activity;GO:0003674 molecular_function
GO:1901265 nucleoside phosphate binding

g;GO:1901363 heterocyclic compound binding
ornithine; (sce)Ornithine biosynthesis, glutamate => ornithine; (sce00220)Arginine biosy
nsporter activity;GO:0015301 anion:anion antiporter activity;GO:0015116 sulfate transmemb

5198 structural molecule activity;GO:0003674 molecular_function

te + erythrose-4P => chorismate; (sce)Shikimate pathway, phosphoenolpyruvate + erythrose-
005048 signal sequence binding;GO:0017056 structural constituent of nuclear pore
ivity;GO:0004672 protein kinase activity;GO:0016773 phosphotransferase activity, alcohol

reticulum
rase activity;GO:0016274 protein-arginine N-methyltransferase activity;GO:0016273 arginin
rsions; (sce00561)Glycerolipid metabolism; (sce00010)Glycolysis / Gluconeogenesis

0019787 ubiquitin-like protein transferase activity;GO:0140096 catalytic activity, acting
de binding;GO:0032561 guanyl ribonucleotide binding
e protein binding;GO:0005515 protein binding
ng;GO:0005488 binding

dase activity, acting on L-amino acid peptides;GO:0008233 peptidase activity

09 kinase activator activity;GO:0019887 protein kinase regulator activity

e; (sce04141)Protein processing in endoplasmic reticulum

reticulum
; (sce00514)Other types of O-glycan biosynthesis; (sce01100)Metabolic pathways
molecular_function

O:0015238 drug transmembrane transporter activity;GO:0022857 transmembrane transporter ac
A replication; (sce03420)Nucleotide excision repair
20)Pyruvate metabolism; (sce01200)Carbon metabolism

O:0008175 tRNA methyltransferase activity;GO:0008173 RNA methyltransferase activity

:0008536 Ran GTPase binding;GO:0140142 nucleocytoplasmic carrier activity
ce03013)RNA transport
hagy - yeast; (sce04113)Meiosis - yeast
osphatase activity;GO:0016462 pyrophosphatase activity
me kinetochore;GO:0000780 condensed nuclear chromosome, centromeric region

(sce03013)RNA transport

endoplasmic reticulum; diploid deletion strain has high budding index

in binding;GO:0005488 binding
in processing in endoplasmic reticulum
n binding;GO:0005488 binding
e biogenesis in eukaryotes
lism; (sce00230)Purine metabolism; (sce01100)Metabolic pathways

5'-UTR binding;GO:0036002 pre-mRNA binding

›tein localizes to mitochondria

;GO:0008252 nucleotidase activity;GO:0016791 phosphatase activity
O:0004672 protein kinase activity;GO:0016773 phosphotransferase activity, alcohol group a

ity;GO:0004721 phosphoprotein phosphatase activity;GO:0016791 phosphatase activity
; (sce00460)Cyanoamino acid metabolism; (sce00480)Glutathione metabolism
004721 phosphoprotein phosphatase activity;GO:0016791 phosphatase activity

tivity;GO:0003824 catalytic activity

phase, glucose 6P => ribulose 5P; (sce)Pentose phosphate pathway, oxidative phase, gluco
emase and epimerase activity, acting on carbohydrates and derivatives;GO:0016854 racemase

ne metabolism; (sce00230)Purine metabolism
27)GABA (gamma-Aminobutyrate) shunt; (sce00650)Butanoate metabolism
oserine binding;GO:0099122 RNA polymerase II C-terminal domain binding;GO:0050815 phospho
rsions; (sce00051)Fructose and mannose metabolism; (sce01100)Metabolic pathways
:0022857 transmembrane transporter activity;GO:0005215 transporter activity
e regulator activity;GO:0031072 heat shock protein binding
16864 intramolecular oxidoreductase activity, transposing S-S bonds;GO:0015035 protein di
g;GO:1901363 heterocyclic compound binding
ecular transducer activity;GO:0003674 molecular_function

itin-like protein transferase activity;GO:0140096 catalytic activity, acting on a protein
O:0004672 protein kinase activity;GO:0016773 phosphotransferase activity, alcohol group a
ein complex binding;GO:0003924 GTPase activity
ansporter activity;GO:0003674 molecular_function

005048 signal sequence binding;GO:0008565 protein transporter activity
yl-CoA; (sce)Pyruvate oxidation, pyruvate => acetyl-CoA; (sce00640)Propanoate metabolism

binding;GO:0003677 DNA binding

03674 molecular_function

488 binding;GO:0003674 molecular_function
anded DNA binding;GO:0140097 catalytic activity, acting on DNA
ce04111)Cell cycle - yeast; (sce04113)Meiosis - yeast
3676 nucleic acid binding
A replication; (sce03420)Nucleotide excision repair

nding;GO:0044877 protein-containing complex binding

vity;GO:0004467 long-chain fatty acid-CoA ligase activity;GO:0015645 fatty acid ligase ac

3285 monocarboxylic acid-transporting ATPase activity;GO:0033284 carboxylic acid-transpor

0000049 tRNA binding;GO:0008135 translation factor activity, RNA binding
e activity;GO:0036459 thiol-dependent ubiquitinyl hydrolase activity;GO:0043022 ribosome
pendent ATPase activity;GO:0042623 ATPase activity, coupled

sis - yeast
sm
y;GO:0016278 lysine N-methyltransferase activity;GO:0008276 protein methyltransferase act
g;GO:0043167 ion binding

amin B6 binding;GO:0019842 vitamin binding
ce04111)Cell cycle - yeast; (sce04113)Meiosis - yeast
tein localizes to the vacuole
1630 ubiquitin protein ligase activity;GO:0061659 ubiquitin-like protein ligase activity
etabolism; (sce01100)Metabolic pathways

uctase activity, acting on peroxide as acceptor;GO:0016209 antioxidant activity
ce-specific DNA binding;GO:0000976 transcription regulatory region sequence-specific DNA

ne metabolism; (sce00230)Purine metabolism

O:0022857 transmembrane transporter activity;GO:0005215 transporter activity

O:0015038 glutathione disulfide oxidoreductase activity;GO:0015037 peptide disulfide oxid
abolic pathways
215 transporter activity;GO:0003674 molecular_function

ydroxyl-kinase activity;GO:0046403 polynucleotide 3'-phosphatase activity;GO:0051733 poly
ce03040)Spliceosome
atory element binding;GO:0030371 translation repressor activity;GO:0090079 translation re
;GO:0051020 GTPase binding;GO:0098772 molecular function regulator
8186 RNA-dependent ATPase activity;GO:0003724 RNA helicase activity
g;GO:0043167 ion binding
y;GO:0016278 lysine N-methyltransferase activity;GO:0008276 protein methyltransferase act

RNA polymerase II transcription factor binding;GO:0003713 transcription coactivator activ
n binding;GO:0005488 binding
O:1901981 phosphatidylinositol phosphate binding;GO:0008565 protein transporter activity
733 peptide-lysine-N-acetyltransferase activity;GO:0034212 peptide N-acetyltransferase ac

amin B6 binding;GO:0019842 vitamin binding

:0048037 cofactor binding
osphatase activity;GO:0016462 pyrophosphatase activity
ce-specific DNA binding;GO:0000987 proximal promoter sequence-specific DNA binding;GO:000

etallopeptidase activity;GO:0004175 endopeptidase activity
0022843 voltage-gated cation channel activity;GO:0005262 calcium channel activity

ity;GO:0008094 DNA-dependent ATPase activity;GO:0042623 ATPase activity, coupled
e => UDP-galactose; (M00554)Nucleotide sugar biosynthesis, galactose => UDP-galactose; (s
ular adaptor activity;GO:0005515 protein binding

:0042123 glucanosyltransferase activity;GO:0016740 transferase activity

ine metabolism; (sce00230)Purine metabolism

some

e; (sce04141)Protein processing in endoplasmic reticulum
00)Metabolic pathways
0008017 microtubule binding;GO:0015631 tubulin binding

sphorylation; (sce01100)Metabolic pathways

etic complications; (sce04144)Endocytosis; (sce04011)MAPK signaling pathway - yeast
0480)Glutathione metabolism

ce01100)Metabolic pathways

nologous recombination; (sce03430)Mismatch repair

movement of ions, phosphorylative mechanism;GO:0042625 ATPase coupled ion transmembrane t
0120013 intermembrane lipid transfer activity;GO:0015248 sterol transporter activity
e regulator activity;GO:0060589 nucleoside-triphosphatase regulator activity

6788 hydrolase activity, acting on ester bonds;GO:0016787 hydrolase activity

specific DNA binding;GO:0003677 DNA binding

ce-specific DNA binding;GO:0000987 proximal promoter sequence-specific DNA binding;GO:000

pendent ATPase activity;GO:0000978 RNA polymerase II proximal promoter sequence-specific
2-oxoglutarate => oxaloacetate; (M00009)Citrate cycle (TCA cycle, Krebs cycle); (M00011)

pase activity;GO:0052689 carboxylic ester hydrolase activity
inding;GO:0008092 cytoskeletal protein binding
g;GO:1901363 heterocyclic compound binding

0019787 ubiquitin-like protein transferase activity;GO:0140096 catalytic activity, acting
rsions; (sce00051)Fructose and mannose metabolism; (sce01100)Metabolic pathways
prolyl cis-trans isomerase activity;GO:0016859 cis-trans isomerase activity
prolyl cis-trans isomerase activity;GO:0016859 cis-trans isomerase activity

sis - yeast

otein deacetylase activity;GO:0019213 deacetylase activity

e activity;GO:0036459 thiol-dependent ubiquitinyl hydrolase activity;GO:0004197 cysteine-
e01100)Metabolic pathways

0008135 translation factor activity, RNA binding;GO:0003723 RNA binding

e04070)Phosphatidylinositol signaling system; (sce01100)Metabolic pathways
e activity;GO:0016773 phosphotransferase activity, alcohol group as acceptor

orter activity;GO:0015291 secondary active transmembrane transporter activity
ein complex binding;GO:0031267 small GTPase binding
ate => H2S; (sce)Assimilatory sulfate reduction, sulfate => H2S; (sce00920)Sulfur metabol

674 molecular_function
nding;GO:0046914 transition metal ion binding
ce04111)Cell cycle - yeast; (sce04113)Meiosis - yeast

ce-specific DNA binding;GO:0000987 proximal promoter sequence-specific DNA binding;GO:000
ator activity;GO:0030695 GTPase regulator activity

y;GO:0008324 cation transmembrane transporter activity;GO:0015075 ion transmembrane trans
59 ubiquitin-like protein ligase activity;GO:0004842 ubiquitin-protein transferase activi

ed DNA binding;GO:0019787 ubiquitin-like protein transferase activity
itin-like protein transferase activity;GO:0140096 catalytic activity, acting on a protein

Sm-like protein family complex;GO:0005681 spliceosomal complex
> CoA; (M00120)Coenzyme A biosynthesis, pantothenate => CoA; (sce00770)Pantothenate and C

phosphatase activity;GO:0043175 RNA polymerase core enzyme binding;GO:0004722 protein ser
binding;GO:0030246 carbohydrate binding
ce04111)Cell cycle - yeast; (sce04113)Meiosis - yeast
pecific DNA binding;GO:0001046 core promoter sequence-specific DNA binding;GO:0001047 cor

histone kinase activity;GO:0004674 protein serine/threonine kinase activity
minus-end-directed;GO:0008574 ATP-dependent microtubule motor activity, plus-end-directed

nthesis of antibiotics; (sce01110)Biosynthesis of secondary metabolites

otein deacetylase activity;GO:0019213 deacetylase activity

ospholipid binding;GO:0008289 lipid binding
:0008139 nuclear localization sequence binding;GO:0140142 nucleocytoplasmic carrier activ

;GO:0034212 peptide N-acetyltransferase activity;GO:0008080 N-acetyltransferase activity
crotubule binding;GO:0015631 tubulin binding

ce04111)Cell cycle - yeast

0003688 DNA replication origin binding;GO:0008301 DNA binding, bending
3676 nucleic acid binding

se => UDP-glucose; (sce)Nucleotide sugar biosynthesis, glucose => UDP-glucose; (sce00040)

hosphatase activity;GO:0016462 pyrophosphatase activity
sphorylation; (sce01100)Metabolic pathways
olic pathways

0016410 N-acyltransferase activity;GO:0016747 transferase activity, transferring acyl gro

GO:0034458 3'-5' RNA helicase activity;GO:0004004 ATP-dependent RNA helicase activity

09982 pseudouridine synthase activity;GO:0016866 intramolecular transferase activity

e transporter activity;GO:0015191 L-methionine transmembrane transporter activity;GO:1901
vity;GO:0005088 Ras guanyl-nucleotide exchange factor activity;GO:0017137 Rab GTPase bind
0019787 ubiquitin-like protein transferase activity;GO:0140096 catalytic activity, acting
A replication; (sce03420)Nucleotide excision repair

GO:0016811 hydrolase activity, acting on carbon-nitrogen (but not peptide) bonds, in line

ne metabolism; (sce00230)Purine metabolism

ine metabolism; (sce00230)Purine metabolism
GO:0016747 transferase activity, transferring acyl groups other than amino-acyl groups;GO

id transporter activity;GO:0005215 transporter activity
O:0004672 protein kinase activity;GO:0016773 phosphotransferase activity, alcohol group a
etabolic pathways
g;GO:0003723 RNA binding

ivity;GO:0005302 L-tyrosine transmembrane transporter activity;GO:0015186 L-glutamine tra
0019787 ubiquitin-like protein transferase activity;GO:0140096 catalytic activity, acting
activity;GO:0015171 amino acid transmembrane transporter activity;GO:0046943 carboxylic

ng;GO:0032559 adenyl ribonucleotide binding
de binding;GO:0032561 guanyl ribonucleotide binding

uence-specific DNA binding;GO:0003677 DNA binding
g;GO:1901363 heterocyclic compound binding
sporter activity;GO:0017016 Ras GTPase binding

lap endonuclease activity;GO:0016888 endodeoxyribonuclease activity, producing 5'-phospho

e01100)Metabolic pathways

; data; partially overlaps dubious ORF YBR056C-B; YBR056W-A has a paralog, YDR034W-B, that

ein complex binding;GO:0044877 protein-containing complex binding

ular adaptor activity;GO:0005515 protein binding
urine metabolism

olic pathways
activity;GO:0008514 organic anion transmembrane transporter activity;GO:0005342 organic a
te => phenylalanine; (sce)Phenylalanine biosynthesis, chorismate => phenylalanine; (sce00

hagy - yeast; (sce04011)MAPK signaling pathway - yeast
ce04141)Protein processing in endoplasmic reticulum
0101 catalytic activity, acting on a tRNA;GO:0140098 catalytic activity, acting on RNA
00230)Purine metabolism

5'-exoribonuclease activity;GO:0016896 exoribonuclease activity, producing 5'-phosphomono

ity;GO:0015606 spermidine transmembrane transporter activity;GO:0015204 urea transmembran
pendent ATPase activity;GO:0042623 ATPase activity, coupled
olic pathways
sis - yeast

g;GO:1901363 heterocyclic compound binding

00)Metabolic pathways

embrane;GO:0044232 organelle membrane contact site

ng pathway

ne metabolism; (sce00230)Purine metabolism

RNA polymerase II-specific;GO:0008270 zinc ion binding;GO:0043565 sequence-specific DNA

;GO:1901682 sulfur compound transmembrane transporter activity;GO:0015103 inorganic anion

hosphatase activity;GO:0016462 pyrophosphatase activity
ty;GO:0016755 transferase activity, transferring amino-acyl groups;GO:0016746 transferase

g;GO:1901363 heterocyclic compound binding

tivity;GO:0046873 metal ion transmembrane transporter activity;GO:0022890 inorganic catio

ferring) activity;GO:0016624 oxidoreductase activity, acting on the aldehyde or oxo group
ty, RNA polymerase II-specific;GO:0000978 RNA polymerase II proximal promoter sequence-sp
vity;GO:0005313 L-glutamate transmembrane transporter activity;GO:0015292 uniporter activ
ansporter activity;GO:0003674 molecular_function
6788 hydrolase activity, acting on ester bonds;GO:0016787 hydrolase activity

hiamine metabolism; (sce04111)Cell cycle - yeast
activity;GO:0140097 catalytic activity, acting on DNA

PRPP + glutamine => IMP; (sce)Inosine monophosphate biosynthesis, PRPP + glutamine => IMP

specific);GO:0018024 histone-lysine N-methyltransferase activity;GO:0016279 protein-lysin

;GO:0015175 neutral amino acid transmembrane transporter activity;GO:0015179 L-amino acid
ity;GO:0052690 trichloro-p-hydroquinone reductive dehalogenase activity;GO:0003959 NADPH
promoter binding;GO:0003714 transcription corepressor activity
440)Homologous recombination

ic compound binding;GO:0097159 organic cyclic compound binding

5)N-glycan precursor biosynthesis; (sce00510)N-Glycan biosynthesis

ubiquitin-protein transferase activity;GO:0019787 ubiquitin-like protein transferase acti

reabsorption; (hsa04722)Neurotrophin signaling pathway

hiamine metabolism; (sce01100)Metabolic pathways
tivity;GO:0005385 zinc ion transmembrane transporter activity;GO:0072509 divalent inorgan
kinase regulator activity;GO:0019887 protein kinase regulator activity;GO:0019207 kinase
NA end binding;GO:0032135 DNA insertion or deletion binding

helicase activity;GO:0004386 helicase activity

iphosphatase activity;GO:0016462 pyrophosphatase activity

0:0004672 protein kinase activity;GO:0016773 phosphotransferase activity, alcohol group a
;GO:0004559 alpha-mannosidase activity;GO:0015923 mannosidase activity

reticulum

lism; (sce01100)Metabolic pathways
GO:0043140 ATP-dependent 3'-5' DNA helicase activity;GO:0043139 5'-3' DNA helicase activi
ansport

098847 sequence-specific single stranded DNA binding;GO:0042162 telomeric DNA binding
0032550 purine ribonucleoside binding
9 ubiquitin-like protein ligase binding;GO:0019899 enzyme binding

733 peptide-lysine-N-acetyltransferase activity;GO:0034212 peptide N-acetyltransferase ac
ce01100)Metabolic pathways

sis - yeast

O:0070273 phosphatidylinositol-4-phosphate binding;GO:1901981 phosphatidylinositol phosph
sphorylation; (sce01100)Metabolic pathways

<i>Ashbya gossypii</i> and other related yeasts; SWAT-GFP and mCherry fusion proteins lc
0008135 translation factor activity, RNA binding;GO:0005085 guanyl-nucleotide exchange fa
n; (hsa02010)ABC transporters; (hsa04976)Bile secretion

e regulator activity;GO:0098772 molecular function regulator
ity;GO:0046943 carboxylic acid transmembrane transporter activity;GO:0008514 organic anio
se => UDP-glucose; (sce)Nucleotide sugar biosynthesis, glucose => UDP-glucose; (sce00051)

042578 phosphoric ester hydrolase activity;GO:0016788 hydrolase activity, acting on ester
etabolic pathways
198 structural molecule activity;GO:0003674 molecular_function
sitol-4,5-bisphosphate binding;GO:1902936 phosphatidylinositol bisphosphate binding
e04011)MAPK signaling pathway - yeast

ase activity;GO:1990189 peptide-serine-N-acetyltransferase activity;GO:0004596 peptide al
ic activity;GO:0003674 molecular_function

hosphatase activity;GO:0016462 pyrophosphatase activity

ular adaptor activity;GO:0005515 protein binding
e regulator activity;GO:0098772 molecular function regulator
198 structural molecule activity;GO:0003674 molecular_function

etabolic pathways
itin-protein transferase activity;GO:0060090 molecular adaptor activity
e regulator activity;GO:0098772 molecular function regulator
cle - yeast; (sce04113)Meiosis - yeast

e activity;GO:0036459 thiol-dependent ubiquitinyl hydrolase activity;GO:0004197 cysteine-

acyltransferase activity;GO:0016407 acetyltransferase activity

PRPP + glutamine => IMP; (sce)Inosine monophosphate biosynthesis, PRPP + glutamine => IMP
RNA polymerase II-specific;GO:0043565 sequence-specific DNA binding;GO:0003700 DNA-bindi

ty;GO:0015175 neutral amino acid transmembrane transporter activity;GO:0015179 L-amino ac

some

r activity;GO:0004012 phospholipid-translocating ATPase activity;GO:0015605 organophospha
(sce03013)RNA transport
ansport
vity;GO:0005088 Ras guanyl-nucleotide exchange factor activity;GO:0017048 Rho GTPase bind
gen (but not peptide) bonds;GO:0016787 hydrolase activity;GO:0003824 catalytic activity
kinase activity;GO:0004693 cyclin-dependent protein serine/threonine kinase activity;GO:0
ty;GO:0008757 S-adenosylmethionine-dependent methyltransferase activity;GO:0008168 methyl
sce01130)Biosynthesis of antibiotics; (sce01110)Biosynthesis of secondary metabolites
hreonine biosynthesis, aspartate => homoserine => threonine; (sce)Threonine biosynthesis,

yl cis-trans isomerase activity;GO:0016859 cis-trans isomerase activity

2-oxoglutarate => oxaloacetate; (M00009)Citrate cycle (TCA cycle, Krebs cycle); (M00011)
sphorylation; (sce01100)Metabolic pathways

ce04111)Cell cycle - yeast; (sce04113)Meiosis - yeast
ity;GO:0016433 rRNA (adenine) methyltransferase activity;GO:0008649 rRNA methyltransferas
sa04919)Thyroid hormone signaling pathway; (hsa05203)Viral carcinogenesis

3420)Nucleotide excision repair
etabolism; (sce01100)Metabolic pathways
3676 nucleic acid binding

5198 structural molecule activity;GO:0003674 molecular_function
0:0043169 cation binding

folded protein binding;GO:0005215 transporter activity

ein complex binding;GO:0044877 protein-containing complex binding
reticulum

ivity;GO:0072542 protein phosphatase activator activity;GO:0019211 phosphatase activator

92 endoribonuclease activity, producing 3'-phosphomonoesters;GO:0004549 tRNA-specific rib
ein complex binding;GO:0008168 methyltransferase activity

tivity;GO:0019707 protein-cysteine S-acyltransferase activity;GO:0016417 S-acyltransferas

3676 nucleic acid binding

signaling pathway - yeast
Pyrimidine metabolism; (sce00230)Purine metabolism

0:1901981 phosphatidylinositol phosphate binding;GO:0035091 phosphatidylinositol binding

8186 RNA-dependent ATPase activity;GO:0003724 RNA helicase activity

y;GO:0072509 divalent inorganic cation transmembrane transporter activity;GO:0046915 tran

8186 RNA-dependent ATPase activity;GO:0003724 RNA helicase activity
;GO:0005488 binding

5198 structural molecule activity;GO:0003674 molecular_function

ing, bridging;GO:0060090 molecular adaptor activity

olic pathways
03674 molecular_function
03674 molecular_function

de binding;GO:0032561 guanyl ribonucleotide binding

otein deacetylase activity;GO:0019213 deacetylase activity
smatch repair; (sce03030)DNA replication
tiple species; (sce04138)Autophagy - yeast; (sce04113)Meiosis - yeast
0019787 ubiquitin-like protein transferase activity;GO:0140096 catalytic activity, acting
on profiling and mass spectrometry
O:0004672 protein kinase activity;GO:0016773 phosphotransferase activity, alcohol group a

00)Metabolic pathways

g;GO:0043167 ion binding

translation initiation factor activity;GO:0008135 translation factor activity, RNA bindi

3420)Nucleotide excision repair

ce-specific DNA binding;GO:0000987 proximal promoter sequence-specific DNA binding;GO:000
733 peptide-lysine-N-acetyltransferase activity;GO:0034212 peptide N-acetyltransferase ac

is; (sce00513)Various types of N-glycan biosynthesis; (sce01100)Metabolic pathways
;GO:1904680 peptide transmembrane transporter activity;GO:0022884 macromolecule transmemb
e-specific DNA binding;GO:0001003 RNA polymerase III type 2 promoter sequence-specific DN
O:0016833 oxo-acid-lyase activity;GO:0010181 FMN binding

hagy - yeast

ed DNA binding;GO:0003723 RNA binding
9 ubiquitin-like protein ligase binding;GO:0019899 enzyme binding
nding;GO:0000977 RNA polymerase II regulatory region sequence-specific DNA binding;GO:000
Glycerophospholipid metabolism; (sce01100)Metabolic pathways

homoserine => threonine; (sce)Threonine biosynthesis, aspartate => homoserine => threoni
intramolecular transferase activity;GO:0016853 isomerase activity
n binding;GO:0005488 binding

3676 nucleic acid binding
lecular_function

ce-specific DNA binding;GO:0000987 proximal promoter sequence-specific DNA binding;GO:000
signaling pathway - yeast

ity;GO:0005385 zinc ion transmembrane transporter activity;GO:0072509 divalent inorganic

O:0004672 protein kinase activity;GO:0016773 phosphotransferase activity, alcohol group a

e-specific DNA binding;GO:0001003 RNA polymerase III type 2 promoter sequence-specific DN

ne metabolism; (sce00230)Purine metabolism
otein localizes to the mitochondrion
0138 ubiquitin-like protein-specific isopeptidase activity;GO:0016929 SUMO-specific prote
1100)Metabolic pathways
sferase activity, transferring one-carbon groups;GO:0016740 transferase activity
n corepressor activity;GO:0003712 transcription coregulator activity
molecular_function
sis - yeast
specific DNA binding;GO:0003677 DNA binding
ular adaptor activity;GO:0005515 protein binding

A binding;GO:0035613 RNA stem-loop binding

676 nucleic acid binding

ing;GO:0070273 phosphatidylinositol-4-phosphate binding;GO:0032266 phosphatidylinositol-3
4 molecular_function
ular adaptor activity;GO:0005515 protein binding
19843 rRNA binding;GO:0003735 structural constituent of ribosome
sce01110)Biosynthesis of secondary metabolites

prolyl cis-trans isomerase activity;GO:0016859 cis-trans isomerase activity

e pole body;GO:0044450 microtubule organizing center part
tivity;GO:0003674 molecular_function

RNA polymerase II-specific;GO:0044212 transcription regulatory region DNA binding;GO:000
92 RNA guanylyltransferase activity;GO:0070568 guanylyltransferase activity
008536 Ran GTPase binding;GO:0005048 signal sequence binding
ein complex binding;GO:0000981 DNA-binding transcription factor activity, RNA polymerase
ome binding;GO:0031490 chromatin DNA binding
ansport; (sce04138)Autophagy - yeast

0044877 protein-containing complex binding;GO:0005488 binding
19843 rRNA binding;GO:0043021 ribonucleoprotein complex binding
008253 5'-nucleotidase activity;GO:0008252 nucleotidase activity

m; (sce)Valine/isoleucine biosynthesis, pyruvate => valine / 2-oxobutanoate => isoleucine
sphorylation; (sce01100)Metabolic pathways
mRNA binding;GO:0003723 RNA binding
NA binding;GO:0003676 nucleic acid binding

tor activity;GO:0019208 phosphatase regulator activity

reticulum
ine metabolism; (sce00230)Purine metabolism

inding;GO:0031267 small GTPase binding
2-oxoglutarate => oxaloacetate; (M00009)Citrate cycle (TCA cycle, Krebs cycle); (M00011)
01130)Biosynthesis of antibiotics; (sce01110)Biosynthesis of secondary metabolites

0032550 purine ribonucleoside binding
hosphodiesterase I activity;GO:0047429 nucleoside-triphosphate diphosphatase activity

ular function regulator;GO:0003674 molecular_function

ce04111)Cell cycle - yeast; (sce04113)Meiosis - yeast
00)Metabolic pathways

;GO:1904680 peptide transmembrane transporter activity;GO:0022884 macromolecule transmemb
5249 voltage-gated potassium channel activity;GO:0022840 leak channel activity

ular adaptor activity;GO:0005515 protein binding
;GO:0003682 chromatin binding
674 molecular_function

GO:0005272 sodium channel activity;GO:0005262 calcium channel activity
ty;GO:0015238 drug transmembrane transporter activity;GO:0022857 transmembrane transporte

lycerol degradation; (sce00561)Glycerolipid metabolism
peroxiredoxin activity;GO:0004601 peroxidase activity

activity, hydrolyzing O-glycosyl compounds;GO:0016798 hydrolase activity, acting on glyco

13)Meiosis - yeast

r hydrolase activity;GO:0016801 hydrolase activity, acting on ether bonds

(sce03013)RNA transport
ic activity;GO:0003674 molecular_function
is (ALS)
activity;GO:0008948 oxaloacetate decarboxylase activity;GO:0016833 oxo-acid-lyase activi

nding;GO:0032559 adenyl ribonucleotide binding
3676 nucleic acid binding
rt
ne metabolism; (sce00230)Purine metabolism

tivity;GO:0046915 transition metal ion transmembrane transporter activity;GO:0015085 calc
82 copper-transporting ATPase activity;GO:0015086 cadmium ion transmembrane transporter a
733 peptide-lysine-N-acetyltransferase activity;GO:0034212 peptide N-acetyltransferase ac

on in cancer

fic DNA binding;GO:0046914 transition metal ion binding
sis - yeast

bolism; (sce01230)Biosynthesis of amino acids; (sce01130)Biosynthesis of antibiotics
ase activity;GO:0016887 ATPase activity

;GO:0060590 ATPase regulator activity;GO:0017056 structural constituent of nuclear pore

y;GO:0004322 ferroxidase activity;GO:0016724 oxidoreductase activity, oxidizing metal ion

4 molecular_function

tein binding;GO:0043022 ribosome binding
sphorylation; (sce01100)Metabolic pathways
in binding;GO:0030234 enzyme regulator activity
ce04111)Cell cycle - yeast
pase activity;GO:0052689 carboxylic ester hydrolase activity

iphosphatase activity;GO:0016462 pyrophosphatase activity
0005543 phospholipid binding;GO:0008289 lipid binding
is - yeast
e regulator activity;GO:0098772 molecular function regulator

ding, bending;GO:1990837 sequence-specific double-stranded DNA binding
reticulum
0005402 carbohydrate:cation symporter activity;GO:0015295 solute:proton symporter activit
0)Tyrosine metabolism; (sce00220)Arginine biosynthesis

cle - yeast; (sce04113)Meiosis - yeast

GO:0003674 molecular_function

pendent ATPase activity;GO:0042623 ATPase activity, coupled
e activity;GO:0036459 thiol-dependent ubiquitinyl hydrolase activity;GO:0004197 cysteine-
0:0003676 nucleic acid binding
ic activity;GO:0003674 molecular_function
GO:0043021 ribonucleoprotein complex binding

0015138 fumarate transmembrane transporter activity;GO:0015141 succinate transmembrane tr
e activity;GO:0036459 thiol-dependent ubiquitinyl hydrolase activity;GO:0019783 ubiquitin
-directed 5'-3' RNA polymerase activity;GO:0034062 5'-3' RNA polymerase activity

:0017111 nucleoside-triphosphatase activity
ty;GO:0015297 antiporter activity;GO:0015291 secondary active transmembrane transporter a

roline; (sce)Proline biosynthesis, glutamate => proline; (sce00330)Arginine and proline m

674 molecular_function

ansporter activity;GO:0003674 molecular_function

translation activator activity;GO:0090079 translation regulator activity, nucleic acid bi

ansferase activity, transferring hexosyl groups;GO:0016757 transferase activity, transfer
0)Mismatch repair; (sce03030)DNA replication
pendent ATPase activity;GO:0042623 ATPase activity, coupled

e04011)MAPK signaling pathway - yeast
e regulator activity;GO:0060589 nucleoside-triphosphatase regulator activity

e; (M00074)N-glycan biosynthesis, high-mannose type; (sce00513)Various types of N-glycan [illegible]
tein-containing complex binding;GO:0003723 RNA binding
ity;GO:0016423 tRNA (guanine) methyltransferase activity;GO:0008175 tRNA methyltransferas
ity;GO:0071077 adenosine 3',5'-bisphosphate transmembrane transporter activity;GO:0000295

5198 structural molecule activity;GO:0003674 molecular_function

sphorylation; (sce01100)Metabolic pathways

236 serine-type peptidase activity;GO:0017171 serine hydrolase activity

s spectrometry
kinase regulator activity;GO:0019887 protein kinase regulator activity;GO:0019207 kinase

inding;GO:0043169 cation binding

sis - yeast
sm; (sce01110)Biosynthesis of secondary metabolites; (sce01100)Metabolic pathways
0)Tryptophan metabolism; (sce00330)Arginine and proline metabolism
40)Spliceosome; (sce03013)RNA transport
nsporter activity;GO:0015301 anion:anion antiporter activity;GO:0015116 sulfate transmemb

nsferase; (sce)N-glycosylation by oligosaccharyltransferase; (sce00510)N-Glycan biosynthe

ing;GO:0048037 cofactor binding

factor binding;GO:0070491 repressing transcription factor binding;GO:0001085 RNA polymer
in-DNA loading ATPase activity;GO:0008094 DNA-dependent ATPase activity
ansport

se => UDP-glucose; (sce)Nucleotide sugar biosynthesis, glucose => UDP-glucose; (sce00040)[illegible]
0019787 ubiquitin-like protein transferase activity;GO:0140096 catalytic activity, acting
cle - yeast; (sce04113)Meiosis - yeast

lism; (sce04213)Longevity regulating pathway - multiple species; (sce01100)Metabolic path
n binding;GO:0005488 binding
g;GO:0051787 misfolded protein binding;GO:0031072 heat shock protein binding
e activity;GO:0036459 thiol-dependent ubiquitinyl hydrolase activity;GO:0004197 cysteine-

mary transcript binding;GO:0034513 box H/ACA snoRNA binding
oserine binding;GO:0099122 RNA polymerase II C-terminal domain binding;GO:0050815 phospho
sporter activity;GO:0051185 coenzyme transmembrane transporter activity;GO:0051184 cofact

ane transporter activity;GO:0015142 tricarboxylic acid transmembrane transporter activity
crotubule binding;GO:0015631 tubulin binding
vity regulating pathway - multiple species; (sce04139)Mitophagy - yeast
nsferase; (sce)N-glycosylation by oligosaccharyltransferase; (sce00510)N-Glycan biosynthe

y;GO:0016278 lysine N-methyltransferase activity;GO:0008276 protein methyltransferase act
ty;GO:0005516 calmodulin binding;GO:0004674 protein serine/threonine kinase activity

olymerase activity;GO:0034061 DNA polymerase activity

de binding;GO:0030554 adenyl nucleotide binding

0003697 single-stranded DNA binding;GO:0005543 phospholipid binding

:0008308 voltage-gated anion channel activity;GO:0015299 solute:proton antiporter activit

e protein binding;GO:0005198 structural molecule activity

198 structural molecule activity;GO:0003674 molecular_function

ity;GO:0046915 transition metal ion transmembrane transporter activity;GO:0046873 metal i
0005402 carbohydrate:cation symporter activity;GO:0015295 solute:proton symporter activit

ma R-mediated phagocytosis; (hsa04360)Axon guidance
ansport

> CoA; (M00120)Coenzyme A biosynthesis, pantothenate => CoA; (sce00770)Pantothenate and C

vity;GO:0016462 pyrophosphatase activity;GO:0016818 hydrolase activity, acting on acid an
5198 structural molecule activity;GO:0003674 molecular_function
binding;GO:0005488 binding
ptidase regulator activity;GO:0004857 enzyme inhibitor activity
ty, RNA polymerase II-specific;GO:0000981 DNA-binding transcription factor activity, RNA
O:0008175 tRNA methyltransferase activity;GO:0016706 oxidoreductase activity, acting on p
signaling pathway - yeast

ity;GO:0016427 tRNA (cytosine) methyltransferase activity;GO:0008175 tRNA methyltransfera
ome biogenesis in eukaryotes

005048 signal sequence binding;GO:0017056 structural constituent of nuclear pore

surveillance pathway; (sce04138)Autophagy - yeast
uclease activity;GO:0004519 endonuclease activity
ity;GO:0004721 phosphoprotein phosphatase activity;GO:0016791 phosphatase activity

733 peptide-lysine-N-acetyltransferase activity;GO:0034212 peptide N-acetyltransferase ac
n, oxaloacetate => 2-oxoglutarate; (sce)Citrate cycle, first carbon oxidation, oxaloaceta
0046906 tetrapyrrole binding

Glycerophospholipid metabolism; (sce04144)Endocytosis
0008135 translation factor activity, RNA binding;GO:0003723 RNA binding

-3 substituted reducing group;GO:0052861 glucan endo-1,3-beta-glucanase activity, C-3 sub

sis - yeast
sequence-specific DNA binding;GO:0001163 RNA polymerase I regulatory region sequence-spec

; (sce00514)Other types of O-glycan biosynthesis; (sce01100)Metabolic pathways

vity;GO:0015665 alcohol transmembrane transporter activity;GO:0042910 xenobiotic transmem

movement of ions, phosphorylative mechanism;GO:0004012 phospholipid-translocating ATPase

ity;GO:0015105 arsenite transmembrane transporter activity;GO:0015297 antiporter activity
3420)Nucleotide excision repair

0004661 protein geranylgeranyltransferase activity;GO:0008318 protein prenyltransferase a

ular adaptor activity;GO:0005515 protein binding

sferase activity, transferring one-carbon groups;GO:0016740 transferase activity
5198 structural molecule activity;GO:0003674 molecular_function
ing pathway - yeast

0043022 ribosome binding;GO:0008135 translation factor activity, RNA binding
4 molecular_function
02010)ABC transporters; (hsa04976)Bile secretion
lism; (sce01100)Metabolic pathways
species

rosclerosis; (hsa04621)NOD-like receptor signaling pathway
3676 nucleic acid binding
;GO:1904680 peptide transmembrane transporter activity;GO:0022884 macromolecule transmemb
vity;GO:1990189 peptide-serine-N-acetyltransferase activity;GO:0004596 peptide alpha-N-ac
0003714 transcription corepressor activity;GO:0003712 transcription coregulator activity

061650 ubiquitin-like protein conjugating enzyme activity;GO:0016874 ligase activity

34061 DNA polymerase activity;GO:0003684 damaged DNA binding
04497 monooxygenase activity;GO:0016705 oxidoreductase activity, acting on paired donors,

;GO:0015214 pyrimidine nucleoside transmembrane transporter activity;GO:0005337 nucleosid

n binding;GO:0008092 cytoskeletal protein binding

6616 oxidoreductase activity, acting on the CH-OH group of donors, NAD or NADP as accepto
activity;GO:0003674 molecular_function

13)RNA transport
ty;GO:0003924 GTPase activity;GO:0005525 GTP binding

20)Sulfur metabolism; (sce00270)Cysteine and methionine metabolism

lycerol degradation; (sce00561)Glycerolipid metabolism

e => UDP-galactose; (M00554)Nucleotide sugar biosynthesis, galactose => UDP-galactose; (s

um ion binding;GO:0030234 enzyme regulator activity
holipase activity;GO:0016298 lipase activity
vity;GO:0005088 Ras guanyl-nucleotide exchange factor activity;GO:0017137 Rab GTPase bind

mologous recombination; (sce03430)Mismatch repair
phosphatase regulator activity;GO:0030346 protein phosphatase 2B binding;GO:0019888 prot
gnaling adaptor activity;GO:0032947 protein-containing complex scaffold activity
e regulator activity;GO:0060589 nucleoside-triphosphatase regulator activity

sis - ganglio series; (hsa01100)Metabolic pathways

;GO:0005488 binding
nding;GO:0032559 adenyl ribonucleotide binding
ein complex binding;GO:0044877 protein-containing complex binding
;GO:1904680 peptide transmembrane transporter activity;GO:0022884 macromolecule transmemb
erine => cysteine; (M00338)Cysteine biosynthesis, homocysteine + serine => cysteine; (sce

kinase regulator activity;GO:0015248 sterol transporter activity;GO:0019887 protein kinas

ity;GO:0015037 peptide disulfide oxidoreductase activity;GO:0015035 protein disulfide oxi
se activity;GO:0004112 cyclic-nucleotide phosphodiesterase activity;GO:0008081 phosphoric
;GO:0015175 neutral amino acid transmembrane transporter activity;GO:0015179 L-amino acid

ty;GO:0022857 transmembrane transporter activity;GO:0005215 transporter activity
rase complex;GO:0043189 H4/H2A histone acetyltransferase complex

sporter activity

er activity
r transmembrane transporter activity

onors, with incorporation or reduction of molecular oxygen, NAD(P)H as one donor, and inc

cycle)

ivity;GO:1901474 azole transmembrane transporter activity

inding
0016491 oxidoreductase activity

on transmembrane transporter activity

inding
coupled to transmembrane movement of ions, phosphorylative mechanism
s acceptor

mbrane transporter activity

s, oxygen as acceptor

se 6P => ribulose 5P; (M00004)Pentose phosphate pathway (Pentose phosphate cycle)

embrane transporter activity

metabolism

mbrane transporter activity

g metal ions, NAD or NADP as acceptor

y

Fructose and mannose metabolism

ι; computational analysis of large-scale protein-protein interaction data suggests a role

id transmembrane transporter activity

, fructose 6P => ribose 5P; (M00004)Pentose phosphate pathway (Pentose phosphate cycle)
015405 P-P-bond-hydrolysis-driven transmembrane transporter activity

orporation or reduction of molecular oxygen

ine-dependent methyltransferase activity

015405 P-P-bond-hydrolysis-driven transmembrane transporter activity

transmembrane transporter activity

membrane transporter activity

UMP; (sce00240)Pyrimidine metabolism

omatic amino acid transmembrane transporter activity

003)Gluconeogenesis, oxaloacetate => fructose-6P

holipid metabolism
er binding
acceptor;GO:0016614 oxidoreductase activity, acting on CH-OH group of donors

species

bone biosynthesis

1228 DNA-binding transcription activator activity, RNA polymerase II-specific
lism

sporter activity

acid transmembrane transporter activity

16701 oxidoreductase activity, acting on single donors with incorporation of molecular ox

bone biosynthesis

ase II transcription factor binding

ed fatty acids
te => 2-oxoglutarate; (M00012)Glyoxylate cycle
of antibiotics

TCA cycle, Krebs cycle)

bone biosynthesis

ansporter activity

g metal ions, NAD or NADP as acceptor

brane transporter activity

043022 ribosome binding

ransporter activity

acceptor;GO:0016614 oxidoreductase activity, acting on CH-OH group of donors
activity, transferring glycosyl groups

the aldehyde or oxo group of donors, NAD or NADP as acceptor

lism

GO:0008556 potassium-transporting ATPase activity

003)Gluconeogenesis, oxaloacetate => fructose-6P

binding

id transmembrane transporter activity

inase activity

syl bonds

, fructose 6P => ribose 5P; (M00004)Pentose phosphate pathway (Pentose phosphate cycle)

vity, acting on the CH-OH group of donors, NAD or NADP as acceptor

s acceptor

factor binding

ty

Autophagy - yeast

ne transporter activity

s acceptor
activity
TCA cycle, Krebs cycle)

organic cation transmembrane transporter activity
P as acceptor;GO:0016903 oxidoreductase activity, acting on the aldehyde or oxo group of

s acceptor
0977 RNA polymerase II regulatory region sequence-specific DNA binding

polymerase II proximal promoter sequence-specific DNA binding

e

s acceptor
bone biosynthesis

P as acceptor;GO:0016903 oxidoreductase activity, acting on the aldehyde or oxo group of

10 transcription regulator activity

oreductase activity

or activity

ose from a single-locus duplication
003)Gluconeogenesis, oxaloacetate => fructose-6P
ific DNA-binding transcription factor binding;GO:0001085 RNA polymerase II transcription

on metal ion transmembrane transporter activity
type endopeptidase activity

tarate => 2-aminoadipate => lysine; (sce00300)Lysine biosynthesis

biotics
;GO:0016655 oxidoreductase activity, acting on NAD(P)H, quinone or similar compound as ac
ase II transcription factor binding
cceptor;GO:0016649 oxidoreductase activity, acting on the CH-NH group of donors, quinone

Fructose and mannose metabolism
s acceptor

acceptor;GO:0016614 oxidoreductase activity, acting on CH-OH group of donors

polymerase II-specific;GO:0043565 sequence-specific DNA binding

070491 repressing transcription factor binding
ivity
:reases in response to DNA replication stress
 inorganic cation transmembrane transporter activity

ty;GO:0015205 nucleobase transmembrane transporter activity

ways

activity

s acceptor

Galactose metabolism

 binding
-like protein-specific protease activity

biosynthesis

anscription factor binding
003)Gluconeogenesis, oxaloacetate => fructose-6P

tivity, coupled to transmembrane movement of substances

003)Gluconeogenesis, oxaloacetate => fructose-6P
polymerase II-specific;GO:0008270 zinc ion binding
porter activity

e activity

ctivity, acting on a sulfur group of donors, quinone or similar compound as acceptor
ysis (Embden-Meyerhof pathway), glucose => pyruvate
compounds

015405 P-P-bond-hydrolysis-driven transmembrane transporter activity

s acceptor

ivity

te => 2-oxoglutarate; (M00012)Glyoxylate cycle

1228 DNA-binding transcription activator activity, RNA polymerase II-specific

, core module involving three-carbon compounds

binding

003)Gluconeogenesis, oxaloacetate => fructose-6P

ctivity

in-specific protease activity

tivator activity

evated by sulfur limitation; YIL166C is a non-essential gene

brane transporter activity

on a protein

tivity

d transmembrane transporter activity

id transmembrane transporter activity

se 6P => ribulose 5P; (M00004)Pentose phosphate pathway (Pentose phosphate cycle)

tabolism

ng transcription factor activity

se 6P => ribulose 5P; (M00004)Pentose phosphate pathway (Pentose phosphate cycle)

-phosphate binding

'ity to 4-(N-(S-glutathionylacetyl)amino) phenylarsenoxide (GSAO)

bone biosynthesis

ansporter activity;GO:0005337 nucleoside transmembrane transporter activity

ontaining groups

ase II transcription factor binding

biosynthesis, AAA pathway, 2-oxoglutarate => 2-aminoadipate => lysine

acceptor;GO:0016614 oxidoreductase activity, acting on CH-OH group of donors

; (M00019)Valine/isoleucine biosynthesis, pyruvate => valine / 2-oxobutanoate => isoleuci

activity
olism

onuclease activity

holipid metabolism

ity

tive transmembrane transporter activity

binding

ate => valine / 2-oxobutanoate => isoleucine; (sce)Isoleucine biosynthesis, threonine =>
015405 P-P-bond-hydrolysis-driven transmembrane transporter activity
oate => ubiquinone; (sce00130)Ubiquinone and other terpenoid-quinone biosynthesis

06018 phosphatidylinositol-3,5-bisphosphate phosphatase activity

transcriptional regulator Ume6p; YIR014W is a non-essential gene

ivity
1228 DNA-binding transcription activator activity, RNA polymerase II-specific

binding

Fructose and mannose metabolism

ctivity

yruvate
UMP; (sce00250)Alanine, aspartate and glutamate metabolism

e

ne; (sce00750)Vitamin B6 metabolism
GO:0008556 potassium-transporting ATPase activity

id transmembrane transporter activity

ivity

ransporter activity

4P => chorismate; (sce00400)Phenylalanine, tyrosine and tryptophan biosynthesis

yltransferase activity
oreductase activity

rane transporter activity

1228 DNA-binding transcription activator activity, RNA polymerase II-specific

on a protein

zing metal ions, NAD or NADP as acceptor

te => 2-oxoglutarate; (M00012)Glyoxylate cycle

ay

ransporter activity

and epimerase activity

m

rane transporter activity
te => 2-oxoglutarate; (M00009)Citrate cycle (TCA cycle, Krebs cycle)

ways

se metabolism

ansferring one-carbon groups

y, forming carbon-sulfur bonds

nding

0977 RNA polymerase II regulatory region sequence-specific DNA binding

acid transmembrane transporter activity

l groups

-CH group of donors, NAD or NADP as acceptor
biosynthesis, AAA pathway, 2-oxoglutarate => 2-aminoadipate => lysine

ds;GO:0016798 hydrolase activity, acting on glycosyl bonds

e activity

polymerase II-specific;GO:0043565 sequence-specific DNA binding
;GO:0000981 DNA-binding transcription factor activity, RNA polymerase II-specific

(NADP+) activity

bone biosynthesis

binding

y

e degradation

rase activity

transmembrane transporter activity

ansmembrane transporter activity

ty

erol O-acyltransferase activity

y;GO:0051998 protein carboxyl O-methyltransferase activity

activity, transferring glycosyl groups

UMP; (sce00250)Alanine, aspartate and glutamate metabolism

anine, tyrosine and tryptophan biosynthesis
rine/threonine kinase activity

ecific DNA binding;GO:0000987 proximal promoter sequence-specific DNA binding

se metabolism

sulfur compound transmembrane transporter activity

oate => ubiquinone; (sce00130)Ubiquinone and other terpenoid-quinone biosynthesis

Galactose metabolism

ing

rane transporter activity
sferase activity

s acceptor

ivity
tivity
0016491 oxidoreductase activity

s acceptor

D activity;GO:0004620 phospholipase activity

hate 1-kinase activity

leotide-sugar transmembrane transporter activity

0977 RNA polymerase II regulatory region sequence-specific DNA binding
s acceptor
ivity
nthesis

polymerase II repressing transcription factor binding

(sce00260)Glycine, serine and threonine metabolism
iption factor recruiting activity;GO:0001030 RNA polymerase III type 1 promoter DNA bindi

4P => chorismate; (sce00400)Phenylalanine, tyrosine and tryptophan biosynthesis

y

polymerase II-specific;GO:0043565 sequence-specific DNA binding

biosynthesis, AAA pathway, 2-oxoglutarate => 2-aminoadipate => lysine

ive with either ribo- or deoxyribonucleic acids and producing 3'-phosphomonoesters
ctivity
e activity

id transmembrane transporter activity

ng transcription factor activity

1228 DNA-binding transcription activator activity, RNA polymerase II-specific

rane transporter activity
ism
anine, tyrosine and tryptophan biosynthesis

ctivity, active with either ribo- or deoxyribonucleic acids and producing 5'-phosphomonoe
transmembrane transporter activity

ide-methionine (S)-S-oxide reductase activity

activity, RNA polymerase II-specific

nsmembrane transporter activity

/threonine kinase activity

type endopeptidase activity

ase activity

ate tetrakisphosphate diphosphatase activity

se 6P => ribulose 5P; (M00004)Pentose phosphate pathway (Pentose phosphate cycle)

brane transporter activity;GO:0035673 oligopeptide transmembrane transporter activity
oA biosynthesis

metabolism

o acid transmembrane transporter activity

group of donors, oxygen as acceptor
DP/CTP; (sce00240)Pyrimidine metabolism

ase II transcription factor binding

ylate transmembrane transporter activity

ing;GO:0001134 transcription regulator recruiting activity

s

e activity
sis

or;GO:0016614 oxidoreductase activity, acting on CH-OH group of donors

ty

e activity, producing 5'-phosphomonoesters

yruvate

icrotubule motor activity

te => 2-oxoglutarate; (M00009)Citrate cycle (TCA cycle, Krebs cycle)

(sce04070)Phosphatidylinositol signaling system

003)Gluconeogenesis, oxaloacetate => fructose-6P

dehydrogenase activity

acid transmembrane transporter activity

;GO:0046943 carboxylic acid transmembrane transporter activity

stituted reducing group;GO:0042973 glucan endo-1,3-beta-D-glucosidase activity

Citrate cycle, second carbon oxidation, 2-oxoglutarate => oxaloacetate

bitor activity

5 organophosphate ester transmembrane transporter activity

nscription coregulator activity

ng transcription factor activity

chinery binding

ity

ase activity

ctivity, active with either ribo- or deoxyribonucleic acids and producing 5'-phosphomonoe
cycle, Krebs cycle)

1 RNA polymerase II basal transcription factor binding
rane transporter activity

onuclease activity

ransporter activity

e transporter activity

ransferase activity

DP/CTP; (sce)Guanine ribonucleotide biosynthesis IMP => GDP,GTP
brane transporter activity
g metal ions, NAD or NADP as acceptor
453 histone demethylase activity (H3-K4 specific)

ptidase activity

er binding

binding

ctivity

tochondria in high-throughput studies; protein abundance increases in response to DNA rep

;GO:0000976 transcription regulatory region sequence-specific DNA binding

ase activity

porter activity
AD-dependent histone deacetylase activity
binding, bending

UMP; (sce00240)Pyrimidine metabolism
rogen bonds

id transmembrane transporter activity

ransmembrane transporter activity

A methyltransferase activity

y

ine (PC) biosynthesis, choline => PC
n transmembrane transporter activity

(sce00290)Valine, leucine and isoleucine biosynthesis
nthesis, 2-oxoisovalerate => 2-oxoisocaproate

ransporter activity;GO:0042626 ATPase activity, coupled to transmembrane movement of subs

g transmembrane transporter activity

transferase activity

ivity

n transmembrane transporter activity

tivity

demethylase activity

amma-subunit complex binding

g metal ions, NAD or NADP as acceptor
polymerase II proximal promoter sequence-specific DNA binding

003)Gluconeogenesis, oxaloacetate => fructose-6P

phosphate ester transmembrane transporter activity

hosphatase activity

l spore wall permeability and sensitivity to beta-glucanase digestion
se activity

0977 RNA polymerase II regulatory region sequence-specific DNA binding

adenine nucleotide transmembrane transporter activity

ase III general transcription initiation factor activity

id transmembrane transporter activity

sferase activity

ity

A

ed fatty acids
erring acyl groups

on the CH-CH group of donors

eme-copper terminal oxidase activity

doreductase activity
ring glycosyl groups
ctivity

binding

sequence-specific double-stranded DNA binding

UMP; (sce00240)Pyrimidine metabolism

73 metal ion transmembrane transporter activity

ing
eric DNA binding

symporter activity

sequence-specific double-stranded DNA binding
, fructose 6P => ribose 5P; (M00004)Pentose phosphate pathway (Pentose phosphate cycle)
00260)Glycine, serine and threonine metabolism

1228 DNA-binding transcription activator activity, RNA polymerase II-specific

ity

ivity

sition metal ion transmembrane transporter activity

brane transporter activity

:tion; expression induced by calcium shortage; YJL147W is a non-essential gene

s acceptor

membrane transporter activity

e activity

, fructose 6P => ribose 5P; (M00004)Pentose phosphate pathway (Pentose phosphate cycle)
, fructose 6P => ribose 5P; (M00004)Pentose phosphate pathway (Pentose phosphate cycle)

oate => ubiquinone; (sce00130)Ubiquinone and other terpenoid-quinone biosynthesis

imilar compound as acceptor

 symporter activity

ty, RNA polymerase II-specific

 binding

ansferring one-carbon groups

binding

ate binding

oate => ubiquinone; (sce00130)Ubiquinone and other terpenoid-quinone biosynthesis

transmembrane transporter activity

003)Gluconeogenesis, oxaloacetate => fructose-6P

s acceptor

in-dependent protein kinase activity

0977 RNA polymerase II regulatory region sequence-specific DNA binding

l groups
Citrate cycle, second carbon oxidation, 2-oxoglutarate => oxaloacetate

y;GO:0005338 nucleotide-sugar transmembrane transporter activity

ific DNA-binding transcription factor binding;GO:0001085 RNA polymerase II transcription
ity;GO:0004722 protein serine/threonine phosphatase activity

oenzyme complex

transmembrane transporter activity

Citrate cycle, second carbon oxidation, 2-oxoglutarate => oxaloacetate

osphatase activity

y

serine/threonine phosphatase activity
inase inhibitor activity

tivity

DP/CTP; (sce00240)Pyrimidine metabolism

ds

id transmembrane transporter activity

on a protein
n a sulfur group of donors

dary metabolites

tidase activity

on transmembrane transporter activity
ng transcription factor activity

tinyl hydrolase activity

e activity

eptide transmembrane transporter activity

ctor activity

chinery binding
organophosphate ester transmembrane transporter activity

r;GO:0071949 FAD binding

type endopeptidase activity

transferase activity

tivity;GO:0030696 tRNA (m5U54) methyltransferase activity

phosphate binding

biosynthesis

binding
s acceptor

0977 RNA polymerase II regulatory region sequence-specific DNA binding

tivity

acid transmembrane transporter activity
0977 RNA polymerase II regulatory region sequence-specific DNA binding

0016896 exoribonuclease activity, producing 5’-phosphomonoesters

e activity

987 proximal promoter sequence-specific DNA binding
bule motor activity

but not peptide) bonds, in cyclic amidines
t

lfur cluster binding

ce-specific DNA binding

n transmembrane transporter activity

osome

binding

ctivity

acceptor;GO:0016614 oxidoreductase activity, acting on CH-OH group of donors

cs

ase II transcription factor binding
0977 RNA polymerase II regulatory region sequence-specific DNA binding

ds

003)Gluconeogenesis, oxaloacetate => fructose-6P

rane transporter activity

1 RNA polymerase II basal transcription factor binding

regulator activity

rane transporter activity

e activity

sis

on the CH-CH group of donors

sis

ivity

ransporter activity

activity

GO:0034595 phosphatidylinositol phosphate 5-phosphatase activity

binding
GO:0008138 protein tyrosine/serine/threonine phosphatase activity
se activity

ption initiation factor activity
biosynthesis

transmembrane transporter activity

ed fatty acids

:0016879 ligase activity, forming carbon-nitrogen bonds

erol O-acyltransferase activity

se activity

ty

anine, tyrosine and tryptophan biosynthesis

ore promoter sequence-specific DNA binding

ase II transcription factor binding

(sce00564)Glycerophospholipid metabolism

DNA binding

ycerophospholipid metabolism

ansferring one-carbon groups
transmembrane transporter activity

:R093C-A, that arose from the whole genome duplication

embrane transporter activity
e activity
2 groups;GO:0016491 oxidoreductase activity

n transmembrane transporter activity

ding;GO:0001013 RNA polymerase I regulatory region DNA binding

tivity

organophosphate ester transmembrane transporter activity

ansferase activity

anic anion transmembrane transporter activity
bolites

g metal ions, NAD or NADP as acceptor

Autophagy - yeast

; (sce00250)Alanine, aspartate and glutamate metabolism

ism
se activity, acting on carbon-nitrogen (but not peptide) bonds

inase activity

monoesters

ansmembrane transporter activity
g

ing;GO:0001134 transcription regulator recruiting activity

003)Gluconeogenesis, oxaloacetate => fructose-6P

romatic amino acid transmembrane transporter activity

tivator activity

605 organophosphate ester transmembrane transporter activity
cleotide transmembrane transporter activity

binding

tivity

ol signaling system

IMP => ADP,ATP

r;GO:0010181 FMN binding
idylinositol (GPI)-anchor biosynthesis

se 6P => ribulose 5P; (M00004)Pentose phosphate pathway (Pentose phosphate cycle)

ctivity

s acceptor
or activity

1228 DNA-binding transcription activator activity, RNA polymerase II-specific

ing

oreductase activity

tivity

e activity

nsmembrane transporter activity

osensitive ion channel activity

clease activity

annosidase activity
s acceptor

sis
1228 DNA-binding transcription activator activity, RNA polymerase II-specific

sferase activity

ne; (sce00300)Lysine biosynthesis

epressor activity

1228 DNA-binding transcription activator activity, RNA polymerase II-specific
1228 DNA-binding transcription activator activity, RNA polymerase II-specific

biosynthesis, AAA pathway, 2-oxoglutarate => 2-aminoadipate => lysine

nsferase activity

as acceptor;GO:0016667 oxidoreductase activity, acting on a sulfur group of donors

ne transporter activity

e activity

te => 2-oxoglutarate; (M00009)Citrate cycle (TCA cycle, Krebs cycle)

porter activity

vity

tivity;GO:0005290 L-histidine transmembrane transporter activity

ADP as acceptor;GO:0016903 oxidoreductase activity, acting on the aldehyde or oxo group o
phosphate binding

; (sce00230)Purine metabolism

0977 RNA polymerase II regulatory region sequence-specific DNA binding
idylinositol (GPI)-anchor biosynthesis

ivity
serine residue binding

ganic cation transmembrane transporter activity

g on the CH-CH group of donors, quinone or related compound as acceptor

erase activity, transferring acyl groups

ption initiation factor activity

ansmembrane transporter activity

0977 RNA polymerase II regulatory region sequence-specific DNA binding

n a sulfur group of donors

l groups

to the mitochondria

ific DNA binding;GO:0001181 RNA polymerase I general transcription initiation factor acti

tivity

on factor activity
) methyltransferase activity

DNA binding

c cation transmembrane transporter activity

ity

merase II-specific

m

porter activity

1228 DNA-binding transcription activator activity, RNA polymerase II-specific

vity

ransferase activity
4P => chorismate; (sce00400)Phenylalanine, tyrosine and tryptophan biosynthesis

ansporter activity

acid transmembrane transporter activity

acid transmembrane transporter activity
ed fatty acids

binding;GO:0001012 RNA polymerase II regulatory region DNA binding

olymerase II transcription cofactor binding

ase II transcription regulator recruiting activity

ine/threonine phosphatase activity

lecular oxidoreductase activity

ivity

idylinositol (GPI)-anchor biosynthesis
cing 5'-phosphomonoesters
a; YKL133C has a paralog, MGR3, that arose from the whole genome duplication

te => 2-oxoglutarate; (M00009)Citrate cycle (TCA cycle, Krebs cycle)

or;GO:0016879 ligase activity, forming carbon-nitrogen bonds

r;GO:0010181 FMN binding
lin-dependent protein serine/threonine kinase regulator activity

003)Gluconeogenesis, oxaloacetate => fructose-6P

3' exodeoxyribonuclease activity

ism
ivity

ate => valine / 2-oxobutanoate => isoleucine; (sce)Isoleucine biosynthesis, threonine =>

ce)Galactose degradation, Leloir pathway, galactose => alpha-D-glucose-1P
A
l bonds
n transmembrane transporter activity
polymerase II-specific;GO:0008270 zinc ion binding

inding

ing
CH-CH group of donors, NAD or NADP as acceptor
s acceptor

symporter activity

003)Gluconeogenesis, oxaloacetate => fructose-6P

on a protein

(sce00290)Valine, leucine and isoleucine biosynthesis

tarate => 2-aminoadipate => lysine; (sce00300)Lysine biosynthesis

e activity
AD-dependent histone deacetylase activity

s acceptor

0 protein transmembrane transporter activity

se 6P => ribulose 5P; (M00004)Pentose phosphate pathway (Pentose phosphate cycle)

n a sulfur group of donors

1228 DNA-binding transcription activator activity, RNA polymerase II-specific

n the CH-NH2 group of donors;GO:0016491 oxidoreductase activity
e activity

ganic cation transmembrane transporter activity

transmembrane transporter activity

sition metal ion transmembrane transporter activity

nsmembrane transporter activity
phosphomonoesters

binding

doreductase activity
syl bonds

as acceptor
s acceptor

ne transporter activity

activity, alcohol group as acceptor
ity, alcohol group as acceptor

Citrate cycle, second carbon oxidation, 2-oxoglutarate => oxaloacetate

1 oxidoreductase activity, acting on NAD(P)H

ty

binding

 metabolism
oA biosynthesis

(sce00290)Valine, leucine and isoleucine biosynthesis

te transmembrane transporter activity
brane transporter activity

ed fatty acids

inase binding

trogen limitation in a GLN3, GAT1-independent manner

1228 DNA-binding transcription activator activity, RNA polymerase II-specific
1228 DNA-binding transcription activator activity, RNA polymerase II-specific

1228 DNA-binding transcription activator activity, RNA polymerase II-specific

 on the CH-CH group of donors
s acceptor
1228 DNA-binding transcription activator activity, RNA polymerase II-specific

binding

saminase activity

ty

te => H2S

 on a protein
s acceptor

sferase activity

097472 cyclin-dependent protein kinase activity

0:0004860 protein kinase inhibitor activity

binding

73 metal ion transmembrane transporter activity

g transcription factor binding

ine, aspartate and glutamate metabolism

ity

sferase activity

metabolites

oreductase activity, oxidizing metal ions

ctivity;GO:0000295 adenine nucleotide transmembrane transporter activity

acid transmembrane transporter activity

097472 cyclin-dependent protein kinase activity

tidase activity

-phosphate binding

ule motor activity
vity, transferring glycosyl groups
003)Gluconeogenesis, oxaloacetate => fructose-6P

anine, tyrosine and tryptophan biosynthesis
Galactose metabolism

ase activity

h incorporation or reduction of molecular oxygen, NAD(P)H as one donor, and incorporation

=> isoleucine; (sce00290)Valine, leucine and isoleucine biosynthesis

le)
idylinositol (GPI)-anchor biosynthesis
polymerase II-specific;GO:0043565 sequence-specific DNA binding

ce-specific DNA binding

nscription factor binding
idylinositol (GPI)-anchor biosynthesis
s

transmembrane transporter activity

tivity

biosynthesis

ing
ng
acceptor;GO:0016614 oxidoreductase activity, acting on CH-OH group of donors

n transmembrane transporter activity

1228 DNA-binding transcription activator activity, RNA polymerase II-specific

UMP; (sce00250)Alanine, aspartate and glutamate metabolism

:0016695 oxidoreductase activity, acting on hydrogen as donor

tivity, coupled to transmembrane movement of substances

400)Phenylalanine, tyrosine and tryptophan biosynthesis

biosynthesis
ng transcription factor activity
0977 RNA polymerase II regulatory region sequence-specific DNA binding

ism

rane transporter activity

ity

1228 DNA-binding transcription activator activity, RNA polymerase II-specific

ate => valine / 2-oxobutanoate => isoleucine; (sce)Isoleucine biosynthesis, threonine =>

idylinositol (GPI)-anchor biosynthesis

g metal ions, NAD or NADP as acceptor

ry metabolites

s acceptor

ET pathway; preference for substrates with downstream transmembrane domains; interacts wi
ity

ygous diploid mutant shows an increase in glycogen accumulation

1228 DNA-binding transcription activator activity, RNA polymerase II-specific
tivity

ing

groups

ltransferase activity

tic activity, acting on a tRNA
e N-methyltransferase activity

bonuclease activity, producing 5’-phosphomonoesters
sis

ring glycosyl groups

r;GO:0010181 FMN binding

n transmembrane transporter activity

e) methyltransferase activity

e

tivity

tion initiation factor activity
anine, tyrosine and tryptophan biosynthesis

tivity, active with either ribo- or deoxyribonucleic acids and producing 5'-phosphomonoes

biosynthesis, AAA pathway, 2-oxoglutarate => 2-aminoadipate => lysine

e N-methyltransferase activity

lyase activity

phosphoprotein phosphatase activity

t rRNA binding

ding

phosphate binding

er activity

A binding;GO:0001030 RNA polymerase III type 1 promoter DNA binding

21 phosphoprotein phosphatase activity

- yeast

ansmembrane transporter activity

0 protein transmembrane transporter activity

regulator activity

ity

=> alpha-D-glucose-1P; (sce00052)Galactose metabolism

leotide-sugar transmembrane transporter activity

=> PE; (sce00564)Glycerophospholipid metabolism
sis

yribonucleotide kinase activity

binding

e activity

ate => valine / 2-oxobutanoate => isoleucine; (sce)Isoleucine biosynthesis, threonine =>

tivity

le)

ule motor activity
bule motor activity

de transmembrane transporter activity

idylinositol (GPI)-anchor biosynthesis

ransporter activity

, producing 5'-phosphomonoesters

O:0016740 transferase activity

acid transmembrane transporter activity

tive transmembrane transporter activity

ransporter activity
e transporter activity

cleotide-sugar transmembrane transporter activity

lease activity, producing 3'-phosphomonoesters

porter activity

activity;GO:0042625 ATPase coupled ion transmembrane transporter activity

n transmembrane transporter activity

(sce00564)Glycerophospholipid metabolism

rginine N-methyltransferase activity

IMP => ADP,ATP

ty

athways

II repressing transcription factor binding
drolase activity, acting on carbon-nitrogen (but not peptide) bonds, in linear amides

thyltransferase activity

ing

e activity

ways

ity, alcohol group as acceptor

-CH group of donors, NAD or NADP as acceptor

894 endonuclease activity, active with either ribo- or deoxyribonucleic acids and produci

ity;GO:0035312 5'-3' exodeoxyribonuclease activity

74 ligase activity

nthesis

sis

ctivity

ansporter activity

; (sce00670)One carbon pool by folate

0977 RNA polymerase II regulatory region sequence-specific DNA binding

Fructose and mannose metabolism

e activity

regulator activity

:ing uptake or incorporation of uracil into RNA; proposed to be involved in the metabolism

on a protein

ansporter activity

Citrate cycle, second carbon oxidation, 2-oxoglutarate => oxaloacetate

imilar compound as acceptor

45 manganese ion binding

ing

type endopeptidase activity

ty
015405 P-P-bond-hydrolysis-driven transmembrane transporter activity

biosynthesis

ransmembrane transporter activity

cing 5'-phosphomonoesters

binding;GO:0001012 RNA polymerase II regulatory region DNA binding

s acceptor
monoesters

ase II transcription factor binding
regulator activity

te ester transmembrane transporter activity

e N-methyltransferase activity

400)Phenylalanine, tyrosine and tryptophan biosynthesis

binding

cs

0977 RNA polymerase II regulatory region sequence-specific DNA binding
, core module involving three-carbon compounds
yl groups other than amino-acyl groups

te => 2-oxoglutarate; (M00009)Citrate cycle (TCA cycle, Krebs cycle)

0977 RNA polymerase II regulatory region sequence-specific DNA binding

metabolism
-like protein-specific protease activity

membrane transporter activity

ing
carbon-sulfur bonds

, fructose 6P => ribose 5P; (sce00040)Pentose and glucuronate interconversions
transcription factor complex binding
xosyl groups

ty

porter activity

A binding;GO:0001030 RNA polymerase III type 1 promoter DNA binding

on a protein

atidylethanolamine (PE) biosynthesis, ethanolamine => PE

asal transcription factor binding

c cation transmembrane transporter activity

ce-specific DNA binding

ctor activity

ty

species

ty
ng

sition metal ion transmembrane transporter activity

oate => ubiquinone; (sce00130)Ubiquinone and other terpenoid-quinone biosynthesis

-phosphate binding
saminase activity

nscription coactivator activity

membrane transporter activity
nosine monophosphate biosynthesis, PRPP + glutamine => IMP

omonoesters

ansferring one-carbon groups

e N-methyltransferase activity

ator activity

ibosome

binding
ing
s acceptor

1228 DNA-binding transcription activator activity, RNA polymerase II-specific
l groups
binding
ase activity, transferring one-carbon groups

s, oxygen as acceptor
idylinositol (GPI)-anchor biosynthesis

biosynthesis

acting on the CH-CH group of donors

ctivity

oximal promoter sequence-specific DNA binding

tarate => 2-aminoadipate => lysine; (sce00300)Lysine biosynthesis
ontaining groups

e activity

binding

le)

0977 RNA polymerase II regulatory region sequence-specific DNA binding

ding;GO:0140223 general transcription initiation factor activity

some biogenesis in eukaryotes

phosphate binding
se activity

activity

tive transmembrane transporter activity

ed fatty acids

DP/CTP; (sce00240)Pyrimidine metabolism

ivity

atidylethanolamine (PE) biosynthesis, ethanolamine => PE

inase inhibitor activity

chinery binding

tone deacetylase activity (H3-K14 specific)

oreductase activity

oA biosynthesis

of donors, oxygen as acceptor

ıighly sensitive to azaserine and resistant to sodium-O-vandate
ding;GO:0001013 RNA polymerase I regulatory region DNA binding

cceptor

activity

syl bonds

/threonine kinase activity

binding

AD-dependent histone deacetylase activity

anscription factor binding

y, forming carbon-sulfur bonds

y;GO:0051998 protein carboxyl O-methyltransferase activity
0287 magnesium ion binding

hylated histone binding

ity

s acceptor

type endopeptidase activity

e transporter activity

activity

=> isoleucine; (sce00290)Valine, leucine and isoleucine biosynthesis
ansferring one-carbon groups

ionine transmembrane transporter activity

UMP; (sce00240)Pyrimidine metabolism
l groups
sporter activity

acid transmembrane transporter activity
serine/threonine phosphatase activity

-like protein-specific protease activity

·expression causes a cell cycle delay or arrest
ase II transcription factor binding
0:0043492 ATPase activity, coupled to movement of substances
on factor binding

s acceptor

tivity

e N-methyltransferase activity

e activity

with incorporation or reduction of molecular oxygen, 2-oxoglutarate as one donor, and in

on a protein

sequence-specific double-stranded DNA binding
etate

; (sce00670)One carbon pool by folate

ity

003)Gluconeogenesis, oxaloacetate => fructose-6P

rane transporter activity

ansferase activity

but not peptide) bonds, in cyclic amidines

ty

aspartate => homoserine => threonine

activity

activity, active with either ribo- or deoxyribonucleic acids and producing 5'-phosphomon

n a sulfur group of donors

; (sce00230)Purine metabolism

transmembrane transporter activity

ecific DNA binding;GO:0000987 proximal promoter sequence-specific DNA binding

regulator activity

l groups

ansmembrane transporter activity

polymerase I regulatory region sequence-specific DNA binding

ity;GO:0000179 rRNA (adenine-N6,N6-)-dimethyltransferase activity

metabolism

yase activity
ibonuclease activity

inding

rane transporter activity

ule motor activity
ways

ansporting ATPase activity

ctivity, acting on a sulfur group of donors, quinone or similar compound as acceptor

chenodeoxycholate; (hsa00120)Primary bile acid biosynthesis

chinery binding

:ET pathway; preference for substrates with downstream transmembrane domains; interacts wi

; (sce00220)Arginine biosynthesis
A binding;GO:0001030 RNA polymerase III type 1 promoter DNA binding

UMP; (sce00240)Pyrimidine metabolism

NA polymerase II-specific;GO:0000981 DNA-binding transcription factor activity, RNA polym

embrane transporter activity

brane anchor

; (sce00330)Arginine and proline metabolism

te ester transmembrane transporter activity

015405 P-P-bond-hydrolysis-driven transmembrane transporter activity
ivity

ansferring one-carbon groups

ransporter activity

vity

regulator activity

ulator activity;GO:0030295 protein kinase activator activity

nthesis

ways
tide) bonds

g on the CH-CH group of donors

s acceptor

s acceptor

binding
ivity

on factor activity

680 peptide transmembrane transporter activity

1228 DNA-binding transcription activator activity, RNA polymerase II-specific

ivity

A binding;GO:0001030 RNA polymerase III type 1 promoter DNA binding

1228 DNA-binding transcription activator activity, RNA polymerase II-specific

onuclease activity

;GO:0000976 transcription regulatory region sequence-specific DNA binding

0 protein transmembrane transporter activity

; (sce00230)Purine metabolism
g metal ions, NAD or NADP as acceptor

hibitor activity
group of donors, oxygen as acceptor

n transmembrane transporter activity

syl bonds

ng agent MMS

vity, transferring glycosyl groups

erase activity, transferring acyl groups

nthesis
rane transporter activity

4P => chorismate; (sce00400)Phenylalanine, tyrosine and tryptophan biosynthesis

group as acceptor

e N-methyltransferase activity

on a protein

tivity

s acceptor

se 6P => ribulose 5P; (M00004)Pentose phosphate pathway (Pentose phosphate cycle)
and epimerase activity

serine residue binding

sulfide oxidoreductase activity

s acceptor

tivity

ting ATPase activity

binding

ivity

binding;GO:0001012 RNA polymerase II regulatory region DNA binding

oreductase activity

deoxyribonucleotide kinase activity

gulator activity, nucleic acid binding

ivity

ity

tivity

1228 DNA-binding transcription activator activity, RNA polymerase II-specific

ce)Galactose degradation, Leloir pathway, galactose => alpha-D-glucose-1P

ransporter activity;GO:0042626 ATPase activity, coupled to transmembrane movement of subs

1228 DNA-binding transcription activator activity, RNA polymerase II-specific

DNA binding
Citrate cycle, second carbon oxidation, 2-oxoglutarate => oxaloacetate

on a protein

type endopeptidase activity

ism

0977 RNA polymerase II regulatory region sequence-specific DNA binding

porter activity
ty

oA biosynthesis

ine/threonine phosphatase activity

e promoter binding

;GO:1990939 ATP-dependent microtubule motor activity

ity

Pentose and glucuronate interconversions

ups other than amino-acyl groups

680 sulfur-containing amino acid secondary active transmembrane transporter activity
ing
on a protein

ar amides;GO:0016810 hydrolase activity, acting on carbon-nitrogen (but not peptide) bond

:0016746 transferase activity, transferring acyl groups

s acceptor

nsmembrane transporter activity
on a protein
acid transmembrane transporter activity

monoesters

arose from the whole genome duplication

cid transmembrane transporter activity
130)Ubiquinone and other terpenoid-quinone biosynthesis

esters

e transporter activity

binding

transmembrane transporter activity

activity, transferring acyl groups

n transmembrane transporter activity

of donors, disulfide as acceptor;GO:0016903 oxidoreductase activity, acting on the aldeh
ecific DNA binding;GO:0000987 proximal promoter sequence-specific DNA binding
ity

; (sce00670)One carbon pool by folate

e N-methyltransferase activity

 transmembrane transporter activity
dehydrogenase activity

vity

ic cation transmembrane transporter activity
regulator activity

s acceptor

ty

tivity

ate binding

ocalize to the vacuole, while SWAT-GFP fusion also localizes to the endoplasmic reticulum
ctor activity

n transmembrane transporter activity
Fructose and mannose metabolism

bonds

pha-N-acetyltransferase activity

type endopeptidase activity

; (sce00230)Purine metabolism
ng transcription factor activity

id transmembrane transporter activity

te ester transmembrane transporter activity

ing

097472 cyclin-dependent protein kinase activity
transferase activity

aspartate => homoserine => threonine

Citrate cycle, second carbon oxidation, 2-oxoglutarate => oxaloacetate

e activity

activity

onuclease activity

e activity

sition metal ion transmembrane transporter activity

on a protein

s acceptor

ng

0977 RNA polymerase II regulatory region sequence-specific DNA binding
tivity

rane transporter activity
A binding;GO:0001030 RNA polymerase III type 1 promoter DNA binding

0976 transcription regulatory region sequence-specific DNA binding

ne; (sce00260)Glycine, serine and threonine metabolism

1228 DNA-binding transcription activator activity, RNA polymerase II-specific

cation transmembrane transporter activity

s acceptor

A binding;GO:0001030 RNA polymerase III type 1 promoter DNA binding

ase activity

-phosphate binding

8270 zinc ion binding

II-specific

; (M00019)Valine/isoleucine biosynthesis, pyruvate => valine / 2-oxobutanoate => isoleuci

Citrate cycle, second carbon oxidation, 2-oxoglutarate => oxaloacetate

rane transporter activity

r activity

syl bonds

ty

ium ion transmembrane transporter activity
ctivity
tivity

s, oxygen as acceptor

y

type endopeptidase activity

ansporter activity
-like protein-specific protease activity

ctivity

etabolism

nding

ring glycosyl groups

biosynthesis

e activity
 adenine nucleotide transmembrane transporter activity

regulator activity

rane transporter activity

sis

ase II transcription factor binding

Pentose and glucuronate interconversions
 on a protein

ways

type endopeptidase activity

serine residue binding
or transmembrane transporter activity

;GO:0046943 carboxylic acid transmembrane transporter activity

sis

ivity

y

on transmembrane transporter activity
y

oA biosynthesis

hydrides, in phosphorus-containing anhydrides

polymerase II-specific;GO:0043565 sequence-specific DNA binding
aired donors, with incorporation or reduction of molecular oxygen, 2-oxoglutarate as one

se activity

tivity
te => 2-oxoglutarate; (M00012)Glyoxylate cycle

stituted reducing group;GO:0042973 glucan endo-1,3-beta-D-glucosidase activity

ific DNA binding;GO:0001181 RNA polymerase I general transcription initiation factor acti

brane transporter activity

activity;GO:0042625 ATPase coupled ion transmembrane transporter activity

ctivity

rane transporter activity
etyltransferase activity

with incorporation or reduction of molecular oxygen

e transmembrane transporter activity

r;GO:0016614 oxidoreductase activity, acting on CH-OH group of donors

ce)Galactose degradation, Leloir pathway, galactose => alpha-D-glucose-1P

ing

ein phosphatase regulator activity

rane transporter activity
00450)Selenocompound metabolism

e regulator activity

doreductase activity
 diester hydrolase activity
 transmembrane transporter activity

orporation of two atoms of oxygen into one donor

in ATP/ADP exchange

ygen;GO:0051213 dioxygenase activity

donors

donors

factor binding

ceptor

or similar compound as acceptor

ne

2-oxobutanoate => isoleucine

ng

sters

sters

›lication stress

tances

factor binding

f donors

vity

2-oxobutanoate => isoleucine

of one atom of oxygen

2-oxobutanoate => isoleucine

th Snd1p, Env10p/Snd2p, and Sec61p-translocon subunits; can compensate for loss of SRP; r

ters

2-oxobutanoate => isoleucine

ng 3'-phosphomonoesters

n of purine and pyrimidine base analogues; deletion mutants are sensitive to HAP and AHA

corporation of one atom each of oxygen into both donors

oesters

th Snd1p, Pho88p/Snd3p, and Sec61p-translocon subunits; can compensate for loss of SRP;

erase II-specific

tances

S

yde or oxo group of donors

ne

donor, and incorporation of one atom each of oxygen into both donors

vity

role in phosphate transport, interacting with pho88, and in the maturation of secretory pr

role in the late endosome-vacuole interface; putative role in secretory protein quality co

[illegible]roteins

ontrol
